# Structure-based prediction of protein-protein interaction network in rice

**DOI:** 10.1590/1678-4685-GMB-2023-0068

**Published:** 2024-02-02

**Authors:** Fangnan Sun, Yaxin Deng, Xiaosong Ma, Yuan Liu, Lingxia Zhao, Shunwu Yu, Lida Zhang

**Affiliations:** 1 Shanghai Jiao Tong University, School of Agriculture and Biology, Department of Plant Science, Shanghai, China.; 2 Shanghai Academy of Agricultural Sciences, Shanghai Agrobiological Gene Center, Shanghai, China.

**Keywords:** Rice, protein-protein interaction, interactome, protein docking

## Abstract

Comprehensive protein-protein interaction (PPI) maps are critical for understanding the functional organization of the proteome, but challenging to produce experimentally. Here, we developed a computational method for predicting PPIs based on protein docking. Evaluation of performance on benchmark sets demonstrated the ability of the docking-based method to accurately identify PPIs using predicted protein structures. By employing the docking-based method, we constructed a structurally resolved PPI network consisting of 24,653 interactions between 2,131 proteins, which greatly extends the current knowledge on the rice protein-protein interactome. Moreover, we mapped the trait-associated single nucleotide polymorphisms (SNPs) to the structural interactome, and computationally identified 14 SNPs that had significant consequences on PPI network. The protein structural interactome map provided a resource to facilitate functional investigation of PPI-perturbing alleles associated with agronomically important traits in rice.

## Introduction

Protein-protein interactions (PPIs) are involved in a wide range of biological processes, including signal transduction, stress responses, plant defense, and organ formation. Comprehensive mapping of PPI networks can provide crucial insights into the control of biological processes in plants. Advances in yeast two-hybrid and affinity purification mass spectrometry have increased the capability to detect PPIs in the model plants (Arabidopsis Interactome Mapping Consortium, 2011; Jones et al., 2014; Altmann et al., 2020; Wierbowski et al., 2020). However, the current available experimental PPI data are still far from a comprehensive map of plant PPI networks. 

Computational approaches offer another means by which to identify plant PPIs via integrating various biological information (Cui et al., 2008; Lin et al., 2011; Zhu et al., 2016). Recently, structural information has been used to improve PPI prediction in plants (Zhang et al., 2016; Liu et al., 2017). There are two typical methods for structure-based PPI predictions: one method based on structural matching with known complex structures and another method based on protein docking. The template-based method predicts PPIs on structural similarity of proteins to complex structures, which is highly dependent on the available structural templates in the database. The docking-based method does not require *a priori* structural templates, and it can identify new PPIs that have different structures from those of known complexes. 

Docking algorithms are primarily designed to analyze the structural characteristics of individual known protein interactions. Identifying large-scale PPIs using docking techniques is computationally expensive. However, progress in computer science has facilitated the application of protein docking to large-scale PPI prediction (Mosca et al., 2009; Wass et al., 2011; Vakser, 2014). Since only a fraction of protein structures have been determined experimentally, the docking-based methods have recently been shifted to rely on protein models instead of on higher resolution experimentally resolved structures (Singh et al., 2020). Low-resolution docking on protein models is especially important in proteome-wide prediction of PPI networks for sequenced organisms in which the available experimental structures are lagging far behind from known protein sequences.

Rice (*Oryza sativa*) is a model plant for studying the biology of cereal crops. Although its complete genome sequence has been available for two decades, the known PPIs are still limited. Currently, there are only a few hundred experimentally determined rice PPIs deposited in the public database (Oughtred et al., 2021). This large gap indicates that there is still a long way to go in elucidating the protein-protein interactome in rice. In this study, we present a docking-based method that can be used to identify PPIs using predicted protein structures that is comparable in performance to the other computational methods. By using the docking-based method, we constructed a structurally resolved PPI interactome consisting of 24,653 interactions, greatly expending the knowledge on the rice protein-protein interactome. Moreover, we mapped the trait-associated single nucleotide polymorphisms (SNPs) to the rice structural interactome, and computationally identified 14 nonsynonymous SNPs that had significant consequences on the PPI network. Our study provides a resource to facilitate prioritization and further characterization of PPI-perturbing alleles associated with agronomically important traits in rice.

## Material and Methods

### Computational modeling of rice proteome

The rice protein sequences were retrieved from the Nipponbare reference genome release 7.0 (http://rice.uga.edu/index.shtml) (Kawahara et al., 2013). After filtering transposable element (TE)-related genes, we obtained 38,864 non-TE-related protein sequences for structure modeling. 

The three-dimensional (3D) structures of proteins were predicted using the batch processing facility of ModPipe (Webb and Sali, 2021). The homology model with the highest ModPipe quality score was selected for each protein according to previously described criteria (Zhang et al., 2016; Liu et al., 2017). To determine the quality of the protein model, the sequence identity and alignment coverage were calculated by aligning the protein with the structural template. A homology model was considered to be of high quality if it exhibited a sequence identity > 50% and an alignment coverage > 80% with the corresponding structural template (Dong et al., 2019).

### PPI prediction based on docking

Protein-protein docking for all possible binary combinations was performed using the ZDOCK program (version 3.0.2) (Pierce et al., 2011). The standardized score (z score) was calculated by comparing the docking score of the top prediction to the distribution of 2000 high-ranked decoys, which was used to assess the possible interaction between the given protein pairs.

### Evaluation of docking performance

The set of 1,122 benchmark interactions was obtained from the Dockground docking benchmark set 4 (http://dockground.compbio.ku.edu) and the GWIDD database (Genome-WIde Docking Database, http://gwidd.compbio.ku.edu). To make the benchmark sets consistent and homogeneous, a homology model was generated for each protein from the benchmark complexes. To avoid self-hits, each model had to have < 95% sequence identity with the template. The negative set of 1,122 pairs of experimental structures and 588 pairs of homology models were generated by randomly shuffling the benchmark interactions.

The test set of 30 rice binary complex structures was collected from the PDB database (Rose et al., 2021). The predicted protein structure was also generated for each chain. We assessed the possible interaction for each pair in all-to-all combinations. Two proteins from each complex were defined as the positive pair, and all shuffled protein pairs were defined as the negative set.

The accuracy of the prediction regarding whether the given members of a protein pair could interact with each other or not was evaluated as true positive (TP), false positive (FP), true negative (TN), and false negative (FN). The overall performance of the docking-based method was assessed by the true positive rate (TPR) or recall (TP/(TP+FN)), the false positive rate (FPR) (FP/(FP+TN)), the precision (TP/(TP+FP)) and the F-measure ((2*Precision*Recall)/(Precision+Recall)). The receiver operating characteristic curve (ROC) and area under the curve (AUC) were also used to measure the performance of the docking-based method in distinguishing the true interactions from random protein pairs. 

### Interolog-based PPI prediction

The orthologs of rice proteins in six organisms, *Arabidopsis thaliana*, *Saccharomyces cerevisiae*, *Caenorhabditis elegans*, *Drosophila melanogaster*, *Mus musculus* and *Homo sapiens*, were identified using InParanoid with default settings (Sonnhammer and Östlund, 2015). The experimentally determined PPI sets were retrieved from the public databases BioGRID (Oughtred et al., 2021), IntAct (Orchard et al., 2014) and Mentha (Calderone et al., 2013). Two rice proteins were predicted to interact with each other if their orthologs interacted in at least one of the six reference organisms.

### Gene coexpression analysis

The coexpression data of rice genes was downloaded from ATTED-II v11 database (https://atted.jp/download/). The standardized coexpression value (coexpression z-score) was constructed using integrative analysis of both RNA-seq and microarray data (Obayashi et al., 2022). 

### Subcellular localization analysis

The subcellular localization information of rice proteins was obtained from the prediction of WoLF PSORT (Horton et al., 2007). If there was more than one subcellular compartment associated with a protein, the winner-takes-all strategy was used to annotate a subcellular location for each protein (Geisler-Lee et al., 2007). Enrichment analysis for the interacting proteins with respect to the subcellular localization were performed based on hypergeometric test. 

### Annotation of trait-associated SNPs

The set of 14,424 rice SNPs associated with agronomically important traits was downloaded from the GWAS Atlas (https://ngdc.cncb.ac.cn/gwas/downloads) (Tian et al., 2020). The effect of each trait-associated SNP was annotated using SnpEff based on the Nipponbare reference genome release 7.0 (Cingolani et al., 2012). A total of 1,915 nonsynonymous SNPs were identified in the coding regions of 1,361 annotated genes in rice. 

### Assessment of SNPs on protein-protein interaction

The interfaces of docked protein structures were predicted by the Prodigy program (https://bianca.science.uu.nl/prodigy/) (Jiménez-García et al., 2019). The changes in protein-protein binding affinity caused by nonsynonymous SNPs were estimated using mCSM-PPI2 software (http://biosig.unimelb.edu.au/mcsm_ppi2/) (Rodrigues et al., 2019). The change in binding affinity (ΔΔG) from each allele was defined as the difference between these two binding energies.

$$∆∆G={∆G}_{REF}-{∆G}_{ALT}$$

## Results

### Structure modeling of rice proteome

**Figure 1 -:**
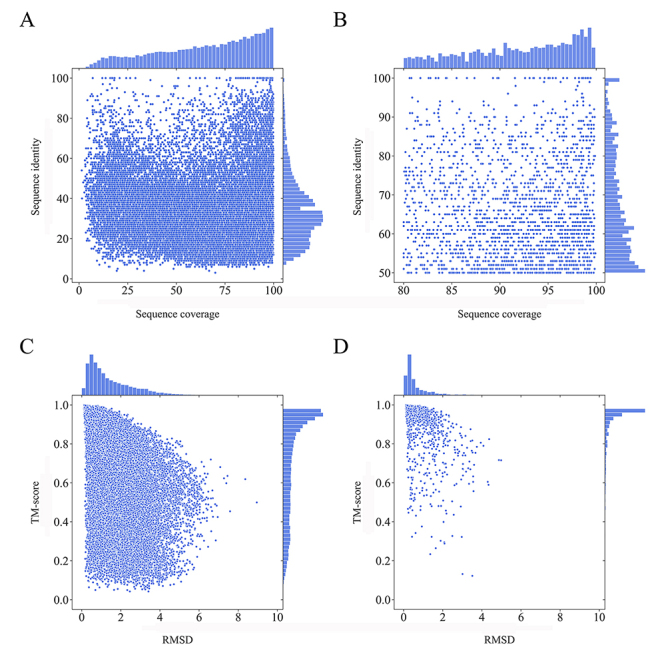
Homology models of rice proteome. Scatter plots of the relationship between sequence identity and alignment coverage of all predicted protein structures (A) and the high-quality models (B). Scatter plots of the relationship between the TM-score and RMSD of all predicted protein structures (C) and the high-quality models (D).

There are currently only 225 experimental structures for rice proteins in the Protein Data Bank. To fill the gap between rice protein sequences and 3D structures, we used homology modeling to predict protein structures, which led to 32,170 models covering 82.8% of the rice proteome. The average alignment identity and coverage were 34% and 64% between the protein models and their corresponding structural templates, respectively (Figure 1A). Protein models with > 50% sequence identity and > 80% alignment coverage were considered to be of high quality. As a result, we obtained a total of 2,083 high-quality protein models, which had on average 66% sequence identity and 92% alignment coverage with the templates (Figure 1B).

To assess the quality of the predicted protein structures, we used the TM-score to measure the topological similarity between the homology models and their corresponding structural templates (Zhang and Skolnick, 2005). Compared with the overall average TM-score of 0.71 (Figure 1C), a larger TM-score of 0.93 was found between the high-quality models and the templates. Of the high-quality models, 98.6% (2054/2083) had a TM-score larger than 0.5 (Figure 1D), indicating that these homology models had the correctly predicted topology (Xu and Zhang, 2010). In addition to the TM-score, we also used the root mean square deviation (RMSD) to measure structural similarity between the protein models and the structural templates. The average RMSD of high-quality models was 0.62 Å, with 94.5% (1968/2083) of models having an RMSD below 2 Å (Figure 1C, D), which indicates that the accuracy of the high-quality models was sufficient for docking analysis (Vakser, 2014).

### Evaluation of docking performance using experimental and predicted structures

**Figure 2 -:**
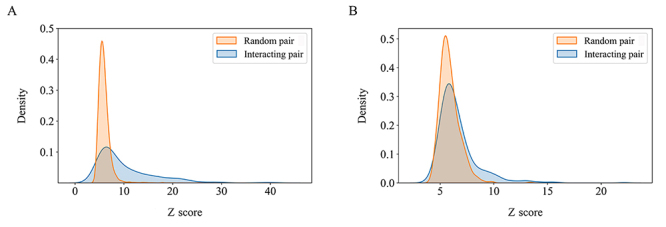
Distribution of docking z scores for interacting proteins and random pairs using (A) experimental structures and (B) homology models.

**Figure 3 -:**
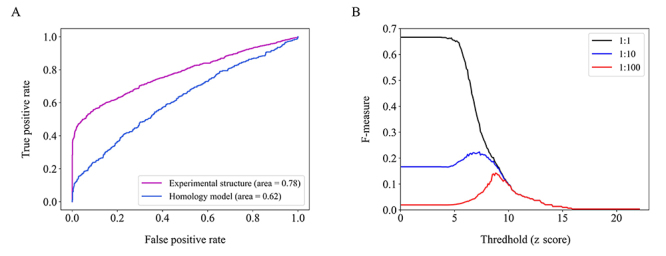
Performance evaluation of the docking-based method. (A) ROC curves for the docking-based method in discriminating true interactions from random protein pairs using experimental structures and homology models. AUC values are reported in parentheses. (B) F-measure as a function of the z score threshold on different ratios of benchmark interactions and random protein pairs using homology models.

To evaluate the ability of a docking method to predict PPIs, we used the ZDOCK program to perform protein docking on 1,122 benchmark interactions using experimental structures (Table S1) (Pierce et al., 2011). The docking z score of the top prediction was then calculated for each protein pair. As shown in Figure 2A, the distribution of the benchmark interactions (mean z score = 10.0) is clearly shifted toward higher docking z scores compared to random protein pairs (mean z score = 5.8) using experimental structures. We also evaluated the performance of ZDOCK on 588 known interactions against predicted protein structures. Similarly, the docking z scores of interacting protein pairs (mean z score = 6.6) were higher than those of random pairs (mean z score = 5.9) (Figure 2B). The enrichment of interacting protein pairs toward the high end of the docking z score distribution indicated that the ZDOCK based method could distinguish true interactions from random pairs using experimental structures and homology models. Furthermore, we used the ROC curves to quantitatively assess the performance of the docking-based method. The area under curve (AUC) was 0.78 for benchmark interactions using experimental structures and 0.62 for known interactions using homology models (Figure 3A), indicating the better performance of the docking-based method over random chance. 

Considering the fact that interaction occurred very infrequently in random protein pairs, the effects on docking performance were evaluated against different ratios of benchmark interactions and random pairs using predicted protein structures. The F-measure values at various docking z score thresholds are shown in Figure 3B. On the balanced dataset, the z score threshold of 3.7 yielded the maximum F-measure value of 0.67 with a precision of 50% and an FPR of 100% (Table 1). However, the high FPR would result in a very large number of false positives in proteome-wide PPI prediction. By changing the ratio between true interactions and random pairs to 1:100, the FPR significantly decreased from 100% to 0.3% at the higher z score threshold of 8.8. The low FPR was similar to the possibility of interactions occurring in random plant protein pairs (Arabidopsis Interactome Mapping Consortium, 2011). Therefore, the z score threshold of 8.8 was used for PPI decisions based on protein docking in this study.

**Table 1 -:** Effects on the docking performance of changing the ratios of positive and negative pairs using homology models.

Ratio (positive:negative)	Optimal z score threshold	TPR	FPR	Precision	F-measure
1:1 (588:588)	3.7	1.0000	1.0000	0.5000	0.6667
1:10 (588:5880)	7.2	0.2245	0.0803	0.2185	0.2215
1:100 (588:58800)	8.8	0.1003	0.0031	0.2458	0.1425

**Figure 4 -:**
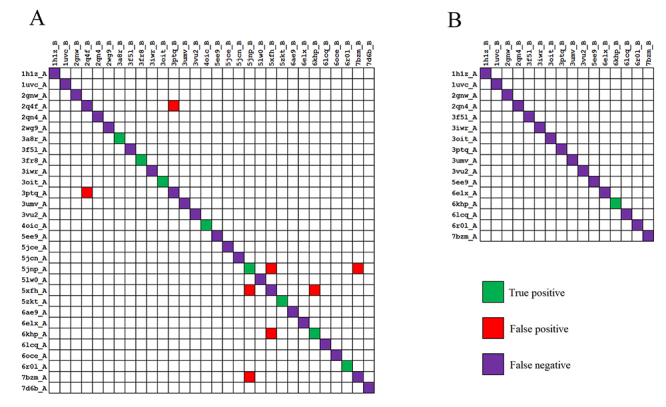
Performance evaluation of the docking-based method on rice binary complexes using (A) experimental structures and (B) homology models. Green, red and purple cells represent true positives, false positives, and false negatives, respectively.

To measure the performance of the docking-based method with the optimal z score threshold, all-to-all docking was carried out on the proteins from the 30 rice binary complexes (Table S2). At the z score threshold of 8.8, the docking-based method predicted 16 PPIs with 8 positives and 8 false positives using experimental structures (Figure 4A), while it identified one known interaction but no false positives using predicted protein structures (Figure 4B). These results indicated that the docking-based method could be used to identify rice PPIs using experimental structures and homology models.

### Structure-based interactome in rice

**Figure 5 -:**
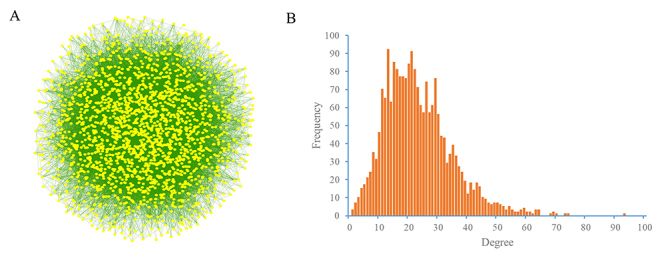
Predicted rice protein-protein interactome. (A) Overview of the predicted rice PPI network. (B) Degree distribution of the node proteins in the PPI network.

A total of 2,132 rice proteins with experimental structures and high-quality models were collected for docking analysis. There were 2,271,646 possible binary interactions without self-interactions that could be formed from these collected rice proteins. We carried out ZDOCK docking on all 2.27 million protein pairs and assessed the possible interaction for each pair using the docking z score. At a z score threshold of 8.8, we generated a structurally resolved rice protein-protein interactome consisting of 24,653 interactions between 2,131 proteins (Figure 5A; Table S3). The degree distribution indicated that one rice protein on average had 23 interacting partners, and approximately 70% of proteins had 10-30 connections with others in the structural protein interactome (Figure 5B). 

To assess the accuracy of the docking-based method in predicting rice PPIs, we compared it with some computational methods on a set of 95 experimentally determined PPIs with 3D structures (Liu et al., 2017; Szklarczyk et al., 2021). The docking-based method yielded comparable performance to the other three methods based on the F-measure (Table 2). Of the docking-inferred PPIs, 1,350 (5.5%) interactions were also detected by at least one of the three methods (Figure S1), which was significantly higher than the probability of random chance. We compared the docking-based method to the state-of-the-art deep learning based AlphaFold-Multimer (AF2-multimer) for PPI prediction (Evans et al., 2021). Of 25 randomly selected docking-inferred PPIs, 2 (8.0%) interactions were supported by the high-accuracy model of AF2-multimer with the ranking confidence > 0.70. We also used coexpression data to validate the PPIs predicted by the docking-based method (Obayashi et al., 2022). The gene expression analysis exhibited a statistically significant trend of coexpression for the predicted PPIs when compared to random gene pairs (Figure S2). Furthermore, the subcellular localization analysis showed that the interacting proteins tended to be located in the same subcellular compartment such as cytosol and nucleus (Figure S3). These results indicated that the docking-based method could predict PPIs using only structural information, which provides another perspective in protein-protein interactome exploration in rice.

**Table 2 -:** Performance comparison of computational methods on the experimental PPIs in rice.

Method	Predicted PPIs with 3D structure	Predicted PPIs verified by experiments	Precision	Recall	F-measure
Interolog	91,372	9	0.00010	0.09474	0.0002
RicePPINet	33,732	4	0.00012	0.04211	0.0002
STRING (v11.5)	383	0	NA	NA	NA
Docking	24,653	1	0.00004	0.01053	0.0001

NA means not available.

### Network effects of trait-associated SNPs on interactome

**Figure 6 -:**
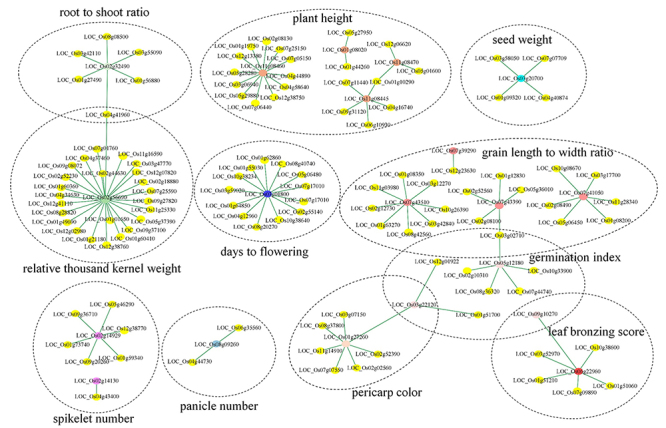
PPI subnetwork with trait-associated nonsynonymous SNPs at the predicted interface.

**Figure 7 -:**
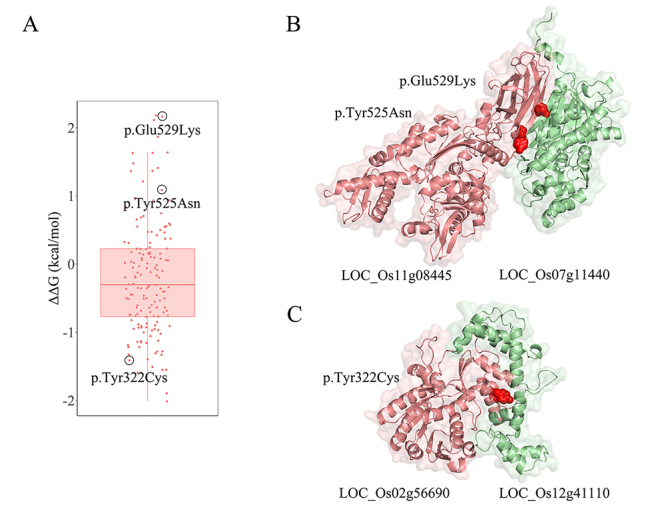
Predicted impact of nonsynonymous SNPs on the binding affinity of PPIs. (A) Distribution of the changes in binding affinity of PPIs caused by all 34 trait-associated nonsynonymous SNPs at the predicted interface. (B) Docked structure between LOC_Os11g08445 and LOC_Os07g11440. (C) Docked structure between LOC_Os02g56690 and LOC_Os12g41110. The nonsynonymous SNPs at the predicted interface are highlighted as red spheres.

Genome-wide association studies have identified 1,915 trait-associated nonsynonymous SNPs within 1,361 protein-coding genes in rice (Tian et al., 2020). Nevertheless, the functional consequences of these trait-associated SNPs remain largely unknown. We mapped these trait-associated SNPs to the structural protein-protein interactome, and identified 34 nonsynonymous SNPs located at the predicted interfaces of 119 PPIs (Figure 6; Table S4). The prediction of binding affinity changes (ΔΔG) showed that these SNPs could affect the stability of PPIs (Table S5), including 8 SNPs significantly increasing the binding affinity of 13 PPIs (ΔΔG > 1 kcal⁄mol) and 7 SNPs having the opposite effect on 24 PPIs (ΔΔG < -1 kcal⁄mol) (Figure 7A). 

Of the 8 favorable variants, 6 in DnaK family genes (LOC_Os11g08445, LOC_Os11g08460 and LOC_Os11g08470) were significantly associated with plant height. The LOC_Os11g08445 allele carrying p.Tyr525Asn and p.Glu529Lys substitutions were predicted to strengthen the interaction between LOC_Os11g08445 and LOC_Os07g11440 (Figure 7B). The locus LOC_Os07g11440 encoded chalcone synthase, which is known to be involved in the biosynthesis of flavonoids and plant circadian rhythm. The relative thousand kernel weight associated variant p.Tyr322Cys in LOC_Os02g56690 significantly decreased the binding affinity of 15 PPIs, including the interaction between LOC_Os02g56690 and LOC_Os12g41110 (calmodulin-like protein 5, CML5) (Figure 7C). The binding affinity between p.Tyr322Cys LOC_Os02g56690 and CML5 was predicted to change by -1.411 kcal⁄mol, suggesting that the strength of interaction with CML5 was perturbed by the p.Tyr322Cys substitution. These results indicated that the structurally resolved protein-protein interactome could help facilitate prioritization of PPI-perturbing alleles associated with agronomically important traits in rice. 

## Discussion

Here, we present a computational approach for predicting PPIs based on protein docking. Our study demonstrated that the docking-based method could accurately distinguish PPIs from random protein pairs using only structural information. We found that the docking-based method performed better using experimental structures compared to homology models. However, docking experimental structures are challenging to use widely to predict the interactomes because only a fraction of protein structures are determined experimentally (Velankar et al., 2021). The technique of inferring PPIs from low-resolution docking could be applied to interactome exploration for sequenced organisms lacking data from experimental structures (Dong et al., 2019).

Rice was the first crop to be fully sequenced, but information about its PPIs is still limited (Struk et al., 2019). Although rice PPI networks have been constructed by computational approaches (Gu et al., 2011; Ho et al., 2012; Liu et al., 2017), the docking-based method using only structural information provides another perspective in rice protein-protein interactome exploration. We applied the docking-based method to the interactome and constructed a structurally resolved PPI network consisting of 24,653 interactions, which greatly expands our knowledge of the protein-protein interactome in rice. 

The identification of genetic variants associated with rice agronomic traits has been facilitated by high-throughput sequencing technologies, but functional characterization and molecular mechanism exploration of trait-associated variants remain major challenges (Huang et al., 2010; Wang et al., 2018; Gupta et al., 2019). Structurally resolved protein-protein interactomes provide more detailed insights into the structural characteristics of PPIs, which have been used to facilitate investigation of the network effects of genetic variants at amino acid resolution (Ghadie and Xia, 2019; Cheng et al., 2021). By mapping the trait-associated SNPs to the structural protein interactome, we identified 34 nonsynonymous SNPs that were located at the PPI interfaces, of which 14 SNPs were predicted to have significant consequences on the PPI network. The structurally resolved protein-protein interactome provides a resource to facilitate prioritization of PPI-perturbing alleles associated with agronomic traits and further functional characterization of genetic variants in rice. 

## Supplementary material

The following online material is available for this article:

Table S1 - List of 1,122 benchmark interactions with experimental structures.

Table S2 - List of 30 rice binary complexes.

Table S3 - List of predicted rice protein-protein interactions.

Table S4 - Trait-associated nonsynonymous SNPs at the predicted interface.

Table S5 - Predicted changes in binding affinity (ΔΔG) caused by nonsynonymous SNPs.

Figure S1 - Venn diagram of PPIs predicted by the docking-based method, interolog mapping, RicePPINet, and STRING.

Figure S2 - Coexpression analysis of PPIs predicted by the docking-based method.

Figure S3- Subcellular localization analysis of the interacting proteins.

## References

[B1] Altmann M, Altmann S, Rodriguez PA, Weller B, Elorduy Vergara L, Palme J, Marín-de la Rosa N, Sauer M, Wenig M, Villaécija-Aguilar JA (2020). Extensive signal integration by the phytohormone protein network. Nature.

[B2] Arabidopsis Interactome Mapping Consortium (2011). Evidence for network evolution in an Arabidopsis interactome map. Science.

[B3] Calderone A, Castagnoli L, Cesareni G (2013). mentha: A resource for browsing integrated protein-interaction networks. Nat Methods.

[B4] Cheng F, Zhao J, Wang Y, Lu W, Liu Z, Zhou Y, Martin WR, Wang R, Huang J, Hao T (2021). Comprehensive characterization of protein-protein interactions perturbed by disease mutations. Nat Genet.

[B5] Cingolani P, Platts A, Wang LL, Coon M, Nguyen T, Wang L, Land SJ, Lu X, Ruden DM (2012). A program for annotating and predicting the effects of single nucleotide polymorphisms, SnpEff: SNPs in the genome of Drosophila melanogaster strain w1118; iso-2; iso-3. Fly (Austin).

[B6] Cui J, Li P, Li G, Xu F, Zhao C, Li Y, Yang Z, Wang G, Yu Q, Li Y (2008). AtPID: Arabidopsis thaliana protein interactome database--an integrative platform for plant systems biology. Nucleic Acids Res.

[B7] Dong S, Lau V, Song R, Ierullo M, Esteban E, Wu Y, Sivieng T, Nahal H, Gaudinier A, Pasha A (2019). Proteome-wide, structure-based prediction of protein-protein interactions/new molecular interactions viewer. Plant Physiol.

[B8] Evans R, O’Neill M, Pritzel A, Antropova N, Senior A, Green T, Žídek A, Bates R, Blackwell S, Yim J (2021). Protein complex prediction with AlphaFold-Multimer. bioRxiv.

[B9] Geisler-Lee J, O’Toole N, Ammar R, Provart NJ, Millar AH, Geisler M (2007). A predicted interactome for Arabidopsis. Plant Physiol.

[B10] Ghadie M, Xia Y (2019). Estimating dispensable content in the human interactome. Nat Commun.

[B11] Gu H, Zhu P, Jiao Y, Meng Y, Chen M (2011). PRIN: A predicted rice interactome network. BMC Bioinformatics.

[B12] Gupta PK, Kulwal PL, Jaiswal V (2019). Association mapping in plants in the post-GWAS genomics era. Adv Genet.

[B13] Ho CL, Wu Y, Shen HB, Provart NJ, Geisler M (2012). A predicted protein interactome for rice. Rice (N Y).

[B14] Horton P, Park KJ, Obayashi T, Fujita N, Harada H, Adams-Collier CJ, Nakai K (2007). WoLF PSORT: Protein localization predictor. Nucleic Acids Res.

[B15] Huang X, Wei X, Sang T, Zhao Q, Feng Q, Zhao Y, Li C, Zhu C, Lu T, Zhang Z (2010). Genome-wide association studies of 14 agronomic traits in rice landraces. Nat Genet.

[B16] Jiménez-García B, Elez K, Koukos PI, Bonvin AM, Vangone A (2019). PRODIGY-crystal: A web-tool for classification of biological interfaces in protein complexes. Bioinformatics.

[B17] Jones AM, Xuan Y, Xu M, Wang RS, Ho CH, Lalonde S, You CH, Sardi MI, Parsa SA, Smith-Valle E (2014). Border control--a membrane-linked interactome of Arabidopsis. Science.

[B18] Kawahara Y, de la Bastide M, Hamilton JP, Kanamori H, McCombie WR, Ouyang S, Schwartz DC, Tanaka T, Wu J, Zhou S (2013). Improvement of the Oryza sativa Nipponbare reference genome using next generation sequence and optical map data. Rice (N Y).

[B19] Lin M, Zhou X, Shen X, Mao C, Chen X (2011). The predicted Arabidopsis interactome resource and network topology-based systems biology analyses. Plant Cell.

[B20] Liu S, Liu Y, Zhao J, Cai S, Qian H, Zuo K, Zhao L, Zhang L (2017). A computational interactome for prioritizing genes associated with complex agronomic traits in rice (Oryza sativa). Plant J.

[B21] Mosca R, Pons C, Fernández-Recio J, Aloy P (2009). Pushing structural information into the yeast interactome by high-throughput protein docking experiments. PLoS Comput Biol.

[B22] Obayashi T, Hibara H, Kagaya Y, Aoki Y, Kinoshita K (2022). ATTED-II v11: A plant gene coexpression database using a sample balancing technique by subagging of principal components. Plant Cell Physiol.

[B23] Orchard S, Ammari M, Aranda B, Breuza L, Briganti L, Broackes-Carter F, Campbell NH, Chavali G, Chen C, del-Toro N (2014). The MIntAct project--IntAct as a common curation platform for 11 molecular interaction databases. Nucleic Acids Res.

[B24] Oughtred R, Rust J, Chang C, Breitkreutz BJ, Stark C, Willems A, Boucher L, Leung G, Kolas N, Zhang F (2021). The BioGRID database: A comprehensive biomedical resource of curated protein, genetic, and chemical interactions. Protein Sci.

[B25] Pierce BG, Hourai Y, Weng Z (2011). Accelerating protein docking in ZDOCK using an advanced 3D convolution library. PLoS One.

[B26] Rodrigues CHM, Myung Y, Pires DEV, Ascher DB (2019). mCSM-PPI2: Predicting the effects of mutations on protein-protein interactions. Nucleic Acids Res.

[B27] Rose Y, Duarte JM, Lowe R, Segura J, Bi C, Bhikadiya C, Chen L, Rose AS, Bittrich S, Burley SK (2021). RCSB protein data bank: Architectural advances towards integrated searching and efficient access to macromolecular structure data from the PDB Archive. J Mol Biol.

[B28] Singh A, Dauzhenka T, Kundrotas PJ, Sternberg MJE, Vakser IA (2020). Application of docking methodologies to modeled proteins. Proteins.

[B29] Sonnhammer EL, Östlund G (2015). InParanoid 8: Orthology analysis between 273 proteomes, mostly eukaryotic. Nucleic Acids Res.

[B30] Struk S, Jacobs A, Sánchez Martín-Fontecha E, Gevaert K, Cubas P, Goormachtig S (2019). Exploring the protein-protein interaction landscape in plants. Plant Cell Environ.

[B31] Szklarczyk D, Gable AL, Nastou KC, Lyon D, Kirsch R, Pyysalo S, Doncheva NT, Legeay M, Fang T, Bork P (2021). The STRING database in 2021: Customizable protein-protein networks, and functional characterization of user-uploaded gene/measurement sets. Nucleic Acids Res.

[B32] Tian D, Wang P, Tang B, Teng X, Li C, Liu X, Zou D, Song S, Zhang Z (2020). GWAS Atlas: A curated resource of genome-wide variant-trait associations in plants and animals. Nucleic Acids Res.

[B33] Vakser IA (2014). Protein-protein docking: From interaction to interactome. Biophys J.

[B34] Velankar S, Burley SK, Kurisu G, Hoch JC, Markley JL (2021). The protein data bank archive. Methods Mol Biol.

[B35] Wang W, Mauleon R, Hu Z, Chebotarov D, Tai S, Wu Z, Li M, Zheng T, Fuentes RR, Zhang F (2018). Genomic variation in 3,010 diverse accessions of Asian cultivated rice. Nature.

[B36] Wass MN, Fuentes G, Pons C, Pazos F, Valencia A (2011). Towards the prediction of protein interaction partners using physical docking. Mol Syst Biol.

[B37] Webb B, Sali A (2021). Protein structure modeling with MODELLER. Methods Mol Biol.

[B38] Wierbowski SD, Vo TV, Falter-Braun P, Jobe TO, Kruse LH, Wei X, Liang J, Meyer MJ, Akturk N, Rivera-Erick CA (2020). A massively parallel barcoded sequencing pipeline enables generation of the first ORFeome and interactome map for rice. Proc Natl Acad Sci U S A.

[B39] Xu J, Zhang Y (2010). How significant is a protein structure similarity with TM-score = 0.5?. Bioinformatics.

[B40] Zhang Y, Skolnick J (2005). TM-align: A protein structure alignment algorithm based on the TM-score. Nucleic Acids Res.

[B41] Zhang F, Liu S, Li L, Zuo K, Zhao L, Zhang L (2016). Genome-Wide inference of protein-protein interaction networks identifies crosstalk in abscisic acid signaling. Plant Physiol.

[B42] Zhu G, Wu A, Xu XJ, Xiao PP, Lu L, Liu J, Cao Y, Chen L, Wu J, Zhao XM (2016). PPIM: A Protein-Protein Interaction Database for Maize. Plant Physiol.

