# Supplementary material for: Structure-based prediction of protein-protein interaction network in rice
Source: Genet Mol Biol. 2024 Feb 2;47(1):e20230068. doi: 10.1590/1678-4685-GMB-2023-0068 (PMC10849033; doi:10.1590/1678-4685-GMB-2023-0068)
Supplement: Table S1 - [file 1415-4757-GMB-47-01-e20230068-s1.pdf]

Genetics and Molecular Biology
https://doi.org/10.1590/1678-4685-GMB-2023-0068

Supplementary Material to “Structure-based prediction of protein-protein interaction network in rice”

Table S1. List of 1,122 benchmark interactions with experimental structures.

Protein chain 1	Protein chain 2	Source
1bdj_A	1bdj_B	dockground
1cc0_E	1cc0_A	dockground
1dfj_I	1dfj_E	dockground
1ewy_A	1ewy_C	dockground
1fle_E	1fle_I	dockground
1fq1_B	1fq1_A	dockground
1g6v_A	1g6v_K	dockground
1gcq_C	1gcq_A	dockground
1gg2_A	1gg2_B	dockground
1gpw_A	1gpw_B	dockground
1i2m_B	1i2m_A	dockground
1i8l_A	1i8l_C	dockground
1ib1_B	1ib1_F	dockground
1jk9_B	1jk9_A	dockground
1jtd_B	1jtd_A	dockground
1k5g_C	1k5g_A	dockground
1kgy_A	1kgy_H	dockground
1lb2_A	1lb2_B	dockground
1lfd_B	1lfd_A	dockground
1mq8_A	1mq8_B	dockground
1nbf_A	1nbf_D	dockground
1nql_A	1nql_B	dockground
1nw9_B	1nw9_A	dockground
1oc0_A	1oc0_B	dockground
1pvh_A	1pvh_B	dockground
1qav_B	1qav_A	dockground
1r8s_E	1r8s_A	dockground
1s1q_A	1s1q_B	dockground
1te1_A	1te1_B	dockground
1tmq_A	1tmq_B	dockground
1uad_A	1uad_C	dockground
1v5i_A	1v5i_B	dockground
1vg0_A	1vg0_B	dockground

Protein chain 1	Protein chain 2	Source
1wq1_G	1wq1_R	dockground
1wrd_A	1wrd_B	dockground
1x86_A	1x86_B	dockground
1xt9_A	1xt9_B	dockground
1y64_B	1y64_A	dockground
1y8x_A	1y8x_B	dockground
1ycs_B	1ycs_A	dockground
1yu6_A	1yu6_C	dockground
1yvb_A	1yvb_I	dockground
2a41_A	2a41_B	dockground
2a5d_B	2a5d_A	dockground
2a9k_B	2a9k_A	dockground
2aq3_H	2aq3_G	dockground
2b4s_D	2b4s_C	dockground
2bcg_G	2bcg_Y	dockground
2bcn_C	2bcn_B	dockground
2bku_B	2bku_A	dockground
2bov_B	2bov_A	dockground
2bwe_T	2bwe_D	dockground
2c2v_B	2c2v_C	dockground
2ch4_A	2ch4_W	dockground
2ejf_A	2ejf_C	dockground
2g45_A	2g45_B	dockground
2grx_A	2grx_C	dockground
2hj9_B	2hj9_C	dockground
2hqs_A	2hqs_H	dockground
2hrk_A	2hrk_B	dockground
2i25_L	2i25_N	dockground
2iwt_B	2iwt_A	dockground
2nxn_A	2nxn_B	dockground
2o25_A	2o25_D	dockground
2o2v_A	2o2v_B	dockground
2o8v_A	2o8v_B	dockground
2oob_B	2oob_A	dockground
2qyi_C	2qyi_D	dockground
2uy7_A	2uy7_B	dockground
2v55_A	2v55_B	dockground
2vrr_A	2vrr_B	dockground
2wbw_A	2wbw_B	dockground
2x0b_F	2x0b_E	dockground
2x9a_D	2x9a_C	dockground
2xgy_B	2xgy_A	dockground
2xwb_F	2xwb_J	dockground

Protein chain 1	Protein chain 2	Source
3a1p_A	3a1p_B	dockground
3a8i_A	3a8i_E	dockground
3av0_A	3av0_B	dockground
3bh6_B	3bh6_A	dockground
3bs5_A	3bs5_B	dockground
3buz_A	3buz_B	dockground
3bx1_A	3bx1_C	dockground
3cu1_D	3cu1_C	dockground
3d3c_A	3d3c_J	dockground
3daw_A	3daw_B	dockground
3e2l_A	3e2l_C	dockground
3f1p_B	3f1p_A	dockground
3f7p_A	3f7p_C	dockground
3fap_A	3fap_B	dockground
3fn1_B	3fn1_A	dockground
3g3a_A	3g3a_B	dockground
3k1i_D	3k1i_A	dockground
3k9m_A	3k9m_C	dockground
3kld_A	3kld_B	dockground
3l1z_A	3l1z_B	dockground
3lb8_A	3lb8_C	dockground
3m18_A	3m18_B	dockground
3mj7_A	3mj7_B	dockground
3mzw_A	3mzw_B	dockground
3oed_A	3oed_C	dockground
3oj4_A	3oj4_B	dockground
3ong_B	3ong_C	dockground
3oun_B	3oun_A	dockground
3prp_A	3prp_B	dockground
3pv6_A	3pv6_B	dockground
3qc8_A	3qc8_B	dockground
3qlu_C	3qlu_A	dockground
3rj3_A	3rj3_D	dockground
3rnk_A	3rnk_B	dockground
3t5g_A	3t5g_B	dockground
3tg1_A	3tg1_B	dockground
3ulq_A	3ulq_B	dockground
3vlb_A	3vlb_B	dockground
3wkt_A	3wkt_C	dockground
4bbn_A	4bbn_F	dockground
4bmp_A	4bmp_B	dockground
4bos_A	4bos_E	dockground
4boz_A	4boz_B	dockground

Protein chain 1	Protein chain 2	Source
4c0o_A	4c0o_C	dockground
4c6t_B	4c6t_A	dockground
4ct4_B	4ct4_A	dockground
4emj_A	4emj_B	dockground
4etw_A	4etw_B	dockground
4f7g_A	4f7g_B	dockground
4ged_A	4ged_B	dockground
4gmj_B	4gmj_A	dockground
4j4l_A	4j4l_C	dockground
4jhp_C	4jhp_B	dockground
4jqw_A	4jqw_C	dockground
4jx1_A	4jx1_C	dockground
4k0v_A	4k0v_B	dockground
4kyi_A	4kyi_B	dockground
4l41_C	4l41_A	dockground
4llo_A	4llo_B	dockground
4lsx_A	4lsx_C	dockground
4m3k_A	4m3k_B	dockground
4mdk_A	4mdk_E	dockground
4mrt_A	4mrt_C	dockground
4n6o_A	4n6o_B	dockground
4nyi_S	4nyi_Q	dockground
4nzl_A	4nzl_B	dockground
4ol0_B	4ol0_A	dockground
4p2a_A	4p2a_B	dockground
4p4q_A	4p4q_B	dockground
4pou_A	4pou_B	dockground
4pw9_A	4pw9_B	dockground
4r62_A	4r62_B	dockground
4rf0_A	4rf0_B	dockground
4rix_B	4rix_A	dockground
4rws_A	4rws_C	dockground
4s10_A	4s10_C	dockground
4txo_C	4txo_D	dockground
4txv_A	4txv_B	dockground
4v2c_A	4v2c_B	dockground
4wm0_A	4wm0_D	dockground
4x6q_B	4x6q_C	dockground
4xs0_B	4xs0_A	dockground
4y5o_B	4y5o_A	dockground
4yeb_B	4yeb_A	dockground
4yoc_A	4yoc_C	dockground
4zfr_A	4zfr_B	dockground

Protein chain 1	Protein chain 2	Source
5bmu_B	5bmu_A	dockground
5brr_I	5brr_E	dockground
5cec_A	5cec_B	dockground
5eg3_A	5eg3_B	dockground
3cbk_A	3cbk_B	dockground
4lx0_B	4lx0_A	dockground
2c0l_A	2c0l_B	dockground
2nqd_B	2nqd_A	dockground
3cw2_A	3cw2_K	dockground
1a02_F	1a02_J	gwidd
1a12_A	1a12_C	gwidd
1a1u_A	1a1u_C	gwidd
1a4i_B	1a4i_A	gwidd
1a4y_D	1a4y_E	gwidd
1a6a_B	1a6a_A	gwidd
1a7x_A	1a7x_B	gwidd
1am4_C	1am4_F	gwidd
1au1_A	1au1_B	gwidd
1aui_A	1aui_B	gwidd
1b3a_A	1b3a_B	gwidd
1b6c_D	1b6c_C	gwidd
1b8m_B	1b8m_A	gwidd
1b9e_B	1b9e_D	gwidd
1bda_A	1bda_B	gwidd
1beh_A	1beh_B	gwidd
1bft_A	1bft_B	gwidd
1bhx_B	1bhx_F	gwidd
1bi8_A	1bi8_B	gwidd
1bmo_A	1bmo_B	gwidd
1bo1_A	1bo1_B	gwidd
1bre_C	1bre_D	gwidd
1bx2_E	1bx2_D	gwidd
1c1y_A	1c1y_B	gwidd
1c9b_Q	1c9b_R	gwidd
1ca9_D	1ca9_F	gwidd
1cbl_B	1cbl_C	gwidd
1cee_B	1cee_A	gwidd
1cm8_A	1cm8_B	gwidd
1cnt_1	1cnt_4	gwidd
1cp3_A	1cp3_B	gwidd
1csb_B	1csb_A	gwidd
1czz_A	1czz_B	gwidd
1d1j_D	1d1j_B	gwidd

Protein chain 1	Protein chain 2	Source
1d1s_B	1d1s_D	gwidd
1d1z_D	1d1z_B	gwidd
1d2v_D	1d2v_B	gwidd
1d4a_B	1d4a_D	gwidd
1d4v_B	1d4v_A	gwidd
1d5z_B	1d5z_A	gwidd
1dan_U	1dan_T	gwidd
1dd1_C	1dd1_A	gwidd
1dfv_B	1dfv_A	gwidd
1djs_A	1djs_B	gwidd
1dle_A	1dle_B	gwidd
1do5_D	1do5_B	gwidd
1dok_A	1dok_B	gwidd
1dux_C	1dux_F	gwidd
1dz1_A	1dz1_B	gwidd
1e51_A	1e51_B	gwidd
1eaj_A	1eaj_B	gwidd
1edm_B	1edm_C	gwidd
1ef7_A	1ef7_B	gwidd
1efv_A	1efv_B	gwidd
1efx_D	1efx_E	gwidd
1ejf_A	1ejf_B	gwidd
1ek6_A	1ek6_B	gwidd
1ern_B	1ern_A	gwidd
1evu_B	1evu_A	gwidd
1ext_A	1ext_B	gwidd
1f05_A	1f05_B	gwidd
1f4j_A	1f4j_D	gwidd
1f66_C	1f66_F	gwidd
1f6a_B	1f6a_D	gwidd
1f9q_D	1f9q_A	gwidd
1fb1_A	1fb1_E	gwidd
1fbv_A	1fbv_C	gwidd
1fdp_A	1fdp_D	gwidd
1fe0_B	1fe0_A	gwidd
1fgu_A	1fgu_B	gwidd
1fj2_A	1fj2_B	gwidd
1fl7_C	1fl7_A	gwidd
1flt_V	1flt_Y	gwidd
1fos_E	1fos_F	gwidd
1fq3_A	1fq3_B	gwidd
1fqv_M	1fqv_O	gwidd
1fv1_E	1fv1_D	gwidd

Protein chain 1	Protein chain 2	Source
1fzd_E	1fzd_H	gwidd
1g82_B	1g82_C	gwidd
1g83_A	1g83_B	gwidd
1gjz_A	1gjz_B	gwidd
1gri_A	1gri_B	gwidd
1grn_B	1grn_A	gwidd
1gvj_B	1gvj_A	gwidd
1gwb_B	1gwb_A	gwidd
1gxc_A	1gxc_D	gwidd
1h1b_A	1h1b_B	gwidd
1h3i_A	1h3i_B	gwidd
1h4r_A	1h4r_B	gwidd
1h88_B	1h88_A	gwidd
1h9u_C	1h9u_D	gwidd
1ha4_A	1ha4_B	gwidd
1hcf_A	1hcf_B	gwidd
1hci_A	1hci_B	gwidd
1hcn_B	1hcn_A	gwidd
1hdy_A	1hdy_B	gwidd
1hkx_B	1hkx_N	gwidd
1hlc_A	1hlc_B	gwidd
1hsa_A	1hsa_D	gwidd
1hvv_A	1hvv_C	gwidd
1hwg_B	1hwg_A	gwidd
1hy7_B	1hy7_A	gwidd
1hyr_C	1hyr_A	gwidd
1hzd_B	1hzd_F	gwidd
1hzw_A	1hzw_B	gwidd
1i0z_A	1i0z_B	gwidd
1i1r_A	1i1r_B	gwidd
1i49_A	1i49_B	gwidd
1i4l_A	1i4l_D	gwidd
1i4o_B	1i4o_D	gwidd
1i8l_A	1i8l_B	gwidd
1ibr_D	1ibr_C	gwidd
1icf_A	1icf_I	gwidd
1ilr_1	1ilr_2	gwidd
1im9_A	1im9_D	gwidd
1ira_Y	1ira_X	gwidd
1irj_H	1irj_G	gwidd
1itu_A	1itu_B	gwidd
1iu1_B	1iu1_A	gwidd
1ivh_A	1ivh_D	gwidd

Protein chain 1	Protein chain 2	Source
1ivo_A	1ivo_B	gwidd
1ivy_A	1ivy_B	gwidd
1j1b_B	1j1b_A	gwidd
1j1j_A	1j1j_D	gwidd
1j88_B	1j88_D	gwidd
1j96_A	1j96_B	gwidd
1jdp_A	1jdp_B	gwidd
1jeq_A	1jeq_B	gwidd
1jfi_B	1jfi_A	gwidd
1jiq_C	1jiq_D	gwidd
1jk8_B	1jk8_A	gwidd
1jkg_B	1jkg_A	gwidd
1jm7_A	1jm7_B	gwidd
1jmj_A	1jmj_B	gwidd
1jnm_B	1jnm_A	gwidd
1joc_A	1joc_B	gwidd
1jpy_Y	1jpy_X	gwidd
1jr2_A	1jr2_B	gwidd
1juo_A	1juo_B	gwidd
1juq_D	1juq_A	gwidd
1jvg_A	1jvg_B	gwidd
1jwm_D	1jwm_A	gwidd
1jws_D	1jws_A	gwidd
1k3y_A	1k3y_B	gwidd
1k5d_A	1k5d_B	gwidd
1k7l_C	1k7l_G	gwidd
1k8f_C	1k8f_D	gwidd
1k8r_A	1k8r_B	gwidd
1k94_A	1k94_B	gwidd
1k9i_A	1k9i_B	gwidd
1k9k_A	1k9k_B	gwidd
1kfu_L	1kfu_S	gwidd
1kgc_E	1kgc_D	gwidd
1khu_A	1khu_C	gwidd
1ki1_B	1ki1_A	gwidd
1kli_H	1kli_L	gwidd
1kqn_A	1kqn_E	gwidd
1kw2_A	1kw2_B	gwidd
1kxg_A	1kxg_F	gwidd
1l1f_A	1l1f_F	gwidd
1l8l_A	1l8l_B	gwidd
1l9x_B	1l9x_D	gwidd
1lgq_A	1lgq_B	gwidd

Protein chain 1	Protein chain 2	Source
1ljr_A	1ljr_B	gwidd
1lph_B	1lph_D	gwidd
1lqf_A	1lqf_D	gwidd
1lzs_A	1lzs_B	gwidd
1m0z_B	1m0z_A	gwidd
1m4r_A	1m4r_B	gwidd
1m4u_A	1m4u_L	gwidd
1m63_E	1m63_G	gwidd
1mhq_B	1mhq_A	gwidd
1mhw_B	1mhw_D	gwidd
1mif_A	1mif_C	gwidd
1mq8_A	1mq8_C	gwidd
1mr8_A	1mr8_B	gwidd
1msg_A	1msg_B	gwidd
1n45_A	1n45_B	gwidd
1n46_A	1n46_B	gwidd
1n7s_B	1n7s_A	gwidd
1n9j_A	1n9j_B	gwidd
1ncn_A	1ncn_B	gwidd
1ncv_A	1ncv_B	gwidd
1nd6_A	1nd6_D	gwidd
1ndd_A	1ndd_D	gwidd
1ne7_A	1ne7_D	gwidd
1nf7_A	1nf7_B	gwidd
1nmk_A	1nmk_B	gwidd
1nn8_S	1nn8_T	gwidd
1nr4_G	1nr4_H	gwidd
1nun_B	1nun_A	gwidd
1nup_A	1nup_B	gwidd
1nwq_A	1nwq_C	gwidd
1nxk_A	1nxk_D	gwidd
1nyu_A	1nyu_B	gwidd
1nzi_B	1nzi_A	gwidd
1o80_A	1o80_B	gwidd
1oa8_D	1oa8_C	gwidd
1oiv_A	1oiv_B	gwidd
1ok3_A	1ok3_B	gwidd
1oki_A	1oki_B	gwidd
1olg_A	1olg_C	gwidd
1oni_I	1oni_G	gwidd
1oo9_A	1oo9_B	gwidd
1ov3_A	1ov3_B	gwidd
1ovz_A	1ovz_B	gwidd

Protein chain 1	Protein chain 2	Source
1p53_A	1p53_B	gwidd
1p60_A	1p60_B	gwidd
1p7h_N	1p7h_O	gwidd
1pbw_B	1pbw_A	gwidd
1pe3_1	1pe3_2	gwidd
1pew_A	1pew_B	gwidd
1pfq_A	1pfq_B	gwidd
1pkx_B	1pkx_A	gwidd
1pl8_A	1pl8_D	gwidd
1psr_A	1psr_B	gwidd
1pvh_C	1pvh_D	gwidd
1pyo_C	1pyo_B	gwidd
1q7l_A	1q7l_C	gwidd
1q7s_A	1q7s_B	gwidd
1qdv_A	1qdv_D	gwidd
1qiz_B	1qiz_D	gwidd
1qja_B	1qja_A	gwidd
1qk1_B	1qk1_H	gwidd
1qku_B	1qku_C	gwidd
1qmv_G	1qmv_J	gwidd
1qnk_A	1qnk_B	gwidd
1qo5_Q	1qo5_R	gwidd
1qr6_A	1qr6_B	gwidd
1qtn_A	1qtn_B	gwidd
1qwt_A	1qwt_B	gwidd
1r0d_A	1r0d_I	gwidd
1r4c_A	1r4c_H	gwidd
1r74_B	1r74_A	gwidd
1rhg_A	1rhg_C	gwidd
1rj7_F	1rj7_M	gwidd
1rk4_A	1rk4_B	gwidd
1rnf_A	1rnf_B	gwidd
1ros_A	1ros_B	gwidd
1rrg_A	1rrg_B	gwidd
1rv6_W	1rv6_V	gwidd
1rwp_A	1rwp_B	gwidd
1ryh_A	1ryh_B	gwidd
1s3k_L	1s3k_H	gwidd
1s95_B	1s95_A	gwidd
1s9i_A	1s9i_B	gwidd
1saw_B	1saw_A	gwidd
1sl6_B	1sl6_F	gwidd
1smo_A	1smo_B	gwidd

Protein chain 1	Protein chain 2	Source
1sqn_B	1sqn_A	gwidd
1szb_A	1szb_B	gwidd
1t0l_A	1t0l_B	gwidd
1t2k_C	1t2k_D	gwidd
1t3g_A	1t3g_B	gwidd
1t3n_A	1t3n_B	gwidd
1t64_A	1t64_B	gwidd
1t6n_A	1t6n_B	gwidd
1t8t_B	1t8t_A	gwidd
1tgz_A	1tgz_B	gwidd
1tnr_A	1tnr_R	gwidd
1tu4_A	1tu4_B	gwidd
1tu6_A	1tu6_B	gwidd
1tvd_A	1tvd_B	gwidd
1u6a_H	1u6a_L	gwidd
1u8f_O	1u8f_R	gwidd
1uj2_A	1uj2_B	gwidd
1ul1_Z	1ul1_C	gwidd
1ur6_A	1ur6_B	gwidd
1v5w_A	1v5w_B	gwidd
1v84_A	1v84_B	gwidd
1vf6_B	1vf6_A	gwidd
1vsc_A	1vsc_B	gwidd
1vyj_A	1vyj_C	gwidd
1w4r_B	1w4r_H	gwidd
1w4v_A	1w4v_F	gwidd
1w6u_A	1w6u_D	gwidd
1w8p_B	1w8p_D	gwidd
1w98_A	1w98_B	gwidd
1w9e_A	1w9e_B	gwidd
1wdz_A	1wdz_B	gwidd
1wio_A	1wio_B	gwidd
1wku_B	1wku_A	gwidd
1wr6_C	1wr6_G	gwidd
1wtl_A	1wtl_B	gwidd
1wwa_X	1wwa_Y	gwidd
1wyw_A	1wyw_B	gwidd
1x27_A	1x27_F	gwidd
1x79_B	1x79_A	gwidd
1xd3_A	1xd3_B	gwidd
1xdc_A	1xdc_B	gwidd
1xfb_A	1xfb_I	gwidd
1xfd_C	1xfd_D	gwidd

Protein chain 1	Protein chain 2	Source
1xg5_D	1xg5_A	gwidd
1xiw_E	1xiw_F	gwidd
1xka_C	1xka_L	gwidd
1xmm_D	1xmm_B	gwidd
1xox_A	1xox_B	gwidd
1xvp_D	1xvp_C	gwidd
1xw5_A	1xw5_B	gwidd
1xw6_A	1xw6_B	gwidd
1y2k_A	1y2k_B	gwidd
1y2o_A	1y2o_B	gwidd
1y4j_B	1y4j_A	gwidd
1y6l_A	1y6l_B	gwidd
1y8o_A	1y8o_B	gwidd
1y97_A	1y97_B	gwidd
1yc0_A	1yc0_I	gwidd
1yi6_A	1yi6_B	gwidd
1yov_B	1yov_D	gwidd
1ypq_B	1ypq_A	gwidd
1yrp_A	1yrp_B	gwidd
1yvl_B	1yvl_A	gwidd
1yxu_B	1yxu_C	gwidd
1yz5_B	1yz5_A	gwidd
1z00_A	1z00_B	gwidd
1z0a_A	1z0a_D	gwidd
1z68_A	1z68_B	gwidd
1z6u_A	1z6u_B	gwidd
1z7x_W	1z7x_X	gwidd
1zag_B	1zag_D	gwidd
1zbq_E	1zbq_F	gwidd
1zdn_A	1zdn_B	gwidd
1zei_D	1zei_F	gwidd
1zjd_A	1zjd_B	gwidd
1zkc_A	1zkc_B	gwidd
1zmd_A	1zmd_F	gwidd
1zmu_B	1zmu_A	gwidd
1zs6_A	1zs6_B	gwidd
1zsv_A	1zsv_D	gwidd
1zt4_C	1zt4_A	gwidd
1zum_A	1zum_D	gwidd
1zxm_B	1zxm_A	gwidd
1zy8_B	1zy8_J	gwidd
1zzj_A	1zzj_C	gwidd
2a07_I	2a07_H	gwidd

Protein chain 1	Protein chain 2	Source
2a1h_A	2a1h_B	gwidd
2a1s_D	2a1s_A	gwidd
2a1u_A	2a1u_B	gwidd
2a24_B	2a24_A	gwidd
2a2r_A	2a2r_B	gwidd
2a72_A	2a72_B	gwidd
2a7l_A	2a7l_B	gwidd
2ach_A	2ach_B	gwidd
2aew_A	2aew_B	gwidd
2afs_A	2afs_B	gwidd
2afw_A	2afw_B	gwidd
2ag5_A	2ag5_D	gwidd
2ajp_A	2ajp_B	gwidd
2akz_A	2akz_B	gwidd
2aw2_X	2aw2_Y	gwidd
2ayo_A	2ayo_B	gwidd
2b0u_D	2b0u_A	gwidd
2b5g_A	2b5g_B	gwidd
2b8w_B	2b8w_A	gwidd
2bew_A	2bew_B	gwidd
2bhl_A	2bhl_B	gwidd
2bjn_B	2bjn_A	gwidd
2bp1_A	2bp1_B	gwidd
2bsk_A	2bsk_B	gwidd
2btp_A	2btp_B	gwidd
2bwg_A	2bwg_B	gwidd
2c10_D	2c10_C	gwidd
2c4k_D	2c4k_F	gwidd
2c62_A	2c62_B	gwidd
2c63_A	2c63_C	gwidd
2c6t_A	2c6t_C	gwidd
2c9o_A	2c9o_B	gwidd
2c9v_A	2c9v_F	gwidd
2car_A	2car_B	gwidd
2cfh_B	2cfh_D	gwidd
2ch5_A	2ch5_C	gwidd
2ckl_A	2ckl_B	gwidd
2coi_A	2coi_B	gwidd
2cv5_D	2cv5_B	gwidd
2cyx_A	2cyx_B	gwidd
2d07_A	2d07_B	gwidd
2d1x_C	2d1x_A	gwidd
2ddk_A	2ddk_B	gwidd

Protein chain 1	Protein chain 2	Source
2den_B	2den_A	gwidd
2djf_B	2djf_C	gwidd
2dn3_B	2dn3_A	gwidd
2dsq_G	2dsq_I	gwidd
2dvs_A	2dvs_B	gwidd
2e8j_A	2e8j_B	gwidd
2egd_B	2egd_A	gwidd
2ela_B	2ela_A	gwidd
2esg_C	2esg_A	gwidd
2ewp_A	2ewp_B	gwidd
2fb8_A	2fb8_B	gwidd
2fd6_U	2fd6_A	gwidd
2fg8_A	2fg8_D	gwidd
2fpe_C	2fpe_H	gwidd
2fu3_B	2fu3_A	gwidd
2fze_A	2fze_B	gwidd
2g45_D	2g45_E	gwidd
2ger_A	2ger_E	gwidd
2gf2_A	2gf2_D	gwidd
2gh0_B	2gh0_D	gwidd
2goo_E	2goo_B	gwidd
2h6f_B	2h6f_A	gwidd
2h7c_A	2h7c_F	gwidd
2h9e_H	2h9e_L	gwidd
2hdp_A	2hdp_B	gwidd
2hhj_A	2hhj_B	gwidd
2hth_B	2hth_A	gwidd
2hvd_A	2hvd_C	gwidd
2hxy_C	2hxy_D	gwidd
2i0e_A	2i0e_B	gwidd
2i1y_B	2i1y_A	gwidd
2iby_D	2iby_A	gwidd
2ihc_C	2ihc_D	gwidd
2il8_A	2il8_B	gwidd
2iyd_A	2iyd_B	gwidd
2j0t_C	2j0t_F	gwidd
2j6k_I	2j6k_J	gwidd
2jbh_B	2jbh_A	gwidd
2jbm_G	2jbm_L	gwidd
2jg8_B	2jg8_C	gwidd
2jgz_A	2jgz_B	gwidd
2k4a_B	2k4a_A	gwidd
2k6d_B	2k6d_A	gwidd

Protein chain 1	Protein chain 2	Source
2k8m_A	2k8m_B	gwidd
2khw_B	2khw_A	gwidd
2ki6_E	2ki6_D	gwidd
2kjh_A	2kjh_B	gwidd
2knv_A	2knv_B	gwidd
2kz1_B	2kz1_A	gwidd
2l0t_B	2l0t_A	gwidd
2l5x_A	2l5x_B	gwidd
2lp2_A	2lp2_B	gwidd
2luc_A	2luc_B	gwidd
2lvo_A	2lvo_C	gwidd
2lxp_A	2lxp_C	gwidd
2mcn_B	2mcn_A	gwidd
2mej_B	2mej_A	gwidd
2mj5_A	2mj5_B	gwidd
2nn2_A	2nn2_B	gwidd
2nps_A	2nps_D	gwidd
2nte_A	2nte_B	gwidd
2nz7_B	2nz7_A	gwidd
2o07_B	2o07_A	gwidd
2o28_A	2o28_B	gwidd
2o53_A	2o53_B	gwidd
2o61_A	2o61_B	gwidd
2oat_A	2oat_B	gwidd
2ofv_A	2ofv_B	gwidd
2ojw_A	2ojw_E	gwidd
2om2_C	2om2_D	gwidd
2onl_A	2onl_C	gwidd
2oo0_A	2oo0_B	gwidd
2opz_B	2opz_D	gwidd
2oq0_B	2oq0_D	gwidd
2p28_B	2p28_A	gwidd
2p4y_A	2p4y_B	gwidd
2p5x_A	2p5x_B	gwidd
2p6x_A	2p6x_B	gwidd
2pab_A	2pab_B	gwidd
2pah_A	2pah_B	gwidd
2pkd_D	2pkd_F	gwidd
2pru_A	2pru_B	gwidd
2px9_A	2px9_B	gwidd
2pzd_A	2pzd_B	gwidd
2q20_B	2q20_A	gwidd
2q4g_W	2q4g_Y	gwidd

Protein chain 1	Protein chain 2	Source
2q4v_B	2q4v_A	gwidd
2q5d_A	2q5d_B	gwidd
2q7d_A	2q7d_B	gwidd
2q80_B	2q80_F	gwidd
2q81_A	2q81_D	gwidd
2q91_A	2q91_B	gwidd
2qag_B	2qag_A	gwidd
2qj2_B	2qj2_A	gwidd
2qjf_B	2qjf_A	gwidd
2qm4_B	2qm4_D	gwidd
2qms_A	2qms_D	gwidd
2qrz_A	2qrz_B	gwidd
2quh_B	2quh_A	gwidd
2qy0_B	2qy0_A	gwidd
2qy7_A	2qy7_C	gwidd
2qyn_A	2qyn_B	gwidd
2r1u_A	2r1u_B	gwidd
2r2q_A	2r2q_B	gwidd
2r3v_A	2r3v_C	gwidd
2r8u_B	2r8u_A	gwidd
2rie_A	2rie_C	gwidd
2rjc_A	2rjc_C	gwidd
2rkb_A	2rkb_E	gwidd
2uvl_B	2uvl_A	gwidd
2uzk_A	2uzk_C	gwidd
2uzp_A	2uzp_C	gwidd
2v5z_A	2v5z_B	gwidd
2v66_B	2v66_E	gwidd
2v76_A	2v76_D	gwidd
2vje_D	2vje_C	gwidd
2vjf_A	2vjf_B	gwidd
2vo1_A	2vo1_B	gwidd
2vwi_D	2vwi_C	gwidd
2vxs_C	2vxs_D	gwidd
2vyi_A	2vyi_B	gwidd
2w1o_A	2w1o_B	gwidd
2w2c_A	2w2c_K	gwidd
2w2m_A	2w2m_E	gwidd
2wcb_A	2wcb_B	gwidd
2wdp_C	2wdp_A	gwidd
2wel_A	2wel_D	gwidd
2wk6_A	2wk6_B	gwidd
2wma_C	2wma_D	gwidd

Protein chain 1	Protein chain 2	Source
2wwp_A	2wwp_B	gwidd
2wwz_B	2wwz_C	gwidd
2x2f_A	2x2f_D	gwidd
2x36_A	2x36_F	gwidd
2x69_B	2x69_E	gwidd
2x6g_A	2x6g_O	gwidd
2x6l_E	2x6l_B	gwidd
2x7o_A	2x7o_B	gwidd
2xa0_A	2xa0_B	gwidd
2xa6_A	2xa6_B	gwidd
2xb1_C	2xb1_A	gwidd
2xre_A	2xre_B	gwidd
2xrf_B	2xrf_C	gwidd
2xsz_A	2xsz_F	gwidd
2xv5_A	2xv5_B	gwidd
2xwr_B	2xwr_A	gwidd
2xwu_B	2xwu_A	gwidd
2y1n_C	2y1n_A	gwidd
2y43_A	2y43_B	gwidd
2y6e_A	2y6e_F	gwidd
2yan_A	2yan_B	gwidd
2ygd_A	2ygd_Q	gwidd
2yho_E	2yho_G	gwidd
2yin_D	2yin_C	gwidd
2yvr_A	2yvr_B	gwidd
2z0v_A	2z0v_B	gwidd
2zeh_A	2zeh_B	gwidd
2zfh_A	2zfh_C	gwidd
2zjd_C	2zjd_A	gwidd
2zmv_B	2zmv_A	gwidd
2zrt_A	2zrt_B	gwidd
3a4s_A	3a4s_B	gwidd
3a6n_A	3a6n_B	gwidd
3a8y_B	3a8y_D	gwidd
3aih_B	3aih_A	gwidd
3aln_A	3aln_B	gwidd
3alq_T	3alq_W	gwidd
3an2_A	3an2_E	gwidd
3b2d_B	3b2d_D	gwidd
3b76_B	3b76_A	gwidd
3bbb_F	3bbb_C	gwidd
3bdw_C	3bdw_D	gwidd
3bg1_A	3bg1_E	gwidd

Protein chain 1	Protein chain 2	Source
3bhh_A	3bhh_C	gwidd
3bji_B	3bji_C	gwidd
3bqp_A	3bqp_B	gwidd
3bsz_F	3bsz_D	gwidd
3c0h_A	3c0h_B	gwidd
3c10_A	3c10_C	gwidd
3cdg_C	3cdg_E	gwidd
3cf2_A	3cf2_B	gwidd
3cog_D	3cog_A	gwidd
3ctb_A	3ctb_B	gwidd
3cu0_A	3cu0_B	gwidd
3cuq_A	3cuq_B	gwidd
3cwg_B	3cwg_A	gwidd
3d3k_B	3d3k_D	gwidd
3d3w_B	3d3w_A	gwidd
3d4j_A	3d4j_B	gwidd
3d9t_B	3d9t_A	gwidd
3dds_A	3dds_B	gwidd
3ddt_A	3ddt_B	gwidd
3dkb_C	3dkb_F	gwidd
3drx_E	3drx_A	gwidd
3ds6_C	3ds6_A	gwidd
3dww_B	3dww_C	gwidd
3dxb_A	3dxb_H	gwidd
3dxe_C	3dxe_A	gwidd
3dy0_A	3dy0_B	gwidd
3dyn_A	3dyn_B	gwidd
3dyu_A	3dyu_B	gwidd
3dzy_D	3dzy_A	gwidd
3e1i_C	3e1i_F	gwidd
3e6p_H	3e6p_L	gwidd
3eb6_B	3eb6_A	gwidd
3ecs_C	3ecs_A	gwidd
3egg_B	3egg_D	gwidd
3eo1_I	3eo1_L	gwidd
3eu9_A	3eu9_C	gwidd
3f2k_A	3f2k_B	gwidd
3f3y_A	3f3y_B	gwidd
3f5o_A	3f5o_D	gwidd
3f6u_H	3f6u_L	gwidd
3fby_A	3fby_C	gwidd
3fd5_B	3fd5_A	gwidd
3fe2_A	3fe2_B	gwidd

Protein chain 1	Protein chain 2	Source
3fqh_A	3fqh_B	gwidd
3fwq_A	3fwq_B	gwidd
3fxi_A	3fxi_B	gwidd
3g2s_A	3g2s_B	gwidd
3gg3_A	3gg3_B	gwidd
3ggf_A	3ggf_B	gwidd
3gj0_A	3gj0_B	gwidd
3gmh_I	3gmh_H	gwidd
3gr4_A	3gr4_C	gwidd
3gtu_D	3gtu_C	gwidd
3gxu_A	3gxu_B	gwidd
3gzn_C	3gzn_J	gwidd
3h0e_B	3h0e_A	gwidd
3h6g_B	3h6g_A	gwidd
3h8v_A	3h8v_B	gwidd
3h91_A	3h91_B	gwidd
3haj_A	3haj_B	gwidd
3hcn_A	3hcn_B	gwidd
3hcs_A	3hcs_B	gwidd
3hcu_B	3hcu_D	gwidd
3hei_O	3hei_P	gwidd
3hf1_B	3hf1_A	gwidd
3hh2_A	3hh2_B	gwidd
3hj2_A	3hj2_B	gwidd
3hn3_A	3hn3_E	gwidd
3hnc_B	3hnc_A	gwidd
3hup_A	3hup_B	gwidd
3i2b_L	3i2b_J	gwidd
3i7i_A	3i7i_B	gwidd
3i90_A	3i90_B	gwidd
3i91_A	3i91_B	gwidd
3ig6_B	3ig6_D	gwidd
3ihl_A	3ihl_B	gwidd
3ihy_A	3ihy_B	gwidd
3ik7_A	3ik7_D	gwidd
3ikk_A	3ikk_B	gwidd
3ikm_F	3ikm_E	gwidd
3iol_A	3iol_B	gwidd
3ixe_A	3ixe_B	gwidd
3j63_B	3j63_C	gwidd
3j7o_H	3j7o_m	gwidd
3jqz_A	3jqz_B	gwidd
3jv5_C	3jv5_D	gwidd

Protein chain 1	Protein chain 2	Source
3jzq_B	3jzq_A	gwidd
3k1x_A	3k1x_E	gwidd
3k2a_B	3k2a_A	gwidd
3k2o_B	3k2o_A	gwidd
3k2s_A	3k2s_B	gwidd
3k9m_B	3k9m_D	gwidd
3kbh_A	3kbh_C	gwidd
3km0_A	3km0_B	gwidd
3kw5_A	3kw5_B	gwidd
3l5j_B	3l5j_A	gwidd
3lcp_B	3lcp_D	gwidd
3ldq_A	3ldq_B	gwidd
3ldz_C	3ldz_F	gwidd
3ljb_A	3ljb_B	gwidd
3llh_B	3llh_A	gwidd
3lmy_B	3lmy_A	gwidd
3lrq_D	3lrq_A	gwidd
3lwe_A	3lwe_B	gwidd
3mi9_A	3mi9_B	gwidd
3mjg_B	3mjg_A	gwidd
3mjk_Y	3mjk_X	gwidd
3mtr_A	3mtr_B	gwidd
3mts_B	3mts_C	gwidd
3n80_E	3n80_H	gwidd
3n9y_B	3n9y_D	gwidd
3nar_A	3nar_B	gwidd
3nau_A	3nau_B	gwidd
3nay_A	3nay_B	gwidd
3ncj_H	3ncj_L	gwidd
3nhe_A	3nhe_B	gwidd
3nj4_C	3nj4_D	gwidd
3nso_A	3nso_B	gwidd
3nvq_A	3nvq_E	gwidd
3nyn_A	3nyn_B	gwidd
3o5z_A	3o5z_B	gwidd
3o65_G	3o65_H	gwidd
3o78_A	3o78_B	gwidd
3o8e_B	3o8e_D	gwidd
3odo_A	3odo_B	gwidd
3ogj_B	3ogj_D	gwidd
3ojy_A	3ojy_C	gwidd
3oll_A	3oll_B	gwidd
3omv_A	3omv_B	gwidd

Protein chain 1	Protein chain 2	Source
3oq9_H	3oq9_L	gwidd
3ovp_B	3ovp_A	gwidd
3p08_B	3p08_A	gwidd
3pgc_B	3pgc_E	gwidd
3pja_L	3pja_K	gwidd
3pos_B	3pos_A	gwidd
3ptz_A	3ptz_E	gwidd
3pv1_A	3pv1_B	gwidd
3pvn_A	3pvn_T	gwidd
3q01_A	3q01_B	gwidd
3q5e_C	3q5e_G	gwidd
3q84_N	3q84_M	gwidd
3q91_D	3q91_C	gwidd
3qb5_K	3qb5_C	gwidd
3qbt_G	3qbt_H	gwidd
3r27_A	3r27_B	gwidd
3r3i_A	3r3i_C	gwidd
3r3m_A	3r3m_B	gwidd
3r9a_A	3r9a_B	gwidd
3ris_B	3ris_D	gwidd
3riy_A	3riy_B	gwidd
3rjr_D	3rjr_A	gwidd
3rlq_B	3rlq_A	gwidd
3rp1_D	3rp1_A	gwidd
3rpp_A	3rpp_C	gwidd
3rw7_A	3rw7_D	gwidd
3rz3_A	3rz3_C	gwidd
3s48_D	3s48_C	gwidd
3s5j_B	3s5j_A	gwidd
3s5z_A	3s5z_B	gwidd
3s8p_B	3s8p_A	gwidd
3s95_A	3s95_B	gwidd
3s9d_D	3s9d_B	gwidd
3sde_A	3sde_B	gwidd
3sek_C	3sek_B	gwidd
3sf4_A	3sf4_B	gwidd
3shi_A	3shi_G	gwidd
3sku_E	3sku_F	gwidd
3soc_B	3soc_A	gwidd
3sop_B	3sop_A	gwidd
3sp9_A	3sp9_B	gwidd
3sqp_A	3sqp_B	gwidd
3srf_H	3srf_D	gwidd

**Protein chain 1**	**Protein chain 2**	**Source**
3szm_C	3szm_F	gwidd
3t1i_D	3t1i_C	gwidd
3t5d_C	3t5d_A	gwidd
3tpx_A	3tpx_C	gwidd
3tq7_A	3tq7_B	gwidd
3tv0_A	3tv0_B	gwidd
3tw2_A	3tw2_B	gwidd
3u8d_A	3u8d_B	gwidd
3uf1_D	3uf1_A	gwidd
3uii_A	3uii_B	gwidd
3uin_A	3uin_B	gwidd
3uit_D	3uit_A	gwidd
3uk6_G	3uk6_F	gwidd
3ull_A	3ull_B	gwidd
3umn_C	3umn_B	gwidd
3uo9_D	3uo9_B	gwidd
3uoa_B	3uoa_C	gwidd
3urf_Z	3urf_A	gwidd
3v33_A	3v33_B	gwidd
3v3l_B	3v3l_A	gwidd
3v5l_D	3v5l_A	gwidd
3v6o_B	3v6o_A	gwidd
3v6s_A	3v6s_B	gwidd
3v83_F	3v83_A	gwidd
3vd0_C	3vd0_L	gwidd
3vd1_L	3vd1_C	gwidd
3vht_B	3vht_C	gwidd
3vjb_B	3vjb_A	gwidd
3vko_A	3vko_B	gwidd
3vvh_A	3vvh_C	gwidd
3w4u_A	3w4u_C	gwidd
3w58_C	3w58_D	gwidd
3w5e_A	3w5e_B	gwidd
3wan_B	3wan_A	gwidd
3wiy_A	3wiy_B	gwidd
3wtp_A	3wtp_B	gwidd
3zgv_B	3zgv_A	gwidd
3zjy_B	3zjy_C	gwidd
3zqs_A	3zqs_B	gwidd
3zxf_B	3zxf_A	gwidd
3zyi_A	3zyi_B	gwidd
3zzy_A	3zzy_B	gwidd
4a4c_A	4a4c_C	gwidd

Protein chain 1	Protein chain 2	Source
4a9z_A	4a9z_B	gwidd
4aa6_F	4aa6_E	gwidd
4akv_B	4akv_A	gwidd
4ap4_A	4ap4_F	gwidd
4ap8_A	4ap8_C	gwidd
4aqh_B	4aqh_A	gwidd
4as4_A	4as4_B	gwidd
4au8_B	4au8_A	gwidd
4avs_A	4avs_E	gwidd
4aya_A	4aya_B	gwidd
4ayc_A	4ayc_B	gwidd
4aze_C	4aze_B	gwidd
4b3z_D	4b3z_B	gwidd
4b7w_C	4b7w_D	gwidd
4b94_D	4b94_B	gwidd
4bej_B	4bej_A	gwidd
4bkx_B	4bkx_A	gwidd
4bmj_C	4bmj_B	gwidd
4bur_B	4bur_D	gwidd
4c3p_A	4c3p_D	gwidd
4c4w_E	4c4w_F	gwidd
4c4z_A	4c4z_B	gwidd
4c5d_A	4c5d_B	gwidd
4c8r_C	4c8r_D	gwidd
4c9b_A	4c9b_B	gwidd
4ccg_B	4ccg_Y	gwidd
4cgv_C	4cgv_D	gwidd
4cq1_H	4cq1_C	gwidd
4csr_A	4csr_B	gwidd
4d18_D	4d18_F	gwidd
4dep_E	4dep_D	gwidd
4deq_A	4deq_B	gwidd
4dnk_B	4dnk_A	gwidd
4dqm_A	4dqm_C	gwidd
4dur_A	4dur_B	gwidd
4e5y_D	4e5y_B	gwidd
4ed5_B	4ed5_A	gwidd
4edy_A	4edy_B	gwidd
4eoz_C	4eoz_A	gwidd
4eq1_A	4eq1_B	gwidd
4equ_B	4equ_A	gwidd
4f02_A	4f02_D	gwidd
4f6u_A	4f6u_B	gwidd

Protein chain 1	Protein chain 2	Source
4f7o_A	4f7o_B	gwidd
4f9k_B	4f9k_A	gwidd
4fao_M	4fao_Q	gwidd
4fcj_A	4fcj_B	gwidd
4fdl_B	4fdl_A	gwidd
4fgl_D	4fgl_C	gwidd
4fjv_C	4fjv_D	gwidd
4fqn_A	4fqn_D	gwidd
4fzs_A	4fzs_B	gwidd
4g0n_A	4g0n_B	gwidd
4g5q_A	4g5q_C	gwidd
4gg2_A	4gg2_B	gwidd
4gln_E	4gln_F	gwidd
4gls_E	4gls_F	gwidd
4gm3_F	4gm3_G	gwidd
4gpz_A	4gpz_B	gwidd
4gt4_A	4gt4_B	gwidd
4gy5_B	4gy5_D	gwidd
4h10_B	4h10_A	gwidd
4h2d_A	4h2d_B	gwidd
4hab_A	4hab_B	gwidd
4haf_A	4haf_B	gwidd
4hhv_A	4hhv_B	gwidd
4hi8_A	4hi8_B	gwidd
4hnj_A	4hnj_B	gwidd
4hon_A	4hon_B	gwidd
4hpm_D	4hpm_B	gwidd
4htp_B	4htp_A	gwidd
4hza_A	4hza_B	gwidd
4i0p_E	4i0p_F	gwidd
4idv_A	4idv_D	gwidd
4igg_B	4igg_A	gwidd
4il1_B	4il1_D	gwidd
4in4_A	4in4_B	gwidd
4inc_B	4inc_A	gwidd
4iz7_A	4iz7_B	gwidd
4j5x_B	4j5x_C	gwidd
4j6g_D	4j6g_B	gwidd
4jnk_D	4jnk_A	gwidd
4joi_B	4joi_C	gwidd
4jok_A	4jok_B	gwidd
4ju5_A	4ju5_B	gwidd
4jze_H	4jze_L	gwidd

Protein chain 1	Protein chain 2	Source
4k4i_A	4k4i_M	gwidd
4kbl_A	4kbl_B	gwidd
4kd7_A	4kd7_B	gwidd
4ko8_A	4ko8_B	gwidd
4kp5_B	4kp5_D	gwidd
4kt1_A	4kt1_E	gwidd
4kww_A	4kww_F	gwidd
4kxy_A	4kxy_B	gwidd
4ky2_A	4ky2_B	gwidd
4ky9_A	4ky9_P	gwidd
4l3v_A	4l3v_C	gwidd
4ldq_B	4ldq_A	gwidd
4lec_A	4lec_B	gwidd
4lg1_C	4lg1_B	gwidd
4li0_F	4li0_E	gwidd
4ll9_B	4ll9_C	gwidd
4llu_A	4llu_B	gwidd
4lp5_A	4lp5_B	gwidd
4lsz_D	4lsz_B	gwidd
4ly1_A	4ly1_C	gwidd
4m0e_B	4m0e_A	gwidd
4m3p_D	4m3p_A	gwidd
4m4e_C	4m4e_A	gwidd
4m4r_A	4m4r_B	gwidd
4m6r_A	4m6r_D	gwidd
4m7r_A	4m7r_B	gwidd
4mel_A	4mel_B	gwidd
4mfv_A	4mfv_B	gwidd
4mjh_A	4mjh_C	gwidd
4mjo_E	4mjo_H	gwidd
4mne_A	4mne_B	gwidd
4ms4_A	4ms4_B	gwidd
4msv_B	4msv_A	gwidd
4mtl_B	4mtl_A	gwidd
4mxv_B	4mxv_D	gwidd
4myg_A	4myg_B	gwidd
4n0f_D	4n0f_A	gwidd
4n7f_A	4n7f_B	gwidd
4n7v_A	4n7v_B	gwidd
4n85_A	4n85_B	gwidd
4n8r_A	4n8r_C	gwidd
4ndn_D	4ndn_C	gwidd
4neu_A	4neu_B	gwidd

Protein chain 1	Protein chain 2	Source
4nhh_L	4nhh_Q	gwidd
4nn0_B	4nn0_A	gwidd
4no0_A	4no0_D	gwidd
4nqa_I	4nqa_B	gwidd
4nqk_C	4nqk_H	gwidd
4nsc_C	4nsc_F	gwidd
4nxt_C	4nxt_D	gwidd
4od9_D	4od9_C	gwidd
4oe5_C	4oe5_D	gwidd
4ogr_A	4ogr_E	gwidd
4okh_A	4okh_B	gwidd
4ole_A	4ole_B	gwidd
4on9_A	4on9_B	gwidd
4or5_B	4or5_G	gwidd
4orh_J	4orh_K	gwidd
4ov5_G	4ov5_M	gwidd
4ova_A	4ova_D	gwidd
4p0p_A	4p0p_B	gwidd
4p0s_D	4p0s_H	gwidd
4p42_A	4p42_B	gwidd
4p5e_A	4p5e_B	gwidd
4poc_B	4poc_A	gwidd
4pw8_A	4pw8_H	gwidd
4pyg_B	4pyg_E	gwidd
4q3h_A	4q3h_B	gwidd
4qqg_E	4qqg_A	gwidd
4qqm_C	4qqm_A	gwidd
4qtb_A	4qtb_B	gwidd
4qxw_A	4qxw_B	gwidd
4r14_A	4r14_B	gwidd
4r2y_D	4r2y_B	gwidd
4rao_D	4rao_A	gwidd
4rdu_D	4rdu_A	gwidd
4rer_G	4rer_B	gwidd
4rg9_B	4rg9_A	gwidd
4rhw_C	4rhw_F	gwidd
4riq_V	4riq_Z	gwidd
4rov_A	4rov_B	gwidd
4rqx_D	4rqx_E	gwidd
4rwd_A	4rwd_B	gwidd
4s1z_C	4s1z_G	gwidd
4tmp_C	4tmp_A	gwidd
4tpk_A	4tpk_B	gwidd

**Protein chain 1**	**Protein chain 2**	**Source**
4tqb_A	4tqb_B	gwidd
4twt_A	4twt_B	gwidd
4u1k_A	4u1k_D	gwidd
4u2m_D	4u2m_C	gwidd
4u2v_B	4u2v_A	gwidd
4u5w_B	4u5w_D	gwidd
4uai_A	4uai_B	gwidd
4umo_D	4umo_B	gwidd
4uq3_A	4uq3_C	gwidd
4ux9_D	4ux9_A	gwidd
4uxn_A	4uxn_B	gwidd
4v5z_Ai	4v5z_Bx	gwidd
4wij_B	4wij_A	gwidd
4wjg_2	4wjg_1	gwidd
4x3g_B	4x3g_A	gwidd
6q21_A	6q21_B	gwidd
9api_A	9api_B	gwidd
