# Supplementary material for: Structure-based prediction of protein-protein interaction network in rice
Source: Genet Mol Biol. 2024 Feb 2;47(1):e20230068. doi: 10.1590/1678-4685-GMB-2023-0068 (PMC10849033; doi:10.1590/1678-4685-GMB-2023-0068)
Supplement: Table S2 - [file 1415-4757-GMB-47-01-e20230068-s2.pdf]

Genetics and Molecular Biology
https://doi.org/10.1590/1678-4685-GMB-2023-0068

Supplementary Material to "Structure-based prediction of protein-protein interaction network in rice"

Table S2. List of 30 rice binary complexes.

Protein chain 1	Protein chain 2
1h1z_A	1h1z_B
1uvc_A	1uvc_B
2gnw_A	2gnw_B
2q4f_A	2q4f_B
2qn4_A	2qn4_B
2wg9_A	2wg9_B
3a8r_A	3a8r_B
3f5l_A	3f5l_B
3fr8_A	3fr8_B
3iwr_A	3iwr_B
3oit_A	3oit_B
3ptq_A	3ptq_B
3umv_A	3umv_B
3vu2_A	3vu2_B
4oic_A	4oic_B
5ee9_A	5ee9_B
5jce_A	5jce_B
5jcn_A	5jcn_B
5jnp_A	5jnp_B
5lw0_A	5lw0_B
5xfh_A	5xfh_B
5zkt_A	5zkt_B
6ae9_A	6ae9_B
6elx_A	6elx_B
6khp_A	6khp_B
6lcq_A	6lcq_B
6oce_A	6oce_B
6r0l_A	6r0l_B
7bzm_A	7bzm_B
7d6b_A	7d6b_B
