# Supplementary material for: Structure-based prediction of protein-protein interaction network in rice
Source: Genet Mol Biol. 2024 Feb 2;47(1):e20230068. doi: 10.1590/1678-4685-GMB-2023-0068 (PMC10849033; doi:10.1590/1678-4685-GMB-2023-0068)
Supplement: Table S3 - [file 1415-4757-GMB-47-01-e20230068-s3.pdf]

Genetics and Molecular Biology
https://doi.org/10.1590/1678-4685-GMB-2023-0068

Supplementary Material to "Structure-based prediction of protein-protein interaction network in rice"

Table S3. List of predicted rice protein-protein interactions.

InteractorA	InteractorB	Z score
LOC_Os02g57630	LOC_Os12g13390	9.83366123
LOC_Os02g57720	LOC_Os06g15990	9.12358511
LOC_Os02g57770	LOC_Os11g37640	8.82593466
LOC_Os02g57854	LOC_Os12g22650	9.79924206
LOC_Os02g57770	LOC_Os04g25990	9.37426066
LOC_Os02g57770	LOC_Os12g38770	9.21876616
LOC_Os02g58340	LOC_Os02g58730	9.14743021
LOC_Os02g57854	LOC_Os08g39140	9.24839706
LOC_Os02g57630	LOC_Os07g05800	8.82505687
LOC_Os02g57720	LOC_Os11g33240	8.86533632
LOC_Os02g57854	LOC_Os05g29880	9.02722015
LOC_Os02g58340	LOC_Os07g28480	9.61822997
LOC_Os02g57770	LOC_Os08g09250	9.28906183
LOC_Os02g57770	LOC_Os09g31502	11.2905118
LOC_Os02g57770	LOC_Os08g28730	8.96111735
LOC_Os02g57450	LOC_Os12g07830	8.99308371
LOC_Os02g58340	LOC_Os06g08600	9.86484086
LOC_Os02g57854	LOC_Os03g25340	9.21766815
LOC_Os02g58340	LOC_Os05g30480	9.35691388
LOC_Os02g58480	LOC_Os08g04540	9.08960218
LOC_Os02g57770	LOC_Os10g36650	8.8646212
LOC_Os02g57450	LOC_Os07g29750	9.0948513
LOC_Os02g58480	LOC_Os08g32620	8.88976524
LOC_Os02g58340	LOC_Os03g64030	9.04409347
LOC_Os02g57854	LOC_Os07g38030	9.0118494
LOC_Os02g57630	LOC_Os04g45490	11.9026304
LOC_Os02g58340	LOC_Os09g12230	9.52010231
LOC_Os02g58340	LOC_Os04g39840	11.3882421
LOC_Os02g57770	LOC_Os04g25400	10.3314576
LOC_Os02g58340	LOC_Os07g34589	9.12512445
LOC_Os02g57630	LOC_Os04g33240	9.82756132
LOC_Os02g58340	LOC_Os05g05470	11.3326409

InteractorA	InteractorB	Z score
LOC_Os02g57450	LOC_Os06g23870	8.80271257
LOC_Os02g58340	LOC_Os04g52361	8.90496093
LOC_Os02g58340	LOC_Os07g12200	9.06383791
LOC_Os02g57770	LOC_Os06g07978	9.34280881
LOC_Os02g58340	LOC_Os04g33720	8.80627297
LOC_Os02g57630	LOC_Os03g15120	9.97207344
LOC_Os02g57770	LOC_Os03g50250	11.1778169
LOC_Os02g58480	LOC_Os03g61330	10.6730377
LOC_Os02g57770	LOC_Os03g18510	9.02584447
LOC_Os02g58340	LOC_Os04g16770	9.62888007
LOC_Os02g57770	LOC_Os10g38640	8.84255866
LOC_Os02g58480	LOC_Os06g35490	8.80246359
LOC_Os02g57720	LOC_Os10g02480	8.85912753
LOC_Os02g58340	LOC_Os06g37080	8.97856667
LOC_Os02g58340	LOC_Os03g59710	8.85453833
LOC_Os02g57770	LOC_Os07g47290	8.91536436
LOC_Os02g57630	LOC_Os10g34520	11.6655209
LOC_Os02g57770	LOC_Os03g12270	9.96826179
LOC_Os02g57630	LOC_Os10g10434	9.17869461
LOC_Os02g58340	LOC_Os05g09500	9.47287744
LOC_Os02g57854	LOC_Os09g10230	9.21083546
LOC_Os02g57770	LOC_Os08g28680	8.84048521
LOC_Os02g57770	LOC_Os06g15990	9.85882594
LOC_Os02g57450	LOC_Os07g47290	10.1331992
LOC_Os02g58480	LOC_Os06g51150	9.61092763
LOC_Os02g57630	LOC_Os10g38670	8.90513248
LOC_Os02g58480	LOC_Os07g07320	9.13156556
LOC_Os02g57630	LOC_Os05g09500	8.9536512
LOC_Os02g58340	LOC_Os05g41060	9.83598944
LOC_Os02g57630	LOC_Os12g12560	9.03289421
LOC_Os02g57450	LOC_Os07g40290	8.89729107
LOC_Os02g57854	LOC_Os03g60580	8.88896822
LOC_Os02g57770	LOC_Os04g01470	8.88312752
LOC_Os02g57770	LOC_Os04g46930	9.20281313
LOC_Os02g57854	LOC_Os12g32240	8.94199981
LOC_Os02g57770	LOC_Os07g29750	8.99567298
LOC_Os02g57854	LOC_Os08g06060	9.56178485
LOC_Os02g57450	LOC_Os10g21344	8.96820059
LOC_Os02g57854	LOC_Os03g45920	10.0478014
LOC_Os02g57720	LOC_Os06g51050	9.1735343
LOC_Os02g57770	LOC_Os07g28480	11.0697245
LOC_Os02g57770	LOC_Os08g28820	10.2232104
LOC_Os02g57770	LOC_Os07g43250	11.0019084

InteractorA	InteractorB	Z score
LOC_Os02g58480	LOC_Os08g34170	9.08444296
LOC_Os02g57450	LOC_Os07g42490	9.7868495
LOC_Os02g58340	LOC_Os06g35560	9.25482832
LOC_Os02g57854	LOC_Os11g02440	9.10057355
LOC_Os02g58340	LOC_Os08g35420	9.62886973
LOC_Os02g58340	LOC_Os08g15030	9.48245693
LOC_Os02g57630	LOC_Os11g24560	8.80808633
LOC_Os02g57854	LOC_Os07g05940	9.24728172
LOC_Os02g58340	LOC_Os11g04954	9.14751211
LOC_Os02g57450	LOC_Os09g39500	8.87224688
LOC_Os02g58340	LOC_Os10g02480	9.03693806
LOC_Os02g57854	LOC_Os11g06390	11.3222768
LOC_Os02g57450	LOC_Os10g35480	9.11490224
LOC_Os02g57450	LOC_Os06g49970	9.42281286
LOC_Os02g57770	LOC_Os06g07878	9.34279809
LOC_Os02g58480	LOC_Os07g26640	8.92672981
LOC_Os02g58340	LOC_Os08g09200	9.81874502
LOC_Os02g57770	LOC_Os09g09270	9.47907919
LOC_Os02g58480	LOC_Os07g46310	10.9577513
LOC_Os02g58340	LOC_Os10g41510	8.81152425
LOC_Os02g58480	LOC_Os07g42950	9.12627099
LOC_Os02g58340	LOC_Os07g16970	8.96054459
LOC_Os02g57450	LOC_Os09g12660	8.901633
LOC_Os02g57770	LOC_Os05g33150	9.34423531
LOC_Os02g58340	LOC_Os04g40874	9.29727378
LOC_Os02g58340	LOC_Os11g07020	8.96300762
LOC_Os02g57770	LOC_Os10g01080	9.15115134
LOC_Os02g57630	LOC_Os04g56730	11.513139
LOC_Os02g57630	LOC_Os06g45120	10.3745445
LOC_Os02g58480	LOC_Os06g37610	9.57666253
LOC_Os02g57770	LOC_Os06g37080	10.3464189
LOC_Os02g57720	LOC_Os07g36140	9.29357403
LOC_Os02g57630	LOC_Os07g39290	8.83596011
LOC_Os02g57630	LOC_Os03g61330	9.0407104
LOC_Os02g58730	LOC_Os03g48310	8.94029095
LOC_Os02g58730	LOC_Os11g31530	8.96070975
LOC_Os02g58730	LOC_Os05g47890	9.23889046
LOC_Os03g01530	LOC_Os07g46280	10.8992207
LOC_Os03g01290	LOC_Os06g40190	8.9394707
LOC_Os03g01530	LOC_Os08g14760	9.01324805
LOC_Os03g01260	LOC_Os06g47320	10.0056889
LOC_Os03g01530	LOC_Os08g04560	9.2682178
LOC_Os02g58730	LOC_Os11g10510	9.76409261

InteractorA	InteractorB	Z score
LOC_Os03g01260	LOC_Os03g62670	9.28065542
LOC_Os03g01120	LOC_Os03g08020	8.85317802
LOC_Os02g58730	LOC_Os11g08340	8.89883834
LOC_Os03g01260	LOC_Os11g32520	9.37396512
LOC_Os02g58730	LOC_Os07g36140	9.46787255
LOC_Os03g01290	LOC_Os09g25390	9.61202832
LOC_Os03g01290	LOC_Os03g56241	9.10452149
LOC_Os03g01260	LOC_Os04g41310	8.89777578
LOC_Os03g01290	LOC_Os06g51084	10.6358823
LOC_Os02g58730	LOC_Os03g18130	9.86905481
LOC_Os03g01530	LOC_Os06g15990	9.06313132
LOC_Os03g01260	LOC_Os04g55960	8.87624917
LOC_Os03g01120	LOC_Os12g25710	8.88398106
LOC_Os02g58730	LOC_Os12g40550	9.73074986
LOC_Os03g01260	LOC_Os03g15120	8.97531936
LOC_Os02g58480	LOC_Os10g33800	10.4826716
LOC_Os03g01290	LOC_Os08g09770	9.38445803
LOC_Os03g01290	LOC_Os04g43410	9.43047409
LOC_Os03g01290	LOC_Os07g48880	9.44030524
LOC_Os03g01170	LOC_Os03g20370	9.56600638
LOC_Os03g01290	LOC_Os07g07320	9.17766377
LOC_Os03g01120	LOC_Os03g10940	9.58626138
LOC_Os03g01290	LOC_Os05g51050	9.1683209
LOC_Os02g58730	LOC_Os03g38020	8.82435497
LOC_Os03g01290	LOC_Os04g37480	9.17094134
LOC_Os03g01170	LOC_Os10g29620	8.90716111
LOC_Os03g01530	LOC_Os04g39020	8.89592713
LOC_Os03g01170	LOC_Os07g30970	8.88815947
LOC_Os03g01530	LOC_Os03g64030	8.92659829
LOC_Os03g01530	LOC_Os06g35490	8.84825879
LOC_Os03g01530	LOC_Os05g02940	8.94947141
LOC_Os03g01170	LOC_Os07g37320	8.85288402
LOC_Os02g58730	LOC_Os08g40140	9.34508472
LOC_Os03g01260	LOC_Os07g23470	9.12061813
LOC_Os03g01290	LOC_Os04g48850	9.01776284
LOC_Os03g01170	LOC_Os07g48780	9.56600638
LOC_Os03g01530	LOC_Os11g19800	8.86470996
LOC_Os03g01120	LOC_Os03g08010	8.85317802
LOC_Os03g01120	LOC_Os04g33970	10.1404367
LOC_Os03g01260	LOC_Os07g26900	8.82618811
LOC_Os03g01260	LOC_Os09g20820	9.27597887
LOC_Os03g01120	LOC_Os12g16410	8.95857884
LOC_Os03g01120	LOC_Os04g24430	9.06354079

InteractorA	InteractorB	Z score
LOC_Os03g01290	LOC_Os10g38489	9.91675397
LOC_Os03g01290	LOC_Os11g19800	8.84910166
LOC_Os03g01530	LOC_Os05g29880	9.444326
LOC_Os02g58730	LOC_Os04g33570	10.7341432
LOC_Os03g01120	LOC_Os11g25260	10.037027
LOC_Os03g01120	LOC_Os10g36650	9.90873713
LOC_Os03g01260	LOC_Os06g06040	9.59742121
LOC_Os02g58730	LOC_Os11g10520	9.32719429
LOC_Os03g01530	LOC_Os03g03910	9.65102977
LOC_Os03g01530	LOC_Os07g30170	8.80551792
LOC_Os03g01120	LOC_Os09g28770	9.86921727
LOC_Os03g01290	LOC_Os03g62500	9.20082752
LOC_Os03g01120	LOC_Os03g61970	9.00951712
LOC_Os03g01290	LOC_Os04g12720	9.60726405
LOC_Os03g01290	LOC_Os08g40930	8.9196565
LOC_Os02g58480	LOC_Os12g08270	10.288795
LOC_Os03g01120	LOC_Os03g08050	8.85317802
LOC_Os03g01290	LOC_Os11g41610	9.10451541
LOC_Os03g01650	LOC_Os03g28400	10.1146151
LOC_Os03g01610	LOC_Os10g25930	9.03692386
LOC_Os03g01640	LOC_Os05g44140	9.60860529
LOC_Os03g01800	LOC_Os05g06480	8.8061359
LOC_Os03g01650	LOC_Os05g46270	9.26189882
LOC_Os03g01610	LOC_Os03g58300	9.05841182
LOC_Os03g01630	LOC_Os12g13390	8.87394675
LOC_Os03g01770	LOC_Os06g36670	8.9738077
LOC_Os03g01610	LOC_Os05g48510	10.0225971
LOC_Os03g01770	LOC_Os03g49600	9.02032363
LOC_Os03g01610	LOC_Os03g17980	10.6860844
LOC_Os03g01640	LOC_Os11g43960	9.16004704
LOC_Os03g01610	LOC_Os04g16826	9.71312933
LOC_Os03g01650	LOC_Os04g55410	9.18363914
LOC_Os03g01630	LOC_Os07g34589	10.1484502
LOC_Os03g01770	LOC_Os03g03910	10.1794255
LOC_Os03g01650	LOC_Os10g35070	9.47101412
LOC_Os03g01800	LOC_Os10g38640	10.1679589
LOC_Os03g01630	LOC_Os11g14040	9.22112068
LOC_Os03g01610	LOC_Os04g54390	8.83606242
LOC_Os03g01800	LOC_Os04g01600	9.15599166
LOC_Os03g01770	LOC_Os12g43440	8.84349654
LOC_Os03g01610	LOC_Os03g28400	10.1146151
LOC_Os03g01800	LOC_Os04g10010	9.21122689
LOC_Os03g01650	LOC_Os10g21344	9.71313015

InteractorA	InteractorB	Z score
LOC_Os03g01610	LOC_Os10g21344	9.71313015
LOC_Os03g01650	LOC_Os03g17980	10.6860844
LOC_Os03g01610	LOC_Os10g10434	9.2389492
LOC_Os03g01770	LOC_Os12g22680	9.23413959
LOC_Os03g01800	LOC_Os10g38234	9.66937382
LOC_Os03g01610	LOC_Os05g46270	9.26189882
LOC_Os03g01800	LOC_Os06g11240	11.0944378
LOC_Os03g01650	LOC_Os03g58300	9.05840991
LOC_Os03g01640	LOC_Os08g20730	9.19994268
LOC_Os03g01650	LOC_Os07g44790	10.1146151
LOC_Os03g01650	LOC_Os10g25930	9.0368942
LOC_Os03g01770	LOC_Os09g27750	8.96938567
LOC_Os03g01640	LOC_Os09g28400	9.16159123
LOC_Os03g01770	LOC_Os11g25220	9.30637785
LOC_Os03g01630	LOC_Os08g08500	9.09016274
LOC_Os03g01640	LOC_Os04g33970	9.49330649
LOC_Os03g01630	LOC_Os06g39708	8.85013525
LOC_Os03g01880	LOC_Os03g58530	9.08236055
LOC_Os03g01800	LOC_Os05g04500	8.83400881
LOC_Os03g01650	LOC_Os03g32270	8.83651418
LOC_Os03g01800	LOC_Os08g39870	9.16545099
LOC_Os03g01770	LOC_Os03g49220	9.61261023
LOC_Os03g01650	LOC_Os04g54390	8.83603027
LOC_Os03g01880	LOC_Os03g49380	9.5887809
LOC_Os03g01770	LOC_Os05g38560	9.57059515
LOC_Os03g01610	LOC_Os08g44370	9.09099385
LOC_Os03g01770	LOC_Os10g37060	10.0992277
LOC_Os03g01610	LOC_Os04g55410	9.18363914
LOC_Os03g01770	LOC_Os07g30170	10.5634282
LOC_Os03g01800	LOC_Os07g17010	9.05957749
LOC_Os03g01800	LOC_Os05g49840	8.84268723
LOC_Os03g01770	LOC_Os03g31210	9.23686002
LOC_Os03g01770	LOC_Os12g02060	11.0561171
LOC_Os03g01800	LOC_Os08g20270	11.1441722
LOC_Os03g01800	LOC_Os03g59020	13.8863737
LOC_Os03g01800	LOC_Os08g40740	10.2326573
LOC_Os03g01630	LOC_Os10g41490	9.02357951
LOC_Os03g01800	LOC_Os08g28800	8.98878653
LOC_Os03g01610	LOC_Os10g35070	9.47095922
LOC_Os03g01640	LOC_Os12g16290	9.97976879
LOC_Os03g01650	LOC_Os10g10434	9.2389492
LOC_Os03g01650	LOC_Os04g16826	9.71312933
LOC_Os03g01800	LOC_Os03g17470	8.98083243

InteractorA	InteractorB	Z score
LOC_Os03g01610	LOC_Os07g44790	10.1146151
LOC_Os03g01800	LOC_Os04g12960	10.2660102
LOC_Os03g01610	LOC_Os03g32270	8.83647176
LOC_Os03g01650	LOC_Os05g48510	10.0225996
LOC_Os03g01650	LOC_Os08g44370	9.09097516
LOC_Os03g01650	LOC_Os03g63330	9.37434311
LOC_Os03g01640	LOC_Os03g11970	9.27811589
LOC_Os03g01640	LOC_Os10g25130	9.3717534
LOC_Os03g01610	LOC_Os03g63330	9.37434311
LOC_Os03g01880	LOC_Os04g39840	10.0689127
LOC_Os03g01640	LOC_Os03g12290	9.35222201
LOC_Os03g01770	LOC_Os04g58580	8.96534536
LOC_Os03g01640	LOC_Os04g37950	9.60413808
LOC_Os03g01880	LOC_Os10g33900	9.64321113
LOC_Os03g02780	LOC_Os03g58050	10.0431435
LOC_Os03g02710	LOC_Os08g06100	9.50102593
LOC_Os03g01900	LOC_Os10g28050	9.48364164
LOC_Os03g02260	LOC_Os03g08800	9.60733833
LOC_Os03g02680	LOC_Os03g50885	9.18020288
LOC_Os03g02920	LOC_Os06g11210	8.83617093
LOC_Os03g02680	LOC_Os09g27820	9.16084024
LOC_Os03g01900	LOC_Os12g38750	10.0711405
LOC_Os03g02710	LOC_Os04g53810	8.86048956
LOC_Os03g01900	LOC_Os03g04169	9.02646582
LOC_Os03g02780	LOC_Os03g61600	8.88669481
LOC_Os03g02260	LOC_Os03g04060	10.3401126
LOC_Os03g01880	LOC_Os06g15990	9.21563032
LOC_Os03g02780	LOC_Os03g07150	9.73068597
LOC_Os03g02780	LOC_Os03g51200	9.10305633
LOC_Os03g01900	LOC_Os03g63330	10.757888
LOC_Os03g02710	LOC_Os07g05400	10.0659449
LOC_Os03g02260	LOC_Os03g56410	10.5593977
LOC_Os03g01900	LOC_Os06g46340	8.81500744
LOC_Os03g02680	LOC_Os11g08445	9.1910638
LOC_Os03g02780	LOC_Os11g10510	8.86156789
LOC_Os03g01880	LOC_Os05g46360	13.3432477
LOC_Os03g01900	LOC_Os06g51084	9.30411008
LOC_Os03g02680	LOC_Os03g16860	8.82225105
LOC_Os03g02680	LOC_Os07g41750	8.89647914
LOC_Os03g02260	LOC_Os10g38234	9.57427049
LOC_Os03g02710	LOC_Os07g43390	9.24208416
LOC_Os03g01880	LOC_Os07g06970	9.51029715
LOC_Os03g02260	LOC_Os03g56280	9.34056913

InteractorA	InteractorB	Z score
LOC_Os03g02680	LOC_Os03g27370	8.86811969
LOC_Os03g02780	LOC_Os07g07709	10.0431435
LOC_Os03g02710	LOC_Os11g08440	8.91625547
LOC_Os03g02710	LOC_Os05g36010	9.0873455
LOC_Os03g01900	LOC_Os05g01675	10.7313132
LOC_Os03g02260	LOC_Os12g41110	10.3813859
LOC_Os03g02260	LOC_Os07g06970	9.09749717
LOC_Os03g01900	LOC_Os12g10600	10.2833159
LOC_Os03g02260	LOC_Os04g26910	9.67784379
LOC_Os03g02260	LOC_Os11g07020	9.64871972
LOC_Os03g02260	LOC_Os03g06940	8.98748179
LOC_Os03g02780	LOC_Os11g31620	9.02282073
LOC_Os03g02920	LOC_Os06g23780	8.88382828
LOC_Os03g02780	LOC_Os03g31300	10.4717978
LOC_Os03g02920	LOC_Os06g36700	10.2245793
LOC_Os03g02260	LOC_Os12g12580	9.91196611
LOC_Os03g02710	LOC_Os08g43170	14.5132149
LOC_Os03g02680	LOC_Os07g17010	8.84182282
LOC_Os03g02920	LOC_Os03g47770	8.93032614
LOC_Os03g02710	LOC_Os09g34960	13.7812001
LOC_Os03g02680	LOC_Os05g40990	9.04701161
LOC_Os03g02260	LOC_Os03g44170	10.1363368
LOC_Os03g02920	LOC_Os03g64030	9.37759251
LOC_Os03g02260	LOC_Os06g37150	9.3228673
LOC_Os03g02920	LOC_Os04g57220	9.2515971
LOC_Os03g01900	LOC_Os03g05730	9.75101963
LOC_Os03g02780	LOC_Os10g22070	9.42850141
LOC_Os03g02260	LOC_Os04g40990	9.13647537
LOC_Os03g02920	LOC_Os03g04250	9.05245408
LOC_Os03g02680	LOC_Os04g09604	9.18490522
LOC_Os03g02680	LOC_Os03g21260	8.93916761
LOC_Os03g02260	LOC_Os06g19960	9.33467176
LOC_Os03g02710	LOC_Os06g51060	9.27137757
LOC_Os03g02710	LOC_Os09g23560	8.93530482
LOC_Os03g02780	LOC_Os11g10480	8.92560379
LOC_Os03g01880	LOC_Os09g37540	10.1021688
LOC_Os03g02680	LOC_Os11g10480	9.75646354
LOC_Os03g02260	LOC_Os05g04510	8.84435105
LOC_Os03g02680	LOC_Os09g23540	9.38408552
LOC_Os03g02710	LOC_Os05g47545	9.20520527
LOC_Os03g01900	LOC_Os10g08670	11.0797199
LOC_Os03g02780	LOC_Os07g07719	8.80395171
LOC_Os03g01900	LOC_Os09g31120	9.33122643

InteractorA	InteractorB	Z score
LOC_Os03g02260	LOC_Os08g09200	9.13808716
LOC_Os03g02780	LOC_Os11g10520	10.9140543
LOC_Os03g01900	LOC_Os04g16760	10.7313132
LOC_Os03g02710	LOC_Os06g05090	8.90585523
LOC_Os03g02780	LOC_Os10g01540	9.10660275
LOC_Os03g02680	LOC_Os04g52280	9.02785089
LOC_Os03g02710	LOC_Os11g26850	9.29456873
LOC_Os03g02260	LOC_Os07g48050	9.41862487
LOC_Os03g02260	LOC_Os09g38620	10.9543724
LOC_Os03g02710	LOC_Os10g26390	8.80147952
LOC_Os03g02780	LOC_Os12g22680	9.95584485
LOC_Os03g02780	LOC_Os10g39170	9.48057701
LOC_Os03g02780	LOC_Os03g18740	9.04603168
LOC_Os03g02710	LOC_Os05g12180	9.18000667
LOC_Os03g01900	LOC_Os04g52280	9.22181082
LOC_Os03g02920	LOC_Os04g02820	9.02491705
LOC_Os03g02710	LOC_Os06g44620	10.0909288
LOC_Os03g02710	LOC_Os05g29880	9.36020199
LOC_Os03g02710	LOC_Os09g31490	10.2791182
LOC_Os03g03720	LOC_Os03g38980	11.6144137
LOC_Os03g03720	LOC_Os03g50440	9.65555096
LOC_Os03g03720	LOC_Os06g45590	11.3424817
LOC_Os03g03720	LOC_Os08g03290	15.2914119
LOC_Os03g03910	LOC_Os07g10720	9.04904059
LOC_Os03g03720	LOC_Os06g37660	9.21439674
LOC_Os03g03910	LOC_Os03g18560	8.81238003
LOC_Os03g03910	LOC_Os09g21770	8.94029344
LOC_Os03g03720	LOC_Os04g29550	10.0335248
LOC_Os03g03720	LOC_Os10g29620	9.12596741
LOC_Os03g03130	LOC_Os07g34580	9.54122269
LOC_Os03g03720	LOC_Os10g38150	10.2733376
LOC_Os03g03720	LOC_Os03g04250	10.815501
LOC_Os03g04060	LOC_Os11g05880	9.06329002
LOC_Os03g03910	LOC_Os11g29190	8.82752948
LOC_Os03g04060	LOC_Os12g31370	8.85692606
LOC_Os03g03130	LOC_Os08g15322	9.2763621
LOC_Os03g03130	LOC_Os11g08440	8.92356746
LOC_Os03g04060	LOC_Os06g04510	8.84204836
LOC_Os03g03130	LOC_Os10g21192	8.81185277
LOC_Os03g02939	LOC_Os11g24560	8.80745705
LOC_Os03g04110	LOC_Os03g58260	9.42841097
LOC_Os03g03910	LOC_Os08g32620	13.1783504
LOC_Os03g04110	LOC_Os08g41830	8.81079377

InteractorA	InteractorB	Z score
LOC_Os03g03130	LOC_Os06g10930	9.09718671
LOC_Os03g03130	LOC_Os09g30360	8.88669556
LOC_Os03g04110	LOC_Os03g49380	9.52614329
LOC_Os03g03910	LOC_Os04g18200	9.38567738
LOC_Os03g03130	LOC_Os07g44450	9.34481947
LOC_Os03g04060	LOC_Os08g04540	9.06607818
LOC_Os03g03910	LOC_Os03g47610	9.62785337
LOC_Os03g03720	LOC_Os08g41830	9.72004162
LOC_Os03g04060	LOC_Os08g36900	8.86749382
LOC_Os03g04060	LOC_Os09g25390	9.24724316
LOC_Os03g03910	LOC_Os04g36700	9.12108859
LOC_Os03g02920	LOC_Os10g37060	9.12922745
LOC_Os03g03910	LOC_Os07g10590	9.00947719
LOC_Os03g03910	LOC_Os05g11550	9.38669127
LOC_Os03g03720	LOC_Os03g16020	9.20640248
LOC_Os03g03720	LOC_Os06g11210	10.6375308
LOC_Os03g04110	LOC_Os04g02050	9.63592665
LOC_Os03g03910	LOC_Os03g09810	9.10842439
LOC_Os03g04110	LOC_Os12g42876	9.6631394
LOC_Os03g02939	LOC_Os06g11210	10.761372
LOC_Os03g04060	LOC_Os04g40874	9.38882064
LOC_Os03g03720	LOC_Os06g01590	8.90125198
LOC_Os03g03910	LOC_Os05g01050	9.5027788
LOC_Os03g02939	LOC_Os10g39170	9.3788033
LOC_Os03g03910	LOC_Os08g43170	8.92296194
LOC_Os03g04110	LOC_Os09g23530	9.29399605
LOC_Os03g02939	LOC_Os06g51150	9.04798011
LOC_Os03g03720	LOC_Os04g35240	9.07008736
LOC_Os03g02939	LOC_Os04g47170	9.34083845
LOC_Os03g03910	LOC_Os05g48290	8.98385673
LOC_Os03g03910	LOC_Os04g24430	9.64866316
LOC_Os03g03720	LOC_Os06g35540	9.9029674
LOC_Os03g03130	LOC_Os04g56580	8.91986351
LOC_Os03g03130	LOC_Os03g44150	9.24627145
LOC_Os03g03720	LOC_Os09g10270	8.83167621
LOC_Os03g04110	LOC_Os07g09680	8.82901265
LOC_Os03g04060	LOC_Os05g47540	9.05079277
LOC_Os03g04060	LOC_Os12g23170	8.92621127
LOC_Os03g03910	LOC_Os06g04270	9.6446237
LOC_Os03g03720	LOC_Os03g25370	10.0216158
LOC_Os03g04110	LOC_Os08g34290	9.37449196
LOC_Os03g03720	LOC_Os05g50380	8.91941101
LOC_Os03g04060	LOC_Os06g37150	9.15329419

InteractorA	InteractorB	Z score
LOC_Os03g03720	LOC_Os06g51150	9.18123926
LOC_Os03g02939	LOC_Os06g19960	8.87560563
LOC_Os03g03130	LOC_Os10g21298	9.2763621
LOC_Os03g03130	LOC_Os12g02980	10.1001899
LOC_Os03g04110	LOC_Os05g26890	9.01531118
LOC_Os03g04060	LOC_Os03g11960	8.98268504
LOC_Os03g03910	LOC_Os08g44530	9.35956791
LOC_Os03g02920	LOC_Os08g20420	8.83519281
LOC_Os03g04060	LOC_Os05g23740	9.00696821
LOC_Os03g03130	LOC_Os04g47170	8.84595525
LOC_Os03g03720	LOC_Os09g07510	9.06765801
LOC_Os03g04110	LOC_Os08g04560	11.3460967
LOC_Os03g03130	LOC_Os10g07229	9.14495074
LOC_Os03g03910	LOC_Os12g42884	9.25755984
LOC_Os03g04060	LOC_Os09g39570	8.86752493
LOC_Os03g03130	LOC_Os06g39728	9.27635584
LOC_Os03g03720	LOC_Os06g35940	10.6029005
LOC_Os03g02939	LOC_Os08g40140	10.2018751
LOC_Os03g04060	LOC_Os05g25490	9.30539864
LOC_Os03g04060	LOC_Os08g20420	9.18637264
LOC_Os03g04110	LOC_Os05g35400	9.62416057
LOC_Os03g04110	LOC_Os07g17010	8.92527792
LOC_Os03g03720	LOC_Os09g28400	8.92329146
LOC_Os03g04060	LOC_Os05g20050	9.01776035
LOC_Os03g02939	LOC_Os04g46560	9.58190259
LOC_Os03g02920	LOC_Os12g31370	9.43194365
LOC_Os03g03720	LOC_Os06g48200	9.42446308
LOC_Os03g03130	LOC_Os07g31270	9.12201873
LOC_Os03g04110	LOC_Os12g38760	8.83053705
LOC_Os03g03910	LOC_Os04g57410	8.89438196
LOC_Os03g03910	LOC_Os12g06660	9.40640383
LOC_Os03g04169	LOC_Os06g11280	9.14872148
LOC_Os03g04250	LOC_Os11g38959	8.96611152
LOC_Os03g04260	LOC_Os10g38670	9.14493286
LOC_Os03g04250	LOC_Os09g32840	9.09874227
LOC_Os03g04970	LOC_Os08g28800	9.07337106
LOC_Os03g04410	LOC_Os03g12500	9.71905007
LOC_Os03g04970	LOC_Os07g48020	9.47659739
LOC_Os03g04260	LOC_Os04g58110	9.33630539
LOC_Os03g04250	LOC_Os07g01780	8.85221038
LOC_Os03g04410	LOC_Os12g06620	9.02820673
LOC_Os03g04260	LOC_Os11g32520	9.0832676
LOC_Os03g04250	LOC_Os07g02350	9.04243359

InteractorA	InteractorB	Z score
LOC_Os03g04260	LOC_Os03g13800	8.84141934
LOC_Os03g04250	LOC_Os07g48430	9.1630744
LOC_Os03g04410	LOC_Os11g32650	9.42091517
LOC_Os03g04970	LOC_Os06g36820	9.43953449
LOC_Os03g04410	LOC_Os12g23170	9.86211135
LOC_Os03g04410	LOC_Os05g44050	8.97837687
LOC_Os03g04169	LOC_Os05g48510	9.198561
LOC_Os03g04410	LOC_Os03g12660	9.14708409
LOC_Os03g05280	LOC_Os06g36700	8.91605588
LOC_Os03g04970	LOC_Os03g49380	8.81334186
LOC_Os03g04250	LOC_Os05g05800	10.575219
LOC_Os03g04260	LOC_Os12g13800	9.36799777
LOC_Os03g04260	LOC_Os11g03980	9.99344327
LOC_Os03g04260	LOC_Os04g44950	9.29982771
LOC_Os03g04169	LOC_Os06g37080	10.9413242
LOC_Os03g04250	LOC_Os09g31410	9.60596402
LOC_Os03g04169	LOC_Os12g42884	8.81032853
LOC_Os03g04250	LOC_Os09g20220	9.12289871
LOC_Os03g04169	LOC_Os04g53920	10.9333168
LOC_Os03g04250	LOC_Os05g46860	9.81974141
LOC_Os03g04260	LOC_Os09g08720	9.03795451
LOC_Os03g04250	LOC_Os04g16680	10.0302289
LOC_Os03g04260	LOC_Os06g07140	9.9377102
LOC_Os03g04410	LOC_Os04g56230	9.41452796
LOC_Os03g04250	LOC_Os06g40170	9.97381685
LOC_Os03g04250	LOC_Os07g05580	8.93702745
LOC_Os03g04410	LOC_Os05g24580	10.4147344
LOC_Os03g04410	LOC_Os11g47600	9.07538619
LOC_Os03g04260	LOC_Os04g58580	9.81373097
LOC_Os03g04260	LOC_Os04g54330	10.3477171
LOC_Os03g04970	LOC_Os08g38300	8.92959898
LOC_Os03g04250	LOC_Os03g17700	8.82722518
LOC_Os03g04970	LOC_Os10g38540	10.518157
LOC_Os03g04169	LOC_Os07g36140	9.80677809
LOC_Os03g04260	LOC_Os11g08940	11.1974368
LOC_Os03g04250	LOC_Os05g33150	9.05312638
LOC_Os03g04410	LOC_Os05g30480	10.6613992
LOC_Os03g04169	LOC_Os11g37550	9.06860575
LOC_Os03g04169	LOC_Os05g41210	9.4374918
LOC_Os03g04410	LOC_Os03g27370	12.3579357
LOC_Os03g04250	LOC_Os03g58400	9.20147936
LOC_Os03g04169	LOC_Os03g63330	8.96710922
LOC_Os03g04169	LOC_Os12g44020	8.87104892

InteractorA	InteractorB	Z score
LOC_Os03g04169	LOC_Os08g33100	8.86944308
LOC_Os03g04410	LOC_Os04g33720	9.25833008
LOC_Os03g04250	LOC_Os03g07840	9.33027412
LOC_Os03g04410	LOC_Os12g40550	9.04469583
LOC_Os03g04250	LOC_Os09g31502	9.67183376
LOC_Os03g04250	LOC_Os04g33720	9.04718615
LOC_Os03g04970	LOC_Os11g19220	10.9114823
LOC_Os03g04169	LOC_Os05g23740	9.00725292
LOC_Os03g04250	LOC_Os03g50250	9.07023529
LOC_Os03g04260	LOC_Os12g31640	8.82626119
LOC_Os03g04970	LOC_Os08g39860	10.2651134
LOC_Os03g04260	LOC_Os06g36770	8.94259065
LOC_Os03g04169	LOC_Os04g33470	8.82059333
LOC_Os03g04410	LOC_Os11g47570	8.9483034
LOC_Os03g04410	LOC_Os05g40990	9.11184401
LOC_Os03g04260	LOC_Os03g20370	9.51865638
LOC_Os03g04250	LOC_Os05g31140	8.87054196
LOC_Os03g04970	LOC_Os09g28400	9.67147489
LOC_Os03g05280	LOC_Os04g33470	8.83264201
LOC_Os03g04169	LOC_Os11g25260	9.1817227
LOC_Os03g04970	LOC_Os10g30580	9.7947933
LOC_Os03g04250	LOC_Os06g13450	8.97851587
LOC_Os03g04970	LOC_Os12g25690	10.1553906
LOC_Os03g04250	LOC_Os08g41990	9.2102121
LOC_Os03g04169	LOC_Os03g19390	11.2160688
LOC_Os03g04410	LOC_Os07g07320	9.54390956
LOC_Os03g04250	LOC_Os06g11240	8.91291522
LOC_Os03g04260	LOC_Os03g42840	8.88425703
LOC_Os03g04260	LOC_Os05g01970	8.83150451
LOC_Os03g04250	LOC_Os03g05980	8.96611152
LOC_Os03g04169	LOC_Os12g22650	10.9338775
LOC_Os03g04260	LOC_Os11g08440	9.05531935
LOC_Os03g04169	LOC_Os09g31502	9.48191681
LOC_Os03g04260	LOC_Os06g06730	9.66245108
LOC_Os03g04970	LOC_Os08g25734	9.39817738
LOC_Os03g04260	LOC_Os04g58710	9.53941204
LOC_Os03g04169	LOC_Os09g39570	9.46490543
LOC_Os03g04169	LOC_Os07g06970	9.1419615
LOC_Os03g04260	LOC_Os05g05800	8.92329369
LOC_Os03g04250	LOC_Os03g57200	8.85027198
LOC_Os03g04250	LOC_Os09g31490	9.76210032
LOC_Os03g04260	LOC_Os07g14590	11.193568
LOC_Os03g04260	LOC_Os09g34250	8.90956655

InteractorA	InteractorB	Z score
LOC_Os03g04260	LOC_Os11g20790	8.85841792
LOC_Os03g04970	LOC_Os04g44470	10.3297968
LOC_Os03g04250	LOC_Os07g05800	9.92197284
LOC_Os03g04250	LOC_Os09g27420	9.79592808
LOC_Os03g04260	LOC_Os06g25010	8.88402975
LOC_Os03g04169	LOC_Os03g61740	10.529806
LOC_Os03g04169	LOC_Os10g02480	9.26082382
LOC_Os03g04250	LOC_Os09g08072	9.96776387
LOC_Os03g04410	LOC_Os12g02980	8.81240641
LOC_Os03g04250	LOC_Os05g41640	8.93273799
LOC_Os03g04410	LOC_Os03g47770	9.35477511
LOC_Os03g04169	LOC_Os12g02980	9.24195525
LOC_Os03g04260	LOC_Os07g48780	9.51865638
LOC_Os03g04250	LOC_Os11g02130	8.95911343
LOC_Os03g04169	LOC_Os10g38710	8.85722001
LOC_Os03g05290	LOC_Os05g24580	8.95982589
LOC_Os03g04260	LOC_Os03g32050	8.87515595
LOC_Os03g04250	LOC_Os10g42720	9.18246303
LOC_Os03g04169	LOC_Os08g28730	9.88804398
LOC_Os03g05290	LOC_Os05g46360	8.88659777
LOC_Os03g04970	LOC_Os12g10720	9.27477496
LOC_Os03g04970	LOC_Os07g43390	9.99485962
LOC_Os03g04250	LOC_Os07g43670	8.85055621
LOC_Os03g04260	LOC_Os07g28480	9.01145244
LOC_Os03g04970	LOC_Os08g28730	8.9816164
LOC_Os03g04169	LOC_Os10g22450	9.43342589
LOC_Os03g04250	LOC_Os03g21900	9.70742748
LOC_Os03g05730	LOC_Os10g38360	8.98792571
LOC_Os03g06330	LOC_Os05g25490	8.89522434
LOC_Os03g05590	LOC_Os10g38740	9.44541242
LOC_Os03g05730	LOC_Os10g01570	9.03707284
LOC_Os03g05590	LOC_Os05g04690	8.88154191
LOC_Os03g05980	LOC_Os07g09890	9.43254287
LOC_Os03g05590	LOC_Os04g52280	9.49883243
LOC_Os03g05730	LOC_Os04g56210	8.85178342
LOC_Os03g05980	LOC_Os05g50710	8.99246812
LOC_Os03g05730	LOC_Os11g36719	9.02971621
LOC_Os03g05980	LOC_Os03g53200	9.25210494
LOC_Os03g05980	LOC_Os06g45710	8.87394812
LOC_Os03g05980	LOC_Os10g25950	9.3119208
LOC_Os03g05980	LOC_Os04g01250	8.85990741
LOC_Os03g05730	LOC_Os09g36450	9.4288433
LOC_Os03g05730	LOC_Os07g35880	11.1486192

InteractorA	InteractorB	Z score
LOC_Os03g06330	LOC_Os10g10434	10.0994296
LOC_Os03g06200	LOC_Os12g12560	9.29703198
LOC_Os03g05980	LOC_Os03g55800	9.08838236
LOC_Os03g06200	LOC_Os07g34260	9.18941968
LOC_Os03g05590	LOC_Os06g11290	9.26722417
LOC_Os03g05980	LOC_Os04g59200	9.26253478
LOC_Os03g05590	LOC_Os04g56230	9.87342681
LOC_Os03g05590	LOC_Os07g38860	10.5109646
LOC_Os03g06330	LOC_Os04g43750	8.95013978
LOC_Os03g05740	LOC_Os03g49260	8.81240538
LOC_Os03g05290	LOC_Os09g36800	9.8825147
LOC_Os03g05290	LOC_Os10g08710	10.052021
LOC_Os03g05590	LOC_Os12g10730	8.80568881
LOC_Os03g06200	LOC_Os12g02060	9.05692943
LOC_Os03g06200	LOC_Os04g01980	8.98232848
LOC_Os03g06330	LOC_Os07g09890	10.0155587
LOC_Os03g05980	LOC_Os06g51150	9.19332416
LOC_Os03g06200	LOC_Os08g37790	8.81325201
LOC_Os03g06330	LOC_Os03g42110	9.29972435
LOC_Os03g05290	LOC_Os12g02980	9.03729809
LOC_Os03g05740	LOC_Os08g10608	9.54297837
LOC_Os03g05730	LOC_Os03g42110	9.12931131
LOC_Os03g05590	LOC_Os10g21266	9.51168603
LOC_Os03g05740	LOC_Os03g60580	8.81172117
LOC_Os03g05980	LOC_Os07g48050	8.88128673
LOC_Os03g06200	LOC_Os09g36900	10.3316153
LOC_Os03g06200	LOC_Os05g24580	9.19735202
LOC_Os03g05590	LOC_Os09g39500	9.3982463
LOC_Os03g05980	LOC_Os05g38550	9.5277446
LOC_Os03g05980	LOC_Os06g06300	9.92712077
LOC_Os03g05730	LOC_Os11g37550	9.16195493
LOC_Os03g06200	LOC_Os10g30580	9.8114579
LOC_Os03g06200	LOC_Os06g40180	8.88955
LOC_Os03g06330	LOC_Os05g19380	10.8115005
LOC_Os03g05740	LOC_Os08g35740	8.92618027
LOC_Os03g05290	LOC_Os09g10260	9.60879278
LOC_Os03g05590	LOC_Os03g55620	10.604822
LOC_Os03g06200	LOC_Os11g24560	10.700684
LOC_Os03g06200	LOC_Os04g28870	9.05259438
LOC_Os03g06330	LOC_Os05g04340	9.55149648
LOC_Os03g05730	LOC_Os04g39840	11.0663577
LOC_Os03g05980	LOC_Os10g26010	9.1038028
LOC_Os03g05730	LOC_Os06g05690	8.8968849

InteractorA	InteractorB	Z score
LOC_Os03g06200	LOC_Os07g44450	9.14129071
LOC_Os03g05980	LOC_Os04g56920	9.24799788
LOC_Os03g06200	LOC_Os04g12480	8.85666169
LOC_Os03g05590	LOC_Os09g20820	9.19106402
LOC_Os03g05740	LOC_Os09g32800	8.83566736
LOC_Os03g06200	LOC_Os12g37960	9.42512473
LOC_Os03g05590	LOC_Os04g45290	9.83823834
LOC_Os03g05740	LOC_Os10g10434	9.92346373
LOC_Os03g05590	LOC_Os12g37360	8.88836639
LOC_Os03g05980	LOC_Os05g48040	8.83042126
LOC_Os03g05980	LOC_Os03g57130	9.8890882
LOC_Os03g06200	LOC_Os06g35590	8.93422804
LOC_Os03g06330	LOC_Os09g31490	9.8325153
LOC_Os03g05730	LOC_Os09g04730	9.49674968
LOC_Os03g05590	LOC_Os03g13170	9.3982463
LOC_Os03g06200	LOC_Os12g18900	8.86868231
LOC_Os03g06330	LOC_Os10g21240	9.13516899
LOC_Os03g05730	LOC_Os04g35240	8.82886878
LOC_Os03g05980	LOC_Os10g38690	11.7228883
LOC_Os03g06200	LOC_Os07g10660	9.07399622
LOC_Os03g05730	LOC_Os05g30480	9.04290152
LOC_Os03g05730	LOC_Os05g33380	9.03543423
LOC_Os03g05590	LOC_Os06g35530	9.28370474
LOC_Os03g05740	LOC_Os08g09770	9.72485751
LOC_Os03g06330	LOC_Os03g57290	10.2984006
LOC_Os03g06200	LOC_Os04g58710	9.05110872
LOC_Os03g06200	LOC_Os07g42940	8.82175936
LOC_Os03g06200	LOC_Os12g12514	8.92374948
LOC_Os03g05590	LOC_Os11g10510	9.79567906
LOC_Os03g05980	LOC_Os09g39440	8.87077835
LOC_Os03g05590	LOC_Os05g48510	10.2323975
LOC_Os03g06330	LOC_Os03g16980	9.12902186
LOC_Os03g06200	LOC_Os06g06100	8.91888523
LOC_Os03g05590	LOC_Os07g25150	8.88057496
LOC_Os03g05590	LOC_Os07g34589	8.85989888
LOC_Os03g06330	LOC_Os05g37390	10.9994863
LOC_Os03g05980	LOC_Os07g16970	8.93436475
LOC_Os03g05730	LOC_Os04g43800	8.90587488
LOC_Os03g05730	LOC_Os06g11210	9.89930755
LOC_Os03g06330	LOC_Os07g49120	9.54008792
LOC_Os03g05730	LOC_Os10g07040	9.7077014
LOC_Os03g05590	LOC_Os11g42350	9.00956575
LOC_Os03g07150	LOC_Os04g56730	9.72526806

InteractorA	InteractorB	Z score
LOC_Os03g06940	LOC_Os03g16030	8.8960396
LOC_Os03g06940	LOC_Os12g02370	10.2951336
LOC_Os03g07150	LOC_Os03g61280	9.06292108
LOC_Os03g08010	LOC_Os10g41689	11.3605138
LOC_Os03g06940	LOC_Os07g29750	10.3367517
LOC_Os03g06620	LOC_Os07g37790	8.99644112
LOC_Os03g07300	LOC_Os04g33480	8.81416032
LOC_Os03g07300	LOC_Os12g25690	9.32969114
LOC_Os03g07300	LOC_Os05g23860	8.88806842
LOC_Os03g07150	LOC_Os10g39410	9.73068597
LOC_Os03g07150	LOC_Os08g06550	8.89242259
LOC_Os03g06940	LOC_Os09g16910	8.80035463
LOC_Os03g06940	LOC_Os12g13380	9.13808878
LOC_Os03g06940	LOC_Os08g15292	8.83147868
LOC_Os03g06620	LOC_Os04g52340	8.99644112
LOC_Os03g07300	LOC_Os07g16970	9.5175362
LOC_Os03g07150	LOC_Os06g11290	9.5790785
LOC_Os03g07840	LOC_Os09g10230	9.01469983
LOC_Os03g08010	LOC_Os07g41750	9.54827161
LOC_Os03g08010	LOC_Os03g12290	8.90686809
LOC_Os03g06940	LOC_Os03g59700	10.4125895
LOC_Os03g07300	LOC_Os03g60620	9.37220032
LOC_Os03g07840	LOC_Os03g58430	8.9844239
LOC_Os03g06620	LOC_Os05g29880	10.4523587
LOC_Os03g07300	LOC_Os09g25390	8.89715434
LOC_Os03g08010	LOC_Os04g59600	9.18223602
LOC_Os03g07150	LOC_Os05g39050	9.73067442
LOC_Os03g06940	LOC_Os07g38430	11.7522926
LOC_Os03g06620	LOC_Os09g12230	8.99168317
LOC_Os03g07150	LOC_Os05g01810	9.27966954
LOC_Os03g07150	LOC_Os10g21326	9.66347214
LOC_Os03g07150	LOC_Os09g38020	9.73068597
LOC_Os03g08010	LOC_Os04g58710	10.6958908
LOC_Os03g08010	LOC_Os10g38340	9.11764341
LOC_Os03g07150	LOC_Os09g37100	8.95186711
LOC_Os03g07840	LOC_Os10g13800	9.97729789
LOC_Os03g06620	LOC_Os09g38030	8.90267402
LOC_Os03g08010	LOC_Os06g02144	9.72026557
LOC_Os03g06940	LOC_Os06g40640	9.12735538
LOC_Os03g08020	LOC_Os03g12290	8.90687148
LOC_Os03g07150	LOC_Os08g43170	9.28112441
LOC_Os03g06620	LOC_Os10g25140	9.83987986
LOC_Os03g06940	LOC_Os08g17784	9.34091406

InteractorA	InteractorB	Z score
LOC_Os03g08010	LOC_Os09g39810	10.8428322
LOC_Os03g07840	LOC_Os09g39380	9.25482471
LOC_Os03g06620	LOC_Os06g51084	10.9412109
LOC_Os03g08010	LOC_Os12g44030	9.67560256
LOC_Os03g06940	LOC_Os10g02040	9.30142037
LOC_Os03g07150	LOC_Os07g42940	8.88665191
LOC_Os03g06620	LOC_Os03g39710	9.66915479
LOC_Os03g07840	LOC_Os04g41960	10.6636506
LOC_Os03g07150	LOC_Os10g25140	9.00179303
LOC_Os03g06620	LOC_Os10g05069	10.4649485
LOC_Os03g07300	LOC_Os09g08072	9.40515692
LOC_Os03g06940	LOC_Os07g41750	9.47261997
LOC_Os03g06940	LOC_Os05g49880	9.34533285
LOC_Os03g07150	LOC_Os07g43820	9.25381702
LOC_Os03g06620	LOC_Os04g40874	9.52409363
LOC_Os03g07840	LOC_Os03g56460	10.5104188
LOC_Os03g06940	LOC_Os11g08460	10.4781087
LOC_Os03g07150	LOC_Os07g36500	9.73068706
LOC_Os03g07840	LOC_Os06g22140	9.08065525
LOC_Os03g06940	LOC_Os04g33740	8.92762939
LOC_Os03g07840	LOC_Os07g07320	8.85225519
LOC_Os03g07150	LOC_Os12g22680	9.10306848
LOC_Os03g06620	LOC_Os12g41110	9.92224691
LOC_Os03g07150	LOC_Os04g39020	9.24021068
LOC_Os03g07150	LOC_Os10g30840	10.8389276
LOC_Os03g07840	LOC_Os07g05160	10.7804487
LOC_Os03g07840	LOC_Os06g48310	8.96459124
LOC_Os03g08010	LOC_Os06g41810	9.12915702
LOC_Os03g07150	LOC_Os04g49420	9.73068597
LOC_Os03g07300	LOC_Os05g38230	11.5932451
LOC_Os03g07840	LOC_Os10g08580	8.86729643
LOC_Os03g08010	LOC_Os09g04680	9.78440009
LOC_Os03g06940	LOC_Os04g39880	8.94206847
LOC_Os03g06940	LOC_Os07g48020	9.30043748
LOC_Os03g06940	LOC_Os03g56460	8.83809649
LOC_Os03g07840	LOC_Os05g47640	9.78568242
LOC_Os03g06620	LOC_Os07g34260	9.63526036
LOC_Os03g07150	LOC_Os04g58200	8.84319468
LOC_Os03g07150	LOC_Os04g36700	9.6052164
LOC_Os03g07300	LOC_Os03g38020	9.12003083
LOC_Os03g07150	LOC_Os05g45420	9.43176954
LOC_Os03g07840	LOC_Os12g12590	10.8983531
LOC_Os03g07150	LOC_Os03g18570	9.7496474

InteractorA	InteractorB	Z score
LOC_Os03g07150	LOC_Os10g26050	8.8505248
LOC_Os03g07300	LOC_Os04g37950	9.11357536
LOC_Os03g07150	LOC_Os09g26340	9.73068706
LOC_Os03g07840	LOC_Os08g39420	9.01641397
LOC_Os03g07840	LOC_Os12g44030	9.0827088
LOC_Os03g08020	LOC_Os03g16920	8.87415406
LOC_Os03g07840	LOC_Os03g21460	9.48067708
LOC_Os03g07840	LOC_Os03g49220	14.8386544
LOC_Os03g06940	LOC_Os04g55960	9.34824468
LOC_Os03g07150	LOC_Os09g08072	8.95668096
LOC_Os03g07150	LOC_Os05g38740	9.73068597
LOC_Os03g07300	LOC_Os12g34450	9.27912537
LOC_Os03g06940	LOC_Os09g31410	10.6217326
LOC_Os03g07150	LOC_Os04g25990	8.89311316
LOC_Os03g06620	LOC_Os04g46560	9.35832685
LOC_Os03g06620	LOC_Os03g12270	8.92096372
LOC_Os03g08010	LOC_Os03g16920	8.87417285
LOC_Os03g06940	LOC_Os06g49970	9.62359173
LOC_Os03g06620	LOC_Os03g07150	9.35466366
LOC_Os03g07300	LOC_Os06g06730	9.09941519
LOC_Os03g06940	LOC_Os03g52970	8.95157896
LOC_Os03g07840	LOC_Os11g26850	8.85651906
LOC_Os03g06330	LOC_Os11g37640	9.43532834
LOC_Os03g06620	LOC_Os07g34520	9.27838308
LOC_Os03g06940	LOC_Os07g05180	9.69008688
LOC_Os03g07840	LOC_Os05g46550	9.05808488
LOC_Os03g07150	LOC_Os04g16844	9.66346605
LOC_Os03g07150	LOC_Os08g33710	10.4209689
LOC_Os03g08560	LOC_Os09g36800	9.34697451
LOC_Os03g08530	LOC_Os07g38970	9.06981248
LOC_Os03g08530	LOC_Os06g35650	8.9266804
LOC_Os03g08560	LOC_Os03g18570	8.93276643
LOC_Os03g08050	LOC_Os04g58710	10.6958908
LOC_Os03g08020	LOC_Os06g41810	9.12917824
LOC_Os03g08800	LOC_Os03g62670	9.83048687
LOC_Os03g08800	LOC_Os08g41880	8.96718835
LOC_Os03g08570	LOC_Os07g07770	8.86730009
LOC_Os03g08800	LOC_Os07g48040	9.60395832
LOC_Os03g08560	LOC_Os04g33970	9.39452159
LOC_Os03g08280	LOC_Os04g10010	8.91316052
LOC_Os03g08530	LOC_Os06g40190	8.87412508
LOC_Os03g08530	LOC_Os11g41130	10.5973547
LOC_Os03g08530	LOC_Os03g16860	10.0142761

InteractorA	InteractorB	Z score
LOC_Os03g08530	LOC_Os07g42600	11.5006144
LOC_Os03g08530	LOC_Os07g01760	9.74990731
LOC_Os03g08530	LOC_Os07g26150	8.98574463
LOC_Os03g08560	LOC_Os12g06660	9.28648321
LOC_Os03g08530	LOC_Os07g48050	9.54236627
LOC_Os03g08800	LOC_Os06g37150	9.22801471
LOC_Os03g08530	LOC_Os08g09370	8.88771531
LOC_Os03g08800	LOC_Os07g03580	8.81428021
LOC_Os03g08280	LOC_Os11g31530	10.0437704
LOC_Os03g08560	LOC_Os08g42410	9.01221116
LOC_Os03g08020	LOC_Os04g59600	9.18223602
LOC_Os03g08050	LOC_Os03g16920	8.87417285
LOC_Os03g08570	LOC_Os12g25700	9.19519494
LOC_Os03g08280	LOC_Os05g12180	9.13624562
LOC_Os03g08530	LOC_Os10g25130	16.2329004
LOC_Os03g08800	LOC_Os04g39880	9.73573767
LOC_Os03g08020	LOC_Os09g04680	9.78434647
LOC_Os03g08560	LOC_Os04g24430	9.41231197
LOC_Os03g08050	LOC_Os04g59600	9.18226798
LOC_Os03g08020	LOC_Os09g39810	10.8428322
LOC_Os03g08050	LOC_Os07g41750	9.54827161
LOC_Os03g08530	LOC_Os05g39690	8.99074447
LOC_Os03g08800	LOC_Os04g01740	10.240818
LOC_Os03g08050	LOC_Os09g04680	9.78438907
LOC_Os03g08560	LOC_Os07g17010	8.80575555
LOC_Os03g08530	LOC_Os05g07880	9.72138637
LOC_Os03g08020	LOC_Os12g44030	9.67560256
LOC_Os03g08800	LOC_Os08g44810	9.61160466
LOC_Os03g08530	LOC_Os11g08445	9.51968946
LOC_Os03g08530	LOC_Os03g16740	10.2527422
LOC_Os03g08800	LOC_Os07g35940	8.81967088
LOC_Os03g08530	LOC_Os03g16020	9.7734514
LOC_Os03g08050	LOC_Os06g41810	9.12925658
LOC_Os03g08050	LOC_Os10g38340	9.11764341
LOC_Os03g08050	LOC_Os10g41689	11.3605138
LOC_Os03g08530	LOC_Os03g44484	8.81907339
LOC_Os03g08280	LOC_Os04g56070	9.5394134
LOC_Os03g08530	LOC_Os05g08960	9.42552914
LOC_Os03g08800	LOC_Os07g38540	8.89959697
LOC_Os03g08570	LOC_Os05g11550	8.97939507
LOC_Os03g08530	LOC_Os08g40170	9.56439618
LOC_Os03g08280	LOC_Os05g23740	8.93983983
LOC_Os03g08020	LOC_Os06g02144	9.72026557

InteractorA	InteractorB	Z score
LOC_Os03g08530	LOC_Os06g21980	8.82060503
LOC_Os03g08050	LOC_Os03g12290	8.90686809
LOC_Os03g08280	LOC_Os08g38920	9.24168312
LOC_Os03g08530	LOC_Os05g48510	10.785723
LOC_Os03g08530	LOC_Os11g39540	9.40967547
LOC_Os03g08570	LOC_Os04g37950	10.4073793
LOC_Os03g08530	LOC_Os03g15120	9.07964413
LOC_Os03g08530	LOC_Os09g25320	9.01765939
LOC_Os03g08280	LOC_Os08g09940	9.28623096
LOC_Os03g08560	LOC_Os05g10780	8.92718772
LOC_Os03g08560	LOC_Os08g29170	9.70235792
LOC_Os03g08050	LOC_Os06g02144	9.72026557
LOC_Os03g08530	LOC_Os03g18740	9.16413973
LOC_Os03g08570	LOC_Os10g38229	8.83625572
LOC_Os03g08280	LOC_Os05g28280	9.78847051
LOC_Os03g08560	LOC_Os05g39690	9.20339279
LOC_Os03g08530	LOC_Os04g46560	10.175363
LOC_Os03g08050	LOC_Os09g39810	10.8428334
LOC_Os03g08530	LOC_Os07g46630	8.97922955
LOC_Os03g08560	LOC_Os11g26850	10.3175701
LOC_Os03g08530	LOC_Os03g30950	9.24472617
LOC_Os03g08530	LOC_Os09g39780	9.80103097
LOC_Os03g08050	LOC_Os12g44030	9.67560969
LOC_Os03g08800	LOC_Os04g38870	9.05176303
LOC_Os03g08800	LOC_Os06g35560	9.17041164
LOC_Os03g08280	LOC_Os08g10010	9.0249933
LOC_Os03g08020	LOC_Os10g41689	11.3605138
LOC_Os03g08560	LOC_Os05g05470	9.30587219
LOC_Os03g08530	LOC_Os09g32840	9.12844379
LOC_Os03g08280	LOC_Os07g06970	8.8464214
LOC_Os03g08530	LOC_Os10g11260	9.63058603
LOC_Os03g08020	LOC_Os07g41750	9.54827161
LOC_Os03g08530	LOC_Os10g01080	9.69743545
LOC_Os03g08560	LOC_Os05g26890	8.93304135
LOC_Os03g08530	LOC_Os10g38229	9.40537924
LOC_Os03g08530	LOC_Os06g44080	9.01765939
LOC_Os03g08020	LOC_Os04g58710	10.6958908
LOC_Os03g08020	LOC_Os10g38340	9.11764341
LOC_Os03g08530	LOC_Os11g08460	8.82116991
LOC_Os03g10190	LOC_Os08g36900	9.23529665
LOC_Os03g10340	LOC_Os04g10010	9.14542486
LOC_Os03g09080	LOC_Os04g10000	9.81184757
LOC_Os03g09810	LOC_Os11g26850	9.13803892

InteractorA	InteractorB	Z score
LOC_Os03g10190	LOC_Os04g32650	9.85414145
LOC_Os03g09080	LOC_Os07g49220	8.95838191
LOC_Os03g09910	LOC_Os05g45590	9.01080675
LOC_Os03g09250	LOC_Os09g10260	9.83600515
LOC_Os03g09910	LOC_Os06g39230	10.7509872
LOC_Os03g09810	LOC_Os06g11240	8.83978802
LOC_Os03g09810	LOC_Os07g03730	9.82144635
LOC_Os03g09250	LOC_Os03g12270	8.8552781
LOC_Os03g10340	LOC_Os03g16920	9.52327245
LOC_Os03g09250	LOC_Os10g31940	9.59623478
LOC_Os03g09080	LOC_Os08g41880	8.9331333
LOC_Os03g09080	LOC_Os09g10230	9.86450412
LOC_Os03g09910	LOC_Os12g05410	8.98229083
LOC_Os03g10120	LOC_Os08g42540	9.73586706
LOC_Os03g09250	LOC_Os03g27370	9.33036684
LOC_Os03g10340	LOC_Os03g13200	10.8946121
LOC_Os03g09250	LOC_Os10g22450	12.5736194
LOC_Os03g09080	LOC_Os08g14760	9.49000382
LOC_Os03g10120	LOC_Os08g43560	8.91880087
LOC_Os03g09080	LOC_Os04g56730	10.5911832
LOC_Os03g09810	LOC_Os09g10230	9.02967305
LOC_Os03g09810	LOC_Os03g59660	9.02945351
LOC_Os03g09810	LOC_Os06g35520	9.35821198
LOC_Os03g09910	LOC_Os07g22950	8.91624957
LOC_Os03g09810	LOC_Os04g37460	10.7422901
LOC_Os03g09250	LOC_Os12g08280	8.88790004
LOC_Os03g09810	LOC_Os08g04460	9.15615858
LOC_Os03g10190	LOC_Os04g29550	9.84499072
LOC_Os03g09250	LOC_Os05g27940	8.95765738
LOC_Os03g09910	LOC_Os08g06060	9.2354798
LOC_Os03g09810	LOC_Os12g12514	10.4242464
LOC_Os03g09250	LOC_Os10g30200	9.00586953
LOC_Os03g09250	LOC_Os03g13210	9.97636539
LOC_Os03g09810	LOC_Os07g40580	9.74102692
LOC_Os03g09250	LOC_Os04g38220	9.25502852
LOC_Os03g09250	LOC_Os10g38600	8.90669019
LOC_Os03g09810	LOC_Os09g26380	9.06845724
LOC_Os03g09810	LOC_Os03g26450	9.63621737
LOC_Os03g09250	LOC_Os10g21326	9.1650967
LOC_Os03g10120	LOC_Os03g49600	9.74191928
LOC_Os03g09810	LOC_Os04g40310	13.0635284
LOC_Os03g09080	LOC_Os07g08660	9.1218608
LOC_Os03g10340	LOC_Os03g57290	10.0187432

InteractorA	InteractorB	Z score
LOC_Os03g10120	LOC_Os03g37950	9.38482858
LOC_Os03g10120	LOC_Os12g02060	9.03287726
LOC_Os03g09810	LOC_Os06g07140	8.95613209
LOC_Os03g10190	LOC_Os10g39840	8.94588535
LOC_Os03g09910	LOC_Os03g50250	9.19866148
LOC_Os03g09910	LOC_Os06g49470	10.1392146
LOC_Os03g09250	LOC_Os03g60620	9.18114302
LOC_Os03g09080	LOC_Os03g13800	8.96104665
LOC_Os03g10120	LOC_Os07g47490	9.45341266
LOC_Os03g09080	LOC_Os05g49890	9.11704572
LOC_Os03g09910	LOC_Os05g44340	11.3943741
LOC_Os03g09810	LOC_Os04g43400	9.71168848
LOC_Os03g10190	LOC_Os06g15420	8.8660565
LOC_Os03g08800	LOC_Os09g10260	9.33565739
LOC_Os03g09910	LOC_Os03g45960	9.70525184
LOC_Os03g09080	LOC_Os10g28080	9.72319244
LOC_Os03g09910	LOC_Os04g57090	9.60647054
LOC_Os03g10120	LOC_Os10g41510	8.9136805
LOC_Os03g10120	LOC_Os07g16970	9.28745384
LOC_Os03g09250	LOC_Os10g38590	9.86323272
LOC_Os03g10190	LOC_Os03g18810	9.94635337
LOC_Os03g09910	LOC_Os06g02144	8.84101574
LOC_Os03g09250	LOC_Os11g47600	10.0007737
LOC_Os03g10340	LOC_Os04g16826	9.97127908
LOC_Os03g09080	LOC_Os10g21248	9.10207306
LOC_Os03g09810	LOC_Os07g02210	9.50119778
LOC_Os03g09080	LOC_Os08g04540	9.27447718
LOC_Os03g09250	LOC_Os04g16844	9.1650967
LOC_Os03g09810	LOC_Os12g25700	8.91952678
LOC_Os03g09080	LOC_Os04g41960	10.4181331
LOC_Os03g10190	LOC_Os03g58530	9.16431793
LOC_Os03g09810	LOC_Os03g11530	9.84705344
LOC_Os03g09080	LOC_Os07g42950	9.34719286
LOC_Os03g09250	LOC_Os10g38470	10.7000294
LOC_Os03g09250	LOC_Os10g38580	9.40808072
LOC_Os03g09080	LOC_Os08g40140	8.87897995
LOC_Os03g09250	LOC_Os12g02980	10.5750688
LOC_Os03g09080	LOC_Os03g15120	9.92665753
LOC_Os03g10190	LOC_Os05g15520	8.87045052
LOC_Os03g10340	LOC_Os03g15880	9.65118312
LOC_Os03g09250	LOC_Os07g49220	9.29168176
LOC_Os03g09910	LOC_Os08g33370	12.1677028
LOC_Os03g09810	LOC_Os10g26010	10.2294287

InteractorA	InteractorB	Z score
LOC_Os03g09080	LOC_Os06g47320	9.38772614
LOC_Os03g09910	LOC_Os08g09200	10.85844
LOC_Os03g09910	LOC_Os07g28480	10.2260959
LOC_Os03g09910	LOC_Os03g44380	11.826347
LOC_Os03g09910	LOC_Os03g50290	8.93099713
LOC_Os03g09250	LOC_Os07g48020	9.69529396
LOC_Os03g10340	LOC_Os03g27280	11.2011634
LOC_Os03g09250	LOC_Os08g28190	9.10557099
LOC_Os03g09910	LOC_Os03g13210	9.54492764
LOC_Os03g09250	LOC_Os06g28550	9.34614673
LOC_Os03g09910	LOC_Os07g02210	9.12918746
LOC_Os03g09910	LOC_Os05g11550	11.6254572
LOC_Os03g09810	LOC_Os04g37480	8.94733003
LOC_Os03g09810	LOC_Os12g44000	9.02841276
LOC_Os03g09810	LOC_Os10g33900	10.7634788
LOC_Os03g09910	LOC_Os11g31620	8.81554878
LOC_Os03g09810	LOC_Os10g42720	9.55170123
LOC_Os03g09910	LOC_Os08g25570	9.31091864
LOC_Os03g09250	LOC_Os07g40290	8.86267986
LOC_Os03g10190	LOC_Os05g45220	9.19275239
LOC_Os03g10190	LOC_Os04g39210	9.0291573
LOC_Os03g09910	LOC_Os12g38760	9.61356276
LOC_Os03g09910	LOC_Os09g28460	9.20011969
LOC_Os01g12830	LOC_Os12g31640	9.17243382
LOC_Os01g11110	LOC_Os11g36719	9.6087301
LOC_Os01g12830	LOC_Os09g20260	9.9299135
LOC_Os01g12160	LOC_Os02g25940	9.33159579
LOC_Os01g12830	LOC_Os07g08170	8.9509559
LOC_Os01g12580	LOC_Os01g71474	9.12944442
LOC_Os01g12580	LOC_Os07g29750	9.05119611
LOC_Os01g11110	LOC_Os09g20820	8.87364556
LOC_Os01g12580	LOC_Os04g16826	8.95006517
LOC_Os01g12830	LOC_Os04g01674	11.9217818
LOC_Os01g12730	LOC_Os02g46970	9.2081239
LOC_Os01g12830	LOC_Os06g35530	10.743156
LOC_Os01g12160	LOC_Os01g57945	8.94172668
LOC_Os01g12730	LOC_Os06g15420	10.053945
LOC_Os01g12160	LOC_Os10g21326	8.94172668
LOC_Os01g11110	LOC_Os11g48110	8.84064345
LOC_Os01g12160	LOC_Os04g20070	8.95962318
LOC_Os01g12830	LOC_Os02g15640	9.7784034
LOC_Os01g12830	LOC_Os03g17700	9.15210887
LOC_Os01g12830	LOC_Os04g01600	19.8193676

InteractorA	InteractorB	Z score
LOC_Os01g12160	LOC_Os07g42490	9.43098337
LOC_Os01g12830	LOC_Os07g47490	9.68873471
LOC_Os01g12830	LOC_Os07g43390	8.96973031
LOC_Os01g12830	LOC_Os03g26970	9.28098906
LOC_Os01g12160	LOC_Os03g46070	9.59154806
LOC_Os01g12580	LOC_Os11g42350	8.85533274
LOC_Os01g12730	LOC_Os10g13800	9.95753262
LOC_Os01g12160	LOC_Os06g11260	11.1522786
LOC_Os01g12830	LOC_Os08g31060	11.4948849
LOC_Os01g11110	LOC_Os11g19320	8.95974231
LOC_Os01g12580	LOC_Os05g06750	9.28674786
LOC_Os01g12160	LOC_Os08g04560	9.62207415
LOC_Os01g12160	LOC_Os03g32270	10.6372739
LOC_Os01g12580	LOC_Os08g10608	8.92501253
LOC_Os01g12830	LOC_Os12g12560	9.00562014
LOC_Os01g13120	LOC_Os03g26450	8.91048718
LOC_Os01g13120	LOC_Os03g64210	8.90960718
LOC_Os01g12580	LOC_Os05g04450	9.09601435
LOC_Os01g12160	LOC_Os08g44280	8.86506869
LOC_Os01g12730	LOC_Os03g04970	9.22909132
LOC_Os01g12580	LOC_Os03g52460	9.22908696
LOC_Os01g12160	LOC_Os02g10070	9.40220108
LOC_Os01g12730	LOC_Os07g43260	9.011562
LOC_Os01g11110	LOC_Os11g08445	8.98687471
LOC_Os01g12580	LOC_Os07g31270	9.14633624
LOC_Os01g12160	LOC_Os05g46360	9.35155008
LOC_Os01g12730	LOC_Os05g29880	8.88224097
LOC_Os01g12730	LOC_Os06g45070	9.17143179
LOC_Os01g12580	LOC_Os04g17650	9.75410585
LOC_Os01g12830	LOC_Os08g06060	9.62338617
LOC_Os01g12580	LOC_Os02g18930	9.28485644
LOC_Os01g13120	LOC_Os03g30470	9.98989899
LOC_Os01g12580	LOC_Os03g57040	9.41979492
LOC_Os01g12830	LOC_Os12g16250	9.8592592
LOC_Os01g12830	LOC_Os02g39850	8.8316188
LOC_Os01g12580	LOC_Os10g21344	8.95006517
LOC_Os01g12830	LOC_Os01g70220	8.83446552
LOC_Os01g12830	LOC_Os03g04410	9.05480414
LOC_Os01g11110	LOC_Os10g35070	10.3768692
LOC_Os01g12160	LOC_Os04g14680	9.45774283
LOC_Os01g12830	LOC_Os01g14860	10.0840648
LOC_Os01g12580	LOC_Os05g49890	9.33247792
LOC_Os01g12160	LOC_Os03g13150	9.29390015

InteractorA	InteractorB	Z score
LOC_Os01g12580	LOC_Os01g46610	9.10814933
LOC_Os01g12830	LOC_Os06g29180	12.5819082
LOC_Os01g12830	LOC_Os03g31300	9.48596261
LOC_Os01g12580	LOC_Os01g57958	8.95006517
LOC_Os01g11110	LOC_Os10g20910	9.60600188
LOC_Os01g12830	LOC_Os06g11260	8.85084244
LOC_Os01g12160	LOC_Os08g28800	9.16602756
LOC_Os01g12830	LOC_Os06g29220	11.9946293
LOC_Os01g12730	LOC_Os02g12730	9.73510622
LOC_Os01g12160	LOC_Os02g12730	9.01365407
LOC_Os01g12160	LOC_Os04g38870	9.04575796
LOC_Os01g12160	LOC_Os07g08880	9.42815069
LOC_Os01g12830	LOC_Os01g73190	9.68547979
LOC_Os01g12580	LOC_Os06g08080	8.85700701
LOC_Os01g12580	LOC_Os04g50880	8.99710899
LOC_Os01g12580	LOC_Os02g41590	10.1974751
LOC_Os01g12160	LOC_Os04g16844	8.94172668
LOC_Os01g12830	LOC_Os03g63330	10.5220086
LOC_Os01g12580	LOC_Os05g33130	9.94328064
LOC_Os01g12830	LOC_Os07g46460	9.1200317
LOC_Os03g11530	LOC_Os08g04540	9.37426891
LOC_Os03g11420	LOC_Os07g49220	9.75439073
LOC_Os03g10340	LOC_Os08g40140	9.10846698
LOC_Os03g11530	LOC_Os06g35480	11.0536667
LOC_Os03g10940	LOC_Os10g35480	9.48725915
LOC_Os03g10340	LOC_Os06g43640	9.93270818
LOC_Os03g10370	LOC_Os05g23740	8.84793813
LOC_Os03g10340	LOC_Os08g34190	9.2574922
LOC_Os03g10620	LOC_Os09g23530	9.0597848
LOC_Os03g10620	LOC_Os06g37150	10.7871195
LOC_Os03g10940	LOC_Os11g37640	8.95115971
LOC_Os03g10340	LOC_Os10g08022	9.02429926
LOC_Os03g10500	LOC_Os03g59740	9.39306739
LOC_Os03g10620	LOC_Os12g18880	8.8311317
LOC_Os03g11530	LOC_Os03g56410	8.83686157
LOC_Os03g10340	LOC_Os12g44020	9.30839632
LOC_Os03g10340	LOC_Os10g21344	9.97127908
LOC_Os03g10940	LOC_Os07g05400	8.99986816
LOC_Os03g10940	LOC_Os11g32610	9.30033743
LOC_Os03g10940	LOC_Os10g26130	9.20457092
LOC_Os03g10940	LOC_Os03g38000	9.65382374
LOC_Os03g10940	LOC_Os05g48510	8.953163
LOC_Os03g10500	LOC_Os05g36290	8.85521722

InteractorA	InteractorB	Z score
LOC_Os03g11530	LOC_Os04g16844	9.37063233
LOC_Os03g11420	LOC_Os05g41900	9.9071066
LOC_Os03g10620	LOC_Os06g51050	9.0864773
LOC_Os03g10620	LOC_Os05g31040	8.80875849
LOC_Os03g10500	LOC_Os07g34140	9.2040882
LOC_Os03g10370	LOC_Os03g51550	9.93963908
LOC_Os03g10620	LOC_Os07g31830	8.82446927
LOC_Os03g11530	LOC_Os05g29880	8.92212327
LOC_Os03g11420	LOC_Os05g49800	8.82191902
LOC_Os03g10940	LOC_Os03g45410	8.99811906
LOC_Os03g10620	LOC_Os04g52280	9.77889401
LOC_Os03g11530	LOC_Os08g20420	9.46492712
LOC_Os03g10620	LOC_Os05g05830	9.40211773
LOC_Os03g10620	LOC_Os07g29440	8.95045406
LOC_Os03g11420	LOC_Os03g32050	9.26763126
LOC_Os03g10500	LOC_Os05g51570	9.21861186
LOC_Os03g10620	LOC_Os06g36160	9.14143066
LOC_Os03g10620	LOC_Os07g25150	8.99135479
LOC_Os03g10500	LOC_Os04g52280	9.00372807
LOC_Os03g10340	LOC_Os11g26860	8.88386225
LOC_Os03g10620	LOC_Os10g11810	9.13575527
LOC_Os03g10340	LOC_Os08g34290	9.43255779
LOC_Os03g10500	LOC_Os05g03820	9.14220529
LOC_Os03g10620	LOC_Os04g52100	8.86151663
LOC_Os03g10940	LOC_Os12g38180	9.47871733
LOC_Os03g11530	LOC_Os04g42250	9.25946847
LOC_Os03g10620	LOC_Os03g58400	9.22812963
LOC_Os03g10340	LOC_Os07g07060	9.98524037
LOC_Os03g10620	LOC_Os12g02370	9.27821696
LOC_Os03g10620	LOC_Os06g07140	9.44432897
LOC_Os03g10340	LOC_Os09g28770	9.25320799
LOC_Os03g11420	LOC_Os11g19800	8.83391977
LOC_Os03g10940	LOC_Os05g08430	9.1051884
LOC_Os03g10940	LOC_Os09g17620	9.09902198
LOC_Os03g10340	LOC_Os06g40640	8.84939129
LOC_Os03g10340	LOC_Os09g20284	9.16203088
LOC_Os03g10940	LOC_Os12g39630	9.7193045
LOC_Os03g10370	LOC_Os05g48510	9.66991467
LOC_Os03g10940	LOC_Os12g24650	9.20992474
LOC_Os03g11530	LOC_Os06g49970	10.1457145
LOC_Os03g10340	LOC_Os05g28280	9.55307691
LOC_Os03g10340	LOC_Os05g25550	9.35662137
LOC_Os03g10620	LOC_Os05g48510	9.21957309

InteractorA	InteractorB	Z score
LOC_Os03g10940	LOC_Os06g01850	11.079552
LOC_Os03g10620	LOC_Os07g07550	10.3360933
LOC_Os03g10620	LOC_Os12g31370	9.21215434
LOC_Os03g10940	LOC_Os05g41640	9.39686278
LOC_Os03g10620	LOC_Os09g39810	9.40880352
LOC_Os03g10500	LOC_Os09g14670	10.5604879
LOC_Os03g10620	LOC_Os05g33380	10.1163109
LOC_Os03g10620	LOC_Os05g33130	9.1401899
LOC_Os03g11420	LOC_Os09g10230	10.3263332
LOC_Os03g10940	LOC_Os04g43800	10.2147207
LOC_Os03g10940	LOC_Os03g59660	9.82496077
LOC_Os03g10620	LOC_Os05g42150	8.970241
LOC_Os03g10620	LOC_Os05g38550	10.0319043
LOC_Os03g10620	LOC_Os07g26900	10.1682767
LOC_Os03g10620	LOC_Os11g14220	8.95042851
LOC_Os03g10940	LOC_Os06g35630	10.8870422
LOC_Os03g10620	LOC_Os03g18570	9.26606769
LOC_Os03g10620	LOC_Os06g04000	9.96723834
LOC_Os03g11530	LOC_Os06g11210	9.11294299
LOC_Os03g11420	LOC_Os07g05940	9.04048974
LOC_Os03g12290	LOC_Os12g06660	8.83652195
LOC_Os03g11960	LOC_Os10g25130	9.07158646
LOC_Os03g11990	LOC_Os03g60740	8.83840975
LOC_Os03g12290	LOC_Os05g12240	9.76663546
LOC_Os03g12270	LOC_Os11g20790	9.959545
LOC_Os03g12290	LOC_Os08g28820	8.96646948
LOC_Os03g12270	LOC_Os04g59040	10.5561003
LOC_Os03g12270	LOC_Os04g56400	8.93011782
LOC_Os03g12270	LOC_Os05g42150	9.57845753
LOC_Os03g12270	LOC_Os03g60400	9.49886768
LOC_Os03g12270	LOC_Os10g34760	9.2751782
LOC_Os03g12290	LOC_Os12g44150	8.83932857
LOC_Os03g12290	LOC_Os07g35940	9.26028704
LOC_Os03g11990	LOC_Os11g03980	9.74533535
LOC_Os03g12270	LOC_Os12g02980	9.42572793
LOC_Os03g11960	LOC_Os10g14150	10.6546182
LOC_Os03g11970	LOC_Os04g16760	9.74929309
LOC_Os03g12270	LOC_Os05g04470	9.37087868
LOC_Os03g12270	LOC_Os07g26630	8.90585967
LOC_Os03g11970	LOC_Os04g55290	8.95317155
LOC_Os03g12290	LOC_Os03g49600	8.97885796
LOC_Os03g12270	LOC_Os07g44370	10.09793
LOC_Os03g12270	LOC_Os10g37060	9.18037569

InteractorA	InteractorB	Z score
LOC_Os03g11960	LOC_Os12g34380	8.88990696
LOC_Os03g11530	LOC_Os12g06620	9.44725006
LOC_Os03g12270	LOC_Os04g39840	9.43223937
LOC_Os03g11960	LOC_Os03g63330	8.91173454
LOC_Os03g12290	LOC_Os11g25100	9.13483976
LOC_Os03g12270	LOC_Os12g06620	9.44152639
LOC_Os03g11990	LOC_Os09g20820	9.26422689
LOC_Os03g12270	LOC_Os05g45810	9.20479142
LOC_Os03g11960	LOC_Os03g27310	8.89100713
LOC_Os03g12290	LOC_Os03g59020	9.33999226
LOC_Os03g11530	LOC_Os10g21326	9.370652
LOC_Os03g12270	LOC_Os12g22680	10.0816676
LOC_Os03g12270	LOC_Os06g36880	9.08714847
LOC_Os03g11990	LOC_Os10g38160	8.89952016
LOC_Os03g12270	LOC_Os03g56840	9.37380413
LOC_Os03g11960	LOC_Os06g04030	8.89100713
LOC_Os03g11900	LOC_Os07g22950	10.6287043
LOC_Os03g11900	LOC_Os03g58430	9.43432095
LOC_Os03g12290	LOC_Os09g09520	8.95262077
LOC_Os03g12290	LOC_Os07g37320	8.80086529
LOC_Os03g11990	LOC_Os03g56280	8.95168702
LOC_Os03g12270	LOC_Os07g43510	8.83653979
LOC_Os03g12290	LOC_Os04g01250	9.62316482
LOC_Os03g12270	LOC_Os08g09770	8.9938368
LOC_Os03g12290	LOC_Os08g25734	9.47745014
LOC_Os03g11970	LOC_Os05g01675	9.74929309
LOC_Os03g11900	LOC_Os03g57290	8.92842963
LOC_Os03g12270	LOC_Os03g32270	10.0856727
LOC_Os03g11970	LOC_Os03g59060	8.94445777
LOC_Os03g12270	LOC_Os12g32240	9.25887526
LOC_Os03g12270	LOC_Os06g35520	9.62359938
LOC_Os03g12270	LOC_Os07g31270	9.40928334
LOC_Os03g12270	LOC_Os10g38360	9.55129221
LOC_Os03g11970	LOC_Os03g57290	9.61466432
LOC_Os03g12290	LOC_Os06g29844	8.83592202
LOC_Os03g11990	LOC_Os03g62060	9.39679638
LOC_Os03g12270	LOC_Os09g36800	8.91106874
LOC_Os03g12290	LOC_Os05g38550	10.9472435
LOC_Os03g12270	LOC_Os10g38600	8.92386983
LOC_Os03g12290	LOC_Os06g08770	9.63696052
LOC_Os03g12270	LOC_Os04g46620	9.77808302
LOC_Os03g11990	LOC_Os03g50490	9.10829411
LOC_Os03g12290	LOC_Os04g01674	10.30936

InteractorA	InteractorB	Z score
LOC_Os03g11970	LOC_Os10g38710	9.29632668
LOC_Os03g12270	LOC_Os04g01980	9.94682703
LOC_Os03g11970	LOC_Os05g30480	9.39014193
LOC_Os03g12290	LOC_Os07g05160	9.64003764
LOC_Os03g12270	LOC_Os07g34140	9.7602795
LOC_Os03g12290	LOC_Os07g31270	9.19422618
LOC_Os03g11900	LOC_Os03g50490	9.03805976
LOC_Os03g12270	LOC_Os03g26960	9.19622676
LOC_Os03g12270	LOC_Os03g25340	9.67861777
LOC_Os03g11990	LOC_Os08g02410	9.04261006
LOC_Os03g11970	LOC_Os09g30412	9.06157734
LOC_Os03g12270	LOC_Os06g06090	9.61580506
LOC_Os03g12290	LOC_Os03g61340	8.81051109
LOC_Os03g12270	LOC_Os05g33730	8.93155135
LOC_Os03g12270	LOC_Os10g38470	9.35373249
LOC_Os03g12270	LOC_Os07g44740	10.4762373
LOC_Os03g12270	LOC_Os11g25700	8.8569391
LOC_Os03g11970	LOC_Os12g04980	9.64081729
LOC_Os03g12290	LOC_Os06g02380	10.65673
LOC_Os03g12290	LOC_Os04g44920	9.14934135
LOC_Os03g11970	LOC_Os03g17700	9.39618435
LOC_Os03g11970	LOC_Os08g17784	8.80983982
LOC_Os03g12270	LOC_Os07g07709	9.43747572
LOC_Os03g12270	LOC_Os10g38140	9.32012857
LOC_Os03g11970	LOC_Os06g10910	8.9873947
LOC_Os03g12290	LOC_Os05g05620	9.50710665
LOC_Os03g11970	LOC_Os12g40830	9.41214345
LOC_Os03g11970	LOC_Os09g39380	10.6391827
LOC_Os03g12270	LOC_Os05g31110	9.12946684
LOC_Os03g11970	LOC_Os05g07690	10.0044579
LOC_Os03g11990	LOC_Os04g42380	9.32306013
LOC_Os03g11970	LOC_Os03g21260	10.6049947
LOC_Os03g11900	LOC_Os06g36880	9.67260242
LOC_Os03g12270	LOC_Os05g43820	9.94235389
LOC_Os03g12270	LOC_Os10g38350	9.62420357
LOC_Os03g12290	LOC_Os07g30200	9.09001915
LOC_Os03g12270	LOC_Os07g43470	8.85178196
LOC_Os03g11990	LOC_Os06g37660	8.91804333
LOC_Os03g11900	LOC_Os12g22680	9.50136648
LOC_Os03g12270	LOC_Os04g43400	9.28866688
LOC_Os03g12290	LOC_Os06g05700	9.44921133
LOC_Os03g12270	LOC_Os11g10480	8.90079217
LOC_Os03g12270	LOC_Os08g35440	8.84445773

InteractorA	InteractorB	Z score
LOC_Os03g11960	LOC_Os06g35540	9.46433762
LOC_Os03g12270	LOC_Os03g55800	10.1444348
LOC_Os03g12290	LOC_Os08g34210	8.9086624
LOC_Os03g12290	LOC_Os03g27370	8.95177499
LOC_Os03g11970	LOC_Os05g49800	8.95630421
LOC_Os03g12290	LOC_Os09g25370	10.5065746
LOC_Os03g12500	LOC_Os03g58430	8.97849907
LOC_Os03g11990	LOC_Os04g17650	9.20258326
LOC_Os03g12270	LOC_Os03g58050	9.43747119
LOC_Os03g11960	LOC_Os04g39814	9.32719727
LOC_Os03g12270	LOC_Os05g51630	8.89604729
LOC_Os03g12270	LOC_Os04g53240	11.3716805
LOC_Os03g11530	LOC_Os09g30412	11.5003205
LOC_Os03g11960	LOC_Os04g56160	10.0066157
LOC_Os03g12290	LOC_Os10g13800	9.00774139
LOC_Os03g13070	LOC_Os04g41960	9.1627664
LOC_Os03g12510	LOC_Os03g57790	9.53739938
LOC_Os03g12660	LOC_Os06g50300	9.67463737
LOC_Os03g13140	LOC_Os10g10434	9.53118581
LOC_Os03g13150	LOC_Os10g29470	8.93958873
LOC_Os03g13140	LOC_Os07g40290	9.01596864
LOC_Os03g12520	LOC_Os10g40090	10.9769353
LOC_Os03g12660	LOC_Os10g37210	8.80926315
LOC_Os03g13140	LOC_Os06g27770	8.95093152
LOC_Os03g12520	LOC_Os06g40190	9.02060169
LOC_Os03g12510	LOC_Os10g21250	8.91289954
LOC_Os03g12500	LOC_Os12g12580	9.28281038
LOC_Os03g12510	LOC_Os03g52460	9.1240193
LOC_Os03g13070	LOC_Os10g08670	9.2670155
LOC_Os03g12660	LOC_Os03g44150	8.83707128
LOC_Os03g13150	LOC_Os05g48510	8.97537411
LOC_Os03g13140	LOC_Os11g26850	9.00695847
LOC_Os03g13140	LOC_Os03g18570	9.12244177
LOC_Os03g12520	LOC_Os11g08445	9.27909284
LOC_Os03g12660	LOC_Os11g47570	9.68473059
LOC_Os03g12520	LOC_Os03g58630	9.19326135
LOC_Os03g12510	LOC_Os08g28190	8.91120401
LOC_Os03g13070	LOC_Os04g32460	10.5190033
LOC_Os03g12520	LOC_Os08g41880	9.62815085
LOC_Os03g12500	LOC_Os12g13380	9.65364288
LOC_Os03g12520	LOC_Os10g08550	9.92972278
LOC_Os03g12520	LOC_Os09g31486	9.22155666
LOC_Os03g12510	LOC_Os04g54390	9.15932239

InteractorA	InteractorB	Z score
LOC_Os03g12660	LOC_Os05g09490	8.85672812
LOC_Os03g12520	LOC_Os11g32540	8.99922874
LOC_Os03g12660	LOC_Os08g39870	9.06477628
LOC_Os03g13070	LOC_Os12g23170	9.37951515
LOC_Os03g12500	LOC_Os07g35940	9.36654012
LOC_Os03g13140	LOC_Os03g21120	9.01079945
LOC_Os03g12660	LOC_Os03g57040	9.79587098
LOC_Os03g12510	LOC_Os07g43470	9.42714071
LOC_Os03g12520	LOC_Os06g11290	9.51806806
LOC_Os03g12660	LOC_Os04g43760	9.34737012
LOC_Os03g12510	LOC_Os07g05400	8.92402047
LOC_Os03g12500	LOC_Os05g49880	8.84170977
LOC_Os03g13070	LOC_Os03g64330	8.97503813
LOC_Os03g13140	LOC_Os06g24990	9.2382964
LOC_Os03g13140	LOC_Os12g44030	9.88623003
LOC_Os03g12500	LOC_Os07g22950	8.82940505
LOC_Os03g13150	LOC_Os05g03820	8.85771207
LOC_Os03g13070	LOC_Os06g23440	9.20375016
LOC_Os03g12660	LOC_Os11g47590	8.96976441
LOC_Os03g13150	LOC_Os10g25674	9.78018508
LOC_Os03g13150	LOC_Os06g42130	9.75448919
LOC_Os03g12520	LOC_Os05g03480	9.02200601
LOC_Os03g13140	LOC_Os03g61970	8.87961071
LOC_Os03g12500	LOC_Os06g51150	9.99270143
LOC_Os03g12520	LOC_Os09g31410	9.33406152
LOC_Os03g12500	LOC_Os09g23540	9.32120127
LOC_Os03g13070	LOC_Os12g12514	8.8155783
LOC_Os03g13140	LOC_Os05g41210	9.1713005
LOC_Os03g13150	LOC_Os09g38620	9.53363721
LOC_Os03g12520	LOC_Os08g43190	9.34208408
LOC_Os03g12660	LOC_Os06g14510	10.4731309
LOC_Os03g12500	LOC_Os06g11290	10.0845871
LOC_Os03g13070	LOC_Os03g60620	10.4107942
LOC_Os03g13070	LOC_Os03g13300	9.39565524
LOC_Os03g12510	LOC_Os09g35800	9.37648269
LOC_Os03g12500	LOC_Os12g02980	8.94691006
LOC_Os03g13070	LOC_Os04g09540	9.06877647
LOC_Os03g13150	LOC_Os09g38030	8.9628573
LOC_Os03g12520	LOC_Os11g32260	10.2020313
LOC_Os03g12510	LOC_Os10g41490	11.5940909
LOC_Os03g13200	LOC_Os03g25300	9.6504374
LOC_Os03g13300	LOC_Os04g56210	9.60952284
LOC_Os03g13180	LOC_Os06g51084	10.1659447

InteractorA	InteractorB	Z score
LOC_Os03g13200	LOC_Os04g33740	9.13229507
LOC_Os03g13200	LOC_Os08g39140	9.67979043
LOC_Os03g13210	LOC_Os08g08070	9.00125803
LOC_Os03g13180	LOC_Os11g08470	9.1791212
LOC_Os03g13200	LOC_Os10g38470	9.09253627
LOC_Os03g13170	LOC_Os07g28480	9.97362247
LOC_Os03g13170	LOC_Os07g44790	9.24937149
LOC_Os03g13200	LOC_Os12g02080	8.92057566
LOC_Os03g13300	LOC_Os05g31110	9.05459187
LOC_Os03g13180	LOC_Os04g16844	10.6640352
LOC_Os03g13170	LOC_Os07g25590	9.24351951
LOC_Os03g13170	LOC_Os08g34210	9.37063025
LOC_Os03g13170	LOC_Os03g60400	9.57800869
LOC_Os03g13160	LOC_Os08g34280	8.96539027
LOC_Os03g13210	LOC_Os07g49220	8.8320056
LOC_Os03g13170	LOC_Os11g05880	9.29571612
LOC_Os03g13170	LOC_Os07g10660	8.81366605
LOC_Os03g13200	LOC_Os05g26890	9.12717905
LOC_Os03g13180	LOC_Os03g14450	9.54610595
LOC_Os03g13170	LOC_Os12g12560	8.87842676
LOC_Os03g13300	LOC_Os07g32800	10.1064861
LOC_Os03g13170	LOC_Os03g47000	10.0752136
LOC_Os03g13170	LOC_Os11g32260	9.57412981
LOC_Os03g13170	LOC_Os03g55090	9.59070505
LOC_Os03g13170	LOC_Os06g35700	8.98244246
LOC_Os03g13170	LOC_Os05g36010	9.09597411
LOC_Os03g13170	LOC_Os05g41230	9.37399777
LOC_Os03g13200	LOC_Os03g25280	9.6504374
LOC_Os03g13210	LOC_Os11g19800	10.9076055
LOC_Os03g13170	LOC_Os12g23170	10.1310427
LOC_Os03g13170	LOC_Os03g25360	8.92496803
LOC_Os03g13180	LOC_Os04g39840	9.56681346
LOC_Os03g13170	LOC_Os03g28400	9.24938543
LOC_Os03g13170	LOC_Os08g10010	8.88669992
LOC_Os03g13200	LOC_Os03g51600	9.00250232
LOC_Os03g13300	LOC_Os05g43510	10.0653525
LOC_Os03g13200	LOC_Os03g32050	8.88423967
LOC_Os03g13210	LOC_Os03g30870	10.2134664
LOC_Os03g13180	LOC_Os12g36950	10.3491123
LOC_Os03g13170	LOC_Os03g56810	10.2191763
LOC_Os03g13170	LOC_Os03g61340	10.4363056
LOC_Os03g13300	LOC_Os07g37550	8.85307437
LOC_Os03g13200	LOC_Os11g34570	10.0672932

InteractorA	InteractorB	Z score
LOC_Os03g13180	LOC_Os03g45960	9.84869155
LOC_Os03g13170	LOC_Os03g59060	9.00904623
LOC_Os03g13160	LOC_Os04g36700	9.80568356
LOC_Os03g13180	LOC_Os10g08550	9.79352842
LOC_Os03g13170	LOC_Os04g59600	10.1900253
LOC_Os03g13180	LOC_Os10g21326	10.6640352
LOC_Os03g13160	LOC_Os12g16410	9.12385075
LOC_Os03g13160	LOC_Os11g08940	9.19843498
LOC_Os03g13200	LOC_Os12g17910	8.91996383
LOC_Os03g13300	LOC_Os09g34214	9.41474274
LOC_Os03g13200	LOC_Os03g22020	8.95356519
LOC_Os03g13800	LOC_Os05g36280	9.57021141
LOC_Os03g13300	LOC_Os07g09890	9.99188104
LOC_Os03g13200	LOC_Os06g30370	8.91767026
LOC_Os03g13210	LOC_Os04g44470	9.83085189
LOC_Os03g13180	LOC_Os12g10720	9.94107684
LOC_Os03g13300	LOC_Os07g07240	9.26606072
LOC_Os03g13200	LOC_Os03g60400	8.82133563
LOC_Os03g13180	LOC_Os10g39840	10.6529477
LOC_Os03g13200	LOC_Os05g45810	9.43578319
LOC_Os03g13300	LOC_Os10g26130	11.0077568
LOC_Os03g13170	LOC_Os06g35490	10.4844628
LOC_Os03g13200	LOC_Os08g34290	10.1274889
LOC_Os03g13300	LOC_Os04g37500	12.519211
LOC_Os03g13150	LOC_Os11g19320	9.86434504
LOC_Os03g13200	LOC_Os03g50250	9.46996769
LOC_Os03g13300	LOC_Os03g51080	14.6327645
LOC_Os03g13170	LOC_Os05g29880	9.74566769
LOC_Os03g13210	LOC_Os04g27980	9.86206293
LOC_Os03g13200	LOC_Os10g35480	9.15106599
LOC_Os03g13300	LOC_Os05g38760	9.26457532
LOC_Os03g13300	LOC_Os05g28180	8.87836722
LOC_Os03g13200	LOC_Os05g28280	9.71184974
LOC_Os03g13210	LOC_Os03g63720	9.50354845
LOC_Os03g13800	LOC_Os06g06510	9.57021141
LOC_Os03g13180	LOC_Os08g35440	8.89333167
LOC_Os03g13800	LOC_Os06g06460	9.57021141
LOC_Os03g13210	LOC_Os03g58430	9.69110679
LOC_Os03g13300	LOC_Os05g29880	8.84757941
LOC_Os03g13200	LOC_Os10g32680	8.97745057
LOC_Os03g13170	LOC_Os08g29370	9.79092432
LOC_Os03g13170	LOC_Os10g41510	9.36453362
LOC_Os03g13210	LOC_Os06g01610	9.02163726

InteractorA	InteractorB	Z score
LOC_Os03g13210	LOC_Os06g25010	9.83928263
LOC_Os03g14450	LOC_Os03g64030	9.00089891
LOC_Os03g13800	LOC_Os11g05730	9.57021141
LOC_Os03g15120	LOC_Os04g59600	9.52922205
LOC_Os03g15120	LOC_Os04g37820	9.21890579
LOC_Os03g14450	LOC_Os04g55040	8.97453439
LOC_Os03g15120	LOC_Os07g05940	8.90690061
LOC_Os03g15360	LOC_Os11g32260	9.1206879
LOC_Os03g15120	LOC_Os05g37390	8.8715618
LOC_Os03g15120	LOC_Os04g25400	10.1038167
LOC_Os03g15360	LOC_Os09g08120	9.52778478
LOC_Os03g15360	LOC_Os11g47570	9.00893816
LOC_Os03g14530	LOC_Os10g34520	9.37706175
LOC_Os03g15360	LOC_Os03g15880	10.461715
LOC_Os03g14450	LOC_Os03g22530	8.82128002
LOC_Os03g15120	LOC_Os07g03409	10.2026878
LOC_Os03g15120	LOC_Os04g47360	8.97608483
LOC_Os03g15120	LOC_Os05g04340	9.26501764
LOC_Os03g13800	LOC_Os08g44350	9.11807059
LOC_Os03g15360	LOC_Os03g57200	9.36959223
LOC_Os03g15360	LOC_Os03g62060	9.13751252
LOC_Os03g15120	LOC_Os10g40710	9.09960869
LOC_Os03g15360	LOC_Os10g21250	9.65850944
LOC_Os03g15120	LOC_Os11g14040	8.98900512
LOC_Os03g15360	LOC_Os06g36770	9.31704261
LOC_Os03g14450	LOC_Os04g31960	9.12840171
LOC_Os03g15360	LOC_Os09g07830	9.7024825
LOC_Os03g15120	LOC_Os07g09890	10.3542898
LOC_Os03g14450	LOC_Os05g45810	8.84817016
LOC_Os03g15360	LOC_Os05g07090	9.34597981
LOC_Os03g15360	LOC_Os05g09500	8.97026455
LOC_Os03g15120	LOC_Os09g24530	9.9198742
LOC_Os03g15360	LOC_Os08g39420	8.86532816
LOC_Os03g14450	LOC_Os03g24380	8.94349776
LOC_Os03g15360	LOC_Os09g04050	9.48158967
LOC_Os03g15880	LOC_Os03g16220	9.07947583
LOC_Os03g14120	LOC_Os07g30200	9.13017047
LOC_Os03g14530	LOC_Os03g16900	9.73262084
LOC_Os03g15360	LOC_Os06g09450	11.0096153
LOC_Os03g15360	LOC_Os12g13380	9.57386874
LOC_Os03g15120	LOC_Os07g30970	11.2681513
LOC_Os03g13950	LOC_Os06g36670	9.32482825
LOC_Os03g15360	LOC_Os06g30970	10.0123364

InteractorA	InteractorB	Z score
LOC_Os03g15120	LOC_Os06g28550	11.3596774
LOC_Os03g15120	LOC_Os05g33400	9.04029635
LOC_Os03g15880	LOC_Os03g45410	12.5054222
LOC_Os03g15120	LOC_Os04g55850	8.93342221
LOC_Os03g15360	LOC_Os12g42876	9.56137576
LOC_Os03g15120	LOC_Os08g43190	10.6929058
LOC_Os03g14530	LOC_Os08g08070	9.5526646
LOC_Os03g14530	LOC_Os09g33500	9.13588466
LOC_Os03g15360	LOC_Os11g48110	9.40450019
LOC_Os03g15120	LOC_Os03g58260	10.5267432
LOC_Os03g15120	LOC_Os08g34210	9.26736333
LOC_Os03g14450	LOC_Os10g07616	10.5665506
LOC_Os03g14120	LOC_Os08g33820	9.03461444
LOC_Os03g15120	LOC_Os04g34630	8.88319917
LOC_Os03g15360	LOC_Os04g57220	9.11061893
LOC_Os03g15120	LOC_Os09g38030	9.32502852
LOC_Os03g15360	LOC_Os11g03230	9.06840046
LOC_Os03g14530	LOC_Os04g33740	11.4257137
LOC_Os03g13800	LOC_Os09g28770	9.75044482
LOC_Os03g15120	LOC_Os07g26150	9.9048974
LOC_Os03g15360	LOC_Os05g08100	9.21899429
LOC_Os03g15120	LOC_Os07g03499	10.2026956
LOC_Os03g14450	LOC_Os12g14070	9.22072011
LOC_Os03g15120	LOC_Os08g06100	9.58297993
LOC_Os03g15360	LOC_Os04g41310	10.3046041
LOC_Os03g15360	LOC_Os10g21230	9.6232418
LOC_Os03g14530	LOC_Os07g47290	9.05448425
LOC_Os03g13800	LOC_Os11g24560	9.64875433
LOC_Os03g15360	LOC_Os07g47420	9.21035678
LOC_Os03g15120	LOC_Os10g38780	9.18234913
LOC_Os03g15360	LOC_Os10g38274	9.6232418
LOC_Os03g15880	LOC_Os03g16920	8.88322433
LOC_Os03g15360	LOC_Os06g40180	10.1012691
LOC_Os03g15120	LOC_Os12g25120	8.88764482
LOC_Os03g15360	LOC_Os09g09270	8.94727976
LOC_Os03g14450	LOC_Os10g36650	10.1592075
LOC_Os03g15120	LOC_Os07g28480	9.40937992
LOC_Os03g15120	LOC_Os10g32550	10.1758338
LOC_Os03g13950	LOC_Os05g50890	9.42975196
LOC_Os03g14450	LOC_Os03g56410	8.80819348
LOC_Os03g14450	LOC_Os10g30200	9.76836907
LOC_Os03g15120	LOC_Os06g06050	8.88569926
LOC_Os03g15120	LOC_Os05g01490	9.01868231

InteractorA	InteractorB	Z score
LOC_Os03g15120	LOC_Os07g03319	10.2027024
LOC_Os03g15120	LOC_Os03g18580	8.8561133
LOC_Os03g13800	LOC_Os09g15400	9.1410712
LOC_Os03g14450	LOC_Os07g42924	8.86318266
LOC_Os03g15120	LOC_Os04g59040	8.82151428
LOC_Os03g15880	LOC_Os07g44450	9.4019124
LOC_Os03g15960	LOC_Os04g12980	9.67622928
LOC_Os03g16020	LOC_Os10g21298	9.1702552
LOC_Os03g16030	LOC_Os06g05110	9.54808184
LOC_Os03g15960	LOC_Os08g33710	9.00439842
LOC_Os03g15880	LOC_Os10g27050	9.59023194
LOC_Os03g16050	LOC_Os06g35540	9.84606859
LOC_Os03g15880	LOC_Os08g39870	9.5275033
LOC_Os03g16020	LOC_Os10g21266	9.40832054
LOC_Os03g16050	LOC_Os07g43670	9.38402687
LOC_Os03g16050	LOC_Os09g23540	9.14399814
LOC_Os03g16050	LOC_Os11g36719	9.78732552
LOC_Os03g16210	LOC_Os04g36700	8.9926054
LOC_Os03g15960	LOC_Os12g34380	8.84777667
LOC_Os03g16030	LOC_Os03g42220	9.37913811
LOC_Os03g16050	LOC_Os08g44530	9.1658988
LOC_Os03g16050	LOC_Os04g58570	9.06790051
LOC_Os03g16020	LOC_Os05g44340	11.6626018
LOC_Os03g16050	LOC_Os05g42350	10.1515112
LOC_Os03g16050	LOC_Os09g37100	10.9219936
LOC_Os03g16050	LOC_Os06g36840	8.81534094
LOC_Os03g15960	LOC_Os05g45590	10.6323905
LOC_Os03g15960	LOC_Os11g32520	9.54934596
LOC_Os03g15880	LOC_Os07g05400	9.0965574
LOC_Os03g16030	LOC_Os08g44340	8.86021064
LOC_Os03g15960	LOC_Os11g04954	10.4018305
LOC_Os03g15960	LOC_Os07g31830	9.17473871
LOC_Os03g16030	LOC_Os12g13800	8.81661093
LOC_Os03g15880	LOC_Os03g59060	10.0194426
LOC_Os03g16020	LOC_Os12g40510	10.7389121
LOC_Os03g16050	LOC_Os04g56760	10.2648455
LOC_Os03g15880	LOC_Os03g59660	9.3819127
LOC_Os03g15960	LOC_Os09g31410	8.95413804
LOC_Os03g16050	LOC_Os05g30410	10.2648455
LOC_Os03g15960	LOC_Os04g36700	9.26261099
LOC_Os03g16210	LOC_Os09g10200	9.35770552
LOC_Os03g15960	LOC_Os07g31270	9.23928845
LOC_Os03g15960	LOC_Os08g31060	10.4300668

InteractorA	InteractorB	Z score
LOC_Os03g16050	LOC_Os10g21240	9.8703672
LOC_Os03g16050	LOC_Os04g33970	9.04488831
LOC_Os03g16050	LOC_Os03g58260	11.599259
LOC_Os03g16030	LOC_Os03g45960	9.3831317
LOC_Os03g16110	LOC_Os07g42950	8.81295727
LOC_Os03g15880	LOC_Os07g38430	10.8248945
LOC_Os03g16050	LOC_Os05g30480	8.91399599
LOC_Os03g15880	LOC_Os09g31490	8.929983
LOC_Os03g15960	LOC_Os04g33470	9.60685715
LOC_Os03g16210	LOC_Os07g30970	9.34588967
LOC_Os03g16020	LOC_Os08g28820	9.04245937
LOC_Os03g15960	LOC_Os07g47490	8.90603981
LOC_Os03g16030	LOC_Os11g14040	9.31077271
LOC_Os03g16110	LOC_Os07g29750	9.04957144
LOC_Os03g16030	LOC_Os10g38690	8.98541139
LOC_Os03g15960	LOC_Os12g10730	8.99980328
LOC_Os03g15960	LOC_Os11g25700	9.4070146
LOC_Os03g16110	LOC_Os09g19954	9.80407292
LOC_Os03g16050	LOC_Os08g10510	9.66118184
LOC_Os03g16030	LOC_Os04g56070	8.981597
LOC_Os03g16110	LOC_Os04g54390	9.02923949
LOC_Os03g15880	LOC_Os07g38540	10.2992202
LOC_Os03g15880	LOC_Os06g29844	9.92008561
LOC_Os03g16020	LOC_Os04g12970	9.02174652
LOC_Os03g16210	LOC_Os06g46284	9.86632673
LOC_Os03g16050	LOC_Os12g37360	9.27800441
LOC_Os03g15880	LOC_Os10g38950	9.09663117
LOC_Os03g16050	LOC_Os10g08580	9.94609026
LOC_Os03g16210	LOC_Os06g07080	10.3222699
LOC_Os03g16050	LOC_Os05g09500	9.56553243
LOC_Os03g15880	LOC_Os08g34790	9.27330179
LOC_Os03g16030	LOC_Os05g01050	9.21236794
LOC_Os03g16110	LOC_Os12g13390	9.36423465
LOC_Os03g15880	LOC_Os04g56580	9.12543972
LOC_Os03g16030	LOC_Os07g46310	10.5156941
LOC_Os03g16110	LOC_Os11g25700	9.33833487
LOC_Os03g16110	LOC_Os05g47890	8.94953246
LOC_Os03g16020	LOC_Os03g42840	9.07674201
LOC_Os03g15880	LOC_Os08g02400	9.95098327
LOC_Os03g15960	LOC_Os07g47290	9.8269981
LOC_Os03g16050	LOC_Os11g37550	9.32586419
LOC_Os03g15960	LOC_Os12g44010	8.81713898
LOC_Os03g16110	LOC_Os10g38660	8.82185688

InteractorA	InteractorB	Z score
LOC_Os03g15880	LOC_Os08g02410	9.22778164
LOC_Os03g16110	LOC_Os06g45100	8.9595311
LOC_Os03g16020	LOC_Os10g38229	8.89608038
LOC_Os03g16020	LOC_Os08g15322	9.1702552
LOC_Os03g15960	LOC_Os07g07060	8.90493659
LOC_Os03g16030	LOC_Os05g05620	9.18978775
LOC_Os03g16030	LOC_Os08g41880	9.01773086
LOC_Os03g15880	LOC_Os06g05250	8.97923423
LOC_Os03g16030	LOC_Os07g02340	9.61836168
LOC_Os03g16050	LOC_Os09g31486	8.80650141
LOC_Os03g16020	LOC_Os06g39728	9.1702552
LOC_Os03g15880	LOC_Os07g01760	9.92574388
LOC_Os03g16050	LOC_Os06g44270	9.70632274
LOC_Os03g15880	LOC_Os04g40130	9.57100433
LOC_Os03g16030	LOC_Os04g32710	8.99536541
LOC_Os03g16020	LOC_Os12g43370	9.79920804
LOC_Os03g16110	LOC_Os05g48980	9.42224726
LOC_Os03g16110	LOC_Os10g28360	9.71377237
LOC_Os03g16020	LOC_Os04g33970	9.17902829
LOC_Os03g16740	LOC_Os05g49800	9.34872954
LOC_Os03g16860	LOC_Os06g45670	9.09931832
LOC_Os03g16900	LOC_Os03g53650	11.0725693
LOC_Os03g16860	LOC_Os03g42840	9.46936818
LOC_Os03g16880	LOC_Os07g08500	8.83441883
LOC_Os03g16860	LOC_Os12g42280	8.81806545
LOC_Os03g16920	LOC_Os03g62070	9.76802112
LOC_Os03g16880	LOC_Os12g16250	8.99317842
LOC_Os03g16740	LOC_Os05g05800	8.89935647
LOC_Os03g16210	LOC_Os11g03230	10.2911012
LOC_Os03g16740	LOC_Os05g38530	9.01126017
LOC_Os03g16900	LOC_Os04g26870	8.96598319
LOC_Os03g16880	LOC_Os07g48020	9.94871804
LOC_Os03g16210	LOC_Os12g16200	8.82315268
LOC_Os03g16740	LOC_Os07g46630	9.06127094
LOC_Os03g16230	LOC_Os07g03368	9.29930478
LOC_Os03g16880	LOC_Os04g56730	11.1888264
LOC_Os03g16920	LOC_Os05g45810	9.80884959
LOC_Os03g16860	LOC_Os03g57120	10.0966639
LOC_Os03g16860	LOC_Os04g16844	9.33641453
LOC_Os03g16900	LOC_Os06g29180	9.38929061
LOC_Os03g16920	LOC_Os05g36270	8.81632821
LOC_Os03g16860	LOC_Os11g25260	8.84717705
LOC_Os03g16860	LOC_Os07g44790	8.9894973

InteractorA	InteractorB	Z score
LOC_Os03g16220	LOC_Os03g56280	8.86053559
LOC_Os03g16860	LOC_Os10g25950	9.24444316
LOC_Os03g16860	LOC_Os12g13380	8.85710708
LOC_Os03g16900	LOC_Os08g04560	9.47882315
LOC_Os03g16740	LOC_Os10g38160	10.8263318
LOC_Os03g16860	LOC_Os10g29620	9.62818138
LOC_Os03g16740	LOC_Os06g35660	8.88386045
LOC_Os03g16900	LOC_Os07g35880	8.98486253
LOC_Os03g16880	LOC_Os06g10340	8.87514144
LOC_Os03g16860	LOC_Os10g21326	9.33643503
LOC_Os03g16740	LOC_Os06g40180	9.27573248
LOC_Os03g16740	LOC_Os11g32610	8.83417244
LOC_Os03g16880	LOC_Os08g37800	8.88318308
LOC_Os03g16900	LOC_Os05g04690	8.96630815
LOC_Os03g16920	LOC_Os04g58200	9.41158641
LOC_Os03g16860	LOC_Os12g16290	9.11966358
LOC_Os03g16860	LOC_Os09g19560	9.04220798
LOC_Os03g16900	LOC_Os05g38550	9.34849337
LOC_Os03g16900	LOC_Os05g11710	8.84862121
LOC_Os03g16740	LOC_Os09g34214	8.94461572
LOC_Os03g16860	LOC_Os10g38730	9.63223024
LOC_Os03g16230	LOC_Os06g36770	9.0003755
LOC_Os03g16230	LOC_Os06g45590	9.92389739
LOC_Os03g16900	LOC_Os09g32570	8.85634238
LOC_Os03g16900	LOC_Os08g42560	9.33612259
LOC_Os03g16900	LOC_Os10g29470	10.9828409
LOC_Os03g16740	LOC_Os06g35520	8.84438007
LOC_Os03g16740	LOC_Os06g46340	9.68739104
LOC_Os03g16920	LOC_Os03g49610	9.57592571
LOC_Os03g16900	LOC_Os04g54330	9.02718983
LOC_Os03g16900	LOC_Os06g01850	9.36873555
LOC_Os03g16220	LOC_Os07g38540	11.2869957
LOC_Os03g16860	LOC_Os05g33150	10.1466197
LOC_Os03g16900	LOC_Os03g49600	9.46796775
LOC_Os03g16880	LOC_Os10g29620	9.98432185
LOC_Os03g16230	LOC_Os10g05069	8.80375995
LOC_Os03g16880	LOC_Os07g07470	8.83916517
LOC_Os03g16860	LOC_Os12g44000	9.30057866
LOC_Os03g16220	LOC_Os10g27050	9.35425528
LOC_Os03g16220	LOC_Os04g08350	10.3842111
LOC_Os03g16860	LOC_Os04g10000	9.42386529
LOC_Os03g16740	LOC_Os07g38540	10.5958567
LOC_Os03g16860	LOC_Os03g63720	8.88075212

InteractorA	InteractorB	Z score
LOC_Os03g16880	LOC_Os03g23970	9.31567827
LOC_Os03g16880	LOC_Os06g39708	8.91214454
LOC_Os03g16220	LOC_Os12g06620	8.81856907
LOC_Os03g16230	LOC_Os03g61340	9.32621575
LOC_Os03g16220	LOC_Os12g40550	9.43637728
LOC_Os03g16860	LOC_Os09g28420	9.9746645
LOC_Os03g16740	LOC_Os12g12514	9.40226578
LOC_Os03g16740	LOC_Os06g44270	9.67264193
LOC_Os03g16900	LOC_Os12g13390	9.15817749
LOC_Os03g16860	LOC_Os11g43360	9.92184286
LOC_Os03g16740	LOC_Os06g22140	9.78441356
LOC_Os03g16740	LOC_Os03g62700	9.39976629
LOC_Os03g16220	LOC_Os08g35740	8.82391436
LOC_Os03g16880	LOC_Os11g47600	9.36479429
LOC_Os03g16880	LOC_Os07g26540	10.4875776
LOC_Os03g16860	LOC_Os08g14770	10.2346697
LOC_Os03g16880	LOC_Os04g38600	10.0147726
LOC_Os03g16900	LOC_Os07g38860	8.84741885
LOC_Os03g16900	LOC_Os05g07090	9.23278154
LOC_Os03g16880	LOC_Os05g49800	10.8535044
LOC_Os03g16860	LOC_Os04g43800	9.16123848
LOC_Os03g16860	LOC_Os12g40510	9.73736354
LOC_Os03g16220	LOC_Os09g30412	8.8809449
LOC_Os03g16900	LOC_Os04g39900	9.0658789
LOC_Os03g16740	LOC_Os06g39875	9.04420173
LOC_Os03g16220	LOC_Os10g25930	9.81548589
LOC_Os03g16230	LOC_Os05g10780	10.0631619
LOC_Os03g16860	LOC_Os12g38750	9.58127635
LOC_Os03g16740	LOC_Os10g26130	9.54401799
LOC_Os03g16860	LOC_Os08g42730	9.30264389
LOC_Os03g16210	LOC_Os11g14040	8.9068147
LOC_Os03g16880	LOC_Os04g56950	9.46960968
LOC_Os03g16900	LOC_Os06g35730	8.84862597
LOC_Os03g16900	LOC_Os07g43390	10.0040077
LOC_Os03g16900	LOC_Os04g44924	10.6203079
LOC_Os03g16210	LOC_Os12g10730	8.80193613
LOC_Os03g16880	LOC_Os04g12980	9.53634979
LOC_Os03g16860	LOC_Os04g44470	8.87751161
LOC_Os03g16860	LOC_Os07g05580	9.64079813
LOC_Os03g16920	LOC_Os04g12970	9.00846632
LOC_Os03g16740	LOC_Os06g37660	8.87874979
LOC_Os03g16900	LOC_Os11g28340	9.83762813
LOC_Os03g16860	LOC_Os06g01850	9.03087737

InteractorA	InteractorB	Z score
LOC_Os03g16220	LOC_Os08g32620	12.4359105
LOC_Os03g16860	LOC_Os04g41960	8.85447986
LOC_Os03g16860	LOC_Os10g38950	10.6094399
LOC_Os03g16860	LOC_Os09g27820	9.30525653
LOC_Os03g16860	LOC_Os09g23540	9.52040936
LOC_Os03g16880	LOC_Os05g41210	8.86351965
LOC_Os03g16860	LOC_Os03g28400	8.9894973
LOC_Os03g16860	LOC_Os04g30420	12.188307
LOC_Os03g16980	LOC_Os04g44920	10.4297526
LOC_Os03g17470	LOC_Os04g52340	9.002861
LOC_Os03g17310	LOC_Os06g07080	8.91938394
LOC_Os03g17690	LOC_Os03g56790	9.18137103
LOC_Os03g17480	LOC_Os04g12960	9.46369908
LOC_Os03g17310	LOC_Os09g08880	9.14750562
LOC_Os03g17470	LOC_Os09g33500	11.1155191
LOC_Os03g17480	LOC_Os03g61920	8.89819071
LOC_Os03g17700	LOC_Os10g25140	10.1394364
LOC_Os03g16980	LOC_Os07g05160	9.67680119
LOC_Os03g17700	LOC_Os09g08120	8.91402913
LOC_Os03g17690	LOC_Os08g03440	9.15347653
LOC_Os03g17700	LOC_Os05g47640	8.94143214
LOC_Os03g17310	LOC_Os11g04954	9.05666175
LOC_Os03g17470	LOC_Os10g21342	9.03620686
LOC_Os03g16920	LOC_Os11g47570	8.98295367
LOC_Os03g17690	LOC_Os03g63330	9.16674343
LOC_Os03g17470	LOC_Os05g50890	8.86182976
LOC_Os03g17690	LOC_Os05g42350	9.42708343
LOC_Os03g17700	LOC_Os10g11810	10.9381547
LOC_Os03g16920	LOC_Os11g26850	8.87227753
LOC_Os03g17470	LOC_Os03g38980	8.94399302
LOC_Os03g16920	LOC_Os10g02070	10.1587799
LOC_Os03g17690	LOC_Os03g58530	8.950186
LOC_Os03g17470	LOC_Os11g19220	9.48017352
LOC_Os03g17480	LOC_Os08g29170	8.89479143
LOC_Os03g17470	LOC_Os04g12950	9.14941595
LOC_Os03g17470	LOC_Os03g27280	10.0693537
LOC_Os03g17690	LOC_Os06g05700	9.62006217
LOC_Os03g17690	LOC_Os05g50890	9.13017875
LOC_Os03g17690	LOC_Os08g44210	8.91234303
LOC_Os03g16980	LOC_Os12g44010	9.01896533
LOC_Os03g17690	LOC_Os09g36800	11.0215335
LOC_Os03g16920	LOC_Os10g02480	10.155022
LOC_Os03g17470	LOC_Os10g28120	8.95466381

InteractorA	InteractorB	Z score
LOC_Os03g17480	LOC_Os06g21820	9.08410116
LOC_Os03g17700	LOC_Os09g07510	8.99659664
LOC_Os03g17470	LOC_Os07g44550	9.26284931
LOC_Os03g17480	LOC_Os10g07040	8.82849733
LOC_Os03g17700	LOC_Os12g25690	9.31518331
LOC_Os03g17470	LOC_Os12g06620	8.9359044
LOC_Os03g17690	LOC_Os11g03230	10.2436793
LOC_Os03g16920	LOC_Os09g08910	9.02385158
LOC_Os03g17700	LOC_Os05g41180	10.5762747
LOC_Os03g17470	LOC_Os04g16828	9.03620686
LOC_Os03g17700	LOC_Os07g41050	9.1570923
LOC_Os03g17480	LOC_Os03g49260	8.96593424
LOC_Os03g17480	LOC_Os12g23170	8.81702892
LOC_Os03g16980	LOC_Os09g09520	8.96248762
LOC_Os03g17470	LOC_Os07g42950	9.58420242
LOC_Os03g17310	LOC_Os10g38730	10.0059458
LOC_Os03g17480	LOC_Os12g43370	9.63165939
LOC_Os03g17470	LOC_Os05g41210	8.87741883
LOC_Os03g17470	LOC_Os08g42410	12.0525024
LOC_Os03g17470	LOC_Os05g35770	9.12102923
LOC_Os03g17690	LOC_Os04g46560	8.86762815
LOC_Os03g17700	LOC_Os07g41750	8.85278947
LOC_Os03g17690	LOC_Os12g07980	9.20397653
LOC_Os03g17700	LOC_Os03g18130	9.03076137
LOC_Os03g17480	LOC_Os03g18130	10.1210673
LOC_Os03g16980	LOC_Os08g02410	9.15200205
LOC_Os03g17310	LOC_Os04g59450	9.89232358
LOC_Os03g17690	LOC_Os03g62670	9.19964858
LOC_Os03g17690	LOC_Os08g32620	8.85993985
LOC_Os03g17480	LOC_Os08g23730	9.36054584
LOC_Os03g16980	LOC_Os06g35630	9.34324257
LOC_Os03g17690	LOC_Os12g01922	9.36811389
LOC_Os03g17470	LOC_Os05g49770	10.7149491
LOC_Os03g17690	LOC_Os05g05670	8.92372216
LOC_Os03g17470	LOC_Os06g24390	8.8592956
LOC_Os03g17700	LOC_Os04g38600	9.39404918
LOC_Os03g17310	LOC_Os03g46070	9.090079
LOC_Os03g17480	LOC_Os12g17540	9.3214435
LOC_Os03g17480	LOC_Os12g13390	9.08726599
LOC_Os03g17470	LOC_Os03g32170	9.45683058
LOC_Os03g17690	LOC_Os04g16740	8.81367954
LOC_Os03g17310	LOC_Os05g46270	9.66971396
LOC_Os03g17690	LOC_Os10g36650	12.6383774

InteractorA	InteractorB	Z score
LOC_Os03g16920	LOC_Os07g48060	12.0234937
LOC_Os03g17690	LOC_Os11g08340	8.85403683
LOC_Os03g17310	LOC_Os07g44430	9.55583019
LOC_Os03g17470	LOC_Os10g25930	9.31115598
LOC_Os03g16920	LOC_Os12g02060	9.16325426
LOC_Os03g17470	LOC_Os10g21250	9.15086466
LOC_Os03g17310	LOC_Os05g38560	10.344127
LOC_Os03g17480	LOC_Os06g10910	9.10952328
LOC_Os03g17690	LOC_Os09g23560	8.80825274
LOC_Os03g16980	LOC_Os05g51670	9.74156027
LOC_Os03g16980	LOC_Os03g31300	8.85138392
LOC_Os03g17470	LOC_Os05g22722	9.03620686
LOC_Os03g17690	LOC_Os03g38980	9.0596899
LOC_Os03g16980	LOC_Os11g08445	9.35113445
LOC_Os03g17690	LOC_Os03g50440	9.06059959
LOC_Os03g17480	LOC_Os05g12190	9.66863156
LOC_Os03g17470	LOC_Os07g37790	9.00283312
LOC_Os03g17700	LOC_Os11g02600	9.1387349
LOC_Os03g17700	LOC_Os04g52100	8.814155
LOC_Os03g17470	LOC_Os03g62670	9.97217831
LOC_Os03g17690	LOC_Os11g02100	8.88296057
LOC_Os03g17980	LOC_Os03g45960	10.1573341
LOC_Os03g17480	LOC_Os12g39630	9.07572259
LOC_Os03g17700	LOC_Os08g04540	9.05406108
LOC_Os03g17690	LOC_Os03g56460	9.09577254
LOC_Os03g17700	LOC_Os12g13320	9.34158262
LOC_Os03g17690	LOC_Os07g23470	9.16302622
LOC_Os03g16920	LOC_Os10g37210	9.48186888
LOC_Os03g16980	LOC_Os06g35540	8.93545002
LOC_Os03g17470	LOC_Os10g28050	9.53135931
LOC_Os03g17480	LOC_Os10g33900	8.93178717
LOC_Os03g17470	LOC_Os03g55874	9.99639185
LOC_Os03g17470	LOC_Os03g19390	9.331987
LOC_Os03g17480	LOC_Os11g19220	9.59257907
LOC_Os03g17480	LOC_Os06g40170	9.03612312
LOC_Os03g17700	LOC_Os07g11440	9.44463278
LOC_Os03g17470	LOC_Os11g08340	9.18713815
LOC_Os03g17480	LOC_Os04g12980	9.78710236
LOC_Os03g17470	LOC_Os08g15276	9.03620686
LOC_Os03g16920	LOC_Os10g31950	9.40558687
LOC_Os03g17480	LOC_Os10g21248	8.96239023
LOC_Os03g16920	LOC_Os09g31430	9.85763319
LOC_Os03g18740	LOC_Os07g36500	9.04603168

InteractorA	InteractorB	Z score
LOC_Os03g18580	LOC_Os06g36700	10.8123748
LOC_Os03g17980	LOC_Os05g29880	9.0241349
LOC_Os03g18510	LOC_Os03g44170	9.32607437
LOC_Os03g17980	LOC_Os10g38580	8.8606885
LOC_Os03g18570	LOC_Os04g44920	11.3055059
LOC_Os03g18560	LOC_Os03g51550	11.6881211
LOC_Os03g18130	LOC_Os05g45590	9.55326789
LOC_Os03g18130	LOC_Os04g25990	9.25997121
LOC_Os03g18130	LOC_Os09g34214	8.80255448
LOC_Os03g18130	LOC_Os04g01470	9.68269567
LOC_Os03g18560	LOC_Os09g30418	9.3793224
LOC_Os03g18510	LOC_Os08g39420	8.96220583
LOC_Os03g18740	LOC_Os10g39410	9.04603168
LOC_Os03g18570	LOC_Os04g56920	9.48064999
LOC_Os03g17980	LOC_Os08g06060	9.18200796
LOC_Os03g18740	LOC_Os08g25734	9.77320757
LOC_Os03g18580	LOC_Os06g04280	10.3029362
LOC_Os03g18740	LOC_Os03g45410	9.56597272
LOC_Os03g18580	LOC_Os12g10730	8.84243796
LOC_Os03g18130	LOC_Os07g31770	9.26300755
LOC_Os03g18580	LOC_Os11g10520	8.97580852
LOC_Os03g18570	LOC_Os05g47640	9.17618767
LOC_Os03g18560	LOC_Os06g37660	9.3872756
LOC_Os03g18130	LOC_Os07g42940	9.24129819
LOC_Os03g18510	LOC_Os04g56210	9.17099844
LOC_Os03g18570	LOC_Os08g33710	8.92180749
LOC_Os03g18560	LOC_Os09g17620	9.5117564
LOC_Os03g18740	LOC_Os03g48780	10.0201604
LOC_Os03g18560	LOC_Os08g04560	9.08437441
LOC_Os03g18130	LOC_Os03g53650	9.90206062
LOC_Os03g17980	LOC_Os07g48880	9.12395847
LOC_Os03g18130	LOC_Os09g32830	8.96846793
LOC_Os03g18570	LOC_Os11g40140	9.58522292
LOC_Os03g18130	LOC_Os08g06060	9.17360682
LOC_Os03g18740	LOC_Os05g39050	9.04603168
LOC_Os03g18580	LOC_Os11g05290	8.92020472
LOC_Os03g18740	LOC_Os07g38540	9.65604253
LOC_Os03g17980	LOC_Os06g05250	9.02247514
LOC_Os03g18580	LOC_Os12g39630	8.84766477
LOC_Os03g18740	LOC_Os09g38020	9.04603168
LOC_Os03g18740	LOC_Os03g64210	8.91516091
LOC_Os03g18130	LOC_Os03g21260	9.34521432
LOC_Os03g17980	LOC_Os04g58570	9.27144378

InteractorA	InteractorB	Z score
LOC_Os03g18740	LOC_Os04g49420	9.04603168
LOC_Os03g18740	LOC_Os07g26900	9.99132145
LOC_Os03g18740	LOC_Os09g08880	9.54820602
LOC_Os03g18580	LOC_Os06g37660	8.88426937
LOC_Os03g18580	LOC_Os05g04450	8.95477486
LOC_Os03g17980	LOC_Os12g04980	10.162319
LOC_Os03g18580	LOC_Os10g28080	8.89414028
LOC_Os03g17980	LOC_Os07g41050	9.11507554
LOC_Os03g18580	LOC_Os05g04690	8.9213686
LOC_Os03g18580	LOC_Os03g50885	8.97477532
LOC_Os03g18580	LOC_Os04g56580	9.07943641
LOC_Os03g18570	LOC_Os09g35800	8.83180022
LOC_Os03g18130	LOC_Os07g10720	8.84347201
LOC_Os03g18570	LOC_Os03g21260	9.28838636
LOC_Os03g18130	LOC_Os05g44760	9.14413739
LOC_Os03g17980	LOC_Os05g28280	8.82140485
LOC_Os03g18130	LOC_Os08g09200	9.68089367
LOC_Os03g18560	LOC_Os10g41510	9.52296211
LOC_Os03g18130	LOC_Os05g37330	8.97369393
LOC_Os03g18580	LOC_Os07g05160	10.1576536
LOC_Os03g18580	LOC_Os05g37700	9.15081873
LOC_Os03g18580	LOC_Os03g61330	9.36456559
LOC_Os03g18570	LOC_Os04g45470	10.0352699
LOC_Os03g18740	LOC_Os09g26340	9.04603168
LOC_Os03g18570	LOC_Os12g06660	10.1085361
LOC_Os03g18130	LOC_Os05g06300	8.81157872
LOC_Os03g18580	LOC_Os05g04520	12.4033826
LOC_Os03g18580	LOC_Os11g14220	9.42858447
LOC_Os03g18130	LOC_Os03g44170	9.26997103
LOC_Os03g18560	LOC_Os08g20730	9.4709237
LOC_Os03g18580	LOC_Os07g23470	8.95073546
LOC_Os03g18740	LOC_Os04g22730	9.15272839
LOC_Os03g18130	LOC_Os03g63090	9.09315748
LOC_Os03g18130	LOC_Os10g22070	10.3807079
LOC_Os03g18570	LOC_Os04g39900	9.0639103
LOC_Os03g18570	LOC_Os11g14040	9.40107909
LOC_Os03g18740	LOC_Os05g38740	9.04603168
LOC_Os03g18510	LOC_Os04g36700	8.89358279
LOC_Os03g18560	LOC_Os04g12950	8.94008377
LOC_Os03g18510	LOC_Os03g20700	9.26927409
LOC_Os03g18510	LOC_Os05g46550	8.85036875
LOC_Os03g18580	LOC_Os04g46620	10.8656553
LOC_Os03g18510	LOC_Os05g05620	11.5447014

InteractorA	InteractorB	Z score
LOC_Os03g18570	LOC_Os10g22450	9.06119914
LOC_Os03g18130	LOC_Os04g55410	9.89260415
LOC_Os03g18560	LOC_Os05g48510	8.83329387
LOC_Os03g17980	LOC_Os06g11200	9.29437921
LOC_Os03g18560	LOC_Os05g28280	9.56103891
LOC_Os03g18130	LOC_Os07g38430	9.10151472
LOC_Os03g17980	LOC_Os04g58880	9.33649449
LOC_Os03g18130	LOC_Os04g56210	9.20884638
LOC_Os03g18580	LOC_Os12g16250	10.726532
LOC_Os03g18580	LOC_Os10g28120	9.93676029
LOC_Os03g18130	LOC_Os10g38660	9.80072866
LOC_Os03g18580	LOC_Os08g42540	8.87271608
LOC_Os03g18130	LOC_Os04g12970	10.8301691
LOC_Os03g17980	LOC_Os08g10010	10.54872
LOC_Os03g17980	LOC_Os06g08080	8.92862543
LOC_Os03g18570	LOC_Os03g56410	9.85341611
LOC_Os03g18580	LOC_Os11g42350	8.8970476
LOC_Os03g18580	LOC_Os07g43170	8.81903418
LOC_Os03g18130	LOC_Os08g28190	11.3434381
LOC_Os03g19930	LOC_Os11g02440	8.96114633
LOC_Os03g20370	LOC_Os04g08350	9.42204058
LOC_Os03g18810	LOC_Os06g44620	10.0013942
LOC_Os03g20710	LOC_Os03g55800	8.8484106
LOC_Os03g20710	LOC_Os04g57590	9.07537648
LOC_Os03g20700	LOC_Os10g27174	9.79089257
LOC_Os03g20700	LOC_Os09g25320	9.03584583
LOC_Os03g19390	LOC_Os05g05830	9.55826463
LOC_Os03g20370	LOC_Os09g20090	9.31345523
LOC_Os03g21120	LOC_Os07g03288	9.56717976
LOC_Os03g20370	LOC_Os07g20544	10.1057086
LOC_Os03g21120	LOC_Os05g09500	8.93853422
LOC_Os03g20700	LOC_Os05g46040	10.1470476
LOC_Os03g20710	LOC_Os06g06730	8.94674552
LOC_Os03g19390	LOC_Os03g31170	9.50835486
LOC_Os03g19930	LOC_Os03g52860	8.90058383
LOC_Os03g18810	LOC_Os08g31060	8.96094869
LOC_Os03g20700	LOC_Os10g22070	9.95438861
LOC_Os03g20700	LOC_Os06g44080	9.03584583
LOC_Os03g18810	LOC_Os04g16770	10.2742215
LOC_Os03g18810	LOC_Os04g43800	9.23151907
LOC_Os03g20370	LOC_Os05g04690	9.20513309
LOC_Os03g18810	LOC_Os04g53920	9.60875065
LOC_Os03g21120	LOC_Os07g03377	9.56717976

InteractorA	InteractorB	Z score
LOC_Os03g21120	LOC_Os05g47545	9.26878436
LOC_Os03g20370	LOC_Os08g37790	8.97101401
LOC_Os03g20700	LOC_Os07g07709	9.17860221
LOC_Os03g19930	LOC_Os10g38780	9.04321157
LOC_Os03g20700	LOC_Os07g38540	9.43197068
LOC_Os03g19930	LOC_Os08g23730	9.52854253
LOC_Os03g20710	LOC_Os05g34770	8.9177114
LOC_Os03g18810	LOC_Os05g49840	9.9621829
LOC_Os03g20370	LOC_Os03g61600	9.11555363
LOC_Os03g21120	LOC_Os07g05940	9.74817234
LOC_Os03g21120	LOC_Os07g03409	9.26260944
LOC_Os03g20370	LOC_Os04g01470	9.71790977
LOC_Os03g18810	LOC_Os06g21820	9.39926516
LOC_Os03g20370	LOC_Os06g09450	8.99844308
LOC_Os03g18810	LOC_Os09g11230	8.84218673
LOC_Os03g18810	LOC_Os04g39210	9.16235999
LOC_Os03g21120	LOC_Os04g33740	9.98122042
LOC_Os03g18810	LOC_Os03g63330	9.01107587
LOC_Os03g19930	LOC_Os03g57790	9.5507815
LOC_Os03g21120	LOC_Os07g03590	9.56717976
LOC_Os03g20370	LOC_Os05g40420	8.99797057
LOC_Os03g19390	LOC_Os08g39140	8.89077498
LOC_Os03g20700	LOC_Os03g58050	9.17860221
LOC_Os03g20700	LOC_Os06g05110	9.02043938
LOC_Os03g20370	LOC_Os11g48110	8.86935763
LOC_Os03g19390	LOC_Os10g08580	9.26057107
LOC_Os03g20710	LOC_Os04g33480	9.83296867
LOC_Os03g19390	LOC_Os11g04954	10.3488406
LOC_Os03g20370	LOC_Os08g17680	8.93750505
LOC_Os03g20700	LOC_Os12g33610	10.2887262
LOC_Os03g19930	LOC_Os07g10660	9.3512268
LOC_Os03g21120	LOC_Os07g03319	9.26260944
LOC_Os03g20700	LOC_Os04g40874	8.9895832
LOC_Os03g21120	LOC_Os05g46270	10.7599899
LOC_Os03g18810	LOC_Os04g17064	8.97516076
LOC_Os03g20370	LOC_Os12g21798	8.86737368
LOC_Os03g20370	LOC_Os03g61280	9.12123091
LOC_Os03g19930	LOC_Os03g53790	9.58268932
LOC_Os03g18810	LOC_Os09g16910	11.1279188
LOC_Os03g18810	LOC_Os09g24530	9.73159243
LOC_Os03g19930	LOC_Os12g05410	9.551899
LOC_Os03g20370	LOC_Os09g36450	9.67449056
LOC_Os03g19390	LOC_Os07g44550	9.3301073

InteractorA	InteractorB	Z score
LOC_Os03g21120	LOC_Os03g48780	9.90183767
LOC_Os03g20370	LOC_Os07g28480	9.43750023
LOC_Os03g19390	LOC_Os09g25150	9.6991848
LOC_Os03g20710	LOC_Os05g44340	9.68527552
LOC_Os03g20710	LOC_Os05g47545	9.21971359
LOC_Os03g20700	LOC_Os03g52840	9.81057807
LOC_Os03g18810	LOC_Os06g01590	9.36590009
LOC_Os03g18810	LOC_Os03g27370	8.96505067
LOC_Os03g20700	LOC_Os03g52460	9.05483075
LOC_Os03g18810	LOC_Os07g49120	9.10882794
LOC_Os03g21120	LOC_Os07g03499	9.26260944
LOC_Os03g18810	LOC_Os06g06980	8.80139887
LOC_Os03g20700	LOC_Os12g25120	9.32260014
LOC_Os03g19930	LOC_Os03g51550	8.98871406
LOC_Os03g20710	LOC_Os05g12190	8.85597368
LOC_Os03g20370	LOC_Os07g32800	9.4039647
LOC_Os03g21120	LOC_Os07g03467	9.56717976
LOC_Os03g20700	LOC_Os03g48471	9.54414834
LOC_Os03g18810	LOC_Os08g35420	10.2742657
LOC_Os03g18810	LOC_Os05g43510	8.91381013
LOC_Os03g18810	LOC_Os05g11550	8.85126401
LOC_Os03g18810	LOC_Os09g10300	9.21852803
LOC_Os03g21120	LOC_Os03g43890	9.273025
LOC_Os03g20710	LOC_Os06g07080	9.14669156
LOC_Os03g20700	LOC_Os04g53800	9.18585965
LOC_Os03g21120	LOC_Os08g02400	9.2912795
LOC_Os03g20710	LOC_Os03g26960	9.31916244
LOC_Os03g19390	LOC_Os10g26110	9.60024726
LOC_Os03g21120	LOC_Os07g46990	9.14719803
LOC_Os03g19930	LOC_Os04g35200	9.07861563
LOC_Os03g21120	LOC_Os03g38980	8.87355771
LOC_Os03g19930	LOC_Os11g10480	8.98426699
LOC_Os03g19390	LOC_Os10g38234	9.2269604
LOC_Os03g19930	LOC_Os03g38000	9.44925519
LOC_Os03g18810	LOC_Os06g08080	9.86038918
LOC_Os03g21120	LOC_Os05g37690	9.3824199
LOC_Os03g20700	LOC_Os03g53230	9.26212066
LOC_Os03g20370	LOC_Os03g52970	8.82726539
LOC_Os03g19930	LOC_Os12g12470	9.59702147
LOC_Os03g18810	LOC_Os11g24560	8.96633103
LOC_Os03g20370	LOC_Os06g01360	9.27554006
LOC_Os01g13190	LOC_Os08g15030	9.14709293
LOC_Os01g13350	LOC_Os12g16220	10.2470824

InteractorA	InteractorB	Z score
LOC_Os01g13190	LOC_Os11g26850	10.1959812
LOC_Os01g14580	LOC_Os01g16900	9.54024058
LOC_Os01g14040	LOC_Os05g35400	8.9028053
LOC_Os01g13190	LOC_Os02g03870	8.84012059
LOC_Os01g13700	LOC_Os05g49880	10.7002044
LOC_Os01g13700	LOC_Os07g02350	11.5055501
LOC_Os01g13120	LOC_Os07g31770	8.85503002
LOC_Os01g13350	LOC_Os05g25850	8.81278409
LOC_Os01g13120	LOC_Os09g10270	8.98876743
LOC_Os01g13700	LOC_Os03g15960	8.97635126
LOC_Os01g14040	LOC_Os03g58530	9.00029332
LOC_Os01g13190	LOC_Os09g27420	9.04909869
LOC_Os01g13700	LOC_Os02g14160	8.94819995
LOC_Os01g14040	LOC_Os03g45410	10.0819967
LOC_Os01g14580	LOC_Os01g27260	9.73134929
LOC_Os01g13190	LOC_Os03g04260	9.06586258
LOC_Os01g13700	LOC_Os02g08490	8.82939293
LOC_Os01g13350	LOC_Os10g38360	9.40098376
LOC_Os01g13120	LOC_Os11g26850	10.9064321
LOC_Os01g13700	LOC_Os11g16590	9.25151935
LOC_Os01g13700	LOC_Os06g35660	10.0620484
LOC_Os01g13350	LOC_Os10g39170	9.14171298
LOC_Os01g13700	LOC_Os07g43470	9.97465839
LOC_Os01g13190	LOC_Os02g38920	10.33279
LOC_Os01g14580	LOC_Os01g49710	8.85385654
LOC_Os01g13350	LOC_Os05g37390	9.4086492
LOC_Os01g13700	LOC_Os06g46284	9.00064949
LOC_Os01g13190	LOC_Os09g20090	9.30411093
LOC_Os01g13700	LOC_Os07g35880	9.05787951
LOC_Os01g13700	LOC_Os04g44920	9.2770687
LOC_Os01g13700	LOC_Os02g51830	9.24685604
LOC_Os01g14580	LOC_Os02g58340	10.7882284
LOC_Os01g14040	LOC_Os04g36700	10.4087996
LOC_Os01g13700	LOC_Os07g34580	9.41201131
LOC_Os01g14040	LOC_Os03g31750	9.53699252
LOC_Os01g13700	LOC_Os04g58110	9.39075185
LOC_Os01g13350	LOC_Os01g51170	8.87757435
LOC_Os01g14580	LOC_Os01g27360	8.85042452
LOC_Os01g13190	LOC_Os08g06100	9.41572922
LOC_Os01g13190	LOC_Os01g14670	8.99814384
LOC_Os01g13190	LOC_Os06g04620	9.04775242
LOC_Os01g13190	LOC_Os03g55090	9.09193178
LOC_Os01g13350	LOC_Os05g02940	9.26932599

InteractorA	InteractorB	Z score
LOC_Os01g13190	LOC_Os09g27820	8.96244984
LOC_Os01g14580	LOC_Os02g05410	9.66115826
LOC_Os01g13700	LOC_Os03g41460	9.54536635
LOC_Os01g14040	LOC_Os03g30870	8.83779753
LOC_Os01g13700	LOC_Os11g40140	9.85201021
LOC_Os01g13190	LOC_Os01g44130	9.97548797
LOC_Os01g13700	LOC_Os02g12580	9.546843
LOC_Os01g13700	LOC_Os06g14510	9.28591575
LOC_Os01g13190	LOC_Os09g26380	9.49447983
LOC_Os01g13700	LOC_Os03g17700	10.746355
LOC_Os01g13190	LOC_Os10g38780	9.02973715
LOC_Os01g13700	LOC_Os12g14070	8.84769536
LOC_Os01g13700	LOC_Os03g50885	9.00220159
LOC_Os01g13700	LOC_Os07g05400	9.07615217
LOC_Os01g13190	LOC_Os01g53930	9.9776323
LOC_Os01g13190	LOC_Os04g26870	8.99800859
LOC_Os01g13190	LOC_Os04g38220	9.05410291
LOC_Os01g14580	LOC_Os01g73200	8.80978225
LOC_Os01g13350	LOC_Os01g32364	9.51006477
LOC_Os01g13190	LOC_Os10g37060	8.98578248
LOC_Os01g13350	LOC_Os12g23170	9.39750453
LOC_Os01g13190	LOC_Os07g09890	10.5838071
LOC_Os01g13190	LOC_Os06g29180	9.08362668
LOC_Os01g13120	LOC_Os05g48510	9.59716173
LOC_Os01g13120	LOC_Os09g10200	9.26809896
LOC_Os01g13700	LOC_Os05g19150	9.26059278
LOC_Os01g13700	LOC_Os02g47610	8.96391535
LOC_Os01g13190	LOC_Os03g58290	9.13071075
LOC_Os01g13190	LOC_Os03g50885	8.89871619
LOC_Os01g13700	LOC_Os03g42220	9.4802174
LOC_Os01g14040	LOC_Os07g31770	9.49191557
LOC_Os01g13350	LOC_Os01g34480	9.02441705
LOC_Os01g13350	LOC_Os01g73680	9.0639723
LOC_Os01g13350	LOC_Os01g59790	9.12148179
LOC_Os01g13700	LOC_Os01g62870	8.94301941
LOC_Os01g13700	LOC_Os04g37500	9.89050447
LOC_Os01g13700	LOC_Os01g62244	9.51857963
LOC_Os01g13350	LOC_Os11g32580	9.19102556
LOC_Os03g21260	LOC_Os08g20730	9.4930686
LOC_Os03g21260	LOC_Os12g04924	9.27464817
LOC_Os03g21950	LOC_Os04g47170	9.34538912
LOC_Os03g22020	LOC_Os12g12470	8.94899161
LOC_Os03g21900	LOC_Os03g50440	8.95234086

InteractorA	InteractorB	Z score
LOC_Os03g21950	LOC_Os08g43190	9.29950092
LOC_Os03g21260	LOC_Os06g05250	9.21413901
LOC_Os03g21260	LOC_Os06g23440	9.52676016
LOC_Os03g22020	LOC_Os11g08470	9.48696286
LOC_Os03g22060	LOC_Os05g01600	9.20984724
LOC_Os03g21460	LOC_Os03g49350	9.8884562
LOC_Os03g21260	LOC_Os12g38180	10.1531732
LOC_Os03g21260	LOC_Os12g08270	8.84073053
LOC_Os03g21900	LOC_Os08g09240	9.02672982
LOC_Os03g21900	LOC_Os03g52460	9.30183063
LOC_Os03g21260	LOC_Os06g11240	9.20630106
LOC_Os03g21260	LOC_Os06g06300	8.99755295
LOC_Os03g22020	LOC_Os05g23740	9.45123589
LOC_Os03g21900	LOC_Os11g03230	9.10330225
LOC_Os03g21260	LOC_Os04g48540	9.93345681
LOC_Os03g22010	LOC_Os09g39810	11.8515672
LOC_Os03g21260	LOC_Os05g42150	9.13885576
LOC_Os03g22020	LOC_Os04g28870	9.67724881
LOC_Os03g21460	LOC_Os09g39810	8.8229941
LOC_Os03g21120	LOC_Os10g25140	10.8913903
LOC_Os03g21950	LOC_Os12g02980	9.01570443
LOC_Os03g21900	LOC_Os12g36950	9.17361287
LOC_Os03g21460	LOC_Os08g06550	9.29948998
LOC_Os03g21950	LOC_Os11g03290	9.3422013
LOC_Os03g21260	LOC_Os05g05670	9.29987971
LOC_Os03g22060	LOC_Os03g25370	9.29036165
LOC_Os03g22010	LOC_Os07g44590	9.06987538
LOC_Os03g22020	LOC_Os09g28460	9.09940444
LOC_Os03g21900	LOC_Os10g40700	8.8015514
LOC_Os03g21950	LOC_Os03g23970	8.88762191
LOC_Os03g21260	LOC_Os09g28400	9.5083227
LOC_Os03g22010	LOC_Os08g28820	9.366545
LOC_Os03g21260	LOC_Os12g44010	9.07474324
LOC_Os03g21900	LOC_Os04g41310	9.28281671
LOC_Os03g21120	LOC_Os11g33270	8.96347601
LOC_Os03g21260	LOC_Os06g27770	9.23744953
LOC_Os03g21950	LOC_Os05g45590	9.77696259
LOC_Os03g21950	LOC_Os03g49580	9.04892656
LOC_Os03g21260	LOC_Os11g28340	9.33916177
LOC_Os03g22010	LOC_Os06g35520	9.02836538
LOC_Os03g21260	LOC_Os04g52280	8.99252826
LOC_Os03g22010	LOC_Os12g25700	9.77221836
LOC_Os03g21260	LOC_Os09g08720	10.1535127

InteractorA	InteractorB	Z score
LOC_Os03g21260	LOC_Os04g59040	9.80859255
LOC_Os03g21260	LOC_Os04g38220	8.92666468
LOC_Os03g21460	LOC_Os08g25734	9.52722305
LOC_Os03g21900	LOC_Os07g42600	9.15628749
LOC_Os03g22020	LOC_Os06g35940	8.84493416
LOC_Os03g21260	LOC_Os08g03290	9.15456431
LOC_Os03g21260	LOC_Os12g43100	10.1025971
LOC_Os03g22060	LOC_Os03g30950	10.8922442
LOC_Os03g21120	LOC_Os10g32870	9.06276227
LOC_Os03g21260	LOC_Os03g60400	9.75516504
LOC_Os03g22010	LOC_Os08g25734	10.0934973
LOC_Os03g21260	LOC_Os11g03290	9.0539977
LOC_Os03g21900	LOC_Os03g55150	9.01236104
LOC_Os03g21260	LOC_Os08g29520	10.0848691
LOC_Os03g21950	LOC_Os03g61600	8.89924781
LOC_Os03g22010	LOC_Os06g29844	9.01104308
LOC_Os03g21120	LOC_Os11g10510	8.82094005
LOC_Os03g21900	LOC_Os09g39380	10.739941
LOC_Os03g21260	LOC_Os06g02380	9.24172372
LOC_Os03g21260	LOC_Os08g10510	8.84395945
LOC_Os03g22060	LOC_Os05g09500	9.20735629
LOC_Os03g21260	LOC_Os08g36320	9.13405714
LOC_Os03g21260	LOC_Os10g34520	10.1918343
LOC_Os03g21460	LOC_Os07g28280	9.39621491
LOC_Os03g21260	LOC_Os11g10510	8.97253312
LOC_Os03g21260	LOC_Os04g01980	9.57118806
LOC_Os03g22010	LOC_Os04g18650	9.30529953
LOC_Os03g21260	LOC_Os04g29550	8.82039799
LOC_Os03g21260	LOC_Os11g07020	10.3139793
LOC_Os03g21460	LOC_Os04g45490	9.517826
LOC_Os03g22020	LOC_Os09g20820	9.04990005
LOC_Os03g22010	LOC_Os07g42940	9.73359966
LOC_Os03g21120	LOC_Os12g43370	9.08025713
LOC_Os03g22020	LOC_Os04g40290	9.17046424
LOC_Os03g22060	LOC_Os03g42840	9.24441096
LOC_Os03g21460	LOC_Os12g06620	8.99489394
LOC_Os03g22010	LOC_Os04g33970	9.65333659
LOC_Os03g21950	LOC_Os03g42840	9.11721291
LOC_Os03g21260	LOC_Os06g45100	9.30421081
LOC_Os03g21900	LOC_Os04g53290	8.98530927
LOC_Os03g22010	LOC_Os05g38230	8.86437898
LOC_Os03g21260	LOC_Os12g41110	8.89019395
LOC_Os03g21260	LOC_Os10g01570	8.94388043

InteractorA	InteractorB	Z score
LOC_Os03g21260	LOC_Os10g28360	11.1690976
LOC_Os03g21260	LOC_Os05g51670	10.1843288
LOC_Os03g22020	LOC_Os03g55874	9.31404832
LOC_Os03g21260	LOC_Os07g39270	10.8395132
LOC_Os03g21260	LOC_Os08g34170	12.4618889
LOC_Os03g21260	LOC_Os12g02370	8.8013291
LOC_Os03g22020	LOC_Os12g12560	8.88102859
LOC_Os03g22010	LOC_Os04g42250	9.1876984
LOC_Os03g22020	LOC_Os09g32840	8.972599
LOC_Os03g22020	LOC_Os03g60620	9.86564498
LOC_Os03g21900	LOC_Os07g06970	9.17848286
LOC_Os03g21260	LOC_Os04g14680	9.28933357
LOC_Os03g22020	LOC_Os09g30418	9.0247209
LOC_Os03g21950	LOC_Os03g25370	9.7189706
LOC_Os03g21950	LOC_Os06g36160	9.06655787
LOC_Os03g21260	LOC_Os10g33800	9.22389635
LOC_Os03g21120	LOC_Os12g10720	8.84355083
LOC_Os03g22020	LOC_Os08g35740	9.17290823
LOC_Os03g25280	LOC_Os07g44450	9.25329823
LOC_Os03g22060	LOC_Os07g31750	8.84028007
LOC_Os03g25300	LOC_Os04g33970	8.93857127
LOC_Os03g22120	LOC_Os04g01250	10.0522113
LOC_Os03g22530	LOC_Os11g14910	10.3551709
LOC_Os03g22530	LOC_Os06g48160	9.01479668
LOC_Os03g22530	LOC_Os07g05940	9.11991066
LOC_Os03g23970	LOC_Os12g06620	9.3331652
LOC_Os03g23970	LOC_Os09g31506	9.01030057
LOC_Os03g24380	LOC_Os04g02050	9.10638071
LOC_Os03g22120	LOC_Os06g10930	9.7568712
LOC_Os03g22120	LOC_Os06g44620	9.27516219
LOC_Os03g25320	LOC_Os03g38980	9.03712833
LOC_Os03g25280	LOC_Os09g10270	9.86449036
LOC_Os03g22120	LOC_Os12g01922	8.90481108
LOC_Os03g23970	LOC_Os06g46340	9.46448949
LOC_Os03g25300	LOC_Os10g21192	8.83500051
LOC_Os03g23970	LOC_Os10g29470	9.37923185
LOC_Os03g22120	LOC_Os03g50250	8.91301746
LOC_Os03g23970	LOC_Os04g28180	9.58367027
LOC_Os03g25300	LOC_Os03g39710	9.84690708
LOC_Os03g22060	LOC_Os12g40510	11.8356418
LOC_Os03g22120	LOC_Os11g29400	9.38431648
LOC_Os03g25280	LOC_Os04g40290	8.99255118
LOC_Os03g22120	LOC_Os03g49600	9.92141436

InteractorA	InteractorB	Z score
LOC_Os03g25280	LOC_Os03g39710	9.84690893
LOC_Os03g25300	LOC_Os08g27840	9.16293873
LOC_Os03g22060	LOC_Os08g25570	10.719825
LOC_Os03g22060	LOC_Os08g14570	8.90112572
LOC_Os03g22120	LOC_Os05g44340	10.5144428
LOC_Os03g22530	LOC_Os09g10230	10.197032
LOC_Os03g22530	LOC_Os12g23170	10.3093147
LOC_Os03g23970	LOC_Os03g27310	10.9900138
LOC_Os03g22120	LOC_Os04g33720	9.37370564
LOC_Os03g23970	LOC_Os04g26870	9.01447172
LOC_Os03g25280	LOC_Os04g33970	8.93858277
LOC_Os03g22060	LOC_Os12g18880	9.39416638
LOC_Os03g22060	LOC_Os12g31370	9.42253731
LOC_Os03g22060	LOC_Os12g10600	9.817359
LOC_Os03g25300	LOC_Os04g40290	8.99253392
LOC_Os03g23970	LOC_Os10g31940	9.4788448
LOC_Os03g25300	LOC_Os09g10270	9.86448553
LOC_Os03g25300	LOC_Os06g46372	10.8880426
LOC_Os03g25280	LOC_Os04g39880	10.0027599
LOC_Os03g22530	LOC_Os10g30200	12.8883344
LOC_Os03g22060	LOC_Os09g23530	9.23666559
LOC_Os03g22120	LOC_Os07g11440	8.96225063
LOC_Os03g22530	LOC_Os07g20544	8.93888234
LOC_Os03g22120	LOC_Os10g38150	9.39052178
LOC_Os03g22120	LOC_Os08g28800	9.03781657
LOC_Os03g23970	LOC_Os06g04030	10.990012
LOC_Os03g22120	LOC_Os07g42950	10.2130308
LOC_Os03g22120	LOC_Os07g47490	8.83499877
LOC_Os03g25280	LOC_Os06g46372	10.8880404
LOC_Os03g22120	LOC_Os07g06970	9.18342661
LOC_Os03g25320	LOC_Os05g04690	9.3292424
LOC_Os03g23970	LOC_Os06g11210	8.8880076
LOC_Os03g22530	LOC_Os05g51570	10.336243
LOC_Os03g24380	LOC_Os09g08720	9.16416596
LOC_Os03g22060	LOC_Os07g30640	9.85145091
LOC_Os03g25320	LOC_Os03g53860	10.9648596
LOC_Os03g22530	LOC_Os03g50250	9.30041311
LOC_Os03g22120	LOC_Os11g16590	10.0408126
LOC_Os03g22530	LOC_Os05g29880	9.49445919
LOC_Os03g22120	LOC_Os04g57590	9.27160944
LOC_Os03g25300	LOC_Os04g39880	10.0030311
LOC_Os03g23970	LOC_Os05g33570	9.00312541
LOC_Os03g25280	LOC_Os10g21192	8.83501519

InteractorA	InteractorB	Z score
LOC_Os03g22530	LOC_Os06g40640	8.84815732
LOC_Os03g23970	LOC_Os12g03816	9.80116135
LOC_Os03g22060	LOC_Os12g32240	9.23437441
LOC_Os03g23970	LOC_Os06g29180	8.90861957
LOC_Os03g23970	LOC_Os05g44340	9.08103093
LOC_Os03g23970	LOC_Os08g41830	8.9522404
LOC_Os03g22120	LOC_Os05g06450	8.89665764
LOC_Os03g25280	LOC_Os08g27840	9.16293873
LOC_Os03g25300	LOC_Os07g44450	9.25329823
LOC_Os03g25320	LOC_Os05g19380	9.3071521
LOC_Os03g23970	LOC_Os03g43890	9.3086854
LOC_Os03g23970	LOC_Os12g10720	9.45275191
LOC_Os03g22120	LOC_Os04g32650	9.35575413
LOC_Os03g22120	LOC_Os07g05940	9.47575174
LOC_Os03g23970	LOC_Os07g36190	9.9512413
LOC_Os03g22530	LOC_Os06g51084	8.8669736
LOC_Os03g25360	LOC_Os08g43190	9.17901872
LOC_Os03g25340	LOC_Os07g44430	9.1457889
LOC_Os03g25360	LOC_Os05g45590	9.78847301
LOC_Os03g25360	LOC_Os10g26390	10.342512
LOC_Os03g26450	LOC_Os06g35940	9.15145899
LOC_Os03g25360	LOC_Os12g12560	11.0796406
LOC_Os03g25340	LOC_Os07g12730	9.19437954
LOC_Os03g26960	LOC_Os06g11210	9.01323805
LOC_Os03g25360	LOC_Os05g46580	8.87663866
LOC_Os03g26229	LOC_Os07g42940	9.01749291
LOC_Os03g26229	LOC_Os04g48850	8.95335796
LOC_Os03g25340	LOC_Os09g31430	10.481497
LOC_Os03g26960	LOC_Os12g44020	8.81748279
LOC_Os03g25370	LOC_Os12g44000	9.22689135
LOC_Os03g25340	LOC_Os08g04560	9.32665078
LOC_Os03g25370	LOC_Os03g45320	9.55294095
LOC_Os03g25320	LOC_Os07g41750	10.0897902
LOC_Os03g25340	LOC_Os03g63330	9.44144283
LOC_Os03g25340	LOC_Os09g23530	9.19962296
LOC_Os03g25360	LOC_Os11g10480	9.27322613
LOC_Os03g25370	LOC_Os03g49380	9.73202041
LOC_Os03g26450	LOC_Os03g58050	9.19624496
LOC_Os03g26970	LOC_Os03g50440	9.91505072
LOC_Os03g25340	LOC_Os05g41230	9.75415576
LOC_Os03g26970	LOC_Os04g33740	10.9284486
LOC_Os03g26960	LOC_Os07g05160	9.53150811
LOC_Os03g25320	LOC_Os08g29170	8.98468597

InteractorA	InteractorB	Z score
LOC_Os03g25360	LOC_Os04g55290	9.44330697
LOC_Os03g26960	LOC_Os04g43400	9.20648557
LOC_Os03g25360	LOC_Os06g11290	9.95743593
LOC_Os03g25370	LOC_Os04g37820	8.88435676
LOC_Os03g25370	LOC_Os07g46310	9.44490285
LOC_Os03g26229	LOC_Os12g40510	9.29941042
LOC_Os03g26450	LOC_Os10g40700	9.7867945
LOC_Os03g26960	LOC_Os06g02390	10.5281539
LOC_Os03g26450	LOC_Os07g07709	9.19624496
LOC_Os03g25360	LOC_Os04g12970	9.14017054
LOC_Os03g26450	LOC_Os06g11280	9.18373871
LOC_Os03g25320	LOC_Os05g51480	8.94486145
LOC_Os03g26229	LOC_Os05g48510	9.64308419
LOC_Os03g26970	LOC_Os04g42920	10.8996673
LOC_Os03g25340	LOC_Os06g23780	9.13975225
LOC_Os03g25370	LOC_Os05g22724	8.87895201
LOC_Os03g26450	LOC_Os06g45710	9.12715198
LOC_Os03g25340	LOC_Os04g59160	9.38688451
LOC_Os03g25360	LOC_Os05g44760	9.51997522
LOC_Os03g26450	LOC_Os11g08330	11.0605177
LOC_Os03g26960	LOC_Os04g40130	10.1689746
LOC_Os03g26960	LOC_Os09g26880	10.60456
LOC_Os03g25340	LOC_Os05g38760	8.97940451
LOC_Os03g25360	LOC_Os07g46280	8.88324016
LOC_Os03g26960	LOC_Os06g02380	9.7781446
LOC_Os03g25340	LOC_Os07g23730	10.3708715
LOC_Os03g25340	LOC_Os06g12790	10.4349364
LOC_Os03g26450	LOC_Os04g10010	10.5015874
LOC_Os03g26450	LOC_Os09g30360	9.22153687
LOC_Os03g26960	LOC_Os10g05069	9.23306964
LOC_Os03g25360	LOC_Os09g39500	8.92496803
LOC_Os03g25370	LOC_Os09g14670	8.98757478
LOC_Os03g25360	LOC_Os04g42920	9.00857604
LOC_Os03g26970	LOC_Os04g29550	10.0380978
LOC_Os03g26960	LOC_Os04g53290	8.9760279
LOC_Os03g25360	LOC_Os05g40990	9.76987744
LOC_Os03g26960	LOC_Os10g27190	8.97126199
LOC_Os03g26450	LOC_Os04g37480	10.2508523
LOC_Os03g25360	LOC_Os04g02820	9.84980531
LOC_Os03g26960	LOC_Os09g25390	9.66336962
LOC_Os03g26450	LOC_Os11g05470	8.93313802
LOC_Os03g26450	LOC_Os05g49800	9.87908808
LOC_Os03g26960	LOC_Os11g32650	8.98853658

InteractorA	InteractorB	Z score
LOC_Os03g25360	LOC_Os12g34062	8.81147664
LOC_Os03g25370	LOC_Os09g23530	12.2759698
LOC_Os03g25320	LOC_Os12g38750	9.29618569
LOC_Os03g25370	LOC_Os06g23780	8.94760271
LOC_Os03g26450	LOC_Os12g16410	8.92418481
LOC_Os03g25340	LOC_Os09g36830	8.85935095
LOC_Os03g26450	LOC_Os12g34380	9.43420852
LOC_Os03g25370	LOC_Os04g45290	9.78255826
LOC_Os03g26960	LOC_Os11g32520	9.67620471
LOC_Os03g25340	LOC_Os06g35650	10.0646368
LOC_Os03g25360	LOC_Os04g45290	9.08425862
LOC_Os03g25360	LOC_Os06g44620	9.4775607
LOC_Os03g26450	LOC_Os08g34190	9.22259801
LOC_Os03g25360	LOC_Os12g12580	10.5761971
LOC_Os03g25370	LOC_Os04g38600	9.28781345
LOC_Os03g26450	LOC_Os04g58640	9.20792904
LOC_Os03g26960	LOC_Os05g34170	9.13494497
LOC_Os03g26450	LOC_Os04g40950	9.48981033
LOC_Os03g25360	LOC_Os06g23780	8.9776821
LOC_Os03g26960	LOC_Os07g44370	9.83189744
LOC_Os03g25340	LOC_Os12g12560	9.31260178
LOC_Os03g26960	LOC_Os03g49600	9.20316165
LOC_Os03g27280	LOC_Os07g36130	8.8637278
LOC_Os03g28330	LOC_Os03g62700	9.00240959
LOC_Os03g27310	LOC_Os10g08022	8.8771383
LOC_Os03g27280	LOC_Os06g11210	9.14509922
LOC_Os03g27370	LOC_Os04g33570	9.0064451
LOC_Os03g26970	LOC_Os10g25140	9.56763441
LOC_Os03g27260	LOC_Os03g63330	9.43748468
LOC_Os03g27310	LOC_Os05g41080	13.2611391
LOC_Os03g28330	LOC_Os04g39880	9.30525602
LOC_Os03g27280	LOC_Os03g59710	9.61686034
LOC_Os03g27280	LOC_Os10g29470	9.94485895
LOC_Os03g27310	LOC_Os08g28730	8.87512854
LOC_Os03g27310	LOC_Os03g46060	9.2525472
LOC_Os03g27370	LOC_Os04g58710	9.97462258
LOC_Os03g27310	LOC_Os04g58710	8.85756566
LOC_Os03g27310	LOC_Os11g03230	9.07767105
LOC_Os03g27260	LOC_Os03g62060	9.58072742
LOC_Os03g27310	LOC_Os05g25850	10.1402595
LOC_Os03g28400	LOC_Os05g37700	9.54843905
LOC_Os03g27310	LOC_Os11g01872	9.64035994
LOC_Os03g27280	LOC_Os06g27770	9.67618039

InteractorA	InteractorB	Z score
LOC_Os03g27260	LOC_Os05g03480	9.06203441
LOC_Os03g27370	LOC_Os04g43410	9.31722019
LOC_Os03g28330	LOC_Os05g45420	9.7573065
LOC_Os03g28400	LOC_Os12g41110	8.81320956
LOC_Os03g27260	LOC_Os12g38180	9.40725115
LOC_Os03g28400	LOC_Os04g37820	8.96388726
LOC_Os03g28400	LOC_Os05g04690	9.67955446
LOC_Os03g27310	LOC_Os09g28400	9.22810199
LOC_Os03g27260	LOC_Os04g45470	10.1259938
LOC_Os03g27310	LOC_Os04g56730	8.97547959
LOC_Os03g27310	LOC_Os03g55070	9.18268536
LOC_Os03g27310	LOC_Os12g22680	8.99889434
LOC_Os03g27370	LOC_Os08g44530	8.83626706
LOC_Os03g27370	LOC_Os06g35660	9.66472262
LOC_Os03g28330	LOC_Os03g60620	9.85478926
LOC_Os03g27260	LOC_Os08g42910	9.87406459
LOC_Os03g27280	LOC_Os08g06100	8.91369323
LOC_Os03g28400	LOC_Os12g34450	9.01882271
LOC_Os03g27260	LOC_Os03g52970	8.96531833
LOC_Os03g28330	LOC_Os11g19800	10.5014625
LOC_Os03g28330	LOC_Os08g44530	9.73401781
LOC_Os03g28330	LOC_Os11g07020	9.285165
LOC_Os03g27370	LOC_Os10g41510	9.65601275
LOC_Os03g28400	LOC_Os08g37490	10.1195673
LOC_Os03g28400	LOC_Os10g37210	9.06321999
LOC_Os03g28330	LOC_Os03g60870	11.5537302
LOC_Os03g28330	LOC_Os11g32610	9.20975556
LOC_Os03g27310	LOC_Os06g11800	9.43562976
LOC_Os03g27370	LOC_Os04g39880	9.59152869
LOC_Os03g27370	LOC_Os06g35520	9.41532917
LOC_Os03g27260	LOC_Os05g42350	9.20461157
LOC_Os03g27310	LOC_Os06g27770	9.95561971
LOC_Os03g28400	LOC_Os04g40130	10.5296736
LOC_Os03g28400	LOC_Os09g39500	9.24938543
LOC_Os03g28330	LOC_Os10g11810	8.83543609
LOC_Os03g27310	LOC_Os12g12560	9.96737247
LOC_Os03g27370	LOC_Os07g38890	9.18619663
LOC_Os03g27310	LOC_Os03g48750	11.3065848
LOC_Os03g27280	LOC_Os04g33720	8.98317379
LOC_Os03g27370	LOC_Os09g10300	9.36484601
LOC_Os03g28400	LOC_Os04g39210	9.82110183
LOC_Os03g27260	LOC_Os07g39270	9.07639468
LOC_Os03g27370	LOC_Os10g25130	9.27840749

InteractorA	InteractorB	Z score
LOC_Os03g28330	LOC_Os07g09890	8.86431854
LOC_Os03g27260	LOC_Os07g46310	10.0724018
LOC_Os03g27370	LOC_Os12g43100	8.8387504
LOC_Os03g27310	LOC_Os05g41610	9.13173855
LOC_Os03g27310	LOC_Os05g38230	8.90528424
LOC_Os03g27370	LOC_Os11g47760	8.89697862
LOC_Os03g28400	LOC_Os09g39440	8.88979566
LOC_Os03g27280	LOC_Os04g20810	9.27516479
LOC_Os03g27310	LOC_Os04g45290	9.10759449
LOC_Os03g28330	LOC_Os11g01872	8.91907355
LOC_Os03g28400	LOC_Os05g33150	9.9427286
LOC_Os03g26970	LOC_Os09g11230	9.02263326
LOC_Os03g27260	LOC_Os12g38770	11.6563097
LOC_Os03g27280	LOC_Os06g37660	8.82158806
LOC_Os03g27310	LOC_Os03g48760	9.00357994
LOC_Os03g27370	LOC_Os07g10720	9.45052716
LOC_Os03g27310	LOC_Os07g05160	9.72198405
LOC_Os03g27370	LOC_Os06g21570	8.88147333
LOC_Os03g28400	LOC_Os04g52100	9.20225793
LOC_Os03g27280	LOC_Os08g09250	9.12622412
LOC_Os03g27260	LOC_Os07g06440	11.0344011
LOC_Os03g28400	LOC_Os08g37800	9.50435258
LOC_Os03g27310	LOC_Os08g39140	8.88560886
LOC_Os03g27310	LOC_Os07g35880	10.4526815
LOC_Os03g27280	LOC_Os05g19380	8.95593862
LOC_Os03g27370	LOC_Os05g19380	8.8206468
LOC_Os03g26970	LOC_Os06g35490	10.5507705
LOC_Os03g27310	LOC_Os06g12090	8.81349276
LOC_Os03g28400	LOC_Os05g08430	9.73728633
LOC_Os03g27370	LOC_Os05g22724	9.03482041
LOC_Os03g26970	LOC_Os08g39300	9.31826672
LOC_Os03g26970	LOC_Os11g05880	8.85651942
LOC_Os03g27260	LOC_Os03g55600	9.09243668
LOC_Os03g28400	LOC_Os11g47570	9.23867108
LOC_Os03g28400	LOC_Os12g31640	8.81498365
LOC_Os03g27370	LOC_Os07g04240	9.39835121
LOC_Os03g27310	LOC_Os06g49970	9.41132242
LOC_Os03g28400	LOC_Os06g47320	10.6619627
LOC_Os03g28330	LOC_Os10g38140	9.0252809
LOC_Os03g28330	LOC_Os07g48010	9.03942851
LOC_Os03g28400	LOC_Os04g40310	9.00384069
LOC_Os03g28330	LOC_Os03g44150	9.82969258
LOC_Os03g28330	LOC_Os04g45290	8.92235132

InteractorA	InteractorB	Z score
LOC_Os03g28330	LOC_Os04g38600	9.71072541
LOC_Os03g28330	LOC_Os04g56070	11.0419832
LOC_Os03g27280	LOC_Os11g32770	8.99069315
LOC_Os03g27310	LOC_Os06g04030	9.32450354
LOC_Os03g27260	LOC_Os08g10608	9.12144534
LOC_Os03g27310	LOC_Os08g44280	9.43284704
LOC_Os03g27310	LOC_Os05g01050	9.82043174
LOC_Os03g28400	LOC_Os11g04954	9.28882849
LOC_Os03g27280	LOC_Os05g04340	10.2507008
LOC_Os03g28400	LOC_Os04g59200	9.89713758
LOC_Os03g27310	LOC_Os09g15320	8.95632466
LOC_Os03g27310	LOC_Os03g60740	10.88884
LOC_Os03g27310	LOC_Os05g42150	8.80060242
LOC_Os03g28400	LOC_Os07g44740	9.15899565
LOC_Os03g28330	LOC_Os08g39860	9.16541661
LOC_Os03g28330	LOC_Os07g05940	10.1543438
LOC_Os03g27280	LOC_Os12g34450	9.6204691
LOC_Os03g28400	LOC_Os03g58530	9.54635236
LOC_Os03g26970	LOC_Os04g58880	9.97842573
LOC_Os03g26970	LOC_Os09g35800	10.6406601
LOC_Os03g27370	LOC_Os07g42600	9.60677485
LOC_Os03g27260	LOC_Os05g15520	11.027179
LOC_Os03g27370	LOC_Os06g42130	9.58874443
LOC_Os03g28400	LOC_Os04g57220	8.86540801
LOC_Os03g27310	LOC_Os05g43820	9.54964268
LOC_Os03g27310	LOC_Os07g44440	10.4293352
LOC_Os03g27370	LOC_Os09g24530	9.1918002
LOC_Os03g28400	LOC_Os04g56210	11.2397997
LOC_Os03g28400	LOC_Os05g01810	8.9625426
LOC_Os03g28400	LOC_Os07g38890	8.8920654
LOC_Os03g28400	LOC_Os12g22650	9.28703203
LOC_Os03g28400	LOC_Os10g32870	9.46386169
LOC_Os03g27260	LOC_Os05g33400	9.64812712
LOC_Os03g27370	LOC_Os04g39864	11.079181
LOC_Os03g27370	LOC_Os04g57410	9.7790013
LOC_Os03g27310	LOC_Os06g02380	9.69248748
LOC_Os03g27280	LOC_Os10g27050	9.16253302
LOC_Os03g28330	LOC_Os03g49350	8.93878992
LOC_Os03g27310	LOC_Os05g11550	8.92033555
LOC_Os03g27370	LOC_Os10g28120	8.97173324
LOC_Os03g26970	LOC_Os10g29620	8.86436783
LOC_Os03g27280	LOC_Os07g09890	8.99907793
LOC_Os03g30950	LOC_Os03g53790	9.82154686

InteractorA	InteractorB	Z score
LOC_Os03g31210	LOC_Os03g50490	8.87894952
LOC_Os03g31210	LOC_Os12g34062	9.43106763
LOC_Os03g31210	LOC_Os03g31750	8.8169095
LOC_Os03g30950	LOC_Os04g35570	8.85496203
LOC_Os03g30870	LOC_Os07g22600	9.93099243
LOC_Os03g31290	LOC_Os04g20070	9.29142852
LOC_Os03g30950	LOC_Os06g44620	10.4305056
LOC_Os03g31290	LOC_Os05g46290	9.52529633
LOC_Os03g29570	LOC_Os09g30412	9.6737586
LOC_Os03g29570	LOC_Os06g04000	9.13299868
LOC_Os03g31170	LOC_Os05g19150	9.8715987
LOC_Os03g30470	LOC_Os07g47490	8.80440237
LOC_Os03g29570	LOC_Os05g48510	11.261718
LOC_Os03g30950	LOC_Os12g34874	8.91099669
LOC_Os03g30870	LOC_Os08g09940	9.40228033
LOC_Os03g31290	LOC_Os08g43170	9.29128338
LOC_Os03g31210	LOC_Os04g31960	10.5175203
LOC_Os03g31210	LOC_Os04g15920	8.97145423
LOC_Os03g31210	LOC_Os11g47600	9.91641961
LOC_Os03g29570	LOC_Os04g57400	9.40938425
LOC_Os03g30470	LOC_Os10g27190	10.1730487
LOC_Os03g31300	LOC_Os03g45320	8.97163243
LOC_Os03g30470	LOC_Os03g57120	8.98026612
LOC_Os03g30950	LOC_Os09g28420	8.96719544
LOC_Os03g30470	LOC_Os05g40990	8.82564684
LOC_Os03g30950	LOC_Os07g07770	9.04854137
LOC_Os03g30950	LOC_Os06g44080	9.22223004
LOC_Os03g31210	LOC_Os10g37210	9.16251837
LOC_Os03g31170	LOC_Os03g55070	9.92873851
LOC_Os03g30870	LOC_Os04g10400	8.91507008
LOC_Os03g30470	LOC_Os06g23780	9.36333295
LOC_Os03g31170	LOC_Os04g01674	9.52133824
LOC_Os03g31210	LOC_Os04g43800	9.41933385
LOC_Os03g30470	LOC_Os04g58110	9.43502588
LOC_Os03g30470	LOC_Os07g09890	9.55344043
LOC_Os03g29570	LOC_Os04g58110	12.6078124
LOC_Os03g30470	LOC_Os04g59150	9.86117729
LOC_Os03g30870	LOC_Os09g10260	8.8838021
LOC_Os03g30870	LOC_Os04g33740	9.52590756
LOC_Os03g31170	LOC_Os07g22930	10.2714772
LOC_Os03g30470	LOC_Os05g24580	9.42721351
LOC_Os03g31170	LOC_Os07g34520	9.00120711
LOC_Os03g31290	LOC_Os05g39690	8.82584279

InteractorA	InteractorB	Z score
LOC_Os03g30470	LOC_Os09g24990	9.05210409
LOC_Os03g30470	LOC_Os06g10930	9.02121628
LOC_Os03g29570	LOC_Os06g41810	9.32813917
LOC_Os03g31210	LOC_Os04g52130	9.26586433
LOC_Os03g30870	LOC_Os05g36010	9.06419673
LOC_Os03g30870	LOC_Os12g43370	9.52338768
LOC_Os03g31290	LOC_Os05g45420	10.1351786
LOC_Os03g30870	LOC_Os08g15030	9.04451467
LOC_Os03g29570	LOC_Os08g06060	10.0071414
LOC_Os03g31170	LOC_Os07g48040	9.04326842
LOC_Os03g31170	LOC_Os04g55040	8.91406885
LOC_Os03g30470	LOC_Os04g30800	9.5885199
LOC_Os03g30950	LOC_Os10g03540	10.4908901
LOC_Os03g30950	LOC_Os08g37800	9.38676714
LOC_Os03g30870	LOC_Os05g49880	8.84474665
LOC_Os03g29570	LOC_Os04g48850	10.0197209
LOC_Os03g30950	LOC_Os05g47545	9.13235937
LOC_Os03g30950	LOC_Os06g11290	9.03326018
LOC_Os03g31210	LOC_Os08g44210	9.51217757
LOC_Os03g29570	LOC_Os11g25260	8.98930935
LOC_Os03g30950	LOC_Os10g08670	9.38248201
LOC_Os03g30870	LOC_Os08g35440	9.98214401
LOC_Os03g31290	LOC_Os04g56400	8.86228828
LOC_Os03g31210	LOC_Os04g32710	8.85046113
LOC_Os03g30870	LOC_Os07g05160	8.80807125
LOC_Os03g30470	LOC_Os08g34170	8.87146049
LOC_Os03g30870	LOC_Os06g10970	9.79248309
LOC_Os03g31170	LOC_Os05g08100	10.4190073
LOC_Os03g29570	LOC_Os04g30800	9.32399246
LOC_Os03g31210	LOC_Os06g29220	9.01204607
LOC_Os03g31290	LOC_Os12g12590	10.0801802
LOC_Os03g30950	LOC_Os07g05180	9.34222752
LOC_Os03g30470	LOC_Os05g47640	9.16528986
LOC_Os03g30470	LOC_Os06g46372	10.2425852
LOC_Os03g30870	LOC_Os03g58430	9.53928842
LOC_Os03g30870	LOC_Os08g44520	8.84205171
LOC_Os03g30950	LOC_Os06g36700	9.11067245
LOC_Os03g31170	LOC_Os10g08670	12.4405201
LOC_Os03g29570	LOC_Os05g07690	9.16484437
LOC_Os03g30470	LOC_Os04g16740	9.41958849
LOC_Os03g30950	LOC_Os08g40930	8.90134483
LOC_Os03g29570	LOC_Os04g48060	8.90373934
LOC_Os03g31290	LOC_Os09g28770	8.97552124

InteractorA	InteractorB	Z score
LOC_Os03g30870	LOC_Os09g36830	9.93261325
LOC_Os03g31170	LOC_Os05g05830	8.90086802
LOC_Os03g31170	LOC_Os09g10260	9.54058509
LOC_Os03g31210	LOC_Os04g52100	10.7652882
LOC_Os03g31210	LOC_Os07g20544	12.0874544
LOC_Os03g30950	LOC_Os10g22310	9.54349452
LOC_Os03g31170	LOC_Os04g38870	8.98242757
LOC_Os03g30870	LOC_Os07g27790	8.86247428
LOC_Os03g31290	LOC_Os04g41960	10.9619313
LOC_Os03g31300	LOC_Os03g59660	9.21895822
LOC_Os03g31170	LOC_Os12g22650	9.31651734
LOC_Os03g29570	LOC_Os04g57950	10.0084612
LOC_Os03g31170	LOC_Os04g48700	8.81910905
LOC_Os03g29570	LOC_Os10g09860	10.0740333
LOC_Os03g30870	LOC_Os10g23900	10.2501057
LOC_Os03g31290	LOC_Os10g38580	9.37105992
LOC_Os03g30950	LOC_Os10g02480	9.18756239
LOC_Os03g31210	LOC_Os08g28820	10.356959
LOC_Os03g30950	LOC_Os09g25320	9.22225013
LOC_Os03g30470	LOC_Os08g32850	9.05081292
LOC_Os03g31210	LOC_Os11g33240	8.80415029
LOC_Os03g30870	LOC_Os04g53810	9.20130297
LOC_Os03g30950	LOC_Os10g38140	9.3217975
LOC_Os03g31300	LOC_Os03g57200	9.16270526
LOC_Os03g29570	LOC_Os04g58580	10.0500842
LOC_Os03g30870	LOC_Os11g32260	9.55432691
LOC_Os03g30950	LOC_Os03g61600	9.87532781
LOC_Os03g30950	LOC_Os08g36910	9.23179599
LOC_Os03g31290	LOC_Os04g26910	11.2054133
LOC_Os03g31210	LOC_Os08g06610	9.80197402
LOC_Os03g31210	LOC_Os08g10608	9.82617053
LOC_Os03g29570	LOC_Os06g01850	9.17257235
LOC_Os03g30470	LOC_Os04g09670	8.86372669
LOC_Os03g37950	LOC_Os08g28800	8.87352318
LOC_Os03g31300	LOC_Os09g38020	10.4718022
LOC_Os03g32170	LOC_Os05g28940	9.25579469
LOC_Os03g32170	LOC_Os04g16846	9.05250982
LOC_Os03g38000	LOC_Os03g48390	9.54381311
LOC_Os03g32050	LOC_Os04g46620	9.54317244
LOC_Os03g31300	LOC_Os05g32140	10.7646737
LOC_Os03g32270	LOC_Os08g28190	9.50776108
LOC_Os03g32270	LOC_Os10g38360	9.0967102
LOC_Os03g32270	LOC_Os12g01922	9.43606336

InteractorA	InteractorB	Z score
LOC_Os03g31300	LOC_Os05g45810	8.95724593
LOC_Os03g38000	LOC_Os03g48471	10.5741987
LOC_Os03g37950	LOC_Os12g12514	10.3704757
LOC_Os03g32050	LOC_Os08g40140	9.8074117
LOC_Os03g37950	LOC_Os04g58570	10.6850425
LOC_Os03g31300	LOC_Os07g31830	9.15406031
LOC_Os03g31300	LOC_Os04g49420	10.4718022
LOC_Os03g32270	LOC_Os08g14770	9.06996113
LOC_Os03g31300	LOC_Os05g45420	9.10520051
LOC_Os03g32270	LOC_Os04g38940	8.93063082
LOC_Os03g32270	LOC_Os10g01080	8.99102097
LOC_Os03g32580	LOC_Os09g15320	9.30806059
LOC_Os03g31750	LOC_Os06g36880	9.21717515
LOC_Os03g31750	LOC_Os05g08100	8.91087096
LOC_Os03g32050	LOC_Os05g50890	9.2858802
LOC_Os03g31750	LOC_Os08g41340	9.0636497
LOC_Os03g32050	LOC_Os06g10970	9.11988578
LOC_Os03g32270	LOC_Os07g42924	10.2515925
LOC_Os03g32170	LOC_Os07g38910	8.85145967
LOC_Os03g32270	LOC_Os03g51550	9.13969998
LOC_Os03g32170	LOC_Os04g58710	11.7964596
LOC_Os03g31300	LOC_Os10g38690	9.27489586
LOC_Os03g32170	LOC_Os12g12590	9.41943739
LOC_Os03g32050	LOC_Os06g06460	8.97592296
LOC_Os03g32170	LOC_Os04g45470	9.41917637
LOC_Os03g32270	LOC_Os08g09200	8.80529597
LOC_Os03g32170	LOC_Os12g44020	10.088894
LOC_Os03g32050	LOC_Os04g45290	10.8285974
LOC_Os03g31750	LOC_Os10g07229	8.9753781
LOC_Os03g37950	LOC_Os03g64210	10.053827
LOC_Os03g31750	LOC_Os04g58680	9.02417761
LOC_Os03g32270	LOC_Os07g22600	9.66651138
LOC_Os03g32270	LOC_Os06g08770	10.1040611
LOC_Os03g31300	LOC_Os04g40290	9.12567598
LOC_Os03g31750	LOC_Os07g36190	9.1850167
LOC_Os03g37950	LOC_Os06g04280	9.59974409
LOC_Os03g32170	LOC_Os09g39570	9.44487849
LOC_Os03g31750	LOC_Os04g58200	10.086396
LOC_Os03g31750	LOC_Os06g35730	9.36743382
LOC_Os03g31300	LOC_Os07g48880	9.03825545
LOC_Os03g32050	LOC_Os03g55620	8.86645615
LOC_Os03g31750	LOC_Os07g34580	9.62807321
LOC_Os03g32270	LOC_Os12g02370	8.86205694

InteractorA	InteractorB	Z score
LOC_Os03g32270	LOC_Os12g16250	8.80022342
LOC_Os03g32170	LOC_Os10g21324	9.05253456
LOC_Os03g32270	LOC_Os03g62070	8.95207635
LOC_Os03g32270	LOC_Os08g09770	8.80600336
LOC_Os03g31300	LOC_Os05g39050	10.4718117
LOC_Os03g31300	LOC_Os10g39410	10.4718022
LOC_Os03g31750	LOC_Os09g25370	9.62976355
LOC_Os03g31300	LOC_Os05g37690	8.97910602
LOC_Os03g32580	LOC_Os06g34690	9.15302378
LOC_Os03g31300	LOC_Os04g30800	11.035415
LOC_Os03g31750	LOC_Os03g61920	9.84224445
LOC_Os03g31750	LOC_Os05g41080	9.00253501
LOC_Os03g32580	LOC_Os09g31120	9.80183252
LOC_Os03g32050	LOC_Os11g08470	10.579972
LOC_Os03g32580	LOC_Os06g43640	8.81841044
LOC_Os03g31300	LOC_Os03g61600	10.113926
LOC_Os03g31750	LOC_Os09g20260	9.79095606
LOC_Os03g32170	LOC_Os11g47570	8.82397606
LOC_Os03g32050	LOC_Os12g38760	8.83821896
LOC_Os03g31300	LOC_Os10g38189	9.87931683
LOC_Os03g32270	LOC_Os03g42110	9.52791874
LOC_Os03g32270	LOC_Os08g28820	8.85180296
LOC_Os03g31300	LOC_Os07g36500	10.4717978
LOC_Os03g31750	LOC_Os12g25710	9.89751388
LOC_Os03g32270	LOC_Os05g09500	9.10247451
LOC_Os03g32170	LOC_Os04g59150	9.29301295
LOC_Os03g32050	LOC_Os07g42950	9.40388728
LOC_Os03g31300	LOC_Os05g38740	10.4717978
LOC_Os03g31750	LOC_Os09g08910	9.13504834
LOC_Os03g32270	LOC_Os07g10720	9.56148972
LOC_Os03g32050	LOC_Os05g36280	8.97592296
LOC_Os03g31750	LOC_Os06g35540	9.29449404
LOC_Os03g37950	LOC_Os06g06100	10.0698367
LOC_Os03g32170	LOC_Os05g37390	9.1892669
LOC_Os03g31750	LOC_Os06g21980	9.35379136
LOC_Os03g32170	LOC_Os05g43510	8.81897733
LOC_Os03g32170	LOC_Os12g08270	8.8565603
LOC_Os03g37950	LOC_Os07g05180	8.95849454
LOC_Os03g32270	LOC_Os09g31506	9.54838773
LOC_Os03g31300	LOC_Os12g42876	9.04298173
LOC_Os03g37950	LOC_Os07g01760	9.73625471
LOC_Os03g31750	LOC_Os05g11710	9.36744347
LOC_Os03g31750	LOC_Os05g47540	9.85430693

InteractorA	InteractorB	Z score
LOC_Os03g32050	LOC_Os06g06510	8.97592296
LOC_Os03g32050	LOC_Os03g32270	8.95710453
LOC_Os03g31750	LOC_Os11g01360	8.91453454
LOC_Os03g37950	LOC_Os07g34580	9.25009871
LOC_Os03g32170	LOC_Os05g49770	9.06488089
LOC_Os03g32050	LOC_Os03g62500	9.29759274
LOC_Os03g32170	LOC_Os06g35530	10.242607
LOC_Os03g32580	LOC_Os08g28800	11.0621568
LOC_Os03g32050	LOC_Os11g05730	8.97592296
LOC_Os03g32050	LOC_Os05g34540	11.5255396
LOC_Os03g31750	LOC_Os05g33570	12.6658151
LOC_Os03g31300	LOC_Os04g43400	9.19513932
LOC_Os03g31300	LOC_Os04g53810	9.50487774
LOC_Os03g32170	LOC_Os04g52280	8.81672725
LOC_Os03g31300	LOC_Os12g22030	8.84761642
LOC_Os03g32050	LOC_Os04g28180	8.97565005
LOC_Os03g31750	LOC_Os06g50300	9.39775473
LOC_Os03g31750	LOC_Os12g41110	10.8760171
LOC_Os03g31300	LOC_Os09g26340	10.4718022
LOC_Os03g32270	LOC_Os04g12690	8.93211551
LOC_Os03g38000	LOC_Os07g47990	9.15301605
LOC_Os03g39710	LOC_Os06g36820	9.46155841
LOC_Os03g38980	LOC_Os12g24650	8.96535049
LOC_Os03g38980	LOC_Os04g33470	8.89454943
LOC_Os03g38980	LOC_Os10g30200	9.16663237
LOC_Os03g38020	LOC_Os04g36700	10.8647881
LOC_Os03g38000	LOC_Os03g60620	9.11680168
LOC_Os03g40330	LOC_Os03g62070	8.93497855
LOC_Os03g39610	LOC_Os06g35730	11.0746836
LOC_Os03g38980	LOC_Os05g11730	8.92803954
LOC_Os03g40330	LOC_Os10g38140	8.83977156
LOC_Os03g39710	LOC_Os07g30200	9.25471587
LOC_Os03g40330	LOC_Os04g53290	9.72167712
LOC_Os03g39710	LOC_Os09g19560	9.00758592
LOC_Os03g38980	LOC_Os04g19740	10.4573708
LOC_Os03g40330	LOC_Os10g08022	9.6544682
LOC_Os03g40720	LOC_Os05g11550	10.2529864
LOC_Os03g38000	LOC_Os04g40950	9.14836895
LOC_Os03g38000	LOC_Os05g41900	9.54138531
LOC_Os03g39610	LOC_Os05g11710	11.0746764
LOC_Os03g38980	LOC_Os06g07878	9.10898176
LOC_Os03g38000	LOC_Os05g48510	9.24554472
LOC_Os03g38000	LOC_Os10g26110	9.83756136

InteractorA	InteractorB	Z score
LOC_Os03g39610	LOC_Os05g35320	8.91574581
LOC_Os03g40720	LOC_Os05g38560	9.01113806
LOC_Os03g40330	LOC_Os09g09270	10.0273549
LOC_Os03g38980	LOC_Os03g51600	9.44234212
LOC_Os03g38980	LOC_Os07g31750	9.13770283
LOC_Os03g38980	LOC_Os06g37560	8.84685294
LOC_Os03g38980	LOC_Os07g06970	9.69894893
LOC_Os03g38000	LOC_Os12g02980	9.07017663
LOC_Os03g40330	LOC_Os03g62700	13.227809
LOC_Os03g40720	LOC_Os04g12710	9.931822
LOC_Os03g40330	LOC_Os04g16740	9.04322411
LOC_Os03g38980	LOC_Os04g52450	9.24251124
LOC_Os03g38980	LOC_Os10g41510	9.4125692
LOC_Os03g40720	LOC_Os07g40290	10.2106837
LOC_Os03g39610	LOC_Os10g38780	8.84319049
LOC_Os03g38980	LOC_Os06g12790	9.1637821
LOC_Os03g38000	LOC_Os07g30640	10.3243139
LOC_Os03g39610	LOC_Os08g02700	9.23945553
LOC_Os03g38020	LOC_Os04g56230	9.57199835
LOC_Os03g38000	LOC_Os04g44920	9.01858925
LOC_Os03g38000	LOC_Os04g42920	9.28244046
LOC_Os03g40330	LOC_Os03g63410	9.30996791
LOC_Os03g40330	LOC_Os05g51670	9.91459375
LOC_Os03g38000	LOC_Os05g32140	9.19759383
LOC_Os03g38980	LOC_Os03g41438	9.50735079
LOC_Os03g38980	LOC_Os10g07229	8.87721301
LOC_Os03g38000	LOC_Os08g38900	9.22463226
LOC_Os03g38000	LOC_Os08g43170	8.99256967
LOC_Os03g39710	LOC_Os03g52460	9.49943524
LOC_Os03g38980	LOC_Os04g33720	9.61191374
LOC_Os03g38980	LOC_Os08g10608	9.16096652
LOC_Os03g38980	LOC_Os04g52130	9.0964276
LOC_Os03g39610	LOC_Os05g27940	9.03284089
LOC_Os03g39610	LOC_Os11g25260	9.09165787
LOC_Os03g38000	LOC_Os07g02350	9.89443044
LOC_Os03g38980	LOC_Os03g56410	8.88143593
LOC_Os03g38000	LOC_Os12g43630	8.98989096
LOC_Os03g38980	LOC_Os04g52340	8.95459572
LOC_Os03g38020	LOC_Os07g02340	8.84897578
LOC_Os03g40720	LOC_Os03g55070	11.3027979
LOC_Os03g40720	LOC_Os04g35200	9.35965797
LOC_Os03g40720	LOC_Os12g12514	10.2191228
LOC_Os03g38000	LOC_Os06g47600	9.01428896

InteractorA	InteractorB	Z score
LOC_Os03g40720	LOC_Os06g01590	8.85312866
LOC_Os03g40720	LOC_Os05g31020	9.06189626
LOC_Os03g38980	LOC_Os04g33240	10.6699167
LOC_Os03g38020	LOC_Os07g49400	10.1543196
LOC_Os03g40720	LOC_Os12g17540	9.86105274
LOC_Os03g38000	LOC_Os09g26880	9.11165563
LOC_Os03g38980	LOC_Os03g39610	8.93780024
LOC_Os03g40720	LOC_Os05g43820	9.54643977
LOC_Os03g40720	LOC_Os06g12090	9.32485662
LOC_Os03g38980	LOC_Os06g07978	9.10898176
LOC_Os03g38980	LOC_Os09g17740	9.0028563
LOC_Os03g38000	LOC_Os03g61330	10.7398778
LOC_Os03g38980	LOC_Os09g11230	8.95484114
LOC_Os03g40330	LOC_Os08g33710	9.12616064
LOC_Os03g38000	LOC_Os12g10600	9.14272252
LOC_Os03g38980	LOC_Os05g33380	8.9536306
LOC_Os03g40330	LOC_Os09g04050	9.12956844
LOC_Os03g38000	LOC_Os04g16846	9.92427819
LOC_Os03g39610	LOC_Os04g37460	9.14021964
LOC_Os03g38980	LOC_Os05g04340	9.4581639
LOC_Os03g38000	LOC_Os04g42930	9.10448565
LOC_Os03g39710	LOC_Os06g11210	8.86517111
LOC_Os03g38020	LOC_Os08g02400	8.92676184
LOC_Os03g40720	LOC_Os07g08170	9.58455874
LOC_Os03g38980	LOC_Os07g42960	10.2103879
LOC_Os03g38020	LOC_Os12g02370	8.8930083
LOC_Os03g38980	LOC_Os04g27060	8.96805598
LOC_Os03g38020	LOC_Os08g44370	8.99444456
LOC_Os03g38980	LOC_Os07g37790	8.95459572
LOC_Os03g38000	LOC_Os09g23530	9.00808548
LOC_Os03g40330	LOC_Os03g63720	10.306513
LOC_Os03g38000	LOC_Os12g21798	10.0345905
LOC_Os03g38980	LOC_Os04g53810	10.7704894
LOC_Os03g38980	LOC_Os05g44340	9.84506771
LOC_Os03g40720	LOC_Os07g48040	9.53410649
LOC_Os03g38000	LOC_Os10g21324	9.92427819
LOC_Os03g38000	LOC_Os06g06560	9.0018701
LOC_Os03g40330	LOC_Os04g55740	9.36419882
LOC_Os03g40330	LOC_Os07g26640	9.4693893
LOC_Os03g38980	LOC_Os03g59700	9.00454563
LOC_Os03g40720	LOC_Os06g06320	8.88754968
LOC_Os03g41438	LOC_Os03g41460	9.3380096
LOC_Os03g38980	LOC_Os07g49220	9.05067627

InteractorA	InteractorB	Z score
LOC_Os03g40330	LOC_Os10g38470	9.27293129
LOC_Os03g40720	LOC_Os04g52450	8.83523065
LOC_Os03g40330	LOC_Os05g05680	9.26320376
LOC_Os03g38980	LOC_Os07g25590	9.38287301
LOC_Os03g40330	LOC_Os09g31502	8.92993508
LOC_Os03g38020	LOC_Os12g04924	8.98922069
LOC_Os03g40330	LOC_Os04g44870	8.86410235
LOC_Os03g40330	LOC_Os09g20284	9.306228
LOC_Os03g40720	LOC_Os08g15040	9.72549479
LOC_Os03g38020	LOC_Os09g08720	9.6545776
LOC_Os03g38000	LOC_Os06g04270	9.24167816
LOC_Os03g38000	LOC_Os10g26390	8.9117184
LOC_Os03g40330	LOC_Os05g10780	9.35901572
LOC_Os03g39610	LOC_Os08g06100	10.3957766
LOC_Os03g38020	LOC_Os05g06750	8.80048502
LOC_Os03g38980	LOC_Os12g44020	9.45428307
LOC_Os03g38020	LOC_Os08g44960	8.82514106
LOC_Os03g39610	LOC_Os05g45810	9.02487284
LOC_Os03g38980	LOC_Os04g36800	13.7122527
LOC_Os03g40720	LOC_Os06g12180	8.82358002
LOC_Os03g38980	LOC_Os04g42380	8.99190559
LOC_Os03g39610	LOC_Os11g26910	9.3895829
LOC_Os03g44150	LOC_Os04g32710	8.95771734
LOC_Os03g41438	LOC_Os04g26920	9.50017502
LOC_Os03g41460	LOC_Os08g40740	9.68977293
LOC_Os03g41438	LOC_Os08g28800	9.4922462
LOC_Os03g44150	LOC_Os07g26640	9.59502593
LOC_Os03g42220	LOC_Os03g50290	8.87649997
LOC_Os03g41460	LOC_Os06g45120	9.09247639
LOC_Os03g42110	LOC_Os08g10608	9.26917641
LOC_Os03g42220	LOC_Os07g38540	8.8773439
LOC_Os03g43890	LOC_Os04g02050	9.0690455
LOC_Os03g41460	LOC_Os05g46270	9.58305988
LOC_Os03g44150	LOC_Os06g43640	8.84181256
LOC_Os03g44150	LOC_Os06g06050	9.46002243
LOC_Os03g41460	LOC_Os12g07720	10.3861932
LOC_Os03g44150	LOC_Os10g39120	9.73844525
LOC_Os03g42110	LOC_Os07g39290	8.80758039
LOC_Os03g41438	LOC_Os04g36700	11.3274324
LOC_Os03g41460	LOC_Os03g48390	8.87443584
LOC_Os03g43890	LOC_Os10g41480	9.01541468
LOC_Os03g44150	LOC_Os07g44590	9.40017876
LOC_Os03g41438	LOC_Os06g12180	9.1779074

InteractorA	InteractorB	Z score
LOC_Os03g42110	LOC_Os11g47600	9.43728398
LOC_Os03g43890	LOC_Os11g37550	8.98580138
LOC_Os03g43890	LOC_Os04g55290	10.3820615
LOC_Os03g42840	LOC_Os05g26890	9.04064936
LOC_Os03g41438	LOC_Os11g43360	9.78107075
LOC_Os03g41460	LOC_Os06g07878	9.06387943
LOC_Os03g41460	LOC_Os04g31960	8.96556768
LOC_Os03g42840	LOC_Os04g33470	9.2589567
LOC_Os03g41460	LOC_Os10g21310	9.18035116
LOC_Os03g41460	LOC_Os10g26130	9.06298225
LOC_Os03g43890	LOC_Os03g57040	9.60098514
LOC_Os03g41460	LOC_Os06g06300	9.408767
LOC_Os03g42110	LOC_Os07g44740	9.70477363
LOC_Os03g41460	LOC_Os10g08670	9.99564316
LOC_Os03g42840	LOC_Os06g05880	9.53492526
LOC_Os03g44150	LOC_Os08g34790	9.09963772
LOC_Os03g44150	LOC_Os10g38730	8.82503051
LOC_Os03g44150	LOC_Os06g27770	8.95706432
LOC_Os03g41438	LOC_Os05g06450	10.0792402
LOC_Os03g42840	LOC_Os11g47570	9.01081298
LOC_Os03g44150	LOC_Os07g02340	10.4291992
LOC_Os03g42840	LOC_Os07g43510	10.3367557
LOC_Os03g43890	LOC_Os11g10520	8.8964972
LOC_Os03g42840	LOC_Os04g55740	11.0894074
LOC_Os03g44150	LOC_Os03g46070	8.87749115
LOC_Os03g44150	LOC_Os07g08880	10.5588117
LOC_Os03g44150	LOC_Os04g14680	8.83747701
LOC_Os03g43890	LOC_Os09g07460	8.8360304
LOC_Os03g44150	LOC_Os07g08500	9.07105832
LOC_Os03g42220	LOC_Os06g50300	9.89741115
LOC_Os03g44150	LOC_Os10g25140	9.20968903
LOC_Os03g44150	LOC_Os06g07140	8.89896947
LOC_Os03g42220	LOC_Os04g46930	8.94528644
LOC_Os03g43890	LOC_Os07g05180	9.19417167
LOC_Os03g42840	LOC_Os11g02130	9.3706161
LOC_Os03g41460	LOC_Os04g57400	9.22462434
LOC_Os03g41438	LOC_Os06g04510	9.56999075
LOC_Os03g43890	LOC_Os08g25570	10.3255144
LOC_Os03g42840	LOC_Os12g13380	8.86919782
LOC_Os03g42840	LOC_Os07g35880	9.29203083
LOC_Os03g41460	LOC_Os04g02820	9.02520602
LOC_Os03g42840	LOC_Os09g24990	9.30723989
LOC_Os03g41460	LOC_Os10g27050	9.44585581

InteractorA	InteractorB	Z score
LOC_Os03g41438	LOC_Os08g37790	8.97796344
LOC_Os03g42220	LOC_Os04g33470	10.5107908
LOC_Os03g41438	LOC_Os07g28480	9.10189071
LOC_Os03g42840	LOC_Os06g35700	8.80833671
LOC_Os03g42840	LOC_Os04g39840	9.03301336
LOC_Os03g42840	LOC_Os11g01872	10.054863
LOC_Os03g44150	LOC_Os07g08660	9.17005881
LOC_Os03g41460	LOC_Os03g64030	10.1012819
LOC_Os03g42840	LOC_Os06g10910	9.46613224
LOC_Os03g42840	LOC_Os08g44810	8.87820309
LOC_Os03g41438	LOC_Os10g38150	9.19923096
LOC_Os03g44150	LOC_Os10g35110	11.6458876
LOC_Os03g42110	LOC_Os07g44460	9.11266574
LOC_Os03g42110	LOC_Os06g36160	8.99523775
LOC_Os03g43890	LOC_Os06g12790	9.88122623
LOC_Os03g44150	LOC_Os03g47610	9.50473083
LOC_Os03g42110	LOC_Os07g14590	9.36511097
LOC_Os03g41460	LOC_Os11g31530	10.122127
LOC_Os03g42840	LOC_Os04g26920	10.2586388
LOC_Os03g42220	LOC_Os11g08340	9.52796784
LOC_Os03g43890	LOC_Os06g06730	8.85710661
LOC_Os03g41460	LOC_Os11g32620	9.27138957
LOC_Os03g42110	LOC_Os08g33100	9.50158319
LOC_Os03g44150	LOC_Os10g38670	8.83747542
LOC_Os03g44150	LOC_Os08g44340	9.01598817
LOC_Os03g41438	LOC_Os08g44340	10.5226106
LOC_Os03g42220	LOC_Os11g10480	9.43831814
LOC_Os03g43890	LOC_Os08g44960	8.81951043
LOC_Os03g42220	LOC_Os08g09950	9.12020993
LOC_Os03g42840	LOC_Os07g03690	8.90422662
LOC_Os03g41438	LOC_Os07g36140	9.63371112
LOC_Os03g41438	LOC_Os08g05910	10.1510178
LOC_Os03g41438	LOC_Os06g07878	10.0511657
LOC_Os03g44150	LOC_Os03g49600	9.37907969
LOC_Os03g42840	LOC_Os06g08600	10.1580699
LOC_Os03g41438	LOC_Os06g01360	9.27537757
LOC_Os03g44150	LOC_Os05g35400	9.00472627
LOC_Os03g41438	LOC_Os11g32580	9.04518073
LOC_Os03g44150	LOC_Os05g50710	8.91128437
LOC_Os03g44150	LOC_Os12g08280	9.67931714
LOC_Os03g42110	LOC_Os08g29370	9.12494267
LOC_Os03g41438	LOC_Os05g05830	9.27676438
LOC_Os03g42220	LOC_Os10g37060	10.1803591

InteractorA	InteractorB	Z score
LOC_Os03g42220	LOC_Os03g63410	9.29081241
LOC_Os03g44170	LOC_Os03g49260	10.1533816
LOC_Os03g44150	LOC_Os11g34450	9.36378321
LOC_Os03g42110	LOC_Os08g42000	9.57345007
LOC_Os03g42220	LOC_Os08g40140	9.13868206
LOC_Os03g44150	LOC_Os06g46340	11.7081177
LOC_Os03g43890	LOC_Os10g08580	9.24632515
LOC_Os03g41438	LOC_Os09g33500	8.81311362
LOC_Os03g42840	LOC_Os03g62700	10.8356596
LOC_Os03g41460	LOC_Os04g39900	8.92792832
LOC_Os03g43890	LOC_Os03g62700	8.87799135
LOC_Os03g44150	LOC_Os05g47640	10.0771684
LOC_Os03g42220	LOC_Os07g35940	9.37991327
LOC_Os03g44150	LOC_Os12g03090	8.8589007
LOC_Os03g44150	LOC_Os04g40130	9.11567113
LOC_Os03g41460	LOC_Os04g26920	9.12937926
LOC_Os03g42220	LOC_Os06g42560	9.19648161
LOC_Os03g44150	LOC_Os07g19040	8.93722696
LOC_Os03g42840	LOC_Os05g12190	8.96319151
LOC_Os03g42110	LOC_Os04g16826	8.8208992
LOC_Os03g42220	LOC_Os11g34450	9.69069388
LOC_Os03g42110	LOC_Os08g38900	9.30061623
LOC_Os03g41438	LOC_Os12g42876	9.07097488
LOC_Os03g42110	LOC_Os07g30990	9.13245776
LOC_Os03g41460	LOC_Os07g43820	9.43789798
LOC_Os03g44150	LOC_Os10g34760	8.96234115
LOC_Os03g41460	LOC_Os04g18650	8.88296621
LOC_Os03g41438	LOC_Os07g46630	9.39054975
LOC_Os03g42220	LOC_Os03g58980	9.09618353
LOC_Os03g41438	LOC_Os06g07978	10.051197
LOC_Os03g42110	LOC_Os10g21344	8.82084294
LOC_Os03g42840	LOC_Os07g26640	8.84806632
LOC_Os03g41460	LOC_Os06g07978	9.06384539
LOC_Os03g44150	LOC_Os11g03400	8.8589007
LOC_Os03g44150	LOC_Os12g37960	9.14265608
LOC_Os03g42840	LOC_Os12g32240	9.4529075
LOC_Os03g44150	LOC_Os04g30800	9.27041276
LOC_Os03g42840	LOC_Os04g17650	9.07405602
LOC_Os03g42840	LOC_Os10g08550	9.44093736
LOC_Os03g42220	LOC_Os09g15790	9.27115383
LOC_Os03g42840	LOC_Os06g23870	9.55871814
LOC_Os03g42110	LOC_Os04g56320	9.16201293
LOC_Os03g44150	LOC_Os11g37950	9.69068201

InteractorA	InteractorB	Z score
LOC_Os03g42220	LOC_Os06g11240	9.25453583
LOC_Os03g42220	LOC_Os08g09770	9.19701247
LOC_Os03g41438	LOC_Os04g53230	9.57358287
LOC_Os03g45410	LOC_Os03g64210	9.12548995
LOC_Os03g45320	LOC_Os07g48050	9.79304161
LOC_Os03g44484	LOC_Os04g16872	9.6324603
LOC_Os03g45410	LOC_Os12g22650	9.26774888
LOC_Os03g45320	LOC_Os11g01872	10.1199805
LOC_Os03g44170	LOC_Os11g32520	11.3575399
LOC_Os03g44484	LOC_Os12g12580	9.85986974
LOC_Os03g44380	LOC_Os08g35740	10.180337
LOC_Os03g45410	LOC_Os03g47000	8.88461188
LOC_Os03g45270	LOC_Os06g24990	8.82934525
LOC_Os03g45320	LOC_Os11g26860	9.4601566
LOC_Os03g44380	LOC_Os06g36160	8.96179124
LOC_Os03g45320	LOC_Os09g08720	10.1620864
LOC_Os03g45320	LOC_Os04g56950	8.89174339
LOC_Os03g45920	LOC_Os12g43100	9.40299226
LOC_Os03g45410	LOC_Os07g07060	10.8266999
LOC_Os03g44170	LOC_Os06g36700	9.24242012
LOC_Os03g44484	LOC_Os06g13450	10.6366876
LOC_Os03g45320	LOC_Os05g09440	9.2749186
LOC_Os03g45920	LOC_Os10g07229	11.1639495
LOC_Os03g45410	LOC_Os10g34520	9.45719514
LOC_Os03g45920	LOC_Os04g30800	9.0138965
LOC_Os03g45270	LOC_Os06g35530	9.12215072
LOC_Os03g45320	LOC_Os05g47890	10.0185422
LOC_Os03g44484	LOC_Os04g33470	8.95554436
LOC_Os03g44380	LOC_Os05g05830	9.93544861
LOC_Os03g45410	LOC_Os06g51084	9.3637891
LOC_Os03g45920	LOC_Os04g58200	9.04528024
LOC_Os03g45920	LOC_Os04g57410	8.825263
LOC_Os03g44484	LOC_Os12g12514	10.1981606
LOC_Os03g45920	LOC_Os10g40730	9.09412972
LOC_Os03g44484	LOC_Os07g40290	9.00934724
LOC_Os03g45320	LOC_Os07g43170	9.49799627
LOC_Os03g45410	LOC_Os12g41110	9.11536313
LOC_Os03g45410	LOC_Os03g62060	9.98603138
LOC_Os03g45320	LOC_Os10g07040	9.00883268
LOC_Os03g44170	LOC_Os05g06750	9.23841505
LOC_Os03g44170	LOC_Os12g12514	9.10864258
LOC_Os03g44484	LOC_Os06g11240	10.3064756
LOC_Os03g45410	LOC_Os10g38600	12.4003008

InteractorA	InteractorB	Z score
LOC_Os03g45920	LOC_Os05g29880	9.58731015
LOC_Os03g45320	LOC_Os04g57410	8.80326575
LOC_Os03g45920	LOC_Os04g28180	10.040116
LOC_Os03g44380	LOC_Os05g40420	8.88730734
LOC_Os03g44170	LOC_Os05g23740	9.05193317
LOC_Os03g45320	LOC_Os10g21310	9.20056733
LOC_Os03g45920	LOC_Os09g31490	9.15745769
LOC_Os03g44380	LOC_Os07g05160	8.89929665
LOC_Os03g44484	LOC_Os10g38740	9.68090053
LOC_Os03g45270	LOC_Os06g06320	8.95173288
LOC_Os03g45410	LOC_Os09g32800	9.68747134
LOC_Os03g44380	LOC_Os08g20420	10.2542889
LOC_Os03g45270	LOC_Os10g40710	9.24730988
LOC_Os03g44484	LOC_Os04g39020	9.57630048
LOC_Os03g45410	LOC_Os03g60620	8.97337559
LOC_Os03g44170	LOC_Os10g38229	9.9315046
LOC_Os03g45320	LOC_Os04g52130	9.30973035
LOC_Os03g45320	LOC_Os07g30200	8.80756048
LOC_Os03g45960	LOC_Os03g64210	8.98051966
LOC_Os03g45270	LOC_Os07g05160	8.88771411
LOC_Os03g45320	LOC_Os09g32830	10.1473706
LOC_Os03g45920	LOC_Os07g06970	10.1671322
LOC_Os03g44170	LOC_Os08g14760	9.35071574
LOC_Os03g45920	LOC_Os07g47290	8.89778089
LOC_Os03g45320	LOC_Os05g49840	9.56173617
LOC_Os03g45270	LOC_Os04g58680	9.03872916
LOC_Os03g45270	LOC_Os04g54330	9.00395538
LOC_Os03g45270	LOC_Os05g51050	8.90370606
LOC_Os03g44170	LOC_Os07g05150	9.23876559
LOC_Os03g45320	LOC_Os04g45290	8.9501084
LOC_Os03g44484	LOC_Os11g37550	9.7526107
LOC_Os03g45270	LOC_Os12g22680	9.61171418
LOC_Os03g44484	LOC_Os10g37060	8.92854093
LOC_Os03g45960	LOC_Os03g52860	9.46757026
LOC_Os03g45320	LOC_Os05g08960	9.63363797
LOC_Os03g45270	LOC_Os04g48540	9.62025789
LOC_Os03g45320	LOC_Os08g34280	9.04242283
LOC_Os03g45920	LOC_Os07g41050	9.2729865
LOC_Os03g44484	LOC_Os06g12790	9.12429017
LOC_Os03g45320	LOC_Os07g48010	9.89636507
LOC_Os03g45320	LOC_Os08g14760	9.27609321
LOC_Os03g45270	LOC_Os09g12570	9.08084694
LOC_Os03g45320	LOC_Os07g44260	8.88144389

InteractorA	InteractorB	Z score
LOC_Os03g44170	LOC_Os06g10330	8.93362102
LOC_Os03g44170	LOC_Os05g06450	9.81053544
LOC_Os03g44380	LOC_Os11g10520	9.99557025
LOC_Os03g45270	LOC_Os05g11730	8.93929578
LOC_Os03g45320	LOC_Os12g06620	9.09369384
LOC_Os03g45270	LOC_Os03g59060	8.93502775
LOC_Os03g45920	LOC_Os05g23740	9.24955027
LOC_Os03g45320	LOC_Os09g19954	9.31942424
LOC_Os03g45320	LOC_Os07g12730	9.15183218
LOC_Os03g45920	LOC_Os07g08880	9.34457757
LOC_Os03g45410	LOC_Os10g08670	8.81580522
LOC_Os03g44484	LOC_Os05g37330	9.28320765
LOC_Os03g45410	LOC_Os08g41830	10.1903745
LOC_Os03g45920	LOC_Os05g05670	8.83981284
LOC_Os03g45320	LOC_Os04g56646	9.1468122
LOC_Os03g45960	LOC_Os04g02050	9.68107757
LOC_Os03g45270	LOC_Os10g40600	8.81097937
LOC_Os03g45920	LOC_Os06g01390	8.91535259
LOC_Os03g45320	LOC_Os10g41689	11.0302585
LOC_Os03g45920	LOC_Os08g33710	8.91172873
LOC_Os03g45320	LOC_Os10g28120	9.52243684
LOC_Os03g45270	LOC_Os05g03820	8.84799815
LOC_Os03g44170	LOC_Os11g47550	8.84740542
LOC_Os03g44484	LOC_Os11g01872	9.66117173
LOC_Os03g44484	LOC_Os12g38180	9.43864025
LOC_Os03g44170	LOC_Os04g54330	10.0749588
LOC_Os03g44170	LOC_Os07g06970	10.188059
LOC_Os03g45320	LOC_Os07g19040	10.0243865
LOC_Os03g45320	LOC_Os11g41130	9.04539103
LOC_Os03g45320	LOC_Os09g24990	9.22988229
LOC_Os03g44484	LOC_Os08g40930	9.3405332
LOC_Os03g44170	LOC_Os08g41990	8.83819
LOC_Os03g46070	LOC_Os04g55410	9.34304636
LOC_Os03g46070	LOC_Os10g39120	9.01476231
LOC_Os03g47000	LOC_Os03g53690	8.94648703
LOC_Os03g47770	LOC_Os12g12590	8.86788111
LOC_Os03g46070	LOC_Os04g57220	10.3341675
LOC_Os03g47610	LOC_Os05g12190	10.153854
LOC_Os03g46070	LOC_Os07g26630	9.2952676
LOC_Os03g47000	LOC_Os09g26880	9.14982862
LOC_Os03g47610	LOC_Os10g26390	9.09739553
LOC_Os03g46060	LOC_Os07g02340	9.28216515
LOC_Os03g47770	LOC_Os10g40720	9.68825938

InteractorA	InteractorB	Z score
LOC_Os03g47000	LOC_Os06g11290	9.72935539
LOC_Os03g47770	LOC_Os03g51550	9.11291359
LOC_Os03g48390	LOC_Os03g60740	9.29291902
LOC_Os03g48390	LOC_Os04g27980	9.17716029
LOC_Os03g47770	LOC_Os04g12720	9.8571501
LOC_Os03g47770	LOC_Os07g46830	11.0278831
LOC_Os03g47610	LOC_Os04g45490	11.3802926
LOC_Os03g48310	LOC_Os08g09200	10.5271806
LOC_Os03g45960	LOC_Os10g40720	9.40212482
LOC_Os03g46070	LOC_Os09g23530	9.07744375
LOC_Os03g47610	LOC_Os09g26880	8.90490678
LOC_Os03g45960	LOC_Os07g05150	8.88681811
LOC_Os03g46070	LOC_Os09g36830	9.22215521
LOC_Os03g47000	LOC_Os05g51480	10.189188
LOC_Os03g46070	LOC_Os05g46860	9.0000877
LOC_Os03g46070	LOC_Os12g12514	8.83529981
LOC_Os03g47000	LOC_Os07g42490	9.33999429
LOC_Os03g47000	LOC_Os09g25390	9.90107824
LOC_Os03g48310	LOC_Os06g12790	9.42763941
LOC_Os03g46070	LOC_Os04g54390	9.58624945
LOC_Os03g47610	LOC_Os09g34970	9.67504507
LOC_Os03g46070	LOC_Os08g33710	8.85072545
LOC_Os03g46070	LOC_Os08g28820	9.18355825
LOC_Os03g47770	LOC_Os05g38230	8.99978012
LOC_Os03g47610	LOC_Os04g15920	9.55724985
LOC_Os03g47770	LOC_Os03g55150	9.33016958
LOC_Os03g48310	LOC_Os07g05400	9.03527626
LOC_Os03g47000	LOC_Os04g57590	11.1213541
LOC_Os03g46070	LOC_Os05g07090	9.33338289
LOC_Os03g47770	LOC_Os09g19954	9.26200752
LOC_Os03g47610	LOC_Os03g55389	9.19116797
LOC_Os03g47770	LOC_Os08g04540	8.84172385
LOC_Os03g47000	LOC_Os09g31486	9.28348281
LOC_Os03g47000	LOC_Os08g36900	8.93988211
LOC_Os03g47770	LOC_Os10g01570	10.3948578
LOC_Os03g46060	LOC_Os05g19380	9.90381628
LOC_Os03g47000	LOC_Os10g38580	9.65500068
LOC_Os03g46060	LOC_Os12g01922	9.15917132
LOC_Os03g48390	LOC_Os04g58680	10.2107426
LOC_Os03g47000	LOC_Os09g39500	10.0752136
LOC_Os03g47610	LOC_Os07g35880	9.24284603
LOC_Os03g47000	LOC_Os05g49880	10.5647207
LOC_Os03g47770	LOC_Os10g07616	9.4435787

InteractorA	InteractorB	Z score
LOC_Os03g46070	LOC_Os03g57120	9.35252634
LOC_Os03g45960	LOC_Os07g03368	9.08053354
LOC_Os03g47610	LOC_Os05g04500	10.3188628
LOC_Os03g47770	LOC_Os05g51050	9.97458054
LOC_Os03g46070	LOC_Os09g32840	11.1807097
LOC_Os03g46060	LOC_Os07g23730	8.88792045
LOC_Os03g48310	LOC_Os12g44150	9.00521029
LOC_Os03g47610	LOC_Os04g32710	8.84297591
LOC_Os03g47610	LOC_Os07g31270	9.19457129
LOC_Os03g47000	LOC_Os09g31506	9.14873325
LOC_Os03g46070	LOC_Os10g41510	9.21503905
LOC_Os03g47770	LOC_Os05g31140	8.80653193
LOC_Os03g46060	LOC_Os06g04030	9.2525472
LOC_Os03g45960	LOC_Os09g38620	8.82153164
LOC_Os03g47000	LOC_Os09g20820	8.85881322
LOC_Os03g47000	LOC_Os07g31270	9.38032927
LOC_Os03g47610	LOC_Os03g48471	9.40132943
LOC_Os03g47000	LOC_Os09g36930	9.06731777
LOC_Os03g47770	LOC_Os12g25690	8.80001149
LOC_Os03g45960	LOC_Os11g47560	9.35378233
LOC_Os03g47000	LOC_Os04g54390	9.18992377
LOC_Os03g47770	LOC_Os12g26290	8.94692277
LOC_Os03g46060	LOC_Os10g05069	9.15417823
LOC_Os03g48310	LOC_Os08g40140	8.87140663
LOC_Os03g48310	LOC_Os06g48160	8.90491421
LOC_Os03g46070	LOC_Os11g19220	9.21718425
LOC_Os03g47770	LOC_Os07g23850	9.14754838
LOC_Os03g46060	LOC_Os05g49760	8.81114002
LOC_Os03g47770	LOC_Os10g10434	11.0842622
LOC_Os03g47770	LOC_Os12g44030	8.99471533
LOC_Os03g47770	LOC_Os07g07320	8.99416512
LOC_Os03g46070	LOC_Os07g09890	10.5099759
LOC_Os03g47000	LOC_Os07g16970	9.2782101
LOC_Os03g46070	LOC_Os09g08880	9.70810769
LOC_Os03g46070	LOC_Os04g38940	8.95049823
LOC_Os03g45960	LOC_Os10g41510	9.18337092
LOC_Os03g47000	LOC_Os04g08350	8.90304942
LOC_Os03g47610	LOC_Os07g48020	10.3096399
LOC_Os01g14860	LOC_Os04g17650	8.89329031
LOC_Os01g14950	LOC_Os01g55540	8.88951405
LOC_Os01g14580	LOC_Os03g58050	8.95234002
LOC_Os01g15010	LOC_Os10g41510	10.1136119
LOC_Os01g14670	LOC_Os09g10230	9.12473138

InteractorA	InteractorB	Z score
LOC_Os01g14580	LOC_Os04g33190	9.06951804
LOC_Os01g14580	LOC_Os10g42720	8.87591992
LOC_Os01g14580	LOC_Os09g25370	10.6287091
LOC_Os01g14860	LOC_Os02g06300	9.01821286
LOC_Os01g14670	LOC_Os12g18880	8.87459975
LOC_Os01g14580	LOC_Os05g49760	12.9013212
LOC_Os01g14860	LOC_Os06g43640	9.4421286
LOC_Os01g14580	LOC_Os09g08072	9.90923036
LOC_Os01g14950	LOC_Os01g46290	9.8811356
LOC_Os01g14950	LOC_Os10g39680	9.168632
LOC_Os01g14860	LOC_Os03g29570	8.95286819
LOC_Os01g15120	LOC_Os01g27230	9.25348544
LOC_Os01g14860	LOC_Os03g58400	9.3033622
LOC_Os01g14580	LOC_Os04g42920	14.7873616
LOC_Os01g14860	LOC_Os10g28080	9.41968075
LOC_Os01g14950	LOC_Os01g59490	9.09075263
LOC_Os01g14860	LOC_Os04g37460	8.8405882
LOC_Os01g15010	LOC_Os05g37390	9.79807153
LOC_Os01g14670	LOC_Os12g12590	9.02273295
LOC_Os01g14580	LOC_Os07g07709	8.95234002
LOC_Os01g14670	LOC_Os08g33820	9.10264244
LOC_Os01g14580	LOC_Os05g36270	8.84343369
LOC_Os01g14670	LOC_Os09g25370	9.68648531
LOC_Os01g14670	LOC_Os01g18170	10.4303082
LOC_Os01g14670	LOC_Os08g02700	8.96560022
LOC_Os01g15010	LOC_Os08g34790	9.70168992
LOC_Os01g14580	LOC_Os07g08030	9.75860517
LOC_Os01g15120	LOC_Os01g49690	9.81022853
LOC_Os01g15010	LOC_Os05g39690	8.82137388
LOC_Os01g14580	LOC_Os03g59020	8.98219411
LOC_Os01g14950	LOC_Os06g06050	10.0429243
LOC_Os01g14860	LOC_Os01g57964	9.27632399
LOC_Os01g14580	LOC_Os03g53650	9.10748763
LOC_Os01g14580	LOC_Os03g39710	9.04621043
LOC_Os01g15010	LOC_Os06g21570	9.55327955
LOC_Os01g14950	LOC_Os12g10720	9.64014926
LOC_Os01g14950	LOC_Os06g50300	9.1184832
LOC_Os01g14580	LOC_Os03g51550	9.49184435
LOC_Os01g15010	LOC_Os01g67160	9.19561083
LOC_Os01g14860	LOC_Os04g16760	9.27632399
LOC_Os01g14860	LOC_Os01g51170	9.72229265
LOC_Os01g14860	LOC_Os06g05090	8.8165841
LOC_Os01g14580	LOC_Os03g15360	10.6027742

InteractorA	InteractorB	Z score
LOC_Os01g15010	LOC_Os05g07720	8.9274429
LOC_Os01g14860	LOC_Os05g01675	9.27632399
LOC_Os01g14580	LOC_Os03g20700	9.48472264
LOC_Os01g15010	LOC_Os02g02210	9.18849732
LOC_Os01g15010	LOC_Os07g06970	8.99760067
LOC_Os01g14950	LOC_Os04g52440	9.24797946
LOC_Os01g14670	LOC_Os04g12690	9.37902806
LOC_Os01g15010	LOC_Os02g46970	9.37262484
LOC_Os01g14950	LOC_Os12g43100	9.57380146
LOC_Os01g14670	LOC_Os03g48750	8.98691728
LOC_Os01g14950	LOC_Os11g43960	9.36582116
LOC_Os01g14860	LOC_Os12g43100	9.82486908
LOC_Os01g14860	LOC_Os08g08070	8.91210289
LOC_Os01g14670	LOC_Os03g58980	10.0804598
LOC_Os01g14860	LOC_Os05g04450	8.86322369
LOC_Os01g14860	LOC_Os03g22010	9.42343946
LOC_Os01g15010	LOC_Os06g11200	10.8784703
LOC_Os01g14860	LOC_Os05g44340	9.44905828
LOC_Os01g14950	LOC_Os01g62230	10.0121335
LOC_Os01g14860	LOC_Os05g35400	11.3216024
LOC_Os01g14860	LOC_Os02g47590	8.85097543
LOC_Os01g14580	LOC_Os11g05570	8.84805148
LOC_Os01g14580	LOC_Os04g27860	8.91423258
LOC_Os01g15010	LOC_Os09g23530	9.28633963
LOC_Os01g14860	LOC_Os09g29200	8.85470333
LOC_Os01g14580	LOC_Os07g41050	8.92856795
LOC_Os03g48780	LOC_Os06g28550	9.03517142
LOC_Os03g48760	LOC_Os06g29220	9.25998211
LOC_Os03g48780	LOC_Os05g19670	10.8715346
LOC_Os03g48760	LOC_Os07g37790	8.83708737
LOC_Os03g48770	LOC_Os03g53860	9.75069247
LOC_Os03g48770	LOC_Os07g01780	9.1279046
LOC_Os03g48750	LOC_Os06g40640	10.3065651
LOC_Os03g49220	LOC_Os08g44960	9.04555078
LOC_Os03g48750	LOC_Os06g04030	11.3065852
LOC_Os03g49220	LOC_Os03g58430	9.94679211
LOC_Os03g48750	LOC_Os03g48770	9.77910016
LOC_Os03g48760	LOC_Os06g05690	8.90233551
LOC_Os03g48471	LOC_Os12g44000	9.23877314
LOC_Os03g49260	LOC_Os03g57120	8.90341333
LOC_Os03g48471	LOC_Os10g40600	8.80796
LOC_Os03g49220	LOC_Os06g46340	9.31112964
LOC_Os03g48770	LOC_Os03g48780	11.4796866

InteractorA	InteractorB	Z score
LOC_Os03g48760	LOC_Os12g07980	9.15654013
LOC_Os03g48390	LOC_Os07g46830	8.92684469
LOC_Os03g48471	LOC_Os06g07080	9.47162386
LOC_Os03g48760	LOC_Os04g52340	8.83708737
LOC_Os03g48471	LOC_Os10g35070	9.97181907
LOC_Os03g48390	LOC_Os12g21798	9.05520958
LOC_Os03g48471	LOC_Os09g23300	8.86777887
LOC_Os03g48760	LOC_Os08g08500	9.04412822
LOC_Os03g48471	LOC_Os11g31530	9.63226462
LOC_Os03g49220	LOC_Os12g34450	9.11729093
LOC_Os03g48780	LOC_Os09g23530	9.73872055
LOC_Os03g48750	LOC_Os07g28480	9.03330414
LOC_Os03g48390	LOC_Os05g48510	8.85647752
LOC_Os03g48760	LOC_Os05g19670	10.5693607
LOC_Os03g48390	LOC_Os06g04000	10.4946204
LOC_Os03g49220	LOC_Os08g06100	9.43744946
LOC_Os03g48390	LOC_Os05g49890	9.84307253
LOC_Os03g48760	LOC_Os03g48770	13.8764099
LOC_Os03g48390	LOC_Os07g05800	9.34457015
LOC_Os03g48750	LOC_Os10g38234	10.2966058
LOC_Os03g48390	LOC_Os07g47990	9.34410524
LOC_Os03g49220	LOC_Os03g55090	9.04285631
LOC_Os03g48780	LOC_Os03g52460	9.97398626
LOC_Os03g48750	LOC_Os05g41080	9.00122467
LOC_Os03g48471	LOC_Os11g24560	10.8451063
LOC_Os03g48770	LOC_Os05g19670	12.5957876
LOC_Os03g48390	LOC_Os08g32620	9.27975831
LOC_Os03g49220	LOC_Os09g10260	10.2823135
LOC_Os03g48780	LOC_Os03g53800	8.80059207
LOC_Os03g48471	LOC_Os06g14510	10.8138869
LOC_Os03g48390	LOC_Os12g23170	9.43269733
LOC_Os03g49260	LOC_Os06g09910	9.00356971
LOC_Os03g49260	LOC_Os06g45120	9.32659495
LOC_Os03g48760	LOC_Os12g12590	9.02401098
LOC_Os03g49220	LOC_Os07g42960	8.92263567
LOC_Os03g49260	LOC_Os03g55280	9.00766404
LOC_Os03g49220	LOC_Os03g61600	9.71638194
LOC_Os03g48750	LOC_Os04g39440	9.25366213
LOC_Os03g48760	LOC_Os03g52840	9.08022366
LOC_Os03g49220	LOC_Os05g38530	8.83187338
LOC_Os03g48760	LOC_Os08g16910	9.36235878
LOC_Os03g49260	LOC_Os05g35400	9.34432767
LOC_Os03g49260	LOC_Os05g48040	9.07327138

InteractorA	InteractorB	Z score
LOC_Os03g49260	LOC_Os05g33730	9.81015941
LOC_Os03g48471	LOC_Os12g38750	8.99073023
LOC_Os03g49260	LOC_Os06g06090	10.3101432
LOC_Os03g48471	LOC_Os04g56320	8.97790013
LOC_Os03g48471	LOC_Os05g09440	10.0735438
LOC_Os03g48770	LOC_Os05g44760	9.41220721
LOC_Os03g49260	LOC_Os05g47890	10.0422343
LOC_Os03g48750	LOC_Os05g19670	12.4352418
LOC_Os03g49220	LOC_Os09g30418	9.19599506
LOC_Os03g48760	LOC_Os12g18900	10.7758184
LOC_Os03g48770	LOC_Os07g20544	9.30140681
LOC_Os03g48780	LOC_Os09g20090	8.88382001
LOC_Os03g49220	LOC_Os08g03040	9.97319462
LOC_Os03g48750	LOC_Os03g48760	10.3290066
LOC_Os03g48760	LOC_Os06g04030	9.00357994
LOC_Os03g48760	LOC_Os05g47640	9.68250301
LOC_Os03g48390	LOC_Os06g37080	8.84600183
LOC_Os03g48760	LOC_Os03g48780	13.316043
LOC_Os03g48471	LOC_Os04g44870	8.96779315
LOC_Os03g49220	LOC_Os05g46360	11.4084637
LOC_Os03g48471	LOC_Os09g07830	9.5927601
LOC_Os03g48780	LOC_Os04g52100	8.87240865
LOC_Os03g48471	LOC_Os07g35880	8.88930536
LOC_Os03g49220	LOC_Os10g25674	9.09524541
LOC_Os03g49260	LOC_Os05g31140	9.45735894
LOC_Os03g48770	LOC_Os08g32870	8.99683582
LOC_Os03g48390	LOC_Os07g47420	9.04551998
LOC_Os03g48750	LOC_Os03g48780	9.87360087
LOC_Os03g50250	LOC_Os07g47420	9.58706709
LOC_Os03g49380	LOC_Os04g08350	9.4100998
LOC_Os03g49350	LOC_Os04g16828	8.95880885
LOC_Os03g49380	LOC_Os07g07770	10.1977595
LOC_Os03g49380	LOC_Os04g56950	10.3187391
LOC_Os03g49600	LOC_Os07g47490	8.8633149
LOC_Os03g49610	LOC_Os08g27840	9.01349217
LOC_Os03g49600	LOC_Os12g41220	9.50413989
LOC_Os03g49600	LOC_Os07g03590	8.99213872
LOC_Os03g49600	LOC_Os06g15420	8.83752876
LOC_Os03g49600	LOC_Os03g52860	9.19687487
LOC_Os03g49600	LOC_Os05g47890	9.4540684
LOC_Os03g49380	LOC_Os10g38340	9.53421999
LOC_Os03g49350	LOC_Os05g33400	9.72035576
LOC_Os03g49600	LOC_Os07g03288	8.99213872

InteractorA	InteractorB	Z score
LOC_Os03g49350	LOC_Os06g06320	9.00140292
LOC_Os03g49580	LOC_Os10g41689	8.85996249
LOC_Os03g49260	LOC_Os08g37800	9.53644137
LOC_Os03g49580	LOC_Os07g48060	9.01344341
LOC_Os03g49600	LOC_Os08g28730	9.6243809
LOC_Os03g49350	LOC_Os09g30418	9.00851038
LOC_Os03g49380	LOC_Os07g44740	8.93820022
LOC_Os03g49380	LOC_Os12g13380	9.44587659
LOC_Os03g49350	LOC_Os04g34630	9.33974958
LOC_Os03g50250	LOC_Os04g35200	10.2274986
LOC_Os03g49600	LOC_Os06g09450	9.49839393
LOC_Os03g49610	LOC_Os10g25930	8.90454065
LOC_Os03g49380	LOC_Os07g26540	9.77580881
LOC_Os03g50250	LOC_Os08g02410	8.99009579
LOC_Os03g49350	LOC_Os04g40310	9.93747931
LOC_Os03g49580	LOC_Os11g32260	9.90086873
LOC_Os03g49350	LOC_Os07g01760	9.5383369
LOC_Os03g49600	LOC_Os07g02340	10.5209467
LOC_Os03g49350	LOC_Os11g47760	8.93258083
LOC_Os03g49600	LOC_Os08g42560	8.96042782
LOC_Os03g50290	LOC_Os05g19380	9.78441776
LOC_Os03g49600	LOC_Os04g55040	9.38647743
LOC_Os03g50250	LOC_Os10g40700	8.96326835
LOC_Os03g49600	LOC_Os11g37550	11.1475295
LOC_Os03g49610	LOC_Os03g55800	9.55462678
LOC_Os03g50250	LOC_Os04g53810	9.83411743
LOC_Os03g49380	LOC_Os03g52860	9.15150799
LOC_Os03g50250	LOC_Os04g58580	8.94245849
LOC_Os03g49380	LOC_Os12g31370	8.93565371
LOC_Os03g50250	LOC_Os09g20090	8.85313042
LOC_Os03g49380	LOC_Os11g24560	8.80939837
LOC_Os03g49350	LOC_Os12g40550	9.40651639
LOC_Os03g50250	LOC_Os04g28180	8.90734147
LOC_Os03g49260	LOC_Os10g08022	8.98478428
LOC_Os03g50250	LOC_Os10g05069	9.62669889
LOC_Os03g49580	LOC_Os03g60620	9.51639153
LOC_Os03g49350	LOC_Os07g06970	9.08669743
LOC_Os03g49350	LOC_Os10g27174	9.11990073
LOC_Os03g49610	LOC_Os05g43510	8.91842789
LOC_Os03g50250	LOC_Os07g41050	8.90037705
LOC_Os03g49380	LOC_Os06g07210	9.37571813
LOC_Os03g49600	LOC_Os05g01050	8.8703429
LOC_Os03g49380	LOC_Os08g31870	8.97508357

InteractorA	InteractorB	Z score
LOC_Os03g49350	LOC_Os10g21342	8.95880012
LOC_Os03g49580	LOC_Os07g49400	8.96054655
LOC_Os03g49610	LOC_Os06g22140	9.87854476
LOC_Os03g50250	LOC_Os04g52450	8.90931633
LOC_Os03g50250	LOC_Os07g12730	10.2174548
LOC_Os03g49260	LOC_Os07g39290	8.80126272
LOC_Os03g50250	LOC_Os05g27940	8.94157675
LOC_Os03g50290	LOC_Os03g56460	9.10599034
LOC_Os03g49600	LOC_Os05g30530	9.0231572
LOC_Os03g49600	LOC_Os04g16680	8.90758769
LOC_Os03g50250	LOC_Os08g35740	9.06233023
LOC_Os03g49380	LOC_Os11g04954	8.88436809
LOC_Os03g49610	LOC_Os05g41900	8.96703111
LOC_Os03g49600	LOC_Os07g32800	9.41787999
LOC_Os03g50250	LOC_Os10g40090	8.93434543
LOC_Os03g50250	LOC_Os05g08430	9.71722922
LOC_Os03g49380	LOC_Os07g35940	8.99905027
LOC_Os03g49600	LOC_Os12g12560	9.94983763
LOC_Os03g50250	LOC_Os11g03400	9.33874014
LOC_Os03g49350	LOC_Os08g43170	9.22661808
LOC_Os03g49350	LOC_Os05g46580	9.22595137
LOC_Os03g49600	LOC_Os03g55389	9.28194877
LOC_Os03g49380	LOC_Os03g57120	11.6078397
LOC_Os03g49380	LOC_Os05g44340	10.5786217
LOC_Os03g49380	LOC_Os03g63720	9.06798716
LOC_Os03g50250	LOC_Os08g04180	8.87185496
LOC_Os03g49350	LOC_Os11g25260	9.11810975
LOC_Os03g49350	LOC_Os08g41880	9.14749159
LOC_Os03g50250	LOC_Os05g48510	9.42786024
LOC_Os03g49380	LOC_Os10g01570	9.45757407
LOC_Os03g49600	LOC_Os07g09890	9.94245348
LOC_Os03g50250	LOC_Os09g31490	9.95376696
LOC_Os03g49610	LOC_Os05g31140	8.97713486
LOC_Os03g49260	LOC_Os12g08270	8.97470694
LOC_Os03g49580	LOC_Os07g30170	9.09447734
LOC_Os03g49600	LOC_Os05g22724	9.25883929
LOC_Os03g50250	LOC_Os05g43510	10.4982425
LOC_Os03g49350	LOC_Os11g10480	9.3436755
LOC_Os03g49600	LOC_Os08g10510	10.5255199
LOC_Os03g49600	LOC_Os12g16240	8.93629933
LOC_Os03g49350	LOC_Os08g20730	9.11236738
LOC_Os03g50250	LOC_Os10g32970	9.82546011
LOC_Os03g50250	LOC_Os09g39400	8.83654722

InteractorA	InteractorB	Z score
LOC_Os03g49380	LOC_Os04g26870	9.33302268
LOC_Os03g49380	LOC_Os06g12090	9.04351332
LOC_Os03g49610	LOC_Os11g47760	8.89578691
LOC_Os03g49600	LOC_Os05g11710	9.73092113
LOC_Os03g49610	LOC_Os07g05180	9.94762313
LOC_Os03g50250	LOC_Os03g63410	9.68263018
LOC_Os03g49600	LOC_Os11g30290	11.0451375
LOC_Os03g49600	LOC_Os07g03377	8.99213872
LOC_Os03g49260	LOC_Os07g07470	9.53430934
LOC_Os03g49350	LOC_Os07g09890	9.56104082
LOC_Os03g49600	LOC_Os05g46270	12.4308936
LOC_Os03g49260	LOC_Os12g24650	10.0153856
LOC_Os03g49600	LOC_Os12g40830	12.1785153
LOC_Os03g49610	LOC_Os03g51740	8.88821517
LOC_Os03g49600	LOC_Os06g35730	9.7309454
LOC_Os03g49380	LOC_Os10g40700	9.47821857
LOC_Os03g49610	LOC_Os06g40180	8.93996837
LOC_Os03g49580	LOC_Os05g03820	9.19261938
LOC_Os03g49350	LOC_Os11g08330	9.19031023
LOC_Os03g49580	LOC_Os11g08340	9.07144687
LOC_Os03g49600	LOC_Os06g35490	9.28181158
LOC_Os03g49380	LOC_Os12g18900	9.99110086
LOC_Os03g49600	LOC_Os06g24390	8.89705556
LOC_Os03g49580	LOC_Os08g32850	9.36782001
LOC_Os03g49350	LOC_Os03g63330	8.85645283
LOC_Os03g49350	LOC_Os05g22722	8.95880012
LOC_Os03g49380	LOC_Os12g06660	9.01046649
LOC_Os03g49260	LOC_Os07g06970	8.99214725
LOC_Os03g49350	LOC_Os08g15276	8.95880012
LOC_Os03g50250	LOC_Os04g09670	9.31726906
LOC_Os03g49380	LOC_Os04g33240	9.44264073
LOC_Os03g49260	LOC_Os09g23560	9.14398847
LOC_Os03g49380	LOC_Os12g16250	8.88658088
LOC_Os03g49380	LOC_Os11g05470	9.81030179
LOC_Os03g49600	LOC_Os07g34140	9.34692457
LOC_Os03g50250	LOC_Os10g42940	8.84428622
LOC_Os03g49600	LOC_Os06g07210	9.46245787
LOC_Os03g50250	LOC_Os12g03090	9.33874014
LOC_Os03g49600	LOC_Os05g01810	8.84473606
LOC_Os03g49600	LOC_Os11g03230	8.90157007
LOC_Os03g50250	LOC_Os12g12590	8.98098454
LOC_Os03g49380	LOC_Os06g45120	9.30132106
LOC_Os03g49350	LOC_Os06g46940	9.40021787

InteractorA	InteractorB	Z score
LOC_Os03g50250	LOC_Os05g02310	9.50415002
LOC_Os03g49380	LOC_Os06g39230	9.71074408
LOC_Os03g50250	LOC_Os06g35560	10.8647036
LOC_Os03g49610	LOC_Os10g26010	9.02887769
LOC_Os03g49600	LOC_Os07g03467	8.99213872
LOC_Os03g50250	LOC_Os06g36700	9.85978228
LOC_Os03g49260	LOC_Os12g25120	8.90168273
LOC_Os03g49380	LOC_Os08g34790	9.59128361
LOC_Os03g50250	LOC_Os04g44470	9.17079249
LOC_Os03g49600	LOC_Os07g31750	9.02722739
LOC_Os03g49380	LOC_Os04g01980	10.322304
LOC_Os03g49260	LOC_Os07g30170	8.88663483
LOC_Os03g49600	LOC_Os10g21266	9.25143439
LOC_Os03g49350	LOC_Os10g22450	9.38770395
LOC_Os03g49600	LOC_Os11g31620	9.63826237
LOC_Os03g49610	LOC_Os06g23780	9.12349561
LOC_Os03g51080	LOC_Os06g24990	10.8052171
LOC_Os03g51040	LOC_Os10g03540	9.03980555
LOC_Os03g51040	LOC_Os03g55874	9.04704954
LOC_Os03g50885	LOC_Os09g12660	10.0464711
LOC_Os03g50490	LOC_Os05g39960	10.0714389
LOC_Os03g50490	LOC_Os04g37820	8.80266804
LOC_Os03g51040	LOC_Os09g36800	9.13922697
LOC_Os03g51080	LOC_Os08g09240	9.84501253
LOC_Os03g50885	LOC_Os10g37210	9.8010691
LOC_Os03g51040	LOC_Os08g40930	8.84694461
LOC_Os03g51200	LOC_Os05g38740	9.10305633
LOC_Os03g50490	LOC_Os07g42950	9.97559974
LOC_Os03g50480	LOC_Os04g24520	9.3990868
LOC_Os03g50885	LOC_Os10g42280	10.0148751
LOC_Os03g50490	LOC_Os08g25734	11.3121393
LOC_Os03g50885	LOC_Os07g03730	9.08879338
LOC_Os03g50885	LOC_Os09g37540	8.86155038
LOC_Os03g51040	LOC_Os04g55960	8.85581483
LOC_Os03g50290	LOC_Os09g11230	9.35328155
LOC_Os03g50480	LOC_Os05g33140	9.45750003
LOC_Os03g50885	LOC_Os10g35070	8.92403435
LOC_Os03g50490	LOC_Os04g44920	9.38163765
LOC_Os03g50490	LOC_Os10g01570	9.65759639
LOC_Os03g51040	LOC_Os11g25260	8.8724288
LOC_Os03g51040	LOC_Os05g38760	8.9302937
LOC_Os03g51200	LOC_Os10g39410	9.10305633
LOC_Os03g50440	LOC_Os09g25390	9.0371387

InteractorA	InteractorB	Z score
LOC_Os03g50480	LOC_Os03g58430	9.35081389
LOC_Os03g51200	LOC_Os04g40874	9.09590953
LOC_Os03g50440	LOC_Os04g38600	10.4375873
LOC_Os03g50885	LOC_Os07g32800	10.0903992
LOC_Os03g51080	LOC_Os04g37500	20.5865606
LOC_Os03g51200	LOC_Os07g47290	9.78753043
LOC_Os03g50480	LOC_Os12g35570	9.31495322
LOC_Os03g50885	LOC_Os09g10260	9.39490284
LOC_Os03g51080	LOC_Os04g53240	10.0522177
LOC_Os03g50490	LOC_Os04g10010	9.59315848
LOC_Os03g50885	LOC_Os05g07880	8.99918492
LOC_Os03g50885	LOC_Os10g25930	8.85315961
LOC_Os03g50480	LOC_Os10g21326	8.91945989
LOC_Os03g51040	LOC_Os06g08080	9.43695943
LOC_Os03g51040	LOC_Os05g29880	9.42528906
LOC_Os03g51080	LOC_Os07g48430	9.28353203
LOC_Os03g50885	LOC_Os12g43440	9.43509904
LOC_Os03g51080	LOC_Os06g34690	9.57006708
LOC_Os03g50480	LOC_Os07g34520	10.4990357
LOC_Os03g50885	LOC_Os03g61970	8.8745675
LOC_Os03g50490	LOC_Os05g04490	8.82249256
LOC_Os03g50885	LOC_Os05g49770	9.08554832
LOC_Os03g51200	LOC_Os05g36010	8.8405372
LOC_Os03g51200	LOC_Os09g09230	9.08239832
LOC_Os03g50480	LOC_Os05g50890	9.73608425
LOC_Os03g50490	LOC_Os06g08770	9.94735829
LOC_Os03g51200	LOC_Os05g24580	8.87087329
LOC_Os03g50885	LOC_Os07g08880	9.21717575
LOC_Os03g50490	LOC_Os05g43820	10.0187986
LOC_Os03g50490	LOC_Os09g09270	9.60698247
LOC_Os03g51200	LOC_Os04g49420	9.10305633
LOC_Os03g50885	LOC_Os07g49400	9.80671761
LOC_Os03g50480	LOC_Os10g30200	9.52126901
LOC_Os03g50885	LOC_Os12g34874	9.07012995
LOC_Os03g50490	LOC_Os07g02340	9.1852884
LOC_Os03g51200	LOC_Os09g26340	9.10305633
LOC_Os03g51040	LOC_Os07g19040	9.2936202
LOC_Os03g51200	LOC_Os08g29170	9.06500245
LOC_Os03g51040	LOC_Os09g30412	8.94537097
LOC_Os03g50490	LOC_Os07g03690	9.57834013
LOC_Os03g50490	LOC_Os09g25370	8.94723504
LOC_Os03g50885	LOC_Os07g42950	8.84782523
LOC_Os03g50490	LOC_Os03g52090	9.0588268

InteractorA	InteractorB	Z score
LOC_Os03g50490	LOC_Os06g15990	8.92751212
LOC_Os03g50885	LOC_Os04g35200	9.95380988
LOC_Os03g51200	LOC_Os09g38020	9.10305633
LOC_Os03g50885	LOC_Os11g42350	9.60820699
LOC_Os03g50440	LOC_Os05g33410	9.08870144
LOC_Os03g51040	LOC_Os06g21980	8.97063458
LOC_Os03g51080	LOC_Os10g31000	8.85954413
LOC_Os03g51200	LOC_Os05g39050	9.10305633
LOC_Os03g50440	LOC_Os12g25710	9.390417
LOC_Os03g51200	LOC_Os07g36500	9.10305633
LOC_Os03g50480	LOC_Os06g11210	9.99326213
LOC_Os03g50885	LOC_Os09g23560	9.78539403
LOC_Os03g51200	LOC_Os09g23560	9.31417866
LOC_Os03g50290	LOC_Os10g33800	9.24081955
LOC_Os03g50490	LOC_Os12g25700	9.0598583
LOC_Os03g50480	LOC_Os04g02050	8.81555472
LOC_Os03g50480	LOC_Os04g16844	8.91945989
LOC_Os03g50290	LOC_Os10g30840	8.95122097
LOC_Os03g51080	LOC_Os05g37700	9.10825845
LOC_Os03g51200	LOC_Os07g36140	9.34349473
LOC_Os03g50885	LOC_Os09g21770	9.4561887
LOC_Os03g50490	LOC_Os07g34190	9.2815244
LOC_Os03g50440	LOC_Os04g41960	8.91854783
LOC_Os03g51200	LOC_Os05g38560	11.8009355
LOC_Os03g50490	LOC_Os10g38140	9.05138455
LOC_Os03g50290	LOC_Os12g33610	9.0752558
LOC_Os03g52860	LOC_Os08g42410	10.0737937
LOC_Os03g52860	LOC_Os04g41310	9.15142133
LOC_Os03g51600	LOC_Os07g43260	8.80106634
LOC_Os03g52860	LOC_Os05g12190	9.66922297
LOC_Os03g52840	LOC_Os10g21248	8.92502847
LOC_Os03g52860	LOC_Os10g30580	11.294425
LOC_Os03g51550	LOC_Os05g46000	8.98048905
LOC_Os03g51740	LOC_Os03g53230	9.60145222
LOC_Os03g52840	LOC_Os08g09250	9.49235142
LOC_Os03g52090	LOC_Os05g30530	9.58136997
LOC_Os03g52840	LOC_Os08g15030	8.88074224
LOC_Os03g52460	LOC_Os10g02040	11.2127056
LOC_Os03g52460	LOC_Os06g11290	9.183646
LOC_Os03g52460	LOC_Os08g31870	8.82250529
LOC_Os03g51600	LOC_Os06g04280	9.6158845
LOC_Os03g52840	LOC_Os10g01570	9.1231154
LOC_Os03g51600	LOC_Os09g16910	8.88688988

InteractorA	InteractorB	Z score
LOC_Os03g51740	LOC_Os05g10780	10.9439561
LOC_Os03g52860	LOC_Os03g56840	9.37353475
LOC_Os03g51740	LOC_Os09g38620	9.64618328
LOC_Os03g52460	LOC_Os06g06090	8.96263897
LOC_Os03g52860	LOC_Os06g15420	9.76848049
LOC_Os03g52090	LOC_Os05g25850	9.45935048
LOC_Os03g51550	LOC_Os10g40720	9.07586265
LOC_Os03g51200	LOC_Os12g01922	9.279408
LOC_Os03g52860	LOC_Os04g47360	9.30173724
LOC_Os03g52860	LOC_Os04g57400	9.28371865
LOC_Os03g52840	LOC_Os10g38740	8.8707622
LOC_Os03g52460	LOC_Os05g41080	8.9861092
LOC_Os03g51600	LOC_Os12g02980	9.78388381
LOC_Os03g52090	LOC_Os07g02340	8.8659962
LOC_Os03g52460	LOC_Os11g18870	8.80679127
LOC_Os03g52860	LOC_Os10g38350	9.45935404
LOC_Os03g51740	LOC_Os08g38920	9.18847518
LOC_Os03g52090	LOC_Os07g05580	9.57249542
LOC_Os03g52860	LOC_Os03g64330	9.49381236
LOC_Os03g52840	LOC_Os09g32830	9.63426556
LOC_Os03g52840	LOC_Os08g28190	9.40780763
LOC_Os03g51740	LOC_Os06g51084	8.85889475
LOC_Os03g52860	LOC_Os08g02410	10.6772914
LOC_Os03g51740	LOC_Os12g40550	10.0587596
LOC_Os03g51600	LOC_Os07g38540	9.11510041
LOC_Os03g51550	LOC_Os06g35560	9.35772332
LOC_Os03g51600	LOC_Os05g28280	9.28171148
LOC_Os03g51740	LOC_Os08g33710	10.4216854
LOC_Os03g51740	LOC_Os05g01490	8.92591611
LOC_Os03g51740	LOC_Os11g03290	9.66873286
LOC_Os03g51600	LOC_Os12g10730	8.80207484
LOC_Os03g52460	LOC_Os10g25930	10.0075828
LOC_Os03g52090	LOC_Os07g48880	8.93763007
LOC_Os03g52460	LOC_Os07g08500	9.90058468
LOC_Os03g52860	LOC_Os04g30420	8.95954653
LOC_Os03g52090	LOC_Os12g12580	10.132916
LOC_Os03g52090	LOC_Os06g27770	9.41911961
LOC_Os03g52860	LOC_Os05g09500	8.85917621
LOC_Os03g51550	LOC_Os03g53690	8.834524
LOC_Os03g52090	LOC_Os06g41810	10.4549375
LOC_Os03g51740	LOC_Os06g21820	8.95629295
LOC_Os03g52090	LOC_Os12g23170	9.37126688
LOC_Os03g51550	LOC_Os10g38780	9.10113718

InteractorA	InteractorB	Z score
LOC_Os03g51740	LOC_Os08g08070	9.42381958
LOC_Os03g52840	LOC_Os08g02410	8.82491842
LOC_Os03g52460	LOC_Os05g50890	8.83801391
LOC_Os03g51740	LOC_Os12g38180	9.99699749
LOC_Os03g52460	LOC_Os09g24924	10.0338381
LOC_Os03g51550	LOC_Os12g44000	8.96028507
LOC_Os03g52860	LOC_Os10g41410	9.45942435
LOC_Os03g52840	LOC_Os10g21240	9.14218376
LOC_Os03g52860	LOC_Os10g38730	8.83632006
LOC_Os03g51740	LOC_Os05g02310	8.90221809
LOC_Os03g52460	LOC_Os03g56410	9.74289172
LOC_Os03g51550	LOC_Os06g45100	8.9527058
LOC_Os03g52090	LOC_Os05g19380	9.55322942
LOC_Os03g52460	LOC_Os09g27420	9.40050217
LOC_Os03g51740	LOC_Os10g38690	10.2520618
LOC_Os03g52970	LOC_Os04g33240	9.41422354
LOC_Os03g51200	LOC_Os12g34874	9.57757988
LOC_Os03g52460	LOC_Os04g37990	9.98091286
LOC_Os03g51740	LOC_Os06g47320	8.87132917
LOC_Os03g51740	LOC_Os06g08080	10.0467643
LOC_Os03g52840	LOC_Os10g38640	9.11387406
LOC_Os03g51600	LOC_Os10g23100	9.16524143
LOC_Os03g52860	LOC_Os12g04924	10.4853678
LOC_Os03g51200	LOC_Os12g10600	8.8582048
LOC_Os03g51600	LOC_Os10g27050	10.5586852
LOC_Os03g52460	LOC_Os07g42960	9.01194055
LOC_Os03g52460	LOC_Os03g64210	9.40830345
LOC_Os03g51740	LOC_Os06g46340	9.71804499
LOC_Os03g52460	LOC_Os06g43660	8.93977017
LOC_Os03g52460	LOC_Os06g46000	9.43002191
LOC_Os03g52840	LOC_Os04g10010	10.6674186
LOC_Os03g52860	LOC_Os05g46550	9.75425385
LOC_Os03g51740	LOC_Os06g01850	8.90231938
LOC_Os03g52840	LOC_Os10g07229	9.68579245
LOC_Os03g52860	LOC_Os07g09890	9.24594618
LOC_Os03g52860	LOC_Os05g45810	8.92364827
LOC_Os03g51740	LOC_Os12g02980	9.43025595
LOC_Os03g52460	LOC_Os04g33970	10.2809309
LOC_Os03g51740	LOC_Os07g31770	9.03577522
LOC_Os03g52860	LOC_Os05g31040	9.21735035
LOC_Os03g51740	LOC_Os05g51480	9.0565519
LOC_Os03g51550	LOC_Os10g01570	9.98961211
LOC_Os03g51550	LOC_Os11g03230	9.10120402

InteractorA	InteractorB	Z score
LOC_Os03g51740	LOC_Os12g04980	9.55889633
LOC_Os03g51740	LOC_Os08g10010	9.20169308
LOC_Os03g53790	LOC_Os09g31486	9.05248321
LOC_Os03g53690	LOC_Os06g46372	9.11084686
LOC_Os03g53690	LOC_Os08g28820	9.23690096
LOC_Os03g53800	LOC_Os04g39020	11.5440381
LOC_Os03g52970	LOC_Os06g22960	8.80077445
LOC_Os03g53230	LOC_Os06g34690	10.217687
LOC_Os03g53200	LOC_Os11g38959	9.25209699
LOC_Os03g53650	LOC_Os04g52450	9.42426259
LOC_Os03g53650	LOC_Os06g43660	9.2935944
LOC_Os03g53800	LOC_Os05g33150	9.46770054
LOC_Os03g53650	LOC_Os03g59710	8.93000407
LOC_Os03g53650	LOC_Os08g14770	9.92340924
LOC_Os03g52970	LOC_Os07g39870	9.11992064
LOC_Os03g53650	LOC_Os08g03040	10.8503484
LOC_Os03g53200	LOC_Os12g13380	9.53640316
LOC_Os03g53650	LOC_Os05g08100	11.4391264
LOC_Os03g53690	LOC_Os05g46580	10.4008661
LOC_Os03g53230	LOC_Os03g64210	9.6770643
LOC_Os03g53800	LOC_Os09g25150	8.9242261
LOC_Os03g53650	LOC_Os04g40990	10.3279028
LOC_Os03g53690	LOC_Os09g21770	8.94755614
LOC_Os03g53650	LOC_Os09g10200	9.3191432
LOC_Os03g53690	LOC_Os10g39840	8.80704713
LOC_Os03g53200	LOC_Os12g12514	9.35909983
LOC_Os03g53230	LOC_Os04g55040	8.94760544
LOC_Os03g53650	LOC_Os12g25700	9.9338834
LOC_Os03g53790	LOC_Os04g55040	8.81696101
LOC_Os03g53860	LOC_Os05g40990	9.73792493
LOC_Os03g52970	LOC_Os10g41550	10.7885533
LOC_Os03g53800	LOC_Os11g26910	11.86234
LOC_Os03g53860	LOC_Os05g05470	9.61697473
LOC_Os03g53200	LOC_Os10g26110	9.16400202
LOC_Os03g53650	LOC_Os06g15990	8.8674966
LOC_Os03g53790	LOC_Os12g17910	8.90466012
LOC_Os03g53230	LOC_Os12g13390	11.5977795
LOC_Os03g53690	LOC_Os09g27750	10.2924463
LOC_Os03g53790	LOC_Os05g47640	9.16741716
LOC_Os03g53650	LOC_Os08g09210	9.02163065
LOC_Os03g53200	LOC_Os06g21980	8.85845628
LOC_Os03g53790	LOC_Os09g14670	8.9680461
LOC_Os03g53800	LOC_Os05g46360	9.16427952

InteractorA	InteractorB	Z score
LOC_Os03g52970	LOC_Os07g03710	9.14864527
LOC_Os03g53790	LOC_Os12g25120	8.94684745
LOC_Os03g53650	LOC_Os05g08960	8.85689886
LOC_Os03g53230	LOC_Os04g58800	8.83814423
LOC_Os03g53790	LOC_Os04g12480	10.0706852
LOC_Os03g53650	LOC_Os12g16290	9.25929545
LOC_Os03g53230	LOC_Os06g04200	9.41071567
LOC_Os03g53800	LOC_Os05g33400	8.87682594
LOC_Os03g53230	LOC_Os07g46310	9.22883499
LOC_Os03g53800	LOC_Os07g05160	9.49412346
LOC_Os03g52970	LOC_Os07g48780	8.82728145
LOC_Os03g53650	LOC_Os06g46284	8.8201255
LOC_Os03g53690	LOC_Os08g06100	10.0758899
LOC_Os03g52970	LOC_Os10g07229	9.732429
LOC_Os03g53200	LOC_Os11g08470	9.58691697
LOC_Os03g53650	LOC_Os06g25010	9.46790559
LOC_Os03g53860	LOC_Os04g58710	10.051104
LOC_Os03g53650	LOC_Os03g55150	9.0860663
LOC_Os03g53200	LOC_Os11g08940	8.94841274
LOC_Os03g53650	LOC_Os07g06410	9.27947583
LOC_Os03g52970	LOC_Os07g12200	8.90788772
LOC_Os03g52970	LOC_Os12g34062	10.1275255
LOC_Os03g52970	LOC_Os12g24650	9.0393317
LOC_Os03g53860	LOC_Os05g46360	9.19699956
LOC_Os03g52970	LOC_Os12g12514	10.8019897
LOC_Os03g53800	LOC_Os06g40170	10.2151076
LOC_Os03g53790	LOC_Os04g55290	11.0454694
LOC_Os03g53200	LOC_Os08g42560	11.1352465
LOC_Os03g53650	LOC_Os03g55874	9.49724992
LOC_Os03g53230	LOC_Os05g41640	9.00390128
LOC_Os03g53860	LOC_Os04g18650	9.17900545
LOC_Os03g53690	LOC_Os04g56230	8.96842354
LOC_Os03g52970	LOC_Os08g43190	9.12059759
LOC_Os03g53800	LOC_Os08g39140	9.31565083
LOC_Os03g53790	LOC_Os08g09250	8.97286916
LOC_Os03g53800	LOC_Os04g31960	9.19119249
LOC_Os03g52970	LOC_Os08g32870	8.95715569
LOC_Os03g53690	LOC_Os09g38620	9.05089642
LOC_Os03g53860	LOC_Os05g09500	9.10751551
LOC_Os03g53690	LOC_Os11g05880	10.2270762
LOC_Os03g53200	LOC_Os06g51084	9.39487248
LOC_Os03g55600	LOC_Os07g36190	8.92129574
LOC_Os03g55090	LOC_Os04g58680	9.42575009

InteractorA	InteractorB	Z score
LOC_Os03g55600	LOC_Os11g32650	9.44044175
LOC_Os03g55070	LOC_Os06g39875	8.92168019
LOC_Os03g55620	LOC_Os09g35800	8.83024015
LOC_Os03g55150	LOC_Os06g35530	9.07790011
LOC_Os03g55090	LOC_Os07g27790	9.84041721
LOC_Os03g55389	LOC_Os05g38310	9.46564311
LOC_Os03g55070	LOC_Os05g38530	8.87692772
LOC_Os03g55090	LOC_Os09g07830	8.85016573
LOC_Os03g55620	LOC_Os06g21570	10.7508278
LOC_Os03g55150	LOC_Os08g14760	9.34423955
LOC_Os03g55090	LOC_Os04g32460	9.18782012
LOC_Os03g55620	LOC_Os04g52450	9.39548676
LOC_Os03g55090	LOC_Os12g12580	9.02126624
LOC_Os03g55600	LOC_Os10g20910	10.9947272
LOC_Os03g55389	LOC_Os11g32540	9.37125954
LOC_Os03g55070	LOC_Os11g10480	9.94916959
LOC_Os03g55600	LOC_Os05g44140	8.8304623
LOC_Os03g55070	LOC_Os06g01390	9.05452115
LOC_Os03g55090	LOC_Os04g43410	10.6786028
LOC_Os03g55280	LOC_Os06g05690	9.48723447
LOC_Os03g55090	LOC_Os12g40830	9.78947935
LOC_Os03g53860	LOC_Os11g47550	8.8703591
LOC_Os03g55620	LOC_Os09g31502	9.12839575
LOC_Os03g55150	LOC_Os09g33860	9.19980207
LOC_Os03g55090	LOC_Os12g02980	10.2143247
LOC_Os03g55620	LOC_Os06g05880	9.4765531
LOC_Os03g55090	LOC_Os04g40130	9.63153334
LOC_Os03g55090	LOC_Os04g40600	9.10869739
LOC_Os03g55600	LOC_Os12g08280	9.36551682
LOC_Os03g55280	LOC_Os10g07040	9.06645835
LOC_Os03g55600	LOC_Os05g01490	9.83130622
LOC_Os03g55280	LOC_Os04g02820	9.16546024
LOC_Os03g55620	LOC_Os03g55800	9.28141322
LOC_Os03g55070	LOC_Os05g41900	8.83045892
LOC_Os03g55620	LOC_Os07g47990	9.34619146
LOC_Os03g55389	LOC_Os11g04954	9.92647507
LOC_Os03g55090	LOC_Os03g58400	9.65027747
LOC_Os03g55070	LOC_Os08g34210	10.1218494
LOC_Os03g55090	LOC_Os07g41750	9.06830567
LOC_Os03g55389	LOC_Os09g38030	9.62823382
LOC_Os03g55389	LOC_Os06g04270	9.774382
LOC_Os03g55070	LOC_Os06g35560	9.37244099
LOC_Os03g55150	LOC_Os08g33820	9.05387659

InteractorA	InteractorB	Z score
LOC_Os03g55600	LOC_Os11g03980	10.9516671
LOC_Os03g55600	LOC_Os12g10720	8.805257
LOC_Os03g55600	LOC_Os07g39870	8.8902604
LOC_Os03g55070	LOC_Os08g31870	9.5921147
LOC_Os03g55070	LOC_Os07g38860	8.91227775
LOC_Os03g55090	LOC_Os04g27060	9.59329827
LOC_Os03g55280	LOC_Os10g23900	9.03550246
LOC_Os03g55620	LOC_Os04g52100	9.1779763
LOC_Os03g55389	LOC_Os04g52280	9.0654431
LOC_Os03g55090	LOC_Os05g36290	8.8220463
LOC_Os03g55620	LOC_Os10g29470	8.89723731
LOC_Os03g55620	LOC_Os06g40190	10.1475927
LOC_Os03g55070	LOC_Os10g28050	8.93759243
LOC_Os03g55090	LOC_Os06g25010	9.51152761
LOC_Os03g55090	LOC_Os06g35940	9.0922393
LOC_Os03g55070	LOC_Os04g41960	9.29146948
LOC_Os03g55070	LOC_Os09g23550	10.9074189
LOC_Os03g55070	LOC_Os06g04030	9.18268536
LOC_Os03g55620	LOC_Os10g34520	9.74355183
LOC_Os03g55150	LOC_Os07g42960	9.73805789
LOC_Os03g55150	LOC_Os11g47600	8.95655935
LOC_Os03g55389	LOC_Os04g35200	8.91728223
LOC_Os03g55600	LOC_Os05g44760	8.85250077
LOC_Os03g55600	LOC_Os10g38160	9.10244861
LOC_Os03g55280	LOC_Os05g47545	9.35537341
LOC_Os03g55600	LOC_Os10g27174	9.0782572
LOC_Os03g55389	LOC_Os09g25370	9.37650505
LOC_Os03g55280	LOC_Os08g04540	9.01161148
LOC_Os03g55090	LOC_Os08g44270	11.0804073
LOC_Os03g55090	LOC_Os06g01850	10.5094319
LOC_Os03g55090	LOC_Os05g40990	9.80340559
LOC_Os03g55280	LOC_Os10g41480	9.31774572
LOC_Os03g55600	LOC_Os09g27750	9.62205449
LOC_Os03g55150	LOC_Os03g59310	10.4277248
LOC_Os03g55280	LOC_Os04g55290	9.18348538
LOC_Os03g55090	LOC_Os05g09440	8.9453827
LOC_Os03g55070	LOC_Os06g40640	9.48175503
LOC_Os03g55620	LOC_Os05g47980	8.89697002
LOC_Os03g55620	LOC_Os07g05940	8.94995719
LOC_Os03g55150	LOC_Os09g28420	10.0316012
LOC_Os03g53860	LOC_Os08g44530	9.13529735
LOC_Os03g55090	LOC_Os09g39500	9.59068928
LOC_Os03g55280	LOC_Os10g25930	9.12928814

InteractorA	InteractorB	Z score
LOC_Os03g55090	LOC_Os07g05580	10.0328764
LOC_Os03g55070	LOC_Os12g25700	10.508368
LOC_Os03g55070	LOC_Os12g40510	10.5838871
LOC_Os03g55070	LOC_Os11g01872	8.91685232
LOC_Os03g55070	LOC_Os12g23630	9.16685665
LOC_Os03g55090	LOC_Os12g02370	8.8005065
LOC_Os03g55150	LOC_Os05g49840	9.23389522
LOC_Os03g55090	LOC_Os08g29170	8.84290609
LOC_Os03g55070	LOC_Os05g08430	9.24496566
LOC_Os03g55280	LOC_Os06g37560	10.1437444
LOC_Os03g55280	LOC_Os07g08500	8.9576189
LOC_Os03g55150	LOC_Os04g12900	8.87838677
LOC_Os03g55070	LOC_Os04g16872	9.13443881
LOC_Os03g55389	LOC_Os08g33100	12.3019163
LOC_Os03g55070	LOC_Os06g01590	8.8501169
LOC_Os03g55280	LOC_Os12g38750	9.04787038
LOC_Os03g55070	LOC_Os09g27420	11.2803301
LOC_Os03g55389	LOC_Os04g01674	8.93433583
LOC_Os03g55150	LOC_Os11g14220	9.11435584
LOC_Os03g55389	LOC_Os10g27190	9.51640608
LOC_Os03g55070	LOC_Os12g18880	10.3242572
LOC_Os03g53860	LOC_Os07g30170	9.49262419
LOC_Os03g55090	LOC_Os06g06560	10.706563
LOC_Os03g55090	LOC_Os12g41110	9.24945046
LOC_Os03g55600	LOC_Os05g28280	8.9862157
LOC_Os03g55620	LOC_Os05g51630	9.08826489
LOC_Os03g55620	LOC_Os09g27820	8.87994033
LOC_Os03g55090	LOC_Os09g15400	9.74126375
LOC_Os03g55070	LOC_Os03g61340	9.27144008
LOC_Os03g55620	LOC_Os08g44530	9.13698476
LOC_Os03g55620	LOC_Os05g31140	10.4006992
LOC_Os03g55070	LOC_Os12g25690	13.6772402
LOC_Os03g55600	LOC_Os07g46310	9.06441964
LOC_Os03g55150	LOC_Os05g45810	10.1110579
LOC_Os03g55150	LOC_Os06g51084	10.3459759
LOC_Os03g55090	LOC_Os08g28800	10.3559893
LOC_Os03g55090	LOC_Os08g27840	10.0637492
LOC_Os03g53860	LOC_Os12g22650	9.42175889
LOC_Os03g55070	LOC_Os04g58570	9.51394998
LOC_Os03g55090	LOC_Os07g05940	9.98205923
LOC_Os03g55090	LOC_Os07g36130	9.17226115
LOC_Os03g55874	LOC_Os04g56160	9.28984583
LOC_Os03g56460	LOC_Os09g39570	9.34378192

InteractorA	InteractorB	Z score
LOC_Os03g55874	LOC_Os10g38140	9.29322484
LOC_Os03g56460	LOC_Os10g21310	8.91292322
LOC_Os03g56241	LOC_Os07g29750	8.88708428
LOC_Os03g56460	LOC_Os06g19960	9.4580766
LOC_Os03g55874	LOC_Os12g44350	12.8643838
LOC_Os03g56280	LOC_Os05g02310	8.80044313
LOC_Os03g56280	LOC_Os07g26690	9.96934872
LOC_Os03g56410	LOC_Os04g55740	8.95363779
LOC_Os03g56810	LOC_Os04g52361	9.10101387
LOC_Os03g56460	LOC_Os04g48850	8.91532024
LOC_Os03g55800	LOC_Os04g43800	9.19578567
LOC_Os03g55874	LOC_Os06g41810	9.39973676
LOC_Os03g56280	LOC_Os04g33190	9.81156374
LOC_Os03g56410	LOC_Os04g47220	10.1487287
LOC_Os03g56410	LOC_Os04g39864	10.254536
LOC_Os03g55800	LOC_Os04g33470	9.31606431
LOC_Os03g55874	LOC_Os03g58260	8.93228387
LOC_Os03g56410	LOC_Os03g62070	10.2618786
LOC_Os03g56460	LOC_Os05g48030	10.7084764
LOC_Os03g55874	LOC_Os10g38489	8.84808972
LOC_Os03g56280	LOC_Os05g43510	9.98601428
LOC_Os03g56410	LOC_Os11g10520	8.96065234
LOC_Os03g56460	LOC_Os06g10930	9.32388571
LOC_Os03g56460	LOC_Os07g41750	8.88931639
LOC_Os03g56280	LOC_Os04g36700	8.86571471
LOC_Os03g55874	LOC_Os12g12590	10.3591648
LOC_Os03g56241	LOC_Os04g18200	9.00929074
LOC_Os03g55874	LOC_Os12g18900	8.99404149
LOC_Os03g55800	LOC_Os04g32710	9.30844615
LOC_Os03g56241	LOC_Os04g25400	8.95980079
LOC_Os03g55874	LOC_Os11g37550	10.0806302
LOC_Os03g55800	LOC_Os04g58710	9.67761078
LOC_Os03g56460	LOC_Os04g45470	8.90539478
LOC_Os03g56280	LOC_Os05g46860	9.03007951
LOC_Os03g56280	LOC_Os04g43800	9.07807505
LOC_Os03g55874	LOC_Os03g62670	10.4063772
LOC_Os03g55620	LOC_Os10g41550	9.02426403
LOC_Os03g56241	LOC_Os05g31040	9.25648682
LOC_Os03g55874	LOC_Os11g29400	9.82932294
LOC_Os03g55874	LOC_Os03g60740	9.26239701
LOC_Os03g56460	LOC_Os07g05940	11.3761522
LOC_Os03g55874	LOC_Os04g41960	8.82487426
LOC_Os03g56460	LOC_Os05g05830	10.1155007

InteractorA	InteractorB	Z score
LOC_Os03g56460	LOC_Os11g29400	8.8678076
LOC_Os03g55874	LOC_Os12g12514	10.3060838
LOC_Os03g55800	LOC_Os04g44730	8.90877644
LOC_Os03g55800	LOC_Os11g38959	9.08840297
LOC_Os03g56241	LOC_Os10g39840	10.0680206
LOC_Os03g56410	LOC_Os06g02490	9.03216711
LOC_Os03g55874	LOC_Os03g61970	8.862667
LOC_Os03g55800	LOC_Os05g05800	9.04243842
LOC_Os03g56810	LOC_Os05g02940	10.2113339
LOC_Os03g56410	LOC_Os10g13800	9.23350344
LOC_Os03g56460	LOC_Os04g44920	9.07747873
LOC_Os03g56460	LOC_Os05g38230	9.10931653
LOC_Os03g56280	LOC_Os07g03690	10.5452518
LOC_Os03g56241	LOC_Os05g42150	9.09940679
LOC_Os03g56410	LOC_Os04g29550	9.21819731
LOC_Os03g56241	LOC_Os05g34540	9.32528594
LOC_Os03g55800	LOC_Os08g40140	8.96301986
LOC_Os03g56241	LOC_Os09g30360	10.8828682
LOC_Os03g56410	LOC_Os08g09940	9.52944462
LOC_Os03g56460	LOC_Os09g07830	9.36926062
LOC_Os03g56280	LOC_Os07g26540	8.85194909
LOC_Os03g55800	LOC_Os12g13390	9.32131915
LOC_Os03g56410	LOC_Os05g07720	10.4477078
LOC_Os03g55620	LOC_Os12g41110	9.97463873
LOC_Os03g55874	LOC_Os05g49880	9.18154169
LOC_Os03g55874	LOC_Os04g09670	8.91921769
LOC_Os03g56460	LOC_Os09g12570	9.19101957
LOC_Os03g56460	LOC_Os04g12900	8.95418072
LOC_Os03g55874	LOC_Os08g40140	9.53492716
LOC_Os03g56280	LOC_Os06g09450	10.5867987
LOC_Os03g56280	LOC_Os03g63330	11.3494219
LOC_Os03g55800	LOC_Os12g34450	11.2954976
LOC_Os03g56810	LOC_Os04g01674	9.35215604
LOC_Os03g56280	LOC_Os12g43630	9.87125436
LOC_Os03g55874	LOC_Os07g20544	11.5207542
LOC_Os03g56241	LOC_Os07g47490	9.82988885
LOC_Os03g55800	LOC_Os04g33970	9.28505627
LOC_Os03g55874	LOC_Os04g27980	9.23526718
LOC_Os03g56410	LOC_Os04g33470	9.09267912
LOC_Os03g55874	LOC_Os03g56460	8.89064338
LOC_Os03g56460	LOC_Os05g50380	8.8772404
LOC_Os03g56280	LOC_Os09g31430	9.43580891
LOC_Os03g56460	LOC_Os05g37390	9.87235137

InteractorA	InteractorB	Z score
LOC_Os03g55800	LOC_Os09g20090	8.80551236
LOC_Os03g56280	LOC_Os03g61970	9.62362857
LOC_Os03g55800	LOC_Os07g07550	9.32326294
LOC_Os03g55620	LOC_Os11g47570	8.90045896
LOC_Os03g56410	LOC_Os09g31486	9.5713805
LOC_Os03g56810	LOC_Os03g63090	8.86586641
LOC_Os03g56410	LOC_Os08g25734	11.0309339
LOC_Os03g55874	LOC_Os03g61360	11.4521137
LOC_Os03g55800	LOC_Os05g29880	10.2952823
LOC_Os03g55874	LOC_Os06g06730	9.75884504
LOC_Os03g55874	LOC_Os04g22730	8.97522796
LOC_Os03g55874	LOC_Os06g11280	11.8850592
LOC_Os03g56460	LOC_Os12g17910	10.3781817
LOC_Os03g56280	LOC_Os12g01922	8.86870268
LOC_Os03g56460	LOC_Os06g06560	9.31489516
LOC_Os03g56280	LOC_Os12g12580	9.27575394
LOC_Os03g55800	LOC_Os06g36820	9.01647465
LOC_Os03g56280	LOC_Os04g20810	9.22320301
LOC_Os03g57290	LOC_Os08g33710	9.80406422
LOC_Os03g56810	LOC_Os07g08880	10.5686393
LOC_Os03g56840	LOC_Os07g35880	10.0436094
LOC_Os03g56810	LOC_Os09g39500	10.2191959
LOC_Os03g56810	LOC_Os12g31640	9.77202578
LOC_Os03g57140	LOC_Os12g34062	10.0616645
LOC_Os03g57200	LOC_Os09g28420	9.13827671
LOC_Os03g57290	LOC_Os08g17680	10.3036055
LOC_Os03g57290	LOC_Os04g57380	9.033749
LOC_Os03g56810	LOC_Os08g06100	10.2570293
LOC_Os03g57040	LOC_Os06g07140	9.27013793
LOC_Os03g56810	LOC_Os06g35814	8.89837401
LOC_Os03g57120	LOC_Os08g15322	8.82296536
LOC_Os03g57120	LOC_Os12g43440	9.69590657
LOC_Os03g57290	LOC_Os04g08350	9.90731894
LOC_Os03g57120	LOC_Os08g42410	10.9032461
LOC_Os03g57120	LOC_Os12g13390	9.15733216
LOC_Os03g57130	LOC_Os08g44530	8.8128265
LOC_Os03g56840	LOC_Os09g10260	9.01315168
LOC_Os03g57140	LOC_Os04g44470	10.4792962
LOC_Os03g57040	LOC_Os03g63330	9.22334118
LOC_Os03g57120	LOC_Os06g03720	8.95665905
LOC_Os03g57120	LOC_Os06g06090	8.83756348
LOC_Os03g57200	LOC_Os05g38560	9.15555366
LOC_Os03g56840	LOC_Os08g37490	8.92180535

InteractorA	InteractorB	Z score
LOC_Os03g57290	LOC_Os07g06970	8.89665127
LOC_Os03g57140	LOC_Os09g39810	10.1828128
LOC_Os03g56840	LOC_Os07g26690	9.02988604
LOC_Os03g56810	LOC_Os05g49840	9.41635337
LOC_Os03g57120	LOC_Os09g23530	9.32574071
LOC_Os03g56840	LOC_Os06g05090	10.2932932
LOC_Os03g56840	LOC_Os12g03816	9.65111039
LOC_Os03g56840	LOC_Os07g46460	8.88579254
LOC_Os03g57130	LOC_Os11g38959	9.8890882
LOC_Os03g56810	LOC_Os10g41490	10.9406038
LOC_Os03g57120	LOC_Os11g05290	9.24267215
LOC_Os03g57120	LOC_Os10g21298	8.82296536
LOC_Os03g57120	LOC_Os12g44150	9.23342403
LOC_Os03g57200	LOC_Os04g56070	8.93350241
LOC_Os03g56810	LOC_Os08g06060	9.68385394
LOC_Os03g56840	LOC_Os04g39030	9.44338043
LOC_Os03g57140	LOC_Os06g35480	8.85330417
LOC_Os03g56840	LOC_Os10g35480	9.2515031
LOC_Os03g57200	LOC_Os05g45590	10.1172888
LOC_Os03g56840	LOC_Os08g20420	9.90764643
LOC_Os03g57200	LOC_Os07g38540	12.3110399
LOC_Os03g57140	LOC_Os10g38730	9.38284103
LOC_Os03g57130	LOC_Os04g55040	8.81117026
LOC_Os03g57120	LOC_Os07g05400	9.08850389
LOC_Os03g57120	LOC_Os05g51480	8.85859915
LOC_Os03g56810	LOC_Os06g03770	9.54931614
LOC_Os03g57140	LOC_Os08g01660	11.3421538
LOC_Os03g57130	LOC_Os04g33480	8.85473176
LOC_Os03g57120	LOC_Os06g39728	8.82296536
LOC_Os03g56840	LOC_Os08g42910	11.1154451
LOC_Os03g57120	LOC_Os07g34260	9.45488616
LOC_Os03g57290	LOC_Os07g05940	9.04060315
LOC_Os03g57200	LOC_Os04g48850	9.40999042
LOC_Os03g57140	LOC_Os12g18880	9.19169793
LOC_Os03g57290	LOC_Os04g27860	8.82256705
LOC_Os03g57120	LOC_Os07g44450	8.99886731
LOC_Os03g57040	LOC_Os09g27420	9.17977535
LOC_Os03g57120	LOC_Os07g11440	9.46989144
LOC_Os03g56840	LOC_Os06g09910	9.21433048
LOC_Os03g57130	LOC_Os09g30360	9.64552549
LOC_Os03g57040	LOC_Os03g62500	9.15327883
LOC_Os03g57290	LOC_Os04g44890	8.99114735
LOC_Os03g57290	LOC_Os07g34140	8.82365547

InteractorA	InteractorB	Z score
LOC_Os03g56840	LOC_Os04g30800	9.09302227
LOC_Os03g56840	LOC_Os07g03319	9.16290106
LOC_Os03g57130	LOC_Os10g30200	9.33575502
LOC_Os03g57140	LOC_Os08g37800	9.18962669
LOC_Os03g56840	LOC_Os07g03409	9.16290777
LOC_Os03g57130	LOC_Os05g47540	9.28253024
LOC_Os03g56840	LOC_Os11g33270	8.89187527
LOC_Os03g57120	LOC_Os08g16910	8.88123326
LOC_Os03g56810	LOC_Os07g05160	8.85756417
LOC_Os03g56810	LOC_Os08g02410	10.3873394
LOC_Os03g57120	LOC_Os07g41750	8.81960396
LOC_Os03g56840	LOC_Os09g23550	8.90513657
LOC_Os03g57140	LOC_Os06g35660	9.22576427
LOC_Os03g57140	LOC_Os08g42910	9.3967189
LOC_Os03g57290	LOC_Os07g39270	11.1161564
LOC_Os03g57120	LOC_Os03g64050	10.7644769
LOC_Os03g57290	LOC_Os04g55960	9.98561083
LOC_Os03g56840	LOC_Os07g03499	9.16290106
LOC_Os03g57120	LOC_Os03g60620	9.9006642
LOC_Os03g57120	LOC_Os09g32952	9.03094896
LOC_Os03g57040	LOC_Os06g01850	10.1409506
LOC_Os03g57200	LOC_Os10g29620	9.01459518
LOC_Os03g56840	LOC_Os07g31830	8.95677715
LOC_Os03g57120	LOC_Os04g18650	9.31862262
LOC_Os03g57290	LOC_Os05g51480	8.87719343
LOC_Os03g57290	LOC_Os04g32460	9.21025839
LOC_Os03g57290	LOC_Os04g57410	10.2077857
LOC_Os03g57130	LOC_Os08g28820	9.05202351
LOC_Os03g56810	LOC_Os12g12560	10.3311569
LOC_Os03g57140	LOC_Os08g40140	9.50402693
LOC_Os03g57140	LOC_Os06g37080	8.89999797
LOC_Os03g57290	LOC_Os05g47640	9.33819654
LOC_Os03g56810	LOC_Os09g12570	9.32778389
LOC_Os03g57040	LOC_Os07g44550	9.1587664
LOC_Os03g57200	LOC_Os05g50380	9.16488252
LOC_Os03g57200	LOC_Os05g31020	8.95373412
LOC_Os03g56840	LOC_Os09g20090	11.5299745
LOC_Os03g58300	LOC_Os06g36670	9.83363293
LOC_Os03g58290	LOC_Os07g48040	10.4622965
LOC_Os03g58050	LOC_Os08g17784	8.93684646
LOC_Os03g58050	LOC_Os05g38740	10.0431435
LOC_Os03g58260	LOC_Os04g52280	10.2747446
LOC_Os03g58260	LOC_Os05g06300	8.94761284

InteractorA	InteractorB	Z score
LOC_Os03g58130	LOC_Os04g40874	8.81374391
LOC_Os03g57290	LOC_Os11g32260	8.98484773
LOC_Os03g57290	LOC_Os12g25690	9.5035893
LOC_Os03g58260	LOC_Os10g02480	10.1728219
LOC_Os03g58050	LOC_Os12g44350	10.5146433
LOC_Os03g58050	LOC_Os05g42150	9.57386949
LOC_Os03g57290	LOC_Os10g25950	9.97340895
LOC_Os03g58050	LOC_Os04g49420	10.0431544
LOC_Os03g58130	LOC_Os04g02820	9.49111702
LOC_Os03g58260	LOC_Os11g40150	8.95408499
LOC_Os03g58320	LOC_Os04g40874	11.0341394
LOC_Os03g58050	LOC_Os10g11260	8.82091671
LOC_Os03g58260	LOC_Os10g21310	9.30751783
LOC_Os03g58320	LOC_Os07g06440	9.05409645
LOC_Os03g58260	LOC_Os09g34214	8.98978437
LOC_Os03g58050	LOC_Os10g38950	9.50407043
LOC_Os03g58130	LOC_Os05g41210	9.03780029
LOC_Os03g57290	LOC_Os10g36650	9.75643847
LOC_Os03g58050	LOC_Os07g36500	10.0431443
LOC_Os03g58050	LOC_Os10g39410	10.0431544
LOC_Os03g58290	LOC_Os11g03980	8.91747155
LOC_Os03g58300	LOC_Os08g28820	8.98854101
LOC_Os03g58300	LOC_Os09g36830	8.88733236
LOC_Os03g58260	LOC_Os04g12480	9.14163767
LOC_Os03g58050	LOC_Os06g04510	8.81715691
LOC_Os03g58260	LOC_Os06g34690	10.1049508
LOC_Os03g57290	LOC_Os10g39840	8.88868389
LOC_Os03g58050	LOC_Os11g08940	9.15809359
LOC_Os03g58050	LOC_Os09g26340	10.0431475
LOC_Os03g58320	LOC_Os06g15990	9.40193224
LOC_Os03g58260	LOC_Os11g32610	9.39770778
LOC_Os03g58260	LOC_Os06g14510	9.48472971
LOC_Os03g57790	LOC_Os06g14510	10.4345337
LOC_Os03g58050	LOC_Os09g38020	10.0431511
LOC_Os03g58050	LOC_Os10g36650	9.81574263
LOC_Os03g57290	LOC_Os10g42720	9.13545551
LOC_Os03g58260	LOC_Os04g41620	9.32233909
LOC_Os03g58050	LOC_Os05g39050	10.0431399
LOC_Os03g58130	LOC_Os06g06040	8.87467186
LOC_Os03g58050	LOC_Os08g31060	9.0691201
LOC_Os03g58050	LOC_Os06g39230	10.7878917
LOC_Os03g58300	LOC_Os05g41210	9.16888662
LOC_Os03g58320	LOC_Os07g43670	9.23690042

InteractorA	InteractorB	Z score
LOC_Os03g58260	LOC_Os10g01540	9.65607129
LOC_Os03g57290	LOC_Os12g12580	9.34245875
LOC_Os03g58320	LOC_Os04g52100	10.0761289
LOC_Os03g58050	LOC_Os05g25490	9.46744137
LOC_Os03g58130	LOC_Os05g42150	9.00733419
LOC_Os03g58260	LOC_Os05g47640	9.08584501
LOC_Os03g58290	LOC_Os10g32550	9.0009466
LOC_Os03g57290	LOC_Os09g17620	9.45693083
LOC_Os03g58050	LOC_Os07g05400	10.762019
LOC_Os03g57290	LOC_Os10g25930	9.17537218
LOC_Os03g58260	LOC_Os08g34210	8.8109338
LOC_Os03g58050	LOC_Os06g12180	8.88132703
LOC_Os03g57290	LOC_Os11g33240	9.19557538
LOC_Os03g58300	LOC_Os09g27820	10.2700857
LOC_Os03g57790	LOC_Os03g61330	9.57648177
LOC_Os03g59020	LOC_Os11g25100	9.06115747
LOC_Os03g58400	LOC_Os05g47890	9.80580912
LOC_Os03g58980	LOC_Os07g05800	8.8700135
LOC_Os03g58630	LOC_Os04g33570	9.32383419
LOC_Os03g58400	LOC_Os05g27940	9.33513711
LOC_Os03g58530	LOC_Os04g17064	8.89040739
LOC_Os03g58980	LOC_Os04g48850	9.79437508
LOC_Os03g59020	LOC_Os04g27860	8.96464452
LOC_Os03g58630	LOC_Os07g05160	9.9784597
LOC_Os03g58430	LOC_Os09g31490	9.05345516
LOC_Os03g59060	LOC_Os07g42600	8.94193747
LOC_Os03g58530	LOC_Os09g37540	8.99196294
LOC_Os03g59020	LOC_Os08g17500	9.20102185
LOC_Os03g59310	LOC_Os05g12210	9.34378271
LOC_Os03g59020	LOC_Os08g09250	9.76606735
LOC_Os03g58980	LOC_Os09g17740	8.96451857
LOC_Os03g58530	LOC_Os09g09520	9.01714871
LOC_Os03g58980	LOC_Os12g13810	9.41406651
LOC_Os03g58430	LOC_Os08g09770	9.41231859
LOC_Os03g58400	LOC_Os05g33730	9.60573244
LOC_Os03g58530	LOC_Os05g44140	8.92685094
LOC_Os03g58400	LOC_Os11g19320	9.3246442
LOC_Os03g58530	LOC_Os06g39708	10.1530149
LOC_Os03g58530	LOC_Os04g41310	8.91420149
LOC_Os03g59020	LOC_Os09g36450	9.35911019
LOC_Os03g58400	LOC_Os09g36450	9.94672001
LOC_Os03g58530	LOC_Os12g23630	8.88475507
LOC_Os03g58530	LOC_Os05g46360	9.95019855

InteractorA	InteractorB	Z score
LOC_Os03g59310	LOC_Os04g25990	10.1843496
LOC_Os03g58400	LOC_Os05g04520	8.86552855
LOC_Os03g59020	LOC_Os08g03440	9.18952969
LOC_Os03g58430	LOC_Os04g02820	9.46933977
LOC_Os03g59060	LOC_Os09g39500	9.00904623
LOC_Os03g59020	LOC_Os10g42720	9.38103112
LOC_Os03g59060	LOC_Os06g14510	9.01347724
LOC_Os03g59020	LOC_Os12g22030	9.03855568
LOC_Os03g59020	LOC_Os04g22660	9.67651761
LOC_Os03g59020	LOC_Os03g63410	8.87815383
LOC_Os03g58530	LOC_Os12g07050	8.82026698
LOC_Os03g58980	LOC_Os09g20284	10.3455164
LOC_Os03g59310	LOC_Os05g33400	9.82536254
LOC_Os03g58400	LOC_Os07g43170	9.40840167
LOC_Os03g58980	LOC_Os04g56070	9.1725254
LOC_Os03g58430	LOC_Os10g38610	9.47830981
LOC_Os03g58430	LOC_Os06g45100	10.1675021
LOC_Os03g58430	LOC_Os05g12210	10.119303
LOC_Os03g59060	LOC_Os08g44340	8.93990333
LOC_Os03g58630	LOC_Os11g32520	8.80288248
LOC_Os03g58400	LOC_Os05g46040	9.12135389
LOC_Os03g58430	LOC_Os10g37210	9.2158601
LOC_Os03g58530	LOC_Os06g22140	9.06423768
LOC_Os03g59020	LOC_Os09g24530	9.62024075
LOC_Os03g58530	LOC_Os11g08440	8.86615304
LOC_Os03g59060	LOC_Os09g39810	9.5045796
LOC_Os03g58530	LOC_Os07g07470	9.24774548
LOC_Os03g58630	LOC_Os05g50890	9.4884524
LOC_Os03g58530	LOC_Os09g17740	9.84216629
LOC_Os03g58400	LOC_Os12g38770	9.92862278
LOC_Os03g58430	LOC_Os09g39570	9.48032621
LOC_Os03g58400	LOC_Os06g49470	8.87590843
LOC_Os03g59060	LOC_Os10g28350	9.01420521
LOC_Os03g58400	LOC_Os05g45810	8.85180185
LOC_Os03g58400	LOC_Os12g34450	9.33671732
LOC_Os03g58430	LOC_Os09g24924	9.69500475
LOC_Os03g58530	LOC_Os07g08660	9.22699073
LOC_Os03g59020	LOC_Os08g04180	8.94322653
LOC_Os03g58400	LOC_Os04g56320	10.1555011
LOC_Os03g59060	LOC_Os03g61340	10.1059408
LOC_Os03g58630	LOC_Os12g10560	10.501929
LOC_Os03g59020	LOC_Os04g16826	9.92048532
LOC_Os03g59020	LOC_Os07g22600	9.83755149

InteractorA	InteractorB	Z score
LOC_Os03g58430	LOC_Os06g44620	11.3065565
LOC_Os03g58980	LOC_Os07g37550	8.83274991
LOC_Os03g59060	LOC_Os05g30410	9.19467287
LOC_Os03g59060	LOC_Os07g22930	8.86581502
LOC_Os03g59020	LOC_Os05g46580	9.15754546
LOC_Os03g58400	LOC_Os06g15990	8.99395379
LOC_Os03g58980	LOC_Os08g33820	9.11649767
LOC_Os03g58630	LOC_Os07g48430	9.12719898
LOC_Os03g59020	LOC_Os12g38760	8.83502128
LOC_Os03g58530	LOC_Os05g46040	9.89859016
LOC_Os03g58530	LOC_Os04g59040	8.8713831
LOC_Os03g59310	LOC_Os04g27060	9.08663461
LOC_Os03g59310	LOC_Os04g44060	9.04225579
LOC_Os03g58400	LOC_Os12g12514	10.5216744
LOC_Os03g58430	LOC_Os04g57220	8.98481968
LOC_Os03g59060	LOC_Os08g36320	9.81489366
LOC_Os03g58430	LOC_Os05g22724	9.22526856
LOC_Os03g58400	LOC_Os04g59160	9.55673901
LOC_Os03g59060	LOC_Os08g15292	9.3554964
LOC_Os03g58530	LOC_Os07g44790	9.54635236
LOC_Os03g58530	LOC_Os10g32550	8.81616811
LOC_Os03g58400	LOC_Os11g37950	9.39880508
LOC_Os03g59060	LOC_Os10g25140	9.79497662
LOC_Os03g58530	LOC_Os09g35800	8.91428614
LOC_Os03g58530	LOC_Os08g37800	10.1253121
LOC_Os03g59020	LOC_Os10g38950	9.36213213
LOC_Os03g58980	LOC_Os10g27050	9.07234497
LOC_Os03g59020	LOC_Os10g21344	9.92048532
LOC_Os03g58530	LOC_Os09g20220	8.97747805
LOC_Os03g59060	LOC_Os12g10720	9.45957818
LOC_Os03g59060	LOC_Os04g56760	9.19467287
LOC_Os03g58430	LOC_Os03g59660	9.82873999
LOC_Os03g59020	LOC_Os04g55960	9.42038503
LOC_Os03g58530	LOC_Os05g51480	8.99357359
LOC_Os03g58530	LOC_Os10g27050	9.02382645
LOC_Os03g59020	LOC_Os05g49830	8.80800356
LOC_Os03g58630	LOC_Os09g15420	9.42383364
LOC_Os03g58430	LOC_Os12g43630	9.39477912
LOC_Os01g15120	LOC_Os11g02440	9.94953018
LOC_Os01g16030	LOC_Os01g37590	8.94634791
LOC_Os01g15120	LOC_Os09g24412	9.15728999
LOC_Os01g15120	LOC_Os07g48160	9.32623559
LOC_Os01g15120	LOC_Os05g49890	9.1976217

InteractorA	InteractorB	Z score
LOC_Os01g15830	LOC_Os01g53900	8.81868351
LOC_Os01g15830	LOC_Os01g44220	9.23777351
LOC_Os01g15130	LOC_Os06g36670	9.14342174
LOC_Os01g15120	LOC_Os01g59340	9.60829585
LOC_Os01g16030	LOC_Os01g66720	9.18378879
LOC_Os01g16030	LOC_Os02g40830	9.20546987
LOC_Os01g16030	LOC_Os06g42130	10.2183049
LOC_Os01g16030	LOC_Os06g09450	9.69752429
LOC_Os01g15130	LOC_Os04g40950	9.67981477
LOC_Os01g15120	LOC_Os03g29570	9.42344437
LOC_Os01g15120	LOC_Os05g46580	8.90619654
LOC_Os01g15130	LOC_Os01g28500	8.98838172
LOC_Os01g15120	LOC_Os08g15266	9.15728999
LOC_Os01g15120	LOC_Os04g16818	9.15728999
LOC_Os01g15120	LOC_Os07g12730	9.04171429
LOC_Os01g15130	LOC_Os01g54490	8.96673219
LOC_Os01g15830	LOC_Os04g16846	9.06584448
LOC_Os01g16030	LOC_Os01g74000	9.20067145
LOC_Os01g15120	LOC_Os12g43630	9.40468373
LOC_Os01g15830	LOC_Os01g51700	9.03430786
LOC_Os01g15830	LOC_Os07g30970	9.41431966
LOC_Os01g15830	LOC_Os03g52860	9.21413501
LOC_Os01g15130	LOC_Os05g09490	11.0211049
LOC_Os01g15120	LOC_Os03g09810	9.982661
LOC_Os01g15830	LOC_Os12g38180	8.84269335
LOC_Os01g15120	LOC_Os07g42940	11.8578897
LOC_Os01g15120	LOC_Os02g49720	8.8735266
LOC_Os01g16030	LOC_Os06g05250	10.4607306
LOC_Os01g15830	LOC_Os07g05820	9.0798672
LOC_Os01g15830	LOC_Os10g22450	9.42061743
LOC_Os01g15120	LOC_Os02g06700	10.3706983
LOC_Os01g15830	LOC_Os09g12660	9.57567837
LOC_Os01g15130	LOC_Os05g35400	9.09132758
LOC_Os01g15120	LOC_Os11g03230	11.1073367
LOC_Os01g15130	LOC_Os11g03230	9.76876219
LOC_Os01g15120	LOC_Os08g29370	9.89190339
LOC_Os01g15120	LOC_Os09g20260	8.83074429
LOC_Os01g15130	LOC_Os08g31870	9.41295445
LOC_Os01g15120	LOC_Os07g05160	10.5651163
LOC_Os01g15120	LOC_Os04g16772	9.15728999
LOC_Os01g15120	LOC_Os10g32550	9.06325323
LOC_Os01g15120	LOC_Os10g21352	9.15728999
LOC_Os01g15120	LOC_Os05g49800	10.5321571

InteractorA	InteractorB	Z score
LOC_Os01g15120	LOC_Os07g38730	8.99649319
LOC_Os01g16030	LOC_Os04g24430	9.46086482
LOC_Os01g16030	LOC_Os03g38980	10.057163
LOC_Os01g16030	LOC_Os06g11290	9.18820994
LOC_Os01g15830	LOC_Os04g58800	9.42553261
LOC_Os01g15830	LOC_Os02g22780	12.1400073
LOC_Os01g15130	LOC_Os10g28120	10.3742542
LOC_Os01g15130	LOC_Os02g10070	10.8284074
LOC_Os01g16030	LOC_Os04g37820	9.6530439
LOC_Os01g16152	LOC_Os02g05410	9.89993267
LOC_Os01g15130	LOC_Os02g35760	9.51980805
LOC_Os01g15130	LOC_Os01g38500	10.1568108
LOC_Os01g15130	LOC_Os01g27230	8.86498475
LOC_Os01g15130	LOC_Os02g53420	9.60407215
LOC_Os01g15130	LOC_Os03g13170	10.5719971
LOC_Os01g15130	LOC_Os10g27050	11.3332936
LOC_Os01g15120	LOC_Os07g14590	8.8321677
LOC_Os01g15120	LOC_Os06g11240	9.49471716
LOC_Os01g15120	LOC_Os07g25024	9.15728999
LOC_Os01g16030	LOC_Os02g55420	10.1593648
LOC_Os01g15130	LOC_Os09g39500	10.5719971
LOC_Os01g16030	LOC_Os07g16970	10.406752
LOC_Os01g15120	LOC_Os02g41470	9.46653041
LOC_Os01g15830	LOC_Os10g21324	9.06583791
LOC_Os01g15120	LOC_Os07g44430	9.89237433
LOC_Os01g16030	LOC_Os02g39850	9.08405617
LOC_Os01g16030	LOC_Os03g61340	8.97061003
LOC_Os01g15830	LOC_Os01g62230	8.88751354
LOC_Os01g15130	LOC_Os03g01260	9.02557561
LOC_Os01g16030	LOC_Os02g16550	9.52141236
LOC_Os01g16030	LOC_Os01g58610	12.5631329
LOC_Os01g15130	LOC_Os09g33850	8.96753557
LOC_Os01g15830	LOC_Os12g43100	9.00465922
LOC_Os01g15120	LOC_Os03g56280	10.4662053
LOC_Os01g16152	LOC_Os01g19450	9.16586219
LOC_Os01g16152	LOC_Os06g05700	9.43407468
LOC_Os01g15130	LOC_Os10g41410	9.28422062
LOC_Os01g15120	LOC_Os12g42884	9.31978201
LOC_Os01g15130	LOC_Os10g21266	9.73396392
LOC_Os01g15830	LOC_Os01g57942	9.06584992
LOC_Os03g59710	LOC_Os05g07690	9.86369221
LOC_Os03g60740	LOC_Os04g16846	8.82009055
LOC_Os03g60620	LOC_Os04g32650	8.92885743

InteractorA	InteractorB	Z score
LOC_Os03g60620	LOC_Os06g06560	9.13515257
LOC_Os03g60580	LOC_Os07g30200	9.03391984
LOC_Os03g60400	LOC_Os04g39020	8.8480438
LOC_Os03g59710	LOC_Os07g38910	10.4046395
LOC_Os03g59740	LOC_Os09g31486	8.87905571
LOC_Os03g60400	LOC_Os05g47545	9.60969951
LOC_Os03g59700	LOC_Os05g40420	9.42682674
LOC_Os03g59740	LOC_Os12g22030	8.91044066
LOC_Os03g59310	LOC_Os10g30840	9.09133051
LOC_Os03g60620	LOC_Os06g51084	10.2298301
LOC_Os03g60620	LOC_Os06g36160	10.2147008
LOC_Os03g59740	LOC_Os10g23100	9.61212317
LOC_Os03g60580	LOC_Os05g11550	10.3714321
LOC_Os03g59660	LOC_Os08g34210	9.17896235
LOC_Os03g59700	LOC_Os06g11260	9.10190685
LOC_Os03g60620	LOC_Os12g10730	8.9957707
LOC_Os03g59660	LOC_Os07g05400	9.52489578
LOC_Os03g60740	LOC_Os04g40874	9.02211097
LOC_Os03g60620	LOC_Os11g32620	9.2343296
LOC_Os03g60740	LOC_Os03g63090	9.4036792
LOC_Os03g59700	LOC_Os11g05570	9.85281844
LOC_Os03g59310	LOC_Os11g36719	9.14317728
LOC_Os03g60620	LOC_Os07g31770	9.99422325
LOC_Os03g60620	LOC_Os08g39860	9.82431362
LOC_Os03g60400	LOC_Os06g35660	8.81429525
LOC_Os03g59700	LOC_Os09g31486	9.32022629
LOC_Os03g60740	LOC_Os04g48540	8.96866244
LOC_Os03g60580	LOC_Os06g37560	9.26293029
LOC_Os03g60580	LOC_Os08g44370	9.41849722
LOC_Os03g59740	LOC_Os05g20050	9.96132362
LOC_Os03g60620	LOC_Os06g47320	9.57552335
LOC_Os03g59740	LOC_Os07g48060	9.48989495
LOC_Os03g60400	LOC_Os05g06300	10.0319486
LOC_Os03g59700	LOC_Os05g15520	9.18068843
LOC_Os03g59700	LOC_Os11g08440	9.29276311
LOC_Os03g60400	LOC_Os09g15790	9.54212899
LOC_Os03g60620	LOC_Os10g25674	9.51208174
LOC_Os03g60740	LOC_Os04g46930	8.97953333
LOC_Os03g59310	LOC_Os12g24650	9.42802615
LOC_Os03g60620	LOC_Os12g07830	9.23750744
LOC_Os03g59660	LOC_Os10g32550	8.84740574
LOC_Os03g60400	LOC_Os07g06970	8.82360511
LOC_Os03g59660	LOC_Os04g42920	9.20252609

InteractorA	InteractorB	Z score
LOC_Os03g60400	LOC_Os09g23560	9.05104882
LOC_Os03g60580	LOC_Os05g51480	9.14011098
LOC_Os03g60620	LOC_Os09g12570	9.75146199
LOC_Os03g60580	LOC_Os10g35480	9.08413671
LOC_Os03g60400	LOC_Os11g19800	9.19435349
LOC_Os03g59710	LOC_Os05g15520	9.52494174
LOC_Os03g60620	LOC_Os10g38150	9.44086675
LOC_Os03g60620	LOC_Os06g35650	13.1211425
LOC_Os03g60620	LOC_Os05g19150	9.20122892
LOC_Os03g59660	LOC_Os12g22650	9.13879613
LOC_Os03g60400	LOC_Os08g29170	9.63211424
LOC_Os03g59740	LOC_Os07g25150	9.45494351
LOC_Os03g60620	LOC_Os05g50710	10.16081
LOC_Os03g60400	LOC_Os05g08430	8.8892281
LOC_Os03g60620	LOC_Os10g07040	10.1182017
LOC_Os03g60400	LOC_Os04g46930	9.2660932
LOC_Os03g59660	LOC_Os10g38540	9.36814317
LOC_Os03g59710	LOC_Os07g05940	9.79571828
LOC_Os03g60580	LOC_Os11g26860	9.18828629
LOC_Os03g60400	LOC_Os09g39500	9.57800553
LOC_Os03g59660	LOC_Os06g39875	9.06663193
LOC_Os03g59310	LOC_Os08g43170	8.97606819
LOC_Os03g59700	LOC_Os12g42876	8.96078673
LOC_Os03g60620	LOC_Os05g01600	8.99830513
LOC_Os03g60740	LOC_Os04g33970	8.85330268
LOC_Os03g60580	LOC_Os11g14220	9.30005766
LOC_Os03g60740	LOC_Os04g41960	9.08865134
LOC_Os03g59700	LOC_Os10g42720	9.35364472
LOC_Os03g59660	LOC_Os07g38430	8.95906207
LOC_Os03g60620	LOC_Os07g05820	11.6519921
LOC_Os03g60620	LOC_Os06g15990	9.75581386
LOC_Os03g59310	LOC_Os11g06390	10.3185822
LOC_Os03g60620	LOC_Os11g20790	9.07794699
LOC_Os03g59740	LOC_Os08g38900	10.0390934
LOC_Os03g59710	LOC_Os06g12790	9.93654388
LOC_Os03g59710	LOC_Os05g35770	10.4645078
LOC_Os03g59700	LOC_Os04g56210	9.43067006
LOC_Os03g59660	LOC_Os08g28800	9.30039129
LOC_Os03g60580	LOC_Os11g37640	9.57146333
LOC_Os03g61360	LOC_Os04g52280	9.14872106
LOC_Os03g61330	LOC_Os08g28680	9.71600225
LOC_Os03g61330	LOC_Os12g13390	10.0835461
LOC_Os03g61360	LOC_Os08g44210	8.80071774

InteractorA	InteractorB	Z score
LOC_Os03g61340	LOC_Os03g64030	9.51927831
LOC_Os03g61600	LOC_Os05g30480	8.86115213
LOC_Os03g61600	LOC_Os03g63720	9.05383493
LOC_Os03g61330	LOC_Os07g48160	10.6603305
LOC_Os03g61340	LOC_Os04g40130	9.79628232
LOC_Os03g61330	LOC_Os09g15320	9.03753908
LOC_Os03g61280	LOC_Os10g05069	9.00109606
LOC_Os03g61330	LOC_Os05g47980	10.2822537
LOC_Os03g61330	LOC_Os05g28180	8.93099736
LOC_Os03g61600	LOC_Os07g36500	8.88670401
LOC_Os03g61340	LOC_Os12g17540	10.0615494
LOC_Os03g61740	LOC_Os09g31120	9.21858241
LOC_Os03g61600	LOC_Os09g26380	11.2205788
LOC_Os03g61740	LOC_Os08g36900	9.01798747
LOC_Os03g61600	LOC_Os10g39410	8.88669481
LOC_Os03g60740	LOC_Os12g12560	9.16395708
LOC_Os03g61280	LOC_Os10g08580	8.90133293
LOC_Os03g61600	LOC_Os04g49420	8.88669481
LOC_Os03g61330	LOC_Os08g23730	9.53586568
LOC_Os03g61600	LOC_Os12g44010	10.2865814
LOC_Os03g61340	LOC_Os09g39500	10.4363056
LOC_Os03g61330	LOC_Os05g29880	9.31674556
LOC_Os03g61600	LOC_Os07g30200	9.55675722
LOC_Os03g61360	LOC_Os10g21250	9.33380552
LOC_Os03g61330	LOC_Os04g41960	9.66871123
LOC_Os03g61340	LOC_Os06g28550	11.254846
LOC_Os03g61340	LOC_Os04g40290	9.74857624
LOC_Os03g61340	LOC_Os05g04340	9.7010219
LOC_Os03g61740	LOC_Os04g45290	9.22391276
LOC_Os03g60740	LOC_Os09g24990	9.74091948
LOC_Os03g61280	LOC_Os08g15292	9.0371346
LOC_Os03g60740	LOC_Os10g30580	9.52831543
LOC_Os03g61330	LOC_Os11g47580	9.16004062
LOC_Os03g61330	LOC_Os05g51670	9.36808779
LOC_Os03g61330	LOC_Os12g42884	10.5729874
LOC_Os03g61740	LOC_Os09g04050	9.05175999
LOC_Os03g61330	LOC_Os12g34450	8.8176529
LOC_Os03g61600	LOC_Os07g23470	9.13617574
LOC_Os03g60740	LOC_Os11g32520	8.94617363
LOC_Os03g60740	LOC_Os10g21324	8.82008264
LOC_Os03g60870	LOC_Os04g55040	8.9433195
LOC_Os03g61330	LOC_Os08g42910	9.06091819
LOC_Os03g61740	LOC_Os04g57590	9.87780218

InteractorA	InteractorB	Z score
LOC_Os03g60740	LOC_Os06g04030	10.88884
LOC_Os03g61330	LOC_Os10g11810	9.82733724
LOC_Os03g61740	LOC_Os04g41310	9.00590925
LOC_Os03g61330	LOC_Os04g54330	9.38199625
LOC_Os03g61600	LOC_Os05g38740	8.88670401
LOC_Os03g61740	LOC_Os05g02310	8.95361291
LOC_Os03g60870	LOC_Os06g35660	10.5329374
LOC_Os03g60870	LOC_Os08g09940	8.92891237
LOC_Os03g61600	LOC_Os09g26340	8.88669711
LOC_Os03g61600	LOC_Os04g44470	9.15961735
LOC_Os03g61740	LOC_Os06g23870	9.86213968
LOC_Os03g61600	LOC_Os07g42950	9.92410122
LOC_Os03g61330	LOC_Os07g29750	10.0979179
LOC_Os03g61600	LOC_Os05g39050	8.88670401
LOC_Os03g61280	LOC_Os06g23440	9.23587213
LOC_Os03g61340	LOC_Os11g08940	11.0352927
LOC_Os03g61340	LOC_Os04g39020	9.75657539
LOC_Os03g60740	LOC_Os09g33860	8.98800967
LOC_Os03g61340	LOC_Os05g47545	9.36881346
LOC_Os03g61340	LOC_Os05g48290	8.82897976
LOC_Os03g61600	LOC_Os09g38020	8.88669481
LOC_Os03g61330	LOC_Os05g04690	8.86656578
LOC_Os03g61740	LOC_Os05g33140	9.02291262
LOC_Os03g61340	LOC_Os07g06970	8.89927758
LOC_Os03g60870	LOC_Os12g25120	8.8996139
LOC_Os03g61600	LOC_Os06g10970	9.71486135
LOC_Os03g61600	LOC_Os07g48780	9.11555363
LOC_Os03g61280	LOC_Os07g48780	9.12123091
LOC_Os03g61340	LOC_Os10g27050	10.6386688
LOC_Os03g61360	LOC_Os07g44430	9.47470325
LOC_Os03g61600	LOC_Os07g22950	9.40680215
LOC_Os03g61360	LOC_Os11g25260	9.50467923
LOC_Os03g60740	LOC_Os08g34190	9.49170764
LOC_Os03g61600	LOC_Os11g36719	9.09649126
LOC_Os03g61330	LOC_Os06g12790	9.27737619
LOC_Os03g61340	LOC_Os04g45290	9.37388562
LOC_Os03g61360	LOC_Os05g38230	10.1571816
LOC_Os03g61280	LOC_Os12g40830	8.99870423
LOC_Os03g61600	LOC_Os12g02980	9.00792614
LOC_Os03g62060	LOC_Os12g03090	9.99985055
LOC_Os03g63090	LOC_Os06g14620	8.92646288
LOC_Os03g62070	LOC_Os07g25024	10.8089422
LOC_Os03g62060	LOC_Os04g43410	9.81996579

InteractorA	InteractorB	Z score
LOC_Os03g62700	LOC_Os12g40510	11.8459817
LOC_Os03g62500	LOC_Os04g17064	9.25477088
LOC_Os03g61920	LOC_Os10g28050	8.86125657
LOC_Os03g62060	LOC_Os06g01850	8.84016865
LOC_Os03g62070	LOC_Os11g30290	8.82716515
LOC_Os03g63090	LOC_Os03g64050	8.88474716
LOC_Os03g61920	LOC_Os06g01390	9.29345361
LOC_Os03g62060	LOC_Os08g35420	9.12524571
LOC_Os03g61920	LOC_Os09g10260	9.02259245
LOC_Os03g62700	LOC_Os04g48850	8.87975555
LOC_Os03g61970	LOC_Os09g39440	9.36934236
LOC_Os03g61920	LOC_Os07g07320	11.5472454
LOC_Os03g61920	LOC_Os04g43400	8.94759186
LOC_Os03g61970	LOC_Os08g29370	9.27179707
LOC_Os03g61970	LOC_Os03g64210	9.55074644
LOC_Os03g62670	LOC_Os07g49400	8.98417859
LOC_Os03g62700	LOC_Os04g44920	9.39641299
LOC_Os03g61920	LOC_Os09g10270	8.88043391
LOC_Os03g62700	LOC_Os05g35290	10.0036964
LOC_Os03g62500	LOC_Os10g01570	9.14867569
LOC_Os03g62700	LOC_Os04g25990	8.94673381
LOC_Os03g62700	LOC_Os11g41130	10.1499471
LOC_Os03g62700	LOC_Os08g32850	9.8700399
LOC_Os03g62060	LOC_Os05g47545	10.2943982
LOC_Os03g62500	LOC_Os06g21820	10.4169514
LOC_Os03g62500	LOC_Os04g49130	9.50862672
LOC_Os03g62070	LOC_Os06g36770	9.13025564
LOC_Os03g62070	LOC_Os04g27060	9.0457308
LOC_Os03g62500	LOC_Os10g32970	9.8033908
LOC_Os03g62070	LOC_Os12g37360	9.2256438
LOC_Os03g62070	LOC_Os10g21352	10.8089344
LOC_Os03g62070	LOC_Os12g36950	9.419477
LOC_Os03g62070	LOC_Os09g26380	9.72067602
LOC_Os03g61970	LOC_Os08g36910	8.97662048
LOC_Os03g63090	LOC_Os04g33570	8.93805683
LOC_Os03g62500	LOC_Os10g38234	9.37582701
LOC_Os03g63090	LOC_Os06g35660	8.93258015
LOC_Os03g62700	LOC_Os12g44000	9.22964627
LOC_Os03g61920	LOC_Os07g44370	9.13277986
LOC_Os03g62500	LOC_Os07g09340	8.99757301
LOC_Os03g62500	LOC_Os08g04540	9.20626511
LOC_Os03g62500	LOC_Os04g10000	9.00423628
LOC_Os03g62070	LOC_Os05g51480	10.0256669

InteractorA	InteractorB	Z score
LOC_Os03g63090	LOC_Os06g36160	9.49448208
LOC_Os03g62070	LOC_Os10g10434	8.86580217
LOC_Os03g61920	LOC_Os04g56160	9.51720626
LOC_Os03g62070	LOC_Os08g34280	9.02567695
LOC_Os03g62670	LOC_Os08g41830	8.85706678
LOC_Os03g62070	LOC_Os04g16772	10.8089344
LOC_Os03g62070	LOC_Os07g05150	9.39992622
LOC_Os03g61740	LOC_Os12g16410	9.39796282
LOC_Os03g62700	LOC_Os08g09770	8.9462694
LOC_Os03g62070	LOC_Os06g48160	9.390904
LOC_Os03g62070	LOC_Os04g31070	9.06006307
LOC_Os03g62060	LOC_Os10g25140	10.5341789
LOC_Os03g62060	LOC_Os11g47760	9.55373577
LOC_Os03g62060	LOC_Os06g37180	9.17529952
LOC_Os03g61970	LOC_Os09g34250	9.47880667
LOC_Os03g62500	LOC_Os12g38180	9.16547314
LOC_Os03g62070	LOC_Os06g35650	9.27752261
LOC_Os03g61920	LOC_Os07g08500	8.84886417
LOC_Os03g62060	LOC_Os04g10400	9.31756745
LOC_Os03g62060	LOC_Os10g33900	9.44325107
LOC_Os03g62670	LOC_Os04g37820	10.6917702
LOC_Os03g62060	LOC_Os08g34190	10.4953428
LOC_Os03g63090	LOC_Os05g25850	9.67842619
LOC_Os03g62700	LOC_Os10g41480	9.12211907
LOC_Os03g62700	LOC_Os05g48040	10.6016056
LOC_Os03g61740	LOC_Os11g24560	9.01176762
LOC_Os03g61970	LOC_Os07g05400	8.93273047
LOC_Os03g61920	LOC_Os04g39020	9.30640884
LOC_Os03g61970	LOC_Os04g53290	8.89783141
LOC_Os03g63090	LOC_Os05g11730	10.4146781
LOC_Os03g61920	LOC_Os06g06090	10.0775524
LOC_Os03g62070	LOC_Os09g24412	10.8089422
LOC_Os03g62500	LOC_Os12g10570	8.84620385
LOC_Os03g62700	LOC_Os11g14220	10.1300641
LOC_Os03g62500	LOC_Os11g10520	9.04168683
LOC_Os03g61970	LOC_Os09g28400	9.2534365
LOC_Os03g61970	LOC_Os07g38730	9.02361103
LOC_Os03g62500	LOC_Os10g07616	9.49650435
LOC_Os03g61970	LOC_Os11g08470	9.21890371
LOC_Os03g62670	LOC_Os04g09670	9.05518287
LOC_Os03g62060	LOC_Os08g15292	8.93745838
LOC_Os03g62670	LOC_Os08g40170	9.78289336
LOC_Os03g62670	LOC_Os07g46310	9.01631983

InteractorA	InteractorB	Z score
LOC_Os03g61740	LOC_Os12g07050	8.85746928
LOC_Os03g62070	LOC_Os08g15266	10.8089422
LOC_Os03g62700	LOC_Os09g39380	9.29298614
LOC_Os03g62060	LOC_Os07g46630	8.83917326
LOC_Os03g63090	LOC_Os04g42920	9.21063757
LOC_Os03g62700	LOC_Os05g45810	9.2979663
LOC_Os03g62070	LOC_Os11g03290	9.81768434
LOC_Os03g61970	LOC_Os09g09270	8.89212656
LOC_Os03g62700	LOC_Os08g04540	8.90315944
LOC_Os03g62670	LOC_Os04g39020	9.43148718
LOC_Os03g61970	LOC_Os05g12190	9.15441733
LOC_Os03g61970	LOC_Os12g17910	9.64878281
LOC_Os03g62670	LOC_Os09g31502	10.7023448
LOC_Os03g62060	LOC_Os05g49880	8.89341944
LOC_Os03g62500	LOC_Os06g40640	9.30441035
LOC_Os03g62070	LOC_Os09g30360	9.37876537
LOC_Os03g62700	LOC_Os08g34170	9.77167004
LOC_Os03g62070	LOC_Os04g40990	8.91840098
LOC_Os03g62070	LOC_Os06g04280	10.0917581
LOC_Os03g62070	LOC_Os04g56400	9.43731184
LOC_Os03g62070	LOC_Os07g07470	10.2631038
LOC_Os03g62500	LOC_Os06g21980	9.98180995
LOC_Os03g62700	LOC_Os06g28550	9.06844529
LOC_Os03g62060	LOC_Os07g16970	9.09988945
LOC_Os03g62060	LOC_Os11g03400	9.99985816
LOC_Os03g61970	LOC_Os06g10970	9.45273748
LOC_Os03g62070	LOC_Os06g35590	9.11900465
LOC_Os03g62700	LOC_Os10g40600	9.55024824
LOC_Os03g62700	LOC_Os04g33480	9.520807
LOC_Os03g61920	LOC_Os12g38770	9.7577794
LOC_Os03g62070	LOC_Os05g50710	10.3906277
LOC_Os03g61970	LOC_Os10g25950	8.8734736
LOC_Os03g62060	LOC_Os07g43670	9.28456642
LOC_Os03g61970	LOC_Os08g33370	8.95453795
LOC_Os03g62070	LOC_Os05g12240	9.20226935
LOC_Os03g62500	LOC_Os09g10260	8.84697975
LOC_Os03g62060	LOC_Os04g16770	9.12524571
LOC_Os03g62700	LOC_Os04g41960	9.30739641
LOC_Os03g62700	LOC_Os04g30800	9.16038741
LOC_Os03g61920	LOC_Os06g19960	10.7679746
LOC_Os03g62060	LOC_Os04g53800	10.6917782
LOC_Os03g61740	LOC_Os11g32770	8.93728772
LOC_Os03g61920	LOC_Os08g14760	8.88441073

InteractorA	InteractorB	Z score
LOC_Os03g62070	LOC_Os04g16818	10.8089346
LOC_Os03g61970	LOC_Os05g38530	9.82774982
LOC_Os03g62500	LOC_Os04g40130	9.13714042
LOC_Os03g61970	LOC_Os08g35440	9.80349825
LOC_Os03g63090	LOC_Os04g40130	10.9704287
LOC_Os03g62060	LOC_Os09g20090	9.77193123
LOC_Os03g62670	LOC_Os05g51480	9.97877469
LOC_Os03g62670	LOC_Os04g36800	9.41277123
LOC_Os03g64050	LOC_Os07g03730	9.06928491
LOC_Os03g64050	LOC_Os09g28420	9.4362149
LOC_Os03g64210	LOC_Os04g50880	8.80088787
LOC_Os03g63090	LOC_Os12g43490	9.54601354
LOC_Os03g64210	LOC_Os04g16818	9.77381439
LOC_Os03g64210	LOC_Os07g03499	9.48111237
LOC_Os03g63330	LOC_Os10g08670	10.5875776
LOC_Os03g64210	LOC_Os07g03319	9.48111772
LOC_Os03g64030	LOC_Os04g58800	8.82451278
LOC_Os03g64210	LOC_Os04g38870	10.5663675
LOC_Os03g64210	LOC_Os11g05470	10.1929519
LOC_Os03g64050	LOC_Os11g40140	8.94190722
LOC_Os03g63330	LOC_Os09g31502	8.80658887
LOC_Os03g63330	LOC_Os11g40140	10.1204521
LOC_Os03g64210	LOC_Os06g45120	9.40242723
LOC_Os03g63720	LOC_Os06g22140	9.17207474
LOC_Os03g64210	LOC_Os08g15266	9.77381439
LOC_Os03g63090	LOC_Os10g11140	9.30925026
LOC_Os04g01250	LOC_Os04g16770	8.92362593
LOC_Os03g63410	LOC_Os10g38950	9.27382321
LOC_Os03g63330	LOC_Os10g39120	10.1495504
LOC_Os03g64030	LOC_Os05g38230	9.07230926
LOC_Os03g64330	LOC_Os07g22950	9.08008416
LOC_Os03g64030	LOC_Os12g02370	9.77453459
LOC_Os03g64210	LOC_Os10g08620	8.94544673
LOC_Os03g63410	LOC_Os04g35200	9.40961429
LOC_Os03g63410	LOC_Os05g05830	9.6844083
LOC_Os03g64210	LOC_Os12g07050	10.5086495
LOC_Os03g64210	LOC_Os07g09340	9.44023937
LOC_Os03g63720	LOC_Os04g59200	10.5697344
LOC_Os03g64030	LOC_Os10g31950	11.5010418
LOC_Os03g63330	LOC_Os04g57590	9.14308957
LOC_Os03g63720	LOC_Os06g04200	10.0649875
LOC_Os03g63410	LOC_Os11g29400	9.0956319
LOC_Os03g63330	LOC_Os07g35940	8.90989551

InteractorA	InteractorB	Z score
LOC_Os03g64210	LOC_Os09g24412	9.77381439
LOC_Os03g63410	LOC_Os07g05800	9.17495285
LOC_Os03g63720	LOC_Os12g22680	8.83095335
LOC_Os03g63720	LOC_Os06g44620	9.90999201
LOC_Os03g63410	LOC_Os09g32840	9.7971408
LOC_Os03g64210	LOC_Os04g16772	9.77379606
LOC_Os03g64210	LOC_Os05g12240	9.26126281
LOC_Os03g64210	LOC_Os10g21352	9.77379606
LOC_Os03g63410	LOC_Os11g25260	9.23513749
LOC_Os03g63410	LOC_Os04g27980	9.74146523
LOC_Os03g63330	LOC_Os09g23550	9.37844294
LOC_Os03g63410	LOC_Os12g12560	9.41198268
LOC_Os03g63720	LOC_Os09g33500	9.36971022
LOC_Os03g63720	LOC_Os05g06450	9.13055708
LOC_Os03g63330	LOC_Os08g40140	9.00659869
LOC_Os03g63720	LOC_Os04g44730	9.09613916
LOC_Os03g63330	LOC_Os04g53290	9.11457673
LOC_Os03g63330	LOC_Os09g10260	8.91625775
LOC_Os03g63090	LOC_Os12g10600	9.92815602
LOC_Os03g64050	LOC_Os12g22650	8.81207129
LOC_Os03g63720	LOC_Os04g44924	8.94600594
LOC_Os03g63720	LOC_Os06g36700	9.86706377
LOC_Os03g64030	LOC_Os04g04230	9.74381893
LOC_Os03g63720	LOC_Os06g51084	8.93380269
LOC_Os03g64030	LOC_Os12g23170	9.87452092
LOC_Os03g63090	LOC_Os07g22600	8.97353856
LOC_Os03g64330	LOC_Os04g10000	9.02247599
LOC_Os03g64210	LOC_Os11g18870	9.19558379
LOC_Os03g64210	LOC_Os04g39210	9.17215425
LOC_Os03g64210	LOC_Os08g32620	9.68120086
LOC_Os03g64210	LOC_Os12g13320	9.00698738
LOC_Os03g64210	LOC_Os11g47580	9.1149601
LOC_Os03g64030	LOC_Os04g29550	9.69102573
LOC_Os03g63330	LOC_Os07g20544	11.1095675
LOC_Os03g64210	LOC_Os07g03409	9.48111772
LOC_Os03g63090	LOC_Os08g09770	10.5893175
LOC_Os03g64330	LOC_Os04g45490	9.57694033
LOC_Os03g63720	LOC_Os05g39580	9.45904691
LOC_Os03g63410	LOC_Os04g33040	9.61530446
LOC_Os03g63410	LOC_Os07g30170	8.91818006
LOC_Os03g63410	LOC_Os08g15040	9.48544482
LOC_Os03g63410	LOC_Os06g02390	9.33756331
LOC_Os03g63410	LOC_Os05g38560	11.2131326

InteractorA	InteractorB	Z score
LOC_Os03g64030	LOC_Os12g06620	9.00038213
LOC_Os03g63410	LOC_Os09g15790	10.4985846
LOC_Os03g63410	LOC_Os07g44440	9.14395213
LOC_Os03g63410	LOC_Os10g38340	8.84217308
LOC_Os03g64030	LOC_Os04g42920	9.03910527
LOC_Os03g64210	LOC_Os09g12570	8.95244908
LOC_Os03g63410	LOC_Os11g14040	9.25904677
LOC_Os03g63090	LOC_Os07g43670	9.05064048
LOC_Os03g63330	LOC_Os11g47760	13.1144812
LOC_Os03g64330	LOC_Os10g25140	8.80109071
LOC_Os03g64330	LOC_Os07g11440	9.19483276
LOC_Os03g63410	LOC_Os05g35330	8.80277663
LOC_Os03g63330	LOC_Os04g25990	9.18625461
LOC_Os03g64210	LOC_Os07g25024	9.77381439
LOC_Os03g63330	LOC_Os08g40740	8.88301206
LOC_Os03g64050	LOC_Os07g41750	9.39550423
LOC_Os03g64330	LOC_Os09g30412	11.1921895
LOC_Os03g63090	LOC_Os10g22070	8.85573528
LOC_Os03g63410	LOC_Os10g29470	11.9111172
LOC_Os03g64050	LOC_Os10g39170	9.2800604
LOC_Os03g63720	LOC_Os07g34260	9.03020057
LOC_Os03g63330	LOC_Os06g06300	9.31663671
LOC_Os03g64030	LOC_Os09g36930	9.55061185
LOC_Os03g63330	LOC_Os09g15790	9.70922283
LOC_Os03g63090	LOC_Os06g48160	9.50162404
LOC_Os03g63090	LOC_Os08g38920	9.18540943
LOC_Os03g64210	LOC_Os05g01810	9.36247073
LOC_Os03g63330	LOC_Os08g02700	9.76099235
LOC_Os03g63410	LOC_Os07g35940	10.5630697
LOC_Os03g64210	LOC_Os07g41750	8.85855868
LOC_Os03g64210	LOC_Os07g48020	9.19025255
LOC_Os03g64330	LOC_Os07g34140	8.92879198
LOC_Os03g63410	LOC_Os04g12690	8.93606017
LOC_Os03g63330	LOC_Os06g35650	9.19734108
LOC_Os03g64210	LOC_Os11g08440	8.89414749
LOC_Os03g63090	LOC_Os06g46340	8.99522869
LOC_Os03g64210	LOC_Os04g41620	9.2488845
LOC_Os03g63090	LOC_Os07g05820	10.4331214
LOC_Os03g64210	LOC_Os10g40090	9.21939626
LOC_Os03g63410	LOC_Os09g10260	9.38010993
LOC_Os03g63330	LOC_Os11g08440	9.01959927
LOC_Os03g64210	LOC_Os04g40130	9.630145
LOC_Os03g63720	LOC_Os10g28080	8.82432437

InteractorA	InteractorB	Z score
LOC_Os03g63410	LOC_Os10g21268	8.80277962
LOC_Os03g63410	LOC_Os04g32650	9.36857705
LOC_Os03g63090	LOC_Os10g26600	9.06041069
LOC_Os03g64210	LOC_Os12g34062	9.53556746
LOC_Os03g64210	LOC_Os04g37990	9.32535539
LOC_Os03g63410	LOC_Os12g01922	9.10775399
LOC_Os03g64210	LOC_Os10g38160	10.6208072
LOC_Os03g63410	LOC_Os06g36880	9.03662437
LOC_Os03g63410	LOC_Os04g58710	9.43027326
LOC_Os03g64210	LOC_Os04g12960	9.03406635
LOC_Os03g64210	LOC_Os06g37500	10.3628481
LOC_Os03g63090	LOC_Os08g09370	8.9452341
LOC_Os03g64210	LOC_Os04g39900	8.98156471
LOC_Os03g64030	LOC_Os10g41490	8.84438253
LOC_Os03g64330	LOC_Os05g49880	9.06166701
LOC_Os03g64210	LOC_Os11g26860	8.80831917
LOC_Os03g64210	LOC_Os07g03730	9.79563842
LOC_Os04g02820	LOC_Os05g45590	12.8956996
LOC_Os04g01980	LOC_Os04g52280	9.12798591
LOC_Os04g01980	LOC_Os12g16250	8.96507027
LOC_Os04g01674	LOC_Os09g27750	9.31712827
LOC_Os04g01740	LOC_Os06g51150	9.57211058
LOC_Os04g02050	LOC_Os08g02410	8.96044813
LOC_Os04g01740	LOC_Os06g15990	8.85221166
LOC_Os04g01980	LOC_Os08g36910	9.72843442
LOC_Os04g01470	LOC_Os10g08022	8.90963017
LOC_Os04g02820	LOC_Os04g16874	9.21478283
LOC_Os04g01600	LOC_Os12g13800	10.7915666
LOC_Os04g01470	LOC_Os05g45810	9.2500043
LOC_Os04g02820	LOC_Os05g09500	10.1173338
LOC_Os04g02050	LOC_Os06g10340	9.62626068
LOC_Os04g02820	LOC_Os07g08500	9.06448229
LOC_Os04g02820	LOC_Os04g43400	12.8101233
LOC_Os04g01250	LOC_Os07g01760	9.22026499
LOC_Os04g02820	LOC_Os07g10720	9.68654947
LOC_Os04g01600	LOC_Os06g10910	8.96769509
LOC_Os04g01600	LOC_Os12g43100	9.18470595
LOC_Os04g01470	LOC_Os08g44350	9.02175044
LOC_Os04g01600	LOC_Os12g31370	10.8415808
LOC_Os04g01674	LOC_Os07g05150	9.24017548
LOC_Os04g01470	LOC_Os10g38740	8.96961413
LOC_Os04g01470	LOC_Os08g28800	9.66782714
LOC_Os04g01470	LOC_Os12g41110	9.0493657

InteractorA	InteractorB	Z score
LOC_Os04g01980	LOC_Os11g26860	9.7860571
LOC_Os04g01600	LOC_Os07g08170	9.28900011
LOC_Os04g02050	LOC_Os07g44440	9.01734914
LOC_Os04g01740	LOC_Os12g17540	8.89167891
LOC_Os04g01600	LOC_Os09g08072	9.10793583
LOC_Os04g01980	LOC_Os06g06100	8.91057049
LOC_Os04g01740	LOC_Os07g38430	9.22821832
LOC_Os04g01740	LOC_Os04g10010	9.38250994
LOC_Os04g01600	LOC_Os10g39840	9.41792187
LOC_Os04g01980	LOC_Os10g38950	8.922158
LOC_Os04g01980	LOC_Os11g20790	8.8849802
LOC_Os04g01674	LOC_Os06g29220	8.93893871
LOC_Os04g01470	LOC_Os12g07980	10.0862825
LOC_Os04g01674	LOC_Os12g44030	8.85741587
LOC_Os04g02050	LOC_Os04g39440	8.92531852
LOC_Os04g01674	LOC_Os04g46930	9.01869137
LOC_Os04g02820	LOC_Os04g53810	8.81423521
LOC_Os04g01470	LOC_Os12g25120	9.90450691
LOC_Os04g01674	LOC_Os05g04690	9.16326955
LOC_Os04g02820	LOC_Os04g40874	9.17136758
LOC_Os04g01600	LOC_Os10g29620	8.82159106
LOC_Os04g01980	LOC_Os10g23900	8.82453476
LOC_Os04g01674	LOC_Os04g40290	8.82828403
LOC_Os04g02050	LOC_Os05g09500	9.28398515
LOC_Os04g01600	LOC_Os09g37100	9.20734651
LOC_Os04g02050	LOC_Os05g31040	9.70876595
LOC_Os04g01250	LOC_Os05g06300	8.8286068
LOC_Os04g01674	LOC_Os09g10260	9.91957849
LOC_Os04g02050	LOC_Os09g30418	10.7445905
LOC_Os04g01470	LOC_Os07g22950	11.2040796
LOC_Os04g02050	LOC_Os07g37550	10.4426216
LOC_Os04g01980	LOC_Os05g46580	9.26529747
LOC_Os04g01600	LOC_Os07g03499	9.46047869
LOC_Os04g01740	LOC_Os10g25140	9.6350908
LOC_Os04g02820	LOC_Os07g10660	9.47487256
LOC_Os04g01470	LOC_Os12g13810	12.3919043
LOC_Os04g01470	LOC_Os07g28480	8.97185228
LOC_Os04g01674	LOC_Os11g14040	8.91260089
LOC_Os04g02050	LOC_Os08g44530	9.20588973
LOC_Os04g01470	LOC_Os08g05910	8.99710834
LOC_Os04g01740	LOC_Os10g02040	9.79756316
LOC_Os04g02820	LOC_Os08g02400	9.45719955
LOC_Os04g01470	LOC_Os12g34450	9.04369268

InteractorA	InteractorB	Z score
LOC_Os04g01250	LOC_Os04g56070	9.01253951
LOC_Os04g01674	LOC_Os09g15420	11.6271964
LOC_Os04g02050	LOC_Os06g47600	11.0517337
LOC_Os04g01250	LOC_Os08g35420	8.92362593
LOC_Os04g01674	LOC_Os08g14570	9.066751
LOC_Os04g01980	LOC_Os09g17740	8.98539979
LOC_Os04g01674	LOC_Os05g02310	8.84944428
LOC_Os04g01470	LOC_Os04g55850	9.72930513
LOC_Os04g01740	LOC_Os12g10570	9.2204264
LOC_Os04g01470	LOC_Os07g48780	9.71790436
LOC_Os04g01600	LOC_Os07g03409	9.46047869
LOC_Os04g02050	LOC_Os06g50300	9.14821163
LOC_Os04g02820	LOC_Os08g44340	8.86880127
LOC_Os04g01250	LOC_Os09g37949	8.99627491
LOC_Os04g01740	LOC_Os10g32550	9.65410486
LOC_Os04g01470	LOC_Os11g26850	11.0390844
LOC_Os04g01740	LOC_Os09g08880	9.03962882
LOC_Os04g02050	LOC_Os11g47550	9.34520567
LOC_Os04g02050	LOC_Os09g26880	8.9139324
LOC_Os04g02050	LOC_Os12g13380	11.1190952
LOC_Os04g02820	LOC_Os06g04510	9.7144132
LOC_Os04g01980	LOC_Os04g52130	8.95274887
LOC_Os04g01250	LOC_Os11g38959	8.85991044
LOC_Os04g01740	LOC_Os09g07460	8.97481245
LOC_Os04g01470	LOC_Os08g06100	12.5999419
LOC_Os04g02820	LOC_Os04g57590	10.2965884
LOC_Os04g02820	LOC_Os06g04270	9.14057224
LOC_Os04g01600	LOC_Os09g30412	9.45873364
LOC_Os04g01600	LOC_Os07g05150	9.33657304
LOC_Os04g01600	LOC_Os04g01674	9.75455251
LOC_Os04g01600	LOC_Os04g10000	8.82247571
LOC_Os04g01980	LOC_Os09g31120	9.45834213
LOC_Os04g02820	LOC_Os06g49970	9.52901472
LOC_Os04g01740	LOC_Os06g27770	9.97191722
LOC_Os04g01980	LOC_Os06g15990	8.9349667
LOC_Os04g01980	LOC_Os04g55960	8.90681257
LOC_Os04g01674	LOC_Os06g07080	11.311829
LOC_Os04g01470	LOC_Os04g09604	19.1801781
LOC_Os04g02050	LOC_Os04g29030	9.70628264
LOC_Os04g01470	LOC_Os05g50380	9.84965667
LOC_Os04g01250	LOC_Os12g03816	10.9581918
LOC_Os04g01980	LOC_Os10g21212	8.88678237
LOC_Os04g01740	LOC_Os07g31770	8.96322548

InteractorA	InteractorB	Z score
LOC_Os04g01600	LOC_Os06g39875	9.41168614
LOC_Os04g01600	LOC_Os07g03710	8.98613709
LOC_Os04g01740	LOC_Os04g57090	9.50312246
LOC_Os04g01600	LOC_Os07g03319	9.46047869
LOC_Os04g01740	LOC_Os05g29880	9.12442126
LOC_Os04g01674	LOC_Os08g28820	8.99931087
LOC_Os04g02050	LOC_Os08g42910	9.00946017
LOC_Os04g02820	LOC_Os07g47490	9.10481528
LOC_Os04g01470	LOC_Os10g38470	9.15267097
LOC_Os04g02820	LOC_Os10g34520	9.66517606
LOC_Os04g01600	LOC_Os10g38360	8.85381973
LOC_Os04g02050	LOC_Os06g39740	9.47023866
LOC_Os04g10010	LOC_Os05g49770	9.66333319
LOC_Os04g09604	LOC_Os04g32650	9.49142517
LOC_Os04g09604	LOC_Os07g05940	9.03801081
LOC_Os04g09604	LOC_Os05g09490	9.77426758
LOC_Os04g10010	LOC_Os06g19960	8.963572
LOC_Os04g09604	LOC_Os08g06100	11.1902452
LOC_Os04g10000	LOC_Os07g30170	9.61252951
LOC_Os04g09604	LOC_Os09g10200	9.2786994
LOC_Os04g09670	LOC_Os06g36160	9.19413892
LOC_Os04g10010	LOC_Os07g02340	11.6008119
LOC_Os04g05080	LOC_Os05g04500	8.84407529
LOC_Os04g10000	LOC_Os08g09210	8.80052969
LOC_Os04g08350	LOC_Os05g08100	9.12636466
LOC_Os04g09604	LOC_Os07g07719	11.0470266
LOC_Os04g08350	LOC_Os04g28180	8.80753161
LOC_Os04g08350	LOC_Os07g03730	9.87763839
LOC_Os04g10000	LOC_Os08g20270	8.95056049
LOC_Os04g09540	LOC_Os09g27750	9.90855165
LOC_Os04g02820	LOC_Os12g18900	9.2976121
LOC_Os04g09604	LOC_Os04g10010	9.30884866
LOC_Os04g10000	LOC_Os06g49970	9.34799464
LOC_Os04g08350	LOC_Os04g42250	9.54070899
LOC_Os04g05080	LOC_Os04g18650	9.16161416
LOC_Os04g10000	LOC_Os09g27420	10.9477803
LOC_Os04g09604	LOC_Os04g28870	10.2513816
LOC_Os04g04230	LOC_Os05g48510	9.24678168
LOC_Os04g10010	LOC_Os05g29880	10.2540331
LOC_Os04g09604	LOC_Os05g45810	10.0375684
LOC_Os04g08350	LOC_Os06g07080	10.0284565
LOC_Os04g09604	LOC_Os06g06560	9.21869981
LOC_Os04g09670	LOC_Os07g44440	9.0667491

InteractorA	InteractorB	Z score
LOC_Os04g08350	LOC_Os04g37950	9.80987702
LOC_Os04g04230	LOC_Os10g22450	9.10543706
LOC_Os04g10010	LOC_Os04g45290	11.6226721
LOC_Os04g10000	LOC_Os08g28820	8.89342418
LOC_Os04g05080	LOC_Os04g26870	8.95710932
LOC_Os04g09604	LOC_Os11g04880	9.25972284
LOC_Os04g08350	LOC_Os07g48780	9.42201064
LOC_Os04g05080	LOC_Os10g38470	9.40837273
LOC_Os04g09670	LOC_Os05g38560	9.19353048
LOC_Os04g09604	LOC_Os06g36840	8.86789684
LOC_Os04g09540	LOC_Os09g29200	9.82015855
LOC_Os04g05080	LOC_Os10g11140	8.97778926
LOC_Os04g09540	LOC_Os10g25140	9.55233054
LOC_Os04g05080	LOC_Os07g05400	8.91916086
LOC_Os04g08350	LOC_Os04g59450	9.53252292
LOC_Os04g02820	LOC_Os12g25690	10.1554879
LOC_Os04g09604	LOC_Os06g25010	9.32452051
LOC_Os04g09670	LOC_Os11g31620	9.08782046
LOC_Os04g05080	LOC_Os05g28280	8.82188279
LOC_Os04g10000	LOC_Os05g35400	8.99744615
LOC_Os04g10000	LOC_Os08g04560	9.69065636
LOC_Os04g09604	LOC_Os10g38160	10.8356771
LOC_Os04g09540	LOC_Os04g45290	9.4721719
LOC_Os04g09670	LOC_Os04g55040	8.8132283
LOC_Os04g09540	LOC_Os04g54390	9.61984653
LOC_Os04g02820	LOC_Os11g40140	9.59282939
LOC_Os04g05080	LOC_Os06g23780	9.10950336
LOC_Os04g09670	LOC_Os05g26890	9.42646571
LOC_Os04g09670	LOC_Os07g26540	8.95953781
LOC_Os04g05080	LOC_Os08g34170	9.2269902
LOC_Os04g09604	LOC_Os12g41110	9.1103325
LOC_Os04g09670	LOC_Os07g07550	9.34383893
LOC_Os04g09604	LOC_Os12g13810	12.2392115
LOC_Os04g05080	LOC_Os11g31530	9.98397639
LOC_Os04g10010	LOC_Os04g33470	9.52885279
LOC_Os04g09604	LOC_Os12g37360	9.24244249
LOC_Os04g09540	LOC_Os07g23730	10.8479911
LOC_Os04g08350	LOC_Os06g11200	10.3905627
LOC_Os04g08350	LOC_Os04g48850	9.96359202
LOC_Os04g09604	LOC_Os05g05800	8.83142929
LOC_Os04g09604	LOC_Os09g23540	9.51520963
LOC_Os04g10000	LOC_Os12g17540	9.2201647
LOC_Os04g10010	LOC_Os04g47360	8.9865213

InteractorA	InteractorB	Z score
LOC_Os04g05080	LOC_Os04g44470	10.3641228
LOC_Os04g09540	LOC_Os04g41310	8.87860763
LOC_Os04g10000	LOC_Os11g26910	8.94726906
LOC_Os04g04230	LOC_Os05g05670	10.086243
LOC_Os04g12710	LOC_Os04g48540	9.23510629
LOC_Os04g12900	LOC_Os07g28480	9.11568082
LOC_Os04g12720	LOC_Os08g28820	9.93046095
LOC_Os04g12900	LOC_Os04g36800	9.23708342
LOC_Os04g10010	LOC_Os10g29470	9.57377233
LOC_Os04g12690	LOC_Os11g07020	8.85015781
LOC_Os04g12720	LOC_Os10g21324	11.2828407
LOC_Os04g12900	LOC_Os11g01872	9.2748406
LOC_Os04g10400	LOC_Os05g30480	9.55705238
LOC_Os04g10010	LOC_Os11g05880	9.0204865
LOC_Os04g12960	LOC_Os06g48180	12.780675
LOC_Os04g12710	LOC_Os09g32840	10.3165838
LOC_Os04g12900	LOC_Os04g52440	9.04538044
LOC_Os04g12480	LOC_Os06g45100	8.91877124
LOC_Os04g10400	LOC_Os12g22030	9.77438414
LOC_Os04g10400	LOC_Os07g11440	9.56201173
LOC_Os04g12720	LOC_Os12g08280	9.15913766
LOC_Os04g10400	LOC_Os09g39380	9.07228764
LOC_Os04g12720	LOC_Os04g16846	11.2828407
LOC_Os04g12480	LOC_Os05g44340	8.80376102
LOC_Os04g12720	LOC_Os05g47545	9.37524372
LOC_Os04g12900	LOC_Os04g17650	8.86553486
LOC_Os04g12900	LOC_Os10g21266	9.0891156
LOC_Os04g12900	LOC_Os06g10950	8.9769519
LOC_Os04g10400	LOC_Os09g27750	8.88431973
LOC_Os04g12720	LOC_Os04g52450	9.01121889
LOC_Os04g12950	LOC_Os06g04620	8.9725679
LOC_Os04g12900	LOC_Os06g04270	8.92001475
LOC_Os04g12720	LOC_Os08g37790	9.27129108
LOC_Os04g12480	LOC_Os09g39400	9.58480039
LOC_Os04g10400	LOC_Os07g10720	12.1859362
LOC_Os04g12900	LOC_Os12g07820	9.33257766
LOC_Os04g12720	LOC_Os12g31370	9.69518545
LOC_Os04g10010	LOC_Os10g31940	9.73174852
LOC_Os04g10400	LOC_Os07g32800	9.56439617
LOC_Os04g12960	LOC_Os05g44140	10.6327813
LOC_Os04g10400	LOC_Os05g35400	9.40799621
LOC_Os04g12710	LOC_Os08g29170	9.92801611
LOC_Os04g12960	LOC_Os05g12190	10.0890175

InteractorA	InteractorB	Z score
LOC_Os04g12900	LOC_Os08g06100	9.37969302
LOC_Os04g12900	LOC_Os04g44470	8.96156696
LOC_Os04g12690	LOC_Os04g48850	8.87428633
LOC_Os04g12950	LOC_Os12g12514	9.66275177
LOC_Os04g12720	LOC_Os08g33710	9.02795025
LOC_Os04g12950	LOC_Os11g47560	8.953457
LOC_Os04g12950	LOC_Os11g34450	9.95730319
LOC_Os04g12690	LOC_Os05g39960	8.83081608
LOC_Os04g10400	LOC_Os09g28460	9.69322342
LOC_Os04g12720	LOC_Os04g38220	9.6545618
LOC_Os04g12710	LOC_Os07g05150	8.97399709
LOC_Os04g12960	LOC_Os05g28280	9.27450529
LOC_Os04g12720	LOC_Os10g13800	9.31944434
LOC_Os04g12720	LOC_Os06g04280	10.3018276
LOC_Os04g12480	LOC_Os11g29400	9.86055845
LOC_Os04g10400	LOC_Os09g31506	9.66963974
LOC_Os04g12900	LOC_Os09g35800	9.43180723
LOC_Os04g12710	LOC_Os06g37560	9.06926434
LOC_Os04g10010	LOC_Os10g09860	8.86676796
LOC_Os04g12950	LOC_Os04g33480	9.32970247
LOC_Os04g12720	LOC_Os07g26640	8.93686174
LOC_Os04g10400	LOC_Os07g03690	9.14651427
LOC_Os04g12900	LOC_Os06g08080	8.94722456
LOC_Os04g12900	LOC_Os12g41110	9.54800491
LOC_Os04g12480	LOC_Os12g39630	8.83082079
LOC_Os04g12900	LOC_Os10g31940	9.37206863
LOC_Os04g12720	LOC_Os10g26390	9.78597564
LOC_Os04g12480	LOC_Os04g52130	9.34146082
LOC_Os04g12720	LOC_Os11g10510	9.23225789
LOC_Os04g12480	LOC_Os05g01050	10.3737281
LOC_Os04g12900	LOC_Os09g12660	10.3892133
LOC_Os04g12690	LOC_Os06g06980	8.81337391
LOC_Os04g12720	LOC_Os09g25320	9.29502475
LOC_Os04g12720	LOC_Os07g38910	8.82361818
LOC_Os04g10400	LOC_Os11g10510	9.0203311
LOC_Os04g12900	LOC_Os04g56070	9.6415998
LOC_Os04g10010	LOC_Os12g07820	9.77251059
LOC_Os04g10400	LOC_Os05g07090	9.72152128
LOC_Os04g12710	LOC_Os09g12660	8.85037837
LOC_Os04g12690	LOC_Os05g37390	9.90615935
LOC_Os04g10010	LOC_Os12g12590	8.90459268
LOC_Os04g12690	LOC_Os07g37550	10.4676193
LOC_Os04g12720	LOC_Os04g40310	9.40837021

InteractorA	InteractorB	Z score
LOC_Os04g12480	LOC_Os12g38760	9.43131959
LOC_Os04g12950	LOC_Os11g33270	9.73448132
LOC_Os04g12480	LOC_Os10g38470	8.95659641
LOC_Os04g12720	LOC_Os08g28800	8.90981178
LOC_Os04g10400	LOC_Os12g08270	9.27629228
LOC_Os04g12720	LOC_Os06g44080	9.29502475
LOC_Os04g10400	LOC_Os04g36700	9.71991355
LOC_Os04g12480	LOC_Os04g37820	9.09850061
LOC_Os04g12480	LOC_Os12g42876	10.5849835
LOC_Os04g12960	LOC_Os12g16250	12.6608769
LOC_Os04g12980	LOC_Os06g45120	10.0959924
LOC_Os04g12970	LOC_Os07g36190	9.02173491
LOC_Os04g16760	LOC_Os06g07978	10.0349712
LOC_Os04g16740	LOC_Os04g52361	9.83279143
LOC_Os04g12960	LOC_Os07g05800	9.20854365
LOC_Os04g15920	LOC_Os10g29470	9.5608778
LOC_Os04g12980	LOC_Os08g35440	8.80011312
LOC_Os04g16760	LOC_Os07g38970	9.27438102
LOC_Os04g16680	LOC_Os12g24650	9.00432453
LOC_Os04g14680	LOC_Os08g34210	9.08850662
LOC_Os04g16760	LOC_Os06g51084	8.85482722
LOC_Os04g15920	LOC_Os07g05400	9.03142197
LOC_Os04g16740	LOC_Os09g31120	8.81147457
LOC_Os04g16770	LOC_Os05g47540	10.057145
LOC_Os04g12970	LOC_Os12g38770	9.53503037
LOC_Os04g16740	LOC_Os11g08445	9.05752789
LOC_Os04g16760	LOC_Os06g35700	10.6247134
LOC_Os04g14680	LOC_Os06g36770	9.48031734
LOC_Os04g16760	LOC_Os06g07878	10.0349712
LOC_Os04g16740	LOC_Os08g39870	8.90537648
LOC_Os04g15920	LOC_Os07g26660	8.82238525
LOC_Os04g12970	LOC_Os06g23440	8.88563401
LOC_Os04g12960	LOC_Os09g07830	8.80982116
LOC_Os04g16770	LOC_Os04g19740	8.92669615
LOC_Os04g12970	LOC_Os12g10600	10.0701657
LOC_Os04g12980	LOC_Os05g38530	8.94197034
LOC_Os04g12980	LOC_Os07g48880	9.03499446
LOC_Os04g16740	LOC_Os07g03409	9.66639149
LOC_Os04g16760	LOC_Os10g38234	10.7294032
LOC_Os04g12960	LOC_Os11g05570	9.21555262
LOC_Os04g14680	LOC_Os07g39870	9.10511921
LOC_Os04g12970	LOC_Os09g37949	8.89973909
LOC_Os04g12970	LOC_Os07g30170	10.1098998

InteractorA	InteractorB	Z score
LOC_Os04g16740	LOC_Os06g23440	9.00572812
LOC_Os04g12970	LOC_Os06g45710	9.30076216
LOC_Os04g12960	LOC_Os07g46460	9.56236943
LOC_Os04g16760	LOC_Os09g10270	8.81894133
LOC_Os04g16740	LOC_Os05g49770	9.21016884
LOC_Os04g12980	LOC_Os05g49830	9.07888641
LOC_Os04g12980	LOC_Os07g49120	9.5645541
LOC_Os04g12970	LOC_Os05g38560	9.11643933
LOC_Os04g16760	LOC_Os07g44550	9.5856457
LOC_Os04g12970	LOC_Os05g49200	9.60483115
LOC_Os04g14680	LOC_Os04g40310	9.05820244
LOC_Os04g16760	LOC_Os10g25140	8.92441439
LOC_Os04g16740	LOC_Os07g03319	9.66639149
LOC_Os04g16740	LOC_Os07g46280	9.31635916
LOC_Os04g12970	LOC_Os09g12660	9.24951681
LOC_Os04g16680	LOC_Os05g36270	9.82971784
LOC_Os04g16760	LOC_Os05g06970	9.7006403
LOC_Os04g16740	LOC_Os10g08670	10.9957608
LOC_Os04g12970	LOC_Os12g10730	10.7078869
LOC_Os04g12980	LOC_Os04g55290	9.30835867
LOC_Os04g12970	LOC_Os11g26910	8.9291735
LOC_Os04g12970	LOC_Os06g37180	8.80505243
LOC_Os04g12980	LOC_Os08g37800	11.2729797
LOC_Os04g12960	LOC_Os09g23540	8.82742738
LOC_Os04g16680	LOC_Os07g23850	9.72879593
LOC_Os04g16740	LOC_Os10g39880	9.07490808
LOC_Os04g12960	LOC_Os08g34190	9.59659591
LOC_Os04g15920	LOC_Os06g36820	9.6516594
LOC_Os04g16680	LOC_Os12g12580	9.11984879
LOC_Os04g14680	LOC_Os05g45810	9.65392584
LOC_Os04g12960	LOC_Os07g48060	9.90999743
LOC_Os04g14680	LOC_Os12g16220	9.53253571
LOC_Os04g12980	LOC_Os08g04540	9.57695208
LOC_Os04g12970	LOC_Os09g31490	8.87255258
LOC_Os04g16760	LOC_Os06g05110	8.98997507
LOC_Os04g14680	LOC_Os08g44340	9.0394665
LOC_Os04g16680	LOC_Os05g28180	9.50657233
LOC_Os04g12980	LOC_Os06g35650	9.37226586
LOC_Os04g16760	LOC_Os08g29520	9.65931695
LOC_Os04g12980	LOC_Os08g35740	8.92743109
LOC_Os04g15920	LOC_Os08g09770	10.1007544
LOC_Os04g15920	LOC_Os12g12514	9.77398928
LOC_Os04g12970	LOC_Os10g22450	9.34948539

InteractorA	InteractorB	Z score
LOC_Os04g16760	LOC_Os07g07320	9.02968884
LOC_Os04g14680	LOC_Os10g29620	9.16313462
LOC_Os04g12980	LOC_Os10g01540	9.80347167
LOC_Os04g16760	LOC_Os07g42950	9.88948228
LOC_Os04g16680	LOC_Os07g07320	9.7697672
LOC_Os04g16760	LOC_Os07g41750	9.0020077
LOC_Os04g16680	LOC_Os11g08460	10.0498023
LOC_Os04g16740	LOC_Os07g03499	9.66639149
LOC_Os04g16760	LOC_Os09g36830	9.42672157
LOC_Os04g12960	LOC_Os09g08120	9.46003764
LOC_Os04g16680	LOC_Os04g53290	10.1578788
LOC_Os04g12970	LOC_Os07g30640	9.13779154
LOC_Os04g16770	LOC_Os04g55040	8.82634571
LOC_Os04g16680	LOC_Os12g17910	9.40496221
LOC_Os04g12970	LOC_Os05g40990	9.74517069
LOC_Os04g15920	LOC_Os12g12560	10.680148
LOC_Os04g12970	LOC_Os05g47545	8.99117381
LOC_Os04g16680	LOC_Os10g41480	9.85563871
LOC_Os04g12980	LOC_Os08g06060	9.15923922
LOC_Os04g16760	LOC_Os04g43760	9.00385669
LOC_Os04g12980	LOC_Os09g08120	11.0771337
LOC_Os04g15920	LOC_Os10g01570	8.81721929
LOC_Os04g16770	LOC_Os05g44140	8.91469402
LOC_Os04g16680	LOC_Os09g25150	9.04063026
LOC_Os04g14680	LOC_Os05g05670	8.95943764
LOC_Os04g16680	LOC_Os07g07060	10.0022765
LOC_Os04g16740	LOC_Os07g35940	10.2394482
LOC_Os04g16760	LOC_Os09g20220	9.12305136
LOC_Os04g12970	LOC_Os05g01600	8.84585151
LOC_Os04g16760	LOC_Os10g38140	9.4849188
LOC_Os04g12970	LOC_Os06g04510	9.82853798
LOC_Os04g16772	LOC_Os05g45220	8.88990277
LOC_Os04g16844	LOC_Os05g33240	9.22941297
LOC_Os04g16818	LOC_Os06g03770	10.0257261
LOC_Os04g16818	LOC_Os04g37820	10.182756
LOC_Os04g16828	LOC_Os07g35880	8.83894616
LOC_Os04g16818	LOC_Os08g41880	9.41889211
LOC_Os04g16826	LOC_Os06g45710	8.88383085
LOC_Os04g16772	LOC_Os04g27980	10.018694
LOC_Os04g16846	LOC_Os05g05800	9.35768804
LOC_Os04g16846	LOC_Os07g48050	10.1211797
LOC_Os04g16826	LOC_Os05g02940	8.8354122
LOC_Os04g16818	LOC_Os12g21798	9.1260617

InteractorA	InteractorB	Z score
LOC_Os04g16772	LOC_Os04g41960	8.82637033
LOC_Os04g16826	LOC_Os09g27820	8.91621318
LOC_Os04g16846	LOC_Os04g37640	9.07180094
LOC_Os04g16818	LOC_Os07g40290	9.68355467
LOC_Os04g16772	LOC_Os07g48040	9.82014011
LOC_Os04g16826	LOC_Os04g56950	8.9203009
LOC_Os04g16770	LOC_Os09g10200	9.48188578
LOC_Os04g16826	LOC_Os10g38274	8.82040937
LOC_Os04g16772	LOC_Os08g41880	9.41887433
LOC_Os04g16826	LOC_Os10g21230	8.82041015
LOC_Os04g16772	LOC_Os05g08430	10.0254748
LOC_Os04g16828	LOC_Os07g38540	10.1302762
LOC_Os04g16818	LOC_Os07g48040	9.82015235
LOC_Os04g16770	LOC_Os11g26860	8.8608346
LOC_Os04g16772	LOC_Os08g06550	10.6531712
LOC_Os04g16818	LOC_Os09g38030	9.58502216
LOC_Os04g16772	LOC_Os09g08910	9.21414864
LOC_Os04g16772	LOC_Os07g40290	9.68350894
LOC_Os04g16874	LOC_Os04g39880	9.66018903
LOC_Os04g16826	LOC_Os10g38660	8.85371418
LOC_Os04g16772	LOC_Os06g35490	9.44932709
LOC_Os04g16770	LOC_Os10g38360	8.8911869
LOC_Os04g16844	LOC_Os05g42350	8.87625392
LOC_Os04g16844	LOC_Os05g27950	9.78723669
LOC_Os04g16872	LOC_Os12g25690	8.93192045
LOC_Os04g16826	LOC_Os04g45290	9.00513371
LOC_Os04g16772	LOC_Os07g48050	10.5204067
LOC_Os04g16844	LOC_Os06g09910	10.0710108
LOC_Os04g16874	LOC_Os05g07880	8.91931483
LOC_Os04g16818	LOC_Os05g45220	8.88990277
LOC_Os04g16844	LOC_Os10g07616	9.08354104
LOC_Os04g16872	LOC_Os11g10510	8.90268373
LOC_Os04g16770	LOC_Os06g23760	9.81946663
LOC_Os04g16872	LOC_Os09g31506	9.43767244
LOC_Os04g16844	LOC_Os10g01080	11.3283559
LOC_Os04g16872	LOC_Os07g07719	10.02779
LOC_Os04g16818	LOC_Os09g08910	9.21414864
LOC_Os04g16772	LOC_Os08g02410	9.39002599
LOC_Os04g16846	LOC_Os08g35440	8.97470838
LOC_Os04g16828	LOC_Os07g07060	9.0071317
LOC_Os04g16844	LOC_Os06g22140	8.84040918
LOC_Os04g16872	LOC_Os06g35730	11.0093511
LOC_Os04g16846	LOC_Os12g12560	9.46659128

InteractorA	InteractorB	Z score
LOC_Os04g16818	LOC_Os10g31000	9.00579729
LOC_Os04g16828	LOC_Os12g38760	9.62756182
LOC_Os04g16818	LOC_Os05g49830	9.91748784
LOC_Os04g16772	LOC_Os05g37700	9.09721717
LOC_Os04g16818	LOC_Os05g30530	9.51726037
LOC_Os04g16872	LOC_Os09g23530	10.1830334
LOC_Os04g16772	LOC_Os11g25330	9.92153133
LOC_Os04g16818	LOC_Os11g25330	9.92153133
LOC_Os04g16826	LOC_Os05g49830	9.86324804
LOC_Os04g16828	LOC_Os10g21248	10.0520913
LOC_Os04g16844	LOC_Os12g25710	11.7484235
LOC_Os04g16772	LOC_Os05g30530	9.51726146
LOC_Os04g16826	LOC_Os04g17064	8.96223349
LOC_Os04g16844	LOC_Os07g07240	9.43572423
LOC_Os04g16872	LOC_Os05g11710	11.0093511
LOC_Os04g16844	LOC_Os10g10434	9.61143316
LOC_Os04g16772	LOC_Os12g21798	9.12606011
LOC_Os04g16770	LOC_Os09g04050	9.48118025
LOC_Os04g16818	LOC_Os04g27980	10.018694
LOC_Os04g16826	LOC_Os05g48980	9.43078744
LOC_Os04g16770	LOC_Os12g16410	9.84537093
LOC_Os04g16818	LOC_Os06g35490	9.44932709
LOC_Os04g16846	LOC_Os04g58710	8.99053063
LOC_Os04g16844	LOC_Os04g56320	9.89082761
LOC_Os04g16770	LOC_Os10g28320	8.9572257
LOC_Os04g16828	LOC_Os04g32460	8.80263928
LOC_Os04g16818	LOC_Os08g06550	10.6531712
LOC_Os04g16772	LOC_Os05g49830	9.91748704
LOC_Os04g16826	LOC_Os10g21250	9.28042324
LOC_Os04g16818	LOC_Os08g02410	9.39002599
LOC_Os04g16772	LOC_Os04g43410	12.7496896
LOC_Os04g16826	LOC_Os10g28320	9.17354926
LOC_Os04g16846	LOC_Os09g37949	9.27511671
LOC_Os04g16770	LOC_Os07g42600	8.86164673
LOC_Os04g16846	LOC_Os04g33740	9.1025958
LOC_Os04g16844	LOC_Os12g08270	10.8243297
LOC_Os04g16772	LOC_Os04g37820	10.182756
LOC_Os04g16844	LOC_Os06g05690	9.6508851
LOC_Os04g16772	LOC_Os10g31000	9.00579729
LOC_Os04g16818	LOC_Os04g43410	12.7496559
LOC_Os04g16844	LOC_Os06g04000	9.16868579
LOC_Os04g16770	LOC_Os07g08030	9.25455127
LOC_Os04g16772	LOC_Os09g38030	9.58502216

InteractorA	InteractorB	Z score
LOC_Os04g16772	LOC_Os06g03770	10.0257261
LOC_Os04g16818	LOC_Os04g41960	8.82637033
LOC_Os04g16818	LOC_Os07g48050	10.5204067
LOC_Os04g16770	LOC_Os07g20544	9.27639535
LOC_Os04g16818	LOC_Os05g37700	9.09721717
LOC_Os04g16844	LOC_Os04g54330	9.15876971
LOC_Os04g16818	LOC_Os05g08430	10.0254748
LOC_Os04g16826	LOC_Os07g05160	9.11210747
LOC_Os04g16828	LOC_Os09g08072	9.19016379
LOC_Os04g16844	LOC_Os10g38740	9.65864361
LOC_Os04g19740	LOC_Os05g27950	8.83114017
LOC_Os04g20070	LOC_Os10g34760	9.00336146
LOC_Os04g19740	LOC_Os10g21192	9.11234005
LOC_Os04g18200	LOC_Os05g36280	9.41814993
LOC_Os04g18650	LOC_Os12g07980	9.03845334
LOC_Os04g17650	LOC_Os11g01872	9.42037014
LOC_Os04g19740	LOC_Os10g35480	10.3313778
LOC_Os04g17650	LOC_Os05g05680	9.12302775
LOC_Os04g18650	LOC_Os07g38860	8.90781097
LOC_Os04g17650	LOC_Os05g01600	9.36165441
LOC_Os04g16874	LOC_Os10g38710	9.32833154
LOC_Os04g18200	LOC_Os06g06460	9.41814993
LOC_Os04g16874	LOC_Os10g41550	8.93536584
LOC_Os04g20810	LOC_Os06g06090	9.71399295
LOC_Os04g17064	LOC_Os04g45290	8.93407694
LOC_Os04g18200	LOC_Os06g06510	9.41814993
LOC_Os04g18650	LOC_Os12g44000	9.24825975
LOC_Os04g18200	LOC_Os11g31620	8.81345883
LOC_Os04g18200	LOC_Os07g35880	9.64149026
LOC_Os04g20810	LOC_Os06g46340	8.90576463
LOC_Os04g18650	LOC_Os04g45290	8.91813499
LOC_Os04g20810	LOC_Os07g49220	9.99867447
LOC_Os04g17650	LOC_Os04g29550	8.9408803
LOC_Os04g19740	LOC_Os11g32520	9.35818741
LOC_Os04g18650	LOC_Os06g27770	9.457481
LOC_Os04g18650	LOC_Os08g23730	8.83779478
LOC_Os04g19740	LOC_Os07g44440	9.92210504
LOC_Os04g17064	LOC_Os10g21344	8.96223349
LOC_Os04g17064	LOC_Os09g09270	9.02656377
LOC_Os04g18650	LOC_Os08g31870	8.81163271
LOC_Os04g17064	LOC_Os07g48020	9.02762625
LOC_Os04g18200	LOC_Os11g07020	8.83430242
LOC_Os04g20810	LOC_Os12g43370	9.70854746

InteractorA	InteractorB	Z score
LOC_Os04g16874	LOC_Os10g38229	9.24243235
LOC_Os04g18200	LOC_Os08g02410	10.0712248
LOC_Os04g18650	LOC_Os08g42730	9.1502923
LOC_Os04g17064	LOC_Os04g43400	9.0280388
LOC_Os04g19740	LOC_Os12g24650	9.33542889
LOC_Os04g17650	LOC_Os06g36880	10.466119
LOC_Os04g16874	LOC_Os05g36270	9.96366288
LOC_Os04g17650	LOC_Os11g32580	10.6389318
LOC_Os04g17064	LOC_Os07g01780	9.32974627
LOC_Os04g17064	LOC_Os09g25150	8.8456523
LOC_Os04g19740	LOC_Os06g04270	13.4564448
LOC_Os04g20070	LOC_Os07g44740	8.90992338
LOC_Os04g17650	LOC_Os06g11210	9.80871146
LOC_Os04g18200	LOC_Os11g41610	9.00929074
LOC_Os04g18200	LOC_Os11g29190	9.00590704
LOC_Os04g17064	LOC_Os06g11210	10.0873623
LOC_Os04g18200	LOC_Os11g05730	9.41814993
LOC_Os04g17650	LOC_Os04g28870	8.85229789
LOC_Os04g20810	LOC_Os06g35490	9.1331391
LOC_Os04g18650	LOC_Os05g41080	9.47393927
LOC_Os04g18200	LOC_Os07g46830	8.94311423
LOC_Os04g17650	LOC_Os11g26860	8.97378745
LOC_Os04g19740	LOC_Os08g35420	8.92669615
LOC_Os04g18200	LOC_Os06g35520	9.40920692
LOC_Os04g17064	LOC_Os04g56230	8.86972749
LOC_Os04g18650	LOC_Os11g32510	9.28722826
LOC_Os04g19740	LOC_Os04g26920	8.99148518
LOC_Os04g20810	LOC_Os10g28050	8.93486397
LOC_Os04g18650	LOC_Os06g04270	9.65436879
LOC_Os04g16874	LOC_Os11g29400	9.0903649
LOC_Os04g17064	LOC_Os12g12590	8.85006296
LOC_Os04g18650	LOC_Os04g55040	10.5074431
LOC_Os04g20810	LOC_Os09g07830	9.12811777
LOC_Os04g17650	LOC_Os06g37660	9.07034757
LOC_Os04g17064	LOC_Os10g21266	8.85517147
LOC_Os04g20810	LOC_Os09g31410	10.7118322
LOC_Os04g18650	LOC_Os08g43560	9.03155366
LOC_Os04g16874	LOC_Os12g43100	8.9656898
LOC_Os04g17064	LOC_Os11g32580	9.45530622
LOC_Os04g19740	LOC_Os06g46940	10.5297679
LOC_Os04g16874	LOC_Os07g03710	9.29848203
LOC_Os04g17650	LOC_Os08g03040	9.18133515
LOC_Os04g17650	LOC_Os08g27840	8.81448034

InteractorA	InteractorB	Z score
LOC_Os04g18200	LOC_Os06g50300	12.2600755
LOC_Os04g16874	LOC_Os10g21250	12.0252863
LOC_Os04g16874	LOC_Os11g19800	9.89643845
LOC_Os04g18650	LOC_Os07g05160	9.34248472
LOC_Os04g18200	LOC_Os08g14760	8.89647425
LOC_Os04g17650	LOC_Os06g42130	8.90224516
LOC_Os04g17650	LOC_Os08g34280	8.87963347
LOC_Os04g19740	LOC_Os10g23900	8.85279533
LOC_Os04g17650	LOC_Os06g35530	9.37952255
LOC_Os01g17180	LOC_Os03g52090	9.1958477
LOC_Os01g17180	LOC_Os03g17470	9.1623997
LOC_Os01g17180	LOC_Os03g55800	10.214078
LOC_Os01g16152	LOC_Os12g42884	9.6395045
LOC_Os01g16240	LOC_Os05g40420	8.99798233
LOC_Os01g16960	LOC_Os06g40940	9.03653238
LOC_Os01g17180	LOC_Os07g13980	9.13047696
LOC_Os01g16900	LOC_Os05g51050	10.4831593
LOC_Os01g16960	LOC_Os03g09910	9.39076146
LOC_Os01g16900	LOC_Os07g17010	8.84353745
LOC_Os01g16900	LOC_Os11g26910	8.85290398
LOC_Os01g16900	LOC_Os06g04620	10.7202091
LOC_Os01g16960	LOC_Os03g17980	9.77930687
LOC_Os01g16960	LOC_Os05g31110	9.44932651
LOC_Os01g16890	LOC_Os11g08940	10.9166614
LOC_Os01g16240	LOC_Os05g04690	9.20513076
LOC_Os01g16900	LOC_Os05g08960	9.10438559
LOC_Os01g16890	LOC_Os08g09250	9.48886128
LOC_Os01g16960	LOC_Os05g04340	9.12068367
LOC_Os01g16960	LOC_Os09g33860	9.55485065
LOC_Os01g16240	LOC_Os01g51170	8.96754087
LOC_Os01g16960	LOC_Os01g52400	8.93147519
LOC_Os01g16960	LOC_Os01g22230	8.99930047
LOC_Os01g16240	LOC_Os12g21798	8.86737368
LOC_Os01g16240	LOC_Os04g01470	9.71790977
LOC_Os01g16900	LOC_Os01g49120	9.09664447
LOC_Os01g16900	LOC_Os06g05250	9.25791308
LOC_Os01g16240	LOC_Os02g41470	11.0949732
LOC_Os01g16900	LOC_Os01g73790	9.07844833
LOC_Os01g17180	LOC_Os02g46130	10.3766144
LOC_Os01g16240	LOC_Os02g33110	9.95248506
LOC_Os01g17180	LOC_Os02g52800	9.24794841
LOC_Os01g16240	LOC_Os03g61280	9.12123091
LOC_Os01g16900	LOC_Os05g36010	9.05400005

InteractorA	InteractorB	Z score
LOC_Os01g16960	LOC_Os07g10590	9.92941128
LOC_Os01g16890	LOC_Os04g59150	8.85834002
LOC_Os01g16240	LOC_Os08g37790	8.97100987
LOC_Os01g16240	LOC_Os09g20090	9.31345523
LOC_Os01g16900	LOC_Os09g20820	8.95616711
LOC_Os01g17180	LOC_Os05g49200	9.42878388
LOC_Os01g16900	LOC_Os04g42920	10.9387301
LOC_Os01g16900	LOC_Os07g43510	12.0622863
LOC_Os01g16240	LOC_Os03g01170	9.56599218
LOC_Os01g16240	LOC_Os07g20544	10.1057086
LOC_Os01g16900	LOC_Os04g58640	9.81095029
LOC_Os01g17180	LOC_Os03g11530	9.18380328
LOC_Os01g16240	LOC_Os02g14430	8.87770923
LOC_Os01g16152	LOC_Os12g42876	9.77377984
LOC_Os01g16890	LOC_Os08g44350	9.23351405
LOC_Os01g17180	LOC_Os03g47000	10.621325
LOC_Os01g16960	LOC_Os12g16220	9.49154156
LOC_Os01g16900	LOC_Os08g04180	8.9120511
LOC_Os01g16900	LOC_Os07g48880	10.2908207
LOC_Os01g16240	LOC_Os03g61600	9.11555583
LOC_Os01g16960	LOC_Os09g28400	9.1492653
LOC_Os01g16890	LOC_Os11g16590	8.89369652
LOC_Os01g16960	LOC_Os06g06040	10.0779294
LOC_Os01g16900	LOC_Os06g49970	8.98079693
LOC_Os01g16960	LOC_Os09g38620	8.99762398
LOC_Os01g16900	LOC_Os04g39210	10.2924703
LOC_Os01g16960	LOC_Os12g22650	8.97896677
LOC_Os01g16240	LOC_Os01g54490	9.82406579
LOC_Os01g16240	LOC_Os02g08490	9.17798558
LOC_Os01g16900	LOC_Os10g25930	10.7865774
LOC_Os01g16900	LOC_Os03g41460	8.86495724
LOC_Os01g16240	LOC_Os01g25484	9.11528943
LOC_Os01g16240	LOC_Os06g09450	8.99844308
LOC_Os01g17180	LOC_Os04g32460	10.1824457
LOC_Os01g16240	LOC_Os09g36450	9.67447622
LOC_Os01g16900	LOC_Os08g33100	9.17880464
LOC_Os01g16890	LOC_Os03g45320	9.17142905
LOC_Os01g16900	LOC_Os10g26010	9.2600427
LOC_Os01g16240	LOC_Os07g32800	9.4039647
LOC_Os01g16900	LOC_Os10g30580	9.08389376
LOC_Os01g16900	LOC_Os02g24354	9.0897609
LOC_Os01g16890	LOC_Os06g35530	11.0533826
LOC_Os01g16900	LOC_Os06g12180	9.10849431

InteractorA	InteractorB	Z score
LOC_Os01g16900	LOC_Os09g31120	9.3220353
LOC_Os01g16900	LOC_Os08g35740	9.03494484
LOC_Os01g16240	LOC_Os02g02560	9.93226131
LOC_Os01g16240	LOC_Os11g48110	8.86939144
LOC_Os01g16240	LOC_Os03g52970	8.82727003
LOC_Os01g16240	LOC_Os04g08350	9.42201064
LOC_Os01g16900	LOC_Os02g08100	9.19150982
LOC_Os01g16900	LOC_Os01g51220	9.07422912
LOC_Os01g16900	LOC_Os02g10310	9.40472882
LOC_Os01g16900	LOC_Os05g33380	9.27979855
LOC_Os01g16240	LOC_Os07g28480	9.43752208
LOC_Os01g17180	LOC_Os03g01640	8.89171126
LOC_Os01g16960	LOC_Os02g52420	9.50752511
LOC_Os01g16900	LOC_Os02g03870	8.9276631
LOC_Os01g16900	LOC_Os05g47640	9.7288503
LOC_Os01g16240	LOC_Os03g04260	9.51865384
LOC_Os01g16890	LOC_Os06g06560	8.92551005
LOC_Os01g16960	LOC_Os05g51480	9.18157339
LOC_Os01g16240	LOC_Os08g17680	8.93751946
LOC_Os01g16900	LOC_Os03g56410	10.1552834
LOC_Os01g16890	LOC_Os07g06970	8.92146874
LOC_Os01g16240	LOC_Os06g01360	9.27554006
LOC_Os04g22730	LOC_Os07g08170	9.05929421
LOC_Os04g26870	LOC_Os08g09240	9.37727407
LOC_Os04g24430	LOC_Os08g40170	9.17586802
LOC_Os04g24430	LOC_Os04g45290	9.40771736
LOC_Os04g25990	LOC_Os07g25590	9.473695
LOC_Os04g22660	LOC_Os05g35400	9.01426943
LOC_Os04g25990	LOC_Os06g02380	8.8943694
LOC_Os04g25990	LOC_Os11g26910	9.06600061
LOC_Os04g26870	LOC_Os07g35940	9.019219
LOC_Os04g26870	LOC_Os08g06100	9.82390741
LOC_Os04g22660	LOC_Os05g48030	8.97734619
LOC_Os04g22660	LOC_Os12g17540	8.89938605
LOC_Os04g24430	LOC_Os04g56160	10.1146531
LOC_Os04g25990	LOC_Os07g30200	8.84615084
LOC_Os04g24430	LOC_Os11g25260	9.12089732
LOC_Os04g25400	LOC_Os10g39840	10.2174178
LOC_Os04g25400	LOC_Os12g40510	9.80071713
LOC_Os04g26870	LOC_Os06g37560	8.93101784
LOC_Os04g25400	LOC_Os04g53230	9.46508769
LOC_Os04g24430	LOC_Os08g34170	11.4844184
LOC_Os04g26910	LOC_Os12g42884	9.00485344

InteractorA	InteractorB	Z score
LOC_Os04g26910	LOC_Os09g11230	9.77146796
LOC_Os04g26870	LOC_Os07g32800	9.48560232
LOC_Os04g22660	LOC_Os10g40720	8.83036067
LOC_Os04g25990	LOC_Os08g44960	9.05298236
LOC_Os04g26870	LOC_Os11g01872	9.19336874
LOC_Os04g22730	LOC_Os11g34450	9.10083895
LOC_Os04g25990	LOC_Os10g26600	8.83167833
LOC_Os04g26910	LOC_Os07g08500	8.98825895
LOC_Os04g25990	LOC_Os05g25850	9.85563854
LOC_Os04g22730	LOC_Os10g26010	8.86598787
LOC_Os04g24430	LOC_Os10g17660	8.96441892
LOC_Os04g22730	LOC_Os06g51150	9.28648843
LOC_Os04g24430	LOC_Os10g17680	8.96441892
LOC_Os04g22730	LOC_Os12g41110	10.0935016
LOC_Os04g24430	LOC_Os12g23170	10.433276
LOC_Os04g25990	LOC_Os07g07770	9.11905619
LOC_Os04g25990	LOC_Os06g44620	10.400994
LOC_Os04g25990	LOC_Os12g13800	9.97879378
LOC_Os04g24430	LOC_Os10g21268	10.5124613
LOC_Os04g22660	LOC_Os10g40090	8.84307565
LOC_Os04g26910	LOC_Os07g44460	10.279112
LOC_Os04g25990	LOC_Os09g24530	9.64081928
LOC_Os04g25400	LOC_Os07g08660	8.82455015
LOC_Os04g22660	LOC_Os09g33500	9.43310913
LOC_Os04g26910	LOC_Os07g30170	8.84747942
LOC_Os04g24430	LOC_Os09g24990	10.5123353
LOC_Os04g26870	LOC_Os12g31370	9.7036828
LOC_Os04g25990	LOC_Os11g03290	8.80378603
LOC_Os04g22730	LOC_Os07g40290	9.11898431
LOC_Os04g22730	LOC_Os09g25150	8.82220211
LOC_Os04g25400	LOC_Os05g28180	8.95392116
LOC_Os04g26870	LOC_Os06g21980	9.85095391
LOC_Os04g24430	LOC_Os05g35330	10.5124613
LOC_Os04g22730	LOC_Os09g39810	9.85604564
LOC_Os04g25990	LOC_Os04g56920	8.87577065
LOC_Os04g26910	LOC_Os08g04180	9.20534191
LOC_Os04g26920	LOC_Os05g30530	9.36263616
LOC_Os04g25400	LOC_Os09g36830	10.8118816
LOC_Os04g25990	LOC_Os07g30990	8.85198734
LOC_Os04g26910	LOC_Os09g34250	8.86912845
LOC_Os04g26910	LOC_Os07g46280	8.80436632
LOC_Os04g24520	LOC_Os12g07980	8.97699992
LOC_Os04g25400	LOC_Os11g41610	8.9598128

InteractorA	InteractorB	Z score
LOC_Os04g24520	LOC_Os04g33240	8.90409163
LOC_Os04g26910	LOC_Os11g47760	10.0239078
LOC_Os04g22730	LOC_Os10g30580	9.74331925
LOC_Os04g24520	LOC_Os12g43630	8.86150773
LOC_Os04g24520	LOC_Os08g40140	9.27770164
LOC_Os04g26910	LOC_Os09g08120	9.40231962
LOC_Os04g26910	LOC_Os06g37080	9.71222795
LOC_Os04g24520	LOC_Os08g28190	9.80304349
LOC_Os04g25400	LOC_Os04g32710	11.2656561
LOC_Os04g26910	LOC_Os10g08580	13.6707436
LOC_Os04g26910	LOC_Os11g08445	10.4218744
LOC_Os04g25400	LOC_Os08g41990	9.58741518
LOC_Os04g26870	LOC_Os08g39870	9.75627753
LOC_Os04g24430	LOC_Os09g38030	9.59429338
LOC_Os04g26910	LOC_Os12g31370	9.22666149
LOC_Os04g24430	LOC_Os08g20730	9.98280805
LOC_Os04g24520	LOC_Os04g58640	9.27653926
LOC_Os04g25400	LOC_Os08g32870	9.50980513
LOC_Os04g25990	LOC_Os09g32800	9.64640903
LOC_Os04g26910	LOC_Os12g07050	9.88012858
LOC_Os04g22730	LOC_Os07g38540	10.3492354
LOC_Os04g22660	LOC_Os07g34140	9.71583285
LOC_Os04g26870	LOC_Os05g28180	9.06306396
LOC_Os04g26910	LOC_Os10g26130	9.96630431
LOC_Os04g22660	LOC_Os11g32520	10.1320091
LOC_Os04g25990	LOC_Os04g57390	9.60773553
LOC_Os04g26910	LOC_Os11g04954	10.4027431
LOC_Os04g26870	LOC_Os12g13390	13.2798309
LOC_Os04g26910	LOC_Os08g06550	9.18135578
LOC_Os04g25990	LOC_Os06g13450	9.44209358
LOC_Os04g24430	LOC_Os07g02210	9.65915018
LOC_Os04g28870	LOC_Os09g38030	8.90910549
LOC_Os04g27060	LOC_Os08g06100	12.7359365
LOC_Os04g28180	LOC_Os11g40150	9.24804711
LOC_Os04g27980	LOC_Os04g57380	9.04210694
LOC_Os04g28870	LOC_Os12g13810	8.82099786
LOC_Os04g26920	LOC_Os08g37490	8.94093003
LOC_Os04g27980	LOC_Os12g40830	8.96377782
LOC_Os04g27980	LOC_Os06g28550	9.24805947
LOC_Os04g29030	LOC_Os05g28180	10.4423147
LOC_Os04g27060	LOC_Os08g37800	10.6668104
LOC_Os04g28180	LOC_Os05g06300	9.37461809
LOC_Os04g28180	LOC_Os09g12590	10.0607672

InteractorA	InteractorB	Z score
LOC_Os04g29550	LOC_Os05g40420	9.09426673
LOC_Os04g28180	LOC_Os08g32620	8.94740449
LOC_Os04g28870	LOC_Os10g08670	8.8148412
LOC_Os04g28870	LOC_Os05g36290	10.4037049
LOC_Os04g29030	LOC_Os07g36130	9.40960641
LOC_Os04g29550	LOC_Os05g12240	8.86409248
LOC_Os04g27980	LOC_Os05g37390	10.5806436
LOC_Os04g28990	LOC_Os11g41130	9.08044493
LOC_Os04g26920	LOC_Os10g29470	9.05810758
LOC_Os04g27060	LOC_Os07g37550	9.01944446
LOC_Os04g27980	LOC_Os12g25710	9.78272915
LOC_Os04g28870	LOC_Os07g46310	10.1964557
LOC_Os04g28180	LOC_Os04g39864	9.15113824
LOC_Os04g27060	LOC_Os07g43820	8.81634334
LOC_Os04g27980	LOC_Os07g05180	9.65134499
LOC_Os04g28180	LOC_Os05g12180	9.15160361
LOC_Os04g27860	LOC_Os04g58880	9.43108767
LOC_Os04g27860	LOC_Os06g14510	10.8229736
LOC_Os04g29550	LOC_Os04g55740	9.12554252
LOC_Os04g27980	LOC_Os10g39120	9.45614153
LOC_Os04g29550	LOC_Os04g57380	9.69598983
LOC_Os04g28870	LOC_Os06g11280	8.81453732
LOC_Os04g27860	LOC_Os10g21268	8.91727258
LOC_Os04g27860	LOC_Os07g29750	9.01948609
LOC_Os04g28870	LOC_Os12g16410	8.87014854
LOC_Os04g29030	LOC_Os05g44340	9.48473479
LOC_Os04g29030	LOC_Os05g51480	9.82260567
LOC_Os04g29030	LOC_Os08g33370	9.56969694
LOC_Os04g29550	LOC_Os06g04620	9.1637049
LOC_Os04g26920	LOC_Os07g31770	9.50056379
LOC_Os04g29550	LOC_Os05g08430	8.96164461
LOC_Os04g28180	LOC_Os12g03816	9.60788583
LOC_Os04g27860	LOC_Os05g35330	8.91729994
LOC_Os04g28180	LOC_Os10g21240	9.91513028
LOC_Os04g27060	LOC_Os11g08120	9.09810248
LOC_Os04g27860	LOC_Os12g44030	10.0962667
LOC_Os04g27980	LOC_Os05g46000	9.70316007
LOC_Os04g28180	LOC_Os04g46620	9.00004091
LOC_Os04g28870	LOC_Os07g28480	8.98202704
LOC_Os04g28870	LOC_Os10g25930	9.07631616
LOC_Os04g27980	LOC_Os07g25024	10.018694
LOC_Os04g28180	LOC_Os12g39630	9.41759169
LOC_Os04g28180	LOC_Os05g03820	9.64977321

InteractorA	InteractorB	Z score
LOC_Os04g28180	LOC_Os06g15990	8.92882102
LOC_Os04g28180	LOC_Os09g08910	8.87869789
LOC_Os04g27860	LOC_Os04g41960	9.69663399
LOC_Os04g28870	LOC_Os07g49120	10.1521302
LOC_Os04g27980	LOC_Os09g24412	10.018694
LOC_Os04g28990	LOC_Os08g35740	10.9628762
LOC_Os04g27980	LOC_Os08g15266	10.0186977
LOC_Os04g28990	LOC_Os12g16240	9.24079463
LOC_Os04g28180	LOC_Os12g08270	9.27131597
LOC_Os04g27860	LOC_Os07g07770	9.48228964
LOC_Os04g29030	LOC_Os06g04270	9.039866
LOC_Os04g28180	LOC_Os12g31370	11.4470324
LOC_Os04g27980	LOC_Os10g21352	10.0186977
LOC_Os04g26920	LOC_Os05g43510	9.09815587
LOC_Os04g27980	LOC_Os05g04340	9.6127384
LOC_Os04g27980	LOC_Os04g59200	8.81043834
LOC_Os04g29550	LOC_Os05g06480	9.68610006
LOC_Os04g28180	LOC_Os09g08120	9.09188858
LOC_Os04g28990	LOC_Os11g25330	9.64016963
LOC_Os04g28870	LOC_Os12g01922	9.53167526
LOC_Os04g29030	LOC_Os07g42924	8.90721909
LOC_Os04g29550	LOC_Os04g33970	8.85051295
LOC_Os04g27980	LOC_Os10g30840	8.92437714
LOC_Os04g27980	LOC_Os05g46860	8.95541532
LOC_Os04g30800	LOC_Os04g39880	8.9988161
LOC_Os04g31070	LOC_Os04g56070	8.8534788
LOC_Os04g32460	LOC_Os09g39780	8.8329505
LOC_Os04g29550	LOC_Os06g21570	8.99669264
LOC_Os04g32650	LOC_Os05g37390	8.85908698
LOC_Os04g32460	LOC_Os08g39870	11.3543712
LOC_Os04g32710	LOC_Os04g39864	10.1598059
LOC_Os04g32650	LOC_Os12g25710	9.34742162
LOC_Os04g30420	LOC_Os06g14510	8.89227966
LOC_Os04g30800	LOC_Os08g43560	10.0483545
LOC_Os04g30420	LOC_Os11g32540	9.27462624
LOC_Os04g31960	LOC_Os06g06100	8.93748636
LOC_Os04g32460	LOC_Os04g57410	9.36907683
LOC_Os04g32460	LOC_Os05g46860	9.02855268
LOC_Os04g30420	LOC_Os12g40830	8.94873775
LOC_Os04g31960	LOC_Os04g48850	10.461838
LOC_Os04g31960	LOC_Os12g38770	8.97994446
LOC_Os04g30420	LOC_Os08g34290	8.8921446
LOC_Os04g30420	LOC_Os12g12580	8.88538641

InteractorA	InteractorB	Z score
LOC_Os04g32650	LOC_Os05g05800	8.86054558
LOC_Os04g30420	LOC_Os06g36820	9.96822291
LOC_Os04g32650	LOC_Os07g34520	8.92327955
LOC_Os04g32650	LOC_Os06g13450	9.04075554
LOC_Os04g32460	LOC_Os04g56320	9.16967568
LOC_Os04g32460	LOC_Os07g20544	10.3409802
LOC_Os04g32460	LOC_Os06g45120	9.10240498
LOC_Os04g31070	LOC_Os08g01660	9.61993782
LOC_Os04g32710	LOC_Os04g53850	8.95422345
LOC_Os04g30420	LOC_Os05g33140	10.2055411
LOC_Os04g32650	LOC_Os05g29880	9.07236757
LOC_Os04g32460	LOC_Os05g22722	8.80263928
LOC_Os04g30420	LOC_Os06g11210	9.00131032
LOC_Os04g29550	LOC_Os09g32810	11.2284691
LOC_Os04g32710	LOC_Os07g02210	9.09845739
LOC_Os04g32650	LOC_Os07g43260	9.87401798
LOC_Os04g29550	LOC_Os08g03040	9.4926914
LOC_Os04g32460	LOC_Os05g48980	9.60676176
LOC_Os04g29550	LOC_Os06g33210	10.2170135
LOC_Os04g29550	LOC_Os12g32240	8.9959724
LOC_Os04g32460	LOC_Os06g11290	8.97622317
LOC_Os04g32010	LOC_Os07g06970	8.99658143
LOC_Os04g32650	LOC_Os10g38540	9.2877535
LOC_Os04g31960	LOC_Os10g42940	9.47350364
LOC_Os04g32460	LOC_Os12g40510	9.65923264
LOC_Os04g32460	LOC_Os12g18880	8.89433648
LOC_Os04g32010	LOC_Os05g06450	9.32629192
LOC_Os04g32460	LOC_Os07g12200	9.29295117
LOC_Os04g32010	LOC_Os06g45100	9.44028059
LOC_Os04g31960	LOC_Os06g11800	9.19821937
LOC_Os04g29550	LOC_Os06g45100	8.87059211
LOC_Os04g30420	LOC_Os07g05940	8.8864238
LOC_Os04g32650	LOC_Os04g36800	12.1242859
LOC_Os04g32460	LOC_Os10g21342	8.80263928
LOC_Os04g32460	LOC_Os06g25010	8.81025009
LOC_Os04g32650	LOC_Os08g42560	12.1521235
LOC_Os04g32460	LOC_Os10g39170	9.68878846
LOC_Os04g32460	LOC_Os10g38150	9.06328816
LOC_Os04g30800	LOC_Os07g08660	8.98370844
LOC_Os04g32460	LOC_Os08g14760	9.90401037
LOC_Os04g32460	LOC_Os06g27770	8.85722463
LOC_Os04g30420	LOC_Os06g35700	11.039474
LOC_Os04g32010	LOC_Os06g11240	8.9769887

InteractorA	InteractorB	Z score
LOC_Os04g29550	LOC_Os12g10720	9.99115533
LOC_Os04g31070	LOC_Os05g11550	11.0971935
LOC_Os04g32710	LOC_Os04g55960	8.94364003
LOC_Os04g32650	LOC_Os12g07980	9.11960274
LOC_Os04g30420	LOC_Os06g07978	8.9395965
LOC_Os04g30420	LOC_Os08g23110	8.80168902
LOC_Os04g30420	LOC_Os11g10520	9.82131584
LOC_Os04g30420	LOC_Os05g46580	9.17092933
LOC_Os04g30420	LOC_Os10g21240	9.88266846
LOC_Os04g29550	LOC_Os08g39870	8.85235221
LOC_Os04g32010	LOC_Os11g43360	10.1479911
LOC_Os04g31960	LOC_Os11g26850	8.9375072
LOC_Os04g30420	LOC_Os12g25700	8.84198064
LOC_Os04g30420	LOC_Os06g39230	8.88965601
LOC_Os04g32460	LOC_Os10g41510	10.763679
LOC_Os04g30420	LOC_Os07g49120	8.872435
LOC_Os04g31070	LOC_Os05g06300	10.2894767
LOC_Os04g29550	LOC_Os07g05800	9.13089824
LOC_Os04g32460	LOC_Os12g12514	8.86577185
LOC_Os04g32010	LOC_Os08g37790	10.5762678
LOC_Os04g32010	LOC_Os09g08910	9.31944677
LOC_Os04g30420	LOC_Os05g45810	9.85113918
LOC_Os04g32460	LOC_Os08g15276	8.80263928
LOC_Os04g29550	LOC_Os09g39400	9.1417654
LOC_Os04g31960	LOC_Os07g37550	8.80123867
LOC_Os04g30420	LOC_Os08g14760	9.13851611
LOC_Os04g32460	LOC_Os04g38870	8.88601702
LOC_Os04g32650	LOC_Os09g23560	9.22679529
LOC_Os04g32710	LOC_Os04g52100	10.0639665
LOC_Os04g32710	LOC_Os04g40290	9.40030807
LOC_Os04g30420	LOC_Os10g38140	9.48887579
LOC_Os04g32650	LOC_Os12g31370	9.89878264
LOC_Os04g32010	LOC_Os07g04240	10.4384834
LOC_Os04g29550	LOC_Os10g38700	8.87798167
LOC_Os04g32460	LOC_Os10g08670	8.9844792
LOC_Os04g31960	LOC_Os05g47545	8.92050659
LOC_Os04g30420	LOC_Os11g47550	9.03655173
LOC_Os04g32460	LOC_Os07g26150	9.20632891
LOC_Os04g31070	LOC_Os09g24924	9.22075808
LOC_Os04g32460	LOC_Os04g53810	8.86683739
LOC_Os04g32460	LOC_Os06g47600	8.94041151
LOC_Os04g31960	LOC_Os06g23780	9.323784
LOC_Os04g32010	LOC_Os09g26380	9.41589907

InteractorA	InteractorB	Z score
LOC_Os04g32460	LOC_Os04g39880	10.8043799
LOC_Os04g32650	LOC_Os05g19380	8.93484034
LOC_Os04g30420	LOC_Os06g33210	9.63198697
LOC_Os04g32460	LOC_Os04g53920	9.46591678
LOC_Os04g30800	LOC_Os06g01850	9.99838641
LOC_Os04g32010	LOC_Os05g47640	8.81350889
LOC_Os04g32650	LOC_Os05g46580	8.83575177
LOC_Os04g30800	LOC_Os04g48850	10.022149
LOC_Os04g32650	LOC_Os06g35560	8.93384512
LOC_Os04g30420	LOC_Os06g07878	8.93957775
LOC_Os04g29550	LOC_Os07g42960	8.92004676
LOC_Os04g32010	LOC_Os06g01590	10.3369603
LOC_Os04g32010	LOC_Os10g28050	8.82304728
LOC_Os04g32650	LOC_Os05g01810	9.58086736
LOC_Os04g32710	LOC_Os05g19150	8.92556383
LOC_Os04g33190	LOC_Os10g22450	9.27391228
LOC_Os04g33190	LOC_Os12g22680	9.36917637
LOC_Os04g33480	LOC_Os10g26010	11.1786274
LOC_Os04g33480	LOC_Os12g42884	10.8211762
LOC_Os04g33240	LOC_Os04g41960	9.5242316
LOC_Os04g33570	LOC_Os09g26380	9.08520342
LOC_Os04g33240	LOC_Os04g53240	9.67241438
LOC_Os04g33040	LOC_Os08g09940	9.20824418
LOC_Os04g33740	LOC_Os07g34260	8.88618602
LOC_Os04g33740	LOC_Os06g42130	9.9496952
LOC_Os04g33720	LOC_Os05g09440	11.4607954
LOC_Os04g33480	LOC_Os10g41510	9.58538508
LOC_Os04g33470	LOC_Os05g46270	9.51901954
LOC_Os04g33240	LOC_Os05g30410	9.49094093
LOC_Os04g33470	LOC_Os05g43510	9.55997877
LOC_Os04g33480	LOC_Os08g34790	8.93598424
LOC_Os04g33740	LOC_Os05g12180	9.7700447
LOC_Os04g33470	LOC_Os11g08470	8.83743849
LOC_Os04g33040	LOC_Os06g23870	10.447945
LOC_Os04g33740	LOC_Os04g48540	9.15585254
LOC_Os04g33240	LOC_Os05g30530	9.03813885
LOC_Os04g33740	LOC_Os07g31830	8.80123454
LOC_Os04g33040	LOC_Os05g35770	10.351716
LOC_Os04g33480	LOC_Os10g34760	9.30062487
LOC_Os04g33040	LOC_Os05g29880	9.2355462
LOC_Os04g33040	LOC_Os06g35730	9.07593064
LOC_Os04g33240	LOC_Os12g10720	9.1798332
LOC_Os04g33740	LOC_Os04g53800	9.77578067

InteractorA	InteractorB	Z score
LOC_Os04g33040	LOC_Os06g06730	8.81053222
LOC_Os04g33480	LOC_Os06g11280	8.94194675
LOC_Os04g33480	LOC_Os04g58110	10.1707246
LOC_Os04g33040	LOC_Os05g15520	9.1544159
LOC_Os04g33040	LOC_Os07g03377	9.35840763
LOC_Os04g33470	LOC_Os05g11710	9.94419605
LOC_Os04g33720	LOC_Os11g47560	8.95572657
LOC_Os04g33240	LOC_Os06g28550	8.87113168
LOC_Os04g33040	LOC_Os05g11710	9.07593175
LOC_Os04g33040	LOC_Os10g26010	8.86633976
LOC_Os04g32710	LOC_Os10g39880	9.22161451
LOC_Os04g33480	LOC_Os05g41080	9.52288856
LOC_Os04g33570	LOC_Os05g33150	9.3596042
LOC_Os04g33470	LOC_Os07g05400	9.11554094
LOC_Os04g33480	LOC_Os10g42720	9.04052659
LOC_Os04g33240	LOC_Os05g31140	9.47898152
LOC_Os04g33040	LOC_Os07g03467	9.35840763
LOC_Os04g33040	LOC_Os12g03816	10.1518648
LOC_Os04g33470	LOC_Os11g37890	8.89660809
LOC_Os04g33570	LOC_Os06g09910	8.85046153
LOC_Os04g33040	LOC_Os08g28730	9.29819443
LOC_Os04g33720	LOC_Os06g46340	8.92507267
LOC_Os04g33240	LOC_Os07g05800	9.27928888
LOC_Os04g33240	LOC_Os10g38160	9.48061931
LOC_Os04g33040	LOC_Os04g43760	8.91195962
LOC_Os04g33040	LOC_Os04g41960	8.81802445
LOC_Os04g33240	LOC_Os11g18870	9.25766262
LOC_Os04g33480	LOC_Os06g40170	8.81635293
LOC_Os04g33040	LOC_Os06g11240	9.34129453
LOC_Os04g33740	LOC_Os05g19380	9.14847131
LOC_Os04g33480	LOC_Os06g51084	9.94828188
LOC_Os04g33480	LOC_Os04g38600	9.29034738
LOC_Os04g33040	LOC_Os11g28340	9.69910086
LOC_Os04g33040	LOC_Os08g44530	8.80271195
LOC_Os04g33480	LOC_Os10g25930	9.84087733
LOC_Os04g32710	LOC_Os12g05410	9.02478163
LOC_Os04g33480	LOC_Os07g08880	12.0050066
LOC_Os04g33240	LOC_Os11g47560	8.8823641
LOC_Os04g33240	LOC_Os05g35400	9.09146358
LOC_Os04g33470	LOC_Os06g11240	9.76531073
LOC_Os04g33480	LOC_Os06g40940	8.83537147
LOC_Os04g32710	LOC_Os07g26150	9.40784027
LOC_Os04g33240	LOC_Os04g56760	9.49094093

InteractorA	InteractorB	Z score
LOC_Os04g33720	LOC_Os05g35320	9.35156943
LOC_Os04g33480	LOC_Os05g49840	10.3193284
LOC_Os04g33720	LOC_Os09g17620	8.93673727
LOC_Os04g33040	LOC_Os09g36800	9.17162828
LOC_Os04g33470	LOC_Os07g26900	8.95364537
LOC_Os04g33240	LOC_Os04g33470	10.6479845
LOC_Os04g33740	LOC_Os07g26540	9.54812755
LOC_Os04g33470	LOC_Os06g10330	8.85164936
LOC_Os04g33190	LOC_Os10g08580	8.80521765
LOC_Os04g33480	LOC_Os09g21770	8.84869296
LOC_Os04g33480	LOC_Os06g27770	9.49483802
LOC_Os04g33740	LOC_Os05g25850	9.29930688
LOC_Os04g33240	LOC_Os08g34280	10.1272806
LOC_Os04g33470	LOC_Os10g26600	9.17023146
LOC_Os04g33720	LOC_Os10g25140	10.6772386
LOC_Os04g33480	LOC_Os06g36770	10.1159741
LOC_Os04g33040	LOC_Os07g48430	10.0054048
LOC_Os04g33480	LOC_Os05g15520	9.02263841
LOC_Os04g33190	LOC_Os10g42940	10.7274684
LOC_Os04g33740	LOC_Os04g45470	9.62777475
LOC_Os04g32710	LOC_Os11g02130	9.55329248
LOC_Os04g33720	LOC_Os07g44440	8.88413551
LOC_Os04g33240	LOC_Os07g03960	8.81114471
LOC_Os04g33480	LOC_Os10g26600	9.48454676
LOC_Os04g33570	LOC_Os05g38230	8.8230245
LOC_Os04g33720	LOC_Os09g11230	11.4791361
LOC_Os04g33240	LOC_Os11g26910	9.10808084
LOC_Os04g33480	LOC_Os10g26050	9.24885071
LOC_Os04g33720	LOC_Os11g14910	8.90056946
LOC_Os04g33040	LOC_Os05g10780	10.3239088
LOC_Os04g33480	LOC_Os06g37660	9.57882826
LOC_Os04g33480	LOC_Os12g25630	10.6749324
LOC_Os04g33470	LOC_Os10g38234	8.98827503
LOC_Os04g33040	LOC_Os11g02100	10.218513
LOC_Os04g33480	LOC_Os06g44080	9.27418989
LOC_Os04g32710	LOC_Os12g07820	8.94914789
LOC_Os04g33480	LOC_Os10g25950	9.54058441
LOC_Os04g33570	LOC_Os07g40290	8.87257221
LOC_Os04g33240	LOC_Os05g25490	11.1039876
LOC_Os04g33480	LOC_Os10g37060	9.61133708
LOC_Os04g33720	LOC_Os04g46930	8.96997651
LOC_Os04g33570	LOC_Os11g32520	9.85693509
LOC_Os04g33740	LOC_Os05g09500	9.50450677

InteractorA	InteractorB	Z score
LOC_Os04g33190	LOC_Os08g34290	9.2929642
LOC_Os04g33480	LOC_Os07g40580	8.85238059
LOC_Os04g33570	LOC_Os11g03980	9.47504133
LOC_Os04g33190	LOC_Os05g38550	9.42256757
LOC_Os04g33040	LOC_Os07g05940	9.41372986
LOC_Os04g33240	LOC_Os06g51084	8.8915685
LOC_Os04g33740	LOC_Os06g45120	9.03036672
LOC_Os04g33040	LOC_Os07g03288	9.35840763
LOC_Os04g33470	LOC_Os06g35730	9.94431591
LOC_Os04g33720	LOC_Os07g02350	8.89349204
LOC_Os04g33480	LOC_Os07g08500	8.9339713
LOC_Os04g33570	LOC_Os07g30200	9.74295773
LOC_Os04g33040	LOC_Os09g08072	9.30380765
LOC_Os04g33240	LOC_Os10g33800	9.41887299
LOC_Os04g33480	LOC_Os08g43560	11.4475535
LOC_Os04g33240	LOC_Os08g31870	9.02329142
LOC_Os04g33740	LOC_Os05g08100	9.33434567
LOC_Os04g33480	LOC_Os06g05250	8.83009014
LOC_Os04g33480	LOC_Os09g25320	9.2741833
LOC_Os04g33720	LOC_Os10g33900	9.3033368
LOC_Os04g33190	LOC_Os06g21570	8.90832188
LOC_Os04g33470	LOC_Os05g47980	9.30832482
LOC_Os04g33480	LOC_Os05g02310	9.26114159
LOC_Os04g33040	LOC_Os07g03590	9.35840763
LOC_Os04g32710	LOC_Os09g12570	9.6588214
LOC_Os04g33480	LOC_Os12g08280	8.84616919
LOC_Os04g33740	LOC_Os08g14570	9.12969463
LOC_Os04g33040	LOC_Os07g42600	9.0440392
LOC_Os04g33240	LOC_Os04g52440	9.3711978
LOC_Os04g33720	LOC_Os10g25950	9.39034264
LOC_Os04g33720	LOC_Os09g23550	8.83805127
LOC_Os04g33240	LOC_Os07g05160	9.45939833
LOC_Os04g33480	LOC_Os07g43390	10.2356047
LOC_Os04g32710	LOC_Os07g48160	9.16688869
LOC_Os04g33970	LOC_Os06g04270	9.83350001
LOC_Os04g35200	LOC_Os04g57090	9.20101592
LOC_Os04g33740	LOC_Os09g28770	9.42145486
LOC_Os04g35240	LOC_Os11g32610	10.1836937
LOC_Os04g33970	LOC_Os05g33410	9.8901152
LOC_Os04g37460	LOC_Os05g35320	10.2205418
LOC_Os04g36800	LOC_Os08g28820	9.55735495
LOC_Os04g35570	LOC_Os10g38234	9.48892149
LOC_Os04g37460	LOC_Os10g38600	9.26663501

InteractorA	InteractorB	Z score
LOC_Os04g36800	LOC_Os08g04180	9.04359406
LOC_Os04g35200	LOC_Os08g39870	8.80635142
LOC_Os04g35200	LOC_Os06g12990	9.02067084
LOC_Os04g35570	LOC_Os05g41640	8.87755722
LOC_Os04g36700	LOC_Os04g39900	11.1479699
LOC_Os04g35240	LOC_Os08g09210	8.92738984
LOC_Os04g34630	LOC_Os06g23780	9.34919717
LOC_Os04g35200	LOC_Os04g56400	8.99581398
LOC_Os04g33970	LOC_Os06g08600	9.48242947
LOC_Os04g35200	LOC_Os08g28190	9.68418801
LOC_Os04g36700	LOC_Os12g25710	10.2063176
LOC_Os04g36800	LOC_Os06g23870	9.06310027
LOC_Os04g33970	LOC_Os10g40730	9.14621679
LOC_Os04g37460	LOC_Os10g39120	8.93476753
LOC_Os04g35240	LOC_Os05g46000	9.33935872
LOC_Os04g33740	LOC_Os11g07020	8.96674561
LOC_Os04g36700	LOC_Os09g39570	9.05886018
LOC_Os04g34630	LOC_Os05g46040	9.69122813
LOC_Os04g37460	LOC_Os09g15420	9.48721545
LOC_Os04g36800	LOC_Os12g16220	9.55673171
LOC_Os04g35200	LOC_Os09g08120	10.4870245
LOC_Os04g35570	LOC_Os10g38600	9.26978675
LOC_Os04g35570	LOC_Os08g15292	9.23825686
LOC_Os04g36800	LOC_Os04g56760	8.97223153
LOC_Os04g37460	LOC_Os07g07240	9.7466057
LOC_Os04g33740	LOC_Os10g21192	8.86204007
LOC_Os04g36800	LOC_Os11g34450	9.1255565
LOC_Os04g36700	LOC_Os06g11210	10.3795521
LOC_Os04g33740	LOC_Os10g39120	9.2400347
LOC_Os04g36800	LOC_Os11g03290	9.46605504
LOC_Os04g35200	LOC_Os05g36010	10.4862902
LOC_Os04g36700	LOC_Os11g07440	9.12928996
LOC_Os04g33970	LOC_Os07g11440	8.97481309
LOC_Os04g33740	LOC_Os10g08022	9.63569844
LOC_Os04g36800	LOC_Os12g22030	10.2672675
LOC_Os04g36700	LOC_Os05g04340	10.6718151
LOC_Os04g35240	LOC_Os10g30840	9.87986335
LOC_Os04g36700	LOC_Os06g15420	8.86657316
LOC_Os04g35200	LOC_Os08g09250	8.81281599
LOC_Os04g33970	LOC_Os07g02340	10.4860573
LOC_Os04g34630	LOC_Os07g42940	8.965939
LOC_Os04g36800	LOC_Os07g02350	8.95188756
LOC_Os04g34630	LOC_Os08g32620	9.75629795

InteractorA	InteractorB	Z score
LOC_Os04g36800	LOC_Os04g44950	8.87622675
LOC_Os04g37460	LOC_Os07g30200	8.84810816
LOC_Os04g35240	LOC_Os07g47290	10.4402564
LOC_Os04g37460	LOC_Os04g37640	8.87180796
LOC_Os04g36800	LOC_Os08g42560	9.94049524
LOC_Os04g33740	LOC_Os10g26390	9.98971453
LOC_Os04g35200	LOC_Os05g01600	10.0306021
LOC_Os04g35570	LOC_Os06g40170	8.80815553
LOC_Os04g33970	LOC_Os04g56160	9.92895354
LOC_Os04g36700	LOC_Os05g10780	9.28484577
LOC_Os04g33740	LOC_Os12g05590	10.2609238
LOC_Os04g33740	LOC_Os09g39570	8.99964221
LOC_Os04g33970	LOC_Os06g35520	9.1110488
LOC_Os04g35200	LOC_Os12g07980	9.1037766
LOC_Os04g33970	LOC_Os10g07616	11.6571188
LOC_Os04g35200	LOC_Os09g08072	9.28886825
LOC_Os04g37460	LOC_Os10g38234	10.4986714
LOC_Os04g33970	LOC_Os04g55850	9.05308667
LOC_Os04g33970	LOC_Os10g01080	8.86293163
LOC_Os04g36800	LOC_Os05g30410	8.97220108
LOC_Os04g34630	LOC_Os12g17910	9.40016545
LOC_Os04g33970	LOC_Os06g36700	9.30106901
LOC_Os04g33740	LOC_Os10g21324	9.10258883
LOC_Os04g36700	LOC_Os10g40700	10.1268125
LOC_Os04g35200	LOC_Os07g46460	10.0597633
LOC_Os04g33970	LOC_Os11g28340	9.17260122
LOC_Os04g34630	LOC_Os10g35110	8.82625847
LOC_Os04g35240	LOC_Os07g43260	9.17975719
LOC_Os04g33970	LOC_Os10g26110	9.17234298
LOC_Os04g33970	LOC_Os04g39880	10.1965289
LOC_Os04g38220	LOC_Os04g48850	9.89841304
LOC_Os04g37820	LOC_Os05g45810	11.6467685
LOC_Os04g37820	LOC_Os07g28280	9.12258904
LOC_Os04g37490	LOC_Os08g14760	10.5193624
LOC_Os04g37820	LOC_Os06g10330	9.56924309
LOC_Os04g37820	LOC_Os05g38560	10.9158967
LOC_Os04g38220	LOC_Os05g35330	9.35840543
LOC_Os04g37480	LOC_Os12g36950	9.45065403
LOC_Os04g37490	LOC_Os07g01780	8.94735908
LOC_Os04g37500	LOC_Os05g43510	9.07233373
LOC_Os04g37950	LOC_Os05g49890	9.12035757
LOC_Os04g37820	LOC_Os07g25024	10.1827624
LOC_Os04g37480	LOC_Os09g08910	9.1233972

InteractorA	InteractorB	Z score
LOC_Os04g37820	LOC_Os07g27790	9.1602516
LOC_Os04g37500	LOC_Os12g14070	8.98159454
LOC_Os04g37640	LOC_Os07g44740	8.85147464
LOC_Os04g37490	LOC_Os04g56580	8.97896809
LOC_Os04g37480	LOC_Os05g30480	9.01883088
LOC_Os04g37820	LOC_Os09g19954	9.05475626
LOC_Os04g37490	LOC_Os06g35520	9.20414362
LOC_Os04g37480	LOC_Os05g04690	9.63921303
LOC_Os04g38600	LOC_Os04g45290	9.43293743
LOC_Os04g37480	LOC_Os05g43510	9.44277849
LOC_Os04g37490	LOC_Os11g39540	9.68509962
LOC_Os04g37500	LOC_Os04g40290	9.50374397
LOC_Os04g37820	LOC_Os10g38730	11.0061838
LOC_Os04g37950	LOC_Os07g07470	9.50667005
LOC_Os04g37480	LOC_Os05g44760	9.31067834
LOC_Os04g37500	LOC_Os08g04540	13.7573398
LOC_Os04g37460	LOC_Os12g13390	9.6605222
LOC_Os04g37480	LOC_Os10g38730	9.51304033
LOC_Os04g38220	LOC_Os05g35320	9.04322693
LOC_Os04g37820	LOC_Os07g38540	8.89397865
LOC_Os04g37500	LOC_Os06g45590	9.69523883
LOC_Os04g38220	LOC_Os05g10780	9.61549104
LOC_Os04g37990	LOC_Os11g01360	9.44595316
LOC_Os04g37480	LOC_Os04g43400	8.93044232
LOC_Os04g37950	LOC_Os04g53230	9.18282244
LOC_Os04g37480	LOC_Os06g11210	9.86753483
LOC_Os04g37480	LOC_Os11g37950	9.34143307
LOC_Os04g37640	LOC_Os08g10608	9.75283135
LOC_Os04g37990	LOC_Os05g11710	9.11203812
LOC_Os04g37820	LOC_Os09g24412	10.182756
LOC_Os04g37480	LOC_Os04g39840	9.68914182
LOC_Os04g37480	LOC_Os07g48040	9.48042962
LOC_Os04g37950	LOC_Os12g38770	9.78796086
LOC_Os04g37640	LOC_Os05g46270	9.2291443
LOC_Os04g37950	LOC_Os09g21770	9.61056982
LOC_Os04g37820	LOC_Os10g21352	10.1827726
LOC_Os04g37500	LOC_Os05g35290	9.15576903
LOC_Os04g37480	LOC_Os11g03980	9.94900257
LOC_Os04g37820	LOC_Os08g01660	9.06229455
LOC_Os04g37820	LOC_Os12g31640	9.65162579
LOC_Os04g37640	LOC_Os07g46460	9.29678918
LOC_Os04g37990	LOC_Os06g35730	9.11207901
LOC_Os04g37950	LOC_Os07g30970	9.53514075

InteractorA	InteractorB	Z score
LOC_Os04g37820	LOC_Os12g22030	9.25704235
LOC_Os04g37820	LOC_Os07g48030	8.80968483
LOC_Os04g37640	LOC_Os12g43370	9.07720487
LOC_Os04g37490	LOC_Os04g55740	9.24961448
LOC_Os04g37950	LOC_Os05g15520	9.21891565
LOC_Os04g37490	LOC_Os08g44270	9.20053547
LOC_Os04g37950	LOC_Os04g56160	8.96998056
LOC_Os04g37480	LOC_Os06g05690	8.86005239
LOC_Os04g37950	LOC_Os12g44020	8.8365005
LOC_Os04g37500	LOC_Os04g38870	11.4606197
LOC_Os04g37480	LOC_Os05g36270	9.48096623
LOC_Os04g37490	LOC_Os09g25370	8.91964014
LOC_Os04g37490	LOC_Os07g02210	8.95960683
LOC_Os04g37640	LOC_Os10g21324	9.07180094
LOC_Os04g38220	LOC_Os08g02410	9.3391151
LOC_Os04g37500	LOC_Os09g38620	9.06576773
LOC_Os04g37480	LOC_Os08g23730	9.10010479
LOC_Os04g37820	LOC_Os07g44790	8.96388726
LOC_Os04g37490	LOC_Os05g41900	9.54076502
LOC_Os04g37640	LOC_Os04g40990	9.51426452
LOC_Os04g37500	LOC_Os05g41060	8.83515151
LOC_Os04g37480	LOC_Os08g42910	9.26419764
LOC_Os04g38220	LOC_Os10g21268	9.35840543
LOC_Os04g37820	LOC_Os05g48510	8.89045718
LOC_Os04g37950	LOC_Os12g24650	8.82002892
LOC_Os04g37480	LOC_Os04g39864	10.9561226
LOC_Os04g37820	LOC_Os10g39120	10.9661844
LOC_Os04g37820	LOC_Os04g52280	9.27345231
LOC_Os04g37500	LOC_Os05g11730	9.56112429
LOC_Os04g37640	LOC_Os12g23630	9.5942656
LOC_Os04g37490	LOC_Os09g23560	8.84906666
LOC_Os04g37950	LOC_Os11g32260	9.19573821
LOC_Os04g37490	LOC_Os09g23530	10.5334469
LOC_Os04g37950	LOC_Os08g37800	11.9154248
LOC_Os04g37640	LOC_Os11g08445	9.08991173
LOC_Os04g37820	LOC_Os10g38470	9.52539976
LOC_Os04g37820	LOC_Os08g15266	10.1827624
LOC_Os04g37950	LOC_Os05g44760	10.2393345
LOC_Os04g37820	LOC_Os12g40510	9.77380274
LOC_Os04g37480	LOC_Os08g44350	9.68772445
LOC_Os04g37490	LOC_Os04g48540	8.96270779
LOC_Os04g37820	LOC_Os06g35590	9.31090041
LOC_Os04g37820	LOC_Os05g50710	9.67697403

InteractorA	InteractorB	Z score
LOC_Os04g37820	LOC_Os12g36950	10.4567758
LOC_Os04g37640	LOC_Os06g45710	10.4067942
LOC_Os04g37490	LOC_Os07g07060	9.12145852
LOC_Os04g37480	LOC_Os04g39880	9.18851634
LOC_Os04g37480	LOC_Os08g20730	9.893147
LOC_Os04g37480	LOC_Os09g31430	8.90684399
LOC_Os04g37990	LOC_Os05g49830	9.8358114
LOC_Os04g37480	LOC_Os07g43260	9.9334118
LOC_Os04g37640	LOC_Os11g16590	9.90699831
LOC_Os04g37480	LOC_Os04g44890	9.79620277
LOC_Os04g37480	LOC_Os09g15420	9.98022632
LOC_Os04g39020	LOC_Os12g02080	10.0014048
LOC_Os04g39814	LOC_Os06g06300	9.89293668
LOC_Os04g38940	LOC_Os12g34380	9.27782654
LOC_Os04g39030	LOC_Os08g40170	8.8547581
LOC_Os04g39210	LOC_Os06g48180	9.10577626
LOC_Os04g39210	LOC_Os09g27820	8.85051037
LOC_Os04g38870	LOC_Os04g47170	8.90872852
LOC_Os04g39210	LOC_Os08g03440	8.95058391
LOC_Os04g38870	LOC_Os08g44340	9.08992339
LOC_Os04g38870	LOC_Os08g31060	9.69314639
LOC_Os04g39814	LOC_Os09g35800	12.3979847
LOC_Os04g39840	LOC_Os06g45070	8.80998092
LOC_Os04g39210	LOC_Os04g47170	10.2246147
LOC_Os04g39840	LOC_Os07g08170	9.10698813
LOC_Os04g38600	LOC_Os11g01360	8.96656319
LOC_Os04g38600	LOC_Os08g39870	9.54955606
LOC_Os04g38600	LOC_Os07g14590	8.96595622
LOC_Os04g38940	LOC_Os05g06300	9.54530942
LOC_Os04g38940	LOC_Os06g40180	10.4311967
LOC_Os04g39840	LOC_Os05g06450	9.95360417
LOC_Os04g38600	LOC_Os07g07320	10.8969982
LOC_Os04g39020	LOC_Os10g39840	8.85404736
LOC_Os04g39814	LOC_Os07g05400	10.136074
LOC_Os04g39840	LOC_Os05g05830	8.93604719
LOC_Os04g38870	LOC_Os05g49830	8.85659958
LOC_Os04g38940	LOC_Os04g42250	8.93065662
LOC_Os04g39210	LOC_Os10g41480	8.94188468
LOC_Os04g38600	LOC_Os10g11260	8.80999951
LOC_Os04g39020	LOC_Os08g20730	10.9743362
LOC_Os04g38870	LOC_Os04g40290	9.71637325
LOC_Os04g39814	LOC_Os07g05150	9.46833695
LOC_Os04g39020	LOC_Os05g04690	9.44020518

InteractorA	InteractorB	Z score
LOC_Os04g39210	LOC_Os07g44790	9.82110183
LOC_Os04g38870	LOC_Os08g17784	8.95725784
LOC_Os04g38940	LOC_Os11g33240	9.29884172
LOC_Os04g38870	LOC_Os04g44730	8.84742786
LOC_Os04g39440	LOC_Os10g38690	10.2591987
LOC_Os04g38870	LOC_Os07g05800	8.8381724
LOC_Os04g39840	LOC_Os04g52280	9.24897975
LOC_Os04g39840	LOC_Os07g07550	9.65153891
LOC_Os04g39440	LOC_Os10g26110	10.3753093
LOC_Os04g38870	LOC_Os11g03230	10.8998367
LOC_Os04g39030	LOC_Os04g40874	9.19167324
LOC_Os04g39020	LOC_Os07g08030	9.34678035
LOC_Os04g39210	LOC_Os12g08280	10.1151644
LOC_Os04g38870	LOC_Os10g07616	11.4541535
LOC_Os04g39440	LOC_Os11g25700	8.89999508
LOC_Os04g39814	LOC_Os05g28180	8.99991772
LOC_Os04g38940	LOC_Os12g05410	8.8643755
LOC_Os04g39030	LOC_Os08g33820	9.30378631
LOC_Os04g38870	LOC_Os08g09210	8.84087537
LOC_Os04g39030	LOC_Os10g27190	9.5823644
LOC_Os04g39210	LOC_Os06g50300	11.9157699
LOC_Os04g38870	LOC_Os06g35650	9.0063973
LOC_Os04g38600	LOC_Os05g40420	8.92485314
LOC_Os04g39210	LOC_Os10g11810	8.99780276
LOC_Os04g39210	LOC_Os04g53810	9.27468313
LOC_Os04g39030	LOC_Os12g22650	8.96842329
LOC_Os04g39440	LOC_Os06g42130	9.71751372
LOC_Os04g38600	LOC_Os08g03290	14.7819853
LOC_Os04g38600	LOC_Os05g49830	10.3878049
LOC_Os04g38870	LOC_Os08g04560	8.92151511
LOC_Os04g39210	LOC_Os05g08960	9.29910165
LOC_Os04g39030	LOC_Os07g42924	9.15263376
LOC_Os04g39840	LOC_Os05g46360	9.06131543
LOC_Os04g38870	LOC_Os08g28800	9.28671089
LOC_Os04g39210	LOC_Os04g56070	9.09631221
LOC_Os04g39814	LOC_Os10g31000	10.0995186
LOC_Os04g38600	LOC_Os07g41750	8.91346212
LOC_Os04g38600	LOC_Os08g44370	9.59317391
LOC_Os04g39440	LOC_Os04g48850	9.29832503
LOC_Os04g38600	LOC_Os06g04270	9.86897265
LOC_Os04g39210	LOC_Os12g07820	10.5663354
LOC_Os04g39030	LOC_Os05g36010	9.46022919
LOC_Os04g38600	LOC_Os09g36800	8.84980285

InteractorA	InteractorB	Z score
LOC_Os04g38600	LOC_Os10g08710	9.5735291
LOC_Os04g39210	LOC_Os07g48880	9.59073023
LOC_Os04g38600	LOC_Os07g46460	10.5707083
LOC_Os04g38600	LOC_Os09g08072	10.0839965
LOC_Os04g39840	LOC_Os04g48850	9.37069507
LOC_Os04g38870	LOC_Os06g06880	8.92662668
LOC_Os04g39440	LOC_Os09g30412	9.77738463
LOC_Os04g38870	LOC_Os12g17910	9.76241607
LOC_Os04g39020	LOC_Os05g44140	8.83694169
LOC_Os04g38940	LOC_Os05g12190	9.25222581
LOC_Os04g39030	LOC_Os08g31060	8.83648507
LOC_Os04g38870	LOC_Os07g07060	9.44632944
LOC_Os04g39840	LOC_Os07g09890	10.4828524
LOC_Os04g39210	LOC_Os08g01660	9.06386642
LOC_Os04g38870	LOC_Os07g16970	9.40938231
LOC_Os04g38870	LOC_Os05g31140	8.8250562
LOC_Os04g38870	LOC_Os09g10230	10.3245168
LOC_Os04g39814	LOC_Os06g35650	8.93802444
LOC_Os04g39840	LOC_Os05g31040	9.37231056
LOC_Os04g39020	LOC_Os08g28190	8.87609842
LOC_Os04g38870	LOC_Os11g10510	9.2316936
LOC_Os04g39840	LOC_Os07g30990	8.98475153
LOC_Os04g39030	LOC_Os05g41080	8.9205989
LOC_Os04g39210	LOC_Os04g58680	9.83184647
LOC_Os04g39210	LOC_Os08g04560	10.0635116
LOC_Os04g39814	LOC_Os09g08072	9.53750583
LOC_Os04g38600	LOC_Os11g36719	9.51250554
LOC_Os04g38870	LOC_Os12g12514	11.7909965
LOC_Os04g39840	LOC_Os04g56210	8.90439349
LOC_Os04g39840	LOC_Os07g03368	9.39821753
LOC_Os04g40310	LOC_Os04g59160	10.1446527
LOC_Os04g40290	LOC_Os04g41260	8.87848594
LOC_Os04g39880	LOC_Os10g26600	8.91366943
LOC_Os04g39840	LOC_Os09g32640	9.5872636
LOC_Os04g40310	LOC_Os11g29400	11.1238933
LOC_Os04g40310	LOC_Os04g43400	9.54495579
LOC_Os04g39900	LOC_Os05g37390	9.2440119
LOC_Os04g40290	LOC_Os08g20730	9.35600983
LOC_Os04g40130	LOC_Os07g44790	10.5296573
LOC_Os04g39840	LOC_Os08g32850	8.80966089
LOC_Os04g39840	LOC_Os10g41510	9.23651114
LOC_Os04g39900	LOC_Os06g29180	9.57809816
LOC_Os04g40310	LOC_Os05g49760	9.87271486

InteractorA	InteractorB	Z score
LOC_Os04g39900	LOC_Os06g33210	8.84650691
LOC_Os04g39864	LOC_Os06g19960	9.53312278
LOC_Os04g39864	LOC_Os09g33500	8.97004412
LOC_Os04g40620	LOC_Os06g36770	8.8534984
LOC_Os04g40310	LOC_Os07g44790	9.00384069
LOC_Os04g40130	LOC_Os09g08910	9.67319522
LOC_Os04g39900	LOC_Os07g07060	9.61894955
LOC_Os04g39864	LOC_Os10g29470	9.09330603
LOC_Os04g40130	LOC_Os07g38430	9.16100872
LOC_Os04g40130	LOC_Os07g42940	10.2819999
LOC_Os04g39840	LOC_Os10g41480	10.6537096
LOC_Os04g40310	LOC_Os06g43640	10.7274433
LOC_Os04g39880	LOC_Os11g14040	10.2154025
LOC_Os04g39880	LOC_Os07g49400	10.1704311
LOC_Os04g39864	LOC_Os07g49120	10.1702061
LOC_Os04g40130	LOC_Os07g34260	9.53241011
LOC_Os04g40130	LOC_Os04g56070	9.50919576
LOC_Os04g40290	LOC_Os06g42130	12.2838665
LOC_Os04g39900	LOC_Os12g06620	9.27228029
LOC_Os04g40310	LOC_Os06g06300	9.2104953
LOC_Os04g40130	LOC_Os07g41750	10.1293486
LOC_Os04g39880	LOC_Os08g36910	8.90669307
LOC_Os04g40130	LOC_Os06g07878	10.2445703
LOC_Os04g40130	LOC_Os10g23100	9.04693433
LOC_Os04g39864	LOC_Os05g45590	9.55623057
LOC_Os04g40310	LOC_Os10g39170	9.16622398
LOC_Os04g40130	LOC_Os09g36710	9.62536084
LOC_Os04g39880	LOC_Os10g38150	9.04484768
LOC_Os04g40290	LOC_Os04g53214	8.95595665
LOC_Os04g39880	LOC_Os08g28800	10.2200151
LOC_Os04g40130	LOC_Os09g33860	9.40009572
LOC_Os04g39900	LOC_Os08g20270	9.47520508
LOC_Os04g40310	LOC_Os06g45100	11.7668882
LOC_Os04g39900	LOC_Os09g25320	9.28295195
LOC_Os04g39864	LOC_Os12g21798	10.3482807
LOC_Os04g39900	LOC_Os05g49890	10.1867174
LOC_Os04g40310	LOC_Os05g38310	9.53612417
LOC_Os04g39864	LOC_Os05g01600	9.30532158
LOC_Os04g40130	LOC_Os10g29470	8.93438537
LOC_Os04g39880	LOC_Os06g37150	11.290936
LOC_Os04g40310	LOC_Os11g32540	8.85256414
LOC_Os04g39880	LOC_Os04g55410	9.41854738
LOC_Os04g40310	LOC_Os11g26910	9.41981546

InteractorA	InteractorB	Z score
LOC_Os04g39900	LOC_Os10g17650	9.04397264
LOC_Os04g39864	LOC_Os09g31486	10.2737911
LOC_Os04g39864	LOC_Os05g11730	9.19771715
LOC_Os04g39900	LOC_Os10g27174	9.04338236
LOC_Os04g40310	LOC_Os07g27790	9.39923755
LOC_Os04g39864	LOC_Os10g38600	9.34379105
LOC_Os04g39900	LOC_Os04g58200	9.14794462
LOC_Os04g39864	LOC_Os07g42600	9.89714433
LOC_Os04g40310	LOC_Os11g10510	9.30136176
LOC_Os04g39900	LOC_Os12g10730	9.83877688
LOC_Os04g40290	LOC_Os04g40990	8.90016049
LOC_Os04g39880	LOC_Os10g28350	9.37750232
LOC_Os04g40130	LOC_Os06g02490	9.14081992
LOC_Os04g39880	LOC_Os05g31040	9.72523417
LOC_Os04g40310	LOC_Os05g42150	10.2965144
LOC_Os04g39864	LOC_Os10g25130	11.2570919
LOC_Os04g39880	LOC_Os09g07510	8.84946554
LOC_Os04g39840	LOC_Os08g20420	9.17208133
LOC_Os04g39900	LOC_Os10g27190	9.30729532
LOC_Os04g39880	LOC_Os11g08470	8.84792576
LOC_Os04g40130	LOC_Os04g59600	9.0176486
LOC_Os04g40130	LOC_Os10g38340	9.82268024
LOC_Os04g40130	LOC_Os09g12230	9.31693888
LOC_Os04g39880	LOC_Os11g19320	8.95804586
LOC_Os04g40290	LOC_Os09g15320	9.49149733
LOC_Os04g39864	LOC_Os12g06620	8.89103141
LOC_Os04g40130	LOC_Os06g07978	10.2445372
LOC_Os04g39840	LOC_Os09g31502	8.87205257
LOC_Os04g39900	LOC_Os10g29620	8.87998039
LOC_Os04g39900	LOC_Os11g03290	9.5245628
LOC_Os04g40290	LOC_Os04g44870	9.76250806
LOC_Os04g40310	LOC_Os05g51480	9.20099098
LOC_Os04g39900	LOC_Os12g10720	8.93784419
LOC_Os04g39900	LOC_Os04g44924	9.09998662
LOC_Os04g40310	LOC_Os08g08500	8.84113731
LOC_Os04g39880	LOC_Os06g10340	9.37133341
LOC_Os04g39864	LOC_Os11g03230	9.19710532
LOC_Os04g40310	LOC_Os08g41880	8.94794481
LOC_Os04g39880	LOC_Os06g28550	9.40653438
LOC_Os04g39900	LOC_Os04g45290	10.9258073
LOC_Os04g39864	LOC_Os05g45420	9.46031687
LOC_Os04g39900	LOC_Os05g03480	9.14603535
LOC_Os04g40310	LOC_Os12g43450	9.36190221

InteractorA	InteractorB	Z score
LOC_Os04g40310	LOC_Os12g12590	10.6046135
LOC_Os04g39864	LOC_Os06g35590	9.71958077
LOC_Os04g39880	LOC_Os05g49890	8.93990962
LOC_Os04g39840	LOC_Os10g08670	10.0169291
LOC_Os04g40130	LOC_Os10g17660	9.74084443
LOC_Os04g39864	LOC_Os08g04540	10.927238
LOC_Os04g40290	LOC_Os07g12730	9.03403147
LOC_Os04g40130	LOC_Os12g35570	8.91307085
LOC_Os04g40290	LOC_Os11g39540	9.3602056
LOC_Os04g39880	LOC_Os12g43630	10.1471415
LOC_Os04g39900	LOC_Os05g05830	11.9709309
LOC_Os04g40130	LOC_Os10g38610	9.7439925
LOC_Os04g39864	LOC_Os05g50710	9.41722386
LOC_Os04g40290	LOC_Os05g35290	8.91966273
LOC_Os04g40130	LOC_Os06g35660	9.18432651
LOC_Os04g39864	LOC_Os05g19380	9.31597046
LOC_Os04g39840	LOC_Os08g20270	10.8518029
LOC_Os04g39900	LOC_Os05g47980	8.8219892
LOC_Os04g39900	LOC_Os06g44080	9.28295195
LOC_Os04g40130	LOC_Os04g57380	9.64399603
LOC_Os04g40130	LOC_Os09g30418	9.23345001
LOC_Os04g40290	LOC_Os04g44730	8.86648391
LOC_Os04g40130	LOC_Os12g22650	8.99478474
LOC_Os04g39864	LOC_Os10g38140	9.65687921
LOC_Os04g39900	LOC_Os06g11200	9.45645893
LOC_Os04g40290	LOC_Os11g08120	8.9848043
LOC_Os04g40130	LOC_Os06g45100	10.0546337
LOC_Os04g40130	LOC_Os10g17680	9.74084443
LOC_Os04g39840	LOC_Os12g17540	9.45450323
LOC_Os04g39900	LOC_Os08g08500	9.02273761
LOC_Os04g39880	LOC_Os06g07580	9.58060739
LOC_Os04g39880	LOC_Os09g31120	9.257327
LOC_Os04g40290	LOC_Os06g36880	10.3127105
LOC_Os04g41960	LOC_Os06g14510	9.17529814
LOC_Os04g40990	LOC_Os05g30480	11.8515559
LOC_Os04g40990	LOC_Os06g04620	9.32613668
LOC_Os04g40874	LOC_Os05g06750	9.56764823
LOC_Os04g41310	LOC_Os11g31530	9.78293294
LOC_Os04g41620	LOC_Os08g40140	11.3017966
LOC_Os04g41960	LOC_Os09g24412	8.82637033
LOC_Os04g41310	LOC_Os09g15790	8.94668315
LOC_Os04g40874	LOC_Os07g48020	10.0607581
LOC_Os04g41310	LOC_Os05g20050	9.34212233

InteractorA	InteractorB	Z score
LOC_Os04g41960	LOC_Os06g35630	10.3512043
LOC_Os04g41960	LOC_Os04g52100	9.88935064
LOC_Os04g41310	LOC_Os05g25490	8.80117396
LOC_Os04g41960	LOC_Os10g21352	8.82637033
LOC_Os04g40990	LOC_Os11g14040	8.89673864
LOC_Os04g40990	LOC_Os11g05570	8.81780337
LOC_Os04g40874	LOC_Os09g24530	9.36852017
LOC_Os04g40990	LOC_Os08g09250	9.02365074
LOC_Os04g41620	LOC_Os09g15420	9.4227191
LOC_Os04g40990	LOC_Os09g31410	9.4659093
LOC_Os04g42250	LOC_Os05g37690	9.4458727
LOC_Os04g41960	LOC_Os07g35880	10.388196
LOC_Os04g41310	LOC_Os09g31430	8.86890181
LOC_Os04g41130	LOC_Os05g01600	9.46791056
LOC_Os04g40874	LOC_Os11g01872	9.2945884
LOC_Os04g40990	LOC_Os06g15990	10.0330128
LOC_Os04g41310	LOC_Os10g28050	10.8479917
LOC_Os04g40950	LOC_Os08g06550	9.12680577
LOC_Os04g41620	LOC_Os12g40510	9.29605987
LOC_Os04g41620	LOC_Os07g49400	8.86009258
LOC_Os04g40990	LOC_Os11g41130	8.86571207
LOC_Os04g40874	LOC_Os06g30370	9.11144565
LOC_Os04g40874	LOC_Os12g07720	8.82181467
LOC_Os04g40950	LOC_Os05g28180	8.97249359
LOC_Os04g41620	LOC_Os05g04490	11.1715862
LOC_Os04g41130	LOC_Os10g40720	9.04387366
LOC_Os04g41960	LOC_Os09g25370	9.72235922
LOC_Os04g41960	LOC_Os09g21770	9.43005863
LOC_Os04g41620	LOC_Os11g34450	9.44714781
LOC_Os04g41960	LOC_Os12g34062	8.81209533
LOC_Os04g41960	LOC_Os07g02350	9.18110301
LOC_Os04g41310	LOC_Os12g25690	8.98709105
LOC_Os04g40990	LOC_Os06g06040	8.80809188
LOC_Os04g40874	LOC_Os07g36130	10.2069856
LOC_Os04g41260	LOC_Os06g11210	9.82646411
LOC_Os04g41310	LOC_Os12g12580	8.83201834
LOC_Os04g40990	LOC_Os11g32510	9.69337977
LOC_Os04g41960	LOC_Os12g36950	9.26749416
LOC_Os04g41310	LOC_Os10g41410	10.4733865
LOC_Os04g40950	LOC_Os11g03290	9.52482168
LOC_Os04g41960	LOC_Os09g15420	10.1017497
LOC_Os04g41310	LOC_Os11g25330	9.50200832
LOC_Os04g40950	LOC_Os12g43450	10.4478603

**InteractorA**	**InteractorB**	**Z score**
LOC_Os04g40874	LOC_Os09g37949	9.09550108
LOC_Os04g40990	LOC_Os07g17010	9.39541071
LOC_Os04g41960	LOC_Os11g47600	9.44976431
LOC_Os04g41960	LOC_Os10g38140	11.129313
LOC_Os04g40950	LOC_Os06g45120	9.20253474
LOC_Os04g41620	LOC_Os07g01020	8.87842324
LOC_Os04g41310	LOC_Os05g05830	9.7135826
LOC_Os04g41620	LOC_Os04g53920	8.84039403
LOC_Os04g41960	LOC_Os06g37180	9.29344133
LOC_Os04g41260	LOC_Os09g37100	10.1095348
LOC_Os04g41960	LOC_Os04g44920	8.93286552
LOC_Os04g42250	LOC_Os05g04690	8.80693405
LOC_Os04g41960	LOC_Os12g02060	9.23253432
LOC_Os04g41960	LOC_Os04g48850	9.51673259
LOC_Os04g41620	LOC_Os05g23860	9.30417391
LOC_Os04g41620	LOC_Os08g04560	8.89119366
LOC_Os04g41310	LOC_Os10g29620	9.89305281
LOC_Os04g40874	LOC_Os08g04560	9.49715712
LOC_Os04g41620	LOC_Os06g02380	9.03635547
LOC_Os04g40990	LOC_Os05g43510	10.1501614
LOC_Os04g40874	LOC_Os10g38140	9.80243267
LOC_Os04g40950	LOC_Os12g42876	8.8046484
LOC_Os04g41130	LOC_Os05g12180	9.43838636
LOC_Os04g41310	LOC_Os12g08270	8.96514234
LOC_Os04g40874	LOC_Os07g11440	10.0989781
LOC_Os04g40990	LOC_Os12g08280	9.57006699
LOC_Os04g41960	LOC_Os10g29620	8.80958903
LOC_Os04g40874	LOC_Os08g35440	8.89662087
LOC_Os04g41960	LOC_Os04g44470	9.653095
LOC_Os04g40990	LOC_Os04g48850	10.5901837
LOC_Os04g41960	LOC_Os05g33730	9.83247522
LOC_Os04g40990	LOC_Os05g44140	8.83761651
LOC_Os04g41960	LOC_Os08g25734	10.5349121
LOC_Os04g42250	LOC_Os05g19150	8.82873686
LOC_Os04g40990	LOC_Os07g25150	9.0629806
LOC_Os04g41130	LOC_Os06g10340	9.82303714
LOC_Os04g40874	LOC_Os10g08580	8.82639735
LOC_Os04g41310	LOC_Os11g14040	10.0817497
LOC_Os04g41960	LOC_Os06g05880	9.97940182
LOC_Os04g41960	LOC_Os07g25024	8.82637033
LOC_Os04g41310	LOC_Os05g07690	9.01144849
LOC_Os04g40990	LOC_Os04g53240	9.07882642
LOC_Os04g40874	LOC_Os06g06090	8.99684111

InteractorA	InteractorB	Z score
LOC_Os04g41130	LOC_Os07g46460	9.05049668
LOC_Os04g40990	LOC_Os10g42720	9.49981424
LOC_Os04g41960	LOC_Os08g15266	8.82637033
LOC_Os04g41260	LOC_Os05g11550	9.18256822
LOC_Os04g41310	LOC_Os04g52130	8.95761204
LOC_Os04g41960	LOC_Os09g31410	9.56711704
LOC_Os04g40874	LOC_Os05g49880	9.18848635
LOC_Os04g40874	LOC_Os11g33240	8.94058832
LOC_Os04g40874	LOC_Os09g31490	9.76417277
LOC_Os04g40950	LOC_Os05g40990	9.67311898
LOC_Os04g41960	LOC_Os10g02040	8.89939537
LOC_Os04g40874	LOC_Os07g06440	8.99048044
LOC_Os04g41620	LOC_Os04g53850	9.24944645
LOC_Os04g40874	LOC_Os05g33240	8.87847223
LOC_Os04g41620	LOC_Os05g05670	9.23475749
LOC_Os04g42920	LOC_Os12g23170	9.593625
LOC_Os04g42380	LOC_Os07g08660	9.18044729
LOC_Os04g43760	LOC_Os07g30170	9.18832878
LOC_Os04g43760	LOC_Os06g23780	9.35269848
LOC_Os04g42250	LOC_Os12g07050	8.85478104
LOC_Os04g43750	LOC_Os11g32610	10.1400851
LOC_Os04g43410	LOC_Os08g31870	9.81360925
LOC_Os04g43400	LOC_Os07g26630	9.65797552
LOC_Os04g43400	LOC_Os09g08120	10.4384249
LOC_Os04g43750	LOC_Os12g39630	9.31023355
LOC_Os04g43410	LOC_Os07g23730	9.59111589
LOC_Os04g43400	LOC_Os07g46460	9.95686817
LOC_Os04g43410	LOC_Os10g08580	8.99928122
LOC_Os04g43750	LOC_Os06g43640	8.82520798
LOC_Os04g42920	LOC_Os08g37800	10.1100818
LOC_Os04g43410	LOC_Os09g10200	10.8284822
LOC_Os04g43410	LOC_Os05g48510	9.18002844
LOC_Os04g42920	LOC_Os12g34874	9.47465784
LOC_Os04g43800	LOC_Os07g48430	10.8617551
LOC_Os04g43750	LOC_Os07g05800	11.0242453
LOC_Os04g42250	LOC_Os08g14760	9.631958
LOC_Os04g43760	LOC_Os11g10520	9.01149523
LOC_Os04g43750	LOC_Os05g25550	9.22676236
LOC_Os04g43400	LOC_Os08g25570	10.3824972
LOC_Os04g43760	LOC_Os10g31000	8.93555352
LOC_Os04g43410	LOC_Os07g34260	8.99440344
LOC_Os04g42920	LOC_Os12g16240	8.91341452
LOC_Os04g43410	LOC_Os09g24412	12.7496896

InteractorA	InteractorB	Z score
LOC_Os04g43760	LOC_Os07g07060	10.3770529
LOC_Os04g43400	LOC_Os12g02060	9.71327182
LOC_Os04g42380	LOC_Os08g10608	10.4250611
LOC_Os04g42920	LOC_Os10g35070	8.83846563
LOC_Os04g42380	LOC_Os09g20090	10.4746941
LOC_Os04g42920	LOC_Os10g22450	10.6056745
LOC_Os04g43760	LOC_Os05g45810	9.20592421
LOC_Os04g43800	LOC_Os05g35290	10.2578632
LOC_Os04g43410	LOC_Os07g25024	12.7497154
LOC_Os04g43400	LOC_Os07g05800	8.819145
LOC_Os04g43400	LOC_Os05g49770	9.2462593
LOC_Os04g43750	LOC_Os12g34450	9.06661147
LOC_Os04g43410	LOC_Os06g12090	10.1031644
LOC_Os04g42930	LOC_Os05g47890	8.89886555
LOC_Os04g42920	LOC_Os10g01540	9.19344162
LOC_Os04g43760	LOC_Os12g40510	9.60850606
LOC_Os04g42380	LOC_Os06g11800	10.0275354
LOC_Os04g43800	LOC_Os05g09490	9.33950119
LOC_Os04g43410	LOC_Os11g08460	9.27111115
LOC_Os04g43760	LOC_Os07g44550	8.8597095
LOC_Os04g42920	LOC_Os06g06460	9.3590828
LOC_Os04g43800	LOC_Os07g28280	9.19929888
LOC_Os04g43800	LOC_Os07g02350	8.81328248
LOC_Os04g43750	LOC_Os06g41810	9.05581617
LOC_Os04g42920	LOC_Os09g31502	9.97270892
LOC_Os04g43410	LOC_Os08g15266	12.7496559
LOC_Os04g43410	LOC_Os06g35560	8.90832193
LOC_Os04g43750	LOC_Os09g27750	9.48269941
LOC_Os04g42380	LOC_Os04g57590	10.1418325
LOC_Os04g42920	LOC_Os08g02410	9.63836375
LOC_Os04g42930	LOC_Os04g46910	9.16518299
LOC_Os04g43400	LOC_Os11g08445	9.11537506
LOC_Os04g43750	LOC_Os04g56320	9.4916517
LOC_Os04g43800	LOC_Os05g48290	9.66133901
LOC_Os04g43750	LOC_Os07g34589	9.21163388
LOC_Os04g43410	LOC_Os11g30290	8.89005464
LOC_Os04g43400	LOC_Os11g48110	9.39985869
LOC_Os04g43760	LOC_Os11g30290	9.46047461
LOC_Os04g42380	LOC_Os09g08072	9.02759467
LOC_Os04g43760	LOC_Os11g48110	11.153868
LOC_Os04g42920	LOC_Os07g01560	9.13583283
LOC_Os04g43400	LOC_Os08g10608	8.94922086
LOC_Os04g43760	LOC_Os05g01675	9.00385669

InteractorA	InteractorB	Z score
LOC_Os04g43760	LOC_Os05g46360	8.81686401
LOC_Os04g42930	LOC_Os08g20420	10.0581968
LOC_Os04g42920	LOC_Os10g28350	8.8062278
LOC_Os04g43760	LOC_Os06g10930	10.7148623
LOC_Os04g43400	LOC_Os11g26850	9.10241286
LOC_Os04g43750	LOC_Os08g41830	8.88239393
LOC_Os04g42920	LOC_Os05g36280	9.35909521
LOC_Os04g42920	LOC_Os05g49760	15.2974339
LOC_Os04g43410	LOC_Os10g21352	12.7496896
LOC_Os04g43410	LOC_Os12g32240	11.1046348
LOC_Os04g43750	LOC_Os12g22680	12.0501199
LOC_Os04g42920	LOC_Os06g06510	9.3590828
LOC_Os04g43800	LOC_Os06g35660	9.00522804
LOC_Os04g43400	LOC_Os05g49830	9.33868364
LOC_Os04g42930	LOC_Os07g48030	8.8064222
LOC_Os04g43400	LOC_Os07g47990	10.0008043
LOC_Os04g43800	LOC_Os07g11440	9.29267491
LOC_Os04g42380	LOC_Os08g42560	10.1273484
LOC_Os04g43750	LOC_Os07g48030	10.5116881
LOC_Os04g43400	LOC_Os05g05620	9.20036033
LOC_Os04g43800	LOC_Os05g46860	8.94974292
LOC_Os04g43410	LOC_Os06g40170	9.43723573
LOC_Os04g42920	LOC_Os10g26390	10.2942813
LOC_Os04g43760	LOC_Os04g43800	18.2934387
LOC_Os04g43410	LOC_Os08g20730	9.38060182
LOC_Os04g43760	LOC_Os05g35290	9.67350235
LOC_Os04g43760	LOC_Os07g44260	9.10555725
LOC_Os04g42920	LOC_Os06g07210	10.2469858
LOC_Os04g42920	LOC_Os05g31110	9.11855337
LOC_Os04g42380	LOC_Os06g10970	8.88941236
LOC_Os04g42250	LOC_Os07g46460	9.09450993
LOC_Os04g43760	LOC_Os12g33610	12.0381978
LOC_Os04g43400	LOC_Os06g47320	9.41166008
LOC_Os04g43750	LOC_Os05g41900	9.46210938
LOC_Os04g43800	LOC_Os09g23540	9.67508892
LOC_Os04g43410	LOC_Os11g26910	9.48313077
LOC_Os04g42920	LOC_Os11g05730	9.35908552
LOC_Os01g17190	LOC_Os02g18550	9.35731439
LOC_Os01g17330	LOC_Os02g19420	8.92303782
LOC_Os01g18110	LOC_Os01g27360	9.05378228
LOC_Os01g18110	LOC_Os04g47170	9.12895215
LOC_Os01g17190	LOC_Os07g26540	9.63137303
LOC_Os01g17180	LOC_Os11g03290	9.41491466

InteractorA	InteractorB	Z score
LOC_Os01g18050	LOC_Os01g23580	9.97258796
LOC_Os01g17250	LOC_Os04g16740	9.25470976
LOC_Os01g17250	LOC_Os08g28800	9.34039578
LOC_Os01g18050	LOC_Os10g28050	9.37704575
LOC_Os01g17190	LOC_Os01g55870	10.2322231
LOC_Os01g17330	LOC_Os02g39064	8.8808661
LOC_Os01g17190	LOC_Os07g03590	9.3578449
LOC_Os01g17190	LOC_Os01g46240	9.22536939
LOC_Os01g18050	LOC_Os10g41490	9.17668605
LOC_Os01g17190	LOC_Os02g49610	9.56587909
LOC_Os01g18110	LOC_Os03g21460	9.18199973
LOC_Os01g17330	LOC_Os03g58430	9.96093244
LOC_Os01g18050	LOC_Os05g07090	9.35302589
LOC_Os01g18110	LOC_Os06g06730	9.16541308
LOC_Os01g17190	LOC_Os01g25484	9.5650428
LOC_Os01g17190	LOC_Os03g06620	12.2177259
LOC_Os01g17190	LOC_Os04g56950	9.01004478
LOC_Os01g17330	LOC_Os05g41210	9.86233635
LOC_Os01g18050	LOC_Os01g73140	9.655924
LOC_Os01g17330	LOC_Os02g03610	9.70800608
LOC_Os01g17250	LOC_Os05g42350	8.86848158
LOC_Os01g17330	LOC_Os04g57950	9.32547558
LOC_Os01g17180	LOC_Os08g33370	8.81686045
LOC_Os01g17190	LOC_Os02g12780	9.75821135
LOC_Os01g18110	LOC_Os03g55070	9.49764603
LOC_Os01g18050	LOC_Os12g10720	9.38618332
LOC_Os01g17250	LOC_Os04g40130	8.81673905
LOC_Os01g18050	LOC_Os02g01280	9.2610631
LOC_Os01g17190	LOC_Os01g54490	11.6602326
LOC_Os01g18050	LOC_Os02g57150	9.8724798
LOC_Os01g17330	LOC_Os11g32260	9.71199362
LOC_Os01g17330	LOC_Os08g28820	9.89678554
LOC_Os01g18050	LOC_Os03g49260	9.63586465
LOC_Os01g17330	LOC_Os03g12270	8.80100946
LOC_Os01g17250	LOC_Os03g04250	10.6809828
LOC_Os01g18110	LOC_Os01g46570	11.5374339
LOC_Os01g17330	LOC_Os11g42350	8.89333125
LOC_Os01g17250	LOC_Os09g10230	9.98041217
LOC_Os01g17190	LOC_Os03g04260	9.32629036
LOC_Os01g17250	LOC_Os01g71320	10.5509118
LOC_Os01g17330	LOC_Os07g05150	9.48648783
LOC_Os01g17190	LOC_Os10g28200	9.64798723
LOC_Os01g17330	LOC_Os01g66720	9.08123706

InteractorA	InteractorB	Z score
LOC_Os01g17190	LOC_Os12g12580	9.60199431
LOC_Os01g17190	LOC_Os12g12514	9.19369298
LOC_Os01g17330	LOC_Os11g08340	9.41238924
LOC_Os01g17190	LOC_Os04g22730	8.82796455
LOC_Os01g17250	LOC_Os02g33110	9.33691609
LOC_Os01g17330	LOC_Os02g42520	10.5564572
LOC_Os01g17190	LOC_Os11g32580	9.92739889
LOC_Os01g17330	LOC_Os09g32840	9.25242258
LOC_Os01g17190	LOC_Os07g48010	10.7383669
LOC_Os01g17190	LOC_Os06g10930	9.2578849
LOC_Os01g17190	LOC_Os03g63330	9.28638599
LOC_Os01g17250	LOC_Os03g61330	8.92904839
LOC_Os01g18110	LOC_Os02g06300	9.05728012
LOC_Os01g17190	LOC_Os04g41130	9.03223676
LOC_Os01g17330	LOC_Os02g06700	9.46151081
LOC_Os01g17190	LOC_Os10g29470	8.85559408
LOC_Os01g17190	LOC_Os01g73140	9.80373595
LOC_Os01g18110	LOC_Os01g71990	8.84492267
LOC_Os01g17190	LOC_Os11g29190	9.35192429
LOC_Os01g18050	LOC_Os11g19800	9.26717613
LOC_Os01g17330	LOC_Os02g06300	8.83842938
LOC_Os01g17180	LOC_Os10g26130	8.9012821
LOC_Os01g17190	LOC_Os07g03467	9.3578191
LOC_Os01g18050	LOC_Os01g37910	9.72218052
LOC_Os01g18050	LOC_Os06g01360	9.24214003
LOC_Os01g18050	LOC_Os09g10230	9.34464403
LOC_Os01g18050	LOC_Os01g60190	9.42917008
LOC_Os01g17330	LOC_Os10g09860	8.86745832
LOC_Os01g17250	LOC_Os05g40420	8.93191166
LOC_Os01g18110	LOC_Os03g09250	11.3376326
LOC_Os01g17330	LOC_Os11g08120	9.20581398
LOC_Os01g18050	LOC_Os07g08880	9.01844006
LOC_Os01g17250	LOC_Os08g28680	9.02067665
LOC_Os01g18050	LOC_Os12g40550	8.80044131
LOC_Os01g17190	LOC_Os03g23970	8.92196376
LOC_Os01g17190	LOC_Os02g19770	9.06803251
LOC_Os01g18110	LOC_Os01g71410	9.47738707
LOC_Os01g17190	LOC_Os04g09540	10.1397844
LOC_Os01g17330	LOC_Os11g32580	8.80495586
LOC_Os01g17190	LOC_Os07g03377	9.3578191
LOC_Os01g17180	LOC_Os12g38760	8.91445702
LOC_Os01g18050	LOC_Os05g51480	9.00524255
LOC_Os01g18050	LOC_Os04g39210	9.51531368

InteractorA	InteractorB	Z score
LOC_Os01g17330	LOC_Os01g61210	9.08057015
LOC_Os01g18110	LOC_Os02g52420	11.0699487
LOC_Os01g17190	LOC_Os02g57240	10.8783455
LOC_Os01g17330	LOC_Os05g31040	10.1327923
LOC_Os01g18050	LOC_Os03g16860	9.66020569
LOC_Os01g18050	LOC_Os02g32370	8.86565213
LOC_Os01g17330	LOC_Os04g24520	9.09297972
LOC_Os01g18110	LOC_Os04g19740	9.02689823
LOC_Os01g18050	LOC_Os02g27760	8.94038775
LOC_Os01g17190	LOC_Os07g03288	9.3578449
LOC_Os01g18110	LOC_Os06g06560	9.90371249
LOC_Os01g18050	LOC_Os01g44980	9.20262906
LOC_Os01g17330	LOC_Os10g36650	8.81960349
LOC_Os01g18050	LOC_Os02g12580	10.2685259
LOC_Os01g17190	LOC_Os04g52340	9.22454997
LOC_Os01g18050	LOC_Os05g38550	8.95017361
LOC_Os01g17330	LOC_Os02g30630	8.98710131
LOC_Os01g18050	LOC_Os01g34614	10.1157421
LOC_Os01g18050	LOC_Os01g38970	11.111859
LOC_Os01g18050	LOC_Os03g44150	8.86733258
LOC_Os01g18110	LOC_Os03g17470	9.52471
LOC_Os01g18110	LOC_Os01g51410	8.86399539
LOC_Os01g17330	LOC_Os03g25340	8.85341682
LOC_Os01g17330	LOC_Os03g55090	8.82216083
LOC_Os01g17190	LOC_Os03g58290	10.1278846
LOC_Os01g17190	LOC_Os05g06480	9.17483946
LOC_Os01g18110	LOC_Os02g38210	9.23752022
LOC_Os01g18050	LOC_Os04g59600	8.8413417
LOC_Os01g18110	LOC_Os01g19750	9.29779876
LOC_Os01g17190	LOC_Os07g37790	9.22458292
LOC_Os01g17330	LOC_Os03g09910	9.04764401
LOC_Os01g18050	LOC_Os06g24990	8.92339363
LOC_Os01g17330	LOC_Os02g02210	9.01278707
LOC_Os01g17190	LOC_Os04g38600	9.32166999
LOC_Os04g44730	LOC_Os06g36840	10.770793
LOC_Os04g44920	LOC_Os12g34380	9.33203454
LOC_Os04g43800	LOC_Os11g48110	10.4853075
LOC_Os04g44890	LOC_Os11g08460	9.4393362
LOC_Os04g44920	LOC_Os11g19800	10.1317135
LOC_Os04g44870	LOC_Os12g32240	9.59949621
LOC_Os04g45290	LOC_Os06g04030	9.10759449
LOC_Os04g44924	LOC_Os05g09440	11.841331
LOC_Os04g44920	LOC_Os09g07460	9.1532181

InteractorA	InteractorB	Z score
LOC_Os04g44950	LOC_Os05g01810	9.51871062
LOC_Os04g44470	LOC_Os09g11230	9.68185966
LOC_Os04g44890	LOC_Os07g31770	8.84184108
LOC_Os04g44730	LOC_Os11g40150	9.05632276
LOC_Os04g44730	LOC_Os06g06730	9.22236005
LOC_Os04g44890	LOC_Os06g39140	8.85443834
LOC_Os04g44870	LOC_Os05g45420	11.319283
LOC_Os04g44920	LOC_Os05g09440	9.90853673
LOC_Os04g44870	LOC_Os07g05940	9.73708775
LOC_Os04g45290	LOC_Os07g30170	10.8758548
LOC_Os04g43800	LOC_Os12g43630	9.70656977
LOC_Os04g44870	LOC_Os06g34690	9.48051217
LOC_Os04g45290	LOC_Os06g34690	8.87848062
LOC_Os04g44924	LOC_Os05g37700	9.59547541
LOC_Os04g45290	LOC_Os05g19150	10.0374834
LOC_Os04g44730	LOC_Os10g27050	10.897361
LOC_Os04g44730	LOC_Os08g09260	9.01253014
LOC_Os04g44920	LOC_Os12g34874	9.33579366
LOC_Os04g44060	LOC_Os07g28280	9.3668852
LOC_Os04g44920	LOC_Os05g47640	8.87285249
LOC_Os04g44920	LOC_Os12g17540	9.78830066
LOC_Os04g44950	LOC_Os07g38970	9.36478409
LOC_Os04g44920	LOC_Os08g34190	8.81958687
LOC_Os04g44920	LOC_Os07g34190	10.2817627
LOC_Os04g44920	LOC_Os10g26110	8.86261014
LOC_Os04g44730	LOC_Os06g29220	9.09234742
LOC_Os04g44870	LOC_Os08g08500	9.92523485
LOC_Os04g44730	LOC_Os08g09770	9.67758026
LOC_Os04g44920	LOC_Os08g44280	9.35517996
LOC_Os04g44920	LOC_Os09g20090	9.03472575
LOC_Os04g44924	LOC_Os11g34450	11.3302274
LOC_Os04g44920	LOC_Os10g28350	8.87320179
LOC_Os04g44730	LOC_Os06g43640	8.91103447
LOC_Os04g45290	LOC_Os06g40190	9.03494137
LOC_Os04g45290	LOC_Os05g04470	9.11548504
LOC_Os04g44950	LOC_Os07g46830	9.37646111
LOC_Os04g44730	LOC_Os07g44620	9.30014993
LOC_Os04g44950	LOC_Os06g35590	9.36729113
LOC_Os04g44730	LOC_Os05g49830	10.037616
LOC_Os04g44890	LOC_Os05g49840	9.8310887
LOC_Os04g44924	LOC_Os11g32580	8.90248349
LOC_Os04g44920	LOC_Os06g08770	8.89118098
LOC_Os04g44920	LOC_Os08g44340	9.25204725

InteractorA	InteractorB	Z score
LOC_Os04g44950	LOC_Os08g44270	9.15533704
LOC_Os04g44730	LOC_Os05g41640	9.03725959
LOC_Os04g44470	LOC_Os05g33380	9.10798435
LOC_Os04g45290	LOC_Os05g04520	9.91039479
LOC_Os04g44950	LOC_Os09g31490	8.82407995
LOC_Os04g44920	LOC_Os04g48700	9.23812396
LOC_Os04g44920	LOC_Os07g46310	10.7594953
LOC_Os04g44730	LOC_Os07g36130	10.4811136
LOC_Os04g44920	LOC_Os04g59450	10.2338087
LOC_Os04g44730	LOC_Os12g22680	10.7044316
LOC_Os04g44470	LOC_Os08g16910	9.73703992
LOC_Os04g44890	LOC_Os09g34960	9.10034171
LOC_Os04g44920	LOC_Os08g14770	9.16718759
LOC_Os04g44870	LOC_Os07g43260	8.81763422
LOC_Os04g44950	LOC_Os11g36719	11.178162
LOC_Os04g44730	LOC_Os12g31640	8.80631346
LOC_Os04g43800	LOC_Os12g33610	9.90954313
LOC_Os04g45290	LOC_Os06g48180	8.92460241
LOC_Os04g44920	LOC_Os07g02350	9.76777487
LOC_Os04g44920	LOC_Os06g08310	9.67324017
LOC_Os04g45290	LOC_Os05g28280	9.03845616
LOC_Os04g45290	LOC_Os05g34770	9.91176709
LOC_Os04g43800	LOC_Os12g31640	8.98423985
LOC_Os04g44870	LOC_Os08g33710	10.8260158
LOC_Os04g44920	LOC_Os07g08030	10.1847548
LOC_Os04g45290	LOC_Os05g41080	8.81432327
LOC_Os04g44890	LOC_Os08g42560	8.99454664
LOC_Os04g44870	LOC_Os10g32550	8.96211242
LOC_Os04g44730	LOC_Os06g44620	10.0288511
LOC_Os04g44060	LOC_Os09g26380	9.94124365
LOC_Os04g44730	LOC_Os08g34290	8.95791714
LOC_Os04g44730	LOC_Os08g44810	9.04263421
LOC_Os04g44870	LOC_Os06g11210	8.95194835
LOC_Os04g44060	LOC_Os12g03816	9.5734189
LOC_Os04g44890	LOC_Os04g52130	8.98156013
LOC_Os04g44730	LOC_Os12g10570	9.93357279
LOC_Os04g44470	LOC_Os07g43470	9.0728512
LOC_Os04g44870	LOC_Os09g10300	9.58781713
LOC_Os04g44924	LOC_Os04g57220	8.8110051
LOC_Os04g44060	LOC_Os07g40290	10.1539883
LOC_Os04g44870	LOC_Os12g03816	9.08873831
LOC_Os04g44890	LOC_Os10g25950	9.46639687
LOC_Os04g47170	LOC_Os09g33860	9.23375685

InteractorA	InteractorB	Z score
LOC_Os04g45470	LOC_Os06g05110	10.3639799
LOC_Os04g46930	LOC_Os10g42720	9.13209352
LOC_Os04g45290	LOC_Os08g06550	9.09769719
LOC_Os04g46460	LOC_Os07g23470	9.5703472
LOC_Os04g47170	LOC_Os04g55290	9.718056
LOC_Os04g45490	LOC_Os11g16590	9.87917717
LOC_Os04g46560	LOC_Os05g19150	9.51212334
LOC_Os04g46930	LOC_Os10g33800	9.54208972
LOC_Os04g46620	LOC_Os04g52280	11.5648271
LOC_Os04g45490	LOC_Os12g22680	9.0862489
LOC_Os04g46560	LOC_Os09g25370	10.3274845
LOC_Os04g46910	LOC_Os07g31770	9.92202856
LOC_Os04g46620	LOC_Os07g10660	9.19596217
LOC_Os04g46460	LOC_Os09g23530	8.89660649
LOC_Os04g46560	LOC_Os09g21770	11.7533613
LOC_Os04g46930	LOC_Os08g16910	9.64731803
LOC_Os04g45290	LOC_Os10g30840	10.973072
LOC_Os04g45470	LOC_Os06g48160	9.10900888
LOC_Os04g45290	LOC_Os12g13800	9.15021149
LOC_Os04g46620	LOC_Os07g49400	9.85857972
LOC_Os04g47170	LOC_Os12g41110	9.41345131
LOC_Os04g46930	LOC_Os05g04470	9.02105459
LOC_Os04g46460	LOC_Os04g48850	10.4021945
LOC_Os04g47170	LOC_Os06g51150	9.22430949
LOC_Os04g47170	LOC_Os07g48050	8.8826914
LOC_Os04g46930	LOC_Os06g10970	8.96011244
LOC_Os04g46460	LOC_Os06g47600	10.1080578
LOC_Os04g45470	LOC_Os09g23560	8.84022491
LOC_Os04g45490	LOC_Os04g56760	8.85270323
LOC_Os04g45490	LOC_Os07g08500	12.8417628
LOC_Os04g46620	LOC_Os05g19380	10.8595433
LOC_Os04g46620	LOC_Os06g10970	10.2364482
LOC_Os04g46560	LOC_Os10g23900	10.5691437
LOC_Os04g46560	LOC_Os08g34280	9.75749069
LOC_Os04g46910	LOC_Os06g10340	8.81156425
LOC_Os04g46560	LOC_Os09g08072	9.00275883
LOC_Os04g46620	LOC_Os05g15520	8.99313743
LOC_Os04g46930	LOC_Os12g17540	8.87508632
LOC_Os04g46620	LOC_Os06g49970	9.64520051
LOC_Os04g46460	LOC_Os09g30412	8.82823937
LOC_Os04g46910	LOC_Os08g14760	8.85831043
LOC_Os04g46460	LOC_Os10g13800	9.05125711
LOC_Os04g46560	LOC_Os07g19040	9.32240532

InteractorA	InteractorB	Z score
LOC_Os04g45490	LOC_Os06g04000	9.34031622
LOC_Os04g46460	LOC_Os06g39875	9.97638184
LOC_Os04g47170	LOC_Os09g11230	8.87259575
LOC_Os04g46910	LOC_Os12g41110	8.94491812
LOC_Os04g45290	LOC_Os11g36719	10.0300871
LOC_Os04g45290	LOC_Os10g37210	8.91368125
LOC_Os04g46460	LOC_Os05g05800	9.07354029
LOC_Os04g45490	LOC_Os05g46860	9.15453325
LOC_Os04g46560	LOC_Os08g44370	9.12866389
LOC_Os04g45470	LOC_Os06g51150	10.0783196
LOC_Os04g47170	LOC_Os07g47420	11.3015259
LOC_Os04g46620	LOC_Os06g23780	9.18621159
LOC_Os04g47170	LOC_Os09g37100	9.73942682
LOC_Os04g46460	LOC_Os11g25330	8.98475969
LOC_Os04g47220	LOC_Os05g06480	8.87906448
LOC_Os04g47170	LOC_Os07g07060	11.6161205
LOC_Os04g45490	LOC_Os05g30410	8.85270323
LOC_Os04g46620	LOC_Os08g28730	8.97837869
LOC_Os04g45490	LOC_Os08g40140	8.85986043
LOC_Os04g47170	LOC_Os06g46000	9.24525395
LOC_Os04g47170	LOC_Os06g49470	8.95699986
LOC_Os04g45470	LOC_Os12g13390	11.7146428
LOC_Os04g46560	LOC_Os06g44620	8.81032979
LOC_Os04g46620	LOC_Os08g39140	9.2260444
LOC_Os04g46910	LOC_Os09g07830	10.9490809
LOC_Os04g47220	LOC_Os05g28940	10.0514922
LOC_Os04g47170	LOC_Os06g21570	9.08571666
LOC_Os04g45490	LOC_Os06g34690	9.94000614
LOC_Os04g46560	LOC_Os10g21192	9.8059372
LOC_Os04g45470	LOC_Os05g51480	10.3051317
LOC_Os04g46460	LOC_Os05g47640	9.31528932
LOC_Os04g45470	LOC_Os11g02440	8.9039982
LOC_Os04g45490	LOC_Os04g46620	10.5932629
LOC_Os04g46560	LOC_Os04g59040	8.95457642
LOC_Os04g46930	LOC_Os08g44350	8.91011966
LOC_Os04g45490	LOC_Os12g13390	8.92864161
LOC_Os04g46560	LOC_Os07g30170	9.26711846
LOC_Os04g45470	LOC_Os07g46830	9.60519443
LOC_Os04g45470	LOC_Os05g12210	9.50250963
LOC_Os04g45290	LOC_Os07g48040	10.0777683
LOC_Os04g46560	LOC_Os05g40420	10.050887
LOC_Os04g47170	LOC_Os06g43640	8.8241909
LOC_Os04g45290	LOC_Os10g21212	9.31056156

InteractorA	InteractorB	Z score
LOC_Os04g45470	LOC_Os05g45220	8.92671507
LOC_Os04g46620	LOC_Os08g38900	10.5090648
LOC_Os04g45290	LOC_Os10g21344	9.0051411
LOC_Os04g45470	LOC_Os07g08500	9.0139632
LOC_Os04g46460	LOC_Os07g07320	9.32876035
LOC_Os04g47170	LOC_Os06g09910	9.21434256
LOC_Os04g47170	LOC_Os09g23530	8.962173
LOC_Os04g45290	LOC_Os12g03816	9.27146869
LOC_Os04g46620	LOC_Os06g21570	9.75544947
LOC_Os04g45290	LOC_Os12g22680	8.98689737
LOC_Os04g45490	LOC_Os05g28280	9.20128691
LOC_Os04g45470	LOC_Os11g26860	11.490809
LOC_Os04g46560	LOC_Os05g45420	8.81492334
LOC_Os04g46460	LOC_Os12g05410	10.9428165
LOC_Os04g46620	LOC_Os05g05470	8.91086436
LOC_Os04g46620	LOC_Os07g31830	9.12461688
LOC_Os04g47170	LOC_Os07g38890	10.2814306
LOC_Os04g48850	LOC_Os08g09210	9.30020783
LOC_Os04g47360	LOC_Os12g05590	8.994555
LOC_Os04g49130	LOC_Os07g22930	10.1653249
LOC_Os04g48060	LOC_Os08g03040	10.0825155
LOC_Os04g47220	LOC_Os05g48510	8.9077219
LOC_Os04g48850	LOC_Os07g42940	11.1248641
LOC_Os04g48060	LOC_Os07g48880	9.00123182
LOC_Os04g48850	LOC_Os06g45590	9.49626759
LOC_Os04g48850	LOC_Os10g30840	8.91350716
LOC_Os04g48540	LOC_Os12g38180	9.13788497
LOC_Os04g48850	LOC_Os11g25260	11.2795261
LOC_Os04g48060	LOC_Os10g30200	8.9025895
LOC_Os04g47220	LOC_Os09g23530	9.12614977
LOC_Os04g48850	LOC_Os12g07980	8.83911657
LOC_Os04g48540	LOC_Os06g06300	8.84389652
LOC_Os04g48060	LOC_Os07g48010	8.82735413
LOC_Os04g50880	LOC_Os09g25390	9.57403227
LOC_Os04g48060	LOC_Os12g08270	9.47410515
LOC_Os04g50880	LOC_Os10g01570	8.82991727
LOC_Os04g49130	LOC_Os05g40420	8.9749775
LOC_Os04g48700	LOC_Os06g11210	9.47158688
LOC_Os04g48700	LOC_Os11g01872	9.44742996
LOC_Os04g48700	LOC_Os10g25950	9.21515348
LOC_Os04g49420	LOC_Os11g10520	10.9141423
LOC_Os04g48850	LOC_Os11g07020	9.34589446
LOC_Os04g48700	LOC_Os05g38760	9.54624565

InteractorA	InteractorB	Z score
LOC_Os04g48060	LOC_Os05g41080	10.6093508
LOC_Os04g48540	LOC_Os08g02410	8.96156785
LOC_Os04g47360	LOC_Os09g28460	8.92432719
LOC_Os04g50880	LOC_Os08g09200	9.32984078
LOC_Os04g48850	LOC_Os12g42884	9.40062906
LOC_Os04g48850	LOC_Os07g39870	9.2885982
LOC_Os04g48540	LOC_Os10g25930	10.2721731
LOC_Os04g47360	LOC_Os12g06620	9.26440293
LOC_Os04g47220	LOC_Os08g32620	9.14737774
LOC_Os04g48850	LOC_Os05g36290	10.9699153
LOC_Os04g48540	LOC_Os09g27750	8.85243473
LOC_Os04g48850	LOC_Os11g10480	9.13588601
LOC_Os04g48850	LOC_Os08g42540	10.5067022
LOC_Os04g48850	LOC_Os10g38690	10.1865725
LOC_Os04g49420	LOC_Os12g22680	9.95584485
LOC_Os04g48700	LOC_Os07g22600	9.35820004
LOC_Os04g48850	LOC_Os05g06750	8.91269458
LOC_Os04g48850	LOC_Os07g03368	8.83693357
LOC_Os04g48850	LOC_Os07g03960	9.9278176
LOC_Os04g49130	LOC_Os12g01922	8.98018594
LOC_Os04g47220	LOC_Os05g41640	9.1588298
LOC_Os04g48540	LOC_Os05g05620	9.4988846
LOC_Os04g48850	LOC_Os08g42730	8.93639633
LOC_Os04g48850	LOC_Os10g38630	9.01989496
LOC_Os04g48850	LOC_Os05g05680	8.90661203
LOC_Os04g48700	LOC_Os07g47290	9.37804054
LOC_Os04g48850	LOC_Os10g38540	8.96819987
LOC_Os04g48850	LOC_Os08g44340	9.6002779
LOC_Os04g49420	LOC_Os10g39170	9.48057701
LOC_Os04g47360	LOC_Os05g37330	9.07468592
LOC_Os04g49130	LOC_Os09g31430	8.84316551
LOC_Os04g47360	LOC_Os07g35880	9.07273669
LOC_Os04g48540	LOC_Os12g25120	8.83781208
LOC_Os04g48850	LOC_Os07g07320	9.21495143
LOC_Os04g48540	LOC_Os06g04200	9.36737366
LOC_Os04g48850	LOC_Os05g44340	9.05404997
LOC_Os04g49420	LOC_Os11g10480	8.92559852
LOC_Os04g48850	LOC_Os07g05160	9.84130812
LOC_Os04g48850	LOC_Os09g31490	9.46491644
LOC_Os04g48850	LOC_Os06g24390	9.19223103
LOC_Os04g48850	LOC_Os05g10780	9.73745085
LOC_Os04g48850	LOC_Os05g06450	10.0811561
LOC_Os04g49420	LOC_Os10g01540	9.10660661

InteractorA	InteractorB	Z score
LOC_Os04g48850	LOC_Os06g35814	10.4164063
LOC_Os04g50880	LOC_Os10g38234	9.04845769
LOC_Os04g50880	LOC_Os11g18870	8.88456912
LOC_Os04g48540	LOC_Os12g10560	9.18209407
LOC_Os04g48850	LOC_Os08g44370	9.87179135
LOC_Os04g48700	LOC_Os06g10950	9.12896059
LOC_Os04g47360	LOC_Os09g07830	10.7010522
LOC_Os04g49420	LOC_Os11g31620	9.02282073
LOC_Os04g48540	LOC_Os06g49470	9.25590998
LOC_Os04g48700	LOC_Os06g03770	9.20968405
LOC_Os04g48850	LOC_Os06g41810	9.6131475
LOC_Os04g48540	LOC_Os12g08270	9.2692287
LOC_Os04g48700	LOC_Os12g02980	9.0132807
LOC_Os04g50880	LOC_Os05g12180	8.89209325
LOC_Os04g48060	LOC_Os07g49220	9.49470099
LOC_Os04g48700	LOC_Os10g38140	11.3287313
LOC_Os04g48850	LOC_Os08g44810	8.87827295
LOC_Os04g49420	LOC_Os07g07709	10.0431435
LOC_Os04g47360	LOC_Os09g12660	9.93531132
LOC_Os04g50880	LOC_Os07g06410	9.9008893
LOC_Os04g48540	LOC_Os07g05940	9.60636919
LOC_Os04g48540	LOC_Os05g41640	9.9453046
LOC_Os04g48060	LOC_Os11g29190	8.97198166
LOC_Os04g47360	LOC_Os09g36830	9.999501
LOC_Os04g48850	LOC_Os10g38360	9.2701223
LOC_Os04g48850	LOC_Os08g44530	9.07917605
LOC_Os04g48850	LOC_Os04g53290	9.60236023
LOC_Os04g48700	LOC_Os09g37100	9.2921233
LOC_Os04g49130	LOC_Os06g40640	9.07308
LOC_Os04g48060	LOC_Os12g23170	9.76320561
LOC_Os04g49420	LOC_Os11g10510	8.86156807
LOC_Os04g48540	LOC_Os12g22030	8.9623446
LOC_Os04g49420	LOC_Os07g07719	8.80395171
LOC_Os04g48850	LOC_Os06g04900	10.8407713
LOC_Os04g50880	LOC_Os06g06320	9.33903872
LOC_Os04g48850	LOC_Os07g47990	10.0806911
LOC_Os04g49130	LOC_Os05g42350	9.1910766
LOC_Os04g48060	LOC_Os07g42490	8.85408105
LOC_Os04g48540	LOC_Os05g40990	9.91103524
LOC_Os04g50880	LOC_Os04g55960	9.43942758
LOC_Os04g49130	LOC_Os12g13810	11.170892
LOC_Os04g49420	LOC_Os10g22070	9.42848024
LOC_Os04g48850	LOC_Os10g41190	9.35698208

InteractorA	InteractorB	Z score
LOC_Os04g49130	LOC_Os06g10950	9.78559507
LOC_Os04g47220	LOC_Os07g03368	9.64363137
LOC_Os04g48540	LOC_Os08g06550	9.38782077
LOC_Os04g52280	LOC_Os06g12790	10.039399
LOC_Os04g52130	LOC_Os05g37700	8.83054415
LOC_Os04g52340	LOC_Os12g16220	8.80794187
LOC_Os04g53214	LOC_Os07g43390	9.36708768
LOC_Os04g52440	LOC_Os08g10510	20.1103778
LOC_Os04g52100	LOC_Os11g37550	9.35487267
LOC_Os04g52100	LOC_Os09g31502	10.585716
LOC_Os04g52340	LOC_Os10g09860	8.88924703
LOC_Os04g52361	LOC_Os11g26910	8.99456073
LOC_Os04g52361	LOC_Os08g02400	9.12707644
LOC_Os04g52280	LOC_Os08g35440	8.93605137
LOC_Os04g52100	LOC_Os10g03540	9.20054331
LOC_Os04g52100	LOC_Os05g46290	10.3269644
LOC_Os04g52100	LOC_Os08g06100	9.62950197
LOC_Os04g52361	LOC_Os04g57950	10.4115503
LOC_Os04g52100	LOC_Os10g34520	10.8962156
LOC_Os04g53214	LOC_Os08g28680	8.87261206
LOC_Os04g53230	LOC_Os08g35440	8.84070563
LOC_Os04g52280	LOC_Os07g11440	9.16585673
LOC_Os04g52280	LOC_Os11g32650	8.81467801
LOC_Os04g52361	LOC_Os09g10260	8.90200825
LOC_Os04g52280	LOC_Os04g58580	8.81706756
LOC_Os04g52450	LOC_Os08g41830	9.3054469
LOC_Os04g52100	LOC_Os06g44080	8.98583288
LOC_Os04g52100	LOC_Os07g44790	9.20226582
LOC_Os04g52100	LOC_Os08g17500	9.65997477
LOC_Os04g52340	LOC_Os11g16590	10.8336299
LOC_Os04g52130	LOC_Os06g04280	8.97132857
LOC_Os04g52130	LOC_Os06g05250	8.93218484
LOC_Os04g52100	LOC_Os04g56646	9.46891733
LOC_Os04g53214	LOC_Os06g06730	10.2783774
LOC_Os04g52450	LOC_Os08g31060	8.96091379
LOC_Os04g53230	LOC_Os05g41210	9.4252079
LOC_Os04g53230	LOC_Os06g11200	9.91860097
LOC_Os04g52361	LOC_Os12g40550	8.92209979
LOC_Os04g53214	LOC_Os12g12470	8.82574102
LOC_Os04g52340	LOC_Os06g45120	9.39991899
LOC_Os04g53214	LOC_Os07g05820	9.12697098
LOC_Os04g52280	LOC_Os07g17010	9.3843237
LOC_Os04g52440	LOC_Os08g41990	9.35618664

InteractorA	InteractorB	Z score
LOC_Os04g52280	LOC_Os06g35630	9.27342371
LOC_Os04g52280	LOC_Os07g48030	10.3346948
LOC_Os04g52340	LOC_Os04g57410	9.25480214
LOC_Os04g52100	LOC_Os10g40090	11.1608678
LOC_Os04g52440	LOC_Os06g40170	9.24044196
LOC_Os04g52100	LOC_Os04g52130	9.07758191
LOC_Os04g52450	LOC_Os07g11440	9.16364923
LOC_Os04g53214	LOC_Os07g42924	8.87815525
LOC_Os04g52280	LOC_Os10g34520	10.165089
LOC_Os04g52440	LOC_Os09g20820	10.1894076
LOC_Os04g53230	LOC_Os09g15400	8.96997784
LOC_Os04g52130	LOC_Os12g41110	9.09516621
LOC_Os04g53230	LOC_Os09g25320	10.2775414
LOC_Os04g52340	LOC_Os04g57400	8.80909569
LOC_Os04g52130	LOC_Os05g38230	12.4739032
LOC_Os04g52280	LOC_Os07g31770	9.47487119
LOC_Os04g52280	LOC_Os05g37390	9.22200822
LOC_Os04g52130	LOC_Os05g44140	8.81026511
LOC_Os04g52340	LOC_Os05g15520	9.0878941
LOC_Os04g53230	LOC_Os06g04200	9.00689465
LOC_Os04g52450	LOC_Os08g44810	11.0700889
LOC_Os04g52280	LOC_Os10g29470	9.12207482
LOC_Os04g52100	LOC_Os09g25320	8.98583288
LOC_Os04g52440	LOC_Os08g14770	11.1585638
LOC_Os04g52340	LOC_Os08g33370	10.0789541
LOC_Os04g52450	LOC_Os07g10720	9.4766559
LOC_Os04g52280	LOC_Os05g44180	9.44744678
LOC_Os04g52100	LOC_Os05g46580	8.87247253
LOC_Os04g52130	LOC_Os07g48020	8.95165598
LOC_Os04g52280	LOC_Os10g08580	8.81913191
LOC_Os04g53230	LOC_Os06g44080	10.2775535
LOC_Os04g52450	LOC_Os08g10510	11.3977147
LOC_Os04g52340	LOC_Os05g07880	10.5623353
LOC_Os04g52100	LOC_Os10g38710	9.47192426
LOC_Os04g52340	LOC_Os05g03820	11.2999445
LOC_Os04g53214	LOC_Os08g15030	9.32858577
LOC_Os04g52100	LOC_Os10g07040	8.86030822
LOC_Os04g52130	LOC_Os08g40170	9.0808411
LOC_Os04g52361	LOC_Os12g42876	8.82671167
LOC_Os04g53214	LOC_Os11g42350	9.45781573
LOC_Os04g53230	LOC_Os06g15990	8.99129698
LOC_Os04g52130	LOC_Os09g32840	9.61597693
LOC_Os04g52440	LOC_Os04g52450	10.3953023

InteractorA	InteractorB	Z score
LOC_Os04g52361	LOC_Os09g26380	9.11650181
LOC_Os04g52280	LOC_Os12g12590	9.5039378
LOC_Os04g52361	LOC_Os05g05620	8.90174145
LOC_Os04g52100	LOC_Os05g05620	8.96315443
LOC_Os04g53214	LOC_Os04g57220	9.12327025
LOC_Os04g53214	LOC_Os12g44350	9.62677419
LOC_Os04g52130	LOC_Os05g36010	10.9818416
LOC_Os04g53230	LOC_Os07g06970	9.12436407
LOC_Os04g53810	LOC_Os11g26860	9.04996705
LOC_Os04g54390	LOC_Os10g26600	9.19575462
LOC_Os04g53920	LOC_Os08g39300	10.7707599
LOC_Os04g53800	LOC_Os05g49770	9.23646773
LOC_Os04g54390	LOC_Os04g56320	9.40030504
LOC_Os04g53240	LOC_Os09g23550	8.9498646
LOC_Os04g53290	LOC_Os06g36770	9.7170012
LOC_Os04g54390	LOC_Os05g04450	9.18399064
LOC_Os04g53230	LOC_Os12g02980	9.33857424
LOC_Os04g53810	LOC_Os05g12190	9.82928392
LOC_Os04g53920	LOC_Os11g36719	8.97943546
LOC_Os04g54390	LOC_Os06g35814	9.20691626
LOC_Os04g53810	LOC_Os07g38910	8.8127428
LOC_Os04g54330	LOC_Os07g05820	9.59308899
LOC_Os04g53810	LOC_Os10g36100	9.20227927
LOC_Os04g55040	LOC_Os05g48040	10.2235037
LOC_Os04g55040	LOC_Os06g39740	8.89886885
LOC_Os04g55040	LOC_Os07g47290	8.95221688
LOC_Os04g53240	LOC_Os08g36320	10.2140475
LOC_Os04g53240	LOC_Os12g13390	10.1199587
LOC_Os04g53850	LOC_Os05g38560	9.45712006
LOC_Os04g54390	LOC_Os11g32540	9.09479004
LOC_Os04g53240	LOC_Os09g23560	9.10154952
LOC_Os04g53810	LOC_Os07g46310	10.0054728
LOC_Os04g55040	LOC_Os06g06090	10.7348199
LOC_Os04g53850	LOC_Os04g55410	9.22557662
LOC_Os04g53810	LOC_Os07g05150	8.81347763
LOC_Os04g54330	LOC_Os06g35660	8.95022314
LOC_Os04g53810	LOC_Os05g45810	8.91953035
LOC_Os04g53920	LOC_Os12g12514	9.12844101
LOC_Os04g53290	LOC_Os05g26890	8.824344
LOC_Os04g54390	LOC_Os11g32520	9.01370839
LOC_Os04g53920	LOC_Os04g57390	9.24431138
LOC_Os04g53290	LOC_Os07g03910	8.85845848
LOC_Os04g53920	LOC_Os06g04620	10.6166315

InteractorA	InteractorB	Z score
LOC_Os04g53810	LOC_Os05g47540	8.9184541
LOC_Os04g54390	LOC_Os08g39300	9.47625363
LOC_Os04g53920	LOC_Os11g47760	9.5602003
LOC_Os04g53290	LOC_Os07g47420	9.06927497
LOC_Os04g53290	LOC_Os05g11550	9.13711619
LOC_Os04g53810	LOC_Os05g44180	9.15398076
LOC_Os04g53920	LOC_Os08g37800	8.91145193
LOC_Os04g53810	LOC_Os11g37550	8.90517134
LOC_Os04g53920	LOC_Os07g32800	10.2088104
LOC_Os04g54330	LOC_Os09g39440	9.54033534
LOC_Os04g54390	LOC_Os08g20420	8.81635954
LOC_Os04g54390	LOC_Os07g48060	8.83540068
LOC_Os04g53230	LOC_Os11g34570	9.1652396
LOC_Os04g53810	LOC_Os11g25700	10.8300178
LOC_Os04g53920	LOC_Os06g40180	8.98571062
LOC_Os04g54390	LOC_Os05g51050	9.9206569
LOC_Os04g55040	LOC_Os05g44050	10.1781996
LOC_Os04g53920	LOC_Os10g21310	8.86301945
LOC_Os04g54390	LOC_Os05g06750	9.91691571
LOC_Os04g53240	LOC_Os07g48880	8.98484187
LOC_Os04g55040	LOC_Os07g39270	9.3239496
LOC_Os04g53290	LOC_Os10g38140	9.08326604
LOC_Os04g54390	LOC_Os12g25630	10.1804915
LOC_Os04g53290	LOC_Os10g38780	9.06329956
LOC_Os04g54390	LOC_Os11g30310	10.4237422
LOC_Os04g54330	LOC_Os06g11210	9.30493872
LOC_Os04g53810	LOC_Os06g24990	9.11201578
LOC_Os04g54330	LOC_Os05g45810	10.4531685
LOC_Os04g53230	LOC_Os12g10720	9.47967558
LOC_Os04g53920	LOC_Os05g47640	9.62778998
LOC_Os04g54330	LOC_Os10g39840	10.6499566
LOC_Os04g53290	LOC_Os07g46630	8.94644802
LOC_Os04g53240	LOC_Os07g43390	8.87129583
LOC_Os04g53810	LOC_Os07g44430	8.93565588
LOC_Os04g54330	LOC_Os10g21326	9.15877661
LOC_Os04g53800	LOC_Os07g38860	10.0881972
LOC_Os04g53290	LOC_Os11g01872	8.80886754
LOC_Os04g54390	LOC_Os08g42560	9.24699489
LOC_Os04g53290	LOC_Os06g41810	9.2897786
LOC_Os04g54330	LOC_Os09g10270	9.11102077
LOC_Os04g53240	LOC_Os12g38760	8.90867088
LOC_Os04g53920	LOC_Os06g46000	9.48000291
LOC_Os04g53290	LOC_Os09g12570	8.95698768

InteractorA	InteractorB	Z score
LOC_Os04g53810	LOC_Os11g32620	10.8557044
LOC_Os04g55040	LOC_Os08g02700	9.27555824
LOC_Os04g53800	LOC_Os11g08470	8.84064403
LOC_Os04g54390	LOC_Os05g41610	9.125157
LOC_Os04g53920	LOC_Os06g14510	9.48467976
LOC_Os04g53800	LOC_Os06g34690	9.72084026
LOC_Os04g54330	LOC_Os09g11230	8.89795144
LOC_Os04g54390	LOC_Os05g02940	8.86741743
LOC_Os04g53810	LOC_Os05g11550	8.97188588
LOC_Os04g53230	LOC_Os12g37360	9.95238152
LOC_Os04g54330	LOC_Os05g50380	9.50745973
LOC_Os04g53800	LOC_Os07g47290	8.85060529
LOC_Os04g53920	LOC_Os09g28400	9.10522699
LOC_Os04g53920	LOC_Os10g10434	8.91863756
LOC_Os04g53850	LOC_Os05g11550	8.89267536
LOC_Os04g53850	LOC_Os05g06750	10.1864675
LOC_Os04g56160	LOC_Os05g48290	10.9223043
LOC_Os04g55960	LOC_Os12g17910	9.56368444
LOC_Os04g55410	LOC_Os05g41900	9.01565239
LOC_Os04g56210	LOC_Os06g06100	8.8244523
LOC_Os04g55410	LOC_Os09g32840	8.82166641
LOC_Os04g55850	LOC_Os08g02400	9.24996566
LOC_Os04g55960	LOC_Os08g17784	8.83370831
LOC_Os04g55410	LOC_Os10g33800	9.86290846
LOC_Os04g55960	LOC_Os10g38740	9.34916207
LOC_Os04g56210	LOC_Os12g03816	9.75113174
LOC_Os04g55410	LOC_Os05g12210	9.13701059
LOC_Os04g55410	LOC_Os10g39680	9.77119401
LOC_Os04g55410	LOC_Os07g38540	10.0326588
LOC_Os04g56070	LOC_Os09g25150	9.25584738
LOC_Os04g56070	LOC_Os11g05570	8.97796523
LOC_Os04g55960	LOC_Os11g47600	10.2690127
LOC_Os04g55850	LOC_Os08g15292	10.2787117
LOC_Os04g56160	LOC_Os04g59200	8.82220915
LOC_Os04g56230	LOC_Os05g49880	12.1693775
LOC_Os04g55740	LOC_Os07g03690	8.93847229
LOC_Os04g55290	LOC_Os06g45590	10.568887
LOC_Os04g56070	LOC_Os10g21248	10.144318
LOC_Os04g56070	LOC_Os10g42720	10.3710314
LOC_Os04g56210	LOC_Os04g56230	14.7260391
LOC_Os04g56210	LOC_Os06g39708	10.1729904
LOC_Os04g56070	LOC_Os12g42884	8.86946146
LOC_Os04g55040	LOC_Os11g01872	9.65489952

InteractorA	InteractorB	Z score
LOC_Os04g55960	LOC_Os07g05180	9.2365197
LOC_Os04g56160	LOC_Os12g16240	8.88698439
LOC_Os04g55290	LOC_Os11g14220	10.7258417
LOC_Os04g55850	LOC_Os09g31502	9.57784722
LOC_Os04g55960	LOC_Os11g08330	9.70976456
LOC_Os04g55410	LOC_Os08g42560	9.67211233
LOC_Os04g56070	LOC_Os11g01872	10.1755476
LOC_Os04g56230	LOC_Os06g39140	8.87675391
LOC_Os04g55410	LOC_Os10g28350	9.19717925
LOC_Os04g55850	LOC_Os09g31430	8.86719298
LOC_Os04g55850	LOC_Os06g24390	8.85511494
LOC_Os04g56210	LOC_Os10g27190	9.00261701
LOC_Os04g56160	LOC_Os11g47580	8.80795879
LOC_Os04g55410	LOC_Os08g37490	9.1536625
LOC_Os04g55740	LOC_Os10g07616	8.93762535
LOC_Os04g55960	LOC_Os11g36719	10.0654861
LOC_Os04g55740	LOC_Os07g34520	8.82444266
LOC_Os04g55740	LOC_Os09g19560	8.82169518
LOC_Os04g55040	LOC_Os10g28200	8.85720382
LOC_Os04g56210	LOC_Os05g47540	9.15720599
LOC_Os04g55410	LOC_Os06g44080	8.86007851
LOC_Os04g56160	LOC_Os06g11210	9.51538278
LOC_Os04g55290	LOC_Os11g32510	9.58113067
LOC_Os04g56070	LOC_Os08g23730	9.88277902
LOC_Os04g55410	LOC_Os06g06730	9.1568822
LOC_Os04g55850	LOC_Os06g35700	10.0595408
LOC_Os04g55960	LOC_Os08g40140	9.4086101
LOC_Os04g55290	LOC_Os12g13380	9.13517172
LOC_Os04g55290	LOC_Os09g14670	9.56206955
LOC_Os04g55960	LOC_Os08g02400	10.0883426
LOC_Os04g56070	LOC_Os08g28730	9.02620096
LOC_Os04g56230	LOC_Os06g29220	9.11532716
LOC_Os04g56230	LOC_Os05g46580	9.9988777
LOC_Os04g56070	LOC_Os10g01540	9.47342994
LOC_Os04g55960	LOC_Os04g58640	9.08766627
LOC_Os04g55960	LOC_Os10g26110	9.53100736
LOC_Os04g55290	LOC_Os06g04270	10.1789289
LOC_Os04g55290	LOC_Os08g10010	8.87215436
LOC_Os04g55040	LOC_Os10g38229	11.860039
LOC_Os04g56160	LOC_Os11g30310	9.59483687
LOC_Os04g55410	LOC_Os07g05180	9.41226846
LOC_Os04g55960	LOC_Os09g07830	8.97339444
LOC_Os04g55850	LOC_Os08g04180	9.10549341

InteractorA	InteractorB	Z score
LOC_Os04g55410	LOC_Os04g57390	9.05426366
LOC_Os04g56230	LOC_Os06g36700	8.98429932
LOC_Os04g56160	LOC_Os05g42350	9.31017895
LOC_Os04g56070	LOC_Os05g05800	9.33849372
LOC_Os04g55410	LOC_Os09g25320	8.86006316
LOC_Os04g56070	LOC_Os11g08460	8.91422776
LOC_Os04g56230	LOC_Os07g09890	10.4797142
LOC_Os04g55040	LOC_Os08g37790	10.7297592
LOC_Os04g55290	LOC_Os12g34062	8.94128539
LOC_Os04g56210	LOC_Os09g31430	8.85287662
LOC_Os04g55410	LOC_Os07g48030	9.59387724
LOC_Os04g56210	LOC_Os12g40510	10.7225985
LOC_Os04g55410	LOC_Os09g10230	10.5273828
LOC_Os04g56160	LOC_Os06g35650	9.23111793
LOC_Os04g56210	LOC_Os07g44790	11.2397496
LOC_Os04g55040	LOC_Os08g44810	9.11497127
LOC_Os04g55410	LOC_Os05g08100	8.87072
LOC_Os04g55960	LOC_Os07g26660	9.05524116
LOC_Os04g55290	LOC_Os12g37960	9.83671468
LOC_Os04g56070	LOC_Os10g38229	9.19835451
LOC_Os04g55960	LOC_Os07g05800	9.76536909
LOC_Os04g56210	LOC_Os06g36700	8.84432336
LOC_Os04g55960	LOC_Os11g08440	10.2209654
LOC_Os04g55040	LOC_Os12g16290	9.63396577
LOC_Os04g56210	LOC_Os05g46580	14.1769503
LOC_Os04g55290	LOC_Os09g19954	9.76928507
LOC_Os04g55740	LOC_Os07g38030	8.83204309
LOC_Os04g55410	LOC_Os10g38640	9.07904715
LOC_Os04g55290	LOC_Os11g20790	12.0527892
LOC_Os04g55850	LOC_Os05g49830	8.84925607
LOC_Os04g55960	LOC_Os10g40730	10.8909505
LOC_Os04g56070	LOC_Os10g38234	9.6213717
LOC_Os04g55960	LOC_Os05g49200	9.12041021
LOC_Os04g55290	LOC_Os09g23550	8.808491
LOC_Os04g56070	LOC_Os08g04560	8.8236139
LOC_Os04g56230	LOC_Os06g27770	9.00405266
LOC_Os04g55040	LOC_Os08g35420	8.82635161
LOC_Os04g55960	LOC_Os06g06730	9.01380427
LOC_Os04g56210	LOC_Os11g05570	9.08126305
LOC_Os04g56230	LOC_Os06g19960	9.83563948
LOC_Os04g56210	LOC_Os09g37540	9.30722672
LOC_Os04g55960	LOC_Os06g51150	9.46412569
LOC_Os04g55960	LOC_Os08g44270	9.25659997

InteractorA	InteractorB	Z score
LOC_Os04g55960	LOC_Os05g41610	8.93170541
LOC_Os04g56210	LOC_Os05g25490	9.10555788
LOC_Os04g56730	LOC_Os05g48510	10.582038
LOC_Os04g56320	LOC_Os08g41880	9.62659612
LOC_Os04g56400	LOC_Os09g24990	9.35475835
LOC_Os04g56730	LOC_Os07g26540	9.10850635
LOC_Os04g56730	LOC_Os05g47545	9.91798045
LOC_Os04g56400	LOC_Os09g34214	9.15044322
LOC_Os04g56580	LOC_Os05g46360	10.4069976
LOC_Os04g57090	LOC_Os06g04280	8.82513834
LOC_Os04g56580	LOC_Os10g40600	9.04608924
LOC_Os04g56580	LOC_Os12g07980	9.02185677
LOC_Os04g56646	LOC_Os06g35480	9.04614197
LOC_Os04g56320	LOC_Os12g10720	8.87857886
LOC_Os04g56320	LOC_Os10g01570	9.33218668
LOC_Os04g56760	LOC_Os06g42130	9.49149339
LOC_Os04g56760	LOC_Os10g21250	8.98967796
LOC_Os04g56400	LOC_Os09g24530	9.61059636
LOC_Os04g56730	LOC_Os08g04560	9.01651476
LOC_Os04g56320	LOC_Os11g40150	9.10120683
LOC_Os04g56580	LOC_Os08g06610	8.82845808
LOC_Os04g56400	LOC_Os07g39290	10.0092452
LOC_Os04g56320	LOC_Os10g25930	10.2888529
LOC_Os04g56400	LOC_Os10g11810	9.14704392
LOC_Os04g56400	LOC_Os10g40720	9.15693329
LOC_Os04g56400	LOC_Os10g37060	12.7276603
LOC_Os04g56730	LOC_Os06g04030	8.97547916
LOC_Os04g56950	LOC_Os07g49400	9.40663817
LOC_Os04g56400	LOC_Os06g02490	8.96373348
LOC_Os04g56646	LOC_Os05g37700	9.30037154
LOC_Os04g56400	LOC_Os05g38550	9.60391683
LOC_Os04g56580	LOC_Os09g28400	9.51740458
LOC_Os04g56230	LOC_Os09g39810	8.88266537
LOC_Os04g56920	LOC_Os10g40090	9.10433297
LOC_Os04g56920	LOC_Os12g16200	9.53048795
LOC_Os04g56730	LOC_Os05g45220	9.25804534
LOC_Os04g56580	LOC_Os08g03440	8.87424314
LOC_Os04g56730	LOC_Os08g04540	9.37827426
LOC_Os04g56400	LOC_Os11g02600	8.82260671
LOC_Os04g56950	LOC_Os10g21344	8.9203009
LOC_Os04g56920	LOC_Os10g41689	9.09342551
LOC_Os04g56320	LOC_Os06g11800	11.7135711
LOC_Os04g57090	LOC_Os07g08840	8.93455021

InteractorA	InteractorB	Z score
LOC_Os04g56920	LOC_Os12g10720	9.27825737
LOC_Os04g56320	LOC_Os10g21326	9.89083567
LOC_Os04g56230	LOC_Os11g08445	10.0312285
LOC_Os04g56646	LOC_Os07g07320	9.1459698
LOC_Os04g56580	LOC_Os12g04980	8.99328726
LOC_Os04g56580	LOC_Os10g23100	9.25186546
LOC_Os04g56400	LOC_Os06g35650	10.1798712
LOC_Os04g56920	LOC_Os05g44140	11.0013783
LOC_Os04g56230	LOC_Os12g07720	9.46205543
LOC_Os04g56320	LOC_Os07g34260	9.56057597
LOC_Os04g56920	LOC_Os07g25150	8.80371178
LOC_Os04g56320	LOC_Os07g06410	9.15938665
LOC_Os04g56230	LOC_Os10g11140	8.96357526
LOC_Os04g56230	LOC_Os12g43100	12.1688668
LOC_Os04g56920	LOC_Os05g48040	9.93563634
LOC_Os04g56920	LOC_Os11g38959	9.24798127
LOC_Os04g56730	LOC_Os11g40140	8.80632686
LOC_Os04g56920	LOC_Os06g51084	9.17345438
LOC_Os04g56400	LOC_Os12g40550	9.40114206
LOC_Os04g56320	LOC_Os05g49770	9.78644521
LOC_Os04g56580	LOC_Os12g41110	9.21340009
LOC_Os04g56730	LOC_Os05g41210	9.58333527
LOC_Os04g56580	LOC_Os05g37884	10.1215537
LOC_Os04g56400	LOC_Os10g21240	8.81139285
LOC_Os04g56760	LOC_Os09g31486	9.28607124
LOC_Os04g56320	LOC_Os08g34190	9.41235819
LOC_Os04g56400	LOC_Os07g26900	9.13269126
LOC_Os04g56730	LOC_Os10g08670	9.11441548
LOC_Os04g56230	LOC_Os11g14220	8.98557113
LOC_Os04g56646	LOC_Os12g41220	9.768726
LOC_Os04g56400	LOC_Os10g38740	9.76657222
LOC_Os04g56400	LOC_Os09g20260	9.854859
LOC_Os04g56950	LOC_Os08g42560	9.47133459
LOC_Os04g56920	LOC_Os12g22650	11.6140298
LOC_Os04g57090	LOC_Os06g37560	8.87490763
LOC_Os04g56400	LOC_Os09g38030	9.20121527
LOC_Os04g56400	LOC_Os10g41550	9.13193802
LOC_Os04g56580	LOC_Os05g25490	11.1308166
LOC_Os04g56760	LOC_Os06g11210	8.8309327
LOC_Os04g56580	LOC_Os09g15420	9.49480445
LOC_Os04g56400	LOC_Os05g05470	9.67077461
LOC_Os04g56230	LOC_Os09g10230	8.85115937
LOC_Os04g56730	LOC_Os05g06450	10.4981347

InteractorA	InteractorB	Z score
LOC_Os04g56320	LOC_Os07g37320	9.09156837
LOC_Os04g56320	LOC_Os05g04690	8.86102615
LOC_Os04g56950	LOC_Os05g29880	10.7977753
LOC_Os04g56580	LOC_Os11g25260	9.17639583
LOC_Os04g56920	LOC_Os05g42150	9.73422613
LOC_Os04g56580	LOC_Os08g36900	9.09015906
LOC_Os04g56646	LOC_Os06g46284	9.11904331
LOC_Os04g56730	LOC_Os07g46830	8.80563603
LOC_Os04g56230	LOC_Os08g40930	9.35232918
LOC_Os04g56230	LOC_Os12g08270	9.42797302
LOC_Os04g56646	LOC_Os12g16240	8.80329749
LOC_Os04g56646	LOC_Os05g35400	11.6400957
LOC_Os04g56950	LOC_Os08g15292	11.0194554
LOC_Os04g56646	LOC_Os09g27820	10.3988352
LOC_Os04g56920	LOC_Os12g40510	10.7626747
LOC_Os04g56230	LOC_Os10g36650	8.98492146
LOC_Os04g56230	LOC_Os12g18900	9.25592159
LOC_Os04g56400	LOC_Os06g05250	8.84846919
LOC_Os04g56730	LOC_Os08g31870	9.4244129
LOC_Os04g56580	LOC_Os07g40580	9.25043412
LOC_Os04g56920	LOC_Os06g35530	8.96377354
LOC_Os04g56730	LOC_Os11g25330	9.94421178
LOC_Os04g57400	LOC_Os08g43190	11.0801329
LOC_Os04g57220	LOC_Os05g28280	9.29122106
LOC_Os04g57220	LOC_Os08g34280	9.81164866
LOC_Os04g58110	LOC_Os05g51570	9.27987512
LOC_Os04g57380	LOC_Os07g16970	10.1669787
LOC_Os04g57400	LOC_Os07g11440	10.617404
LOC_Os04g57590	LOC_Os09g25390	9.86843649
LOC_Os04g57400	LOC_Os10g39840	10.0501613
LOC_Os04g57950	LOC_Os08g31870	9.95557299
LOC_Os04g57410	LOC_Os05g06450	8.83111516
LOC_Os04g57950	LOC_Os12g13320	9.31047398
LOC_Os04g57090	LOC_Os10g34760	8.95608912
LOC_Os04g58110	LOC_Os10g21266	8.82899934
LOC_Os04g57390	LOC_Os05g36010	8.88461507
LOC_Os04g57590	LOC_Os07g05400	8.81856138
LOC_Os04g57220	LOC_Os05g31020	9.18678307
LOC_Os04g57390	LOC_Os12g12470	9.26239453
LOC_Os04g57220	LOC_Os08g02410	9.29989183
LOC_Os04g57590	LOC_Os06g36670	8.86694804
LOC_Os04g58110	LOC_Os09g12660	9.3574015
LOC_Os04g57220	LOC_Os09g08720	9.23176157

InteractorA	InteractorB	Z score
LOC_Os04g57380	LOC_Os09g38030	9.10986318
LOC_Os04g57410	LOC_Os08g14770	9.05268033
LOC_Os04g57400	LOC_Os10g38340	9.24294224
LOC_Os04g58110	LOC_Os07g31830	8.97207908
LOC_Os04g57090	LOC_Os09g26380	9.10468763
LOC_Os04g57400	LOC_Os09g07830	9.11553822
LOC_Os04g57390	LOC_Os08g09200	11.2047166
LOC_Os04g57400	LOC_Os07g06440	8.90115401
LOC_Os04g57220	LOC_Os12g12514	10.294158
LOC_Os04g57410	LOC_Os07g37790	9.25478473
LOC_Os04g57590	LOC_Os07g20544	9.75712062
LOC_Os04g57380	LOC_Os11g25700	8.94310688
LOC_Os04g57950	LOC_Os11g32650	9.26500537
LOC_Os04g58200	LOC_Os05g38530	8.92875062
LOC_Os04g57400	LOC_Os08g38920	8.99003501
LOC_Os04g58200	LOC_Os07g22930	9.90503314
LOC_Os04g57390	LOC_Os06g37080	9.44716756
LOC_Os04g57390	LOC_Os09g23530	9.12458044
LOC_Os04g57220	LOC_Os12g40550	9.15240441
LOC_Os04g57950	LOC_Os05g24580	12.546598
LOC_Os04g57590	LOC_Os12g07720	9.26321481
LOC_Os04g57950	LOC_Os08g04560	9.64420249
LOC_Os04g57390	LOC_Os04g59040	9.40598007
LOC_Os04g57220	LOC_Os07g44790	8.86540801
LOC_Os04g57410	LOC_Os12g22650	9.25933512
LOC_Os04g57390	LOC_Os11g10520	9.05117337
LOC_Os04g57090	LOC_Os12g25700	9.78379152
LOC_Os04g57950	LOC_Os08g41830	9.90763526
LOC_Os04g57590	LOC_Os06g19960	9.95029744
LOC_Os04g57410	LOC_Os08g35740	9.6212714
LOC_Os04g58200	LOC_Os06g45120	9.32515612
LOC_Os04g57590	LOC_Os07g30170	9.06226313
LOC_Os04g57590	LOC_Os09g38030	10.6610926
LOC_Os04g57220	LOC_Os06g36670	9.28039069
LOC_Os04g58110	LOC_Os05g23740	10.3336019
LOC_Os04g57950	LOC_Os09g36800	10.1722766
LOC_Os04g57950	LOC_Os09g31486	10.2795931
LOC_Os04g57410	LOC_Os08g03040	9.58915405
LOC_Os04g57410	LOC_Os12g10560	9.37449066
LOC_Os04g58200	LOC_Os08g35740	11.0499316
LOC_Os04g57220	LOC_Os06g51060	9.18659599
LOC_Os04g57220	LOC_Os07g38030	9.78947922
LOC_Os04g57410	LOC_Os10g38340	9.19097016

InteractorA	InteractorB	Z score
LOC_Os04g57390	LOC_Os05g48510	9.00284592
LOC_Os04g57400	LOC_Os06g05250	9.73638998
LOC_Os04g57400	LOC_Os06g46372	9.13177268
LOC_Os04g57220	LOC_Os09g37100	9.95751611
LOC_Os04g57380	LOC_Os09g31490	9.54533916
LOC_Os04g57410	LOC_Os08g42000	9.20794358
LOC_Os04g58110	LOC_Os05g41080	8.90502031
LOC_Os04g57400	LOC_Os07g37790	8.80917546
LOC_Os04g57400	LOC_Os08g44520	9.00007438
LOC_Os04g57400	LOC_Os06g11290	9.39482005
LOC_Os04g57380	LOC_Os07g34520	9.25451215
LOC_Os04g57220	LOC_Os04g58110	9.41193448
LOC_Os04g57590	LOC_Os12g03816	10.8778016
LOC_Os04g57090	LOC_Os11g03230	9.69878464
LOC_Os04g58110	LOC_Os07g08170	8.81212685
LOC_Os04g57590	LOC_Os09g28420	9.20523177
LOC_Os04g58110	LOC_Os09g28400	10.0655052
LOC_Os04g57400	LOC_Os12g13800	9.34717559
LOC_Os04g57400	LOC_Os12g12580	12.5953232
LOC_Os04g57220	LOC_Os09g34970	9.73692087
LOC_Os04g57590	LOC_Os08g20730	8.8600548
LOC_Os04g58110	LOC_Os12g14070	8.94736454
LOC_Os04g57410	LOC_Os05g42150	9.64487577
LOC_Os04g57390	LOC_Os12g36950	9.54503596
LOC_Os04g57220	LOC_Os08g34290	9.15088063
LOC_Os04g58110	LOC_Os06g03720	8.84305147
LOC_Os04g57380	LOC_Os05g48290	11.1990564
LOC_Os04g57380	LOC_Os07g44460	8.99563014
LOC_Os04g57400	LOC_Os08g27840	10.1033513
LOC_Os04g58110	LOC_Os09g36830	9.67939815
LOC_Os04g57590	LOC_Os06g12090	9.34345396
LOC_Os04g58200	LOC_Os06g42130	11.0137425
LOC_Os04g57590	LOC_Os08g09770	9.68744839
LOC_Os04g58580	LOC_Os10g38234	9.61240218
LOC_Os04g59040	LOC_Os12g43630	9.85262076
LOC_Os04g58570	LOC_Os06g11210	8.8297924
LOC_Os04g58200	LOC_Os10g38690	9.88765936
LOC_Os04g58570	LOC_Os06g48180	10.2344405
LOC_Os04g58880	LOC_Os07g07060	9.23376949
LOC_Os04g58680	LOC_Os06g45670	8.86519721
LOC_Os04g58640	LOC_Os07g07550	9.01529064
LOC_Os04g58200	LOC_Os09g32830	9.19852379
LOC_Os04g58710	LOC_Os12g41110	9.42767452

InteractorA	InteractorB	Z score
LOC_Os04g58570	LOC_Os06g14510	9.97832597
LOC_Os04g58580	LOC_Os07g03467	9.15893994
LOC_Os04g58580	LOC_Os11g47760	8.95331812
LOC_Os04g58570	LOC_Os05g44140	9.12338916
LOC_Os04g58710	LOC_Os05g15520	9.76359284
LOC_Os04g58710	LOC_Os06g04030	8.85756651
LOC_Os04g58800	LOC_Os05g47980	9.04958707
LOC_Os04g58710	LOC_Os11g47590	8.8801621
LOC_Os04g58580	LOC_Os07g22950	9.33929467
LOC_Os04g58200	LOC_Os11g16590	9.9341092
LOC_Os04g59040	LOC_Os12g42876	8.97777443
LOC_Os04g58200	LOC_Os09g23530	9.18499517
LOC_Os04g59040	LOC_Os08g28820	8.97548222
LOC_Os04g58580	LOC_Os07g03590	9.15893994
LOC_Os04g58680	LOC_Os05g26890	9.39823035
LOC_Os04g59040	LOC_Os10g27174	9.24642677
LOC_Os04g58570	LOC_Os12g25690	10.0382175
LOC_Os04g58640	LOC_Os09g26380	8.83713394
LOC_Os04g58640	LOC_Os12g08270	9.30659907
LOC_Os04g58580	LOC_Os10g37210	9.81058468
LOC_Os04g58570	LOC_Os07g38430	9.41016303
LOC_Os04g58880	LOC_Os05g06450	9.4004295
LOC_Os04g58680	LOC_Os05g05830	8.81243628
LOC_Os04g58580	LOC_Os08g09770	10.201132
LOC_Os04g58880	LOC_Os05g39960	10.1555965
LOC_Os04g58640	LOC_Os09g17740	9.28718895
LOC_Os04g58880	LOC_Os07g05180	10.1525611
LOC_Os04g58570	LOC_Os07g47490	9.12161295
LOC_Os04g58580	LOC_Os07g03377	9.15893994
LOC_Os04g58680	LOC_Os10g36650	8.90842544
LOC_Os04g58640	LOC_Os07g34580	9.95661715
LOC_Os04g59040	LOC_Os05g24580	8.87570931
LOC_Os04g58800	LOC_Os11g31620	11.6654374
LOC_Os04g58580	LOC_Os10g41510	8.97230211
LOC_Os04g58880	LOC_Os08g33370	8.90203577
LOC_Os04g59040	LOC_Os07g26540	8.9020285
LOC_Os04g58640	LOC_Os11g08460	9.42304761
LOC_Os04g58880	LOC_Os12g43490	9.4580526
LOC_Os04g58680	LOC_Os06g07080	9.42931051
LOC_Os04g58570	LOC_Os10g09860	8.9745781
LOC_Os04g58580	LOC_Os12g06620	9.32121032
LOC_Os04g59040	LOC_Os08g42560	9.30645768
LOC_Os04g58880	LOC_Os09g28400	10.1232492

InteractorA	InteractorB	Z score
LOC_Os04g59040	LOC_Os06g19960	9.53423925
LOC_Os04g58710	LOC_Os10g21324	8.99053063
LOC_Os04g58570	LOC_Os09g39810	10.4703941
LOC_Os04g58880	LOC_Os12g40830	13.4249667
LOC_Os04g58800	LOC_Os12g22650	8.85253359
LOC_Os04g58570	LOC_Os05g04470	8.84951548
LOC_Os04g58710	LOC_Os08g33820	8.93650064
LOC_Os04g59150	LOC_Os08g38300	9.01811282
LOC_Os04g58580	LOC_Os08g03440	9.30319129
LOC_Os04g58800	LOC_Os08g33370	8.87261977
LOC_Os04g58580	LOC_Os09g11230	9.10008219
LOC_Os04g58710	LOC_Os12g22650	8.94548447
LOC_Os04g58640	LOC_Os05g06300	9.637974
LOC_Os04g58580	LOC_Os11g32650	9.84717971
LOC_Os04g58710	LOC_Os08g15030	8.92585287
LOC_Os04g58680	LOC_Os07g28280	9.18810934
LOC_Os04g58580	LOC_Os07g03288	9.15893994
LOC_Os04g58800	LOC_Os12g13390	9.61429254
LOC_Os04g58570	LOC_Os09g10260	9.10648405
LOC_Os04g58200	LOC_Os10g38160	10.6941637
LOC_Os04g58580	LOC_Os06g01390	8.97572938
LOC_Os04g58580	LOC_Os12g43100	8.80107006
LOC_Os04g58570	LOC_Os08g09940	9.90811682
LOC_Os04g58580	LOC_Os12g01922	10.120233
LOC_Os05g01675	LOC_Os08g29520	9.65931695
LOC_Os05g01050	LOC_Os07g34589	9.12636803
LOC_Os04g59600	LOC_Os10g02040	10.5251309
LOC_Os05g01600	LOC_Os08g04560	9.47720696
LOC_Os05g01810	LOC_Os05g28280	9.64962893
LOC_Os04g59450	LOC_Os10g38189	9.31228968
LOC_Os04g59450	LOC_Os11g32770	9.01763799
LOC_Os04g59450	LOC_Os11g10510	9.44876985
LOC_Os05g01810	LOC_Os07g23730	8.99296234
LOC_Os04g59200	LOC_Os07g47490	10.0906164
LOC_Os05g01050	LOC_Os09g39380	9.011051
LOC_Os05g01600	LOC_Os12g17540	10.7275197
LOC_Os05g01675	LOC_Os06g07878	10.0349712
LOC_Os04g59450	LOC_Os06g40180	9.83789821
LOC_Os05g01675	LOC_Os09g10270	8.81894133
LOC_Os05g01600	LOC_Os10g34760	9.28750633
LOC_Os04g59450	LOC_Os06g36160	9.38564593
LOC_Os05g01600	LOC_Os12g02080	9.09796827
LOC_Os04g59200	LOC_Os08g37490	10.1027452

InteractorA	InteractorB	Z score
LOC_Os05g01050	LOC_Os08g33710	9.35972211
LOC_Os04g59200	LOC_Os10g08620	8.97381515
LOC_Os05g01675	LOC_Os06g51084	8.85485601
LOC_Os04g59200	LOC_Os07g43170	9.57522737
LOC_Os04g59200	LOC_Os10g33800	9.06702382
LOC_Os04g59200	LOC_Os09g39380	8.9809707
LOC_Os05g01675	LOC_Os06g07978	10.0349712
LOC_Os05g01675	LOC_Os07g41750	9.0020077
LOC_Os05g01600	LOC_Os08g10608	10.236485
LOC_Os04g59160	LOC_Os07g38910	8.9369999
LOC_Os05g01810	LOC_Os07g35940	10.1383227
LOC_Os05g01600	LOC_Os11g08470	9.12382478
LOC_Os05g01810	LOC_Os05g40420	9.23337068
LOC_Os05g01600	LOC_Os05g38530	9.66014154
LOC_Os05g01600	LOC_Os08g28190	9.08303493
LOC_Os05g01050	LOC_Os06g06460	9.16957199
LOC_Os05g01675	LOC_Os06g35700	10.6247134
LOC_Os04g59200	LOC_Os07g44790	9.89708638
LOC_Os04g59160	LOC_Os10g30200	9.34911107
LOC_Os05g01675	LOC_Os06g05110	8.98997507
LOC_Os04g59450	LOC_Os10g25140	10.538051
LOC_Os05g01050	LOC_Os07g05150	10.9259015
LOC_Os05g01050	LOC_Os09g36830	9.76677852
LOC_Os05g01600	LOC_Os10g38470	9.54595217
LOC_Os05g01050	LOC_Os12g14070	8.98357534
LOC_Os05g01490	LOC_Os10g38640	9.86798167
LOC_Os05g01810	LOC_Os07g44790	8.96255247
LOC_Os05g01600	LOC_Os06g01590	9.21839465
LOC_Os05g01675	LOC_Os07g42950	9.88948228
LOC_Os04g59160	LOC_Os10g38950	9.26796225
LOC_Os05g01675	LOC_Os10g25140	8.92441439
LOC_Os05g01675	LOC_Os09g20220	9.12305136
LOC_Os04g59200	LOC_Os10g39120	8.98941945
LOC_Os05g01810	LOC_Os07g08660	9.54489579
LOC_Os04g59160	LOC_Os11g37550	9.77068311
LOC_Os04g59200	LOC_Os05g46270	8.83533875
LOC_Os05g01675	LOC_Os07g44550	9.5856457
LOC_Os05g01050	LOC_Os12g13320	9.46116505
LOC_Os04g59200	LOC_Os05g30480	8.81444877
LOC_Os04g59450	LOC_Os06g23440	9.36717201
LOC_Os04g59200	LOC_Os11g38959	9.26253478
LOC_Os05g01050	LOC_Os08g09250	10.2295674
LOC_Os04g59200	LOC_Os11g32260	9.12967709

InteractorA	InteractorB	Z score
LOC_Os04g59200	LOC_Os08g17784	9.55514445
LOC_Os04g59450	LOC_Os09g28770	9.08571971
LOC_Os05g01050	LOC_Os06g04030	9.82043174
LOC_Os04g59450	LOC_Os11g05290	9.35087065
LOC_Os05g01050	LOC_Os08g35740	8.91696893
LOC_Os04g59200	LOC_Os06g51084	9.07173381
LOC_Os04g59200	LOC_Os09g26380	9.39185833
LOC_Os04g59200	LOC_Os05g44140	9.72214486
LOC_Os05g01810	LOC_Os11g43360	8.95782867
LOC_Os05g01050	LOC_Os05g36280	9.16957199
LOC_Os04g59200	LOC_Os10g02040	9.54608127
LOC_Os05g01810	LOC_Os09g09230	9.06092929
LOC_Os04g59200	LOC_Os10g22070	8.90881623
LOC_Os05g01675	LOC_Os10g38234	10.7294032
LOC_Os04g59600	LOC_Os09g39500	10.1900253
LOC_Os05g01050	LOC_Os11g05730	9.16957199
LOC_Os04g59200	LOC_Os06g01850	9.32420249
LOC_Os05g01810	LOC_Os07g06440	9.50002674
LOC_Os04g59200	LOC_Os12g22680	10.2017666
LOC_Os05g01600	LOC_Os08g34280	9.1070932
LOC_Os05g01050	LOC_Os08g37490	9.31272774
LOC_Os04g59600	LOC_Os08g04540	10.5837525
LOC_Os04g59200	LOC_Os06g50300	8.91405977
LOC_Os04g59200	LOC_Os10g26130	9.78153202
LOC_Os05g01810	LOC_Os11g10510	9.91329104
LOC_Os05g01675	LOC_Os10g38140	9.4849188
LOC_Os04g59600	LOC_Os10g38140	10.3339246
LOC_Os05g01600	LOC_Os12g42876	8.94164704
LOC_Os04g59450	LOC_Os07g48020	10.7025486
LOC_Os05g01675	LOC_Os07g38970	9.27438102
LOC_Os05g01675	LOC_Os09g36830	9.42672157
LOC_Os04g59600	LOC_Os09g31502	8.85563335
LOC_Os05g01810	LOC_Os08g14760	9.03035476
LOC_Os05g01050	LOC_Os08g02410	10.1309023
LOC_Os05g01050	LOC_Os09g10260	9.0181302
LOC_Os04g59600	LOC_Os08g04560	9.06720907
LOC_Os04g59450	LOC_Os07g01760	9.34729071
LOC_Os05g01675	LOC_Os07g07320	9.02969487
LOC_Os05g01050	LOC_Os06g06510	9.16957199
LOC_Os05g01600	LOC_Os06g06320	8.8108085
LOC_Os05g01675	LOC_Os05g06970	9.7006403
LOC_Os01g18170	LOC_Os06g29180	8.85026424
LOC_Os01g19150	LOC_Os01g38680	8.81189287

InteractorA	InteractorB	Z score
LOC_Os01g18120	LOC_Os04g56070	10.7834693
LOC_Os01g18110	LOC_Os11g10480	9.53124279
LOC_Os01g18400	LOC_Os10g35840	9.02281606
LOC_Os01g18110	LOC_Os07g47290	10.6711297
LOC_Os01g18120	LOC_Os01g47410	9.10924784
LOC_Os01g18110	LOC_Os10g41510	9.28307511
LOC_Os01g18110	LOC_Os10g22070	8.81037103
LOC_Os01g18110	LOC_Os07g44260	11.6109275
LOC_Os01g18120	LOC_Os10g01570	8.86141689
LOC_Os01g18400	LOC_Os11g01872	9.70159045
LOC_Os01g18110	LOC_Os07g30990	8.86027242
LOC_Os01g18400	LOC_Os03g10340	8.92718057
LOC_Os01g18110	LOC_Os07g25590	8.95070207
LOC_Os01g18400	LOC_Os02g02560	9.43548818
LOC_Os01g18400	LOC_Os06g10910	9.1636105
LOC_Os01g18400	LOC_Os05g33140	9.32067109
LOC_Os01g18860	LOC_Os02g32030	9.90090257
LOC_Os01g18120	LOC_Os12g22680	10.3203847
LOC_Os01g18860	LOC_Os01g37910	10.175995
LOC_Os01g18860	LOC_Os05g24580	9.88965011
LOC_Os01g18400	LOC_Os09g07830	9.46734126
LOC_Os01g18170	LOC_Os03g48780	9.49831418
LOC_Os01g18170	LOC_Os03g41438	9.07977382
LOC_Os01g18120	LOC_Os04g39840	8.8576036
LOC_Os01g18120	LOC_Os09g36800	9.21464004
LOC_Os01g18400	LOC_Os04g32460	8.90214513
LOC_Os01g18120	LOC_Os11g25330	9.32487505
LOC_Os01g18400	LOC_Os06g04200	9.38679218
LOC_Os01g18400	LOC_Os10g33900	8.81174841
LOC_Os01g18400	LOC_Os02g41680	9.61438821
LOC_Os01g18120	LOC_Os01g27360	8.84475024
LOC_Os01g18110	LOC_Os08g02410	10.7094169
LOC_Os01g18400	LOC_Os05g44050	9.13950164
LOC_Os01g18120	LOC_Os11g25700	8.89016333
LOC_Os01g19150	LOC_Os03g61280	9.51378708
LOC_Os01g18400	LOC_Os05g31110	9.81570451
LOC_Os01g18400	LOC_Os09g04050	9.77260475
LOC_Os01g18110	LOC_Os10g21230	8.99025491
LOC_Os01g18110	LOC_Os08g38300	9.16740081
LOC_Os01g18400	LOC_Os04g52280	9.30365612
LOC_Os01g18170	LOC_Os03g52460	9.38513607
LOC_Os01g18170	LOC_Os03g48750	9.73860241
LOC_Os01g18170	LOC_Os06g04270	9.38791067

InteractorA	InteractorB	Z score
LOC_Os01g19150	LOC_Os03g49350	9.37782698
LOC_Os01g18400	LOC_Os10g02480	9.00185567
LOC_Os01g18120	LOC_Os04g37460	9.55417649
LOC_Os01g18860	LOC_Os02g34860	10.4548435
LOC_Os01g19150	LOC_Os03g51200	9.24113349
LOC_Os01g18400	LOC_Os03g05590	8.8696142
LOC_Os01g18860	LOC_Os11g47570	8.89928654
LOC_Os01g18170	LOC_Os02g07720	10.3807372
LOC_Os01g18400	LOC_Os04g40874	9.08585396
LOC_Os01g18120	LOC_Os12g44030	10.4176134
LOC_Os01g18110	LOC_Os07g36190	9.14022097
LOC_Os01g18400	LOC_Os04g58580	9.92162226
LOC_Os01g18120	LOC_Os08g04460	10.3006386
LOC_Os01g18860	LOC_Os10g23900	9.43988924
LOC_Os01g18110	LOC_Os08g15292	9.12632046
LOC_Os01g18110	LOC_Os10g29620	9.78167635
LOC_Os01g18400	LOC_Os10g39170	8.86873588
LOC_Os01g18400	LOC_Os07g42490	9.02015187
LOC_Os01g18400	LOC_Os05g50890	9.39758423
LOC_Os01g18120	LOC_Os01g50030	9.66387966
LOC_Os01g18120	LOC_Os05g45810	9.76454816
LOC_Os01g18860	LOC_Os04g53214	9.36579968
LOC_Os01g18110	LOC_Os07g03409	8.93650217
LOC_Os01g18120	LOC_Os11g10480	9.78300805
LOC_Os01g18400	LOC_Os11g08440	10.6994637
LOC_Os01g18170	LOC_Os01g56380	9.49281973
LOC_Os01g18400	LOC_Os11g26850	9.26516955
LOC_Os01g18120	LOC_Os12g40550	9.8800479
LOC_Os01g18170	LOC_Os03g48770	11.9769645
LOC_Os01g18110	LOC_Os07g03499	8.93650217
LOC_Os01g18400	LOC_Os03g59740	8.92302484
LOC_Os01g18860	LOC_Os02g01510	9.38515927
LOC_Os01g19150	LOC_Os02g46970	12.7600117
LOC_Os01g18110	LOC_Os07g03319	8.93650217
LOC_Os01g18860	LOC_Os12g44000	9.13638197
LOC_Os01g18120	LOC_Os10g39680	8.93544908
LOC_Os01g18120	LOC_Os01g60190	9.20579941
LOC_Os01g18400	LOC_Os06g35700	11.089779
LOC_Os01g19150	LOC_Os04g41310	8.88978604
LOC_Os01g18110	LOC_Os10g38274	8.99024796
LOC_Os01g18400	LOC_Os07g26540	8.8406465
LOC_Os05g04340	LOC_Os08g44530	9.10205869
LOC_Os05g02940	LOC_Os07g05150	8.82686556

InteractorA	InteractorB	Z score
LOC_Os05g02310	LOC_Os12g43630	10.6407328
LOC_Os05g04470	LOC_Os05g45810	8.96472234
LOC_Os05g04470	LOC_Os08g39140	9.1192233
LOC_Os05g04450	LOC_Os06g51150	9.08549553
LOC_Os05g01970	LOC_Os06g02490	8.86018367
LOC_Os05g03820	LOC_Os05g45810	10.2250743
LOC_Os05g02310	LOC_Os09g26380	9.70388787
LOC_Os05g04450	LOC_Os11g43200	8.96105805
LOC_Os05g04470	LOC_Os08g39300	10.6661382
LOC_Os05g04470	LOC_Os05g50380	9.26032451
LOC_Os05g04470	LOC_Os05g08100	8.90989255
LOC_Os05g04340	LOC_Os12g25710	8.85032416
LOC_Os05g02940	LOC_Os06g36700	8.85520743
LOC_Os05g03480	LOC_Os10g39880	9.43847513
LOC_Os05g04340	LOC_Os10g38229	9.32600823
LOC_Os05g02940	LOC_Os08g28820	8.84054635
LOC_Os05g01970	LOC_Os12g43370	9.47321173
LOC_Os05g04450	LOC_Os11g08120	8.88603911
LOC_Os05g04470	LOC_Os08g44520	9.54391714
LOC_Os05g04450	LOC_Os10g41490	9.34187044
LOC_Os05g04470	LOC_Os06g08770	9.10492667
LOC_Os05g03820	LOC_Os07g30990	10.0949455
LOC_Os05g04450	LOC_Os06g35590	8.80596991
LOC_Os05g04470	LOC_Os06g30370	11.1992388
LOC_Os05g03820	LOC_Os09g27820	9.52205249
LOC_Os05g02310	LOC_Os05g25850	9.25413906
LOC_Os05g02310	LOC_Os09g37949	9.27622397
LOC_Os05g04470	LOC_Os08g02340	8.92855271
LOC_Os05g02310	LOC_Os07g05400	9.00550157
LOC_Os05g03820	LOC_Os07g37790	11.2999273
LOC_Os05g04340	LOC_Os12g17910	9.25630829
LOC_Os05g04340	LOC_Os05g49770	10.3822042
LOC_Os05g02310	LOC_Os07g48040	9.5885219
LOC_Os05g04340	LOC_Os07g38430	8.8211966
LOC_Os05g04340	LOC_Os06g49470	10.97331
LOC_Os05g03480	LOC_Os05g46580	8.96381787
LOC_Os05g01970	LOC_Os12g13800	9.05202719
LOC_Os05g01970	LOC_Os09g33500	8.95744238
LOC_Os05g03820	LOC_Os05g04690	9.48121791
LOC_Os05g02310	LOC_Os07g49400	10.2642936
LOC_Os05g02940	LOC_Os07g36190	9.96640864
LOC_Os05g04490	LOC_Os07g06410	8.80378836
LOC_Os05g02310	LOC_Os08g40140	9.61379649

InteractorA	InteractorB	Z score
LOC_Os05g04470	LOC_Os11g01872	10.9117868
LOC_Os05g04490	LOC_Os08g25570	9.63904877
LOC_Os05g03820	LOC_Os07g48160	9.24728171
LOC_Os05g02940	LOC_Os05g50380	9.02867856
LOC_Os05g04490	LOC_Os11g26860	10.4193238
LOC_Os05g04490	LOC_Os12g34450	8.87521699
LOC_Os05g04490	LOC_Os06g12990	8.83506208
LOC_Os05g04470	LOC_Os06g45670	8.83952024
LOC_Os05g03820	LOC_Os07g01020	9.5693241
LOC_Os05g04470	LOC_Os12g40830	8.9082369
LOC_Os05g02310	LOC_Os06g35630	9.56631244
LOC_Os05g02310	LOC_Os05g51670	9.76185762
LOC_Os05g03820	LOC_Os10g33900	9.36278423
LOC_Os05g04340	LOC_Os08g04560	10.2161073
LOC_Os05g02310	LOC_Os06g19960	9.62618519
LOC_Os05g03820	LOC_Os05g37330	8.9387096
LOC_Os05g02310	LOC_Os10g27174	9.31437567
LOC_Os05g03820	LOC_Os05g41610	9.30800744
LOC_Os05g03820	LOC_Os07g44460	9.16312489
LOC_Os05g02940	LOC_Os05g05680	8.80872623
LOC_Os05g03480	LOC_Os12g22680	9.91038219
LOC_Os05g04340	LOC_Os07g14590	9.66760901
LOC_Os05g04450	LOC_Os09g31410	10.2871307
LOC_Os05g04470	LOC_Os12g40510	8.8982943
LOC_Os05g01810	LOC_Os12g38770	9.39931097
LOC_Os05g03820	LOC_Os05g23740	9.36661768
LOC_Os05g03480	LOC_Os11g08470	9.37216396
LOC_Os05g04490	LOC_Os05g28280	9.30028945
LOC_Os05g04340	LOC_Os08g04540	10.2286697
LOC_Os05g04470	LOC_Os11g08445	8.95367242
LOC_Os05g02940	LOC_Os07g26540	8.91985278
LOC_Os05g04340	LOC_Os06g50300	8.85980091
LOC_Os05g04340	LOC_Os10g08550	9.3171138
LOC_Os05g03480	LOC_Os12g38760	9.63391863
LOC_Os05g04450	LOC_Os05g34170	9.16265243
LOC_Os05g04450	LOC_Os10g28320	9.65696795
LOC_Os05g03480	LOC_Os07g03600	9.10538708
LOC_Os05g04450	LOC_Os10g08580	10.063604
LOC_Os05g04340	LOC_Os10g22450	9.07855668
LOC_Os05g03820	LOC_Os05g33130	8.85195428
LOC_Os05g04470	LOC_Os05g46550	8.87838833
LOC_Os05g04490	LOC_Os07g03690	9.19425889
LOC_Os05g02310	LOC_Os05g09500	9.41117901

InteractorA	InteractorB	Z score
LOC_Os05g04340	LOC_Os05g29880	9.37653788
LOC_Os05g04450	LOC_Os06g06300	9.52205823
LOC_Os05g04470	LOC_Os08g16910	9.13038307
LOC_Os05g04490	LOC_Os07g06970	9.27806263
LOC_Os05g04450	LOC_Os10g29470	9.6571281
LOC_Os05g02310	LOC_Os08g44530	10.2237209
LOC_Os05g04500	LOC_Os05g46550	8.95841
LOC_Os05g04490	LOC_Os05g11550	10.8865816
LOC_Os05g04490	LOC_Os09g10270	8.81682798
LOC_Os05g03480	LOC_Os06g37080	10.9957421
LOC_Os05g04500	LOC_Os06g21570	9.25876925
LOC_Os05g02940	LOC_Os10g21344	8.83540672
LOC_Os05g04520	LOC_Os05g51050	8.95524141
LOC_Os05g05670	LOC_Os07g07060	9.06863815
LOC_Os05g05830	LOC_Os07g01020	9.31620571
LOC_Os05g05800	LOC_Os11g01010	9.05480991
LOC_Os05g04520	LOC_Os10g41510	9.15018973
LOC_Os05g04510	LOC_Os07g36130	8.94671693
LOC_Os05g05620	LOC_Os11g25330	8.87101946
LOC_Os05g04520	LOC_Os06g04200	9.19581748
LOC_Os05g05620	LOC_Os11g31620	9.88302975
LOC_Os05g04690	LOC_Os11g32580	9.4651771
LOC_Os05g05620	LOC_Os09g31410	10.6274429
LOC_Os05g05620	LOC_Os08g42410	8.89264507
LOC_Os05g05800	LOC_Os09g24990	9.4888901
LOC_Os05g05670	LOC_Os12g31370	9.17992522
LOC_Os05g04690	LOC_Os07g47990	10.1901662
LOC_Os05g05800	LOC_Os05g41180	9.59885712
LOC_Os05g04520	LOC_Os06g35560	9.14620887
LOC_Os05g05670	LOC_Os05g39690	9.18873544
LOC_Os05g04690	LOC_Os06g12180	9.21353139
LOC_Os05g05620	LOC_Os06g11210	9.22545251
LOC_Os05g05680	LOC_Os07g43670	8.88476337
LOC_Os05g05620	LOC_Os08g36900	9.00088245
LOC_Os05g05670	LOC_Os06g36770	9.5024133
LOC_Os05g04500	LOC_Os07g28480	9.97267869
LOC_Os05g04690	LOC_Os06g37500	9.33499046
LOC_Os05g05620	LOC_Os05g49830	9.16057212
LOC_Os05g05800	LOC_Os10g21324	9.35768804
LOC_Os05g05620	LOC_Os12g38760	9.3413139
LOC_Os05g05620	LOC_Os09g25150	9.30099579
LOC_Os05g05800	LOC_Os07g19040	9.60606347
LOC_Os05g04690	LOC_Os07g48780	9.20513309

InteractorA	InteractorB	Z score
LOC_Os05g05830	LOC_Os06g50300	9.46087366
LOC_Os05g05670	LOC_Os08g34190	9.46899498
LOC_Os05g05680	LOC_Os08g34170	10.7855295
LOC_Os05g05800	LOC_Os07g20544	9.85248732
LOC_Os05g05670	LOC_Os08g28800	9.02811249
LOC_Os05g05680	LOC_Os09g32810	9.44602196
LOC_Os05g05680	LOC_Os07g23470	9.09910797
LOC_Os05g05680	LOC_Os06g36880	10.8304366
LOC_Os05g05800	LOC_Os05g42150	9.26047043
LOC_Os05g05670	LOC_Os11g39540	9.36201911
LOC_Os05g04690	LOC_Os11g32610	10.79213
LOC_Os05g05680	LOC_Os06g44620	9.07069169
LOC_Os05g04520	LOC_Os12g10730	8.99662934
LOC_Os05g04690	LOC_Os06g41810	9.23081351
LOC_Os05g04510	LOC_Os05g07880	8.9952546
LOC_Os05g05670	LOC_Os05g36270	9.31763208
LOC_Os05g04510	LOC_Os12g44000	9.51118476
LOC_Os05g05620	LOC_Os09g16910	10.1855192
LOC_Os05g04500	LOC_Os06g44620	8.85705047
LOC_Os05g05680	LOC_Os05g12180	11.1969094
LOC_Os05g05680	LOC_Os08g09250	9.94684321
LOC_Os05g04690	LOC_Os06g14510	12.1953937
LOC_Os05g05830	LOC_Os07g07240	8.97367623
LOC_Os05g04520	LOC_Os06g01850	10.2456739
LOC_Os05g04500	LOC_Os11g02440	11.6472129
LOC_Os05g04510	LOC_Os05g31110	8.92133475
LOC_Os05g04690	LOC_Os06g39740	8.91750925
LOC_Os05g04690	LOC_Os10g21250	9.20990448
LOC_Os05g05800	LOC_Os10g39170	9.60207564
LOC_Os05g05830	LOC_Os08g09210	10.0583323
LOC_Os05g05470	LOC_Os07g16970	9.90560705
LOC_Os05g05670	LOC_Os05g47980	8.80678818
LOC_Os05g04520	LOC_Os06g10950	8.80956283
LOC_Os05g05800	LOC_Os06g01850	9.12392688
LOC_Os05g04510	LOC_Os05g24580	8.91277346
LOC_Os05g04690	LOC_Os07g44790	9.67955446
LOC_Os05g04510	LOC_Os09g08072	9.4857185
LOC_Os05g04500	LOC_Os09g28770	8.94042134
LOC_Os05g05830	LOC_Os05g07880	11.4415466
LOC_Os05g04500	LOC_Os06g45710	9.93962672
LOC_Os05g05680	LOC_Os07g06440	9.6819527
LOC_Os05g04500	LOC_Os09g30418	9.34177278
LOC_Os05g04520	LOC_Os07g26540	9.61567146

InteractorA	InteractorB	Z score
LOC_Os05g05620	LOC_Os05g51480	8.84092243
LOC_Os05g04520	LOC_Os08g34290	8.91135745
LOC_Os05g04690	LOC_Os11g03980	8.82160905
LOC_Os05g05800	LOC_Os07g46830	10.0573518
LOC_Os05g05680	LOC_Os10g20910	9.00177957
LOC_Os05g04500	LOC_Os12g02370	9.5206341
LOC_Os05g04520	LOC_Os05g38760	9.08231252
LOC_Os05g05830	LOC_Os09g08120	9.09765573
LOC_Os05g05680	LOC_Os05g37390	10.8632075
LOC_Os05g04510	LOC_Os05g30480	10.4351425
LOC_Os05g04500	LOC_Os09g10270	9.22415718
LOC_Os05g05620	LOC_Os10g35110	9.18212825
LOC_Os05g04520	LOC_Os05g46270	9.34806696
LOC_Os05g04520	LOC_Os07g40580	9.04354599
LOC_Os05g04520	LOC_Os08g31060	9.98891294
LOC_Os05g04520	LOC_Os08g32850	9.15138012
LOC_Os05g05680	LOC_Os06g29220	10.2778994
LOC_Os05g05670	LOC_Os11g47760	8.84021774
LOC_Os05g05800	LOC_Os06g06730	8.84100651
LOC_Os05g04520	LOC_Os09g16910	8.99658389
LOC_Os05g05470	LOC_Os05g41230	9.30286942
LOC_Os05g05830	LOC_Os07g48880	10.2476182
LOC_Os05g05620	LOC_Os06g49970	9.89435928
LOC_Os05g04520	LOC_Os07g06970	8.97518887
LOC_Os05g04690	LOC_Os05g25550	9.04084997
LOC_Os05g04690	LOC_Os06g28550	10.967288
LOC_Os05g04690	LOC_Os11g08470	8.98024604
LOC_Os05g04510	LOC_Os07g47420	8.97659512
LOC_Os05g05800	LOC_Os05g10780	9.21658182
LOC_Os05g07690	LOC_Os08g32620	8.98296012
LOC_Os05g07880	LOC_Os07g47990	9.09461934
LOC_Os05g06750	LOC_Os10g26110	9.21598283
LOC_Os05g07880	LOC_Os12g16220	9.06973553
LOC_Os05g06750	LOC_Os05g41080	8.83840319
LOC_Os05g05830	LOC_Os10g37060	10.1643103
LOC_Os05g07880	LOC_Os09g31490	11.4486434
LOC_Os05g06300	LOC_Os07g05180	8.83693261
LOC_Os05g07720	LOC_Os05g38760	9.00680527
LOC_Os05g06300	LOC_Os09g15320	9.14203721
LOC_Os05g07090	LOC_Os05g41210	9.25265714
LOC_Os05g06970	LOC_Os07g39290	12.6040849
LOC_Os05g07880	LOC_Os11g31620	8.90013929
LOC_Os05g07690	LOC_Os08g34170	8.92295725

InteractorA	InteractorB	Z score
LOC_Os05g07880	LOC_Os11g31530	9.33983861
LOC_Os05g07720	LOC_Os08g41880	9.24758005
LOC_Os05g07690	LOC_Os08g43560	9.17164896
LOC_Os05g06970	LOC_Os12g21798	9.90574115
LOC_Os05g07880	LOC_Os07g38540	9.61505984
LOC_Os05g07880	LOC_Os09g32840	10.4753916
LOC_Os05g07880	LOC_Os08g34190	9.05797582
LOC_Os05g07880	LOC_Os05g33380	9.13849424
LOC_Os05g07090	LOC_Os08g04560	10.9305269
LOC_Os05g07690	LOC_Os05g47545	8.90979905
LOC_Os05g06450	LOC_Os07g46310	8.87381376
LOC_Os05g07720	LOC_Os05g35320	9.64122861
LOC_Os05g06750	LOC_Os05g45810	9.07690583
LOC_Os05g07880	LOC_Os10g42720	10.3104989
LOC_Os05g07090	LOC_Os10g22450	9.45165308
LOC_Os05g07090	LOC_Os12g12470	8.86332988
LOC_Os05g07720	LOC_Os05g42350	8.92061978
LOC_Os05g06300	LOC_Os06g14510	8.89908831
LOC_Os05g06450	LOC_Os06g01390	9.00484596
LOC_Os05g07690	LOC_Os07g42924	9.87749031
LOC_Os05g06450	LOC_Os06g39230	10.0147321
LOC_Os05g05830	LOC_Os09g33860	9.30874958
LOC_Os05g06300	LOC_Os06g09910	9.60126883
LOC_Os05g07880	LOC_Os06g37080	8.889806
LOC_Os05g06480	LOC_Os10g32550	9.0194128
LOC_Os05g06300	LOC_Os12g25120	9.20055331
LOC_Os05g07720	LOC_Os10g22450	10.0457612
LOC_Os05g07880	LOC_Os08g28820	8.85322926
LOC_Os05g07690	LOC_Os07g34589	10.2994862
LOC_Os05g07880	LOC_Os09g37100	9.11498178
LOC_Os05g06300	LOC_Os08g02700	9.92818475
LOC_Os05g06970	LOC_Os06g43640	9.16324811
LOC_Os05g07880	LOC_Os09g20260	9.31882601
LOC_Os05g06450	LOC_Os07g08170	11.4339884
LOC_Os05g07880	LOC_Os05g36010	9.24975208
LOC_Os05g06970	LOC_Os12g38760	9.94533192
LOC_Os05g07880	LOC_Os09g09230	8.89060871
LOC_Os05g07880	LOC_Os06g21820	9.27443931
LOC_Os05g06300	LOC_Os09g08910	9.04603911
LOC_Os05g06450	LOC_Os07g41050	9.12476779
LOC_Os05g06970	LOC_Os09g23560	9.21245812
LOC_Os05g06970	LOC_Os12g43100	8.85619707
LOC_Os05g06300	LOC_Os11g37890	10.0629861

InteractorA	InteractorB	Z score
LOC_Os05g06300	LOC_Os10g10434	8.82627547
LOC_Os05g06480	LOC_Os10g30840	8.96123945
LOC_Os05g07880	LOC_Os12g13800	9.92839304
LOC_Os05g06300	LOC_Os06g45070	9.69861882
LOC_Os05g06970	LOC_Os05g45810	9.01509829
LOC_Os05g07090	LOC_Os05g49830	11.4790344
LOC_Os05g06970	LOC_Os08g02410	9.43907914
LOC_Os05g06480	LOC_Os09g37100	9.63688568
LOC_Os05g06450	LOC_Os09g10200	9.1933821
LOC_Os05g06480	LOC_Os11g05880	9.48958746
LOC_Os05g06750	LOC_Os10g28360	9.17832667
LOC_Os05g07690	LOC_Os07g12730	9.08089518
LOC_Os05g05830	LOC_Os11g03980	9.14933402
LOC_Os05g06450	LOC_Os06g45070	8.85732612
LOC_Os05g06970	LOC_Os06g39875	9.79210927
LOC_Os05g07880	LOC_Os08g06060	9.58963821
LOC_Os05g07090	LOC_Os05g41640	9.44753071
LOC_Os05g06750	LOC_Os12g06620	8.82020705
LOC_Os05g06970	LOC_Os06g51150	9.71315797
LOC_Os05g07880	LOC_Os09g12590	10.2802367
LOC_Os05g06300	LOC_Os10g25950	9.20236786
LOC_Os05g06480	LOC_Os12g32240	9.49956751
LOC_Os05g06480	LOC_Os12g44020	9.18556741
LOC_Os05g07880	LOC_Os05g12190	9.08749563
LOC_Os05g07880	LOC_Os08g02410	10.5422804
LOC_Os05g06300	LOC_Os06g35520	9.43499032
LOC_Os05g05830	LOC_Os10g38234	9.13307401
LOC_Os05g06480	LOC_Os06g51150	9.65585317
LOC_Os05g06300	LOC_Os10g39680	9.15650678
LOC_Os05g06300	LOC_Os10g37210	9.69676582
LOC_Os05g07880	LOC_Os12g42884	9.24966825
LOC_Os05g07880	LOC_Os09g07460	9.71471505
LOC_Os05g06970	LOC_Os12g44010	9.56504828
LOC_Os05g06450	LOC_Os05g47540	9.07834808
LOC_Os05g06970	LOC_Os08g34280	8.86934747
LOC_Os05g07880	LOC_Os10g08580	10.1989728
LOC_Os05g06750	LOC_Os08g44350	9.78431383
LOC_Os05g06300	LOC_Os07g03710	9.5286622
LOC_Os05g06450	LOC_Os06g04000	9.2701891
LOC_Os05g07880	LOC_Os05g45420	9.19852196
LOC_Os05g06450	LOC_Os07g02210	9.23490193
LOC_Os05g06480	LOC_Os07g02210	8.85535271
LOC_Os05g07720	LOC_Os07g42490	8.8133667

InteractorA	InteractorB	Z score
LOC_Os05g07880	LOC_Os07g37790	10.5623353
LOC_Os05g05830	LOC_Os11g01872	9.77182368
LOC_Os05g07880	LOC_Os06g01590	9.64825376
LOC_Os05g06300	LOC_Os10g01570	8.91277097
LOC_Os05g06480	LOC_Os12g12580	9.220638
LOC_Os05g09500	LOC_Os05g38760	9.17379317
LOC_Os05g08100	LOC_Os06g02390	9.08324777
LOC_Os05g11710	LOC_Os08g34210	9.00380089
LOC_Os05g09440	LOC_Os08g34210	8.83139932
LOC_Os05g10780	LOC_Os05g31110	9.19604423
LOC_Os05g08100	LOC_Os07g08880	10.4121281
LOC_Os05g10780	LOC_Os11g03290	10.3562306
LOC_Os05g11730	LOC_Os08g10010	9.2368143
LOC_Os05g09500	LOC_Os10g25140	8.95294072
LOC_Os05g11550	LOC_Os05g22724	8.95904082
LOC_Os05g09490	LOC_Os08g04540	9.82561351
LOC_Os05g11710	LOC_Os10g02480	9.24245646
LOC_Os05g10780	LOC_Os09g10260	8.93862426
LOC_Os05g09490	LOC_Os11g14910	9.98177985
LOC_Os05g11550	LOC_Os08g44280	9.60214101
LOC_Os05g11710	LOC_Os06g46000	9.27590503
LOC_Os05g09500	LOC_Os05g51670	9.30870228
LOC_Os05g09500	LOC_Os08g38900	11.4965373
LOC_Os05g11710	LOC_Os07g46990	8.86908878
LOC_Os05g09500	LOC_Os12g43630	8.97518286
LOC_Os05g08100	LOC_Os08g39140	9.39042283
LOC_Os05g09440	LOC_Os06g44620	11.1788498
LOC_Os05g10780	LOC_Os09g31490	10.1276829
LOC_Os05g10780	LOC_Os07g03960	9.08445662
LOC_Os05g08960	LOC_Os10g32550	8.87233371
LOC_Os05g08430	LOC_Os07g29440	8.84725474
LOC_Os05g11710	LOC_Os10g23900	10.2039625
LOC_Os05g11730	LOC_Os06g04510	9.06005769
LOC_Os05g10780	LOC_Os07g09340	9.70577388
LOC_Os05g10780	LOC_Os07g34190	10.3511393
LOC_Os05g11550	LOC_Os06g35700	9.84150519
LOC_Os05g11730	LOC_Os09g24924	8.96989866
LOC_Os05g09500	LOC_Os07g44450	9.76085173
LOC_Os05g11550	LOC_Os05g44180	8.97275753
LOC_Os05g10780	LOC_Os07g05400	8.97586488
LOC_Os05g09490	LOC_Os06g12090	9.46428427
LOC_Os05g11710	LOC_Os09g04050	9.16957755
LOC_Os05g09490	LOC_Os07g31770	9.4913699

InteractorA	InteractorB	Z score
LOC_Os05g08100	LOC_Os08g34280	8.82717912
LOC_Os05g09440	LOC_Os06g39875	8.9676915
LOC_Os05g11710	LOC_Os07g22650	9.53780804
LOC_Os05g11550	LOC_Os06g04030	8.9202798
LOC_Os05g11550	LOC_Os06g37150	8.894886
LOC_Os05g09490	LOC_Os05g31040	8.95812113
LOC_Os05g08100	LOC_Os06g49970	9.20004798
LOC_Os05g09440	LOC_Os10g38140	8.82119679
LOC_Os05g09440	LOC_Os09g27420	10.1710422
LOC_Os05g09440	LOC_Os07g48040	9.75981957
LOC_Os05g11730	LOC_Os08g15292	9.30549658
LOC_Os05g11550	LOC_Os07g26540	9.28410519
LOC_Os05g11710	LOC_Os12g23170	9.19370626
LOC_Os05g11550	LOC_Os06g06980	11.1869543
LOC_Os05g11710	LOC_Os05g32140	9.12622241
LOC_Os05g09490	LOC_Os05g47540	8.95619818
LOC_Os05g09500	LOC_Os12g34874	10.6311369
LOC_Os05g08430	LOC_Os12g01922	9.00323956
LOC_Os05g10780	LOC_Os09g28400	10.4192971
LOC_Os05g11710	LOC_Os08g36900	9.21442914
LOC_Os05g11730	LOC_Os07g48430	8.8014267
LOC_Os05g11550	LOC_Os09g07830	9.13472896
LOC_Os05g09440	LOC_Os09g34214	9.39273593
LOC_Os05g09490	LOC_Os08g39300	8.99676349
LOC_Os05g11710	LOC_Os08g14760	8.93898398
LOC_Os05g09490	LOC_Os12g08280	10.5916859
LOC_Os05g09500	LOC_Os05g25490	9.7629638
LOC_Os05g09500	LOC_Os07g26690	8.95089926
LOC_Os05g10780	LOC_Os08g04460	9.14870181
LOC_Os05g11710	LOC_Os05g51480	8.90406062
LOC_Os05g09440	LOC_Os05g50890	10.7269483
LOC_Os05g11710	LOC_Os10g21248	8.80715925
LOC_Os05g09440	LOC_Os06g10330	10.8284046
LOC_Os05g10780	LOC_Os12g18880	9.9616903
LOC_Os05g11710	LOC_Os05g36270	8.93599419
LOC_Os05g11550	LOC_Os06g22140	11.5483433
LOC_Os05g08100	LOC_Os07g40290	9.65277679
LOC_Os05g10780	LOC_Os07g05180	10.8287995
LOC_Os05g09440	LOC_Os07g26640	9.37077434
LOC_Os05g09440	LOC_Os07g38540	9.97458781
LOC_Os05g11550	LOC_Os05g45810	8.94551368
LOC_Os05g10780	LOC_Os10g39880	10.8279511
LOC_Os05g09490	LOC_Os06g35540	8.92582239

InteractorA	InteractorB	Z score
LOC_Os05g09440	LOC_Os10g21250	8.88756979
LOC_Os05g09490	LOC_Os07g34520	9.0211772
LOC_Os05g08100	LOC_Os09g21770	9.4377764
LOC_Os05g09440	LOC_Os10g38229	8.86507549
LOC_Os05g09500	LOC_Os11g31530	8.87626614
LOC_Os05g11710	LOC_Os08g32620	8.95864719
LOC_Os05g10780	LOC_Os08g08070	8.89980105
LOC_Os05g09490	LOC_Os06g07210	9.54243111
LOC_Os05g10780	LOC_Os12g06620	10.0608199
LOC_Os05g09490	LOC_Os09g10230	9.94497528
LOC_Os05g09500	LOC_Os08g09200	10.2568602
LOC_Os05g08430	LOC_Os12g38760	9.96575112
LOC_Os05g10780	LOC_Os10g41190	8.96285682
LOC_Os05g10780	LOC_Os08g20730	9.00765135
LOC_Os05g11550	LOC_Os12g44350	9.38700239
LOC_Os05g11710	LOC_Os09g19560	8.88393765
LOC_Os05g09490	LOC_Os10g27050	9.53022418
LOC_Os05g08960	LOC_Os11g33240	9.18672169
LOC_Os05g09500	LOC_Os05g49880	9.35391987
LOC_Os05g10780	LOC_Os09g10300	9.05526941
LOC_Os05g11710	LOC_Os09g28400	9.55115965
LOC_Os05g09440	LOC_Os05g49200	8.87680252
LOC_Os05g09440	LOC_Os12g02980	8.94418667
LOC_Os05g09440	LOC_Os06g51060	8.84827009
LOC_Os05g11710	LOC_Os09g39380	8.817943
LOC_Os05g08430	LOC_Os07g44790	9.73728347
LOC_Os05g11550	LOC_Os09g32840	9.41300724
LOC_Os05g09440	LOC_Os06g07580	8.81291278
LOC_Os05g08960	LOC_Os07g31770	8.97595592
LOC_Os05g10780	LOC_Os12g24650	10.2358575
LOC_Os05g08430	LOC_Os08g15266	10.0254748
LOC_Os05g11550	LOC_Os08g39140	9.39696068
LOC_Os05g11710	LOC_Os05g42350	9.35557001
LOC_Os05g09440	LOC_Os11g01360	9.33798456
LOC_Os05g10780	LOC_Os12g07820	9.0400313
LOC_Os05g11550	LOC_Os07g23850	9.35182391
LOC_Os05g11550	LOC_Os07g48880	8.84790556
LOC_Os05g09490	LOC_Os11g32610	8.90929588
LOC_Os05g09500	LOC_Os10g39840	9.25960906
LOC_Os05g09500	LOC_Os09g10230	8.83982134
LOC_Os05g08430	LOC_Os09g24412	10.0254735
LOC_Os05g09490	LOC_Os11g34450	11.7716545
LOC_Os05g11550	LOC_Os05g36010	9.60228208

InteractorA	InteractorB	Z score
LOC_Os05g10780	LOC_Os05g44140	8.93658395
LOC_Os05g09490	LOC_Os06g47320	9.3717837
LOC_Os05g11710	LOC_Os10g40700	10.6767768
LOC_Os05g09500	LOC_Os06g07080	9.2593069
LOC_Os05g11550	LOC_Os11g30290	10.5799014
LOC_Os05g08430	LOC_Os07g25024	10.0254748
LOC_Os05g09440	LOC_Os06g51150	9.55861099
LOC_Os05g08100	LOC_Os07g49220	8.94037316
LOC_Os05g09490	LOC_Os11g32510	10.1672735
LOC_Os05g10780	LOC_Os10g09860	9.16899427
LOC_Os05g09440	LOC_Os05g41080	9.63043026
LOC_Os05g11710	LOC_Os06g04000	11.0436435
LOC_Os05g11550	LOC_Os05g49890	8.95177648
LOC_Os05g08430	LOC_Os10g21352	10.0254644
LOC_Os05g12190	LOC_Os05g45810	9.96423321
LOC_Os05g19670	LOC_Os07g05180	8.82136775
LOC_Os05g12190	LOC_Os06g10330	9.91653114
LOC_Os05g15520	LOC_Os08g08070	8.99066471
LOC_Os05g12190	LOC_Os06g11280	8.86913859
LOC_Os05g19380	LOC_Os07g46630	9.47774211
LOC_Os05g12180	LOC_Os10g23900	9.07194688
LOC_Os05g12210	LOC_Os11g01360	9.06559261
LOC_Os05g19670	LOC_Os08g27840	9.21651862
LOC_Os05g19380	LOC_Os07g35940	9.38007507
LOC_Os05g15520	LOC_Os08g29170	8.96600516
LOC_Os05g19150	LOC_Os11g03230	9.65111023
LOC_Os05g12190	LOC_Os12g42280	10.5160507
LOC_Os05g15520	LOC_Os12g10600	8.98545661
LOC_Os05g12240	LOC_Os10g38189	8.84193745
LOC_Os05g12210	LOC_Os08g40930	10.872465
LOC_Os05g12180	LOC_Os10g11810	8.82744853
LOC_Os05g12240	LOC_Os11g32610	9.19036803
LOC_Os05g15520	LOC_Os05g49770	9.1050922
LOC_Os05g19150	LOC_Os07g08170	11.6428198
LOC_Os05g20050	LOC_Os06g35814	9.08596668
LOC_Os05g12210	LOC_Os07g30970	9.38627626
LOC_Os05g12240	LOC_Os07g11440	10.6586715
LOC_Os05g11730	LOC_Os10g38160	9.01254602
LOC_Os05g20050	LOC_Os06g09450	8.80315738
LOC_Os05g12180	LOC_Os10g33900	9.85677884
LOC_Os05g15520	LOC_Os05g51570	10.0604988
LOC_Os05g12240	LOC_Os07g06440	9.43993549
LOC_Os05g19380	LOC_Os09g32620	10.1190562

InteractorA	InteractorB	Z score
LOC_Os05g12210	LOC_Os06g44620	9.42596712
LOC_Os05g15520	LOC_Os12g16290	9.26703182
LOC_Os05g19380	LOC_Os12g17540	9.21048426
LOC_Os05g15520	LOC_Os06g40170	8.97533271
LOC_Os05g12240	LOC_Os06g11210	9.97108093
LOC_Os05g19150	LOC_Os07g08500	9.19604035
LOC_Os05g11730	LOC_Os11g10480	8.92134252
LOC_Os05g12210	LOC_Os09g10300	13.1468763
LOC_Os05g12210	LOC_Os09g20090	9.73781942
LOC_Os05g15520	LOC_Os05g44140	10.7140792
LOC_Os05g12180	LOC_Os07g08170	10.4621954
LOC_Os05g12190	LOC_Os06g23870	8.80871506
LOC_Os05g19380	LOC_Os08g44270	9.52603638
LOC_Os05g12190	LOC_Os10g02480	10.1893743
LOC_Os05g12240	LOC_Os11g05470	11.4392141
LOC_Os05g19380	LOC_Os09g20260	9.03126682
LOC_Os05g12240	LOC_Os09g31490	9.46040849
LOC_Os05g20050	LOC_Os05g48980	8.82689624
LOC_Os05g22722	LOC_Os07g07060	9.0071317
LOC_Os05g19670	LOC_Os06g21570	9.0826262
LOC_Os05g12180	LOC_Os10g41190	9.02141588
LOC_Os05g19150	LOC_Os12g08280	9.07151826
LOC_Os05g19150	LOC_Os05g44760	8.80404294
LOC_Os05g12190	LOC_Os11g01010	9.27809096
LOC_Os05g12240	LOC_Os06g13450	8.9397772
LOC_Os05g20050	LOC_Os05g46550	9.17541976
LOC_Os05g12190	LOC_Os11g37550	9.47167597
LOC_Os05g12240	LOC_Os06g23780	10.8764622
LOC_Os05g19380	LOC_Os07g38730	9.65407598
LOC_Os05g12180	LOC_Os07g44740	9.98268821
LOC_Os05g19380	LOC_Os09g32810	9.41393896
LOC_Os05g12210	LOC_Os06g35530	11.0068563
LOC_Os05g12180	LOC_Os06g10330	8.98194109
LOC_Os05g19380	LOC_Os07g34190	9.38597374
LOC_Os05g19380	LOC_Os12g42876	8.83208761
LOC_Os05g12210	LOC_Os05g33140	10.0870497
LOC_Os05g19670	LOC_Os05g45420	10.0233899
LOC_Os05g19380	LOC_Os09g28420	8.93273818
LOC_Os05g19380	LOC_Os05g38530	8.96535627
LOC_Os05g19150	LOC_Os06g51150	9.71142114
LOC_Os05g19380	LOC_Os09g38030	9.40546147
LOC_Os05g12210	LOC_Os11g26910	9.17996581
LOC_Os05g19380	LOC_Os07g26900	10.0638755

InteractorA	InteractorB	Z score
LOC_Os05g19380	LOC_Os09g31410	9.01085788
LOC_Os05g15520	LOC_Os06g40940	9.09870852
LOC_Os05g20050	LOC_Os07g49220	10.9898935
LOC_Os05g20050	LOC_Os07g12730	9.2545798
LOC_Os05g12210	LOC_Os09g10260	9.7551608
LOC_Os05g15520	LOC_Os07g37790	9.08796536
LOC_Os05g19380	LOC_Os08g32620	9.29069184
LOC_Os05g19670	LOC_Os06g46940	9.77027838
LOC_Os05g12210	LOC_Os10g42940	9.26876024
LOC_Os05g19380	LOC_Os06g51050	10.0638004
LOC_Os05g19150	LOC_Os06g11200	9.16184418
LOC_Os05g12240	LOC_Os06g37150	8.90120897
LOC_Os05g19380	LOC_Os07g49120	9.42998728
LOC_Os05g12210	LOC_Os08g44960	8.87372673
LOC_Os05g12240	LOC_Os12g23170	10.0894532
LOC_Os05g12190	LOC_Os05g45590	8.8262908
LOC_Os05g12180	LOC_Os11g28340	8.96755681
LOC_Os05g15520	LOC_Os10g03540	9.17197334
LOC_Os05g19380	LOC_Os07g47490	9.71997747
LOC_Os05g15520	LOC_Os06g35590	9.05230025
LOC_Os05g12180	LOC_Os08g36320	9.01124138
LOC_Os05g19670	LOC_Os08g33820	9.00029376
LOC_Os05g19380	LOC_Os05g41080	8.82106012
LOC_Os05g19380	LOC_Os09g25150	10.6042738
LOC_Os05g12190	LOC_Os09g39400	9.30686266
LOC_Os05g19380	LOC_Os08g04560	9.48163216
LOC_Os05g19380	LOC_Os11g36719	8.9909322
LOC_Os05g15520	LOC_Os05g45590	9.03484877
LOC_Os05g12210	LOC_Os09g23530	9.54285442
LOC_Os05g12180	LOC_Os07g34190	11.2151519
LOC_Os05g19380	LOC_Os09g36900	11.1429177
LOC_Os05g12240	LOC_Os06g23870	9.67080963
LOC_Os05g15520	LOC_Os08g06060	8.92857812
LOC_Os05g19380	LOC_Os07g40290	9.14468948
LOC_Os05g19150	LOC_Os09g20260	8.80742205
LOC_Os05g19380	LOC_Os05g42150	9.36685433
LOC_Os05g12240	LOC_Os08g09250	10.3724448
LOC_Os05g12180	LOC_Os07g43250	10.0199534
LOC_Os05g15520	LOC_Os12g02980	9.27621048
LOC_Os05g20050	LOC_Os06g01850	9.93249729
LOC_Os05g12180	LOC_Os08g38300	9.00671224
LOC_Os05g25850	LOC_Os11g19320	9.61747922
LOC_Os05g26890	LOC_Os11g25330	9.62275581

InteractorA	InteractorB	Z score
LOC_Os05g25490	LOC_Os06g36840	9.42806581
LOC_Os05g27950	LOC_Os05g38530	8.94014362
LOC_Os05g25490	LOC_Os09g37100	9.62377046
LOC_Os05g25490	LOC_Os08g34280	9.82959345
LOC_Os05g23740	LOC_Os12g25700	10.3520098
LOC_Os05g25490	LOC_Os09g31120	10.0917751
LOC_Os05g22722	LOC_Os12g38760	9.62748404
LOC_Os05g25550	LOC_Os08g03040	8.87557861
LOC_Os05g27940	LOC_Os10g28360	8.89810138
LOC_Os05g22722	LOC_Os07g38540	10.1302762
LOC_Os05g27940	LOC_Os09g24990	9.58668048
LOC_Os05g22724	LOC_Os10g27050	9.2390389
LOC_Os05g23860	LOC_Os09g32800	9.52371959
LOC_Os05g25490	LOC_Os05g36010	10.1851422
LOC_Os05g25490	LOC_Os07g38860	9.19912445
LOC_Os05g25490	LOC_Os09g24924	8.89893299
LOC_Os05g23740	LOC_Os09g16910	9.88606259
LOC_Os05g26890	LOC_Os08g15292	8.80554458
LOC_Os05g24580	LOC_Os08g40140	9.50367846
LOC_Os05g23860	LOC_Os11g26910	9.23107856
LOC_Os05g26890	LOC_Os11g07440	8.98951009
LOC_Os05g24580	LOC_Os05g45220	9.31578087
LOC_Os05g23740	LOC_Os10g27050	9.416174
LOC_Os05g25850	LOC_Os11g33240	9.23989245
LOC_Os05g25490	LOC_Os07g07709	9.46746844
LOC_Os05g25490	LOC_Os07g05160	9.47227406
LOC_Os05g22724	LOC_Os09g31486	9.23145395
LOC_Os05g25490	LOC_Os05g26890	9.31489314
LOC_Os05g25850	LOC_Os06g04030	10.1402595
LOC_Os05g24580	LOC_Os12g25120	10.2305116
LOC_Os05g22722	LOC_Os09g08072	9.19014086
LOC_Os05g24580	LOC_Os05g46860	9.54790464
LOC_Os05g23860	LOC_Os05g44050	9.60933482
LOC_Os05g25550	LOC_Os10g41550	10.6256471
LOC_Os05g25490	LOC_Os12g07980	9.43480368
LOC_Os05g25550	LOC_Os12g12590	10.0547602
LOC_Os05g27940	LOC_Os11g03290	9.21529107
LOC_Os05g25490	LOC_Os12g10720	8.98478683
LOC_Os05g22724	LOC_Os10g25130	9.00052088
LOC_Os05g24580	LOC_Os05g33130	8.83558364
LOC_Os05g25550	LOC_Os10g41510	9.18112848
LOC_Os05g25490	LOC_Os11g47560	10.578687
LOC_Os05g25850	LOC_Os07g46310	9.02475487

InteractorA	InteractorB	Z score
LOC_Os05g22722	LOC_Os07g35880	8.83894616
LOC_Os05g25490	LOC_Os07g02210	8.99735195
LOC_Os05g23860	LOC_Os11g32540	8.89149021
LOC_Os05g24580	LOC_Os11g08330	9.19099434
LOC_Os05g22722	LOC_Os10g21248	10.052107
LOC_Os05g25550	LOC_Os09g39380	8.84630545
LOC_Os05g24580	LOC_Os07g26640	8.98557553
LOC_Os05g22724	LOC_Os10g34760	9.71685411
LOC_Os05g25490	LOC_Os10g40730	9.48402416
LOC_Os05g22724	LOC_Os12g12590	9.23381575
LOC_Os05g24580	LOC_Os05g41180	9.76302249
LOC_Os05g23860	LOC_Os05g41900	9.07403409
LOC_Os05g24580	LOC_Os07g26150	9.21578478
LOC_Os05g26890	LOC_Os07g31270	9.87749741
LOC_Os05g25490	LOC_Os06g36770	8.92170907
LOC_Os05g27940	LOC_Os08g02700	10.3828171
LOC_Os05g27940	LOC_Os12g44350	9.26317769
LOC_Os05g25490	LOC_Os06g05690	9.53480181
LOC_Os05g25490	LOC_Os10g25140	10.1878877
LOC_Os05g23740	LOC_Os11g16590	8.90881114
LOC_Os05g24580	LOC_Os10g01080	10.7818493
LOC_Os05g23740	LOC_Os11g08445	10.039113
LOC_Os05g24580	LOC_Os06g21570	10.0622372
LOC_Os05g27940	LOC_Os08g41340	8.96340459
LOC_Os05g25490	LOC_Os12g40550	10.3466715
LOC_Os05g26890	LOC_Os05g47640	9.18857184
LOC_Os05g26890	LOC_Os10g41510	10.3701237
LOC_Os05g23860	LOC_Os05g48290	8.95796727
LOC_Os05g24580	LOC_Os08g17680	8.99530162
LOC_Os05g25850	LOC_Os11g32650	8.88142191
LOC_Os05g24580	LOC_Os09g31506	8.89732954
LOC_Os05g22724	LOC_Os12g24650	8.89843554
LOC_Os05g25490	LOC_Os07g03910	8.86732828
LOC_Os05g23860	LOC_Os06g06320	9.63402882
LOC_Os05g25490	LOC_Os10g10434	9.38810017
LOC_Os05g23740	LOC_Os08g43170	9.22531896
LOC_Os05g26890	LOC_Os10g01080	9.41155552
LOC_Os05g25490	LOC_Os09g39380	10.6009918
LOC_Os05g22724	LOC_Os10g27190	9.00603109
LOC_Os05g24580	LOC_Os05g51570	10.2669929
LOC_Os05g24580	LOC_Os06g15990	11.0095449
LOC_Os05g23860	LOC_Os12g43100	9.11489791
LOC_Os05g25490	LOC_Os10g38590	8.91180862

InteractorA	InteractorB	Z score
LOC_Os05g24580	LOC_Os06g01850	8.93152085
LOC_Os05g27940	LOC_Os06g06980	9.43645933
LOC_Os05g26890	LOC_Os06g42130	9.53698643
LOC_Os05g25850	LOC_Os07g34140	10.527794
LOC_Os05g24580	LOC_Os06g45100	9.2481348
LOC_Os05g23740	LOC_Os12g42884	9.30560638
LOC_Os05g23740	LOC_Os09g25150	9.89597692
LOC_Os05g25490	LOC_Os06g14510	11.1559508
LOC_Os05g26890	LOC_Os09g10200	8.84489767
LOC_Os05g23740	LOC_Os07g26150	9.6904099
LOC_Os05g23740	LOC_Os07g08660	8.83526109
LOC_Os05g28180	LOC_Os09g32620	8.81139123
LOC_Os05g27950	LOC_Os11g34570	9.76063205
LOC_Os05g29880	LOC_Os09g39500	9.74567014
LOC_Os05g28280	LOC_Os09g39570	8.80484798
LOC_Os05g30480	LOC_Os10g29620	8.86418026
LOC_Os05g30480	LOC_Os10g38160	12.4604753
LOC_Os05g30530	LOC_Os08g34280	8.82113205
LOC_Os05g28180	LOC_Os08g04180	9.06291883
LOC_Os05g28180	LOC_Os10g07229	9.43786727
LOC_Os05g30480	LOC_Os10g27174	8.81329887
LOC_Os05g31040	LOC_Os07g34520	8.9211008
LOC_Os05g28940	LOC_Os11g05880	8.90452522
LOC_Os05g30410	LOC_Os10g21250	8.98967868
LOC_Os05g30530	LOC_Os08g39300	9.12204836
LOC_Os05g28180	LOC_Os07g26540	10.0919912
LOC_Os05g31020	LOC_Os11g40140	8.80004503
LOC_Os05g31040	LOC_Os08g10608	8.86671434
LOC_Os05g31020	LOC_Os07g48050	9.15877114
LOC_Os05g31020	LOC_Os05g44340	9.44868488
LOC_Os05g29880	LOC_Os12g12470	9.35346865
LOC_Os05g28280	LOC_Os06g30370	10.3211683
LOC_Os05g31020	LOC_Os06g30970	9.11163651
LOC_Os05g27950	LOC_Os08g40140	10.1964266
LOC_Os05g28280	LOC_Os08g15292	9.23433017
LOC_Os05g29880	LOC_Os06g35700	10.1922394
LOC_Os05g31040	LOC_Os11g07020	10.2928858
LOC_Os05g28180	LOC_Os09g07460	9.77934075
LOC_Os05g29880	LOC_Os09g07460	9.17839678
LOC_Os05g29880	LOC_Os08g42730	9.83762113
LOC_Os05g29880	LOC_Os08g28800	9.07067722
LOC_Os05g31040	LOC_Os08g09210	9.15494203
LOC_Os05g30530	LOC_Os10g30840	9.60045007

InteractorA	InteractorB	Z score
LOC_Os05g28940	LOC_Os10g40720	9.37672777
LOC_Os05g28280	LOC_Os05g39690	9.48999988
LOC_Os05g28940	LOC_Os05g31110	8.84098037
LOC_Os05g28280	LOC_Os09g32640	9.65363847
LOC_Os05g27950	LOC_Os12g03816	9.05729104
LOC_Os05g28280	LOC_Os12g13810	9.01576978
LOC_Os05g27950	LOC_Os08g42730	9.12159895
LOC_Os05g31020	LOC_Os11g32650	9.10576492
LOC_Os05g28280	LOC_Os06g37150	9.77182526
LOC_Os05g28280	LOC_Os11g10510	8.87057232
LOC_Os05g28180	LOC_Os07g38910	9.29467202
LOC_Os05g30530	LOC_Os10g21352	9.51726037
LOC_Os05g29880	LOC_Os11g07020	9.19682833
LOC_Os05g31020	LOC_Os05g48510	8.92793522
LOC_Os05g28180	LOC_Os06g51050	9.12827145
LOC_Os05g30530	LOC_Os05g48510	9.03626852
LOC_Os05g28180	LOC_Os10g38740	10.3017173
LOC_Os05g28180	LOC_Os08g09210	9.51962565
LOC_Os05g28180	LOC_Os10g38229	9.27108884
LOC_Os05g31040	LOC_Os12g08270	8.93134884
LOC_Os05g29880	LOC_Os10g37210	9.97678522
LOC_Os05g28180	LOC_Os05g33140	9.19947634
LOC_Os05g29880	LOC_Os08g40140	9.30779425
LOC_Os05g31020	LOC_Os05g39690	8.80211924
LOC_Os05g28280	LOC_Os10g02380	9.26677843
LOC_Os05g28940	LOC_Os07g32800	8.98887809
LOC_Os05g31020	LOC_Os12g38770	10.8857489
LOC_Os05g28280	LOC_Os07g38730	9.42733966
LOC_Os05g29880	LOC_Os07g01760	9.48250654
LOC_Os05g28180	LOC_Os07g22950	8.95768678
LOC_Os05g27950	LOC_Os12g13320	9.81157532
LOC_Os05g29880	LOC_Os12g25710	10.1547198
LOC_Os05g28180	LOC_Os07g46280	9.91006917
LOC_Os05g29880	LOC_Os07g42960	9.09678793
LOC_Os05g30530	LOC_Os08g15266	9.51726037
LOC_Os05g30530	LOC_Os09g24412	9.51726037
LOC_Os05g29880	LOC_Os11g08460	9.06175505
LOC_Os05g30480	LOC_Os05g33130	9.91064847
LOC_Os05g30480	LOC_Os05g30530	9.53162106
LOC_Os05g28280	LOC_Os07g26540	8.91239922
LOC_Os05g28180	LOC_Os05g38550	9.01675378
LOC_Os05g30410	LOC_Os06g42130	9.49149339
LOC_Os05g29880	LOC_Os08g29370	8.88031744

InteractorA	InteractorB	Z score
LOC_Os05g31040	LOC_Os09g10230	8.85494874
LOC_Os05g30480	LOC_Os07g43470	9.52375708
LOC_Os05g31040	LOC_Os06g09450	9.16379165
LOC_Os05g30410	LOC_Os06g11210	8.8309327
LOC_Os05g28180	LOC_Os08g42560	9.87429968
LOC_Os05g31020	LOC_Os06g03720	9.24327671
LOC_Os05g27950	LOC_Os11g03290	8.9560916
LOC_Os05g31040	LOC_Os09g33850	9.34093719
LOC_Os05g29880	LOC_Os11g19220	9.21063605
LOC_Os05g27950	LOC_Os07g38430	9.01088147
LOC_Os05g31020	LOC_Os07g35940	9.45885614
LOC_Os05g31040	LOC_Os07g08170	8.89429737
LOC_Os05g30530	LOC_Os06g36820	10.8247626
LOC_Os05g29880	LOC_Os08g44270	9.43786654
LOC_Os05g27950	LOC_Os11g08470	8.81356534
LOC_Os05g29880	LOC_Os06g42560	9.60402572
LOC_Os05g29880	LOC_Os10g28350	9.03956732
LOC_Os05g29880	LOC_Os05g50890	9.15063736
LOC_Os05g28940	LOC_Os09g25390	9.52851842
LOC_Os05g28180	LOC_Os07g07320	10.4753873
LOC_Os05g28280	LOC_Os10g26110	9.4151825
LOC_Os05g30530	LOC_Os12g31370	9.07139632
LOC_Os05g29880	LOC_Os05g41900	9.02357892
LOC_Os05g31040	LOC_Os11g41610	9.25648682
LOC_Os05g31040	LOC_Os08g09950	10.1391819
LOC_Os05g29880	LOC_Os10g38580	8.99659859
LOC_Os05g29880	LOC_Os11g32650	9.3019099
LOC_Os05g30530	LOC_Os06g11260	10.1987419
LOC_Os05g29880	LOC_Os10g38710	8.85136623
LOC_Os05g28180	LOC_Os09g33860	9.32305003
LOC_Os05g31040	LOC_Os11g14220	10.7800903
LOC_Os05g28280	LOC_Os07g05820	9.04205173
LOC_Os05g28280	LOC_Os12g18900	10.7145043
LOC_Os05g29880	LOC_Os11g31530	8.87576499
LOC_Os05g30480	LOC_Os07g28480	9.52870467
LOC_Os05g30480	LOC_Os11g08470	10.459282
LOC_Os05g30480	LOC_Os07g40580	9.76906278
LOC_Os05g30480	LOC_Os10g26390	9.22480103
LOC_Os05g27950	LOC_Os06g49970	9.72815461
LOC_Os05g30530	LOC_Os09g10270	8.87196557
LOC_Os05g27950	LOC_Os09g23530	8.87790358
LOC_Os05g28280	LOC_Os05g35330	9.78704903
LOC_Os05g28940	LOC_Os05g37390	11.5903026

InteractorA	InteractorB	Z score
LOC_Os05g29880	LOC_Os11g25220	9.02143006
LOC_Os05g29880	LOC_Os06g36880	9.16811131
LOC_Os05g29880	LOC_Os07g43670	8.86237843
LOC_Os05g29880	LOC_Os12g17910	8.82747473
LOC_Os05g28180	LOC_Os07g01020	10.6154392
LOC_Os05g29880	LOC_Os06g35814	9.75431388
LOC_Os05g30480	LOC_Os08g44270	8.87714185
LOC_Os05g28280	LOC_Os05g38530	11.1860146
LOC_Os05g28180	LOC_Os05g45810	8.97860064
LOC_Os05g30530	LOC_Os07g31750	9.47087822
LOC_Os05g31040	LOC_Os09g07830	10.3380473
LOC_Os05g28180	LOC_Os11g32540	9.44175524
LOC_Os05g30480	LOC_Os05g35320	9.79286653
LOC_Os05g28940	LOC_Os12g16240	9.217918
LOC_Os05g31020	LOC_Os05g44760	9.09945317
LOC_Os05g30480	LOC_Os10g31940	9.87388019
LOC_Os05g30480	LOC_Os06g35540	9.61209982
LOC_Os05g29880	LOC_Os05g44050	8.83123147
LOC_Os05g31040	LOC_Os06g35650	9.24827715
LOC_Os05g28180	LOC_Os12g44350	11.1860022
LOC_Os05g28280	LOC_Os11g08460	9.64937781
LOC_Os05g29880	LOC_Os12g16240	9.49445632
LOC_Os05g27950	LOC_Os10g21326	9.78723669
LOC_Os05g30410	LOC_Os09g31486	9.28607124
LOC_Os05g29880	LOC_Os06g48310	8.97511071
LOC_Os05g30530	LOC_Os07g25024	9.51726037
LOC_Os05g28280	LOC_Os05g46040	9.77014436
LOC_Os05g28280	LOC_Os10g21268	9.78702304
LOC_Os05g29880	LOC_Os11g43200	10.0611869
LOC_Os05g29880	LOC_Os06g04620	9.1960955
LOC_Os05g28940	LOC_Os07g07719	9.29218875
LOC_Os05g28180	LOC_Os11g32650	9.49167133
LOC_Os05g31140	LOC_Os08g44960	10.6469655
LOC_Os05g33130	LOC_Os12g16250	9.28873677
LOC_Os05g32140	LOC_Os10g27190	8.82211303
LOC_Os05g33140	LOC_Os05g34540	9.10229286
LOC_Os05g33130	LOC_Os08g43190	9.17937918
LOC_Os05g33140	LOC_Os11g47570	10.3506905
LOC_Os05g33140	LOC_Os11g32260	8.85918415
LOC_Os05g33240	LOC_Os07g26900	9.20901925
LOC_Os05g33240	LOC_Os06g11200	9.50211292
LOC_Os05g33410	LOC_Os07g34520	8.96724223
LOC_Os05g31110	LOC_Os08g33710	11.5672812

InteractorA	InteractorB	Z score
LOC_Os05g33150	LOC_Os12g34450	9.02535625
LOC_Os05g33240	LOC_Os11g37960	9.32946218
LOC_Os05g33380	LOC_Os12g39630	8.8460657
LOC_Os05g33130	LOC_Os11g16590	9.54357025
LOC_Os05g33380	LOC_Os06g34690	10.0056991
LOC_Os05g33400	LOC_Os12g24650	9.06038407
LOC_Os05g31140	LOC_Os09g34970	9.18815412
LOC_Os05g33130	LOC_Os07g46310	9.89288147
LOC_Os05g31110	LOC_Os10g25140	10.4440204
LOC_Os05g33150	LOC_Os10g28320	9.07902207
LOC_Os05g32140	LOC_Os08g28800	9.92710382
LOC_Os05g33130	LOC_Os07g44590	8.98710978
LOC_Os05g33130	LOC_Os11g28340	9.22739121
LOC_Os05g33140	LOC_Os06g22140	9.75118725
LOC_Os05g31140	LOC_Os08g35740	11.2400684
LOC_Os05g33240	LOC_Os10g21326	9.22941297
LOC_Os05g33140	LOC_Os11g16590	8.93410388
LOC_Os05g33130	LOC_Os11g10480	9.32667204
LOC_Os05g33140	LOC_Os12g05410	9.52761541
LOC_Os05g31140	LOC_Os12g10560	10.2051975
LOC_Os05g33130	LOC_Os09g07830	9.46830163
LOC_Os05g32140	LOC_Os06g35730	9.12623874
LOC_Os05g31140	LOC_Os11g26910	9.5558518
LOC_Os05g33240	LOC_Os07g13980	9.06465348
LOC_Os05g33380	LOC_Os10g26390	8.84305592
LOC_Os05g33380	LOC_Os10g20910	8.81411027
LOC_Os05g33140	LOC_Os07g05400	9.06133027
LOC_Os05g33400	LOC_Os06g02390	9.59815662
LOC_Os05g33400	LOC_Os06g36770	9.55108049
LOC_Os05g33130	LOC_Os10g22070	9.17452505
LOC_Os05g33380	LOC_Os12g13390	9.0794544
LOC_Os05g33150	LOC_Os11g01872	9.11338466
LOC_Os05g31140	LOC_Os07g48160	9.06763024
LOC_Os05g31140	LOC_Os05g46860	9.1297748
LOC_Os05g33130	LOC_Os06g11280	9.87658593
LOC_Os05g33150	LOC_Os07g05940	9.20615648
LOC_Os05g33130	LOC_Os08g29370	9.43098605
LOC_Os05g31140	LOC_Os05g45810	9.75740923
LOC_Os05g33140	LOC_Os11g10510	8.80805033
LOC_Os05g33130	LOC_Os09g27750	8.97428626
LOC_Os05g33140	LOC_Os05g47540	9.51705432
LOC_Os05g33150	LOC_Os07g41050	8.87631514
LOC_Os05g31140	LOC_Os10g38140	9.17392729

InteractorA	InteractorB	Z score
LOC_Os05g31140	LOC_Os12g32240	9.32355676
LOC_Os05g31140	LOC_Os05g33400	9.50599012
LOC_Os05g33150	LOC_Os07g44790	9.94273313
LOC_Os05g33130	LOC_Os08g32850	9.59831304
LOC_Os05g33410	LOC_Os07g43390	9.79969842
LOC_Os05g33400	LOC_Os11g32650	9.12240254
LOC_Os05g31110	LOC_Os12g25690	9.87171184
LOC_Os05g33240	LOC_Os07g30970	9.52858262
LOC_Os05g33130	LOC_Os12g44020	8.94856368
LOC_Os05g33130	LOC_Os10g37060	9.37188047
LOC_Os05g31140	LOC_Os12g16200	9.17632457
LOC_Os05g31110	LOC_Os07g47490	9.86881132
LOC_Os05g33400	LOC_Os06g51150	9.55711542
LOC_Os05g33410	LOC_Os05g38560	9.05333724
LOC_Os05g33400	LOC_Os07g44440	9.50803308
LOC_Os05g33410	LOC_Os06g01360	9.01413176
LOC_Os05g31110	LOC_Os10g38340	9.10031252
LOC_Os05g33140	LOC_Os06g27770	9.62234209
LOC_Os05g33240	LOC_Os07g08840	10.2515765
LOC_Os05g33130	LOC_Os08g31060	9.01753347
LOC_Os05g33380	LOC_Os07g34580	8.85785426
LOC_Os05g33240	LOC_Os09g27750	9.31392404
LOC_Os05g31140	LOC_Os12g44020	9.37356394
LOC_Os05g33130	LOC_Os09g25390	10.2713873
LOC_Os05g33380	LOC_Os06g48160	9.89366953
LOC_Os05g31110	LOC_Os09g23530	8.87903833
LOC_Os05g33140	LOC_Os11g32650	10.2163103
LOC_Os05g31110	LOC_Os09g10270	9.79718312
LOC_Os05g33730	LOC_Os10g08670	8.83414853
LOC_Os05g35290	LOC_Os08g36900	10.4792596
LOC_Os05g35320	LOC_Os09g15790	8.91224699
LOC_Os05g34540	LOC_Os12g25710	8.94890741
LOC_Os05g33570	LOC_Os07g29440	9.60548179
LOC_Os05g34540	LOC_Os11g26860	10.254425
LOC_Os05g33570	LOC_Os11g18870	9.07238752
LOC_Os05g34540	LOC_Os12g34380	9.58011868
LOC_Os05g35330	LOC_Os07g43470	9.86212499
LOC_Os05g35320	LOC_Os05g44140	8.80880694
LOC_Os05g33730	LOC_Os11g05730	9.52246675
LOC_Os05g34770	LOC_Os06g04200	9.12679583
LOC_Os05g34770	LOC_Os08g44530	9.09335915
LOC_Os05g35770	LOC_Os09g17740	9.54483401
LOC_Os05g34540	LOC_Os09g38030	9.01337727

InteractorA	InteractorB	Z score
LOC_Os05g35320	LOC_Os07g05180	10.9155575
LOC_Os05g35330	LOC_Os09g28460	9.09103268
LOC_Os05g35400	LOC_Os07g38430	8.85155736
LOC_Os05g35320	LOC_Os08g09200	9.32359219
LOC_Os05g34540	LOC_Os09g15400	9.78358817
LOC_Os05g34540	LOC_Os10g38690	9.72802945
LOC_Os05g34540	LOC_Os11g41610	9.32528594
LOC_Os05g35400	LOC_Os05g47540	9.12191587
LOC_Os05g33570	LOC_Os09g10200	10.5398692
LOC_Os05g34170	LOC_Os07g47490	9.31849255
LOC_Os05g35770	LOC_Os08g09770	9.30116497
LOC_Os05g35330	LOC_Os10g32550	8.88791156
LOC_Os05g34540	LOC_Os10g28350	9.64325627
LOC_Os05g35330	LOC_Os10g26130	9.21134016
LOC_Os05g34540	LOC_Os08g43560	9.91577686
LOC_Os05g35290	LOC_Os07g02350	10.4530846
LOC_Os05g34540	LOC_Os06g46940	9.43929173
LOC_Os05g35320	LOC_Os12g06620	8.93427056
LOC_Os05g35400	LOC_Os05g38530	8.92690645
LOC_Os05g34170	LOC_Os10g30200	9.26941267
LOC_Os05g35400	LOC_Os06g35540	9.08851046
LOC_Os05g34170	LOC_Os10g28050	9.24379559
LOC_Os05g34540	LOC_Os10g22450	8.8147776
LOC_Os05g35290	LOC_Os11g48110	15.6563609
LOC_Os05g33730	LOC_Os05g36280	9.52247566
LOC_Os05g35330	LOC_Os07g42950	9.2923256
LOC_Os05g33730	LOC_Os06g06460	9.52247566
LOC_Os05g33730	LOC_Os12g38770	9.19576656
LOC_Os05g35330	LOC_Os11g03290	9.58652964
LOC_Os05g33730	LOC_Os12g12580	13.3765537
LOC_Os05g35770	LOC_Os08g33710	9.8523325
LOC_Os05g34770	LOC_Os07g05800	9.50315288
LOC_Os05g34170	LOC_Os12g17910	8.93735794
LOC_Os05g35770	LOC_Os08g16910	8.9935451
LOC_Os05g35320	LOC_Os12g37360	9.30940549
LOC_Os05g35330	LOC_Os09g15320	9.50336223
LOC_Os05g34170	LOC_Os08g06100	9.88122828
LOC_Os05g35330	LOC_Os11g32260	8.99125062
LOC_Os05g35330	LOC_Os06g12090	9.22536408
LOC_Os05g34170	LOC_Os07g30200	10.9894242
LOC_Os05g35400	LOC_Os08g06060	8.83603471
LOC_Os05g33730	LOC_Os12g12560	9.00311667
LOC_Os05g35770	LOC_Os09g37100	9.27191455

InteractorA	InteractorB	Z score
LOC_Os05g35330	LOC_Os12g31640	9.23914745
LOC_Os05g35400	LOC_Os12g34874	10.4984777
LOC_Os05g33730	LOC_Os09g34214	10.034232
LOC_Os05g35770	LOC_Os09g25150	8.87485739
LOC_Os05g34770	LOC_Os09g39380	9.85410433
LOC_Os05g35290	LOC_Os07g22930	9.18489927
LOC_Os05g35320	LOC_Os09g36800	8.83244961
LOC_Os05g35400	LOC_Os06g39230	9.67414843
LOC_Os05g35400	LOC_Os07g38890	8.87490729
LOC_Os05g35290	LOC_Os08g42000	9.43272365
LOC_Os05g34170	LOC_Os06g11280	9.00993664
LOC_Os05g34770	LOC_Os08g44520	10.4865149
LOC_Os05g34770	LOC_Os11g16590	9.46145748
LOC_Os05g33730	LOC_Os06g06510	9.52246675
LOC_Os05g33730	LOC_Os09g30360	9.74006325
LOC_Os05g34170	LOC_Os11g19800	8.88685054
LOC_Os05g34540	LOC_Os07g48880	8.82050327
LOC_Os05g35290	LOC_Os12g33610	9.0176027
LOC_Os05g33410	LOC_Os11g32580	9.4653482
LOC_Os05g34170	LOC_Os07g08880	11.0626245
LOC_Os05g34540	LOC_Os10g38160	9.06564469
LOC_Os05g35290	LOC_Os08g02340	9.73615261
LOC_Os05g35330	LOC_Os10g21268	15.0941818
LOC_Os05g34770	LOC_Os07g38890	9.37532776
LOC_Os05g33730	LOC_Os11g29400	9.66748571
LOC_Os05g35320	LOC_Os05g44180	8.95055922
LOC_Os05g34770	LOC_Os11g05470	9.1869353
LOC_Os05g35400	LOC_Os09g15400	8.93511178
LOC_Os05g35400	LOC_Os06g37500	9.13310346
LOC_Os05g34540	LOC_Os10g38360	8.83789472
LOC_Os05g35770	LOC_Os06g35540	10.2753256
LOC_Os05g35330	LOC_Os10g09860	9.09834083
LOC_Os05g33570	LOC_Os06g11210	10.4348346
LOC_Os05g37700	LOC_Os09g24412	9.09721717
LOC_Os05g37690	LOC_Os12g06620	9.77976757
LOC_Os05g37390	LOC_Os07g05150	11.3415328
LOC_Os05g36010	LOC_Os05g39690	9.8843431
LOC_Os05g37690	LOC_Os10g33800	9.43231757
LOC_Os05g36270	LOC_Os12g43630	8.84106748
LOC_Os05g37690	LOC_Os08g02410	10.5001129
LOC_Os05g36010	LOC_Os08g40930	9.9266694
LOC_Os05g37690	LOC_Os12g04924	8.9836576
LOC_Os05g37330	LOC_Os12g31370	9.30438613

InteractorA	InteractorB	Z score
LOC_Os05g36280	LOC_Os10g07616	9.5223222
LOC_Os05g37390	LOC_Os11g25330	9.8420253
LOC_Os05g36290	LOC_Os07g49400	8.87151864
LOC_Os05g37700	LOC_Os07g39870	9.00514705
LOC_Os05g37390	LOC_Os08g10010	9.39931085
LOC_Os05g36280	LOC_Os06g06510	11.0654453
LOC_Os05g38230	LOC_Os08g39860	9.21223259
LOC_Os05g37330	LOC_Os05g50710	10.0623345
LOC_Os05g37690	LOC_Os07g05820	8.91269304
LOC_Os05g37700	LOC_Os07g42490	9.6744632
LOC_Os05g36010	LOC_Os06g04510	10.6961635
LOC_Os05g38230	LOC_Os06g04030	8.90528424
LOC_Os05g36280	LOC_Os09g32620	10.0128063
LOC_Os05g36010	LOC_Os11g37890	9.14459746
LOC_Os05g37390	LOC_Os12g43370	9.17935792
LOC_Os05g37690	LOC_Os07g06970	9.30216118
LOC_Os05g36280	LOC_Os10g38350	9.2380179
LOC_Os05g36290	LOC_Os05g49830	9.16182789
LOC_Os05g36280	LOC_Os12g22680	9.68892299
LOC_Os05g36010	LOC_Os05g51050	9.72552064
LOC_Os05g36010	LOC_Os05g41210	10.7821661
LOC_Os05g36010	LOC_Os09g04680	9.18902255
LOC_Os05g37330	LOC_Os10g11140	9.21845431
LOC_Os05g36290	LOC_Os09g26380	10.8700562
LOC_Os05g37390	LOC_Os08g27840	10.0207134
LOC_Os05g38230	LOC_Os06g21570	10.0419949
LOC_Os05g37330	LOC_Os06g12090	9.31369415
LOC_Os05g36010	LOC_Os09g39500	9.09597411
LOC_Os05g36010	LOC_Os07g48430	9.28557886
LOC_Os05g36010	LOC_Os11g03290	10.1746959
LOC_Os05g38230	LOC_Os12g02980	9.3002451
LOC_Os05g37700	LOC_Os07g35940	9.65130995
LOC_Os05g37700	LOC_Os07g25024	9.09721717
LOC_Os05g36010	LOC_Os09g34960	9.45975098
LOC_Os05g37700	LOC_Os08g15266	9.09721717
LOC_Os05g36280	LOC_Os06g37180	9.2220947
LOC_Os05g36280	LOC_Os12g10730	9.13435634
LOC_Os05g38230	LOC_Os11g29400	9.87436347
LOC_Os05g37700	LOC_Os05g39580	9.35404225
LOC_Os05g36280	LOC_Os06g06460	11.0654308
LOC_Os05g37330	LOC_Os12g02060	9.07477683
LOC_Os05g37884	LOC_Os11g10510	10.0364977
LOC_Os05g36290	LOC_Os10g10434	9.56513006

InteractorA	InteractorB	Z score
LOC_Os05g38230	LOC_Os09g21770	9.43493713
LOC_Os05g36280	LOC_Os08g04180	8.84954596
LOC_Os05g36290	LOC_Os09g10260	8.90767036
LOC_Os05g37390	LOC_Os05g38560	9.51216951
LOC_Os05g36290	LOC_Os05g46860	9.30946221
LOC_Os05g36010	LOC_Os07g42490	8.85489016
LOC_Os05g37330	LOC_Os06g23760	9.65541608
LOC_Os05g37690	LOC_Os11g19320	9.21516763
LOC_Os05g37690	LOC_Os10g21248	9.79206971
LOC_Os05g36280	LOC_Os09g12590	10.6579397
LOC_Os05g37330	LOC_Os08g44960	9.02233962
LOC_Os05g37390	LOC_Os05g46290	9.21343314
LOC_Os05g38230	LOC_Os06g05700	9.85102297
LOC_Os05g38230	LOC_Os07g44550	10.3285924
LOC_Os05g37330	LOC_Os09g36830	9.25979146
LOC_Os05g37690	LOC_Os12g43630	9.01397371
LOC_Os05g37390	LOC_Os06g37150	9.7555488
LOC_Os05g36270	LOC_Os06g35700	9.25951446
LOC_Os05g37700	LOC_Os12g36950	8.99256354
LOC_Os05g37690	LOC_Os08g25734	9.19646495
LOC_Os05g38230	LOC_Os05g49890	8.96792782
LOC_Os05g36280	LOC_Os11g05730	11.0654308
LOC_Os05g38230	LOC_Os09g34250	9.27071411
LOC_Os05g36270	LOC_Os06g06730	9.11653927
LOC_Os05g37690	LOC_Os12g13030	8.98589719
LOC_Os05g36270	LOC_Os10g32550	8.91174348
LOC_Os05g36010	LOC_Os06g43640	8.94036247
LOC_Os05g36270	LOC_Os06g29844	8.89359001
LOC_Os05g37390	LOC_Os06g49470	8.85627636
LOC_Os05g36010	LOC_Os07g31830	9.1491009
LOC_Os05g36270	LOC_Os06g48180	10.1060473
LOC_Os05g38230	LOC_Os12g25630	9.00950372
LOC_Os05g36270	LOC_Os06g35730	8.93599419
LOC_Os05g37390	LOC_Os09g10300	10.0206182
LOC_Os05g36280	LOC_Os12g07980	10.6969586
LOC_Os05g37690	LOC_Os06g23760	9.63844221
LOC_Os05g36270	LOC_Os06g04270	9.44606774
LOC_Os05g37700	LOC_Os07g44790	9.54844264
LOC_Os05g38230	LOC_Os07g07719	10.4145018
LOC_Os05g36280	LOC_Os06g36770	9.28017027
LOC_Os05g38230	LOC_Os09g38620	8.87212581
LOC_Os05g36010	LOC_Os08g42560	10.3238425
LOC_Os05g36010	LOC_Os12g38770	9.95848837

InteractorA	InteractorB	Z score
LOC_Os05g36290	LOC_Os08g06060	10.7256163
LOC_Os05g37390	LOC_Os08g02700	8.89496138
LOC_Os05g37700	LOC_Os10g21352	9.09721717
LOC_Os05g36010	LOC_Os07g23470	8.99011615
LOC_Os05g37390	LOC_Os08g40740	9.1927705
LOC_Os05g38230	LOC_Os10g41689	8.98722345
LOC_Os05g36010	LOC_Os07g46310	8.85187957
LOC_Os05g38230	LOC_Os12g42876	9.43853643
LOC_Os05g37390	LOC_Os05g47640	9.11210177
LOC_Os05g37700	LOC_Os06g05250	9.20621556
LOC_Os05g37700	LOC_Os11g25330	9.98315975
LOC_Os05g37390	LOC_Os08g44210	8.99761181
LOC_Os05g36010	LOC_Os10g28200	9.53992145
LOC_Os05g36290	LOC_Os06g01590	9.99534356
LOC_Os05g36010	LOC_Os05g36290	8.96720647
LOC_Os05g37690	LOC_Os05g46360	9.23068424
LOC_Os05g36010	LOC_Os10g38160	8.85410318
LOC_Os05g37690	LOC_Os10g35480	9.08059256
LOC_Os05g36010	LOC_Os10g07229	8.81471727
LOC_Os05g37390	LOC_Os12g34874	10.3196207
LOC_Os05g37390	LOC_Os06g45590	9.41054985
LOC_Os05g37690	LOC_Os07g05150	9.03884938
LOC_Os05g36280	LOC_Os10g38160	8.95621906
LOC_Os05g37330	LOC_Os06g35560	9.3085619
LOC_Os05g37700	LOC_Os09g12590	9.28620942
LOC_Os05g37700	LOC_Os09g24924	8.83160781
LOC_Os05g36270	LOC_Os10g08550	9.34492095
LOC_Os05g38230	LOC_Os07g47990	10.0260571
LOC_Os05g38230	LOC_Os05g40990	9.06197965
LOC_Os05g38230	LOC_Os12g36950	10.0251981
LOC_Os05g36010	LOC_Os07g43390	8.92268301
LOC_Os05g36280	LOC_Os07g44550	10.5558631
LOC_Os05g37690	LOC_Os07g35940	8.86494247
LOC_Os01g21820	LOC_Os02g13140	8.9421039
LOC_Os01g21820	LOC_Os11g39540	10.0875913
LOC_Os01g21820	LOC_Os02g08490	10.7733023
LOC_Os01g19450	LOC_Os02g52700	9.59236098
LOC_Os01g21970	LOC_Os02g42810	8.88415761
LOC_Os01g19750	LOC_Os04g53810	9.86318855
LOC_Os01g21820	LOC_Os09g36930	9.51734726
LOC_Os01g21820	LOC_Os07g14590	10.5209043
LOC_Os01g19750	LOC_Os08g33710	8.85735209
LOC_Os01g19150	LOC_Os06g10910	9.05353518

InteractorA	InteractorB	Z score
LOC_Os01g19750	LOC_Os01g27340	9.1872829
LOC_Os01g19150	LOC_Os05g11730	9.19605455
LOC_Os01g19450	LOC_Os06g48200	9.50890054
LOC_Os01g19150	LOC_Os09g34970	8.93200444
LOC_Os01g19450	LOC_Os10g26390	10.1347508
LOC_Os01g21970	LOC_Os01g46570	9.13964043
LOC_Os01g21820	LOC_Os01g42690	9.94554063
LOC_Os01g19450	LOC_Os03g55600	15.8688046
LOC_Os01g19150	LOC_Os06g01390	9.42360311
LOC_Os01g19750	LOC_Os07g02210	9.25852053
LOC_Os01g19450	LOC_Os06g04000	9.09905316
LOC_Os01g19450	LOC_Os03g16900	10.8335497
LOC_Os01g19750	LOC_Os02g57770	9.33252238
LOC_Os01g21820	LOC_Os02g55140	9.44252429
LOC_Os01g19150	LOC_Os08g04540	9.34389677
LOC_Os01g19450	LOC_Os09g37100	12.4535138
LOC_Os01g19450	LOC_Os07g01760	9.70141425
LOC_Os01g19450	LOC_Os03g50480	9.18419506
LOC_Os01g21820	LOC_Os12g23630	9.01919873
LOC_Os01g19450	LOC_Os12g16250	9.67834087
LOC_Os01g21970	LOC_Os01g58220	8.87489815
LOC_Os01g21820	LOC_Os10g39880	8.88735027
LOC_Os01g19450	LOC_Os01g46070	9.8509073
LOC_Os01g19750	LOC_Os02g52420	8.95415821
LOC_Os01g21970	LOC_Os01g64660	10.3496575
LOC_Os01g19450	LOC_Os02g57720	8.82250356
LOC_Os01g19450	LOC_Os02g52710	9.59236098
LOC_Os01g19450	LOC_Os07g38430	9.16860302
LOC_Os01g19750	LOC_Os01g44220	8.90254624
LOC_Os01g19750	LOC_Os01g71400	8.83467545
LOC_Os01g21970	LOC_Os01g27210	8.86713233
LOC_Os01g19150	LOC_Os07g06970	9.19485114
LOC_Os01g19450	LOC_Os01g49120	9.01268247
LOC_Os01g19450	LOC_Os06g09910	9.23810605
LOC_Os01g19450	LOC_Os07g40580	9.01147391
LOC_Os01g19450	LOC_Os12g10560	9.47244149
LOC_Os01g19150	LOC_Os10g11810	9.37186023
LOC_Os01g19150	LOC_Os09g12590	9.54347083
LOC_Os01g19150	LOC_Os06g43640	10.1040125
LOC_Os01g19150	LOC_Os06g35530	9.2029714
LOC_Os01g19450	LOC_Os03g59740	8.88421331
LOC_Os01g21820	LOC_Os05g23740	11.0690471
LOC_Os01g21970	LOC_Os01g57945	9.31894578

InteractorA	InteractorB	Z score
LOC_Os01g21180	LOC_Os01g25065	9.65159926
LOC_Os01g19450	LOC_Os03g60620	9.39521632
LOC_Os01g19450	LOC_Os04g43400	9.0527387
LOC_Os01g21820	LOC_Os01g71340	9.75750705
LOC_Os01g19450	LOC_Os07g47990	8.91184894
LOC_Os01g19450	LOC_Os04g10010	9.60938788
LOC_Os01g21820	LOC_Os07g20544	10.0664561
LOC_Os01g19750	LOC_Os12g16240	9.05707601
LOC_Os01g19450	LOC_Os02g41630	9.46941398
LOC_Os01g19150	LOC_Os05g44180	9.77441527
LOC_Os01g21820	LOC_Os06g42130	9.31852313
LOC_Os01g19750	LOC_Os05g36270	8.9623648
LOC_Os01g19450	LOC_Os03g56280	9.54734351
LOC_Os01g21180	LOC_Os03g45320	10.1978401
LOC_Os01g21820	LOC_Os03g62670	8.83258896
LOC_Os01g21820	LOC_Os10g32550	8.83143476
LOC_Os01g19150	LOC_Os07g43390	9.26566978
LOC_Os01g21820	LOC_Os09g04050	10.4986953
LOC_Os01g21180	LOC_Os02g56690	8.87150997
LOC_Os01g19450	LOC_Os05g04500	10.888353
LOC_Os01g19450	LOC_Os10g41490	9.88111705
LOC_Os01g19450	LOC_Os02g47790	9.2080322
LOC_Os01g21180	LOC_Os09g38620	9.93380603
LOC_Os01g19450	LOC_Os01g63270	10.0926536
LOC_Os01g21820	LOC_Os12g35570	8.81974755
LOC_Os01g19450	LOC_Os01g73220	11.7856153
LOC_Os01g21820	LOC_Os03g03910	10.4124378
LOC_Os01g19450	LOC_Os04g56646	9.06198668
LOC_Os01g19450	LOC_Os04g29550	9.6181939
LOC_Os01g19450	LOC_Os07g48050	9.74926534
LOC_Os01g19450	LOC_Os06g35520	8.82208323
LOC_Os01g19150	LOC_Os07g42924	9.50036431
LOC_Os01g19450	LOC_Os08g36900	9.41355592
LOC_Os01g21180	LOC_Os04g37480	9.06495857
LOC_Os01g21180	LOC_Os10g08670	8.96066948
LOC_Os01g19450	LOC_Os01g21970	9.53810564
LOC_Os01g21820	LOC_Os03g51600	9.23817881
LOC_Os01g21180	LOC_Os05g06450	9.43891566
LOC_Os01g21180	LOC_Os02g47790	9.08179252
LOC_Os01g19450	LOC_Os04g57380	9.70520694
LOC_Os01g19450	LOC_Os10g28080	10.4547007
LOC_Os01g19150	LOC_Os09g26380	8.95010943
LOC_Os01g21970	LOC_Os03g20700	8.80505362

InteractorA	InteractorB	Z score
LOC_Os01g19450	LOC_Os07g05820	10.5789121
LOC_Os01g19450	LOC_Os03g08280	9.0922384
LOC_Os01g21820	LOC_Os06g49970	8.99773308
LOC_Os01g21820	LOC_Os06g06040	10.1659896
LOC_Os01g19450	LOC_Os04g25400	9.78071074
LOC_Os01g19150	LOC_Os11g37890	8.87102784
LOC_Os01g19450	LOC_Os10g26010	8.84953162
LOC_Os01g19450	LOC_Os03g22120	8.89718463
LOC_Os01g21820	LOC_Os12g42280	8.89585468
LOC_Os01g19450	LOC_Os01g22249	9.75216066
LOC_Os01g21180	LOC_Os02g13330	9.03730741
LOC_Os01g21180	LOC_Os05g37390	8.94459155
LOC_Os01g21820	LOC_Os03g55800	8.96136307
LOC_Os01g19450	LOC_Os04g16770	9.00879836
LOC_Os01g19450	LOC_Os08g03290	9.12458543
LOC_Os01g19750	LOC_Os10g08670	10.1221134
LOC_Os01g19450	LOC_Os08g40740	8.99389316
LOC_Os01g19450	LOC_Os08g35420	9.00879836
LOC_Os01g21970	LOC_Os03g02680	9.30434549
LOC_Os01g19750	LOC_Os02g08100	9.71952853
LOC_Os01g19450	LOC_Os07g30170	10.0590669
LOC_Os01g19750	LOC_Os05g27940	9.58259295
LOC_Os01g19450	LOC_Os12g10720	9.50603086
LOC_Os01g21820	LOC_Os10g38670	10.4494853
LOC_Os01g21820	LOC_Os12g07720	9.67896631
LOC_Os01g19450	LOC_Os04g16874	9.86601684
LOC_Os01g21820	LOC_Os12g06660	10.2487686
LOC_Os01g19450	LOC_Os11g02600	9.54932451
LOC_Os01g19450	LOC_Os12g17910	8.82008507
LOC_Os01g19450	LOC_Os08g04460	9.094895
LOC_Os01g21820	LOC_Os08g14570	9.95515158
LOC_Os01g19450	LOC_Os11g06390	9.84296874
LOC_Os01g19750	LOC_Os08g40140	10.2738318
LOC_Os01g21970	LOC_Os03g16740	8.95048504
LOC_Os01g21820	LOC_Os03g55874	9.19056912
LOC_Os01g19450	LOC_Os01g49690	8.95270926
LOC_Os01g19450	LOC_Os08g20730	9.48702237
LOC_Os01g21820	LOC_Os03g55090	9.92665303
LOC_Os01g21820	LOC_Os03g21460	8.86007062
LOC_Os01g21180	LOC_Os08g27840	9.17581816
LOC_Os01g19750	LOC_Os09g26380	9.70049338
LOC_Os01g19750	LOC_Os11g08460	11.1106061
LOC_Os01g21820	LOC_Os03g28330	8.87157067

InteractorA	InteractorB	Z score
LOC_Os01g19750	LOC_Os08g42410	9.50438914
LOC_Os01g19750	LOC_Os03g27260	9.24908081
LOC_Os01g21970	LOC_Os01g62244	11.277991
LOC_Os01g21820	LOC_Os05g45220	8.98733386
LOC_Os01g19450	LOC_Os05g48040	9.98998207
LOC_Os01g19750	LOC_Os08g25734	8.87977111
LOC_Os01g21180	LOC_Os08g32620	9.2129868
LOC_Os01g21820	LOC_Os12g44000	9.7454854
LOC_Os01g21820	LOC_Os09g37100	8.9048894
LOC_Os01g21820	LOC_Os07g47290	11.0536293
LOC_Os05g39960	LOC_Os11g10520	9.53455926
LOC_Os05g39580	LOC_Os08g41830	10.4656692
LOC_Os05g38560	LOC_Os05g48510	9.16385788
LOC_Os05g38530	LOC_Os07g25150	9.49990084
LOC_Os05g39960	LOC_Os06g08770	9.03060357
LOC_Os05g38760	LOC_Os06g30970	9.14028196
LOC_Os05g38310	LOC_Os09g20090	9.28843359
LOC_Os05g38560	LOC_Os12g25120	9.7639978
LOC_Os05g38740	LOC_Os07g07709	10.0431544
LOC_Os05g38760	LOC_Os09g07830	9.59197817
LOC_Os05g38760	LOC_Os08g38900	9.6815753
LOC_Os05g38560	LOC_Os10g38540	9.91386089
LOC_Os05g38550	LOC_Os05g42350	8.9183529
LOC_Os05g39690	LOC_Os06g51084	10.208134
LOC_Os05g38550	LOC_Os09g14670	9.30886929
LOC_Os05g38530	LOC_Os11g31620	8.924725
LOC_Os05g38310	LOC_Os06g44270	8.96675347
LOC_Os05g38560	LOC_Os05g50890	8.89072484
LOC_Os05g38740	LOC_Os11g10480	8.92560379
LOC_Os05g39690	LOC_Os11g48110	9.28252549
LOC_Os05g39580	LOC_Os06g21570	9.01872896
LOC_Os05g40420	LOC_Os06g06100	10.2594983
LOC_Os05g38530	LOC_Os07g03690	9.21935055
LOC_Os05g38560	LOC_Os11g34570	8.86262547
LOC_Os05g38550	LOC_Os09g34214	10.1203123
LOC_Os05g39960	LOC_Os09g24990	9.22630795
LOC_Os05g39690	LOC_Os07g07770	9.35438744
LOC_Os05g39690	LOC_Os08g09200	9.06730226
LOC_Os05g39050	LOC_Os10g39170	9.48057701
LOC_Os05g38760	LOC_Os06g06980	9.27980612
LOC_Os05g39580	LOC_Os10g07229	8.83301183
LOC_Os05g38560	LOC_Os09g26880	10.0899819
LOC_Os05g38530	LOC_Os12g07830	9.33698149

InteractorA	InteractorB	Z score
LOC_Os05g39690	LOC_Os08g40140	9.12568788
LOC_Os05g39580	LOC_Os08g02410	9.16842622
LOC_Os05g39580	LOC_Os12g38770	9.01389315
LOC_Os05g38560	LOC_Os07g23470	9.35682435
LOC_Os05g38560	LOC_Os12g17540	9.38489277
LOC_Os05g38560	LOC_Os08g33100	8.84396728
LOC_Os05g39960	LOC_Os08g42910	9.5962737
LOC_Os05g38530	LOC_Os07g23470	8.97081677
LOC_Os05g38740	LOC_Os10g22070	9.42846446
LOC_Os05g38530	LOC_Os07g08500	9.34017364
LOC_Os05g38530	LOC_Os05g45810	9.31942857
LOC_Os05g39960	LOC_Os06g03770	9.96444288
LOC_Os05g39690	LOC_Os12g13810	8.96178758
LOC_Os05g39690	LOC_Os07g34140	9.77480918
LOC_Os05g39960	LOC_Os11g10480	12.2017092
LOC_Os05g38740	LOC_Os10g01540	9.10660275
LOC_Os05g38560	LOC_Os08g40170	8.87182525
LOC_Os05g39690	LOC_Os05g49800	9.47195854
LOC_Os05g38530	LOC_Os05g39690	9.15370961
LOC_Os05g38560	LOC_Os10g22450	9.08815182
LOC_Os05g38550	LOC_Os11g38959	9.5277446
LOC_Os05g38560	LOC_Os12g34062	8.96832534
LOC_Os05g38310	LOC_Os07g05800	8.98508631
LOC_Os05g38310	LOC_Os05g45810	9.17243885
LOC_Os05g40420	LOC_Os06g06050	8.90558835
LOC_Os05g39580	LOC_Os06g14510	8.88590714
LOC_Os05g39580	LOC_Os06g19960	9.36364424
LOC_Os05g38760	LOC_Os11g04954	8.81556153
LOC_Os05g38530	LOC_Os11g37950	8.89249339
LOC_Os05g38760	LOC_Os05g45420	9.78482311
LOC_Os05g38530	LOC_Os10g02480	8.87581583
LOC_Os05g39050	LOC_Os11g31620	9.02281446
LOC_Os05g39050	LOC_Os11g10510	8.86154942
LOC_Os05g38550	LOC_Os07g26900	8.91218277
LOC_Os05g38530	LOC_Os12g16290	9.24707169
LOC_Os05g38760	LOC_Os08g34790	11.8755741
LOC_Os05g38740	LOC_Os11g31620	9.02280822
LOC_Os05g38560	LOC_Os06g05250	8.94715986
LOC_Os05g39960	LOC_Os06g36700	9.26767829
LOC_Os05g40420	LOC_Os05g49800	9.29327886
LOC_Os05g38560	LOC_Os08g44530	10.2277233
LOC_Os05g38530	LOC_Os12g16220	9.15388945
LOC_Os05g38530	LOC_Os09g23530	8.8671273

InteractorA	InteractorB	Z score
LOC_Os05g38760	LOC_Os12g24650	9.21375112
LOC_Os05g38310	LOC_Os07g40290	10.5084262
LOC_Os05g38560	LOC_Os08g20730	8.88655611
LOC_Os05g38550	LOC_Os08g09770	9.32250657
LOC_Os05g39580	LOC_Os09g32830	8.92602297
LOC_Os05g39690	LOC_Os10g40090	9.01222291
LOC_Os05g39690	LOC_Os05g45220	8.92574867
LOC_Os05g39050	LOC_Os07g07709	10.0431544
LOC_Os05g38740	LOC_Os10g39170	9.48057701
LOC_Os05g39960	LOC_Os08g43170	10.1375312
LOC_Os05g38530	LOC_Os10g07040	9.33886932
LOC_Os05g38550	LOC_Os11g03290	10.9146757
LOC_Os05g38740	LOC_Os07g07719	8.80395226
LOC_Os05g38550	LOC_Os11g47600	8.9895045
LOC_Os05g38560	LOC_Os06g30970	9.26919842
LOC_Os05g39690	LOC_Os07g41050	8.83168763
LOC_Os05g39050	LOC_Os07g07719	8.80395171
LOC_Os05g38560	LOC_Os07g43820	9.23001289
LOC_Os05g39690	LOC_Os07g48020	9.3574448
LOC_Os05g38560	LOC_Os11g43360	10.0664161
LOC_Os05g38740	LOC_Os12g22680	9.95585475
LOC_Os05g38760	LOC_Os12g44020	9.31060991
LOC_Os05g40420	LOC_Os06g35560	10.544721
LOC_Os05g38530	LOC_Os12g13380	9.03085237
LOC_Os05g39580	LOC_Os12g21798	9.90795406
LOC_Os05g38530	LOC_Os10g11810	10.2551659
LOC_Os05g39690	LOC_Os08g39420	8.93640652
LOC_Os05g38310	LOC_Os06g23760	10.0084217
LOC_Os05g38310	LOC_Os08g41830	9.30616119
LOC_Os05g39690	LOC_Os06g09910	8.88282531
LOC_Os05g38530	LOC_Os09g34214	9.73545617
LOC_Os05g39580	LOC_Os06g37150	10.0519572
LOC_Os05g39050	LOC_Os11g10480	8.92560379
LOC_Os05g40420	LOC_Os06g36670	9.01472321
LOC_Os05g38530	LOC_Os05g39960	11.034257
LOC_Os05g38740	LOC_Os11g10520	10.9140543
LOC_Os05g38740	LOC_Os11g10510	8.86156807
LOC_Os05g39050	LOC_Os10g01540	9.10660661
LOC_Os05g39050	LOC_Os11g10520	10.9141423
LOC_Os05g38760	LOC_Os10g26110	9.65837426
LOC_Os05g39050	LOC_Os12g22680	9.95584485
LOC_Os05g39580	LOC_Os12g05590	9.50382697
LOC_Os05g39960	LOC_Os07g42960	10.1985365

InteractorA	InteractorB	Z score
LOC_Os05g38530	LOC_Os07g37320	9.38566623
LOC_Os05g39050	LOC_Os10g22070	9.42850276
LOC_Os05g38310	LOC_Os07g46460	9.06086785
LOC_Os05g40420	LOC_Os07g01780	9.29313386
LOC_Os05g38560	LOC_Os07g36130	9.36108273
LOC_Os05g39580	LOC_Os05g41610	9.19585235
LOC_Os05g41900	LOC_Os07g01780	8.89634443
LOC_Os05g41080	LOC_Os10g38340	9.68051164
LOC_Os05g41080	LOC_Os08g28820	8.89156905
LOC_Os05g41060	LOC_Os06g35560	9.12672838
LOC_Os05g40420	LOC_Os12g03816	8.98435593
LOC_Os05g42150	LOC_Os07g07709	9.57386949
LOC_Os05g40420	LOC_Os12g41110	12.407289
LOC_Os05g40990	LOC_Os05g43510	10.8976564
LOC_Os05g40420	LOC_Os08g39140	11.2925055
LOC_Os05g41080	LOC_Os09g33860	10.7276557
LOC_Os05g41640	LOC_Os12g41110	9.16302265
LOC_Os05g40990	LOC_Os06g23760	8.80987696
LOC_Os05g41180	LOC_Os07g06970	9.04726762
LOC_Os05g40420	LOC_Os07g08500	9.74530363
LOC_Os05g41210	LOC_Os12g13800	9.07884425
LOC_Os05g41060	LOC_Os10g38360	9.0225569
LOC_Os05g41610	LOC_Os09g25320	9.03225386
LOC_Os05g41640	LOC_Os08g15292	8.84924646
LOC_Os05g42150	LOC_Os06g04030	8.80060252
LOC_Os05g41610	LOC_Os08g09950	9.45269744
LOC_Os05g41640	LOC_Os12g16410	9.03646059
LOC_Os05g41210	LOC_Os09g30360	10.7331163
LOC_Os05g41610	LOC_Os10g05069	9.46302954
LOC_Os05g42150	LOC_Os05g47640	8.8552001
LOC_Os05g40420	LOC_Os10g08710	8.90186653
LOC_Os05g41640	LOC_Os09g32830	9.32031821
LOC_Os05g41230	LOC_Os07g48060	9.5471361
LOC_Os05g42150	LOC_Os06g35540	8.89185252
LOC_Os05g41080	LOC_Os07g01560	8.99185679
LOC_Os05g41210	LOC_Os11g48110	8.95520285
LOC_Os05g41610	LOC_Os09g12570	9.28531397
LOC_Os05g40990	LOC_Os07g10720	9.23684604
LOC_Os05g41180	LOC_Os12g23170	10.7560024
LOC_Os05g41210	LOC_Os07g48060	9.30469423
LOC_Os05g41640	LOC_Os06g13450	9.87527558
LOC_Os05g42150	LOC_Os05g49800	9.50218319
LOC_Os05g41180	LOC_Os09g23550	8.81718322

InteractorA	InteractorB	Z score
LOC_Os05g40990	LOC_Os08g14570	10.100276
LOC_Os05g41210	LOC_Os12g22650	8.99865718
LOC_Os05g40990	LOC_Os05g42350	9.43189826
LOC_Os05g40420	LOC_Os07g38860	11.307089
LOC_Os05g40420	LOC_Os07g48780	8.99798233
LOC_Os05g41210	LOC_Os09g36450	9.5599181
LOC_Os05g41610	LOC_Os05g43820	8.84138023
LOC_Os05g40990	LOC_Os07g34580	8.80445906
LOC_Os05g41060	LOC_Os07g03499	8.80721794
LOC_Os05g40420	LOC_Os09g38030	9.71289148
LOC_Os05g41210	LOC_Os10g27174	9.45346658
LOC_Os05g41060	LOC_Os12g34062	11.0186975
LOC_Os05g41080	LOC_Os10g40600	8.80243263
LOC_Os05g40990	LOC_Os06g06100	8.97348671
LOC_Os05g41080	LOC_Os06g04030	13.2611391
LOC_Os05g41640	LOC_Os06g36670	9.94930344
LOC_Os05g41210	LOC_Os08g34190	8.80682679
LOC_Os05g41180	LOC_Os06g45120	10.7319141
LOC_Os05g41080	LOC_Os06g36840	8.95738509
LOC_Os05g41230	LOC_Os07g07240	9.09832165
LOC_Os05g41060	LOC_Os07g03319	8.80720248
LOC_Os05g41210	LOC_Os12g34874	9.23821365
LOC_Os05g40420	LOC_Os09g33860	9.23756172
LOC_Os05g41610	LOC_Os11g08445	9.06078774
LOC_Os05g42150	LOC_Os08g04540	9.04370972
LOC_Os05g41610	LOC_Os07g03288	9.68256852
LOC_Os05g41210	LOC_Os09g11230	8.81385897
LOC_Os05g41610	LOC_Os07g03590	9.68256852
LOC_Os05g41210	LOC_Os06g36770	9.06484757
LOC_Os05g41080	LOC_Os10g08550	8.91273413
LOC_Os05g41080	LOC_Os08g09370	8.9211404
LOC_Os05g41610	LOC_Os07g03467	9.68256852
LOC_Os05g42150	LOC_Os09g31120	8.85725611
LOC_Os05g40990	LOC_Os11g10520	8.96193723
LOC_Os05g42150	LOC_Os07g38540	9.89623629
LOC_Os05g41060	LOC_Os07g03409	8.80720248
LOC_Os05g41210	LOC_Os08g23110	9.09277993
LOC_Os05g42150	LOC_Os08g37490	9.73250985
LOC_Os05g41210	LOC_Os08g03440	9.38880098
LOC_Os05g41210	LOC_Os11g05570	8.95428408
LOC_Os05g41210	LOC_Os06g23440	9.3212363
LOC_Os05g41210	LOC_Os07g09890	11.4961004
LOC_Os05g41900	LOC_Os10g29620	9.36382074

InteractorA	InteractorB	Z score
LOC_Os05g42150	LOC_Os07g42940	8.96691575
LOC_Os05g41640	LOC_Os09g25370	9.11663379
LOC_Os05g41610	LOC_Os10g39170	8.96335806
LOC_Os05g41640	LOC_Os11g04880	8.98519833
LOC_Os05g41640	LOC_Os09g11230	9.34721181
LOC_Os05g41610	LOC_Os06g44080	9.03225686
LOC_Os05g41080	LOC_Os10g38470	8.94911506
LOC_Os05g41640	LOC_Os11g43360	9.15785065
LOC_Os05g41210	LOC_Os05g46290	9.83154744
LOC_Os05g41610	LOC_Os06g04030	9.13173855
LOC_Os05g41640	LOC_Os07g48010	9.26525664
LOC_Os05g40420	LOC_Os09g08072	9.61216178
LOC_Os05g41080	LOC_Os10g28350	9.57920743
LOC_Os05g41640	LOC_Os12g25700	10.3541804
LOC_Os05g41610	LOC_Os12g22680	9.94065839
LOC_Os05g41210	LOC_Os10g21310	11.2051261
LOC_Os05g41180	LOC_Os09g21770	8.87206262
LOC_Os05g40420	LOC_Os07g07550	9.09162287
LOC_Os05g41610	LOC_Os08g17784	9.11381515
LOC_Os05g41060	LOC_Os09g04730	11.1660966
LOC_Os05g41180	LOC_Os08g04560	9.05754854
LOC_Os05g40990	LOC_Os10g26010	9.23959527
LOC_Os05g41080	LOC_Os08g15292	8.89893569
LOC_Os05g41900	LOC_Os07g47490	9.55880391
LOC_Os05g40420	LOC_Os07g23730	8.88290975
LOC_Os05g41210	LOC_Os05g49890	9.03493835
LOC_Os05g41060	LOC_Os06g46372	10.2708534
LOC_Os05g41060	LOC_Os07g48060	9.00293471
LOC_Os05g41180	LOC_Os11g36719	8.85579482
LOC_Os05g41180	LOC_Os07g05800	9.47845714
LOC_Os05g41080	LOC_Os08g02400	8.83933257
LOC_Os05g40420	LOC_Os10g01570	9.6606558
LOC_Os05g41210	LOC_Os06g44620	11.5146751
LOC_Os05g40420	LOC_Os11g10480	8.83475247
LOC_Os05g42150	LOC_Os05g46290	8.96549
LOC_Os05g41610	LOC_Os07g03377	9.68256852
LOC_Os05g41900	LOC_Os05g48510	9.9611657
LOC_Os05g41080	LOC_Os10g25674	8.83342681
LOC_Os05g41230	LOC_Os09g39500	9.37399682
LOC_Os05g41900	LOC_Os12g02370	9.47765976
LOC_Os05g40420	LOC_Os11g08445	11.9431466
LOC_Os05g41080	LOC_Os11g37640	10.2494296
LOC_Os05g41640	LOC_Os08g44530	8.80338718

InteractorA	InteractorB	Z score
LOC_Os05g40420	LOC_Os10g25130	8.89754345
LOC_Os05g42150	LOC_Os06g39140	8.97771819
LOC_Os05g41640	LOC_Os12g13800	11.0709119
LOC_Os05g41080	LOC_Os07g22650	9.03956504
LOC_Os05g41610	LOC_Os06g05250	9.16916507
LOC_Os05g41060	LOC_Os11g32620	9.39823197
LOC_Os05g44760	LOC_Os08g34170	9.87653809
LOC_Os05g43510	LOC_Os11g32620	10.1609056
LOC_Os05g43510	LOC_Os12g12514	9.14775127
LOC_Os05g45220	LOC_Os06g21980	9.05472125
LOC_Os05g43510	LOC_Os09g32570	9.18475105
LOC_Os05g44760	LOC_Os07g43670	9.97299546
LOC_Os05g43510	LOC_Os12g22680	9.45702429
LOC_Os05g44340	LOC_Os08g44280	9.46779746
LOC_Os05g44760	LOC_Os07g03467	9.58870019
LOC_Os05g45220	LOC_Os05g49840	9.73178821
LOC_Os05g44340	LOC_Os12g06620	9.98327352
LOC_Os05g44140	LOC_Os10g29470	9.13239202
LOC_Os05g42350	LOC_Os06g34690	9.30136248
LOC_Os05g44180	LOC_Os06g11210	9.11183641
LOC_Os05g43510	LOC_Os07g38540	10.0453293
LOC_Os05g43820	LOC_Os10g29470	8.91122949
LOC_Os05g43510	LOC_Os09g37540	8.86098621
LOC_Os05g44760	LOC_Os07g34589	10.4134574
LOC_Os05g43510	LOC_Os12g07720	9.4232499
LOC_Os05g44760	LOC_Os07g03590	9.58872153
LOC_Os05g44340	LOC_Os11g08340	8.9812811
LOC_Os05g44180	LOC_Os06g19960	11.1618352
LOC_Os05g42350	LOC_Os08g42000	9.10336691
LOC_Os05g43510	LOC_Os10g31950	9.48599688
LOC_Os05g43510	LOC_Os08g06100	9.99036985
LOC_Os05g44340	LOC_Os11g08460	9.49603087
LOC_Os05g44340	LOC_Os09g20260	8.82496718
LOC_Os05g44340	LOC_Os07g08170	9.02735725
LOC_Os05g44340	LOC_Os11g03230	9.03359471
LOC_Os05g45220	LOC_Os10g28120	10.1302377
LOC_Os05g42350	LOC_Os06g29220	9.3690338
LOC_Os05g44050	LOC_Os06g23440	10.1754768
LOC_Os05g44140	LOC_Os09g31430	8.96878974
LOC_Os05g42350	LOC_Os08g44340	9.78240868
LOC_Os05g45220	LOC_Os10g21352	8.88991535
LOC_Os05g42350	LOC_Os07g42950	10.9976826
LOC_Os05g45220	LOC_Os06g45100	9.04169684

InteractorA	InteractorB	Z score
LOC_Os05g45220	LOC_Os12g31370	8.8040358
LOC_Os05g44340	LOC_Os12g32240	9.21447089
LOC_Os05g44140	LOC_Os11g29190	8.94366681
LOC_Os05g42350	LOC_Os08g06100	9.16875716
LOC_Os05g44340	LOC_Os09g08072	9.48388663
LOC_Os05g42350	LOC_Os08g10608	8.96277373
LOC_Os05g44760	LOC_Os07g04240	9.17791239
LOC_Os05g42350	LOC_Os06g35730	9.35559735
LOC_Os05g42350	LOC_Os07g48880	9.1659148
LOC_Os05g44760	LOC_Os07g03288	9.58867227
LOC_Os05g44760	LOC_Os10g07616	9.04029782
LOC_Os05g42150	LOC_Os11g32770	8.86962312
LOC_Os05g44340	LOC_Os10g38710	9.32091567
LOC_Os05g44140	LOC_Os07g08660	9.77241447
LOC_Os05g42150	LOC_Os10g41689	8.92323129
LOC_Os05g44760	LOC_Os07g02210	10.3445286
LOC_Os05g44140	LOC_Os12g05590	9.34109835
LOC_Os05g44340	LOC_Os10g23900	8.81703659
LOC_Os05g44760	LOC_Os11g10520	9.42496492
LOC_Os05g44140	LOC_Os09g04730	9.76971666
LOC_Os05g44050	LOC_Os09g25150	9.34193738
LOC_Os05g44760	LOC_Os05g46580	9.3345993
LOC_Os05g44140	LOC_Os11g47590	9.13216865
LOC_Os05g44340	LOC_Os11g33270	8.8780774
LOC_Os05g45220	LOC_Os06g10910	8.87182775
LOC_Os05g43510	LOC_Os11g26860	10.0297106
LOC_Os05g43820	LOC_Os12g44150	9.00592746
LOC_Os05g44340	LOC_Os12g12580	9.0182295
LOC_Os05g44140	LOC_Os12g43100	9.54987905
LOC_Os05g42350	LOC_Os07g39270	9.97471194
LOC_Os05g43510	LOC_Os08g39140	9.1830842
LOC_Os05g43510	LOC_Os06g19960	9.18666215
LOC_Os05g44760	LOC_Os07g03377	9.58867227
LOC_Os05g42350	LOC_Os06g45100	9.02988701
LOC_Os05g44340	LOC_Os09g14670	8.99921728
LOC_Os05g43510	LOC_Os06g36700	9.20322852
LOC_Os05g44140	LOC_Os08g35420	8.91467951
LOC_Os05g44760	LOC_Os05g49890	8.96256177
LOC_Os05g42190	LOC_Os12g22680	8.91200175
LOC_Os05g44340	LOC_Os09g12660	8.89892705
LOC_Os05g44140	LOC_Os06g35490	8.85130049
LOC_Os05g43820	LOC_Os07g42600	9.05037929
LOC_Os05g45220	LOC_Os08g34290	9.52044015

InteractorA	InteractorB	Z score
LOC_Os05g44340	LOC_Os07g03600	8.83827066
LOC_Os05g43510	LOC_Os08g04560	14.1123371
LOC_Os05g43510	LOC_Os12g40510	9.20441089
LOC_Os05g43510	LOC_Os07g17010	9.18895142
LOC_Os05g45220	LOC_Os09g24412	8.88990111
LOC_Os05g44050	LOC_Os05g47540	9.80311851
LOC_Os05g42150	LOC_Os10g28120	9.96275222
LOC_Os05g42350	LOC_Os12g31640	8.95431809
LOC_Os05g42350	LOC_Os06g36700	9.89121105
LOC_Os05g44340	LOC_Os10g27050	9.38274027
LOC_Os05g45220	LOC_Os09g32952	9.18926137
LOC_Os05g43510	LOC_Os08g09370	9.11624161
LOC_Os05g42150	LOC_Os12g12590	10.2079028
LOC_Os05g42150	LOC_Os12g12514	9.64883539
LOC_Os05g44760	LOC_Os08g06100	10.0437511
LOC_Os05g42350	LOC_Os08g02410	8.91626668
LOC_Os05g43510	LOC_Os06g51150	9.20576067
LOC_Os05g43510	LOC_Os07g46460	9.48460008
LOC_Os05g42350	LOC_Os10g21326	8.87625392
LOC_Os05g43510	LOC_Os10g26110	18.3806857
LOC_Os05g44140	LOC_Os10g11140	9.25339711
LOC_Os05g44340	LOC_Os09g37949	8.84239461
LOC_Os05g45220	LOC_Os08g15266	8.88990277
LOC_Os05g42150	LOC_Os10g38229	9.76379522
LOC_Os05g42150	LOC_Os11g41610	9.09940679
LOC_Os05g44050	LOC_Os07g42950	9.91914939
LOC_Os05g44180	LOC_Os12g34380	9.79536421
LOC_Os05g42350	LOC_Os05g51480	10.4580171
LOC_Os05g44760	LOC_Os11g25260	9.70690709
LOC_Os05g43820	LOC_Os06g04030	9.54964268
LOC_Os05g42150	LOC_Os11g26910	10.221591
LOC_Os05g42350	LOC_Os05g43510	8.83014828
LOC_Os05g44050	LOC_Os12g13810	9.63567307
LOC_Os05g43820	LOC_Os11g32650	9.4241481
LOC_Os05g44180	LOC_Os07g05150	9.65445224
LOC_Os05g42150	LOC_Os10g40710	8.92256026
LOC_Os05g42350	LOC_Os06g37610	9.57446632
LOC_Os05g44340	LOC_Os12g12514	9.31772319
LOC_Os05g42150	LOC_Os10g27174	9.20401858
LOC_Os05g44180	LOC_Os06g11800	9.40361454
LOC_Os05g44050	LOC_Os07g46830	8.94238873
LOC_Os05g44050	LOC_Os07g06440	9.01864567
LOC_Os05g44760	LOC_Os07g06410	9.82202836

InteractorA	InteractorB	Z score
LOC_Os05g44140	LOC_Os11g30310	8.91674006
LOC_Os05g43510	LOC_Os10g23900	15.6932198
LOC_Os05g45220	LOC_Os11g03290	9.57576243
LOC_Os05g44140	LOC_Os07g38860	10.0110976
LOC_Os05g44760	LOC_Os11g25100	11.0617764
LOC_Os05g44050	LOC_Os07g34580	9.6319952
LOC_Os05g42350	LOC_Os07g14590	9.99650854
LOC_Os05g42350	LOC_Os08g25570	8.80233707
LOC_Os05g43510	LOC_Os08g04540	21.8878924
LOC_Os05g42350	LOC_Os09g08910	9.23942003
LOC_Os05g45220	LOC_Os07g25024	8.88990111
LOC_Os05g45220	LOC_Os08g35740	9.31214236
LOC_Os05g46270	LOC_Os09g28770	9.43357593
LOC_Os05g45590	LOC_Os10g39590	9.12356083
LOC_Os05g46580	LOC_Os11g31620	9.48857059
LOC_Os05g45810	LOC_Os08g14570	9.41069034
LOC_Os05g46040	LOC_Os09g08720	9.21982341
LOC_Os05g45810	LOC_Os09g23550	9.23289892
LOC_Os05g45810	LOC_Os10g08550	9.0258808
LOC_Os05g46040	LOC_Os08g39860	9.22186959
LOC_Os05g46360	LOC_Os10g33900	12.4649348
LOC_Os05g45810	LOC_Os11g48110	9.45673073
LOC_Os05g46290	LOC_Os07g43470	9.10272431
LOC_Os05g45810	LOC_Os11g14910	8.86155606
LOC_Os05g46290	LOC_Os07g30640	8.99604942
LOC_Os05g45590	LOC_Os11g08445	9.11785184
LOC_Os05g46040	LOC_Os08g28680	10.5409191
LOC_Os05g45590	LOC_Os11g31620	8.85653638
LOC_Os05g45810	LOC_Os12g42876	10.1225864
LOC_Os05g46290	LOC_Os08g20270	10.8539367
LOC_Os05g45590	LOC_Os10g39840	9.80114531
LOC_Os05g45420	LOC_Os08g40170	9.34689765
LOC_Os05g45810	LOC_Os06g35560	8.91599305
LOC_Os05g46290	LOC_Os07g38860	8.8278445
LOC_Os05g45810	LOC_Os06g27770	9.69411917
LOC_Os05g45590	LOC_Os10g26050	11.0410992
LOC_Os05g45810	LOC_Os06g23870	9.63104182
LOC_Os05g46580	LOC_Os10g41550	9.06086998
LOC_Os05g46290	LOC_Os08g28800	9.03562657
LOC_Os05g46040	LOC_Os09g10270	8.92011658
LOC_Os05g46270	LOC_Os08g33820	10.6072933
LOC_Os05g46550	LOC_Os07g38430	9.68494672
LOC_Os05g46040	LOC_Os06g35814	9.13701361

InteractorA	InteractorB	Z score
LOC_Os05g45810	LOC_Os06g15420	10.4451873
LOC_Os05g46040	LOC_Os10g38140	9.00923075
LOC_Os05g46000	LOC_Os08g39860	8.8172275
LOC_Os05g46270	LOC_Os09g32640	8.90698851
LOC_Os05g46270	LOC_Os10g17660	8.85818773
LOC_Os05g46360	LOC_Os06g44270	8.85073474
LOC_Os05g46360	LOC_Os12g43450	10.0379885
LOC_Os05g45420	LOC_Os06g39708	8.96442887
LOC_Os05g45810	LOC_Os09g30412	9.42859065
LOC_Os05g45810	LOC_Os06g23760	8.80340324
LOC_Os05g45590	LOC_Os07g36130	9.10734848
LOC_Os05g46270	LOC_Os07g22930	9.06228909
LOC_Os05g46360	LOC_Os09g36800	10.4262143
LOC_Os05g46860	LOC_Os09g23560	9.86653427
LOC_Os05g46040	LOC_Os10g32550	9.7007844
LOC_Os05g45420	LOC_Os09g24924	9.88449769
LOC_Os05g46270	LOC_Os07g31750	9.20539659
LOC_Os05g46290	LOC_Os09g10230	9.66766445
LOC_Os05g45420	LOC_Os08g28820	9.10120389
LOC_Os05g46290	LOC_Os08g02400	9.15176088
LOC_Os05g45810	LOC_Os06g39875	9.35486651
LOC_Os05g46550	LOC_Os07g38540	8.95612675
LOC_Os05g46270	LOC_Os10g08580	9.4188509
LOC_Os05g45810	LOC_Os11g37640	9.74784903
LOC_Os05g45590	LOC_Os09g17620	9.3439765
LOC_Os05g45810	LOC_Os12g33610	8.82329175
LOC_Os05g46580	LOC_Os12g34062	8.92482791
LOC_Os05g46550	LOC_Os10g23100	11.003427
LOC_Os05g45590	LOC_Os07g41750	9.17990045
LOC_Os05g45810	LOC_Os11g47760	9.21729685
LOC_Os05g45590	LOC_Os06g03770	8.969981
LOC_Os05g46270	LOC_Os07g49400	9.10377808
LOC_Os05g46360	LOC_Os09g08072	9.19774231
LOC_Os05g46040	LOC_Os08g41990	9.51093918
LOC_Os05g45590	LOC_Os06g23440	8.93509662
LOC_Os05g46040	LOC_Os06g09450	9.27194817
LOC_Os05g45810	LOC_Os06g37180	9.08106003
LOC_Os05g46040	LOC_Os07g08660	9.25245994
LOC_Os05g45420	LOC_Os09g10230	10.0047261
LOC_Os05g46290	LOC_Os10g34520	9.53621891
LOC_Os05g46040	LOC_Os09g10300	9.20516634
LOC_Os05g46040	LOC_Os06g24390	8.8669435
LOC_Os05g46580	LOC_Os07g22930	9.62926386

InteractorA	InteractorB	Z score
LOC_Os05g46270	LOC_Os07g04240	9.03766688
LOC_Os05g45420	LOC_Os09g31490	9.31842255
LOC_Os05g45590	LOC_Os07g08880	11.130464
LOC_Os05g45810	LOC_Os10g34520	9.57309401
LOC_Os05g46290	LOC_Os10g31940	9.42155812
LOC_Os05g45420	LOC_Os10g40730	8.9309939
LOC_Os05g46550	LOC_Os09g10260	9.84822027
LOC_Os05g45590	LOC_Os12g22030	9.70958967
LOC_Os05g45810	LOC_Os12g13380	9.69556535
LOC_Os05g46270	LOC_Os07g34140	9.15107911
LOC_Os05g45420	LOC_Os07g46830	9.19129507
LOC_Os05g45810	LOC_Os11g10520	9.14451425
LOC_Os05g45590	LOC_Os09g24990	8.87072296
LOC_Os05g46270	LOC_Os07g48880	8.82697385
LOC_Os05g46580	LOC_Os05g47540	8.86538271
LOC_Os05g46860	LOC_Os07g22950	9.08311092
LOC_Os05g45810	LOC_Os07g03730	9.38034823
LOC_Os05g46270	LOC_Os07g46310	11.2489899
LOC_Os05g45590	LOC_Os05g48040	9.35023253
LOC_Os05g46360	LOC_Os08g32620	9.30680681
LOC_Os05g46550	LOC_Os07g01780	9.15495861
LOC_Os05g46040	LOC_Os06g36670	9.40988488
LOC_Os05g46290	LOC_Os10g28360	9.33985031
LOC_Os05g46270	LOC_Os10g17680	8.85818773
LOC_Os05g45810	LOC_Os07g46460	9.09410976
LOC_Os05g46040	LOC_Os09g23560	9.27342954
LOC_Os05g45590	LOC_Os09g23560	11.1363977
LOC_Os05g45590	LOC_Os06g21980	9.20102631
LOC_Os05g45420	LOC_Os09g07460	9.10996171
LOC_Os05g46290	LOC_Os11g08340	8.85646233
LOC_Os05g46550	LOC_Os12g01922	8.9285218
LOC_Os05g46270	LOC_Os08g04180	9.65742376
LOC_Os05g46860	LOC_Os06g49970	8.8173702
LOC_Os05g45590	LOC_Os07g06970	9.99207489
LOC_Os05g46270	LOC_Os08g44280	12.0681516
LOC_Os05g45810	LOC_Os06g46372	9.55489747
LOC_Os05g46580	LOC_Os06g08600	9.19621449
LOC_Os05g45420	LOC_Os11g34450	9.03445187
LOC_Os05g45420	LOC_Os07g46310	9.42299234
LOC_Os05g46290	LOC_Os12g44010	8.88178123
LOC_Os05g45420	LOC_Os06g23440	9.28907824
LOC_Os05g46270	LOC_Os08g39420	9.51137617
LOC_Os05g46550	LOC_Os11g32650	8.99906242

InteractorA	InteractorB	Z score
LOC_Os05g45590	LOC_Os12g40830	9.47011698
LOC_Os05g45810	LOC_Os07g22650	8.84342872
LOC_Os05g46290	LOC_Os08g28190	9.41821293
LOC_Os05g45590	LOC_Os07g01760	10.0981005
LOC_Os05g46860	LOC_Os08g34290	9.08054041
LOC_Os05g45590	LOC_Os07g39870	9.03848687
LOC_Os05g46040	LOC_Os12g10720	8.82561228
LOC_Os05g45810	LOC_Os09g28460	9.76476761
LOC_Os05g45810	LOC_Os05g49830	8.82005547
LOC_Os05g45420	LOC_Os08g34210	9.74507384
LOC_Os05g46580	LOC_Os07g49120	9.8308969
LOC_Os05g45590	LOC_Os11g25100	8.93370093
LOC_Os05g46270	LOC_Os12g02980	10.6362766
LOC_Os05g45590	LOC_Os06g45100	9.63436675
LOC_Os05g46360	LOC_Os09g37540	9.41417746
LOC_Os05g48510	LOC_Os07g46630	8.81550438
LOC_Os05g48510	LOC_Os07g34260	9.42614481
LOC_Os05g47540	LOC_Os06g50300	8.90386603
LOC_Os05g47980	LOC_Os07g32800	8.86569092
LOC_Os05g48510	LOC_Os09g25390	11.3196192
LOC_Os05g48030	LOC_Os11g32620	9.32607507
LOC_Os05g47545	LOC_Os06g39708	8.97223826
LOC_Os05g48290	LOC_Os10g27174	10.9402731
LOC_Os05g47540	LOC_Os10g39120	10.5003069
LOC_Os05g48510	LOC_Os10g13800	10.3949628
LOC_Os05g48040	LOC_Os06g02144	9.78608249
LOC_Os05g48980	LOC_Os07g46280	9.87377767
LOC_Os05g48290	LOC_Os09g20284	9.21607653
LOC_Os05g47640	LOC_Os06g39740	9.26987444
LOC_Os05g47890	LOC_Os10g29470	8.82743466
LOC_Os05g48510	LOC_Os10g22450	9.31550146
LOC_Os05g47545	LOC_Os07g48430	10.6019176
LOC_Os05g47545	LOC_Os12g34450	8.94222029
LOC_Os05g48510	LOC_Os09g24924	9.09789079
LOC_Os05g48290	LOC_Os11g29190	9.30635014
LOC_Os05g47890	LOC_Os11g03230	9.74611419
LOC_Os05g48980	LOC_Os06g30970	11.4250528
LOC_Os05g48040	LOC_Os12g13380	9.17245402
LOC_Os05g47980	LOC_Os10g25130	8.85153981
LOC_Os05g48510	LOC_Os06g11240	9.1927546
LOC_Os05g47980	LOC_Os11g31530	8.92833843
LOC_Os05g46860	LOC_Os10g32870	9.08992313
LOC_Os05g47540	LOC_Os12g10560	8.88634671

InteractorA	InteractorB	Z score
LOC_Os05g48290	LOC_Os10g30580	9.24554333
LOC_Os05g48980	LOC_Os06g35480	9.43882215
LOC_Os05g47540	LOC_Os12g33610	9.08646208
LOC_Os05g46860	LOC_Os12g40830	9.10026159
LOC_Os05g48510	LOC_Os07g44740	9.19597571
LOC_Os05g47540	LOC_Os11g32650	12.5034
LOC_Os05g48040	LOC_Os11g38959	8.83043952
LOC_Os05g47980	LOC_Os08g06060	9.26469165
LOC_Os05g47980	LOC_Os06g40180	9.24384913
LOC_Os05g47640	LOC_Os12g01922	10.2514697
LOC_Os05g47640	LOC_Os09g34214	9.10163467
LOC_Os05g48510	LOC_Os08g40170	8.87210538
LOC_Os05g48510	LOC_Os07g40290	8.85722138
LOC_Os05g47540	LOC_Os08g44210	9.48186359
LOC_Os05g47540	LOC_Os09g28770	10.1248431
LOC_Os05g48510	LOC_Os09g21770	10.5582328
LOC_Os05g48980	LOC_Os11g25100	9.15075016
LOC_Os05g48030	LOC_Os11g03290	8.81420194
LOC_Os05g48510	LOC_Os10g39680	11.9615121
LOC_Os05g48040	LOC_Os11g26850	10.2858792
LOC_Os05g47540	LOC_Os06g06090	9.21012615
LOC_Os05g47640	LOC_Os10g08670	9.32995399
LOC_Os05g48510	LOC_Os12g14070	9.02774439
LOC_Os05g47540	LOC_Os06g35700	9.7594538
LOC_Os05g47640	LOC_Os06g01850	9.31590265
LOC_Os05g48980	LOC_Os10g21344	9.43078092
LOC_Os05g47540	LOC_Os12g08270	9.93365824
LOC_Os05g48510	LOC_Os11g25260	9.04205946
LOC_Os05g47545	LOC_Os12g12470	9.45983159
LOC_Os05g47545	LOC_Os12g38770	9.15700645
LOC_Os05g47640	LOC_Os08g44960	8.85303471
LOC_Os05g47540	LOC_Os10g21240	10.1754919
LOC_Os05g48290	LOC_Os06g34690	8.84051176
LOC_Os05g48040	LOC_Os06g36820	9.28823335
LOC_Os05g47980	LOC_Os11g42350	9.70392993
LOC_Os05g48030	LOC_Os08g37490	9.64716801
LOC_Os05g47540	LOC_Os08g06060	9.38376726
LOC_Os05g47640	LOC_Os06g39140	9.64764832
LOC_Os05g47540	LOC_Os08g39870	8.94309644
LOC_Os05g48290	LOC_Os10g27050	9.63187286
LOC_Os05g48980	LOC_Os08g36910	9.53425673
LOC_Os05g47540	LOC_Os10g06630	9.32610643
LOC_Os05g47640	LOC_Os05g51670	9.98299006

InteractorA	InteractorB	Z score
LOC_Os05g47640	LOC_Os12g36950	9.36876407
LOC_Os05g48290	LOC_Os07g40290	8.84077589
LOC_Os05g47640	LOC_Os11g25260	9.74639889
LOC_Os05g47980	LOC_Os09g38030	9.17492413
LOC_Os05g47545	LOC_Os05g49800	9.11527834
LOC_Os05g48510	LOC_Os08g28190	9.5897803
LOC_Os05g48290	LOC_Os11g33270	9.98180581
LOC_Os05g47890	LOC_Os10g05069	9.05584444
LOC_Os05g48510	LOC_Os12g24650	9.4106957
LOC_Os05g47640	LOC_Os05g49760	8.89998792
LOC_Os05g48030	LOC_Os06g22140	9.40138649
LOC_Os05g47540	LOC_Os10g21266	10.9205488
LOC_Os05g48510	LOC_Os10g37210	9.20788049
LOC_Os05g48290	LOC_Os12g41220	9.26674042
LOC_Os05g47545	LOC_Os12g22650	10.523427
LOC_Os05g48290	LOC_Os10g08580	10.277902
LOC_Os05g47540	LOC_Os05g50380	9.59476273
LOC_Os05g48510	LOC_Os10g38610	8.91743864
LOC_Os05g47545	LOC_Os12g18880	9.3937655
LOC_Os05g47540	LOC_Os08g35420	10.0571609
LOC_Os05g48510	LOC_Os06g11290	9.33283397
LOC_Os05g47640	LOC_Os06g29180	9.1128261
LOC_Os05g48980	LOC_Os07g05400	9.87361173
LOC_Os05g47640	LOC_Os09g27820	10.2256903
LOC_Os05g47890	LOC_Os07g39290	9.04278022
LOC_Os05g48290	LOC_Os09g16910	10.0700801
LOC_Os05g47640	LOC_Os07g05180	8.86935932
LOC_Os05g47540	LOC_Os10g21192	8.85545019
LOC_Os05g48510	LOC_Os07g34140	8.86872831
LOC_Os05g48510	LOC_Os10g42940	9.86705221
LOC_Os05g48040	LOC_Os07g30990	9.57432323
LOC_Os05g47540	LOC_Os07g14590	8.82371141
LOC_Os05g49760	LOC_Os09g30418	9.13403416
LOC_Os05g49770	LOC_Os07g27790	9.39001857
LOC_Os05g50890	LOC_Os09g31506	9.28483345
LOC_Os05g49770	LOC_Os08g37490	9.78943309
LOC_Os05g49760	LOC_Os11g32650	8.82550393
LOC_Os05g49830	LOC_Os12g14070	10.4615935
LOC_Os05g49840	LOC_Os10g37210	9.10644435
LOC_Os05g49800	LOC_Os12g42876	9.82052212
LOC_Os05g49830	LOC_Os06g11290	9.38375316
LOC_Os05g50380	LOC_Os09g25320	9.65102591
LOC_Os05g49890	LOC_Os10g02040	9.72354985

InteractorA	InteractorB	Z score
LOC_Os05g50710	LOC_Os07g44260	8.82994524
LOC_Os05g50890	LOC_Os09g23540	8.81547364
LOC_Os05g50380	LOC_Os08g34210	10.1908092
LOC_Os05g50890	LOC_Os09g10200	10.1355727
LOC_Os05g49830	LOC_Os09g20090	8.84342551
LOC_Os05g49830	LOC_Os11g25260	11.7252962
LOC_Os05g49880	LOC_Os09g10260	10.7902634
LOC_Os05g49770	LOC_Os11g48110	8.8988179
LOC_Os05g49760	LOC_Os12g42884	9.26775723
LOC_Os05g49880	LOC_Os11g32650	9.01482888
LOC_Os05g50890	LOC_Os12g37360	9.28992381
LOC_Os05g50890	LOC_Os07g37550	9.4605601
LOC_Os05g50890	LOC_Os11g25700	9.22306361
LOC_Os05g49830	LOC_Os09g24412	9.91748704
LOC_Os05g49880	LOC_Os08g32620	8.93250464
LOC_Os05g50710	LOC_Os08g44340	9.25014778
LOC_Os05g49770	LOC_Os09g30418	9.08177321
LOC_Os05g49760	LOC_Os06g40170	9.19136774
LOC_Os05g49760	LOC_Os10g22450	9.20437686
LOC_Os05g49800	LOC_Os06g40940	8.94109105
LOC_Os05g49770	LOC_Os06g35520	8.9038398
LOC_Os05g49830	LOC_Os09g39380	11.2470937
LOC_Os05g50380	LOC_Os10g38580	9.14757538
LOC_Os05g50710	LOC_Os09g35800	10.3368874
LOC_Os05g49880	LOC_Os07g46280	8.98831033
LOC_Os05g49880	LOC_Os10g11140	9.15108229
LOC_Os05g49770	LOC_Os12g34380	9.76858478
LOC_Os05g50380	LOC_Os10g39680	8.84328398
LOC_Os05g49830	LOC_Os07g07320	8.91425069
LOC_Os05g49760	LOC_Os07g39870	9.16658503
LOC_Os05g49770	LOC_Os12g10720	10.1826806
LOC_Os05g49800	LOC_Os05g49890	10.2699256
LOC_Os05g50710	LOC_Os11g32610	9.15275903
LOC_Os05g50710	LOC_Os07g14590	9.21196052
LOC_Os05g49840	LOC_Os12g41110	9.18906489
LOC_Os05g49770	LOC_Os06g40180	9.73267323
LOC_Os05g49760	LOC_Os07g28280	8.96537608
LOC_Os05g50380	LOC_Os07g48050	8.96147208
LOC_Os05g49770	LOC_Os07g30970	8.96802302
LOC_Os05g49770	LOC_Os10g21248	9.36175319
LOC_Os05g49770	LOC_Os08g39140	8.91902961
LOC_Os05g50380	LOC_Os06g44080	9.65105156
LOC_Os05g49830	LOC_Os10g21344	9.86322433

InteractorA	InteractorB	Z score
LOC_Os05g49830	LOC_Os07g25024	9.91747665
LOC_Os05g50710	LOC_Os08g44810	9.25179109
LOC_Os05g50710	LOC_Os09g07830	9.15076707
LOC_Os05g49760	LOC_Os06g21570	8.87072622
LOC_Os05g49890	LOC_Os07g46830	8.92881415
LOC_Os05g49770	LOC_Os10g40090	9.57429159
LOC_Os05g49830	LOC_Os05g51480	10.5364107
LOC_Os05g50890	LOC_Os11g30310	9.14031425
LOC_Os05g49760	LOC_Os07g39270	12.2904915
LOC_Os05g49760	LOC_Os10g07616	9.45798972
LOC_Os05g49770	LOC_Os06g04280	9.33249625
LOC_Os05g49830	LOC_Os09g14670	8.8391534
LOC_Os05g49760	LOC_Os10g30840	8.83584607
LOC_Os05g49200	LOC_Os08g35440	9.43854724
LOC_Os05g49880	LOC_Os10g02040	9.19386923
LOC_Os05g49830	LOC_Os09g09520	8.87903757
LOC_Os05g49760	LOC_Os11g01360	8.80075901
LOC_Os05g49800	LOC_Os07g05820	9.39969351
LOC_Os05g50890	LOC_Os07g04240	8.87577598
LOC_Os05g49840	LOC_Os06g39875	10.2116855
LOC_Os05g49830	LOC_Os07g05160	9.20609537
LOC_Os05g49830	LOC_Os10g21352	9.91748704
LOC_Os05g49760	LOC_Os11g34570	9.6982489
LOC_Os05g50710	LOC_Os11g38959	8.99246812
LOC_Os05g49770	LOC_Os10g07616	9.10678506
LOC_Os05g51050	LOC_Os06g06100	8.85357593
LOC_Os05g50710	LOC_Os11g08440	8.83702819
LOC_Os05g49840	LOC_Os10g27050	9.1296225
LOC_Os05g49830	LOC_Os06g37150	9.31598076
LOC_Os05g50890	LOC_Os08g44270	9.89491188
LOC_Os05g49890	LOC_Os12g22650	9.20991912
LOC_Os05g50710	LOC_Os12g44030	9.35070495
LOC_Os05g49200	LOC_Os12g12470	9.48594854
LOC_Os05g49880	LOC_Os12g10730	8.80693106
LOC_Os05g49880	LOC_Os08g41340	8.8077132
LOC_Os05g49770	LOC_Os12g36950	9.99776425
LOC_Os05g49770	LOC_Os12g16410	10.2774472
LOC_Os05g50380	LOC_Os11g14040	9.19019177
LOC_Os05g49800	LOC_Os12g06620	8.87366722
LOC_Os05g49760	LOC_Os11g01872	9.61490304
LOC_Os05g49760	LOC_Os11g16590	9.08064174
LOC_Os05g50890	LOC_Os08g41830	9.43818593
LOC_Os05g50890	LOC_Os07g38540	9.03137539

InteractorA	InteractorB	Z score
LOC_Os05g50890	LOC_Os06g11200	9.02856005
LOC_Os05g49800	LOC_Os11g36719	9.23011686
LOC_Os05g49880	LOC_Os11g33240	10.3653496
LOC_Os05g49890	LOC_Os08g33710	9.05970696
LOC_Os05g51050	LOC_Os06g45710	9.35490431
LOC_Os05g49800	LOC_Os11g32540	8.86330793
LOC_Os05g49830	LOC_Os08g15266	9.91748704
LOC_Os05g49840	LOC_Os12g25120	11.365271
LOC_Os05g50710	LOC_Os10g41510	9.11145134
LOC_Os05g49890	LOC_Os07g07550	9.57399503
LOC_Os05g50890	LOC_Os08g28800	9.73452197
LOC_Os05g49770	LOC_Os07g43390	9.8828923
LOC_Os05g49770	LOC_Os08g34170	8.84103353
LOC_Os05g49760	LOC_Os06g09450	8.99218458
LOC_Os05g49880	LOC_Os10g32680	9.30477816
LOC_Os05g50380	LOC_Os10g14150	8.85897687
LOC_Os05g49880	LOC_Os10g32970	9.16529972
LOC_Os05g49800	LOC_Os09g31410	10.0190317
LOC_Os05g49880	LOC_Os09g10200	9.06971631
LOC_Os05g49760	LOC_Os08g20730	9.24544175
LOC_Os05g49880	LOC_Os06g06050	9.09784991
LOC_Os05g51670	LOC_Os07g44460	8.85217753
LOC_Os06g01850	LOC_Os12g03816	8.92499487
LOC_Os06g01850	LOC_Os10g08620	10.4156606
LOC_Os05g51050	LOC_Os11g37970	8.93451922
LOC_Os06g02144	LOC_Os11g06390	8.88629639
LOC_Os06g01610	LOC_Os11g08470	8.82150584
LOC_Os05g51480	LOC_Os08g43560	9.10446092
LOC_Os06g01390	LOC_Os06g29180	8.85776914
LOC_Os05g51630	LOC_Os08g09370	8.91383647
LOC_Os05g51050	LOC_Os10g35480	8.81948431
LOC_Os06g02144	LOC_Os09g08910	9.81746414
LOC_Os06g01590	LOC_Os10g38740	9.2210812
LOC_Os06g01360	LOC_Os09g08072	9.02423058
LOC_Os05g51480	LOC_Os06g09450	8.81435751
LOC_Os06g01610	LOC_Os09g36830	8.82951956
LOC_Os06g01360	LOC_Os07g48780	9.27550788
LOC_Os05g51630	LOC_Os11g19220	9.12551801
LOC_Os05g51670	LOC_Os11g03290	9.18477329
LOC_Os05g51480	LOC_Os07g28480	10.3985348
LOC_Os05g51570	LOC_Os06g35630	9.48440111
LOC_Os05g51480	LOC_Os09g10200	9.48552296
LOC_Os05g51480	LOC_Os08g28800	10.0835459

InteractorA	InteractorB	Z score
LOC_Os05g51570	LOC_Os09g20820	9.68124405
LOC_Os05g51480	LOC_Os08g15030	9.03928082
LOC_Os06g01850	LOC_Os09g25150	8.86215142
LOC_Os05g51630	LOC_Os06g08770	8.81840039
LOC_Os05g51670	LOC_Os10g30840	9.50324938
LOC_Os06g01360	LOC_Os11g08120	9.71986361
LOC_Os05g51570	LOC_Os10g08670	9.21953131
LOC_Os05g51670	LOC_Os10g32680	8.854118
LOC_Os05g51050	LOC_Os09g27750	9.2766991
LOC_Os05g51670	LOC_Os10g38470	8.94761664
LOC_Os06g01390	LOC_Os09g31430	9.05276316
LOC_Os05g51570	LOC_Os08g02400	10.3151422
LOC_Os05g51670	LOC_Os11g10510	10.0924057
LOC_Os06g01850	LOC_Os11g34570	9.01864583
LOC_Os06g02144	LOC_Os09g30360	9.15951973
LOC_Os06g01610	LOC_Os08g40140	9.4358253
LOC_Os06g01360	LOC_Os07g03368	9.71088795
LOC_Os05g51480	LOC_Os06g35730	8.90406062
LOC_Os06g02144	LOC_Os12g37360	8.98696855
LOC_Os06g01390	LOC_Os08g42560	8.93349288
LOC_Os06g01850	LOC_Os12g40830	9.26745137
LOC_Os06g01850	LOC_Os09g31430	10.1276422
LOC_Os06g01590	LOC_Os08g39860	9.59715772
LOC_Os06g01850	LOC_Os08g14770	8.90089421
LOC_Os05g51570	LOC_Os07g43470	8.9215881
LOC_Os05g51480	LOC_Os11g32260	8.87668926
LOC_Os06g01390	LOC_Os11g32620	9.97942917
LOC_Os05g51570	LOC_Os09g31506	9.49687135
LOC_Os05g51050	LOC_Os08g28800	9.14888514
LOC_Os05g51670	LOC_Os10g28320	8.96097038
LOC_Os05g51570	LOC_Os07g05150	8.84946217
LOC_Os06g01850	LOC_Os07g44370	8.8455067
LOC_Os06g01390	LOC_Os10g34020	9.41902583
LOC_Os06g01850	LOC_Os07g31770	9.72484233
LOC_Os05g51480	LOC_Os07g39870	9.30817558
LOC_Os05g51050	LOC_Os09g38030	9.07322555
LOC_Os06g01850	LOC_Os12g16220	9.54321757
LOC_Os05g51630	LOC_Os12g02980	9.63279961
LOC_Os06g01390	LOC_Os10g40710	9.42504588
LOC_Os06g01360	LOC_Os12g22650	8.96561351
LOC_Os06g01360	LOC_Os06g09450	9.47439209
LOC_Os05g51570	LOC_Os11g26860	10.3329444
LOC_Os05g51570	LOC_Os06g29180	9.70511311

InteractorA	InteractorB	Z score
LOC_Os05g51570	LOC_Os06g04200	9.89110691
LOC_Os06g01850	LOC_Os06g36700	9.1790345
LOC_Os05g51480	LOC_Os07g34140	8.94261186
LOC_Os06g01360	LOC_Os06g36770	9.25068952
LOC_Os06g01590	LOC_Os12g25710	9.01082644
LOC_Os05g51050	LOC_Os10g38140	8.99448818
LOC_Os05g51050	LOC_Os07g23730	9.66017096
LOC_Os06g01390	LOC_Os10g28050	9.54900075
LOC_Os06g01590	LOC_Os11g02440	9.04081759
LOC_Os06g02144	LOC_Os07g43820	9.13027816
LOC_Os06g01390	LOC_Os08g37800	9.35115117
LOC_Os05g51670	LOC_Os12g05410	10.286643
LOC_Os05g51570	LOC_Os08g09940	9.80343
LOC_Os05g51670	LOC_Os11g19220	9.97604945
LOC_Os06g04030	LOC_Os06g27770	9.95561971
LOC_Os06g02390	LOC_Os07g49120	9.51117741
LOC_Os06g04200	LOC_Os10g13800	9.05937546
LOC_Os06g02490	LOC_Os12g31370	8.9807471
LOC_Os06g02380	LOC_Os06g04030	9.69247786
LOC_Os06g02380	LOC_Os07g06970	10.2701855
LOC_Os06g04000	LOC_Os10g38234	8.93851916
LOC_Os06g04030	LOC_Os10g08022	8.87716861
LOC_Os06g04030	LOC_Os11g01872	9.64037799
LOC_Os06g02490	LOC_Os10g30840	9.33834858
LOC_Os06g04270	LOC_Os10g38950	9.33280753
LOC_Os06g02220	LOC_Os11g10510	9.09500103
LOC_Os06g02380	LOC_Os08g34210	9.36974291
LOC_Os06g03770	LOC_Os11g42350	9.41194526
LOC_Os06g04200	LOC_Os07g07719	8.81693673
LOC_Os06g03770	LOC_Os12g43370	9.26193823
LOC_Os06g02490	LOC_Os06g12790	11.0428586
LOC_Os06g02220	LOC_Os10g38340	9.12981161
LOC_Os06g04000	LOC_Os09g23560	8.88567381
LOC_Os06g04200	LOC_Os08g40140	8.81551995
LOC_Os06g04200	LOC_Os09g24990	11.8344534
LOC_Os06g04510	LOC_Os06g11210	9.00739716
LOC_Os06g02390	LOC_Os09g25390	9.63286004
LOC_Os06g04030	LOC_Os06g12090	8.81349276
LOC_Os06g03720	LOC_Os12g13810	9.10664668
LOC_Os06g02390	LOC_Os09g10260	9.37878673
LOC_Os06g04000	LOC_Os07g44430	9.28582192
LOC_Os06g03720	LOC_Os06g40180	9.77504229
LOC_Os06g03720	LOC_Os12g16200	8.90692019

InteractorA	InteractorB	Z score
LOC_Os06g03720	LOC_Os07g47290	9.07806456
LOC_Os06g04270	LOC_Os06g40180	8.86671057
LOC_Os06g04030	LOC_Os06g49970	9.41132496
LOC_Os06g04030	LOC_Os07g35880	10.452679
LOC_Os06g03770	LOC_Os12g40550	11.1642874
LOC_Os06g04200	LOC_Os06g44620	10.4557188
LOC_Os06g04200	LOC_Os09g32952	9.13929775
LOC_Os06g04200	LOC_Os10g38340	10.6810697
LOC_Os06g04030	LOC_Os08g28730	8.87512854
LOC_Os06g04200	LOC_Os08g02400	10.2404856
LOC_Os06g04200	LOC_Os08g34170	9.21230978
LOC_Os06g04000	LOC_Os10g21326	9.16868579
LOC_Os06g04200	LOC_Os07g38910	9.33579659
LOC_Os06g04280	LOC_Os10g21310	9.79799675
LOC_Os06g04270	LOC_Os09g09230	9.03272848
LOC_Os06g03770	LOC_Os09g24412	10.0257261
LOC_Os06g03770	LOC_Os08g15266	10.0257261
LOC_Os06g04000	LOC_Os06g35730	11.0436435
LOC_Os06g03770	LOC_Os10g21352	10.0257261
LOC_Os06g03770	LOC_Os08g09210	9.31064074
LOC_Os06g04280	LOC_Os11g40150	8.91725999
LOC_Os06g04030	LOC_Os08g44280	9.43284704
LOC_Os06g02390	LOC_Os11g26910	9.03586333
LOC_Os06g04030	LOC_Os07g05160	9.72198133
LOC_Os06g04000	LOC_Os07g38860	9.80556163
LOC_Os06g02390	LOC_Os11g08445	9.40811611
LOC_Os06g04280	LOC_Os08g40140	10.1860705
LOC_Os06g02390	LOC_Os11g16590	9.97101944
LOC_Os06g04280	LOC_Os12g44010	9.08661986
LOC_Os06g04030	LOC_Os08g39140	8.88560886
LOC_Os06g04030	LOC_Os09g28400	9.22810199
LOC_Os06g03770	LOC_Os11g26860	8.8630294
LOC_Os06g03770	LOC_Os07g25024	10.0257261
LOC_Os06g04510	LOC_Os07g07709	8.81715555
LOC_Os06g03770	LOC_Os11g01872	8.9681894
LOC_Os06g03720	LOC_Os06g09450	8.94573566
LOC_Os06g04000	LOC_Os06g11210	9.44947065
LOC_Os06g04000	LOC_Os08g44210	10.2728704
LOC_Os06g04030	LOC_Os12g22680	8.99889434
LOC_Os06g03720	LOC_Os11g40140	10.9032046
LOC_Os06g02380	LOC_Os10g21248	9.13004436
LOC_Os06g04030	LOC_Os11g03230	9.07767105
LOC_Os06g02490	LOC_Os08g41880	9.32027618

InteractorA	InteractorB	Z score
LOC_Os06g02380	LOC_Os08g09250	8.94259264
LOC_Os06g04280	LOC_Os09g39810	9.59607541
LOC_Os06g04030	LOC_Os09g15320	8.95631276
LOC_Os06g04000	LOC_Os12g16240	9.2241688
LOC_Os06g04200	LOC_Os12g25700	8.99283577
LOC_Os06g02380	LOC_Os10g33800	9.23335351
LOC_Os06g04030	LOC_Os06g11800	9.43564951
LOC_Os06g04270	LOC_Os10g32550	9.49455777
LOC_Os06g03770	LOC_Os12g44030	8.94092251
LOC_Os06g02390	LOC_Os06g47320	8.95521542
LOC_Os06g04280	LOC_Os06g09910	10.1275899
LOC_Os06g04000	LOC_Os08g03290	9.35029366
LOC_Os06g02380	LOC_Os06g51084	9.20155178
LOC_Os06g04030	LOC_Os12g12560	9.96735601
LOC_Os06g04270	LOC_Os11g47560	8.89827089
LOC_Os06g04000	LOC_Os07g44550	8.94789551
LOC_Os06g04270	LOC_Os09g28400	10.5464453
LOC_Os06g04280	LOC_Os06g36670	9.6243723
LOC_Os06g04200	LOC_Os10g33900	8.99035777
LOC_Os06g04270	LOC_Os06g14620	10.1368827
LOC_Os06g04200	LOC_Os11g01360	9.3484532
LOC_Os06g04270	LOC_Os06g15990	9.87229898
LOC_Os06g04200	LOC_Os09g32640	9.32479646
LOC_Os06g02390	LOC_Os12g10730	9.141078
LOC_Os06g04270	LOC_Os06g35520	9.38864345
LOC_Os06g03770	LOC_Os10g38160	8.83488016
LOC_Os06g04000	LOC_Os11g04954	9.12882519
LOC_Os06g04270	LOC_Os06g51084	10.5798676
LOC_Os06g02490	LOC_Os07g03368	8.90657612
LOC_Os06g04280	LOC_Os07g43470	9.19567033
LOC_Os06g04030	LOC_Os07g44440	10.4293262
LOC_Os06g06040	LOC_Os07g10590	9.40277364
LOC_Os06g06050	LOC_Os07g01760	8.85032433
LOC_Os06g05250	LOC_Os08g28800	9.90984917
LOC_Os06g05250	LOC_Os12g25690	8.96060741
LOC_Os06g05110	LOC_Os07g01020	9.72987076
LOC_Os06g05690	LOC_Os10g41490	9.22478733
LOC_Os06g06090	LOC_Os08g28800	10.7007192
LOC_Os06g05690	LOC_Os09g10200	9.4452955
LOC_Os06g05690	LOC_Os10g38160	11.9252168
LOC_Os06g06050	LOC_Os07g01560	10.5579818
LOC_Os06g04620	LOC_Os11g34570	9.33276246
LOC_Os06g04510	LOC_Os12g36950	9.03086484

InteractorA	InteractorB	Z score
LOC_Os06g06090	LOC_Os06g06100	10.5495623
LOC_Os06g05250	LOC_Os08g32850	11.3520353
LOC_Os06g06050	LOC_Os06g46436	10.0798589
LOC_Os06g06050	LOC_Os08g44530	9.02170618
LOC_Os06g05090	LOC_Os10g08620	9.29248256
LOC_Os06g05110	LOC_Os09g23560	8.82606103
LOC_Os06g05090	LOC_Os09g36900	9.37443882
LOC_Os06g06050	LOC_Os11g02130	9.56926389
LOC_Os06g04900	LOC_Os08g20730	8.80555082
LOC_Os06g05250	LOC_Os10g08550	10.8993396
LOC_Os06g06050	LOC_Os07g22498	10.0798331
LOC_Os06g06040	LOC_Os12g12514	9.22721527
LOC_Os06g04620	LOC_Os08g06550	9.03270345
LOC_Os06g06050	LOC_Os07g05160	9.93969322
LOC_Os06g04620	LOC_Os06g36670	9.40319065
LOC_Os06g06090	LOC_Os08g04540	8.86360994
LOC_Os06g04510	LOC_Os07g28280	9.43304026
LOC_Os06g06040	LOC_Os12g44020	9.51807826
LOC_Os06g05250	LOC_Os06g21570	9.57566666
LOC_Os06g06050	LOC_Os12g39630	10.3699632
LOC_Os06g05110	LOC_Os08g34210	9.28452104
LOC_Os06g05110	LOC_Os07g49220	10.155538
LOC_Os06g05690	LOC_Os07g46310	10.1555996
LOC_Os06g05880	LOC_Os08g04560	11.2635098
LOC_Os06g06050	LOC_Os09g10260	8.80379556
LOC_Os06g06050	LOC_Os10g40720	9.33182164
LOC_Os06g05700	LOC_Os10g26110	9.23629627
LOC_Os06g05250	LOC_Os10g22450	9.01710956
LOC_Os06g05880	LOC_Os12g40510	11.6689236
LOC_Os06g05880	LOC_Os06g15990	9.10046772
LOC_Os06g05690	LOC_Os06g40180	9.30915288
LOC_Os06g06050	LOC_Os06g46340	8.88731914
LOC_Os06g06050	LOC_Os11g32260	9.18554052
LOC_Os06g06040	LOC_Os07g05400	9.18911381
LOC_Os06g05700	LOC_Os06g48180	9.39203478
LOC_Os06g06050	LOC_Os11g48110	9.98748011
LOC_Os06g04510	LOC_Os12g12514	9.11989999
LOC_Os06g06090	LOC_Os06g09910	9.89281788
LOC_Os06g05690	LOC_Os12g42876	9.87311645
LOC_Os06g06050	LOC_Os07g46310	10.0401
LOC_Os06g04620	LOC_Os11g32580	9.7170151
LOC_Os06g06050	LOC_Os08g02340	9.23344827
LOC_Os06g05110	LOC_Os07g44430	9.15312631

InteractorA	InteractorB	Z score
LOC_Os06g05250	LOC_Os07g48430	9.58300306
LOC_Os06g05090	LOC_Os11g04880	8.96657357
LOC_Os06g05700	LOC_Os10g02380	9.16107278
LOC_Os06g05250	LOC_Os11g47580	9.31278781
LOC_Os06g04510	LOC_Os08g09210	9.25789494
LOC_Os06g04620	LOC_Os12g16250	9.77243517
LOC_Os06g05250	LOC_Os08g34210	10.9308546
LOC_Os06g06050	LOC_Os12g22030	8.80369358
LOC_Os06g05110	LOC_Os11g08445	8.86037246
LOC_Os06g06040	LOC_Os06g44080	8.94620436
LOC_Os06g05700	LOC_Os06g11800	9.23458375
LOC_Os06g05690	LOC_Os10g38340	9.88489504
LOC_Os06g05690	LOC_Os12g38770	10.2479618
LOC_Os06g06050	LOC_Os12g22680	10.4264618
LOC_Os06g06050	LOC_Os08g09770	8.92883549
LOC_Os06g06050	LOC_Os10g21406	10.0798331
LOC_Os06g04620	LOC_Os10g38580	9.73291515
LOC_Os06g06040	LOC_Os06g06880	10.4535534
LOC_Os06g05690	LOC_Os10g21326	9.6508851
LOC_Os06g05250	LOC_Os12g10560	9.17938768
LOC_Os06g05250	LOC_Os08g09770	10.356956
LOC_Os06g05690	LOC_Os11g26910	9.35983187
LOC_Os06g05690	LOC_Os08g09250	9.00159689
LOC_Os06g05700	LOC_Os11g08330	8.84507453
LOC_Os06g05250	LOC_Os09g20260	10.1635137
LOC_Os06g06090	LOC_Os08g10010	9.40097856
LOC_Os06g06040	LOC_Os09g25320	8.94620436
LOC_Os06g05250	LOC_Os07g12200	11.5049532
LOC_Os06g05700	LOC_Os09g04050	9.34235874
LOC_Os06g04510	LOC_Os09g23560	8.99270202
LOC_Os06g05700	LOC_Os06g42130	9.10861974
LOC_Os06g06050	LOC_Os07g38730	8.86990696
LOC_Os06g06050	LOC_Os07g38030	9.35578785
LOC_Os06g05110	LOC_Os08g31060	8.88156684
LOC_Os06g06050	LOC_Os06g35530	9.43394856
LOC_Os06g06040	LOC_Os09g27820	8.854045
LOC_Os06g04620	LOC_Os10g40710	9.06797527
LOC_Os06g05250	LOC_Os09g37949	10.3674641
LOC_Os06g06050	LOC_Os09g09270	10.6914639
LOC_Os06g05250	LOC_Os10g41550	8.87233489
LOC_Os06g05700	LOC_Os07g49120	9.75159464
LOC_Os06g06560	LOC_Os11g14910	10.247663
LOC_Os06g06090	LOC_Os09g31490	9.45747609

InteractorA	InteractorB	Z score
LOC_Os06g06510	LOC_Os06g36770	9.28017027
LOC_Os06g06560	LOC_Os11g42350	9.25203393
LOC_Os06g06460	LOC_Os12g22680	9.68891059
LOC_Os06g06510	LOC_Os08g04180	8.84954596
LOC_Os06g06560	LOC_Os09g08880	12.3357955
LOC_Os06g07080	LOC_Os11g33270	8.95582594
LOC_Os06g06100	LOC_Os12g12590	9.12243994
LOC_Os06g06980	LOC_Os09g24990	9.03956834
LOC_Os06g06460	LOC_Os10g38160	8.95621906
LOC_Os06g07080	LOC_Os06g48180	8.92572672
LOC_Os06g06460	LOC_Os06g37180	9.2221175
LOC_Os06g06560	LOC_Os07g03319	9.5937957
LOC_Os06g06100	LOC_Os11g26910	9.99901703
LOC_Os06g06100	LOC_Os07g22930	9.77585679
LOC_Os06g06510	LOC_Os06g37180	9.2220983
LOC_Os06g06090	LOC_Os12g43450	9.03892735
LOC_Os06g06320	LOC_Os10g28050	9.38078659
LOC_Os06g07080	LOC_Os06g43660	9.41233905
LOC_Os06g06730	LOC_Os12g07980	9.32436919
LOC_Os06g06460	LOC_Os10g07616	9.5223222
LOC_Os06g06090	LOC_Os10g28320	9.09325178
LOC_Os06g06880	LOC_Os09g04050	9.43022503
LOC_Os06g06320	LOC_Os09g12660	8.99252191
LOC_Os06g06090	LOC_Os09g27820	9.67484886
LOC_Os06g06460	LOC_Os08g04180	8.84954596
LOC_Os06g06560	LOC_Os07g03409	9.59381805
LOC_Os06g06510	LOC_Os07g44550	10.5558631
LOC_Os06g06560	LOC_Os07g03499	9.59381692
LOC_Os06g06730	LOC_Os12g38770	9.94065539
LOC_Os06g07080	LOC_Os06g12990	10.2830677
LOC_Os06g06510	LOC_Os09g12590	10.6579221
LOC_Os06g06460	LOC_Os12g10730	9.13435634
LOC_Os06g06560	LOC_Os07g47290	9.13979387
LOC_Os06g06730	LOC_Os10g30200	8.99508529
LOC_Os06g06980	LOC_Os07g05940	9.04645254
LOC_Os06g06560	LOC_Os09g10270	8.87961462
LOC_Os06g06090	LOC_Os09g32952	8.98499677
LOC_Os06g06510	LOC_Os09g32620	10.0128063
LOC_Os06g06560	LOC_Os08g33370	9.03416811
LOC_Os06g06460	LOC_Os06g36770	9.2801602
LOC_Os06g06980	LOC_Os11g10510	10.3540597
LOC_Os06g06320	LOC_Os08g05910	10.0254324
LOC_Os06g06460	LOC_Os09g32620	10.0128063

InteractorA	InteractorB	Z score
LOC_Os06g06560	LOC_Os06g39230	12.0399168
LOC_Os06g06980	LOC_Os10g35110	9.20332845
LOC_Os06g06980	LOC_Os07g30970	9.16165131
LOC_Os06g06510	LOC_Os12g07980	10.6969586
LOC_Os06g07080	LOC_Os07g48010	10.0889738
LOC_Os06g06320	LOC_Os08g28820	9.25941023
LOC_Os06g06090	LOC_Os10g29620	11.1707147
LOC_Os06g06560	LOC_Os12g02060	9.19690253
LOC_Os06g06090	LOC_Os10g02040	8.96232678
LOC_Os06g06300	LOC_Os12g12514	9.69623173
LOC_Os06g06460	LOC_Os12g07980	10.6969586
LOC_Os06g06090	LOC_Os10g27174	8.9808119
LOC_Os06g07140	LOC_Os08g41880	8.83029276
LOC_Os06g07080	LOC_Os09g27820	9.5092036
LOC_Os06g06560	LOC_Os12g41110	8.80321853
LOC_Os06g06730	LOC_Os06g23780	9.27096473
LOC_Os06g06730	LOC_Os08g33710	8.93083714
LOC_Os06g06460	LOC_Os07g44550	10.5558631
LOC_Os06g06510	LOC_Os12g22680	9.68890602
LOC_Os06g07080	LOC_Os06g37500	8.96365729
LOC_Os06g06560	LOC_Os09g23560	10.2445845
LOC_Os06g07080	LOC_Os06g08080	9.29236171
LOC_Os06g06880	LOC_Os07g46280	9.14944604
LOC_Os06g06730	LOC_Os07g06410	8.8762914
LOC_Os06g07140	LOC_Os08g16910	10.4018065
LOC_Os06g06090	LOC_Os09g32830	13.2980599
LOC_Os06g06510	LOC_Os12g10730	9.13435634
LOC_Os06g07080	LOC_Os06g40170	8.81152434
LOC_Os06g06510	LOC_Os10g38350	9.2380179
LOC_Os06g06460	LOC_Os06g06510	11.0654287
LOC_Os06g06560	LOC_Os10g35070	8.98566779
LOC_Os06g06510	LOC_Os10g38160	8.95621906
LOC_Os06g06730	LOC_Os09g16910	8.82800914
LOC_Os06g07080	LOC_Os10g40730	9.17906594
LOC_Os06g06510	LOC_Os10g07616	9.5223222
LOC_Os06g06560	LOC_Os11g02600	9.08003603
LOC_Os06g06980	LOC_Os10g09860	10.2791202
LOC_Os06g06460	LOC_Os09g12590	10.6579221
LOC_Os06g06100	LOC_Os10g07229	9.55019567
LOC_Os06g06460	LOC_Os10g38350	9.2380179
LOC_Os06g07140	LOC_Os10g34520	9.56173014
LOC_Os06g06730	LOC_Os07g42950	8.82821686
LOC_Os06g06300	LOC_Os12g43490	9.39099914

InteractorA	InteractorB	Z score
LOC_Os06g06510	LOC_Os11g05730	11.0654287
LOC_Os06g06320	LOC_Os07g41750	10.0259247
LOC_Os06g06090	LOC_Os10g37060	9.56825117
LOC_Os06g06730	LOC_Os07g43170	8.90319202
LOC_Os06g06300	LOC_Os11g38959	9.92712077
LOC_Os06g06320	LOC_Os10g27050	10.4694763
LOC_Os06g06320	LOC_Os06g46000	9.37767581
LOC_Os06g06560	LOC_Os11g37550	9.26487943
LOC_Os06g06980	LOC_Os07g22650	9.3064977
LOC_Os06g06100	LOC_Os06g49970	8.85627952
LOC_Os06g06980	LOC_Os07g49120	9.91776639
LOC_Os06g06460	LOC_Os11g05730	11.0654305
LOC_Os01g22249	LOC_Os04g12960	11.1386717
LOC_Os01g22249	LOC_Os04g33970	9.80180557
LOC_Os01g22010	LOC_Os07g44260	9.13829357
LOC_Os01g22336	LOC_Os05g48980	9.22491645
LOC_Os01g22249	LOC_Os08g40140	8.86217957
LOC_Os01g22010	LOC_Os02g56460	8.84678942
LOC_Os01g22249	LOC_Os02g38200	9.98013312
LOC_Os01g22010	LOC_Os02g49720	9.25794571
LOC_Os01g21970	LOC_Os04g40130	9.78958742
LOC_Os01g22336	LOC_Os01g57956	9.03713513
LOC_Os01g22336	LOC_Os05g04690	9.07265268
LOC_Os01g22230	LOC_Os09g32800	9.69116596
LOC_Os01g22249	LOC_Os05g35400	8.97612533
LOC_Os01g22336	LOC_Os11g25260	9.05640415
LOC_Os01g22336	LOC_Os02g50860	10.1440892
LOC_Os01g22249	LOC_Os07g48160	9.05138146
LOC_Os01g22230	LOC_Os05g45420	9.24617622
LOC_Os01g21970	LOC_Os06g46940	10.4042641
LOC_Os01g21970	LOC_Os07g31770	9.96546735
LOC_Os01g22010	LOC_Os02g24354	9.94151869
LOC_Os01g21970	LOC_Os03g59740	8.80567589
LOC_Os01g22230	LOC_Os03g18130	9.18899125
LOC_Os01g22249	LOC_Os09g34214	9.62695099
LOC_Os01g22010	LOC_Os07g28480	9.05994679
LOC_Os01g21970	LOC_Os05g27950	9.06644621
LOC_Os01g22010	LOC_Os06g50300	8.94559803
LOC_Os01g22230	LOC_Os06g36770	9.94639969
LOC_Os01g22249	LOC_Os11g47760	9.01496256
LOC_Os01g21970	LOC_Os10g21326	9.31894952
LOC_Os01g22352	LOC_Os02g07720	9.01141031
LOC_Os01g22249	LOC_Os12g10730	9.258541

InteractorA	InteractorB	Z score
LOC_Os01g21970	LOC_Os09g32620	9.15334381
LOC_Os01g22230	LOC_Os02g22780	9.87850859
LOC_Os01g22230	LOC_Os05g07720	9.6508178
LOC_Os01g22336	LOC_Os04g40990	9.95499572
LOC_Os01g22010	LOC_Os02g47590	8.8825231
LOC_Os01g22336	LOC_Os08g15276	9.03713513
LOC_Os01g22249	LOC_Os08g33710	8.87298536
LOC_Os01g21970	LOC_Os05g04340	10.1899994
LOC_Os01g22230	LOC_Os07g05180	9.00132328
LOC_Os01g22230	LOC_Os09g39380	8.87990239
LOC_Os01g22249	LOC_Os09g11230	9.02305534
LOC_Os01g22230	LOC_Os03g45960	8.80106133
LOC_Os01g22249	LOC_Os05g43820	9.17497118
LOC_Os01g22230	LOC_Os01g48280	9.53557274
LOC_Os01g22249	LOC_Os03g03910	9.59570532
LOC_Os01g22336	LOC_Os03g57290	9.316799
LOC_Os01g22249	LOC_Os06g35940	9.01771697
LOC_Os01g22230	LOC_Os09g31502	8.8859736
LOC_Os01g22230	LOC_Os07g29750	9.50587401
LOC_Os01g22230	LOC_Os12g04980	9.1755317
LOC_Os01g22336	LOC_Os09g34214	9.00875054
LOC_Os01g22336	LOC_Os03g20710	9.54180436
LOC_Os01g22249	LOC_Os08g35740	9.01991054
LOC_Os01g22010	LOC_Os04g16680	9.24354386
LOC_Os01g22336	LOC_Os05g46270	9.30522334
LOC_Os01g22336	LOC_Os07g14590	9.13158542
LOC_Os01g22336	LOC_Os11g14040	9.94096462
LOC_Os01g22010	LOC_Os04g27860	9.4570003
LOC_Os01g22230	LOC_Os06g24390	9.31893887
LOC_Os01g22230	LOC_Os02g03610	9.7132202
LOC_Os01g22336	LOC_Os12g44020	10.0006644
LOC_Os01g22249	LOC_Os04g59600	9.20795208
LOC_Os01g22249	LOC_Os02g40830	8.82904854
LOC_Os01g22336	LOC_Os02g49720	9.81926107
LOC_Os01g22230	LOC_Os08g08070	9.27865165
LOC_Os01g22336	LOC_Os12g43630	9.58439445
LOC_Os01g22336	LOC_Os06g40170	8.87601539
LOC_Os01g22352	LOC_Os02g08420	9.02527609
LOC_Os01g22010	LOC_Os11g40140	9.02978337
LOC_Os01g22230	LOC_Os02g35310	8.8285275
LOC_Os01g22230	LOC_Os02g12580	8.97209541
LOC_Os01g22336	LOC_Os04g16828	9.03713513
LOC_Os01g22230	LOC_Os12g22680	8.88410514

InteractorA	InteractorB	Z score
LOC_Os01g22230	LOC_Os01g55540	10.4030272
LOC_Os01g22336	LOC_Os02g47600	11.1443473
LOC_Os01g22249	LOC_Os04g55850	9.16043032
LOC_Os01g22010	LOC_Os08g44340	8.8454526
LOC_Os01g22230	LOC_Os02g08100	8.94076598
LOC_Os01g22010	LOC_Os02g38210	9.01485787
LOC_Os01g22336	LOC_Os03g64210	9.50626494
LOC_Os01g22230	LOC_Os02g51100	9.3826323
LOC_Os01g21970	LOC_Os10g41550	8.85015794
LOC_Os01g22010	LOC_Os02g10070	9.77384468
LOC_Os01g21970	LOC_Os04g16844	9.31895051
LOC_Os01g22336	LOC_Os09g30412	11.7405355
LOC_Os01g22336	LOC_Os05g22722	9.03713513
LOC_Os01g21970	LOC_Os07g22950	8.98082747
LOC_Os01g22230	LOC_Os03g49600	9.76367673
LOC_Os01g21970	LOC_Os11g05570	10.2491123
LOC_Os01g22336	LOC_Os03g38000	9.77961848
LOC_Os01g22249	LOC_Os10g39170	8.8333844
LOC_Os01g22336	LOC_Os10g21342	9.03713513
LOC_Os01g22336	LOC_Os01g23620	9.94753119
LOC_Os06g08080	LOC_Os09g20820	9.63819652
LOC_Os06g08080	LOC_Os10g27190	9.15399541
LOC_Os06g07210	LOC_Os08g34170	9.23920536
LOC_Os06g09450	LOC_Os06g23780	9.29268784
LOC_Os06g07878	LOC_Os11g32260	9.14097457
LOC_Os06g08770	LOC_Os07g26690	9.69467536
LOC_Os06g07978	LOC_Os08g40930	9.53169629
LOC_Os06g08080	LOC_Os06g43660	10.0235068
LOC_Os06g07978	LOC_Os12g16240	9.74043588
LOC_Os06g08770	LOC_Os12g40510	9.18855556
LOC_Os06g07878	LOC_Os08g40930	9.53169629
LOC_Os06g09910	LOC_Os08g14760	8.88753699
LOC_Os06g07978	LOC_Os11g05880	9.53717686
LOC_Os06g10330	LOC_Os07g28480	8.84593685
LOC_Os06g08770	LOC_Os10g13800	9.07617831
LOC_Os06g09450	LOC_Os07g01760	9.69438944
LOC_Os06g07878	LOC_Os12g18880	10.8887149
LOC_Os06g07960	LOC_Os11g08470	8.88586638
LOC_Os06g07580	LOC_Os06g49470	8.80687914
LOC_Os06g09910	LOC_Os10g40090	9.48730421
LOC_Os06g07580	LOC_Os09g08072	9.24315614
LOC_Os06g08770	LOC_Os06g39230	9.59680249
LOC_Os06g07210	LOC_Os11g25260	10.5571835

InteractorA	InteractorB	Z score
LOC_Os06g07878	LOC_Os11g25260	9.50097075
LOC_Os06g09450	LOC_Os07g49120	9.44215025
LOC_Os06g08600	LOC_Os08g02410	8.8662757
LOC_Os06g09450	LOC_Os07g42490	9.64501617
LOC_Os06g07978	LOC_Os08g04560	9.27106869
LOC_Os06g09450	LOC_Os08g44350	12.2228775
LOC_Os06g08600	LOC_Os09g23530	8.97032887
LOC_Os06g08770	LOC_Os06g24390	8.80343777
LOC_Os06g07580	LOC_Os12g05410	8.81617052
LOC_Os06g08600	LOC_Os08g34170	9.10693737
LOC_Os06g07878	LOC_Os11g05880	9.53715597
LOC_Os06g08080	LOC_Os07g07770	8.80839743
LOC_Os06g07878	LOC_Os08g44810	8.81930853
LOC_Os06g09450	LOC_Os12g12514	8.94151124
LOC_Os06g08310	LOC_Os10g01540	9.94850543
LOC_Os06g09450	LOC_Os10g30580	9.34281107
LOC_Os06g07878	LOC_Os08g04560	9.27106869
LOC_Os06g08080	LOC_Os08g35740	10.4731346
LOC_Os06g08600	LOC_Os10g32550	10.2995953
LOC_Os06g07210	LOC_Os07g48020	11.344746
LOC_Os06g07210	LOC_Os08g44340	9.38214976
LOC_Os06g09450	LOC_Os10g39840	9.243404
LOC_Os06g10330	LOC_Os07g46830	9.11588695
LOC_Os06g08770	LOC_Os08g32870	8.8969334
LOC_Os06g09450	LOC_Os07g13980	8.95650518
LOC_Os06g08770	LOC_Os11g43960	9.95397531
LOC_Os06g07210	LOC_Os07g46280	10.4815986
LOC_Os06g08600	LOC_Os07g07550	9.27396868
LOC_Os06g07210	LOC_Os06g21980	10.3203029
LOC_Os06g08310	LOC_Os10g30580	9.06492645
LOC_Os06g08770	LOC_Os07g07550	9.43693675
LOC_Os06g09450	LOC_Os09g39810	8.88812526
LOC_Os06g07210	LOC_Os12g31640	9.20432262
LOC_Os06g07978	LOC_Os12g18880	10.8887149
LOC_Os06g07210	LOC_Os08g44530	8.97668391
LOC_Os06g07978	LOC_Os08g44810	8.81930853
LOC_Os06g07978	LOC_Os11g25260	9.50096045
LOC_Os06g09450	LOC_Os10g21240	9.04273315
LOC_Os06g07210	LOC_Os07g38860	9.5338287
LOC_Os06g07210	LOC_Os08g36320	9.24992594
LOC_Os06g08770	LOC_Os09g11230	8.82612955
LOC_Os06g08080	LOC_Os11g32650	9.55117811
LOC_Os06g09450	LOC_Os09g23540	11.1686485

InteractorA	InteractorB	Z score
LOC_Os06g09450	LOC_Os07g48780	8.99844308
LOC_Os06g07978	LOC_Os11g32260	9.14097457
LOC_Os06g07580	LOC_Os09g32840	9.4259021
LOC_Os06g07580	LOC_Os09g28420	8.86385934
LOC_Os06g07210	LOC_Os08g37490	9.41194725
LOC_Os06g08770	LOC_Os06g10330	10.1698885
LOC_Os06g07210	LOC_Os12g23630	8.89193507
LOC_Os06g09910	LOC_Os10g21326	10.0710108
LOC_Os06g08770	LOC_Os09g10300	9.48810961
LOC_Os06g07960	LOC_Os10g25674	8.81609277
LOC_Os06g07878	LOC_Os12g16240	9.74043959
LOC_Os06g08310	LOC_Os12g40550	8.95475924
LOC_Os06g08080	LOC_Os07g07550	9.85076817
LOC_Os06g08770	LOC_Os12g06620	9.45766359
LOC_Os06g09450	LOC_Os11g10510	8.89299829
LOC_Os06g08770	LOC_Os12g31640	10.1011905
LOC_Os06g07210	LOC_Os12g08280	9.99784643
LOC_Os06g08080	LOC_Os10g41510	9.68745912
LOC_Os06g07140	LOC_Os12g14070	9.46697964
LOC_Os06g09450	LOC_Os12g12470	9.21660658
LOC_Os06g09910	LOC_Os06g39140	9.55542292
LOC_Os06g08310	LOC_Os11g08120	8.84374743
LOC_Os06g10950	LOC_Os06g35590	9.28022813
LOC_Os06g11240	LOC_Os07g46830	9.5156463
LOC_Os06g11290	LOC_Os10g38229	9.90701693
LOC_Os06g11280	LOC_Os10g39170	8.8946349
LOC_Os06g11210	LOC_Os06g12180	13.8824393
LOC_Os06g11200	LOC_Os08g37800	9.94608915
LOC_Os06g10330	LOC_Os10g38740	9.39906238
LOC_Os06g11280	LOC_Os11g42350	9.86271139
LOC_Os06g10910	LOC_Os10g03540	8.98820558
LOC_Os06g11210	LOC_Os11g34450	9.3706054
LOC_Os06g11240	LOC_Os08g38920	8.93172625
LOC_Os06g10950	LOC_Os06g36670	8.94521812
LOC_Os06g10330	LOC_Os08g40140	8.81120081
LOC_Os06g11290	LOC_Os06g44080	9.93988328
LOC_Os06g10930	LOC_Os11g43360	8.88252604
LOC_Os06g11210	LOC_Os07g48060	9.01020459
LOC_Os06g10930	LOC_Os08g44340	9.1699045
LOC_Os06g10340	LOC_Os11g03980	9.38158835
LOC_Os06g11290	LOC_Os11g08460	8.82435873
LOC_Os06g10910	LOC_Os06g50300	9.98510828
LOC_Os06g11210	LOC_Os10g41689	10.8548141

InteractorA	InteractorB	Z score
LOC_Os06g11240	LOC_Os06g30970	8.81468845
LOC_Os06g11260	LOC_Os08g09940	9.23294891
LOC_Os06g10970	LOC_Os08g23730	9.28555453
LOC_Os06g10340	LOC_Os07g46460	8.89413352
LOC_Os06g11210	LOC_Os11g26850	8.87788859
LOC_Os06g10970	LOC_Os12g34062	9.40201913
LOC_Os06g11240	LOC_Os12g08280	8.81765021
LOC_Os06g10970	LOC_Os06g45100	10.7640213
LOC_Os06g10340	LOC_Os07g29750	9.01866059
LOC_Os06g11240	LOC_Os10g11140	9.71741117
LOC_Os06g11280	LOC_Os06g36700	8.8859211
LOC_Os06g11240	LOC_Os06g36700	9.74848289
LOC_Os06g11240	LOC_Os08g20730	10.4962703
LOC_Os06g11290	LOC_Os07g02340	9.91383517
LOC_Os06g10910	LOC_Os11g47550	9.03382453
LOC_Os06g11200	LOC_Os07g10720	9.72051597
LOC_Os06g11290	LOC_Os11g08440	8.95387282
LOC_Os06g11290	LOC_Os09g11230	9.13057632
LOC_Os06g11210	LOC_Os09g39380	9.03973091
LOC_Os06g11280	LOC_Os09g36450	9.33333636
LOC_Os06g11290	LOC_Os06g24390	8.98329754
LOC_Os06g11240	LOC_Os07g06410	8.87943837
LOC_Os06g11290	LOC_Os09g25320	9.93988328
LOC_Os06g11210	LOC_Os08g33370	9.31214562
LOC_Os06g11280	LOC_Os08g35740	9.32291517
LOC_Os06g10930	LOC_Os12g33610	10.1306933
LOC_Os06g10970	LOC_Os06g21980	9.49709347
LOC_Os06g10950	LOC_Os06g37150	9.64263395
LOC_Os06g11280	LOC_Os07g34140	8.89211234
LOC_Os06g11290	LOC_Os07g05400	9.5300244
LOC_Os06g11210	LOC_Os11g31620	8.88039275
LOC_Os06g10950	LOC_Os10g13800	9.38881562
LOC_Os06g11240	LOC_Os09g24990	8.98723624
LOC_Os06g10910	LOC_Os07g03368	8.97613608
LOC_Os06g11240	LOC_Os12g13390	8.89151367
LOC_Os06g10950	LOC_Os08g02700	8.82208978
LOC_Os06g11210	LOC_Os07g49400	8.81410399
LOC_Os06g11260	LOC_Os12g16200	9.31538433
LOC_Os06g11260	LOC_Os07g41750	11.4411756
LOC_Os06g11240	LOC_Os10g32870	9.89141832
LOC_Os06g10950	LOC_Os10g38740	9.0973233
LOC_Os06g11240	LOC_Os07g43390	9.5828958
LOC_Os06g11290	LOC_Os12g23630	9.42739844

InteractorA	InteractorB	Z score
LOC_Os06g11210	LOC_Os06g35660	9.45441822
LOC_Os06g10340	LOC_Os10g21266	8.86758108
LOC_Os06g11240	LOC_Os11g25100	9.5710786
LOC_Os06g11210	LOC_Os09g17620	8.9519062
LOC_Os06g11280	LOC_Os12g10560	9.55692032
LOC_Os06g11290	LOC_Os07g36190	9.16128335
LOC_Os06g11210	LOC_Os08g40140	9.82948573
LOC_Os06g10910	LOC_Os07g42960	11.5317921
LOC_Os06g11210	LOC_Os11g25220	9.24624954
LOC_Os06g10930	LOC_Os07g47290	8.95611507
LOC_Os06g10950	LOC_Os10g05069	9.4230764
LOC_Os06g11200	LOC_Os09g34960	10.4624849
LOC_Os06g11210	LOC_Os06g35480	8.90737152
LOC_Os06g11240	LOC_Os08g06550	10.0379575
LOC_Os06g10950	LOC_Os11g40150	9.24306433
LOC_Os06g11240	LOC_Os11g03230	9.13381439
LOC_Os06g11200	LOC_Os09g20284	9.06044765
LOC_Os06g10340	LOC_Os09g04050	9.47463267
LOC_Os06g11210	LOC_Os07g44620	9.06942241
LOC_Os06g11240	LOC_Os10g39840	11.6232782
LOC_Os06g10330	LOC_Os09g31490	9.61594296
LOC_Os06g10970	LOC_Os08g28190	8.94901651
LOC_Os06g11280	LOC_Os06g30370	8.89490641
LOC_Os06g11280	LOC_Os11g26860	9.75136155
LOC_Os06g10970	LOC_Os10g38580	8.99869813
LOC_Os06g11260	LOC_Os12g02980	9.43197581
LOC_Os06g11210	LOC_Os07g42490	9.55238759
LOC_Os06g11280	LOC_Os08g09200	8.89753163
LOC_Os06g11240	LOC_Os11g40150	10.2375126
LOC_Os06g11280	LOC_Os08g43560	9.14719956
LOC_Os06g10930	LOC_Os11g08445	9.72433722
LOC_Os06g11240	LOC_Os06g37660	8.94371542
LOC_Os06g11290	LOC_Os06g19960	11.3623993
LOC_Os06g11200	LOC_Os06g35630	8.83476745
LOC_Os06g11210	LOC_Os08g17500	9.58893824
LOC_Os06g11210	LOC_Os10g25674	10.1455115
LOC_Os06g11240	LOC_Os11g24560	8.92965108
LOC_Os06g11260	LOC_Os08g06610	8.94384718
LOC_Os06g11210	LOC_Os10g08550	8.87327137
LOC_Os06g11280	LOC_Os09g27750	10.3196287
LOC_Os06g11260	LOC_Os12g22650	9.0310941
LOC_Os06g11290	LOC_Os10g26390	9.2045825
LOC_Os06g10340	LOC_Os11g25220	8.90328461

InteractorA	InteractorB	Z score
LOC_Os06g11290	LOC_Os07g14590	8.90131263
LOC_Os06g11240	LOC_Os11g41130	9.13461682
LOC_Os06g11260	LOC_Os09g23530	9.26544401
LOC_Os06g11210	LOC_Os08g15030	9.37008083
LOC_Os06g10930	LOC_Os08g04540	8.94144583
LOC_Os06g10970	LOC_Os10g38660	10.4465652
LOC_Os06g11260	LOC_Os07g05400	9.21646588
LOC_Os06g11280	LOC_Os08g32620	10.8490174
LOC_Os06g11240	LOC_Os06g35630	8.95574289
LOC_Os06g10930	LOC_Os08g32850	9.45020761
LOC_Os06g11210	LOC_Os09g08072	9.20957144
LOC_Os06g11280	LOC_Os06g49470	10.9776102
LOC_Os06g12790	LOC_Os07g09340	9.33530136
LOC_Os06g11800	LOC_Os08g16910	9.01128858
LOC_Os06g15990	LOC_Os10g25140	10.1549402
LOC_Os06g15990	LOC_Os08g39860	8.8829669
LOC_Os06g12180	LOC_Os12g12580	9.34451088
LOC_Os06g12180	LOC_Os06g35650	9.03584503
LOC_Os06g12990	LOC_Os06g51150	9.10112125
LOC_Os06g19960	LOC_Os08g44520	10.416925
LOC_Os06g15990	LOC_Os10g22310	9.66112664
LOC_Os06g14510	LOC_Os07g35880	8.95079323
LOC_Os06g13450	LOC_Os07g30970	8.84238154
LOC_Os06g14510	LOC_Os09g10300	10.2806114
LOC_Os06g12090	LOC_Os07g05150	9.33587541
LOC_Os06g19960	LOC_Os08g02700	9.92948016
LOC_Os06g15990	LOC_Os12g13030	8.87358507
LOC_Os06g19960	LOC_Os09g37949	9.23884037
LOC_Os06g15990	LOC_Os12g37360	8.81016235
LOC_Os06g11800	LOC_Os07g40290	8.81949738
LOC_Os06g14510	LOC_Os09g32810	10.5112254
LOC_Os06g14510	LOC_Os08g02410	9.53719864
LOC_Os06g19960	LOC_Os10g38740	9.66766371
LOC_Os06g12090	LOC_Os08g34790	9.82992839
LOC_Os06g11800	LOC_Os10g35110	9.14875348
LOC_Os06g12180	LOC_Os07g26540	8.9142513
LOC_Os06g14620	LOC_Os08g35440	8.96137506
LOC_Os06g12790	LOC_Os09g20820	9.27033603
LOC_Os06g14510	LOC_Os06g23780	9.84732187
LOC_Os06g15990	LOC_Os11g33240	8.86414001
LOC_Os06g15420	LOC_Os10g03540	8.86007533
LOC_Os06g12090	LOC_Os08g14760	8.94095716
LOC_Os06g12090	LOC_Os06g37150	9.33004424

InteractorA	InteractorB	Z score
LOC_Os06g19960	LOC_Os06g43640	9.10481783
LOC_Os06g15420	LOC_Os07g30200	9.16359
LOC_Os06g12990	LOC_Os07g48880	8.93966265
LOC_Os06g14510	LOC_Os12g07820	10.2587146
LOC_Os06g19960	LOC_Os10g38670	9.46383271
LOC_Os06g21570	LOC_Os07g44550	9.46436052
LOC_Os06g14510	LOC_Os12g18900	8.81312329
LOC_Os06g12090	LOC_Os08g32620	9.31100908
LOC_Os06g12990	LOC_Os11g03980	12.5901108
LOC_Os06g19960	LOC_Os08g06550	8.81318461
LOC_Os06g21570	LOC_Os09g28460	9.49510116
LOC_Os06g12990	LOC_Os12g38770	9.27060108
LOC_Os06g12790	LOC_Os09g36830	9.38719519
LOC_Os06g15420	LOC_Os10g32680	9.0708919
LOC_Os06g12090	LOC_Os11g26850	9.50386233
LOC_Os06g15420	LOC_Os07g31270	9.39950631
LOC_Os06g15990	LOC_Os12g16220	8.97406362
LOC_Os06g19960	LOC_Os11g40150	9.42688872
LOC_Os06g15420	LOC_Os09g38620	9.76541804
LOC_Os06g21570	LOC_Os06g46284	9.73160303
LOC_Os06g19960	LOC_Os10g41550	9.59667146
LOC_Os06g12990	LOC_Os11g04880	9.05704744
LOC_Os06g21570	LOC_Os09g19560	12.9874818
LOC_Os06g12990	LOC_Os09g08120	9.43056998
LOC_Os06g12090	LOC_Os10g21268	9.2253715
LOC_Os06g12990	LOC_Os07g05180	9.24275343
LOC_Os06g13450	LOC_Os10g38229	10.5436125
LOC_Os06g15990	LOC_Os09g33850	9.94488251
LOC_Os06g21570	LOC_Os08g34290	9.6126423
LOC_Os06g15420	LOC_Os07g07550	9.38552818
LOC_Os06g15420	LOC_Os08g09770	9.14736017
LOC_Os06g21570	LOC_Os06g27770	9.2706871
LOC_Os06g15420	LOC_Os08g05910	8.80998258
LOC_Os06g12090	LOC_Os08g20270	9.49752138
LOC_Os06g14510	LOC_Os10g21212	8.89793578
LOC_Os06g11800	LOC_Os06g40940	8.90861614
LOC_Os06g12990	LOC_Os10g28360	9.84697057
LOC_Os06g12790	LOC_Os08g16910	9.33042291
LOC_Os06g15990	LOC_Os10g33800	8.80601574
LOC_Os06g14510	LOC_Os09g04730	9.34527039
LOC_Os06g21570	LOC_Os07g28480	11.203564
LOC_Os06g15420	LOC_Os10g38360	9.41207105
LOC_Os06g12790	LOC_Os12g44000	8.94329009

InteractorA	InteractorB	Z score
LOC_Os06g12790	LOC_Os12g22680	8.92349574
LOC_Os06g15990	LOC_Os10g08580	9.18868566
LOC_Os06g15420	LOC_Os11g25100	8.91997694
LOC_Os06g12090	LOC_Os07g39290	8.96994573
LOC_Os06g13450	LOC_Os11g47600	9.18688841
LOC_Os06g11800	LOC_Os07g46460	8.88340017
LOC_Os06g12990	LOC_Os11g14910	8.91127363
LOC_Os06g12990	LOC_Os07g42960	9.81005246
LOC_Os06g11800	LOC_Os07g29440	9.83446501
LOC_Os06g12090	LOC_Os06g12790	9.81850285
LOC_Os06g12990	LOC_Os07g02350	8.91932744
LOC_Os06g12990	LOC_Os08g44960	9.43512296
LOC_Os06g14620	LOC_Os07g34190	9.16791188
LOC_Os06g12990	LOC_Os06g40190	9.91121085
LOC_Os06g15420	LOC_Os09g36930	8.82739037
LOC_Os06g19960	LOC_Os07g47420	9.85150143
LOC_Os06g19960	LOC_Os12g25630	10.1075596
LOC_Os06g12990	LOC_Os10g31940	9.88412725
LOC_Os06g12790	LOC_Os09g39380	9.28458366
LOC_Os06g14620	LOC_Os06g35650	9.47580069
LOC_Os06g15990	LOC_Os11g04880	8.81896352
LOC_Os06g15420	LOC_Os07g48160	8.886921
LOC_Os06g12180	LOC_Os07g07320	9.25116826
LOC_Os06g11800	LOC_Os10g37060	9.39313753
LOC_Os06g15420	LOC_Os12g44350	9.25125049
LOC_Os06g12790	LOC_Os07g05160	9.857009
LOC_Os06g19960	LOC_Os08g28800	10.0355574
LOC_Os06g12180	LOC_Os07g07709	8.88134079
LOC_Os06g12090	LOC_Os07g48880	8.91757224
LOC_Os06g15990	LOC_Os10g17680	8.88609579
LOC_Os06g14510	LOC_Os11g02600	9.31232608
LOC_Os06g21570	LOC_Os07g05160	9.26885119
LOC_Os06g15990	LOC_Os10g17660	8.88611183
LOC_Os06g14510	LOC_Os12g12470	9.04708691
LOC_Os06g15420	LOC_Os09g34970	9.1940678
LOC_Os06g12990	LOC_Os08g42000	9.90015151
LOC_Os06g27770	LOC_Os06g45710	11.0326427
LOC_Os06g23440	LOC_Os10g02040	9.04316323
LOC_Os06g27770	LOC_Os06g29220	8.91489143
LOC_Os06g22960	LOC_Os07g09890	8.86506473
LOC_Os06g23780	LOC_Os10g21240	9.19384569
LOC_Os06g23760	LOC_Os08g35420	9.81946663
LOC_Os06g24390	LOC_Os07g38030	8.92171025

InteractorA	InteractorB	Z score
LOC_Os06g23780	LOC_Os10g42280	9.6952346
LOC_Os06g23780	LOC_Os10g41480	9.41371021
LOC_Os06g24390	LOC_Os07g22600	8.85995664
LOC_Os06g22140	LOC_Os10g29470	8.94287669
LOC_Os06g23870	LOC_Os07g05400	9.59711883
LOC_Os06g23440	LOC_Os06g40180	9.27841911
LOC_Os06g24390	LOC_Os06g41810	9.33432859
LOC_Os06g22140	LOC_Os08g32620	9.78290789
LOC_Os06g27770	LOC_Os11g31530	8.95733206
LOC_Os06g23440	LOC_Os06g29220	8.93361887
LOC_Os06g23780	LOC_Os11g40140	9.19813621
LOC_Os06g23780	LOC_Os09g28420	9.58415449
LOC_Os06g23780	LOC_Os09g39570	8.84421694
LOC_Os06g23780	LOC_Os12g05410	8.89083615
LOC_Os06g23870	LOC_Os09g27750	9.61570475
LOC_Os06g22140	LOC_Os11g48110	9.05673207
LOC_Os06g23440	LOC_Os06g46340	8.9064194
LOC_Os06g23760	LOC_Os08g09770	9.12667191
LOC_Os06g23780	LOC_Os07g42960	9.82466514
LOC_Os06g24990	LOC_Os09g28400	9.01719441
LOC_Os06g23870	LOC_Os08g43190	9.81378846
LOC_Os06g23870	LOC_Os07g48050	9.1765362
LOC_Os06g27770	LOC_Os08g39300	10.1039285
LOC_Os06g21570	LOC_Os12g23630	8.99105901
LOC_Os06g23760	LOC_Os10g23900	9.68543434
LOC_Os06g21980	LOC_Os11g26860	9.69684464
LOC_Os06g21570	LOC_Os12g10600	9.19674996
LOC_Os06g23440	LOC_Os06g27770	9.16914093
LOC_Os06g23870	LOC_Os11g02130	8.99910027
LOC_Os06g21980	LOC_Os10g38640	9.18732129
LOC_Os06g24990	LOC_Os07g02210	9.85400751
LOC_Os06g21820	LOC_Os07g08500	9.16992498
LOC_Os06g27770	LOC_Os06g36670	8.93402082
LOC_Os06g22960	LOC_Os10g38600	9.39031827
LOC_Os06g23780	LOC_Os08g02410	8.96940136
LOC_Os06g22140	LOC_Os09g31486	9.94170772
LOC_Os06g24390	LOC_Os06g37610	9.60792294
LOC_Os06g23440	LOC_Os06g46372	9.4062115
LOC_Os06g27770	LOC_Os08g14570	9.58309691
LOC_Os06g27770	LOC_Os06g29844	10.0741439
LOC_Os06g21980	LOC_Os07g39870	9.2606429
LOC_Os06g27770	LOC_Os07g44740	11.8804807
LOC_Os06g25010	LOC_Os09g09520	8.90937017

InteractorA	InteractorB	Z score
LOC_Os06g27770	LOC_Os09g39810	9.0879335
LOC_Os06g23440	LOC_Os06g46284	9.14314074
LOC_Os06g23780	LOC_Os11g31620	9.41654173
LOC_Os06g25010	LOC_Os10g39120	9.56650931
LOC_Os06g27770	LOC_Os08g03290	8.86545544
LOC_Os06g23760	LOC_Os07g40580	8.91318523
LOC_Os06g23870	LOC_Os10g34520	9.20360511
LOC_Os06g21820	LOC_Os08g35740	8.81646277
LOC_Os06g23780	LOC_Os07g46310	8.8623192
LOC_Os06g23780	LOC_Os07g48030	9.03617105
LOC_Os06g25010	LOC_Os08g29520	8.80968863
LOC_Os06g23780	LOC_Os11g05570	9.28479356
LOC_Os06g23440	LOC_Os11g40140	9.532746
LOC_Os06g25010	LOC_Os07g06440	10.0172299
LOC_Os06g23440	LOC_Os10g27050	8.85544411
LOC_Os06g21980	LOC_Os06g35530	9.50515346
LOC_Os06g23440	LOC_Os07g40290	9.88617669
LOC_Os06g25010	LOC_Os10g25130	9.14084634
LOC_Os06g23440	LOC_Os10g30840	10.0989896
LOC_Os06g23780	LOC_Os06g43640	9.03237271
LOC_Os06g23760	LOC_Os11g16590	8.89753098
LOC_Os06g25010	LOC_Os10g23100	11.6265438
LOC_Os06g22140	LOC_Os06g25010	8.97193419
LOC_Os06g22140	LOC_Os10g21326	8.84040918
LOC_Os06g23780	LOC_Os12g16240	11.0393936
LOC_Os06g27770	LOC_Os06g51084	9.2369563
LOC_Os06g21820	LOC_Os08g44530	8.85556125
LOC_Os06g22960	LOC_Os09g10270	8.86969156
LOC_Os06g29220	LOC_Os10g26390	8.88933616
LOC_Os06g35490	LOC_Os06g35540	9.0110843
LOC_Os06g34690	LOC_Os08g33100	8.88768159
LOC_Os06g35520	LOC_Os06g48160	8.88898912
LOC_Os06g33210	LOC_Os08g34210	8.92542159
LOC_Os06g27770	LOC_Os12g44150	9.87974305
LOC_Os06g28550	LOC_Os12g16200	9.02906771
LOC_Os06g35490	LOC_Os10g30840	10.6161675
LOC_Os06g35490	LOC_Os09g24412	9.44930416
LOC_Os06g29220	LOC_Os08g15292	9.83096781
LOC_Os06g35490	LOC_Os06g45670	9.33523843
LOC_Os06g28550	LOC_Os08g34170	8.85418219
LOC_Os06g33210	LOC_Os12g43100	8.92229858
LOC_Os06g35480	LOC_Os07g29750	9.73472203
LOC_Os06g35480	LOC_Os10g38730	9.35813094

InteractorA	InteractorB	Z score
LOC_Os06g35530	LOC_Os07g43170	9.08162171
LOC_Os06g29220	LOC_Os11g26850	9.51786146
LOC_Os06g34690	LOC_Os11g32620	9.83042074
LOC_Os06g35480	LOC_Os09g10270	9.117967
LOC_Os06g35490	LOC_Os07g25024	9.44932709
LOC_Os06g33210	LOC_Os10g38580	9.06577856
LOC_Os06g34690	LOC_Os12g02060	9.9517505
LOC_Os06g30970	LOC_Os07g06410	8.98059012
LOC_Os06g35480	LOC_Os07g47420	10.3935689
LOC_Os06g28550	LOC_Os08g40170	9.02451958
LOC_Os06g34690	LOC_Os10g25930	9.23322059
LOC_Os06g35530	LOC_Os09g31506	9.12758039
LOC_Os06g35530	LOC_Os11g10480	10.4365939
LOC_Os06g35490	LOC_Os10g21352	9.44932709
LOC_Os06g29844	LOC_Os10g41490	8.99960431
LOC_Os06g35480	LOC_Os12g34874	9.58710872
LOC_Os06g28550	LOC_Os10g42280	8.83149349
LOC_Os06g35530	LOC_Os10g41480	9.75627027
LOC_Os06g34690	LOC_Os11g16590	9.29230366
LOC_Os06g35520	LOC_Os10g26390	8.93970516
LOC_Os06g29220	LOC_Os11g32510	9.1276398
LOC_Os06g28550	LOC_Os06g35630	9.21814824
LOC_Os06g35490	LOC_Os09g39500	10.4844628
LOC_Os06g30370	LOC_Os09g17620	9.23033677
LOC_Os06g35490	LOC_Os07g31830	9.8662098
LOC_Os06g35480	LOC_Os08g25570	10.4882301
LOC_Os06g34690	LOC_Os07g37550	10.0910156
LOC_Os06g28550	LOC_Os07g31770	9.40083375
LOC_Os06g34690	LOC_Os12g16410	10.6799788
LOC_Os06g28550	LOC_Os07g07320	9.51548219
LOC_Os06g28550	LOC_Os10g07229	9.14433737
LOC_Os06g29220	LOC_Os09g36710	8.94389857
LOC_Os06g30370	LOC_Os08g09770	9.08972267
LOC_Os06g35520	LOC_Os06g35560	8.95016806
LOC_Os06g34690	LOC_Os10g39590	9.66860513
LOC_Os06g28550	LOC_Os08g44350	8.9725822
LOC_Os06g30370	LOC_Os12g22650	10.0185395
LOC_Os06g30970	LOC_Os10g41410	9.32671285
LOC_Os06g35520	LOC_Os07g44590	8.90177382
LOC_Os06g29220	LOC_Os08g44210	9.18828625
LOC_Os06g29844	LOC_Os09g23530	8.89693832
LOC_Os06g30970	LOC_Os12g44010	9.50914563
LOC_Os06g35520	LOC_Os11g26860	10.0129168

InteractorA	InteractorB	Z score
LOC_Os06g29180	LOC_Os09g36710	10.0426925
LOC_Os06g28550	LOC_Os07g13980	9.53067239
LOC_Os06g35530	LOC_Os09g15790	9.08896427
LOC_Os06g34690	LOC_Os07g38860	9.32504891
LOC_Os06g28550	LOC_Os08g20270	10.0319249
LOC_Os06g35490	LOC_Os08g06060	10.4580908
LOC_Os06g29180	LOC_Os06g29220	12.8143492
LOC_Os06g29220	LOC_Os07g03690	9.01047146
LOC_Os06g35490	LOC_Os07g34580	9.78596855
LOC_Os06g35490	LOC_Os12g34874	8.95645637
LOC_Os06g28550	LOC_Os09g31502	9.08513084
LOC_Os06g30970	LOC_Os10g09860	9.8137846
LOC_Os06g35520	LOC_Os08g34170	9.11350551
LOC_Os06g34690	LOC_Os12g07720	9.01366371
LOC_Os06g29180	LOC_Os12g04924	8.91096822
LOC_Os06g29844	LOC_Os09g10230	9.64939265
LOC_Os06g28550	LOC_Os08g36320	10.2485231
LOC_Os06g35530	LOC_Os09g08720	9.50112106
LOC_Os06g34690	LOC_Os09g12230	10.1420781
LOC_Os06g30970	LOC_Os10g33800	8.99967637
LOC_Os06g35480	LOC_Os12g40510	11.5585883
LOC_Os06g35530	LOC_Os08g04540	10.1680723
LOC_Os06g30970	LOC_Os10g38140	9.81377273
LOC_Os06g30370	LOC_Os07g39870	9.27235589
LOC_Os06g35480	LOC_Os12g14070	9.5136473
LOC_Os06g28550	LOC_Os12g40830	9.35406917
LOC_Os06g27770	LOC_Os12g43440	11.2275234
LOC_Os06g35530	LOC_Os11g05290	9.41265251
LOC_Os06g30370	LOC_Os06g50300	8.84510555
LOC_Os06g27770	LOC_Os12g25690	8.83165369
LOC_Os06g29180	LOC_Os07g35940	9.16526934
LOC_Os06g29844	LOC_Os11g33240	10.6824917
LOC_Os06g34690	LOC_Os10g21250	9.48089221
LOC_Os06g35520	LOC_Os10g14150	9.93385967
LOC_Os06g30970	LOC_Os12g13320	9.18168288
LOC_Os06g35490	LOC_Os08g15266	9.44932709
LOC_Os06g30970	LOC_Os08g34170	9.44635924
LOC_Os06g35530	LOC_Os07g48880	8.84393576
LOC_Os06g30370	LOC_Os06g36840	10.4579515
LOC_Os06g28550	LOC_Os09g35800	9.39015271
LOC_Os06g35530	LOC_Os08g44370	9.08070773
LOC_Os06g35530	LOC_Os11g10510	10.7584522
LOC_Os06g35530	LOC_Os12g36950	9.07238508

InteractorA	InteractorB	Z score
LOC_Os06g27770	LOC_Os12g10730	9.41037803
LOC_Os06g28550	LOC_Os06g47320	8.9085607
LOC_Os06g35730	LOC_Os10g40700	10.6767768
LOC_Os06g35630	LOC_Os08g23730	8.81018781
LOC_Os06g35700	LOC_Os08g15040	8.95752295
LOC_Os06g35650	LOC_Os11g19800	10.9658024
LOC_Os06g35660	LOC_Os09g32640	9.19949625
LOC_Os06g35814	LOC_Os07g48880	9.42545106
LOC_Os06g35660	LOC_Os10g38740	9.38506902
LOC_Os06g35650	LOC_Os12g16240	10.6581512
LOC_Os06g35700	LOC_Os12g34450	9.41616557
LOC_Os06g35700	LOC_Os09g24530	9.64883152
LOC_Os06g35660	LOC_Os09g36450	10.3772398
LOC_Os06g35650	LOC_Os07g03710	9.28761904
LOC_Os06g35560	LOC_Os08g04540	10.6763726
LOC_Os06g36670	LOC_Os07g06410	8.89507471
LOC_Os06g36700	LOC_Os08g17784	9.10226494
LOC_Os06g35630	LOC_Os09g25320	11.6590233
LOC_Os06g36670	LOC_Os09g10300	9.03419825
LOC_Os06g35700	LOC_Os08g44810	9.57541082
LOC_Os06g35540	LOC_Os07g36190	8.88466669
LOC_Os06g35650	LOC_Os07g08170	9.92713166
LOC_Os06g35660	LOC_Os07g42950	9.2397533
LOC_Os06g35660	LOC_Os12g13390	8.80004735
LOC_Os06g35660	LOC_Os11g31530	9.21927206
LOC_Os06g35560	LOC_Os08g09250	9.12539458
LOC_Os06g35730	LOC_Os09g19560	8.88393765
LOC_Os06g36160	LOC_Os08g39420	9.052122
LOC_Os06g35660	LOC_Os06g45590	9.42460592
LOC_Os06g36160	LOC_Os09g23540	9.14835462
LOC_Os06g36670	LOC_Os09g36830	10.027827
LOC_Os06g35814	LOC_Os09g15320	8.83055198
LOC_Os06g35814	LOC_Os07g46460	9.49174925
LOC_Os06g35940	LOC_Os08g20270	9.47743002
LOC_Os06g35560	LOC_Os10g26050	8.88710022
LOC_Os06g35730	LOC_Os06g46000	9.27590503
LOC_Os06g35560	LOC_Os10g29620	9.31646838
LOC_Os06g35560	LOC_Os11g33240	10.0821328
LOC_Os06g35630	LOC_Os09g31120	8.81810194
LOC_Os06g35630	LOC_Os09g32800	10.5891439
LOC_Os06g35660	LOC_Os07g38540	9.3350466
LOC_Os06g35730	LOC_Os08g32620	8.95864719
LOC_Os06g35700	LOC_Os09g28460	9.46528876

InteractorA	InteractorB	Z score
LOC_Os06g35650	LOC_Os07g44460	10.040323
LOC_Os06g35560	LOC_Os06g37500	8.87565414
LOC_Os06g35590	LOC_Os09g36450	9.54969306
LOC_Os06g35730	LOC_Os07g46990	8.86908878
LOC_Os06g35590	LOC_Os11g08340	9.91768428
LOC_Os06g35560	LOC_Os11g32510	9.69792624
LOC_Os06g35730	LOC_Os10g02480	9.24245646
LOC_Os06g35660	LOC_Os07g44620	8.93524233
LOC_Os06g35650	LOC_Os12g44030	10.4667859
LOC_Os06g35730	LOC_Os08g14760	8.93895674
LOC_Os06g35560	LOC_Os09g10300	8.94198756
LOC_Os06g35650	LOC_Os07g03409	9.23727963
LOC_Os06g36700	LOC_Os09g07510	8.94595817
LOC_Os06g35540	LOC_Os11g37640	9.10679743
LOC_Os06g35730	LOC_Os12g23170	9.19370626
LOC_Os06g35940	LOC_Os07g34589	8.84971655
LOC_Os06g35560	LOC_Os10g34520	9.64602581
LOC_Os06g35540	LOC_Os08g01660	8.82155905
LOC_Os06g35540	LOC_Os10g21248	12.0026925
LOC_Os06g35540	LOC_Os07g47490	10.0818365
LOC_Os06g36670	LOC_Os09g21770	9.05421441
LOC_Os06g35630	LOC_Os12g07980	8.85726447
LOC_Os06g36160	LOC_Os08g39870	9.64479103
LOC_Os06g35590	LOC_Os12g10600	10.2105459
LOC_Os06g35630	LOC_Os11g26860	8.90184436
LOC_Os06g35540	LOC_Os10g28200	9.81271792
LOC_Os06g35540	LOC_Os07g37550	12.1393843
LOC_Os06g35560	LOC_Os08g09260	9.33666114
LOC_Os06g36700	LOC_Os08g35740	8.82476808
LOC_Os06g35560	LOC_Os12g12590	8.82110567
LOC_Os06g35814	LOC_Os08g15292	9.52959835
LOC_Os06g35730	LOC_Os07g22650	9.53780804
LOC_Os06g35560	LOC_Os12g25690	8.83049108
LOC_Os06g35940	LOC_Os11g08330	8.81414933
LOC_Os06g35700	LOC_Os10g07040	11.3848119
LOC_Os06g35660	LOC_Os12g33610	8.80310003
LOC_Os06g35650	LOC_Os08g06060	11.3073632
LOC_Os06g36670	LOC_Os09g10270	9.31783613
LOC_Os06g35560	LOC_Os11g25330	8.87223535
LOC_Os06g35730	LOC_Os09g28400	9.55113504
LOC_Os06g35650	LOC_Os07g03499	9.23727963
LOC_Os06g35630	LOC_Os11g26850	9.61315436
LOC_Os06g35560	LOC_Os12g02370	10.1305758

InteractorA	InteractorB	Z score
LOC_Os06g35630	LOC_Os10g39590	10.5356321
LOC_Os06g35940	LOC_Os11g31620	11.310358
LOC_Os06g36160	LOC_Os12g10720	9.15189169
LOC_Os06g35590	LOC_Os09g34970	11.0630559
LOC_Os06g35650	LOC_Os07g06410	9.07843742
LOC_Os06g35560	LOC_Os07g22930	9.90527144
LOC_Os06g35650	LOC_Os10g39840	10.3922628
LOC_Os06g35560	LOC_Os07g31770	9.47455202
LOC_Os06g35560	LOC_Os09g23550	8.85014456
LOC_Os06g35660	LOC_Os10g38630	9.08075218
LOC_Os06g35730	LOC_Os08g36900	9.21442016
LOC_Os06g35730	LOC_Os08g34210	9.00380089
LOC_Os06g35650	LOC_Os10g34760	10.0265928
LOC_Os06g35560	LOC_Os11g26910	8.97195429
LOC_Os06g35540	LOC_Os12g18880	9.29731945
LOC_Os06g35630	LOC_Os07g08170	8.85475997
LOC_Os06g35630	LOC_Os09g10200	10.0209089
LOC_Os06g35814	LOC_Os07g35940	9.46708299
LOC_Os06g35630	LOC_Os06g43640	9.16674522
LOC_Os06g35700	LOC_Os11g10510	10.2524118
LOC_Os06g35700	LOC_Os09g39500	8.98244246
LOC_Os06g36700	LOC_Os08g09250	9.68534888
LOC_Os06g35540	LOC_Os11g37890	9.48226631
LOC_Os06g35630	LOC_Os11g20790	8.83415002
LOC_Os06g35540	LOC_Os11g26910	9.78867177
LOC_Os06g35940	LOC_Os06g46940	10.2862974
LOC_Os06g35700	LOC_Os08g09210	10.1218254
LOC_Os06g35730	LOC_Os10g21248	8.80715925
LOC_Os06g35560	LOC_Os07g06440	9.54078931
LOC_Os06g35730	LOC_Os10g23900	10.2039625
LOC_Os06g35590	LOC_Os07g05160	9.28446126
LOC_Os06g35630	LOC_Os06g44080	11.6590233
LOC_Os06g36670	LOC_Os07g43260	9.55907475
LOC_Os06g35814	LOC_Os11g08445	8.91820722
LOC_Os06g36670	LOC_Os07g31770	9.17702541
LOC_Os06g35650	LOC_Os07g03319	9.23727963
LOC_Os06g35730	LOC_Os09g04050	9.16959703
LOC_Os06g35650	LOC_Os10g07229	10.04766
LOC_Os06g35630	LOC_Os12g44350	10.0423088
LOC_Os06g35560	LOC_Os09g11230	10.3761487
LOC_Os06g35560	LOC_Os11g16590	8.88226899
LOC_Os06g35940	LOC_Os06g47320	8.83797014
LOC_Os06g36670	LOC_Os12g34450	9.40705944

InteractorA	InteractorB	Z score
LOC_Os06g35560	LOC_Os07g26900	10.1803398
LOC_Os06g35630	LOC_Os08g28820	9.36818955
LOC_Os06g35650	LOC_Os07g16970	9.36940685
LOC_Os06g36160	LOC_Os08g04180	8.91325707
LOC_Os06g35940	LOC_Os07g05400	9.60629917
LOC_Os06g35660	LOC_Os12g02370	9.22053861
LOC_Os06g35940	LOC_Os07g40290	8.92270919
LOC_Os06g36670	LOC_Os06g44270	8.92997246
LOC_Os06g35700	LOC_Os11g47560	9.14213977
LOC_Os06g35660	LOC_Os08g04540	8.87803289
LOC_Os06g35560	LOC_Os08g40740	9.07846363
LOC_Os06g35730	LOC_Os09g39380	8.817943
LOC_Os06g36700	LOC_Os07g05400	8.81986504
LOC_Os06g37660	LOC_Os07g48160	8.80611715
LOC_Os06g36880	LOC_Os11g37890	9.15501048
LOC_Os06g36770	LOC_Os10g40700	9.83662274
LOC_Os06g36840	LOC_Os09g36900	9.77586016
LOC_Os06g37150	LOC_Os08g14770	10.0664351
LOC_Os06g36840	LOC_Os12g38770	9.94612899
LOC_Os06g37660	LOC_Os12g12470	9.28564323
LOC_Os06g37660	LOC_Os07g16970	8.8605894
LOC_Os06g36840	LOC_Os11g04954	9.5645935
LOC_Os06g36820	LOC_Os11g32540	8.82303736
LOC_Os06g36820	LOC_Os10g26050	9.2467666
LOC_Os06g36770	LOC_Os11g05730	9.28016674
LOC_Os06g37150	LOC_Os07g46830	8.96582797
LOC_Os06g37610	LOC_Os12g13320	8.9354973
LOC_Os06g37500	LOC_Os10g38580	9.20391025
LOC_Os06g39140	LOC_Os11g31620	9.14962428
LOC_Os06g36880	LOC_Os12g08280	9.71750184
LOC_Os06g37560	LOC_Os10g09860	9.70816014
LOC_Os06g36840	LOC_Os08g09940	9.26965959
LOC_Os06g37610	LOC_Os10g08580	9.1245382
LOC_Os06g37500	LOC_Os11g32580	9.13755779
LOC_Os06g37180	LOC_Os07g03690	9.14759288
LOC_Os06g37080	LOC_Os08g36320	9.08640158
LOC_Os06g36770	LOC_Os10g22070	9.7578782
LOC_Os06g36820	LOC_Os10g21266	9.82984941
LOC_Os06g36700	LOC_Os10g02040	12.4234718
LOC_Os06g37150	LOC_Os09g31506	9.31632884
LOC_Os06g37080	LOC_Os10g07616	8.98699101
LOC_Os06g37080	LOC_Os09g38030	9.10092906
LOC_Os06g36700	LOC_Os12g25630	9.59726026

InteractorA	InteractorB	Z score
LOC_Os06g37150	LOC_Os07g48880	9.31916616
LOC_Os06g37150	LOC_Os10g32680	9.29374603
LOC_Os06g37150	LOC_Os06g49970	11.0390895
LOC_Os06g36820	LOC_Os10g08580	10.5630251
LOC_Os06g37660	LOC_Os07g39870	8.91852242
LOC_Os06g37660	LOC_Os12g42280	8.8749916
LOC_Os06g37500	LOC_Os08g44350	9.49174049
LOC_Os06g36840	LOC_Os09g38030	10.1638067
LOC_Os06g37660	LOC_Os08g03040	11.0859913
LOC_Os06g37560	LOC_Os10g38640	9.99054861
LOC_Os06g36840	LOC_Os10g37060	9.18927907
LOC_Os06g37080	LOC_Os09g27750	9.29295688
LOC_Os06g37660	LOC_Os07g08030	9.23187991
LOC_Os06g37150	LOC_Os12g17540	9.64096904
LOC_Os06g39140	LOC_Os08g23730	8.81923288
LOC_Os06g36770	LOC_Os08g23730	8.81177842
LOC_Os06g36770	LOC_Os08g37790	11.1011353
LOC_Os06g37150	LOC_Os08g28680	9.1574546
LOC_Os06g37150	LOC_Os10g28320	8.85223411
LOC_Os06g37080	LOC_Os11g08460	9.14286885
LOC_Os06g39140	LOC_Os07g08500	10.7661582
LOC_Os06g36770	LOC_Os08g33100	9.72004251
LOC_Os06g36770	LOC_Os09g12230	9.25637839
LOC_Os06g36880	LOC_Os10g32870	8.96017653
LOC_Os06g36840	LOC_Os10g29470	9.66890385
LOC_Os06g37660	LOC_Os12g31370	9.92025366
LOC_Os06g37180	LOC_Os11g05730	9.2221175
LOC_Os06g36700	LOC_Os10g08550	10.9705889
LOC_Os06g36840	LOC_Os08g39870	9.48885727
LOC_Os06g36820	LOC_Os09g11230	8.97657949
LOC_Os06g37150	LOC_Os08g36900	10.001033
LOC_Os06g37560	LOC_Os11g08120	8.97927891
LOC_Os06g37150	LOC_Os07g08170	9.97927241
LOC_Os06g36700	LOC_Os12g41110	11.6470845
LOC_Os06g37660	LOC_Os10g25140	11.3118287
LOC_Os06g36770	LOC_Os08g44520	9.39977733
LOC_Os06g37150	LOC_Os07g43250	8.86468569
LOC_Os06g36700	LOC_Os11g26850	9.38191553
LOC_Os06g37150	LOC_Os12g14070	9.52112115
LOC_Os06g37560	LOC_Os09g23540	8.80687493
LOC_Os06g36820	LOC_Os12g36950	8.85410726
LOC_Os06g37150	LOC_Os11g47580	9.84551201
LOC_Os06g36820	LOC_Os06g41810	9.34537954

InteractorA	InteractorB	Z score
LOC_Os06g37150	LOC_Os07g31750	9.81610009
LOC_Os06g36770	LOC_Os11g14910	8.85688395
LOC_Os06g37610	LOC_Os09g08880	10.2116269
LOC_Os06g37150	LOC_Os07g42924	9.10234704
LOC_Os06g36770	LOC_Os10g38780	10.189876
LOC_Os06g36820	LOC_Os11g16590	10.3663245
LOC_Os06g37610	LOC_Os10g32550	8.86428171
LOC_Os06g37660	LOC_Os10g38234	9.15953976
LOC_Os06g37150	LOC_Os10g38360	10.1687225
LOC_Os06g37610	LOC_Os06g48180	9.38317757
LOC_Os06g37080	LOC_Os11g20790	8.89889365
LOC_Os06g37610	LOC_Os07g46310	9.18006608
LOC_Os06g36820	LOC_Os07g46310	9.4439677
LOC_Os06g37560	LOC_Os12g31640	8.96402447
LOC_Os06g37150	LOC_Os07g03600	9.00531665
LOC_Os06g37660	LOC_Os10g25130	11.9428943
LOC_Os06g36770	LOC_Os07g48430	8.94878476
LOC_Os06g37610	LOC_Os12g16250	8.97343226
LOC_Os06g36770	LOC_Os07g36130	14.005392
LOC_Os06g36770	LOC_Os10g39120	8.8187667
LOC_Os06g36820	LOC_Os10g32550	8.92119063
LOC_Os06g39140	LOC_Os09g27420	9.12501276
LOC_Os06g37610	LOC_Os08g03440	10.1879083
LOC_Os06g37150	LOC_Os08g40140	8.81200727
LOC_Os06g39140	LOC_Os07g48050	10.078242
LOC_Os06g37610	LOC_Os07g43170	9.59645571
LOC_Os06g36770	LOC_Os07g47290	8.80357197
LOC_Os06g37560	LOC_Os10g21192	9.1005443
LOC_Os06g39140	LOC_Os12g42876	10.3933512
LOC_Os06g37610	LOC_Os06g48200	8.97449694
LOC_Os06g37500	LOC_Os11g19800	9.08117655
LOC_Os06g36770	LOC_Os12g40830	9.02469595
LOC_Os06g37560	LOC_Os12g14070	8.87348172
LOC_Os06g36770	LOC_Os11g26860	9.05736024
LOC_Os06g36880	LOC_Os09g39780	9.25778845
LOC_Os06g36880	LOC_Os07g22950	9.95128692
LOC_Os06g37180	LOC_Os12g07720	9.79616145
LOC_Os06g37150	LOC_Os06g47320	9.21933599
LOC_Os06g37610	LOC_Os09g39570	9.00980363
LOC_Os06g36880	LOC_Os08g42560	10.3538563
LOC_Os06g36700	LOC_Os10g26600	8.8388955
LOC_Os06g37660	LOC_Os10g35070	9.41389048
LOC_Os06g36880	LOC_Os10g02070	8.80806439

InteractorA	InteractorB	Z score
LOC_Os06g37660	LOC_Os07g42924	11.3476276
LOC_Os06g36840	LOC_Os08g15040	9.52309513
LOC_Os06g40170	LOC_Os10g01540	9.88611541
LOC_Os06g40180	LOC_Os08g15292	10.6746569
LOC_Os06g40170	LOC_Os07g43170	8.82158015
LOC_Os06g40190	LOC_Os07g48010	9.7243039
LOC_Os06g39230	LOC_Os08g32850	8.83784595
LOC_Os06g39708	LOC_Os08g09940	8.80590889
LOC_Os06g41810	LOC_Os10g23900	9.14659154
LOC_Os06g40180	LOC_Os11g30290	10.432886
LOC_Os06g40170	LOC_Os10g25140	9.25533496
LOC_Os06g39740	LOC_Os11g26910	9.01141126
LOC_Os06g40940	LOC_Os08g27840	10.2833275
LOC_Os06g39875	LOC_Os08g14760	9.05756991
LOC_Os06g40170	LOC_Os12g02370	9.52287564
LOC_Os06g39740	LOC_Os07g31270	9.62353808
LOC_Os06g42130	LOC_Os11g10480	8.90408519
LOC_Os06g39230	LOC_Os07g43820	9.18712528
LOC_Os06g39230	LOC_Os12g13800	10.4864439
LOC_Os06g39230	LOC_Os10g27174	9.02021232
LOC_Os06g40180	LOC_Os10g21266	9.16268728
LOC_Os06g39875	LOC_Os08g02400	10.6300351
LOC_Os06g40180	LOC_Os10g41490	9.38360818
LOC_Os06g39875	LOC_Os12g42280	8.8911889
LOC_Os06g39740	LOC_Os07g38430	8.86643501
LOC_Os06g40190	LOC_Os07g05820	10.2068607
LOC_Os06g40180	LOC_Os12g44000	9.04709966
LOC_Os06g42130	LOC_Os11g14040	8.99312433
LOC_Os06g40180	LOC_Os09g23560	9.4673646
LOC_Os06g39728	LOC_Os12g10570	9.65921952
LOC_Os06g41810	LOC_Os08g10608	8.81628268
LOC_Os06g41810	LOC_Os09g33500	9.77160648
LOC_Os06g39708	LOC_Os07g42490	9.62622194
LOC_Os06g40180	LOC_Os10g05069	9.18414553
LOC_Os06g42130	LOC_Os07g08660	9.19785417
LOC_Os06g40170	LOC_Os11g02600	8.96202904
LOC_Os06g40940	LOC_Os07g30970	9.28553597
LOC_Os06g39740	LOC_Os07g02210	9.06813716
LOC_Os06g40940	LOC_Os10g29470	8.92016866
LOC_Os06g42130	LOC_Os07g43260	8.94907032
LOC_Os06g42130	LOC_Os12g12580	8.87381443
LOC_Os06g42560	LOC_Os08g44340	10.9902632
LOC_Os06g39728	LOC_Os12g38750	8.96503255

InteractorA	InteractorB	Z score
LOC_Os06g39875	LOC_Os11g30310	11.6424727
LOC_Os06g40640	LOC_Os09g08120	8.9590006
LOC_Os06g40180	LOC_Os10g38360	9.36430645
LOC_Os06g42560	LOC_Os09g39380	9.11317965
LOC_Os06g40940	LOC_Os12g38180	8.80889959
LOC_Os06g40170	LOC_Os10g27050	9.23447586
LOC_Os06g42560	LOC_Os07g31750	9.03164787
LOC_Os06g42130	LOC_Os08g42730	9.16685324
LOC_Os06g42130	LOC_Os07g07240	9.17595754
LOC_Os06g39875	LOC_Os10g11810	10.2401595
LOC_Os06g40640	LOC_Os06g51150	9.2118243
LOC_Os06g40940	LOC_Os08g15292	9.13442852
LOC_Os06g41810	LOC_Os07g26540	10.0982607
LOC_Os06g40190	LOC_Os12g02060	9.79495607
LOC_Os06g41810	LOC_Os07g05160	9.15053929
LOC_Os06g39728	LOC_Os12g41220	9.67764773
LOC_Os06g40170	LOC_Os08g15292	9.17661427
LOC_Os06g40170	LOC_Os12g12580	9.78842362
LOC_Os06g39875	LOC_Os10g08580	9.05940412
LOC_Os06g39708	LOC_Os07g06970	9.8111903
LOC_Os06g40190	LOC_Os12g22650	8.80388676
LOC_Os06g40640	LOC_Os08g01660	9.54534498
LOC_Os06g39740	LOC_Os12g16250	8.82313651
LOC_Os06g41810	LOC_Os09g08910	9.78682677
LOC_Os06g40170	LOC_Os08g34280	9.39425957
LOC_Os06g39708	LOC_Os08g28800	9.45094474
LOC_Os06g39708	LOC_Os10g38580	9.2053359
LOC_Os06g40940	LOC_Os08g38900	9.30699139
LOC_Os06g40940	LOC_Os09g10300	8.82457272
LOC_Os06g40170	LOC_Os12g08270	9.39301124
LOC_Os06g39740	LOC_Os06g44620	9.31648076
LOC_Os06g39708	LOC_Os09g39380	9.07479682
LOC_Os06g40170	LOC_Os10g39120	9.52098879
LOC_Os06g39875	LOC_Os07g47420	9.09969575
LOC_Os06g39708	LOC_Os08g09250	9.25295888
LOC_Os06g39708	LOC_Os07g38430	9.63488483
LOC_Os06g42130	LOC_Os10g41490	9.22306962
LOC_Os06g39740	LOC_Os07g11440	8.90986491
LOC_Os06g40640	LOC_Os10g08670	9.14723474
LOC_Os06g40180	LOC_Os12g07720	9.97163226
LOC_Os06g39230	LOC_Os09g31410	8.85138699
LOC_Os06g40170	LOC_Os12g18880	9.4112569
LOC_Os06g39230	LOC_Os07g07709	10.7878969

InteractorA	InteractorB	Z score
LOC_Os06g39875	LOC_Os09g36710	10.2237454
LOC_Os06g42130	LOC_Os06g51150	9.15564796
LOC_Os06g39230	LOC_Os09g23530	8.8072326
LOC_Os06g40940	LOC_Os08g38920	9.34279451
LOC_Os06g41810	LOC_Os09g23530	9.29845508
LOC_Os06g42130	LOC_Os11g26860	9.15422493
LOC_Os06g40190	LOC_Os10g38710	10.867892
LOC_Os06g41810	LOC_Os10g38140	9.34691517
LOC_Os06g40170	LOC_Os08g38900	9.03278249
LOC_Os06g39875	LOC_Os08g39870	8.88509617
LOC_Os06g40170	LOC_Os07g43250	9.9546117
LOC_Os06g39875	LOC_Os10g39170	9.27247197
LOC_Os06g39230	LOC_Os08g09200	10.5502247
LOC_Os06g40940	LOC_Os11g03290	9.71609323
LOC_Os06g40180	LOC_Os11g25260	9.04788878
LOC_Os06g40180	LOC_Os07g31830	8.95713418
LOC_Os06g40170	LOC_Os07g36190	8.83351419
LOC_Os06g40180	LOC_Os06g46940	9.22422018
LOC_Os06g39875	LOC_Os11g32260	9.14527179
LOC_Os06g45100	LOC_Os08g20270	9.24755532
LOC_Os06g44620	LOC_Os11g32770	9.45943759
LOC_Os06g44620	LOC_Os12g34450	10.7870999
LOC_Os06g44270	LOC_Os09g14670	9.24617037
LOC_Os06g45100	LOC_Os12g07830	8.97033863
LOC_Os06g46000	LOC_Os09g10230	9.08249316
LOC_Os06g44080	LOC_Os07g44260	9.08307914
LOC_Os06g43640	LOC_Os07g03377	8.8020271
LOC_Os06g46284	LOC_Os10g09860	8.85767745
LOC_Os06g43640	LOC_Os11g30310	9.63139761
LOC_Os06g44620	LOC_Os09g20284	8.99371387
LOC_Os06g44620	LOC_Os06g45670	9.41429786
LOC_Os06g45710	LOC_Os07g35880	10.55853
LOC_Os06g43640	LOC_Os12g44020	8.95878149
LOC_Os06g44080	LOC_Os10g07229	10.6696577
LOC_Os06g45710	LOC_Os11g47760	10.651791
LOC_Os06g45070	LOC_Os12g43440	9.64624701
LOC_Os06g44620	LOC_Os09g10230	9.0557826
LOC_Os06g44270	LOC_Os09g28770	8.8119496
LOC_Os06g43640	LOC_Os07g03288	8.8020271
LOC_Os06g45590	LOC_Os10g26050	8.91641339
LOC_Os06g44270	LOC_Os10g02480	9.53584589
LOC_Os06g43640	LOC_Os11g01872	10.315239
LOC_Os06g45670	LOC_Os09g24990	9.90289849

InteractorA	InteractorB	Z score
LOC_Os06g44620	LOC_Os12g38180	9.68738114
LOC_Os06g43640	LOC_Os07g03467	8.8020271
LOC_Os06g46000	LOC_Os07g22600	9.48411356
LOC_Os06g44270	LOC_Os11g10480	9.22710209
LOC_Os06g44080	LOC_Os08g40140	9.60340545
LOC_Os06g45120	LOC_Os09g38030	8.90660507
LOC_Os06g45590	LOC_Os07g34589	9.39373928
LOC_Os06g46000	LOC_Os11g20790	9.1731832
LOC_Os06g44080	LOC_Os09g39570	9.87040807
LOC_Os06g43640	LOC_Os12g34874	10.2547203
LOC_Os06g43640	LOC_Os12g01922	9.10595121
LOC_Os06g43640	LOC_Os06g51150	8.89976362
LOC_Os06g45590	LOC_Os12g16410	11.247996
LOC_Os06g43660	LOC_Os12g44020	9.6162399
LOC_Os06g45100	LOC_Os08g44270	9.23226968
LOC_Os06g43640	LOC_Os11g34450	9.27150332
LOC_Os06g46000	LOC_Os12g04980	9.28899611
LOC_Os06g45710	LOC_Os08g06060	9.09736703
LOC_Os06g44080	LOC_Os07g38540	9.11876718
LOC_Os06g45710	LOC_Os11g38959	8.8739361
LOC_Os06g44620	LOC_Os10g22450	9.18414764
LOC_Os06g45590	LOC_Os07g48880	8.84914056
LOC_Os06g46000	LOC_Os06g48180	9.01134947
LOC_Os06g45590	LOC_Os10g21310	8.83649314
LOC_Os06g46284	LOC_Os09g30418	9.56199542
LOC_Os06g44620	LOC_Os08g38920	9.71494705
LOC_Os06g45120	LOC_Os12g10560	8.9321167
LOC_Os06g45590	LOC_Os08g33370	9.26774805
LOC_Os06g45590	LOC_Os09g24530	9.51937525
LOC_Os06g46000	LOC_Os12g42280	8.82063695
LOC_Os06g45120	LOC_Os11g14040	9.63736394
LOC_Os06g45100	LOC_Os09g28420	9.92629619
LOC_Os06g45670	LOC_Os09g15420	8.97646863
LOC_Os06g44620	LOC_Os10g21266	9.37847981
LOC_Os06g43640	LOC_Os12g36950	9.19894661
LOC_Os06g44620	LOC_Os08g39300	8.85702971
LOC_Os06g44620	LOC_Os07g08660	9.22266553
LOC_Os06g45710	LOC_Os10g21344	8.8838254
LOC_Os06g45120	LOC_Os10g42940	9.15676013
LOC_Os06g43640	LOC_Os11g37890	8.88352762
LOC_Os06g44270	LOC_Os08g44340	8.86748994
LOC_Os06g45070	LOC_Os09g28420	10.3287371
LOC_Os06g43640	LOC_Os08g16910	9.42527265

InteractorA	InteractorB	Z score
LOC_Os06g45100	LOC_Os10g29620	11.6268584
LOC_Os06g44080	LOC_Os10g21266	9.07782601
LOC_Os06g45590	LOC_Os08g03290	14.3214955
LOC_Os06g45100	LOC_Os07g08030	8.91525813
LOC_Os06g45120	LOC_Os07g38030	9.19763928
LOC_Os06g44080	LOC_Os12g02980	8.99499252
LOC_Os06g45670	LOC_Os11g47600	9.16827973
LOC_Os06g46284	LOC_Os09g36450	8.95588143
LOC_Os06g44620	LOC_Os11g37960	9.02069268
LOC_Os06g44620	LOC_Os06g51084	9.49257889
LOC_Os06g44620	LOC_Os08g14770	9.62285014
LOC_Os06g45120	LOC_Os10g08670	8.84969357
LOC_Os06g43660	LOC_Os10g01570	9.90319519
LOC_Os06g43640	LOC_Os07g38860	12.5481081
LOC_Os06g45120	LOC_Os07g37790	9.39991899
LOC_Os06g46000	LOC_Os09g30360	8.81221775
LOC_Os06g44080	LOC_Os10g35480	9.10483858
LOC_Os06g45710	LOC_Os10g34520	8.83425886
LOC_Os06g45710	LOC_Os11g14040	9.17912221
LOC_Os06g44080	LOC_Os12g31640	9.41668496
LOC_Os06g44620	LOC_Os08g34190	9.82708439
LOC_Os06g46284	LOC_Os09g31502	9.25411242
LOC_Os06g44620	LOC_Os12g33610	9.23265015
LOC_Os06g45590	LOC_Os09g38030	8.86516396
LOC_Os06g43640	LOC_Os10g11810	10.9511931
LOC_Os06g43660	LOC_Os08g41830	10.3156422
LOC_Os06g44620	LOC_Os09g10260	9.30740392
LOC_Os06g45100	LOC_Os11g47580	9.75428095
LOC_Os06g44620	LOC_Os09g28770	9.74145839
LOC_Os06g43660	LOC_Os12g13380	9.22186505
LOC_Os06g44620	LOC_Os12g17540	8.99142756
LOC_Os06g43640	LOC_Os07g03590	8.8020271
LOC_Os06g43640	LOC_Os06g49470	9.16983481
LOC_Os06g43640	LOC_Os10g25950	10.5783194
LOC_Os06g45120	LOC_Os06g48200	8.81756464
LOC_Os06g45710	LOC_Os09g34970	8.86821131
LOC_Os06g46000	LOC_Os10g28050	12.2353991
LOC_Os06g45120	LOC_Os11g19220	8.9054858
LOC_Os06g45670	LOC_Os07g38860	9.67607916
LOC_Os06g46000	LOC_Os07g26540	9.38408811
LOC_Os06g43660	LOC_Os11g05880	8.80600552
LOC_Os06g45100	LOC_Os09g08120	9.78488345
LOC_Os06g43640	LOC_Os12g44010	8.91958143

InteractorA	InteractorB	Z score
LOC_Os06g49970	LOC_Os12g40510	9.13469357
LOC_Os06g50300	LOC_Os07g38430	9.12421361
LOC_Os06g49970	LOC_Os12g43630	9.14807671
LOC_Os06g47320	LOC_Os10g07616	10.9146575
LOC_Os06g49470	LOC_Os12g42280	9.0834405
LOC_Os06g50300	LOC_Os09g38030	9.1410358
LOC_Os06g49970	LOC_Os08g44960	9.29868125
LOC_Os06g46940	LOC_Os06g51060	9.28175525
LOC_Os06g48180	LOC_Os07g47290	8.95593417
LOC_Os06g48160	LOC_Os09g37100	8.89191939
LOC_Os06g48180	LOC_Os08g28730	8.8981965
LOC_Os06g46940	LOC_Os08g34170	8.91500241
LOC_Os06g47600	LOC_Os11g03230	10.1076529
LOC_Os06g48160	LOC_Os10g39590	9.56082843
LOC_Os06g49470	LOC_Os11g31530	8.99104529
LOC_Os06g50300	LOC_Os07g37550	9.14539645
LOC_Os06g49970	LOC_Os12g12560	9.35961022
LOC_Os06g46940	LOC_Os09g31506	9.29172562
LOC_Os06g48200	LOC_Os11g32260	9.45078165
LOC_Os06g48200	LOC_Os12g16220	9.04270658
LOC_Os06g50300	LOC_Os08g31060	9.44768611
LOC_Os06g46436	LOC_Os11g33270	9.01427351
LOC_Os06g48200	LOC_Os10g38640	9.00514539
LOC_Os06g48200	LOC_Os08g44280	11.2836799
LOC_Os06g50300	LOC_Os08g06060	9.20416016
LOC_Os06g48180	LOC_Os11g37950	9.31863965
LOC_Os06g48180	LOC_Os09g35800	9.17155138
LOC_Os06g48180	LOC_Os06g51084	8.81336045
LOC_Os06g46340	LOC_Os09g25390	9.16840913
LOC_Os06g49970	LOC_Os08g42410	10.4192856
LOC_Os06g49970	LOC_Os08g36900	8.92189707
LOC_Os06g48180	LOC_Os09g20284	9.11427889
LOC_Os06g49970	LOC_Os09g39570	9.00502925
LOC_Os06g48180	LOC_Os10g22450	9.78220766
LOC_Os06g49970	LOC_Os12g34380	9.1543848
LOC_Os06g48310	LOC_Os08g44340	9.05842313
LOC_Os06g50300	LOC_Os07g03580	11.1068134
LOC_Os06g46940	LOC_Os12g25690	9.40975582
LOC_Os06g50300	LOC_Os10g26130	8.95095593
LOC_Os06g47600	LOC_Os09g39380	9.76899378
LOC_Os06g48160	LOC_Os12g10720	9.15856517
LOC_Os06g47600	LOC_Os09g10270	10.2025434
LOC_Os06g46372	LOC_Os10g14150	9.17111768

InteractorA	InteractorB	Z score
LOC_Os06g47600	LOC_Os07g37320	9.0440625
LOC_Os06g49470	LOC_Os10g27174	9.81938974
LOC_Os06g50300	LOC_Os11g05880	11.0155188
LOC_Os06g48180	LOC_Os08g23730	9.49108999
LOC_Os06g46340	LOC_Os09g10200	8.92042096
LOC_Os06g48180	LOC_Os09g39380	9.28670192
LOC_Os06g50300	LOC_Os10g38470	10.6994128
LOC_Os06g48160	LOC_Os07g09340	9.70241865
LOC_Os06g50300	LOC_Os12g10600	9.89734848
LOC_Os06g49470	LOC_Os07g36130	9.01333433
LOC_Os06g47600	LOC_Os07g41750	9.76657667
LOC_Os06g49970	LOC_Os10g25130	9.19060623
LOC_Os06g48180	LOC_Os12g31640	9.51102059
LOC_Os06g46372	LOC_Os07g28480	9.22372647
LOC_Os06g49970	LOC_Os07g36130	10.3878843
LOC_Os06g48200	LOC_Os09g36830	8.88957877
LOC_Os06g46436	LOC_Os07g23730	8.91809993
LOC_Os06g49470	LOC_Os07g03580	9.79325637
LOC_Os06g46372	LOC_Os08g06550	8.89198317
LOC_Os06g46340	LOC_Os09g32640	9.45850941
LOC_Os06g48180	LOC_Os12g43490	10.9424078
LOC_Os06g49970	LOC_Os07g10720	9.83141841
LOC_Os06g47320	LOC_Os08g15292	9.05563104
LOC_Os06g47320	LOC_Os07g44790	10.6619712
LOC_Os06g50300	LOC_Os07g39870	9.31971743
LOC_Os06g50300	LOC_Os11g25220	9.92625607
LOC_Os06g50300	LOC_Os07g08170	9.31954608
LOC_Os06g50300	LOC_Os10g38690	9.93576296
LOC_Os06g48200	LOC_Os09g10200	9.61695728
LOC_Os06g48160	LOC_Os10g08620	9.0596339
LOC_Os06g49970	LOC_Os12g25700	9.12763627
LOC_Os06g48200	LOC_Os11g42350	9.01347812
LOC_Os06g46340	LOC_Os09g28420	9.45605096
LOC_Os06g48180	LOC_Os11g32260	11.5193176
LOC_Os06g47600	LOC_Os08g34170	10.0447345
LOC_Os06g48160	LOC_Os08g33710	9.20153246
LOC_Os06g47320	LOC_Os09g23550	10.1037053
LOC_Os06g50300	LOC_Os11g26910	8.98884827
LOC_Os06g48180	LOC_Os11g26910	9.60707874
LOC_Os06g46940	LOC_Os08g41830	8.87681527
LOC_Os06g50300	LOC_Os12g06620	9.19179976
LOC_Os06g48160	LOC_Os10g41550	9.33403276
LOC_Os06g49470	LOC_Os08g40930	8.92465117

InteractorA	InteractorB	Z score
LOC_Os06g49970	LOC_Os08g35440	9.04689015
LOC_Os06g49970	LOC_Os12g02980	10.2186095
LOC_Os06g47320	LOC_Os11g06390	8.96469101
LOC_Os06g49470	LOC_Os10g30840	9.32514904
LOC_Os01g22490	LOC_Os06g04200	9.82259836
LOC_Os01g22352	LOC_Os05g11710	9.48024947
LOC_Os01g22490	LOC_Os05g28280	8.8303013
LOC_Os01g22520	LOC_Os02g13530	9.29100469
LOC_Os01g22352	LOC_Os11g14910	9.0143144
LOC_Os01g23580	LOC_Os03g45960	8.97718339
LOC_Os01g22520	LOC_Os01g59490	9.34947871
LOC_Os01g23580	LOC_Os06g11200	9.40156994
LOC_Os01g22490	LOC_Os07g30200	11.7129431
LOC_Os01g22520	LOC_Os04g10000	9.15476695
LOC_Os01g22352	LOC_Os03g40720	9.28166325
LOC_Os01g23580	LOC_Os01g49890	11.0060909
LOC_Os01g22490	LOC_Os04g37820	10.0057839
LOC_Os01g22370	LOC_Os01g58020	9.26305074
LOC_Os01g23580	LOC_Os05g04520	10.1179453
LOC_Os01g22370	LOC_Os09g23530	8.98537838
LOC_Os01g22370	LOC_Os11g29400	8.8897431
LOC_Os01g22520	LOC_Os02g56700	8.90977954
LOC_Os01g22520	LOC_Os03g07840	8.87725373
LOC_Os01g22490	LOC_Os12g12560	11.7796326
LOC_Os01g22520	LOC_Os03g55620	9.58618003
LOC_Os01g22352	LOC_Os08g36900	9.00386004
LOC_Os01g22520	LOC_Os10g21212	11.6284373
LOC_Os01g22370	LOC_Os06g04200	9.41663132
LOC_Os01g22490	LOC_Os02g57040	9.31081878
LOC_Os01g22520	LOC_Os10g38470	10.6891014
LOC_Os01g22490	LOC_Os03g48471	9.91249477
LOC_Os01g22370	LOC_Os04g02820	10.6104636
LOC_Os01g22490	LOC_Os09g26380	8.96741448
LOC_Os01g23580	LOC_Os06g05700	10.6098038
LOC_Os01g22370	LOC_Os05g35330	9.26306013
LOC_Os01g23580	LOC_Os03g44150	9.85846539
LOC_Os01g22490	LOC_Os04g33740	8.90898464
LOC_Os01g22520	LOC_Os04g18650	8.86034399
LOC_Os01g22520	LOC_Os05g06750	13.0393012
LOC_Os01g22490	LOC_Os09g25320	9.14316035
LOC_Os01g23580	LOC_Os11g14910	10.5164071
LOC_Os01g22520	LOC_Os12g16410	9.70802898
LOC_Os01g22370	LOC_Os06g35560	9.02631775

InteractorA	InteractorB	Z score
LOC_Os01g22490	LOC_Os10g38140	9.02022744
LOC_Os01g22490	LOC_Os11g32520	9.66127798
LOC_Os01g23620	LOC_Os01g50050	9.39380455
LOC_Os01g22490	LOC_Os06g35490	8.82749953
LOC_Os01g22352	LOC_Os06g35730	9.48024947
LOC_Os01g23580	LOC_Os08g33370	9.25710734
LOC_Os01g22490	LOC_Os01g44220	9.4473376
LOC_Os01g22490	LOC_Os06g14510	11.8335845
LOC_Os01g22520	LOC_Os06g35590	8.97547307
LOC_Os01g22370	LOC_Os02g38210	10.9755422
LOC_Os01g22520	LOC_Os04g45470	11.2731191
LOC_Os01g23580	LOC_Os05g06480	9.22645459
LOC_Os01g22370	LOC_Os08g09250	8.98007957
LOC_Os01g22352	LOC_Os10g30200	11.1203298
LOC_Os01g22490	LOC_Os03g13200	9.00087032
LOC_Os01g22352	LOC_Os03g31210	8.86761068
LOC_Os01g22490	LOC_Os08g34190	9.14138995
LOC_Os01g22370	LOC_Os10g21268	9.26305074
LOC_Os01g22520	LOC_Os04g49130	8.85523884
LOC_Os01g22490	LOC_Os02g52290	9.12967482
LOC_Os01g22490	LOC_Os01g71320	9.08882378
LOC_Os01g22352	LOC_Os07g07060	9.12454662
LOC_Os01g22352	LOC_Os08g29370	10.7021654
LOC_Os01g22352	LOC_Os03g48390	9.01738173
LOC_Os01g23580	LOC_Os02g55890	9.82936135
LOC_Os01g22520	LOC_Os05g44340	8.80596334
LOC_Os01g22370	LOC_Os03g48310	9.76062164
LOC_Os01g22352	LOC_Os10g40710	9.24257056
LOC_Os01g22520	LOC_Os10g30200	9.387464
LOC_Os01g22490	LOC_Os01g39270	9.73871049
LOC_Os01g22370	LOC_Os04g56230	8.92864351
LOC_Os01g23580	LOC_Os12g18900	9.47506677
LOC_Os01g22490	LOC_Os01g71400	9.88504056
LOC_Os01g22352	LOC_Os03g09910	9.06063766
LOC_Os01g22352	LOC_Os12g12470	9.19015445
LOC_Os01g22490	LOC_Os08g06550	10.1659142
LOC_Os01g22370	LOC_Os06g51050	9.7740012
LOC_Os01g22490	LOC_Os01g50050	8.90321416
LOC_Os01g22520	LOC_Os07g46830	9.11648366
LOC_Os01g22352	LOC_Os05g41610	8.86489872
LOC_Os01g22352	LOC_Os03g51080	9.35955335
LOC_Os01g22370	LOC_Os06g27770	9.08508868
LOC_Os01g22352	LOC_Os04g12970	8.88991814

InteractorA	InteractorB	Z score
LOC_Os01g22370	LOC_Os02g07870	8.84170408
LOC_Os01g23580	LOC_Os06g46284	10.4010785
LOC_Os01g22352	LOC_Os06g35940	8.9994344
LOC_Os01g23620	LOC_Os01g64100	9.25527325
LOC_Os01g22352	LOC_Os08g25570	9.1180926
LOC_Os01g22352	LOC_Os11g25330	9.70554262
LOC_Os01g22370	LOC_Os03g16900	10.2897764
LOC_Os01g22370	LOC_Os08g02700	11.007957
LOC_Os01g22370	LOC_Os11g05470	9.47170129
LOC_Os01g22370	LOC_Os08g40140	10.6078531
LOC_Os01g22370	LOC_Os05g33130	9.41297611
LOC_Os01g22520	LOC_Os04g22660	9.72172445
LOC_Os01g22490	LOC_Os01g49890	9.24077022
LOC_Os01g23580	LOC_Os09g30412	10.0909789
LOC_Os01g22352	LOC_Os07g34580	9.8041115
LOC_Os01g22520	LOC_Os12g07830	9.41807427
LOC_Os01g23580	LOC_Os08g10010	9.10541699
LOC_Os01g23580	LOC_Os02g18880	10.7952428
LOC_Os01g22490	LOC_Os12g13380	10.8743935
LOC_Os01g22520	LOC_Os02g03610	10.1627521
LOC_Os01g22490	LOC_Os03g55874	10.4677798
LOC_Os01g22370	LOC_Os07g05400	9.10789548
LOC_Os01g23580	LOC_Os01g24750	8.9093738
LOC_Os01g22352	LOC_Os04g57590	8.87943448
LOC_Os01g22352	LOC_Os03g32580	8.90163286
LOC_Os01g23580	LOC_Os06g23870	9.69357565
LOC_Os01g22520	LOC_Os08g44810	9.02184134
LOC_Os01g22490	LOC_Os10g26390	8.9835214
LOC_Os01g22370	LOC_Os02g52230	9.09684221
LOC_Os01g22370	LOC_Os06g35520	10.6841671
LOC_Os01g22352	LOC_Os09g39380	10.0078965
LOC_Os01g22490	LOC_Os02g18880	10.1537666
LOC_Os01g22520	LOC_Os07g01760	10.2050656
LOC_Os01g22490	LOC_Os06g44080	9.14315508
LOC_Os01g22352	LOC_Os10g41490	9.02791408
LOC_Os01g23580	LOC_Os08g39870	9.03555647
LOC_Os01g22490	LOC_Os09g30418	9.31553038
LOC_Os01g22370	LOC_Os05g34540	9.24478162
LOC_Os01g22520	LOC_Os02g57720	9.06002152
LOC_Os01g22352	LOC_Os11g03980	10.2798376
LOC_Os01g22370	LOC_Os09g26380	10.2813681
LOC_Os01g22490	LOC_Os12g07980	10.0798684
LOC_Os01g01060	LOC_Os07g23730	8.90307512

InteractorA	InteractorB	Z score
LOC_Os01g01060	LOC_Os02g52390	9.19032606
LOC_Os01g01650	LOC_Os04g39210	9.33637967
LOC_Os01g01830	LOC_Os07g41750	9.43872858
LOC_Os01g01660	LOC_Os02g58730	8.90965486
LOC_Os01g01660	LOC_Os01g18110	9.52686972
LOC_Os01g01660	LOC_Os11g03400	9.95230316
LOC_Os01g01660	LOC_Os04g20070	9.12060919
LOC_Os01g01660	LOC_Os09g20820	10.0146029
LOC_Os01g01650	LOC_Os05g44140	9.10132233
LOC_Os01g01650	LOC_Os01g46070	9.49026251
LOC_Os01g01660	LOC_Os02g08490	8.81181316
LOC_Os01g01660	LOC_Os01g59490	10.5886972
LOC_Os01g01830	LOC_Os01g51210	8.94605481
LOC_Os01g01060	LOC_Os01g61400	9.48004127
LOC_Os01g01060	LOC_Os12g22680	9.92338641
LOC_Os01g01660	LOC_Os03g55070	10.0997271
LOC_Os01g01650	LOC_Os07g43470	9.71974081
LOC_Os01g01650	LOC_Os04g48700	9.29178267
LOC_Os01g01660	LOC_Os03g25340	8.83652333
LOC_Os01g01660	LOC_Os08g39870	9.70930887
LOC_Os01g01650	LOC_Os03g41460	10.8870267
LOC_Os01g01660	LOC_Os06g11240	11.7336049
LOC_Os01g01650	LOC_Os01g50460	9.23273453
LOC_Os01g01660	LOC_Os09g31430	11.2798805
LOC_Os01g01650	LOC_Os06g02390	9.41679822
LOC_Os01g01660	LOC_Os02g40000	9.92729316
LOC_Os01g01650	LOC_Os05g01810	8.88274408
LOC_Os01g01650	LOC_Os01g51060	10.1906502
LOC_Os01g01660	LOC_Os01g47550	8.84093645
LOC_Os01g01060	LOC_Os05g35400	9.30107739
LOC_Os01g01660	LOC_Os10g35480	11.508387
LOC_Os01g01650	LOC_Os12g06620	9.25706541
LOC_Os01g01650	LOC_Os10g37060	8.97526322
LOC_Os01g02020	LOC_Os06g44620	9.0947155
LOC_Os01g01830	LOC_Os10g38489	8.97461625
LOC_Os01g01650	LOC_Os09g34970	9.37711798
LOC_Os01g01650	LOC_Os01g62290	11.2673823
LOC_Os01g02020	LOC_Os05g47545	9.09422249
LOC_Os01g01830	LOC_Os04g43760	9.56501283
LOC_Os01g01660	LOC_Os04g58110	9.45491716
LOC_Os01g01650	LOC_Os12g04980	10.1562312
LOC_Os01g01650	LOC_Os04g37480	9.3040945
LOC_Os01g01650	LOC_Os01g18120	9.68818336

InteractorA	InteractorB	Z score
LOC_Os01g01660	LOC_Os05g50380	10.150921
LOC_Os01g01660	LOC_Os07g30170	9.23580351
LOC_Os01g01830	LOC_Os07g48010	9.7965907
LOC_Os01g01660	LOC_Os02g41680	10.4725483
LOC_Os01g01060	LOC_Os03g50490	9.51842396
LOC_Os01g01650	LOC_Os12g43370	9.8848305
LOC_Os01g01650	LOC_Os01g64100	8.85326327
LOC_Os01g01650	LOC_Os07g46280	9.23673706
LOC_Os01g01650	LOC_Os06g30970	9.07495531
LOC_Os01g01650	LOC_Os01g71474	9.40604451
LOC_Os01g01060	LOC_Os03g07150	9.23346402
LOC_Os01g02020	LOC_Os04g56210	9.3645722
LOC_Os01g01650	LOC_Os01g51200	11.7388519
LOC_Os01g01830	LOC_Os10g38160	9.68007375
LOC_Os01g01650	LOC_Os08g09950	8.90100232
LOC_Os01g02020	LOC_Os05g11710	9.02735847
LOC_Os01g01650	LOC_Os02g49610	9.27693679
LOC_Os01g01660	LOC_Os03g12290	9.54099093
LOC_Os01g02020	LOC_Os03g04410	8.90576362
LOC_Os01g01660	LOC_Os12g43440	9.03889267
LOC_Os01g01660	LOC_Os11g25220	10.0407728
LOC_Os01g01660	LOC_Os03g51080	9.33841501
LOC_Os01g01650	LOC_Os03g07150	9.07738066
LOC_Os01g01650	LOC_Os07g03319	11.2352385
LOC_Os01g01650	LOC_Os04g43400	10.2679421
LOC_Os01g01650	LOC_Os11g37640	9.59176149
LOC_Os01g01650	LOC_Os01g27360	10.1165329
LOC_Os01g01650	LOC_Os02g42710	8.83236183
LOC_Os01g01660	LOC_Os07g14590	8.96461426
LOC_Os01g01660	LOC_Os04g56230	11.2546339
LOC_Os01g01660	LOC_Os04g43410	10.144236
LOC_Os01g01650	LOC_Os03g62070	9.05351674
LOC_Os01g02020	LOC_Os07g07060	9.80394389
LOC_Os01g01060	LOC_Os01g60190	11.4400897
LOC_Os01g01660	LOC_Os07g05400	11.0162382
LOC_Os01g01650	LOC_Os03g11960	9.367355
LOC_Os01g02020	LOC_Os04g24520	9.88289628
LOC_Os01g01650	LOC_Os01g27390	9.03114371
LOC_Os01g02020	LOC_Os05g33140	10.764082
LOC_Os01g01650	LOC_Os03g18570	9.26133194
LOC_Os01g01650	LOC_Os02g21460	10.4030961
LOC_Os01g01650	LOC_Os02g34600	10.0533054
LOC_Os01g02020	LOC_Os03g28330	8.83671959

InteractorA	InteractorB	Z score
LOC_Os01g01060	LOC_Os04g17064	9.33246767
LOC_Os01g01660	LOC_Os01g23620	9.33822175
LOC_Os01g01660	LOC_Os01g41510	8.84442601
LOC_Os01g01660	LOC_Os02g12580	9.59250582
LOC_Os01g02020	LOC_Os01g39830	10.8138642
LOC_Os01g01660	LOC_Os10g26110	11.3080194
LOC_Os01g01650	LOC_Os09g37100	9.41077702
LOC_Os01g01650	LOC_Os10g38140	9.50666288
LOC_Os01g01650	LOC_Os06g06730	9.48404855
LOC_Os01g01650	LOC_Os07g03409	11.2352385
LOC_Os01g01650	LOC_Os04g57590	9.07420199
LOC_Os01g02020	LOC_Os07g05800	11.0135959
LOC_Os01g01830	LOC_Os02g33110	10.5462516
LOC_Os01g01830	LOC_Os07g38890	10.2258942
LOC_Os01g02020	LOC_Os07g07470	10.5701507
LOC_Os01g01650	LOC_Os07g19040	10.6302321
LOC_Os01g01060	LOC_Os12g44030	8.94284143
LOC_Os01g01660	LOC_Os05g44050	10.4704809
LOC_Os01g01660	LOC_Os11g32650	8.81428851
LOC_Os01g01650	LOC_Os01g59790	9.18407278
LOC_Os01g01650	LOC_Os03g03130	10.5175355
LOC_Os01g01660	LOC_Os03g64210	9.63135932
LOC_Os01g01060	LOC_Os10g38700	8.84230555
LOC_Os01g01660	LOC_Os03g55800	10.0666559
LOC_Os01g01660	LOC_Os03g01170	9.77979095
LOC_Os01g01830	LOC_Os08g15040	9.41036172
LOC_Os01g01650	LOC_Os03g16880	10.1422738
LOC_Os01g01830	LOC_Os03g58530	8.83098823
LOC_Os01g01650	LOC_Os03g60400	9.29982022
LOC_Os01g01660	LOC_Os02g55400	9.6351506
LOC_Os01g01650	LOC_Os12g07820	9.54380837
LOC_Os01g01660	LOC_Os08g42560	9.56770935
LOC_Os01g01060	LOC_Os12g22650	9.26156353
LOC_Os01g01660	LOC_Os01g05630	9.00648454
LOC_Os01g01650	LOC_Os02g56690	9.2414598
LOC_Os01g01650	LOC_Os01g14950	9.55795871
LOC_Os01g01660	LOC_Os06g39140	9.76377503
LOC_Os01g01660	LOC_Os09g28400	10.5483547
LOC_Os01g01650	LOC_Os02g14160	9.24432001
LOC_Os01g01650	LOC_Os03g46060	9.23662442
LOC_Os01g01830	LOC_Os07g43390	8.84498889
LOC_Os01g01650	LOC_Os01g14040	9.15298381
LOC_Os01g01660	LOC_Os06g44080	9.13389842

InteractorA	InteractorB	Z score
LOC_Os01g01660	LOC_Os02g08420	10.2895958
LOC_Os01g01650	LOC_Os03g02920	9.2241431
LOC_Os01g01060	LOC_Os03g21950	8.80543296
LOC_Os01g01060	LOC_Os05g01970	11.253256
LOC_Os01g02020	LOC_Os01g09510	10.0939557
LOC_Os01g01830	LOC_Os02g27940	9.19496629
LOC_Os01g01060	LOC_Os10g11140	11.4559085
LOC_Os01g01650	LOC_Os11g18870	9.0383865
LOC_Os01g01660	LOC_Os02g22140	9.1598642
LOC_Os01g01660	LOC_Os09g25320	9.13388603
LOC_Os01g01660	LOC_Os04g12710	10.6919325
LOC_Os01g01650	LOC_Os08g29520	12.0850128
LOC_Os01g01650	LOC_Os09g20820	9.55307816
LOC_Os01g01650	LOC_Os09g04050	8.8623311
LOC_Os01g01660	LOC_Os07g46460	9.0828485
LOC_Os01g01650	LOC_Os07g46630	9.09653294
LOC_Os01g01650	LOC_Os10g38730	10.7286682
LOC_Os01g01650	LOC_Os09g21770	8.86427153
LOC_Os01g01660	LOC_Os06g15990	8.9516427
LOC_Os01g01650	LOC_Os01g67510	9.45005967
LOC_Os01g01660	LOC_Os04g58570	9.99539106
LOC_Os01g01660	LOC_Os01g64970	9.66220185
LOC_Os01g01650	LOC_Os07g03499	11.2352419
LOC_Os01g01660	LOC_Os05g41210	9.52412867
LOC_Os01g01660	LOC_Os06g06100	9.10631617
LOC_Os01g01650	LOC_Os08g17500	10.754651
LOC_Os01g01650	LOC_Os08g32850	9.54327651
LOC_Os01g01650	LOC_Os09g31430	9.25096903
LOC_Os01g01660	LOC_Os03g45960	11.2781814
LOC_Os01g01660	LOC_Os07g43470	9.35220285
LOC_Os01g01060	LOC_Os03g51080	10.250857
LOC_Os01g01650	LOC_Os03g11970	9.60954968
LOC_Os01g01830	LOC_Os03g04110	9.39657774
LOC_Os01g01830	LOC_Os03g58260	9.18231738
LOC_Os01g02020	LOC_Os06g35730	9.02732039
LOC_Os01g01660	LOC_Os04g09604	10.0226468
LOC_Os01g01060	LOC_Os07g02340	10.9227501
LOC_Os01g01650	LOC_Os09g24990	9.06002604
LOC_Os01g01830	LOC_Os12g43100	9.08063722
LOC_Os01g01650	LOC_Os03g16860	9.83468962
LOC_Os01g01060	LOC_Os03g22060	9.5857764
LOC_Os01g01650	LOC_Os03g30870	10.2383975
LOC_Os01g01650	LOC_Os02g46970	9.03620233

InteractorA	InteractorB	Z score
LOC_Os01g01830	LOC_Os02g08410	9.73010736
LOC_Os01g01650	LOC_Os06g11280	9.28285671
LOC_Os01g02020	LOC_Os03g02260	9.01884339
LOC_Os01g01660	LOC_Os08g34170	9.73970477
LOC_Os01g01660	LOC_Os12g03090	9.95230316
LOC_Os01g01650	LOC_Os01g71310	9.427367
LOC_Os01g01660	LOC_Os01g18120	9.91100396
LOC_Os01g01660	LOC_Os07g16970	11.4224173
LOC_Os01g01650	LOC_Os03g58300	9.86412215
LOC_Os01g01650	LOC_Os01g25100	9.36882082
LOC_Os01g01650	LOC_Os11g47560	9.01731003
LOC_Os01g01660	LOC_Os01g32364	9.33580412
LOC_Os01g01660	LOC_Os04g32650	10.1986144
LOC_Os01g01660	LOC_Os05g05620	9.04274544
LOC_Os01g01660	LOC_Os01g55870	9.38135359
LOC_Os01g01660	LOC_Os07g46310	9.17548932
LOC_Os01g01660	LOC_Os02g01280	9.02052811
LOC_Os01g01060	LOC_Os01g27340	9.31006247
LOC_Os01g01650	LOC_Os08g34170	9.23643913
LOC_Os01g01650	LOC_Os04g40874	9.65806196
LOC_Os01g01650	LOC_Os04g38220	9.21671865
LOC_Os01g01650	LOC_Os03g61740	8.91563672
LOC_Os01g01650	LOC_Os02g08520	8.9660749
LOC_Os01g01660	LOC_Os01g61400	9.88492473
LOC_Os01g01660	LOC_Os02g57630	9.25348354
LOC_Os01g01660	LOC_Os03g25370	10.964669
LOC_Os01g01650	LOC_Os03g17310	9.50992077
LOC_Os01g02020	LOC_Os02g14059	9.12295103
LOC_Os01g01650	LOC_Os11g02600	9.64096285
LOC_Os01g01830	LOC_Os10g41510	9.9167625
LOC_Os01g01660	LOC_Os01g71380	9.02773841
LOC_Os01g01830	LOC_Os06g15990	9.50260533
LOC_Os01g01660	LOC_Os04g33480	9.3204932
LOC_Os01g01650	LOC_Os01g74650	10.4241215
LOC_Os01g01660	LOC_Os07g47420	9.04727785
LOC_Os01g01650	LOC_Os06g35480	9.65798715
LOC_Os01g02020	LOC_Os02g10070	9.12416902
LOC_Os01g01650	LOC_Os12g16250	9.30564967
LOC_Os01g02020	LOC_Os01g73310	8.99790747
LOC_Os01g01060	LOC_Os10g08580	9.27540898
LOC_Os01g01650	LOC_Os03g45960	9.33351058
LOC_Os01g01650	LOC_Os07g05400	9.72356514
LOC_Os01g01660	LOC_Os01g15010	11.057739

InteractorA	InteractorB	Z score
LOC_Os01g01650	LOC_Os05g05620	9.7081051
LOC_Os01g01830	LOC_Os11g10480	9.32453393
LOC_Os01g01650	LOC_Os04g10000	9.33867823
LOC_Os01g01650	LOC_Os07g47490	9.5396172
LOC_Os01g01650	LOC_Os08g09250	9.63649699
LOC_Os01g01660	LOC_Os05g31040	9.23230755
LOC_Os01g01650	LOC_Os10g21192	9.01279695
LOC_Os01g01660	LOC_Os07g26640	12.8257565
LOC_Os06g51150	LOC_Os08g42410	9.40033516
LOC_Os07g02350	LOC_Os10g32550	9.26515817
LOC_Os06g51150	LOC_Os11g02440	8.9614939
LOC_Os06g51084	LOC_Os07g02210	12.3735843
LOC_Os06g51084	LOC_Os12g40550	8.81704199
LOC_Os06g51060	LOC_Os07g08170	9.33341455
LOC_Os07g01560	LOC_Os12g34062	9.11366462
LOC_Os07g02340	LOC_Os11g32610	9.04752148
LOC_Os07g01780	LOC_Os11g40140	8.92758356
LOC_Os07g01760	LOC_Os10g25140	10.0068027
LOC_Os06g51084	LOC_Os07g44620	9.49647673
LOC_Os06g51084	LOC_Os07g29750	11.1124792
LOC_Os07g01020	LOC_Os10g40090	9.71936754
LOC_Os07g01780	LOC_Os10g38630	9.00110452
LOC_Os07g02340	LOC_Os08g41340	10.2319693
LOC_Os06g51060	LOC_Os09g30412	9.61297105
LOC_Os07g01760	LOC_Os07g42600	10.2191777
LOC_Os07g03288	LOC_Os08g06060	11.3079453
LOC_Os06g51050	LOC_Os10g01080	10.8424574
LOC_Os07g01020	LOC_Os07g05160	9.41166929
LOC_Os07g01760	LOC_Os12g38760	10.7536582
LOC_Os07g01780	LOC_Os09g10230	9.82260825
LOC_Os06g51150	LOC_Os10g38590	10.1333122
LOC_Os06g51084	LOC_Os08g44340	8.80013407
LOC_Os07g03319	LOC_Os07g05820	10.2146054
LOC_Os07g03288	LOC_Os10g35840	9.43621867
LOC_Os06g51050	LOC_Os08g28820	9.86181049
LOC_Os06g51150	LOC_Os11g32540	9.91369964
LOC_Os07g03319	LOC_Os07g14590	9.22184712
LOC_Os06g51150	LOC_Os07g07550	10.9675422
LOC_Os06g51084	LOC_Os07g08840	9.58495704
LOC_Os06g51150	LOC_Os09g32840	9.50829803
LOC_Os06g51150	LOC_Os10g39680	10.1392395
LOC_Os06g51084	LOC_Os08g35440	8.95309742
LOC_Os07g03288	LOC_Os10g29470	9.51682519

InteractorA	InteractorB	Z score
LOC_Os06g51084	LOC_Os08g05910	9.1341796
LOC_Os06g51150	LOC_Os08g32620	10.6493041
LOC_Os07g03288	LOC_Os12g10720	9.02960992
LOC_Os07g01020	LOC_Os07g31770	9.08125773
LOC_Os07g01760	LOC_Os07g48880	10.7944744
LOC_Os06g51150	LOC_Os10g07616	9.11104352
LOC_Os07g02210	LOC_Os08g32620	12.1214392
LOC_Os07g01020	LOC_Os11g33240	9.17383329
LOC_Os06g51084	LOC_Os12g12514	9.51450011
LOC_Os06g51060	LOC_Os12g04924	8.98230763
LOC_Os06g51084	LOC_Os08g38900	8.85832999
LOC_Os07g03319	LOC_Os08g09200	11.489394
LOC_Os06g51150	LOC_Os11g38959	9.19332416
LOC_Os06g51060	LOC_Os10g34520	11.1557406
LOC_Os06g51084	LOC_Os07g40580	9.81312382
LOC_Os07g01560	LOC_Os12g02980	8.83521947
LOC_Os07g01760	LOC_Os11g08445	9.01607693
LOC_Os06g51084	LOC_Os10g32550	12.7903892
LOC_Os07g01760	LOC_Os10g25130	16.0899421
LOC_Os07g02340	LOC_Os07g20544	9.41415721
LOC_Os07g01780	LOC_Os08g29170	8.85089104
LOC_Os06g51084	LOC_Os07g22600	9.98726661
LOC_Os07g01020	LOC_Os09g10230	8.86523656
LOC_Os06g51084	LOC_Os11g14040	8.84073331
LOC_Os07g01560	LOC_Os12g24650	9.64562005
LOC_Os06g51084	LOC_Os09g20260	8.88304709
LOC_Os07g03319	LOC_Os09g27750	9.7130089
LOC_Os06g51060	LOC_Os07g05940	8.84121033
LOC_Os07g02350	LOC_Os10g26600	10.0349002
LOC_Os07g02340	LOC_Os09g20090	9.13909217
LOC_Os06g51084	LOC_Os10g31000	8.8852019
LOC_Os07g01020	LOC_Os08g39300	8.92188252
LOC_Os07g01760	LOC_Os08g32620	10.4827184
LOC_Os07g01760	LOC_Os07g26640	9.20078964
LOC_Os07g01020	LOC_Os09g32840	10.7482026
LOC_Os06g51084	LOC_Os09g20090	11.2271854
LOC_Os07g01760	LOC_Os10g27190	11.2451826
LOC_Os07g02210	LOC_Os10g40090	11.6942346
LOC_Os07g01020	LOC_Os07g03690	10.3548512
LOC_Os06g51060	LOC_Os08g39870	9.0806208
LOC_Os06g51050	LOC_Os07g36130	8.94428998
LOC_Os06g51084	LOC_Os12g16220	8.89969339
LOC_Os07g03368	LOC_Os08g44810	8.97393843

InteractorA	InteractorB	Z score
LOC_Os06g51084	LOC_Os10g38690	8.85516204
LOC_Os07g03288	LOC_Os12g40510	9.73504481
LOC_Os06g51084	LOC_Os07g04240	8.89044254
LOC_Os06g51084	LOC_Os12g25630	9.70597127
LOC_Os07g01020	LOC_Os07g16970	10.5005792
LOC_Os06g51150	LOC_Os07g43670	9.46471199
LOC_Os07g02350	LOC_Os07g48880	8.99421474
LOC_Os07g01760	LOC_Os07g42924	9.84266501
LOC_Os06g51050	LOC_Os08g39860	8.96731641
LOC_Os07g02350	LOC_Os08g39140	8.86362051
LOC_Os07g03319	LOC_Os09g23560	9.28370275
LOC_Os06g51084	LOC_Os12g32240	9.61928295
LOC_Os06g51060	LOC_Os11g47550	9.23945051
LOC_Os06g51150	LOC_Os12g25710	11.2471059
LOC_Os06g51084	LOC_Os09g25150	8.85630386
LOC_Os07g01020	LOC_Os09g12660	8.92008961
LOC_Os07g03288	LOC_Os07g34589	9.35306186
LOC_Os07g03690	LOC_Os07g07550	9.55888919
LOC_Os07g03377	LOC_Os10g35840	9.43621867
LOC_Os07g03590	LOC_Os08g06060	11.3079453
LOC_Os07g03467	LOC_Os08g06060	11.3079453
LOC_Os07g03467	LOC_Os12g10720	9.02963745
LOC_Os07g03499	LOC_Os09g23560	9.28370275
LOC_Os07g05150	LOC_Os07g34190	9.29672242
LOC_Os07g03710	LOC_Os10g01570	10.3603794
LOC_Os07g03368	LOC_Os11g06390	8.8731945
LOC_Os07g03690	LOC_Os10g28320	8.8708202
LOC_Os07g04240	LOC_Os10g38229	8.84286325
LOC_Os07g03377	LOC_Os08g06060	11.3079453
LOC_Os07g03600	LOC_Os09g27750	9.67179185
LOC_Os07g04240	LOC_Os10g38360	9.30286202
LOC_Os07g03690	LOC_Os08g33370	9.62338736
LOC_Os07g03730	LOC_Os12g31370	8.84830146
LOC_Os07g04240	LOC_Os12g13810	9.89412342
LOC_Os07g04240	LOC_Os11g06390	9.47164533
LOC_Os07g03580	LOC_Os12g05410	8.82910331
LOC_Os07g04240	LOC_Os11g05570	10.3552098
LOC_Os07g03590	LOC_Os12g10720	9.02964682
LOC_Os07g03409	LOC_Os09g27750	9.7130089
LOC_Os07g03710	LOC_Os07g34589	9.19518372
LOC_Os07g03600	LOC_Os12g40510	11.1623086
LOC_Os07g03377	LOC_Os07g34589	9.35306186
LOC_Os07g03590	LOC_Os07g34589	9.35306186

InteractorA	InteractorB	Z score
LOC_Os07g03590	LOC_Os10g29470	9.51682519
LOC_Os07g03730	LOC_Os07g42490	10.4797338
LOC_Os07g03690	LOC_Os11g32620	9.33623729
LOC_Os07g04240	LOC_Os12g17540	9.42029036
LOC_Os07g04240	LOC_Os08g34210	8.88720856
LOC_Os07g03960	LOC_Os07g47420	9.50078229
LOC_Os07g03467	LOC_Os10g29470	9.51682519
LOC_Os07g03377	LOC_Os10g29470	9.51682519
LOC_Os07g03590	LOC_Os12g40510	9.73504481
LOC_Os07g03368	LOC_Os10g38350	9.00505449
LOC_Os07g03600	LOC_Os11g03230	8.86495491
LOC_Os07g03409	LOC_Os08g09200	11.4893869
LOC_Os07g03710	LOC_Os10g26110	9.51426414
LOC_Os07g04240	LOC_Os07g38430	9.22043495
LOC_Os07g03499	LOC_Os08g09200	11.4893692
LOC_Os07g03467	LOC_Os07g34589	9.35306186
LOC_Os07g03467	LOC_Os10g35840	9.43621867
LOC_Os07g03377	LOC_Os12g10720	9.02963745
LOC_Os07g03690	LOC_Os12g13380	9.00932403
LOC_Os07g03580	LOC_Os07g05400	9.47306194
LOC_Os07g03409	LOC_Os07g14590	9.22182427
LOC_Os07g03499	LOC_Os07g05820	10.2146054
LOC_Os07g03467	LOC_Os12g40510	9.73504481
LOC_Os07g03409	LOC_Os07g05820	10.2146054
LOC_Os07g03960	LOC_Os09g08072	9.16396983
LOC_Os07g03499	LOC_Os07g14590	9.22182778
LOC_Os07g03409	LOC_Os09g23560	9.28370275
LOC_Os07g03368	LOC_Os09g35800	10.6158469
LOC_Os07g03368	LOC_Os10g38340	9.19266604
LOC_Os07g03600	LOC_Os08g44530	8.88183347
LOC_Os07g03590	LOC_Os10g35840	9.43621867
LOC_Os07g04240	LOC_Os08g02400	8.88421024
LOC_Os07g03580	LOC_Os10g27050	9.71668522
LOC_Os07g03368	LOC_Os10g38470	8.82498404
LOC_Os07g03710	LOC_Os10g21310	9.20638546
LOC_Os07g04240	LOC_Os08g31870	10.7808948
LOC_Os07g03377	LOC_Os12g40510	9.73504481
LOC_Os07g03730	LOC_Os08g43190	8.95029521
LOC_Os07g03600	LOC_Os11g08470	9.61092866
LOC_Os07g03710	LOC_Os08g25734	9.37579292
LOC_Os07g03730	LOC_Os12g40510	9.45040607
LOC_Os07g03499	LOC_Os09g27750	9.7130089
LOC_Os07g05400	LOC_Os11g26860	10.4915455

InteractorA	InteractorB	Z score
LOC_Os07g07320	LOC_Os12g22030	9.18525886
LOC_Os07g05820	LOC_Os08g14770	8.88433456
LOC_Os07g06970	LOC_Os11g37550	9.13084143
LOC_Os07g05160	LOC_Os10g01540	9.90792786
LOC_Os07g05400	LOC_Os07g28280	9.72011805
LOC_Os07g05180	LOC_Os07g07240	10.6000894
LOC_Os07g06410	LOC_Os10g08550	9.28023112
LOC_Os07g06440	LOC_Os11g25700	11.4504799
LOC_Os07g05940	LOC_Os07g08170	9.00814267
LOC_Os07g06970	LOC_Os07g07550	8.86664049
LOC_Os07g05400	LOC_Os09g34960	9.03580158
LOC_Os07g06970	LOC_Os12g08280	10.5439616
LOC_Os07g05160	LOC_Os08g33370	8.83200338
LOC_Os07g07320	LOC_Os10g21250	9.99526
LOC_Os07g05150	LOC_Os07g44590	9.19950735
LOC_Os07g05180	LOC_Os10g21192	9.3441342
LOC_Os07g06970	LOC_Os11g40140	10.0717909
LOC_Os07g05820	LOC_Os09g04050	9.43709962
LOC_Os07g05940	LOC_Os11g32610	10.8371363
LOC_Os07g05180	LOC_Os10g31000	8.96491641
LOC_Os07g07320	LOC_Os09g23560	10.2387173
LOC_Os07g07320	LOC_Os07g26540	9.55115083
LOC_Os07g05800	LOC_Os09g20260	10.4504573
LOC_Os07g06440	LOC_Os07g30170	9.18495221
LOC_Os07g05800	LOC_Os09g24990	9.38975016
LOC_Os07g07060	LOC_Os08g03040	10.9961623
LOC_Os07g05160	LOC_Os10g38610	11.5792677
LOC_Os07g07060	LOC_Os08g41880	9.13598022
LOC_Os07g05800	LOC_Os08g31060	9.62465934
LOC_Os07g06970	LOC_Os10g26010	9.14463026
LOC_Os07g05160	LOC_Os07g38860	9.53251281
LOC_Os07g05180	LOC_Os09g25150	9.587207
LOC_Os07g05940	LOC_Os12g02980	9.00565422
LOC_Os07g05400	LOC_Os12g39630	9.13171482
LOC_Os07g06970	LOC_Os09g09520	9.18956063
LOC_Os07g05940	LOC_Os10g28120	9.32276131
LOC_Os07g05160	LOC_Os07g11440	9.98074789
LOC_Os07g05400	LOC_Os08g43170	9.02434103
LOC_Os07g07060	LOC_Os08g28730	10.2563891
LOC_Os07g07240	LOC_Os09g36710	8.93591408
LOC_Os07g05150	LOC_Os08g23110	10.7038639
LOC_Os07g06440	LOC_Os10g41510	9.55249032
LOC_Os07g07240	LOC_Os11g47600	8.96132558

InteractorA	InteractorB	Z score
LOC_Os07g05800	LOC_Os12g34380	9.21486023
LOC_Os07g07060	LOC_Os10g21342	9.00713107
LOC_Os07g05940	LOC_Os09g28420	9.98301171
LOC_Os07g05160	LOC_Os10g21344	9.11210747
LOC_Os07g05820	LOC_Os07g06410	10.3684914
LOC_Os07g06440	LOC_Os11g08460	9.12488849
LOC_Os07g05150	LOC_Os10g01540	11.1856617
LOC_Os07g05940	LOC_Os07g08840	10.0920197
LOC_Os07g07060	LOC_Os08g15276	9.00713107
LOC_Os07g05180	LOC_Os10g07616	10.0551521
LOC_Os07g05940	LOC_Os12g16290	10.176864
LOC_Os07g05180	LOC_Os07g13980	9.93873032
LOC_Os07g05180	LOC_Os08g16910	9.7027564
LOC_Os07g07060	LOC_Os07g31770	8.94033712
LOC_Os07g05820	LOC_Os08g02700	11.0845897
LOC_Os07g05940	LOC_Os08g17680	9.16454259
LOC_Os07g05180	LOC_Os07g30990	9.5949036
LOC_Os07g07320	LOC_Os09g31502	9.47809506
LOC_Os07g05400	LOC_Os07g23470	8.91520875
LOC_Os07g05940	LOC_Os09g32640	9.33224844
LOC_Os07g07320	LOC_Os12g16220	9.49295303
LOC_Os07g05150	LOC_Os11g08460	8.86761265
LOC_Os07g06440	LOC_Os12g34450	9.31403877
LOC_Os07g05160	LOC_Os07g05180	9.43018489
LOC_Os07g05940	LOC_Os11g39540	8.881157
LOC_Os07g05150	LOC_Os09g26880	9.83980017
LOC_Os07g07240	LOC_Os07g43470	8.89382546
LOC_Os07g05150	LOC_Os11g41130	10.2909225
LOC_Os07g07060	LOC_Os12g12560	9.13633541
LOC_Os07g05400	LOC_Os07g44370	9.78165009
LOC_Os07g05940	LOC_Os09g31502	11.4388099
LOC_Os07g05150	LOC_Os11g47600	9.34031217
LOC_Os07g05180	LOC_Os08g40930	10.5296572
LOC_Os07g06970	LOC_Os07g31750	9.04168402
LOC_Os07g05400	LOC_Os07g07709	10.762019
LOC_Os07g06970	LOC_Os07g26690	8.97278624
LOC_Os07g05400	LOC_Os08g44530	9.82177874
LOC_Os07g05940	LOC_Os12g44350	9.09869872
LOC_Os07g05800	LOC_Os10g42720	8.83422153
LOC_Os07g05940	LOC_Os10g39680	9.22593693
LOC_Os07g07240	LOC_Os08g10010	9.14263168
LOC_Os07g07320	LOC_Os11g20790	10.2599722
LOC_Os07g05180	LOC_Os12g12560	9.86178524

InteractorA	InteractorB	Z score
LOC_Os07g05580	LOC_Os09g28770	8.98791477
LOC_Os07g05180	LOC_Os08g04180	8.80026844
LOC_Os07g05800	LOC_Os10g27050	10.0214299
LOC_Os07g05150	LOC_Os12g07830	10.0323839
LOC_Os07g07060	LOC_Os10g40600	9.31535999
LOC_Os07g05400	LOC_Os09g36800	9.29717271
LOC_Os07g05150	LOC_Os12g38760	9.15976248
LOC_Os07g05150	LOC_Os10g40710	9.81954716
LOC_Os07g05150	LOC_Os10g34760	10.1705674
LOC_Os07g06410	LOC_Os10g42280	8.96209471
LOC_Os07g06440	LOC_Os08g44340	9.51509577
LOC_Os07g06970	LOC_Os07g36130	8.87874488
LOC_Os07g06410	LOC_Os11g25700	9.02604772
LOC_Os07g05180	LOC_Os08g34790	9.1118708
LOC_Os07g05820	LOC_Os08g41830	9.04542877
LOC_Os07g07240	LOC_Os10g21326	9.4357324
LOC_Os07g07320	LOC_Os10g30200	9.92386428
LOC_Os07g05150	LOC_Os10g40700	11.8246902
LOC_Os07g05150	LOC_Os08g20420	10.3666915
LOC_Os07g05150	LOC_Os09g34970	9.83782546
LOC_Os07g06970	LOC_Os07g38540	9.26382001
LOC_Os07g06410	LOC_Os09g23300	10.2209568
LOC_Os07g05940	LOC_Os12g31370	9.20205188
LOC_Os07g05400	LOC_Os09g32640	8.8935813
LOC_Os07g05400	LOC_Os07g23850	9.15364996
LOC_Os07g05160	LOC_Os07g35940	9.16367243
LOC_Os07g05940	LOC_Os08g17500	9.46920635
LOC_Os07g06970	LOC_Os07g35940	8.80771947
LOC_Os07g07060	LOC_Os07g29440	9.64493526
LOC_Os07g05160	LOC_Os08g40930	8.87396359
LOC_Os07g05400	LOC_Os12g12590	10.3043062
LOC_Os07g07240	LOC_Os12g05410	8.86479875
LOC_Os07g05400	LOC_Os08g09250	8.83224774
LOC_Os07g06970	LOC_Os07g25150	8.935419
LOC_Os07g05580	LOC_Os07g27790	8.98239877
LOC_Os07g06410	LOC_Os09g31490	10.6662985
LOC_Os07g05940	LOC_Os10g10434	9.18256869
LOC_Os07g07320	LOC_Os10g28350	9.0737513
LOC_Os07g05150	LOC_Os10g22070	9.54609185
LOC_Os07g06440	LOC_Os12g39630	9.03233688
LOC_Os07g05800	LOC_Os10g38640	9.39220244
LOC_Os07g07320	LOC_Os10g38229	10.5013412
LOC_Os07g05940	LOC_Os07g48050	9.67777471

InteractorA	InteractorB	Z score
LOC_Os07g06440	LOC_Os08g33710	12.892426
LOC_Os07g05160	LOC_Os07g34190	9.18461853
LOC_Os07g06970	LOC_Os10g32970	9.99323065
LOC_Os07g09890	LOC_Os11g38959	9.4325292
LOC_Os07g07709	LOC_Os08g31060	9.0691201
LOC_Os07g08660	LOC_Os10g25130	9.08986224
LOC_Os07g07719	LOC_Os07g48050	9.06731141
LOC_Os07g08660	LOC_Os07g46630	8.8373866
LOC_Os07g07719	LOC_Os09g38020	8.80395293
LOC_Os07g08840	LOC_Os10g08670	9.36211733
LOC_Os07g08500	LOC_Os07g14590	9.55190718
LOC_Os07g07470	LOC_Os08g09250	9.45614504
LOC_Os07g08170	LOC_Os12g07720	9.66230279
LOC_Os07g07550	LOC_Os09g12570	9.07885613
LOC_Os07g08170	LOC_Os10g26050	9.18852132
LOC_Os07g07709	LOC_Os07g36500	10.0431544
LOC_Os07g08660	LOC_Os09g25370	9.96454024
LOC_Os07g09340	LOC_Os10g36650	9.97419406
LOC_Os07g07709	LOC_Os08g17784	8.93684646
LOC_Os07g08880	LOC_Os12g03816	10.3221164
LOC_Os07g08030	LOC_Os07g10660	9.20511922
LOC_Os07g09890	LOC_Os07g44370	8.8219816
LOC_Os07g07719	LOC_Os10g39410	8.80395171
LOC_Os07g07550	LOC_Os07g44450	11.193832
LOC_Os07g07770	LOC_Os07g41050	9.02013473
LOC_Os07g09340	LOC_Os11g25260	9.32697895
LOC_Os07g08170	LOC_Os10g21192	8.85393581
LOC_Os07g07719	LOC_Os08g20270	9.13685511
LOC_Os07g10590	LOC_Os08g28800	8.95134119
LOC_Os07g07709	LOC_Os12g44350	10.5146433
LOC_Os07g07709	LOC_Os10g38950	9.50407043
LOC_Os07g07550	LOC_Os07g08170	9.67765522
LOC_Os07g08170	LOC_Os07g43670	8.96016282
LOC_Os07g07770	LOC_Os07g46630	9.62634875
LOC_Os07g08660	LOC_Os11g37550	9.16476716
LOC_Os07g09890	LOC_Os10g30200	10.5887297
LOC_Os07g08170	LOC_Os10g38610	9.22331818
LOC_Os07g07719	LOC_Os12g10600	9.22775446
LOC_Os07g07719	LOC_Os12g10720	9.27478425
LOC_Os07g08170	LOC_Os10g08670	8.84308895
LOC_Os07g07550	LOC_Os09g35800	8.89696238
LOC_Os07g07719	LOC_Os08g44340	9.16156622
LOC_Os07g08030	LOC_Os08g35420	9.25455127

InteractorA	InteractorB	Z score
LOC_Os07g07719	LOC_Os11g26860	9.78586725
LOC_Os07g07550	LOC_Os09g30418	8.91042308
LOC_Os07g07550	LOC_Os10g38630	8.82010193
LOC_Os07g08170	LOC_Os12g04980	8.91781228
LOC_Os07g07550	LOC_Os07g22950	9.08489341
LOC_Os07g07719	LOC_Os07g36500	8.80394968
LOC_Os07g07470	LOC_Os10g28080	9.22519785
LOC_Os07g07550	LOC_Os08g03290	9.49862102
LOC_Os07g07719	LOC_Os09g20284	9.89060847
LOC_Os07g09890	LOC_Os11g10480	8.81312865
LOC_Os07g08880	LOC_Os10g08670	8.97484111
LOC_Os07g07550	LOC_Os09g07830	10.1777348
LOC_Os07g07550	LOC_Os12g43630	9.4434071
LOC_Os07g07550	LOC_Os10g38590	9.01720952
LOC_Os07g07709	LOC_Os09g38020	10.0431511
LOC_Os07g09890	LOC_Os12g12470	8.8767017
LOC_Os07g07770	LOC_Os12g07820	9.10943812
LOC_Os07g07719	LOC_Os08g33710	9.06634949
LOC_Os07g07709	LOC_Os09g26340	10.0431544
LOC_Os07g09890	LOC_Os10g40700	9.01694304
LOC_Os07g08660	LOC_Os12g01922	9.08150208
LOC_Os07g07770	LOC_Os08g34790	9.40428928
LOC_Os07g07770	LOC_Os08g40140	8.83691375
LOC_Os07g07719	LOC_Os10g31000	11.7930393
LOC_Os07g07770	LOC_Os10g38340	8.98462076
LOC_Os07g08500	LOC_Os07g49120	9.8762769
LOC_Os07g08500	LOC_Os11g05290	9.17315914
LOC_Os07g08840	LOC_Os09g12570	8.99135213
LOC_Os07g08500	LOC_Os09g04680	9.30575638
LOC_Os07g10590	LOC_Os07g12730	9.2604595
LOC_Os07g09890	LOC_Os07g44550	9.64510687
LOC_Os07g07550	LOC_Os08g44350	8.86496693
LOC_Os07g08840	LOC_Os07g42960	9.87686443
LOC_Os07g09680	LOC_Os10g01570	10.0804037
LOC_Os07g07709	LOC_Os10g11260	8.82089326
LOC_Os07g07550	LOC_Os10g38740	9.00977256
LOC_Os07g09890	LOC_Os10g39170	9.24217918
LOC_Os07g07719	LOC_Os09g26340	8.80395293
LOC_Os07g09890	LOC_Os11g37960	9.97333734
LOC_Os07g07709	LOC_Os11g08940	9.15809382
LOC_Os07g09890	LOC_Os07g36130	10.3593127
LOC_Os07g08170	LOC_Os12g42876	10.4597342
LOC_Os07g07770	LOC_Os10g01080	8.80337654

InteractorA	InteractorB	Z score
LOC_Os07g07550	LOC_Os11g14220	10.0200589
LOC_Os07g08170	LOC_Os10g40730	11.2190126
LOC_Os07g08500	LOC_Os08g14760	9.28036732
LOC_Os07g07709	LOC_Os10g39410	10.0431544
LOC_Os07g07719	LOC_Os08g04460	10.5463495
LOC_Os07g07709	LOC_Os10g36650	9.81574129
LOC_Os07g07550	LOC_Os10g38730	9.22702387
LOC_Os07g10720	LOC_Os08g37790	9.26056707
LOC_Os07g20544	LOC_Os11g47600	10.1227996
LOC_Os07g12200	LOC_Os10g26600	9.02529641
LOC_Os07g20544	LOC_Os11g39540	9.17341033
LOC_Os07g17010	LOC_Os08g37790	9.38077402
LOC_Os07g14590	LOC_Os08g40930	9.37405608
LOC_Os07g20544	LOC_Os09g08910	9.14665918
LOC_Os07g13980	LOC_Os08g15030	9.29921945
LOC_Os07g12730	LOC_Os12g10730	8.95576306
LOC_Os07g10720	LOC_Os08g09200	9.22941675
LOC_Os07g14590	LOC_Os10g29620	11.2410876
LOC_Os07g10720	LOC_Os08g28800	8.8860189
LOC_Os07g17010	LOC_Os12g38760	9.18833509
LOC_Os07g19040	LOC_Os08g34280	8.95287913
LOC_Os07g20544	LOC_Os10g21240	9.18230813
LOC_Os07g17010	LOC_Os07g34260	12.8093354
LOC_Os07g19040	LOC_Os08g09950	8.88401221
LOC_Os07g20544	LOC_Os09g34214	8.98706558
LOC_Os07g19040	LOC_Os10g26050	9.45205582
LOC_Os07g17010	LOC_Os12g12514	9.96358115
LOC_Os07g22600	LOC_Os09g24530	9.43028585
LOC_Os07g17010	LOC_Os11g32580	11.1272995
LOC_Os07g17010	LOC_Os11g32650	15.6743502
LOC_Os07g16970	LOC_Os08g40140	8.93345389
LOC_Os07g14590	LOC_Os08g29170	11.1183025
LOC_Os07g14590	LOC_Os08g44530	9.46927983
LOC_Os07g14590	LOC_Os11g43960	9.16693575
LOC_Os07g10720	LOC_Os09g28420	12.4606762
LOC_Os07g11440	LOC_Os10g29470	9.00642865
LOC_Os07g20544	LOC_Os08g44340	8.92994815
LOC_Os07g17010	LOC_Os08g28800	9.81969078
LOC_Os07g20544	LOC_Os09g15400	10.8224036
LOC_Os07g19040	LOC_Os10g30840	9.96760615
LOC_Os07g13980	LOC_Os07g42960	9.04061364
LOC_Os07g20544	LOC_Os07g35880	8.83077226
LOC_Os07g11440	LOC_Os11g08445	9.56796678

InteractorA	InteractorB	Z score
LOC_Os07g22498	LOC_Os11g33270	9.01425107
LOC_Os07g17010	LOC_Os08g36910	10.7305406
LOC_Os07g17010	LOC_Os08g09950	10.564966
LOC_Os07g17010	LOC_Os10g25130	11.7875472
LOC_Os07g20544	LOC_Os08g09940	10.1802131
LOC_Os07g10660	LOC_Os11g40150	9.54309884
LOC_Os07g20544	LOC_Os08g35420	9.27633969
LOC_Os07g19040	LOC_Os11g05570	9.42547965
LOC_Os07g11440	LOC_Os11g32580	15.3163313
LOC_Os07g12730	LOC_Os08g43190	9.30945314
LOC_Os07g20544	LOC_Os07g48780	10.1056838
LOC_Os07g22600	LOC_Os10g39680	8.81693278
LOC_Os07g11440	LOC_Os10g08580	9.37960464
LOC_Os07g20544	LOC_Os12g23170	9.20885435
LOC_Os07g22600	LOC_Os10g32870	9.22727488
LOC_Os07g12200	LOC_Os07g46310	9.13889948
LOC_Os07g17010	LOC_Os11g32610	12.1875781
LOC_Os07g22650	LOC_Os07g47490	9.21674719
LOC_Os07g12200	LOC_Os08g04540	10.2079721
LOC_Os07g17010	LOC_Os10g09860	12.1230036
LOC_Os07g20544	LOC_Os09g31120	9.33164592
LOC_Os07g10720	LOC_Os11g08340	9.23490887
LOC_Os07g10590	LOC_Os09g16910	9.1129994
LOC_Os07g14590	LOC_Os10g39840	10.5649482
LOC_Os07g17010	LOC_Os09g32640	9.93942751
LOC_Os07g19040	LOC_Os11g03980	9.54248227
LOC_Os07g20544	LOC_Os09g15320	11.1500686
LOC_Os07g22498	LOC_Os07g23730	8.91809993
LOC_Os07g11440	LOC_Os07g34260	9.50287204
LOC_Os07g10720	LOC_Os09g08120	8.90365239
LOC_Os07g20544	LOC_Os11g32650	10.2634627
LOC_Os07g17010	LOC_Os07g34190	16.9182596
LOC_Os07g12730	LOC_Os09g14670	9.12410955
LOC_Os07g13980	LOC_Os07g26540	10.2901969
LOC_Os07g10720	LOC_Os09g28400	9.08513594
LOC_Os07g17010	LOC_Os08g25570	9.37114133
LOC_Os07g20544	LOC_Os11g10480	10.1998498
LOC_Os07g10720	LOC_Os08g15292	9.3126901
LOC_Os07g16970	LOC_Os10g05069	9.35904522
LOC_Os07g14590	LOC_Os12g13380	9.14122099
LOC_Os07g11440	LOC_Os11g47760	10.3665968
LOC_Os07g16970	LOC_Os10g26390	9.08776887
LOC_Os07g14590	LOC_Os07g43820	9.03132319

InteractorA	InteractorB	Z score
LOC_Os07g16970	LOC_Os11g38959	8.93436175
LOC_Os07g14590	LOC_Os10g39880	9.17478069
LOC_Os07g10660	LOC_Os09g39500	8.81366605
LOC_Os07g16970	LOC_Os10g02040	9.91490993
LOC_Os07g10660	LOC_Os08g31060	8.97237664
LOC_Os07g20544	LOC_Os12g16250	9.56007954
LOC_Os07g13980	LOC_Os09g36670	9.21137882
LOC_Os07g16970	LOC_Os11g08330	8.86531141
LOC_Os07g11440	LOC_Os08g35440	9.03512418
LOC_Os07g11440	LOC_Os07g17010	11.8059456
LOC_Os07g10660	LOC_Os10g28320	8.84784138
LOC_Os07g12730	LOC_Os07g35940	9.14736318
LOC_Os07g13980	LOC_Os10g32550	9.3332882
LOC_Os07g22600	LOC_Os08g37800	9.53700445
LOC_Os07g11440	LOC_Os07g31770	12.1891553
LOC_Os07g11440	LOC_Os10g08620	13.4041814
LOC_Os07g22650	LOC_Os11g25260	9.48385455
LOC_Os07g13980	LOC_Os12g10560	9.15348918
LOC_Os07g23730	LOC_Os09g23530	8.93106118
LOC_Os07g26900	LOC_Os10g31000	9.49758185
LOC_Os07g22930	LOC_Os10g36650	9.12034193
LOC_Os07g25590	LOC_Os09g39500	9.24351951
LOC_Os07g26150	LOC_Os08g44270	9.45231591
LOC_Os07g26900	LOC_Os07g35940	10.6733975
LOC_Os07g25150	LOC_Os08g04560	8.83950848
LOC_Os07g22950	LOC_Os11g10520	8.89273974
LOC_Os07g25150	LOC_Os10g40720	8.83136205
LOC_Os07g23850	LOC_Os12g17910	8.84195789
LOC_Os07g26540	LOC_Os07g38860	9.24761828
LOC_Os07g25024	LOC_Os08g41880	9.41889211
LOC_Os07g25590	LOC_Os10g25130	9.03278053
LOC_Os07g23470	LOC_Os11g47570	11.5935052
LOC_Os07g25590	LOC_Os10g21250	8.95770136
LOC_Os07g23730	LOC_Os12g10730	9.20164999
LOC_Os07g26900	LOC_Os10g38600	8.88060166
LOC_Os07g26690	LOC_Os12g12580	9.59663735
LOC_Os07g23730	LOC_Os08g34170	8.88403258
LOC_Os07g22950	LOC_Os07g48060	9.01419285
LOC_Os07g26640	LOC_Os12g17910	8.81342545
LOC_Os07g26640	LOC_Os11g14910	9.77806261
LOC_Os07g25150	LOC_Os10g38470	9.67157408
LOC_Os07g22930	LOC_Os12g08280	10.8273736
LOC_Os07g23470	LOC_Os07g48010	10.6849405

InteractorA	InteractorB	Z score
LOC_Os07g23470	LOC_Os09g12570	8.87881966
LOC_Os07g22650	LOC_Os12g44030	8.96220971
LOC_Os07g26540	LOC_Os10g38730	9.33041658
LOC_Os07g22650	LOC_Os12g02980	10.6416789
LOC_Os07g25024	LOC_Os07g40290	9.68350894
LOC_Os07g26150	LOC_Os07g35880	9.09080976
LOC_Os07g25024	LOC_Os11g25330	9.92153133
LOC_Os07g26900	LOC_Os08g14760	9.46145966
LOC_Os07g26660	LOC_Os09g31506	9.11099451
LOC_Os07g26900	LOC_Os08g03040	8.9073934
LOC_Os07g26540	LOC_Os10g41410	9.09276411
LOC_Os07g26630	LOC_Os08g16910	11.0335859
LOC_Os07g25024	LOC_Os07g48040	9.82014011
LOC_Os07g26660	LOC_Os12g02370	10.1601112
LOC_Os07g23730	LOC_Os11g10520	10.9336889
LOC_Os07g23850	LOC_Os12g40510	9.24642197
LOC_Os07g26640	LOC_Os11g03230	8.85312879
LOC_Os07g26900	LOC_Os09g32800	9.77079222
LOC_Os07g26540	LOC_Os07g41050	9.67994083
LOC_Os07g26690	LOC_Os07g42600	9.34465598
LOC_Os07g26630	LOC_Os08g06100	10.2664
LOC_Os07g23470	LOC_Os09g39810	9.24119831
LOC_Os07g25150	LOC_Os09g07830	9.13189996
LOC_Os07g22930	LOC_Os08g34280	9.10024212
LOC_Os07g23730	LOC_Os08g14770	9.04019902
LOC_Os07g25024	LOC_Os10g31000	9.00579729
LOC_Os07g23730	LOC_Os10g21406	8.91809993
LOC_Os07g26540	LOC_Os07g47290	9.39009112
LOC_Os07g23470	LOC_Os09g35800	10.4026244
LOC_Os07g25024	LOC_Os09g38030	9.58499466
LOC_Os07g23850	LOC_Os07g38970	8.81950472
LOC_Os07g26900	LOC_Os08g44350	9.21823104
LOC_Os07g26690	LOC_Os07g34140	9.12954956
LOC_Os07g25590	LOC_Os08g04540	24.6631453
LOC_Os07g25024	LOC_Os08g02410	9.39002599
LOC_Os07g26690	LOC_Os12g16240	9.66395599
LOC_Os07g26540	LOC_Os07g36130	10.8537595
LOC_Os07g26150	LOC_Os07g48880	8.89160723
LOC_Os07g23730	LOC_Os12g06620	9.91468832
LOC_Os07g23470	LOC_Os11g33240	9.75866612
LOC_Os07g25590	LOC_Os10g26110	17.7633473
LOC_Os07g22930	LOC_Os10g40720	8.81206444
LOC_Os07g23470	LOC_Os10g01540	9.00383474

InteractorA	InteractorB	Z score
LOC_Os07g23470	LOC_Os09g04050	9.25989827
LOC_Os07g25024	LOC_Os12g21798	9.12606016
LOC_Os07g26150	LOC_Os11g25700	9.06776229
LOC_Os07g23470	LOC_Os08g04560	9.27571824
LOC_Os07g25024	LOC_Os09g08910	9.21414864
LOC_Os07g26630	LOC_Os10g11810	9.17049208
LOC_Os07g26660	LOC_Os09g37100	8.81477745
LOC_Os07g26540	LOC_Os08g40140	9.30362985
LOC_Os07g23730	LOC_Os09g36710	9.55715926
LOC_Os07g23470	LOC_Os09g04680	9.14402872
LOC_Os07g26150	LOC_Os11g08340	9.75407908
LOC_Os07g26640	LOC_Os09g07510	9.16434063
LOC_Os07g26540	LOC_Os10g31950	9.12336967
LOC_Os07g25590	LOC_Os11g32770	8.94550491
LOC_Os07g23730	LOC_Os08g28190	9.17978924
LOC_Os07g26900	LOC_Os08g40930	9.21174433
LOC_Os07g26540	LOC_Os08g44370	9.31673452
LOC_Os07g25150	LOC_Os11g08460	11.4004393
LOC_Os07g26540	LOC_Os11g32650	9.39543489
LOC_Os07g25024	LOC_Os07g48050	10.5204067
LOC_Os07g25024	LOC_Os08g06550	10.6531712
LOC_Os07g25590	LOC_Os10g32550	9.93842793
LOC_Os07g22930	LOC_Os10g41510	9.0466274
LOC_Os07g23470	LOC_Os10g40090	11.44646
LOC_Os07g26540	LOC_Os11g43360	10.7932992
LOC_Os07g23470	LOC_Os09g23540	10.1778299
LOC_Os07g25150	LOC_Os12g06660	9.03059771
LOC_Os07g22930	LOC_Os08g38920	9.2045078
LOC_Os07g22950	LOC_Os10g25930	9.4593708
LOC_Os07g23470	LOC_Os10g40730	8.89287558
LOC_Os07g26540	LOC_Os10g26050	9.8954505
LOC_Os07g27790	LOC_Os10g39590	9.36588771
LOC_Os07g28280	LOC_Os09g14670	8.96764748
LOC_Os07g30990	LOC_Os07g48880	9.09553649
LOC_Os07g28480	LOC_Os09g36830	8.81295803
LOC_Os07g30090	LOC_Os08g32850	11.7229573
LOC_Os07g27790	LOC_Os12g26290	9.30790821
LOC_Os07g30170	LOC_Os10g10434	9.64274686
LOC_Os07g31770	LOC_Os11g33270	9.160315
LOC_Os07g29440	LOC_Os10g28080	8.8732256
LOC_Os07g28480	LOC_Os08g31060	8.84890146
LOC_Os07g30170	LOC_Os08g04540	8.96190822
LOC_Os07g29440	LOC_Os10g28360	9.02767089

InteractorA	InteractorB	Z score
LOC_Os07g29440	LOC_Os12g25710	9.35402781
LOC_Os07g31830	LOC_Os09g08120	9.16911583
LOC_Os07g31830	LOC_Os09g20090	8.90152562
LOC_Os07g28480	LOC_Os08g37800	9.84979568
LOC_Os07g30200	LOC_Os09g08120	9.76796496
LOC_Os07g29750	LOC_Os10g28050	12.6503304
LOC_Os07g29440	LOC_Os11g26910	9.11578774
LOC_Os07g28480	LOC_Os08g14770	10.0580788
LOC_Os07g30970	LOC_Os12g13380	11.0269863
LOC_Os07g29750	LOC_Os07g34140	8.87263053
LOC_Os07g28480	LOC_Os10g27050	9.75748282
LOC_Os07g31270	LOC_Os10g39120	9.78153003
LOC_Os07g30970	LOC_Os09g34214	9.87572726
LOC_Os07g30170	LOC_Os09g09230	9.82966896
LOC_Os07g30170	LOC_Os11g05570	9.82282632
LOC_Os07g29750	LOC_Os09g24990	9.01002527
LOC_Os07g30170	LOC_Os09g38620	8.87633225
LOC_Os07g29750	LOC_Os08g40740	10.4028685
LOC_Os07g30990	LOC_Os10g35110	9.319129
LOC_Os07g30170	LOC_Os11g31530	10.6232679
LOC_Os07g31750	LOC_Os08g42540	9.16093592
LOC_Os07g28280	LOC_Os08g06060	11.6649805
LOC_Os07g28480	LOC_Os07g48780	9.43752208
LOC_Os07g29440	LOC_Os09g39400	9.53240221
LOC_Os07g29750	LOC_Os11g34570	9.97181971
LOC_Os07g31750	LOC_Os08g34210	9.81044498
LOC_Os07g30200	LOC_Os10g40710	9.50357616
LOC_Os07g30170	LOC_Os07g38910	9.23104715
LOC_Os07g30200	LOC_Os08g08500	9.70034073
LOC_Os07g29750	LOC_Os08g41830	9.96066922
LOC_Os07g26900	LOC_Os12g38760	9.66407775
LOC_Os07g31270	LOC_Os10g29620	9.33469264
LOC_Os07g28480	LOC_Os08g38300	9.07711422
LOC_Os07g31270	LOC_Os10g08670	9.08445116
LOC_Os07g31270	LOC_Os10g40090	9.55834174
LOC_Os07g30990	LOC_Os09g08910	9.37671858
LOC_Os07g30970	LOC_Os09g31430	10.0435113
LOC_Os07g29440	LOC_Os10g23900	9.02072863
LOC_Os07g30200	LOC_Os11g37550	9.04742218
LOC_Os07g30990	LOC_Os09g28460	9.52404383
LOC_Os07g26900	LOC_Os12g38750	9.79281996
LOC_Os07g28280	LOC_Os09g31430	9.92836528
LOC_Os07g31270	LOC_Os08g02400	9.58586559

InteractorA	InteractorB	Z score
LOC_Os07g31830	LOC_Os08g34280	9.35404784
LOC_Os07g31750	LOC_Os07g40580	9.07053772
LOC_Os07g27790	LOC_Os08g06060	9.57353188
LOC_Os07g28480	LOC_Os10g38140	8.94631264
LOC_Os07g30970	LOC_Os09g25390	9.69251299
LOC_Os07g30970	LOC_Os07g38430	8.96636034
LOC_Os07g27790	LOC_Os11g37890	8.9036241
LOC_Os07g31750	LOC_Os07g39870	9.38030522
LOC_Os07g31750	LOC_Os07g49220	9.1620891
LOC_Os07g30990	LOC_Os08g09210	9.17450961
LOC_Os07g29440	LOC_Os08g02340	8.90598123
LOC_Os07g30990	LOC_Os07g38890	9.22681277
LOC_Os07g31750	LOC_Os10g38600	9.31187515
LOC_Os07g29750	LOC_Os12g40830	8.91649677
LOC_Os07g30990	LOC_Os08g09370	9.34356763
LOC_Os07g29750	LOC_Os11g41610	8.88708428
LOC_Os07g29750	LOC_Os07g43670	9.61112399
LOC_Os07g31830	LOC_Os10g38580	8.92627332
LOC_Os07g30200	LOC_Os12g07720	9.41738083
LOC_Os07g27790	LOC_Os10g40090	8.92045229
LOC_Os07g31270	LOC_Os12g02370	9.13606529
LOC_Os07g28280	LOC_Os12g22680	9.31608162
LOC_Os07g30170	LOC_Os09g36830	8.91375612
LOC_Os07g31830	LOC_Os09g31120	9.07711394
LOC_Os07g30200	LOC_Os07g39870	8.88593297
LOC_Os07g29750	LOC_Os10g39120	8.83952494
LOC_Os07g31830	LOC_Os11g03290	11.1915472
LOC_Os07g28280	LOC_Os08g15292	9.09312632
LOC_Os07g30170	LOC_Os12g43440	8.8764159
LOC_Os07g30640	LOC_Os12g12470	9.49094549
LOC_Os07g28480	LOC_Os09g39500	9.97362247
LOC_Os07g30970	LOC_Os09g24530	9.13352977
LOC_Os07g34190	LOC_Os12g25690	9.04851991
LOC_Os07g32800	LOC_Os07g35940	10.0477593
LOC_Os07g34190	LOC_Os08g33710	8.95603579
LOC_Os07g35880	LOC_Os11g25330	9.0675725
LOC_Os07g34140	LOC_Os09g31430	9.11184691
LOC_Os07g32800	LOC_Os08g36320	10.3537112
LOC_Os07g34140	LOC_Os12g41110	10.2975471
LOC_Os07g34520	LOC_Os07g38970	8.93963862
LOC_Os07g34520	LOC_Os08g44810	8.8545385
LOC_Os07g35880	LOC_Os10g30580	9.5523293
LOC_Os07g36500	LOC_Os11g10480	8.92560379

InteractorA	InteractorB	Z score
LOC_Os07g35880	LOC_Os12g16290	8.89516174
LOC_Os07g35940	LOC_Os07g48020	9.27315365
LOC_Os07g36130	LOC_Os08g38300	9.73055993
LOC_Os07g36500	LOC_Os12g22680	9.95584485
LOC_Os07g32800	LOC_Os07g42600	10.8943717
LOC_Os07g34589	LOC_Os11g04880	9.31929732
LOC_Os07g34580	LOC_Os09g23560	9.76385184
LOC_Os07g35880	LOC_Os10g21342	8.83894616
LOC_Os07g34140	LOC_Os09g07830	11.3442673
LOC_Os07g34589	LOC_Os10g38580	8.8167283
LOC_Os07g35940	LOC_Os09g16910	9.12132029
LOC_Os07g34190	LOC_Os10g08670	8.90838964
LOC_Os07g34190	LOC_Os07g48050	9.22556772
LOC_Os07g36140	LOC_Os11g08470	8.86076184
LOC_Os07g36190	LOC_Os08g14570	9.03163261
LOC_Os07g35940	LOC_Os07g38910	9.47552128
LOC_Os07g36130	LOC_Os08g38900	9.16617
LOC_Os07g37550	LOC_Os08g20270	9.71564754
LOC_Os07g37320	LOC_Os07g46310	11.1237166
LOC_Os07g34260	LOC_Os08g08500	10.5953377
LOC_Os07g34580	LOC_Os10g01540	10.0810189
LOC_Os07g34580	LOC_Os11g31620	10.363833
LOC_Os07g34580	LOC_Os07g48880	9.655465
LOC_Os07g34140	LOC_Os08g44530	8.84990091
LOC_Os07g34520	LOC_Os10g32970	9.05629267
LOC_Os07g34260	LOC_Os09g37100	10.3487215
LOC_Os07g34589	LOC_Os10g38660	9.02387537
LOC_Os07g36190	LOC_Os10g41410	9.62503322
LOC_Os07g34190	LOC_Os08g41880	9.38501615
LOC_Os07g34589	LOC_Os07g47290	9.03106631
LOC_Os07g34580	LOC_Os10g36650	9.59314309
LOC_Os07g37550	LOC_Os08g04560	9.56580612
LOC_Os07g36500	LOC_Os10g01540	9.10660661
LOC_Os07g36190	LOC_Os09g27420	9.55206345
LOC_Os07g36140	LOC_Os09g24530	9.37582893
LOC_Os07g34589	LOC_Os07g44450	9.11147922
LOC_Os07g34260	LOC_Os12g41110	9.12134368
LOC_Os07g34190	LOC_Os07g35880	8.8644393
LOC_Os07g34190	LOC_Os08g15292	8.97781518
LOC_Os07g34589	LOC_Os10g38640	9.08867345
LOC_Os07g35940	LOC_Os09g10200	9.55447508
LOC_Os07g37320	LOC_Os08g33370	8.92475643
LOC_Os07g34580	LOC_Os09g31490	9.11100401

InteractorA	InteractorB	Z score
LOC_Os07g37320	LOC_Os12g16240	10.4461518
LOC_Os07g36190	LOC_Os08g08500	8.80626709
LOC_Os07g35880	LOC_Os08g36900	9.41200758
LOC_Os07g36500	LOC_Os11g10510	8.86156807
LOC_Os07g35880	LOC_Os08g15276	8.83894616
LOC_Os07g34589	LOC_Os07g38860	11.9685787
LOC_Os07g34140	LOC_Os07g42600	9.08570446
LOC_Os07g34589	LOC_Os09g30360	10.2842853
LOC_Os07g36500	LOC_Os10g39170	9.48057701
LOC_Os07g37550	LOC_Os08g34290	9.78148437
LOC_Os07g34190	LOC_Os08g44810	9.53920419
LOC_Os07g34589	LOC_Os07g44740	8.82748009
LOC_Os07g37320	LOC_Os10g28120	9.49289536
LOC_Os07g34580	LOC_Os07g46830	9.4669041
LOC_Os07g34140	LOC_Os08g28820	9.02465082
LOC_Os07g35940	LOC_Os10g30840	9.18387053
LOC_Os07g36140	LOC_Os07g38430	8.80561156
LOC_Os07g34580	LOC_Os10g23100	9.53858138
LOC_Os07g35940	LOC_Os12g12580	9.08716545
LOC_Os07g34140	LOC_Os10g07616	9.99352006
LOC_Os07g36500	LOC_Os11g10520	10.9141423
LOC_Os07g35880	LOC_Os09g31486	10.0172544
LOC_Os07g35940	LOC_Os09g23540	9.72860915
LOC_Os07g36500	LOC_Os11g31620	9.02280822
LOC_Os07g34140	LOC_Os10g39120	9.86120587
LOC_Os07g36500	LOC_Os10g22070	9.42845533
LOC_Os07g34140	LOC_Os10g09860	10.216265
LOC_Os07g36190	LOC_Os10g27050	9.09688559
LOC_Os07g34190	LOC_Os07g43390	8.92321657
LOC_Os07g34520	LOC_Os12g14070	9.77106583
LOC_Os07g34260	LOC_Os09g10230	9.05646328
LOC_Os07g35880	LOC_Os09g10200	11.4122362
LOC_Os07g37790	LOC_Os08g33370	10.0789541
LOC_Os07g35940	LOC_Os10g39840	10.6733167
LOC_Os07g34260	LOC_Os11g32580	9.21268754
LOC_Os07g35940	LOC_Os12g31370	9.35414891
LOC_Os07g36130	LOC_Os09g23560	9.15070728
LOC_Os07g35880	LOC_Os10g10434	9.99498835
LOC_Os07g34140	LOC_Os07g47420	11.2262634
LOC_Os07g37550	LOC_Os12g13320	10.096048
LOC_Os07g34140	LOC_Os10g38140	10.3088194
LOC_Os07g37550	LOC_Os10g28350	9.42531473
LOC_Os07g34520	LOC_Os10g11810	8.81360446

InteractorA	InteractorB	Z score
LOC_Os07g36140	LOC_Os12g13390	8.92497335
LOC_Os07g34580	LOC_Os11g07020	9.05408229
LOC_Os07g37550	LOC_Os09g25370	9.15636886
LOC_Os07g34190	LOC_Os08g09210	8.93630373
LOC_Os07g34580	LOC_Os12g07050	10.3406158
LOC_Os07g36140	LOC_Os09g21770	9.31294596
LOC_Os07g34140	LOC_Os07g42960	8.85163713
LOC_Os07g34140	LOC_Os10g28200	9.18762571
LOC_Os07g34589	LOC_Os07g48430	9.2642792
LOC_Os07g36130	LOC_Os07g46310	8.90958509
LOC_Os07g34190	LOC_Os09g30418	9.0094093
LOC_Os07g32800	LOC_Os07g48780	9.4039647
LOC_Os07g38910	LOC_Os11g47550	8.98143151
LOC_Os07g38890	LOC_Os07g44790	8.8920654
LOC_Os07g37790	LOC_Os12g16220	8.8079735
LOC_Os07g38970	LOC_Os12g12514	9.20891598
LOC_Os07g39270	LOC_Os11g32650	9.13634604
LOC_Os07g38540	LOC_Os10g21342	10.1302762
LOC_Os07g40290	LOC_Os09g37100	8.80260582
LOC_Os07g42490	LOC_Os08g02400	9.01571263
LOC_Os07g39290	LOC_Os07g42490	9.15038768
LOC_Os07g39290	LOC_Os11g14910	9.53028252
LOC_Os07g40580	LOC_Os08g09770	9.68876616
LOC_Os07g38430	LOC_Os09g10200	9.85639114
LOC_Os07g37790	LOC_Os11g16590	10.8336299
LOC_Os07g38890	LOC_Os12g31370	8.81383919
LOC_Os07g39270	LOC_Os09g04050	8.88896111
LOC_Os07g40290	LOC_Os10g21352	9.68350894
LOC_Os07g40290	LOC_Os10g42720	8.89610125
LOC_Os07g38430	LOC_Os08g40140	10.0676352
LOC_Os07g38430	LOC_Os12g44020	8.89314698
LOC_Os07g38540	LOC_Os08g42410	8.86443227
LOC_Os07g38890	LOC_Os11g07020	9.20181039
LOC_Os07g38730	LOC_Os12g12514	9.46351086
LOC_Os07g39290	LOC_Os12g23630	8.94735893
LOC_Os07g39870	LOC_Os12g07830	9.19360586
LOC_Os07g38430	LOC_Os10g01570	9.73138339
LOC_Os07g38540	LOC_Os07g44460	9.95477032
LOC_Os07g38540	LOC_Os10g23900	9.92551406
LOC_Os07g38860	LOC_Os12g06660	9.50346366
LOC_Os07g38890	LOC_Os11g10480	9.61331421
LOC_Os07g38910	LOC_Os09g30360	8.94804362
LOC_Os07g38860	LOC_Os07g48160	9.5804617

InteractorA	InteractorB	Z score
LOC_Os07g39870	LOC_Os11g02440	8.85947469
LOC_Os07g38730	LOC_Os07g43260	10.2701286
LOC_Os07g38540	LOC_Os11g25260	9.71772649
LOC_Os07g38540	LOC_Os09g25320	9.11876577
LOC_Os07g38730	LOC_Os10g41510	9.9232415
LOC_Os07g41750	LOC_Os07g48880	10.2068626
LOC_Os07g38030	LOC_Os08g08500	9.52867426
LOC_Os07g40290	LOC_Os12g03090	9.22747425
LOC_Os07g40290	LOC_Os08g34170	9.17070975
LOC_Os07g40290	LOC_Os08g15266	9.68355467
LOC_Os07g38540	LOC_Os11g24560	10.0409666
LOC_Os07g38860	LOC_Os10g38489	10.4597054
LOC_Os07g40290	LOC_Os12g14070	9.48416397
LOC_Os07g38730	LOC_Os10g35110	9.81877433
LOC_Os07g38540	LOC_Os10g32970	9.0554057
LOC_Os07g38030	LOC_Os07g46310	9.26297366
LOC_Os07g40580	LOC_Os08g36900	8.91563259
LOC_Os07g38540	LOC_Os12g02980	9.90929852
LOC_Os07g38030	LOC_Os10g41410	10.130526
LOC_Os07g38730	LOC_Os08g34170	10.8895319
LOC_Os07g38540	LOC_Os11g29190	8.98132268
LOC_Os07g38910	LOC_Os08g16910	9.0485811
LOC_Os07g40290	LOC_Os09g24412	9.68350894
LOC_Os07g39290	LOC_Os10g09860	9.16857139
LOC_Os07g38890	LOC_Os12g40510	9.31391913
LOC_Os07g39870	LOC_Os09g28460	9.11907165
LOC_Os07g38540	LOC_Os12g12470	9.82712537
LOC_Os07g40580	LOC_Os10g37060	8.95798566
LOC_Os07g39870	LOC_Os09g28420	9.07426543
LOC_Os07g40580	LOC_Os10g38360	9.12890907
LOC_Os07g38890	LOC_Os08g40140	9.26288722
LOC_Os07g39870	LOC_Os12g07720	10.0676344
LOC_Os07g41750	LOC_Os11g02130	9.06585358
LOC_Os07g38540	LOC_Os08g15276	10.1302762
LOC_Os07g41750	LOC_Os08g34280	9.68134649
LOC_Os07g38430	LOC_Os08g02340	8.94959101
LOC_Os07g41750	LOC_Os08g44340	9.6450942
LOC_Os07g38540	LOC_Os08g04560	9.40798473
LOC_Os07g40290	LOC_Os07g44590	8.89368604
LOC_Os07g38730	LOC_Os07g48040	10.9907576
LOC_Os07g39870	LOC_Os09g07460	9.15584436
LOC_Os07g42490	LOC_Os09g27750	9.4767784
LOC_Os07g41050	LOC_Os09g25390	9.6077297

InteractorA	InteractorB	Z score
LOC_Os07g41050	LOC_Os10g08670	8.95926606
LOC_Os07g39270	LOC_Os07g44440	9.19480428
LOC_Os07g38860	LOC_Os08g09240	9.84047287
LOC_Os07g39270	LOC_Os12g37960	8.83651503
LOC_Os07g41750	LOC_Os09g26380	8.89238392
LOC_Os07g38030	LOC_Os10g38470	9.66813216
LOC_Os07g38860	LOC_Os09g10260	9.29690808
LOC_Os07g41750	LOC_Os08g06060	10.1883026
LOC_Os07g40580	LOC_Os09g36450	8.91485333
LOC_Os07g39870	LOC_Os12g40830	9.72983151
LOC_Os07g38540	LOC_Os08g02700	9.93854242
LOC_Os07g38860	LOC_Os08g37490	10.6585845
LOC_Os07g40290	LOC_Os09g36710	9.45633113
LOC_Os07g41050	LOC_Os08g43170	8.86589913
LOC_Os07g40580	LOC_Os10g26390	10.447311
LOC_Os07g38540	LOC_Os10g08022	10.5561641
LOC_Os07g37790	LOC_Os10g09860	8.88924703
LOC_Os07g39870	LOC_Os08g37790	9.88093191
LOC_Os07g42490	LOC_Os08g08500	9.60691472
LOC_Os07g41750	LOC_Os11g26850	8.94628232
LOC_Os07g39870	LOC_Os11g07440	9.2569496
LOC_Os07g40290	LOC_Os10g09860	10.2975502
LOC_Os07g41050	LOC_Os11g28340	9.82181881
LOC_Os07g38540	LOC_Os10g08670	9.752755
LOC_Os07g38860	LOC_Os10g26600	9.21739907
LOC_Os07g40580	LOC_Os09g12230	9.05903149
LOC_Os07g39270	LOC_Os11g32610	10.6511007
LOC_Os07g40290	LOC_Os11g05570	9.61795695
LOC_Os07g41750	LOC_Os08g33710	9.76652578
LOC_Os07g38860	LOC_Os10g39590	9.53969468
LOC_Os07g41750	LOC_Os09g04050	8.96749609
LOC_Os07g39290	LOC_Os10g29620	9.25501449
LOC_Os07g38430	LOC_Os09g30418	9.43842992
LOC_Os07g38540	LOC_Os08g34790	9.35816931
LOC_Os07g39870	LOC_Os11g32520	8.84031608
LOC_Os07g38860	LOC_Os10g38700	9.0356253
LOC_Os07g41750	LOC_Os11g10520	9.60576087
LOC_Os07g38430	LOC_Os10g35480	8.86365058
LOC_Os07g39870	LOC_Os12g13390	9.11393491
LOC_Os07g38860	LOC_Os10g21212	9.85716858
LOC_Os07g38910	LOC_Os11g31530	9.72702545
LOC_Os07g39290	LOC_Os12g34450	9.61348288
LOC_Os07g38890	LOC_Os08g28800	10.0197591

InteractorA	InteractorB	Z score
LOC_Os07g40290	LOC_Os11g03400	9.2275006
LOC_Os07g40580	LOC_Os09g38030	9.20331172
LOC_Os07g43510	LOC_Os11g03290	10.1439432
LOC_Os07g43170	LOC_Os11g03230	9.84651692
LOC_Os07g43820	LOC_Os09g20090	9.10514834
LOC_Os07g44430	LOC_Os09g34960	9.13394377
LOC_Os07g42950	LOC_Os07g46280	9.46912344
LOC_Os07g42924	LOC_Os11g32260	9.48164256
LOC_Os07g42600	LOC_Os12g38760	8.92959086
LOC_Os07g44260	LOC_Os09g10260	9.60477391
LOC_Os07g44260	LOC_Os08g33820	9.05817523
LOC_Os07g43260	LOC_Os08g16910	9.38407384
LOC_Os07g43170	LOC_Os08g25570	9.17398045
LOC_Os07g42600	LOC_Os09g26380	16.2323351
LOC_Os07g43260	LOC_Os09g36670	8.84977252
LOC_Os07g42940	LOC_Os09g26380	9.49664724
LOC_Os07g42960	LOC_Os10g26010	8.92852811
LOC_Os07g42490	LOC_Os11g26850	9.92860819
LOC_Os07g42600	LOC_Os11g01872	8.96622624
LOC_Os07g43250	LOC_Os07g44260	9.02762711
LOC_Os07g44370	LOC_Os12g40550	9.11722513
LOC_Os07g44260	LOC_Os12g13380	8.86034877
LOC_Os07g43510	LOC_Os10g26390	10.8260777
LOC_Os07g43170	LOC_Os08g29170	9.12238752
LOC_Os07g44260	LOC_Os11g10510	9.66087772
LOC_Os07g42950	LOC_Os11g01360	9.62902225
LOC_Os07g43510	LOC_Os08g42560	10.4343262
LOC_Os07g42950	LOC_Os12g13320	9.49201809
LOC_Os07g43390	LOC_Os09g10260	9.63506351
LOC_Os07g44430	LOC_Os09g11230	9.00627598
LOC_Os07g43260	LOC_Os08g02700	8.99792971
LOC_Os07g42490	LOC_Os12g10730	9.1651897
LOC_Os07g43510	LOC_Os11g03980	8.95394515
LOC_Os07g44370	LOC_Os08g39140	9.24229341
LOC_Os07g43670	LOC_Os11g25700	9.80071721
LOC_Os07g43170	LOC_Os07g44590	9.41191075
LOC_Os07g43260	LOC_Os10g30580	8.93172247
LOC_Os07g43170	LOC_Os07g48050	9.92280341
LOC_Os07g42924	LOC_Os12g43100	8.97846804
LOC_Os07g43260	LOC_Os08g42540	9.03975455
LOC_Os07g42924	LOC_Os08g34170	11.5137485
LOC_Os07g43250	LOC_Os12g42876	9.99640727
LOC_Os07g42600	LOC_Os12g44010	9.15016432

InteractorA	InteractorB	Z score
LOC_Os07g42950	LOC_Os07g49120	10.8046825
LOC_Os07g42600	LOC_Os08g36910	10.1108377
LOC_Os07g43390	LOC_Os08g41880	10.4155443
LOC_Os07g44370	LOC_Os09g07830	9.04230576
LOC_Os07g43260	LOC_Os09g20820	9.01901349
LOC_Os07g42600	LOC_Os07g43820	9.1076642
LOC_Os07g43390	LOC_Os11g26910	9.4422989
LOC_Os07g43470	LOC_Os10g21268	9.86212499
LOC_Os07g44260	LOC_Os08g09210	8.92369157
LOC_Os07g44430	LOC_Os07g44590	9.30698382
LOC_Os07g43470	LOC_Os11g26850	8.90813927
LOC_Os07g43470	LOC_Os10g29620	9.31668277
LOC_Os07g43250	LOC_Os12g38760	10.177279
LOC_Os07g42600	LOC_Os10g25140	11.2011089
LOC_Os07g43670	LOC_Os12g05410	9.44643117
LOC_Os07g42960	LOC_Os10g13800	10.3505593
LOC_Os07g43250	LOC_Os10g25674	9.62534175
LOC_Os07g44260	LOC_Os09g25320	9.08307914
LOC_Os07g43260	LOC_Os10g39120	9.05297992
LOC_Os07g43470	LOC_Os09g32800	9.35610056
LOC_Os07g42950	LOC_Os12g10730	9.33845577
LOC_Os07g43170	LOC_Os12g07720	9.56652146
LOC_Os07g44430	LOC_Os07g44440	11.0408821
LOC_Os07g43260	LOC_Os10g17650	9.41568223
LOC_Os07g43510	LOC_Os09g23540	9.10838596
LOC_Os07g42600	LOC_Os09g20090	8.80896766
LOC_Os07g42600	LOC_Os10g39880	9.69450845
LOC_Os07g44430	LOC_Os09g20220	8.80137437
LOC_Os07g42600	LOC_Os08g35420	8.86164673
LOC_Os07g42950	LOC_Os10g28050	9.68993398
LOC_Os07g44370	LOC_Os12g38180	9.10424738
LOC_Os07g43670	LOC_Os12g43440	9.21299551
LOC_Os07g42600	LOC_Os12g13800	10.2346874
LOC_Os07g42600	LOC_Os09g38030	9.37431878
LOC_Os07g43170	LOC_Os11g32770	8.9122183
LOC_Os07g42950	LOC_Os08g39300	9.22442589
LOC_Os07g44430	LOC_Os10g26010	11.0025485
LOC_Os07g43510	LOC_Os08g35740	8.92526056
LOC_Os07g42940	LOC_Os08g05910	10.1655175
LOC_Os07g43670	LOC_Os09g08120	10.1353794
LOC_Os07g42960	LOC_Os12g22650	9.07057887
LOC_Os07g42950	LOC_Os11g41130	9.69768587
LOC_Os07g44430	LOC_Os08g03040	8.85547357

InteractorA	InteractorB	Z score
LOC_Os07g44430	LOC_Os10g35480	8.99950449
LOC_Os07g42950	LOC_Os10g38229	10.5421133
LOC_Os07g44260	LOC_Os12g17540	8.93305967
LOC_Os07g43820	LOC_Os12g41110	8.9092221
LOC_Os07g43470	LOC_Os07g46280	9.01415706
LOC_Os07g42950	LOC_Os07g46990	9.21356648
LOC_Os07g43260	LOC_Os08g41830	8.88313064
LOC_Os07g43260	LOC_Os12g08280	8.82665999
LOC_Os07g44370	LOC_Os10g39170	8.89403013
LOC_Os07g43470	LOC_Os08g03040	9.2631579
LOC_Os07g42940	LOC_Os10g13800	9.07805406
LOC_Os07g42950	LOC_Os11g07440	9.47510554
LOC_Os07g43670	LOC_Os09g36710	8.91665243
LOC_Os07g42940	LOC_Os08g37490	9.25413661
LOC_Os07g42490	LOC_Os11g47760	9.26907602
LOC_Os07g44430	LOC_Os08g09770	8.92900786
LOC_Os07g42960	LOC_Os10g07616	9.27753233
LOC_Os07g42940	LOC_Os08g34790	9.34110539
LOC_Os07g43170	LOC_Os08g15030	9.56152884
LOC_Os07g44260	LOC_Os12g07820	8.83168936
LOC_Os07g42950	LOC_Os10g21268	9.29232637
LOC_Os07g43820	LOC_Os08g31870	8.97935631
LOC_Os07g43390	LOC_Os10g08710	9.44718911
LOC_Os07g42600	LOC_Os10g25130	11.9461272
LOC_Os07g44370	LOC_Os10g38470	8.86834973
LOC_Os07g44260	LOC_Os09g30360	9.43943078
LOC_Os01g25065	LOC_Os07g03690	9.10105824
LOC_Os01g24680	LOC_Os06g12790	10.8768697
LOC_Os01g24710	LOC_Os11g25100	9.49511787
LOC_Os01g23620	LOC_Os04g52130	9.15987384
LOC_Os01g24680	LOC_Os02g45940	9.12755763
LOC_Os01g24680	LOC_Os11g48110	10.2537982
LOC_Os01g24680	LOC_Os02g57720	8.9623006
LOC_Os01g25065	LOC_Os03g57290	9.10392749
LOC_Os01g24680	LOC_Os04g09670	9.41067244
LOC_Os01g24680	LOC_Os05g04500	11.8418697
LOC_Os01g24680	LOC_Os12g40550	10.2002049
LOC_Os01g24680	LOC_Os05g47640	8.91061061
LOC_Os01g24750	LOC_Os09g20260	10.8417818
LOC_Os01g24680	LOC_Os01g61920	9.12753317
LOC_Os01g24680	LOC_Os05g11730	10.8085144
LOC_Os01g24680	LOC_Os10g38489	9.47565888
LOC_Os01g25065	LOC_Os05g05830	10.435782

InteractorA	InteractorB	Z score
LOC_Os01g24710	LOC_Os04g33470	9.18959161
LOC_Os01g24680	LOC_Os01g27260	12.0660622
LOC_Os01g25065	LOC_Os09g10200	8.82677253
LOC_Os01g24680	LOC_Os11g01010	8.91336898
LOC_Os01g24680	LOC_Os11g32770	9.39829528
LOC_Os01g23620	LOC_Os05g38230	9.80982638
LOC_Os01g24680	LOC_Os03g08280	8.81050827
LOC_Os01g24680	LOC_Os05g07090	8.82798851
LOC_Os01g25065	LOC_Os09g25370	9.68611948
LOC_Os01g23620	LOC_Os03g02260	10.1808055
LOC_Os01g24680	LOC_Os01g64660	9.29075669
LOC_Os01g24680	LOC_Os05g40990	9.18056065
LOC_Os01g24680	LOC_Os11g19800	11.6127977
LOC_Os01g24680	LOC_Os05g39050	9.12754999
LOC_Os01g24710	LOC_Os01g53900	8.82690044
LOC_Os01g24680	LOC_Os01g54370	9.96841704
LOC_Os01g24710	LOC_Os03g60400	10.2957728
LOC_Os01g24710	LOC_Os07g48020	9.5620896
LOC_Os01g24680	LOC_Os03g64050	9.51822461
LOC_Os01g25065	LOC_Os10g01540	10.2653466
LOC_Os01g24710	LOC_Os07g48880	9.31284918
LOC_Os01g24680	LOC_Os09g26340	9.12754073
LOC_Os01g24750	LOC_Os09g24924	9.19055764
LOC_Os01g23620	LOC_Os06g35560	9.14293702
LOC_Os01g24680	LOC_Os10g38160	10.775078
LOC_Os01g23620	LOC_Os10g21266	8.94309294
LOC_Os01g24680	LOC_Os12g41110	11.2944272
LOC_Os01g25065	LOC_Os08g05910	8.82317011
LOC_Os01g24680	LOC_Os07g35880	9.36338767
LOC_Os01g23620	LOC_Os10g26600	9.36764764
LOC_Os01g25065	LOC_Os05g12240	9.4749455
LOC_Os01g24680	LOC_Os03g02780	9.12756807
LOC_Os01g24680	LOC_Os06g28550	10.2802143
LOC_Os01g25065	LOC_Os10g37210	9.25724341
LOC_Os01g24680	LOC_Os04g24520	9.36726743
LOC_Os01g24680	LOC_Os02g29020	9.06357912
LOC_Os01g23620	LOC_Os06g27770	9.50470808
LOC_Os01g24680	LOC_Os05g48290	9.91812768
LOC_Os01g23620	LOC_Os02g51830	8.91497073
LOC_Os01g24680	LOC_Os10g39410	9.12754999
LOC_Os01g24710	LOC_Os11g26860	8.89521004
LOC_Os01g24680	LOC_Os03g26960	9.27329878
LOC_Os01g24750	LOC_Os02g22780	10.4858486

InteractorA	InteractorB	Z score
LOC_Os01g23620	LOC_Os01g73790	9.25898887
LOC_Os01g24750	LOC_Os11g32650	10.0144897
LOC_Os01g24680	LOC_Os12g40510	9.13035838
LOC_Os01g24680	LOC_Os12g44150	8.81775955
LOC_Os01g24680	LOC_Os01g50050	9.04880771
LOC_Os01g24750	LOC_Os01g40870	9.37455482
LOC_Os01g24750	LOC_Os04g46910	9.9044378
LOC_Os01g24680	LOC_Os04g49420	9.12754073
LOC_Os01g24680	LOC_Os03g04260	9.65574297
LOC_Os01g24710	LOC_Os07g26540	11.5449718
LOC_Os01g23620	LOC_Os03g15960	9.33266513
LOC_Os01g24680	LOC_Os01g59600	9.00822264
LOC_Os01g25065	LOC_Os08g39860	9.31572608
LOC_Os01g24680	LOC_Os08g41340	9.30503627
LOC_Os01g24680	LOC_Os08g14760	8.89734842
LOC_Os01g25065	LOC_Os02g33140	9.05296584
LOC_Os01g24710	LOC_Os09g31490	9.48228954
LOC_Os01g25065	LOC_Os07g22498	9.1038008
LOC_Os01g23620	LOC_Os03g17690	9.44120154
LOC_Os01g24750	LOC_Os09g28420	10.2977319
LOC_Os01g24680	LOC_Os01g71400	12.590675
LOC_Os01g24750	LOC_Os04g46460	9.54513244
LOC_Os01g24750	LOC_Os12g13390	8.96791344
LOC_Os01g25065	LOC_Os12g31370	9.14177397
LOC_Os01g24680	LOC_Os04g47170	9.02920596
LOC_Os01g24680	LOC_Os12g17910	9.60685738
LOC_Os01g24680	LOC_Os09g38020	9.12756807
LOC_Os01g24750	LOC_Os09g28770	9.6561696
LOC_Os01g25065	LOC_Os01g39270	8.86149337
LOC_Os01g24680	LOC_Os01g46070	10.5096372
LOC_Os01g24750	LOC_Os05g50710	9.68045031
LOC_Os01g24680	LOC_Os10g29620	8.82861732
LOC_Os01g24680	LOC_Os06g37660	8.96152658
LOC_Os01g25065	LOC_Os06g46436	9.1038008
LOC_Os01g24750	LOC_Os02g47790	9.25318431
LOC_Os01g24750	LOC_Os03g28330	10.687088
LOC_Os01g24680	LOC_Os04g02050	9.10825659
LOC_Os01g24680	LOC_Os04g52130	9.02589004
LOC_Os01g23620	LOC_Os03g30950	9.16469949
LOC_Os01g24710	LOC_Os09g39380	9.84778778
LOC_Os01g24680	LOC_Os05g38740	9.12756807
LOC_Os01g24680	LOC_Os07g36500	9.12756499
LOC_Os01g23620	LOC_Os02g10310	9.44297485

InteractorA	InteractorB	Z score
LOC_Os01g25065	LOC_Os04g46560	10.676632
LOC_Os01g24680	LOC_Os08g35740	9.04765462
LOC_Os01g23620	LOC_Os09g10270	9.22777195
LOC_Os01g24710	LOC_Os05g09500	10.781766
LOC_Os01g24680	LOC_Os01g70770	10.1223596
LOC_Os01g24680	LOC_Os04g37820	9.1159242
LOC_Os01g25065	LOC_Os10g26600	8.85397346
LOC_Os01g23620	LOC_Os09g33500	9.05796778
LOC_Os01g25065	LOC_Os10g21406	9.1038008
LOC_Os01g24680	LOC_Os10g38690	8.8878632
LOC_Os01g23620	LOC_Os02g33080	9.10214395
LOC_Os01g24750	LOC_Os06g09450	9.05814797
LOC_Os01g24680	LOC_Os04g59150	8.81219012
LOC_Os01g23620	LOC_Os10g41510	9.33198589
LOC_Os01g24680	LOC_Os05g05830	10.973333
LOC_Os01g24680	LOC_Os06g51084	9.2008311
LOC_Os01g25065	LOC_Os06g05090	11.5164472
LOC_Os01g24680	LOC_Os07g09340	9.20005715
LOC_Os01g24680	LOC_Os03g26970	9.54301909
LOC_Os01g24680	LOC_Os04g33040	9.66208864
LOC_Os07g44450	LOC_Os09g15790	9.07385993
LOC_Os07g46280	LOC_Os10g29470	9.0907314
LOC_Os07g44460	LOC_Os10g32970	9.37015225
LOC_Os07g44790	LOC_Os08g37800	9.50434559
LOC_Os07g44440	LOC_Os11g14910	9.54892011
LOC_Os07g46460	LOC_Os12g16200	9.16032602
LOC_Os07g44790	LOC_Os12g41110	8.81320956
LOC_Os07g44740	LOC_Os08g20270	9.22024062
LOC_Os07g44790	LOC_Os10g32870	9.46386169
LOC_Os07g46310	LOC_Os08g08070	9.38234664
LOC_Os07g44620	LOC_Os12g13800	8.95224762
LOC_Os07g44450	LOC_Os11g10520	9.0572895
LOC_Os07g46280	LOC_Os12g16240	8.84644889
LOC_Os07g46280	LOC_Os08g33710	10.5590963
LOC_Os07g46310	LOC_Os07g47420	11.6739596
LOC_Os07g46630	LOC_Os12g06620	8.99323192
LOC_Os07g44450	LOC_Os09g39810	8.89681172
LOC_Os07g46310	LOC_Os10g38540	9.67576536
LOC_Os07g47420	LOC_Os08g09940	8.93527335
LOC_Os07g46830	LOC_Os10g25950	8.98632844
LOC_Os07g46460	LOC_Os11g29400	9.39165465
LOC_Os07g46460	LOC_Os09g27750	10.3192298
LOC_Os07g44620	LOC_Os11g26850	9.01752497

InteractorA	InteractorB	Z score
LOC_Os07g46830	LOC_Os12g44020	10.2468203
LOC_Os07g46460	LOC_Os08g33820	9.71953068
LOC_Os07g46280	LOC_Os08g08500	9.57654991
LOC_Os07g44620	LOC_Os08g36900	9.47408951
LOC_Os07g44790	LOC_Os10g37210	9.06321999
LOC_Os07g46990	LOC_Os10g38140	9.45158788
LOC_Os07g47490	LOC_Os09g12230	10.1592647
LOC_Os07g44740	LOC_Os09g23540	9.60698788
LOC_Os07g46830	LOC_Os12g04980	8.87653416
LOC_Os07g44440	LOC_Os11g40140	10.0195903
LOC_Os07g47290	LOC_Os08g37790	9.01096439
LOC_Os07g44740	LOC_Os08g28820	9.3593312
LOC_Os07g46310	LOC_Os09g31486	9.07627041
LOC_Os07g44550	LOC_Os09g23540	8.87823721
LOC_Os07g47420	LOC_Os12g13380	10.4455585
LOC_Os07g44440	LOC_Os10g03540	10.2361414
LOC_Os07g47420	LOC_Os10g22450	9.66178942
LOC_Os07g46460	LOC_Os09g07830	9.33844181
LOC_Os07g46460	LOC_Os10g38730	9.87141072
LOC_Os07g44620	LOC_Os10g37060	9.5329251
LOC_Os07g46310	LOC_Os12g17910	9.14848532
LOC_Os07g44460	LOC_Os08g06060	9.09158893
LOC_Os07g46830	LOC_Os11g03980	10.7488162
LOC_Os07g46310	LOC_Os09g27820	9.18513232
LOC_Os07g44460	LOC_Os11g32610	9.21686271
LOC_Os07g47490	LOC_Os08g33370	8.8845247
LOC_Os07g44620	LOC_Os12g13390	9.05890206
LOC_Os07g44550	LOC_Os11g05730	10.5558631
LOC_Os07g44460	LOC_Os12g22650	9.41627228
LOC_Os07g46630	LOC_Os09g39440	9.53822574
LOC_Os07g46460	LOC_Os12g17910	9.05373841
LOC_Os07g47420	LOC_Os10g38640	9.3928095
LOC_Os07g46280	LOC_Os09g08120	9.32267206
LOC_Os07g44790	LOC_Os08g37490	10.1195406
LOC_Os07g44790	LOC_Os09g39440	8.88975744
LOC_Os07g46310	LOC_Os12g16410	11.3118218
LOC_Os07g46310	LOC_Os09g17740	9.48273763
LOC_Os07g46460	LOC_Os08g23730	8.8400502
LOC_Os07g44790	LOC_Os12g34450	9.01882547
LOC_Os07g44460	LOC_Os10g38140	9.06907072
LOC_Os07g47420	LOC_Os08g41830	8.9813207
LOC_Os07g44740	LOC_Os12g07980	8.92196208
LOC_Os07g44450	LOC_Os12g42876	9.21020787

InteractorA	InteractorB	Z score
LOC_Os07g46310	LOC_Os10g38489	9.41262424
LOC_Os07g44740	LOC_Os07g44790	9.15899565
LOC_Os07g44740	LOC_Os09g23560	8.83777195
LOC_Os07g46310	LOC_Os11g34570	8.89968363
LOC_Os07g47490	LOC_Os09g10230	8.880352
LOC_Os07g44440	LOC_Os10g21212	9.06514157
LOC_Os07g47420	LOC_Os11g32520	9.04637143
LOC_Os07g44740	LOC_Os09g38030	10.8550851
LOC_Os07g44590	LOC_Os08g40140	10.5829412
LOC_Os07g46830	LOC_Os12g38760	9.12226795
LOC_Os07g46280	LOC_Os12g42280	8.85065221
LOC_Os07g44740	LOC_Os10g36650	8.85902163
LOC_Os07g46830	LOC_Os12g13800	9.02824872
LOC_Os07g46310	LOC_Os09g27750	9.13678325
LOC_Os07g46310	LOC_Os07g46630	9.08584705
LOC_Os07g46310	LOC_Os12g44030	8.87979516
LOC_Os07g44790	LOC_Os11g47570	9.23867108
LOC_Os07g44790	LOC_Os11g04954	9.28882849
LOC_Os07g44740	LOC_Os09g34214	9.14171123
LOC_Os07g44790	LOC_Os12g22650	9.28701722
LOC_Os07g44790	LOC_Os09g39500	9.24937149
LOC_Os07g47420	LOC_Os09g28400	9.00197543
LOC_Os07g46310	LOC_Os12g38180	9.43790304
LOC_Os07g46830	LOC_Os12g12514	9.46757831
LOC_Os07g46280	LOC_Os12g21798	9.21948938
LOC_Os07g46990	LOC_Os10g39880	8.8909234
LOC_Os07g46280	LOC_Os11g33240	9.19907671
LOC_Os07g46630	LOC_Os12g31370	8.92720706
LOC_Os07g46990	LOC_Os09g23560	9.29904682
LOC_Os07g46280	LOC_Os08g23730	9.56452466
LOC_Os07g44740	LOC_Os11g14040	9.04736254
LOC_Os07g47490	LOC_Os10g09860	10.4130238
LOC_Os07g44740	LOC_Os09g15400	8.84948274
LOC_Os07g44460	LOC_Os09g20260	9.87651253
LOC_Os07g46310	LOC_Os10g35480	9.86315943
LOC_Os07g44450	LOC_Os10g34520	8.87728455
LOC_Os07g46310	LOC_Os10g38470	10.3649697
LOC_Os07g46830	LOC_Os09g10230	9.51489296
LOC_Os07g44790	LOC_Os12g31640	8.81498403
LOC_Os07g46460	LOC_Os10g23100	9.52882688
LOC_Os07g44430	LOC_Os11g40140	9.05270087
LOC_Os07g44620	LOC_Os09g23560	9.05792229
LOC_Os07g47420	LOC_Os12g12514	8.94771952

InteractorA	InteractorB	Z score
LOC_Os07g46310	LOC_Os09g07830	9.11931412
LOC_Os07g44460	LOC_Os10g08670	8.98175174
LOC_Os07g44450	LOC_Os10g29470	9.75879106
LOC_Os07g47420	LOC_Os09g23530	8.96883347
LOC_Os07g46460	LOC_Os11g01872	8.87555206
LOC_Os07g49400	LOC_Os11g08440	9.475814
LOC_Os07g48040	LOC_Os12g13380	9.26028627
LOC_Os08g02400	LOC_Os08g39140	10.6482603
LOC_Os08g02340	LOC_Os11g32520	9.03646374
LOC_Os07g48880	LOC_Os08g29520	9.92060664
LOC_Os07g49400	LOC_Os08g37490	8.94060045
LOC_Os07g48430	LOC_Os08g44280	8.87866244
LOC_Os07g48060	LOC_Os10g08620	10.3137026
LOC_Os07g47490	LOC_Os11g20790	9.07776208
LOC_Os08g02400	LOC_Os09g04050	9.19691043
LOC_Os07g48050	LOC_Os10g27174	9.05181673
LOC_Os07g49120	LOC_Os12g12514	9.49057158
LOC_Os07g48020	LOC_Os12g25700	8.81365859
LOC_Os07g49220	LOC_Os10g38670	10.1089919
LOC_Os07g48010	LOC_Os09g10300	9.93724234
LOC_Os07g49400	LOC_Os12g42280	9.2915438
LOC_Os07g49120	LOC_Os10g22070	9.74542291
LOC_Os07g48430	LOC_Os08g38920	9.21114106
LOC_Os07g48040	LOC_Os08g15266	9.82017103
LOC_Os07g49220	LOC_Os09g38030	9.20844007
LOC_Os08g02400	LOC_Os10g38470	9.22821561
LOC_Os08g02340	LOC_Os09g31430	9.59228642
LOC_Os07g48050	LOC_Os11g38959	8.88128673
LOC_Os07g47990	LOC_Os09g04730	8.96934618
LOC_Os07g48780	LOC_Os09g20090	9.31345523
LOC_Os07g49220	LOC_Os10g38160	8.81415145
LOC_Os07g47990	LOC_Os12g10570	9.11085248
LOC_Os08g01660	LOC_Os12g25690	10.2319933
LOC_Os07g49120	LOC_Os11g26910	9.5981458
LOC_Os07g49220	LOC_Os08g42000	8.89351819
LOC_Os08g02400	LOC_Os10g26010	9.8293945
LOC_Os08g01660	LOC_Os12g43630	9.03926031
LOC_Os07g47990	LOC_Os08g39860	8.88056327
LOC_Os07g48780	LOC_Os09g36450	9.67449056
LOC_Os07g48050	LOC_Os12g44030	9.58640512
LOC_Os08g01660	LOC_Os10g11810	8.89341763
LOC_Os07g48430	LOC_Os09g07830	8.86321938
LOC_Os07g48880	LOC_Os10g38640	9.69805104

InteractorA	InteractorB	Z score
LOC_Os07g47490	LOC_Os11g41610	9.8298661
LOC_Os07g48040	LOC_Os09g24412	9.82014011
LOC_Os07g48880	LOC_Os08g33710	9.50641526
LOC_Os07g48040	LOC_Os10g39680	9.05680971
LOC_Os08g02400	LOC_Os09g28460	9.81216976
LOC_Os07g48430	LOC_Os12g12514	10.4409845
LOC_Os07g48010	LOC_Os10g38660	9.12635465
LOC_Os08g01660	LOC_Os08g09770	9.46274827
LOC_Os07g48010	LOC_Os12g43440	9.89248161
LOC_Os07g49220	LOC_Os12g25630	8.90047264
LOC_Os07g49120	LOC_Os10g32550	10.1233886
LOC_Os08g01660	LOC_Os09g09230	8.80111061
LOC_Os07g48060	LOC_Os12g17910	9.37903231
LOC_Os07g48430	LOC_Os08g04540	8.94394391
LOC_Os07g49220	LOC_Os10g38600	9.59691589
LOC_Os07g49400	LOC_Os09g38620	11.0245583
LOC_Os07g48430	LOC_Os10g35110	8.95849841
LOC_Os07g48430	LOC_Os08g25570	11.92682
LOC_Os07g49220	LOC_Os10g38700	8.90685025
LOC_Os07g47490	LOC_Os10g39680	8.81495293
LOC_Os07g48780	LOC_Os08g17680	8.93751033
LOC_Os08g02400	LOC_Os11g43360	9.2921542
LOC_Os07g48880	LOC_Os10g38630	10.0600106
LOC_Os08g02340	LOC_Os08g33820	8.83193701
LOC_Os08g02400	LOC_Os08g04560	9.11927448
LOC_Os08g02340	LOC_Os08g09200	10.4419413
LOC_Os07g48010	LOC_Os07g49220	10.4379513
LOC_Os07g48780	LOC_Os11g48110	8.86939144
LOC_Os07g49400	LOC_Os10g36650	10.1657761
LOC_Os08g02340	LOC_Os10g21250	8.98268388
LOC_Os07g49220	LOC_Os08g06100	9.22319607
LOC_Os07g48880	LOC_Os11g08470	9.78618053
LOC_Os07g49400	LOC_Os10g26600	9.59559006
LOC_Os07g48430	LOC_Os08g10010	8.90530484
LOC_Os07g48020	LOC_Os10g22450	9.75858283
LOC_Os07g48040	LOC_Os10g21352	9.82014011
LOC_Os07g48880	LOC_Os08g34210	9.28975824
LOC_Os07g48060	LOC_Os10g30200	9.02382109
LOC_Os07g48020	LOC_Os10g08550	9.55502638
LOC_Os07g48030	LOC_Os08g44810	8.95239387
LOC_Os07g48050	LOC_Os09g24412	10.5204067
LOC_Os07g49220	LOC_Os11g02600	8.9368083
LOC_Os07g48040	LOC_Os12g12580	9.38600412

InteractorA	InteractorB	Z score
LOC_Os07g48040	LOC_Os11g08340	8.81322453
LOC_Os07g49120	LOC_Os08g25570	9.15978145
LOC_Os07g48780	LOC_Os12g21798	8.86737368
LOC_Os07g49220	LOC_Os10g28120	9.3220171
LOC_Os08g02340	LOC_Os09g07510	8.87156962
LOC_Os07g48430	LOC_Os08g09770	10.9377439
LOC_Os07g49120	LOC_Os07g49220	9.10124086
LOC_Os07g48430	LOC_Os10g26110	8.84693597
LOC_Os07g49120	LOC_Os10g02480	9.26954399
LOC_Os07g48040	LOC_Os11g19320	9.68912731
LOC_Os07g48010	LOC_Os11g03980	9.29525545
LOC_Os07g48050	LOC_Os10g21324	10.1211797
LOC_Os07g48060	LOC_Os10g40700	10.6872792
LOC_Os08g01660	LOC_Os10g32870	8.98977976
LOC_Os07g48880	LOC_Os12g36950	9.68008824
LOC_Os08g02340	LOC_Os09g10260	9.47986785
LOC_Os07g48060	LOC_Os09g12660	9.23134871
LOC_Os07g48060	LOC_Os08g17680	8.82057036
LOC_Os08g01660	LOC_Os08g25570	10.0820855
LOC_Os07g48050	LOC_Os08g15266	10.5204067
LOC_Os07g48020	LOC_Os09g12590	9.0271062
LOC_Os07g48780	LOC_Os08g37790	8.97099667
LOC_Os07g49220	LOC_Os10g38360	9.92184307
LOC_Os08g02400	LOC_Os11g08440	8.80812402
LOC_Os07g48880	LOC_Os09g08880	9.7827029
LOC_Os07g48050	LOC_Os08g04540	10.0814814
LOC_Os08g02400	LOC_Os10g01540	10.2081746
LOC_Os07g47990	LOC_Os09g23560	10.3792155
LOC_Os07g49400	LOC_Os08g35740	8.95575528
LOC_Os07g48020	LOC_Os08g44960	8.85016784
LOC_Os07g48050	LOC_Os10g21352	10.5204067
LOC_Os07g48060	LOC_Os10g41510	10.6466632
LOC_Os07g49120	LOC_Os11g19800	9.31031964
LOC_Os07g48060	LOC_Os11g25100	9.30113341
LOC_Os08g09200	LOC_Os10g41689	9.0056531
LOC_Os08g04540	LOC_Os08g37800	11.9599314
LOC_Os08g04540	LOC_Os08g04560	16.0928644
LOC_Os08g02410	LOC_Os09g33860	9.88580504
LOC_Os08g04180	LOC_Os11g05730	8.84954596
LOC_Os08g04180	LOC_Os11g26910	10.2191438
LOC_Os08g02410	LOC_Os10g34760	9.20776432
LOC_Os08g03040	LOC_Os09g04680	9.07849979
LOC_Os08g02410	LOC_Os09g36930	8.99711101

InteractorA	InteractorB	Z score
LOC_Os08g08500	LOC_Os09g30412	9.77420596
LOC_Os08g06060	LOC_Os10g38660	9.1450829
LOC_Os08g04540	LOC_Os12g12514	9.22201582
LOC_Os08g06100	LOC_Os12g10720	9.0641367
LOC_Os08g04540	LOC_Os12g38750	9.1572476
LOC_Os08g04560	LOC_Os09g09230	8.98229083
LOC_Os08g09200	LOC_Os09g09520	8.94732347
LOC_Os08g02410	LOC_Os08g16910	9.78934747
LOC_Os08g04560	LOC_Os10g23900	10.2597153
LOC_Os08g02410	LOC_Os09g24412	9.39002599
LOC_Os08g05910	LOC_Os08g17784	8.87835012
LOC_Os08g03040	LOC_Os08g31870	9.09787716
LOC_Os08g06550	LOC_Os10g25930	9.54826528
LOC_Os08g02410	LOC_Os10g41550	9.30946829
LOC_Os08g02410	LOC_Os10g39170	9.4575451
LOC_Os08g06610	LOC_Os09g26380	8.92737101
LOC_Os08g04180	LOC_Os10g41510	10.5975217
LOC_Os08g03040	LOC_Os10g30200	8.89258432
LOC_Os08g04560	LOC_Os12g38180	9.0688237
LOC_Os08g06100	LOC_Os09g36930	9.27626316
LOC_Os08g02700	LOC_Os12g22650	9.39061233
LOC_Os08g04180	LOC_Os11g03400	10.5004839
LOC_Os08g03290	LOC_Os12g42876	9.40769722
LOC_Os08g09200	LOC_Os10g41410	8.82537104
LOC_Os08g06550	LOC_Os08g31060	8.85784393
LOC_Os08g04560	LOC_Os10g28050	9.05818956
LOC_Os08g02700	LOC_Os10g20910	9.08547005
LOC_Os08g02700	LOC_Os12g07980	8.80552091
LOC_Os08g06060	LOC_Os10g38640	10.3779139
LOC_Os08g02410	LOC_Os09g08720	9.81976069
LOC_Os08g02410	LOC_Os10g21352	9.39002599
LOC_Os08g08070	LOC_Os12g16290	9.27059708
LOC_Os08g02410	LOC_Os11g02440	8.89351758
LOC_Os08g02700	LOC_Os11g26910	9.06259779
LOC_Os08g06100	LOC_Os12g13810	12.1728574
LOC_Os08g06060	LOC_Os12g16240	9.90855472
LOC_Os08g08070	LOC_Os09g37100	9.40034679
LOC_Os08g02410	LOC_Os09g30412	9.27594686
LOC_Os08g08500	LOC_Os08g20730	9.061307
LOC_Os08g06550	LOC_Os10g26600	10.4269632
LOC_Os08g03290	LOC_Os12g22030	9.20802464
LOC_Os08g08500	LOC_Os11g26860	9.3552461
LOC_Os08g02700	LOC_Os10g34520	10.0590752

InteractorA	InteractorB	Z score
LOC_Os08g04540	LOC_Os09g10270	9.37650133
LOC_Os08g06100	LOC_Os12g44000	9.13523679
LOC_Os08g04180	LOC_Os12g03090	10.5005017
LOC_Os08g02410	LOC_Os08g42910	9.33340394
LOC_Os08g06610	LOC_Os08g29520	9.03775402
LOC_Os08g04560	LOC_Os10g10434	9.32225936
LOC_Os08g04560	LOC_Os10g26110	21.6781851
LOC_Os08g04180	LOC_Os10g07040	10.1079342
LOC_Os08g05910	LOC_Os08g32620	9.35256971
LOC_Os08g06550	LOC_Os10g21352	10.6531712
LOC_Os08g06100	LOC_Os08g10010	9.38908081
LOC_Os08g06060	LOC_Os12g01922	8.91851523
LOC_Os08g04540	LOC_Os10g23900	27.2389952
LOC_Os08g04560	LOC_Os08g33370	10.291881
LOC_Os08g04540	LOC_Os10g26110	22.2184609
LOC_Os08g06100	LOC_Os08g09210	10.2470775
LOC_Os08g08500	LOC_Os09g39570	9.08776447
LOC_Os08g06550	LOC_Os08g44210	9.94104291
LOC_Os08g02410	LOC_Os10g38234	9.58753492
LOC_Os08g04560	LOC_Os09g28770	9.68573168
LOC_Os08g04540	LOC_Os08g39860	9.63407194
LOC_Os08g03040	LOC_Os08g25570	9.36570111
LOC_Os08g09200	LOC_Os10g08620	9.44416553
LOC_Os08g04540	LOC_Os12g06620	10.3563794
LOC_Os08g06610	LOC_Os09g07460	9.81876082
LOC_Os08g02700	LOC_Os08g39300	9.21795262
LOC_Os08g06060	LOC_Os10g39120	8.87774279
LOC_Os08g05910	LOC_Os12g34062	9.94425115
LOC_Os08g02410	LOC_Os11g02130	9.65255524
LOC_Os08g02410	LOC_Os10g02070	10.4196248
LOC_Os08g06060	LOC_Os10g39680	9.34373183
LOC_Os08g06060	LOC_Os09g31120	9.31228829
LOC_Os08g09200	LOC_Os08g34170	9.4209106
LOC_Os08g04540	LOC_Os11g19220	9.15567878
LOC_Os08g03440	LOC_Os11g08340	8.94429493
LOC_Os08g02410	LOC_Os08g15266	9.39002599
LOC_Os08g02410	LOC_Os10g07040	9.42183295
LOC_Os08g03040	LOC_Os11g30310	9.2605759
LOC_Os08g04180	LOC_Os08g41880	9.45346654
LOC_Os08g08500	LOC_Os11g32260	9.83859526
LOC_Os08g03040	LOC_Os08g09200	9.16585375
LOC_Os08g02700	LOC_Os11g08330	8.94222067
LOC_Os08g06550	LOC_Os09g24412	10.6531712

InteractorA	InteractorB	Z score
LOC_Os08g05490	LOC_Os09g10270	10.0769946
LOC_Os08g04460	LOC_Os09g08720	9.03547061
LOC_Os08g09200	LOC_Os09g28770	8.82314282
LOC_Os08g04180	LOC_Os12g22650	8.83783992
LOC_Os08g02410	LOC_Os09g09230	9.61238146
LOC_Os08g06550	LOC_Os08g15266	10.6531712
LOC_Os08g04560	LOC_Os10g28320	11.51593
LOC_Os08g02700	LOC_Os08g33710	9.9475446
LOC_Os08g09200	LOC_Os12g38760	10.6424353
LOC_Os08g09770	LOC_Os08g34170	10.1695974
LOC_Os08g09240	LOC_Os08g36320	9.38856681
LOC_Os08g09950	LOC_Os09g20820	9.19953859
LOC_Os08g15030	LOC_Os09g37100	9.96547693
LOC_Os08g14760	LOC_Os09g39380	9.84880647
LOC_Os08g09200	LOC_Os12g44030	9.13372229
LOC_Os08g09250	LOC_Os11g31620	9.13632797
LOC_Os08g09370	LOC_Os09g36800	9.11691788
LOC_Os08g09770	LOC_Os12g10600	9.31826401
LOC_Os08g14760	LOC_Os12g13390	9.48894757
LOC_Os08g09770	LOC_Os08g37790	9.03747283
LOC_Os08g09770	LOC_Os11g32770	9.04174833
LOC_Os08g15276	LOC_Os09g08072	9.19016379
LOC_Os08g09210	LOC_Os08g34210	9.20347903
LOC_Os08g09940	LOC_Os08g41340	9.81338645
LOC_Os08g09770	LOC_Os11g25700	8.82617037
LOC_Os08g15276	LOC_Os12g38760	9.62748404
LOC_Os08g09200	LOC_Os12g06660	10.1958829
LOC_Os08g09210	LOC_Os08g09770	9.08977123
LOC_Os08g09200	LOC_Os12g43450	10.2405344
LOC_Os08g09210	LOC_Os12g43450	8.84835569
LOC_Os08g09950	LOC_Os09g10300	8.920207
LOC_Os08g09210	LOC_Os12g16250	9.02679854
LOC_Os08g15276	LOC_Os10g21248	10.0520778
LOC_Os08g14770	LOC_Os09g25390	13.4552905
LOC_Os08g14760	LOC_Os10g07040	11.8556978
LOC_Os08g09210	LOC_Os12g32240	9.04093929
LOC_Os08g09940	LOC_Os10g39840	8.97222352
LOC_Os08g09250	LOC_Os08g42410	9.66911503
LOC_Os08g14770	LOC_Os08g38920	9.21745528
LOC_Os08g14570	LOC_Os11g40150	10.0105652
LOC_Os08g10010	LOC_Os09g39500	8.8867062
LOC_Os08g14760	LOC_Os09g33860	9.5800843
LOC_Os08g15040	LOC_Os11g01872	9.04025981

InteractorA	InteractorB	Z score
LOC_Os08g10608	LOC_Os09g25390	9.70327348
LOC_Os08g14570	LOC_Os10g07229	10.506295
LOC_Os08g15266	LOC_Os10g31000	9.00579729
LOC_Os08g14570	LOC_Os10g41510	8.84146459
LOC_Os08g14760	LOC_Os08g28800	10.9367101
LOC_Os08g09770	LOC_Os12g34380	9.26665775
LOC_Os08g09250	LOC_Os12g17910	8.85092828
LOC_Os08g10010	LOC_Os09g20820	11.5773655
LOC_Os08g14770	LOC_Os12g43440	8.80837402
LOC_Os08g14760	LOC_Os09g10230	8.99807961
LOC_Os08g10010	LOC_Os12g38760	8.99060446
LOC_Os08g14760	LOC_Os08g27840	8.88232443
LOC_Os08g14760	LOC_Os10g38700	9.06230433
LOC_Os08g09200	LOC_Os12g10730	8.95366921
LOC_Os08g15266	LOC_Os08g41880	9.41889211
LOC_Os08g14760	LOC_Os11g42350	9.15014833
LOC_Os08g15266	LOC_Os11g25330	9.92153133
LOC_Os08g10510	LOC_Os08g31870	10.6976281
LOC_Os08g10608	LOC_Os08g44530	8.85434226
LOC_Os08g15030	LOC_Os10g02480	10.6205104
LOC_Os08g15292	LOC_Os08g17680	8.86507095
LOC_Os08g09210	LOC_Os10g40090	8.9821738
LOC_Os08g09250	LOC_Os09g26380	9.08247323
LOC_Os08g09210	LOC_Os11g25220	9.12464147
LOC_Os08g14760	LOC_Os12g43370	10.7862165
LOC_Os08g09950	LOC_Os10g32550	8.86174817
LOC_Os08g09940	LOC_Os11g32770	8.81375901
LOC_Os08g14570	LOC_Os11g08330	9.8093912
LOC_Os08g15266	LOC_Os09g08910	9.21414864
LOC_Os08g14760	LOC_Os09g04050	10.5270148
LOC_Os08g09240	LOC_Os10g14150	9.67952113
LOC_Os08g14770	LOC_Os10g34760	9.89728711
LOC_Os08g14760	LOC_Os12g07720	9.16802141
LOC_Os08g15266	LOC_Os09g38030	9.58499466
LOC_Os08g15030	LOC_Os08g20730	8.89615652
LOC_Os08g09210	LOC_Os09g23540	8.9253838
LOC_Os08g14770	LOC_Os11g08440	8.87395568
LOC_Os08g10608	LOC_Os11g05570	9.71390716
LOC_Os08g14770	LOC_Os10g27174	9.10741743
LOC_Os08g15266	LOC_Os12g21798	9.12606016
LOC_Os08g14760	LOC_Os10g26110	8.85215998
LOC_Os08g14770	LOC_Os10g38350	9.13695572
LOC_Os08g15030	LOC_Os10g10434	9.5351664

InteractorA	InteractorB	Z score
LOC_Os08g14760	LOC_Os10g07616	10.1147805
LOC_Os08g09240	LOC_Os12g43370	8.87598584
LOC_Os08g10608	LOC_Os11g07440	9.07156948
LOC_Os08g09250	LOC_Os08g36900	9.78713657
LOC_Os08g09250	LOC_Os11g37640	8.97149206
LOC_Os08g09940	LOC_Os09g34250	9.41362044
LOC_Os08g14760	LOC_Os12g16410	8.92452102
LOC_Os08g14760	LOC_Os12g06620	10.3872838
LOC_Os08g09770	LOC_Os09g31430	9.33738688
LOC_Os08g09210	LOC_Os08g36900	9.75187766
LOC_Os08g09210	LOC_Os08g44960	9.39923379
LOC_Os08g09250	LOC_Os11g05290	10.8854425
LOC_Os08g14760	LOC_Os09g28420	9.54400423
LOC_Os08g15030	LOC_Os10g28360	8.94031588
LOC_Os08g09250	LOC_Os08g38900	9.31775266
LOC_Os08g09250	LOC_Os11g14910	9.97069867
LOC_Os08g10510	LOC_Os08g44810	11.8057352
LOC_Os08g15030	LOC_Os10g39840	8.83141238
LOC_Os08g10510	LOC_Os12g44350	9.46100124
LOC_Os08g28190	LOC_Os10g41689	9.1141606
LOC_Os08g28190	LOC_Os08g32870	9.51803393
LOC_Os08g28730	LOC_Os10g28320	9.46625804
LOC_Os08g25734	LOC_Os10g38229	8.98583897
LOC_Os08g28680	LOC_Os08g40740	9.01523512
LOC_Os08g28820	LOC_Os11g19220	11.2894408
LOC_Os08g28800	LOC_Os12g42280	9.20789548
LOC_Os08g16910	LOC_Os09g39810	9.47311905
LOC_Os08g25734	LOC_Os12g22680	10.3286056
LOC_Os08g17500	LOC_Os09g08072	10.4627849
LOC_Os08g20270	LOC_Os10g28120	11.3310248
LOC_Os08g15292	LOC_Os09g28420	10.4763434
LOC_Os08g25570	LOC_Os09g23560	9.28846653
LOC_Os08g27840	LOC_Os10g28050	9.93818242
LOC_Os08g20270	LOC_Os09g25390	9.07540239
LOC_Os08g28190	LOC_Os10g32680	11.355248
LOC_Os08g25570	LOC_Os12g34380	8.82107975
LOC_Os08g23730	LOC_Os10g31950	8.88419498
LOC_Os08g28190	LOC_Os10g38150	9.55003524
LOC_Os08g27840	LOC_Os10g25674	10.1363395
LOC_Os08g27840	LOC_Os08g39870	8.80849466
LOC_Os08g28190	LOC_Os08g28820	9.80366583
LOC_Os08g25734	LOC_Os10g39120	9.72020997
LOC_Os08g28820	LOC_Os12g38760	9.62866932

InteractorA	InteractorB	Z score
LOC_Os08g23730	LOC_Os09g28400	11.7191428
LOC_Os08g17784	LOC_Os12g22680	10.5725674
LOC_Os08g15292	LOC_Os12g10730	8.83194003
LOC_Os08g28800	LOC_Os10g23900	10.1763177
LOC_Os08g28820	LOC_Os10g37210	9.47451854
LOC_Os08g28820	LOC_Os12g44350	10.568458
LOC_Os08g17500	LOC_Os12g12470	8.87447931
LOC_Os08g20270	LOC_Os08g23730	10.1360821
LOC_Os08g28820	LOC_Os10g05069	8.99708291
LOC_Os08g16910	LOC_Os09g10270	9.78185948
LOC_Os08g28820	LOC_Os11g31620	9.03453195
LOC_Os08g28730	LOC_Os08g29170	9.85445029
LOC_Os08g20270	LOC_Os08g28820	9.05711135
LOC_Os08g17500	LOC_Os09g10300	8.84294806
LOC_Os08g23730	LOC_Os11g36719	8.83965554
LOC_Os08g27840	LOC_Os12g02980	9.77763151
LOC_Os08g28680	LOC_Os12g13380	9.12935207
LOC_Os08g28820	LOC_Os11g19800	10.6148138
LOC_Os08g25734	LOC_Os12g12560	10.114218
LOC_Os08g25570	LOC_Os10g42280	9.05655465
LOC_Os08g16910	LOC_Os12g06620	10.6225347
LOC_Os08g28190	LOC_Os12g02080	9.40786846
LOC_Os08g25570	LOC_Os11g03290	9.22624208
LOC_Os08g27840	LOC_Os10g38690	9.21092014
LOC_Os08g23730	LOC_Os12g16200	8.90335388
LOC_Os08g25734	LOC_Os12g12580	9.34385242
LOC_Os08g15292	LOC_Os10g41480	9.57547871
LOC_Os08g25734	LOC_Os12g18900	9.40568737
LOC_Os08g25734	LOC_Os11g01010	9.55204762
LOC_Os08g17500	LOC_Os08g43190	9.08827725
LOC_Os08g28190	LOC_Os10g38780	8.86710035
LOC_Os08g27840	LOC_Os09g38620	9.61828751
LOC_Os08g28680	LOC_Os10g02480	8.87545906
LOC_Os08g25570	LOC_Os10g33800	9.15552948
LOC_Os08g15292	LOC_Os12g34062	9.02888816
LOC_Os08g23730	LOC_Os09g10270	9.49296899
LOC_Os08g15322	LOC_Os12g41220	9.67764773
LOC_Os08g15322	LOC_Os12g10570	9.65922781
LOC_Os08g28680	LOC_Os10g22450	9.3136248
LOC_Os08g28820	LOC_Os08g42730	9.22080057
LOC_Os08g28820	LOC_Os10g38690	9.11979155
LOC_Os08g15292	LOC_Os10g25950	9.21005221
LOC_Os08g28820	LOC_Os08g34790	10.8477344

InteractorA	InteractorB	Z score
LOC_Os08g15292	LOC_Os10g23900	9.74335655
LOC_Os08g28800	LOC_Os12g38180	10.0956961
LOC_Os08g17500	LOC_Os10g27174	9.12539338
LOC_Os08g16910	LOC_Os09g36930	8.98454578
LOC_Os08g28820	LOC_Os11g25700	8.98297875
LOC_Os08g17680	LOC_Os11g25700	8.80536803
LOC_Os08g16910	LOC_Os09g39440	9.05810241
LOC_Os08g28800	LOC_Os09g11230	8.97015832
LOC_Os08g28730	LOC_Os09g10200	8.94790308
LOC_Os08g17680	LOC_Os11g26850	8.86984092
LOC_Os08g15322	LOC_Os12g38750	8.96503255
LOC_Os08g28800	LOC_Os12g42884	9.05747494
LOC_Os08g25570	LOC_Os11g08330	9.56147758
LOC_Os08g16910	LOC_Os09g23560	10.0950396
LOC_Os08g20270	LOC_Os10g25130	9.04917263
LOC_Os08g20730	LOC_Os12g38180	9.74452045
LOC_Os08g20270	LOC_Os09g24990	10.1832853
LOC_Os08g20270	LOC_Os09g08072	9.12455029
LOC_Os08g28680	LOC_Os12g44020	10.3124496
LOC_Os08g20420	LOC_Os11g33240	8.8893531
LOC_Os08g23730	LOC_Os10g41689	9.23751914
LOC_Os08g20420	LOC_Os08g27840	9.95018865
LOC_Os08g27840	LOC_Os10g07616	9.33210947
LOC_Os08g20270	LOC_Os09g31430	9.75799386
LOC_Os08g27840	LOC_Os10g38700	10.8882678
LOC_Os08g16910	LOC_Os09g10300	10.3842814
LOC_Os08g25734	LOC_Os09g37949	8.88972607
LOC_Os08g25570	LOC_Os10g11810	8.8688825
LOC_Os08g28190	LOC_Os12g43370	9.19697288
LOC_Os08g28820	LOC_Os09g27420	9.35081846
LOC_Os08g28800	LOC_Os09g24530	8.8103499
LOC_Os08g28800	LOC_Os11g32610	8.90257571
LOC_Os08g25570	LOC_Os09g24530	8.90412645
LOC_Os08g33820	LOC_Os11g08340	11.1525114
LOC_Os08g35740	LOC_Os09g24990	10.7093519
LOC_Os08g34280	LOC_Os10g22450	8.89903534
LOC_Os08g33370	LOC_Os11g26860	9.44491574
LOC_Os08g31060	LOC_Os09g23300	10.0227428
LOC_Os08g34790	LOC_Os08g37800	9.53263085
LOC_Os08g34280	LOC_Os09g23530	9.15597826
LOC_Os08g33100	LOC_Os12g43630	8.97182641
LOC_Os08g33820	LOC_Os10g23900	9.44894502
LOC_Os08g29170	LOC_Os10g32970	8.99741783

InteractorA	InteractorB	Z score
LOC_Os08g34170	LOC_Os09g27750	9.19579585
LOC_Os08g33370	LOC_Os08g34790	10.0587142
LOC_Os08g34790	LOC_Os11g02440	9.35264491
LOC_Os08g34290	LOC_Os09g11230	10.3756479
LOC_Os08g35420	LOC_Os09g04050	9.48116783
LOC_Os08g35440	LOC_Os08g37490	9.94357414
LOC_Os08g29370	LOC_Os09g39500	9.79093828
LOC_Os08g35440	LOC_Os10g21324	8.974716
LOC_Os08g31870	LOC_Os10g23900	9.40491453
LOC_Os08g32620	LOC_Os08g42730	9.68083206
LOC_Os08g31060	LOC_Os10g30200	9.0234645
LOC_Os08g33820	LOC_Os12g34450	10.5403904
LOC_Os08g34790	LOC_Os11g05290	9.5226076
LOC_Os08g29370	LOC_Os12g01922	9.303375
LOC_Os08g34790	LOC_Os10g34520	9.01911652
LOC_Os08g31060	LOC_Os12g13810	9.15920317
LOC_Os08g31060	LOC_Os08g43560	10.601525
LOC_Os08g32850	LOC_Os11g08445	9.3417834
LOC_Os08g31870	LOC_Os12g10600	10.6257482
LOC_Os08g34170	LOC_Os12g12590	9.00715743
LOC_Os08g33820	LOC_Os09g23540	9.2739904
LOC_Os08g34170	LOC_Os09g32640	9.29781553
LOC_Os08g33820	LOC_Os12g16410	9.03268288
LOC_Os08g31060	LOC_Os09g11230	11.00473
LOC_Os08g31060	LOC_Os11g32520	9.31042164
LOC_Os08g31060	LOC_Os11g16590	9.69476148
LOC_Os08g35420	LOC_Os10g28320	8.95723253
LOC_Os08g31060	LOC_Os12g08270	9.68738241
LOC_Os08g33820	LOC_Os11g16590	10.2142219
LOC_Os08g32620	LOC_Os10g08670	9.67502196
LOC_Os08g33370	LOC_Os08g44350	8.88253276
LOC_Os08g32620	LOC_Os10g35070	9.58059019
LOC_Os08g33370	LOC_Os11g10480	10.1950387
LOC_Os08g31870	LOC_Os11g37640	9.72060858
LOC_Os08g32620	LOC_Os11g03230	9.8159245
LOC_Os08g34280	LOC_Os11g24560	8.98072456
LOC_Os08g32620	LOC_Os08g40740	8.92699605
LOC_Os08g31870	LOC_Os09g27420	9.37049363
LOC_Os08g33710	LOC_Os12g25700	9.74182537
LOC_Os08g29370	LOC_Os11g37950	8.94069604
LOC_Os08g33710	LOC_Os10g38234	9.53115863
LOC_Os08g33820	LOC_Os11g01872	9.41118683
LOC_Os08g34290	LOC_Os12g02980	9.09540313

InteractorA	InteractorB	Z score
LOC_Os08g34280	LOC_Os10g11140	9.63064916
LOC_Os08g33370	LOC_Os12g07980	9.64838513
LOC_Os08g35740	LOC_Os09g28420	9.49156318
LOC_Os08g32620	LOC_Os09g09520	9.00485564
LOC_Os08g31060	LOC_Os11g08120	9.31788949
LOC_Os08g29370	LOC_Os10g08620	10.5974271
LOC_Os08g33100	LOC_Os12g14070	9.55369927
LOC_Os08g32870	LOC_Os11g43960	9.09624574
LOC_Os08g33710	LOC_Os10g39170	10.1815176
LOC_Os08g32850	LOC_Os12g14070	9.4198124
LOC_Os08g34210	LOC_Os09g15420	8.99594335
LOC_Os08g35420	LOC_Os10g38360	8.89120048
LOC_Os08g34280	LOC_Os10g21310	8.93925664
LOC_Os08g29520	LOC_Os09g27750	10.5018218
LOC_Os08g33370	LOC_Os12g06660	9.07075176
LOC_Os08g34210	LOC_Os11g14220	9.07881305
LOC_Os08g34170	LOC_Os11g29190	8.81837254
LOC_Os08g35420	LOC_Os12g16410	9.84537093
LOC_Os08g34790	LOC_Os10g09860	9.25404618
LOC_Os08g29370	LOC_Os12g10570	9.168248
LOC_Os08g35420	LOC_Os11g26860	8.86084107
LOC_Os08g33370	LOC_Os12g07720	8.97786082
LOC_Os08g35420	LOC_Os09g10200	9.48188578
LOC_Os08g33710	LOC_Os12g02060	9.12152691
LOC_Os08g34210	LOC_Os09g39500	9.37058074
LOC_Os08g34170	LOC_Os08g40740	9.03399776
LOC_Os08g29520	LOC_Os10g32970	9.25725086
LOC_Os08g35740	LOC_Os08g42560	8.92485414
LOC_Os08g34210	LOC_Os10g35070	9.02948577
LOC_Os08g34210	LOC_Os11g43360	8.88478193
LOC_Os08g32850	LOC_Os09g10230	10.1468955
LOC_Os08g34790	LOC_Os09g30418	10.3176021
LOC_Os08g34170	LOC_Os11g30290	8.98471016
LOC_Os08g34290	LOC_Os12g34380	10.4728085
LOC_Os08g34290	LOC_Os09g10270	9.7423961
LOC_Os08g34280	LOC_Os09g26380	9.57266283
LOC_Os08g33370	LOC_Os12g17910	9.88585835
LOC_Os08g32620	LOC_Os11g31530	10.1326784
LOC_Os08g34280	LOC_Os10g29620	9.16237102
LOC_Os08g33100	LOC_Os11g34570	11.6138062
LOC_Os08g35740	LOC_Os09g09230	8.84246256
LOC_Os08g35440	LOC_Os10g26110	12.2246983
LOC_Os08g34210	LOC_Os09g28420	8.80481335

InteractorA	InteractorB	Z score
LOC_Os08g32620	LOC_Os08g44350	8.99613397
LOC_Os08g33370	LOC_Os11g43960	9.86605655
LOC_Os08g32620	LOC_Os09g23550	8.87703758
LOC_Os08g34170	LOC_Os11g34570	9.78036212
LOC_Os08g33710	LOC_Os12g38760	10.5615255
LOC_Os08g32620	LOC_Os12g16410	9.64110005
LOC_Os08g34280	LOC_Os11g08470	10.3126704
LOC_Os08g31870	LOC_Os10g34760	9.1983091
LOC_Os08g33370	LOC_Os11g34450	9.2389696
LOC_Os08g29520	LOC_Os12g03816	9.33578308
LOC_Os08g34170	LOC_Os10g08670	11.1434894
LOC_Os08g40170	LOC_Os11g01872	10.1058834
LOC_Os08g37490	LOC_Os11g02440	9.58561248
LOC_Os08g36900	LOC_Os11g32510	8.83251867
LOC_Os08g36910	LOC_Os10g38489	9.67153577
LOC_Os08g37800	LOC_Os12g23170	9.90966115
LOC_Os08g38300	LOC_Os12g17910	10.6157617
LOC_Os08g38300	LOC_Os09g30418	9.0251527
LOC_Os08g41340	LOC_Os10g26390	10.0186189
LOC_Os08g41830	LOC_Os11g04880	9.52240346
LOC_Os08g37800	LOC_Os12g40830	9.43431245
LOC_Os08g36910	LOC_Os11g25700	9.61111185
LOC_Os08g37800	LOC_Os10g38690	9.30920683
LOC_Os08g40740	LOC_Os10g01570	9.09897806
LOC_Os08g40140	LOC_Os12g02080	9.11092749
LOC_Os08g40930	LOC_Os10g08710	8.89736683
LOC_Os08g37490	LOC_Os11g47580	8.80550203
LOC_Os08g40140	LOC_Os10g14150	9.21884297
LOC_Os08g36910	LOC_Os08g39860	10.1777332
LOC_Os08g35740	LOC_Os12g25690	9.24124467
LOC_Os08g36910	LOC_Os10g01570	9.02439778
LOC_Os08g36900	LOC_Os10g38630	9.04479531
LOC_Os08g38920	LOC_Os10g28120	9.62923591
LOC_Os08g37800	LOC_Os09g20820	9.59806389
LOC_Os08g37800	LOC_Os09g38620	9.85251132
LOC_Os08g38920	LOC_Os12g42876	9.57677437
LOC_Os08g35740	LOC_Os11g25220	9.58271581
LOC_Os08g39860	LOC_Os09g10270	10.1580651
LOC_Os08g41340	LOC_Os11g10510	8.89126019
LOC_Os08g37790	LOC_Os10g37060	12.3412269
LOC_Os08g36910	LOC_Os11g32650	9.20279018
LOC_Os08g38300	LOC_Os12g25120	9.738744
LOC_Os08g38300	LOC_Os11g25330	9.31004067

InteractorA	InteractorB	Z score
LOC_Os08g40140	LOC_Os10g35070	9.15279182
LOC_Os08g40170	LOC_Os09g23540	9.72992662
LOC_Os08g40740	LOC_Os10g01540	8.82509122
LOC_Os08g37490	LOC_Os12g13380	9.87083301
LOC_Os08g39420	LOC_Os10g11810	10.3657207
LOC_Os08g37800	LOC_Os10g38710	9.2189793
LOC_Os08g40740	LOC_Os12g17910	9.26057243
LOC_Os08g40170	LOC_Os11g36719	9.27016179
LOC_Os08g39870	LOC_Os11g19220	9.7329794
LOC_Os08g39300	LOC_Os09g28460	9.20074076
LOC_Os08g36900	LOC_Os10g38350	9.68338858
LOC_Os08g40140	LOC_Os12g43630	8.83659938
LOC_Os08g37490	LOC_Os12g01922	9.03130934
LOC_Os08g39870	LOC_Os11g32520	8.97749973
LOC_Os08g35740	LOC_Os10g40710	9.07810857
LOC_Os08g40170	LOC_Os12g12514	9.24592774
LOC_Os08g40140	LOC_Os11g02130	9.39921402
LOC_Os08g35740	LOC_Os12g06660	9.49186424
LOC_Os08g39140	LOC_Os11g31620	10.0487181
LOC_Os08g36320	LOC_Os11g47560	9.48646844
LOC_Os08g36910	LOC_Os10g35840	8.81923267
LOC_Os08g36320	LOC_Os12g34380	8.90541074
LOC_Os08g40930	LOC_Os10g30840	10.0293573
LOC_Os08g41340	LOC_Os10g26600	9.13325681
LOC_Os08g39870	LOC_Os12g12560	9.20669064
LOC_Os08g36900	LOC_Os12g12560	9.11169447
LOC_Os08g38300	LOC_Os10g26390	9.21004693
LOC_Os08g39300	LOC_Os09g07830	9.00267674
LOC_Os08g37490	LOC_Os10g26600	10.2665545
LOC_Os08g41340	LOC_Os10g29620	9.20475003
LOC_Os08g38900	LOC_Os08g38920	10.1097686
LOC_Os08g35740	LOC_Os09g37100	9.3365934
LOC_Os08g39140	LOC_Os12g38770	9.08669024
LOC_Os08g39420	LOC_Os09g17740	9.31172617
LOC_Os08g41340	LOC_Os09g36830	8.87825683
LOC_Os08g39140	LOC_Os12g22650	9.63310524
LOC_Os08g41830	LOC_Os12g07720	8.89290488
LOC_Os08g41340	LOC_Os11g26860	9.14085432
LOC_Os08g39300	LOC_Os10g39880	11.0108436
LOC_Os08g37490	LOC_Os08g40140	9.20757355
LOC_Os08g41340	LOC_Os12g12590	9.05572697
LOC_Os08g39140	LOC_Os10g29470	8.90730052
LOC_Os08g40740	LOC_Os09g31490	8.886185

InteractorA	InteractorB	Z score
LOC_Os08g37790	LOC_Os10g35840	9.24970924
LOC_Os08g38920	LOC_Os12g22680	10.3812229
LOC_Os08g38900	LOC_Os12g16410	9.11831421
LOC_Os08g39420	LOC_Os08g40140	9.33640761
LOC_Os08g38300	LOC_Os12g22650	9.65325652
LOC_Os08g41340	LOC_Os10g28120	9.34136571
LOC_Os08g39870	LOC_Os11g26910	9.19182926
LOC_Os08g40140	LOC_Os12g35570	9.69180209
LOC_Os08g40740	LOC_Os11g03400	9.43579586
LOC_Os08g40140	LOC_Os09g25320	9.60343262
LOC_Os08g40140	LOC_Os11g32540	8.8930202
LOC_Os08g38920	LOC_Os12g25710	8.82558001
LOC_Os08g37800	LOC_Os12g31640	9.33569616
LOC_Os08g37800	LOC_Os12g43370	9.24412125
LOC_Os08g40930	LOC_Os09g20260	9.24152016
LOC_Os08g37800	LOC_Os09g25390	9.9519058
LOC_Os08g36910	LOC_Os12g02980	9.11483114
LOC_Os08g40740	LOC_Os12g03090	9.43579586
LOC_Os08g37490	LOC_Os09g31120	11.778458
LOC_Os08g37800	LOC_Os09g31490	8.80421058
LOC_Os08g40170	LOC_Os09g36830	11.4913542
LOC_Os08g40740	LOC_Os09g24990	10.0460107
LOC_Os08g40140	LOC_Os11g47580	9.95818252
LOC_Os08g36910	LOC_Os10g25130	8.83404443
LOC_Os08g40140	LOC_Os10g30840	9.18291895
LOC_Os08g40140	LOC_Os11g02440	9.6686224
LOC_Os08g41830	LOC_Os10g30840	9.3320304
LOC_Os08g40930	LOC_Os12g12590	8.92100466
LOC_Os08g41830	LOC_Os10g32680	11.8053869
LOC_Os08g39860	LOC_Os09g16910	9.13542321
LOC_Os08g37800	LOC_Os12g13380	9.10548484
LOC_Os08g37790	LOC_Os09g25390	9.22640006
LOC_Os08g38920	LOC_Os09g12660	8.99290396
LOC_Os08g38900	LOC_Os10g22310	9.61226548
LOC_Os08g40140	LOC_Os10g26110	10.0521599
LOC_Os08g38300	LOC_Os09g31490	8.82796546
LOC_Os08g37800	LOC_Os10g38140	10.4729998
LOC_Os08g39870	LOC_Os10g26600	9.37684378
LOC_Os08g37790	LOC_Os09g04050	8.97447988
LOC_Os08g40740	LOC_Os11g32650	10.0889555
LOC_Os08g38900	LOC_Os09g23300	8.83931469
LOC_Os08g37800	LOC_Os10g28360	8.88929864
LOC_Os08g37800	LOC_Os11g01010	9.50542605

InteractorA	InteractorB	Z score
LOC_Os08g38920	LOC_Os09g10300	9.83714122
LOC_Os08g37490	LOC_Os12g40550	10.5634897
LOC_Os08g37800	LOC_Os10g23900	9.05824489
LOC_Os08g37790	LOC_Os09g31490	9.82410691
LOC_Os08g39870	LOC_Os10g11810	9.83020544
LOC_Os08g38300	LOC_Os12g38770	9.18578342
LOC_Os08g37800	LOC_Os12g02980	9.21151966
LOC_Os08g41340	LOC_Os09g24990	8.97487497
LOC_Os08g35740	LOC_Os10g05069	9.13546132
LOC_Os08g40140	LOC_Os11g10510	10.2169598
LOC_Os08g37490	LOC_Os12g14070	9.45678402
LOC_Os08g39860	LOC_Os10g30840	9.14763067
LOC_Os08g38920	LOC_Os09g31502	9.94091564
LOC_Os08g37800	LOC_Os12g33946	9.05095396
LOC_Os08g37800	LOC_Os11g06390	10.0788544
LOC_Os08g40930	LOC_Os09g36830	8.88177263
LOC_Os08g37800	LOC_Os09g38030	9.69601993
LOC_Os08g37790	LOC_Os11g14220	9.02944952
LOC_Os08g40930	LOC_Os10g31950	9.67521945
LOC_Os08g39870	LOC_Os12g12514	8.88503929
LOC_Os08g41830	LOC_Os09g36710	9.21134328
LOC_Os08g40140	LOC_Os08g43170	8.95172338
LOC_Os08g37800	LOC_Os11g37550	9.62007637
LOC_Os08g40140	LOC_Os12g41110	10.3445846
LOC_Os08g43170	LOC_Os09g34960	10.6277886
LOC_Os08g42560	LOC_Os09g08120	9.95595087
LOC_Os08g42410	LOC_Os09g28400	9.09644462
LOC_Os08g41830	LOC_Os12g44030	9.3264836
LOC_Os08g44270	LOC_Os12g08280	9.36994273
LOC_Os08g44270	LOC_Os10g21212	9.0597048
LOC_Os08g44340	LOC_Os12g10730	10.3120319
LOC_Os08g44520	LOC_Os10g20910	8.84973465
LOC_Os08g42410	LOC_Os10g38350	9.49106035
LOC_Os08g44340	LOC_Os11g40140	8.856382
LOC_Os08g44280	LOC_Os10g38340	9.14637862
LOC_Os08g42910	LOC_Os09g07830	8.83990774
LOC_Os08g44520	LOC_Os10g08022	8.83207942
LOC_Os08g41990	LOC_Os10g27174	10.1794509
LOC_Os08g43170	LOC_Os09g28400	9.26485977
LOC_Os08g44270	LOC_Os09g23540	10.1420995
LOC_Os08g42000	LOC_Os11g04954	9.49247046
LOC_Os08g44960	LOC_Os10g08022	9.00748099
LOC_Os08g44520	LOC_Os09g08072	9.1564766

InteractorA	InteractorB	Z score
LOC_Os08g42910	LOC_Os10g02070	8.971026
LOC_Os08g41880	LOC_Os11g08330	9.57544402
LOC_Os08g41880	LOC_Os11g01872	9.92273947
LOC_Os08g42410	LOC_Os11g41130	9.06442107
LOC_Os08g42560	LOC_Os10g26600	12.5119122
LOC_Os08g41880	LOC_Os09g39440	9.08708405
LOC_Os08g44520	LOC_Os10g38660	8.87018912
LOC_Os08g44340	LOC_Os09g31506	10.0768481
LOC_Os08g44810	LOC_Os09g24530	9.05058751
LOC_Os08g43170	LOC_Os09g07460	9.39155519
LOC_Os08g42730	LOC_Os09g15320	9.31957605
LOC_Os08g42560	LOC_Os12g17910	9.22675051
LOC_Os08g43170	LOC_Os09g37100	9.45884587
LOC_Os08g43560	LOC_Os12g43100	8.9760096
LOC_Os08g43190	LOC_Os10g28120	8.86550533
LOC_Os08g43190	LOC_Os11g30290	9.10769262
LOC_Os08g41880	LOC_Os08g44270	9.43312277
LOC_Os08g44960	LOC_Os09g23550	9.00071051
LOC_Os08g44810	LOC_Os11g03230	10.8899639
LOC_Os08g42540	LOC_Os11g25260	9.31453812
LOC_Os08g41880	LOC_Os12g07830	8.90444171
LOC_Os08g43170	LOC_Os10g31940	9.43329564
LOC_Os08g41880	LOC_Os09g30418	8.97943924
LOC_Os09g04050	LOC_Os10g23100	8.86698212
LOC_Os08g41880	LOC_Os12g44150	9.09474707
LOC_Os08g44340	LOC_Os10g28360	9.08059246
LOC_Os08g44530	LOC_Os10g21240	9.14174283
LOC_Os08g43560	LOC_Os09g23560	9.73499489
LOC_Os08g41880	LOC_Os08g44210	10.1398681
LOC_Os08g44810	LOC_Os12g13800	9.83334672
LOC_Os08g44280	LOC_Os12g43630	8.93866496
LOC_Os08g41880	LOC_Os10g23900	11.6662468
LOC_Os08g44810	LOC_Os10g40710	9.504742
LOC_Os08g44340	LOC_Os11g33240	9.44522434
LOC_Os08g43170	LOC_Os09g23550	12.8350203
LOC_Os08g44530	LOC_Os09g12230	8.87287872
LOC_Os08g44340	LOC_Os11g47550	9.55148274
LOC_Os08g42540	LOC_Os10g26130	9.8219786
LOC_Os08g44530	LOC_Os12g40510	8.93554784
LOC_Os08g42910	LOC_Os11g33240	9.27435813
LOC_Os08g44340	LOC_Os10g38234	8.86518419
LOC_Os08g43190	LOC_Os10g42280	10.1557055
LOC_Os08g41880	LOC_Os12g10730	9.02674105

InteractorA	InteractorB	Z score
LOC_Os08g41880	LOC_Os09g24412	9.41885124
LOC_Os08g42560	LOC_Os10g26010	8.99167101
LOC_Os08g41990	LOC_Os11g32260	9.28146071
LOC_Os08g44810	LOC_Os09g39570	8.88822151
LOC_Os08g42910	LOC_Os11g08460	9.30991652
LOC_Os09g04050	LOC_Os10g38150	8.86071044
LOC_Os08g44530	LOC_Os11g08330	9.28747602
LOC_Os08g44340	LOC_Os12g06620	8.9690293
LOC_Os08g44810	LOC_Os12g34380	9.47216652
LOC_Os08g42560	LOC_Os09g24924	8.81029292
LOC_Os08g42560	LOC_Os10g02380	9.94763955
LOC_Os08g44960	LOC_Os09g28420	9.64348684
LOC_Os08g42410	LOC_Os12g12514	9.02913093
LOC_Os08g42560	LOC_Os10g41510	9.12458751
LOC_Os08g42560	LOC_Os12g33610	8.99547332
LOC_Os08g44530	LOC_Os09g36900	9.74349855
LOC_Os08g42910	LOC_Os11g08440	9.36614245
LOC_Os08g43190	LOC_Os09g32810	9.01788307
LOC_Os08g44280	LOC_Os11g33240	9.66342033
LOC_Os08g43560	LOC_Os12g12514	9.62791679
LOC_Os08g41880	LOC_Os11g31620	9.00287118
LOC_Os08g42410	LOC_Os09g33500	12.8578623
LOC_Os08g42560	LOC_Os12g22030	9.35846125
LOC_Os08g42560	LOC_Os11g08340	9.80311058
LOC_Os08g41880	LOC_Os10g21352	9.41887433
LOC_Os08g44530	LOC_Os11g24560	9.00713886
LOC_Os08g43170	LOC_Os12g12514	8.82046053
LOC_Os08g44530	LOC_Os12g25700	9.19385048
LOC_Os08g44530	LOC_Os12g12590	10.1662755
LOC_Os08g44340	LOC_Os09g25390	8.89392895
LOC_Os08g44530	LOC_Os11g10480	9.00836014
LOC_Os09g08910	LOC_Os11g32650	9.87936536
LOC_Os09g08120	LOC_Os10g41550	8.81537105
LOC_Os09g11230	LOC_Os10g42280	9.74140724
LOC_Os09g04680	LOC_Os12g40510	8.81001829
LOC_Os09g10200	LOC_Os10g20910	9.07759831
LOC_Os09g08072	LOC_Os10g01570	9.08413244
LOC_Os09g10230	LOC_Os11g37960	8.82532012
LOC_Os09g10270	LOC_Os12g06620	9.67606464
LOC_Os09g08720	LOC_Os12g13800	8.80538252
LOC_Os09g08120	LOC_Os10g38340	9.70254315
LOC_Os09g10200	LOC_Os11g19220	8.9330974
LOC_Os09g04050	LOC_Os12g41110	9.06324644

InteractorA	InteractorB	Z score
LOC_Os09g10270	LOC_Os12g13390	9.50644732
LOC_Os09g04050	LOC_Os12g06620	10.3011327
LOC_Os09g04730	LOC_Os10g03540	11.6401121
LOC_Os09g12570	LOC_Os10g38340	9.30410051
LOC_Os09g09270	LOC_Os10g32550	9.13937145
LOC_Os09g10270	LOC_Os10g07616	9.55559702
LOC_Os09g11230	LOC_Os11g32610	8.95058833
LOC_Os09g10300	LOC_Os11g32580	9.1927737
LOC_Os09g10300	LOC_Os12g02980	9.25136185
LOC_Os09g08072	LOC_Os10g28320	11.1563734
LOC_Os09g07460	LOC_Os12g16290	9.37272126
LOC_Os09g07830	LOC_Os09g39380	8.93266511
LOC_Os09g10230	LOC_Os10g25930	9.18493372
LOC_Os09g07510	LOC_Os09g27820	11.290788
LOC_Os09g10300	LOC_Os11g32650	9.02414378
LOC_Os09g10230	LOC_Os12g44010	9.7672792
LOC_Os09g12570	LOC_Os11g16590	9.26233011
LOC_Os09g10270	LOC_Os12g16250	9.36994605
LOC_Os09g11230	LOC_Os11g14910	9.00793148
LOC_Os09g04730	LOC_Os09g27750	8.93564919
LOC_Os09g07510	LOC_Os10g26390	10.2570941
LOC_Os09g04680	LOC_Os10g41510	12.4090448
LOC_Os09g08720	LOC_Os11g47570	8.84983228
LOC_Os09g07830	LOC_Os09g31490	11.0583823
LOC_Os09g07510	LOC_Os09g37100	9.23384884
LOC_Os09g10260	LOC_Os10g26110	10.1204988
LOC_Os09g07830	LOC_Os11g02600	8.8324828
LOC_Os09g10260	LOC_Os09g31502	8.88219638
LOC_Os09g12570	LOC_Os10g42940	8.84694664
LOC_Os09g08720	LOC_Os09g23530	11.4539767
LOC_Os09g08910	LOC_Os09g36830	9.97832907
LOC_Os09g10200	LOC_Os10g42280	9.2217127
LOC_Os09g08120	LOC_Os12g13800	9.20155697
LOC_Os09g11230	LOC_Os12g25690	8.96844267
LOC_Os09g12570	LOC_Os09g27750	9.21465637
LOC_Os09g10230	LOC_Os11g33240	9.13580712
LOC_Os09g07510	LOC_Os10g21192	9.29574486
LOC_Os09g07830	LOC_Os09g23530	9.68129119
LOC_Os09g04680	LOC_Os09g23560	8.81395814
LOC_Os09g10260	LOC_Os10g29470	10.4657732
LOC_Os09g09270	LOC_Os11g33240	8.98023898
LOC_Os09g04050	LOC_Os11g25330	8.93365523
LOC_Os09g07830	LOC_Os10g38740	8.94784117

InteractorA	InteractorB	Z score
LOC_Os09g04730	LOC_Os12g34450	9.15261946
LOC_Os09g10270	LOC_Os11g08340	9.15015663
LOC_Os09g10270	LOC_Os10g30580	10.5032148
LOC_Os09g07830	LOC_Os12g12590	8.96536778
LOC_Os09g08910	LOC_Os09g24412	9.21414864
LOC_Os09g10270	LOC_Os09g36930	9.84782357
LOC_Os09g09230	LOC_Os10g38470	9.79631307
LOC_Os09g10270	LOC_Os11g39540	10.9615841
LOC_Os09g10230	LOC_Os10g34520	9.27482247
LOC_Os09g08072	LOC_Os10g02040	9.09556512
LOC_Os09g09520	LOC_Os12g04980	9.59936589
LOC_Os09g08720	LOC_Os10g38150	9.30202341
LOC_Os09g10260	LOC_Os09g33850	9.08844466
LOC_Os09g10260	LOC_Os10g40720	9.14597713
LOC_Os09g08910	LOC_Os11g03980	9.19595793
LOC_Os09g10230	LOC_Os09g12230	9.57242332
LOC_Os09g12230	LOC_Os09g23560	9.16285829
LOC_Os09g10260	LOC_Os10g40710	11.4412821
LOC_Os09g08910	LOC_Os10g22450	12.1975698
LOC_Os09g07830	LOC_Os10g35110	8.92995868
LOC_Os09g07830	LOC_Os12g03816	9.37976329
LOC_Os09g11230	LOC_Os11g14040	9.03680935
LOC_Os09g09230	LOC_Os09g12570	9.73843547
LOC_Os09g07460	LOC_Os09g30418	8.98203065
LOC_Os09g08072	LOC_Os11g06390	9.12235592
LOC_Os09g09230	LOC_Os11g07440	8.81760571
LOC_Os09g12660	LOC_Os10g02480	9.05975556
LOC_Os09g07460	LOC_Os12g42280	9.15507113
LOC_Os09g10230	LOC_Os09g39570	9.46662863
LOC_Os09g10270	LOC_Os12g26290	9.12438331
LOC_Os09g12230	LOC_Os12g13320	9.12498919
LOC_Os09g07830	LOC_Os09g23540	9.3312964
LOC_Os09g10270	LOC_Os11g32610	12.2231915
LOC_Os09g10230	LOC_Os11g43960	8.82398648
LOC_Os09g08880	LOC_Os11g32610	8.92085264
LOC_Os09g10270	LOC_Os11g08940	9.12229222
LOC_Os09g08910	LOC_Os11g02440	9.78622151
LOC_Os09g04050	LOC_Os12g07720	9.41243159
LOC_Os09g08720	LOC_Os10g32680	8.84850018
LOC_Os09g08720	LOC_Os09g23540	9.06553523
LOC_Os09g08910	LOC_Os09g23550	10.3139763
LOC_Os09g08120	LOC_Os09g31506	8.94738604
LOC_Os09g10270	LOC_Os09g38620	11.1580118

InteractorA	InteractorB	Z score
LOC_Os09g08910	LOC_Os09g23540	9.20658611
LOC_Os09g08910	LOC_Os10g21352	9.21414864
LOC_Os09g09230	LOC_Os12g13380	8.8051973
LOC_Os09g10230	LOC_Os10g25130	9.28617968
LOC_Os09g04050	LOC_Os11g37640	11.8916026
LOC_Os09g09230	LOC_Os11g29190	9.08376351
LOC_Os09g10230	LOC_Os10g09860	11.2315388
LOC_Os09g11230	LOC_Os12g38760	10.6875612
LOC_Os09g11230	LOC_Os11g47550	9.19235786
LOC_Os09g10300	LOC_Os09g31506	8.87604837
LOC_Os09g10200	LOC_Os11g47600	11.1976305
LOC_Os09g07510	LOC_Os09g15420	8.87313029
LOC_Os09g12570	LOC_Os10g25140	9.07448807
LOC_Os09g10230	LOC_Os12g36950	8.93356702
LOC_Os09g08120	LOC_Os10g11810	9.06929249
LOC_Os09g12590	LOC_Os12g44010	9.40404477
LOC_Os09g08120	LOC_Os09g23550	9.03769264
LOC_Os09g08880	LOC_Os12g38750	9.21773582
LOC_Os09g04050	LOC_Os12g22030	9.58257349
LOC_Os09g12590	LOC_Os11g05730	10.6579248
LOC_Os09g08720	LOC_Os10g08580	9.13638522
LOC_Os09g08072	LOC_Os10g21342	9.19016379
LOC_Os09g12590	LOC_Os11g42350	9.23631563
LOC_Os09g10200	LOC_Os10g31950	9.82168966
LOC_Os09g04680	LOC_Os12g25690	9.98358338
LOC_Os09g07830	LOC_Os12g06660	9.12742061
LOC_Os09g12660	LOC_Os09g30360	9.84980725
LOC_Os09g11230	LOC_Os12g02980	8.8914732
LOC_Os09g10230	LOC_Os12g23170	9.08465435
LOC_Os09g12570	LOC_Os10g37210	9.02098733
LOC_Os09g08120	LOC_Os10g38160	11.7017775
LOC_Os09g23530	LOC_Os10g38229	8.99601395
LOC_Os09g21770	LOC_Os12g13800	8.84877779
LOC_Os09g14670	LOC_Os11g32260	9.25480821
LOC_Os09g15420	LOC_Os09g26880	8.94528082
LOC_Os09g23550	LOC_Os11g10480	8.82177652
LOC_Os09g15790	LOC_Os09g19560	9.05983284
LOC_Os09g23560	LOC_Os09g32952	8.85457997
LOC_Os09g20090	LOC_Os11g26860	9.08774242
LOC_Os09g23530	LOC_Os10g31950	8.90232812
LOC_Os09g19954	LOC_Os12g12514	9.41788243
LOC_Os09g23540	LOC_Os12g08280	9.77786254
LOC_Os09g15320	LOC_Os11g32620	9.12230544

InteractorA	InteractorB	Z score
LOC_Os09g23550	LOC_Os10g40710	9.34326893
LOC_Os09g24412	LOC_Os12g21798	9.12606011
LOC_Os09g19560	LOC_Os10g26010	8.92506955
LOC_Os09g24412	LOC_Os09g38030	9.58502216
LOC_Os09g23530	LOC_Os10g30840	9.18120444
LOC_Os09g21770	LOC_Os12g12590	9.07928887
LOC_Os09g21770	LOC_Os12g10720	9.55527286
LOC_Os09g23530	LOC_Os10g17680	9.08754071
LOC_Os09g24530	LOC_Os11g03290	9.42178947
LOC_Os09g23550	LOC_Os11g08120	9.56729366
LOC_Os09g21770	LOC_Os09g28770	9.11484048
LOC_Os09g23550	LOC_Os09g33860	9.00138224
LOC_Os09g23530	LOC_Os09g23560	9.5865317
LOC_Os09g12660	LOC_Os11g18870	9.59830176
LOC_Os09g23530	LOC_Os12g26290	9.25672191
LOC_Os09g23560	LOC_Os11g32510	9.46311475
LOC_Os09g12660	LOC_Os10g38540	10.0920146
LOC_Os09g21770	LOC_Os09g25370	10.234381
LOC_Os09g23560	LOC_Os11g10520	8.98440061
LOC_Os09g24530	LOC_Os09g25370	8.88992242
LOC_Os09g15320	LOC_Os12g42876	8.82959621
LOC_Os09g19560	LOC_Os10g42720	8.99154396
LOC_Os09g24530	LOC_Os12g06620	8.91623511
LOC_Os09g23530	LOC_Os11g37550	9.07375057
LOC_Os09g20820	LOC_Os12g12514	9.00661355
LOC_Os09g12660	LOC_Os10g38160	9.99113142
LOC_Os09g23550	LOC_Os09g36900	9.70351057
LOC_Os09g23550	LOC_Os10g29470	10.1904193
LOC_Os09g23530	LOC_Os09g34214	9.0197562
LOC_Os09g23530	LOC_Os09g39570	8.98009878
LOC_Os09g19954	LOC_Os11g14220	9.15654375
LOC_Os09g17740	LOC_Os11g20790	9.04311272
LOC_Os09g19560	LOC_Os12g31370	11.4034034
LOC_Os09g23530	LOC_Os10g38340	10.1856245
LOC_Os09g23530	LOC_Os12g22680	9.59301944
LOC_Os09g23560	LOC_Os10g38360	10.0072943
LOC_Os09g15320	LOC_Os09g37100	9.78275621
LOC_Os09g15320	LOC_Os11g03290	9.02775433
LOC_Os09g20820	LOC_Os10g08550	9.67482866
LOC_Os09g20220	LOC_Os10g38229	9.22898563
LOC_Os09g23550	LOC_Os12g16240	9.41302381
LOC_Os09g23530	LOC_Os10g39880	8.90693682
LOC_Os09g24412	LOC_Os11g25330	9.92153133

InteractorA	InteractorB	Z score
LOC_Os09g20090	LOC_Os09g30412	11.4829604
LOC_Os09g24530	LOC_Os10g38150	11.1291706
LOC_Os09g23550	LOC_Os10g38580	8.80829812
LOC_Os09g14670	LOC_Os11g47760	8.82643422
LOC_Os09g20090	LOC_Os11g06390	9.60042153
LOC_Os09g24530	LOC_Os10g35840	8.93090679
LOC_Os09g15320	LOC_Os12g22680	9.14834561
LOC_Os09g23300	LOC_Os09g24990	9.43164207
LOC_Os09g20220	LOC_Os12g43100	10.1052518
LOC_Os09g24412	LOC_Os10g31000	9.00579729
LOC_Os09g20260	LOC_Os11g16590	9.48255916
LOC_Os09g20820	LOC_Os10g11260	9.90175624
LOC_Os09g15400	LOC_Os10g28320	9.97787768
LOC_Os09g20260	LOC_Os10g27050	10.1489477
LOC_Os09g23530	LOC_Os10g38600	8.98970122
LOC_Os09g23560	LOC_Os09g28420	9.18407744
LOC_Os09g12660	LOC_Os10g30840	8.96300083
LOC_Os09g17740	LOC_Os10g10434	8.85298471
LOC_Os09g15320	LOC_Os10g21268	9.50336074
LOC_Os09g23530	LOC_Os12g12590	10.4025116
LOC_Os09g23560	LOC_Os10g10434	9.18507212
LOC_Os09g20284	LOC_Os11g26850	10.1065882
LOC_Os09g20284	LOC_Os12g03090	9.97440976
LOC_Os09g12660	LOC_Os10g38350	9.03785844
LOC_Os09g16910	LOC_Os09g24924	10.2525478
LOC_Os09g21770	LOC_Os11g32520	9.95642924
LOC_Os09g12660	LOC_Os12g40550	9.01518343
LOC_Os09g15420	LOC_Os11g08330	9.4807982
LOC_Os09g24530	LOC_Os10g01570	10.7852512
LOC_Os09g23560	LOC_Os11g29190	8.82235558
LOC_Os09g16910	LOC_Os10g39680	9.18312288
LOC_Os09g15420	LOC_Os10g30840	10.108969
LOC_Os09g23530	LOC_Os09g31120	9.33308622
LOC_Os09g23530	LOC_Os10g17660	9.08754071
LOC_Os09g14670	LOC_Os11g40150	8.88182101
LOC_Os09g17740	LOC_Os12g38770	9.68811801
LOC_Os09g24530	LOC_Os12g43440	8.86319511
LOC_Os09g20260	LOC_Os09g39810	10.123001
LOC_Os09g17620	LOC_Os11g25220	10.1206273
LOC_Os09g23300	LOC_Os10g25130	9.32657304
LOC_Os09g23530	LOC_Os11g19220	9.16864741
LOC_Os09g23530	LOC_Os10g41510	9.2844274
LOC_Os09g20284	LOC_Os11g03400	9.97442358

InteractorA	InteractorB	Z score
LOC_Os09g23530	LOC_Os12g34450	9.25000847
LOC_Os09g20820	LOC_Os10g05069	9.08379586
LOC_Os09g15320	LOC_Os12g44020	9.86482348
LOC_Os09g20284	LOC_Os12g13390	9.84939344
LOC_Os09g20284	LOC_Os11g42350	9.52181243
LOC_Os09g20820	LOC_Os11g48110	8.88471756
LOC_Os09g23560	LOC_Os09g28770	9.48274073
LOC_Os09g23530	LOC_Os11g34450	9.81561716
LOC_Os01g27210	LOC_Os07g03319	9.35141784
LOC_Os01g27210	LOC_Os07g03499	9.35143213
LOC_Os01g25100	LOC_Os01g56880	9.36825323
LOC_Os01g25484	LOC_Os08g39140	11.0128504
LOC_Os01g25610	LOC_Os01g55940	9.33300153
LOC_Os01g27210	LOC_Os01g32364	9.50122307
LOC_Os01g25100	LOC_Os10g32680	9.38972549
LOC_Os01g25610	LOC_Os03g21900	9.09511393
LOC_Os01g25484	LOC_Os07g48780	9.11527763
LOC_Os01g25100	LOC_Os01g44260	11.2492505
LOC_Os01g25610	LOC_Os08g15266	9.0617228
LOC_Os01g27210	LOC_Os01g60790	9.33303719
LOC_Os01g25280	LOC_Os05g23860	9.51306128
LOC_Os01g25610	LOC_Os04g33720	9.00099837
LOC_Os01g27210	LOC_Os06g34690	9.64339457
LOC_Os01g25280	LOC_Os01g62040	9.17135601
LOC_Os01g25610	LOC_Os09g24412	9.0617228
LOC_Os01g25484	LOC_Os06g12180	8.9651449
LOC_Os01g25100	LOC_Os11g01872	9.29178933
LOC_Os01g25280	LOC_Os04g33720	9.17890174
LOC_Os01g25280	LOC_Os09g30360	9.49089143
LOC_Os01g25484	LOC_Os09g34250	9.14532586
LOC_Os01g25484	LOC_Os01g41630	8.99659872
LOC_Os01g25280	LOC_Os02g16040	10.8594442
LOC_Os01g25484	LOC_Os03g50885	10.2403516
LOC_Os01g25100	LOC_Os01g48680	8.82793489
LOC_Os01g25280	LOC_Os02g18930	9.15323724
LOC_Os01g25100	LOC_Os03g59710	9.2989001
LOC_Os01g25280	LOC_Os09g10230	9.01225351
LOC_Os01g25610	LOC_Os03g06620	10.2323059
LOC_Os01g25610	LOC_Os04g16772	9.0617228
LOC_Os01g25610	LOC_Os04g01600	9.8345631
LOC_Os01g27210	LOC_Os06g35520	9.39853148
LOC_Os01g25100	LOC_Os12g24650	8.96715226
LOC_Os01g25100	LOC_Os01g71400	8.97737863

InteractorA	InteractorB	Z score
LOC_Os01g25610	LOC_Os04g38600	9.3320509
LOC_Os01g25100	LOC_Os05g33240	10.0171029
LOC_Os01g25484	LOC_Os12g07980	8.99385206
LOC_Os01g27210	LOC_Os05g49760	9.26216992
LOC_Os01g25610	LOC_Os04g16818	9.0617228
LOC_Os01g25610	LOC_Os12g34380	8.91042762
LOC_Os01g25100	LOC_Os06g11210	8.83277086
LOC_Os01g27210	LOC_Os03g59660	9.05529587
LOC_Os01g25610	LOC_Os11g01872	9.22562086
LOC_Os01g25610	LOC_Os01g71300	8.90750196
LOC_Os01g25484	LOC_Os03g48390	9.12862178
LOC_Os01g25610	LOC_Os10g21352	9.0617228
LOC_Os01g25100	LOC_Os01g27490	9.40581081
LOC_Os01g25610	LOC_Os10g01540	9.38525536
LOC_Os01g25610	LOC_Os05g23740	9.30927895
LOC_Os01g25280	LOC_Os02g21460	9.25698142
LOC_Os01g25484	LOC_Os03g20370	9.11527763
LOC_Os01g27210	LOC_Os07g03409	9.35143213
LOC_Os01g25100	LOC_Os10g03540	8.85207197
LOC_Os01g25610	LOC_Os06g36820	9.73245227
LOC_Os01g25280	LOC_Os06g11200	9.0994569
LOC_Os01g27210	LOC_Os01g67160	9.32579682
LOC_Os01g25610	LOC_Os12g17910	9.1896424
LOC_Os01g25484	LOC_Os08g44340	8.8359557
LOC_Os01g27210	LOC_Os09g31486	8.90185177
LOC_Os01g25280	LOC_Os06g10910	9.17589115
LOC_Os01g27210	LOC_Os02g53790	8.87068977
LOC_Os01g25280	LOC_Os07g26540	9.13468179
LOC_Os01g25610	LOC_Os07g25024	9.0617228
LOC_Os01g25610	LOC_Os10g07229	9.24072925
LOC_Os01g25610	LOC_Os05g39960	10.5147573
LOC_Os01g27210	LOC_Os08g17784	8.8180396
LOC_Os01g25610	LOC_Os03g45320	9.27921978
LOC_Os01g25610	LOC_Os03g44484	9.04215176
LOC_Os01g25610	LOC_Os11g04954	8.9763561
LOC_Os01g25100	LOC_Os08g20270	9.22356629
LOC_Os01g25100	LOC_Os02g08100	9.6548934
LOC_Os01g25100	LOC_Os02g08490	9.29114419
LOC_Os01g25100	LOC_Os01g67220	9.29552181
LOC_Os01g25610	LOC_Os02g32030	8.87989632
LOC_Os01g25484	LOC_Os10g38140	9.38474739
LOC_Os01g25484	LOC_Os07g48040	9.07149864
LOC_Os01g25484	LOC_Os03g20700	9.28374706

InteractorA	InteractorB	Z score
LOC_Os01g25610	LOC_Os05g03820	9.10753552
LOC_Os01g27210	LOC_Os06g45590	9.03907979
LOC_Os01g25610	LOC_Os06g36160	8.88647492
LOC_Os01g25610	LOC_Os05g48040	8.91016192
LOC_Os09g30418	LOC_Os12g25120	9.10053992
LOC_Os09g27820	LOC_Os12g12514	9.08662531
LOC_Os09g28420	LOC_Os12g08280	10.4132119
LOC_Os09g24924	LOC_Os11g25330	9.26885268
LOC_Os09g30360	LOC_Os11g41610	10.8828682
LOC_Os09g28460	LOC_Os10g21268	9.09103268
LOC_Os09g31120	LOC_Os11g08445	9.73402823
LOC_Os09g31410	LOC_Os11g14040	9.27121875
LOC_Os09g28400	LOC_Os10g25140	9.59033456
LOC_Os09g31486	LOC_Os10g25140	11.3678824
LOC_Os09g26380	LOC_Os10g39170	9.33124677
LOC_Os09g31490	LOC_Os10g38340	9.69561183
LOC_Os09g31120	LOC_Os09g36710	9.07670285
LOC_Os09g27750	LOC_Os10g36100	9.43537566
LOC_Os09g26340	LOC_Os11g10480	8.92560379
LOC_Os09g25370	LOC_Os12g13380	9.92959909
LOC_Os09g26880	LOC_Os11g08460	9.27846232
LOC_Os09g26340	LOC_Os10g39170	9.48057701
LOC_Os09g28770	LOC_Os11g34450	9.90845949
LOC_Os09g31490	LOC_Os12g24650	8.88059588
LOC_Os09g31120	LOC_Os10g25130	8.97249079
LOC_Os09g26380	LOC_Os11g20790	8.88003145
LOC_Os09g26380	LOC_Os12g44010	9.1681749
LOC_Os09g31410	LOC_Os12g43100	11.5296689
LOC_Os09g26340	LOC_Os10g01540	9.10660661
LOC_Os09g27750	LOC_Os09g28770	8.80695636
LOC_Os09g26380	LOC_Os09g34960	10.420747
LOC_Os09g25320	LOC_Os12g31640	9.41668496
LOC_Os09g26880	LOC_Os10g38140	10.2121722
LOC_Os09g26380	LOC_Os12g43450	9.03475829
LOC_Os09g26880	LOC_Os09g36830	9.24635847
LOC_Os09g25150	LOC_Os12g41110	9.73708423
LOC_Os09g26380	LOC_Os10g25140	17.0756404
LOC_Os09g31410	LOC_Os10g38140	9.7077916
LOC_Os09g24990	LOC_Os10g25950	10.2008061
LOC_Os09g27750	LOC_Os09g33500	9.24173096
LOC_Os09g25390	LOC_Os09g31120	8.89620308
LOC_Os09g31486	LOC_Os11g43960	10.4477269
LOC_Os09g28400	LOC_Os12g12560	9.02558092

InteractorA	InteractorB	Z score
LOC_Os09g25320	LOC_Os10g35480	9.10485339
LOC_Os09g28400	LOC_Os10g30580	11.5301814
LOC_Os09g25320	LOC_Os10g21266	9.07782601
LOC_Os09g26340	LOC_Os11g31620	9.02281446
LOC_Os09g29200	LOC_Os12g12514	9.72505081
LOC_Os09g25150	LOC_Os10g21310	9.15554377
LOC_Os09g31486	LOC_Os09g31506	10.2224139
LOC_Os09g30418	LOC_Os10g38189	8.92464342
LOC_Os09g26340	LOC_Os11g10520	10.9141423
LOC_Os09g26880	LOC_Os12g01922	8.99301699
LOC_Os09g25150	LOC_Os12g06620	8.96270355
LOC_Os09g28460	LOC_Os12g22650	9.05900584
LOC_Os09g28420	LOC_Os11g14910	9.58779752
LOC_Os09g28420	LOC_Os10g38350	9.68437763
LOC_Os09g26340	LOC_Os12g22680	9.95584485
LOC_Os09g25390	LOC_Os12g33946	9.19934101
LOC_Os09g26880	LOC_Os10g21250	9.37218442
LOC_Os09g26380	LOC_Os10g09860	9.46949796
LOC_Os09g31120	LOC_Os10g38730	9.5368885
LOC_Os09g30418	LOC_Os09g36800	9.88734811
LOC_Os09g26880	LOC_Os11g26910	10.9872517
LOC_Os09g26380	LOC_Os10g25130	16.5678179
LOC_Os09g28460	LOC_Os10g37060	9.40023573
LOC_Os09g28770	LOC_Os10g01080	9.53414356
LOC_Os09g31430	LOC_Os12g40510	10.9711326
LOC_Os09g26380	LOC_Os12g40550	9.95087655
LOC_Os09g31502	LOC_Os11g28340	9.05381077
LOC_Os09g31120	LOC_Os10g21240	8.83850094
LOC_Os09g28400	LOC_Os10g02380	9.07117085
LOC_Os09g25390	LOC_Os10g38234	9.64136476
LOC_Os09g25390	LOC_Os09g32840	10.397662
LOC_Os09g27820	LOC_Os11g40150	8.80256686
LOC_Os09g31430	LOC_Os12g39630	9.61519648
LOC_Os09g24990	LOC_Os10g38234	8.93337701
LOC_Os09g25390	LOC_Os11g19800	9.91151044
LOC_Os09g26880	LOC_Os11g10520	8.93547576
LOC_Os09g30412	LOC_Os10g25930	8.97239988
LOC_Os09g26340	LOC_Os10g22070	9.42850276
LOC_Os09g25390	LOC_Os11g08440	9.34788637
LOC_Os09g31490	LOC_Os10g11810	10.3794095
LOC_Os09g28770	LOC_Os10g02070	8.88981294
LOC_Os09g28420	LOC_Os10g03540	9.45552505
LOC_Os09g30360	LOC_Os12g10720	8.86807134

InteractorA	InteractorB	Z score
LOC_Os09g25390	LOC_Os10g21250	9.49067451
LOC_Os09g28420	LOC_Os09g37100	8.91153967
LOC_Os09g30418	LOC_Os11g36719	9.86022404
LOC_Os09g27820	LOC_Os10g21344	8.91621715
LOC_Os09g25390	LOC_Os12g40510	8.97618974
LOC_Os09g30418	LOC_Os12g40550	9.46224004
LOC_Os09g28420	LOC_Os12g02980	9.05287428
LOC_Os09g26380	LOC_Os11g47560	9.03882973
LOC_Os09g24990	LOC_Os09g30418	9.10258242
LOC_Os09g31430	LOC_Os12g10730	9.73906059
LOC_Os09g31430	LOC_Os12g04924	8.87121746
LOC_Os09g26380	LOC_Os10g08620	11.5835447
LOC_Os09g27750	LOC_Os11g31620	10.6738736
LOC_Os09g30360	LOC_Os12g07980	9.10459763
LOC_Os09g25370	LOC_Os12g42876	10.8107972
LOC_Os09g26380	LOC_Os10g41550	9.15177519
LOC_Os09g25370	LOC_Os11g04880	9.68716351
LOC_Os09g30418	LOC_Os10g42940	9.25476178
LOC_Os09g26380	LOC_Os10g39840	9.24871305
LOC_Os09g28400	LOC_Os10g07040	10.594105
LOC_Os09g24990	LOC_Os10g21248	9.30701092
LOC_Os09g30418	LOC_Os10g40600	9.63200064
LOC_Os09g25390	LOC_Os10g38710	9.89268117
LOC_Os09g25320	LOC_Os10g07229	10.6696577
LOC_Os09g27750	LOC_Os09g32840	8.90041399
LOC_Os09g25320	LOC_Os09g39570	9.87040221
LOC_Os09g27750	LOC_Os10g38740	9.27309244
LOC_Os09g25320	LOC_Os12g02980	8.99499252
LOC_Os09g27420	LOC_Os09g30412	10.362607
LOC_Os09g31430	LOC_Os10g42720	9.54404031
LOC_Os09g26880	LOC_Os09g31490	9.20844366
LOC_Os09g30412	LOC_Os11g29400	9.63794721
LOC_Os09g25150	LOC_Os09g28420	9.24599201
LOC_Os09g31430	LOC_Os11g02440	10.914988
LOC_Os09g27750	LOC_Os11g10480	8.92872173
LOC_Os09g26880	LOC_Os11g10480	9.86895583
LOC_Os09g27820	LOC_Os10g30580	9.24042146
LOC_Os09g25390	LOC_Os10g21240	9.08990222
LOC_Os09g28420	LOC_Os12g06620	10.6807241
LOC_Os09g26380	LOC_Os12g12514	10.3571333
LOC_Os09g31502	LOC_Os09g34250	8.83778671
LOC_Os09g28420	LOC_Os10g33800	9.19375143
LOC_Os09g30360	LOC_Os10g31950	9.39631587

InteractorA	InteractorB	Z score
LOC_Os09g28770	LOC_Os12g04980	9.16982414
LOC_Os09g26380	LOC_Os12g12590	9.00752142
LOC_Os09g24990	LOC_Os10g28080	9.2035186
LOC_Os09g28420	LOC_Os11g25220	9.52360636
LOC_Os09g28770	LOC_Os11g05570	8.84010511
LOC_Os09g26340	LOC_Os11g10510	8.86156789
LOC_Os09g31502	LOC_Os10g38470	10.2422499
LOC_Os09g26880	LOC_Os11g20790	9.02265503
LOC_Os09g31502	LOC_Os10g22450	9.58891866
LOC_Os09g27750	LOC_Os09g39780	9.92569504
LOC_Os09g28770	LOC_Os12g23630	8.92084928
LOC_Os09g31410	LOC_Os11g03290	9.80294656
LOC_Os09g26380	LOC_Os12g31640	9.95266031
LOC_Os09g26380	LOC_Os10g36650	9.02829061
LOC_Os09g32620	LOC_Os12g25710	10.2370186
LOC_Os09g32952	LOC_Os12g44000	9.56648944
LOC_Os09g34250	LOC_Os12g17910	9.03180992
LOC_Os09g36900	LOC_Os11g32260	9.94250553
LOC_Os09g37100	LOC_Os12g12514	9.73744768
LOC_Os09g32952	LOC_Os10g25950	8.86074339
LOC_Os09g36900	LOC_Os12g10600	9.56843106
LOC_Os09g37949	LOC_Os09g38620	9.57079954
LOC_Os09g37100	LOC_Os12g35570	9.44967506
LOC_Os09g34250	LOC_Os10g11140	9.0409732
LOC_Os09g37949	LOC_Os12g14070	8.9389127
LOC_Os09g38020	LOC_Os10g22070	9.42850349
LOC_Os09g33500	LOC_Os10g29470	9.83801498
LOC_Os09g32830	LOC_Os11g26860	9.33830823
LOC_Os09g37100	LOC_Os09g38030	8.87217783
LOC_Os09g34214	LOC_Os10g36650	9.37897058
LOC_Os09g37540	LOC_Os12g01922	8.82294947
LOC_Os09g36830	LOC_Os10g21250	8.93787773
LOC_Os09g32840	LOC_Os10g27190	9.73674307
LOC_Os09g32840	LOC_Os10g28120	11.919734
LOC_Os09g36710	LOC_Os11g19800	9.26792732
LOC_Os09g32570	LOC_Os11g25330	10.1048044
LOC_Os09g34250	LOC_Os10g07616	9.04434684
LOC_Os09g37949	LOC_Os11g02440	8.98864094
LOC_Os09g34960	LOC_Os09g39380	9.13366952
LOC_Os09g33860	LOC_Os12g13810	10.0162767
LOC_Os09g36830	LOC_Os11g31620	9.64338039
LOC_Os09g33500	LOC_Os11g34450	10.3694159
LOC_Os09g34960	LOC_Os10g28350	10.4406779

InteractorA	InteractorB	Z score
LOC_Os09g32952	LOC_Os10g33900	9.26605627
LOC_Os09g37540	LOC_Os10g33900	11.3003909
LOC_Os09g31506	LOC_Os11g08330	9.27499459
LOC_Os09g33500	LOC_Os10g21266	9.05829602
LOC_Os09g37949	LOC_Os10g28080	8.84969212
LOC_Os09g35800	LOC_Os11g40150	9.77769503
LOC_Os09g36670	LOC_Os11g03980	8.90915665
LOC_Os09g32830	LOC_Os10g01540	9.24667183
LOC_Os09g32570	LOC_Os11g24560	9.21855816
LOC_Os09g36930	LOC_Os10g08550	9.4104968
LOC_Os09g38020	LOC_Os10g01540	9.10660661
LOC_Os09g34960	LOC_Os12g02370	8.85010722
LOC_Os09g37949	LOC_Os12g24650	10.613648
LOC_Os09g36830	LOC_Os10g25140	10.1513357
LOC_Os09g33500	LOC_Os10g31950	9.13751093
LOC_Os09g36900	LOC_Os10g21266	9.63705231
LOC_Os09g37949	LOC_Os10g30580	8.81986826
LOC_Os09g34214	LOC_Os10g38140	9.30554238
LOC_Os09g34214	LOC_Os11g08460	9.05991524
LOC_Os09g32952	LOC_Os10g42280	11.1448991
LOC_Os09g36450	LOC_Os09g38620	9.38324665
LOC_Os09g37100	LOC_Os11g10480	10.1052387
LOC_Os09g37100	LOC_Os10g38640	9.35788532
LOC_Os09g35800	LOC_Os10g08620	9.30889603
LOC_Os09g32840	LOC_Os09g37100	10.0481519
LOC_Os09g32640	LOC_Os11g26910	10.1505349
LOC_Os09g36830	LOC_Os10g30580	10.5338115
LOC_Os09g32800	LOC_Os11g10510	9.50479224
LOC_Os09g36930	LOC_Os10g25140	10.6224326
LOC_Os09g37949	LOC_Os10g21324	9.27511671
LOC_Os09g34214	LOC_Os09g37949	8.82587525
LOC_Os09g36450	LOC_Os10g38160	10.6054554
LOC_Os09g36830	LOC_Os11g48110	9.65167179
LOC_Os09g32840	LOC_Os10g32870	8.83341198
LOC_Os09g36830	LOC_Os10g28320	9.79610165
LOC_Os09g32620	LOC_Os11g05730	10.0128063
LOC_Os09g31506	LOC_Os10g35110	9.46483372
LOC_Os09g37949	LOC_Os12g33610	8.93375637
LOC_Os09g34250	LOC_Os12g44000	10.1172444
LOC_Os09g32830	LOC_Os09g39380	9.44703119
LOC_Os09g36800	LOC_Os10g34760	9.0743499
LOC_Os09g34960	LOC_Os11g26860	9.02983087
LOC_Os09g36710	LOC_Os12g31370	9.01027811

InteractorA	InteractorB	Z score
LOC_Os09g36900	LOC_Os11g39540	10.3987088
LOC_Os09g36830	LOC_Os12g38760	9.71707824
LOC_Os09g33500	LOC_Os11g32260	8.97714409
LOC_Os09g33860	LOC_Os10g31950	10.129509
LOC_Os10g02480	LOC_Os12g34450	9.15708959
LOC_Os10g08670	LOC_Os11g43200	9.20638686
LOC_Os09g39400	LOC_Os10g35480	9.11381773
LOC_Os10g07229	LOC_Os12g40550	9.15112973
LOC_Os10g01080	LOC_Os10g21326	11.3283499
LOC_Os09g39500	LOC_Os10g41510	9.36453362
LOC_Os10g08580	LOC_Os12g40830	9.79039911
LOC_Os09g38020	LOC_Os11g10480	8.92560379
LOC_Os10g09860	LOC_Os10g21268	9.09834083
LOC_Os10g08670	LOC_Os11g33270	9.50255374
LOC_Os09g38620	LOC_Os10g38229	10.0406425
LOC_Os10g08670	LOC_Os11g08330	9.06540612
LOC_Os10g07040	LOC_Os12g25700	11.0935294
LOC_Os10g08022	LOC_Os12g02060	9.09177522
LOC_Os10g01080	LOC_Os10g26130	8.83218427
LOC_Os10g07229	LOC_Os10g38630	9.46605774
LOC_Os10g08022	LOC_Os10g11140	8.87726809
LOC_Os10g08710	LOC_Os11g08330	8.94655664
LOC_Os09g39380	LOC_Os12g10570	9.40968192
LOC_Os09g38020	LOC_Os11g10510	8.86154942
LOC_Os10g09860	LOC_Os12g43370	8.81827407
LOC_Os10g09860	LOC_Os11g01010	9.36535116
LOC_Os10g09860	LOC_Os12g14070	9.14365085
LOC_Os10g08710	LOC_Os12g12514	8.96305538
LOC_Os09g38020	LOC_Os12g22680	9.95584485
LOC_Os09g38020	LOC_Os10g39170	9.48057701
LOC_Os10g02040	LOC_Os12g44030	10.5436441
LOC_Os10g02040	LOC_Os12g10720	8.82962502
LOC_Os10g07040	LOC_Os10g28080	9.20709481
LOC_Os10g08670	LOC_Os10g26050	9.37445217
LOC_Os10g08670	LOC_Os12g07050	8.8898595
LOC_Os09g39570	LOC_Os12g44030	8.81728302
LOC_Os09g39440	LOC_Os11g38959	8.87077696
LOC_Os09g39400	LOC_Os10g40710	9.08723828
LOC_Os10g07040	LOC_Os11g47760	9.35924896
LOC_Os10g08022	LOC_Os10g38234	9.92929689
LOC_Os10g02480	LOC_Os11g10520	8.88397549
LOC_Os10g01570	LOC_Os11g24560	9.86485114
LOC_Os09g39380	LOC_Os10g11810	9.18834941

InteractorA	InteractorB	Z score
LOC_Os09g39570	LOC_Os10g41410	10.2836821
LOC_Os10g05069	LOC_Os11g32620	8.83274822
LOC_Os10g01570	LOC_Os10g30840	9.16703897
LOC_Os09g39380	LOC_Os12g02980	9.28126823
LOC_Os09g39810	LOC_Os12g40550	9.1400646
LOC_Os10g09860	LOC_Os12g22030	9.05640476
LOC_Os10g08670	LOC_Os12g37960	9.14587248
LOC_Os09g38030	LOC_Os10g29620	10.4802287
LOC_Os10g07616	LOC_Os10g39590	8.91987033
LOC_Os10g03540	LOC_Os11g25100	8.91406247
LOC_Os10g07229	LOC_Os12g25710	8.89282123
LOC_Os10g10434	LOC_Os10g22070	9.55880828
LOC_Os09g39500	LOC_Os12g23170	10.1310427
LOC_Os09g39570	LOC_Os11g30310	9.83214879
LOC_Os09g39380	LOC_Os12g13380	9.41416849
LOC_Os09g39400	LOC_Os10g25130	10.286045
LOC_Os09g39440	LOC_Os12g17910	9.51085046
LOC_Os10g08620	LOC_Os10g38234	10.3637664
LOC_Os09g39500	LOC_Os11g32260	9.57412115
LOC_Os09g38030	LOC_Os10g28200	9.71022844
LOC_Os10g07229	LOC_Os10g38340	8.99387644
LOC_Os09g39780	LOC_Os10g31950	9.38092266
LOC_Os10g08550	LOC_Os10g39840	10.3611645
LOC_Os09g38030	LOC_Os12g31370	9.44416149
LOC_Os09g39400	LOC_Os12g07820	10.1932774
LOC_Os10g08670	LOC_Os11g06390	9.26999415
LOC_Os10g05069	LOC_Os11g01872	10.5283149
LOC_Os10g08022	LOC_Os10g23900	8.94460004
LOC_Os10g08580	LOC_Os10g27174	10.1857092
LOC_Os09g39570	LOC_Os10g38730	10.0625212
LOC_Os09g38620	LOC_Os11g26860	9.08597489
LOC_Os10g05069	LOC_Os11g14910	9.60824176
LOC_Os10g07616	LOC_Os11g05730	9.5223222
LOC_Os10g08022	LOC_Os12g08280	9.3710727
LOC_Os10g08550	LOC_Os12g22680	10.1250215
LOC_Os10g08710	LOC_Os12g16290	10.2869982
LOC_Os10g05069	LOC_Os10g27190	8.8770991
LOC_Os10g08022	LOC_Os12g40510	9.88927507
LOC_Os10g07229	LOC_Os12g43450	9.04759131
LOC_Os09g38620	LOC_Os11g05290	9.75813227
LOC_Os09g38030	LOC_Os10g21352	9.58499466
LOC_Os10g03540	LOC_Os12g16220	8.95170863
LOC_Os09g39810	LOC_Os12g25630	9.32831614

InteractorA	InteractorB	Z score
LOC_Os10g01540	LOC_Os10g39410	9.10660275
LOC_Os09g38020	LOC_Os11g31620	9.02282073
LOC_Os10g08550	LOC_Os12g12514	9.63880355
LOC_Os10g02070	LOC_Os11g19320	9.41325353
LOC_Os10g08670	LOC_Os11g47560	9.11372527
LOC_Os10g08670	LOC_Os10g38140	8.87534232
LOC_Os10g02040	LOC_Os10g05069	9.29647368
LOC_Os09g39440	LOC_Os11g03230	9.05373952
LOC_Os09g38620	LOC_Os10g29470	8.80930072
LOC_Os10g07040	LOC_Os12g13320	9.58018089
LOC_Os09g38020	LOC_Os11g10520	10.9140543
LOC_Os10g08710	LOC_Os10g31950	8.88267616
LOC_Os10g08580	LOC_Os12g25690	10.8521424
LOC_Os09g38030	LOC_Os10g28120	9.1044433
LOC_Os10g02040	LOC_Os11g47760	8.85184537
LOC_Os09g39810	LOC_Os10g37060	9.04255012
LOC_Os10g08710	LOC_Os10g28360	9.01026891
LOC_Os10g02480	LOC_Os12g44000	9.17735868
LOC_Os10g03540	LOC_Os12g12590	9.85733837
LOC_Os10g01570	LOC_Os11g40150	9.32214579
LOC_Os10g01570	LOC_Os11g19320	9.01896405
LOC_Os10g01570	LOC_Os11g33240	10.5004471
LOC_Os10g10434	LOC_Os10g21326	9.61143952
LOC_Os10g07616	LOC_Os10g21326	9.08354113
LOC_Os10g08710	LOC_Os12g06620	8.86695814
LOC_Os09g39380	LOC_Os10g28360	11.57308
LOC_Os09g39500	LOC_Os12g12560	8.87840379
LOC_Os10g01570	LOC_Os10g39880	8.8658863
LOC_Os10g08670	LOC_Os11g19220	11.8528654
LOC_Os09g38030	LOC_Os10g21266	8.83887599
LOC_Os10g07040	LOC_Os12g40830	8.98047193
LOC_Os09g39810	LOC_Os12g02370	9.2760738
LOC_Os09g39500	LOC_Os11g05880	9.29571612
LOC_Os10g08620	LOC_Os10g42720	9.44278931
LOC_Os10g02380	LOC_Os12g16200	9.55134675
LOC_Os09g39570	LOC_Os10g07229	8.91326396
LOC_Os10g10434	LOC_Os11g48110	9.0144204
LOC_Os10g21406	LOC_Os11g33270	9.01425107
LOC_Os10g21268	LOC_Os11g03290	9.58649364
LOC_Os10g20910	LOC_Os11g10480	9.82616426
LOC_Os10g21298	LOC_Os12g38750	8.96503893
LOC_Os10g21268	LOC_Os12g31640	9.23914745
LOC_Os10g21248	LOC_Os12g34062	9.34974371

InteractorA	InteractorB	Z score
LOC_Os10g21230	LOC_Os12g10600	9.13999373
LOC_Os10g22070	LOC_Os12g41110	10.2406884
LOC_Os10g10434	LOC_Os10g38470	9.83756743
LOC_Os10g23900	LOC_Os10g25130	9.99027309
LOC_Os10g21266	LOC_Os10g37060	9.44522846
LOC_Os10g22070	LOC_Os10g39410	9.42850276
LOC_Os10g21352	LOC_Os10g31000	9.00579729
LOC_Os10g21212	LOC_Os11g19800	10.0142285
LOC_Os10g17650	LOC_Os12g17540	9.12856147
LOC_Os10g21266	LOC_Os10g28050	10.3896113
LOC_Os10g21266	LOC_Os12g40510	9.69455308
LOC_Os10g21250	LOC_Os11g47550	10.0509175
LOC_Os10g22310	LOC_Os12g25120	9.63486897
LOC_Os10g21326	LOC_Os10g38740	9.65864361
LOC_Os10g20910	LOC_Os10g25140	9.09043352
LOC_Os10g11810	LOC_Os10g34520	9.37279275
LOC_Os10g11810	LOC_Os10g38640	9.54454434
LOC_Os10g23900	LOC_Os11g26860	9.45144567
LOC_Os10g21250	LOC_Os11g48110	8.90218701
LOC_Os10g21344	LOC_Os10g38274	8.82040254
LOC_Os10g17650	LOC_Os10g25140	9.51931889
LOC_Os10g11810	LOC_Os11g48110	10.1320885
LOC_Os10g21298	LOC_Os12g10570	9.65921952
LOC_Os10g22310	LOC_Os12g12560	8.94425696
LOC_Os10g21192	LOC_Os11g03230	10.7700588
LOC_Os10g11810	LOC_Os11g37890	9.08736911
LOC_Os10g17680	LOC_Os12g12560	9.43409894
LOC_Os10g20910	LOC_Os11g32510	9.75560504
LOC_Os10g11260	LOC_Os10g29470	9.80964132
LOC_Os10g14150	LOC_Os10g28320	9.08092236
LOC_Os10g23900	LOC_Os12g41110	8.80954606
LOC_Os10g17660	LOC_Os12g12470	9.56549765
LOC_Os10g22310	LOC_Os11g32510	9.1354718
LOC_Os10g11260	LOC_Os12g38180	8.85266881
LOC_Os10g21352	LOC_Os11g25330	9.92153133
LOC_Os10g21266	LOC_Os11g29400	8.83546156
LOC_Os10g21352	LOC_Os12g21798	9.12606011
LOC_Os10g21248	LOC_Os10g21342	10.0521053
LOC_Os10g17650	LOC_Os11g03290	9.022599
LOC_Os10g21310	LOC_Os12g17910	9.22622652
LOC_Os10g25130	LOC_Os10g39880	9.82803204
LOC_Os10g11140	LOC_Os10g36650	9.00438978
LOC_Os10g21298	LOC_Os12g41220	9.67765764

InteractorA	InteractorB	Z score
LOC_Os10g21344	LOC_Os10g38660	8.85371839
LOC_Os10g21248	LOC_Os12g07050	9.38186602
LOC_Os10g25140	LOC_Os10g27050	9.79000542
LOC_Os10g21268	LOC_Os10g32550	8.88786754
LOC_Os10g11810	LOC_Os10g26010	9.64782619
LOC_Os10g21342	LOC_Os12g38760	9.62756182
LOC_Os10g21250	LOC_Os12g07050	9.01243765
LOC_Os10g21250	LOC_Os11g08470	9.53630171
LOC_Os10g21250	LOC_Os10g21344	9.28042324
LOC_Os10g11810	LOC_Os11g47760	9.65793143
LOC_Os10g11810	LOC_Os11g05470	9.13687298
LOC_Os10g25130	LOC_Os10g26600	9.70396746
LOC_Os10g21240	LOC_Os12g36950	10.3138906
LOC_Os10g21344	LOC_Os10g28320	9.17357561
LOC_Os10g21248	LOC_Os11g34450	10.3317891
LOC_Os10g25130	LOC_Os12g02080	9.40439297
LOC_Os10g10434	LOC_Os11g32520	9.22776908
LOC_Os10g25140	LOC_Os12g12580	9.04850446
LOC_Os10g21324	LOC_Os12g12560	9.46658096
LOC_Os10g13800	LOC_Os10g28120	9.02034504
LOC_Os10g21268	LOC_Os11g32260	8.99125062
LOC_Os10g22310	LOC_Os11g19800	9.49870038
LOC_Os10g17660	LOC_Os12g12560	9.43409894
LOC_Os10g23900	LOC_Os10g38690	9.21481774
LOC_Os10g23900	LOC_Os11g30290	10.2333707
LOC_Os10g11140	LOC_Os10g31940	9.17720764
LOC_Os10g21268	LOC_Os10g26130	9.21134016
LOC_Os10g23900	LOC_Os11g03230	10.1137594
LOC_Os10g17680	LOC_Os12g12470	9.56549765
LOC_Os10g21248	LOC_Os12g44000	9.65119656
LOC_Os10g21326	LOC_Os12g08270	10.8243297
LOC_Os10g14150	LOC_Os12g44000	8.91328453
LOC_Os10g23900	LOC_Os10g26110	20.2955572
LOC_Os10g11810	LOC_Os11g07440	10.2660743
LOC_Os10g11810	LOC_Os12g21798	10.3450957
LOC_Os10g21212	LOC_Os10g38600	9.61951058
LOC_Os10g21212	LOC_Os12g02060	9.15722748
LOC_Os10g21230	LOC_Os11g14910	9.43989293
LOC_Os10g20910	LOC_Os10g38710	12.1044373
LOC_Os10g21326	LOC_Os12g25710	11.7484235
LOC_Os10g22450	LOC_Os11g26860	8.9695146
LOC_Os10g11810	LOC_Os12g16240	9.94492876
LOC_Os10g21240	LOC_Os11g33240	9.36005432

InteractorA	InteractorB	Z score
LOC_Os10g25130	LOC_Os12g44030	11.1655392
LOC_Os10g21230	LOC_Os11g01360	9.89071682
LOC_Os10g22450	LOC_Os11g37890	9.46692223
LOC_Os10g13800	LOC_Os10g42280	9.36831832
LOC_Os10g21230	LOC_Os10g21344	8.82040254
LOC_Os10g26600	LOC_Os12g43630	9.47609814
LOC_Os10g27190	LOC_Os12g39630	9.67055786
LOC_Os10g30840	LOC_Os12g25630	8.9574729
LOC_Os10g26010	LOC_Os10g27174	8.82395693
LOC_Os10g27174	LOC_Os12g16410	8.91261309
LOC_Os10g25674	LOC_Os11g04954	10.9223509
LOC_Os10g29620	LOC_Os10g41510	8.86409966
LOC_Os10g34020	LOC_Os12g16240	9.33015074
LOC_Os10g27190	LOC_Os11g08470	9.65112456
LOC_Os10g30840	LOC_Os11g08470	8.96907197
LOC_Os10g27174	LOC_Os11g10520	9.07851985
LOC_Os10g29470	LOC_Os10g34760	9.32440343
LOC_Os10g28320	LOC_Os11g32520	8.93919493
LOC_Os10g30840	LOC_Os11g36719	8.94895211
LOC_Os10g27174	LOC_Os12g16220	8.85735753
LOC_Os10g28050	LOC_Os12g16410	10.2479133
LOC_Os10g26010	LOC_Os10g31000	9.0255885
LOC_Os10g28120	LOC_Os10g38730	9.35012984
LOC_Os10g25674	LOC_Os11g32510	8.80553974
LOC_Os10g26110	LOC_Os10g37210	9.22577892
LOC_Os10g30200	LOC_Os10g38640	10.6115005
LOC_Os10g26010	LOC_Os11g29400	9.96917537
LOC_Os10g26010	LOC_Os10g38470	10.272077
LOC_Os10g29620	LOC_Os12g38770	9.36612259
LOC_Os10g26010	LOC_Os11g38959	9.1038028
LOC_Os10g25950	LOC_Os11g37550	9.01118093
LOC_Os10g34760	LOC_Os12g16220	8.82589033
LOC_Os10g30840	LOC_Os11g37550	8.91398547
LOC_Os10g25930	LOC_Os12g43440	9.65606813
LOC_Os10g28350	LOC_Os11g26860	9.85699211
LOC_Os10g28050	LOC_Os11g33240	11.1672024
LOC_Os10g34760	LOC_Os12g16250	9.50496901
LOC_Os10g26010	LOC_Os12g10730	9.31078013
LOC_Os10g28360	LOC_Os11g10510	9.13489935
LOC_Os10g25140	LOC_Os12g44000	9.80272568
LOC_Os10g28320	LOC_Os12g43440	9.41917402
LOC_Os10g30200	LOC_Os11g06390	9.4391619
LOC_Os10g30580	LOC_Os12g25630	10.79668

InteractorA	InteractorB	Z score
LOC_Os10g32680	LOC_Os11g25260	10.2066188
LOC_Os10g27050	LOC_Os10g35840	9.21060878
LOC_Os10g34520	LOC_Os10g41510	9.39550694
LOC_Os10g34520	LOC_Os11g03290	9.17502712
LOC_Os10g28120	LOC_Os10g35110	9.00142795
LOC_Os10g28050	LOC_Os12g10720	9.1987023
LOC_Os10g25950	LOC_Os12g06660	9.91998762
LOC_Os10g28080	LOC_Os12g16220	10.4867562
LOC_Os10g27190	LOC_Os12g34450	8.80048902
LOC_Os10g28320	LOC_Os11g42350	8.85669109
LOC_Os10g30200	LOC_Os10g38690	9.25059187
LOC_Os10g32550	LOC_Os10g37060	9.4598552
LOC_Os10g28200	LOC_Os11g19320	8.9054616
LOC_Os10g33900	LOC_Os10g38730	9.25678208
LOC_Os10g30200	LOC_Os10g38670	10.076125
LOC_Os10g26130	LOC_Os12g05410	11.4723248
LOC_Os10g25674	LOC_Os10g32870	9.69746156
LOC_Os10g25674	LOC_Os10g38470	8.84459808
LOC_Os10g26010	LOC_Os10g38690	8.86674352
LOC_Os10g25674	LOC_Os10g38670	9.17671486
LOC_Os10g25950	LOC_Os10g38780	10.1632618
LOC_Os10g35070	LOC_Os10g38340	9.17000922
LOC_Os10g31950	LOC_Os10g41490	9.03479203
LOC_Os10g27190	LOC_Os11g26850	11.4653057
LOC_Os10g25674	LOC_Os10g32550	8.92590994
LOC_Os10g29470	LOC_Os12g42280	9.12622415
LOC_Os10g30580	LOC_Os11g37550	10.9776778
LOC_Os10g28120	LOC_Os11g25100	8.95514093
LOC_Os10g26130	LOC_Os11g10480	9.2983774
LOC_Os10g30840	LOC_Os12g07980	9.20723701
LOC_Os10g25930	LOC_Os11g37550	9.06423948
LOC_Os10g30200	LOC_Os12g17540	11.7494486
LOC_Os10g31940	LOC_Os12g07820	9.18079111
LOC_Os10g26110	LOC_Os11g10510	9.30757237
LOC_Os10g28350	LOC_Os12g16200	9.02395202
LOC_Os10g26600	LOC_Os12g22650	9.91672831
LOC_Os10g26390	LOC_Os12g34450	9.2445042
LOC_Os10g25930	LOC_Os10g28050	9.31970891
LOC_Os10g29470	LOC_Os12g44020	9.20824082
LOC_Os10g29620	LOC_Os12g25710	8.87895287
LOC_Os10g26010	LOC_Os12g08280	9.56359263
LOC_Os10g31950	LOC_Os12g10730	10.0262853
LOC_Os10g28360	LOC_Os11g06390	9.02024706

InteractorA	InteractorB	Z score
LOC_Os10g26390	LOC_Os11g03230	9.1518991
LOC_Os10g26390	LOC_Os11g01872	9.05373007
LOC_Os10g28360	LOC_Os11g10480	9.30184277
LOC_Os10g35070	LOC_Os11g24560	8.88043444
LOC_Os10g28050	LOC_Os10g38640	9.30089546
LOC_Os10g28080	LOC_Os12g12514	10.6665748
LOC_Os10g25930	LOC_Os12g44000	10.2440896
LOC_Os10g34520	LOC_Os11g02440	9.10783124
LOC_Os10g29470	LOC_Os12g40510	9.83796118
LOC_Os10g30580	LOC_Os12g41110	8.92325618
LOC_Os10g28360	LOC_Os10g38234	9.74058653
LOC_Os10g25950	LOC_Os11g38959	9.31189732
LOC_Os10g28350	LOC_Os12g41110	9.57549031
LOC_Os10g32970	LOC_Os12g07050	8.8763822
LOC_Os10g27190	LOC_Os10g31950	9.84748132
LOC_Os10g25674	LOC_Os12g17910	8.86111751
LOC_Os10g33800	LOC_Os12g25120	10.4252552
LOC_Os10g29470	LOC_Os12g12560	9.73206033
LOC_Os10g30200	LOC_Os11g05290	9.31181978
LOC_Os10g27190	LOC_Os11g32520	8.83770697
LOC_Os10g28050	LOC_Os10g38160	9.16524243
LOC_Os10g32550	LOC_Os12g10560	9.67308266
LOC_Os10g25674	LOC_Os10g38150	9.00529463
LOC_Os10g28120	LOC_Os11g26860	9.1867587
LOC_Os10g25674	LOC_Os11g32580	8.89972686
LOC_Os10g32550	LOC_Os11g41130	9.37421985
LOC_Os10g25930	LOC_Os11g40150	9.29019282
LOC_Os10g34760	LOC_Os12g10720	8.96121908
LOC_Os10g26390	LOC_Os11g24560	9.42346417
LOC_Os10g32680	LOC_Os11g10480	9.16772968
LOC_Os10g39680	LOC_Os12g12470	10.0387815
LOC_Os10g38360	LOC_Os10g38740	8.96242161
LOC_Os10g38340	LOC_Os12g34874	10.4499441
LOC_Os10g38470	LOC_Os12g10730	8.83053633
LOC_Os10g38640	LOC_Os11g25260	8.98604992
LOC_Os10g36650	LOC_Os11g01360	9.24262779
LOC_Os10g39680	LOC_Os12g05410	8.92689123
LOC_Os10g38340	LOC_Os12g22650	10.4174391
LOC_Os10g38660	LOC_Os12g12560	8.94283937
LOC_Os10g38690	LOC_Os12g10720	9.7342784
LOC_Os10g38670	LOC_Os11g37550	10.0905032
LOC_Os10g38140	LOC_Os12g12514	9.89804716
LOC_Os10g39880	LOC_Os11g25100	9.44543145

InteractorA	InteractorB	Z score
LOC_Os10g39410	LOC_Os11g31620	9.02281446
LOC_Os10g38140	LOC_Os10g41410	10.5884594
LOC_Os10g38740	LOC_Os12g10600	10.3615443
LOC_Os10g38780	LOC_Os12g10720	10.8123316
LOC_Os10g38690	LOC_Os11g05290	9.1394736
LOC_Os10g38600	LOC_Os12g22650	9.01998418
LOC_Os10g39880	LOC_Os11g04880	8.912063
LOC_Os10g38660	LOC_Os12g44010	9.02272621
LOC_Os10g37060	LOC_Os12g33946	8.86939263
LOC_Os10g39880	LOC_Os12g22030	8.82637011
LOC_Os10g38600	LOC_Os11g26860	8.81977561
LOC_Os10g38160	LOC_Os11g05730	8.95621906
LOC_Os10g39170	LOC_Os10g39410	9.48057701
LOC_Os10g38160	LOC_Os12g10730	8.96249536
LOC_Os10g38470	LOC_Os12g13380	8.99589335
LOC_Os10g38360	LOC_Os12g39630	9.86586221
LOC_Os10g39840	LOC_Os11g05880	8.840848
LOC_Os10g38670	LOC_Os12g03090	8.95597316
LOC_Os10g35110	LOC_Os11g08940	9.16431038
LOC_Os10g38340	LOC_Os12g10600	12.4545372
LOC_Os10g38600	LOC_Os12g07980	9.64635951
LOC_Os10g38274	LOC_Os12g10600	9.13999363
LOC_Os10g38690	LOC_Os12g22030	9.31788172
LOC_Os10g38670	LOC_Os12g10720	9.51293519
LOC_Os10g39170	LOC_Os12g34380	9.09982275
LOC_Os10g38274	LOC_Os11g14910	9.43990273
LOC_Os10g38160	LOC_Os12g06620	9.09951817
LOC_Os10g39120	LOC_Os10g41410	9.70137739
LOC_Os10g38600	LOC_Os11g32260	10.9040265
LOC_Os10g38229	LOC_Os10g38580	9.47881261
LOC_Os10g38140	LOC_Os11g14910	12.9421288
LOC_Os10g35110	LOC_Os12g07050	9.08512361
LOC_Os10g38160	LOC_Os12g12470	10.6986442
LOC_Os10g38630	LOC_Os11g03230	9.16582088
LOC_Os10g39410	LOC_Os11g10510	8.86156807
LOC_Os10g38160	LOC_Os12g08270	8.81045022
LOC_Os10g39840	LOC_Os11g07440	9.38292451
LOC_Os10g35480	LOC_Os12g41110	9.3921802
LOC_Os10g38690	LOC_Os11g38959	11.7228867
LOC_Os10g38340	LOC_Os12g44020	8.95049427
LOC_Os10g38234	LOC_Os11g07440	9.34328479
LOC_Os10g40600	LOC_Os11g26910	9.61288807
LOC_Os10g38580	LOC_Os10g38730	9.87687724

InteractorA	InteractorB	Z score
LOC_Os10g38234	LOC_Os10g40090	11.6693576
LOC_Os10g38630	LOC_Os12g13800	10.3120242
LOC_Os10g35110	LOC_Os10g38740	9.07066965
LOC_Os10g38340	LOC_Os12g12470	8.84833216
LOC_Os10g38630	LOC_Os12g43440	8.94846187
LOC_Os10g39410	LOC_Os11g10480	8.92560379
LOC_Os10g38360	LOC_Os12g21798	9.74038856
LOC_Os10g39880	LOC_Os12g13030	9.27189857
LOC_Os10g38670	LOC_Os11g03400	8.95597316
LOC_Os10g38700	LOC_Os12g32240	9.27089983
LOC_Os10g38229	LOC_Os10g38660	9.04058046
LOC_Os10g38360	LOC_Os11g14040	9.65488902
LOC_Os10g38350	LOC_Os11g05730	9.2380179
LOC_Os10g38700	LOC_Os10g40600	9.19767589
LOC_Os10g38234	LOC_Os11g07020	10.9804453
LOC_Os10g39840	LOC_Os11g20790	8.97010805
LOC_Os10g38730	LOC_Os12g16410	8.88082615
LOC_Os10g38350	LOC_Os10g38690	12.2042716
LOC_Os10g35110	LOC_Os12g07720	9.36463321
LOC_Os10g38160	LOC_Os12g22650	8.89265062
LOC_Os10g38660	LOC_Os12g06620	9.59399716
LOC_Os10g39680	LOC_Os11g08470	10.2509026
LOC_Os10g38140	LOC_Os12g10570	9.14719203
LOC_Os10g37060	LOC_Os11g19320	10.2281465
LOC_Os10g39120	LOC_Os12g34450	9.78195249
LOC_Os10g38229	LOC_Os10g38730	9.10007066
LOC_Os10g39880	LOC_Os11g03230	9.24753478
LOC_Os10g38950	LOC_Os11g01872	8.8562619
LOC_Os10g40600	LOC_Os11g32650	9.34138637
LOC_Os10g38660	LOC_Os12g18900	8.88904464
LOC_Os10g38630	LOC_Os11g37550	8.96469517
LOC_Os10g38360	LOC_Os12g22650	9.45870794
LOC_Os10g39410	LOC_Os11g10520	10.9140911
LOC_Os10g38690	LOC_Os10g40600	9.12965142
LOC_Os10g38489	LOC_Os12g22030	10.2247204
LOC_Os10g38234	LOC_Os12g16240	8.9100681
LOC_Os10g38610	LOC_Os11g32620	9.22973693
LOC_Os10g38274	LOC_Os11g01360	9.89071667
LOC_Os10g36650	LOC_Os11g47550	9.41737939
LOC_Os10g38234	LOC_Os11g26860	8.92338076
LOC_Os10g38340	LOC_Os10g41689	9.1485566
LOC_Os10g38690	LOC_Os12g32240	9.01308201
LOC_Os10g39410	LOC_Os12g22680	9.95585412

InteractorA	InteractorB	Z score
LOC_Os10g38150	LOC_Os12g40510	11.9998619
LOC_Os10g40600	LOC_Os11g03980	11.0406053
LOC_Os10g39840	LOC_Os11g41610	10.0680206
LOC_Os10g38540	LOC_Os11g37550	9.26517408
LOC_Os10g38160	LOC_Os12g12560	9.02137335
LOC_Os11g08460	LOC_Os12g13380	10.4609221
LOC_Os10g41550	LOC_Os12g12514	9.48799842
LOC_Os10g40730	LOC_Os12g25120	9.24124923
LOC_Os11g10510	LOC_Os11g10520	12.4835161
LOC_Os10g40730	LOC_Os11g32520	9.5752575
LOC_Os11g05880	LOC_Os11g39540	8.96068568
LOC_Os11g01872	LOC_Os11g08470	10.3632314
LOC_Os11g01010	LOC_Os11g31530	9.62773584
LOC_Os10g42940	LOC_Os11g32520	9.50475026
LOC_Os11g03290	LOC_Os11g20790	9.14471398
LOC_Os10g40700	LOC_Os11g31530	8.85057032
LOC_Os11g05470	LOC_Os12g23170	9.27973667
LOC_Os11g03290	LOC_Os12g05410	8.82819293
LOC_Os10g40720	LOC_Os12g13380	9.60630705
LOC_Os11g08440	LOC_Os12g13320	8.94726578
LOC_Os11g08120	LOC_Os11g42350	9.21466719
LOC_Os11g10480	LOC_Os12g04924	8.9825081
LOC_Os11g10510	LOC_Os12g43450	9.10850948
LOC_Os11g02130	LOC_Os12g23630	8.91173362
LOC_Os11g02130	LOC_Os12g23170	9.42163539
LOC_Os11g03980	LOC_Os11g37550	9.8970355
LOC_Os11g10480	LOC_Os12g43490	9.3046843
LOC_Os11g07020	LOC_Os12g16220	8.80820962
LOC_Os11g01872	LOC_Os12g17540	9.08721189
LOC_Os11g04954	LOC_Os11g32650	9.60881492
LOC_Os10g42940	LOC_Os11g36719	8.81241816
LOC_Os11g10480	LOC_Os12g35570	8.87112442
LOC_Os10g40720	LOC_Os12g03816	9.05080831
LOC_Os10g40720	LOC_Os10g41490	9.50737121
LOC_Os11g02440	LOC_Os11g20790	9.94602793
LOC_Os11g08120	LOC_Os12g31370	10.1363032
LOC_Os11g05730	LOC_Os12g07980	10.6969479
LOC_Os11g08470	LOC_Os11g19800	9.13148284
LOC_Os11g20790	LOC_Os11g32650	8.98547333
LOC_Os10g41689	LOC_Os12g44000	8.9258131
LOC_Os11g03980	LOC_Os11g25100	9.15872857
LOC_Os11g05730	LOC_Os12g10730	9.13435634
LOC_Os11g08470	LOC_Os11g37960	9.06892064

InteractorA	InteractorB	Z score
LOC_Os11g10520	LOC_Os12g12514	8.87792329
LOC_Os11g03230	LOC_Os11g14040	9.11566929
LOC_Os11g01010	LOC_Os12g13390	11.0345625
LOC_Os10g41510	LOC_Os12g10730	8.99468863
LOC_Os11g10510	LOC_Os12g32240	9.11656274
LOC_Os10g42940	LOC_Os12g13810	9.22726733
LOC_Os11g05290	LOC_Os12g13810	9.09335779
LOC_Os11g03290	LOC_Os11g36719	9.05893549
LOC_Os11g19220	LOC_Os12g02980	8.83506763
LOC_Os11g08330	LOC_Os12g44020	11.0675483
LOC_Os11g10510	LOC_Os12g05410	9.3517532
LOC_Os10g41510	LOC_Os12g43370	8.85109004
LOC_Os11g08340	LOC_Os11g47760	10.6576087
LOC_Os11g05570	LOC_Os11g08460	9.28732212
LOC_Os11g19220	LOC_Os11g47590	8.88243737
LOC_Os11g10480	LOC_Os11g10520	8.89341202
LOC_Os11g10510	LOC_Os11g32520	9.32491371
LOC_Os11g04954	LOC_Os12g31370	9.04482393
LOC_Os11g08120	LOC_Os12g43450	9.25004532
LOC_Os11g03290	LOC_Os12g41110	8.91936146
LOC_Os10g41510	LOC_Os11g10510	8.80748852
LOC_Os11g01872	LOC_Os12g14070	8.90634167
LOC_Os11g02440	LOC_Os12g16200	9.30024787
LOC_Os11g01872	LOC_Os11g03230	9.16040121
LOC_Os11g08470	LOC_Os12g42884	10.0236669
LOC_Os11g04954	LOC_Os11g25100	8.93776256
LOC_Os11g08340	LOC_Os12g42876	9.89276377
LOC_Os10g42720	LOC_Os11g47760	8.94546549
LOC_Os11g04954	LOC_Os11g40150	8.93056287
LOC_Os11g10480	LOC_Os12g43370	10.470695
LOC_Os10g41689	LOC_Os11g34450	9.26558332
LOC_Os11g02600	LOC_Os11g26850	8.85062745
LOC_Os11g05730	LOC_Os12g22680	9.68892171
LOC_Os11g04954	LOC_Os12g12560	10.4269298
LOC_Os11g01360	LOC_Os11g32620	9.33526354
LOC_Os11g08445	LOC_Os12g07820	8.90288156
LOC_Os11g04954	LOC_Os12g04980	9.15354371
LOC_Os10g41689	LOC_Os12g44350	9.19814588
LOC_Os11g07440	LOC_Os12g44030	9.63475716
LOC_Os11g08460	LOC_Os12g38750	9.12919904
LOC_Os11g08340	LOC_Os12g24650	9.82084156
LOC_Os10g40730	LOC_Os12g12514	9.48893756
LOC_Os11g14040	LOC_Os12g13380	9.74078049

InteractorA	InteractorB	Z score
LOC_Os11g02440	LOC_Os11g04954	9.32658888
LOC_Os11g01360	LOC_Os12g01922	9.08286244
LOC_Os11g06390	LOC_Os12g17910	8.93053703
LOC_Os11g03230	LOC_Os11g40150	9.64965283
LOC_Os11g08340	LOC_Os12g25690	8.95587658
LOC_Os11g19220	LOC_Os11g33240	9.58292445
LOC_Os10g41550	LOC_Os12g39630	9.01489098
LOC_Os11g04954	LOC_Os12g12590	9.19339582
LOC_Os11g03400	LOC_Os11g47580	9.7578856
LOC_Os11g08440	LOC_Os11g32520	8.963383
LOC_Os11g03980	LOC_Os11g24560	9.02346011
LOC_Os11g10510	LOC_Os12g10720	10.6539592
LOC_Os10g41550	LOC_Os12g02370	9.99306973
LOC_Os11g16590	LOC_Os12g08280	9.24808501
LOC_Os11g10480	LOC_Os11g31530	10.7570344
LOC_Os11g01872	LOC_Os12g13810	9.07322704
LOC_Os10g41480	LOC_Os11g25260	8.9949495
LOC_Os11g08440	LOC_Os11g32650	9.18877594
LOC_Os10g41510	LOC_Os12g44000	9.18595299
LOC_Os11g06390	LOC_Os11g43360	8.93524546
LOC_Os11g10510	LOC_Os11g47590	10.0900154
LOC_Os10g41689	LOC_Os12g34874	9.46178314
LOC_Os10g41490	LOC_Os10g41510	10.3587194
LOC_Os11g01872	LOC_Os12g13380	9.33586399
LOC_Os11g14910	LOC_Os11g26860	8.92181183
LOC_Os11g02440	LOC_Os12g40550	8.84961711
LOC_Os11g08470	LOC_Os12g06620	9.12392242
LOC_Os11g31530	LOC_Os12g12580	9.8888418
LOC_Os12g16240	LOC_Os12g22650	9.52797035
LOC_Os12g07980	LOC_Os12g16290	9.14227085
LOC_Os11g43360	LOC_Os12g38770	9.01537802
LOC_Os11g47590	LOC_Os12g24650	10.5704645
LOC_Os12g08270	LOC_Os12g22680	9.34476652
LOC_Os12g13320	LOC_Os12g44020	9.09219399
LOC_Os12g12514	LOC_Os12g23630	8.80132452
LOC_Os11g37950	LOC_Os12g33610	9.74813118
LOC_Os11g25700	LOC_Os12g43490	9.19083305
LOC_Os12g12514	LOC_Os12g40510	9.13379773
LOC_Os11g34570	LOC_Os12g12470	9.43018286
LOC_Os11g26910	LOC_Os12g12514	9.98104798
LOC_Os11g47580	LOC_Os12g03090	9.7578856
LOC_Os11g29400	LOC_Os12g06660	9.06788217
LOC_Os11g47560	LOC_Os12g43100	10.7611563

InteractorA	InteractorB	Z score
LOC_Os11g40140	LOC_Os12g12470	9.22616979
LOC_Os12g07720	LOC_Os12g16410	8.90500441
LOC_Os12g07980	LOC_Os12g34380	8.8333523
LOC_Os11g41130	LOC_Os12g18900	9.15399892
LOC_Os12g13380	LOC_Os12g40830	9.52933863
LOC_Os11g32520	LOC_Os11g32770	9.51532638
LOC_Os11g47760	LOC_Os12g01922	11.2363632
LOC_Os11g33240	LOC_Os11g47600	11.5368348
LOC_Os12g07050	LOC_Os12g13380	10.103902
LOC_Os12g12470	LOC_Os12g38770	9.3203047
LOC_Os11g25260	LOC_Os12g34380	9.28536191
LOC_Os11g43960	LOC_Os12g41110	9.0704275
LOC_Os12g02980	LOC_Os12g22650	10.6131187
LOC_Os11g47560	LOC_Os12g14070	9.69096244
LOC_Os11g32580	LOC_Os12g12514	10.7350159
LOC_Os12g14070	LOC_Os12g24650	9.04449642
LOC_Os12g02980	LOC_Os12g13390	9.98731382
LOC_Os11g48110	LOC_Os12g16410	8.89442621
LOC_Os11g37960	LOC_Os12g44000	9.96196172
LOC_Os11g34450	LOC_Os12g17910	10.3184483
LOC_Os11g26910	LOC_Os11g30310	9.26799996
LOC_Os12g12590	LOC_Os12g13320	8.83340046
LOC_Os12g03816	LOC_Os12g34380	9.19736657
LOC_Os11g47760	LOC_Os12g44000	9.00288875
LOC_Os12g02980	LOC_Os12g07720	9.70378517
LOC_Os11g33240	LOC_Os12g41220	9.03979132
LOC_Os12g12514	LOC_Os12g13320	8.93585282
LOC_Os12g13380	LOC_Os12g31640	9.41330328
LOC_Os11g26860	LOC_Os12g12580	9.2736424
LOC_Os11g32610	LOC_Os12g13380	9.53327119
LOC_Os11g37550	LOC_Os12g12580	9.0473894
LOC_Os12g07720	LOC_Os12g36950	8.94495455
LOC_Os12g13320	LOC_Os12g40510	8.888124
LOC_Os11g26910	LOC_Os12g02980	8.8106964
LOC_Os11g26910	LOC_Os12g38760	10.5893894
LOC_Os11g40150	LOC_Os12g04980	14.0577031
LOC_Os11g26910	LOC_Os11g32510	10.5775307
LOC_Os11g25260	LOC_Os12g04980	9.00499535
LOC_Os11g26850	LOC_Os11g32650	9.50311357
LOC_Os12g05410	LOC_Os12g31640	9.50947179
LOC_Os12g13390	LOC_Os12g25700	9.51940833
LOC_Os11g37550	LOC_Os11g40150	8.8597117
LOC_Os11g32520	LOC_Os11g47560	9.32954029

InteractorA	InteractorB	Z score
LOC_Os12g12514	LOC_Os12g43370	11.8946254
LOC_Os11g32520	LOC_Os11g32580	8.89229647
LOC_Os11g29400	LOC_Os11g40150	11.6750702
LOC_Os11g32610	LOC_Os11g32650	13.4441347
LOC_Os11g24560	LOC_Os12g44010	9.56319034
LOC_Os11g26910	LOC_Os12g38770	8.86876599
LOC_Os11g32770	LOC_Os12g16410	10.194021
LOC_Os12g01922	LOC_Os12g08280	8.88205739
LOC_Os12g14070	LOC_Os12g40830	9.08137514
LOC_Os11g47570	LOC_Os12g42876	10.3644541
LOC_Os11g32620	LOC_Os12g18900	9.93077696
LOC_Os11g36719	LOC_Os12g12514	8.88658026
LOC_Os12g02080	LOC_Os12g43450	10.6192026
LOC_Os12g02060	LOC_Os12g12590	9.20325388
LOC_Os11g25330	LOC_Os12g38180	10.1189494
LOC_Os12g05590	LOC_Os12g25700	9.30105831
LOC_Os11g32650	LOC_Os12g12590	9.27853316
LOC_Os12g07720	LOC_Os12g38760	9.76149846
LOC_Os11g32520	LOC_Os12g35570	9.92040544
LOC_Os11g37640	LOC_Os12g07980	8.92854748
LOC_Os12g16220	LOC_Os12g33946	9.29870515
LOC_Os11g41130	LOC_Os12g44020	9.2112935
LOC_Os11g26910	LOC_Os11g33270	9.79230796
LOC_Os12g02080	LOC_Os12g13390	9.2464976
LOC_Os12g13380	LOC_Os12g31370	9.26192627
LOC_Os11g25260	LOC_Os12g25690	9.28219651
LOC_Os11g25330	LOC_Os12g13380	10.6689455
LOC_Os12g02060	LOC_Os12g44030	9.01846157
LOC_Os12g02060	LOC_Os12g08270	9.7655897
LOC_Os12g12514	LOC_Os12g34062	9.7188777
LOC_Os12g12580	LOC_Os12g13390	9.23679632
LOC_Os11g29190	LOC_Os12g14070	9.18163131
LOC_Os12g08280	LOC_Os12g44000	8.99388829
LOC_Os12g02080	LOC_Os12g40510	10.2359122
LOC_Os11g33270	LOC_Os12g43630	10.3538198
LOC_Os11g40150	LOC_Os11g43200	8.80636836
LOC_Os12g06660	LOC_Os12g25700	8.91139642
LOC_Os11g48110	LOC_Os12g33610	14.921379
LOC_Os12g16240	LOC_Os12g34380	8.93740928
LOC_Os11g34450	LOC_Os12g38760	9.49250652
LOC_Os12g06620	LOC_Os12g21798	9.2911656
LOC_Os11g47580	LOC_Os12g23630	9.67009369
LOC_Os11g31620	LOC_Os11g41130	9.35412629

InteractorA	InteractorB	Z score
LOC_Os12g06660	LOC_Os12g17540	9.13801971
LOC_Os12g07720	LOC_Os12g17910	8.91840776
LOC_Os12g10600	LOC_Os12g13380	9.37365469
LOC_Os12g02060	LOC_Os12g25690	10.1245081
LOC_Os12g16250	LOC_Os12g40550	9.22230394
LOC_Os12g04924	LOC_Os12g22680	9.22127079
LOC_Os11g47550	LOC_Os12g31640	9.87505745
LOC_Os11g26850	LOC_Os12g31370	11.2452575
LOC_Os11g32260	LOC_Os12g43490	9.55065637
LOC_Os12g02980	LOC_Os12g31370	9.25570493
LOC_Os11g47760	LOC_Os12g40510	9.90376325
LOC_Os12g13390	LOC_Os12g42884	10.6552219
LOC_Os11g33240	LOC_Os12g26290	8.97382852
LOC_Os11g47760	LOC_Os12g40550	8.87777662
LOC_Os11g32540	LOC_Os11g37550	9.03918509
LOC_Os12g12560	LOC_Os12g12590	9.21847821
LOC_Os11g47570	LOC_Os12g01922	9.68315653
LOC_Os12g10570	LOC_Os12g22650	9.08698778
LOC_Os12g36950	LOC_Os12g39630	9.33984518
LOC_Os12g16410	LOC_Os12g40830	9.70284123
LOC_Os12g38760	LOC_Os12g44150	9.29784383
LOC_Os12g33610	LOC_Os12g40510	9.69626444
LOC_Os12g16290	LOC_Os12g44030	9.28376699
LOC_Os12g25690	LOC_Os12g25700	12.0037406
LOC_Os12g17910	LOC_Os12g23170	9.30704918
LOC_Os12g16290	LOC_Os12g43370	9.18970781
LOC_Os12g22650	LOC_Os12g37360	9.13210781
LOC_Os12g16290	LOC_Os12g38760	10.2465355
LOC_Os12g22650	LOC_Os12g31640	8.93999988
LOC_Os12g16290	LOC_Os12g34874	9.51033655
LOC_Os12g35570	LOC_Os12g41110	8.94901205
LOC_Os12g17910	LOC_Os12g44010	9.61919542
LOC_Os12g25120	LOC_Os12g25690	9.09972957
LOC_Os12g38180	LOC_Os12g42884	8.87492745
LOC_Os12g39630	LOC_Os12g42280	8.81025778
LOC_Os12g36950	LOC_Os12g43370	11.3230593
LOC_Os12g24650	LOC_Os12g39630	9.0624765
LOC_Os12g34874	LOC_Os12g38750	9.38130434
LOC_Os12g36950	LOC_Os12g38770	9.30406991
LOC_Os12g44020	LOC_Os12g44030	8.836369
LOC_Os01g27230	LOC_Os01g55950	9.32355592
LOC_Os01g27230	LOC_Os07g07320	9.81529031
LOC_Os01g27230	LOC_Os01g71830	8.92634838

InteractorA	InteractorB	Z score
LOC_Os01g27360	LOC_Os07g03467	8.82120898
LOC_Os01g27340	LOC_Os06g04200	9.62535016
LOC_Os01g27300	LOC_Os06g06050	9.17484916
LOC_Os01g27340	LOC_Os04g53230	8.89515851
LOC_Os01g27230	LOC_Os10g34020	10.0568007
LOC_Os01g27300	LOC_Os08g09950	9.70582257
LOC_Os01g27260	LOC_Os11g14910	8.80918488
LOC_Os01g27230	LOC_Os10g21212	10.6362855
LOC_Os01g27300	LOC_Os01g52470	8.82057767
LOC_Os01g27300	LOC_Os09g27750	9.34543484
LOC_Os01g27360	LOC_Os05g44760	9.07852716
LOC_Os01g27230	LOC_Os04g12960	8.91086951
LOC_Os01g27300	LOC_Os01g61210	9.44168221
LOC_Os01g27340	LOC_Os03g10940	8.95113517
LOC_Os01g27230	LOC_Os05g41210	10.4520193
LOC_Os01g27300	LOC_Os02g32030	9.07589343
LOC_Os01g27300	LOC_Os09g25390	9.72451285
LOC_Os01g27360	LOC_Os09g32810	8.99285922
LOC_Os01g27230	LOC_Os03g04410	9.27397762
LOC_Os01g27360	LOC_Os03g42840	9.44590339
LOC_Os01g27230	LOC_Os12g06660	8.86927729
LOC_Os01g27230	LOC_Os01g54490	9.22277726
LOC_Os01g27340	LOC_Os02g13330	8.8379497
LOC_Os01g27360	LOC_Os02g40000	9.04423133
LOC_Os01g27260	LOC_Os08g35740	8.91226595
LOC_Os01g27230	LOC_Os01g51210	10.0302015
LOC_Os01g27360	LOC_Os06g12790	10.2417584
LOC_Os01g27230	LOC_Os03g58130	9.59283679
LOC_Os01g27340	LOC_Os04g44730	9.79795792
LOC_Os01g27300	LOC_Os12g44010	9.02429609
LOC_Os01g27360	LOC_Os04g37460	9.05752447
LOC_Os01g27230	LOC_Os05g42350	9.52132553
LOC_Os01g27230	LOC_Os03g31750	8.85526467
LOC_Os01g27230	LOC_Os12g42884	9.03686879
LOC_Os01g27230	LOC_Os03g42840	9.46926508
LOC_Os01g27360	LOC_Os06g01850	9.41430087
LOC_Os01g27360	LOC_Os01g62244	9.40323446
LOC_Os01g27340	LOC_Os04g37480	9.9040479
LOC_Os01g27340	LOC_Os10g38340	9.21907619
LOC_Os01g27260	LOC_Os07g07550	9.23883799
LOC_Os01g27260	LOC_Os03g07150	8.8472977
LOC_Os01g27230	LOC_Os06g33210	9.11680868
LOC_Os01g27230	LOC_Os07g49120	8.80632311

InteractorA	InteractorB	Z score
LOC_Os01g27230	LOC_Os10g33900	9.95763322
LOC_Os01g27360	LOC_Os05g43510	11.0321336
LOC_Os01g27230	LOC_Os08g37790	9.70249151
LOC_Os01g27230	LOC_Os03g07840	9.08479916
LOC_Os01g27230	LOC_Os04g37460	8.81232128
LOC_Os01g27230	LOC_Os10g25950	9.28829686
LOC_Os01g27230	LOC_Os04g27060	8.88223568
LOC_Os01g27300	LOC_Os11g25260	8.96054385
LOC_Os01g27230	LOC_Os02g24632	8.93667576
LOC_Os01g27360	LOC_Os06g01390	9.10687833
LOC_Os01g27260	LOC_Os03g22120	9.17188655
LOC_Os01g27230	LOC_Os01g57962	9.55531655
LOC_Os01g27340	LOC_Os03g50885	9.21518032
LOC_Os01g27360	LOC_Os08g33100	9.23468345
LOC_Os01g27360	LOC_Os07g03288	8.82119943
LOC_Os01g27230	LOC_Os10g38234	11.2614237
LOC_Os01g27360	LOC_Os06g22140	8.8877133
LOC_Os01g27300	LOC_Os08g09210	8.87986475
LOC_Os01g27230	LOC_Os01g54370	9.02017904
LOC_Os01g27260	LOC_Os05g05670	9.13510972
LOC_Os01g27360	LOC_Os08g16910	10.1141498
LOC_Os01g27300	LOC_Os02g01220	10.2827705
LOC_Os01g27360	LOC_Os05g41900	9.63009704
LOC_Os01g27360	LOC_Os03g49380	9.58954558
LOC_Os01g27360	LOC_Os07g39290	9.19664493
LOC_Os01g27260	LOC_Os02g52390	11.6498486
LOC_Os01g27260	LOC_Os02g02560	9.78145665
LOC_Os01g27360	LOC_Os07g22650	9.79940801
LOC_Os01g27340	LOC_Os04g33040	10.5260921
LOC_Os01g27210	LOC_Os12g05410	10.3757169
LOC_Os01g27230	LOC_Os07g10720	8.98412562
LOC_Os01g27360	LOC_Os02g13140	11.7669605
LOC_Os01g27230	LOC_Os05g46360	9.48886548
LOC_Os01g27230	LOC_Os02g10830	8.92220975
LOC_Os01g27230	LOC_Os09g28400	10.642189
LOC_Os01g27260	LOC_Os02g13140	9.78513709
LOC_Os01g27360	LOC_Os04g33480	8.94080695
LOC_Os01g27360	LOC_Os09g11230	10.2681168
LOC_Os01g27360	LOC_Os08g33710	8.96310131
LOC_Os01g27360	LOC_Os04g40130	9.90392263
LOC_Os01g27360	LOC_Os07g03590	8.82120898
LOC_Os01g27260	LOC_Os11g07020	9.78284497
LOC_Os01g27340	LOC_Os07g07550	10.4336376

InteractorA	InteractorB	Z score
LOC_Os01g27300	LOC_Os07g23850	9.74602159
LOC_Os01g27230	LOC_Os06g36770	8.97042812
LOC_Os01g27230	LOC_Os07g08880	11.901254
LOC_Os01g27230	LOC_Os06g39708	9.16788159
LOC_Os01g27340	LOC_Os02g35760	8.85012207
LOC_Os01g27340	LOC_Os03g07300	9.01135005
LOC_Os01g27300	LOC_Os03g63090	8.98475278
LOC_Os01g27260	LOC_Os08g37800	8.80735252
LOC_Os01g27230	LOC_Os04g40990	8.86705641
LOC_Os01g27230	LOC_Os06g37610	10.1517639
LOC_Os01g27340	LOC_Os03g44170	9.00404007
LOC_Os01g27340	LOC_Os06g34690	8.95752244
LOC_Os01g27340	LOC_Os02g53790	8.93930931
LOC_Os01g27360	LOC_Os05g47980	9.00909382
LOC_Os01g27230	LOC_Os12g13380	8.9636812
LOC_Os01g27300	LOC_Os06g36840	10.451931
LOC_Os01g27340	LOC_Os10g35070	9.45261434
LOC_Os01g27360	LOC_Os07g03377	8.82120898
LOC_Os01g27360	LOC_Os01g64630	9.99099596
LOC_Os01g27230	LOC_Os06g34690	8.9052379
LOC_Os01g27230	LOC_Os03g60400	9.56602456
LOC_Os01g27230	LOC_Os02g39850	8.94371776
LOC_Os01g27230	LOC_Os08g31060	8.9246066
LOC_Os01g27490	LOC_Os05g38740	12.2930441
LOC_Os01g27390	LOC_Os09g31410	10.1561205
LOC_Os01g27390	LOC_Os09g08720	8.90744367
LOC_Os01g27380	LOC_Os10g29470	9.05433029
LOC_Os01g27380	LOC_Os02g52640	9.28750723
LOC_Os01g27490	LOC_Os06g27770	10.5532777
LOC_Os01g27390	LOC_Os03g27280	8.94756512
LOC_Os01g27380	LOC_Os04g40990	10.0709862
LOC_Os01g27390	LOC_Os03g01650	9.62923985
LOC_Os01g27490	LOC_Os01g64850	9.01659335
LOC_Os01g27360	LOC_Os12g01922	9.06315275
LOC_Os01g27380	LOC_Os05g51480	10.0926204
LOC_Os01g27630	LOC_Os02g35760	9.38411019
LOC_Os01g27380	LOC_Os10g21212	9.55466411
LOC_Os01g27490	LOC_Os06g10970	9.98942913
LOC_Os01g27480	LOC_Os12g07720	9.60749395
LOC_Os01g27380	LOC_Os04g29550	10.3858422
LOC_Os01g27380	LOC_Os09g31486	8.85706629
LOC_Os01g27480	LOC_Os02g08420	8.84275519
LOC_Os01g27480	LOC_Os09g10270	9.01209872

InteractorA	InteractorB	Z score
LOC_Os01g27480	LOC_Os07g03319	9.4418076
LOC_Os01g27490	LOC_Os02g45940	12.2930441
LOC_Os01g27480	LOC_Os01g46926	9.19288654
LOC_Os01g27480	LOC_Os02g14170	8.89974727
LOC_Os01g27390	LOC_Os03g01610	9.62923985
LOC_Os01g27480	LOC_Os09g39380	11.0064887
LOC_Os01g27490	LOC_Os01g49690	8.85928785
LOC_Os01g27490	LOC_Os09g38020	12.2930441
LOC_Os01g27490	LOC_Os04g29550	9.79983668
LOC_Os01g27490	LOC_Os03g28400	9.60709667
LOC_Os01g27490	LOC_Os11g32260	8.80660783
LOC_Os01g27480	LOC_Os11g33240	8.83339277
LOC_Os01g27380	LOC_Os05g50890	9.5233804
LOC_Os01g27380	LOC_Os05g40420	10.8497607
LOC_Os01g27490	LOC_Os02g52230	9.22318214
LOC_Os01g27630	LOC_Os01g39830	8.82907012
LOC_Os01g27630	LOC_Os05g34170	9.38938702
LOC_Os01g27480	LOC_Os06g35520	9.18989992
LOC_Os01g27380	LOC_Os03g57290	8.95777436
LOC_Os01g27490	LOC_Os02g32490	9.30512834
LOC_Os01g27490	LOC_Os01g27750	10.3117824
LOC_Os01g27380	LOC_Os02g08410	9.58350012
LOC_Os01g27490	LOC_Os04g49420	12.2930441
LOC_Os01g27380	LOC_Os01g64850	9.22876257
LOC_Os01g27480	LOC_Os03g55389	9.70420353
LOC_Os01g27480	LOC_Os12g25700	9.14913847
LOC_Os01g27480	LOC_Os09g37100	9.03395743
LOC_Os01g27630	LOC_Os04g57410	10.0918113
LOC_Os01g27480	LOC_Os08g17500	8.90666766
LOC_Os01g27390	LOC_Os10g08620	9.00425261
LOC_Os01g27480	LOC_Os01g48600	9.87185098
LOC_Os01g27490	LOC_Os09g32640	8.89722381
LOC_Os01g27490	LOC_Os05g04500	9.44824383
LOC_Os01g27630	LOC_Os05g35770	9.05241006
LOC_Os01g27380	LOC_Os04g16872	8.96135126
LOC_Os01g27480	LOC_Os03g45410	8.87001228
LOC_Os01g27490	LOC_Os10g38274	8.97450984
LOC_Os01g27480	LOC_Os07g03409	9.4418076
LOC_Os01g27380	LOC_Os03g15360	9.13907858
LOC_Os01g27380	LOC_Os05g37690	10.4383284
LOC_Os01g27490	LOC_Os08g10608	8.85556236
LOC_Os01g27490	LOC_Os12g31370	8.80029274
LOC_Os01g27480	LOC_Os03g49380	9.39499334

InteractorA	InteractorB	Z score
LOC_Os01g27360	LOC_Os10g41510	9.10124867
LOC_Os01g27490	LOC_Os02g01920	10.9601044
LOC_Os01g27630	LOC_Os05g04690	10.7706283
LOC_Os01g27490	LOC_Os08g41990	10.1138963
LOC_Os01g27490	LOC_Os04g28180	9.927563
LOC_Os01g27390	LOC_Os03g63410	8.82310111
LOC_Os01g27380	LOC_Os02g35760	8.89321449
LOC_Os01g27380	LOC_Os12g38760	9.75438163
LOC_Os01g27380	LOC_Os04g20070	9.26354234
LOC_Os01g27490	LOC_Os03g15120	9.48810576
LOC_Os01g27490	LOC_Os02g55300	10.1221331
LOC_Os01g27490	LOC_Os09g31506	9.5644575
LOC_Os01g27490	LOC_Os10g21230	8.97450774
LOC_Os01g27490	LOC_Os07g38540	9.49206135
LOC_Os01g27390	LOC_Os02g36400	9.09715909
LOC_Os01g27490	LOC_Os07g36500	12.2930441
LOC_Os01g27490	LOC_Os09g26340	12.2930441
LOC_Os01g27390	LOC_Os02g07230	9.11122302
LOC_Os01g27490	LOC_Os03g05290	9.04564641
LOC_Os01g27490	LOC_Os12g08280	8.83085958
LOC_Os01g27380	LOC_Os01g44980	9.57519156
LOC_Os01g27390	LOC_Os03g63090	8.92019326
LOC_Os01g27480	LOC_Os02g51910	9.22635686
LOC_Os01g27490	LOC_Os01g59790	9.82591735
LOC_Os01g27380	LOC_Os03g53200	8.80400649
LOC_Os01g27630	LOC_Os02g14170	8.88536461
LOC_Os01g27380	LOC_Os02g43010	9.83075725
LOC_Os01g27380	LOC_Os04g39864	9.20729408
LOC_Os01g27480	LOC_Os04g37950	9.16253752
LOC_Os01g27480	LOC_Os06g06980	8.9322747
LOC_Os01g27480	LOC_Os07g37550	8.80459887
LOC_Os01g27390	LOC_Os12g34874	9.1095561
LOC_Os01g27490	LOC_Os10g39410	12.2930441
LOC_Os01g27490	LOC_Os06g15420	9.76387192
LOC_Os01g27480	LOC_Os12g06620	9.33805564
LOC_Os01g27390	LOC_Os07g32800	9.58480477
LOC_Os01g27480	LOC_Os12g12590	11.0553504
LOC_Os01g27480	LOC_Os07g03499	9.4418076
LOC_Os01g27380	LOC_Os10g01080	9.55868084
LOC_Os01g27490	LOC_Os07g44790	9.60709667
LOC_Os01g27380	LOC_Os07g46830	9.49890512
LOC_Os01g27380	LOC_Os04g39880	10.5633465
LOC_Os01g27380	LOC_Os04g58580	8.82967188

InteractorA	InteractorB	Z score
LOC_Os01g27380	LOC_Os01g55240	9.08187123
LOC_Os01g27390	LOC_Os03g45320	8.95970202
LOC_Os01g27490	LOC_Os02g56700	9.65515285
LOC_Os01g27490	LOC_Os03g02780	12.2930441
LOC_Os01g27490	LOC_Os02g12580	8.98747282
LOC_Os01g27490	LOC_Os12g40550	8.8179773
LOC_Os01g27490	LOC_Os01g61920	12.2930441
LOC_Os01g27380	LOC_Os04g12960	8.95087615
LOC_Os01g27380	LOC_Os06g11240	13.1714064
LOC_Os01g27490	LOC_Os06g35660	9.48723571
LOC_Os01g27480	LOC_Os03g41460	8.8606492
LOC_Os01g27490	LOC_Os06g06050	8.94369198
LOC_Os01g27380	LOC_Os03g58400	9.2959736
LOC_Os01g27630	LOC_Os05g40420	9.69588875
LOC_Os01g27380	LOC_Os03g05730	9.62400003
LOC_Os01g27480	LOC_Os09g39400	9.08243364
LOC_Os01g27490	LOC_Os05g39050	12.2930441
LOC_Os01g27480	LOC_Os04g09670	8.88390529
LOC_Os01g27380	LOC_Os09g28420	9.01116575
LOC_Os01g27750	LOC_Os07g05180	8.95908364
LOC_Os01g28450	LOC_Os07g26630	10.2753829
LOC_Os01g27750	LOC_Os01g60190	9.67016351
LOC_Os01g29780	LOC_Os02g47590	8.92602639
LOC_Os01g27770	LOC_Os12g06620	10.4861051
LOC_Os01g28450	LOC_Os12g17540	9.14704723
LOC_Os01g28450	LOC_Os01g66180	9.09199762
LOC_Os01g27770	LOC_Os09g38030	9.02329641
LOC_Os01g28450	LOC_Os08g34280	8.87788044
LOC_Os01g27750	LOC_Os06g06880	9.53138554
LOC_Os01g27750	LOC_Os03g21260	9.0023735
LOC_Os01g28450	LOC_Os07g01760	11.2158041
LOC_Os01g27770	LOC_Os03g59060	8.85223989
LOC_Os01g29780	LOC_Os01g38500	9.74553417
LOC_Os01g28500	LOC_Os06g36670	9.32124651
LOC_Os01g27630	LOC_Os09g21770	9.49885788
LOC_Os01g27750	LOC_Os04g53230	9.17446197
LOC_Os01g28500	LOC_Os07g06970	9.24502407
LOC_Os01g28450	LOC_Os08g31060	9.38661281
LOC_Os01g27770	LOC_Os03g08020	9.58206882
LOC_Os01g27750	LOC_Os07g31830	11.0758019
LOC_Os01g28450	LOC_Os10g41480	9.65200318
LOC_Os01g27770	LOC_Os02g57150	9.41323485
LOC_Os01g27770	LOC_Os03g21260	10.9460045

InteractorA	InteractorB	Z score
LOC_Os01g28500	LOC_Os03g51550	9.03020348
LOC_Os01g28450	LOC_Os07g42960	10.6489541
LOC_Os01g28500	LOC_Os10g02480	9.6828689
LOC_Os01g27750	LOC_Os09g27420	9.6438474
LOC_Os01g29780	LOC_Os07g44450	8.83311474
LOC_Os01g27750	LOC_Os06g43640	9.28318184
LOC_Os01g27750	LOC_Os08g38300	10.4251346
LOC_Os01g27770	LOC_Os03g09250	9.05845689
LOC_Os01g27770	LOC_Os05g49760	8.85126541
LOC_Os01g27750	LOC_Os09g28400	8.82157303
LOC_Os01g27770	LOC_Os04g45290	9.2633792
LOC_Os01g27770	LOC_Os05g06300	8.82824365
LOC_Os01g27750	LOC_Os05g32140	9.46731858
LOC_Os01g27770	LOC_Os02g35760	9.71960482
LOC_Os01g28500	LOC_Os11g10510	8.97290076
LOC_Os01g27770	LOC_Os09g27820	9.16843542
LOC_Os01g27770	LOC_Os03g55280	9.2419758
LOC_Os01g28500	LOC_Os04g56210	9.26582532
LOC_Os01g28450	LOC_Os05g45810	9.45772048
LOC_Os01g27770	LOC_Os10g32870	8.97166227
LOC_Os01g28450	LOC_Os07g44790	9.2337353
LOC_Os01g29780	LOC_Os03g41460	8.84888884
LOC_Os01g27770	LOC_Os06g49970	9.90138513
LOC_Os01g28450	LOC_Os06g35520	9.38024238
LOC_Os01g27770	LOC_Os10g41480	10.8970493
LOC_Os01g27770	LOC_Os03g60580	9.84111211
LOC_Os01g27770	LOC_Os09g12590	9.01392511
LOC_Os01g28450	LOC_Os11g34450	9.07238102
LOC_Os01g27770	LOC_Os07g16970	9.11013712
LOC_Os01g29780	LOC_Os05g06300	9.07054644
LOC_Os01g29780	LOC_Os05g30410	9.1115495
LOC_Os01g27770	LOC_Os03g08010	9.58206882
LOC_Os01g28450	LOC_Os02g41860	9.14744677
LOC_Os01g29780	LOC_Os07g47420	9.29560878
LOC_Os01g27770	LOC_Os11g16590	8.83610782
LOC_Os01g28500	LOC_Os01g55350	9.04621126
LOC_Os01g28450	LOC_Os03g01170	9.15363768
LOC_Os01g27750	LOC_Os04g22660	9.16470363
LOC_Os01g27750	LOC_Os07g39290	9.72989316
LOC_Os01g27750	LOC_Os01g43390	9.96256567
LOC_Os01g28450	LOC_Os03g53200	9.17922353
LOC_Os01g27770	LOC_Os04g52280	10.2578161
LOC_Os01g27750	LOC_Os12g22030	9.71263388

InteractorA	InteractorB	Z score
LOC_Os01g27770	LOC_Os04g56730	9.64765323
LOC_Os01g28500	LOC_Os03g15120	8.84595605
LOC_Os01g27750	LOC_Os07g38030	9.35113506
LOC_Os01g29780	LOC_Os01g51170	9.07959641
LOC_Os01g27750	LOC_Os07g26900	10.8335275
LOC_Os01g27750	LOC_Os12g07050	9.05510184
LOC_Os01g27750	LOC_Os04g41960	10.1814903
LOC_Os01g27770	LOC_Os10g39680	9.09514428
LOC_Os01g27770	LOC_Os03g07150	9.65518939
LOC_Os01g27750	LOC_Os02g57770	9.42916876
LOC_Os01g27770	LOC_Os02g12580	8.98856812
LOC_Os01g29780	LOC_Os04g56760	9.11153493
LOC_Os01g27770	LOC_Os03g47610	11.4272669
LOC_Os01g29780	LOC_Os07g11440	9.37750999
LOC_Os01g27770	LOC_Os06g37660	10.2174964
LOC_Os01g27770	LOC_Os03g08050	9.58203044
LOC_Os01g27750	LOC_Os05g08960	9.50942304
LOC_Os01g27750	LOC_Os04g12710	9.47858553
LOC_Os01g27750	LOC_Os01g27770	10.3679174
LOC_Os01g27770	LOC_Os01g51570	8.92295775
LOC_Os01g27750	LOC_Os02g03870	9.34010791
LOC_Os01g27750	LOC_Os01g49890	9.29374027
LOC_Os01g27770	LOC_Os11g10510	9.00207308
LOC_Os01g27770	LOC_Os07g48160	8.81727228
LOC_Os01g28500	LOC_Os03g31750	9.78625552
LOC_Os01g28500	LOC_Os12g05410	8.89118747
LOC_Os01g28450	LOC_Os03g22060	9.82552676
LOC_Os01g27770	LOC_Os07g44370	8.96757908
LOC_Os01g27750	LOC_Os07g48040	9.51567367
LOC_Os01g27770	LOC_Os02g41680	9.89095957
LOC_Os01g27750	LOC_Os07g48050	9.67852066
LOC_Os01g28450	LOC_Os08g33100	9.48107616
LOC_Os01g27770	LOC_Os06g47320	9.9887964
LOC_Os01g27750	LOC_Os10g26110	8.82613411
LOC_Os01g27750	LOC_Os08g41340	8.89787957
LOC_Os01g28450	LOC_Os03g28400	9.2337353
LOC_Os01g27750	LOC_Os04g18650	9.19027503
LOC_Os01g27770	LOC_Os01g55950	9.37277476
LOC_Os01g28450	LOC_Os01g49120	9.17492454
LOC_Os01g27750	LOC_Os05g11550	9.5030514
LOC_Os01g27750	LOC_Os05g51050	9.55966837
LOC_Os01g27750	LOC_Os08g35440	9.13424724
LOC_Os01g28450	LOC_Os07g46830	9.42804513

InteractorA	InteractorB	Z score
LOC_Os01g27770	LOC_Os04g01470	9.0230314
LOC_Os01g32364	LOC_Os04g37460	9.55009466
LOC_Os01g34480	LOC_Os03g08280	9.34391334
LOC_Os01g33160	LOC_Os06g35660	9.16873319
LOC_Os01g33160	LOC_Os05g49760	8.99378005
LOC_Os01g34480	LOC_Os12g02980	9.92154156
LOC_Os01g34480	LOC_Os07g44740	9.93633503
LOC_Os01g34480	LOC_Os11g31530	10.08308
LOC_Os01g34614	LOC_Os03g45920	11.3961026
LOC_Os01g33160	LOC_Os07g46460	9.33829962
LOC_Os01g34480	LOC_Os07g36140	10.6011736
LOC_Os01g34480	LOC_Os03g07150	9.15589448
LOC_Os01g34480	LOC_Os08g04460	9.92045474
LOC_Os01g32364	LOC_Os01g38970	9.13910541
LOC_Os01g35850	LOC_Os01g49290	9.37221083
LOC_Os01g34480	LOC_Os09g08072	9.37379854
LOC_Os01g34480	LOC_Os05g26890	10.6869466
LOC_Os01g34480	LOC_Os10g38690	10.0719044
LOC_Os01g33160	LOC_Os03g51740	9.06477334
LOC_Os01g32364	LOC_Os03g57140	10.1677406
LOC_Os01g33160	LOC_Os05g27950	9.00237702
LOC_Os01g34614	LOC_Os01g45400	8.83834426
LOC_Os01g29780	LOC_Os08g31870	9.54206838
LOC_Os01g34480	LOC_Os03g58630	11.7680168
LOC_Os01g34614	LOC_Os06g06880	8.8347271
LOC_Os01g34614	LOC_Os01g70300	9.63606782
LOC_Os01g33160	LOC_Os02g52610	9.29888484
LOC_Os01g32364	LOC_Os06g51084	8.83002494
LOC_Os01g34480	LOC_Os08g04540	9.65575019
LOC_Os01g32364	LOC_Os12g21798	9.11277537
LOC_Os01g32364	LOC_Os05g06450	9.50182674
LOC_Os01g34480	LOC_Os10g30840	9.33585473
LOC_Os01g35850	LOC_Os06g35530	9.44414306
LOC_Os01g33160	LOC_Os10g39880	8.83092518
LOC_Os01g32364	LOC_Os03g04410	8.92409008
LOC_Os01g34480	LOC_Os03g10620	12.4945893
LOC_Os01g34614	LOC_Os10g27174	9.85331936
LOC_Os01g33160	LOC_Os07g26900	9.42610611
LOC_Os01g34614	LOC_Os12g36950	9.89321746
LOC_Os01g34480	LOC_Os12g12470	9.26117445
LOC_Os01g34480	LOC_Os02g47600	9.20687
LOC_Os01g34480	LOC_Os02g32490	10.0363471
LOC_Os01g32364	LOC_Os10g08670	9.78383976

InteractorA	InteractorB	Z score
LOC_Os01g34480	LOC_Os01g52400	8.85303627
LOC_Os01g34480	LOC_Os07g34580	9.61874915
LOC_Os01g34614	LOC_Os09g08720	10.3898688
LOC_Os01g34480	LOC_Os10g39120	9.25957925
LOC_Os01g34480	LOC_Os10g31940	11.1719127
LOC_Os01g34480	LOC_Os09g12660	9.77180249
LOC_Os01g34480	LOC_Os02g02960	9.38664552
LOC_Os01g34614	LOC_Os03g44484	9.61455464
LOC_Os01g34614	LOC_Os08g34280	9.86875793
LOC_Os01g32364	LOC_Os08g32620	9.0634197
LOC_Os01g34480	LOC_Os03g62070	10.5466192
LOC_Os01g34614	LOC_Os05g27940	8.9293419
LOC_Os01g35850	LOC_Os07g17010	8.86873187
LOC_Os01g34480	LOC_Os01g64970	11.184977
LOC_Os01g34480	LOC_Os07g05940	9.61875082
LOC_Os01g34480	LOC_Os02g14430	9.97279093
LOC_Os01g34480	LOC_Os01g71340	10.0443658
LOC_Os01g34480	LOC_Os07g20544	11.3446765
LOC_Os01g34480	LOC_Os03g16020	9.47078865
LOC_Os01g34480	LOC_Os06g07080	8.85841208
LOC_Os01g34480	LOC_Os01g55950	8.89260605
LOC_Os01g33160	LOC_Os04g18200	9.38846428
LOC_Os01g34480	LOC_Os05g44340	10.0904755
LOC_Os01g34480	LOC_Os12g04980	9.09086438
LOC_Os01g34480	LOC_Os03g30950	11.5274467
LOC_Os01g34614	LOC_Os10g38160	8.81534906
LOC_Os01g34480	LOC_Os03g03130	9.88292684
LOC_Os01g34480	LOC_Os04g38940	8.87697411
LOC_Os01g32364	LOC_Os01g62080	8.82840992
LOC_Os01g33160	LOC_Os07g08660	9.70128837
LOC_Os01g34480	LOC_Os09g28420	10.4770438
LOC_Os01g34480	LOC_Os04g58680	9.17100485
LOC_Os01g34480	LOC_Os02g01590	9.92840152
LOC_Os01g34480	LOC_Os11g25100	9.35115168
LOC_Os01g34480	LOC_Os04g54390	9.29592802
LOC_Os01g34480	LOC_Os01g62870	9.70241694
LOC_Os01g34480	LOC_Os04g45290	9.25468131
LOC_Os01g34614	LOC_Os03g16740	9.98062071
LOC_Os01g34480	LOC_Os05g02940	9.77557285
LOC_Os01g29780	LOC_Os09g37100	9.19496761
LOC_Os01g34480	LOC_Os04g46930	8.922659
LOC_Os01g34480	LOC_Os04g43410	9.09785466
LOC_Os01g34480	LOC_Os05g08100	10.7322629

InteractorA	InteractorB	Z score
LOC_Os01g34614	LOC_Os05g32140	8.87348528
LOC_Os01g34480	LOC_Os07g03710	9.53288363
LOC_Os01g34480	LOC_Os04g56646	10.0801792
LOC_Os01g33160	LOC_Os04g42920	9.39726612
LOC_Os01g34480	LOC_Os11g30290	9.25365683
LOC_Os01g35850	LOC_Os03g62060	9.5360315
LOC_Os01g29780	LOC_Os10g08022	8.85934732
LOC_Os01g35850	LOC_Os05g19150	10.2287866
LOC_Os01g34614	LOC_Os03g16900	9.37301231
LOC_Os01g34480	LOC_Os03g53200	9.36680341
LOC_Os01g33160	LOC_Os02g14460	9.53290644
LOC_Os01g34480	LOC_Os07g23470	11.8129488
LOC_Os01g34480	LOC_Os02g12580	9.02312745
LOC_Os01g35850	LOC_Os05g34170	8.80697529
LOC_Os01g32364	LOC_Os08g23730	9.26040917
LOC_Os01g32364	LOC_Os10g01570	9.40116811
LOC_Os01g33160	LOC_Os06g35480	8.91855147
LOC_Os01g32364	LOC_Os09g26380	9.6683119
LOC_Os01g34480	LOC_Os07g02210	9.68142238
LOC_Os01g34614	LOC_Os01g59150	10.0065791
LOC_Os01g32364	LOC_Os03g53650	9.54360129
LOC_Os01g32364	LOC_Os04g48060	9.40569388
LOC_Os01g32364	LOC_Os06g11280	9.09493383
LOC_Os01g34480	LOC_Os04g59200	9.05121459
LOC_Os01g34614	LOC_Os05g29880	9.69734253
LOC_Os01g33160	LOC_Os09g31120	9.53399333
LOC_Os01g34480	LOC_Os04g33480	9.67393398
LOC_Os01g32364	LOC_Os03g20700	11.0667443
LOC_Os01g34480	LOC_Os01g73580	8.88068763
LOC_Os01g35850	LOC_Os06g42130	8.91512755
LOC_Os01g36070	LOC_Os03g04250	9.74267732
LOC_Os01g37590	LOC_Os06g14510	9.24462552
LOC_Os01g36240	LOC_Os06g21570	9.86630906
LOC_Os01g37800	LOC_Os03g51740	9.11659978
LOC_Os01g36070	LOC_Os03g04110	9.74041796
LOC_Os01g37590	LOC_Os10g38600	8.84800127
LOC_Os01g37590	LOC_Os02g44780	9.74092478
LOC_Os01g37590	LOC_Os07g08880	8.88316158
LOC_Os01g36070	LOC_Os03g26970	9.37194513
LOC_Os01g36240	LOC_Os06g45120	10.9282573
LOC_Os01g37590	LOC_Os07g44590	9.93843451
LOC_Os01g36070	LOC_Os12g14070	10.2218447
LOC_Os01g36240	LOC_Os08g39300	9.50670728

InteractorA	InteractorB	Z score
LOC_Os01g37750	LOC_Os11g25260	9.43546978
LOC_Os01g37590	LOC_Os02g33110	8.88357877
LOC_Os01g36070	LOC_Os08g04540	9.2356293
LOC_Os01g37590	LOC_Os03g58530	9.20457398
LOC_Os01g35850	LOC_Os09g04730	9.0249777
LOC_Os01g36070	LOC_Os07g34580	8.9979364
LOC_Os01g37750	LOC_Os05g45590	9.03183495
LOC_Os01g36240	LOC_Os07g05180	8.98740178
LOC_Os01g37590	LOC_Os01g63270	8.83216082
LOC_Os01g37590	LOC_Os05g45590	9.13628542
LOC_Os01g35850	LOC_Os09g26880	8.86498643
LOC_Os01g37590	LOC_Os03g58050	11.1221325
LOC_Os01g37590	LOC_Os02g51910	9.19127829
LOC_Os01g37590	LOC_Os09g29200	9.33336897
LOC_Os01g36070	LOC_Os06g48200	10.3872548
LOC_Os01g37590	LOC_Os09g15420	9.58161134
LOC_Os01g37590	LOC_Os04g57410	8.87810459
LOC_Os01g37750	LOC_Os11g14910	9.60133345
LOC_Os01g37800	LOC_Os03g49380	9.41142581
LOC_Os01g37750	LOC_Os01g43390	9.383089
LOC_Os01g37800	LOC_Os01g70300	9.02344904
LOC_Os01g36240	LOC_Os07g46310	8.80209093
LOC_Os01g37800	LOC_Os06g04620	9.14247299
LOC_Os01g35850	LOC_Os11g26910	8.82549604
LOC_Os01g36070	LOC_Os10g26390	9.46228838
LOC_Os01g36070	LOC_Os02g01590	8.89450514
LOC_Os01g36070	LOC_Os07g07550	9.0897854
LOC_Os01g36240	LOC_Os01g48600	9.81217348
LOC_Os01g36240	LOC_Os11g08445	10.2721513
LOC_Os01g36240	LOC_Os02g51830	10.120509
LOC_Os01g37750	LOC_Os07g07320	9.3515245
LOC_Os01g37590	LOC_Os09g21770	9.09858035
LOC_Os01g37590	LOC_Os07g07709	11.1221325
LOC_Os01g36070	LOC_Os07g34589	8.88784911
LOC_Os01g36240	LOC_Os03g41438	9.14802261
LOC_Os01g37590	LOC_Os03g40330	9.22908572
LOC_Os01g37590	LOC_Os10g28120	9.69153269
LOC_Os01g36240	LOC_Os09g28770	10.589553
LOC_Os01g37750	LOC_Os10g38140	8.87012047
LOC_Os01g36070	LOC_Os05g33150	9.45507796
LOC_Os01g36240	LOC_Os04g40290	9.33806566
LOC_Os01g37750	LOC_Os10g38340	9.59149867
LOC_Os01g37590	LOC_Os09g12570	8.86991062

InteractorA	InteractorB	Z score
LOC_Os01g37750	LOC_Os05g29880	8.92354962
LOC_Os01g36070	LOC_Os10g32550	8.81793373
LOC_Os01g35850	LOC_Os12g38180	9.76939698
LOC_Os01g35850	LOC_Os12g38770	9.80973212
LOC_Os01g37590	LOC_Os12g25690	8.94078212
LOC_Os01g37750	LOC_Os06g36670	9.05060958
LOC_Os01g37837	LOC_Os07g30090	8.86437176
LOC_Os01g38670	LOC_Os02g06700	9.22917961
LOC_Os01g37960	LOC_Os10g38740	10.6041461
LOC_Os01g38500	LOC_Os01g51570	9.97550679
LOC_Os01g38500	LOC_Os01g57610	9.04390862
LOC_Os01g37910	LOC_Os01g43390	8.85486995
LOC_Os01g37837	LOC_Os04g32650	11.358784
LOC_Os01g37960	LOC_Os04g58110	10.3450111
LOC_Os01g37837	LOC_Os06g39230	9.16859052
LOC_Os01g37837	LOC_Os10g05069	8.98701287
LOC_Os01g38500	LOC_Os08g09210	10.1219888
LOC_Os01g37910	LOC_Os07g41750	8.88642508
LOC_Os01g37960	LOC_Os02g04100	9.61966204
LOC_Os01g37960	LOC_Os12g17910	11.2583805
LOC_Os01g38500	LOC_Os03g51550	9.40409722
LOC_Os01g37960	LOC_Os04g43760	9.21993841
LOC_Os01g37960	LOC_Os05g12210	9.22655888
LOC_Os01g37960	LOC_Os11g02440	9.11684228
LOC_Os01g37960	LOC_Os01g38670	9.77345019
LOC_Os01g38670	LOC_Os04g44924	8.82125451
LOC_Os01g37960	LOC_Os01g49890	8.83131154
LOC_Os01g38500	LOC_Os01g71820	10.6394391
LOC_Os01g37960	LOC_Os05g43510	9.37817923
LOC_Os01g37960	LOC_Os06g04270	9.95904827
LOC_Os01g37910	LOC_Os03g50490	8.81229177
LOC_Os01g37960	LOC_Os03g22020	9.69883434
LOC_Os01g37837	LOC_Os03g10190	12.88362
LOC_Os01g38500	LOC_Os04g53230	8.81646704
LOC_Os01g38500	LOC_Os02g04950	12.1853438
LOC_Os01g37960	LOC_Os04g37460	9.63002686
LOC_Os01g37837	LOC_Os10g29620	11.5976922
LOC_Os01g37837	LOC_Os02g20850	9.80690826
LOC_Os01g38500	LOC_Os03g12290	8.93535129
LOC_Os01g37800	LOC_Os10g08580	9.15127742
LOC_Os01g38500	LOC_Os04g39020	10.3101783
LOC_Os01g37837	LOC_Os02g01510	8.89807246
LOC_Os01g37837	LOC_Os03g55280	9.28893308

InteractorA	InteractorB	Z score
LOC_Os01g37910	LOC_Os08g14770	8.90843114
LOC_Os01g37960	LOC_Os12g10730	10.6659106
LOC_Os01g38670	LOC_Os07g01760	8.9849421
LOC_Os01g37960	LOC_Os03g37950	9.76841697
LOC_Os01g38670	LOC_Os02g07260	9.05241802
LOC_Os01g37910	LOC_Os03g56410	9.98077736
LOC_Os01g37837	LOC_Os04g43400	8.85963344
LOC_Os01g38500	LOC_Os03g38980	9.66831157
LOC_Os01g37837	LOC_Os06g15420	9.05792133
LOC_Os01g37960	LOC_Os02g46970	9.63604139
LOC_Os01g37960	LOC_Os02g52630	9.32612049
LOC_Os01g37960	LOC_Os10g41490	8.81021304
LOC_Os01g38500	LOC_Os10g21326	9.89373466
LOC_Os01g38500	LOC_Os01g71990	8.80075742
LOC_Os01g37960	LOC_Os03g44170	9.33033009
LOC_Os01g37910	LOC_Os09g12570	9.34301614
LOC_Os01g37837	LOC_Os02g52640	11.2482781
LOC_Os01g37837	LOC_Os07g05150	8.85346779
LOC_Os01g38500	LOC_Os03g50490	9.07382823
LOC_Os01g38670	LOC_Os02g55420	9.40298488
LOC_Os01g38670	LOC_Os05g19380	9.6289429
LOC_Os01g37960	LOC_Os05g51570	9.16010317
LOC_Os01g38500	LOC_Os04g52440	9.30280079
LOC_Os01g37837	LOC_Os09g10270	9.17287281
LOC_Os01g38500	LOC_Os02g50240	9.32253695
LOC_Os01g37960	LOC_Os01g72460	11.9662235
LOC_Os01g37960	LOC_Os07g02210	11.7023654
LOC_Os01g37960	LOC_Os02g50860	9.97384474
LOC_Os01g37960	LOC_Os03g01900	9.90453713
LOC_Os01g37960	LOC_Os11g02130	8.8535315
LOC_Os01g37800	LOC_Os12g06660	10.022521
LOC_Os01g38500	LOC_Os06g44620	9.0124055
LOC_Os01g37837	LOC_Os04g57410	9.29278856
LOC_Os01g37837	LOC_Os03g15360	9.14429922
LOC_Os01g37910	LOC_Os05g39580	9.26091552
LOC_Os01g37960	LOC_Os10g39840	9.77834121
LOC_Os01g37800	LOC_Os12g43370	9.8227432
LOC_Os01g37960	LOC_Os02g36974	8.96422597
LOC_Os01g37960	LOC_Os07g34580	10.3178574
LOC_Os01g37960	LOC_Os01g71310	9.01742475
LOC_Os01g38670	LOC_Os01g70140	9.0216046
LOC_Os01g37837	LOC_Os03g21950	9.36506278
LOC_Os01g37960	LOC_Os04g42250	8.97110893

InteractorA	InteractorB	Z score
LOC_Os01g38500	LOC_Os05g48510	11.7463646
LOC_Os01g37837	LOC_Os08g41880	9.39319642
LOC_Os01g37960	LOC_Os12g41220	8.99816547
LOC_Os01g38500	LOC_Os07g43260	9.06544147
LOC_Os01g38500	LOC_Os01g57945	9.89373466
LOC_Os01g37837	LOC_Os11g25700	9.16174425
LOC_Os01g37837	LOC_Os03g04410	9.14282049
LOC_Os01g37910	LOC_Os05g46360	9.17457672
LOC_Os01g38500	LOC_Os04g16844	9.89372673
LOC_Os01g37960	LOC_Os01g73740	8.83824289
LOC_Os01g39830	LOC_Os02g15640	9.63463014
LOC_Os01g38670	LOC_Os10g11140	8.99797088
LOC_Os01g38970	LOC_Os01g71380	10.1847728
LOC_Os01g38970	LOC_Os12g12560	8.81908494
LOC_Os01g38680	LOC_Os01g38970	11.0332236
LOC_Os01g38970	LOC_Os03g08010	10.9115205
LOC_Os01g38970	LOC_Os07g39870	8.82627347
LOC_Os01g38970	LOC_Os07g22650	9.66915957
LOC_Os01g38970	LOC_Os01g71474	8.9365821
LOC_Os01g39270	LOC_Os08g06100	8.85936844
LOC_Os01g39270	LOC_Os03g61740	8.840726
LOC_Os01g39280	LOC_Os06g05690	9.16247773
LOC_Os01g39270	LOC_Os01g54370	11.0210321
LOC_Os01g38970	LOC_Os02g01920	8.94039715
LOC_Os01g39280	LOC_Os04g37480	8.98943169
LOC_Os01g38970	LOC_Os10g11140	9.39842943
LOC_Os01g38970	LOC_Os06g03770	11.200609
LOC_Os01g38970	LOC_Os04g17650	10.8335812
LOC_Os01g39280	LOC_Os03g20710	9.10750455
LOC_Os01g39280	LOC_Os08g23730	9.79951221
LOC_Os01g39830	LOC_Os06g01590	9.33782185
LOC_Os01g38680	LOC_Os06g40940	9.06476307
LOC_Os01g38970	LOC_Os08g16910	9.83876792
LOC_Os01g38970	LOC_Os12g25630	8.88998837
LOC_Os01g39270	LOC_Os07g42960	9.38259297
LOC_Os01g38970	LOC_Os06g45710	9.0275734
LOC_Os01g39270	LOC_Os07g12200	8.88376222
LOC_Os01g39270	LOC_Os06g46372	9.52458998
LOC_Os01g39270	LOC_Os03g45270	8.9063867
LOC_Os01g39270	LOC_Os09g31120	8.88748068
LOC_Os01g38970	LOC_Os05g46580	10.8010711
LOC_Os01g39270	LOC_Os05g33730	9.30474906
LOC_Os01g38970	LOC_Os04g22660	9.06195158

InteractorA	InteractorB	Z score
LOC_Os01g39830	LOC_Os01g55940	9.41347435
LOC_Os01g39830	LOC_Os04g05080	8.8380288
LOC_Os01g39270	LOC_Os08g03040	8.98145829
LOC_Os01g38670	LOC_Os07g49400	9.0074883
LOC_Os01g38970	LOC_Os03g16920	9.39077307
LOC_Os01g39270	LOC_Os06g23440	8.98849935
LOC_Os01g39270	LOC_Os04g55040	8.9632509
LOC_Os01g38970	LOC_Os04g29030	9.33291605
LOC_Os01g38970	LOC_Os02g33080	8.97248021
LOC_Os01g38970	LOC_Os06g06980	10.2371599
LOC_Os01g38970	LOC_Os07g08500	10.849583
LOC_Os01g38670	LOC_Os09g26380	8.95843781
LOC_Os01g38970	LOC_Os12g42280	9.56488076
LOC_Os01g39270	LOC_Os03g10940	9.07844716
LOC_Os01g38970	LOC_Os06g51150	8.94401088
LOC_Os01g39830	LOC_Os03g37950	9.15548291
LOC_Os01g38680	LOC_Os12g12580	9.5258086
LOC_Os01g38970	LOC_Os01g50050	9.6919948
LOC_Os01g39830	LOC_Os06g11280	8.88042487
LOC_Os01g38970	LOC_Os03g45410	9.23220695
LOC_Os01g39270	LOC_Os03g60580	9.19774274
LOC_Os01g38970	LOC_Os07g44790	9.18427829
LOC_Os01g39280	LOC_Os10g26600	9.22259754
LOC_Os01g38970	LOC_Os10g25950	8.84981981
LOC_Os01g38970	LOC_Os03g28400	9.18427787
LOC_Os01g39280	LOC_Os07g29440	9.16646855
LOC_Os01g38970	LOC_Os07g26900	8.86638723
LOC_Os01g38970	LOC_Os07g48010	15.0030093
LOC_Os01g39270	LOC_Os06g40190	8.83936341
LOC_Os01g39280	LOC_Os06g51084	8.95071463
LOC_Os01g39270	LOC_Os03g11990	9.10318994
LOC_Os01g38970	LOC_Os07g48020	8.91921409
LOC_Os01g38970	LOC_Os01g74000	9.31042048
LOC_Os01g38670	LOC_Os12g16220	10.5194358
LOC_Os01g38970	LOC_Os12g35570	9.03405377
LOC_Os01g39270	LOC_Os05g41080	9.64161286
LOC_Os01g38670	LOC_Os12g12580	8.90092717
LOC_Os01g38970	LOC_Os07g23730	8.92881173
LOC_Os01g39280	LOC_Os05g47540	8.84114737
LOC_Os01g39270	LOC_Os02g10990	9.87285812
LOC_Os01g39270	LOC_Os03g59060	9.16235828
LOC_Os01g39270	LOC_Os02g02410	9.16300268
LOC_Os01g38970	LOC_Os05g19380	9.03097253

InteractorA	InteractorB	Z score
LOC_Os01g38680	LOC_Os01g70140	8.96065476
LOC_Os01g38970	LOC_Os03g31300	9.30696288
LOC_Os01g38670	LOC_Os10g25140	10.2310744
LOC_Os01g38970	LOC_Os02g53420	9.00806382
LOC_Os01g38970	LOC_Os04g44924	8.81430757
LOC_Os01g38970	LOC_Os02g50880	9.31208072
LOC_Os01g38970	LOC_Os06g34690	9.1169932
LOC_Os01g38970	LOC_Os11g05570	9.20699195
LOC_Os01g38680	LOC_Os05g49760	10.2040132
LOC_Os01g38970	LOC_Os07g08880	8.98741157
LOC_Os01g38970	LOC_Os12g10720	9.99766469
LOC_Os01g38970	LOC_Os11g37890	11.6538949
LOC_Os01g39280	LOC_Os06g06050	10.5705966
LOC_Os01g39830	LOC_Os04g33040	9.03972968
LOC_Os01g39270	LOC_Os03g20700	10.7300699
LOC_Os01g38970	LOC_Os03g08050	10.9115205
LOC_Os01g38970	LOC_Os04g39900	8.98956806
LOC_Os01g38970	LOC_Os01g66720	9.21909304
LOC_Os01g38970	LOC_Os02g57150	8.80528462
LOC_Os01g38970	LOC_Os03g59740	10.5584933
LOC_Os01g38970	LOC_Os03g08020	10.9115205
LOC_Os01g39830	LOC_Os02g02840	8.90400443
LOC_Os01g38970	LOC_Os05g12240	8.95661941
LOC_Os01g38970	LOC_Os12g23630	9.20206867
LOC_Os01g38970	LOC_Os02g14929	9.55635066
LOC_Os01g38970	LOC_Os09g37100	9.68433872
LOC_Os01g38970	LOC_Os03g61600	11.8084132
LOC_Os01g39830	LOC_Os01g59730	9.13925903
LOC_Os01g38970	LOC_Os08g28800	8.81334781
LOC_Os01g38970	LOC_Os10g08620	11.2642163
LOC_Os01g38970	LOC_Os01g39830	8.84242426
LOC_Os01g39830	LOC_Os04g39880	9.48376811
LOC_Os01g39270	LOC_Os08g17784	8.80982869
LOC_Os01g39270	LOC_Os04g26920	9.00768454
LOC_Os01g38970	LOC_Os05g34170	9.15805892
LOC_Os01g38970	LOC_Os02g19770	9.15275692
LOC_Os01g39270	LOC_Os02g51830	9.28242713
LOC_Os01g38970	LOC_Os07g43260	9.23782922
LOC_Os01g39270	LOC_Os01g73790	9.42465039
LOC_Os01g38970	LOC_Os11g05470	10.8796279
LOC_Os01g38970	LOC_Os11g32260	9.23987444
LOC_Os01g39280	LOC_Os02g08420	8.9553906
LOC_Os01g40860	LOC_Os04g39020	8.97638847

InteractorA	InteractorB	Z score
LOC_Os01g40840	LOC_Os08g37790	9.11050434
LOC_Os01g41510	LOC_Os02g56680	10.3291829
LOC_Os01g40860	LOC_Os08g44270	9.04951899
LOC_Os01g40860	LOC_Os04g53850	9.24700094
LOC_Os01g40860	LOC_Os05g31040	9.06781479
LOC_Os01g41510	LOC_Os01g46070	9.04415003
LOC_Os01g40870	LOC_Os03g28400	9.5697627
LOC_Os01g40870	LOC_Os11g47560	8.92859687
LOC_Os01g40400	LOC_Os08g09210	9.555825
LOC_Os01g40870	LOC_Os03g50490	8.9505872
LOC_Os01g40860	LOC_Os06g10950	8.81180251
LOC_Os01g39830	LOC_Os12g34062	9.00802474
LOC_Os01g40870	LOC_Os08g31060	9.10717117
LOC_Os01g40400	LOC_Os02g58340	8.98955401
LOC_Os01g41510	LOC_Os02g09150	9.40236155
LOC_Os01g40400	LOC_Os12g39630	9.67453097
LOC_Os01g40400	LOC_Os11g08940	9.29445994
LOC_Os01g40870	LOC_Os03g05590	9.54887612
LOC_Os01g40400	LOC_Os04g10400	8.90204443
LOC_Os01g40870	LOC_Os02g07760	9.32429869
LOC_Os01g40400	LOC_Os06g10910	9.76463526
LOC_Os01g40860	LOC_Os12g44030	9.38218682
LOC_Os01g40860	LOC_Os05g28280	9.38108968
LOC_Os01g40400	LOC_Os02g48290	9.04585303
LOC_Os01g40860	LOC_Os12g13320	9.00829357
LOC_Os01g41510	LOC_Os07g05580	10.8842644
LOC_Os01g40840	LOC_Os12g10730	9.28923786
LOC_Os01g40860	LOC_Os04g12980	8.89976026
LOC_Os01g40400	LOC_Os01g60190	8.90118731
LOC_Os01g40400	LOC_Os07g07719	9.41941858
LOC_Os01g41510	LOC_Os07g26690	9.02369452
LOC_Os01g40860	LOC_Os01g49190	9.35461285
LOC_Os01g40400	LOC_Os05g11730	8.93206277
LOC_Os01g40400	LOC_Os05g22724	10.0750286
LOC_Os01g41510	LOC_Os05g47540	11.5674324
LOC_Os01g40860	LOC_Os08g37490	9.46537424
LOC_Os01g40870	LOC_Os07g48010	9.16769839
LOC_Os01g40400	LOC_Os05g41210	9.49275942
LOC_Os01g40840	LOC_Os11g31620	8.91438327
LOC_Os01g40840	LOC_Os12g40550	8.86097038
LOC_Os01g40400	LOC_Os01g50050	9.78407204
LOC_Os01g40860	LOC_Os02g07760	10.1789067
LOC_Os01g41510	LOC_Os06g02380	8.83812879

InteractorA	InteractorB	Z score
LOC_Os01g40860	LOC_Os04g50880	9.00245188
LOC_Os01g40870	LOC_Os01g49890	9.27049528
LOC_Os01g40840	LOC_Os05g06970	9.68344794
LOC_Os01g40870	LOC_Os04g41310	10.0392524
LOC_Os01g40840	LOC_Os12g07830	9.02876073
LOC_Os01g40870	LOC_Os04g28870	8.81098368
LOC_Os01g41510	LOC_Os02g56720	9.30256522
LOC_Os01g40840	LOC_Os03g49600	10.9767132
LOC_Os01g40400	LOC_Os04g29550	10.592019
LOC_Os01g40400	LOC_Os07g35940	9.67745737
LOC_Os01g41510	LOC_Os04g30420	9.47311729
LOC_Os01g41510	LOC_Os05g38310	9.64579563
LOC_Os01g40840	LOC_Os05g46290	8.87122288
LOC_Os01g40870	LOC_Os01g58380	9.73045961
LOC_Os01g40840	LOC_Os08g04540	9.78226335
LOC_Os01g39830	LOC_Os11g32260	9.2329334
LOC_Os01g40860	LOC_Os05g36010	8.86069839
LOC_Os01g40870	LOC_Os04g02050	9.2696617
LOC_Os01g41510	LOC_Os06g06300	8.93591609
LOC_Os01g40860	LOC_Os10g39590	8.84837983
LOC_Os01g41510	LOC_Os03g38020	9.3606038
LOC_Os01g40840	LOC_Os05g27950	9.06198722
LOC_Os01g41510	LOC_Os02g08420	9.9124287
LOC_Os01g40840	LOC_Os02g52420	9.39893684
LOC_Os01g40870	LOC_Os03g15960	8.9908441
LOC_Os01g40840	LOC_Os06g37660	9.51560159
LOC_Os01g40840	LOC_Os03g09080	9.70253141
LOC_Os01g40840	LOC_Os08g35740	9.70991188
LOC_Os01g40840	LOC_Os11g41130	9.2803773
LOC_Os01g40870	LOC_Os07g43170	10.2183804
LOC_Os01g40840	LOC_Os05g38550	9.2610345
LOC_Os01g41510	LOC_Os03g53200	9.15032804
LOC_Os01g40860	LOC_Os01g40870	11.8777859
LOC_Os01g40870	LOC_Os02g24632	8.8757467
LOC_Os01g40870	LOC_Os07g05820	10.1615578
LOC_Os01g39830	LOC_Os10g07040	10.6129416
LOC_Os01g40400	LOC_Os11g25330	10.8244791
LOC_Os01g41510	LOC_Os04g14680	10.0014535
LOC_Os01g40840	LOC_Os01g71474	9.17933979
LOC_Os01g40860	LOC_Os07g08170	9.6335269
LOC_Os01g40860	LOC_Os09g39440	9.13534544
LOC_Os01g41510	LOC_Os02g52710	9.65478151
LOC_Os01g40860	LOC_Os02g47790	9.07007335

InteractorA	InteractorB	Z score
LOC_Os01g40840	LOC_Os04g12710	8.89977166
LOC_Os01g40840	LOC_Os02g44780	9.03681527
LOC_Os01g41510	LOC_Os01g60190	8.92661556
LOC_Os01g39830	LOC_Os10g28200	9.31620397
LOC_Os01g41510	LOC_Os06g35730	8.95471092
LOC_Os01g41510	LOC_Os04g53920	9.15884778
LOC_Os01g40400	LOC_Os05g19380	9.76900725
LOC_Os01g40860	LOC_Os05g33570	8.82528161
LOC_Os01g40400	LOC_Os12g40550	10.5992999
LOC_Os01g41510	LOC_Os01g59930	9.1989428
LOC_Os01g41510	LOC_Os06g04280	8.80964601
LOC_Os01g40400	LOC_Os06g35660	8.9832125
LOC_Os01g40860	LOC_Os01g64970	9.37175599
LOC_Os01g41510	LOC_Os04g39840	11.8251023
LOC_Os01g40400	LOC_Os09g36900	10.4433731
LOC_Os01g40870	LOC_Os04g39020	9.38915706
LOC_Os01g41510	LOC_Os02g51910	8.85322764
LOC_Os01g40840	LOC_Os12g38180	9.37437747
LOC_Os01g40840	LOC_Os12g06620	9.46517923
LOC_Os01g40870	LOC_Os11g04954	9.85134604
LOC_Os01g40860	LOC_Os10g40720	8.91518786
LOC_Os01g41510	LOC_Os03g17690	10.2046753
LOC_Os01g41510	LOC_Os02g52700	9.6548224
LOC_Os01g39830	LOC_Os12g01922	10.2408464
LOC_Os01g39830	LOC_Os07g44460	9.38673343
LOC_Os01g40870	LOC_Os10g39590	11.8378401
LOC_Os01g41510	LOC_Os05g11710	8.95475712
LOC_Os01g41510	LOC_Os01g71310	9.33280752
LOC_Os01g40860	LOC_Os09g14670	8.92308835
LOC_Os01g40840	LOC_Os02g57040	9.89318264
LOC_Os01g40870	LOC_Os05g28280	9.32340176
LOC_Os01g40400	LOC_Os11g26850	10.9984224
LOC_Os01g41510	LOC_Os01g57570	9.02376902
LOC_Os01g40870	LOC_Os07g44790	9.5697627
LOC_Os01g40870	LOC_Os06g21570	9.54924812
LOC_Os01g40400	LOC_Os07g05820	10.2495939
LOC_Os01g40400	LOC_Os05g38560	9.46465225
LOC_Os01g40860	LOC_Os03g52460	9.00859123
LOC_Os01g40860	LOC_Os03g51200	9.51880161
LOC_Os01g40860	LOC_Os10g38189	8.94310162
LOC_Os01g40840	LOC_Os08g15292	11.7175276
LOC_Os01g40400	LOC_Os03g63720	8.95255986
LOC_Os01g40860	LOC_Os06g09450	9.82744831

InteractorA	InteractorB	Z score
LOC_Os01g40400	LOC_Os02g44780	9.89473948
LOC_Os01g41510	LOC_Os04g46560	9.0402565
LOC_Os01g39830	LOC_Os11g26910	9.41145008
LOC_Os01g41510	LOC_Os02g35500	9.48541883
LOC_Os01g40870	LOC_Os04g12710	9.82248497
LOC_Os01g40860	LOC_Os01g73190	9.19090313
LOC_Os01g40860	LOC_Os01g74000	10.4038643
LOC_Os01g40400	LOC_Os02g51910	9.20573948
LOC_Os01g02020	LOC_Os09g26380	8.92819953
LOC_Os01g02120	LOC_Os12g13390	10.0261694
LOC_Os01g03730	LOC_Os09g23530	8.8510922
LOC_Os01g02120	LOC_Os05g04340	9.24314494
LOC_Os01g03730	LOC_Os06g05250	9.5771862
LOC_Os01g02880	LOC_Os10g21248	9.6099414
LOC_Os01g03950	LOC_Os03g12270	9.27087493
LOC_Os01g03730	LOC_Os04g42930	8.94846695
LOC_Os01g03740	LOC_Os04g44920	9.13693881
LOC_Os01g03730	LOC_Os07g16970	9.09326339
LOC_Os01g03740	LOC_Os02g32030	9.07042106
LOC_Os01g02120	LOC_Os06g37610	10.3396217
LOC_Os01g02880	LOC_Os05g44760	8.94462228
LOC_Os01g03950	LOC_Os02g55060	9.28959452
LOC_Os01g02120	LOC_Os07g05160	8.93372012
LOC_Os01g02120	LOC_Os03g13140	8.81945313
LOC_Os01g02880	LOC_Os12g04980	9.96863613
LOC_Os01g02880	LOC_Os11g05570	8.91753993
LOC_Os01g02120	LOC_Os08g14760	10.7530191
LOC_Os01g03950	LOC_Os01g71350	8.86490838
LOC_Os01g02120	LOC_Os07g48780	8.90954767
LOC_Os01g02120	LOC_Os01g27490	8.87232807
LOC_Os01g02020	LOC_Os09g38030	8.99594347
LOC_Os01g02020	LOC_Os11g01010	9.87905213
LOC_Os01g03730	LOC_Os12g01922	9.21846139
LOC_Os01g02120	LOC_Os09g31486	9.18845884
LOC_Os01g02880	LOC_Os03g20370	9.84085047
LOC_Os01g02880	LOC_Os01g40860	8.95173648
LOC_Os01g02120	LOC_Os12g08270	9.14105446
LOC_Os01g02120	LOC_Os03g31300	10.1881043
LOC_Os01g02880	LOC_Os11g01872	8.99156424
LOC_Os01g02120	LOC_Os10g21310	9.03242773
LOC_Os01g02120	LOC_Os04g55410	9.44920802
LOC_Os01g03740	LOC_Os12g39630	9.10312268
LOC_Os01g02120	LOC_Os03g20370	8.90954788

InteractorA	InteractorB	Z score
LOC_Os01g02880	LOC_Os10g38340	9.24234146
LOC_Os01g02880	LOC_Os01g16240	9.84083085
LOC_Os01g02880	LOC_Os03g50490	9.40638174
LOC_Os01g03730	LOC_Os02g39064	9.37477447
LOC_Os01g02120	LOC_Os02g14110	8.85174223
LOC_Os01g03730	LOC_Os04g10400	9.24149609
LOC_Os01g02880	LOC_Os04g37820	8.94986502
LOC_Os01g02020	LOC_Os08g34790	10.5412742
LOC_Os01g02120	LOC_Os05g31020	9.03101386
LOC_Os01g02020	LOC_Os10g26600	9.3858851
LOC_Os01g02880	LOC_Os03g53800	9.66681086
LOC_Os01g02880	LOC_Os07g48780	9.84086459
LOC_Os01g02020	LOC_Os09g37949	9.30971812
LOC_Os01g03730	LOC_Os03g04250	11.2573091
LOC_Os01g02020	LOC_Os08g33100	9.40155639
LOC_Os01g02880	LOC_Os11g04954	9.05220077
LOC_Os01g03740	LOC_Os02g04950	9.21891221
LOC_Os01g02120	LOC_Os05g29880	10.8323213
LOC_Os01g03730	LOC_Os09g10270	9.00479079
LOC_Os01g02120	LOC_Os10g32970	8.85294961
LOC_Os01g02880	LOC_Os10g08022	9.20371105
LOC_Os01g02120	LOC_Os03g12510	9.25016545
LOC_Os01g03950	LOC_Os01g17190	9.2712072
LOC_Os01g02120	LOC_Os01g16240	8.90955874
LOC_Os01g03730	LOC_Os01g61400	8.88176224
LOC_Os01g02120	LOC_Os01g46070	8.99519892
LOC_Os01g03740	LOC_Os12g40550	9.22343731
LOC_Os01g03740	LOC_Os02g52560	9.79247093
LOC_Os01g42470	LOC_Os03g18580	9.00256956
LOC_Os01g42470	LOC_Os09g27820	9.67017833
LOC_Os01g41630	LOC_Os02g47600	10.4516288
LOC_Os01g43090	LOC_Os05g41230	10.8415854
LOC_Os01g41630	LOC_Os06g10930	9.21354447
LOC_Os01g42470	LOC_Os09g16910	8.93631206
LOC_Os01g41630	LOC_Os04g33040	9.25594147
LOC_Os01g41630	LOC_Os05g07090	9.18440276
LOC_Os01g42470	LOC_Os09g15320	9.08285769
LOC_Os01g41630	LOC_Os04g59200	10.1823951
LOC_Os01g41630	LOC_Os08g44520	9.75558122
LOC_Os01g41630	LOC_Os10g21406	8.96863032
LOC_Os01g43090	LOC_Os05g29880	9.22184113
LOC_Os01g43090	LOC_Os04g27980	10.2351451
LOC_Os01g41630	LOC_Os03g17310	10.6613327

InteractorA	InteractorB	Z score
LOC_Os01g42690	LOC_Os09g36830	10.0585682
LOC_Os01g43090	LOC_Os02g18880	9.30276965
LOC_Os01g41710	LOC_Os09g26880	11.9296181
LOC_Os01g42690	LOC_Os04g24430	9.40849051
LOC_Os01g42470	LOC_Os12g07980	8.93819779
LOC_Os01g42470	LOC_Os08g02700	9.63119232
LOC_Os01g41630	LOC_Os09g23530	9.07501012
LOC_Os01g41630	LOC_Os10g01570	8.81956524
LOC_Os01g42470	LOC_Os05g48290	8.88217422
LOC_Os01g43090	LOC_Os06g21570	9.78157757
LOC_Os01g43090	LOC_Os07g23730	9.70422884
LOC_Os01g42470	LOC_Os03g05590	9.22903706
LOC_Os01g42470	LOC_Os07g35940	9.48738127
LOC_Os01g43090	LOC_Os05g11550	10.4372777
LOC_Os01g41630	LOC_Os07g22498	8.96864745
LOC_Os01g41510	LOC_Os10g25674	9.70368059
LOC_Os01g41630	LOC_Os01g47550	9.03459863
LOC_Os01g41630	LOC_Os03g19390	9.15539291
LOC_Os01g43090	LOC_Os05g35320	9.51726507
LOC_Os01g41630	LOC_Os01g59600	10.2766069
LOC_Os01g43090	LOC_Os02g41590	9.28991886
LOC_Os01g42690	LOC_Os11g26860	9.3281206
LOC_Os01g42470	LOC_Os11g05880	9.29772462
LOC_Os01g41710	LOC_Os05g19380	8.92131771
LOC_Os01g43090	LOC_Os06g48180	8.94176988
LOC_Os01g42470	LOC_Os04g54330	9.81204034
LOC_Os01g42690	LOC_Os08g39140	9.21762372
LOC_Os01g41710	LOC_Os04g56070	10.0784555
LOC_Os01g41510	LOC_Os10g33800	8.82651798
LOC_Os01g42470	LOC_Os02g47610	9.55085472
LOC_Os01g42690	LOC_Os10g30200	9.02844237
LOC_Os01g41510	LOC_Os08g39860	9.11007505
LOC_Os01g42470	LOC_Os03g57290	9.49169021
LOC_Os01g42470	LOC_Os07g43390	8.87602905
LOC_Os01g41710	LOC_Os08g34790	9.1582083
LOC_Os01g41630	LOC_Os02g10830	8.81569232
LOC_Os01g41630	LOC_Os03g21900	9.48233534
LOC_Os01g41630	LOC_Os06g46436	8.96864745
LOC_Os01g43090	LOC_Os02g18930	9.09560312
LOC_Os01g41710	LOC_Os07g46310	9.19596698
LOC_Os01g41630	LOC_Os05g19150	8.94808729
LOC_Os01g41710	LOC_Os02g32490	8.80854768
LOC_Os01g41630	LOC_Os01g62870	9.29862495

InteractorA	InteractorB	Z score
LOC_Os01g43090	LOC_Os04g36800	8.96795659
LOC_Os01g41630	LOC_Os09g20260	9.04926784
LOC_Os01g41710	LOC_Os12g26290	9.74778424
LOC_Os01g42470	LOC_Os02g25940	8.91961786
LOC_Os01g42470	LOC_Os07g05400	9.16110384
LOC_Os01g42470	LOC_Os08g44270	9.18068376
LOC_Os01g43090	LOC_Os07g01760	9.28474459
LOC_Os01g41630	LOC_Os10g37210	9.50406892
LOC_Os01g42470	LOC_Os07g28280	9.41625912
LOC_Os01g42690	LOC_Os09g31490	9.08589446
LOC_Os01g41630	LOC_Os01g51570	8.97945353
LOC_Os01g43090	LOC_Os04g37480	9.36496363
LOC_Os01g42470	LOC_Os04g12710	8.80038519
LOC_Os01g41630	LOC_Os02g07160	9.15293766
LOC_Os01g42470	LOC_Os08g02410	9.20038078
LOC_Os01g43090	LOC_Os05g41900	9.64398108
LOC_Os01g42470	LOC_Os05g28180	10.7274772
LOC_Os01g42470	LOC_Os02g01280	9.49195973
LOC_Os01g42470	LOC_Os09g04730	8.82995822
LOC_Os01g43090	LOC_Os07g05940	10.0350406
LOC_Os01g41630	LOC_Os10g28320	8.97651673
LOC_Os01g41710	LOC_Os04g37460	8.962796
LOC_Os01g41630	LOC_Os09g31486	9.95947514
LOC_Os01g41630	LOC_Os01g71830	9.21684636
LOC_Os01g41630	LOC_Os02g01510	9.07566269
LOC_Os01g41630	LOC_Os02g47610	9.20532402
LOC_Os01g41710	LOC_Os02g55400	9.55240455
LOC_Os01g43090	LOC_Os01g52490	9.1324944
LOC_Os01g42470	LOC_Os06g07960	9.19831452
LOC_Os01g41710	LOC_Os09g36800	9.16012464
LOC_Os01g41630	LOC_Os04g32650	9.05294224
LOC_Os01g42470	LOC_Os06g43640	8.84834058
LOC_Os01g43090	LOC_Os03g62060	9.10164733
LOC_Os01g43090	LOC_Os01g57962	8.82637762
LOC_Os01g41710	LOC_Os05g06750	10.2178146
LOC_Os01g41630	LOC_Os04g40620	9.18235488
LOC_Os01g42690	LOC_Os09g10230	9.31026521
LOC_Os01g42690	LOC_Os05g31140	9.08761176
LOC_Os01g42690	LOC_Os01g48600	9.16072214
LOC_Os01g41510	LOC_Os09g24530	9.93925926
LOC_Os01g41630	LOC_Os02g50880	9.0144887
LOC_Os01g43390	LOC_Os04g33480	9.71414256
LOC_Os01g44130	LOC_Os09g38020	9.80971577

InteractorA	InteractorB	Z score
LOC_Os01g43090	LOC_Os10g38234	10.0387452
LOC_Os01g44130	LOC_Os01g74350	9.66251861
LOC_Os01g44130	LOC_Os12g38180	8.89530575
LOC_Os01g43530	LOC_Os08g40140	9.90297546
LOC_Os01g44260	LOC_Os03g04970	9.02119102
LOC_Os01g43390	LOC_Os10g26110	8.98350837
LOC_Os01g43390	LOC_Os06g06090	9.10565925
LOC_Os01g44220	LOC_Os02g35760	8.81308735
LOC_Os01g43390	LOC_Os10g02070	9.10122849
LOC_Os01g44130	LOC_Os09g26340	9.80971577
LOC_Os01g44220	LOC_Os05g04340	9.03069105
LOC_Os01g44130	LOC_Os05g31040	11.0370178
LOC_Os01g43390	LOC_Os06g10340	8.90724612
LOC_Os01g44130	LOC_Os02g51830	9.02817775
LOC_Os01g43390	LOC_Os05g12240	9.94647509
LOC_Os01g43390	LOC_Os03g59060	9.91902298
LOC_Os01g44260	LOC_Os07g31770	8.8061234
LOC_Os01g43090	LOC_Os09g10260	9.84852604
LOC_Os01g43530	LOC_Os05g44760	9.43138974
LOC_Os01g43390	LOC_Os04g39900	10.2901283
LOC_Os01g44260	LOC_Os03g50290	9.29567852
LOC_Os01g43390	LOC_Os08g23730	9.21696054
LOC_Os01g44130	LOC_Os03g04169	8.86242433
LOC_Os01g44130	LOC_Os02g45940	9.80973651
LOC_Os01g44130	LOC_Os03g60400	9.53517388
LOC_Os01g43530	LOC_Os10g01570	9.20494201
LOC_Os01g44260	LOC_Os05g48030	8.82870237
LOC_Os01g44260	LOC_Os03g45410	9.06598544
LOC_Os01g44130	LOC_Os09g04050	9.07485889
LOC_Os01g44220	LOC_Os06g37560	9.30333474
LOC_Os01g43390	LOC_Os03g58980	8.84567943
LOC_Os01g44220	LOC_Os02g14460	8.87091044
LOC_Os01g44130	LOC_Os11g10520	9.77824158
LOC_Os01g44130	LOC_Os05g39050	9.80972127
LOC_Os01g43390	LOC_Os12g44000	9.22702725
LOC_Os01g43530	LOC_Os01g62860	9.54056181
LOC_Os01g44130	LOC_Os06g40180	9.07647111
LOC_Os01g43530	LOC_Os01g62870	9.87676076
LOC_Os01g44220	LOC_Os01g52470	8.89616774
LOC_Os01g44260	LOC_Os02g02840	9.36853847
LOC_Os01g44130	LOC_Os01g63890	10.1613857
LOC_Os01g44260	LOC_Os06g35520	9.3856206
LOC_Os01g44130	LOC_Os03g11530	8.91372271

InteractorA	InteractorB	Z score
LOC_Os01g43390	LOC_Os06g39230	10.4695004
LOC_Os01g44130	LOC_Os01g71320	8.85935217
LOC_Os01g44260	LOC_Os01g46070	10.2000509
LOC_Os01g43090	LOC_Os09g10200	8.82405807
LOC_Os01g44220	LOC_Os03g51740	9.0893118
LOC_Os01g44220	LOC_Os05g11730	9.34076557
LOC_Os01g43390	LOC_Os09g36830	8.83935158
LOC_Os01g44130	LOC_Os02g55370	10.1328089
LOC_Os01g43390	LOC_Os04g57400	8.86342902
LOC_Os01g43390	LOC_Os12g10570	9.35803281
LOC_Os01g44130	LOC_Os04g35240	9.6476813
LOC_Os01g44220	LOC_Os03g01170	9.11068926
LOC_Os01g43390	LOC_Os01g73200	9.17529639
LOC_Os01g44130	LOC_Os10g38150	9.85711928
LOC_Os01g44220	LOC_Os03g58980	10.3499833
LOC_Os01g43530	LOC_Os02g12580	10.6025903
LOC_Os01g44130	LOC_Os07g36500	9.80972127
LOC_Os01g44220	LOC_Os07g34520	8.93680957
LOC_Os01g43390	LOC_Os10g25674	9.42950071
LOC_Os01g43390	LOC_Os04g44870	8.91023128
LOC_Os01g44260	LOC_Os03g10120	8.88760489
LOC_Os01g44220	LOC_Os01g71400	8.8402975
LOC_Os01g43530	LOC_Os10g40720	9.22347709
LOC_Os01g43390	LOC_Os05g49890	9.13385461
LOC_Os01g43090	LOC_Os08g32850	8.81575375
LOC_Os01g44260	LOC_Os05g28180	8.95821498
LOC_Os01g44130	LOC_Os01g55030	10.2397753
LOC_Os01g44220	LOC_Os03g39710	10.5332742
LOC_Os01g44260	LOC_Os04g32710	8.89149532
LOC_Os01g43530	LOC_Os07g26900	9.92181701
LOC_Os01g44130	LOC_Os04g55290	10.1613793
LOC_Os01g43530	LOC_Os04g47170	8.98063774
LOC_Os01g44220	LOC_Os01g50760	9.44119695
LOC_Os01g43090	LOC_Os08g33820	8.99404353
LOC_Os01g43530	LOC_Os07g30990	9.71203587
LOC_Os01g44220	LOC_Os05g46580	9.28670424
LOC_Os01g44260	LOC_Os05g04520	9.01251994
LOC_Os01g43390	LOC_Os01g52490	8.91347974
LOC_Os01g44220	LOC_Os01g62880	9.72068431
LOC_Os01g43530	LOC_Os08g38900	9.26111659
LOC_Os01g43390	LOC_Os02g41650	9.33696289
LOC_Os01g44130	LOC_Os03g55800	9.37907607
LOC_Os01g44130	LOC_Os03g01290	9.25466298

InteractorA	InteractorB	Z score
LOC_Os01g43390	LOC_Os02g22130	9.54187123
LOC_Os01g44260	LOC_Os03g52840	8.96553748
LOC_Os01g44220	LOC_Os07g10590	11.4015176
LOC_Os01g43390	LOC_Os03g61600	9.91682161
LOC_Os01g44130	LOC_Os03g02780	9.80972984
LOC_Os01g44130	LOC_Os02g08490	9.64616738
LOC_Os01g43530	LOC_Os05g51480	9.20332032
LOC_Os01g44260	LOC_Os04g01980	8.95280647
LOC_Os01g44260	LOC_Os07g38860	9.36048292
LOC_Os01g44130	LOC_Os10g39410	9.80972984
LOC_Os01g43390	LOC_Os07g34520	10.7664523
LOC_Os01g43390	LOC_Os06g15420	8.81650369
LOC_Os01g43390	LOC_Os03g04060	10.17359
LOC_Os01g44220	LOC_Os01g73980	8.93743136
LOC_Os01g43390	LOC_Os07g05820	9.16505186
LOC_Os01g43390	LOC_Os05g11550	10.9594561
LOC_Os01g44130	LOC_Os04g38870	9.14132505
LOC_Os01g43390	LOC_Os05g04500	9.72227137
LOC_Os01g44260	LOC_Os06g51084	9.32333401
LOC_Os01g44260	LOC_Os04g32650	9.40144575
LOC_Os01g43530	LOC_Os01g71380	9.2412747
LOC_Os01g43530	LOC_Os03g12290	9.56416373
LOC_Os01g43390	LOC_Os10g38350	8.89100184
LOC_Os01g43390	LOC_Os08g41830	10.3599187
LOC_Os01g44130	LOC_Os04g49420	9.80972127
LOC_Os01g44260	LOC_Os02g51830	8.96627665
LOC_Os01g43090	LOC_Os09g24990	8.87877575
LOC_Os01g44260	LOC_Os03g48471	10.8680738
LOC_Os01g44260	LOC_Os03g55090	9.39636665
LOC_Os01g44130	LOC_Os05g38740	9.80971577
LOC_Os01g44130	LOC_Os07g22930	8.97190068
LOC_Os01g44260	LOC_Os08g40170	9.06421703
LOC_Os01g44220	LOC_Os10g38234	8.83701127
LOC_Os01g43530	LOC_Os02g04950	10.6112254
LOC_Os01g43390	LOC_Os07g47420	10.0162079
LOC_Os01g44260	LOC_Os03g16030	9.80052722
LOC_Os01g44220	LOC_Os03g03720	10.0988935
LOC_Os01g44260	LOC_Os03g50480	8.884805
LOC_Os01g44130	LOC_Os04g12970	9.37112612
LOC_Os01g44130	LOC_Os04g25990	9.18131339
LOC_Os01g44260	LOC_Os04g52100	9.2319713
LOC_Os01g43390	LOC_Os02g41630	8.94080926
LOC_Os01g44130	LOC_Os03g55874	10.344672

InteractorA	InteractorB	Z score
LOC_Os01g44130	LOC_Os01g61920	9.80971577
LOC_Os01g44980	LOC_Os05g38560	9.56174199
LOC_Os01g45420	LOC_Os11g48110	9.98670973
LOC_Os01g45420	LOC_Os03g58050	10.9064277
LOC_Os01g45420	LOC_Os07g35940	8.83678943
LOC_Os01g44980	LOC_Os06g39875	9.40819664
LOC_Os01g46240	LOC_Os06g08310	8.89185753
LOC_Os01g44980	LOC_Os06g40180	9.04550098
LOC_Os01g45400	LOC_Os02g17390	8.89625144
LOC_Os01g46070	LOC_Os09g23530	9.00455135
LOC_Os01g44260	LOC_Os12g10600	9.7421555
LOC_Os01g46070	LOC_Os10g08580	9.23976702
LOC_Os01g46070	LOC_Os06g27770	9.1207818
LOC_Os01g44980	LOC_Os03g41460	10.0445777
LOC_Os01g46240	LOC_Os04g40310	8.80374835
LOC_Os01g46240	LOC_Os03g21900	9.36936144
LOC_Os01g46070	LOC_Os03g64330	9.69153018
LOC_Os01g45400	LOC_Os07g49220	8.87776934
LOC_Os01g45420	LOC_Os09g24530	9.19075135
LOC_Os01g46240	LOC_Os03g04970	9.38724787
LOC_Os01g44980	LOC_Os07g39290	9.18328884
LOC_Os01g45420	LOC_Os12g14070	10.5620942
LOC_Os01g45400	LOC_Os10g39880	10.9956431
LOC_Os01g44260	LOC_Os12g02980	9.56641409
LOC_Os01g45420	LOC_Os04g56210	9.73640438
LOC_Os01g44980	LOC_Os10g37210	9.55461641
LOC_Os01g45420	LOC_Os01g74000	10.3332875
LOC_Os01g46240	LOC_Os07g35940	10.1373011
LOC_Os01g45420	LOC_Os04g37820	9.55824802
LOC_Os01g46070	LOC_Os04g56730	9.20335585
LOC_Os01g46070	LOC_Os05g45810	8.94480653
LOC_Os01g46240	LOC_Os03g52860	8.9333665
LOC_Os01g44260	LOC_Os09g28420	9.42687914
LOC_Os01g45420	LOC_Os12g07980	9.93886048
LOC_Os01g46240	LOC_Os08g34790	9.26176569
LOC_Os01g46070	LOC_Os04g43800	9.02077079
LOC_Os01g46240	LOC_Os01g62230	9.23439983
LOC_Os01g45420	LOC_Os06g44620	9.35629848
LOC_Os01g45420	LOC_Os05g26890	8.97569804
LOC_Os01g46070	LOC_Os05g12210	8.89541636
LOC_Os01g44260	LOC_Os10g02070	10.4381951
LOC_Os01g44260	LOC_Os12g43370	10.9420593
LOC_Os01g46240	LOC_Os08g32620	9.2012895

InteractorA	InteractorB	Z score
LOC_Os01g46070	LOC_Os04g12900	10.3573738
LOC_Os01g44980	LOC_Os04g56646	11.0046045
LOC_Os01g45400	LOC_Os05g37690	9.22694992
LOC_Os01g44980	LOC_Os12g31370	8.95520543
LOC_Os01g46240	LOC_Os09g25390	9.81404134
LOC_Os01g46070	LOC_Os03g08530	8.86658591
LOC_Os01g45420	LOC_Os02g52800	9.20855345
LOC_Os01g44980	LOC_Os07g44440	9.04901415
LOC_Os01g44980	LOC_Os04g48850	9.67346478
LOC_Os01g45420	LOC_Os01g64110	9.52371973
LOC_Os01g45400	LOC_Os11g32650	8.94900381
LOC_Os01g45420	LOC_Os10g38189	8.84768249
LOC_Os01g45420	LOC_Os05g28180	8.83230935
LOC_Os01g46240	LOC_Os09g31486	10.47151
LOC_Os01g45400	LOC_Os05g42350	9.14912705
LOC_Os01g46070	LOC_Os07g17010	8.8040178
LOC_Os01g46070	LOC_Os08g23730	9.00204996
LOC_Os01g46240	LOC_Os03g49260	9.10476026
LOC_Os01g44980	LOC_Os08g02410	8.97694192
LOC_Os01g46070	LOC_Os09g31486	9.01226113
LOC_Os01g46070	LOC_Os02g32490	9.54894694
LOC_Os01g45420	LOC_Os04g12720	11.0034799
LOC_Os01g44980	LOC_Os08g44340	8.85834813
LOC_Os01g44260	LOC_Os11g47760	8.82236811
LOC_Os01g45400	LOC_Os05g35320	8.94292781
LOC_Os01g45420	LOC_Os03g55090	8.93826736
LOC_Os01g46070	LOC_Os02g14460	9.08758488
LOC_Os01g44980	LOC_Os07g26540	9.01440257
LOC_Os01g44980	LOC_Os03g18740	8.93935232
LOC_Os01g46070	LOC_Os12g37960	9.71939523
LOC_Os01g46070	LOC_Os04g42250	8.85329922
LOC_Os01g46070	LOC_Os06g08600	8.92667648
LOC_Os01g46070	LOC_Os09g08072	9.2015515
LOC_Os01g46240	LOC_Os03g01530	8.82240799
LOC_Os01g45420	LOC_Os07g07709	10.906422
LOC_Os01g46240	LOC_Os07g30970	8.84657636
LOC_Os01g46240	LOC_Os02g58730	8.93231085
LOC_Os01g46240	LOC_Os06g10910	9.01119723
LOC_Os01g44980	LOC_Os02g14059	9.6665962
LOC_Os01g46070	LOC_Os12g12560	10.047527
LOC_Os01g46240	LOC_Os02g21460	8.98721234
LOC_Os01g46240	LOC_Os01g55940	9.21981472
LOC_Os01g45420	LOC_Os08g34170	8.89596321

InteractorA	InteractorB	Z score
LOC_Os01g44980	LOC_Os03g12660	9.27863679
LOC_Os01g44980	LOC_Os02g53420	9.05411347
LOC_Os01g45400	LOC_Os04g53230	9.2765982
LOC_Os01g45420	LOC_Os09g34960	9.30336118
LOC_Os01g45400	LOC_Os12g21798	9.50736063
LOC_Os01g46070	LOC_Os02g01160	9.28868659
LOC_Os01g45420	LOC_Os01g59600	9.84043348
LOC_Os01g45400	LOC_Os11g47550	9.36789376
LOC_Os01g46070	LOC_Os06g46000	9.45289456
LOC_Os01g45400	LOC_Os05g23740	9.25365136
LOC_Os01g46070	LOC_Os08g02700	8.9847447
LOC_Os01g44980	LOC_Os08g01660	8.94832221
LOC_Os01g46070	LOC_Os04g46620	9.23482864
LOC_Os01g46240	LOC_Os08g44350	11.638159
LOC_Os01g45400	LOC_Os03g22060	9.05992268
LOC_Os01g45420	LOC_Os03g02710	9.52813076
LOC_Os01g46070	LOC_Os05g49840	9.07139719
LOC_Os01g46240	LOC_Os08g28820	9.29183314
LOC_Os01g46070	LOC_Os03g06940	10.643659
LOC_Os01g45400	LOC_Os04g44870	9.05109382
LOC_Os01g44260	LOC_Os09g20090	9.26674897
LOC_Os01g46070	LOC_Os05g28940	8.88992396
LOC_Os01g46070	LOC_Os05g36290	9.1038055
LOC_Os01g44260	LOC_Os09g32952	8.88546736
LOC_Os01g44260	LOC_Os08g44530	9.24233344
LOC_Os01g46070	LOC_Os02g47020	9.73121515
LOC_Os01g45420	LOC_Os04g56400	9.07090392
LOC_Os01g46240	LOC_Os09g27750	8.91407928
LOC_Os01g46240	LOC_Os04g54330	9.10546661
LOC_Os01g46570	LOC_Os03g07300	9.13514188
LOC_Os01g46290	LOC_Os02g52430	9.03309689
LOC_Os01g46610	LOC_Os02g47790	9.01749403
LOC_Os01g46570	LOC_Os04g24520	8.83276338
LOC_Os01g46610	LOC_Os04g16846	9.06043981
LOC_Os01g46380	LOC_Os10g28320	9.95624453
LOC_Os01g46380	LOC_Os06g04030	9.33197866
LOC_Os01g46240	LOC_Os11g25100	9.79809814
LOC_Os01g46380	LOC_Os07g25590	9.05579717
LOC_Os01g46380	LOC_Os06g37500	9.26093824
LOC_Os01g46570	LOC_Os02g38200	9.88033416
LOC_Os01g46240	LOC_Os12g10560	10.164528
LOC_Os01g46290	LOC_Os12g22680	9.2097169
LOC_Os01g46926	LOC_Os02g17390	8.9833437

InteractorA	InteractorB	Z score
LOC_Os01g46380	LOC_Os12g04980	9.0089712
LOC_Os01g46290	LOC_Os01g73580	9.76530719
LOC_Os01g46570	LOC_Os01g50050	9.51636272
LOC_Os01g46380	LOC_Os01g64640	9.47499616
LOC_Os01g46610	LOC_Os04g19740	9.52930163
LOC_Os01g46290	LOC_Os02g08490	9.2095164
LOC_Os01g46290	LOC_Os02g35310	9.14628167
LOC_Os01g46380	LOC_Os04g33470	8.84505132
LOC_Os01g46610	LOC_Os02g01280	9.29604623
LOC_Os01g46610	LOC_Os10g39120	8.88039297
LOC_Os01g46610	LOC_Os03g12660	9.54155784
LOC_Os01g46380	LOC_Os04g40290	8.9888839
LOC_Os01g46610	LOC_Os01g57942	9.06042032
LOC_Os01g46380	LOC_Os03g44484	8.85164375
LOC_Os01g46610	LOC_Os10g38740	9.0978975
LOC_Os01g46290	LOC_Os05g45590	8.80810191
LOC_Os01g46380	LOC_Os05g46290	9.6166591
LOC_Os01g46610	LOC_Os07g03730	8.86159239
LOC_Os01g46610	LOC_Os07g03368	8.90096764
LOC_Os01g46610	LOC_Os02g46130	10.109551
LOC_Os01g46380	LOC_Os05g36280	9.47500965
LOC_Os01g46926	LOC_Os10g38140	10.6210957
LOC_Os01g46290	LOC_Os11g08445	9.85248002
LOC_Os01g46926	LOC_Os07g38030	9.65872779
LOC_Os01g46610	LOC_Os04g35570	8.90770901
LOC_Os01g46570	LOC_Os04g16740	9.22938094
LOC_Os01g46380	LOC_Os03g27310	9.33197866
LOC_Os01g46570	LOC_Os03g61280	9.51243862
LOC_Os01g46570	LOC_Os11g10520	8.91942244
LOC_Os01g46570	LOC_Os02g38210	9.06799782
LOC_Os01g46290	LOC_Os03g06940	9.02542736
LOC_Os01g46610	LOC_Os07g46280	9.86112681
LOC_Os01g46570	LOC_Os09g26880	9.38040861
LOC_Os01g46926	LOC_Os05g38560	10.3881705
LOC_Os01g46610	LOC_Os08g03440	8.89858539
LOC_Os01g46610	LOC_Os05g49760	12.8242247
LOC_Os01g46610	LOC_Os03g56460	9.1878189
LOC_Os01g46240	LOC_Os10g21266	9.4138758
LOC_Os01g46570	LOC_Os04g58640	9.39664061
LOC_Os01g46380	LOC_Os06g43640	8.97278031
LOC_Os01g46380	LOC_Os01g57942	8.89908959
LOC_Os01g46290	LOC_Os10g25130	9.056383
LOC_Os01g46380	LOC_Os02g01510	9.34712689

InteractorA	InteractorB	Z score
LOC_Os01g46380	LOC_Os04g16846	8.89909882
LOC_Os01g46570	LOC_Os12g17910	8.88845526
LOC_Os01g46380	LOC_Os03g17690	8.91237883
LOC_Os01g46570	LOC_Os03g55800	9.22693752
LOC_Os01g46570	LOC_Os03g56840	9.03014016
LOC_Os01g46570	LOC_Os07g47990	9.01838269
LOC_Os01g46380	LOC_Os06g15990	9.52561231
LOC_Os01g46610	LOC_Os08g37800	9.4840839
LOC_Os01g46380	LOC_Os11g05730	9.47500965
LOC_Os01g46610	LOC_Os04g58580	9.46044656
LOC_Os01g46610	LOC_Os12g13800	9.13095813
LOC_Os01g46380	LOC_Os02g41470	9.31355075
LOC_Os01g46380	LOC_Os10g21324	8.89909013
LOC_Os01g46926	LOC_Os07g38860	9.32269004
LOC_Os01g46926	LOC_Os03g02260	9.09847098
LOC_Os01g46570	LOC_Os04g36700	8.84672202
LOC_Os01g46570	LOC_Os03g58130	8.96564774
LOC_Os01g46290	LOC_Os12g34380	9.20476051
LOC_Os01g46380	LOC_Os06g06510	9.47499616
LOC_Os01g46380	LOC_Os05g49800	11.5726921
LOC_Os01g46570	LOC_Os11g08940	8.87742459
LOC_Os01g46240	LOC_Os10g28080	9.20176417
LOC_Os01g46290	LOC_Os03g07150	9.3785785
LOC_Os01g46610	LOC_Os04g42920	9.29767176
LOC_Os01g46610	LOC_Os10g37060	9.14664976
LOC_Os01g46610	LOC_Os10g21324	9.06042032
LOC_Os01g46570	LOC_Os10g20910	8.99551021
LOC_Os01g46240	LOC_Os12g13380	8.92448377
LOC_Os01g46290	LOC_Os04g33720	9.96366248
LOC_Os01g46926	LOC_Os04g41310	8.87869817
LOC_Os01g46290	LOC_Os05g08430	9.13498391
LOC_Os01g46926	LOC_Os07g12730	8.81611173
LOC_Os01g46610	LOC_Os05g45420	9.47806846
LOC_Os01g46610	LOC_Os04g36700	8.98414838
LOC_Os01g46380	LOC_Os05g47980	9.20052825
LOC_Os01g46380	LOC_Os04g38600	9.54505214
LOC_Os01g46290	LOC_Os01g52470	9.28788513
LOC_Os01g46610	LOC_Os07g42940	8.90651094
LOC_Os01g46290	LOC_Os05g40990	8.87529227
LOC_Os01g46570	LOC_Os03g20710	9.29153323
LOC_Os01g46570	LOC_Os03g53650	8.80544372
LOC_Os01g46240	LOC_Os10g02070	9.08203649
LOC_Os01g46610	LOC_Os01g55950	8.84760377

InteractorA	InteractorB	Z score
LOC_Os01g46610	LOC_Os01g61400	10.2510052
LOC_Os01g46570	LOC_Os03g13150	8.91499738
LOC_Os01g46610	LOC_Os02g12730	9.05023701
LOC_Os01g46610	LOC_Os05g11730	9.95252506
LOC_Os01g46570	LOC_Os08g33820	9.53314858
LOC_Os01g46926	LOC_Os04g40130	10.8772017
LOC_Os01g46570	LOC_Os01g73170	9.85178536
LOC_Os01g46380	LOC_Os06g06460	9.47499616
LOC_Os01g46610	LOC_Os08g35740	11.989828
LOC_Os01g46380	LOC_Os04g53810	8.95226336
LOC_Os01g46570	LOC_Os02g37420	9.10632231
LOC_Os01g46290	LOC_Os05g48290	8.90661816
LOC_Os01g46570	LOC_Os10g42720	9.44134218
LOC_Os01g46610	LOC_Os12g13390	10.0577212
LOC_Os01g47400	LOC_Os03g16880	9.64050573
LOC_Os01g47400	LOC_Os10g25130	9.06269075
LOC_Os01g47400	LOC_Os10g38234	8.93229941
LOC_Os01g47410	LOC_Os07g07550	9.0170283
LOC_Os01g47400	LOC_Os03g46060	9.12706509
LOC_Os01g47400	LOC_Os03g53800	9.43972841
LOC_Os01g47400	LOC_Os04g01674	9.04380879
LOC_Os01g47400	LOC_Os04g53810	8.89553461
LOC_Os01g47410	LOC_Os04g28180	8.86524352
LOC_Os01g47070	LOC_Os05g42150	8.85202245
LOC_Os01g47550	LOC_Os06g15420	9.4411195
LOC_Os01g48280	LOC_Os08g06060	10.6421847
LOC_Os01g48280	LOC_Os10g38690	9.0184223
LOC_Os01g48280	LOC_Os08g31060	10.1311672
LOC_Os01g48280	LOC_Os01g73580	9.07658567
LOC_Os01g47400	LOC_Os04g56070	9.6975332
LOC_Os01g47400	LOC_Os10g38229	9.97531971
LOC_Os01g47070	LOC_Os08g02410	8.95715663
LOC_Os01g47410	LOC_Os04g32010	9.88330741
LOC_Os01g47400	LOC_Os06g07080	9.58770932
LOC_Os01g48280	LOC_Os03g23970	8.87560525
LOC_Os01g47410	LOC_Os02g05410	9.34781517
LOC_Os01g47400	LOC_Os04g37990	10.7741315
LOC_Os01g47550	LOC_Os02g53790	9.5109944
LOC_Os01g48280	LOC_Os03g22010	8.80726075
LOC_Os01g47400	LOC_Os03g27310	8.84268057
LOC_Os01g47550	LOC_Os03g45270	9.18888151
LOC_Os01g47400	LOC_Os09g28460	9.66796771
LOC_Os01g47400	LOC_Os02g57854	10.215477

InteractorA	InteractorB	Z score
LOC_Os01g47550	LOC_Os11g08340	9.7588602
LOC_Os01g47400	LOC_Os12g06620	8.91444022
LOC_Os01g47410	LOC_Os03g29570	9.75996742
LOC_Os01g47550	LOC_Os10g21310	9.23312459
LOC_Os01g47400	LOC_Os05g05830	12.2979701
LOC_Os01g47400	LOC_Os05g49760	9.68775854
LOC_Os01g47400	LOC_Os07g28480	10.4306931
LOC_Os01g48280	LOC_Os04g57090	9.86031676
LOC_Os01g47070	LOC_Os01g71820	9.01617162
LOC_Os01g47400	LOC_Os07g39290	10.454139
LOC_Os01g48280	LOC_Os08g37800	9.18102942
LOC_Os01g47070	LOC_Os03g40330	9.1840376
LOC_Os01g47400	LOC_Os03g10370	10.552773
LOC_Os01g47410	LOC_Os04g35240	8.92930053
LOC_Os01g47400	LOC_Os06g46000	9.12275176
LOC_Os01g47400	LOC_Os05g33400	9.49535065
LOC_Os01g47410	LOC_Os03g49610	9.13506442
LOC_Os01g48280	LOC_Os04g32010	8.97535894
LOC_Os01g47070	LOC_Os06g05110	8.98977325
LOC_Os01g47550	LOC_Os02g14460	9.32286011
LOC_Os01g47400	LOC_Os01g58380	8.97846472
LOC_Os01g47070	LOC_Os09g28460	9.19798739
LOC_Os01g47550	LOC_Os05g23860	9.0813344
LOC_Os01g47070	LOC_Os05g42350	9.81719056
LOC_Os01g47070	LOC_Os03g31290	8.8999355
LOC_Os01g47400	LOC_Os07g17010	10.6252363
LOC_Os01g47400	LOC_Os11g03980	9.89525213
LOC_Os01g47550	LOC_Os03g37950	11.0177863
LOC_Os01g47400	LOC_Os02g01880	8.94218191
LOC_Os01g47400	LOC_Os03g60870	8.86459293
LOC_Os01g47400	LOC_Os12g42280	8.83728176
LOC_Os01g48280	LOC_Os02g50240	10.0036996
LOC_Os01g48280	LOC_Os02g57770	9.04688045
LOC_Os01g47400	LOC_Os11g26850	9.40017801
LOC_Os01g47070	LOC_Os07g05940	8.8041673
LOC_Os01g47410	LOC_Os02g51830	9.23067613
LOC_Os01g47550	LOC_Os04g57220	9.41621725
LOC_Os01g47550	LOC_Os01g49320	9.41470124
LOC_Os01g47400	LOC_Os08g29520	8.8319385
LOC_Os01g47070	LOC_Os05g38310	9.02937843
LOC_Os01g47400	LOC_Os02g52640	8.84588234
LOC_Os01g47400	LOC_Os09g24990	11.1920132
LOC_Os01g47070	LOC_Os05g46860	10.5942289

InteractorA	InteractorB	Z score
LOC_Os01g47070	LOC_Os07g08880	10.5260421
LOC_Os01g47400	LOC_Os08g41880	9.13184234
LOC_Os01g47400	LOC_Os06g06560	9.55053376
LOC_Os01g47410	LOC_Os01g60190	8.83713987
LOC_Os01g47410	LOC_Os02g27940	9.15955488
LOC_Os01g48280	LOC_Os09g28420	9.64869144
LOC_Os01g47400	LOC_Os02g07490	9.34559961
LOC_Os01g47550	LOC_Os09g36830	9.75654614
LOC_Os01g47070	LOC_Os03g56280	9.00020561
LOC_Os01g48280	LOC_Os02g56680	8.89728037
LOC_Os01g47550	LOC_Os12g13390	8.87621079
LOC_Os01g47400	LOC_Os07g09680	8.87798172
LOC_Os01g47410	LOC_Os07g05160	9.18253286
LOC_Os01g48280	LOC_Os04g32710	10.6001815
LOC_Os01g47410	LOC_Os03g51600	8.89690546
LOC_Os01g47410	LOC_Os11g08470	8.85441421
LOC_Os01g47070	LOC_Os08g23730	9.19242591
LOC_Os01g47410	LOC_Os06g47600	10.803875
LOC_Os01g47400	LOC_Os08g34280	10.7950839
LOC_Os01g47410	LOC_Os02g56130	9.51131472
LOC_Os01g48280	LOC_Os07g05820	8.96342211
LOC_Os01g47400	LOC_Os06g04030	8.84268057
LOC_Os01g47410	LOC_Os02g52800	9.51989911
LOC_Os01g48280	LOC_Os06g37080	8.82541044
LOC_Os01g47410	LOC_Os03g04169	8.94561089
LOC_Os01g47550	LOC_Os07g07320	8.92377754
LOC_Os01g48280	LOC_Os11g37550	9.68976617
LOC_Os01g47400	LOC_Os03g28330	8.83069706
LOC_Os01g47400	LOC_Os04g01600	9.49950366
LOC_Os01g47550	LOC_Os01g73170	9.07447696
LOC_Os01g47070	LOC_Os02g18880	9.04096672
LOC_Os01g47400	LOC_Os02g09150	10.1778305
LOC_Os01g48280	LOC_Os06g04280	8.9911614
LOC_Os01g47400	LOC_Os03g61340	9.09902801
LOC_Os01g47400	LOC_Os06g35700	9.5292927
LOC_Os01g47550	LOC_Os06g11290	9.65803238
LOC_Os01g47410	LOC_Os07g38890	9.21059694
LOC_Os01g48280	LOC_Os09g39810	10.2246491
LOC_Os01g47400	LOC_Os04g46460	10.3947453
LOC_Os01g47400	LOC_Os10g23100	9.51528972
LOC_Os01g48280	LOC_Os12g12470	9.29460468
LOC_Os01g47400	LOC_Os05g51050	8.94450789
LOC_Os01g47410	LOC_Os02g17780	9.13793011

InteractorA	InteractorB	Z score
LOC_Os01g47400	LOC_Os09g39400	8.83910681
LOC_Os01g47550	LOC_Os08g28820	9.32729701
LOC_Os01g47400	LOC_Os07g48040	13.539589
LOC_Os01g47550	LOC_Os06g37660	9.01694259
LOC_Os01g47550	LOC_Os03g52840	9.60065286
LOC_Os01g47400	LOC_Os04g10000	9.53119087
LOC_Os01g47400	LOC_Os02g57450	8.88834056
LOC_Os01g47550	LOC_Os06g40940	9.1176624
LOC_Os01g47550	LOC_Os04g12900	9.96606863
LOC_Os01g47400	LOC_Os05g46290	9.41136908
LOC_Os01g47550	LOC_Os12g13810	9.63688118
LOC_Os01g47070	LOC_Os03g13160	10.1548855
LOC_Os01g47550	LOC_Os02g42320	11.2931124
LOC_Os01g47400	LOC_Os03g01290	9.57443923
LOC_Os01g47070	LOC_Os05g20050	9.05217458
LOC_Os01g47070	LOC_Os03g13150	10.0590001
LOC_Os01g47410	LOC_Os06g27770	9.12807921
LOC_Os01g47400	LOC_Os01g48420	10.2025618
LOC_Os01g48280	LOC_Os09g28400	11.0834924
LOC_Os01g48280	LOC_Os11g31620	9.44261994
LOC_Os01g47550	LOC_Os10g39840	9.52613716
LOC_Os01g47410	LOC_Os09g10270	10.471783
LOC_Os01g46926	LOC_Os12g40550	8.83802124
LOC_Os01g48600	LOC_Os02g17390	10.6559834
LOC_Os01g48600	LOC_Os07g20544	10.6312184
LOC_Os01g48680	LOC_Os08g41880	9.22952646
LOC_Os01g48760	LOC_Os08g39140	9.30194899
LOC_Os01g48600	LOC_Os04g35240	9.75194103
LOC_Os01g48760	LOC_Os12g23630	8.88249531
LOC_Os01g48760	LOC_Os01g56380	9.5640347
LOC_Os01g48600	LOC_Os03g10940	9.69463382
LOC_Os01g49120	LOC_Os12g16240	9.4268957
LOC_Os01g49120	LOC_Os01g59530	9.50542612
LOC_Os01g48680	LOC_Os09g37100	8.83119512
LOC_Os01g48600	LOC_Os03g16900	9.42687805
LOC_Os01g49120	LOC_Os08g41830	9.11896058
LOC_Os01g49120	LOC_Os03g21260	9.20999446
LOC_Os01g48600	LOC_Os07g35880	9.23288746
LOC_Os01g48760	LOC_Os01g71680	9.10553443
LOC_Os01g48680	LOC_Os02g56690	8.85502544
LOC_Os01g48680	LOC_Os06g07140	10.1462638
LOC_Os01g48760	LOC_Os08g40170	8.84866387
LOC_Os01g48420	LOC_Os01g70300	8.84346358

InteractorA	InteractorB	Z score
LOC_Os01g48760	LOC_Os02g01340	9.72375034
LOC_Os01g49120	LOC_Os10g02480	9.37422834
LOC_Os01g48680	LOC_Os05g34540	9.49182676
LOC_Os01g49120	LOC_Os08g28820	10.8423326
LOC_Os01g48600	LOC_Os03g55600	9.45262295
LOC_Os01g48760	LOC_Os05g45420	10.2469502
LOC_Os01g49190	LOC_Os01g61400	9.34092174
LOC_Os01g48600	LOC_Os04g16680	9.95592047
LOC_Os01g48680	LOC_Os05g46040	11.5776377
LOC_Os01g48760	LOC_Os10g08710	10.4806938
LOC_Os01g48760	LOC_Os10g21240	8.96081441
LOC_Os01g48600	LOC_Os08g25734	10.1429471
LOC_Os01g48680	LOC_Os02g50880	9.22630693
LOC_Os01g48680	LOC_Os05g12190	9.94266611
LOC_Os01g48760	LOC_Os02g51100	9.34445255
LOC_Os01g48680	LOC_Os02g50240	8.86060498
LOC_Os01g48680	LOC_Os08g06060	9.10939598
LOC_Os01g48760	LOC_Os11g25700	9.27673894
LOC_Os01g48760	LOC_Os03g62060	9.11969715
LOC_Os01g48420	LOC_Os01g49690	8.911477
LOC_Os01g48600	LOC_Os06g41810	8.94774534
LOC_Os01g48600	LOC_Os09g04050	9.58447542
LOC_Os01g49190	LOC_Os01g52470	9.27815096
LOC_Os01g48680	LOC_Os05g44180	9.55903862
LOC_Os01g49120	LOC_Os01g50760	9.62163485
LOC_Os01g49120	LOC_Os01g60190	9.67733028
LOC_Os01g49120	LOC_Os07g42600	8.82869846
LOC_Os01g48680	LOC_Os09g37949	9.862507
LOC_Os01g48420	LOC_Os05g30480	8.83540494
LOC_Os01g49120	LOC_Os05g35320	8.90685391
LOC_Os01g48760	LOC_Os04g37480	8.95930714
LOC_Os01g48680	LOC_Os07g43250	9.01810023
LOC_Os01g48600	LOC_Os04g40130	9.23109898
LOC_Os01g48680	LOC_Os07g07550	8.85568601
LOC_Os01g49190	LOC_Os01g52450	8.92967199
LOC_Os01g48680	LOC_Os05g04520	9.00191943
LOC_Os01g48600	LOC_Os05g11550	9.42003621
LOC_Os01g49120	LOC_Os10g28050	9.79749427
LOC_Os01g48680	LOC_Os07g42940	10.5721367
LOC_Os01g48600	LOC_Os02g38840	9.09410834
LOC_Os01g48420	LOC_Os12g22680	9.34792994
LOC_Os01g48600	LOC_Os05g29880	9.11752969
LOC_Os01g48420	LOC_Os04g36800	9.1461727

InteractorA	InteractorB	Z score
LOC_Os01g48760	LOC_Os01g64850	9.76691096
LOC_Os01g49120	LOC_Os01g71320	8.88298658
LOC_Os01g48600	LOC_Os11g32650	8.90696579
LOC_Os01g48760	LOC_Os01g50760	8.95726608
LOC_Os01g48600	LOC_Os07g14590	9.22093148
LOC_Os01g49120	LOC_Os03g18570	8.83999631
LOC_Os01g49320	LOC_Os07g05180	9.13889581
LOC_Os01g49320	LOC_Os08g10510	8.9770987
LOC_Os01g49290	LOC_Os04g16680	8.98274173
LOC_Os01g49720	LOC_Os02g07260	8.96269424
LOC_Os01g49320	LOC_Os12g04980	8.94795468
LOC_Os01g49320	LOC_Os05g51050	9.08042406
LOC_Os01g49690	LOC_Os07g08170	9.9195199
LOC_Os01g49190	LOC_Os04g47360	8.92684387
LOC_Os01g49710	LOC_Os10g38140	9.74529822
LOC_Os01g49320	LOC_Os08g33710	9.09344026
LOC_Os01g49710	LOC_Os03g58290	10.4497649
LOC_Os01g49710	LOC_Os01g71350	8.83720603
LOC_Os01g49690	LOC_Os02g52640	8.80524851
LOC_Os01g49710	LOC_Os02g08420	9.21000167
LOC_Os01g49290	LOC_Os07g13980	8.93096299
LOC_Os01g49710	LOC_Os10g23900	9.90318782
LOC_Os01g49320	LOC_Os03g18570	8.81231465
LOC_Os01g49190	LOC_Os12g43450	9.18958532
LOC_Os01g49690	LOC_Os04g56760	10.7074395
LOC_Os01g49690	LOC_Os05g48290	9.07226956
LOC_Os01g49190	LOC_Os10g28320	9.10679389
LOC_Os01g49720	LOC_Os01g59600	9.76062381
LOC_Os01g49710	LOC_Os03g58050	9.1153023
LOC_Os01g49710	LOC_Os07g07709	9.11530528
LOC_Os01g49320	LOC_Os03g60620	9.51586672
LOC_Os01g49320	LOC_Os11g33240	8.93830256
LOC_Os01g49290	LOC_Os08g39860	8.914015
LOC_Os01g49190	LOC_Os10g38360	8.85339858
LOC_Os01g49690	LOC_Os09g39810	9.02372375
LOC_Os01g49720	LOC_Os02g38200	11.2137476
LOC_Os01g49690	LOC_Os06g37660	9.22329146
LOC_Os01g49190	LOC_Os03g06620	8.84714093
LOC_Os01g49690	LOC_Os10g26600	8.90925877
LOC_Os01g49690	LOC_Os11g25330	9.24935998
LOC_Os01g49190	LOC_Os07g37550	8.9010991
LOC_Os01g49690	LOC_Os02g56690	8.95206599
LOC_Os01g49690	LOC_Os10g07229	11.0146284

InteractorA	InteractorB	Z score
LOC_Os01g49190	LOC_Os06g07580	9.73246632
LOC_Os01g49320	LOC_Os04g32650	9.18195786
LOC_Os01g49290	LOC_Os03g26970	10.1361393
LOC_Os01g49190	LOC_Os03g25360	9.47206045
LOC_Os01g49190	LOC_Os04g02050	8.87042884
LOC_Os01g49290	LOC_Os05g43510	9.21258759
LOC_Os01g49320	LOC_Os07g34589	8.99975031
LOC_Os01g49710	LOC_Os03g27370	9.74285869
LOC_Os01g49710	LOC_Os02g08520	8.81038579
LOC_Os01g49190	LOC_Os03g53230	9.86045438
LOC_Os01g49190	LOC_Os12g02080	10.3307347
LOC_Os01g49320	LOC_Os02g02210	11.5540621
LOC_Os01g49690	LOC_Os09g04680	8.86769031
LOC_Os01g49690	LOC_Os02g52420	9.70652352
LOC_Os01g49320	LOC_Os08g04560	10.908667
LOC_Os01g49290	LOC_Os02g58480	8.93294226
LOC_Os01g49190	LOC_Os02g17390	9.72618896
LOC_Os01g49710	LOC_Os07g14590	8.95190256
LOC_Os01g49320	LOC_Os03g45410	9.75436324
LOC_Os01g49290	LOC_Os11g05880	8.80234434
LOC_Os01g49690	LOC_Os05g46550	8.97845389
LOC_Os01g49690	LOC_Os08g34280	8.99085167
LOC_Os01g49690	LOC_Os02g47610	9.43437426
LOC_Os01g49690	LOC_Os05g02310	9.73970209
LOC_Os01g49690	LOC_Os02g36400	8.81050185
LOC_Os01g49720	LOC_Os01g70300	9.45952154
LOC_Os01g49690	LOC_Os03g61340	10.4822127
LOC_Os01g49190	LOC_Os05g44050	9.38621233
LOC_Os01g49320	LOC_Os04g42920	9.24972712
LOC_Os01g49190	LOC_Os04g33480	8.9821167
LOC_Os01g49710	LOC_Os12g25690	9.77946561
LOC_Os01g49320	LOC_Os09g23540	12.137311
LOC_Os01g49690	LOC_Os12g43450	9.17896077
LOC_Os01g49690	LOC_Os07g42950	9.93905621
LOC_Os01g49290	LOC_Os12g03816	9.10607388
LOC_Os01g49190	LOC_Os06g45120	9.01587029
LOC_Os01g49690	LOC_Os03g13150	9.05492694
LOC_Os01g49190	LOC_Os04g57400	8.91055486
LOC_Os01g49320	LOC_Os05g30410	9.66114658
LOC_Os01g49190	LOC_Os08g44270	9.41626626
LOC_Os01g49320	LOC_Os07g39870	8.94911876
LOC_Os01g49190	LOC_Os02g56014	9.73246632
LOC_Os01g49690	LOC_Os03g22120	9.68754823

InteractorA	InteractorB	Z score
LOC_Os01g49290	LOC_Os10g21250	9.03555761
LOC_Os01g49690	LOC_Os11g48110	11.2873704
LOC_Os01g49320	LOC_Os03g18810	9.00856121
LOC_Os01g49190	LOC_Os07g44620	9.40032032
LOC_Os01g49290	LOC_Os06g35530	8.99248279
LOC_Os01g49320	LOC_Os04g56760	9.66114658
LOC_Os01g49190	LOC_Os10g32970	9.28974495
LOC_Os01g49690	LOC_Os12g36950	9.16522345
LOC_Os01g49710	LOC_Os03g30950	9.1239517
LOC_Os01g49690	LOC_Os03g50250	10.76036
LOC_Os01g49320	LOC_Os03g62700	8.83859814
LOC_Os01g49690	LOC_Os05g30410	10.7074395
LOC_Os01g49320	LOC_Os08g37790	11.9347959
LOC_Os01g49720	LOC_Os02g51100	9.0561454
LOC_Os01g49690	LOC_Os11g10520	11.4146027
LOC_Os01g49190	LOC_Os06g50300	8.95468389
LOC_Os01g49690	LOC_Os04g01470	9.71208118
LOC_Os01g49690	LOC_Os03g59060	8.91817989
LOC_Os01g49710	LOC_Os08g38900	9.94868802
LOC_Os01g49190	LOC_Os05g04690	9.37799334
LOC_Os01g49190	LOC_Os02g17780	9.00277489
LOC_Os01g49320	LOC_Os03g58130	8.89603617
LOC_Os01g49690	LOC_Os03g41438	8.9958324
LOC_Os01g49190	LOC_Os02g07490	9.86646684
LOC_Os01g49190	LOC_Os10g41490	9.18927209
LOC_Os01g49290	LOC_Os03g49350	9.20800321
LOC_Os01g49290	LOC_Os08g02340	9.94647993
LOC_Os01g49290	LOC_Os02g10310	10.2550675
LOC_Os01g49710	LOC_Os06g42130	10.149807
LOC_Os01g49290	LOC_Os02g14130	10.0195653
LOC_Os01g49710	LOC_Os04g36800	9.01468376
LOC_Os01g49320	LOC_Os07g44460	9.0941694
LOC_Os01g50050	LOC_Os07g08030	8.85495402
LOC_Os01g50050	LOC_Os01g50760	10.582742
LOC_Os01g50030	LOC_Os07g47420	9.51911735
LOC_Os01g49890	LOC_Os01g51060	10.0043396
LOC_Os01g50050	LOC_Os08g33370	8.87669095
LOC_Os01g50030	LOC_Os03g58430	9.09645246
LOC_Os01g50050	LOC_Os10g28080	9.41014034
LOC_Os01g49890	LOC_Os05g51670	8.86529357
LOC_Os01g50050	LOC_Os03g04250	9.01433856
LOC_Os01g49720	LOC_Os07g30170	11.5562173
LOC_Os01g50030	LOC_Os07g39290	9.27754806

InteractorA	InteractorB	Z score
LOC_Os01g50030	LOC_Os08g23110	8.94498317
LOC_Os01g50050	LOC_Os06g06560	9.99979295
LOC_Os01g50030	LOC_Os08g39300	9.40080556
LOC_Os01g49890	LOC_Os07g48430	9.10903754
LOC_Os01g50032	LOC_Os04g09540	9.34032765
LOC_Os01g50032	LOC_Os06g12090	9.56862606
LOC_Os01g50050	LOC_Os03g37950	8.92221875
LOC_Os01g50030	LOC_Os01g63270	8.95256032
LOC_Os01g50032	LOC_Os03g18580	9.99711203
LOC_Os01g50050	LOC_Os02g33140	9.39985055
LOC_Os01g50050	LOC_Os06g35590	9.89707286
LOC_Os01g50030	LOC_Os03g57130	9.36197325
LOC_Os01g50030	LOC_Os07g34140	8.98818614
LOC_Os01g50030	LOC_Os06g37610	9.14699165
LOC_Os01g49890	LOC_Os04g33740	9.7961017
LOC_Os01g49890	LOC_Os09g20090	10.0356413
LOC_Os01g49890	LOC_Os08g37800	10.2269861
LOC_Os01g50050	LOC_Os03g52840	9.466679
LOC_Os01g49720	LOC_Os03g12270	9.26619641
LOC_Os01g50050	LOC_Os11g08470	9.04313077
LOC_Os01g49890	LOC_Os04g22730	8.82990186
LOC_Os01g50050	LOC_Os04g56210	15.1129967
LOC_Os01g50032	LOC_Os01g50050	8.84258754
LOC_Os01g50050	LOC_Os03g56280	9.02721
LOC_Os01g50032	LOC_Os01g71990	10.2492457
LOC_Os01g49890	LOC_Os08g02400	8.96546376
LOC_Os01g50050	LOC_Os12g43630	10.1518177
LOC_Os01g50030	LOC_Os03g01610	9.43091661
LOC_Os01g50050	LOC_Os04g17064	9.61166783
LOC_Os01g50050	LOC_Os12g38750	8.85778088
LOC_Os01g50030	LOC_Os09g31430	9.27328448
LOC_Os01g50032	LOC_Os10g41689	9.25085004
LOC_Os01g49720	LOC_Os12g13800	8.82721651
LOC_Os01g49890	LOC_Os07g22600	12.8169442
LOC_Os01g49890	LOC_Os01g73580	9.281789
LOC_Os01g50050	LOC_Os05g04690	8.97084955
LOC_Os01g50050	LOC_Os12g12580	8.96156394
LOC_Os01g49890	LOC_Os01g53600	8.96181731
LOC_Os01g50050	LOC_Os05g46580	14.1155544
LOC_Os01g49890	LOC_Os07g04240	9.23183191
LOC_Os01g50030	LOC_Os07g31270	8.83020648
LOC_Os01g50032	LOC_Os08g34790	9.48724995
LOC_Os01g50032	LOC_Os03g18740	9.61182673

InteractorA	InteractorB	Z score
LOC_Os01g49720	LOC_Os07g34589	8.86899145
LOC_Os01g49890	LOC_Os02g19420	9.62354297
LOC_Os01g50030	LOC_Os12g03816	9.30055893
LOC_Os01g50030	LOC_Os03g01650	9.430986
LOC_Os01g50050	LOC_Os12g38770	9.59604315
LOC_Os01g49720	LOC_Os11g32650	9.71417113
LOC_Os01g50030	LOC_Os12g38760	8.83643709
LOC_Os01g50460	LOC_Os01g62230	9.05037527
LOC_Os01g50030	LOC_Os09g26880	8.85227804
LOC_Os01g49890	LOC_Os10g02070	8.98966215
LOC_Os01g50032	LOC_Os03g64050	9.33857047
LOC_Os01g49890	LOC_Os07g23730	9.49616347
LOC_Os01g50030	LOC_Os03g07840	8.94618085
LOC_Os01g49890	LOC_Os04g39840	9.37687166
LOC_Os01g49890	LOC_Os12g43490	9.22082597
LOC_Os01g49890	LOC_Os04g16874	9.65977204
LOC_Os01g49890	LOC_Os04g26910	9.05213811
LOC_Os01g50032	LOC_Os12g25700	9.50662073
LOC_Os01g49890	LOC_Os07g31770	11.8980336
LOC_Os01g49720	LOC_Os03g08560	9.02702854
LOC_Os01g50050	LOC_Os02g14180	9.82785794
LOC_Os01g49890	LOC_Os01g51700	8.83494302
LOC_Os01g50050	LOC_Os06g29220	9.58911103
LOC_Os01g50050	LOC_Os02g35590	9.033862
LOC_Os01g50030	LOC_Os10g08710	9.03857163
LOC_Os01g49720	LOC_Os03g16740	10.1893331
LOC_Os01g50030	LOC_Os11g47560	9.35158845
LOC_Os01g50032	LOC_Os02g15550	9.3967172
LOC_Os01g50030	LOC_Os08g06060	8.99013524
LOC_Os01g49890	LOC_Os10g23100	10.1539761
LOC_Os01g49890	LOC_Os02g41680	9.33724462
LOC_Os01g50030	LOC_Os08g44370	9.14258104
LOC_Os01g49890	LOC_Os08g34280	10.3050197
LOC_Os01g50030	LOC_Os06g06880	10.3492486
LOC_Os01g49890	LOC_Os02g14059	9.26892217
LOC_Os01g49890	LOC_Os05g12180	8.87696129
LOC_Os01g50030	LOC_Os10g39120	10.6827992
LOC_Os01g49720	LOC_Os04g58200	10.0576725
LOC_Os01g50050	LOC_Os02g07760	9.8359514
LOC_Os01g50050	LOC_Os04g56230	11.0678385
LOC_Os01g50030	LOC_Os03g47000	9.69065148
LOC_Os01g49890	LOC_Os10g38360	9.82533718
LOC_Os01g50030	LOC_Os07g38890	10.144998

InteractorA	InteractorB	Z score
LOC_Os01g50050	LOC_Os04g14680	8.8835852
LOC_Os01g49890	LOC_Os10g07040	9.30806704
LOC_Os01g49890	LOC_Os03g55600	9.21499746
LOC_Os01g50050	LOC_Os05g45590	9.2327158
LOC_Os01g49890	LOC_Os07g22950	9.87436604
LOC_Os01g49890	LOC_Os10g38350	9.38456921
LOC_Os01g50030	LOC_Os09g39570	9.86932478
LOC_Os01g49890	LOC_Os05g04510	8.95876411
LOC_Os01g49890	LOC_Os09g15790	9.87206561
LOC_Os01g50050	LOC_Os06g39140	9.75143662
LOC_Os01g50030	LOC_Os03g14530	10.4446122
LOC_Os01g50030	LOC_Os03g04260	9.25772871
LOC_Os01g50030	LOC_Os02g52640	9.33586842
LOC_Os01g50030	LOC_Os03g07150	9.00856563
LOC_Os01g49890	LOC_Os03g30470	9.06176717
LOC_Os01g49890	LOC_Os07g27790	9.08305067
LOC_Os01g49890	LOC_Os10g08022	9.40408478
LOC_Os01g50770	LOC_Os10g11810	9.39947506
LOC_Os01g51170	LOC_Os06g05690	8.88836463
LOC_Os01g51170	LOC_Os09g14670	9.38128416
LOC_Os01g51170	LOC_Os06g06300	9.46764378
LOC_Os01g50760	LOC_Os06g39140	8.92136784
LOC_Os01g51170	LOC_Os02g32760	8.89574537
LOC_Os01g50760	LOC_Os07g05180	8.98904157
LOC_Os01g51170	LOC_Os02g05410	9.15887567
LOC_Os01g50770	LOC_Os01g55540	11.4310661
LOC_Os01g51170	LOC_Os02g22780	9.96449804
LOC_Os01g51200	LOC_Os03g64050	10.3835844
LOC_Os01g50760	LOC_Os02g25940	9.13751431
LOC_Os01g50760	LOC_Os02g18930	8.82301772
LOC_Os01g51060	LOC_Os01g53900	9.91857588
LOC_Os01g50760	LOC_Os04g56210	13.3199595
LOC_Os01g51060	LOC_Os09g08720	10.8707694
LOC_Os01g51170	LOC_Os01g64850	9.91404493
LOC_Os01g51060	LOC_Os02g14929	9.57888288
LOC_Os01g51200	LOC_Os03g44150	9.96595654
LOC_Os01g51060	LOC_Os08g17500	9.31669247
LOC_Os01g51060	LOC_Os04g33740	8.80338086
LOC_Os01g51170	LOC_Os02g56460	10.7456542
LOC_Os01g50760	LOC_Os05g04520	11.3489268
LOC_Os01g50770	LOC_Os09g23540	9.37930698
LOC_Os01g51060	LOC_Os05g24580	12.5752825
LOC_Os01g51170	LOC_Os05g42190	9.08250619

InteractorA	InteractorB	Z score
LOC_Os01g51060	LOC_Os04g12960	8.8276221
LOC_Os01g50760	LOC_Os05g46580	11.5274347
LOC_Os01g50760	LOC_Os07g30200	9.28056824
LOC_Os01g50770	LOC_Os05g50380	9.13122492
LOC_Os01g51170	LOC_Os03g17480	9.16295806
LOC_Os01g51060	LOC_Os06g23440	9.67975133
LOC_Os01g51060	LOC_Os07g43260	8.9225012
LOC_Os01g51170	LOC_Os06g08310	9.35758561
LOC_Os01g50460	LOC_Os10g39840	8.89137428
LOC_Os01g51170	LOC_Os07g30970	9.82275753
LOC_Os01g51200	LOC_Os01g51220	8.90534362
LOC_Os01g50770	LOC_Os06g11280	9.02119982
LOC_Os01g51060	LOC_Os08g06060	10.0348536
LOC_Os01g51170	LOC_Os01g55870	8.86319738
LOC_Os01g50770	LOC_Os01g61400	8.89261141
LOC_Os01g51170	LOC_Os02g56680	10.9969857
LOC_Os01g51170	LOC_Os05g27950	8.81583784
LOC_Os01g51170	LOC_Os04g45290	10.6665069
LOC_Os01g50760	LOC_Os05g07880	9.691598
LOC_Os01g51060	LOC_Os09g26380	10.5729069
LOC_Os01g51170	LOC_Os12g24650	9.53154412
LOC_Os01g51200	LOC_Os03g15880	9.81231228
LOC_Os01g50460	LOC_Os07g48030	8.91317784
LOC_Os01g51170	LOC_Os05g45420	8.84818519
LOC_Os01g51200	LOC_Os04g42920	9.82333958
LOC_Os01g51060	LOC_Os07g05940	9.34534171
LOC_Os01g51200	LOC_Os04g16740	8.86064779
LOC_Os01g50760	LOC_Os04g56230	10.8265724
LOC_Os01g50770	LOC_Os04g18200	9.08304153
LOC_Os01g51200	LOC_Os01g64970	9.08476947
LOC_Os01g51200	LOC_Os03g22060	9.34980647
LOC_Os01g50760	LOC_Os08g36900	10.4529637
LOC_Os01g51200	LOC_Os04g30420	9.26383713
LOC_Os01g50760	LOC_Os05g45590	9.30048929
LOC_Os01g51200	LOC_Os03g02710	9.4545708
LOC_Os01g50770	LOC_Os12g22650	9.00031891
LOC_Os01g51170	LOC_Os04g32460	10.0514228
LOC_Os01g50760	LOC_Os05g45810	8.85366965
LOC_Os01g50770	LOC_Os07g42600	8.96924332
LOC_Os01g50760	LOC_Os10g27190	8.83776757
LOC_Os01g50770	LOC_Os03g57200	9.00001863
LOC_Os01g51170	LOC_Os08g44370	8.89009803
LOC_Os01g51170	LOC_Os03g26960	9.54586412

InteractorA	InteractorB	Z score
LOC_Os01g50760	LOC_Os01g51200	8.95955941
LOC_Os01g51170	LOC_Os07g48780	8.96755882
LOC_Os01g51170	LOC_Os06g08770	9.01764411
LOC_Os01g50460	LOC_Os10g38489	9.32134561
LOC_Os01g51060	LOC_Os02g04950	9.38820531
LOC_Os01g50460	LOC_Os04g17650	9.46741818
LOC_Os01g51060	LOC_Os03g49350	10.0161728
LOC_Os01g50760	LOC_Os07g26540	9.70043106
LOC_Os01g50460	LOC_Os06g48180	9.68359963
LOC_Os01g51170	LOC_Os03g20370	8.96754087
LOC_Os01g51200	LOC_Os03g63720	9.31663408
LOC_Os01g51170	LOC_Os02g10310	9.01556668
LOC_Os01g51170	LOC_Os05g36010	10.9639334
LOC_Os01g51170	LOC_Os04g38870	8.93579186
LOC_Os01g51170	LOC_Os05g46360	9.6665587
LOC_Os01g51060	LOC_Os09g39810	9.14121324
LOC_Os01g51170	LOC_Os06g05090	10.0431608
LOC_Os01g51200	LOC_Os03g62060	9.7187511
LOC_Os01g51060	LOC_Os02g14440	8.82920261
LOC_Os01g51170	LOC_Os03g48310	9.56500438
LOC_Os01g51170	LOC_Os08g44960	8.89331216
LOC_Os01g50770	LOC_Os05g04520	9.84771109
LOC_Os01g50760	LOC_Os11g37550	10.0611489
LOC_Os01g51200	LOC_Os03g57120	9.83170591
LOC_Os01g50770	LOC_Os07g43260	9.92680702
LOC_Os01g51060	LOC_Os09g15420	10.1640707
LOC_Os01g51060	LOC_Os02g01340	8.88102153
LOC_Os01g50460	LOC_Os07g41050	8.86655425
LOC_Os01g51200	LOC_Os01g62230	8.86952608
LOC_Os01g51200	LOC_Os04g53230	8.81365145
LOC_Os01g51060	LOC_Os03g58400	9.21074872
LOC_Os01g51060	LOC_Os06g22960	8.85394692
LOC_Os01g51220	LOC_Os11g47580	8.92505692
LOC_Os01g51220	LOC_Os02g56100	9.08764407
LOC_Os01g51210	LOC_Os03g21900	9.14900055
LOC_Os01g51570	LOC_Os04g44870	9.54101389
LOC_Os01g51220	LOC_Os07g05940	8.83605399
LOC_Os01g51410	LOC_Os04g58200	9.74532436
LOC_Os01g51220	LOC_Os10g17680	8.97004268
LOC_Os01g51210	LOC_Os09g30418	10.4354394
LOC_Os01g51220	LOC_Os04g55960	8.89249552
LOC_Os01g51410	LOC_Os03g10500	8.85547488
LOC_Os01g51570	LOC_Os04g38940	9.09441626

InteractorA	InteractorB	Z score
LOC_Os01g51210	LOC_Os09g25370	9.32629509
LOC_Os01g51410	LOC_Os04g57090	8.84922876
LOC_Os01g51410	LOC_Os03g01770	9.29281572
LOC_Os01g51220	LOC_Os04g01470	9.08915482
LOC_Os01g51220	LOC_Os01g60360	8.99052322
LOC_Os01g51220	LOC_Os03g55150	9.33067856
LOC_Os01g51220	LOC_Os02g19770	8.88164608
LOC_Os01g51220	LOC_Os07g44790	9.8197243
LOC_Os01g51220	LOC_Os01g60410	8.99052322
LOC_Os01g51220	LOC_Os02g08490	8.89464739
LOC_Os01g51570	LOC_Os03g32580	9.69729782
LOC_Os01g51410	LOC_Os12g34450	8.84106068
LOC_Os01g51570	LOC_Os04g31960	8.8751304
LOC_Os01g51570	LOC_Os04g56160	9.88780974
LOC_Os01g51570	LOC_Os03g12660	9.08441855
LOC_Os01g51410	LOC_Os01g57964	8.89521151
LOC_Os01g51210	LOC_Os04g56070	8.84403803
LOC_Os01g51570	LOC_Os04g12980	9.81738503
LOC_Os01g51220	LOC_Os09g36450	10.8038479
LOC_Os01g51210	LOC_Os09g33500	8.83654059
LOC_Os01g51210	LOC_Os11g10510	9.32408124
LOC_Os01g51220	LOC_Os02g58340	9.8613078
LOC_Os01g51200	LOC_Os07g35880	9.11794016
LOC_Os01g51210	LOC_Os04g12900	9.51662847
LOC_Os01g51220	LOC_Os10g28360	9.66641234
LOC_Os01g51200	LOC_Os10g38600	9.38448607
LOC_Os01g51210	LOC_Os06g22960	9.2386257
LOC_Os01g51410	LOC_Os04g16760	8.89511186
LOC_Os01g51210	LOC_Os05g46360	13.7428813
LOC_Os01g51210	LOC_Os03g01880	11.3309406
LOC_Os01g51410	LOC_Os12g21798	9.28915211
LOC_Os01g51210	LOC_Os03g55874	9.04797075
LOC_Os01g51220	LOC_Os01g54490	9.77759284
LOC_Os01g51380	LOC_Os08g42560	11.0472384
LOC_Os01g51570	LOC_Os06g08080	9.08201423
LOC_Os01g51200	LOC_Os09g38030	9.82364103
LOC_Os01g51220	LOC_Os01g67510	9.89174615
LOC_Os01g51220	LOC_Os08g42410	9.07247106
LOC_Os01g51200	LOC_Os10g37060	9.44872216
LOC_Os01g51210	LOC_Os10g33900	12.2400335
LOC_Os01g51220	LOC_Os02g38200	10.6006181
LOC_Os01g51220	LOC_Os12g12514	9.02145412
LOC_Os01g51410	LOC_Os07g46280	8.82773434

InteractorA	InteractorB	Z score
LOC_Os01g51570	LOC_Os03g18130	8.80808253
LOC_Os01g51210	LOC_Os02g13110	8.89999124
LOC_Os01g51220	LOC_Os03g56810	8.86885701
LOC_Os01g51410	LOC_Os02g06300	8.80573012
LOC_Os01g51200	LOC_Os10g38160	9.00749013
LOC_Os01g51570	LOC_Os01g59490	8.96662025
LOC_Os01g51220	LOC_Os10g17660	8.96996391
LOC_Os01g51200	LOC_Os07g30170	10.1553609
LOC_Os01g51220	LOC_Os10g08670	9.5014041
LOC_Os01g51410	LOC_Os02g07230	8.87529624
LOC_Os01g51200	LOC_Os07g25590	10.1260879
LOC_Os01g51410	LOC_Os06g36700	9.62521023
LOC_Os01g51210	LOC_Os03g58530	10.0126176
LOC_Os01g51410	LOC_Os02g50240	8.93116489
LOC_Os01g51220	LOC_Os03g51600	9.83249578
LOC_Os01g51200	LOC_Os12g02370	9.87140171
LOC_Os01g51380	LOC_Os04g01740	8.87488327
LOC_Os01g51380	LOC_Os02g47840	9.20035256
LOC_Os01g51410	LOC_Os07g09890	9.13074801
LOC_Os01g51410	LOC_Os10g21248	10.4458466
LOC_Os01g51570	LOC_Os05g10780	9.33137156
LOC_Os01g51410	LOC_Os04g44920	9.9892842
LOC_Os01g51220	LOC_Os06g11240	9.37794313
LOC_Os01g51220	LOC_Os06g47320	10.9105451
LOC_Os01g51380	LOC_Os02g19880	9.20575988
LOC_Os01g51220	LOC_Os04g17064	9.26926769
LOC_Os01g51220	LOC_Os06g05880	8.95347171
LOC_Os01g51410	LOC_Os01g54490	9.52178963
LOC_Os01g51380	LOC_Os12g02980	9.26526854
LOC_Os01g51410	LOC_Os04g55850	10.2454417
LOC_Os01g51220	LOC_Os04g56400	8.86168416
LOC_Os01g51210	LOC_Os07g28480	9.05996427
LOC_Os01g51220	LOC_Os06g35480	8.86348067
LOC_Os01g51410	LOC_Os02g47020	8.8375458
LOC_Os01g51220	LOC_Os04g02050	8.80587151
LOC_Os01g51220	LOC_Os07g23850	9.14741947
LOC_Os01g51570	LOC_Os03g04260	9.97088609
LOC_Os01g51380	LOC_Os01g73790	9.60250886
LOC_Os01g51200	LOC_Os10g38590	9.7648318
LOC_Os01g51410	LOC_Os08g27840	9.06193604
LOC_Os01g51570	LOC_Os03g08530	9.39033067
LOC_Os01g51220	LOC_Os02g04320	9.2284683
LOC_Os01g51570	LOC_Os01g73790	9.62422826

InteractorA	InteractorB	Z score
LOC_Os01g51200	LOC_Os06g07210	10.1827503
LOC_Os01g51220	LOC_Os02g55890	9.5272476
LOC_Os01g51380	LOC_Os10g05069	10.7117241
LOC_Os01g51210	LOC_Os01g70220	9.32857053
LOC_Os01g51380	LOC_Os10g26050	8.97686827
LOC_Os01g51200	LOC_Os07g05400	10.6644564
LOC_Os01g51220	LOC_Os05g46040	8.94054305
LOC_Os01g51380	LOC_Os08g41880	9.36108678
LOC_Os01g51220	LOC_Os03g28400	9.8197243
LOC_Os01g51380	LOC_Os06g48200	9.85588938
LOC_Os01g51570	LOC_Os06g15420	9.50351552
LOC_Os01g51410	LOC_Os05g01675	8.89520869
LOC_Os01g51210	LOC_Os07g44440	8.96180217
LOC_Os01g51200	LOC_Os05g23860	9.37177201
LOC_Os01g51220	LOC_Os12g44000	8.91183002
LOC_Os01g51570	LOC_Os03g62070	9.01208533
LOC_Os01g51210	LOC_Os09g37540	9.24271395
LOC_Os01g51410	LOC_Os03g16860	9.98729163
LOC_Os01g51410	LOC_Os07g01020	8.91664913
LOC_Os01g51410	LOC_Os01g71320	10.0646835
LOC_Os01g51200	LOC_Os06g40170	9.69540809
LOC_Os01g04340	LOC_Os11g40140	9.29769164
LOC_Os01g04340	LOC_Os02g02560	8.80076394
LOC_Os01g04370	LOC_Os07g37320	9.66725751
LOC_Os01g04370	LOC_Os08g44340	9.41935009
LOC_Os01g03950	LOC_Os08g04460	9.23600689
LOC_Os01g04340	LOC_Os10g07040	9.33384543
LOC_Os01g04360	LOC_Os06g04620	9.26788228
LOC_Os01g04340	LOC_Os10g35840	10.1680449
LOC_Os01g04370	LOC_Os01g28450	9.73330748
LOC_Os01g04380	LOC_Os01g71810	8.83281746
LOC_Os01g04370	LOC_Os03g59310	9.84996369
LOC_Os01g04370	LOC_Os10g25950	10.1120581
LOC_Os01g04370	LOC_Os06g23780	8.86472945
LOC_Os01g04370	LOC_Os12g25630	8.85365935
LOC_Os01g04340	LOC_Os12g06620	9.02292848
LOC_Os01g04380	LOC_Os03g11900	9.92822594
LOC_Os01g04360	LOC_Os07g30990	9.21039534
LOC_Os01g03950	LOC_Os10g41510	9.01780312
LOC_Os01g04380	LOC_Os10g38690	9.22151128
LOC_Os01g04370	LOC_Os08g41880	10.267037
LOC_Os01g03950	LOC_Os09g37100	8.87183931
LOC_Os01g04340	LOC_Os01g71300	9.04699438

InteractorA	InteractorB	Z score
LOC_Os01g04370	LOC_Os12g07820	9.4889027
LOC_Os01g04340	LOC_Os11g32580	8.86187458
LOC_Os01g03950	LOC_Os04g43800	8.86977496
LOC_Os01g04370	LOC_Os11g30290	9.36053886
LOC_Os01g03950	LOC_Os10g22310	8.99240711
LOC_Os01g04340	LOC_Os11g16590	9.10563559
LOC_Os01g04380	LOC_Os07g30970	9.2587723
LOC_Os01g04360	LOC_Os02g03870	9.92848624
LOC_Os01g04370	LOC_Os01g38670	9.39869861
LOC_Os01g04340	LOC_Os07g22950	9.2891567
LOC_Os01g04360	LOC_Os04g59600	9.23182094
LOC_Os01g04360	LOC_Os12g10730	10.4648944
LOC_Os01g04370	LOC_Os11g40140	9.10750161
LOC_Os01g04360	LOC_Os11g05880	9.72081401
LOC_Os01g03950	LOC_Os06g36670	8.87119892
LOC_Os01g04370	LOC_Os02g01920	8.80152868
LOC_Os01g04360	LOC_Os07g35940	9.05441932
LOC_Os01g03950	LOC_Os07g01560	9.34263936
LOC_Os01g04360	LOC_Os05g48030	9.20559421
LOC_Os01g04340	LOC_Os03g59020	9.2381193
LOC_Os01g04340	LOC_Os09g07510	9.32956736
LOC_Os01g04360	LOC_Os03g45320	9.53990413
LOC_Os01g04360	LOC_Os03g31750	8.95590593
LOC_Os01g04370	LOC_Os10g09860	9.31963105
LOC_Os01g04360	LOC_Os04g10010	9.8627909
LOC_Os01g04380	LOC_Os01g64630	9.01617596
LOC_Os01g04340	LOC_Os07g02340	9.00410119
LOC_Os01g04370	LOC_Os01g08350	8.9094894
LOC_Os01g04380	LOC_Os03g53800	9.35518386
LOC_Os01g04380	LOC_Os06g02144	9.05997007
LOC_Os01g04370	LOC_Os10g21250	9.55993001
LOC_Os01g04340	LOC_Os03g05290	9.0944773
LOC_Os01g04340	LOC_Os02g32370	10.3872575
LOC_Os01g04340	LOC_Os09g07460	8.95183478
LOC_Os01g04340	LOC_Os02g47600	9.03271923
LOC_Os01g03950	LOC_Os07g02210	9.61383599
LOC_Os01g03950	LOC_Os06g03720	10.2394727
LOC_Os01g05490	LOC_Os01g51220	8.80424431
LOC_Os01g05490	LOC_Os01g62420	10.2769284
LOC_Os01g04380	LOC_Os05g23740	9.03105634
LOC_Os01g04370	LOC_Os08g43560	9.07235396
LOC_Os01g04370	LOC_Os07g48430	8.88439178
LOC_Os01g04340	LOC_Os02g33110	9.69328957

InteractorA	InteractorB	Z score
LOC_Os01g03950	LOC_Os05g42350	9.4428413
LOC_Os01g04380	LOC_Os12g31640	10.0699499
LOC_Os01g04340	LOC_Os12g37960	9.28243555
LOC_Os01g04380	LOC_Os05g47640	9.70770141
LOC_Os01g04340	LOC_Os02g44080	9.40767494
LOC_Os01g04360	LOC_Os01g40400	9.38629766
LOC_Os01g04380	LOC_Os10g41190	9.19205722
LOC_Os01g04370	LOC_Os05g12240	9.0448893
LOC_Os01g04380	LOC_Os04g44890	9.07463507
LOC_Os01g04370	LOC_Os08g40930	11.1247822
LOC_Os01g04360	LOC_Os12g38760	9.96409355
LOC_Os01g03950	LOC_Os10g42720	9.19254875
LOC_Os01g04360	LOC_Os03g07840	9.06751756
LOC_Os01g04340	LOC_Os06g29844	9.57297632
LOC_Os01g04370	LOC_Os01g71310	9.62377573
LOC_Os01g04340	LOC_Os08g34190	9.70596266
LOC_Os01g04370	LOC_Os03g64050	9.08592981
LOC_Os01g04340	LOC_Os05g39690	10.0285335
LOC_Os01g04340	LOC_Os01g73220	9.5911274
LOC_Os01g04380	LOC_Os05g08430	9.31774751
LOC_Os01g04360	LOC_Os04g56210	10.0493917
LOC_Os01g04360	LOC_Os03g51550	9.46474866
LOC_Os01g04360	LOC_Os02g38340	8.87583356
LOC_Os01g04360	LOC_Os04g55960	9.93257566
LOC_Os01g04370	LOC_Os02g10310	9.34779304
LOC_Os01g03950	LOC_Os06g04270	8.84861339
LOC_Os01g04360	LOC_Os11g30290	9.63159342
LOC_Os01g04370	LOC_Os07g26150	8.89286391
LOC_Os01g04380	LOC_Os05g51630	9.68606449
LOC_Os01g05490	LOC_Os01g22336	9.71092148
LOC_Os01g04340	LOC_Os04g37820	9.18899349
LOC_Os01g03950	LOC_Os10g36650	9.37322116
LOC_Os01g04380	LOC_Os06g05250	9.62580564
LOC_Os01g04380	LOC_Os10g30580	10.4012325
LOC_Os01g04370	LOC_Os11g25260	9.32350107
LOC_Os01g04340	LOC_Os04g57090	8.99880053
LOC_Os01g04370	LOC_Os10g41190	11.013044
LOC_Os01g04340	LOC_Os04g39880	8.91289962
LOC_Os01g04360	LOC_Os03g11420	8.89303819
LOC_Os01g04380	LOC_Os03g59310	9.51905123
LOC_Os01g04360	LOC_Os01g21180	10.5232078
LOC_Os01g04340	LOC_Os01g18120	10.5558745
LOC_Os01g04340	LOC_Os07g26900	8.87830586

InteractorA	InteractorB	Z score
LOC_Os01g04360	LOC_Os04g33480	8.87408869
LOC_Os01g04360	LOC_Os02g56130	10.5684066
LOC_Os01g04340	LOC_Os01g19150	9.6786688
LOC_Os01g52450	LOC_Os03g64210	10.6272578
LOC_Os01g51570	LOC_Os09g39380	9.0298542
LOC_Os01g51700	LOC_Os08g43560	8.84323726
LOC_Os01g51570	LOC_Os06g45100	10.0575092
LOC_Os01g52400	LOC_Os10g26050	10.4398345
LOC_Os01g52470	LOC_Os03g62060	9.36524625
LOC_Os01g52450	LOC_Os03g15960	8.90409408
LOC_Os01g52240	LOC_Os12g24650	12.2530017
LOC_Os01g51570	LOC_Os12g23630	9.35860543
LOC_Os01g52240	LOC_Os01g53900	9.03192612
LOC_Os01g52470	LOC_Os01g62244	8.82041425
LOC_Os01g52400	LOC_Os07g05940	9.98776389
LOC_Os01g52400	LOC_Os11g10520	9.21310915
LOC_Os01g52450	LOC_Os02g07160	9.77187126
LOC_Os01g51570	LOC_Os08g40140	10.4068346
LOC_Os01g52400	LOC_Os03g08010	9.92260905
LOC_Os01g51700	LOC_Os03g22120	9.69411752
LOC_Os01g51570	LOC_Os12g41110	9.13106875
LOC_Os01g52240	LOC_Os11g43360	9.04218182
LOC_Os01g52400	LOC_Os08g40140	12.2512204
LOC_Os01g52470	LOC_Os02g09490	8.91686772
LOC_Os01g52400	LOC_Os05g41080	9.0294867
LOC_Os01g51700	LOC_Os04g55960	8.80208127
LOC_Os01g51700	LOC_Os07g09890	8.80065687
LOC_Os01g51570	LOC_Os08g44210	8.97679843
LOC_Os01g52400	LOC_Os08g41990	8.9231632
LOC_Os01g51570	LOC_Os11g20790	9.02908177
LOC_Os01g52400	LOC_Os09g10270	8.95522412
LOC_Os01g52470	LOC_Os03g56460	9.13762628
LOC_Os01g52240	LOC_Os02g14059	9.54117799
LOC_Os01g52400	LOC_Os10g38140	9.3733413
LOC_Os01g51570	LOC_Os11g33270	8.87138235
LOC_Os01g52450	LOC_Os05g34540	9.3336701
LOC_Os01g52400	LOC_Os03g62670	10.7872527
LOC_Os01g52450	LOC_Os01g55240	8.88271336
LOC_Os01g51700	LOC_Os07g46990	10.4471921
LOC_Os01g52450	LOC_Os05g41640	9.07594445
LOC_Os01g52400	LOC_Os01g71340	8.91304321
LOC_Os01g52470	LOC_Os08g32850	8.91475715
LOC_Os01g51700	LOC_Os02g14170	8.84845769

InteractorA	InteractorB	Z score
LOC_Os01g52400	LOC_Os05g12210	9.22270141
LOC_Os01g52240	LOC_Os05g50710	9.02144238
LOC_Os01g52470	LOC_Os03g53790	9.9530375
LOC_Os01g52240	LOC_Os10g38229	9.10927976
LOC_Os01g51700	LOC_Os10g34760	11.0107535
LOC_Os01g52470	LOC_Os04g12900	9.47049809
LOC_Os01g52450	LOC_Os05g04340	10.1357776
LOC_Os01g52400	LOC_Os07g42950	9.05538666
LOC_Os01g52240	LOC_Os03g58980	11.8761696
LOC_Os01g52470	LOC_Os03g38000	9.18342194
LOC_Os01g52450	LOC_Os09g28420	9.68815465
LOC_Os01g52400	LOC_Os12g01922	9.13917466
LOC_Os01g52450	LOC_Os02g07060	8.91807454
LOC_Os01g51570	LOC_Os07g06440	9.32392706
LOC_Os01g52400	LOC_Os04g35240	8.9240682
LOC_Os01g51570	LOC_Os10g30840	8.96246319
LOC_Os01g52450	LOC_Os06g03770	10.2099588
LOC_Os01g52400	LOC_Os03g40330	8.86357062
LOC_Os01g52470	LOC_Os07g34580	10.2392033
LOC_Os01g52470	LOC_Os07g07060	8.88583729
LOC_Os01g52470	LOC_Os08g16910	9.15971516
LOC_Os01g52450	LOC_Os01g74450	10.7790102
LOC_Os01g51700	LOC_Os12g12590	10.3165979
LOC_Os01g52450	LOC_Os04g46460	9.42648112
LOC_Os01g52400	LOC_Os03g10620	8.88225021
LOC_Os01g52470	LOC_Os04g58640	9.2017611
LOC_Os01g52470	LOC_Os02g44080	9.12878414
LOC_Os01g52400	LOC_Os05g35400	9.00111645
LOC_Os01g52450	LOC_Os04g02820	9.07225759
LOC_Os01g52470	LOC_Os01g52500	8.86565303
LOC_Os01g52450	LOC_Os03g04970	11.5813783
LOC_Os01g52400	LOC_Os03g08020	9.92257476
LOC_Os01g52450	LOC_Os05g29880	9.0653923
LOC_Os01g51570	LOC_Os11g40150	8.80460225
LOC_Os01g52450	LOC_Os03g22530	8.81937536
LOC_Os01g52470	LOC_Os06g04270	9.05681439
LOC_Os01g52470	LOC_Os05g41080	9.6060311
LOC_Os01g52400	LOC_Os01g70770	9.1797294
LOC_Os01g52450	LOC_Os03g19390	9.0065087
LOC_Os01g52240	LOC_Os03g04970	10.1935718
LOC_Os01g52450	LOC_Os03g62670	9.2718804
LOC_Os01g52240	LOC_Os04g36700	8.84130026
LOC_Os01g51700	LOC_Os12g02980	9.28099897

InteractorA	InteractorB	Z score
LOC_Os01g51570	LOC_Os07g39290	9.43517761
LOC_Os01g52400	LOC_Os10g11810	9.24706793
LOC_Os01g51570	LOC_Os08g44530	8.94805873
LOC_Os01g52400	LOC_Os07g07550	9.97357604
LOC_Os01g52400	LOC_Os03g08050	9.92257476
LOC_Os01g52450	LOC_Os03g07840	12.0563254
LOC_Os01g52470	LOC_Os03g04410	10.1296795
LOC_Os01g51570	LOC_Os09g12660	9.31312055
LOC_Os01g52470	LOC_Os05g25550	8.95689173
LOC_Os01g52470	LOC_Os03g64030	9.55523442
LOC_Os01g52450	LOC_Os08g06100	9.19469308
LOC_Os01g52470	LOC_Os05g26890	9.02206391
LOC_Os01g51700	LOC_Os02g09490	10.0380555
LOC_Os01g52450	LOC_Os02g24354	9.00039793
LOC_Os01g52450	LOC_Os02g57720	9.66260778
LOC_Os01g52470	LOC_Os04g12690	9.68652567
LOC_Os01g52470	LOC_Os03g01290	8.82069502
LOC_Os01g51570	LOC_Os12g12590	9.05300366
LOC_Os01g52470	LOC_Os02g27760	9.29590861
LOC_Os01g52450	LOC_Os03g20700	8.84042775
LOC_Os01g51570	LOC_Os08g31870	9.54362019
LOC_Os01g52400	LOC_Os03g22120	10.6984988
LOC_Os01g52450	LOC_Os03g61970	9.71963188
LOC_Os01g51700	LOC_Os08g43190	9.15407563
LOC_Os01g51700	LOC_Os05g05670	9.81983798
LOC_Os01g52470	LOC_Os04g57220	9.07913335
LOC_Os01g52490	LOC_Os11g33240	10.2582582
LOC_Os01g52500	LOC_Os02g52800	9.20629908
LOC_Os01g53280	LOC_Os09g24412	10.0092299
LOC_Os01g52500	LOC_Os04g59450	9.08834164
LOC_Os01g53600	LOC_Os08g28820	9.90785729
LOC_Os01g52490	LOC_Os04g48700	9.02987671
LOC_Os01g53280	LOC_Os01g60190	9.86480931
LOC_Os01g53280	LOC_Os02g12900	9.22623176
LOC_Os01g52500	LOC_Os05g31140	10.3166106
LOC_Os01g53280	LOC_Os04g16772	10.0092288
LOC_Os01g53600	LOC_Os02g51100	10.7084712
LOC_Os01g52470	LOC_Os09g28400	9.22189565
LOC_Os01g53280	LOC_Os01g72460	8.84469623
LOC_Os01g52470	LOC_Os10g30200	9.00974621
LOC_Os01g52500	LOC_Os01g73580	9.9461996
LOC_Os01g52500	LOC_Os02g13110	9.06314452
LOC_Os01g52470	LOC_Os09g08120	9.03710035

InteractorA	InteractorB	Z score
LOC_Os01g53280	LOC_Os11g25330	8.83016786
LOC_Os01g53280	LOC_Os02g33140	8.90454951
LOC_Os01g52500	LOC_Os07g34520	10.2877914
LOC_Os01g52500	LOC_Os01g53280	11.6852691
LOC_Os01g53280	LOC_Os07g25024	10.0092288
LOC_Os01g53280	LOC_Os12g37960	9.83840475
LOC_Os01g53600	LOC_Os06g45710	10.0605102
LOC_Os01g52500	LOC_Os08g37800	9.38691799
LOC_Os01g52500	LOC_Os03g48310	9.25501796
LOC_Os01g53079	LOC_Os04g37640	9.81862577
LOC_Os01g53600	LOC_Os04g16872	8.80108325
LOC_Os01g53280	LOC_Os10g21352	10.0092299
LOC_Os01g53280	LOC_Os10g39840	9.17439265
LOC_Os01g52500	LOC_Os02g55300	9.40280212
LOC_Os01g52470	LOC_Os09g39380	8.87721875
LOC_Os01g52500	LOC_Os11g26910	8.89316774
LOC_Os01g53280	LOC_Os07g20544	8.90189788
LOC_Os01g53280	LOC_Os04g25990	9.94686334
LOC_Os01g52470	LOC_Os08g37800	8.93894862
LOC_Os01g52500	LOC_Os09g08072	9.81316128
LOC_Os01g53280	LOC_Os03g31300	9.2216018
LOC_Os01g52500	LOC_Os12g40550	9.49937913
LOC_Os01g52500	LOC_Os04g44060	9.48129389
LOC_Os01g53600	LOC_Os07g05150	10.0434188
LOC_Os01g52500	LOC_Os05g23740	10.1209362
LOC_Os01g53280	LOC_Os07g38730	9.06915737
LOC_Os01g52500	LOC_Os02g52590	9.61098487
LOC_Os01g52490	LOC_Os06g37660	9.18212618
LOC_Os01g53280	LOC_Os10g02480	9.77561951
LOC_Os01g52500	LOC_Os10g02040	10.8810663
LOC_Os01g52500	LOC_Os01g54030	9.15469675
LOC_Os01g53280	LOC_Os07g48430	9.7225431
LOC_Os01g52500	LOC_Os03g62070	9.16409348
LOC_Os01g52500	LOC_Os05g50890	10.3986829
LOC_Os01g53280	LOC_Os03g57140	9.57944272
LOC_Os01g53079	LOC_Os04g26910	8.93347459
LOC_Os01g53280	LOC_Os03g14450	9.02013421
LOC_Os01g52500	LOC_Os03g16740	8.8971157
LOC_Os01g52500	LOC_Os03g64210	9.15468213
LOC_Os01g53280	LOC_Os07g26540	10.3461719
LOC_Os01g52490	LOC_Os04g17650	9.09542267
LOC_Os01g52500	LOC_Os08g35740	10.1376399
LOC_Os01g53600	LOC_Os01g73680	9.3598166

InteractorA	InteractorB	Z score
LOC_Os01g53600	LOC_Os05g38560	8.94429218
LOC_Os01g53280	LOC_Os08g15266	10.0092302
LOC_Os01g53280	LOC_Os02g19420	10.3658628
LOC_Os01g53600	LOC_Os10g37060	9.88495342
LOC_Os01g52500	LOC_Os05g33400	10.1701515
LOC_Os01g52500	LOC_Os09g10230	8.95036264
LOC_Os01g52500	LOC_Os11g02100	8.82938549
LOC_Os01g52500	LOC_Os11g07020	9.5992956
LOC_Os01g52500	LOC_Os11g41130	9.37499528
LOC_Os01g53280	LOC_Os01g74650	9.5149029
LOC_Os01g52470	LOC_Os12g01922	9.57974148
LOC_Os01g53280	LOC_Os10g38150	8.94468261
LOC_Os01g52490	LOC_Os04g16874	8.89666479
LOC_Os01g53280	LOC_Os10g14150	9.19742922
LOC_Os01g52500	LOC_Os06g33210	9.2441233
LOC_Os01g53280	LOC_Os04g16818	10.0092299
LOC_Os01g53600	LOC_Os03g42110	10.1495142
LOC_Os01g53079	LOC_Os09g25390	9.08043528
LOC_Os01g53930	LOC_Os02g07230	9.1365699
LOC_Os01g54030	LOC_Os06g36670	10.174808
LOC_Os01g53900	LOC_Os06g11290	11.4364395
LOC_Os01g53900	LOC_Os02g10070	9.54715616
LOC_Os01g54030	LOC_Os02g51910	9.6122224
LOC_Os01g53900	LOC_Os12g44000	9.72613175
LOC_Os01g54370	LOC_Os07g08170	9.12184413
LOC_Os01g54030	LOC_Os12g44000	8.81532119
LOC_Os01g54030	LOC_Os02g49980	8.83839339
LOC_Os01g54030	LOC_Os07g48160	9.17192312
LOC_Os01g53900	LOC_Os10g23900	9.66188035
LOC_Os01g53900	LOC_Os12g42876	9.25095509
LOC_Os01g53930	LOC_Os05g47540	10.9932893
LOC_Os01g53900	LOC_Os06g10910	9.45935877
LOC_Os01g55030	LOC_Os01g56510	9.41609604
LOC_Os01g53930	LOC_Os04g43800	9.05707529
LOC_Os01g54030	LOC_Os02g47800	9.99996176
LOC_Os01g53900	LOC_Os10g21248	9.11633853
LOC_Os01g54370	LOC_Os07g03288	9.28710661
LOC_Os01g53900	LOC_Os06g46372	8.91210169
LOC_Os01g54370	LOC_Os07g03377	9.28710661
LOC_Os01g54030	LOC_Os06g21570	9.20133799
LOC_Os01g53930	LOC_Os02g49720	9.13388357
LOC_Os01g54490	LOC_Os10g21268	9.42292313
LOC_Os01g53930	LOC_Os04g56160	10.1304824

InteractorA	InteractorB	Z score
LOC_Os01g53930	LOC_Os02g56180	8.95167706
LOC_Os01g53900	LOC_Os07g06970	9.03814398
LOC_Os01g54370	LOC_Os07g03467	9.28710661
LOC_Os01g54030	LOC_Os05g07880	9.97496467
LOC_Os01g53900	LOC_Os04g57410	8.89689805
LOC_Os01g54490	LOC_Os05g12180	9.39019462
LOC_Os01g54490	LOC_Os05g20050	9.40778049
LOC_Os01g53900	LOC_Os06g02380	10.4726324
LOC_Os01g53900	LOC_Os03g48471	9.87243448
LOC_Os01g54490	LOC_Os06g40940	10.9055412
LOC_Os01g54490	LOC_Os01g58020	9.42293838
LOC_Os01g54370	LOC_Os07g02210	10.1301813
LOC_Os01g53930	LOC_Os03g04110	8.81345013
LOC_Os01g54030	LOC_Os09g08072	8.92934494
LOC_Os01g54490	LOC_Os01g64630	9.23558158
LOC_Os01g54370	LOC_Os02g56180	10.1532966
LOC_Os01g53900	LOC_Os04g33240	9.68920477
LOC_Os01g54490	LOC_Os02g52710	10.825377
LOC_Os01g53930	LOC_Os03g05980	9.73742468
LOC_Os01g53930	LOC_Os06g39708	9.53204201
LOC_Os01g54370	LOC_Os05g33130	9.14175745
LOC_Os01g54370	LOC_Os07g05180	9.04991155
LOC_Os01g54030	LOC_Os04g45490	10.2104868
LOC_Os01g53900	LOC_Os12g33610	10.2971614
LOC_Os01g54030	LOC_Os04g14680	9.91968937
LOC_Os01g54030	LOC_Os12g34450	9.37344303
LOC_Os01g54030	LOC_Os04g31070	9.33037645
LOC_Os01g54490	LOC_Os03g61340	9.31083515
LOC_Os01g53900	LOC_Os01g59440	9.07050502
LOC_Os01g55030	LOC_Os01g71340	9.39459387
LOC_Os01g54030	LOC_Os09g34960	8.83467508
LOC_Os01g54030	LOC_Os04g45290	9.40489621
LOC_Os01g54030	LOC_Os03g03910	9.47238452
LOC_Os01g53930	LOC_Os02g14460	9.4437494
LOC_Os01g54370	LOC_Os07g03590	9.28710661
LOC_Os01g53900	LOC_Os11g34450	9.54698227
LOC_Os01g54030	LOC_Os05g29880	9.96395176
LOC_Os01g54030	LOC_Os04g16680	9.06795166
LOC_Os01g54030	LOC_Os05g09440	13.1825761
LOC_Os01g53930	LOC_Os02g06700	8.88149542
LOC_Os01g54490	LOC_Os04g12980	8.8884109
LOC_Os01g54490	LOC_Os03g15360	10.9513242
LOC_Os01g53930	LOC_Os12g40550	9.37600074

InteractorA	InteractorB	Z score
LOC_Os01g53930	LOC_Os04g25990	8.90969603
LOC_Os01g55030	LOC_Os01g73580	9.06792201
LOC_Os01g54370	LOC_Os03g16740	8.9055812
LOC_Os01g53900	LOC_Os06g01850	9.73616016
LOC_Os01g53900	LOC_Os04g40130	9.23633013
LOC_Os01g54370	LOC_Os04g49130	9.04562962
LOC_Os01g54490	LOC_Os03g20370	9.82406579
LOC_Os01g54030	LOC_Os02g47790	9.56941252
LOC_Os01g54490	LOC_Os03g22060	10.0351931
LOC_Os01g53900	LOC_Os02g52560	8.86637575
LOC_Os01g54030	LOC_Os10g26130	9.02959032
LOC_Os01g53930	LOC_Os01g70220	8.97768615
LOC_Os01g53930	LOC_Os06g45120	9.22429343
LOC_Os01g54030	LOC_Os04g58880	9.05054328
LOC_Os01g54490	LOC_Os05g35330	9.42292313
LOC_Os01g54370	LOC_Os05g30480	9.84636523
LOC_Os01g53900	LOC_Os10g38360	8.96690434
LOC_Os01g53930	LOC_Os02g51830	9.88403653
LOC_Os01g54030	LOC_Os10g38229	9.7933479
LOC_Os01g53900	LOC_Os11g06390	8.87030377
LOC_Os01g54490	LOC_Os07g48780	9.82406579
LOC_Os01g54370	LOC_Os12g32240	9.20435024
LOC_Os01g54030	LOC_Os01g71340	8.89440662
LOC_Os01g54370	LOC_Os09g15790	9.61648945
LOC_Os01g54490	LOC_Os10g09860	9.49821596
LOC_Os01g53930	LOC_Os11g38959	9.73739881
LOC_Os01g54490	LOC_Os02g52700	10.8254058
LOC_Os01g54490	LOC_Os06g40180	9.12397986
LOC_Os01g53930	LOC_Os07g07240	8.82471983
LOC_Os01g54030	LOC_Os04g40310	8.85152714
LOC_Os01g53930	LOC_Os06g39230	9.05308043
LOC_Os01g54370	LOC_Os07g43260	9.30539526
LOC_Os01g54490	LOC_Os06g51150	9.1304597
LOC_Os01g54490	LOC_Os02g52560	10.48919
LOC_Os01g54370	LOC_Os03g53800	9.03260146
LOC_Os01g54370	LOC_Os09g34214	10.4304821
LOC_Os01g53930	LOC_Os06g04280	9.48815662
LOC_Os01g53900	LOC_Os10g41480	9.75863447
LOC_Os01g53930	LOC_Os03g01640	9.56777922
LOC_Os01g54370	LOC_Os06g37080	9.11168027
LOC_Os01g53900	LOC_Os06g39708	9.17740569
LOC_Os01g54030	LOC_Os04g16740	9.42531701
LOC_Os01g54030	LOC_Os12g25630	11.5731457

InteractorA	InteractorB	Z score
LOC_Os01g53900	LOC_Os02g20850	9.48999942
LOC_Os01g53900	LOC_Os03g18570	11.4651877
LOC_Os01g54370	LOC_Os05g06970	10.104827
LOC_Os01g53900	LOC_Os02g08130	8.97917814
LOC_Os01g53930	LOC_Os02g41670	9.40746401
LOC_Os01g54030	LOC_Os04g44730	10.1558634
LOC_Os01g54490	LOC_Os04g39814	10.7291351
LOC_Os01g53930	LOC_Os09g23530	9.23513203
LOC_Os01g54370	LOC_Os06g11240	9.39886323
LOC_Os01g53930	LOC_Os05g33240	8.89304723
LOC_Os01g53930	LOC_Os03g49260	12.2689601
LOC_Os01g54490	LOC_Os04g55960	10.8890545
LOC_Os01g54370	LOC_Os04g39840	8.91401065
LOC_Os01g54490	LOC_Os06g23780	9.33338184
LOC_Os01g54370	LOC_Os06g06980	9.65384923
LOC_Os01g53900	LOC_Os10g11140	10.0126662
LOC_Os01g54370	LOC_Os03g51550	9.11113987
LOC_Os01g53930	LOC_Os12g32240	9.20925148
LOC_Os01g54030	LOC_Os09g04050	9.03021345
LOC_Os01g54030	LOC_Os06g06980	9.34772386
LOC_Os01g54030	LOC_Os05g46580	8.90706097
LOC_Os01g53930	LOC_Os07g35880	9.02788966
LOC_Os01g55870	LOC_Os02g47800	9.16014216
LOC_Os01g55830	LOC_Os10g21192	8.93155719
LOC_Os01g55350	LOC_Os08g32620	9.76406727
LOC_Os01g55030	LOC_Os04g52340	11.6282349
LOC_Os01g55030	LOC_Os08g17500	9.14675544
LOC_Os01g55350	LOC_Os06g46284	8.82342886
LOC_Os01g55830	LOC_Os08g09950	8.99949228
LOC_Os01g55870	LOC_Os02g10320	8.99717139
LOC_Os01g55030	LOC_Os03g01800	8.89668862
LOC_Os01g55030	LOC_Os03g53650	10.4586487
LOC_Os01g55350	LOC_Os10g34760	10.2111109
LOC_Os01g55240	LOC_Os04g56730	9.59636385
LOC_Os01g55870	LOC_Os03g18570	9.64698061
LOC_Os01g55350	LOC_Os05g44760	9.1953093
LOC_Os01g55240	LOC_Os07g44460	10.279913
LOC_Os01g55830	LOC_Os04g40990	10.131784
LOC_Os01g55350	LOC_Os12g12560	8.99583176
LOC_Os01g55540	LOC_Os04g16740	9.51293858
LOC_Os01g55350	LOC_Os09g32830	8.81616934
LOC_Os01g55540	LOC_Os12g13390	12.1123517
LOC_Os01g55540	LOC_Os11g08470	9.22386066

InteractorA	InteractorB	Z score
LOC_Os01g55540	LOC_Os06g21570	9.13984429
LOC_Os01g55030	LOC_Os11g33270	9.77056253
LOC_Os01g55350	LOC_Os04g40130	8.8872903
LOC_Os01g55350	LOC_Os08g44340	8.9678072
LOC_Os01g55030	LOC_Os11g19800	9.70563007
LOC_Os01g55540	LOC_Os05g04510	10.5116846
LOC_Os01g55870	LOC_Os03g25320	11.6903746
LOC_Os01g55540	LOC_Os03g52090	9.16726313
LOC_Os01g55030	LOC_Os12g38180	10.1382708
LOC_Os01g55350	LOC_Os03g11960	9.29544644
LOC_Os01g55350	LOC_Os04g32650	8.88525917
LOC_Os01g55830	LOC_Os09g38030	9.29750052
LOC_Os01g55350	LOC_Os10g42280	8.90426724
LOC_Os01g55240	LOC_Os02g14170	9.23672405
LOC_Os01g55350	LOC_Os09g10230	10.5467248
LOC_Os01g55540	LOC_Os06g46940	10.1152274
LOC_Os01g55030	LOC_Os06g10970	8.85673728
LOC_Os01g55240	LOC_Os11g32580	8.90827485
LOC_Os01g55830	LOC_Os07g42950	8.98544673
LOC_Os01g55240	LOC_Os02g50880	8.95877561
LOC_Os01g55030	LOC_Os07g29750	9.849485
LOC_Os01g55240	LOC_Os10g26130	9.46473842
LOC_Os01g55350	LOC_Os05g38560	9.3317293
LOC_Os01g55540	LOC_Os07g30170	9.3655934
LOC_Os01g55030	LOC_Os03g53690	9.28362364
LOC_Os01g55540	LOC_Os02g52230	8.81662644
LOC_Os01g55830	LOC_Os08g34280	9.22895047
LOC_Os01g55830	LOC_Os10g22070	8.94917651
LOC_Os01g55540	LOC_Os04g47360	8.86531646
LOC_Os01g55540	LOC_Os04g19740	8.86289434
LOC_Os01g55350	LOC_Os01g73140	9.41182471
LOC_Os01g55240	LOC_Os02g51830	8.84766785
LOC_Os01g55350	LOC_Os09g36450	9.8022075
LOC_Os01g55830	LOC_Os06g11280	8.9577039
LOC_Os01g55240	LOC_Os02g07160	9.01308291
LOC_Os01g55030	LOC_Os04g56730	9.32622539
LOC_Os01g55830	LOC_Os10g32680	10.3712737
LOC_Os01g55830	LOC_Os06g12990	9.38923586
LOC_Os01g55540	LOC_Os06g35540	12.1614856
LOC_Os01g55240	LOC_Os04g39880	9.64020641
LOC_Os01g55830	LOC_Os07g07550	9.39748264
LOC_Os01g55830	LOC_Os09g20284	9.19798171
LOC_Os01g55030	LOC_Os12g08270	9.19458295

InteractorA	InteractorB	Z score
LOC_Os01g55540	LOC_Os06g08080	8.97562029
LOC_Os01g55830	LOC_Os08g23730	10.8423837
LOC_Os01g55830	LOC_Os09g39380	8.81969929
LOC_Os01g55350	LOC_Os04g37640	9.01631739
LOC_Os01g55350	LOC_Os10g09860	9.1158576
LOC_Os01g55350	LOC_Os03g59310	9.32212205
LOC_Os01g55240	LOC_Os03g17980	9.18299525
LOC_Os01g55540	LOC_Os06g02490	9.67924272
LOC_Os01g55350	LOC_Os03g04110	8.96804353
LOC_Os01g55540	LOC_Os03g26450	9.32939247
LOC_Os01g55830	LOC_Os08g42410	8.92285021
LOC_Os01g55540	LOC_Os06g11210	9.56097196
LOC_Os01g55350	LOC_Os05g33410	10.803824
LOC_Os01g55350	LOC_Os09g36710	9.0395351
LOC_Os01g55540	LOC_Os04g33720	9.04861943
LOC_Os01g55030	LOC_Os04g47360	9.25695953
LOC_Os01g55240	LOC_Os03g55874	9.3011545
LOC_Os01g55350	LOC_Os06g40190	9.59217938
LOC_Os01g55350	LOC_Os04g53290	9.00843452
LOC_Os01g55830	LOC_Os03g07150	9.26407493
LOC_Os01g55350	LOC_Os11g19220	8.90404017
LOC_Os01g55540	LOC_Os03g09910	10.1783266
LOC_Os01g55240	LOC_Os09g07830	9.1387026
LOC_Os01g55830	LOC_Os02g52390	9.02115314
LOC_Os01g55350	LOC_Os04g57410	9.40445954
LOC_Os01g55350	LOC_Os08g28190	9.47966079
LOC_Os01g55870	LOC_Os02g56460	9.88051706
LOC_Os01g55540	LOC_Os05g41640	8.83641624
LOC_Os01g55350	LOC_Os03g15120	10.9604685
LOC_Os01g55030	LOC_Os07g37790	11.6282349
LOC_Os01g55830	LOC_Os09g04050	8.81954782
LOC_Os01g55350	LOC_Os03g38980	8.89634804
LOC_Os01g55830	LOC_Os05g15520	9.80098746
LOC_Os01g55240	LOC_Os07g43170	9.52562508
LOC_Os01g55830	LOC_Os05g07690	8.92198863
LOC_Os01g55830	LOC_Os06g06320	8.89906305
LOC_Os01g55240	LOC_Os06g06090	9.67397904
LOC_Os01g55830	LOC_Os04g57410	8.89931744
LOC_Os01g55240	LOC_Os05g43820	9.12925816
LOC_Os01g55030	LOC_Os06g07080	9.25595424
LOC_Os01g55240	LOC_Os04g39900	9.05269702
LOC_Os01g55940	LOC_Os04g46910	9.28933619
LOC_Os01g56810	LOC_Os02g32030	9.74866719

InteractorA	InteractorB	Z score
LOC_Os01g56380	LOC_Os08g04560	23.3826016
LOC_Os01g55950	LOC_Os12g10560	9.36117666
LOC_Os01g55940	LOC_Os07g16970	9.43807723
LOC_Os01g55940	LOC_Os02g02960	8.80522725
LOC_Os01g56380	LOC_Os07g07770	10.0913821
LOC_Os01g56380	LOC_Os05g31140	9.04414714
LOC_Os01g56380	LOC_Os05g43510	19.1339869
LOC_Os01g55950	LOC_Os02g42810	8.92149225
LOC_Os01g55870	LOC_Os08g02700	9.09949323
LOC_Os01g56810	LOC_Os01g70140	9.16748675
LOC_Os01g56380	LOC_Os02g35760	9.30064175
LOC_Os01g56810	LOC_Os05g25550	9.00682647
LOC_Os01g55950	LOC_Os04g41960	10.1146534
LOC_Os01g55950	LOC_Os04g05080	9.34447338
LOC_Os01g55940	LOC_Os09g23560	9.38351554
LOC_Os01g55950	LOC_Os10g27050	9.22000883
LOC_Os01g55940	LOC_Os06g42560	9.37863349
LOC_Os01g55950	LOC_Os03g26450	8.81402843
LOC_Os01g56810	LOC_Os03g27310	9.48000467
LOC_Os01g55950	LOC_Os06g19960	9.13804908
LOC_Os01g55940	LOC_Os12g25690	9.38465798
LOC_Os01g55940	LOC_Os11g32510	9.80886097
LOC_Os01g56380	LOC_Os02g40830	10.0590071
LOC_Os01g55940	LOC_Os02g51100	9.45554791
LOC_Os01g55940	LOC_Os07g44790	8.97637459
LOC_Os01g56380	LOC_Os04g19740	8.85614881
LOC_Os01g55940	LOC_Os12g13380	9.31088891
LOC_Os01g56380	LOC_Os10g26110	18.4719136
LOC_Os01g55950	LOC_Os08g15030	8.80161551
LOC_Os01g55940	LOC_Os07g03710	8.9766125
LOC_Os01g56380	LOC_Os07g35940	8.91070014
LOC_Os01g56380	LOC_Os09g27820	9.36294782
LOC_Os01g56810	LOC_Os06g02144	9.57228015
LOC_Os01g56380	LOC_Os01g63270	10.6467421
LOC_Os01g55950	LOC_Os01g57770	9.63680701
LOC_Os01g55940	LOC_Os06g42130	9.01464991
LOC_Os01g55950	LOC_Os03g18560	8.87075856
LOC_Os01g56510	LOC_Os07g02210	9.97195394
LOC_Os01g55870	LOC_Os05g19670	8.90494885
LOC_Os01g55940	LOC_Os08g25570	9.39187252
LOC_Os01g56510	LOC_Os07g39290	8.82400659
LOC_Os01g56380	LOC_Os02g30630	8.92631956
LOC_Os01g56510	LOC_Os04g22730	8.8056439

InteractorA	InteractorB	Z score
LOC_Os01g56810	LOC_Os02g51830	9.68426208
LOC_Os01g55940	LOC_Os06g03770	8.91044092
LOC_Os01g56810	LOC_Os03g27260	8.86958803
LOC_Os01g56810	LOC_Os02g02560	8.9233768
LOC_Os01g55940	LOC_Os03g28400	8.9763637
LOC_Os01g55950	LOC_Os06g05250	9.59842013
LOC_Os01g55950	LOC_Os02g57260	8.87357928
LOC_Os01g55950	LOC_Os02g47600	9.0786326
LOC_Os01g55950	LOC_Os04g47170	8.82688422
LOC_Os01g56380	LOC_Os04g33480	9.28716289
LOC_Os01g55950	LOC_Os05g09500	8.94965444
LOC_Os01g56380	LOC_Os03g41438	9.03237062
LOC_Os01g55950	LOC_Os02g57040	9.14999421
LOC_Os01g56380	LOC_Os04g45290	8.8842995
LOC_Os01g56380	LOC_Os08g04540	20.6787374
LOC_Os01g55870	LOC_Os08g29170	8.82215607
LOC_Os01g55950	LOC_Os09g10270	9.85689985
LOC_Os01g55940	LOC_Os03g47610	8.85897405
LOC_Os01g55940	LOC_Os12g22650	9.90222854
LOC_Os01g55940	LOC_Os12g44150	9.919232
LOC_Os01g55940	LOC_Os03g06200	8.81743616
LOC_Os01g56510	LOC_Os07g38430	8.97687906
LOC_Os01g56510	LOC_Os04g53800	8.94612705
LOC_Os01g56510	LOC_Os01g64850	8.91457892
LOC_Os01g55870	LOC_Os05g51480	9.41229376
LOC_Os01g55940	LOC_Os01g57942	8.91423657
LOC_Os01g55940	LOC_Os05g05620	11.0955822
LOC_Os01g56380	LOC_Os04g24520	9.20767765
LOC_Os01g55950	LOC_Os10g39170	9.14548193
LOC_Os01g55870	LOC_Os06g35530	9.05366446
LOC_Os01g56510	LOC_Os04g57410	9.81583906
LOC_Os01g56510	LOC_Os05g43510	9.20045748
LOC_Os01g56380	LOC_Os04g37500	9.74669351
LOC_Os01g55950	LOC_Os04g01600	9.27306257
LOC_Os01g55940	LOC_Os04g48700	9.17659325
LOC_Os01g56380	LOC_Os09g19560	10.0117172
LOC_Os01g56380	LOC_Os02g41590	9.16251192
LOC_Os01g55940	LOC_Os11g08470	9.06855758
LOC_Os01g56380	LOC_Os04g30420	10.3361828
LOC_Os01g55950	LOC_Os03g11990	8.81080696
LOC_Os01g56380	LOC_Os09g32640	10.1191805
LOC_Os01g56810	LOC_Os05g46860	9.07733696
LOC_Os01g55950	LOC_Os03g04410	9.51296035

InteractorA	InteractorB	Z score
LOC_Os01g55950	LOC_Os04g12710	9.94918821
LOC_Os01g56510	LOC_Os04g41310	9.89289712
LOC_Os01g55940	LOC_Os05g49760	9.08667149
LOC_Os01g55940	LOC_Os02g35760	8.85019316
LOC_Os01g56380	LOC_Os03g44484	9.35409623
LOC_Os01g55940	LOC_Os03g56790	9.1386008
LOC_Os01g55870	LOC_Os04g58710	9.28304298
LOC_Os01g55940	LOC_Os05g19670	9.68981268
LOC_Os01g56380	LOC_Os03g05590	9.64575826
LOC_Os01g55870	LOC_Os12g12514	9.36292355
LOC_Os01g55940	LOC_Os03g60620	8.92612643
LOC_Os01g56380	LOC_Os04g58200	11.4472576
LOC_Os01g56510	LOC_Os10g27190	9.7754735
LOC_Os01g56380	LOC_Os06g05250	9.02868266
LOC_Os01g55950	LOC_Os11g25330	9.60000055
LOC_Os01g55950	LOC_Os12g16250	8.9457238
LOC_Os01g56380	LOC_Os03g44150	8.95794574
LOC_Os01g55940	LOC_Os04g16846	8.91423657
LOC_Os01g55870	LOC_Os04g28990	9.08896612
LOC_Os01g55950	LOC_Os02g51100	9.34029431
LOC_Os01g56380	LOC_Os11g31620	9.60832689
LOC_Os01g56510	LOC_Os01g73680	10.088856
LOC_Os01g55940	LOC_Os01g73580	10.1794542
LOC_Os01g55950	LOC_Os03g51200	10.6361644
LOC_Os01g55940	LOC_Os07g44370	9.13700116
LOC_Os01g56510	LOC_Os05g47640	10.4784623
LOC_Os01g56510	LOC_Os11g47550	9.01813525
LOC_Os01g56380	LOC_Os09g36830	8.89499972
LOC_Os01g56510	LOC_Os06g51150	10.0922178
LOC_Os01g56810	LOC_Os02g36400	8.85788312
LOC_Os01g56510	LOC_Os04g38870	9.19294387
LOC_Os01g55940	LOC_Os05g34770	9.66326533
LOC_Os01g55870	LOC_Os05g45220	9.77074205
LOC_Os01g56510	LOC_Os06g35530	8.95394314
LOC_Os01g56380	LOC_Os10g23900	17.2113134
LOC_Os01g55950	LOC_Os12g44020	8.97800602
LOC_Os01g55870	LOC_Os09g10260	11.5539359
LOC_Os01g56510	LOC_Os03g13150	9.07434224
LOC_Os01g55940	LOC_Os10g38229	9.88354308
LOC_Os01g55950	LOC_Os04g01470	9.07653868
LOC_Os01g56380	LOC_Os11g47550	9.06366212
LOC_Os01g55870	LOC_Os05g11730	9.34480731
LOC_Os01g55940	LOC_Os10g21324	8.91423657

InteractorA	InteractorB	Z score
LOC_Os01g55870	LOC_Os10g21240	9.09358266
LOC_Os01g56380	LOC_Os04g29550	8.94635071
LOC_Os01g56510	LOC_Os02g01220	8.97500766
LOC_Os01g55940	LOC_Os02g52230	9.08202642
LOC_Os01g55940	LOC_Os03g48760	9.25293901
LOC_Os01g55950	LOC_Os05g51630	8.83872288
LOC_Os01g55870	LOC_Os04g40290	9.71371188
LOC_Os01g55940	LOC_Os08g20420	10.1354216
LOC_Os01g56380	LOC_Os07g25590	14.167794
LOC_Os01g55950	LOC_Os07g49220	10.0781785
LOC_Os01g55870	LOC_Os07g30200	9.01966477
LOC_Os01g56380	LOC_Os12g12560	9.49781645
LOC_Os01g55940	LOC_Os10g26130	9.20049769
LOC_Os01g55940	LOC_Os05g33150	8.86167455
LOC_Os01g55950	LOC_Os03g13160	8.92787766
LOC_Os01g55870	LOC_Os11g34570	9.70527067
LOC_Os01g55940	LOC_Os11g08445	8.97988722
LOC_Os01g56880	LOC_Os02g30200	9.56135638
LOC_Os01g57610	LOC_Os08g32620	9.33082222
LOC_Os01g57610	LOC_Os02g01920	9.00901313
LOC_Os01g56880	LOC_Os01g73790	9.26570439
LOC_Os01g57610	LOC_Os06g11240	10.4811654
LOC_Os01g57610	LOC_Os06g05700	9.14574412
LOC_Os01g56880	LOC_Os05g33730	10.4497177
LOC_Os01g56880	LOC_Os06g11290	9.42513126
LOC_Os01g56810	LOC_Os08g23730	8.84838321
LOC_Os01g57942	LOC_Os04g37640	9.07180094
LOC_Os01g57770	LOC_Os07g38860	9.2327312
LOC_Os01g56810	LOC_Os10g26010	8.85542083
LOC_Os01g56880	LOC_Os02g44080	10.912535
LOC_Os01g56880	LOC_Os06g43640	9.18410511
LOC_Os01g57610	LOC_Os05g38550	9.54706407
LOC_Os01g57770	LOC_Os08g40140	8.86033058
LOC_Os01g57610	LOC_Os05g44140	11.8057851
LOC_Os01g56810	LOC_Os09g34214	9.16737573
LOC_Os01g57570	LOC_Os09g30360	9.43780449
LOC_Os01g57770	LOC_Os03g40330	9.00136724
LOC_Os01g56880	LOC_Os04g32460	9.02471998
LOC_Os01g57610	LOC_Os07g22930	8.87385753
LOC_Os01g57570	LOC_Os06g05250	9.71983384
LOC_Os01g56880	LOC_Os02g32490	9.41570088
LOC_Os01g57942	LOC_Os04g58710	8.99053063
LOC_Os01g57770	LOC_Os04g02820	10.1595737

InteractorA	InteractorB	Z score
LOC_Os01g57610	LOC_Os04g39900	9.52953299
LOC_Os01g57570	LOC_Os09g04050	9.1319547
LOC_Os01g56880	LOC_Os08g27840	8.82386246
LOC_Os01g56880	LOC_Os05g09500	10.6662681
LOC_Os01g56810	LOC_Os09g04680	8.99849217
LOC_Os01g56880	LOC_Os02g41590	9.04755002
LOC_Os01g57610	LOC_Os05g09490	8.86520024
LOC_Os01g57610	LOC_Os06g06090	8.98701487
LOC_Os01g57770	LOC_Os09g10230	9.61565602
LOC_Os01g57770	LOC_Os03g49610	9.10919164
LOC_Os01g57770	LOC_Os02g15620	9.28684718
LOC_Os01g57610	LOC_Os04g01470	9.49837262
LOC_Os01g57610	LOC_Os04g40990	9.48580942
LOC_Os01g57610	LOC_Os09g07830	9.34513059
LOC_Os01g57610	LOC_Os02g47800	8.88842643
LOC_Os01g57610	LOC_Os02g51830	9.60640336
LOC_Os01g57610	LOC_Os06g27770	10.2148028
LOC_Os01g57942	LOC_Os08g35440	8.97470838
LOC_Os01g57610	LOC_Os01g73680	8.87069552
LOC_Os01g57942	LOC_Os04g33740	9.10258883
LOC_Os01g57770	LOC_Os06g37560	8.90450787
LOC_Os01g56880	LOC_Os06g35650	8.997851
LOC_Os01g57610	LOC_Os04g12950	10.2792472
LOC_Os01g57610	LOC_Os06g22140	9.16261431
LOC_Os01g56880	LOC_Os11g31530	9.47352514
LOC_Os01g57770	LOC_Os03g04410	8.91542128
LOC_Os01g56880	LOC_Os04g09540	8.96134389
LOC_Os01g57610	LOC_Os03g53650	10.0871325
LOC_Os01g57570	LOC_Os01g59530	10.7809917
LOC_Os01g57610	LOC_Os02g24354	9.3978077
LOC_Os01g56880	LOC_Os06g43660	8.96103066
LOC_Os01g57610	LOC_Os03g18580	9.16491427
LOC_Os01g57570	LOC_Os08g16910	9.50729627
LOC_Os01g57610	LOC_Os05g07720	8.90652531
LOC_Os01g57610	LOC_Os03g26450	9.31358685
LOC_Os01g57570	LOC_Os07g23730	11.1632871
LOC_Os01g57570	LOC_Os09g20090	8.83293206
LOC_Os01g57610	LOC_Os04g27980	8.89229351
LOC_Os01g56880	LOC_Os12g21798	10.8274758
LOC_Os01g57942	LOC_Os05g05800	9.35768804
LOC_Os01g56880	LOC_Os01g73580	8.84525844
LOC_Os01g56810	LOC_Os06g04030	9.48002156
LOC_Os01g57610	LOC_Os06g15420	9.35223528

InteractorA	InteractorB	Z score
LOC_Os01g57570	LOC_Os01g62230	10.1243853
LOC_Os01g57610	LOC_Os09g34960	8.94464669
LOC_Os01g56880	LOC_Os05g50710	9.87697477
LOC_Os01g57770	LOC_Os12g40510	9.25607211
LOC_Os01g57610	LOC_Os03g60620	9.17971727
LOC_Os01g56810	LOC_Os10g38470	9.26656367
LOC_Os01g57770	LOC_Os05g26890	9.5117362
LOC_Os01g57770	LOC_Os05g44140	9.64129415
LOC_Os01g56880	LOC_Os05g41080	9.18277629
LOC_Os01g56880	LOC_Os07g47490	10.2853599
LOC_Os01g56880	LOC_Os04g43760	9.84397739
LOC_Os01g56880	LOC_Os05g06970	8.80304714
LOC_Os01g57610	LOC_Os03g57120	9.08397236
LOC_Os01g57610	LOC_Os02g52420	9.08550143
LOC_Os01g57610	LOC_Os03g02920	9.21583389
LOC_Os01g57942	LOC_Os03g60740	8.82009055
LOC_Os01g56880	LOC_Os06g39875	9.29737655
LOC_Os01g57942	LOC_Os03g38000	9.92427819
LOC_Os01g56880	LOC_Os10g28360	9.25888453
LOC_Os01g57610	LOC_Os07g07719	9.42478509
LOC_Os01g57570	LOC_Os07g38430	9.44652801
LOC_Os01g56880	LOC_Os04g17650	9.54816323
LOC_Os01g57610	LOC_Os04g39440	9.63284712
LOC_Os01g57610	LOC_Os09g38030	9.06805886
LOC_Os01g56810	LOC_Os07g06970	8.98954547
LOC_Os01g57610	LOC_Os07g47420	8.925301
LOC_Os01g56880	LOC_Os03g17470	8.98499551
LOC_Os01g57770	LOC_Os12g38760	9.67904105
LOC_Os01g57942	LOC_Os07g48050	10.1211797
LOC_Os01g57942	LOC_Os03g32170	9.05253456
LOC_Os01g57942	LOC_Os04g12720	11.2828597
LOC_Os01g56880	LOC_Os06g06980	9.20058877
LOC_Os01g57610	LOC_Os08g20420	8.84937972
LOC_Os01g57770	LOC_Os03g04250	9.67748353
LOC_Os01g57610	LOC_Os03g05730	8.83383713
LOC_Os01g57610	LOC_Os06g45070	9.24208444
LOC_Os01g56880	LOC_Os06g29220	9.86178224
LOC_Os01g57610	LOC_Os03g04169	9.11793809
LOC_Os01g56880	LOC_Os07g14590	10.0069638
LOC_Os01g57770	LOC_Os05g28280	9.43848768
LOC_Os01g57945	LOC_Os06g04000	9.16868594
LOC_Os01g57964	LOC_Os07g38970	9.27438102
LOC_Os01g57956	LOC_Os02g50240	8.90161941

InteractorA	InteractorB	Z score
LOC_Os01g57945	LOC_Os06g22140	8.84040918
LOC_Os01g57962	LOC_Os08g25734	8.85489103
LOC_Os01g57958	LOC_Os06g45710	8.8838254
LOC_Os01g57945	LOC_Os12g08270	10.8243297
LOC_Os01g57962	LOC_Os05g23860	9.0062716
LOC_Os01g57958	LOC_Os05g48980	9.43078744
LOC_Os01g57945	LOC_Os05g27950	9.78725072
LOC_Os01g57962	LOC_Os05g37690	10.1203962
LOC_Os01g57958	LOC_Os09g27820	8.9161854
LOC_Os01g57964	LOC_Os10g38140	9.4849188
LOC_Os01g57945	LOC_Os10g01080	11.3283499
LOC_Os01g57964	LOC_Os03g11970	9.74929309
LOC_Os01g57945	LOC_Os04g54330	9.15876971
LOC_Os01g57956	LOC_Os03g49350	8.95880012
LOC_Os01g57945	LOC_Os03g50480	8.9194474
LOC_Os01g57964	LOC_Os07g07320	9.02968884
LOC_Os01g57962	LOC_Os06g35730	9.17643967
LOC_Os01g57962	LOC_Os02g35760	8.97615933
LOC_Os01g57962	LOC_Os05g11710	9.17643967
LOC_Os01g57962	LOC_Os04g02050	9.03795567
LOC_Os01g57958	LOC_Os05g02940	8.83541426
LOC_Os01g57962	LOC_Os10g28360	8.8125043
LOC_Os01g57956	LOC_Os02g51100	9.08766358
LOC_Os01g57964	LOC_Os07g42950	9.88948228
LOC_Os01g57958	LOC_Os03g59020	9.92048532
LOC_Os01g57964	LOC_Os08g29520	9.65931695
LOC_Os01g57958	LOC_Os05g49830	9.86322433
LOC_Os01g57945	LOC_Os02g04320	8.8334079
LOC_Os01g57964	LOC_Os03g01900	10.7313132
LOC_Os01g57958	LOC_Os04g56950	8.92029852
LOC_Os01g57945	LOC_Os07g07240	9.4357324
LOC_Os01g57964	LOC_Os06g07978	10.0349712
LOC_Os01g57958	LOC_Os02g57450	8.96820059
LOC_Os01g57964	LOC_Os06g51084	8.85485601
LOC_Os01g57958	LOC_Os04g17064	8.96226963
LOC_Os01g57964	LOC_Os02g52560	11.919849
LOC_Os01g57945	LOC_Os02g38920	8.85182696
LOC_Os01g57964	LOC_Os01g71310	8.97525615
LOC_Os01g57962	LOC_Os06g35540	8.9630795
LOC_Os01g57962	LOC_Os09g31486	11.0139428
LOC_Os01g57945	LOC_Os03g13180	10.6640352
LOC_Os01g57958	LOC_Os03g42110	8.82084294
LOC_Os01g57945	LOC_Os05g42350	8.87625392

InteractorA	InteractorB	Z score
LOC_Os01g57958	LOC_Os07g05160	9.11210747
LOC_Os01g57962	LOC_Os03g49610	8.97693796
LOC_Os01g57962	LOC_Os03g04410	10.1731706
LOC_Os01g57945	LOC_Os10g38740	9.65864361
LOC_Os01g57958	LOC_Os10g38274	8.82040254
LOC_Os01g57958	LOC_Os03g01650	9.71313015
LOC_Os01g57945	LOC_Os03g16860	9.33643247
LOC_Os01g57964	LOC_Os05g06970	9.7006403
LOC_Os01g57962	LOC_Os02g32490	8.81879136
LOC_Os01g57945	LOC_Os10g07616	9.08354113
LOC_Os01g57956	LOC_Os09g08072	9.19017966
LOC_Os01g57964	LOC_Os07g41750	9.0020077
LOC_Os01g57962	LOC_Os11g26860	9.50495914
LOC_Os01g57962	LOC_Os06g11800	9.0329241
LOC_Os01g57956	LOC_Os04g32460	8.80263928
LOC_Os01g57942	LOC_Os09g37949	9.275137
LOC_Os01g57958	LOC_Os03g01610	9.71313015
LOC_Os01g57964	LOC_Os10g25140	8.92441439
LOC_Os01g57956	LOC_Os07g07060	9.00713107
LOC_Os01g57964	LOC_Os04g43760	9.00385669
LOC_Os01g57958	LOC_Os10g38660	8.85371839
LOC_Os01g57964	LOC_Os06g07878	10.0349712
LOC_Os01g57964	LOC_Os09g10270	8.81894133
LOC_Os01g57945	LOC_Os10g10434	9.61143952
LOC_Os01g57964	LOC_Os10g38234	10.7294032
LOC_Os01g57962	LOC_Os12g44000	9.51205246
LOC_Os01g57945	LOC_Os03g09250	9.1650967
LOC_Os01g57945	LOC_Os05g33240	9.22941297
LOC_Os01g57945	LOC_Os06g09910	10.0710108
LOC_Os01g57945	LOC_Os06g05690	9.6508851
LOC_Os01g57956	LOC_Os07g38540	10.1302762
LOC_Os01g57962	LOC_Os04g43410	9.3997763
LOC_Os01g57958	LOC_Os10g21250	9.28042324
LOC_Os01g57945	LOC_Os03g11530	9.37063233
LOC_Os01g57956	LOC_Os07g35880	8.83894616
LOC_Os01g57962	LOC_Os02g36974	10.3910492
LOC_Os01g57962	LOC_Os07g38890	9.4217454
LOC_Os01g57964	LOC_Os09g20220	9.12305136
LOC_Os01g57964	LOC_Os02g14130	8.81976054
LOC_Os01g57958	LOC_Os10g28320	9.17356642
LOC_Os01g57962	LOC_Os12g02370	8.91066917
LOC_Os01g57958	LOC_Os04g45290	9.0051411
LOC_Os01g57956	LOC_Os03g17470	9.03620686

InteractorA	InteractorB	Z score
LOC_Os01g57958	LOC_Os10g21230	8.82040254
LOC_Os01g57962	LOC_Os07g38970	8.85354448
LOC_Os01g57962	LOC_Os02g47800	12.2482279
LOC_Os01g57964	LOC_Os07g44550	9.5856457
LOC_Os01g57958	LOC_Os03g10340	9.97127908
LOC_Os01g57964	LOC_Os06g35700	10.6247134
LOC_Os01g57942	LOC_Os12g12560	9.46659128
LOC_Os01g57964	LOC_Os06g05110	8.98996128
LOC_Os01g57945	LOC_Os04g56320	9.89083027
LOC_Os01g57964	LOC_Os09g36830	9.42672157
LOC_Os01g57956	LOC_Os10g21248	10.0520778
LOC_Os01g57945	LOC_Os03g07150	9.6634735
LOC_Os01g57958	LOC_Os02g55370	9.16387694
LOC_Os01g57945	LOC_Os12g25710	11.7484235
LOC_Os01g57962	LOC_Os05g05830	10.4242376
LOC_Os01g57945	LOC_Os02g02560	9.34767326
LOC_Os01g57956	LOC_Os12g38760	9.62755638
LOC_Os01g57964	LOC_Os02g14430	10.2929598
LOC_Os01g57962	LOC_Os01g62244	8.81555712
LOC_Os01g58610	LOC_Os05g05830	8.82725303
LOC_Os01g58730	LOC_Os02g27760	9.11612908
LOC_Os01g58020	LOC_Os07g42950	9.29232637
LOC_Os01g58380	LOC_Os02g07260	10.4217473
LOC_Os01g58020	LOC_Os04g27860	8.91730451
LOC_Os01g58730	LOC_Os07g22950	9.34811198
LOC_Os01g58380	LOC_Os03g15360	10.6713032
LOC_Os01g58220	LOC_Os08g38300	9.60177829
LOC_Os01g58730	LOC_Os03g17980	8.95352082
LOC_Os01g58610	LOC_Os03g44170	10.014371
LOC_Os01g58730	LOC_Os03g56280	9.14069785
LOC_Os01g58730	LOC_Os12g25690	8.91438067
LOC_Os01g58020	LOC_Os09g15320	9.50337472
LOC_Os01g58020	LOC_Os11g32260	8.99125062
LOC_Os01g58730	LOC_Os04g12980	9.34234758
LOC_Os01g58610	LOC_Os08g09950	8.90525272
LOC_Os01g58730	LOC_Os02g01920	9.00304869
LOC_Os01g58020	LOC_Os02g30200	10.2178693
LOC_Os01g58730	LOC_Os10g35840	9.82968732
LOC_Os01g58380	LOC_Os10g35480	9.00109949
LOC_Os01g59150	LOC_Os03g45320	9.36435485
LOC_Os01g58380	LOC_Os09g32840	8.93880437
LOC_Os01g58610	LOC_Os08g29170	8.81360849
LOC_Os01g58610	LOC_Os12g10730	9.48697311

InteractorA	InteractorB	Z score
LOC_Os01g58380	LOC_Os06g29180	9.13734257
LOC_Os01g58730	LOC_Os07g44430	8.91246961
LOC_Os01g58220	LOC_Os04g45290	9.10665005
LOC_Os01g58380	LOC_Os03g13180	9.14157094
LOC_Os01g58730	LOC_Os11g08330	9.66279299
LOC_Os01g58380	LOC_Os02g47790	9.95676008
LOC_Os01g58380	LOC_Os03g32170	9.83256682
LOC_Os01g58020	LOC_Os03g63410	8.80277962
LOC_Os01g58380	LOC_Os04g58710	9.17288187
LOC_Os01g58020	LOC_Os02g10390	10.0243569
LOC_Os01g58730	LOC_Os08g02400	11.5231814
LOC_Os01g58730	LOC_Os05g37390	8.80802102
LOC_Os01g58730	LOC_Os12g40830	9.05587784
LOC_Os01g58020	LOC_Os12g31640	9.23915043
LOC_Os01g58730	LOC_Os06g44620	9.47323248
LOC_Os01g58020	LOC_Os10g21268	15.0941818
LOC_Os01g58020	LOC_Os04g24430	10.5124613
LOC_Os01g58020	LOC_Os10g32550	8.88786754
LOC_Os01g58730	LOC_Os07g06970	8.95494878
LOC_Os01g58380	LOC_Os10g39120	8.85983583
LOC_Os01g58380	LOC_Os05g49770	9.31495282
LOC_Os01g58730	LOC_Os04g49130	9.38124599
LOC_Os01g58220	LOC_Os02g18880	8.96659224
LOC_Os01g59150	LOC_Os01g64680	8.89590307
LOC_Os01g58020	LOC_Os11g03290	9.58652964
LOC_Os01g59150	LOC_Os02g51930	10.0015818
LOC_Os01g58610	LOC_Os06g51150	8.82979652
LOC_Os01g58020	LOC_Os10g26130	9.21134016
LOC_Os01g58020	LOC_Os05g28280	9.78702304
LOC_Os01g58220	LOC_Os01g64850	9.08360727
LOC_Os01g58380	LOC_Os09g10300	9.54608561
LOC_Os01g58730	LOC_Os03g08570	9.35275722
LOC_Os01g58730	LOC_Os06g09450	8.96536482
LOC_Os01g58730	LOC_Os04g24520	10.1001953
LOC_Os01g58020	LOC_Os07g43470	9.86212499
LOC_Os01g58610	LOC_Os08g38300	8.97740458
LOC_Os01g58730	LOC_Os09g39400	9.0494092
LOC_Os01g58220	LOC_Os03g58530	9.38729108
LOC_Os01g58380	LOC_Os10g34520	11.1043458
LOC_Os01g58380	LOC_Os07g20544	9.07658706
LOC_Os01g58730	LOC_Os10g21266	11.046673
LOC_Os01g58610	LOC_Os01g73220	11.6423096
LOC_Os01g58730	LOC_Os06g06050	9.51970416

InteractorA	InteractorB	Z score
LOC_Os01g58730	LOC_Os06g14510	9.50190458
LOC_Os01g58730	LOC_Os09g32840	9.76880875
LOC_Os01g58020	LOC_Os10g09860	9.0982956
LOC_Os01g58380	LOC_Os02g35500	10.4691253
LOC_Os01g58380	LOC_Os05g33140	8.97085242
LOC_Os01g58380	LOC_Os02g53790	9.27447768
LOC_Os01g58380	LOC_Os03g18740	8.99284242
LOC_Os01g58610	LOC_Os03g25360	10.3854061
LOC_Os01g58610	LOC_Os04g46930	9.68745291
LOC_Os01g58380	LOC_Os08g43190	9.78328694
LOC_Os01g58020	LOC_Os04g38220	9.35844802
LOC_Os01g58730	LOC_Os01g73140	9.13467061
LOC_Os01g58730	LOC_Os07g05940	9.54548829
LOC_Os01g58610	LOC_Os08g28800	9.09015778
LOC_Os01g58020	LOC_Os02g46970	10.7945882
LOC_Os01g58380	LOC_Os02g17390	8.84276074
LOC_Os01g58020	LOC_Os09g28460	9.09103268
LOC_Os01g58610	LOC_Os03g21460	9.0409973
LOC_Os01g58380	LOC_Os09g34960	8.91644246
LOC_Os01g58380	LOC_Os10g29470	9.30052583
LOC_Os01g58020	LOC_Os06g12090	9.2253715
LOC_Os01g58020	LOC_Os05g35330	15.0941818
LOC_Os01g59340	LOC_Os12g13390	9.01496972
LOC_Os01g59530	LOC_Os01g70220	9.98374801
LOC_Os01g59600	LOC_Os02g24632	9.27559624
LOC_Os01g59530	LOC_Os08g16910	8.8920039
LOC_Os01g59490	LOC_Os09g28400	10.5067126
LOC_Os01g59440	LOC_Os04g46910	9.16021026
LOC_Os01g59490	LOC_Os11g02440	9.92539204
LOC_Os01g59530	LOC_Os05g33240	8.89441465
LOC_Os01g59340	LOC_Os12g02370	9.97403504
LOC_Os01g59490	LOC_Os12g07720	10.1811582
LOC_Os01g59530	LOC_Os05g45590	11.0061095
LOC_Os01g59600	LOC_Os03g58400	9.19768116
LOC_Os01g59340	LOC_Os07g41750	10.2385072
LOC_Os01g59340	LOC_Os10g03540	9.09244326
LOC_Os01g59490	LOC_Os12g36950	9.03406221
LOC_Os01g59530	LOC_Os07g08880	9.37746336
LOC_Os01g59340	LOC_Os02g07160	9.15531122
LOC_Os01g59530	LOC_Os06g09450	9.46011355
LOC_Os01g59150	LOC_Os12g13390	8.83840407
LOC_Os01g59490	LOC_Os10g41689	9.47385974
LOC_Os01g59340	LOC_Os05g02310	9.38590642

InteractorA	InteractorB	Z score
LOC_Os01g59150	LOC_Os08g06610	8.87366569
LOC_Os01g59530	LOC_Os03g42840	9.10980293
LOC_Os01g59490	LOC_Os11g32580	9.04656494
LOC_Os01g59490	LOC_Os01g73580	9.21345326
LOC_Os01g59440	LOC_Os08g35740	8.99743988
LOC_Os01g59440	LOC_Os12g39630	9.70118111
LOC_Os01g59490	LOC_Os05g11550	8.94208873
LOC_Os01g59530	LOC_Os02g01880	8.8733979
LOC_Os01g59490	LOC_Os02g24634	11.169517
LOC_Os01g59490	LOC_Os11g08340	9.64445888
LOC_Os01g59530	LOC_Os05g40420	9.04451964
LOC_Os01g59530	LOC_Os04g45290	9.5005004
LOC_Os01g59490	LOC_Os06g06980	9.3766534
LOC_Os01g59340	LOC_Os05g37690	8.86475126
LOC_Os01g59150	LOC_Os11g14220	8.94623981
LOC_Os01g59150	LOC_Os12g31640	9.42649145
LOC_Os01g59600	LOC_Os04g55410	9.84675317
LOC_Os01g59150	LOC_Os08g38900	8.8440629
LOC_Os01g59340	LOC_Os02g14929	8.83274443
LOC_Os01g59530	LOC_Os06g36160	8.89328298
LOC_Os01g59490	LOC_Os10g31950	9.60949377
LOC_Os01g59490	LOC_Os10g38140	9.7104394
LOC_Os01g59530	LOC_Os08g15292	9.6340191
LOC_Os01g59440	LOC_Os02g08490	9.36063816
LOC_Os01g59340	LOC_Os05g25550	9.24462498
LOC_Os01g59440	LOC_Os08g44810	9.99978745
LOC_Os01g59440	LOC_Os08g03040	9.10553969
LOC_Os01g59490	LOC_Os09g31502	8.81502043
LOC_Os01g59340	LOC_Os06g04000	8.8437446
LOC_Os01g59340	LOC_Os05g51670	9.97656235
LOC_Os01g59340	LOC_Os02g47610	9.09771816
LOC_Os01g59530	LOC_Os06g11210	9.96430752
LOC_Os01g59340	LOC_Os02g08490	9.56088346
LOC_Os01g59150	LOC_Os05g45590	8.89230734
LOC_Os01g59150	LOC_Os07g49220	9.42285786
LOC_Os01g59150	LOC_Os12g12560	8.818634
LOC_Os01g59530	LOC_Os09g31486	11.0920473
LOC_Os01g59600	LOC_Os02g41670	9.5313425
LOC_Os01g59340	LOC_Os02g12780	10.0207134
LOC_Os01g59440	LOC_Os03g42840	9.03505255
LOC_Os01g59530	LOC_Os10g26390	9.36094829
LOC_Os01g59490	LOC_Os07g38910	9.23139164
LOC_Os01g59150	LOC_Os07g47490	9.00822035

InteractorA	InteractorB	Z score
LOC_Os01g59530	LOC_Os11g24560	8.8019662
LOC_Os01g59600	LOC_Os05g28180	8.88921572
LOC_Os01g59490	LOC_Os03g16900	9.36109734
LOC_Os01g59530	LOC_Os04g40990	8.87957526
LOC_Os01g59150	LOC_Os08g28820	9.34827822
LOC_Os01g59530	LOC_Os03g16860	10.2593048
LOC_Os01g59440	LOC_Os10g08580	9.5564202
LOC_Os01g59340	LOC_Os03g63410	9.95286025
LOC_Os01g59490	LOC_Os06g39708	8.82036186
LOC_Os01g59490	LOC_Os04g12710	9.3007784
LOC_Os01g59440	LOC_Os05g46040	8.802078
LOC_Os01g59530	LOC_Os05g35290	9.27700694
LOC_Os01g59530	LOC_Os11g08340	9.1870672
LOC_Os01g59150	LOC_Os06g37500	9.44325817
LOC_Os01g59340	LOC_Os04g56730	9.24458518
LOC_Os01g59490	LOC_Os09g36800	8.93281374
LOC_Os01g59490	LOC_Os02g46970	8.85728937
LOC_Os01g59340	LOC_Os05g40420	9.16301907
LOC_Os01g59340	LOC_Os03g39610	8.9412982
LOC_Os01g59530	LOC_Os03g15120	9.38355648
LOC_Os01g59930	LOC_Os01g71474	9.5440086
LOC_Os01g60190	LOC_Os02g51100	9.31314713
LOC_Os01g60190	LOC_Os11g31530	9.51318836
LOC_Os01g59600	LOC_Os10g38730	9.43798792
LOC_Os01g60360	LOC_Os07g35940	10.3544763
LOC_Os01g60190	LOC_Os02g24354	9.01123495
LOC_Os01g59790	LOC_Os12g34062	10.4639648
LOC_Os01g59790	LOC_Os04g09604	9.17225876
LOC_Os01g59730	LOC_Os12g25630	10.1880796
LOC_Os01g60360	LOC_Os07g48880	8.97926199
LOC_Os01g60190	LOC_Os05g07720	10.6704253
LOC_Os01g60190	LOC_Os12g34380	9.50782564
LOC_Os01g60360	LOC_Os05g45220	9.00947998
LOC_Os01g60190	LOC_Os05g47980	8.87670541
LOC_Os01g60190	LOC_Os04g57090	9.35553399
LOC_Os01g59930	LOC_Os10g32680	9.11482248
LOC_Os01g60190	LOC_Os05g34770	8.86121671
LOC_Os01g59930	LOC_Os04g52280	8.84480291
LOC_Os01g59790	LOC_Os07g48020	8.87900286
LOC_Os01g59790	LOC_Os03g42840	9.03403754
LOC_Os01g59790	LOC_Os08g44960	9.7831611
LOC_Os01g59930	LOC_Os03g48750	9.68878232
LOC_Os01g60190	LOC_Os08g28730	9.37397914

InteractorA	InteractorB	Z score
LOC_Os01g60190	LOC_Os05g28180	8.84490639
LOC_Os01g60190	LOC_Os02g41860	9.50672726
LOC_Os01g59790	LOC_Os02g44550	8.83202635
LOC_Os01g59790	LOC_Os07g48030	8.826504
LOC_Os01g60190	LOC_Os04g39210	8.80040015
LOC_Os01g59930	LOC_Os03g56790	9.14305807
LOC_Os01g60360	LOC_Os05g38760	9.26317461
LOC_Os01g60360	LOC_Os04g40130	10.3194617
LOC_Os01g60190	LOC_Os12g43100	9.06950458
LOC_Os01g59790	LOC_Os09g31502	8.90839109
LOC_Os01g60190	LOC_Os04g01674	9.11744822
LOC_Os01g59790	LOC_Os11g47600	8.96342241
LOC_Os01g60190	LOC_Os12g06660	8.91124697
LOC_Os01g59730	LOC_Os11g16590	9.61370666
LOC_Os01g60190	LOC_Os10g39840	9.42154634
LOC_Os01g59930	LOC_Os09g23560	9.22661691
LOC_Os01g59790	LOC_Os02g10320	9.07989146
LOC_Os01g59790	LOC_Os08g32620	8.8151567
LOC_Os01g60360	LOC_Os09g17740	9.29789953
LOC_Os01g59730	LOC_Os09g04050	9.46363944
LOC_Os01g59600	LOC_Os11g40150	8.84387982
LOC_Os01g59930	LOC_Os10g31940	9.59755243
LOC_Os01g59730	LOC_Os03g59710	9.02938964
LOC_Os01g59600	LOC_Os12g23170	9.83164301
LOC_Os01g60190	LOC_Os04g58580	9.09215633
LOC_Os01g59730	LOC_Os04g18650	9.23700246
LOC_Os01g59730	LOC_Os07g48430	9.04599746
LOC_Os01g59730	LOC_Os03g40720	9.93683356
LOC_Os01g60360	LOC_Os09g24530	9.40584263
LOC_Os01g59930	LOC_Os04g40874	10.2327133
LOC_Os01g60190	LOC_Os10g41550	8.86831341
LOC_Os01g60190	LOC_Os07g07470	9.64895299
LOC_Os01g59930	LOC_Os04g16680	8.85221155
LOC_Os01g60190	LOC_Os03g64330	8.85384911
LOC_Os01g59790	LOC_Os12g05590	8.9192062
LOC_Os01g60190	LOC_Os03g63090	10.6302254
LOC_Os01g60360	LOC_Os06g35730	9.571816
LOC_Os01g59790	LOC_Os01g74000	9.19590871
LOC_Os01g60190	LOC_Os02g35590	9.78684994
LOC_Os01g59930	LOC_Os10g29470	8.94624518
LOC_Os01g59730	LOC_Os07g43260	10.039205
LOC_Os01g60360	LOC_Os03g62700	8.9622374
LOC_Os01g59790	LOC_Os02g14460	8.93975158

InteractorA	InteractorB	Z score
LOC_Os01g59930	LOC_Os02g47790	9.0896305
LOC_Os01g60190	LOC_Os02g07720	9.68833021
LOC_Os01g59730	LOC_Os10g28080	9.32548505
LOC_Os01g60190	LOC_Os06g51050	9.43789808
LOC_Os01g60360	LOC_Os05g11710	9.571816
LOC_Os01g59730	LOC_Os05g01810	8.82661913
LOC_Os01g59790	LOC_Os10g28080	9.02191582
LOC_Os01g59790	LOC_Os12g25120	9.1117378
LOC_Os01g59930	LOC_Os02g14059	12.3118937
LOC_Os01g60190	LOC_Os02g04950	10.3168524
LOC_Os01g60190	LOC_Os07g49120	9.09758218
LOC_Os01g60360	LOC_Os09g15420	8.89450184
LOC_Os01g59730	LOC_Os10g40720	8.94187906
LOC_Os01g59930	LOC_Os03g38980	9.63591351
LOC_Os01g59930	LOC_Os07g05400	9.10873592
LOC_Os01g60190	LOC_Os07g20544	8.99015466
LOC_Os01g60190	LOC_Os02g01590	9.15361537
LOC_Os01g60190	LOC_Os11g03290	11.193305
LOC_Os01g59930	LOC_Os07g47990	9.01189127
LOC_Os01g60190	LOC_Os06g11260	9.07541225
LOC_Os01g59730	LOC_Os02g12730	8.83602564
LOC_Os01g59730	LOC_Os03g53800	8.98018013
LOC_Os01g59600	LOC_Os09g10300	9.54536069
LOC_Os01g59930	LOC_Os06g23760	11.0767867
LOC_Os01g59930	LOC_Os08g09210	9.09631411
LOC_Os01g59790	LOC_Os09g04730	11.0338068
LOC_Os01g59790	LOC_Os10g21212	10.8894531
LOC_Os01g60360	LOC_Os02g56690	9.2848981
LOC_Os01g59600	LOC_Os10g38580	9.07922708
LOC_Os01g59730	LOC_Os10g27174	9.13165305
LOC_Os01g60190	LOC_Os12g41110	9.89527884
LOC_Os01g59730	LOC_Os07g06970	8.92851381
LOC_Os01g60190	LOC_Os08g29370	9.25399632
LOC_Os01g60190	LOC_Os04g01740	9.14044792
LOC_Os01g60190	LOC_Os08g37800	8.94208315
LOC_Os01g05630	LOC_Os03g58320	9.25769182
LOC_Os01g05630	LOC_Os10g02480	8.84293211
LOC_Os01g05970	LOC_Os05g24580	8.82531415
LOC_Os01g05610	LOC_Os03g51080	8.82108033
LOC_Os01g05630	LOC_Os02g51830	9.35473347
LOC_Os01g05970	LOC_Os03g04410	8.8951123
LOC_Os01g05610	LOC_Os04g45470	9.91306768
LOC_Os01g05630	LOC_Os01g71270	8.99955853

InteractorA	InteractorB	Z score
LOC_Os01g05970	LOC_Os01g08200	9.06134356
LOC_Os01g05630	LOC_Os04g33480	10.240557
LOC_Os01g06010	LOC_Os01g71670	10.0810156
LOC_Os01g05610	LOC_Os05g12180	9.20235476
LOC_Os01g05610	LOC_Os04g59150	9.52252643
LOC_Os01g06010	LOC_Os05g31020	10.1945158
LOC_Os01g05970	LOC_Os01g49720	9.50612675
LOC_Os01g05490	LOC_Os12g04980	9.83294661
LOC_Os01g05490	LOC_Os11g32260	11.2229524
LOC_Os01g05630	LOC_Os10g22070	8.90021849
LOC_Os01g05490	LOC_Os03g41438	8.87495766
LOC_Os01g05610	LOC_Os07g48030	8.80365069
LOC_Os01g05970	LOC_Os07g30990	9.43452592
LOC_Os01g05970	LOC_Os03g13170	10.8314555
LOC_Os01g06010	LOC_Os01g16960	9.93786845
LOC_Os01g05610	LOC_Os03g24380	8.93172368
LOC_Os01g06010	LOC_Os03g16110	9.3170347
LOC_Os01g05970	LOC_Os07g47420	9.68806534
LOC_Os01g05610	LOC_Os03g37950	10.5093686
LOC_Os01g05610	LOC_Os04g54390	9.44590974
LOC_Os01g05970	LOC_Os08g44210	8.80738791
LOC_Os01g06010	LOC_Os03g09810	10.009788
LOC_Os01g05970	LOC_Os01g45400	10.1519758
LOC_Os01g05630	LOC_Os03g01630	8.84693352
LOC_Os01g05490	LOC_Os02g56700	8.97535935
LOC_Os01g05630	LOC_Os01g60790	11.144667
LOC_Os01g05610	LOC_Os09g28420	9.38003573
LOC_Os01g05610	LOC_Os12g38770	8.83321925
LOC_Os01g05610	LOC_Os04g52130	10.205334
LOC_Os01g05970	LOC_Os10g38360	11.6168498
LOC_Os01g05970	LOC_Os09g39500	10.8314758
LOC_Os01g05630	LOC_Os03g09810	9.31653058
LOC_Os01g05970	LOC_Os04g01674	8.99495764
LOC_Os01g05970	LOC_Os08g33100	10.6794514
LOC_Os01g05610	LOC_Os03g53690	10.433144
LOC_Os01g05610	LOC_Os11g05730	9.05755487
LOC_Os01g05630	LOC_Os10g30840	9.64425795
LOC_Os01g05970	LOC_Os10g38189	8.82363782
LOC_Os01g05610	LOC_Os03g32270	9.03559456
LOC_Os01g05630	LOC_Os07g36130	9.99871226
LOC_Os01g05610	LOC_Os09g28460	9.51741463
LOC_Os01g05970	LOC_Os10g08710	9.2521459
LOC_Os01g05970	LOC_Os01g52470	9.42675037

InteractorA	InteractorB	Z score
LOC_Os01g05490	LOC_Os03g18580	9.2472258
LOC_Os01g05970	LOC_Os02g42710	9.44801192
LOC_Os01g05970	LOC_Os01g36070	9.00765412
LOC_Os01g06010	LOC_Os02g01920	9.05445116
LOC_Os01g05630	LOC_Os05g48980	9.01287081
LOC_Os01g05610	LOC_Os06g07210	11.1881395
LOC_Os01g05630	LOC_Os10g08580	9.48478518
LOC_Os01g05610	LOC_Os08g42560	8.85361556
LOC_Os01g05610	LOC_Os04g47360	9.50716852
LOC_Os01g05970	LOC_Os07g12200	8.92194332
LOC_Os01g05490	LOC_Os06g35730	9.02709919
LOC_Os01g05610	LOC_Os01g32364	9.3616277
LOC_Os01g05970	LOC_Os06g21570	11.1514992
LOC_Os01g05970	LOC_Os05g12190	9.37825313
LOC_Os01g06010	LOC_Os01g67340	10.4215017
LOC_Os01g05610	LOC_Os03g51200	11.4643001
LOC_Os01g06010	LOC_Os09g15790	9.21875221
LOC_Os01g05970	LOC_Os11g19800	9.71933837
LOC_Os01g05970	LOC_Os05g45220	9.48794707
LOC_Os01g05610	LOC_Os06g06320	9.7125851
LOC_Os01g06010	LOC_Os02g51830	8.9439178
LOC_Os01g05490	LOC_Os02g04950	12.0305973
LOC_Os01g05970	LOC_Os03g13070	9.98513555
LOC_Os01g05610	LOC_Os08g33100	12.8278673
LOC_Os01g05490	LOC_Os03g57790	9.52197466
LOC_Os01g05970	LOC_Os08g31870	10.296094
LOC_Os01g05970	LOC_Os10g38340	8.83368507
LOC_Os01g05610	LOC_Os05g29880	10.2498968
LOC_Os01g05630	LOC_Os02g04100	10.5373385
LOC_Os01g05490	LOC_Os02g14929	10.9100256
LOC_Os01g05610	LOC_Os07g36140	10.8134237
LOC_Os01g05970	LOC_Os09g12230	9.21748626
LOC_Os01g05610	LOC_Os05g41210	8.84239701
LOC_Os01g05970	LOC_Os12g04980	9.43276789
LOC_Os01g06010	LOC_Os05g39580	10.0917346
LOC_Os01g05970	LOC_Os06g35700	9.23806723
LOC_Os01g05610	LOC_Os05g36280	9.05755513
LOC_Os01g05630	LOC_Os11g14040	9.37330887
LOC_Os01g05630	LOC_Os11g19220	11.469765
LOC_Os01g05490	LOC_Os09g04680	8.94516099
LOC_Os01g05610	LOC_Os11g01872	8.93477096
LOC_Os01g05630	LOC_Os05g08100	9.08639683
LOC_Os01g05630	LOC_Os09g15790	10.0251174

InteractorA	InteractorB	Z score
LOC_Os01g05610	LOC_Os11g39540	8.98530689
LOC_Os01g05610	LOC_Os02g17780	9.4803264
LOC_Os01g06010	LOC_Os01g55950	9.67703892
LOC_Os01g05970	LOC_Os04g12970	11.4314244
LOC_Os01g05630	LOC_Os03g61340	9.34246057
LOC_Os01g05630	LOC_Os01g62420	8.94484664
LOC_Os01g05630	LOC_Os03g47770	10.0671682
LOC_Os01g05970	LOC_Os12g44020	9.36348949
LOC_Os01g05610	LOC_Os04g48540	8.93348398
LOC_Os01g05630	LOC_Os05g19670	9.27770648
LOC_Os01g05490	LOC_Os05g11710	9.02711407
LOC_Os01g05610	LOC_Os04g39210	9.16126063
LOC_Os01g05630	LOC_Os08g14570	9.59400627
LOC_Os01g06010	LOC_Os01g62230	10.2507454
LOC_Os01g06010	LOC_Os03g41438	9.02590367
LOC_Os01g05970	LOC_Os03g56840	9.34175375
LOC_Os01g05970	LOC_Os03g62670	8.92363537
LOC_Os01g05610	LOC_Os01g50030	8.8005004
LOC_Os01g05610	LOC_Os03g52460	10.1661489
LOC_Os01g05970	LOC_Os08g38300	12.1439122
LOC_Os01g06010	LOC_Os04g56920	8.9491828
LOC_Os01g06010	LOC_Os01g58730	8.86883829
LOC_Os01g05970	LOC_Os10g26390	9.24459299
LOC_Os01g05610	LOC_Os08g09950	9.53332271
LOC_Os01g05490	LOC_Os09g36450	10.1111946
LOC_Os01g05970	LOC_Os01g62880	8.99949091
LOC_Os01g05630	LOC_Os10g23900	9.50764845
LOC_Os01g05630	LOC_Os04g29550	13.671181
LOC_Os01g05610	LOC_Os10g41510	9.06194582
LOC_Os01g05970	LOC_Os04g53240	9.50685655
LOC_Os01g05610	LOC_Os01g12160	9.04934423
LOC_Os01g05630	LOC_Os12g17910	9.27540499
LOC_Os01g05630	LOC_Os07g48050	9.5041802
LOC_Os01g05610	LOC_Os02g57260	9.37136397
LOC_Os01g05630	LOC_Os02g14190	8.94125681
LOC_Os01g05970	LOC_Os08g41340	8.92917032
LOC_Os01g05630	LOC_Os03g16860	10.6667392
LOC_Os01g05610	LOC_Os04g12980	8.82700819
LOC_Os01g06010	LOC_Os01g27230	9.17425184
LOC_Os01g05630	LOC_Os07g47490	8.91201316
LOC_Os01g05630	LOC_Os05g45810	9.64690924
LOC_Os01g05970	LOC_Os02g02830	9.32962353
LOC_Os01g05610	LOC_Os05g37390	9.16791207

InteractorA	InteractorB	Z score
LOC_Os01g05630	LOC_Os01g51380	8.85967714
LOC_Os01g05490	LOC_Os05g29880	9.94820079
LOC_Os01g05630	LOC_Os12g38180	9.4584251
LOC_Os01g05630	LOC_Os01g46290	9.39307325
LOC_Os01g05630	LOC_Os01g51410	9.23488202
LOC_Os01g05610	LOC_Os01g64640	9.05755648
LOC_Os01g05610	LOC_Os06g06510	9.0575336
LOC_Os01g05970	LOC_Os11g26860	9.2583455
LOC_Os01g05970	LOC_Os03g38980	8.8359673
LOC_Os01g05610	LOC_Os10g32680	9.23238622
LOC_Os01g05610	LOC_Os02g14190	9.30620082
LOC_Os01g05970	LOC_Os07g36130	9.96065193
LOC_Os01g05970	LOC_Os03g51200	10.2361032
LOC_Os01g05970	LOC_Os01g48600	9.12204584
LOC_Os01g05970	LOC_Os02g04100	8.80280955
LOC_Os01g05970	LOC_Os06g11240	10.6111105
LOC_Os01g05630	LOC_Os01g58220	10.4105376
LOC_Os01g05610	LOC_Os01g57962	9.26286251
LOC_Os01g05970	LOC_Os03g55600	10.1844773
LOC_Os01g06010	LOC_Os04g37500	9.01218355
LOC_Os01g06010	LOC_Os03g56280	9.10354225
LOC_Os01g05490	LOC_Os03g50440	9.30663292
LOC_Os01g05610	LOC_Os12g43450	9.16000118
LOC_Os01g05610	LOC_Os03g17310	9.92852497
LOC_Os01g05610	LOC_Os06g06460	9.05754822
LOC_Os01g61400	LOC_Os02g55420	9.33761511
LOC_Os01g61920	LOC_Os11g10510	8.86156807
LOC_Os01g61400	LOC_Os07g02210	9.24955013
LOC_Os01g61920	LOC_Os11g10480	8.92560379
LOC_Os01g60410	LOC_Os12g07980	9.18720156
LOC_Os01g61400	LOC_Os07g47990	10.2489746
LOC_Os01g61400	LOC_Os09g31430	9.17944137
LOC_Os01g60410	LOC_Os09g24530	9.40584263
LOC_Os01g61400	LOC_Os07g23730	9.0873468
LOC_Os01g61400	LOC_Os02g38340	9.01009361
LOC_Os01g61920	LOC_Os03g51200	9.10305633
LOC_Os01g61920	LOC_Os07g07719	8.80394968
LOC_Os01g61920	LOC_Os07g07709	10.0431455
LOC_Os01g60410	LOC_Os09g17740	9.29789856
LOC_Os01g60410	LOC_Os12g01922	10.0062999
LOC_Os01g60790	LOC_Os03g06330	10.7911032
LOC_Os01g60410	LOC_Os05g38760	9.26316134
LOC_Os01g60410	LOC_Os05g11710	9.571816

InteractorA	InteractorB	Z score
LOC_Os01g61400	LOC_Os04g33190	8.85095957
LOC_Os01g61400	LOC_Os03g06200	9.2559493
LOC_Os01g60790	LOC_Os09g12660	9.01782344
LOC_Os01g60790	LOC_Os07g29440	10.3881347
LOC_Os01g61400	LOC_Os03g31170	9.05796315
LOC_Os01g61400	LOC_Os03g56280	10.0116843
LOC_Os01g61210	LOC_Os01g71990	8.8760831
LOC_Os01g61920	LOC_Os10g39170	9.48057701
LOC_Os01g61400	LOC_Os06g09910	8.86877141
LOC_Os01g60790	LOC_Os08g36900	9.47569901
LOC_Os01g61400	LOC_Os09g39440	9.03226883
LOC_Os01g61400	LOC_Os04g40950	9.76060106
LOC_Os01g61400	LOC_Os03g53650	9.62400656
LOC_Os01g61400	LOC_Os12g10730	9.08490561
LOC_Os01g60790	LOC_Os10g41510	10.1146056
LOC_Os01g61920	LOC_Os11g31620	9.02280822
LOC_Os01g61400	LOC_Os02g01560	9.70606256
LOC_Os01g61400	LOC_Os05g06750	8.96612981
LOC_Os01g60410	LOC_Os07g48880	8.97926199
LOC_Os01g61400	LOC_Os03g27280	8.8792244
LOC_Os01g60410	LOC_Os11g29400	8.93671245
LOC_Os01g60360	LOC_Os12g01922	10.0062653
LOC_Os01g61400	LOC_Os11g14910	8.91120836
LOC_Os01g61210	LOC_Os10g41510	9.52588948
LOC_Os01g60790	LOC_Os08g38300	9.54679109
LOC_Os01g60410	LOC_Os03g62700	8.96224544
LOC_Os01g61400	LOC_Os11g47600	9.61448819
LOC_Os01g60410	LOC_Os11g40150	9.21005589
LOC_Os01g61400	LOC_Os08g03290	12.1452053
LOC_Os01g61400	LOC_Os11g36719	8.85821687
LOC_Os01g61210	LOC_Os07g01780	10.1419718
LOC_Os01g60790	LOC_Os04g25990	9.67224507
LOC_Os01g61400	LOC_Os07g44260	8.97084102
LOC_Os01g61400	LOC_Os10g32680	9.35123072
LOC_Os01g60790	LOC_Os01g62230	9.44973077
LOC_Os01g61400	LOC_Os02g32030	9.84674661
LOC_Os01g61210	LOC_Os10g31940	9.28475818
LOC_Os01g61400	LOC_Os04g20070	10.3324019
LOC_Os01g61400	LOC_Os03g22010	8.88216875
LOC_Os01g61920	LOC_Os02g04950	8.80847463
LOC_Os01g61920	LOC_Os03g61600	8.88669711
LOC_Os01g61400	LOC_Os04g53230	8.85899968
LOC_Os01g61400	LOC_Os04g30420	9.30726003

InteractorA	InteractorB	Z score
LOC_Os01g61400	LOC_Os03g57790	8.83184601
LOC_Os01g60410	LOC_Os09g15420	8.89453019
LOC_Os01g61920	LOC_Os03g31300	10.4717978
LOC_Os01g61400	LOC_Os07g43260	9.02793042
LOC_Os01g61400	LOC_Os05g42350	8.8393776
LOC_Os01g60410	LOC_Os05g45220	9.00946438
LOC_Os01g60410	LOC_Os06g35730	9.571816
LOC_Os01g60410	LOC_Os04g40130	10.3195291
LOC_Os01g60790	LOC_Os09g25320	9.136594
LOC_Os01g60790	LOC_Os07g48880	9.12430486
LOC_Os01g60790	LOC_Os06g51060	9.37903606
LOC_Os01g61400	LOC_Os06g36670	8.91735014
LOC_Os01g60360	LOC_Os12g13320	11.4814143
LOC_Os01g60790	LOC_Os09g32840	10.5046817
LOC_Os01g61400	LOC_Os03g64210	9.2181161
LOC_Os01g60790	LOC_Os04g28870	9.13873231
LOC_Os01g61400	LOC_Os11g08470	9.00198782
LOC_Os01g61400	LOC_Os02g46970	9.41652904
LOC_Os01g60790	LOC_Os12g13800	9.54148754
LOC_Os01g61400	LOC_Os03g18130	9.23621886
LOC_Os01g61920	LOC_Os03g07150	9.73068597
LOC_Os01g60790	LOC_Os06g44080	9.136594
LOC_Os01g61400	LOC_Os03g11420	10.4784806
LOC_Os01g61400	LOC_Os01g63890	9.37132928
LOC_Os01g61920	LOC_Os02g56460	10.7060006
LOC_Os01g61400	LOC_Os11g47760	10.4641834
LOC_Os01g60410	LOC_Os02g56690	9.2848981
LOC_Os01g62020	LOC_Os02g38920	9.05804067
LOC_Os01g60360	LOC_Os12g07980	9.18720813
LOC_Os01g61400	LOC_Os03g22120	8.92024613
LOC_Os01g61400	LOC_Os04g41960	10.1980128
LOC_Os01g61210	LOC_Os02g27760	9.44388865
LOC_Os01g62020	LOC_Os02g48290	9.79602413
LOC_Os01g61920	LOC_Os02g21460	10.1402953
LOC_Os01g61400	LOC_Os07g19040	8.87023095
LOC_Os01g61210	LOC_Os07g48020	9.19956544
LOC_Os01g60410	LOC_Os07g35940	10.3544745
LOC_Os01g61400	LOC_Os12g10600	9.32712676
LOC_Os01g61920	LOC_Os10g22070	9.42850276
LOC_Os01g61210	LOC_Os12g10730	9.134625
LOC_Os01g61210	LOC_Os12g16220	8.88703063
LOC_Os01g61920	LOC_Os03g18740	9.04603168
LOC_Os01g61400	LOC_Os12g06620	9.50529423

InteractorA	InteractorB	Z score
LOC_Os01g60790	LOC_Os04g20810	8.97792181
LOC_Os01g60790	LOC_Os09g36830	9.07124372
LOC_Os01g61400	LOC_Os11g05290	9.14217942
LOC_Os01g61400	LOC_Os10g26130	8.94496292
LOC_Os01g60410	LOC_Os12g13320	11.4814178
LOC_Os01g61400	LOC_Os02g17390	9.05681046
LOC_Os01g60360	LOC_Os11g40150	9.21007026
LOC_Os01g61210	LOC_Os12g08280	8.80130889
LOC_Os01g61400	LOC_Os04g25400	10.063128
LOC_Os01g61920	LOC_Os10g01540	9.10660661
LOC_Os01g61400	LOC_Os08g37800	9.2019427
LOC_Os01g61400	LOC_Os04g55290	9.37142109
LOC_Os01g61920	LOC_Os03g58050	10.0431544
LOC_Os01g60360	LOC_Os11g29400	8.93670773
LOC_Os01g61920	LOC_Os11g10520	10.9140543
LOC_Os01g60790	LOC_Os02g56720	9.10509572
LOC_Os01g61920	LOC_Os12g22680	9.95584485
LOC_Os01g62020	LOC_Os02g51830	8.99243158
LOC_Os01g62244	LOC_Os06g11260	9.32725193
LOC_Os01g62244	LOC_Os12g12514	10.283049
LOC_Os01g62230	LOC_Os07g35880	8.92762555
LOC_Os01g62040	LOC_Os10g28350	9.67460086
LOC_Os01g62230	LOC_Os01g72430	9.83456644
LOC_Os01g62230	LOC_Os04g25400	12.1727482
LOC_Os01g62230	LOC_Os12g02980	8.82844226
LOC_Os01g62244	LOC_Os12g25700	9.07940342
LOC_Os01g62244	LOC_Os04g33190	8.91053486
LOC_Os01g62290	LOC_Os04g12980	10.2107515
LOC_Os01g62290	LOC_Os04g58110	9.17875747
LOC_Os01g62080	LOC_Os08g42560	9.15947323
LOC_Os01g62040	LOC_Os06g14620	10.1036019
LOC_Os01g62080	LOC_Os03g08010	10.2923934
LOC_Os01g62230	LOC_Os07g44430	8.92467476
LOC_Os01g62020	LOC_Os09g09230	9.24362476
LOC_Os01g62230	LOC_Os07g38860	9.19860106
LOC_Os01g62290	LOC_Os02g52700	9.31251304
LOC_Os01g62290	LOC_Os02g14460	9.38685181
LOC_Os01g62244	LOC_Os05g45420	9.51516181
LOC_Os01g62040	LOC_Os04g44920	9.9964215
LOC_Os01g62040	LOC_Os11g05470	9.11564925
LOC_Os01g62020	LOC_Os10g41689	11.4583922
LOC_Os01g62230	LOC_Os06g11240	8.8839907
LOC_Os01g62230	LOC_Os03g50480	9.36927402

InteractorA	InteractorB	Z score
LOC_Os01g62040	LOC_Os03g31170	9.59474889
LOC_Os01g62244	LOC_Os10g38360	9.28405947
LOC_Os01g62230	LOC_Os10g38660	8.82023411
LOC_Os01g62230	LOC_Os08g38300	10.3832985
LOC_Os01g62230	LOC_Os06g39740	9.74577892
LOC_Os01g62040	LOC_Os04g33480	10.077437
LOC_Os01g62080	LOC_Os04g16874	9.39453426
LOC_Os01g62040	LOC_Os09g32800	10.0284625
LOC_Os01g62230	LOC_Os10g25674	8.87045736
LOC_Os01g62230	LOC_Os08g31870	8.97277408
LOC_Os01g62230	LOC_Os06g47320	9.45998533
LOC_Os01g62244	LOC_Os09g31486	9.34610761
LOC_Os01g62040	LOC_Os03g31290	9.30388495
LOC_Os01g62040	LOC_Os04g29550	9.37249047
LOC_Os01g62290	LOC_Os02g04950	8.82171137
LOC_Os01g62230	LOC_Os03g61340	9.41952906
LOC_Os01g62290	LOC_Os02g53420	9.3003279
LOC_Os01g62020	LOC_Os03g05980	9.68605121
LOC_Os01g62244	LOC_Os02g52390	9.71459449
LOC_Os01g62020	LOC_Os03g07840	9.29671192
LOC_Os01g62230	LOC_Os04g12980	11.2358337
LOC_Os01g62080	LOC_Os05g34540	9.13603064
LOC_Os01g62230	LOC_Os05g46550	8.98961414
LOC_Os01g62040	LOC_Os04g58880	9.71398309
LOC_Os01g62230	LOC_Os01g73200	8.95846693
LOC_Os01g62230	LOC_Os02g48450	10.0480162
LOC_Os01g62040	LOC_Os05g12210	9.46316774
LOC_Os01g62230	LOC_Os12g25120	9.34858623
LOC_Os01g62230	LOC_Os08g44210	10.0954766
LOC_Os01g62290	LOC_Os02g52710	9.31250157
LOC_Os01g62290	LOC_Os03g55874	8.81242037
LOC_Os01g62040	LOC_Os01g67220	8.80785267
LOC_Os01g62080	LOC_Os03g08050	10.2923934
LOC_Os01g62244	LOC_Os04g19740	9.43381902
LOC_Os01g62230	LOC_Os04g20810	9.00839396
LOC_Os01g62244	LOC_Os04g44470	9.07073687
LOC_Os01g62040	LOC_Os08g39140	9.12868712
LOC_Os01g62040	LOC_Os02g08420	10.8649133
LOC_Os01g62244	LOC_Os07g42960	8.88411756
LOC_Os01g62080	LOC_Os02g55300	9.39339215
LOC_Os01g62230	LOC_Os10g23100	9.92011138
LOC_Os01g62230	LOC_Os04g39840	9.61956426
LOC_Os01g62040	LOC_Os03g04060	9.6962456

InteractorA	InteractorB	Z score
LOC_Os01g62040	LOC_Os10g26600	10.4871034
LOC_Os01g62080	LOC_Os03g08020	10.2923934
LOC_Os01g62244	LOC_Os07g05180	10.0034008
LOC_Os01g62290	LOC_Os03g01630	8.90986149
LOC_Os01g62290	LOC_Os04g48540	9.33543668
LOC_Os01g62080	LOC_Os06g37560	10.2169669
LOC_Os01g62040	LOC_Os05g11550	9.26221801
LOC_Os01g62230	LOC_Os08g33100	13.9704411
LOC_Os01g62290	LOC_Os02g52390	10.1557754
LOC_Os01g62230	LOC_Os04g45470	10.3708259
LOC_Os01g62040	LOC_Os06g28550	10.9143313
LOC_Os01g62230	LOC_Os09g20260	10.7159471
LOC_Os01g62020	LOC_Os07g05400	9.48923529
LOC_Os01g62230	LOC_Os09g07830	8.91315953
LOC_Os01g62020	LOC_Os05g46270	9.34011625
LOC_Os01g62020	LOC_Os03g11900	10.0564265
LOC_Os01g62290	LOC_Os02g56700	8.85804989
LOC_Os01g62230	LOC_Os02g41470	9.72894024
LOC_Os01g62080	LOC_Os05g48980	9.24064668
LOC_Os01g62230	LOC_Os05g44760	11.4361245
LOC_Os01g62040	LOC_Os11g47760	9.27400074
LOC_Os01g62230	LOC_Os02g55300	9.12226624
LOC_Os01g62020	LOC_Os03g62670	8.99518979
LOC_Os01g62244	LOC_Os04g41620	8.87411258
LOC_Os01g62244	LOC_Os06g35560	9.48259413
LOC_Os01g62230	LOC_Os09g26880	9.41706354
LOC_Os01g62040	LOC_Os12g16410	10.0620514
LOC_Os01g62080	LOC_Os05g04520	9.20325937
LOC_Os01g62230	LOC_Os06g06730	9.30830844
LOC_Os01g62020	LOC_Os04g46620	10.0085435
LOC_Os01g62040	LOC_Os05g04340	8.92920978
LOC_Os01g62230	LOC_Os02g17390	9.89764143
LOC_Os01g62244	LOC_Os11g32520	9.98904522
LOC_Os01g62244	LOC_Os09g23540	9.66201927
LOC_Os01g62040	LOC_Os09g31502	9.35959176
LOC_Os01g62040	LOC_Os07g11440	10.2482664
LOC_Os01g62020	LOC_Os06g11210	9.51385296
LOC_Os01g62020	LOC_Os06g11240	11.1090016
LOC_Os01g62080	LOC_Os04g33720	8.97374679
LOC_Os01g62040	LOC_Os01g63270	9.06438078
LOC_Os01g62230	LOC_Os12g13390	8.87375912
LOC_Os01g62290	LOC_Os03g57120	10.4925397
LOC_Os01g62230	LOC_Os06g04510	8.94119222

InteractorA	InteractorB	Z score
LOC_Os01g62290	LOC_Os04g45490	8.83666951
LOC_Os01g62230	LOC_Os10g05069	9.91663453
LOC_Os01g62244	LOC_Os03g08280	9.27776453
LOC_Os01g62020	LOC_Os02g57770	8.83921331
LOC_Os01g62080	LOC_Os07g12200	9.30643626
LOC_Os01g62230	LOC_Os03g22010	9.36026705
LOC_Os01g62230	LOC_Os04g44924	10.2879025
LOC_Os01g62244	LOC_Os06g06560	8.86196167
LOC_Os01g62040	LOC_Os03g45960	8.81775266
LOC_Os01g62020	LOC_Os12g40830	10.663935
LOC_Os01g62040	LOC_Os10g21212	9.5111025
LOC_Os01g62230	LOC_Os03g62670	8.88382068
LOC_Os01g62244	LOC_Os02g08490	9.59797294
LOC_Os01g62230	LOC_Os05g09500	9.1208587
LOC_Os01g62020	LOC_Os03g04110	8.87419331
LOC_Os01g62230	LOC_Os09g34970	8.89033409
LOC_Os01g62080	LOC_Os12g01922	9.84332397
LOC_Os01g62230	LOC_Os03g29570	9.39542992
LOC_Os01g62020	LOC_Os08g04540	8.89815135
LOC_Os01g62040	LOC_Os03g55090	9.3043697
LOC_Os01g62290	LOC_Os05g19380	9.33181031
LOC_Os01g62244	LOC_Os03g63090	9.3478865
LOC_Os01g62020	LOC_Os11g38959	9.68604644
LOC_Os01g62040	LOC_Os11g37550	8.80127654
LOC_Os01g62040	LOC_Os07g12730	9.04564039
LOC_Os01g62244	LOC_Os02g58730	10.1496779
LOC_Os01g62080	LOC_Os10g25130	9.1481194
LOC_Os01g62290	LOC_Os03g50250	9.93466362
LOC_Os01g62950	LOC_Os08g41340	10.6679051
LOC_Os01g62950	LOC_Os08g40170	9.29379501
LOC_Os01g62870	LOC_Os03g07840	9.83164786
LOC_Os01g62860	LOC_Os03g01800	10.1594042
LOC_Os01g62950	LOC_Os01g64670	9.22202714
LOC_Os01g62870	LOC_Os04g26910	9.59448641
LOC_Os01g62290	LOC_Os09g08072	9.0730416
LOC_Os01g62870	LOC_Os10g23900	9.18796999
LOC_Os01g62950	LOC_Os01g64100	9.91777969
LOC_Os01g62860	LOC_Os08g08070	10.2535859
LOC_Os01g62870	LOC_Os03g63410	9.51270604
LOC_Os01g62290	LOC_Os12g16240	9.89071768
LOC_Os01g62860	LOC_Os03g61280	9.84584026
LOC_Os01g62950	LOC_Os09g11230	9.73453876
LOC_Os01g62880	LOC_Os10g25950	8.92738828

InteractorA	InteractorB	Z score
LOC_Os01g62880	LOC_Os08g36910	9.32271586
LOC_Os01g62420	LOC_Os09g25390	8.80086431
LOC_Os01g62880	LOC_Os12g40830	9.55666933
LOC_Os01g62860	LOC_Os12g07980	9.07399998
LOC_Os01g62950	LOC_Os04g08350	9.50525736
LOC_Os01g62290	LOC_Os12g40510	9.32358553
LOC_Os01g62290	LOC_Os07g48060	9.87511601
LOC_Os01g62880	LOC_Os08g34290	9.3732646
LOC_Os01g62950	LOC_Os05g06300	9.09299341
LOC_Os01g62420	LOC_Os03g22060	8.82326116
LOC_Os01g62950	LOC_Os07g44450	8.88570161
LOC_Os01g62880	LOC_Os11g26860	8.87500814
LOC_Os01g62880	LOC_Os03g16740	9.0883548
LOC_Os01g62860	LOC_Os06g49970	9.10745927
LOC_Os01g62950	LOC_Os01g71380	9.16802565
LOC_Os01g62870	LOC_Os12g13390	9.63413917
LOC_Os01g62420	LOC_Os03g53650	9.08185372
LOC_Os01g62860	LOC_Os04g33740	8.95651631
LOC_Os01g62420	LOC_Os08g01660	8.93111282
LOC_Os01g62880	LOC_Os10g38140	11.4024145
LOC_Os01g62880	LOC_Os02g08100	8.88287485
LOC_Os01g62290	LOC_Os10g32870	13.2175042
LOC_Os01g62880	LOC_Os04g55410	9.02194682
LOC_Os01g62420	LOC_Os03g41438	8.94963747
LOC_Os01g62860	LOC_Os07g03690	8.9864581
LOC_Os01g62950	LOC_Os06g35630	9.07682507
LOC_Os01g62860	LOC_Os04g55740	10.3738923
LOC_Os01g62860	LOC_Os06g01390	9.46198878
LOC_Os01g62880	LOC_Os05g42350	9.23484692
LOC_Os01g62870	LOC_Os02g02560	9.3787876
LOC_Os01g62860	LOC_Os04g25990	9.15086772
LOC_Os01g62950	LOC_Os03g63410	9.44977495
LOC_Os01g62860	LOC_Os04g27860	8.82324204
LOC_Os01g62880	LOC_Os09g19954	9.57300945
LOC_Os01g62880	LOC_Os07g34520	9.91442532
LOC_Os01g62290	LOC_Os09g26380	9.46299734
LOC_Os01g62880	LOC_Os03g20710	8.85368463
LOC_Os01g62870	LOC_Os03g57290	9.19591448
LOC_Os01g62420	LOC_Os05g29880	9.9763871
LOC_Os01g62860	LOC_Os07g22930	8.86993357
LOC_Os01g62860	LOC_Os10g33800	9.01492879
LOC_Os01g62860	LOC_Os11g34450	9.07270487
LOC_Os01g62860	LOC_Os02g01920	8.8653577

InteractorA	InteractorB	Z score
LOC_Os01g62860	LOC_Os05g49840	10.3916735
LOC_Os01g62880	LOC_Os03g01640	8.92821022
LOC_Os01g62860	LOC_Os01g64850	9.05750579
LOC_Os01g62880	LOC_Os03g55280	8.88498773
LOC_Os01g62860	LOC_Os05g26890	9.36044936
LOC_Os01g62870	LOC_Os09g25390	8.9381864
LOC_Os01g62420	LOC_Os02g35590	9.39210286
LOC_Os01g62860	LOC_Os09g19954	9.47208206
LOC_Os01g62880	LOC_Os02g12580	9.39648315
LOC_Os01g62290	LOC_Os10g29470	8.88410664
LOC_Os01g62950	LOC_Os07g03690	11.5391683
LOC_Os01g62880	LOC_Os01g71320	9.55374869
LOC_Os01g62860	LOC_Os03g14450	9.7599262
LOC_Os01g62290	LOC_Os07g23470	9.18155548
LOC_Os01g62880	LOC_Os05g39690	9.82900631
LOC_Os01g62860	LOC_Os06g04510	8.96182285
LOC_Os01g62880	LOC_Os07g48050	9.00990292
LOC_Os01g62870	LOC_Os12g24650	10.1715593
LOC_Os01g62860	LOC_Os02g13110	10.2787743
LOC_Os01g62880	LOC_Os12g07720	9.23354368
LOC_Os01g62870	LOC_Os04g58110	9.22698189
LOC_Os01g62950	LOC_Os02g17780	10.0084367
LOC_Os01g62880	LOC_Os11g14040	9.16765405
LOC_Os01g62420	LOC_Os09g38620	8.92546502
LOC_Os01g62950	LOC_Os02g01340	9.87433373
LOC_Os01g62870	LOC_Os05g04690	9.14567437
LOC_Os01g62860	LOC_Os10g27174	9.01215339
LOC_Os01g62880	LOC_Os07g07550	9.98215089
LOC_Os01g62420	LOC_Os03g49600	9.09849185
LOC_Os01g62870	LOC_Os02g02960	8.88918075
LOC_Os01g62290	LOC_Os10g39680	9.16076586
LOC_Os01g62880	LOC_Os04g47170	9.36666938
LOC_Os01g62880	LOC_Os03g04060	9.12539582
LOC_Os01g62950	LOC_Os05g46550	9.37521252
LOC_Os01g62290	LOC_Os10g42940	9.26574668
LOC_Os01g62880	LOC_Os05g20050	8.82081972
LOC_Os01g62870	LOC_Os03g08530	8.83623187
LOC_Os01g62880	LOC_Os05g44760	9.29651346
LOC_Os01g62290	LOC_Os05g34540	8.9157018
LOC_Os01g62950	LOC_Os05g37700	9.19597941
LOC_Os01g62870	LOC_Os03g62700	9.12713691
LOC_Os01g62950	LOC_Os05g20050	8.92770247
LOC_Os01g62950	LOC_Os01g74000	9.29688087

InteractorA	InteractorB	Z score
LOC_Os01g63420	LOC_Os02g40830	8.8925724
LOC_Os01g63420	LOC_Os03g13140	9.41109218
LOC_Os01g63420	LOC_Os03g45960	9.83861939
LOC_Os01g64100	LOC_Os04g39864	8.97463071
LOC_Os01g63420	LOC_Os10g37210	9.93915045
LOC_Os01g63270	LOC_Os02g24632	9.80901457
LOC_Os01g63890	LOC_Os03g11970	8.95317155
LOC_Os01g64110	LOC_Os02g47800	8.84366994
LOC_Os01g64100	LOC_Os02g32950	9.09905155
LOC_Os01g63420	LOC_Os03g63720	9.16754071
LOC_Os01g63420	LOC_Os11g19220	9.14588405
LOC_Os01g64100	LOC_Os04g58880	8.93306631
LOC_Os01g64100	LOC_Os03g61360	9.74805739
LOC_Os01g64110	LOC_Os04g53920	10.1762698
LOC_Os01g63890	LOC_Os02g07260	10.0845577
LOC_Os01g63890	LOC_Os08g10010	8.87220425
LOC_Os01g64110	LOC_Os06g06980	9.77023427
LOC_Os01g63270	LOC_Os04g36800	9.2468285
LOC_Os01g64100	LOC_Os02g51100	8.96865955
LOC_Os01g63890	LOC_Os02g56130	8.89694611
LOC_Os01g64100	LOC_Os02g47610	8.93802893
LOC_Os01g64110	LOC_Os06g12790	8.93772009
LOC_Os01g63270	LOC_Os02g57150	8.80758144
LOC_Os01g64100	LOC_Os07g31270	8.81383802
LOC_Os01g63270	LOC_Os07g05400	9.92013716
LOC_Os01g63420	LOC_Os03g58320	9.73821564
LOC_Os01g64100	LOC_Os08g17680	10.9725402
LOC_Os01g63270	LOC_Os03g01900	9.61650926
LOC_Os01g64100	LOC_Os03g63720	8.92612571
LOC_Os01g64110	LOC_Os04g56920	9.14495545
LOC_Os01g63270	LOC_Os02g53420	9.79081917
LOC_Os01g63270	LOC_Os12g36950	9.53132202
LOC_Os01g64100	LOC_Os03g42220	10.4158124
LOC_Os01g63420	LOC_Os06g02144	9.11662361
LOC_Os01g64100	LOC_Os10g38140	9.25561062
LOC_Os01g64110	LOC_Os07g05400	8.85229791
LOC_Os01g63890	LOC_Os03g43890	10.3820615
LOC_Os01g63270	LOC_Os04g57400	8.95344291
LOC_Os01g63420	LOC_Os04g26870	9.34586227
LOC_Os01g63270	LOC_Os07g43510	9.09792615
LOC_Os01g63270	LOC_Os04g57380	9.71879007
LOC_Os01g64100	LOC_Os11g38959	10.1304277
LOC_Os01g63270	LOC_Os04g43760	9.06991703

InteractorA	InteractorB	Z score
LOC_Os01g63420	LOC_Os12g31640	9.45866569
LOC_Os01g63890	LOC_Os03g53790	11.0454694
LOC_Os01g63270	LOC_Os03g04110	9.13382034
LOC_Os01g63890	LOC_Os09g14670	9.56206955
LOC_Os01g63420	LOC_Os09g36830	9.14885264
LOC_Os01g64110	LOC_Os04g58580	11.221742
LOC_Os01g63890	LOC_Os06g45590	10.568887
LOC_Os01g63890	LOC_Os11g14220	10.7258336
LOC_Os01g64100	LOC_Os07g23730	9.94452625
LOC_Os01g64100	LOC_Os09g26380	9.3772747
LOC_Os01g63420	LOC_Os09g27420	8.96805638
LOC_Os01g64110	LOC_Os04g41960	8.89499259
LOC_Os01g63420	LOC_Os03g62670	9.13182322
LOC_Os01g63890	LOC_Os01g70525	9.49811053
LOC_Os01g64100	LOC_Os03g05980	10.1304013
LOC_Os01g63420	LOC_Os11g32610	9.63540989
LOC_Os01g63420	LOC_Os02g07230	8.81073813
LOC_Os01g63890	LOC_Os12g13380	9.13517172
LOC_Os01g63270	LOC_Os07g30990	9.88882808
LOC_Os01g64100	LOC_Os09g23300	8.81297535
LOC_Os01g63890	LOC_Os09g23550	8.80851904
LOC_Os01g64100	LOC_Os01g71380	9.18225641
LOC_Os01g63270	LOC_Os01g71380	8.98660536
LOC_Os01g63270	LOC_Os03g25340	9.26334549
LOC_Os01g63890	LOC_Os03g55280	9.18348538
LOC_Os01g63270	LOC_Os08g34190	9.17946714
LOC_Os01g63420	LOC_Os02g35760	8.94235448
LOC_Os01g64110	LOC_Os05g49830	9.38997779
LOC_Os01g63890	LOC_Os11g20790	12.052789
LOC_Os01g63890	LOC_Os06g04270	10.1789289
LOC_Os01g64100	LOC_Os05g04490	9.06655588
LOC_Os01g63420	LOC_Os07g44740	10.4361256
LOC_Os01g63270	LOC_Os09g25150	8.9595608
LOC_Os01g63420	LOC_Os06g51084	9.26887176
LOC_Os01g63420	LOC_Os04g58110	9.24366361
LOC_Os01g63420	LOC_Os08g02410	10.5637474
LOC_Os01g64110	LOC_Os08g27840	9.51650257
LOC_Os01g64630	LOC_Os02g33080	8.91087364
LOC_Os01g63420	LOC_Os02g04320	9.76697565
LOC_Os01g64100	LOC_Os11g32520	8.85060849
LOC_Os01g63890	LOC_Os11g32510	9.58113067
LOC_Os01g63420	LOC_Os07g39870	9.12773874
LOC_Os01g63420	LOC_Os10g28350	8.91533497

InteractorA	InteractorB	Z score
LOC_Os01g63890	LOC_Os01g71830	11.6124977
LOC_Os01g63270	LOC_Os11g16590	9.5513525
LOC_Os01g63420	LOC_Os03g49260	11.2499554
LOC_Os01g63270	LOC_Os05g12240	10.307918
LOC_Os01g63890	LOC_Os04g47170	9.71806493
LOC_Os01g64100	LOC_Os01g71990	8.8642312
LOC_Os01g64100	LOC_Os09g10260	9.62577424
LOC_Os01g64110	LOC_Os12g38750	8.86711456
LOC_Os01g63890	LOC_Os02g52560	9.42143224
LOC_Os01g64110	LOC_Os04g33040	9.52165014
LOC_Os01g64110	LOC_Os05g20050	9.68789742
LOC_Os01g63420	LOC_Os02g35590	8.99116879
LOC_Os01g64110	LOC_Os04g57590	9.64432314
LOC_Os01g64100	LOC_Os04g30420	9.23120032
LOC_Os01g63420	LOC_Os06g35814	8.86731538
LOC_Os01g63420	LOC_Os04g16680	8.95255067
LOC_Os01g63420	LOC_Os04g31070	9.60035485
LOC_Os01g64110	LOC_Os07g26690	9.18237998
LOC_Os01g63890	LOC_Os02g27760	8.82394458
LOC_Os01g63890	LOC_Os12g34062	8.94128842
LOC_Os01g64100	LOC_Os09g19954	9.80015571
LOC_Os01g63270	LOC_Os08g15040	9.30027614
LOC_Os01g63420	LOC_Os09g36710	9.22204373
LOC_Os01g64110	LOC_Os06g09910	9.13299544
LOC_Os01g63420	LOC_Os07g35880	9.98874145
LOC_Os01g64100	LOC_Os09g10300	9.0546406
LOC_Os01g64110	LOC_Os04g30420	10.042534
LOC_Os01g63890	LOC_Os09g19954	9.76927667
LOC_Os01g63890	LOC_Os04g12980	9.30827205
LOC_Os01g63420	LOC_Os02g09490	8.91969379
LOC_Os01g63890	LOC_Os01g71680	10.4738214
LOC_Os01g63890	LOC_Os12g37960	9.83671468
LOC_Os01g64660	LOC_Os08g36910	8.88666549
LOC_Os01g64660	LOC_Os03g53200	9.54957483
LOC_Os01g64680	LOC_Os04g41960	9.131109
LOC_Os01g64660	LOC_Os12g13800	9.15580311
LOC_Os01g64850	LOC_Os03g01800	9.36785401
LOC_Os01g64630	LOC_Os10g26050	9.24663661
LOC_Os01g64630	LOC_Os11g47760	9.27117028
LOC_Os01g64640	LOC_Os02g50860	8.89202896
LOC_Os01g64640	LOC_Os02g25940	9.13714022
LOC_Os01g64660	LOC_Os02g02410	8.84222464
LOC_Os01g64670	LOC_Os05g11550	8.94729491

InteractorA	InteractorB	Z score
LOC_Os01g64640	LOC_Os01g71350	9.11600881
LOC_Os01g64640	LOC_Os04g18200	9.41814993
LOC_Os01g64680	LOC_Os01g71340	8.83299929
LOC_Os01g64630	LOC_Os03g51740	9.47520775
LOC_Os01g64680	LOC_Os01g73580	8.92208302
LOC_Os01g64660	LOC_Os10g41510	10.2907797
LOC_Os01g64630	LOC_Os09g10260	8.87074009
LOC_Os01g64660	LOC_Os01g72430	9.83349025
LOC_Os01g64660	LOC_Os06g45100	9.03664093
LOC_Os01g64670	LOC_Os04g33720	9.19404186
LOC_Os01g64670	LOC_Os12g02370	9.48448328
LOC_Os01g64680	LOC_Os08g27840	9.33485925
LOC_Os01g64660	LOC_Os04g52100	9.91043767
LOC_Os01g64660	LOC_Os11g08440	10.8034292
LOC_Os01g64670	LOC_Os04g40130	9.22688027
LOC_Os01g64640	LOC_Os09g32620	10.0128063
LOC_Os01g64640	LOC_Os10g38160	8.95621906
LOC_Os01g64660	LOC_Os03g25370	9.40727253
LOC_Os01g64630	LOC_Os06g35530	10.4315036
LOC_Os01g64660	LOC_Os02g38840	10.0555265
LOC_Os01g64660	LOC_Os09g34214	9.34150496
LOC_Os01g64680	LOC_Os03g56810	9.41346776
LOC_Os01g64640	LOC_Os06g37180	9.2220983
LOC_Os01g64640	LOC_Os06g06510	11.0654312
LOC_Os01g64660	LOC_Os07g03690	9.38151231
LOC_Os01g64660	LOC_Os06g01360	9.46850584
LOC_Os01g64660	LOC_Os03g49220	9.0440354
LOC_Os01g64660	LOC_Os10g38690	9.8884918
LOC_Os01g64630	LOC_Os04g09604	9.28477027
LOC_Os01g64850	LOC_Os03g13140	9.17804554
LOC_Os01g64680	LOC_Os11g16590	9.05072781
LOC_Os01g64640	LOC_Os10g38350	9.2380179
LOC_Os01g64630	LOC_Os08g42560	8.82805578
LOC_Os01g64640	LOC_Os06g06460	11.0654308
LOC_Os01g64850	LOC_Os05g49770	9.10695995
LOC_Os01g64670	LOC_Os04g44950	9.15395824
LOC_Os01g64680	LOC_Os08g34190	9.16439882
LOC_Os01g64660	LOC_Os02g14770	11.2972932
LOC_Os01g64640	LOC_Os09g12590	10.6579355
LOC_Os01g64850	LOC_Os03g13070	9.77822034
LOC_Os01g64680	LOC_Os07g03710	9.1140867
LOC_Os01g64630	LOC_Os07g49400	9.52526315
LOC_Os01g64640	LOC_Os10g07616	9.5223222

InteractorA	InteractorB	Z score
LOC_Os01g64680	LOC_Os02g45540	8.97099204
LOC_Os01g64630	LOC_Os06g45710	9.43098917
LOC_Os01g64680	LOC_Os11g33240	8.83982775
LOC_Os01g64660	LOC_Os05g19380	9.01838808
LOC_Os01g64680	LOC_Os12g04980	8.86809805
LOC_Os01g64680	LOC_Os04g48700	9.25029755
LOC_Os01g64680	LOC_Os02g57040	9.44252512
LOC_Os01g64640	LOC_Os05g01050	9.16957199
LOC_Os01g64640	LOC_Os12g10730	9.13435634
LOC_Os01g64660	LOC_Os12g40510	9.68581376
LOC_Os01g64680	LOC_Os10g07616	9.20173003
LOC_Os01g64670	LOC_Os05g45810	9.72877815
LOC_Os01g64670	LOC_Os03g59660	9.02981984
LOC_Os01g64850	LOC_Os05g45220	9.80102473
LOC_Os01g64670	LOC_Os04g33040	9.97830834
LOC_Os01g64670	LOC_Os05g28940	9.19366369
LOC_Os01g64680	LOC_Os03g41460	9.07900622
LOC_Os01g64660	LOC_Os04g39020	9.71796826
LOC_Os01g64670	LOC_Os11g02440	11.199198
LOC_Os01g64660	LOC_Os07g17010	9.26630763
LOC_Os01g64640	LOC_Os07g44550	10.5558631
LOC_Os01g64850	LOC_Os04g12480	9.83385153
LOC_Os01g64680	LOC_Os06g46000	9.89847193
LOC_Os01g64660	LOC_Os08g29370	8.941592
LOC_Os01g64640	LOC_Os08g04180	8.84954596
LOC_Os01g64640	LOC_Os11g05730	11.0654354
LOC_Os01g64660	LOC_Os07g10660	9.20703455
LOC_Os01g64680	LOC_Os07g22950	9.05547391
LOC_Os01g64640	LOC_Os03g13800	9.57021141
LOC_Os01g64640	LOC_Os12g22680	9.68892421
LOC_Os01g64850	LOC_Os03g51550	9.21653326
LOC_Os01g64660	LOC_Os03g42840	10.9085401
LOC_Os01g64660	LOC_Os08g41880	9.16816214
LOC_Os01g64850	LOC_Os04g57950	9.41797556
LOC_Os01g64640	LOC_Os03g32050	8.97592296
LOC_Os01g64680	LOC_Os05g49760	9.01314278
LOC_Os01g64640	LOC_Os05g33730	9.52246675
LOC_Os01g64680	LOC_Os04g10010	9.25115198
LOC_Os01g64850	LOC_Os05g08430	9.32844926
LOC_Os01g64680	LOC_Os03g49600	9.16915391
LOC_Os01g64630	LOC_Os03g04410	9.43268531
LOC_Os01g64630	LOC_Os03g62500	9.47619749
LOC_Os01g64670	LOC_Os03g30870	9.6191604

InteractorA	InteractorB	Z score
LOC_Os01g64850	LOC_Os04g53214	8.8229977
LOC_Os01g64640	LOC_Os05g36280	11.0654287
LOC_Os01g64640	LOC_Os06g36770	9.28017027
LOC_Os01g64850	LOC_Os04g39900	9.05992737
LOC_Os01g64850	LOC_Os05g39960	10.0126948
LOC_Os01g64670	LOC_Os07g44620	9.18453269
LOC_Os01g64660	LOC_Os06g37500	9.49119529
LOC_Os01g64640	LOC_Os01g73740	8.99509146
LOC_Os01g64640	LOC_Os12g07980	10.6969479
LOC_Os01g64850	LOC_Os05g43820	9.36911556
LOC_Os01g64680	LOC_Os04g46460	9.72873571
LOC_Os01g64640	LOC_Os04g42920	9.3590828
LOC_Os01g64680	LOC_Os11g10510	9.48351579
LOC_Os01g64850	LOC_Os10g37210	9.40250861
LOC_Os01g66180	LOC_Os09g23530	8.85295907
LOC_Os01g66720	LOC_Os07g03368	8.89686089
LOC_Os01g64970	LOC_Os03g16860	9.95589772
LOC_Os01g65090	LOC_Os06g43660	12.1372157
LOC_Os01g65090	LOC_Os10g25674	9.165581
LOC_Os01g65090	LOC_Os03g17470	9.31865363
LOC_Os01g65090	LOC_Os09g32620	9.59465609
LOC_Os01g66720	LOC_Os09g10230	9.01386103
LOC_Os01g64970	LOC_Os10g29620	10.2397769
LOC_Os01g65090	LOC_Os05g49890	9.65335227
LOC_Os01g64850	LOC_Os07g46630	9.29282222
LOC_Os01g66720	LOC_Os10g25930	9.50082669
LOC_Os01g66720	LOC_Os03g52460	9.64583441
LOC_Os01g65090	LOC_Os11g10480	11.721107
LOC_Os01g64850	LOC_Os11g08940	9.61876236
LOC_Os01g65090	LOC_Os09g20220	8.95890044
LOC_Os01g66720	LOC_Os09g04050	9.00026385
LOC_Os01g66180	LOC_Os05g15520	8.89237824
LOC_Os01g65090	LOC_Os11g18870	9.57596328
LOC_Os01g64970	LOC_Os06g40940	9.08283591
LOC_Os01g65090	LOC_Os02g39064	9.26012099
LOC_Os01g64970	LOC_Os04g33040	10.1047108
LOC_Os01g66720	LOC_Os02g51830	10.8834745
LOC_Os01g65830	LOC_Os04g10010	8.88173701
LOC_Os01g66720	LOC_Os07g43260	10.7579497
LOC_Os01g65090	LOC_Os05g04490	8.95079114
LOC_Os01g66720	LOC_Os01g67860	9.4728967
LOC_Os01g65090	LOC_Os07g28480	9.81282586
LOC_Os01g65830	LOC_Os05g28180	10.382104

InteractorA	InteractorB	Z score
LOC_Os01g66720	LOC_Os01g71320	8.85122816
LOC_Os01g65090	LOC_Os08g27840	9.42062931
LOC_Os01g64970	LOC_Os06g35650	8.83301158
LOC_Os01g64970	LOC_Os10g41510	10.5231137
LOC_Os01g64970	LOC_Os12g21798	9.2034854
LOC_Os01g65830	LOC_Os04g46930	8.93578511
LOC_Os01g65090	LOC_Os07g22600	8.93143682
LOC_Os01g66720	LOC_Os04g58580	9.82119833
LOC_Os01g65090	LOC_Os10g38580	9.32394592
LOC_Os01g64970	LOC_Os02g01220	11.7236895
LOC_Os01g66720	LOC_Os03g07840	9.87226108
LOC_Os01g65830	LOC_Os05g10780	9.57726961
LOC_Os01g65090	LOC_Os08g05910	8.94330094
LOC_Os01g65090	LOC_Os02g49610	8.89867621
LOC_Os01g64850	LOC_Os09g10270	8.85271696
LOC_Os01g65830	LOC_Os03g51600	9.03359416
LOC_Os01g64970	LOC_Os02g32370	9.04707547
LOC_Os01g64970	LOC_Os04g05080	8.95792854
LOC_Os01g65090	LOC_Os03g30950	9.14805996
LOC_Os01g65830	LOC_Os07g38730	9.20169586
LOC_Os01g64970	LOC_Os08g44270	9.21707966
LOC_Os01g66720	LOC_Os06g36700	9.20211973
LOC_Os01g65090	LOC_Os02g22130	9.98343246
LOC_Os01g65090	LOC_Os02g07720	10.6127643
LOC_Os01g66720	LOC_Os06g07080	8.90429463
LOC_Os01g65090	LOC_Os04g10000	9.08275716
LOC_Os01g66180	LOC_Os08g37790	8.93766691
LOC_Os01g65830	LOC_Os10g03540	8.88183158
LOC_Os01g64850	LOC_Os08g40930	9.71601747
LOC_Os01g66180	LOC_Os04g54390	9.4206689
LOC_Os01g66720	LOC_Os08g33710	9.08466962
LOC_Os01g65830	LOC_Os08g09950	8.86139508
LOC_Os01g64970	LOC_Os10g07616	8.82445932
LOC_Os01g66720	LOC_Os08g06550	9.94548274
LOC_Os01g66720	LOC_Os06g28550	10.2915082
LOC_Os01g66180	LOC_Os10g39590	8.94274981
LOC_Os01g66720	LOC_Os07g05400	10.4873153
LOC_Os01g66720	LOC_Os08g25734	11.6743999
LOC_Os01g64970	LOC_Os04g27860	9.1270481
LOC_Os01g66720	LOC_Os10g22310	10.5763843
LOC_Os01g65090	LOC_Os03g60580	8.80270608
LOC_Os01g64850	LOC_Os08g34170	9.25165802
LOC_Os01g65830	LOC_Os08g03440	8.96262807

InteractorA	InteractorB	Z score
LOC_Os01g65830	LOC_Os08g10010	9.65446965
LOC_Os01g65090	LOC_Os05g41230	9.45550101
LOC_Os01g65090	LOC_Os01g71380	9.24231476
LOC_Os01g64970	LOC_Os05g41230	10.1326105
LOC_Os01g64850	LOC_Os10g31950	9.49401446
LOC_Os01g64850	LOC_Os11g25700	10.5024101
LOC_Os01g66180	LOC_Os12g13390	8.86797176
LOC_Os01g65090	LOC_Os12g13030	9.18284
LOC_Os01g66720	LOC_Os03g55389	9.52097942
LOC_Os01g64850	LOC_Os06g04000	8.97059567
LOC_Os01g65090	LOC_Os03g52090	9.23013249
LOC_Os01g64970	LOC_Os02g58340	10.1658733
LOC_Os01g64970	LOC_Os10g41550	9.9843792
LOC_Os01g64970	LOC_Os05g50710	9.09729969
LOC_Os01g65090	LOC_Os04g27860	8.87456956
LOC_Os01g66720	LOC_Os04g44924	9.54624061
LOC_Os01g64970	LOC_Os03g58430	8.89791045
LOC_Os01g66720	LOC_Os02g45540	9.24341432
LOC_Os01g66720	LOC_Os10g35110	9.55578062
LOC_Os01g64970	LOC_Os07g47290	9.51317766
LOC_Os01g65830	LOC_Os02g30200	10.1659539
LOC_Os01g66180	LOC_Os12g12514	8.95689197
LOC_Os01g64970	LOC_Os01g73740	9.09540406
LOC_Os01g64970	LOC_Os02g55370	8.80447402
LOC_Os01g64970	LOC_Os02g10320	9.54684839
LOC_Os01g64970	LOC_Os12g37360	10.5902406
LOC_Os01g66720	LOC_Os09g23550	8.91214947
LOC_Os01g64850	LOC_Os11g16590	9.40006253
LOC_Os01g65090	LOC_Os12g16290	9.25874622
LOC_Os01g65090	LOC_Os12g02080	9.33957989
LOC_Os01g65830	LOC_Os12g25630	9.31210398
LOC_Os01g66720	LOC_Os03g01120	9.36339988
LOC_Os01g66720	LOC_Os03g47770	10.9456606
LOC_Os01g65090	LOC_Os03g22120	10.8767843
LOC_Os01g64970	LOC_Os07g03690	8.9832488
LOC_Os01g66180	LOC_Os03g09080	10.4938389
LOC_Os01g65090	LOC_Os03g57200	10.9995024
LOC_Os01g64850	LOC_Os10g39840	8.91594814
LOC_Os01g65090	LOC_Os09g04050	9.08004571
LOC_Os01g66720	LOC_Os09g21770	9.67974456
LOC_Os01g65090	LOC_Os11g03230	10.4006367
LOC_Os01g64850	LOC_Os06g42560	8.86559937
LOC_Os01g66720	LOC_Os09g32830	8.84122839

InteractorA	InteractorB	Z score
LOC_Os01g64970	LOC_Os11g16590	9.13361059
LOC_Os01g64970	LOC_Os06g40180	9.06742381
LOC_Os01g64970	LOC_Os10g39880	8.95031308
LOC_Os01g64970	LOC_Os03g55800	8.95118879
LOC_Os01g65090	LOC_Os03g17690	9.45282648
LOC_Os01g65090	LOC_Os03g27370	9.42761869
LOC_Os01g64970	LOC_Os02g48290	9.18001651
LOC_Os01g66180	LOC_Os03g49600	8.94216661
LOC_Os01g64970	LOC_Os05g29880	9.45254436
LOC_Os01g66720	LOC_Os09g28420	9.10846131
LOC_Os01g64850	LOC_Os12g02060	9.37823661
LOC_Os01g69080	LOC_Os02g10390	10.5061135
LOC_Os01g67860	LOC_Os10g35480	8.89262355
LOC_Os01g67510	LOC_Os09g36710	8.90297858
LOC_Os01g69080	LOC_Os01g72800	10.302904
LOC_Os01g67220	LOC_Os07g48030	8.82685773
LOC_Os01g67860	LOC_Os04g01600	9.65068815
LOC_Os01g67340	LOC_Os04g48850	9.10856167
LOC_Os01g67160	LOC_Os01g73220	9.34931728
LOC_Os01g67160	LOC_Os09g31486	9.91023752
LOC_Os01g67340	LOC_Os06g27770	8.89092805
LOC_Os01g67510	LOC_Os07g36190	9.30973481
LOC_Os01g67340	LOC_Os06g02490	9.89367048
LOC_Os01g67340	LOC_Os04g16872	8.816166
LOC_Os01g67220	LOC_Os09g36930	9.19292523
LOC_Os01g67160	LOC_Os08g06610	8.96549372
LOC_Os01g67220	LOC_Os03g55280	8.96625576
LOC_Os01g67340	LOC_Os03g59740	8.84495109
LOC_Os01g67220	LOC_Os08g44350	9.40133238
LOC_Os01g67340	LOC_Os03g27280	9.97448319
LOC_Os01g67340	LOC_Os04g38220	9.20524421
LOC_Os01g67510	LOC_Os12g12580	9.65750458
LOC_Os01g67340	LOC_Os06g11800	9.00411233
LOC_Os01g67340	LOC_Os02g12730	10.5403558
LOC_Os01g67340	LOC_Os04g46930	9.13317674
LOC_Os01g67340	LOC_Os05g09490	10.1651521
LOC_Os01g67860	LOC_Os04g58680	9.63046619
LOC_Os01g67510	LOC_Os09g16910	10.5586335
LOC_Os01g67160	LOC_Os07g01780	8.86742764
LOC_Os01g67160	LOC_Os03g43890	10.6830365
LOC_Os01g67340	LOC_Os08g37490	9.69340241
LOC_Os01g67860	LOC_Os03g40330	9.43835552
LOC_Os01g67510	LOC_Os11g26860	8.9478111

InteractorA	InteractorB	Z score
LOC_Os01g67220	LOC_Os03g16920	9.56426154
LOC_Os01g67510	LOC_Os08g34290	8.80887967
LOC_Os01g67340	LOC_Os07g39290	9.26630003
LOC_Os01g67860	LOC_Os01g73310	9.10219163
LOC_Os01g67860	LOC_Os03g62060	9.79922849
LOC_Os01g67340	LOC_Os03g25370	10.6949962
LOC_Os01g67340	LOC_Os09g31502	9.40993787
LOC_Os01g67860	LOC_Os12g16220	9.99026625
LOC_Os01g67160	LOC_Os02g01920	8.91638378
LOC_Os01g67160	LOC_Os11g26910	9.49921711
LOC_Os01g67860	LOC_Os03g08560	9.95897889
LOC_Os01g67860	LOC_Os04g41960	9.04683976
LOC_Os01g67860	LOC_Os05g46580	8.86811709
LOC_Os01g67510	LOC_Os10g40730	9.08323495
LOC_Os01g67860	LOC_Os03g06330	9.19895551
LOC_Os01g67510	LOC_Os12g13390	9.92388933
LOC_Os01g67340	LOC_Os12g06620	10.1608827
LOC_Os01g67340	LOC_Os10g38660	9.03033748
LOC_Os01g67220	LOC_Os12g16250	9.08179931
LOC_Os01g67220	LOC_Os10g38229	9.43498514
LOC_Os01g67160	LOC_Os02g57150	9.58542641
LOC_Os01g67340	LOC_Os03g08010	9.04121115
LOC_Os01g67220	LOC_Os12g34450	8.97058094
LOC_Os01g69080	LOC_Os03g17980	9.67078641
LOC_Os01g67860	LOC_Os03g01770	11.5361505
LOC_Os01g67160	LOC_Os07g05180	8.90435993
LOC_Os01g67860	LOC_Os03g61970	9.26266645
LOC_Os01g67860	LOC_Os06g42130	8.83466958
LOC_Os01g67860	LOC_Os10g26390	9.2296602
LOC_Os01g67220	LOC_Os04g37500	9.55193113
LOC_Os01g67220	LOC_Os06g36160	8.93115154
LOC_Os01g67860	LOC_Os04g55410	9.0146851
LOC_Os01g67160	LOC_Os06g48180	8.98115673
LOC_Os01g67220	LOC_Os02g02840	9.01711901
LOC_Os01g67220	LOC_Os03g27370	9.02117536
LOC_Os01g67860	LOC_Os07g01780	9.16937515
LOC_Os01g67860	LOC_Os07g34589	8.97299839
LOC_Os01g67340	LOC_Os08g38300	11.829474
LOC_Os01g67860	LOC_Os08g34290	9.72211309
LOC_Os01g67160	LOC_Os03g04250	9.2555007
LOC_Os01g67340	LOC_Os05g05830	9.12112934
LOC_Os01g67340	LOC_Os08g40930	9.07609032
LOC_Os01g67340	LOC_Os03g08020	9.04122482

InteractorA	InteractorB	Z score
LOC_Os01g67860	LOC_Os09g23540	9.04493924
LOC_Os01g67340	LOC_Os12g43100	10.2666942
LOC_Os01g67340	LOC_Os03g22020	8.95724727
LOC_Os01g67860	LOC_Os08g09200	9.61397967
LOC_Os01g67860	LOC_Os08g35740	9.70191641
LOC_Os01g67340	LOC_Os02g39850	9.77693164
LOC_Os01g67510	LOC_Os05g31020	8.95425966
LOC_Os01g67160	LOC_Os02g55420	9.3918575
LOC_Os01g67340	LOC_Os03g08050	9.04122482
LOC_Os01g67340	LOC_Os08g38920	8.88686062
LOC_Os01g67220	LOC_Os09g25320	9.26118302
LOC_Os01g67220	LOC_Os04g29550	8.93228659
LOC_Os01g67340	LOC_Os03g13070	9.77065787
LOC_Os01g69080	LOC_Os03g18810	9.46404344
LOC_Os01g67860	LOC_Os08g03440	9.20508234
LOC_Os01g67340	LOC_Os12g42280	9.9531875
LOC_Os01g67860	LOC_Os10g25130	8.92710034
LOC_Os01g67340	LOC_Os08g34190	8.92974319
LOC_Os01g66720	LOC_Os11g08940	9.29371671
LOC_Os01g67860	LOC_Os03g08530	9.04422202
LOC_Os01g67510	LOC_Os03g64030	9.51399557
LOC_Os01g67340	LOC_Os09g38030	9.21877226
LOC_Os01g67510	LOC_Os07g49400	8.82288691
LOC_Os01g67160	LOC_Os07g37550	9.19032589
LOC_Os01g67220	LOC_Os06g44080	9.26120968
LOC_Os01g67220	LOC_Os03g48390	9.78603177
LOC_Os01g67860	LOC_Os03g56460	9.03198481
LOC_Os01g66720	LOC_Os12g08270	9.06471861
LOC_Os01g67160	LOC_Os03g63090	9.79804673
LOC_Os01g67510	LOC_Os04g56400	8.95107192
LOC_Os01g67220	LOC_Os02g02830	8.81021319
LOC_Os01g67860	LOC_Os10g02040	9.19343973
LOC_Os01g67340	LOC_Os09g36930	10.3212244
LOC_Os01g67510	LOC_Os05g05470	10.1763888
LOC_Os01g67160	LOC_Os03g56410	8.80169699
LOC_Os01g67160	LOC_Os09g31502	9.59335133
LOC_Os01g67510	LOC_Os01g71410	9.47579691
LOC_Os01g67220	LOC_Os05g45810	9.22335912
LOC_Os01g67510	LOC_Os03g11900	9.15206888
LOC_Os01g67860	LOC_Os03g56280	10.1107651
LOC_Os01g67340	LOC_Os09g23550	8.95606207
LOC_Os01g67220	LOC_Os10g39880	9.10865494
LOC_Os01g67220	LOC_Os03g01260	9.77668453

InteractorA	InteractorB	Z score
LOC_Os01g67220	LOC_Os06g51150	10.7259287
LOC_Os01g67860	LOC_Os11g08330	9.45553547
LOC_Os01g70220	LOC_Os08g06060	9.00116046
LOC_Os01g70300	LOC_Os03g12270	9.79506985
LOC_Os01g69080	LOC_Os11g47580	9.0882269
LOC_Os01g70300	LOC_Os09g32840	10.5182085
LOC_Os01g70300	LOC_Os11g47570	8.90000876
LOC_Os01g69080	LOC_Os08g34170	10.2357873
LOC_Os01g70220	LOC_Os12g44000	8.93024212
LOC_Os01g70220	LOC_Os04g47360	8.92321545
LOC_Os01g70300	LOC_Os02g53790	8.88902459
LOC_Os01g70220	LOC_Os04g39900	9.69769466
LOC_Os01g70220	LOC_Os09g10270	9.70611095
LOC_Os01g70220	LOC_Os01g71650	10.0363911
LOC_Os01g70220	LOC_Os07g46310	9.96782678
LOC_Os01g70770	LOC_Os06g40170	9.21843519
LOC_Os01g70220	LOC_Os07g38730	9.11371485
LOC_Os01g70525	LOC_Os09g30418	9.04536382
LOC_Os01g70140	LOC_Os05g02940	8.81055704
LOC_Os01g70220	LOC_Os04g25990	9.62396485
LOC_Os01g70220	LOC_Os05g34770	9.31716507
LOC_Os01g70300	LOC_Os11g32520	9.17525613
LOC_Os01g70220	LOC_Os07g39870	11.8059372
LOC_Os01g70220	LOC_Os09g10260	9.62556162
LOC_Os01g70220	LOC_Os11g32510	9.67646863
LOC_Os01g69080	LOC_Os04g33970	8.83832735
LOC_Os01g70140	LOC_Os03g31300	9.4004247
LOC_Os01g70220	LOC_Os08g39860	9.58832017
LOC_Os01g70300	LOC_Os02g08130	8.82493773
LOC_Os01g70300	LOC_Os10g08670	8.98993854
LOC_Os01g70300	LOC_Os04g53290	8.8432897
LOC_Os01g70525	LOC_Os02g33080	9.42692452
LOC_Os01g70140	LOC_Os10g26130	9.53317205
LOC_Os01g70300	LOC_Os06g08770	9.55943534
LOC_Os01g70300	LOC_Os04g34630	9.52664182
LOC_Os01g70525	LOC_Os06g37610	8.93945337
LOC_Os01g70220	LOC_Os03g28330	8.80514502
LOC_Os01g70300	LOC_Os02g10830	11.4402985
LOC_Os01g70220	LOC_Os07g31750	8.86257009
LOC_Os01g70300	LOC_Os03g62070	9.85233268
LOC_Os01g70140	LOC_Os04g43400	9.5808877
LOC_Os01g70220	LOC_Os05g04470	9.86806711
LOC_Os01g70525	LOC_Os05g19380	9.14349263

InteractorA	InteractorB	Z score
LOC_Os01g70140	LOC_Os12g34062	8.80989673
LOC_Os01g70770	LOC_Os06g12090	9.62951229
LOC_Os01g70220	LOC_Os07g03319	8.85740184
LOC_Os01g70300	LOC_Os06g37150	10.4145095
LOC_Os01g70140	LOC_Os03g04169	9.62013141
LOC_Os01g70220	LOC_Os12g07820	9.17366053
LOC_Os01g70300	LOC_Os06g46372	9.42457627
LOC_Os01g69080	LOC_Os05g08960	8.84452947
LOC_Os01g70220	LOC_Os08g39140	9.13603662
LOC_Os01g70300	LOC_Os10g02480	8.81199553
LOC_Os01g69080	LOC_Os06g36770	9.4033834
LOC_Os01g70770	LOC_Os05g04450	10.5917665
LOC_Os01g70220	LOC_Os10g40090	10.093003
LOC_Os01g70300	LOC_Os08g34210	9.47756664
LOC_Os01g70220	LOC_Os09g35800	11.1062844
LOC_Os01g70300	LOC_Os05g01970	8.82892895
LOC_Os01g70770	LOC_Os04g33040	9.18886632
LOC_Os01g70220	LOC_Os09g32620	9.61398216
LOC_Os01g69080	LOC_Os07g48430	10.4505177
LOC_Os01g70220	LOC_Os03g61330	10.28695
LOC_Os01g70220	LOC_Os04g12960	11.6136092
LOC_Os01g70770	LOC_Os05g06480	9.18300606
LOC_Os01g69080	LOC_Os07g26900	9.02650639
LOC_Os01g70220	LOC_Os11g10480	8.85200086
LOC_Os01g70220	LOC_Os03g05590	8.89752436
LOC_Os01g70220	LOC_Os05g01600	9.29772153
LOC_Os01g70300	LOC_Os04g57090	13.3349926
LOC_Os01g70525	LOC_Os06g07080	8.97394073
LOC_Os01g70220	LOC_Os07g43510	9.97853722
LOC_Os01g70770	LOC_Os06g45070	9.13020654
LOC_Os01g70300	LOC_Os04g54390	8.89773332
LOC_Os01g70220	LOC_Os05g38550	9.17415008
LOC_Os01g70300	LOC_Os04g01740	9.35546632
LOC_Os01g70220	LOC_Os07g03409	8.85740184
LOC_Os01g70220	LOC_Os06g24390	10.0736336
LOC_Os01g70140	LOC_Os04g52100	9.33610035
LOC_Os01g70300	LOC_Os02g14929	8.81564773
LOC_Os01g70300	LOC_Os09g19560	8.93035717
LOC_Os01g70525	LOC_Os09g26380	9.28961064
LOC_Os01g70770	LOC_Os03g14450	9.16077138
LOC_Os01g70525	LOC_Os08g15292	9.34297062
LOC_Os01g70300	LOC_Os03g18810	9.22206342
LOC_Os01g70770	LOC_Os06g30370	9.88952577

InteractorA	InteractorB	Z score
LOC_Os01g70220	LOC_Os10g29620	13.7457352
LOC_Os01g70300	LOC_Os02g47600	9.40310123
LOC_Os01g69080	LOC_Os06g50300	9.04245414
LOC_Os01g70525	LOC_Os04g55290	9.49811053
LOC_Os01g70220	LOC_Os02g51830	8.92184065
LOC_Os01g70300	LOC_Os01g72430	8.81019535
LOC_Os01g70220	LOC_Os07g03710	9.11880069
LOC_Os01g70770	LOC_Os06g44620	9.48483342
LOC_Os01g70300	LOC_Os02g12580	8.83208167
LOC_Os01g70220	LOC_Os07g03499	8.85740184
LOC_Os01g70220	LOC_Os12g17910	11.2347596
LOC_Os01g70300	LOC_Os09g36830	8.80472165
LOC_Os01g70300	LOC_Os10g41510	8.83369022
LOC_Os01g70220	LOC_Os03g29570	9.62971751
LOC_Os01g70300	LOC_Os03g63330	21.622249
LOC_Os01g70220	LOC_Os03g01800	9.02494026
LOC_Os01g70300	LOC_Os05g42190	8.81750915
LOC_Os01g70525	LOC_Os12g07980	9.53407281
LOC_Os01g70140	LOC_Os04g33040	9.73893629
LOC_Os01g70220	LOC_Os08g29170	11.745241
LOC_Os01g70300	LOC_Os02g41470	9.77359906
LOC_Os01g70300	LOC_Os10g29470	9.47674643
LOC_Os01g69080	LOC_Os12g35570	9.43194116
LOC_Os01g70220	LOC_Os12g01922	9.99339696
LOC_Os01g70300	LOC_Os02g38840	9.29006007
LOC_Os01g70220	LOC_Os09g30360	10.4451438
LOC_Os01g70300	LOC_Os07g20544	11.2024969
LOC_Os01g70220	LOC_Os03g17690	9.08088582
LOC_Os01g69080	LOC_Os04g10000	9.07431593
LOC_Os01g70220	LOC_Os06g35590	8.91300325
LOC_Os01g70220	LOC_Os05g33380	10.3226659
LOC_Os01g70220	LOC_Os12g34450	9.44269232
LOC_Os01g70220	LOC_Os04g45490	9.51923753
LOC_Os01g70220	LOC_Os03g31290	11.627278
LOC_Os01g69080	LOC_Os04g42250	9.42231374
LOC_Os01g70220	LOC_Os04g39880	8.80041793
LOC_Os01g70300	LOC_Os11g48110	8.82523288
LOC_Os01g69080	LOC_Os04g56070	9.01365178
LOC_Os01g70525	LOC_Os03g41438	8.81295767
LOC_Os01g71310	LOC_Os02g52390	8.90277827
LOC_Os01g71320	LOC_Os08g44210	9.55410605
LOC_Os01g71320	LOC_Os09g38030	10.5064252
LOC_Os01g71310	LOC_Os04g12480	9.32382347

InteractorA	InteractorB	Z score
LOC_Os01g71310	LOC_Os02g52230	9.06580944
LOC_Os01g71320	LOC_Os04g17650	8.9399846
LOC_Os01g71310	LOC_Os04g16760	8.97525615
LOC_Os01g71310	LOC_Os12g16240	10.0403071
LOC_Os01g71320	LOC_Os08g37490	9.18474415
LOC_Os01g71090	LOC_Os05g06750	9.51888016
LOC_Os01g71320	LOC_Os07g44740	9.86866049
LOC_Os01g70770	LOC_Os11g29190	9.41120657
LOC_Os01g71310	LOC_Os03g30870	8.9666431
LOC_Os01g71270	LOC_Os10g01080	9.30134881
LOC_Os01g71270	LOC_Os03g26960	9.15507823
LOC_Os01g71320	LOC_Os09g39780	8.99718452
LOC_Os01g71270	LOC_Os06g10340	9.07194356
LOC_Os01g71320	LOC_Os03g57120	9.01544855
LOC_Os01g71320	LOC_Os03g02710	8.8581528
LOC_Os01g71320	LOC_Os06g35490	9.29998616
LOC_Os01g71270	LOC_Os03g06620	8.87291712
LOC_Os01g71090	LOC_Os03g18510	8.92843484
LOC_Os01g71090	LOC_Os02g46970	9.06735723
LOC_Os01g71270	LOC_Os10g30200	9.56077435
LOC_Os01g71090	LOC_Os02g02410	9.64785592
LOC_Os01g70770	LOC_Os10g08550	9.3320906
LOC_Os01g71310	LOC_Os12g12514	9.49802212
LOC_Os01g71270	LOC_Os08g41990	11.1789559
LOC_Os01g70770	LOC_Os12g07720	9.83465266
LOC_Os01g71320	LOC_Os04g58110	9.21544794
LOC_Os01g70770	LOC_Os09g32952	8.80270798
LOC_Os01g71270	LOC_Os08g16910	9.49437781
LOC_Os01g71300	LOC_Os11g31620	11.3370282
LOC_Os01g71270	LOC_Os06g49970	10.0941098
LOC_Os01g70770	LOC_Os08g20270	9.47724302
LOC_Os01g70770	LOC_Os12g02060	9.00805177
LOC_Os01g71270	LOC_Os06g06300	8.82537088
LOC_Os01g71090	LOC_Os10g09860	8.93486356
LOC_Os01g71310	LOC_Os07g44450	9.59416712
LOC_Os01g71320	LOC_Os08g23730	9.22718551
LOC_Os01g71340	LOC_Os02g27760	9.08583905
LOC_Os01g71320	LOC_Os12g07980	8.87274818
LOC_Os01g71300	LOC_Os11g03980	8.93353459
LOC_Os01g71320	LOC_Os04g27860	8.84431959
LOC_Os01g71320	LOC_Os12g07720	9.5313878
LOC_Os01g71320	LOC_Os03g50440	9.52320122
LOC_Os01g71320	LOC_Os09g39810	9.68007651

InteractorA	InteractorB	Z score
LOC_Os01g71320	LOC_Os09g30418	9.19812034
LOC_Os01g71090	LOC_Os05g31020	10.2913982
LOC_Os01g71310	LOC_Os06g28550	10.2323759
LOC_Os01g71300	LOC_Os02g14059	9.82615967
LOC_Os01g71310	LOC_Os02g35310	9.25222073
LOC_Os01g71310	LOC_Os03g49350	8.90404267
LOC_Os01g71310	LOC_Os07g34190	8.87146087
LOC_Os01g71320	LOC_Os06g01360	9.62139164
LOC_Os01g71090	LOC_Os10g21248	9.36798875
LOC_Os01g71270	LOC_Os06g48310	9.56159233
LOC_Os01g71090	LOC_Os02g57630	9.60864044
LOC_Os01g71310	LOC_Os04g37950	8.84684605
LOC_Os01g71300	LOC_Os03g60580	9.49470938
LOC_Os01g71320	LOC_Os02g14110	9.32195169
LOC_Os01g71310	LOC_Os10g38229	9.25399671
LOC_Os01g70770	LOC_Os10g21250	8.97236477
LOC_Os01g71320	LOC_Os05g09490	9.46484956
LOC_Os01g71320	LOC_Os02g02890	9.79243772
LOC_Os01g71320	LOC_Os05g07880	9.08575857
LOC_Os01g71270	LOC_Os08g42410	10.9327833
LOC_Os01g71320	LOC_Os09g33860	9.92328608
LOC_Os01g71270	LOC_Os07g04240	9.7242349
LOC_Os01g71300	LOC_Os07g03690	8.88152083
LOC_Os01g71310	LOC_Os03g49380	9.10990046
LOC_Os01g71270	LOC_Os03g28330	9.23448724
LOC_Os01g71310	LOC_Os10g21266	9.6164978
LOC_Os01g71310	LOC_Os07g34140	9.25427131
LOC_Os01g71320	LOC_Os06g02380	9.99599447
LOC_Os01g71310	LOC_Os04g40310	8.81923201
LOC_Os01g70770	LOC_Os12g44150	9.39515177
LOC_Os01g71300	LOC_Os08g09250	8.96025892
LOC_Os01g71310	LOC_Os06g39740	10.1550137
LOC_Os01g71300	LOC_Os07g03730	9.10641913
LOC_Os01g71310	LOC_Os03g26960	8.80002772
LOC_Os01g71300	LOC_Os03g37950	9.1522793
LOC_Os01g71300	LOC_Os05g47545	9.04967414
LOC_Os01g71310	LOC_Os03g27370	11.2569593
LOC_Os01g71300	LOC_Os07g44370	9.15746203
LOC_Os01g71320	LOC_Os02g45540	9.51267965
LOC_Os01g71270	LOC_Os05g44760	9.68199883
LOC_Os01g71270	LOC_Os06g11800	9.27421985
LOC_Os01g70770	LOC_Os11g10480	9.60255637
LOC_Os01g71300	LOC_Os07g05400	9.12773264

InteractorA	InteractorB	Z score
LOC_Os01g71270	LOC_Os08g03290	8.96091743
LOC_Os01g71310	LOC_Os03g16230	8.87005229
LOC_Os01g71310	LOC_Os04g55740	9.70817721
LOC_Os01g71320	LOC_Os03g15360	8.97062863
LOC_Os01g70770	LOC_Os12g38770	9.5967236
LOC_Os01g71310	LOC_Os05g33410	9.06505445
LOC_Os01g71270	LOC_Os06g42560	9.21013889
LOC_Os01g71300	LOC_Os06g11280	12.1210168
LOC_Os01g71310	LOC_Os05g37700	8.92845975
LOC_Os01g71310	LOC_Os07g47990	8.83640874
LOC_Os01g71320	LOC_Os02g34600	9.10951498
LOC_Os01g71310	LOC_Os11g32650	8.87117057
LOC_Os01g71310	LOC_Os05g01675	8.97525615
LOC_Os01g71320	LOC_Os10g03540	9.77135131
LOC_Os01g71270	LOC_Os09g33500	9.96050726
LOC_Os01g71320	LOC_Os03g53800	8.96274735
LOC_Os01g71310	LOC_Os02g02210	9.70109507
LOC_Os01g71310	LOC_Os07g02340	9.01351087
LOC_Os01g71090	LOC_Os10g38660	8.845163
LOC_Os01g71270	LOC_Os04g56730	9.48624187
LOC_Os01g71310	LOC_Os05g45810	10.0817879
LOC_Os01g71350	LOC_Os09g27750	9.14450639
LOC_Os01g71380	LOC_Os03g55090	10.3813642
LOC_Os01g71340	LOC_Os03g57120	9.3145211
LOC_Os01g71400	LOC_Os07g34260	8.8193386
LOC_Os01g71380	LOC_Os03g31170	9.6854309
LOC_Os01g71410	LOC_Os02g10830	9.1217874
LOC_Os01g71340	LOC_Os03g01640	9.59791284
LOC_Os01g71410	LOC_Os03g22010	9.08831358
LOC_Os01g71380	LOC_Os04g10400	8.81405047
LOC_Os01g71410	LOC_Os02g49610	9.94077521
LOC_Os01g71410	LOC_Os02g10070	9.29433942
LOC_Os01g71474	LOC_Os02g18880	11.1300819
LOC_Os01g71380	LOC_Os02g08100	9.28956068
LOC_Os01g71380	LOC_Os09g34960	9.17866501
LOC_Os01g71340	LOC_Os08g06100	9.83957733
LOC_Os01g71474	LOC_Os03g49610	10.686189
LOC_Os01g71350	LOC_Os12g16200	8.9145541
LOC_Os01g71350	LOC_Os05g36280	9.11600881
LOC_Os01g71474	LOC_Os03g18130	9.65318999
LOC_Os01g71350	LOC_Os04g33570	8.94212884
LOC_Os01g71350	LOC_Os11g26860	9.07937227
LOC_Os01g71340	LOC_Os06g35530	9.7933873

InteractorA	InteractorB	Z score
LOC_Os01g71350	LOC_Os10g40710	9.57330855
LOC_Os01g71350	LOC_Os05g12190	8.96370213
LOC_Os01g71474	LOC_Os03g08800	9.31648638
LOC_Os01g71380	LOC_Os03g11970	9.52661143
LOC_Os01g71340	LOC_Os03g15360	9.16146464
LOC_Os01g71340	LOC_Os04g59200	10.5202563
LOC_Os01g71380	LOC_Os12g13390	8.89102785
LOC_Os01g71474	LOC_Os02g52700	10.2791327
LOC_Os01g71400	LOC_Os03g04260	10.2920053
LOC_Os01g71400	LOC_Os04g38600	8.95401378
LOC_Os01g71410	LOC_Os08g02410	10.4848494
LOC_Os01g71350	LOC_Os02g57770	8.84845056
LOC_Os01g71340	LOC_Os07g49120	9.51630929
LOC_Os01g71350	LOC_Os10g25930	10.6721938
LOC_Os01g71340	LOC_Os12g16290	9.09882173
LOC_Os01g71350	LOC_Os11g37960	8.8501776
LOC_Os01g71350	LOC_Os06g06460	9.11600881
LOC_Os01g71350	LOC_Os03g55150	9.63544003
LOC_Os01g71380	LOC_Os04g10010	9.01924218
LOC_Os01g71474	LOC_Os01g73190	9.06089675
LOC_Os01g71400	LOC_Os08g38900	8.98816678
LOC_Os01g71380	LOC_Os06g35730	8.97897554
LOC_Os01g71350	LOC_Os06g04200	9.71467227
LOC_Os01g71340	LOC_Os10g25950	10.9014763
LOC_Os01g71474	LOC_Os06g12990	9.35693681
LOC_Os01g71350	LOC_Os04g56400	9.23146506
LOC_Os01g71400	LOC_Os03g05290	9.1326638
LOC_Os01g71340	LOC_Os03g60620	8.87119382
LOC_Os01g71410	LOC_Os05g41060	9.16720376
LOC_Os01g71400	LOC_Os07g49120	9.24136271
LOC_Os01g71350	LOC_Os06g23440	8.88204106
LOC_Os01g71350	LOC_Os05g10780	9.06784281
LOC_Os01g71350	LOC_Os07g05940	9.08497201
LOC_Os01g71350	LOC_Os02g46970	10.4313042
LOC_Os01g71410	LOC_Os02g35310	8.88148111
LOC_Os01g71340	LOC_Os11g25100	9.79358852
LOC_Os01g71410	LOC_Os03g08570	9.3722743
LOC_Os01g71400	LOC_Os03g56840	9.42098133
LOC_Os01g71380	LOC_Os08g02700	9.91293506
LOC_Os01g71474	LOC_Os03g27310	8.95925189
LOC_Os01g71400	LOC_Os03g49220	9.89825567
LOC_Os01g71400	LOC_Os07g26900	9.56831838
LOC_Os01g71350	LOC_Os09g24990	9.30080739

InteractorA	InteractorB	Z score
LOC_Os01g71400	LOC_Os02g24634	9.14173504
LOC_Os01g71380	LOC_Os05g11710	8.97892254
LOC_Os01g71410	LOC_Os10g38350	9.76140996
LOC_Os01g71410	LOC_Os04g41960	9.06306238
LOC_Os01g71350	LOC_Os08g32620	11.0398711
LOC_Os01g71380	LOC_Os09g07830	9.05728509
LOC_Os01g71340	LOC_Os03g58260	9.02694295
LOC_Os01g71340	LOC_Os04g01980	9.10031898
LOC_Os01g71400	LOC_Os03g30950	10.3406114
LOC_Os01g71400	LOC_Os06g08770	10.377988
LOC_Os01g71380	LOC_Os03g04110	9.76388716
LOC_Os01g71410	LOC_Os04g39880	9.88174492
LOC_Os01g71474	LOC_Os06g35630	9.39661979
LOC_Os01g71380	LOC_Os11g25100	8.81110077
LOC_Os01g71400	LOC_Os03g49260	9.97651817
LOC_Os01g71400	LOC_Os10g21310	9.09741092
LOC_Os01g71400	LOC_Os11g08470	10.3439172
LOC_Os01g71410	LOC_Os04g18200	9.31495918
LOC_Os01g71474	LOC_Os05g33400	9.11878926
LOC_Os01g71350	LOC_Os03g49350	9.22687507
LOC_Os01g71474	LOC_Os06g04030	8.95925189
LOC_Os01g71474	LOC_Os06g11800	8.80813259
LOC_Os01g71380	LOC_Os04g28870	9.10673419
LOC_Os01g71340	LOC_Os09g08880	8.93277218
LOC_Os01g71410	LOC_Os03g18130	9.01506013
LOC_Os01g71400	LOC_Os12g23630	10.3579231
LOC_Os01g71400	LOC_Os11g37970	9.27598476
LOC_Os01g71380	LOC_Os04g41620	9.00353548
LOC_Os01g71350	LOC_Os02g14160	10.1815649
LOC_Os01g71410	LOC_Os07g02210	8.82016904
LOC_Os01g71474	LOC_Os04g52100	9.73244745
LOC_Os01g71474	LOC_Os02g14430	9.54106912
LOC_Os01g71410	LOC_Os04g56160	9.0188937
LOC_Os01g71474	LOC_Os04g10000	10.1050193
LOC_Os01g71474	LOC_Os04g41960	9.42106609
LOC_Os01g71474	LOC_Os03g42840	9.93190089
LOC_Os01g71400	LOC_Os12g06620	9.36064311
LOC_Os01g71400	LOC_Os04g55040	9.09020811
LOC_Os01g71380	LOC_Os04g59450	9.50690491
LOC_Os01g71380	LOC_Os09g25390	9.38265226
LOC_Os01g71340	LOC_Os03g03130	9.52963181
LOC_Os01g71340	LOC_Os05g05670	9.36597086
LOC_Os01g71340	LOC_Os07g01020	8.88946069

InteractorA	InteractorB	Z score
LOC_Os01g71400	LOC_Os11g16590	9.82344761
LOC_Os01g71350	LOC_Os06g06510	9.11600881
LOC_Os01g71380	LOC_Os10g10434	8.84243496
LOC_Os01g71380	LOC_Os03g64030	13.8312049
LOC_Os01g71350	LOC_Os01g71680	9.85123888
LOC_Os01g71380	LOC_Os03g05730	8.90118501
LOC_Os01g71400	LOC_Os07g07719	9.8644362
LOC_Os01g71400	LOC_Os07g05400	10.0315653
LOC_Os01g71380	LOC_Os01g72460	9.03695733
LOC_Os01g71350	LOC_Os07g43670	10.5845421
LOC_Os01g71474	LOC_Os04g33190	10.8388374
LOC_Os01g71350	LOC_Os12g02060	10.7763761
LOC_Os01g71400	LOC_Os03g47770	9.50304417
LOC_Os01g71400	LOC_Os04g52280	10.2929617
LOC_Os01g71400	LOC_Os09g10300	9.18648246
LOC_Os01g71340	LOC_Os09g31120	9.28320821
LOC_Os01g71400	LOC_Os09g34214	8.90817047
LOC_Os01g71474	LOC_Os05g05830	9.21706814
LOC_Os01g71400	LOC_Os02g53790	9.57253251
LOC_Os01g71380	LOC_Os02g18930	9.11178819
LOC_Os01g71400	LOC_Os08g27840	11.8964466
LOC_Os01g71350	LOC_Os06g29844	9.49223998
LOC_Os01g71340	LOC_Os10g27050	8.90822152
LOC_Os01g71400	LOC_Os08g02400	9.53248714
LOC_Os01g71400	LOC_Os06g11240	10.1970391
LOC_Os01g71474	LOC_Os02g52710	10.2791476
LOC_Os01g71350	LOC_Os11g05730	9.11600881
LOC_Os01g71474	LOC_Os01g74350	9.16927909
LOC_Os01g71350	LOC_Os07g48020	9.57185555
LOC_Os01g06740	LOC_Os03g08280	10.167657
LOC_Os01g07376	LOC_Os05g31110	9.05911839
LOC_Os01g06600	LOC_Os01g47550	8.92136627
LOC_Os01g07760	LOC_Os06g27770	8.8102301
LOC_Os01g06740	LOC_Os01g22249	9.0002021
LOC_Os01g07960	LOC_Os03g10500	9.11967305
LOC_Os01g06740	LOC_Os06g48180	9.41506418
LOC_Os01g07760	LOC_Os08g23730	9.44875511
LOC_Os01g06740	LOC_Os08g37490	9.2855307
LOC_Os01g07376	LOC_Os06g11200	9.25263199
LOC_Os01g07760	LOC_Os02g29020	10.1604799
LOC_Os01g07760	LOC_Os09g31490	9.45452748
LOC_Os01g06600	LOC_Os05g47980	9.54983963
LOC_Os01g07376	LOC_Os05g12180	9.79542964

InteractorA	InteractorB	Z score
LOC_Os01g06740	LOC_Os01g59490	10.1941355
LOC_Os01g07376	LOC_Os01g38970	8.84063397
LOC_Os01g07760	LOC_Os06g22140	9.29521426
LOC_Os01g07760	LOC_Os11g01360	9.61197879
LOC_Os01g06740	LOC_Os04g48850	9.56405989
LOC_Os01g06740	LOC_Os04g01600	9.78089526
LOC_Os01g06740	LOC_Os01g64680	9.05957318
LOC_Os01g07760	LOC_Os06g02390	8.91257307
LOC_Os01g06740	LOC_Os08g32620	8.96071484
LOC_Os01g06740	LOC_Os08g43190	9.01897975
LOC_Os01g06740	LOC_Os10g34760	9.94293647
LOC_Os01g07760	LOC_Os01g70220	10.2964215
LOC_Os01g07760	LOC_Os09g25370	8.82472536
LOC_Os01g06010	LOC_Os12g31370	10.0339058
LOC_Os01g07376	LOC_Os02g17390	9.37357197
LOC_Os01g06740	LOC_Os02g36974	9.25311459
LOC_Os01g07760	LOC_Os07g44430	9.32490027
LOC_Os01g07760	LOC_Os03g02710	9.07898843
LOC_Os01g06600	LOC_Os04g43400	8.88744428
LOC_Os01g07760	LOC_Os06g50300	8.88884093
LOC_Os01g07760	LOC_Os11g03980	10.5022574
LOC_Os01g07376	LOC_Os08g35440	9.55345881
LOC_Os01g07760	LOC_Os04g39900	10.359415
LOC_Os01g07760	LOC_Os06g41810	10.4161131
LOC_Os01g06740	LOC_Os04g48540	9.53755051
LOC_Os01g06600	LOC_Os06g10930	10.162438
LOC_Os01g07760	LOC_Os04g58710	8.8594598
LOC_Os01g06740	LOC_Os09g33500	8.84715354
LOC_Os01g07376	LOC_Os07g07320	9.91852421
LOC_Os01g07760	LOC_Os03g08280	9.46577027
LOC_Os01g07376	LOC_Os07g46310	10.8162148
LOC_Os01g07376	LOC_Os06g08600	9.5114167
LOC_Os01g06740	LOC_Os05g23740	9.23759087
LOC_Os01g07760	LOC_Os05g46290	11.0930244
LOC_Os01g06740	LOC_Os04g33480	9.40888756
LOC_Os01g07760	LOC_Os05g49840	10.7042205
LOC_Os01g07760	LOC_Os03g05980	9.10822672
LOC_Os01g07960	LOC_Os05g15520	9.87221919
LOC_Os01g06740	LOC_Os05g49830	9.75443216
LOC_Os01g06740	LOC_Os02g01340	11.0349527
LOC_Os01g07760	LOC_Os07g11440	8.93707832
LOC_Os01g07376	LOC_Os03g16110	8.90179718
LOC_Os01g07760	LOC_Os04g16874	9.01557017

InteractorA	InteractorB	Z score
LOC_Os01g07760	LOC_Os05g12240	9.11378265
LOC_Os01g07376	LOC_Os03g51600	8.8890791
LOC_Os01g06600	LOC_Os06g47320	9.23510395
LOC_Os01g07376	LOC_Os05g12240	9.01680757
LOC_Os01g07760	LOC_Os05g47640	9.6690973
LOC_Os01g07760	LOC_Os10g30840	9.63580036
LOC_Os01g07376	LOC_Os01g65090	10.8233574
LOC_Os01g06600	LOC_Os04g08350	9.22298443
LOC_Os01g06600	LOC_Os02g52590	9.52920278
LOC_Os01g07376	LOC_Os08g02700	10.0314654
LOC_Os01g06600	LOC_Os05g47640	9.22341057
LOC_Os01g06600	LOC_Os05g45420	9.56279474
LOC_Os01g06740	LOC_Os01g38970	9.62533848
LOC_Os01g07760	LOC_Os01g71310	8.96685467
LOC_Os01g07376	LOC_Os03g61970	9.91103055
LOC_Os01g06600	LOC_Os08g06550	9.57879298
LOC_Os01g06740	LOC_Os03g55620	9.43409194
LOC_Os01g07760	LOC_Os01g52470	9.62036285
LOC_Os01g06740	LOC_Os04g15920	8.95670405
LOC_Os01g07376	LOC_Os09g39400	8.93739225
LOC_Os01g06600	LOC_Os09g39400	9.37539312
LOC_Os01g07760	LOC_Os09g10300	9.50507893
LOC_Os01g06600	LOC_Os03g08010	8.93210621
LOC_Os01g06600	LOC_Os03g12270	8.98079122
LOC_Os01g07760	LOC_Os11g08445	9.08711659
LOC_Os01g07760	LOC_Os04g02050	9.07976304
LOC_Os01g07760	LOC_Os07g47990	9.45513761
LOC_Os01g06740	LOC_Os03g61340	9.75998265
LOC_Os01g06600	LOC_Os12g25630	8.88882785
LOC_Os01g06740	LOC_Os01g24680	9.00602935
LOC_Os01g06010	LOC_Os12g10720	8.90392099
LOC_Os01g06740	LOC_Os09g23540	9.71890116
LOC_Os01g07760	LOC_Os03g58260	9.93559178
LOC_Os01g06740	LOC_Os03g45320	9.21912682
LOC_Os01g06600	LOC_Os01g19450	9.53805531
LOC_Os01g06600	LOC_Os03g08020	8.93208864
LOC_Os01g06740	LOC_Os12g40510	9.78207868
LOC_Os01g07760	LOC_Os01g09430	10.2634925
LOC_Os01g06740	LOC_Os10g22310	9.1870762
LOC_Os01g06600	LOC_Os03g08050	8.93210122
LOC_Os01g07760	LOC_Os11g38959	9.10825147
LOC_Os01g07960	LOC_Os03g08530	8.90513736
LOC_Os01g06740	LOC_Os03g49610	8.97442683

InteractorA	InteractorB	Z score
LOC_Os01g07760	LOC_Os03g16920	9.03979531
LOC_Os01g06600	LOC_Os02g20850	8.9577623
LOC_Os01g06740	LOC_Os08g33710	9.80551855
LOC_Os01g07760	LOC_Os01g53280	9.00596441
LOC_Os01g07760	LOC_Os06g06980	9.93329367
LOC_Os01g06740	LOC_Os03g15120	10.4041853
LOC_Os01g71820	LOC_Os05g37690	10.0719438
LOC_Os01g71650	LOC_Os04g48540	9.2696587
LOC_Os01g71670	LOC_Os05g36290	9.55845092
LOC_Os01g71680	LOC_Os05g38230	10.4984887
LOC_Os01g71474	LOC_Os06g44620	10.0776202
LOC_Os01g71680	LOC_Os06g35660	10.4794345
LOC_Os01g71680	LOC_Os12g07720	8.84885571
LOC_Os01g71820	LOC_Os03g15120	9.55908423
LOC_Os01g71650	LOC_Os02g14130	9.16021013
LOC_Os01g71820	LOC_Os09g31486	9.62406628
LOC_Os01g71474	LOC_Os11g01010	8.84040917
LOC_Os01g71810	LOC_Os02g14770	9.05548352
LOC_Os01g71810	LOC_Os04g39210	9.59207818
LOC_Os01g71810	LOC_Os08g44530	10.6897732
LOC_Os01g71810	LOC_Os05g49770	9.99696292
LOC_Os01g71650	LOC_Os03g23970	10.0949801
LOC_Os01g71650	LOC_Os03g04060	9.45101326
LOC_Os01g71670	LOC_Os11g33240	9.4953746
LOC_Os01g71820	LOC_Os12g36950	9.48325402
LOC_Os01g71650	LOC_Os10g31940	8.96477648
LOC_Os01g71680	LOC_Os05g06970	8.90237473
LOC_Os01g71680	LOC_Os05g27950	9.82862715
LOC_Os01g71680	LOC_Os03g22060	10.415677
LOC_Os01g71820	LOC_Os05g31140	9.49070808
LOC_Os01g71670	LOC_Os05g38230	9.22215996
LOC_Os01g71474	LOC_Os08g44960	9.27743821
LOC_Os01g71820	LOC_Os08g09210	10.0436663
LOC_Os01g71680	LOC_Os06g07210	9.90821459
LOC_Os01g71820	LOC_Os10g08670	8.95465242
LOC_Os01g71474	LOC_Os11g02440	9.2091349
LOC_Os01g71680	LOC_Os11g36719	8.91289866
LOC_Os01g71670	LOC_Os02g18880	10.0411694
LOC_Os01g71680	LOC_Os05g07690	9.20150961
LOC_Os01g71670	LOC_Os03g58530	9.01012591
LOC_Os01g71680	LOC_Os01g73680	9.2413335
LOC_Os01g71474	LOC_Os07g32800	9.06914618
LOC_Os01g71670	LOC_Os05g45220	9.38262909

InteractorA	InteractorB	Z score
LOC_Os01g71820	LOC_Os06g02144	9.40355402
LOC_Os01g71680	LOC_Os08g27840	9.7356896
LOC_Os01g71810	LOC_Os03g13200	8.89947199
LOC_Os01g71680	LOC_Os03g01640	9.8568236
LOC_Os01g71810	LOC_Os07g34520	9.16001573
LOC_Os01g71820	LOC_Os04g01250	9.06016237
LOC_Os01g71670	LOC_Os03g17690	9.59426712
LOC_Os01g71680	LOC_Os02g33140	9.07276924
LOC_Os01g71820	LOC_Os11g41610	9.26693539
LOC_Os01g71670	LOC_Os04g55960	9.1026977
LOC_Os01g71810	LOC_Os06g21570	9.6844491
LOC_Os01g71820	LOC_Os03g56241	9.26693539
LOC_Os01g71820	LOC_Os09g26380	9.28186215
LOC_Os01g71820	LOC_Os05g07880	9.21852298
LOC_Os01g71810	LOC_Os05g30480	8.80616114
LOC_Os01g71820	LOC_Os08g35740	10.9932216
LOC_Os01g71810	LOC_Os03g58530	8.81715433
LOC_Os01g71820	LOC_Os04g43800	8.80720537
LOC_Os01g71680	LOC_Os04g55290	10.4738214
LOC_Os01g71680	LOC_Os05g09500	11.0554402
LOC_Os01g71680	LOC_Os04g12960	9.15126824
LOC_Os01g71670	LOC_Os06g21570	8.86675727
LOC_Os01g71474	LOC_Os12g08280	9.44910017
LOC_Os01g71650	LOC_Os06g10340	9.08975741
LOC_Os01g71670	LOC_Os09g39570	9.6172044
LOC_Os01g71670	LOC_Os08g41990	8.89207317
LOC_Os01g71680	LOC_Os02g38340	9.55362762
LOC_Os01g71650	LOC_Os10g35070	9.11028334
LOC_Os01g71650	LOC_Os11g41130	8.8104794
LOC_Os01g71820	LOC_Os08g04540	9.04474603
LOC_Os01g71820	LOC_Os03g49260	9.17187163
LOC_Os01g71650	LOC_Os02g40000	9.15982719
LOC_Os01g71680	LOC_Os04g35240	9.03292083
LOC_Os01g71670	LOC_Os04g12720	9.1965739
LOC_Os01g71680	LOC_Os05g45590	9.3561142
LOC_Os01g71680	LOC_Os04g01674	9.45391971
LOC_Os01g71650	LOC_Os05g04500	9.60432291
LOC_Os01g71820	LOC_Os10g26110	12.3508092
LOC_Os01g71680	LOC_Os12g12590	9.38495646
LOC_Os01g71810	LOC_Os07g38540	9.26433523
LOC_Os01g71670	LOC_Os09g10260	10.0716337
LOC_Os01g71810	LOC_Os05g04470	9.91908182
LOC_Os01g71670	LOC_Os02g27769	11.4871301

InteractorA	InteractorB	Z score
LOC_Os01g71680	LOC_Os04g32710	9.33208277
LOC_Os01g71670	LOC_Os03g17470	8.88771507
LOC_Os01g71670	LOC_Os09g10270	9.25511231
LOC_Os01g71650	LOC_Os10g26130	9.29485361
LOC_Os01g71680	LOC_Os03g50440	8.81415895
LOC_Os01g71820	LOC_Os06g22140	8.9145288
LOC_Os01g71820	LOC_Os03g58530	9.79839315
LOC_Os01g71810	LOC_Os06g49470	8.84198614
LOC_Os01g71670	LOC_Os05g19380	8.85142263
LOC_Os01g71650	LOC_Os07g40580	9.06981187
LOC_Os01g71680	LOC_Os06g11210	8.91893082
LOC_Os01g71810	LOC_Os11g03230	9.06424756
LOC_Os01g71810	LOC_Os04g52100	9.45297453
LOC_Os01g71680	LOC_Os10g23900	8.93925147
LOC_Os01g71474	LOC_Os09g16910	8.83398762
LOC_Os01g71670	LOC_Os11g08440	8.82795319
LOC_Os01g71680	LOC_Os07g29750	9.01668752
LOC_Os01g71820	LOC_Os02g35760	8.85959686
LOC_Os01g71810	LOC_Os03g30950	8.99611767
LOC_Os01g71810	LOC_Os06g10930	9.57741832
LOC_Os01g71670	LOC_Os04g48700	9.00895717
LOC_Os01g71474	LOC_Os11g24560	8.99807077
LOC_Os01g71810	LOC_Os08g44280	9.04117178
LOC_Os01g71820	LOC_Os03g01170	8.93554131
LOC_Os01g71820	LOC_Os02g10390	9.28279397
LOC_Os01g71680	LOC_Os12g23630	10.5383859
LOC_Os01g71474	LOC_Os11g25330	9.02821371
LOC_Os01g71820	LOC_Os03g04169	9.78016165
LOC_Os01g71820	LOC_Os09g20820	8.84802334
LOC_Os01g71670	LOC_Os03g46070	9.15647046
LOC_Os01g71680	LOC_Os05g25550	8.83484646
LOC_Os01g71474	LOC_Os10g41510	9.08857713
LOC_Os01g71820	LOC_Os03g47610	9.16313446
LOC_Os01g71830	LOC_Os02g22780	10.1587511
LOC_Os01g72800	LOC_Os04g02050	9.80527498
LOC_Os01g71990	LOC_Os09g31490	9.92959264
LOC_Os01g71990	LOC_Os07g46310	12.548662
LOC_Os01g72800	LOC_Os10g38340	9.20006837
LOC_Os01g71830	LOC_Os02g51100	9.46509092
LOC_Os01g71990	LOC_Os07g47490	8.95363035
LOC_Os01g71830	LOC_Os04g28870	8.9314776
LOC_Os01g71830	LOC_Os05g46290	8.91462353
LOC_Os01g71990	LOC_Os01g72460	8.89017411

InteractorA	InteractorB	Z score
LOC_Os01g72800	LOC_Os08g33710	9.1285233
LOC_Os01g72430	LOC_Os03g09080	10.1790908
LOC_Os01g71990	LOC_Os08g09940	9.81858911
LOC_Os01g71990	LOC_Os03g17690	9.22702846
LOC_Os01g71830	LOC_Os04g55290	11.6124878
LOC_Os01g71830	LOC_Os03g40330	9.16745537
LOC_Os01g71830	LOC_Os03g31300	9.29283549
LOC_Os01g71830	LOC_Os05g04470	9.58113797
LOC_Os01g71990	LOC_Os09g38620	8.82913655
LOC_Os01g72430	LOC_Os03g05980	9.53274476
LOC_Os01g73140	LOC_Os03g45320	9.29997993
LOC_Os01g71990	LOC_Os11g32580	9.97357693
LOC_Os01g72800	LOC_Os10g21240	10.1594237
LOC_Os01g72800	LOC_Os01g73980	9.19650059
LOC_Os01g71830	LOC_Os02g46970	9.12583325
LOC_Os01g71830	LOC_Os12g44000	8.85013082
LOC_Os01g71990	LOC_Os06g04030	9.53964784
LOC_Os01g71830	LOC_Os05g38230	8.86071893
LOC_Os01g72800	LOC_Os02g27940	9.16677393
LOC_Os01g73140	LOC_Os02g56460	9.33269458
LOC_Os01g72460	LOC_Os07g26640	8.82482606
LOC_Os01g71990	LOC_Os03g12520	10.0651488
LOC_Os01g71990	LOC_Os04g42920	9.43865954
LOC_Os01g72430	LOC_Os06g10910	8.83062984
LOC_Os01g71990	LOC_Os07g26540	9.46098732
LOC_Os01g71990	LOC_Os02g10310	9.23446388
LOC_Os01g72800	LOC_Os05g49800	9.66133486
LOC_Os01g72800	LOC_Os07g07320	9.44193255
LOC_Os01g71990	LOC_Os07g42960	9.26181158
LOC_Os01g72460	LOC_Os05g46270	8.97634105
LOC_Os01g71990	LOC_Os09g14670	8.8030274
LOC_Os01g72430	LOC_Os04g12480	9.43677605
LOC_Os01g72460	LOC_Os03g12270	9.02536849
LOC_Os01g71830	LOC_Os06g35630	9.77146862
LOC_Os01g71990	LOC_Os03g27310	9.53963035
LOC_Os01g71830	LOC_Os05g05800	8.83868415
LOC_Os01g73140	LOC_Os05g28180	9.51197044
LOC_Os01g71830	LOC_Os09g25150	8.84461002
LOC_Os01g73140	LOC_Os03g08530	9.21569577
LOC_Os01g72430	LOC_Os11g38959	9.53274082
LOC_Os01g71830	LOC_Os06g01390	9.05842944
LOC_Os01g72460	LOC_Os08g33710	9.81006754
LOC_Os01g72800	LOC_Os02g08490	9.15396381

InteractorA	InteractorB	Z score
LOC_Os01g72800	LOC_Os06g25010	9.03729468
LOC_Os01g72800	LOC_Os06g04200	9.00774481
LOC_Os01g72800	LOC_Os03g19930	9.93090526
LOC_Os01g71990	LOC_Os03g05730	8.81035648
LOC_Os01g72460	LOC_Os07g05800	10.5873778
LOC_Os01g71830	LOC_Os04g10400	9.67863341
LOC_Os01g71990	LOC_Os10g06630	9.03094093
LOC_Os01g72800	LOC_Os05g19380	8.90373682
LOC_Os01g71830	LOC_Os04g33740	9.02516583
LOC_Os01g73140	LOC_Os03g26450	9.01525034
LOC_Os01g71990	LOC_Os08g33710	9.96289983
LOC_Os01g71830	LOC_Os07g40290	9.50696346
LOC_Os01g72800	LOC_Os06g46284	9.17350094
LOC_Os01g71830	LOC_Os11g01010	9.06077878
LOC_Os01g72800	LOC_Os07g43170	8.91541182
LOC_Os01g71990	LOC_Os04g02050	10.3766762
LOC_Os01g72800	LOC_Os05g26890	10.3045731
LOC_Os01g71990	LOC_Os02g01510	10.6647055
LOC_Os01g72430	LOC_Os03g46070	8.84484666
LOC_Os01g71990	LOC_Os01g74350	8.88195643
LOC_Os01g72430	LOC_Os10g11810	9.15032626
LOC_Os01g72800	LOC_Os05g31040	9.59361164
LOC_Os01g72460	LOC_Os10g30200	10.3031042
LOC_Os01g72460	LOC_Os03g47770	9.71994587
LOC_Os01g71990	LOC_Os05g25550	9.27880531
LOC_Os01g71990	LOC_Os04g18200	8.84184442
LOC_Os01g72430	LOC_Os05g40420	9.20064876
LOC_Os01g72430	LOC_Os09g10270	9.30932039
LOC_Os01g71990	LOC_Os07g05800	9.64390181
LOC_Os01g73140	LOC_Os01g73580	9.10554158
LOC_Os01g71990	LOC_Os10g32970	12.5078275
LOC_Os01g73140	LOC_Os05g41080	8.8516961
LOC_Os01g72800	LOC_Os06g35480	9.10340656
LOC_Os01g72800	LOC_Os08g28680	9.69033523
LOC_Os01g72460	LOC_Os12g08270	12.358159
LOC_Os01g71990	LOC_Os12g44000	8.87828369
LOC_Os01g73140	LOC_Os03g05590	10.6247505
LOC_Os01g71990	LOC_Os02g10390	9.36662954
LOC_Os01g73140	LOC_Os04g18650	8.92012444
LOC_Os01g71990	LOC_Os09g25150	9.1130607
LOC_Os01g71990	LOC_Os05g05670	10.603665
LOC_Os01g71990	LOC_Os05g04340	9.69484685
LOC_Os01g73310	LOC_Os05g36010	8.8503208

InteractorA	InteractorB	Z score
LOC_Os01g73190	LOC_Os12g13380	8.87995798
LOC_Os01g73170	LOC_Os03g02920	10.5131558
LOC_Os01g73170	LOC_Os11g19220	8.8962211
LOC_Os01g73220	LOC_Os06g45710	9.02162047
LOC_Os01g73310	LOC_Os03g55070	8.82022662
LOC_Os01g73310	LOC_Os03g17690	10.829911
LOC_Os01g73220	LOC_Os06g28550	9.95872228
LOC_Os01g73170	LOC_Os04g43760	9.00468516
LOC_Os01g73170	LOC_Os04g19740	9.35032975
LOC_Os01g73220	LOC_Os08g34170	9.47388536
LOC_Os01g73190	LOC_Os11g05570	9.19055215
LOC_Os01g73220	LOC_Os10g38234	8.93788673
LOC_Os01g73220	LOC_Os05g06300	10.1322512
LOC_Os01g73190	LOC_Os05g41640	10.3201303
LOC_Os01g73170	LOC_Os11g32650	9.35821506
LOC_Os01g73310	LOC_Os04g52340	9.27304884
LOC_Os01g73200	LOC_Os04g55040	10.0920593
LOC_Os01g73190	LOC_Os02g07260	8.85536156
LOC_Os01g73190	LOC_Os02g53790	9.34762089
LOC_Os01g73170	LOC_Os07g44460	10.5121239
LOC_Os01g73220	LOC_Os04g43410	8.80304671
LOC_Os01g73170	LOC_Os03g44170	9.10808378
LOC_Os01g73220	LOC_Os06g37180	9.33299759
LOC_Os01g73190	LOC_Os11g32260	8.84554726
LOC_Os01g73140	LOC_Os10g29470	9.04867174
LOC_Os01g73220	LOC_Os11g08330	9.06032117
LOC_Os01g73220	LOC_Os07g05160	9.47749654
LOC_Os01g73310	LOC_Os04g12720	8.87200924
LOC_Os01g73190	LOC_Os03g45410	9.58488998
LOC_Os01g73220	LOC_Os02g12730	12.2999454
LOC_Os01g73140	LOC_Os12g07720	11.1468049
LOC_Os01g73140	LOC_Os11g29400	9.87872394
LOC_Os01g73310	LOC_Os02g55400	9.24726899
LOC_Os01g73200	LOC_Os05g49760	10.3163589
LOC_Os01g73140	LOC_Os08g14770	9.19434349
LOC_Os01g73200	LOC_Os12g07980	8.99990083
LOC_Os01g73310	LOC_Os02g12780	9.18009198
LOC_Os01g73220	LOC_Os12g43630	8.81084758
LOC_Os01g73310	LOC_Os07g37790	9.27307067
LOC_Os01g73190	LOC_Os02g49720	9.06475291
LOC_Os01g73190	LOC_Os02g42810	9.17350454
LOC_Os01g73220	LOC_Os04g16680	9.03506241
LOC_Os01g73220	LOC_Os02g36710	8.97147968

InteractorA	InteractorB	Z score
LOC_Os01g73170	LOC_Os04g39840	11.342675
LOC_Os01g73170	LOC_Os04g24430	9.65816351
LOC_Os01g73310	LOC_Os05g38530	9.0124905
LOC_Os01g73310	LOC_Os02g14130	8.97991996
LOC_Os01g73170	LOC_Os03g52860	8.98230556
LOC_Os01g73170	LOC_Os07g05180	9.52670862
LOC_Os01g73190	LOC_Os07g41050	9.03305702
LOC_Os01g73310	LOC_Os11g42350	8.82207818
LOC_Os01g73170	LOC_Os04g44920	8.85792228
LOC_Os01g73190	LOC_Os07g37320	10.748971
LOC_Os01g73200	LOC_Os02g58340	8.95947702
LOC_Os01g73220	LOC_Os07g38860	10.8004347
LOC_Os01g73310	LOC_Os12g21798	10.058975
LOC_Os01g73170	LOC_Os07g44550	9.49098729
LOC_Os01g73200	LOC_Os12g23630	9.41041568
LOC_Os01g73170	LOC_Os03g44484	10.1075775
LOC_Os01g73170	LOC_Os03g64330	10.0181372
LOC_Os01g73170	LOC_Os10g21266	9.28126391
LOC_Os01g73310	LOC_Os08g03440	9.55627318
LOC_Os01g73190	LOC_Os08g06100	9.69466556
LOC_Os01g73200	LOC_Os08g28820	8.95806752
LOC_Os01g73140	LOC_Os09g14670	8.99601719
LOC_Os01g73200	LOC_Os09g10300	9.27773831
LOC_Os01g73200	LOC_Os07g48880	9.93711526
LOC_Os01g73190	LOC_Os03g17470	8.94303227
LOC_Os01g73220	LOC_Os05g04490	8.86015062
LOC_Os01g73310	LOC_Os10g38640	9.19149437
LOC_Os01g73310	LOC_Os05g45590	10.5254272
LOC_Os01g73190	LOC_Os08g01660	8.97575292
LOC_Os01g73310	LOC_Os07g34580	10.3314739
LOC_Os01g73190	LOC_Os08g28820	8.87988239
LOC_Os01g73190	LOC_Os06g50300	9.7797021
LOC_Os01g73310	LOC_Os11g08440	9.73404521
LOC_Os01g73310	LOC_Os04g48850	10.2678347
LOC_Os01g73190	LOC_Os10g20910	8.82103988
LOC_Os01g73190	LOC_Os03g04410	9.83823247
LOC_Os01g73200	LOC_Os10g03540	9.1654371
LOC_Os01g73220	LOC_Os02g22780	8.934779
LOC_Os01g73220	LOC_Os12g44000	9.57270124
LOC_Os01g73200	LOC_Os04g55740	9.10720328
LOC_Os01g73310	LOC_Os12g17540	10.9502746
LOC_Os01g73200	LOC_Os11g02100	9.15746558
LOC_Os01g73220	LOC_Os02g39850	10.0796115

InteractorA	InteractorB	Z score
LOC_Os01g73310	LOC_Os04g14680	9.13425479
LOC_Os01g73200	LOC_Os02g14460	9.19063607
LOC_Os01g73310	LOC_Os06g04200	9.180756
LOC_Os01g73170	LOC_Os07g35940	9.53203415
LOC_Os01g73170	LOC_Os12g43440	9.08735936
LOC_Os01g73140	LOC_Os06g46284	9.11770112
LOC_Os01g73190	LOC_Os02g47590	9.37048898
LOC_Os01g73310	LOC_Os05g07880	9.58598553
LOC_Os01g73220	LOC_Os12g01922	9.36386474
LOC_Os01g73190	LOC_Os04g22730	9.75464438
LOC_Os01g74000	LOC_Os03g13180	8.94692751
LOC_Os01g74000	LOC_Os02g16040	9.07099776
LOC_Os01g73740	LOC_Os02g14160	9.23876233
LOC_Os01g73580	LOC_Os03g19390	10.1139035
LOC_Os01g73580	LOC_Os02g50240	11.1660017
LOC_Os01g73980	LOC_Os06g08310	9.34384474
LOC_Os01g73740	LOC_Os03g15880	10.2350686
LOC_Os01g73790	LOC_Os03g01640	9.36754722
LOC_Os01g73580	LOC_Os08g17784	9.8228121
LOC_Os01g73580	LOC_Os08g35740	9.33463875
LOC_Os01g73740	LOC_Os02g55400	9.92509798
LOC_Os01g73580	LOC_Os02g52710	9.86566927
LOC_Os01g73580	LOC_Os07g30200	9.41351574
LOC_Os01g73680	LOC_Os03g01610	9.44078249
LOC_Os01g73580	LOC_Os03g22060	9.66608587
LOC_Os01g73740	LOC_Os04g40130	9.93276971
LOC_Os01g73980	LOC_Os11g43360	8.91845805
LOC_Os01g73980	LOC_Os12g17910	9.22311563
LOC_Os01g73740	LOC_Os06g06460	8.99509146
LOC_Os01g73680	LOC_Os05g02310	9.28907891
LOC_Os01g73680	LOC_Os03g01260	9.2435385
LOC_Os01g74000	LOC_Os03g56810	10.2715225
LOC_Os01g73740	LOC_Os03g58400	9.03561895
LOC_Os01g73580	LOC_Os12g17540	9.04270477
LOC_Os01g73580	LOC_Os12g44000	8.91926853
LOC_Os01g73680	LOC_Os06g41810	9.78566639
LOC_Os01g73580	LOC_Os06g05880	8.87620228
LOC_Os01g73740	LOC_Os04g41310	8.8336997
LOC_Os01g73580	LOC_Os03g53200	10.9946823
LOC_Os01g73740	LOC_Os07g38030	10.0686279
LOC_Os01g73580	LOC_Os05g44760	9.28561323
LOC_Os01g73580	LOC_Os02g52700	9.86570265
LOC_Os01g73980	LOC_Os03g15120	9.14047922

InteractorA	InteractorB	Z score
LOC_Os01g73680	LOC_Os04g59040	8.96183594
LOC_Os01g73980	LOC_Os03g51740	9.46277753
LOC_Os01g73580	LOC_Os11g32620	9.6354599
LOC_Os01g73580	LOC_Os04g55410	9.26124337
LOC_Os01g73680	LOC_Os03g57290	10.6553065
LOC_Os01g73980	LOC_Os10g31940	8.88092665
LOC_Os01g73980	LOC_Os04g53230	8.8187294
LOC_Os01g73580	LOC_Os03g58260	10.3719769
LOC_Os01g73680	LOC_Os07g02340	8.81134275
LOC_Os01g73680	LOC_Os11g34570	9.82166539
LOC_Os01g73580	LOC_Os09g27820	8.93084786
LOC_Os01g73580	LOC_Os04g02820	9.59637489
LOC_Os01g73740	LOC_Os04g45290	9.01158364
LOC_Os01g73680	LOC_Os02g49720	8.8184564
LOC_Os01g73680	LOC_Os03g01650	9.44079818
LOC_Os01g73580	LOC_Os02g08130	9.8850508
LOC_Os01g73790	LOC_Os05g28280	9.34617776
LOC_Os01g73680	LOC_Os12g13390	8.94025422
LOC_Os01g74000	LOC_Os05g10780	10.6250761
LOC_Os01g73680	LOC_Os05g09490	8.80733239
LOC_Os01g73680	LOC_Os03g56460	8.82117519
LOC_Os01g73790	LOC_Os09g10260	9.11173951
LOC_Os01g73580	LOC_Os05g47640	9.70913123
LOC_Os01g73580	LOC_Os04g47170	9.79029574
LOC_Os01g73580	LOC_Os04g59600	9.4423339
LOC_Os01g73980	LOC_Os03g06200	9.24348142
LOC_Os01g73740	LOC_Os02g14929	9.34852957
LOC_Os01g73980	LOC_Os03g50490	9.5045441
LOC_Os01g73790	LOC_Os12g12514	12.7314766
LOC_Os01g73790	LOC_Os03g16900	9.52636388
LOC_Os01g73680	LOC_Os05g27950	8.97593764
LOC_Os01g73980	LOC_Os12g43100	8.80057058
LOC_Os01g73980	LOC_Os05g06450	9.80644506
LOC_Os01g73790	LOC_Os06g51084	9.93574217
LOC_Os01g73740	LOC_Os07g10590	9.40322014
LOC_Os01g73680	LOC_Os04g56580	9.79125536
LOC_Os01g73680	LOC_Os04g49130	10.4964613
LOC_Os01g74000	LOC_Os04g16772	9.72055382
LOC_Os01g73580	LOC_Os06g06560	8.88636021
LOC_Os01g74000	LOC_Os03g18580	9.03803202
LOC_Os01g73790	LOC_Os12g13390	9.15775795
LOC_Os01g73980	LOC_Os04g12900	9.77444376
LOC_Os01g73680	LOC_Os04g47360	8.97519406

InteractorA	InteractorB	Z score
LOC_Os01g73790	LOC_Os12g25690	9.20133738
LOC_Os01g73580	LOC_Os08g28800	9.58191636
LOC_Os01g73680	LOC_Os02g42520	9.36372934
LOC_Os01g73980	LOC_Os12g23170	9.42686838
LOC_Os01g74000	LOC_Os02g18880	9.11757728
LOC_Os01g73740	LOC_Os06g06090	9.44409821
LOC_Os01g73980	LOC_Os03g16920	10.0780987
LOC_Os01g73980	LOC_Os05g29880	9.16150235
LOC_Os01g73580	LOC_Os08g04560	9.24946386
LOC_Os01g73980	LOC_Os03g11420	9.06425049
LOC_Os01g73680	LOC_Os04g32460	9.28681226
LOC_Os01g73740	LOC_Os09g23530	9.30792594
LOC_Os01g74000	LOC_Os04g16818	9.72055461
LOC_Os01g73680	LOC_Os10g38590	10.2683127
LOC_Os01g73680	LOC_Os03g47770	8.88568524
LOC_Os01g73740	LOC_Os04g12970	9.4022855
LOC_Os01g73980	LOC_Os06g11240	8.96761222
LOC_Os01g73980	LOC_Os08g41990	8.89635292
LOC_Os01g73580	LOC_Os10g30580	8.9042667
LOC_Os01g73980	LOC_Os02g12730	9.0798551
LOC_Os01g73580	LOC_Os02g14180	9.66340724
LOC_Os01g73580	LOC_Os09g23550	10.161609
LOC_Os01g73740	LOC_Os02g14440	9.46057685
LOC_Os01g73980	LOC_Os02g52390	8.80378264
LOC_Os01g73580	LOC_Os02g39850	8.81730681
LOC_Os01g73680	LOC_Os03g09250	9.92075654
LOC_Os01g73740	LOC_Os09g32840	8.81922145
LOC_Os01g73790	LOC_Os07g05180	9.03493543
LOC_Os01g73680	LOC_Os03g09080	8.86404185
LOC_Os01g73580	LOC_Os08g08500	10.224106
LOC_Os01g73580	LOC_Os06g06980	10.4941175
LOC_Os01g73740	LOC_Os05g36280	8.99509146
LOC_Os01g73980	LOC_Os03g52840	9.10943775
LOC_Os01g73980	LOC_Os10g29470	9.30573932
LOC_Os01g74000	LOC_Os02g08490	9.39584767
LOC_Os01g73740	LOC_Os03g25360	10.0657684
LOC_Os01g73580	LOC_Os08g43170	10.278277
LOC_Os01g73580	LOC_Os02g42810	10.5554026
LOC_Os01g73980	LOC_Os07g48040	8.86596387
LOC_Os01g73680	LOC_Os03g55090	9.80229777
LOC_Os01g73680	LOC_Os04g10010	11.5006564
LOC_Os01g73680	LOC_Os03g22530	9.80691893
LOC_Os01g73680	LOC_Os12g12514	9.06380426

InteractorA	InteractorB	Z score
LOC_Os01g74000	LOC_Os03g45960	9.28037247
LOC_Os01g73740	LOC_Os11g05730	8.99509146
LOC_Os01g73580	LOC_Os04g44870	9.11530691
LOC_Os01g73790	LOC_Os09g10200	9.13090886
LOC_Os01g73580	LOC_Os05g31020	9.05465586
LOC_Os01g73740	LOC_Os07g44590	9.48240272
LOC_Os01g73740	LOC_Os12g02370	9.94411602
LOC_Os01g73680	LOC_Os09g31502	10.4304965
LOC_Os01g74000	LOC_Os02g55300	10.5613509
LOC_Os01g73980	LOC_Os05g41230	8.86724744
LOC_Os01g73580	LOC_Os05g48980	8.94516042
LOC_Os01g73790	LOC_Os02g32370	10.5863893
LOC_Os01g73980	LOC_Os06g10930	9.27165082
LOC_Os01g73580	LOC_Os08g37790	9.09124497
LOC_Os01g73680	LOC_Os05g48030	8.99497023
LOC_Os01g73740	LOC_Os08g16910	8.8148862
LOC_Os01g73740	LOC_Os06g06510	8.99509146
LOC_Os01g73580	LOC_Os06g36160	9.42964971
LOC_Os01g74000	LOC_Os04g45490	9.17979618
LOC_Os01g73790	LOC_Os03g56810	8.86695541
LOC_Os01g73580	LOC_Os05g38550	10.7744057
LOC_Os01g73740	LOC_Os05g45590	9.25441675
LOC_Os01g73790	LOC_Os06g35530	9.10920671
LOC_Os01g73680	LOC_Os02g08100	8.98524218
LOC_Os01g73790	LOC_Os05g37390	10.0617175
LOC_Os01g73580	LOC_Os07g06410	8.82808266
LOC_Os01g74350	LOC_Os11g08940	9.97722381
LOC_Os02g01160	LOC_Os02g04950	8.93997126
LOC_Os01g74350	LOC_Os04g41960	9.35423399
LOC_Os01g74650	LOC_Os06g36840	9.27956852
LOC_Os01g74350	LOC_Os12g12590	9.84547314
LOC_Os01g74000	LOC_Os07g06970	10.3351069
LOC_Os02g01160	LOC_Os05g45590	9.02728523
LOC_Os02g01160	LOC_Os04g28870	9.44998711
LOC_Os01g74450	LOC_Os03g45320	9.71799425
LOC_Os02g01220	LOC_Os05g35400	9.19809556
LOC_Os01g74450	LOC_Os05g51570	9.42181125
LOC_Os02g01160	LOC_Os03g01630	9.1660732
LOC_Os01g74350	LOC_Os04g42920	8.93323539
LOC_Os02g01220	LOC_Os09g11230	9.63373978
LOC_Os02g01160	LOC_Os09g08120	9.07009605
LOC_Os02g01160	LOC_Os04g30420	9.21784637
LOC_Os01g74350	LOC_Os07g48040	10.7123972

InteractorA	InteractorB	Z score
LOC_Os02g01220	LOC_Os06g34690	9.42481401
LOC_Os02g01160	LOC_Os02g55400	8.99355387
LOC_Os02g01160	LOC_Os07g14590	8.98736185
LOC_Os02g01220	LOC_Os03g15120	9.05978066
LOC_Os02g01220	LOC_Os09g28420	10.9428729
LOC_Os02g01160	LOC_Os03g55800	10.018493
LOC_Os02g01220	LOC_Os02g14110	9.09686899
LOC_Os01g74350	LOC_Os08g44960	9.75244191
LOC_Os01g74350	LOC_Os02g51830	8.85838164
LOC_Os01g74350	LOC_Os04g55960	9.12040945
LOC_Os01g74650	LOC_Os05g42150	8.91076808
LOC_Os01g74000	LOC_Os10g30200	8.94507714
LOC_Os01g74000	LOC_Os08g15266	9.72055382
LOC_Os02g01220	LOC_Os11g03980	9.00549166
LOC_Os02g01220	LOC_Os05g31110	10.7992047
LOC_Os02g01220	LOC_Os02g18880	11.235836
LOC_Os01g74000	LOC_Os09g24412	9.72055461
LOC_Os01g74000	LOC_Os11g47600	9.31616465
LOC_Os02g01220	LOC_Os05g49760	9.74207478
LOC_Os02g01160	LOC_Os06g10930	10.2408292
LOC_Os01g74650	LOC_Os03g05980	8.92175144
LOC_Os01g74450	LOC_Os11g32510	10.1927327
LOC_Os02g01220	LOC_Os04g37950	9.38779537
LOC_Os02g01220	LOC_Os05g44050	9.08680525
LOC_Os02g01220	LOC_Os04g16740	9.41385127
LOC_Os01g74350	LOC_Os03g04970	8.83185152
LOC_Os01g74650	LOC_Os11g38959	8.92174659
LOC_Os02g01160	LOC_Os06g06100	8.97314628
LOC_Os02g01160	LOC_Os05g36270	8.92860142
LOC_Os02g01160	LOC_Os02g38340	9.23372489
LOC_Os02g01220	LOC_Os09g12570	8.89787459
LOC_Os02g01160	LOC_Os03g49220	10.1694131
LOC_Os02g01160	LOC_Os12g14070	9.13345234
LOC_Os02g01220	LOC_Os04g39840	9.30386367
LOC_Os01g74000	LOC_Os10g21352	9.72055382
LOC_Os01g74450	LOC_Os03g42840	9.24776149
LOC_Os02g01160	LOC_Os03g61330	10.48214
LOC_Os01g74000	LOC_Os06g19960	8.90151791
LOC_Os02g01160	LOC_Os02g41470	10.1459823
LOC_Os01g74350	LOC_Os10g25140	8.82574011
LOC_Os01g74000	LOC_Os07g25024	9.72055382
LOC_Os02g01220	LOC_Os06g10910	9.3371259
LOC_Os02g01160	LOC_Os10g29620	9.05329749

InteractorA	InteractorB	Z score
LOC_Os02g01220	LOC_Os02g18930	9.91697394
LOC_Os01g74350	LOC_Os07g43670	9.19435537
LOC_Os01g74350	LOC_Os06g05700	9.00379472
LOC_Os02g01220	LOC_Os05g46270	9.87833839
LOC_Os02g01160	LOC_Os04g39210	9.05901397
LOC_Os01g74350	LOC_Os11g08120	10.6074155
LOC_Os01g74350	LOC_Os05g08100	9.41738717
LOC_Os02g01160	LOC_Os09g39380	9.16151862
LOC_Os01g74350	LOC_Os02g53420	10.2253391
LOC_Os01g74350	LOC_Os06g45710	8.87809973
LOC_Os01g74350	LOC_Os12g43630	11.7556536
LOC_Os02g01160	LOC_Os06g06090	8.93012748
LOC_Os02g01220	LOC_Os02g47800	9.04295203
LOC_Os02g01160	LOC_Os02g17780	9.36853754
LOC_Os01g74350	LOC_Os03g21260	9.42428038
LOC_Os02g01160	LOC_Os04g44950	11.2414509
LOC_Os02g01220	LOC_Os04g57590	9.13322541
LOC_Os01g74000	LOC_Os09g20090	9.04907272
LOC_Os02g01280	LOC_Os02g07160	9.58160932
LOC_Os02g01220	LOC_Os07g42924	8.98540975
LOC_Os02g01220	LOC_Os08g04560	8.91370555
LOC_Os02g01160	LOC_Os04g45290	9.89387844
LOC_Os02g01590	LOC_Os07g47290	8.9450435
LOC_Os02g01340	LOC_Os11g05570	9.91967771
LOC_Os02g01280	LOC_Os12g25710	9.76704874
LOC_Os02g01510	LOC_Os02g52710	10.0088273
LOC_Os02g01590	LOC_Os02g21460	9.49996601
LOC_Os02g01280	LOC_Os08g40930	9.35297946
LOC_Os02g01510	LOC_Os05g47640	8.90848276
LOC_Os02g01510	LOC_Os08g43190	9.37449659
LOC_Os02g01560	LOC_Os04g25990	12.497034
LOC_Os02g01510	LOC_Os04g48700	9.54429887
LOC_Os02g01510	LOC_Os11g08470	9.31864855
LOC_Os02g01590	LOC_Os03g55874	9.40143682
LOC_Os02g01590	LOC_Os03g01770	9.21801718
LOC_Os02g01340	LOC_Os09g07510	9.59450975
LOC_Os02g01340	LOC_Os03g42840	8.91758548
LOC_Os02g01560	LOC_Os03g53200	9.20218642
LOC_Os02g01510	LOC_Os02g46970	9.31654915
LOC_Os02g01760	LOC_Os02g56130	9.28534089
LOC_Os02g01510	LOC_Os02g02830	9.08546413
LOC_Os02g01560	LOC_Os08g37800	9.60057981
LOC_Os02g01560	LOC_Os07g22600	9.36502599

InteractorA	InteractorB	Z score
LOC_Os02g01560	LOC_Os07g34140	9.18770127
LOC_Os02g01340	LOC_Os05g46580	9.11208607
LOC_Os02g01560	LOC_Os12g12560	9.08434555
LOC_Os02g01510	LOC_Os05g26890	9.4743968
LOC_Os02g01510	LOC_Os02g52700	10.0088404
LOC_Os02g01590	LOC_Os08g06060	9.38114567
LOC_Os02g01560	LOC_Os07g44260	9.45893496
LOC_Os02g01280	LOC_Os03g12290	9.64769411
LOC_Os02g01590	LOC_Os04g39900	8.89087018
LOC_Os02g01280	LOC_Os05g23740	9.59884091
LOC_Os02g01340	LOC_Os03g12660	9.01229247
LOC_Os02g01510	LOC_Os04g47170	9.0847535
LOC_Os02g01760	LOC_Os05g22724	10.1117611
LOC_Os02g01510	LOC_Os08g35440	8.82092899
LOC_Os02g01590	LOC_Os10g40720	8.99092892
LOC_Os02g01510	LOC_Os02g41470	8.93064884
LOC_Os02g01280	LOC_Os06g10970	9.47867892
LOC_Os02g01280	LOC_Os11g02130	9.09006556
LOC_Os02g01340	LOC_Os07g42940	8.97233323
LOC_Os02g01340	LOC_Os10g35110	9.62402399
LOC_Os02g01280	LOC_Os08g02400	8.80090098
LOC_Os02g01510	LOC_Os07g23470	10.308856
LOC_Os02g01590	LOC_Os03g08530	10.0157804
LOC_Os02g01340	LOC_Os05g44050	8.87116001
LOC_Os02g01340	LOC_Os10g05069	8.9338075
LOC_Os02g01510	LOC_Os03g16920	9.27023715
LOC_Os02g01340	LOC_Os02g10320	9.10447462
LOC_Os02g01280	LOC_Os03g21950	9.3309912
LOC_Os02g01280	LOC_Os06g46340	9.10372198
LOC_Os02g01590	LOC_Os03g44484	8.87420559
LOC_Os02g01510	LOC_Os05g48980	9.14269009
LOC_Os02g01510	LOC_Os05g12180	9.2050562
LOC_Os02g01280	LOC_Os08g44210	9.29487455
LOC_Os02g01340	LOC_Os03g13070	9.98922468
LOC_Os02g01760	LOC_Os05g28940	9.07667859
LOC_Os02g01590	LOC_Os03g21260	9.51840871
LOC_Os02g01760	LOC_Os02g24634	9.53723766
LOC_Os02g01340	LOC_Os03g60740	8.98433412
LOC_Os02g01510	LOC_Os06g11260	8.90544022
LOC_Os02g01560	LOC_Os03g56840	9.21961012
LOC_Os02g01560	LOC_Os03g30470	9.74863029
LOC_Os02g01280	LOC_Os04g56230	8.85391252
LOC_Os02g01340	LOC_Os05g37700	9.30672015

InteractorA	InteractorB	Z score
LOC_Os02g01280	LOC_Os08g04560	9.0343425
LOC_Os02g01560	LOC_Os07g08660	8.88746741
LOC_Os02g01340	LOC_Os05g49890	9.07050825
LOC_Os02g01760	LOC_Os04g27980	9.46389806
LOC_Os02g01560	LOC_Os05g06480	9.30471045
LOC_Os02g01560	LOC_Os02g07060	9.09863537
LOC_Os02g01340	LOC_Os03g18130	10.0415494
LOC_Os02g01280	LOC_Os07g35940	10.5850697
LOC_Os02g01560	LOC_Os04g40290	8.96279225
LOC_Os02g01340	LOC_Os04g59450	9.47198887
LOC_Os02g01340	LOC_Os04g57390	9.9902866
LOC_Os02g01340	LOC_Os11g03290	10.5854123
LOC_Os02g01340	LOC_Os05g05680	9.55425835
LOC_Os02g01510	LOC_Os05g32140	8.83775311
LOC_Os02g01590	LOC_Os03g63090	10.3154472
LOC_Os02g01340	LOC_Os05g46860	9.36055371
LOC_Os02g01760	LOC_Os05g44140	9.79262969
LOC_Os02g01510	LOC_Os07g44740	9.04971494
LOC_Os02g01340	LOC_Os06g19960	9.42486676
LOC_Os02g01560	LOC_Os07g44460	9.66786312
LOC_Os02g01280	LOC_Os06g30970	9.89259943
LOC_Os02g01590	LOC_Os12g31370	9.85921471
LOC_Os02g01280	LOC_Os06g37610	8.94900568
LOC_Os02g01280	LOC_Os02g50240	8.98154307
LOC_Os02g01340	LOC_Os07g27790	8.84956271
LOC_Os02g01510	LOC_Os02g55140	10.041266
LOC_Os02g01590	LOC_Os09g23540	9.71792109
LOC_Os02g01280	LOC_Os04g25990	8.95258679
LOC_Os02g01560	LOC_Os04g40990	8.90005806
LOC_Os02g01340	LOC_Os03g17700	9.3789704
LOC_Os02g01340	LOC_Os03g13160	10.4503351
LOC_Os02g01340	LOC_Os10g11140	8.87928345
LOC_Os02g01760	LOC_Os02g45540	9.34591532
LOC_Os02g01280	LOC_Os04g43410	9.86080429
LOC_Os02g01340	LOC_Os04g22730	9.14259186
LOC_Os02g01880	LOC_Os04g17650	9.14806267
LOC_Os02g02210	LOC_Os07g42950	9.33921396
LOC_Os02g02210	LOC_Os06g51050	10.4286631
LOC_Os02g02560	LOC_Os10g21326	9.34767326
LOC_Os02g01760	LOC_Os08g38920	9.6389301
LOC_Os02g01920	LOC_Os03g16740	9.22290064
LOC_Os02g01920	LOC_Os07g26660	9.8958766
LOC_Os02g02410	LOC_Os09g12660	9.24837306

InteractorA	InteractorB	Z score
LOC_Os02g01880	LOC_Os06g35520	9.51969169
LOC_Os02g02560	LOC_Os04g32010	8.99781848
LOC_Os02g02410	LOC_Os04g16680	11.3024059
LOC_Os02g02410	LOC_Os04g46620	9.43188415
LOC_Os02g01920	LOC_Os10g21212	10.1740391
LOC_Os02g02410	LOC_Os03g55600	9.82152625
LOC_Os02g01880	LOC_Os04g41960	9.60478858
LOC_Os02g02560	LOC_Os07g09340	8.96485423
LOC_Os02g02210	LOC_Os08g40140	9.94849736
LOC_Os02g02410	LOC_Os09g23530	8.8082382
LOC_Os02g02560	LOC_Os04g39840	9.21435817
LOC_Os02g01880	LOC_Os05g41210	8.83766631
LOC_Os02g02560	LOC_Os07g40580	10.124165
LOC_Os02g01920	LOC_Os07g34190	8.98785584
LOC_Os02g02410	LOC_Os06g43640	9.561493
LOC_Os02g01880	LOC_Os08g43190	9.08353833
LOC_Os02g01920	LOC_Os09g23540	8.85880548
LOC_Os02g01880	LOC_Os07g46310	9.24123863
LOC_Os02g01920	LOC_Os10g27050	9.88897524
LOC_Os02g01880	LOC_Os06g46284	9.14038079
LOC_Os02g02560	LOC_Os05g48290	8.99124824
LOC_Os02g01920	LOC_Os03g51740	10.0824762
LOC_Os02g01880	LOC_Os12g40510	9.9955837
LOC_Os02g02560	LOC_Os10g39840	10.7613451
LOC_Os02g02210	LOC_Os04g52440	16.8451287
LOC_Os02g01880	LOC_Os03g32270	9.91032857
LOC_Os02g01880	LOC_Os06g45120	9.0469109
LOC_Os02g01760	LOC_Os12g40550	8.93625084
LOC_Os02g02410	LOC_Os10g07616	9.1962609
LOC_Os02g01760	LOC_Os08g28820	9.46796932
LOC_Os02g02560	LOC_Os09g24990	8.99296049
LOC_Os02g02560	LOC_Os07g02210	8.81039222
LOC_Os02g01920	LOC_Os03g50480	9.23419735
LOC_Os02g02560	LOC_Os11g08940	9.22669086
LOC_Os02g02560	LOC_Os03g20370	9.9322303
LOC_Os02g02830	LOC_Os02g08420	9.99717101
LOC_Os02g02410	LOC_Os03g15360	8.90184299
LOC_Os02g02560	LOC_Os07g26640	8.88537059
LOC_Os02g01920	LOC_Os05g49830	11.5891388
LOC_Os02g02210	LOC_Os02g57260	9.41733182
LOC_Os02g01920	LOC_Os10g02040	8.88062752
LOC_Os02g01920	LOC_Os03g10370	8.82049102
LOC_Os02g01880	LOC_Os07g05180	9.42152201

InteractorA	InteractorB	Z score
LOC_Os02g02210	LOC_Os07g04240	8.9011161
LOC_Os02g02410	LOC_Os04g40874	9.52959341
LOC_Os02g01920	LOC_Os12g12514	9.06234311
LOC_Os02g02560	LOC_Os04g56230	8.95011289
LOC_Os02g02560	LOC_Os11g03980	8.96045395
LOC_Os02g01920	LOC_Os04g39864	10.2644967
LOC_Os02g01880	LOC_Os11g37550	8.9565402
LOC_Os02g02560	LOC_Os04g16844	9.34767979
LOC_Os02g02210	LOC_Os02g49720	9.28981951
LOC_Os02g02560	LOC_Os09g12570	9.32304652
LOC_Os02g02410	LOC_Os06g39875	10.022663
LOC_Os02g02560	LOC_Os07g34190	9.42194214
LOC_Os02g01920	LOC_Os07g41750	10.4454701
LOC_Os02g01880	LOC_Os02g52630	9.13363079
LOC_Os02g02410	LOC_Os03g61740	9.60613584
LOC_Os02g02410	LOC_Os03g16880	9.03394602
LOC_Os02g01920	LOC_Os11g08330	9.44761082
LOC_Os02g02560	LOC_Os02g41670	9.11414758
LOC_Os02g01880	LOC_Os04g09604	9.45554604
LOC_Os02g02560	LOC_Os07g48780	9.9322303
LOC_Os02g02560	LOC_Os12g04980	9.08488012
LOC_Os02g02410	LOC_Os11g33240	11.3637803
LOC_Os02g02410	LOC_Os10g31950	11.3117507
LOC_Os02g02410	LOC_Os04g12900	11.4332943
LOC_Os02g02560	LOC_Os06g49970	9.8356793
LOC_Os02g02560	LOC_Os04g26910	9.13949622
LOC_Os02g02410	LOC_Os03g61360	8.81201779
LOC_Os02g02210	LOC_Os08g10510	12.4686168
LOC_Os02g01920	LOC_Os09g34250	11.2013409
LOC_Os02g02560	LOC_Os03g55874	9.0042644
LOC_Os02g01880	LOC_Os03g43890	8.92769168
LOC_Os02g01880	LOC_Os10g38160	9.75933339
LOC_Os02g02560	LOC_Os04g57380	9.26071642
LOC_Os02g02410	LOC_Os09g25320	9.65971833
LOC_Os02g02410	LOC_Os09g31410	10.1119308
LOC_Os02g01880	LOC_Os04g58710	9.41219724
LOC_Os02g01920	LOC_Os04g47360	9.20809518
LOC_Os02g01920	LOC_Os06g43640	8.95124067
LOC_Os02g01880	LOC_Os03g57120	10.7774283
LOC_Os02g01920	LOC_Os10g13800	10.2972015
LOC_Os02g02560	LOC_Os12g22650	8.95419719
LOC_Os02g01920	LOC_Os04g56210	9.26410399
LOC_Os02g01880	LOC_Os10g39840	11.3329481

InteractorA	InteractorB	Z score
LOC_Os02g01920	LOC_Os11g10480	9.09156317
LOC_Os02g02560	LOC_Os10g21248	9.30946297
LOC_Os02g02560	LOC_Os02g18550	10.4205295
LOC_Os02g02410	LOC_Os03g30870	9.77340521
LOC_Os02g01920	LOC_Os07g26690	8.94423315
LOC_Os02g02210	LOC_Os10g08620	9.10145883
LOC_Os02g01880	LOC_Os04g54330	9.22884873
LOC_Os02g01920	LOC_Os09g39380	9.79085048
LOC_Os02g02410	LOC_Os05g45810	9.05944917
LOC_Os02g02210	LOC_Os08g14770	11.0973022
LOC_Os02g02560	LOC_Os11g31620	10.7939045
LOC_Os02g02410	LOC_Os03g22060	9.18363199
LOC_Os02g01920	LOC_Os10g21310	8.84287446
LOC_Os02g02560	LOC_Os08g34290	9.17898313
LOC_Os02g01760	LOC_Os09g15420	8.84210276
LOC_Os02g02410	LOC_Os07g46830	9.07518462
LOC_Os02g01920	LOC_Os07g47990	9.12024948
LOC_Os02g02410	LOC_Os09g39810	9.01867639
LOC_Os02g02210	LOC_Os11g37550	9.90270962
LOC_Os02g02560	LOC_Os08g14760	8.86449258
LOC_Os02g01920	LOC_Os06g37500	9.69569119
LOC_Os02g02210	LOC_Os06g23870	8.8025105
LOC_Os02g02210	LOC_Os04g52450	12.8718581
LOC_Os02g01920	LOC_Os03g01530	9.36555897
LOC_Os02g02560	LOC_Os06g45710	9.24155176
LOC_Os02g01920	LOC_Os03g49600	9.33335362
LOC_Os02g02560	LOC_Os04g53290	9.06444025
LOC_Os02g02210	LOC_Os04g01740	9.01638405
LOC_Os02g02210	LOC_Os09g20820	10.6228561
LOC_Os02g02560	LOC_Os03g50440	8.91503656
LOC_Os02g02560	LOC_Os04g57410	10.3610589
LOC_Os02g02410	LOC_Os07g01760	8.80614259
LOC_Os02g01920	LOC_Os12g37360	8.85738748
LOC_Os02g01920	LOC_Os07g34580	9.70053287
LOC_Os02g02560	LOC_Os10g28200	9.06829505
LOC_Os02g02410	LOC_Os10g27050	9.10480596
LOC_Os02g01920	LOC_Os08g41340	8.82493736
LOC_Os02g01920	LOC_Os02g46130	10.5953373
LOC_Os02g01880	LOC_Os04g39900	9.14002153
LOC_Os02g02410	LOC_Os06g44080	9.65971833
LOC_Os02g02410	LOC_Os12g12560	9.55037066
LOC_Os02g01880	LOC_Os10g38740	9.51277826
LOC_Os02g01920	LOC_Os05g51480	9.2463937

InteractorA	InteractorB	Z score
LOC_Os02g01920	LOC_Os07g22930	9.13018945
LOC_Os02g01880	LOC_Os07g05400	10.0420787
LOC_Os02g02210	LOC_Os10g26600	8.93559396
LOC_Os02g01880	LOC_Os10g25950	8.80520909
LOC_Os02g01920	LOC_Os03g64050	8.87834824
LOC_Os02g01760	LOC_Os08g28800	9.7407851
LOC_Os02g01880	LOC_Os10g21248	8.90940508
LOC_Os02g01920	LOC_Os10g28120	10.247295
LOC_Os02g02960	LOC_Os03g02939	9.31140145
LOC_Os02g02890	LOC_Os12g10720	10.0192684
LOC_Os02g03610	LOC_Os06g06730	8.91879478
LOC_Os02g02890	LOC_Os08g09940	9.49957319
LOC_Os02g02840	LOC_Os03g57120	8.95687215
LOC_Os02g02830	LOC_Os10g38140	9.14475858
LOC_Os02g02960	LOC_Os03g55280	9.28008784
LOC_Os02g02840	LOC_Os04g48540	9.26487376
LOC_Os02g03870	LOC_Os03g44150	8.8205813
LOC_Os02g02960	LOC_Os06g24990	8.99063522
LOC_Os02g03610	LOC_Os05g12190	9.40640574
LOC_Os02g03610	LOC_Os06g41810	8.87429182
LOC_Os02g03610	LOC_Os05g48510	8.84382399
LOC_Os02g02960	LOC_Os04g12480	10.4236817
LOC_Os02g03610	LOC_Os03g42840	11.0217552
LOC_Os02g02830	LOC_Os05g38530	8.94466247
LOC_Os02g02830	LOC_Os09g23540	9.43096625
LOC_Os02g02890	LOC_Os05g28180	9.01141732
LOC_Os02g02960	LOC_Os05g47540	9.08922019
LOC_Os02g03610	LOC_Os10g25140	8.83750928
LOC_Os02g02960	LOC_Os05g50890	9.33198269
LOC_Os02g02840	LOC_Os10g09860	9.94690727
LOC_Os02g02960	LOC_Os11g08470	10.3966758
LOC_Os02g02960	LOC_Os03g46060	10.2393397
LOC_Os02g02960	LOC_Os09g07460	8.81278558
LOC_Os02g02960	LOC_Os09g24530	9.09849555
LOC_Os02g02960	LOC_Os04g50880	8.97643113
LOC_Os02g03610	LOC_Os06g05700	9.23065991
LOC_Os02g03610	LOC_Os02g33080	8.8481067
LOC_Os02g02890	LOC_Os09g26380	8.84420902
LOC_Os02g03610	LOC_Os03g55800	8.91330565
LOC_Os02g02960	LOC_Os06g35490	9.07653753
LOC_Os02g02960	LOC_Os07g48040	8.80984445
LOC_Os02g02830	LOC_Os03g61330	9.31191161
LOC_Os02g03610	LOC_Os08g33370	9.1256441

InteractorA	InteractorB	Z score
LOC_Os02g02840	LOC_Os05g45810	9.00833107
LOC_Os02g02890	LOC_Os11g03980	10.6213582
LOC_Os02g02840	LOC_Os10g38340	9.58001878
LOC_Os02g03610	LOC_Os03g63410	8.97881065
LOC_Os02g02890	LOC_Os06g01850	10.0014183
LOC_Os02g03610	LOC_Os05g45590	9.47214686
LOC_Os02g03610	LOC_Os08g04180	8.93555838
LOC_Os02g02960	LOC_Os03g61740	9.78709416
LOC_Os02g03870	LOC_Os04g46620	10.0189898
LOC_Os02g02840	LOC_Os07g30970	9.57164674
LOC_Os02g02960	LOC_Os03g16050	8.80966704
LOC_Os02g02960	LOC_Os10g02480	8.92789838
LOC_Os02g02830	LOC_Os11g29400	9.22866979
LOC_Os02g03610	LOC_Os03g49350	10.781522
LOC_Os02g02830	LOC_Os07g35940	9.34073736
LOC_Os02g02840	LOC_Os03g49260	9.38375987
LOC_Os02g03870	LOC_Os03g21950	9.18144798
LOC_Os02g02960	LOC_Os02g08130	9.17278156
LOC_Os02g02960	LOC_Os03g01900	10.2723578
LOC_Os02g02890	LOC_Os04g39030	9.63488211
LOC_Os02g03870	LOC_Os06g27770	10.7504622
LOC_Os02g02840	LOC_Os03g18810	9.24375167
LOC_Os02g02830	LOC_Os07g12730	9.82544699
LOC_Os02g03870	LOC_Os07g29750	8.82533552
LOC_Os02g02840	LOC_Os04g33190	9.01147027
LOC_Os02g02830	LOC_Os07g07550	9.67261269
LOC_Os02g03610	LOC_Os03g53200	9.07824275
LOC_Os02g02890	LOC_Os06g36820	9.31600941
LOC_Os02g02840	LOC_Os05g34170	10.1122228
LOC_Os02g02840	LOC_Os03g07300	9.71254118
LOC_Os02g02830	LOC_Os12g06620	10.2142013
LOC_Os02g03610	LOC_Os07g38540	8.89107447
LOC_Os02g02960	LOC_Os04g40950	9.29587595
LOC_Os02g03870	LOC_Os02g52420	9.54955736
LOC_Os02g03610	LOC_Os08g37800	9.49302055
LOC_Os02g02830	LOC_Os08g15292	10.1159632
LOC_Os02g03870	LOC_Os04g44920	8.97522123
LOC_Os02g02840	LOC_Os08g39140	9.63420649
LOC_Os02g02830	LOC_Os04g53810	9.51280803
LOC_Os02g02840	LOC_Os06g35630	9.76998407
LOC_Os02g02890	LOC_Os06g23440	9.56519845
LOC_Os02g03610	LOC_Os09g36900	9.25184424
LOC_Os02g02960	LOC_Os04g40310	12.4669078

InteractorA	InteractorB	Z score
LOC_Os02g02960	LOC_Os11g08460	9.32314094
LOC_Os02g02840	LOC_Os04g37490	9.79414731
LOC_Os02g02890	LOC_Os04g33470	9.57145588
LOC_Os02g02890	LOC_Os10g32550	9.62654651
LOC_Os02g02960	LOC_Os09g32810	8.82501289
LOC_Os02g02830	LOC_Os09g24990	10.0731013
LOC_Os02g02840	LOC_Os04g56400	9.49502005
LOC_Os02g02960	LOC_Os06g10970	9.60284219
LOC_Os02g02840	LOC_Os07g27790	9.18442191
LOC_Os02g04950	LOC_Os04g49420	8.80847463
LOC_Os02g04100	LOC_Os07g03368	8.80422134
LOC_Os02g04320	LOC_Os02g49720	9.12825185
LOC_Os02g04950	LOC_Os03g19930	9.00382189
LOC_Os02g04950	LOC_Os04g56230	8.89330281
LOC_Os02g04950	LOC_Os02g07720	8.97482477
LOC_Os02g04480	LOC_Os04g10400	9.65806346
LOC_Os02g04950	LOC_Os09g26340	8.80845212
LOC_Os02g05410	LOC_Os05g34170	9.33504372
LOC_Os02g04480	LOC_Os12g02370	8.85256016
LOC_Os02g04100	LOC_Os03g56280	9.03173994
LOC_Os02g05410	LOC_Os04g40990	9.05570372
LOC_Os02g04950	LOC_Os07g36500	8.80847463
LOC_Os02g06300	LOC_Os03g61740	8.80735943
LOC_Os02g03870	LOC_Os11g08330	10.7649492
LOC_Os02g04950	LOC_Os07g41050	8.97669995
LOC_Os02g04950	LOC_Os02g45940	8.80847463
LOC_Os02g04320	LOC_Os07g48030	9.82685979
LOC_Os02g04100	LOC_Os02g13140	9.31362976
LOC_Os02g04320	LOC_Os06g46000	9.34598833
LOC_Os02g06300	LOC_Os03g16230	9.92699369
LOC_Os02g05410	LOC_Os06g19960	9.97544608
LOC_Os02g04950	LOC_Os03g02780	8.80847463
LOC_Os02g04100	LOC_Os03g51550	9.3746329
LOC_Os02g04100	LOC_Os09g39570	9.78884624
LOC_Os02g05410	LOC_Os04g48850	8.9204251
LOC_Os02g04320	LOC_Os06g10930	9.02350033
LOC_Os02g04480	LOC_Os08g28820	9.009969
LOC_Os02g04100	LOC_Os11g33240	11.3414236
LOC_Os02g03870	LOC_Os09g26880	11.2997006
LOC_Os02g04320	LOC_Os10g21326	8.8334079
LOC_Os02g04320	LOC_Os04g58680	8.90769173
LOC_Os02g04480	LOC_Os03g16740	9.56919229
LOC_Os02g04480	LOC_Os04g43750	9.16465378

InteractorA	InteractorB	Z score
LOC_Os02g05410	LOC_Os05g04690	9.6140973
LOC_Os02g04950	LOC_Os10g08670	8.92326544
LOC_Os02g04950	LOC_Os10g28120	8.80034948
LOC_Os02g04480	LOC_Os03g58630	9.25667829
LOC_Os02g04480	LOC_Os05g25490	10.9530442
LOC_Os02g04950	LOC_Os05g09500	9.7853693
LOC_Os02g04950	LOC_Os09g08880	8.91448108
LOC_Os02g04950	LOC_Os06g36700	12.6762834
LOC_Os02g05410	LOC_Os03g01800	9.3573543
LOC_Os02g05410	LOC_Os07g38540	9.04754615
LOC_Os02g04320	LOC_Os04g57590	9.64615595
LOC_Os02g04950	LOC_Os04g59450	9.27861346
LOC_Os02g04950	LOC_Os08g09370	8.86247875
LOC_Os02g04320	LOC_Os09g34214	9.487816
LOC_Os02g04480	LOC_Os04g53800	9.02863078
LOC_Os02g04950	LOC_Os03g50885	9.11464999
LOC_Os02g04100	LOC_Os04g28180	9.40851432
LOC_Os02g04100	LOC_Os07g49220	10.0951907
LOC_Os02g05410	LOC_Os11g48110	8.92310396
LOC_Os02g05410	LOC_Os05g51480	8.85983943
LOC_Os02g04950	LOC_Os03g02920	9.53434546
LOC_Os02g04320	LOC_Os07g42600	9.8870884
LOC_Os02g04480	LOC_Os07g34520	9.01474858
LOC_Os02g04950	LOC_Os05g38740	8.80847634
LOC_Os02g04480	LOC_Os06g45710	9.62508515
LOC_Os02g04100	LOC_Os02g52640	10.9378496
LOC_Os02g06300	LOC_Os03g13200	9.55057933
LOC_Os02g04100	LOC_Os03g04410	9.60958636
LOC_Os02g04950	LOC_Os06g04510	8.97477152
LOC_Os02g04950	LOC_Os04g45290	9.22109565
LOC_Os02g04480	LOC_Os11g03290	9.04469466
LOC_Os02g04320	LOC_Os07g30640	8.95603873
LOC_Os02g04950	LOC_Os04g54330	8.93949394
LOC_Os02g04950	LOC_Os05g39050	8.80847463
LOC_Os02g04950	LOC_Os05g49760	8.85218851
LOC_Os02g04320	LOC_Os07g05160	10.0772823
LOC_Os02g04320	LOC_Os04g39900	9.71292645
LOC_Os02g04950	LOC_Os10g39410	8.80847463
LOC_Os02g04480	LOC_Os03g01900	8.95027668
LOC_Os02g05410	LOC_Os09g24990	9.04113438
LOC_Os02g04950	LOC_Os06g43640	10.2393989
LOC_Os02g04950	LOC_Os06g10910	9.95451065
LOC_Os02g05410	LOC_Os04g55850	9.4458024

InteractorA	InteractorB	Z score
LOC_Os02g04950	LOC_Os09g38020	8.80847463
LOC_Os02g04100	LOC_Os03g16900	8.94124942
LOC_Os02g04100	LOC_Os08g32620	9.83164938
LOC_Os02g04320	LOC_Os05g12190	8.83989444
LOC_Os02g04480	LOC_Os04g40290	9.3993296
LOC_Os02g04950	LOC_Os02g51830	9.36539899
LOC_Os02g04320	LOC_Os03g21900	8.96878246
LOC_Os02g04320	LOC_Os05g11550	10.5691066
LOC_Os02g04320	LOC_Os06g45070	8.93558738
LOC_Os02g04950	LOC_Os11g26850	8.83579578
LOC_Os02g04950	LOC_Os03g16880	11.6924519
LOC_Os02g04950	LOC_Os07g39290	12.0492286
LOC_Os02g04950	LOC_Os09g14670	9.34909521
LOC_Os02g04320	LOC_Os07g47990	9.08391015
LOC_Os02g04320	LOC_Os10g25130	8.96319902
LOC_Os02g04100	LOC_Os09g10270	9.53894997
LOC_Os02g04320	LOC_Os04g16844	8.83340435
LOC_Os02g03870	LOC_Os11g32650	9.13951677
LOC_Os02g04950	LOC_Os05g41210	10.0969057
LOC_Os02g06300	LOC_Os03g15960	9.20452962
LOC_Os02g04320	LOC_Os06g06100	8.82164788
LOC_Os02g04100	LOC_Os03g17980	9.10138014
LOC_Os02g05410	LOC_Os05g01810	10.5077202
LOC_Os02g06300	LOC_Os06g04270	10.9076735
LOC_Os02g06300	LOC_Os12g08280	8.99035878
LOC_Os02g06700	LOC_Os09g08120	9.50882124
LOC_Os02g07060	LOC_Os03g12270	9.81221165
LOC_Os02g06540	LOC_Os04g48850	10.3462097
LOC_Os02g07230	LOC_Os05g30480	10.0852862
LOC_Os02g07230	LOC_Os06g06980	8.94586979
LOC_Os02g06300	LOC_Os06g35560	11.3631309
LOC_Os02g06700	LOC_Os11g25700	10.2772748
LOC_Os02g06300	LOC_Os07g46830	9.34280676
LOC_Os02g06300	LOC_Os06g49470	9.20578369
LOC_Os02g06300	LOC_Os04g56320	9.21268005
LOC_Os02g07160	LOC_Os06g44620	9.0109967
LOC_Os02g07060	LOC_Os09g04050	9.59566816
LOC_Os02g06700	LOC_Os02g16550	9.00453824
LOC_Os02g07230	LOC_Os02g55300	9.09744863
LOC_Os02g07060	LOC_Os07g08880	9.95199382
LOC_Os02g06300	LOC_Os09g10270	9.17719218
LOC_Os02g07060	LOC_Os09g10260	8.88369085
LOC_Os02g06700	LOC_Os10g07229	9.47328117

InteractorA	InteractorB	Z score
LOC_Os02g07230	LOC_Os09g31486	9.94104768
LOC_Os02g06300	LOC_Os06g15420	9.20037295
LOC_Os02g07230	LOC_Os05g37390	9.28832204
LOC_Os02g07160	LOC_Os02g52610	9.20874321
LOC_Os02g06700	LOC_Os10g28350	10.0443368
LOC_Os02g06700	LOC_Os02g46970	8.87589115
LOC_Os02g06300	LOC_Os07g48430	9.51420487
LOC_Os02g07060	LOC_Os11g32610	8.82677907
LOC_Os02g07160	LOC_Os10g32870	9.80510445
LOC_Os02g06700	LOC_Os03g49260	9.08528743
LOC_Os02g07060	LOC_Os06g34690	9.92776818
LOC_Os02g07160	LOC_Os05g38560	10.3971256
LOC_Os02g07230	LOC_Os05g20050	9.80725246
LOC_Os02g06700	LOC_Os05g05800	9.26296067
LOC_Os02g07160	LOC_Os04g44950	8.96369538
LOC_Os02g06300	LOC_Os10g38640	9.09627677
LOC_Os02g06700	LOC_Os04g48540	9.1102282
LOC_Os02g06700	LOC_Os10g31950	9.31204465
LOC_Os02g07060	LOC_Os07g02350	9.0230866
LOC_Os02g07060	LOC_Os06g07080	9.069707
LOC_Os02g06700	LOC_Os12g05590	10.4504937
LOC_Os02g07230	LOC_Os04g42920	8.82513019
LOC_Os02g06700	LOC_Os03g15960	8.84206754
LOC_Os02g06540	LOC_Os05g11730	8.84664595
LOC_Os02g06700	LOC_Os09g36830	8.91851288
LOC_Os02g06700	LOC_Os10g02070	10.2916252
LOC_Os02g07060	LOC_Os07g07240	8.87295109
LOC_Os02g06300	LOC_Os05g04470	9.02198731
LOC_Os02g06700	LOC_Os04g33570	12.0956286
LOC_Os02g06540	LOC_Os06g30970	9.03398758
LOC_Os02g06700	LOC_Os09g36710	9.11694144
LOC_Os02g06540	LOC_Os04g42250	8.89855055
LOC_Os02g06300	LOC_Os10g17680	9.65686377
LOC_Os02g07160	LOC_Os12g16240	9.84775129
LOC_Os02g06700	LOC_Os11g47760	9.39093931
LOC_Os02g06700	LOC_Os03g59020	9.40857483
LOC_Os02g07160	LOC_Os09g04050	9.07662732
LOC_Os02g06300	LOC_Os06g35650	8.80799684
LOC_Os02g06300	LOC_Os10g17660	9.65687369
LOC_Os02g06700	LOC_Os03g27370	10.2156256
LOC_Os02g07160	LOC_Os07g05800	9.08918321
LOC_Os02g06700	LOC_Os05g19150	9.36386671
LOC_Os02g06700	LOC_Os12g07820	9.0000603

InteractorA	InteractorB	Z score
LOC_Os02g06300	LOC_Os06g07960	9.32059326
LOC_Os02g06300	LOC_Os04g45290	10.0381274
LOC_Os02g07060	LOC_Os09g39380	9.12121725
LOC_Os02g06300	LOC_Os11g25100	9.16127932
LOC_Os02g06540	LOC_Os03g27310	10.385487
LOC_Os02g06700	LOC_Os08g28680	10.0766198
LOC_Os02g07060	LOC_Os04g33570	9.14185485
LOC_Os02g07230	LOC_Os06g01850	8.9290558
LOC_Os02g07160	LOC_Os05g41640	10.0167018
LOC_Os02g06700	LOC_Os11g41130	9.28345556
LOC_Os02g07060	LOC_Os03g47000	8.85483784
LOC_Os02g06700	LOC_Os04g02050	10.0375526
LOC_Os02g07230	LOC_Os09g26380	10.784686
LOC_Os02g06700	LOC_Os04g33190	9.05305233
LOC_Os02g07160	LOC_Os03g56460	9.03552531
LOC_Os02g07160	LOC_Os04g01600	9.15318623
LOC_Os02g06700	LOC_Os02g14440	9.43736349
LOC_Os02g06700	LOC_Os04g39900	10.4700672
LOC_Os02g07160	LOC_Os11g03230	9.24593423
LOC_Os02g07060	LOC_Os07g47990	8.80285359
LOC_Os02g06300	LOC_Os05g28180	9.03490279
LOC_Os02g07160	LOC_Os07g40580	9.0513486
LOC_Os02g06540	LOC_Os02g27769	9.6098408
LOC_Os02g06300	LOC_Os11g03230	8.85490975
LOC_Os02g07230	LOC_Os06g48180	10.3135904
LOC_Os02g06540	LOC_Os06g04030	10.385487
LOC_Os02g06300	LOC_Os04g47170	8.99174845
LOC_Os02g07160	LOC_Os03g50885	9.34523195
LOC_Os02g07160	LOC_Os06g37150	10.2198953
LOC_Os02g07160	LOC_Os03g49380	8.85604932
LOC_Os02g06540	LOC_Os12g04924	8.91504357
LOC_Os02g06300	LOC_Os12g18900	11.6480707
LOC_Os02g07230	LOC_Os05g37330	8.90638788
LOC_Os02g07230	LOC_Os03g12270	10.2064235
LOC_Os02g07060	LOC_Os02g12730	8.98596677
LOC_Os02g07060	LOC_Os08g32620	8.80887131
LOC_Os02g07060	LOC_Os07g22930	10.4582632
LOC_Os02g06300	LOC_Os10g08670	8.9478353
LOC_Os01g08350	LOC_Os11g40150	9.17664886
LOC_Os01g09260	LOC_Os01g62020	9.05445075
LOC_Os01g08020	LOC_Os02g38840	9.17565383
LOC_Os01g09260	LOC_Os11g03230	8.99907692
LOC_Os01g08350	LOC_Os08g38920	9.32876056

InteractorA	InteractorB	Z score
LOC_Os01g08020	LOC_Os12g41110	10.2166402
LOC_Os01g09320	LOC_Os01g64660	9.98685162
LOC_Os01g08020	LOC_Os04g09604	10.4518517
LOC_Os01g08350	LOC_Os05g46860	10.2634692
LOC_Os01g08350	LOC_Os04g14680	9.86452738
LOC_Os01g09320	LOC_Os03g20700	9.74719183
LOC_Os01g09320	LOC_Os02g01160	9.25906156
LOC_Os01g09260	LOC_Os09g12590	9.74938955
LOC_Os01g08020	LOC_Os08g17784	9.01996399
LOC_Os01g09320	LOC_Os02g51930	9.27284586
LOC_Os01g09260	LOC_Os01g40400	8.82106661
LOC_Os01g08020	LOC_Os11g03290	8.87886043
LOC_Os01g08200	LOC_Os03g57040	9.32470058
LOC_Os01g09260	LOC_Os09g39500	9.18625042
LOC_Os01g08350	LOC_Os12g10730	10.6070781
LOC_Os01g08350	LOC_Os07g34190	8.92082501
LOC_Os01g08350	LOC_Os05g04470	9.49237565
LOC_Os01g08020	LOC_Os02g18880	9.07871294
LOC_Os01g08200	LOC_Os06g01610	9.35217612
LOC_Os01g09260	LOC_Os02g08420	8.91654149
LOC_Os01g09260	LOC_Os10g38340	8.91611612
LOC_Os01g09260	LOC_Os03g13170	9.18625559
LOC_Os01g08200	LOC_Os08g32620	9.94226711
LOC_Os01g08020	LOC_Os10g28320	9.20225734
LOC_Os01g09320	LOC_Os02g19770	8.92365397
LOC_Os01g08200	LOC_Os01g64970	8.93051067
LOC_Os01g09260	LOC_Os07g38890	9.67510404
LOC_Os01g08350	LOC_Os04g10010	8.92531154
LOC_Os01g08350	LOC_Os05g28180	12.7267187
LOC_Os01g08350	LOC_Os04g56070	9.16530479
LOC_Os01g09260	LOC_Os02g35310	9.28097147
LOC_Os01g09320	LOC_Os03g10370	8.9655204
LOC_Os01g08200	LOC_Os12g43450	9.34684325
LOC_Os01g08020	LOC_Os09g34960	9.01008057
LOC_Os01g09260	LOC_Os03g49600	9.1226702
LOC_Os01g08350	LOC_Os01g18120	9.24456025
LOC_Os01g08350	LOC_Os03g63090	9.63587226
LOC_Os01g08200	LOC_Os07g25150	9.26192431
LOC_Os01g08200	LOC_Os10g35070	9.4581472
LOC_Os01g08350	LOC_Os03g09250	9.59133821
LOC_Os01g08350	LOC_Os04g36800	9.91229147
LOC_Os01g08200	LOC_Os09g04050	9.17632609
LOC_Os01g08020	LOC_Os05g27950	9.06766537

InteractorA	InteractorB	Z score
LOC_Os01g08350	LOC_Os03g61600	10.2953015
LOC_Os01g08200	LOC_Os01g55950	9.70239735
LOC_Os01g08200	LOC_Os02g52560	9.36568688
LOC_Os01g08200	LOC_Os06g09450	9.33802565
LOC_Os01g08350	LOC_Os06g04030	8.92035498
LOC_Os01g08350	LOC_Os01g41630	10.3027839
LOC_Os01g08020	LOC_Os07g06410	9.46118214
LOC_Os01g09260	LOC_Os11g16590	9.64823895
LOC_Os01g08020	LOC_Os03g05730	8.87379377
LOC_Os01g09320	LOC_Os01g22352	9.25141022
LOC_Os01g08200	LOC_Os06g06560	9.84444161
LOC_Os01g08350	LOC_Os02g18880	8.96593476
LOC_Os01g07960	LOC_Os06g29844	8.85733291
LOC_Os01g08200	LOC_Os09g10230	9.23126098
LOC_Os01g08200	LOC_Os10g39840	9.67318889
LOC_Os01g08200	LOC_Os07g41050	9.69224492
LOC_Os01g08350	LOC_Os07g43510	9.76454825
LOC_Os01g08350	LOC_Os09g08120	9.65847053
LOC_Os01g08200	LOC_Os06g03770	9.40539542
LOC_Os01g08350	LOC_Os01g21970	9.1417746
LOC_Os01g08350	LOC_Os02g07870	9.43640189
LOC_Os01g08020	LOC_Os01g10820	8.86289032
LOC_Os01g08200	LOC_Os05g09490	9.91827052
LOC_Os01g08350	LOC_Os11g32610	9.6927112
LOC_Os01g08350	LOC_Os03g27310	8.92034503
LOC_Os01g08350	LOC_Os07g02340	9.10151981
LOC_Os01g08350	LOC_Os11g14220	10.3613534
LOC_Os01g09320	LOC_Os01g57964	9.9131642
LOC_Os01g08200	LOC_Os10g22070	8.98448422
LOC_Os01g08020	LOC_Os12g43370	8.80221073
LOC_Os01g08350	LOC_Os01g16890	10.6539509
LOC_Os01g08020	LOC_Os09g36450	9.36964756
LOC_Os01g08020	LOC_Os06g37080	9.04010594
LOC_Os01g08350	LOC_Os12g42876	9.72683615
LOC_Os01g09320	LOC_Os01g56880	13.5519832
LOC_Os01g08350	LOC_Os03g59020	10.3053492
LOC_Os01g09260	LOC_Os07g05400	9.82638277
LOC_Os01g08020	LOC_Os07g48880	8.87861007
LOC_Os01g08350	LOC_Os10g38780	9.89239091
LOC_Os01g09260	LOC_Os08g34170	10.2873553
LOC_Os01g08020	LOC_Os02g01220	9.23057785
LOC_Os01g08350	LOC_Os09g31490	9.05502954
LOC_Os01g08200	LOC_Os06g11260	10.8850736

InteractorA	InteractorB	Z score
LOC_Os01g08350	LOC_Os09g39440	9.43501921
LOC_Os01g08200	LOC_Os07g20544	10.138188
LOC_Os01g07960	LOC_Os10g38690	8.88140198
LOC_Os01g08200	LOC_Os07g43670	8.99160865
LOC_Os01g08200	LOC_Os06g43640	8.98124239
LOC_Os01g08020	LOC_Os01g44260	8.83033296
LOC_Os01g08350	LOC_Os08g41340	9.24741206
LOC_Os01g09320	LOC_Os01g59790	9.13098597
LOC_Os01g09320	LOC_Os01g53280	8.86095906
LOC_Os01g08350	LOC_Os01g37960	8.95648074
LOC_Os01g09260	LOC_Os10g30200	9.18467013
LOC_Os01g09320	LOC_Os01g44130	9.22217078
LOC_Os01g09260	LOC_Os03g58530	9.04378733
LOC_Os01g08350	LOC_Os08g37790	9.50713626
LOC_Os01g09260	LOC_Os02g52800	9.15876026
LOC_Os01g09320	LOC_Os01g52500	13.4955898
LOC_Os01g08020	LOC_Os07g06970	8.92202196
LOC_Os01g08020	LOC_Os01g21970	9.53665911
LOC_Os02g07490	LOC_Os03g13300	9.0505772
LOC_Os02g07260	LOC_Os07g46830	10.474498
LOC_Os02g07410	LOC_Os10g31950	8.97654495
LOC_Os02g07260	LOC_Os04g22660	9.39244639
LOC_Os02g07720	LOC_Os06g12990	9.24264433
LOC_Os02g07760	LOC_Os06g12790	9.36703234
LOC_Os02g07230	LOC_Os12g34874	9.99197018
LOC_Os02g07260	LOC_Os11g02440	8.87301211
LOC_Os02g07870	LOC_Os04g55850	11.7156861
LOC_Os02g07870	LOC_Os03g49350	8.93221
LOC_Os02g07760	LOC_Os08g02400	9.06879044
LOC_Os02g07410	LOC_Os02g57450	8.88973687
LOC_Os02g07260	LOC_Os11g37550	9.0793104
LOC_Os02g07720	LOC_Os02g34860	8.82158203
LOC_Os02g07490	LOC_Os06g45590	12.0641382
LOC_Os02g07870	LOC_Os03g17980	9.71557909
LOC_Os02g07720	LOC_Os09g28460	9.48148663
LOC_Os02g07720	LOC_Os11g47600	9.14541547
LOC_Os02g07490	LOC_Os02g49720	9.03593185
LOC_Os02g07760	LOC_Os03g16210	9.24758546
LOC_Os02g07870	LOC_Os02g15550	9.05499707
LOC_Os02g07870	LOC_Os03g48390	9.73279635
LOC_Os02g07260	LOC_Os12g16410	9.09830117
LOC_Os02g07760	LOC_Os10g38540	9.31276702
LOC_Os02g07760	LOC_Os05g27940	9.2465587

InteractorA	InteractorB	Z score
LOC_Os02g07720	LOC_Os02g56700	8.88835926
LOC_Os02g07260	LOC_Os06g50300	9.26866308
LOC_Os02g07720	LOC_Os04g43800	9.10705593
LOC_Os02g07760	LOC_Os02g57630	9.96281896
LOC_Os02g07260	LOC_Os03g22120	10.8401739
LOC_Os02g07760	LOC_Os03g06940	9.14831386
LOC_Os02g07490	LOC_Os07g05400	9.060874
LOC_Os02g07490	LOC_Os02g42810	9.03006141
LOC_Os02g07870	LOC_Os05g45420	9.32237927
LOC_Os02g07870	LOC_Os02g41470	9.03974927
LOC_Os02g07760	LOC_Os02g41670	9.52835363
LOC_Os02g07410	LOC_Os06g36770	9.81081823
LOC_Os02g07870	LOC_Os02g40000	8.9401981
LOC_Os02g07870	LOC_Os03g08010	10.7774728
LOC_Os02g07490	LOC_Os03g03720	9.91498035
LOC_Os02g07760	LOC_Os10g25950	9.61682735
LOC_Os02g07410	LOC_Os04g25400	8.93470438
LOC_Os02g07260	LOC_Os03g57200	9.03491228
LOC_Os02g07870	LOC_Os02g52590	10.3176932
LOC_Os02g07720	LOC_Os03g22530	8.86114977
LOC_Os02g07760	LOC_Os11g02130	9.20949804
LOC_Os02g07760	LOC_Os02g38200	10.4644669
LOC_Os02g07760	LOC_Os07g42600	9.51051118
LOC_Os02g07720	LOC_Os03g10340	10.2365676
LOC_Os02g07260	LOC_Os07g44590	9.44158814
LOC_Os02g07870	LOC_Os02g36974	9.06545543
LOC_Os02g07260	LOC_Os08g36910	9.8574777
LOC_Os02g07720	LOC_Os09g37100	8.89592517
LOC_Os02g07720	LOC_Os06g09450	9.98972257
LOC_Os02g07870	LOC_Os04g39864	9.52816554
LOC_Os02g07870	LOC_Os02g52420	9.05277315
LOC_Os02g07760	LOC_Os04g56730	8.8506904
LOC_Os02g07490	LOC_Os06g34690	10.094542
LOC_Os02g07760	LOC_Os03g50290	9.16722327
LOC_Os02g07720	LOC_Os04g47220	9.48933416
LOC_Os02g07720	LOC_Os06g21570	8.9447119
LOC_Os02g07870	LOC_Os03g02920	9.57625649
LOC_Os02g07260	LOC_Os04g43410	9.01123811
LOC_Os02g07720	LOC_Os07g30640	9.19336072
LOC_Os02g07260	LOC_Os10g41490	9.82215185
LOC_Os02g07490	LOC_Os04g45290	9.14189585
LOC_Os02g07490	LOC_Os04g52100	9.74579823
LOC_Os02g07260	LOC_Os09g39440	8.94129066

InteractorA	InteractorB	Z score
LOC_Os02g07260	LOC_Os04g59600	8.87234317
LOC_Os02g07490	LOC_Os03g51080	9.96642812
LOC_Os02g07260	LOC_Os09g32830	8.99035637
LOC_Os02g07260	LOC_Os02g07870	8.874294
LOC_Os02g07870	LOC_Os02g47610	10.4873789
LOC_Os02g07260	LOC_Os02g08130	10.0312857
LOC_Os02g07760	LOC_Os06g33210	9.61395417
LOC_Os02g07410	LOC_Os02g47790	9.01028896
LOC_Os02g07760	LOC_Os03g18570	9.55969906
LOC_Os02g07720	LOC_Os04g40874	9.38262908
LOC_Os02g07260	LOC_Os03g04250	12.2332122
LOC_Os02g07870	LOC_Os03g44150	9.68442857
LOC_Os02g07760	LOC_Os10g25130	10.3774282
LOC_Os02g07720	LOC_Os10g25130	9.30798912
LOC_Os02g07870	LOC_Os03g08050	10.7775579
LOC_Os02g07260	LOC_Os06g36880	9.10623981
LOC_Os02g07260	LOC_Os08g34170	9.18416629
LOC_Os02g07760	LOC_Os05g35320	9.27972689
LOC_Os02g07760	LOC_Os07g42940	8.85732719
LOC_Os02g07490	LOC_Os05g47980	8.91686717
LOC_Os02g07490	LOC_Os12g07720	10.1645521
LOC_Os02g07760	LOC_Os06g15420	8.94419953
LOC_Os02g07490	LOC_Os06g23760	8.89734684
LOC_Os02g07490	LOC_Os03g52860	10.0078748
LOC_Os02g07720	LOC_Os04g33740	9.13782678
LOC_Os02g07490	LOC_Os03g45960	9.41204157
LOC_Os02g07870	LOC_Os03g53860	9.60469704
LOC_Os02g07260	LOC_Os06g27770	11.0830098
LOC_Os02g07410	LOC_Os07g42924	9.54872004
LOC_Os02g07260	LOC_Os10g03540	9.03586824
LOC_Os02g07260	LOC_Os04g55290	10.0845577
LOC_Os02g07760	LOC_Os03g07840	9.76522388
LOC_Os02g07870	LOC_Os03g08020	10.777571
LOC_Os02g07870	LOC_Os06g37660	9.76370145
LOC_Os02g07870	LOC_Os03g62060	8.82746212
LOC_Os02g07260	LOC_Os07g38430	9.27330644
LOC_Os02g07490	LOC_Os04g46560	8.88812921
LOC_Os02g07760	LOC_Os06g35590	9.29092536
LOC_Os02g07760	LOC_Os05g01970	9.24328605
LOC_Os02g07260	LOC_Os03g46060	9.66227452
LOC_Os02g07760	LOC_Os03g02920	8.82240798
LOC_Os02g08100	LOC_Os10g40710	9.35401006
LOC_Os02g07870	LOC_Os07g29750	9.36105986

InteractorA	InteractorB	Z score
LOC_Os02g08130	LOC_Os06g07580	9.17565891
LOC_Os02g08490	LOC_Os04g53810	9.17040315
LOC_Os02g08130	LOC_Os02g56100	8.86205668
LOC_Os02g08420	LOC_Os08g44350	8.93124257
LOC_Os02g08420	LOC_Os04g32010	9.36317018
LOC_Os02g08490	LOC_Os11g31530	8.82353499
LOC_Os02g08410	LOC_Os03g49600	8.82644546
LOC_Os02g08130	LOC_Os06g23440	9.78014078
LOC_Os02g08420	LOC_Os12g16250	10.3916051
LOC_Os02g08100	LOC_Os09g20090	9.37810657
LOC_Os02g08100	LOC_Os12g17540	9.24589073
LOC_Os02g08410	LOC_Os03g13160	9.29359802
LOC_Os02g08410	LOC_Os09g24990	10.5911386
LOC_Os02g08490	LOC_Os10g08620	9.30767964
LOC_Os02g08490	LOC_Os10g08670	10.58263
LOC_Os02g08490	LOC_Os09g36800	8.95736405
LOC_Os02g08130	LOC_Os09g08720	9.22172411
LOC_Os02g08100	LOC_Os05g49880	9.64900115
LOC_Os02g08130	LOC_Os02g32030	9.2648051
LOC_Os02g08130	LOC_Os04g53214	10.0908043
LOC_Os02g08100	LOC_Os03g53230	9.42709688
LOC_Os02g08490	LOC_Os04g56320	9.02021976
LOC_Os02g08130	LOC_Os03g04410	9.40463865
LOC_Os02g08100	LOC_Os07g04240	9.23826739
LOC_Os02g08100	LOC_Os08g41990	10.0611463
LOC_Os02g08100	LOC_Os03g58260	10.0029414
LOC_Os02g08100	LOC_Os10g01080	8.92061417
LOC_Os02g08490	LOC_Os03g58530	9.63132411
LOC_Os02g08410	LOC_Os06g45670	9.4922754
LOC_Os02g08520	LOC_Os03g47000	8.89186552
LOC_Os02g08100	LOC_Os10g38610	9.01704218
LOC_Os02g07870	LOC_Os11g47580	9.51370904
LOC_Os02g07870	LOC_Os10g28320	8.83412471
LOC_Os02g08490	LOC_Os10g29620	9.05471507
LOC_Os02g08100	LOC_Os08g41830	9.47403477
LOC_Os02g08420	LOC_Os03g56410	10.043446
LOC_Os02g08130	LOC_Os04g57590	10.375773
LOC_Os02g08490	LOC_Os03g53650	9.20527821
LOC_Os02g08130	LOC_Os07g30200	8.8785937
LOC_Os02g08100	LOC_Os02g16040	8.93862796
LOC_Os02g08410	LOC_Os03g64050	9.12654334
LOC_Os02g08410	LOC_Os03g62070	9.64995844
LOC_Os02g08490	LOC_Os12g12580	8.81611258

InteractorA	InteractorB	Z score
LOC_Os02g08420	LOC_Os09g23540	8.92552002
LOC_Os02g08490	LOC_Os09g37100	9.54463797
LOC_Os02g08100	LOC_Os09g20284	9.00651148
LOC_Os02g08100	LOC_Os02g37420	9.24580655
LOC_Os02g07870	LOC_Os06g46340	11.0972933
LOC_Os02g08100	LOC_Os09g32952	9.04136709
LOC_Os02g08100	LOC_Os09g10230	8.89461846
LOC_Os02g08130	LOC_Os06g09910	9.36115191
LOC_Os02g08100	LOC_Os07g47420	9.27661254
LOC_Os02g08130	LOC_Os12g43370	9.975877
LOC_Os02g08490	LOC_Os03g16110	9.37321853
LOC_Os02g08490	LOC_Os06g46000	8.87467768
LOC_Os02g08490	LOC_Os09g10230	9.26974261
LOC_Os02g08100	LOC_Os02g35310	9.30232675
LOC_Os02g08410	LOC_Os04g02820	8.83432134
LOC_Os02g08130	LOC_Os05g08100	11.7741208
LOC_Os02g08130	LOC_Os03g01800	9.46741321
LOC_Os02g08420	LOC_Os07g25150	9.12488661
LOC_Os02g08490	LOC_Os05g36270	9.75851331
LOC_Os02g08490	LOC_Os04g41960	9.65794191
LOC_Os02g08130	LOC_Os06g07878	8.81085593
LOC_Os02g08410	LOC_Os07g47490	9.08731452
LOC_Os02g08100	LOC_Os05g30480	9.59206425
LOC_Os02g08100	LOC_Os05g42350	12.0915047
LOC_Os02g08420	LOC_Os06g06040	8.95858049
LOC_Os02g08130	LOC_Os06g07978	8.81085593
LOC_Os02g08490	LOC_Os11g05880	10.0474867
LOC_Os02g08420	LOC_Os12g31370	9.02027467
LOC_Os02g08420	LOC_Os06g35660	9.42798688
LOC_Os02g08100	LOC_Os03g30950	8.87710064
LOC_Os02g08490	LOC_Os07g48780	9.17798558
LOC_Os02g08130	LOC_Os07g05150	9.29044641
LOC_Os02g08100	LOC_Os09g30360	8.82525549
LOC_Os02g08410	LOC_Os06g42130	9.38290156
LOC_Os02g08490	LOC_Os07g41050	9.62455535
LOC_Os02g08100	LOC_Os07g43390	9.2138987
LOC_Os02g08420	LOC_Os06g37180	8.99828203
LOC_Os02g08420	LOC_Os03g55874	12.0697048
LOC_Os02g08130	LOC_Os12g05410	10.2102494
LOC_Os02g08100	LOC_Os07g44740	9.06739934
LOC_Os02g08130	LOC_Os07g02340	9.0047668
LOC_Os02g08100	LOC_Os02g08520	9.04021741
LOC_Os02g08490	LOC_Os05g07880	9.19695591

InteractorA	InteractorB	Z score
LOC_Os02g08130	LOC_Os04g44920	9.6399392
LOC_Os02g08130	LOC_Os07g03730	10.7414815
LOC_Os02g08100	LOC_Os02g47800	9.2359184
LOC_Os02g08490	LOC_Os03g21950	9.11814322
LOC_Os02g08490	LOC_Os05g34770	10.6465228
LOC_Os02g08100	LOC_Os06g10910	9.76459569
LOC_Os02g08130	LOC_Os08g37490	9.2554201
LOC_Os02g08410	LOC_Os06g02490	8.91702853
LOC_Os02g08100	LOC_Os03g17700	8.92232362
LOC_Os02g08130	LOC_Os05g37390	9.00462677
LOC_Os02g08490	LOC_Os12g33946	9.99524335
LOC_Os02g08410	LOC_Os04g55410	9.40282637
LOC_Os02g08130	LOC_Os10g08022	8.95212963
LOC_Os02g08100	LOC_Os08g39420	9.00504333
LOC_Os02g08100	LOC_Os10g08022	10.1859204
LOC_Os02g07870	LOC_Os07g12730	8.97550966
LOC_Os02g08130	LOC_Os12g40510	9.12863692
LOC_Os02g08130	LOC_Os03g58530	9.33693538
LOC_Os02g08490	LOC_Os12g10720	9.71178354
LOC_Os02g08410	LOC_Os02g41680	9.19798056
LOC_Os02g08490	LOC_Os06g51060	10.2806925
LOC_Os02g08490	LOC_Os07g09340	8.81008882
LOC_Os02g08490	LOC_Os08g28820	13.4609285
LOC_Os02g08410	LOC_Os07g42940	9.04686235
LOC_Os02g08490	LOC_Os07g36140	8.82262529
LOC_Os02g08410	LOC_Os04g30800	9.39779821
LOC_Os02g08100	LOC_Os07g35880	8.86398375
LOC_Os02g08100	LOC_Os11g05470	9.75049597
LOC_Os02g08490	LOC_Os03g55090	10.1591477
LOC_Os02g08130	LOC_Os02g56014	9.17567592
LOC_Os02g08100	LOC_Os03g32170	9.38897629
LOC_Os02g07870	LOC_Os08g01660	9.03258637
LOC_Os02g08420	LOC_Os02g27940	9.49092216
LOC_Os02g08420	LOC_Os06g42130	9.88096339
LOC_Os02g08420	LOC_Os11g02600	10.0485928
LOC_Os02g08490	LOC_Os04g57390	8.81891305
LOC_Os02g08420	LOC_Os08g31870	8.97226699
LOC_Os02g08490	LOC_Os11g47760	9.24444402
LOC_Os02g08410	LOC_Os02g14110	8.98450772
LOC_Os02g08490	LOC_Os05g39960	9.07769311
LOC_Os02g08410	LOC_Os09g37100	9.53306995
LOC_Os02g08130	LOC_Os11g10480	8.95899482
LOC_Os02g08420	LOC_Os02g18880	10.8572272

InteractorA	InteractorB	Z score
LOC_Os02g08100	LOC_Os06g39140	10.088374
LOC_Os02g08130	LOC_Os09g10200	8.98207575
LOC_Os02g08490	LOC_Os04g46910	8.97593189
LOC_Os02g08490	LOC_Os04g26920	9.19418485
LOC_Os02g08130	LOC_Os10g40720	9.05404669
LOC_Os02g08490	LOC_Os03g20370	9.17798558
LOC_Os02g08100	LOC_Os10g31000	9.56810715
LOC_Os02g08100	LOC_Os03g18130	8.902591
LOC_Os02g08100	LOC_Os09g31430	9.42341577
LOC_Os02g08490	LOC_Os10g42940	9.74739941
LOC_Os02g08100	LOC_Os05g35770	9.60907154
LOC_Os02g08410	LOC_Os12g40510	9.34186462
LOC_Os02g08420	LOC_Os07g38730	8.93049674
LOC_Os02g08130	LOC_Os11g08460	10.4508428
LOC_Os02g08100	LOC_Os06g12090	9.33820687
LOC_Os02g08420	LOC_Os07g35940	9.79363924
LOC_Os02g08420	LOC_Os10g01570	9.15084987
LOC_Os02g08490	LOC_Os02g27760	9.12804927
LOC_Os02g08100	LOC_Os09g30412	9.45337011
LOC_Os02g08100	LOC_Os03g22120	9.56567736
LOC_Os02g08490	LOC_Os07g46990	8.85182521
LOC_Os02g08490	LOC_Os10g28200	10.1048932
LOC_Os02g08130	LOC_Os12g16220	10.2893213
LOC_Os02g08490	LOC_Os05g42190	9.99306478
LOC_Os02g08100	LOC_Os06g39230	10.7817943
LOC_Os02g08130	LOC_Os04g33190	9.14867939
LOC_Os02g08490	LOC_Os03g16210	9.82062553
LOC_Os02g08410	LOC_Os02g22140	9.60068352
LOC_Os02g08100	LOC_Os10g36650	9.72860215
LOC_Os02g08490	LOC_Os09g31486	8.94413607
LOC_Os02g08130	LOC_Os07g46990	9.13806848
LOC_Os02g08490	LOC_Os02g52230	8.80726004
LOC_Os02g08130	LOC_Os10g25140	10.1157908
LOC_Os02g08100	LOC_Os10g26010	10.0562859
LOC_Os02g08420	LOC_Os06g21570	9.65964618
LOC_Os02g08420	LOC_Os03g58300	10.0703014
LOC_Os02g08100	LOC_Os07g02340	9.15221785
LOC_Os02g07870	LOC_Os09g39380	9.11403134
LOC_Os02g08100	LOC_Os07g16970	9.257525
LOC_Os02g08100	LOC_Os03g61920	9.96822816
LOC_Os02g08100	LOC_Os03g64030	8.87609525
LOC_Os02g08130	LOC_Os03g15120	9.40727823
LOC_Os02g09490	LOC_Os10g23900	9.23086318

InteractorA	InteractorB	Z score
LOC_Os02g10070	LOC_Os08g42910	9.75221825
LOC_Os02g09490	LOC_Os10g09860	9.14301122
LOC_Os02g09490	LOC_Os09g36900	9.16239732
LOC_Os02g09490	LOC_Os02g52230	9.15274473
LOC_Os02g10070	LOC_Os05g04340	10.2581041
LOC_Os02g10070	LOC_Os04g40874	9.17312756
LOC_Os02g10310	LOC_Os04g43410	9.0240762
LOC_Os02g10070	LOC_Os06g06090	10.7162673
LOC_Os02g10320	LOC_Os03g21260	9.63187297
LOC_Os02g10310	LOC_Os05g12180	8.96737292
LOC_Os02g09490	LOC_Os08g42910	9.30305798
LOC_Os02g09490	LOC_Os10g29470	10.5528488
LOC_Os02g10070	LOC_Os12g37360	10.9425659
LOC_Os02g09490	LOC_Os08g44520	9.43983238
LOC_Os02g10320	LOC_Os03g49350	8.8890254
LOC_Os02g09490	LOC_Os10g17660	8.87284125
LOC_Os02g10070	LOC_Os03g09910	8.86541467
LOC_Os02g09150	LOC_Os02g14770	9.22538233
LOC_Os02g10310	LOC_Os10g08670	8.86191023
LOC_Os02g09150	LOC_Os03g17690	8.86368689
LOC_Os02g10070	LOC_Os12g22680	8.86251276
LOC_Os02g09490	LOC_Os07g06970	8.86402712
LOC_Os02g10070	LOC_Os03g61330	9.1673903
LOC_Os02g10320	LOC_Os07g46990	8.81482565
LOC_Os02g10070	LOC_Os04g53230	9.23660025
LOC_Os02g10310	LOC_Os10g39840	9.59606941
LOC_Os02g10320	LOC_Os03g22020	8.85237069
LOC_Os02g09490	LOC_Os09g23560	9.46773853
LOC_Os02g10070	LOC_Os11g33240	25.1332007
LOC_Os02g09150	LOC_Os10g20910	8.94326399
LOC_Os02g10070	LOC_Os06g11290	9.48788223
LOC_Os02g09490	LOC_Os02g56700	8.97716461
LOC_Os02g09150	LOC_Os12g44020	8.98699354
LOC_Os02g09150	LOC_Os07g34140	9.15871763
LOC_Os02g10310	LOC_Os02g49720	9.59224609
LOC_Os02g09490	LOC_Os03g13200	9.21849235
LOC_Os02g09490	LOC_Os06g21820	9.1326158
LOC_Os02g10070	LOC_Os10g31950	10.8293053
LOC_Os02g09150	LOC_Os03g51740	8.83781922
LOC_Os02g09490	LOC_Os07g46310	8.93734976
LOC_Os02g10070	LOC_Os08g42730	9.65957494
LOC_Os02g09490	LOC_Os08g17680	9.28431403
LOC_Os02g10310	LOC_Os04g43800	9.2161637

InteractorA	InteractorB	Z score
LOC_Os02g10310	LOC_Os03g47610	9.47180953
LOC_Os02g09150	LOC_Os09g23550	9.30957187
LOC_Os02g10310	LOC_Os03g55280	10.2572833
LOC_Os02g10070	LOC_Os10g42280	9.70881097
LOC_Os02g09490	LOC_Os03g01630	9.36486859
LOC_Os02g09490	LOC_Os06g12790	9.08109716
LOC_Os02g10320	LOC_Os09g26880	9.17161393
LOC_Os02g10070	LOC_Os12g16200	9.4669486
LOC_Os02g10310	LOC_Os05g37690	8.91220399
LOC_Os02g09490	LOC_Os07g22650	8.83090324
LOC_Os02g09490	LOC_Os04g32460	9.11024521
LOC_Os02g10310	LOC_Os04g33740	9.25429988
LOC_Os02g09490	LOC_Os02g10320	9.18346789
LOC_Os02g10310	LOC_Os03g41438	9.62513879
LOC_Os02g10070	LOC_Os09g25390	9.53630631
LOC_Os02g09490	LOC_Os12g17540	9.2390488
LOC_Os02g10310	LOC_Os11g39540	12.0953641
LOC_Os02g10070	LOC_Os04g54330	9.76156953
LOC_Os02g08520	LOC_Os07g05180	9.64673341
LOC_Os02g10070	LOC_Os02g41470	8.9087576
LOC_Os02g10310	LOC_Os06g35940	9.2821127
LOC_Os02g10310	LOC_Os03g44484	9.85524329
LOC_Os02g09490	LOC_Os12g40830	9.70618379
LOC_Os02g08520	LOC_Os09g31490	9.46857901
LOC_Os02g08520	LOC_Os10g01570	8.89317805
LOC_Os02g10320	LOC_Os08g28680	10.0089943
LOC_Os02g09490	LOC_Os04g15920	9.42717026
LOC_Os02g09490	LOC_Os04g57090	9.27027503
LOC_Os02g09490	LOC_Os02g16040	9.44451332
LOC_Os02g10320	LOC_Os07g02350	9.13173286
LOC_Os02g09490	LOC_Os10g28050	9.29834904
LOC_Os02g09150	LOC_Os03g58430	9.57419798
LOC_Os02g09490	LOC_Os04g52280	9.2541966
LOC_Os02g10310	LOC_Os03g05590	9.210481
LOC_Os02g09490	LOC_Os04g24520	9.29932371
LOC_Os02g10320	LOC_Os02g33110	8.97768986
LOC_Os02g09150	LOC_Os05g47890	8.89350394
LOC_Os02g08520	LOC_Os06g35480	8.81692721
LOC_Os02g09490	LOC_Os07g28480	9.20724075
LOC_Os02g10310	LOC_Os09g38030	9.34889966
LOC_Os02g10320	LOC_Os07g48020	8.84941043
LOC_Os02g10070	LOC_Os02g41670	9.31413825
LOC_Os02g10320	LOC_Os03g53650	9.00395678

InteractorA	InteractorB	Z score
LOC_Os02g09490	LOC_Os04g38600	9.11436789
LOC_Os02g08520	LOC_Os09g10230	9.3542479
LOC_Os02g10070	LOC_Os03g26450	10.6259721
LOC_Os02g10320	LOC_Os09g39380	8.851486
LOC_Os02g10310	LOC_Os03g02680	8.86009373
LOC_Os02g10070	LOC_Os05g46550	9.52292336
LOC_Os02g09490	LOC_Os06g36820	9.42229185
LOC_Os02g10070	LOC_Os12g39630	9.73689197
LOC_Os02g09490	LOC_Os09g39570	9.3220931
LOC_Os02g10070	LOC_Os03g55070	9.58799217
LOC_Os02g09490	LOC_Os10g17680	8.87284125
LOC_Os02g09490	LOC_Os07g26660	9.21119905
LOC_Os02g08520	LOC_Os05g05830	10.0134571
LOC_Os02g10310	LOC_Os11g10510	12.6077768
LOC_Os02g09490	LOC_Os10g28350	9.8495566
LOC_Os02g08520	LOC_Os08g43190	9.05363092
LOC_Os02g10070	LOC_Os07g34190	10.2612545
LOC_Os02g10070	LOC_Os02g24632	9.23509477
LOC_Os02g10310	LOC_Os06g27770	9.37503835
LOC_Os02g09490	LOC_Os03g10620	9.44012101
LOC_Os02g10310	LOC_Os04g41960	9.4117501
LOC_Os02g09490	LOC_Os04g59150	10.8888522
LOC_Os02g09490	LOC_Os03g21260	9.14185638
LOC_Os02g10320	LOC_Os04g37460	9.00211229
LOC_Os02g10070	LOC_Os06g36880	9.18249515
LOC_Os02g10320	LOC_Os03g48770	9.29612515
LOC_Os02g08520	LOC_Os06g42130	8.90651735
LOC_Os02g10070	LOC_Os03g10190	9.38114237
LOC_Os02g09490	LOC_Os03g59710	9.55473968
LOC_Os02g10070	LOC_Os02g19770	9.79287841
LOC_Os02g10310	LOC_Os03g55070	8.99342542
LOC_Os02g10070	LOC_Os08g02700	10.6156412
LOC_Os02g09490	LOC_Os12g36950	8.82989493
LOC_Os02g09490	LOC_Os02g18880	9.19609803
LOC_Os02g09490	LOC_Os04g31960	9.46126039
LOC_Os02g10070	LOC_Os06g04620	9.01706971
LOC_Os02g09490	LOC_Os03g12660	10.3384425
LOC_Os02g10320	LOC_Os09g36800	9.61459938
LOC_Os02g09490	LOC_Os09g23540	9.16891062
LOC_Os02g09150	LOC_Os10g29620	9.17015303
LOC_Os02g10310	LOC_Os06g07140	9.57746392
LOC_Os02g10320	LOC_Os06g30970	8.94071734
LOC_Os02g10070	LOC_Os02g14130	8.94522251

InteractorA	InteractorB	Z score
LOC_Os02g10070	LOC_Os03g59700	10.6519495
LOC_Os02g10070	LOC_Os02g45540	9.04203198
LOC_Os02g10070	LOC_Os10g38670	9.52215142
LOC_Os02g10390	LOC_Os02g50860	9.35814
LOC_Os02g12730	LOC_Os12g21798	9.43216709
LOC_Os02g12730	LOC_Os12g44030	9.15012863
LOC_Os02g12730	LOC_Os06g27770	9.00451052
LOC_Os02g12730	LOC_Os03g09910	9.54222468
LOC_Os02g12780	LOC_Os04g17650	8.83840025
LOC_Os02g12730	LOC_Os05g44340	9.95568172
LOC_Os02g10390	LOC_Os07g08500	9.57179653
LOC_Os02g10830	LOC_Os07g28480	9.08330333
LOC_Os02g12580	LOC_Os09g20090	10.3360902
LOC_Os02g12730	LOC_Os08g31870	9.23478462
LOC_Os02g12580	LOC_Os05g36290	9.1357938
LOC_Os02g10830	LOC_Os08g34170	8.98856268
LOC_Os02g12580	LOC_Os09g39570	9.87056159
LOC_Os02g12730	LOC_Os04g40310	8.95477744
LOC_Os02g10390	LOC_Os09g21770	9.32870832
LOC_Os02g12580	LOC_Os02g35310	9.45002448
LOC_Os02g10390	LOC_Os06g10330	8.90350306
LOC_Os02g12730	LOC_Os05g01050	9.20012646
LOC_Os02g10390	LOC_Os05g35330	10.0243569
LOC_Os02g10390	LOC_Os02g32490	8.82961623
LOC_Os02g12580	LOC_Os03g11530	9.71721615
LOC_Os02g12780	LOC_Os06g40940	9.35310944
LOC_Os02g10390	LOC_Os07g19040	8.9918327
LOC_Os02g10990	LOC_Os03g04970	9.39765428
LOC_Os02g12730	LOC_Os02g50880	9.23845813
LOC_Os02g10830	LOC_Os03g08530	9.04748441
LOC_Os02g12730	LOC_Os07g36130	8.93848681
LOC_Os02g12730	LOC_Os02g50860	9.49901453
LOC_Os02g12580	LOC_Os08g09370	10.5758449
LOC_Os02g12730	LOC_Os08g28190	8.98071375
LOC_Os02g12780	LOC_Os04g09540	9.10330538
LOC_Os02g10990	LOC_Os12g10570	9.32474232
LOC_Os02g12780	LOC_Os05g44140	9.44600387
LOC_Os02g10990	LOC_Os06g46372	8.89005451
LOC_Os02g12730	LOC_Os09g39500	8.96511223
LOC_Os02g10390	LOC_Os07g38430	9.07508187
LOC_Os02g10390	LOC_Os06g07080	13.4926508
LOC_Os02g10830	LOC_Os07g11440	9.75829822
LOC_Os02g12730	LOC_Os06g37660	9.23638392

InteractorA	InteractorB	Z score
LOC_Os02g12580	LOC_Os12g34380	9.87652149
LOC_Os02g12580	LOC_Os07g39290	9.42644787
LOC_Os02g10830	LOC_Os07g08170	8.94549741
LOC_Os02g10390	LOC_Os05g27950	9.44306562
LOC_Os02g12730	LOC_Os10g40700	10.2614813
LOC_Os02g12580	LOC_Os09g34960	9.56607064
LOC_Os02g10390	LOC_Os05g23860	9.12716416
LOC_Os02g12580	LOC_Os04g52450	10.9479021
LOC_Os02g12580	LOC_Os10g25130	12.2464934
LOC_Os02g12730	LOC_Os03g19930	9.61685642
LOC_Os02g12730	LOC_Os07g43510	9.46027175
LOC_Os02g12580	LOC_Os04g40620	9.32103354
LOC_Os02g12780	LOC_Os02g18930	8.96299981
LOC_Os02g12730	LOC_Os09g38620	9.12369575
LOC_Os02g10390	LOC_Os06g03770	9.18914144
LOC_Os02g10830	LOC_Os05g46580	9.42729963
LOC_Os02g12580	LOC_Os02g56690	9.07014185
LOC_Os02g12730	LOC_Os04g58880	9.01005397
LOC_Os02g12730	LOC_Os11g47760	10.4142902
LOC_Os02g10990	LOC_Os04g50880	9.45154946
LOC_Os02g12730	LOC_Os12g07720	8.82075558
LOC_Os02g12730	LOC_Os08g40170	8.90061915
LOC_Os02g10390	LOC_Os11g29400	9.28871794
LOC_Os02g10990	LOC_Os12g33610	8.85350342
LOC_Os02g12730	LOC_Os07g07719	9.59876862
LOC_Os02g12580	LOC_Os07g38970	8.90524213
LOC_Os02g10990	LOC_Os05g28180	9.02316466
LOC_Os02g10390	LOC_Os03g44150	10.5166932
LOC_Os02g12730	LOC_Os10g13800	9.70244645
LOC_Os02g12730	LOC_Os10g26600	8.93568247
LOC_Os02g10990	LOC_Os12g23170	9.26088018
LOC_Os02g10390	LOC_Os04g24520	8.85311672
LOC_Os02g10830	LOC_Os09g23540	9.72486588
LOC_Os02g12580	LOC_Os02g30200	8.87756
LOC_Os02g12730	LOC_Os08g37490	9.58095228
LOC_Os02g12580	LOC_Os10g25930	9.79022105
LOC_Os02g12580	LOC_Os07g12730	8.90937653
LOC_Os02g12730	LOC_Os03g21900	10.6345392
LOC_Os02g10320	LOC_Os11g14040	10.5213573
LOC_Os02g10390	LOC_Os11g10510	11.215806
LOC_Os02g12580	LOC_Os10g21310	9.61124023
LOC_Os02g10390	LOC_Os02g53180	9.30211073
LOC_Os02g10830	LOC_Os02g13110	9.13283173

InteractorA	InteractorB	Z score
LOC_Os02g10320	LOC_Os11g08120	9.11221219
LOC_Os02g10990	LOC_Os04g26910	9.58508488
LOC_Os02g12580	LOC_Os03g50490	8.92098507
LOC_Os02g12580	LOC_Os12g25120	9.97952635
LOC_Os02g10990	LOC_Os07g34520	9.25598579
LOC_Os02g10320	LOC_Os12g22650	9.08011363
LOC_Os02g10390	LOC_Os03g51600	8.90478898
LOC_Os02g12580	LOC_Os09g39380	9.07906425
LOC_Os02g12730	LOC_Os03g13170	8.96508177
LOC_Os02g12730	LOC_Os02g18550	9.04414519
LOC_Os02g12580	LOC_Os02g17780	8.91283513
LOC_Os02g12580	LOC_Os09g20260	10.2557457
LOC_Os02g12730	LOC_Os06g35520	9.08316043
LOC_Os02g10390	LOC_Os10g21268	10.0243745
LOC_Os02g10990	LOC_Os10g30200	9.31969707
LOC_Os02g12730	LOC_Os03g31210	8.92909132
LOC_Os02g10390	LOC_Os07g05800	8.95860002
LOC_Os02g12730	LOC_Os12g10730	9.23812756
LOC_Os02g12580	LOC_Os03g51740	9.23894897
LOC_Os02g13330	LOC_Os05g38760	9.67305804
LOC_Os02g13530	LOC_Os03g16860	9.77220312
LOC_Os02g13110	LOC_Os12g24650	9.17237653
LOC_Os02g13830	LOC_Os02g53790	8.90606803
LOC_Os02g13140	LOC_Os06g35560	9.78309754
LOC_Os02g13140	LOC_Os07g06970	8.80539792
LOC_Os02g13140	LOC_Os07g03368	10.591768
LOC_Os02g13530	LOC_Os02g16040	10.2855376
LOC_Os02g13530	LOC_Os04g37990	8.90988548
LOC_Os02g13330	LOC_Os09g10300	9.08315158
LOC_Os02g13530	LOC_Os03g16740	9.26710529
LOC_Os02g13110	LOC_Os11g26910	9.19639269
LOC_Os02g12780	LOC_Os11g37950	8.92389858
LOC_Os02g13530	LOC_Os03g13170	9.7092682
LOC_Os02g12900	LOC_Os04g16872	10.557821
LOC_Os02g13110	LOC_Os06g51060	8.81774501
LOC_Os02g13530	LOC_Os08g36910	9.2523385
LOC_Os02g13530	LOC_Os07g34589	9.28727984
LOC_Os02g13140	LOC_Os03g52970	9.3543823
LOC_Os02g13330	LOC_Os03g18580	10.1834666
LOC_Os02g13140	LOC_Os03g13950	8.94478978
LOC_Os02g13110	LOC_Os07g42950	9.00323002
LOC_Os02g13140	LOC_Os02g15640	9.118854
LOC_Os02g13110	LOC_Os07g25150	9.89584047

InteractorA	InteractorB	Z score
LOC_Os02g12780	LOC_Os12g06660	9.03153329
LOC_Os02g12900	LOC_Os06g37150	8.95965017
LOC_Os02g13530	LOC_Os06g35940	9.90291663
LOC_Os02g13530	LOC_Os02g15640	8.82500042
LOC_Os02g13140	LOC_Os11g07440	9.08131503
LOC_Os02g13140	LOC_Os06g49970	8.8740388
LOC_Os02g13330	LOC_Os05g26890	9.07128513
LOC_Os02g13110	LOC_Os11g01872	8.95889818
LOC_Os02g13530	LOC_Os03g01900	8.99963906
LOC_Os02g13330	LOC_Os02g57150	12.2395527
LOC_Os02g13110	LOC_Os08g38900	9.66019566
LOC_Os02g13140	LOC_Os10g38189	9.2023003
LOC_Os02g13530	LOC_Os10g08580	9.07821913
LOC_Os02g12900	LOC_Os05g38760	10.0298391
LOC_Os02g12900	LOC_Os08g08070	9.45015451
LOC_Os02g13330	LOC_Os12g23630	9.81046695
LOC_Os02g13530	LOC_Os07g38890	9.49805304
LOC_Os02g13530	LOC_Os02g19770	10.0830462
LOC_Os02g13140	LOC_Os03g58290	8.83774596
LOC_Os02g12900	LOC_Os05g05830	9.89168722
LOC_Os02g13140	LOC_Os05g37690	9.06186868
LOC_Os02g13140	LOC_Os10g38740	8.8511413
LOC_Os02g12900	LOC_Os05g40420	9.77207058
LOC_Os02g13110	LOC_Os05g05800	9.03254024
LOC_Os02g13830	LOC_Os03g44484	8.87019467
LOC_Os02g13530	LOC_Os12g13320	8.93677568
LOC_Os02g12780	LOC_Os08g28800	8.91767997
LOC_Os02g13330	LOC_Os03g55389	9.89288704
LOC_Os02g13330	LOC_Os09g10200	10.226885
LOC_Os02g13110	LOC_Os12g12514	9.43133369
LOC_Os02g13140	LOC_Os02g52390	11.9516348
LOC_Os02g13330	LOC_Os03g39610	9.39206818
LOC_Os02g13330	LOC_Os03g53860	9.4997016
LOC_Os02g13530	LOC_Os10g39680	10.1263946
LOC_Os02g12900	LOC_Os04g12900	9.18645132
LOC_Os02g13530	LOC_Os12g44030	10.0925253
LOC_Os02g13530	LOC_Os11g47580	8.83257802
LOC_Os02g13110	LOC_Os03g47610	9.29987292
LOC_Os02g13140	LOC_Os02g55420	10.1209913
LOC_Os02g12900	LOC_Os07g36190	9.20483337
LOC_Os02g13110	LOC_Os03g01900	9.08034747
LOC_Os02g13140	LOC_Os12g38760	9.41608579
LOC_Os02g12900	LOC_Os12g07830	8.87262714

InteractorA	InteractorB	Z score
LOC_Os02g12900	LOC_Os08g44340	9.25752051
LOC_Os02g13140	LOC_Os08g14760	8.81766386
LOC_Os02g13140	LOC_Os11g08120	9.24547712
LOC_Os02g13330	LOC_Os05g33140	9.00618357
LOC_Os02g12900	LOC_Os08g04560	9.08402854
LOC_Os02g13110	LOC_Os08g42410	11.0510652
LOC_Os02g13110	LOC_Os07g28280	8.93237263
LOC_Os02g12780	LOC_Os11g01872	8.82971885
LOC_Os02g13110	LOC_Os06g49970	9.57040882
LOC_Os02g13830	LOC_Os03g26450	9.90611329
LOC_Os02g13530	LOC_Os09g39500	9.70924841
LOC_Os02g13530	LOC_Os03g55874	8.93692555
LOC_Os02g13140	LOC_Os07g07060	9.10363263
LOC_Os02g12900	LOC_Os10g10434	9.16790659
LOC_Os02g13330	LOC_Os06g46372	9.65300411
LOC_Os02g13110	LOC_Os03g59020	9.15897342
LOC_Os02g13330	LOC_Os02g40000	9.03697308
LOC_Os02g14059	LOC_Os10g28200	9.07590367
LOC_Os02g14130	LOC_Os12g42884	8.82507665
LOC_Os02g14110	LOC_Os06g37080	9.074326
LOC_Os02g14110	LOC_Os10g28200	8.99792404
LOC_Os02g14130	LOC_Os11g20790	9.88101614
LOC_Os02g14130	LOC_Os09g08720	9.42504963
LOC_Os02g14110	LOC_Os09g10230	9.6530893
LOC_Os02g14130	LOC_Os08g42560	9.82544246
LOC_Os02g14059	LOC_Os05g39580	9.80706789
LOC_Os02g14110	LOC_Os08g28820	8.97758129
LOC_Os02g13830	LOC_Os05g37700	8.92613427
LOC_Os02g14059	LOC_Os05g11550	8.9765614
LOC_Os02g14110	LOC_Os04g20070	8.80680597
LOC_Os02g13830	LOC_Os06g29844	8.97810105
LOC_Os02g14059	LOC_Os09g32800	9.04343648
LOC_Os02g14130	LOC_Os03g30470	9.27802841
LOC_Os02g14059	LOC_Os02g56460	8.96996132
LOC_Os02g14130	LOC_Os05g31040	10.5288039
LOC_Os02g14110	LOC_Os05g38560	9.80011091
LOC_Os02g14160	LOC_Os03g21120	9.62281451
LOC_Os02g14160	LOC_Os05g33730	9.20868413
LOC_Os02g14130	LOC_Os04g16760	8.81976054
LOC_Os02g13830	LOC_Os10g41550	10.3258952
LOC_Os02g14110	LOC_Os08g43170	8.90087618
LOC_Os02g14110	LOC_Os06g43660	9.6934925
LOC_Os02g14110	LOC_Os05g08430	9.25609992

InteractorA	InteractorB	Z score
LOC_Os02g13830	LOC_Os09g32840	10.2791923
LOC_Os02g14130	LOC_Os07g31770	9.67084332
LOC_Os02g14130	LOC_Os03g53230	10.9518478
LOC_Os02g14059	LOC_Os03g04410	9.24071865
LOC_Os02g14110	LOC_Os05g01600	8.92184391
LOC_Os02g14110	LOC_Os07g05150	10.750946
LOC_Os02g14130	LOC_Os02g57720	9.44641085
LOC_Os02g14110	LOC_Os03g17480	9.01301259
LOC_Os02g14170	LOC_Os05g45810	9.82678621
LOC_Os02g13830	LOC_Os04g32460	8.80325102
LOC_Os02g14160	LOC_Os11g48110	9.06988503
LOC_Os02g14059	LOC_Os07g46630	8.98730778
LOC_Os02g14130	LOC_Os11g48110	8.95147655
LOC_Os02g14130	LOC_Os07g43470	8.81471771
LOC_Os02g14059	LOC_Os04g16770	8.90050553
LOC_Os02g14130	LOC_Os03g61340	9.9574489
LOC_Os02g14160	LOC_Os12g40830	8.87015051
LOC_Os02g13830	LOC_Os05g31040	9.29248932
LOC_Os02g13830	LOC_Os05g27940	9.64841944
LOC_Os02g14059	LOC_Os10g11140	8.97815765
LOC_Os02g14170	LOC_Os02g51930	9.22681187
LOC_Os02g13830	LOC_Os06g46340	10.8297465
LOC_Os02g14130	LOC_Os03g58980	9.39290162
LOC_Os02g14059	LOC_Os05g36270	9.531528
LOC_Os02g14130	LOC_Os04g43400	9.6054804
LOC_Os02g14170	LOC_Os12g41110	8.84429324
LOC_Os02g13830	LOC_Os07g40580	11.0788868
LOC_Os02g14160	LOC_Os05g47640	8.8909315
LOC_Os02g13830	LOC_Os06g36670	9.23668785
LOC_Os02g14059	LOC_Os08g04560	10.3258219
LOC_Os02g14059	LOC_Os12g08280	8.82436443
LOC_Os02g14110	LOC_Os07g08660	9.02808644
LOC_Os02g14160	LOC_Os03g17980	8.88932745
LOC_Os02g14110	LOC_Os03g16740	9.65131792
LOC_Os02g14130	LOC_Os02g42520	8.9906504
LOC_Os02g14130	LOC_Os10g25930	9.75372626
LOC_Os02g14130	LOC_Os02g46970	9.19615581
LOC_Os02g14110	LOC_Os05g12190	9.24536452
LOC_Os02g14059	LOC_Os03g48471	9.37999504
LOC_Os02g14130	LOC_Os02g46130	9.88787002
LOC_Os02g14130	LOC_Os10g38600	9.15334371
LOC_Os02g14130	LOC_Os05g01675	8.81976054
LOC_Os02g14059	LOC_Os05g29880	9.33114792

InteractorA	InteractorB	Z score
LOC_Os02g14130	LOC_Os06g10910	10.5824892
LOC_Os02g14110	LOC_Os03g53200	8.86693956
LOC_Os02g14170	LOC_Os03g13200	8.9643187
LOC_Os02g14110	LOC_Os11g19320	8.90059425
LOC_Os02g14170	LOC_Os09g09270	9.44527106
LOC_Os02g14130	LOC_Os10g17650	9.35135134
LOC_Os02g14110	LOC_Os04g33740	9.67499983
LOC_Os02g14110	LOC_Os02g14929	10.9831631
LOC_Os02g14170	LOC_Os09g36830	9.61872554
LOC_Os02g13830	LOC_Os04g39814	9.48489481
LOC_Os02g14059	LOC_Os03g53860	9.07984182
LOC_Os02g14170	LOC_Os05g11550	9.77409099
LOC_Os02g14110	LOC_Os02g55420	12.7892452
LOC_Os02g14130	LOC_Os03g60620	9.91405741
LOC_Os02g14160	LOC_Os08g40140	10.0240986
LOC_Os02g14059	LOC_Os10g30200	8.90291811
LOC_Os02g14059	LOC_Os08g35420	8.90050553
LOC_Os02g14059	LOC_Os07g04240	10.1311325
LOC_Os02g14130	LOC_Os03g10500	10.2749605
LOC_Os02g14160	LOC_Os08g33710	8.86600113
LOC_Os02g14059	LOC_Os02g42320	8.96349719
LOC_Os02g14130	LOC_Os04g57400	9.00028524
LOC_Os02g14170	LOC_Os05g41210	9.05037657
LOC_Os02g14059	LOC_Os11g20790	9.13504971
LOC_Os02g14160	LOC_Os09g26380	9.41874382
LOC_Os02g14170	LOC_Os10g40090	9.18618373
LOC_Os02g14460	LOC_Os07g35940	8.87656158
LOC_Os02g14430	LOC_Os03g12290	9.02729118
LOC_Os02g14430	LOC_Os04g16760	10.2929665
LOC_Os02g14190	LOC_Os03g06330	9.60856635
LOC_Os02g14430	LOC_Os04g22660	8.88066884
LOC_Os02g14430	LOC_Os10g21250	9.28337901
LOC_Os02g14180	LOC_Os04g32010	8.97889173
LOC_Os02g14440	LOC_Os02g56100	9.62144941
LOC_Os02g14430	LOC_Os05g01675	10.2929598
LOC_Os02g14460	LOC_Os05g42350	8.85777323
LOC_Os02g14460	LOC_Os09g26380	10.5565864
LOC_Os02g14180	LOC_Os05g45810	8.80306709
LOC_Os02g14770	LOC_Os03g04970	9.01222175
LOC_Os02g14190	LOC_Os11g04954	9.01379636
LOC_Os02g14440	LOC_Os03g49350	9.67096237
LOC_Os02g14770	LOC_Os04g58880	10.0139569
LOC_Os02g14770	LOC_Os05g23860	9.02235425

InteractorA	InteractorB	Z score
LOC_Os02g14430	LOC_Os12g44010	9.76541074
LOC_Os02g14770	LOC_Os06g50300	8.9838949
LOC_Os02g14180	LOC_Os09g32810	9.09062744
LOC_Os02g14430	LOC_Os04g41310	9.51120574
LOC_Os02g14460	LOC_Os05g45420	10.9100697
LOC_Os02g14180	LOC_Os04g58200	9.10352597
LOC_Os02g14460	LOC_Os05g30480	10.59923
LOC_Os02g14190	LOC_Os11g16590	9.39772125
LOC_Os02g14440	LOC_Os04g45290	9.67412094
LOC_Os02g14180	LOC_Os06g45120	10.8423501
LOC_Os02g14460	LOC_Os12g16240	9.66916178
LOC_Os02g14770	LOC_Os08g20270	9.68051035
LOC_Os02g14770	LOC_Os05g46550	9.70589553
LOC_Os02g14460	LOC_Os12g38180	9.09768708
LOC_Os02g14440	LOC_Os05g01050	9.04329321
LOC_Os02g14460	LOC_Os06g37080	9.02195679
LOC_Os02g14440	LOC_Os03g45320	8.93056663
LOC_Os02g14460	LOC_Os03g16740	8.95526959
LOC_Os02g14190	LOC_Os10g11810	9.97618059
LOC_Os02g14430	LOC_Os07g48780	8.87771347
LOC_Os02g14190	LOC_Os05g38550	9.2137142
LOC_Os02g14440	LOC_Os06g11210	9.12091864
LOC_Os02g14460	LOC_Os03g13200	11.0513836
LOC_Os02g14770	LOC_Os05g37690	8.8438147
LOC_Os02g14180	LOC_Os03g45320	9.92807793
LOC_Os02g14460	LOC_Os08g28820	8.90596986
LOC_Os02g14460	LOC_Os11g05880	9.00216306
LOC_Os02g14190	LOC_Os09g27750	11.5721989
LOC_Os02g14460	LOC_Os12g44030	9.08905962
LOC_Os02g14770	LOC_Os02g38340	8.98455582
LOC_Os02g14460	LOC_Os12g31370	9.78945977
LOC_Os02g14190	LOC_Os11g40150	10.1338333
LOC_Os02g14770	LOC_Os07g39870	9.26721855
LOC_Os02g14440	LOC_Os02g25940	9.67160785
LOC_Os02g14770	LOC_Os06g07210	9.97003888
LOC_Os02g14460	LOC_Os02g36974	10.6974742
LOC_Os02g14190	LOC_Os03g18130	8.88555863
LOC_Os02g14430	LOC_Os02g46970	9.02087585
LOC_Os02g14770	LOC_Os07g38890	8.88892942
LOC_Os02g14190	LOC_Os09g07510	8.86775976
LOC_Os02g14440	LOC_Os08g20270	10.1416413
LOC_Os02g14180	LOC_Os02g52420	9.40734005
LOC_Os02g14460	LOC_Os04g40990	9.00230514

InteractorA	InteractorB	Z score
LOC_Os02g14430	LOC_Os12g08280	9.1435674
LOC_Os02g14180	LOC_Os08g28800	10.3264814
LOC_Os02g14460	LOC_Os04g12900	9.60317839
LOC_Os02g14460	LOC_Os02g34600	8.82034023
LOC_Os02g14460	LOC_Os03g18810	9.74165892
LOC_Os02g14190	LOC_Os04g46930	9.32205297
LOC_Os02g14770	LOC_Os03g13200	8.84891476
LOC_Os02g14770	LOC_Os03g47770	10.4450182
LOC_Os02g14180	LOC_Os06g19960	9.37858542
LOC_Os02g14440	LOC_Os03g16900	9.71858237
LOC_Os02g14460	LOC_Os10g28320	9.1091485
LOC_Os02g14180	LOC_Os10g25130	9.04164015
LOC_Os02g14190	LOC_Os07g31770	9.31217134
LOC_Os02g14430	LOC_Os03g21950	9.15727393
LOC_Os02g14770	LOC_Os03g10340	9.25500655
LOC_Os02g14460	LOC_Os09g37949	8.80596641
LOC_Os02g14770	LOC_Os03g01260	9.88126292
LOC_Os02g14430	LOC_Os03g45410	8.96341313
LOC_Os02g14440	LOC_Os03g38000	11.5240825
LOC_Os02g14180	LOC_Os06g50300	10.4312392
LOC_Os02g14770	LOC_Os07g46310	8.84302194
LOC_Os02g14770	LOC_Os08g34210	10.5936021
LOC_Os02g14460	LOC_Os02g52420	8.8097827
LOC_Os02g14190	LOC_Os10g38470	8.8560307
LOC_Os02g14180	LOC_Os04g48850	9.2117685
LOC_Os02g14460	LOC_Os05g37330	9.51949499
LOC_Os02g14460	LOC_Os03g27370	9.35847106
LOC_Os02g14430	LOC_Os04g53290	10.064473
LOC_Os02g14460	LOC_Os02g18930	9.21941934
LOC_Os02g14460	LOC_Os12g10720	10.235957
LOC_Os02g14460	LOC_Os10g39840	9.01402304
LOC_Os02g14180	LOC_Os04g29030	10.5706622
LOC_Os02g14190	LOC_Os06g28550	9.32153706
LOC_Os02g14190	LOC_Os07g29440	8.90059202
LOC_Os02g14770	LOC_Os04g40874	8.9666807
LOC_Os02g14460	LOC_Os03g63330	9.02165073
LOC_Os02g14180	LOC_Os07g49220	9.60818165
LOC_Os02g14430	LOC_Os07g34589	9.05549826
LOC_Os02g14440	LOC_Os07g38910	9.104105
LOC_Os02g14430	LOC_Os05g35400	9.16956979
LOC_Os02g14180	LOC_Os02g50240	9.08279204
LOC_Os02g14430	LOC_Os07g46460	10.1148067
LOC_Os02g14440	LOC_Os03g58430	8.98603363

InteractorA	InteractorB	Z score
LOC_Os02g14770	LOC_Os07g07470	9.60472376
LOC_Os02g14770	LOC_Os04g33480	9.4812227
LOC_Os02g14180	LOC_Os02g46970	9.64848891
LOC_Os02g14430	LOC_Os02g47610	9.01251602
LOC_Os02g14770	LOC_Os03g42840	10.3181158
LOC_Os02g14440	LOC_Os07g34260	8.89207298
LOC_Os02g14430	LOC_Os03g20370	8.87771347
LOC_Os02g14460	LOC_Os03g22060	9.00820016
LOC_Os02g14180	LOC_Os03g25320	9.69646776
LOC_Os02g14770	LOC_Os06g04280	9.00065353
LOC_Os02g14770	LOC_Os08g10608	8.84301134
LOC_Os02g14460	LOC_Os07g44370	10.0407303
LOC_Os02g14770	LOC_Os04g01674	9.01686865
LOC_Os02g15550	LOC_Os03g04410	9.34951409
LOC_Os02g15640	LOC_Os06g35940	8.83753914
LOC_Os02g14929	LOC_Os09g04730	9.31718513
LOC_Os02g15620	LOC_Os07g49120	10.7165564
LOC_Os02g16040	LOC_Os07g43260	9.92511292
LOC_Os02g15620	LOC_Os03g57120	9.12399206
LOC_Os02g15640	LOC_Os08g02400	9.69159137
LOC_Os02g15550	LOC_Os03g51740	9.07324752
LOC_Os02g14929	LOC_Os07g20544	9.31768932
LOC_Os02g14929	LOC_Os05g44340	9.08877851
LOC_Os02g15640	LOC_Os05g49880	9.25935724
LOC_Os02g15640	LOC_Os07g05800	9.95863421
LOC_Os02g15620	LOC_Os12g16220	9.27840811
LOC_Os02g15620	LOC_Os06g06730	9.91734102
LOC_Os02g14929	LOC_Os12g38770	10.7190746
LOC_Os02g15640	LOC_Os08g33710	9.79134368
LOC_Os02g15620	LOC_Os05g11710	9.70793844
LOC_Os02g15640	LOC_Os04g40990	10.1067307
LOC_Os02g15640	LOC_Os05g47980	9.4143078
LOC_Os02g14770	LOC_Os09g04680	8.82918432
LOC_Os02g15550	LOC_Os06g11210	9.6685092
LOC_Os02g14929	LOC_Os05g04690	9.73313564
LOC_Os02g15550	LOC_Os03g60580	8.9159638
LOC_Os02g16550	LOC_Os06g06090	9.82076525
LOC_Os02g16040	LOC_Os09g30412	10.4926675
LOC_Os02g15550	LOC_Os11g01872	9.10022125
LOC_Os02g14929	LOC_Os05g06300	9.59776722
LOC_Os02g14929	LOC_Os09g36450	8.83991517
LOC_Os02g14929	LOC_Os03g58530	9.22534162
LOC_Os02g14929	LOC_Os09g20260	10.3774158

InteractorA	InteractorB	Z score
LOC_Os02g15640	LOC_Os12g13390	9.31994546
LOC_Os02g15640	LOC_Os07g48050	9.18373922
LOC_Os02g14770	LOC_Os11g14040	8.82053688
LOC_Os02g14929	LOC_Os05g20050	9.40228273
LOC_Os02g15640	LOC_Os04g57090	9.1523041
LOC_Os02g16040	LOC_Os10g26010	8.85348919
LOC_Os02g16040	LOC_Os09g36710	10.1800561
LOC_Os02g15640	LOC_Os02g51830	9.95967176
LOC_Os02g15640	LOC_Os10g30580	9.34695811
LOC_Os02g15640	LOC_Os03g50250	8.82309406
LOC_Os02g14929	LOC_Os09g36710	9.06518556
LOC_Os02g15620	LOC_Os04g44890	9.12999821
LOC_Os02g14770	LOC_Os12g25120	8.9702166
LOC_Os02g15550	LOC_Os07g34190	9.09018385
LOC_Os02g16040	LOC_Os04g40990	9.19798981
LOC_Os02g15620	LOC_Os02g47840	10.3227379
LOC_Os02g14929	LOC_Os08g32620	10.6168058
LOC_Os02g15550	LOC_Os11g02440	9.41674626
LOC_Os02g14929	LOC_Os11g08445	10.0659927
LOC_Os02g15620	LOC_Os02g38840	9.13199526
LOC_Os02g14929	LOC_Os03g60400	8.93761493
LOC_Os02g16040	LOC_Os04g47360	9.06708827
LOC_Os02g16040	LOC_Os10g29470	9.84865684
LOC_Os02g15640	LOC_Os03g08530	11.4406709
LOC_Os02g14770	LOC_Os11g03290	8.96506021
LOC_Os02g15640	LOC_Os11g32520	8.80123729
LOC_Os02g14770	LOC_Os10g08710	9.33465044
LOC_Os02g15550	LOC_Os12g02370	10.4111209
LOC_Os02g14929	LOC_Os12g12580	8.97094633
LOC_Os02g14929	LOC_Os10g07229	10.0099972
LOC_Os02g14770	LOC_Os09g25390	8.85719302
LOC_Os02g15640	LOC_Os03g38020	9.06391601
LOC_Os02g14929	LOC_Os06g01850	9.59037878
LOC_Os02g14929	LOC_Os05g40990	10.1279084
LOC_Os02g15550	LOC_Os04g41960	9.01668268
LOC_Os02g16040	LOC_Os05g42350	9.43357482
LOC_Os02g16040	LOC_Os03g49600	9.46649019
LOC_Os02g15640	LOC_Os10g41410	8.95659688
LOC_Os02g16040	LOC_Os11g10520	8.81871123
LOC_Os02g15620	LOC_Os11g32650	9.19103384
LOC_Os02g14770	LOC_Os09g32800	10.0686808
LOC_Os02g14929	LOC_Os05g46290	9.72537717
LOC_Os02g14929	LOC_Os03g21120	8.93902353

InteractorA	InteractorB	Z score
LOC_Os02g16550	LOC_Os02g57770	8.85240842
LOC_Os02g14929	LOC_Os03g64050	9.06068898
LOC_Os02g15640	LOC_Os02g40000	9.22454484
LOC_Os02g16040	LOC_Os07g39270	8.85011858
LOC_Os02g15620	LOC_Os06g35730	9.70793844
LOC_Os02g14770	LOC_Os10g13800	9.68082766
LOC_Os02g16040	LOC_Os09g23530	9.23234374
LOC_Os02g16040	LOC_Os06g34690	10.1428317
LOC_Os02g16040	LOC_Os12g12560	9.84334895
LOC_Os02g16040	LOC_Os02g17390	9.15359616
LOC_Os02g15640	LOC_Os04g53810	8.88432067
LOC_Os02g14929	LOC_Os09g29200	8.99780919
LOC_Os02g14929	LOC_Os04g50880	9.21841914
LOC_Os02g17780	LOC_Os02g47020	8.95204038
LOC_Os02g18880	LOC_Os03g11530	9.16802417
LOC_Os02g17780	LOC_Os12g44010	9.56917552
LOC_Os02g17780	LOC_Os04g44890	8.97077497
LOC_Os02g18180	LOC_Os08g33820	9.34296403
LOC_Os02g16550	LOC_Os07g03368	11.6079871
LOC_Os02g18880	LOC_Os06g02390	9.30013584
LOC_Os02g18180	LOC_Os07g38430	9.06039956
LOC_Os02g18880	LOC_Os07g30970	9.96144605
LOC_Os02g17390	LOC_Os04g58710	9.78201942
LOC_Os02g18880	LOC_Os06g02380	8.93838721
LOC_Os02g18180	LOC_Os07g26900	9.01404875
LOC_Os02g18880	LOC_Os07g44370	9.68668932
LOC_Os02g18880	LOC_Os07g44790	9.13303202
LOC_Os02g18930	LOC_Os03g51550	9.54289052
LOC_Os02g18880	LOC_Os05g11730	9.79917862
LOC_Os02g17780	LOC_Os06g34690	9.50924103
LOC_Os02g18880	LOC_Os03g14120	9.10772589
LOC_Os02g17390	LOC_Os06g05250	9.79996598
LOC_Os02g18180	LOC_Os05g05620	10.1018066
LOC_Os02g18930	LOC_Os03g02710	13.0733207
LOC_Os02g17390	LOC_Os06g47600	9.22205831
LOC_Os02g17780	LOC_Os05g48290	8.87316604
LOC_Os02g18880	LOC_Os04g28870	8.80608555
LOC_Os02g17780	LOC_Os12g40550	11.5982531
LOC_Os02g18550	LOC_Os02g44780	9.28835068
LOC_Os02g18180	LOC_Os04g46560	8.93314786
LOC_Os02g18880	LOC_Os10g07229	9.87159497
LOC_Os02g18930	LOC_Os03g47770	8.98510222
LOC_Os02g17780	LOC_Os12g34450	8.85101269

InteractorA	InteractorB	Z score
LOC_Os02g18550	LOC_Os03g21260	8.80536839
LOC_Os02g17390	LOC_Os03g02260	8.90795259
LOC_Os02g17390	LOC_Os02g41470	9.63534237
LOC_Os02g18180	LOC_Os05g38760	8.80566137
LOC_Os02g18550	LOC_Os08g14570	9.15468269
LOC_Os02g18880	LOC_Os05g44340	9.81311045
LOC_Os02g18550	LOC_Os06g23870	9.46480283
LOC_Os02g17780	LOC_Os05g23740	8.95384828
LOC_Os02g17780	LOC_Os03g08530	8.94068118
LOC_Os02g18930	LOC_Os03g27280	9.08492036
LOC_Os02g18550	LOC_Os09g36800	9.06292957
LOC_Os02g18880	LOC_Os11g08440	9.20493743
LOC_Os02g18880	LOC_Os03g16740	8.81030308
LOC_Os02g17390	LOC_Os12g25700	9.29472355
LOC_Os02g18880	LOC_Os11g10480	8.97035725
LOC_Os02g18880	LOC_Os10g41410	9.02659613
LOC_Os02g16550	LOC_Os09g33500	8.90558703
LOC_Os02g18550	LOC_Os07g07770	8.86997809
LOC_Os02g18180	LOC_Os11g47760	8.83026707
LOC_Os02g17780	LOC_Os03g55090	9.2427007
LOC_Os02g17390	LOC_Os03g16220	8.92845733
LOC_Os02g17780	LOC_Os07g35940	9.59950945
LOC_Os02g17390	LOC_Os06g51060	9.28205124
LOC_Os02g18180	LOC_Os09g38030	9.20851796
LOC_Os02g17780	LOC_Os07g37320	8.96392652
LOC_Os02g18880	LOC_Os02g56720	9.84288569
LOC_Os02g16550	LOC_Os09g31502	8.91580461
LOC_Os02g18930	LOC_Os03g55150	9.24506025
LOC_Os02g18180	LOC_Os12g16240	9.34383196
LOC_Os02g18880	LOC_Os06g37080	9.16581671
LOC_Os02g18880	LOC_Os03g47770	9.12399946
LOC_Os02g18550	LOC_Os04g19740	9.55977576
LOC_Os02g18880	LOC_Os03g28400	9.13303202
LOC_Os02g18880	LOC_Os02g53180	8.96476913
LOC_Os02g18880	LOC_Os11g47760	9.04380404
LOC_Os02g18880	LOC_Os08g15040	8.84956211
LOC_Os02g18880	LOC_Os11g14040	9.22917396
LOC_Os02g18180	LOC_Os03g22010	10.7300922
LOC_Os02g18550	LOC_Os03g49260	9.29604201
LOC_Os02g18180	LOC_Os05g35400	9.8944858
LOC_Os02g18550	LOC_Os06g04620	10.1843505
LOC_Os02g18180	LOC_Os08g40140	13.0087724
LOC_Os02g18880	LOC_Os03g22530	9.29668412

InteractorA	InteractorB	Z score
LOC_Os02g18550	LOC_Os10g41689	8.83272715
LOC_Os02g17780	LOC_Os05g19380	9.282315
LOC_Os02g17780	LOC_Os03g18810	10.6154554
LOC_Os02g18550	LOC_Os03g57790	10.2275552
LOC_Os02g18880	LOC_Os12g43370	8.98148299
LOC_Os02g18930	LOC_Os02g44550	11.228066
LOC_Os02g18930	LOC_Os02g39850	8.8679806
LOC_Os02g18880	LOC_Os06g46372	9.45124175
LOC_Os02g18880	LOC_Os05g33730	8.90614928
LOC_Os02g18930	LOC_Os03g53200	8.92428594
LOC_Os02g18880	LOC_Os03g15360	9.00296192
LOC_Os02g18880	LOC_Os06g06040	11.9698159
LOC_Os02g17390	LOC_Os02g47790	8.86116488
LOC_Os02g17390	LOC_Os05g45420	9.19038696
LOC_Os02g18180	LOC_Os03g10120	10.3063202
LOC_Os02g17390	LOC_Os03g60740	9.13762012
LOC_Os02g18880	LOC_Os05g38310	10.2223497
LOC_Os02g18930	LOC_Os03g06940	8.95602745
LOC_Os02g17390	LOC_Os03g15360	8.80531372
LOC_Os02g17390	LOC_Os04g01250	10.0162619
LOC_Os02g17780	LOC_Os09g36930	9.00854635
LOC_Os02g17780	LOC_Os04g45490	9.1617671
LOC_Os02g17390	LOC_Os03g01630	9.06725713
LOC_Os02g18880	LOC_Os02g56690	11.3552401
LOC_Os02g17390	LOC_Os02g45540	9.27095512
LOC_Os02g18930	LOC_Os03g20710	9.12472508
LOC_Os02g18880	LOC_Os06g36160	8.8552084
LOC_Os02g18930	LOC_Os02g56720	10.3586785
LOC_Os02g17780	LOC_Os05g41210	12.4873265
LOC_Os02g18880	LOC_Os09g04680	10.1913099
LOC_Os02g18180	LOC_Os10g38340	9.04970499
LOC_Os02g17390	LOC_Os03g03910	9.25140648
LOC_Os02g17780	LOC_Os05g06300	9.03186546
LOC_Os02g18880	LOC_Os04g41310	8.89219618
LOC_Os02g18880	LOC_Os04g29030	8.90378463
LOC_Os02g18880	LOC_Os04g59040	9.25115553
LOC_Os02g17390	LOC_Os03g58400	8.92393104
LOC_Os02g17780	LOC_Os03g55389	8.95622205
LOC_Os02g18880	LOC_Os04g12970	9.15850084
LOC_Os02g17390	LOC_Os09g39780	8.85065468
LOC_Os02g17780	LOC_Os12g12514	9.00219359
LOC_Os02g18880	LOC_Os10g25674	11.3579393
LOC_Os02g18550	LOC_Os06g15420	8.83247812

InteractorA	InteractorB	Z score
LOC_Os02g17390	LOC_Os06g23780	9.19580134
LOC_Os02g17780	LOC_Os03g43890	8.93793759
LOC_Os02g17780	LOC_Os07g32800	10.6089543
LOC_Os02g18880	LOC_Os02g56180	10.0515384
LOC_Os02g17780	LOC_Os08g37800	8.93789454
LOC_Os02g17390	LOC_Os05g04520	9.66874351
LOC_Os02g18550	LOC_Os04g43760	9.81916359
LOC_Os02g18550	LOC_Os02g29020	10.0691331
LOC_Os02g16550	LOC_Os09g36830	8.97124803
LOC_Os02g18180	LOC_Os09g35800	8.9263685
LOC_Os02g18880	LOC_Os07g17010	9.81740879
LOC_Os02g17780	LOC_Os12g37360	9.04207624
LOC_Os02g16550	LOC_Os10g07040	8.81200661
LOC_Os02g17390	LOC_Os03g46070	9.03688237
LOC_Os02g17780	LOC_Os09g10270	8.80381216
LOC_Os02g18880	LOC_Os06g46940	9.71100461
LOC_Os02g18930	LOC_Os03g63410	10.3872245
LOC_Os02g17390	LOC_Os03g48471	8.99350218
LOC_Os02g17390	LOC_Os08g42910	9.29496475
LOC_Os02g18930	LOC_Os03g38020	8.87437097
LOC_Os02g17390	LOC_Os12g36950	8.88747125
LOC_Os02g18180	LOC_Os10g38730	10.7453323
LOC_Os02g18550	LOC_Os04g53810	9.06758561
LOC_Os02g18880	LOC_Os06g23870	13.1118786
LOC_Os02g17780	LOC_Os08g44350	10.3076901
LOC_Os02g17390	LOC_Os04g30420	9.34606961
LOC_Os02g18880	LOC_Os11g31620	9.25277354
LOC_Os02g18880	LOC_Os11g10520	9.20090485
LOC_Os02g18550	LOC_Os04g39840	9.31154826
LOC_Os02g17390	LOC_Os07g41750	9.56602732
LOC_Os02g18880	LOC_Os08g34790	9.23718966
LOC_Os02g18930	LOC_Os04g20070	8.97872784
LOC_Os02g18880	LOC_Os11g37640	10.1600367
LOC_Os02g18550	LOC_Os03g40720	8.82672084
LOC_Os02g17390	LOC_Os02g47590	8.85634009
LOC_Os02g17390	LOC_Os03g03130	9.09824224
LOC_Os02g18880	LOC_Os09g39400	9.17817686
LOC_Os02g18550	LOC_Os06g25010	9.01032928
LOC_Os02g19880	LOC_Os06g49970	9.26981797
LOC_Os02g18930	LOC_Os07g22930	9.04938288
LOC_Os02g19770	LOC_Os06g01850	10.4751179
LOC_Os02g21460	LOC_Os09g04050	9.43504828
LOC_Os02g19770	LOC_Os05g11730	9.39798369

InteractorA	InteractorB	Z score
LOC_Os02g18930	LOC_Os08g14760	9.02616315
LOC_Os02g21460	LOC_Os09g26340	10.1402953
LOC_Os02g21460	LOC_Os10g38360	8.80788185
LOC_Os02g19770	LOC_Os05g05800	9.54955295
LOC_Os02g18930	LOC_Os09g25390	9.69579791
LOC_Os02g19770	LOC_Os10g23900	8.80519701
LOC_Os02g21460	LOC_Os07g49220	10.1574506
LOC_Os02g19420	LOC_Os07g10660	9.41602796
LOC_Os02g18930	LOC_Os07g38430	9.93500501
LOC_Os02g19420	LOC_Os11g19320	9.46159082
LOC_Os02g18930	LOC_Os12g02080	10.2652751
LOC_Os02g18930	LOC_Os11g32650	11.4703114
LOC_Os02g19770	LOC_Os05g28180	9.59662758
LOC_Os02g19770	LOC_Os10g11140	8.89729224
LOC_Os02g18930	LOC_Os06g23870	10.7455456
LOC_Os02g18930	LOC_Os06g39230	10.0673956
LOC_Os02g21460	LOC_Os06g46000	9.07435366
LOC_Os02g21460	LOC_Os05g38740	10.1402924
LOC_Os02g19770	LOC_Os09g15400	9.16323571
LOC_Os02g21460	LOC_Os03g05980	10.3568126
LOC_Os02g19770	LOC_Os06g35590	9.48458384
LOC_Os02g18930	LOC_Os12g38180	10.4838322
LOC_Os02g19770	LOC_Os11g25100	9.61816025
LOC_Os02g21460	LOC_Os07g25150	10.6217637
LOC_Os02g18930	LOC_Os04g33480	8.96603598
LOC_Os02g20850	LOC_Os09g31486	9.84244262
LOC_Os02g20850	LOC_Os02g21460	8.99096481
LOC_Os02g21460	LOC_Os02g45940	10.1402953
LOC_Os02g21460	LOC_Os07g36500	10.1402924
LOC_Os02g21460	LOC_Os06g36670	8.86338965
LOC_Os02g20850	LOC_Os07g07320	8.8752302
LOC_Os02g18930	LOC_Os04g39880	9.22872544
LOC_Os02g20850	LOC_Os03g04110	10.3766503
LOC_Os02g19420	LOC_Os03g58530	9.10535469
LOC_Os02g18930	LOC_Os10g07229	8.80933538
LOC_Os02g18930	LOC_Os06g15420	10.3375498
LOC_Os02g20850	LOC_Os08g44350	9.50837159
LOC_Os02g18930	LOC_Os11g37640	9.0699252
LOC_Os02g18930	LOC_Os04g32010	9.53934176
LOC_Os02g18930	LOC_Os04g37820	9.6465507
LOC_Os02g19770	LOC_Os04g45490	8.91128638
LOC_Os02g18930	LOC_Os10g28360	8.8022381
LOC_Os02g21460	LOC_Os05g31040	9.7007925

InteractorA	InteractorB	Z score
LOC_Os02g21460	LOC_Os04g10010	9.60075024
LOC_Os02g20850	LOC_Os04g39864	9.10853213
LOC_Os02g19770	LOC_Os06g09450	9.16641978
LOC_Os02g18930	LOC_Os04g44730	9.4039604
LOC_Os02g21460	LOC_Os06g51084	9.64560981
LOC_Os02g19420	LOC_Os08g09200	9.07327917
LOC_Os02g21460	LOC_Os11g29400	9.77167563
LOC_Os02g19770	LOC_Os03g61340	9.40079285
LOC_Os02g22130	LOC_Os02g52430	9.25343651
LOC_Os02g19880	LOC_Os07g43390	8.9785568
LOC_Os02g19770	LOC_Os09g29200	8.87324647
LOC_Os02g19420	LOC_Os04g12980	9.05142565
LOC_Os02g19420	LOC_Os06g45120	9.89330474
LOC_Os02g21460	LOC_Os04g49420	10.1402953
LOC_Os02g21460	LOC_Os04g27860	9.26341461
LOC_Os02g18930	LOC_Os05g04470	9.01431729
LOC_Os02g21460	LOC_Os05g39050	10.1402953
LOC_Os02g18930	LOC_Os06g10970	8.84204836
LOC_Os02g20850	LOC_Os12g43440	10.0346386
LOC_Os02g21460	LOC_Os11g38959	10.3568126
LOC_Os02g19770	LOC_Os07g43390	9.23620081
LOC_Os02g20850	LOC_Os10g38470	8.95939463
LOC_Os02g18930	LOC_Os11g14040	9.9481477
LOC_Os02g20850	LOC_Os03g59740	9.14758049
LOC_Os02g19420	LOC_Os06g41810	8.85451622
LOC_Os02g18930	LOC_Os08g08500	9.36391465
LOC_Os02g18930	LOC_Os05g04500	9.34438318
LOC_Os02g19880	LOC_Os12g13810	8.99337477
LOC_Os02g21460	LOC_Os07g47990	10.8565899
LOC_Os02g21460	LOC_Os03g25370	9.03323161
LOC_Os02g18930	LOC_Os08g28190	9.27895249
LOC_Os02g21460	LOC_Os03g02780	10.1402983
LOC_Os02g20850	LOC_Os03g04169	10.1789541
LOC_Os02g18930	LOC_Os10g31940	9.28527297
LOC_Os02g21460	LOC_Os08g44340	10.059234
LOC_Os02g20850	LOC_Os05g26890	9.07549548
LOC_Os02g18930	LOC_Os08g39870	8.83571116
LOC_Os02g19770	LOC_Os12g24650	10.5895636
LOC_Os02g18930	LOC_Os10g32550	8.94706317
LOC_Os02g19770	LOC_Os11g08460	9.34786326
LOC_Os02g19420	LOC_Os02g55140	8.93720051
LOC_Os02g19880	LOC_Os07g05180	9.08428911
LOC_Os02g19770	LOC_Os03g18570	9.01072785

InteractorA	InteractorB	Z score
LOC_Os02g18930	LOC_Os08g09250	11.7862934
LOC_Os02g20850	LOC_Os04g08350	9.34463909
LOC_Os02g20850	LOC_Os11g08440	9.0056805
LOC_Os02g21460	LOC_Os10g39410	10.1402924
LOC_Os02g19770	LOC_Os06g12180	8.92602057
LOC_Os02g19880	LOC_Os08g36910	8.98167266
LOC_Os02g18930	LOC_Os09g08880	10.0962218
LOC_Os02g21460	LOC_Os03g44484	9.83342435
LOC_Os02g21460	LOC_Os09g39570	8.9648716
LOC_Os02g19770	LOC_Os03g53790	8.97294785
LOC_Os02g19770	LOC_Os07g38890	8.88956573
LOC_Os02g20850	LOC_Os12g42884	9.79227517
LOC_Os02g21460	LOC_Os06g46372	9.45288914
LOC_Os02g21460	LOC_Os06g24390	8.93452945
LOC_Os02g19770	LOC_Os06g15420	9.00964984
LOC_Os02g21460	LOC_Os09g38020	10.1402953
LOC_Os02g20850	LOC_Os08g14760	9.35914116
LOC_Os02g18930	LOC_Os10g41490	8.85320087
LOC_Os02g19770	LOC_Os11g26910	9.05235021
LOC_Os02g18930	LOC_Os08g42410	8.89826427
LOC_Os02g19770	LOC_Os07g30970	9.5905605
LOC_Os02g18930	LOC_Os06g06560	9.76169344
LOC_Os02g20850	LOC_Os06g06880	8.99809161
LOC_Os02g20850	LOC_Os10g39170	9.16791376
LOC_Os02g20850	LOC_Os02g49720	10.3745006
LOC_Os02g19770	LOC_Os03g45320	9.454214
LOC_Os02g18930	LOC_Os06g46372	10.8346247
LOC_Os02g18930	LOC_Os04g30420	8.81565191
LOC_Os01g09430	LOC_Os01g19450	10.8687664
LOC_Os01g09460	LOC_Os04g01740	8.87855578
LOC_Os01g09460	LOC_Os09g15320	10.751032
LOC_Os01g09430	LOC_Os03g55800	10.0134542
LOC_Os01g09580	LOC_Os03g20700	9.16359482
LOC_Os01g09460	LOC_Os12g10720	8.89236345
LOC_Os01g09430	LOC_Os01g62040	9.18514265
LOC_Os01g09460	LOC_Os05g49800	9.31839753
LOC_Os01g09460	LOC_Os04g33040	9.02210626
LOC_Os01g09510	LOC_Os03g52840	9.04854229
LOC_Os01g09430	LOC_Os02g42520	10.3733245
LOC_Os01g09460	LOC_Os05g28940	10.1195439
LOC_Os01g09430	LOC_Os06g10910	8.97587361
LOC_Os01g09580	LOC_Os07g35940	10.6024394
LOC_Os01g09510	LOC_Os08g44530	8.87583487

InteractorA	InteractorB	Z score
LOC_Os01g09460	LOC_Os07g39270	9.82269412
LOC_Os01g09580	LOC_Os01g34480	10.2024013
LOC_Os01g09430	LOC_Os04g56950	9.05486306
LOC_Os01g09430	LOC_Os12g22650	8.89732078
LOC_Os01g09510	LOC_Os04g40990	8.83378956
LOC_Os01g09320	LOC_Os09g08072	9.24017531
LOC_Os01g09460	LOC_Os07g17010	10.7407631
LOC_Os01g09320	LOC_Os04g28180	10.0776917
LOC_Os01g09510	LOC_Os11g08330	9.05585007
LOC_Os01g09580	LOC_Os06g36700	9.74096326
LOC_Os01g09320	LOC_Os05g50890	9.19805083
LOC_Os01g09430	LOC_Os02g50880	9.32706277
LOC_Os01g09460	LOC_Os08g03290	9.02064408
LOC_Os01g09580	LOC_Os01g51220	10.0444302
LOC_Os01g09430	LOC_Os11g25100	11.8458856
LOC_Os01g09460	LOC_Os09g33500	11.8045895
LOC_Os01g09460	LOC_Os10g09860	10.2325532
LOC_Os01g09460	LOC_Os06g10970	9.15141463
LOC_Os01g09320	LOC_Os03g62670	8.83670936
LOC_Os01g09580	LOC_Os04g16680	9.03467858
LOC_Os01g09430	LOC_Os04g33570	9.02031947
LOC_Os01g09580	LOC_Os05g22724	13.0562095
LOC_Os01g09580	LOC_Os07g38890	10.0269879
LOC_Os01g09580	LOC_Os03g08560	9.39498978
LOC_Os01g09430	LOC_Os03g52460	10.0448685
LOC_Os01g09460	LOC_Os01g71400	9.57124238
LOC_Os01g09320	LOC_Os04g45290	10.0584478
LOC_Os01g09430	LOC_Os01g57610	9.94349316
LOC_Os01g09430	LOC_Os07g43820	10.5195902
LOC_Os01g09510	LOC_Os10g36650	9.30065929
LOC_Os01g09580	LOC_Os01g11110	9.01456086
LOC_Os01g09580	LOC_Os09g19954	10.2812745
LOC_Os01g09320	LOC_Os05g09440	10.4498346
LOC_Os01g09580	LOC_Os01g23580	9.82344137
LOC_Os01g09510	LOC_Os07g42950	9.23132497
LOC_Os01g09320	LOC_Os10g26010	10.7592887
LOC_Os01g09430	LOC_Os05g48030	9.37032845
LOC_Os01g09430	LOC_Os12g41220	9.65357703
LOC_Os01g09460	LOC_Os02g14440	9.72463453
LOC_Os01g09510	LOC_Os01g15120	8.9943098
LOC_Os01g09460	LOC_Os11g19220	9.33050073
LOC_Os01g09460	LOC_Os02g27760	10.1508772
LOC_Os01g09510	LOC_Os08g33100	9.20724897

InteractorA	InteractorB	Z score
LOC_Os01g09510	LOC_Os11g41130	9.00826689
LOC_Os01g09460	LOC_Os08g40140	10.1991292
LOC_Os01g09460	LOC_Os06g12790	9.90346467
LOC_Os01g09460	LOC_Os10g21240	10.277726
LOC_Os01g09580	LOC_Os01g73580	9.02976531
LOC_Os01g09510	LOC_Os01g18170	9.4509866
LOC_Os01g09460	LOC_Os07g08880	10.2697261
LOC_Os01g09460	LOC_Os03g01900	10.5520271
LOC_Os01g09460	LOC_Os01g60790	9.23744099
LOC_Os01g09460	LOC_Os10g25140	11.8776485
LOC_Os01g09460	LOC_Os06g45120	9.16004801
LOC_Os01g09580	LOC_Os01g71350	9.82231131
LOC_Os01g09430	LOC_Os05g37690	9.62073374
LOC_Os01g09580	LOC_Os10g38489	11.2652201
LOC_Os01g09430	LOC_Os06g35700	9.18788659
LOC_Os01g09320	LOC_Os10g40710	9.40966082
LOC_Os01g09580	LOC_Os01g46240	10.050991
LOC_Os01g09580	LOC_Os08g10608	10.7472618
LOC_Os01g09580	LOC_Os02g18930	8.9285953
LOC_Os01g09320	LOC_Os03g56460	8.88269799
LOC_Os01g09320	LOC_Os08g02400	9.03843288
LOC_Os01g09460	LOC_Os10g38730	9.17060451
LOC_Os01g09580	LOC_Os05g05680	13.7520964
LOC_Os01g09430	LOC_Os02g47610	9.3422181
LOC_Os01g09580	LOC_Os01g10840	9.06691835
LOC_Os01g09430	LOC_Os09g38620	9.35609393
LOC_Os01g09320	LOC_Os03g64330	9.18738871
LOC_Os01g09460	LOC_Os11g43960	9.69805268
LOC_Os01g09580	LOC_Os12g25690	8.83655343
LOC_Os01g09580	LOC_Os07g23850	10.0741906
LOC_Os01g09580	LOC_Os01g64850	9.46095348
LOC_Os01g09460	LOC_Os03g57200	9.03553891
LOC_Os01g09460	LOC_Os03g53200	8.85624298
LOC_Os01g09460	LOC_Os08g39300	9.09504209
LOC_Os01g09460	LOC_Os07g27790	9.17553963
LOC_Os01g09460	LOC_Os05g09500	9.24813166
LOC_Os01g09320	LOC_Os12g16290	8.93675552
LOC_Os01g09320	LOC_Os04g16760	9.91324123
LOC_Os01g09320	LOC_Os08g02410	9.67063275
LOC_Os01g09460	LOC_Os02g10390	11.4920444
LOC_Os01g09460	LOC_Os04g02820	11.1091917
LOC_Os01g09430	LOC_Os01g52500	9.93703403
LOC_Os01g09580	LOC_Os10g38540	9.51728954

InteractorA	InteractorB	Z score
LOC_Os01g09580	LOC_Os08g42000	9.16231071
LOC_Os01g09460	LOC_Os06g35700	8.8788097
LOC_Os01g09580	LOC_Os06g35540	9.13449181
LOC_Os01g09430	LOC_Os07g26640	9.04956192
LOC_Os01g09460	LOC_Os09g27820	8.85759737
LOC_Os01g09460	LOC_Os03g58260	9.23561939
LOC_Os01g09430	LOC_Os01g55830	9.88667629
LOC_Os01g09460	LOC_Os02g51100	10.3559881
LOC_Os01g09580	LOC_Os04g33240	9.50649458
LOC_Os01g09460	LOC_Os05g35320	9.0798234
LOC_Os01g09320	LOC_Os05g01675	9.91316762
LOC_Os01g09320	LOC_Os04g36700	9.07691637
LOC_Os01g09460	LOC_Os03g51040	9.03318467
LOC_Os01g09510	LOC_Os06g46340	10.5493919
LOC_Os01g09460	LOC_Os04g27860	9.23462759
LOC_Os01g09430	LOC_Os06g50300	9.88085625
LOC_Os01g09430	LOC_Os04g56920	9.03476951
LOC_Os01g09580	LOC_Os10g38780	9.65641684
LOC_Os01g09510	LOC_Os03g18740	9.20653792
LOC_Os01g09430	LOC_Os03g59310	9.30397351
LOC_Os01g09510	LOC_Os03g16110	9.4192233
LOC_Os01g09510	LOC_Os04g40874	8.94713297
LOC_Os01g09430	LOC_Os08g17680	9.3482657
LOC_Os01g09460	LOC_Os03g51740	10.1059049
LOC_Os01g09460	LOC_Os04g54330	9.28844285
LOC_Os01g09460	LOC_Os12g04980	10.5190948
LOC_Os01g09460	LOC_Os01g73580	8.9000798
LOC_Os01g09320	LOC_Os09g04680	10.2594764
LOC_Os02g24632	LOC_Os04g58570	9.24169793
LOC_Os02g22130	LOC_Os12g41110	9.3701535
LOC_Os02g24354	LOC_Os04g09604	10.0751613
LOC_Os02g24354	LOC_Os05g49890	9.152996
LOC_Os02g24634	LOC_Os04g56070	9.12113696
LOC_Os02g24354	LOC_Os03g31210	8.9258898
LOC_Os02g24354	LOC_Os12g02370	8.80276672
LOC_Os02g22130	LOC_Os12g01922	10.1487486
LOC_Os02g24632	LOC_Os05g41210	9.15465934
LOC_Os02g24354	LOC_Os11g47760	9.15679275
LOC_Os02g22780	LOC_Os08g09210	10.3353902
LOC_Os02g24632	LOC_Os12g16410	9.15080391
LOC_Os02g24634	LOC_Os07g38540	9.455522
LOC_Os02g24632	LOC_Os10g38600	9.27458629
LOC_Os02g24632	LOC_Os10g38710	9.105116

InteractorA	InteractorB	Z score
LOC_Os02g24632	LOC_Os08g38300	10.5780225
LOC_Os02g24632	LOC_Os07g04240	9.22578559
LOC_Os02g22130	LOC_Os08g08500	9.08090183
LOC_Os02g24354	LOC_Os10g38274	9.68694866
LOC_Os02g24354	LOC_Os10g21230	9.68694866
LOC_Os02g24634	LOC_Os05g11710	9.44045108
LOC_Os02g22130	LOC_Os03g12270	9.48556717
LOC_Os02g24634	LOC_Os08g40140	9.79572989
LOC_Os02g22780	LOC_Os05g51480	8.93200922
LOC_Os02g24354	LOC_Os05g41900	9.39927936
LOC_Os02g22780	LOC_Os04g12900	9.98181763
LOC_Os02g22130	LOC_Os08g39870	9.886923
LOC_Os02g24354	LOC_Os03g57290	9.26061093
LOC_Os02g22780	LOC_Os10g38540	8.88827026
LOC_Os02g22780	LOC_Os10g35070	8.82795927
LOC_Os02g24354	LOC_Os07g41750	12.6648002
LOC_Os02g22140	LOC_Os05g30480	8.97560593
LOC_Os02g24632	LOC_Os04g43800	9.29871326
LOC_Os02g22780	LOC_Os03g58300	8.92627474
LOC_Os02g22780	LOC_Os05g41900	8.84311732
LOC_Os02g22780	LOC_Os06g06050	9.72547066
LOC_Os02g22780	LOC_Os04g40130	10.5238159
LOC_Os02g22130	LOC_Os04g41620	9.19096213
LOC_Os02g22130	LOC_Os07g38730	9.03714776
LOC_Os02g22130	LOC_Os10g23900	10.246675
LOC_Os02g24634	LOC_Os11g05470	9.8805061
LOC_Os02g22780	LOC_Os11g07440	9.79592374
LOC_Os02g22780	LOC_Os02g39850	9.76799651
LOC_Os02g22780	LOC_Os07g08660	9.5684273
LOC_Os02g22130	LOC_Os03g17690	8.85920514
LOC_Os02g22130	LOC_Os06g35590	9.58102879
LOC_Os02g22140	LOC_Os04g48850	9.37597281
LOC_Os02g24634	LOC_Os08g33370	8.97643334
LOC_Os02g24632	LOC_Os12g12560	8.91685263
LOC_Os02g22130	LOC_Os05g19380	9.02193773
LOC_Os02g22130	LOC_Os03g09910	11.0441784
LOC_Os02g22780	LOC_Os06g39740	11.0425039
LOC_Os02g22140	LOC_Os05g23860	9.12824696
LOC_Os02g24632	LOC_Os03g56840	9.8248869
LOC_Os02g22780	LOC_Os05g49800	9.84152547
LOC_Os02g22780	LOC_Os04g58200	9.24591022
LOC_Os02g22140	LOC_Os08g23730	9.4078157
LOC_Os02g22130	LOC_Os05g05800	10.0800977

InteractorA	InteractorB	Z score
LOC_Os02g24632	LOC_Os03g48471	9.21064103
LOC_Os02g24354	LOC_Os12g25630	8.94557249
LOC_Os02g24634	LOC_Os03g04410	9.32117577
LOC_Os02g24632	LOC_Os02g39850	10.5097999
LOC_Os02g24354	LOC_Os04g40290	9.17136547
LOC_Os02g24354	LOC_Os06g51150	8.97542446
LOC_Os02g22140	LOC_Os07g46310	9.79975807
LOC_Os02g22780	LOC_Os06g12090	8.86175219
LOC_Os02g22780	LOC_Os03g08530	9.93432494
LOC_Os02g24354	LOC_Os07g07320	8.99328697
LOC_Os02g22780	LOC_Os08g03040	8.8219862
LOC_Os02g22780	LOC_Os08g44960	9.40595196
LOC_Os02g22780	LOC_Os04g45490	9.81711884
LOC_Os02g22780	LOC_Os03g58530	9.67478466
LOC_Os02g22780	LOC_Os07g36140	10.155124
LOC_Os02g24634	LOC_Os08g34280	9.29588316
LOC_Os02g22130	LOC_Os09g32952	9.07548332
LOC_Os02g22780	LOC_Os11g19320	9.21987737
LOC_Os02g22780	LOC_Os07g40290	8.88627493
LOC_Os02g22780	LOC_Os02g52800	8.83962254
LOC_Os02g24632	LOC_Os04g02820	9.02304761
LOC_Os02g24354	LOC_Os04g55040	9.28308285
LOC_Os02g22130	LOC_Os12g25120	9.4225639
LOC_Os02g22780	LOC_Os10g28320	9.77673759
LOC_Os02g22130	LOC_Os03g18130	9.07059327
LOC_Os02g24634	LOC_Os08g42560	9.73233501
LOC_Os02g24634	LOC_Os06g35730	9.44045108
LOC_Os02g22780	LOC_Os09g20220	9.04806277
LOC_Os02g24634	LOC_Os05g23740	9.11082529
LOC_Os02g24634	LOC_Os04g53214	8.84458913
LOC_Os02g22780	LOC_Os06g40170	9.65526796
LOC_Os02g24632	LOC_Os11g20790	9.18667611
LOC_Os02g24354	LOC_Os05g07720	10.9148878
LOC_Os02g22780	LOC_Os05g26890	11.4525086
LOC_Os02g24634	LOC_Os03g21120	10.4975335
LOC_Os02g22780	LOC_Os03g46070	10.3356978
LOC_Os02g24354	LOC_Os03g27370	9.1090536
LOC_Os02g24354	LOC_Os03g50250	9.09713778
LOC_Os02g22780	LOC_Os04g39864	11.2252359
LOC_Os02g24634	LOC_Os08g31870	8.80917318
LOC_Os02g24632	LOC_Os07g08500	8.94457347
LOC_Os02g24354	LOC_Os08g05910	8.98899694
LOC_Os02g24632	LOC_Os12g41220	9.53795854

InteractorA	InteractorB	Z score
LOC_Os02g22780	LOC_Os07g05940	9.22055089
LOC_Os02g27940	LOC_Os04g43750	9.50139655
LOC_Os02g28810	LOC_Os02g36710	8.92263868
LOC_Os02g25940	LOC_Os10g38700	9.35348171
LOC_Os02g25940	LOC_Os06g36770	9.41513161
LOC_Os02g25940	LOC_Os09g35800	9.10342732
LOC_Os02g25940	LOC_Os07g07550	9.05801222
LOC_Os02g27940	LOC_Os04g14680	9.12120185
LOC_Os02g28810	LOC_Os12g01922	8.97618253
LOC_Os02g27760	LOC_Os10g28050	9.39832235
LOC_Os02g29020	LOC_Os03g46070	9.41106183
LOC_Os02g28810	LOC_Os10g21212	9.65635314
LOC_Os02g27940	LOC_Os09g07460	8.8964176
LOC_Os02g27940	LOC_Os07g07770	8.91495599
LOC_Os02g27769	LOC_Os03g51550	10.3015154
LOC_Os02g25940	LOC_Os04g53850	9.211137
LOC_Os02g27940	LOC_Os03g03720	11.1269322
LOC_Os02g27760	LOC_Os08g32620	10.3240238
LOC_Os02g27940	LOC_Os03g42840	8.95851644
LOC_Os02g28810	LOC_Os11g02600	9.45553612
LOC_Os02g25940	LOC_Os08g09200	11.2075096
LOC_Os02g25940	LOC_Os04g12710	9.02070334
LOC_Os02g25940	LOC_Os06g06460	9.1371411
LOC_Os02g27940	LOC_Os09g31490	9.03863742
LOC_Os02g25940	LOC_Os07g07060	9.42010422
LOC_Os02g25940	LOC_Os05g25850	10.484506
LOC_Os02g27760	LOC_Os10g30200	8.85457257
LOC_Os02g27760	LOC_Os04g56920	9.46832801
LOC_Os02g24634	LOC_Os11g32260	8.990722
LOC_Os02g27769	LOC_Os04g39864	11.2335992
LOC_Os02g25940	LOC_Os02g53420	8.84518563
LOC_Os02g27769	LOC_Os06g06090	9.5730655
LOC_Os02g27940	LOC_Os09g31502	8.83047803
LOC_Os02g25940	LOC_Os05g38760	8.92935901
LOC_Os02g27760	LOC_Os09g20820	9.31436571
LOC_Os02g25940	LOC_Os11g05730	9.13714022
LOC_Os02g28810	LOC_Os12g04980	9.41291863
LOC_Os02g25940	LOC_Os02g46970	9.39455978
LOC_Os02g27940	LOC_Os04g22660	8.83142493
LOC_Os02g27769	LOC_Os10g42720	8.94474018
LOC_Os02g27940	LOC_Os06g37080	10.3638565
LOC_Os02g25940	LOC_Os05g42350	8.96308275
LOC_Os02g27760	LOC_Os03g26450	8.83570946

InteractorA	InteractorB	Z score
LOC_Os02g27940	LOC_Os03g40720	9.41414905
LOC_Os02g25940	LOC_Os05g19150	9.06204002
LOC_Os02g27760	LOC_Os10g23100	9.31648648
LOC_Os02g25940	LOC_Os09g31430	8.99544423
LOC_Os02g24634	LOC_Os12g12560	9.53025061
LOC_Os02g25940	LOC_Os03g18510	10.114284
LOC_Os02g27940	LOC_Os06g36770	9.86679586
LOC_Os02g27940	LOC_Os03g38020	8.94169641
LOC_Os02g27940	LOC_Os04g28990	9.30506506
LOC_Os02g27940	LOC_Os02g47610	10.0072884
LOC_Os02g27760	LOC_Os04g33040	9.16725937
LOC_Os02g25940	LOC_Os05g39580	9.03015339
LOC_Os02g25940	LOC_Os06g09910	9.70464284
LOC_Os02g25940	LOC_Os05g41080	10.6820633
LOC_Os02g28810	LOC_Os10g07616	9.04109867
LOC_Os02g28810	LOC_Os09g14670	8.84322237
LOC_Os02g25940	LOC_Os03g01650	10.5298371
LOC_Os02g29020	LOC_Os05g07880	9.31385863
LOC_Os02g28810	LOC_Os05g28280	8.88741961
LOC_Os02g29020	LOC_Os06g30970	9.03494924
LOC_Os02g25940	LOC_Os06g45670	9.5576023
LOC_Os02g25940	LOC_Os08g34190	8.8290153
LOC_Os02g27769	LOC_Os02g52610	9.75408218
LOC_Os02g29020	LOC_Os05g19670	15.1501435
LOC_Os02g25940	LOC_Os05g39690	9.23911859
LOC_Os02g25940	LOC_Os03g01610	10.5298449
LOC_Os02g27769	LOC_Os03g12660	8.84204352
LOC_Os02g25940	LOC_Os03g57140	9.55637072
LOC_Os02g29020	LOC_Os03g48780	9.50593513
LOC_Os02g25940	LOC_Os08g44370	9.34691695
LOC_Os02g25940	LOC_Os12g22650	9.15203571
LOC_Os02g28810	LOC_Os12g07980	8.84386021
LOC_Os02g27760	LOC_Os04g55290	8.82394458
LOC_Os02g25940	LOC_Os05g36280	9.13715037
LOC_Os02g25940	LOC_Os05g01970	9.47799341
LOC_Os02g27940	LOC_Os05g28180	9.35752715
LOC_Os02g28810	LOC_Os06g11290	9.28703883
LOC_Os02g25940	LOC_Os11g37960	9.00400582
LOC_Os02g25940	LOC_Os04g37640	9.26573177
LOC_Os02g25940	LOC_Os06g06510	9.13714022
LOC_Os02g27760	LOC_Os07g08880	8.90120581
LOC_Os02g27760	LOC_Os02g56130	8.93157655
LOC_Os02g27760	LOC_Os11g19320	9.42807286

InteractorA	InteractorB	Z score
LOC_Os02g27760	LOC_Os03g01800	9.47828607
LOC_Os02g25940	LOC_Os03g64050	8.93372039
LOC_Os02g28810	LOC_Os04g37820	9.8020441
LOC_Os02g29020	LOC_Os03g48760	14.4674204
LOC_Os02g25940	LOC_Os10g41510	9.25809368
LOC_Os02g28810	LOC_Os02g35310	9.60219297
LOC_Os02g25940	LOC_Os07g40290	10.3505761
LOC_Os02g24634	LOC_Os11g34450	9.30206511
LOC_Os02g28810	LOC_Os02g32030	9.41148571
LOC_Os02g27940	LOC_Os06g29844	9.42753753
LOC_Os02g27940	LOC_Os04g56070	9.07074385
LOC_Os02g27760	LOC_Os04g49130	9.09116803
LOC_Os02g27940	LOC_Os03g07300	9.27169609
LOC_Os02g27760	LOC_Os05g30480	9.71594005
LOC_Os02g32030	LOC_Os07g49120	10.5451765
LOC_Os02g32490	LOC_Os04g41620	9.32893929
LOC_Os02g32370	LOC_Os02g55300	9.77253276
LOC_Os02g30200	LOC_Os04g56320	9.92142885
LOC_Os02g32030	LOC_Os08g44270	9.12089862
LOC_Os02g30630	LOC_Os07g46830	9.14984315
LOC_Os02g32370	LOC_Os04g44920	8.95250939
LOC_Os02g32370	LOC_Os07g34190	9.78223087
LOC_Os02g30200	LOC_Os08g09770	9.11717136
LOC_Os02g32490	LOC_Os02g52560	9.6498285
LOC_Os02g32030	LOC_Os07g26540	11.4853894
LOC_Os02g30200	LOC_Os06g45120	9.21012097
LOC_Os02g32660	LOC_Os06g37660	9.12863037
LOC_Os02g30200	LOC_Os04g32010	9.26907075
LOC_Os02g32490	LOC_Os06g40190	9.27822184
LOC_Os02g32370	LOC_Os12g08280	10.1815517
LOC_Os02g32490	LOC_Os03g55090	9.19184209
LOC_Os02g32370	LOC_Os10g26110	8.83379861
LOC_Os02g32490	LOC_Os05g41900	9.38557301
LOC_Os02g30200	LOC_Os07g08170	9.10378556
LOC_Os02g30630	LOC_Os11g03230	8.85496931
LOC_Os02g32030	LOC_Os02g39064	9.67110799
LOC_Os02g30200	LOC_Os06g36770	9.6292144
LOC_Os02g32030	LOC_Os03g05590	9.03063976
LOC_Os02g32370	LOC_Os04g33480	9.92438897
LOC_Os02g32490	LOC_Os03g01900	9.01024364
LOC_Os02g30200	LOC_Os06g04030	9.68889904
LOC_Os02g32370	LOC_Os12g43370	8.83419947
LOC_Os02g30200	LOC_Os02g50860	8.90098101

InteractorA	InteractorB	Z score
LOC_Os02g32030	LOC_Os07g07709	10.2365047
LOC_Os02g30630	LOC_Os12g16250	9.20417492
LOC_Os02g32660	LOC_Os06g19960	8.99247152
LOC_Os02g32030	LOC_Os12g08270	8.82119427
LOC_Os02g30200	LOC_Os02g39850	10.0560465
LOC_Os02g32030	LOC_Os04g58680	8.89220721
LOC_Os02g32370	LOC_Os10g35110	8.99947024
LOC_Os02g30630	LOC_Os05g07880	8.83202413
LOC_Os02g30200	LOC_Os11g41130	9.33327358
LOC_Os02g32030	LOC_Os03g08530	9.12969903
LOC_Os02g32370	LOC_Os09g32830	9.33604744
LOC_Os02g32490	LOC_Os12g43370	8.99509225
LOC_Os02g32030	LOC_Os09g31506	9.15918084
LOC_Os02g30630	LOC_Os12g36950	9.66975291
LOC_Os02g32490	LOC_Os05g28180	8.91836194
LOC_Os02g30630	LOC_Os02g51930	9.10499198
LOC_Os02g32370	LOC_Os06g42130	9.81315688
LOC_Os02g32370	LOC_Os03g12520	9.05944254
LOC_Os02g32030	LOC_Os09g33860	10.5999062
LOC_Os02g30200	LOC_Os03g59060	10.6450663
LOC_Os02g32370	LOC_Os03g03910	9.00958063
LOC_Os02g30200	LOC_Os12g43100	8.87731195
LOC_Os02g32490	LOC_Os04g41960	8.81212327
LOC_Os02g29020	LOC_Os09g07830	9.83699445
LOC_Os02g32370	LOC_Os06g11240	9.74684414
LOC_Os02g32660	LOC_Os04g56210	8.81633048
LOC_Os02g32030	LOC_Os12g41110	8.86089783
LOC_Os02g30200	LOC_Os10g21268	10.2178561
LOC_Os02g32030	LOC_Os11g32770	9.33004461
LOC_Os02g30630	LOC_Os03g49350	10.0420346
LOC_Os02g30630	LOC_Os09g23540	9.1707517
LOC_Os02g30200	LOC_Os07g29750	9.2140127
LOC_Os02g32490	LOC_Os04g59450	8.92433569
LOC_Os02g32370	LOC_Os04g33040	9.7349018
LOC_Os02g32660	LOC_Os03g06330	9.0297278
LOC_Os02g32030	LOC_Os10g07229	9.65918394
LOC_Os02g30200	LOC_Os02g53420	8.82497107
LOC_Os02g29020	LOC_Os12g16290	9.77013756
LOC_Os02g32370	LOC_Os06g45100	8.89873368
LOC_Os02g32370	LOC_Os02g33140	9.17413404
LOC_Os02g32030	LOC_Os03g16860	10.3348152
LOC_Os02g30200	LOC_Os05g47640	9.9886677
LOC_Os02g30630	LOC_Os11g40140	9.33347536

InteractorA	InteractorB	Z score
LOC_Os02g32030	LOC_Os06g04280	8.88583338
LOC_Os02g32030	LOC_Os05g47980	9.94025382
LOC_Os02g30200	LOC_Os11g03230	9.50476925
LOC_Os02g30200	LOC_Os05g35320	9.139188
LOC_Os02g32490	LOC_Os09g30412	9.97731213
LOC_Os02g32030	LOC_Os04g24520	9.11454613
LOC_Os02g32370	LOC_Os08g34280	9.10494234
LOC_Os02g32030	LOC_Os03g01260	8.89084941
LOC_Os02g30200	LOC_Os03g27310	9.68889904
LOC_Os02g32660	LOC_Os05g19380	8.88665513
LOC_Os02g32370	LOC_Os08g31060	10.3118258
LOC_Os02g29020	LOC_Os12g12590	9.49379489
LOC_Os02g30200	LOC_Os06g51084	9.20880479
LOC_Os02g30630	LOC_Os08g34210	9.6812512
LOC_Os02g32030	LOC_Os03g17480	9.33772425
LOC_Os02g32490	LOC_Os11g10480	9.98628584
LOC_Os02g32490	LOC_Os08g08500	9.41852127
LOC_Os02g32030	LOC_Os07g32800	9.42886646
LOC_Os02g32490	LOC_Os12g41220	9.09374812
LOC_Os02g32490	LOC_Os08g28820	10.9344292
LOC_Os02g32370	LOC_Os06g08770	9.09678698
LOC_Os02g32370	LOC_Os10g38350	9.28488214
LOC_Os02g32030	LOC_Os10g38489	8.93654707
LOC_Os02g30630	LOC_Os10g25130	9.37569529
LOC_Os02g30200	LOC_Os05g03480	9.56473582
LOC_Os02g32490	LOC_Os03g02920	8.9615037
LOC_Os02g32370	LOC_Os08g41990	10.0477878
LOC_Os02g32030	LOC_Os09g37100	10.1697935
LOC_Os02g32370	LOC_Os11g08445	9.91483691
LOC_Os02g32030	LOC_Os07g38540	10.4630508
LOC_Os02g32490	LOC_Os03g42110	8.96760223
LOC_Os02g32030	LOC_Os11g37550	9.23920691
LOC_Os02g30200	LOC_Os05g35330	10.217883
LOC_Os02g32490	LOC_Os10g31000	10.605095
LOC_Os02g32490	LOC_Os07g41050	9.48342573
LOC_Os02g32030	LOC_Os03g58050	10.2365047
LOC_Os02g32030	LOC_Os03g12500	9.21723182
LOC_Os02g32490	LOC_Os07g38540	9.53825141
LOC_Os02g32030	LOC_Os04g37460	9.13551971
LOC_Os02g32490	LOC_Os06g39875	10.2313949
LOC_Os02g30200	LOC_Os04g44470	9.22947196
LOC_Os02g32030	LOC_Os08g02700	10.6684653
LOC_Os02g32370	LOC_Os09g31120	8.90129086

InteractorA	InteractorB	Z score
LOC_Os02g32370	LOC_Os03g15120	9.69372703
LOC_Os02g33110	LOC_Os06g04510	10.2288572
LOC_Os02g33080	LOC_Os02g53790	9.91548649
LOC_Os02g33080	LOC_Os06g14510	8.86974329
LOC_Os02g33080	LOC_Os03g27370	9.10834552
LOC_Os02g32950	LOC_Os05g11550	9.38819072
LOC_Os02g33140	LOC_Os07g41750	9.23660883
LOC_Os02g33080	LOC_Os07g43470	9.50901375
LOC_Os02g33140	LOC_Os03g58050	9.60143473
LOC_Os02g32760	LOC_Os06g35480	8.93653678
LOC_Os02g33140	LOC_Os10g03540	9.70784497
LOC_Os02g34600	LOC_Os03g01800	9.37158315
LOC_Os02g33140	LOC_Os05g24580	9.44573283
LOC_Os02g33080	LOC_Os04g49130	9.03359192
LOC_Os02g34600	LOC_Os05g04340	9.93613616
LOC_Os02g33110	LOC_Os04g10010	8.94457788
LOC_Os02g33140	LOC_Os04g40990	9.68359885
LOC_Os02g33110	LOC_Os07g05160	9.035577
LOC_Os02g33110	LOC_Os03g01290	10.4955931
LOC_Os02g33110	LOC_Os03g10940	9.32742301
LOC_Os02g33140	LOC_Os05g47545	10.1655406
LOC_Os02g33140	LOC_Os02g35500	9.22659474
LOC_Os02g33080	LOC_Os10g28360	10.4820364
LOC_Os02g33080	LOC_Os10g42280	9.47022183
LOC_Os02g33140	LOC_Os02g36974	9.21786108
LOC_Os02g33080	LOC_Os05g47980	9.29007953
LOC_Os02g34600	LOC_Os04g33720	8.80220765
LOC_Os02g33080	LOC_Os05g44180	9.47730679
LOC_Os02g33140	LOC_Os10g36650	10.2882751
LOC_Os02g33080	LOC_Os12g06620	9.13888548
LOC_Os02g33080	LOC_Os06g45670	9.04959269
LOC_Os02g33110	LOC_Os03g44170	10.1527449
LOC_Os02g32950	LOC_Os11g16590	9.38768702
LOC_Os02g33080	LOC_Os06g08310	9.14008769
LOC_Os02g33140	LOC_Os07g07709	9.60143473
LOC_Os02g32950	LOC_Os04g33740	8.80076534
LOC_Os02g33080	LOC_Os10g27050	9.14824769
LOC_Os02g33080	LOC_Os09g32952	10.1106403
LOC_Os02g33110	LOC_Os12g35570	9.126965
LOC_Os02g33110	LOC_Os07g06970	9.08975317
LOC_Os02g33110	LOC_Os07g47490	9.09056035
LOC_Os02g33110	LOC_Os11g34570	8.84376138
LOC_Os02g32760	LOC_Os03g58290	10.1882874

InteractorA	InteractorB	Z score
LOC_Os02g33110	LOC_Os09g10230	8.90094657
LOC_Os02g32950	LOC_Os05g24580	9.93061225
LOC_Os02g33080	LOC_Os06g10340	9.28073566
LOC_Os02g33080	LOC_Os03g10500	8.80660232
LOC_Os02g32660	LOC_Os08g25734	9.12042151
LOC_Os02g33080	LOC_Os11g19320	8.97002988
LOC_Os02g32950	LOC_Os12g40510	9.45005291
LOC_Os02g33110	LOC_Os10g41480	8.85941093
LOC_Os02g33110	LOC_Os08g27840	8.82819778
LOC_Os02g32950	LOC_Os10g28080	10.2945225
LOC_Os02g33140	LOC_Os03g41438	9.10833174
LOC_Os02g33080	LOC_Os10g23900	10.1224681
LOC_Os02g34600	LOC_Os02g52230	8.9163006
LOC_Os02g33080	LOC_Os11g28340	9.7300057
LOC_Os02g33080	LOC_Os02g39850	9.77122739
LOC_Os02g33110	LOC_Os07g31770	10.0474412
LOC_Os02g33110	LOC_Os07g48780	9.95248082
LOC_Os02g33080	LOC_Os05g47540	9.38079937
LOC_Os02g33080	LOC_Os03g04110	9.0469854
LOC_Os02g33080	LOC_Os12g25630	8.85099551
LOC_Os02g32760	LOC_Os06g46284	9.94816284
LOC_Os02g32950	LOC_Os09g31486	9.0736713
LOC_Os02g33080	LOC_Os03g28330	10.1457518
LOC_Os02g33080	LOC_Os03g49600	8.9392455
LOC_Os02g33140	LOC_Os07g46630	8.86931711
LOC_Os02g33080	LOC_Os02g38340	9.15577351
LOC_Os02g32760	LOC_Os03g16110	10.3495615
LOC_Os02g33110	LOC_Os03g20370	9.95248506
LOC_Os02g33110	LOC_Os03g12290	8.96520271
LOC_Os02g33110	LOC_Os04g47360	8.83320801
LOC_Os02g33080	LOC_Os11g02600	9.33837556
LOC_Os02g34600	LOC_Os03g59660	9.17090554
LOC_Os02g33080	LOC_Os05g51480	10.8799222
LOC_Os02g33080	LOC_Os04g45290	9.42401487
LOC_Os02g33080	LOC_Os04g17650	8.9241585
LOC_Os02g32660	LOC_Os07g40580	12.3868036
LOC_Os02g32760	LOC_Os10g02070	9.75927714
LOC_Os02g33110	LOC_Os03g61340	8.81150079
LOC_Os02g33140	LOC_Os05g41610	9.10067001
LOC_Os02g33080	LOC_Os07g14590	9.45077951
LOC_Os02g33080	LOC_Os08g23730	9.74418025
LOC_Os02g33080	LOC_Os04g58110	10.9281546
LOC_Os02g32660	LOC_Os08g27840	9.01831666

InteractorA	InteractorB	Z score
LOC_Os02g33080	LOC_Os05g04340	9.2536064
LOC_Os02g33080	LOC_Os03g25340	9.79286216
LOC_Os02g33110	LOC_Os09g20284	10.1010563
LOC_Os02g33140	LOC_Os02g57260	10.3261367
LOC_Os02g33140	LOC_Os12g22680	9.67175055
LOC_Os02g33140	LOC_Os03g61280	9.41358396
LOC_Os02g33080	LOC_Os04g01250	9.74414469
LOC_Os02g33080	LOC_Os09g24924	8.96563877
LOC_Os02g33140	LOC_Os04g29030	11.240299
LOC_Os02g32950	LOC_Os09g31410	9.39472436
LOC_Os02g33110	LOC_Os04g53920	9.22889666
LOC_Os02g32760	LOC_Os09g19560	9.32692542
LOC_Os02g33110	LOC_Os05g33570	10.1525946
LOC_Os02g33080	LOC_Os09g25370	9.14151389
LOC_Os02g33080	LOC_Os05g45590	9.95761177
LOC_Os02g33080	LOC_Os05g05680	9.47253445
LOC_Os02g33140	LOC_Os03g57120	9.4833974
LOC_Os02g32950	LOC_Os09g21770	8.83045808
LOC_Os02g32950	LOC_Os08g41830	9.31418173
LOC_Os02g33140	LOC_Os04g12690	8.90514052
LOC_Os02g32760	LOC_Os03g12500	9.39617164
LOC_Os02g35310	LOC_Os03g02920	9.27830042
LOC_Os02g35590	LOC_Os07g30200	11.5501699
LOC_Os02g35760	LOC_Os07g44590	9.58989783
LOC_Os02g35500	LOC_Os03g21260	10.8234402
LOC_Os02g35500	LOC_Os09g32840	8.93447236
LOC_Os02g35590	LOC_Os06g36700	9.4398793
LOC_Os02g35760	LOC_Os02g53420	9.49337889
LOC_Os02g35310	LOC_Os04g39210	11.0500277
LOC_Os02g35310	LOC_Os11g05470	8.85704428
LOC_Os02g34860	LOC_Os03g01900	8.91077206
LOC_Os02g35760	LOC_Os07g07709	8.8146364
LOC_Os02g35590	LOC_Os12g31640	11.5593011
LOC_Os02g36400	LOC_Os03g38000	10.8989426
LOC_Os02g35310	LOC_Os06g04200	8.86038316
LOC_Os02g35760	LOC_Os03g58050	8.8146364
LOC_Os02g35760	LOC_Os10g38234	9.71399081
LOC_Os02g36400	LOC_Os05g02940	11.5027926
LOC_Os02g34600	LOC_Os09g36830	9.13725665
LOC_Os02g35310	LOC_Os06g15420	9.72194891
LOC_Os02g35310	LOC_Os03g04250	10.5808044
LOC_Os02g35500	LOC_Os06g46372	12.5021796
LOC_Os02g34600	LOC_Os10g29620	10.673603

InteractorA	InteractorB	Z score
LOC_Os02g35590	LOC_Os10g26110	11.1887721
LOC_Os02g35310	LOC_Os03g52090	9.49552856
LOC_Os02g35310	LOC_Os10g21240	9.96272917
LOC_Os02g35500	LOC_Os10g40700	10.3880259
LOC_Os02g35310	LOC_Os06g42130	9.92439729
LOC_Os02g35590	LOC_Os05g25850	10.2620319
LOC_Os02g35310	LOC_Os05g35320	9.32415312
LOC_Os02g35310	LOC_Os04g56730	8.9647439
LOC_Os02g35590	LOC_Os10g38360	8.90739945
LOC_Os02g35590	LOC_Os08g39860	9.10041237
LOC_Os02g35500	LOC_Os06g05250	9.1454019
LOC_Os02g35500	LOC_Os03g31210	9.20975885
LOC_Os02g35500	LOC_Os03g01260	9.62045434
LOC_Os02g35500	LOC_Os12g02370	9.06909189
LOC_Os02g35310	LOC_Os05g31140	9.79256205
LOC_Os02g35500	LOC_Os05g28940	9.37749738
LOC_Os02g35310	LOC_Os03g25370	9.02883972
LOC_Os02g35500	LOC_Os03g44170	9.43947575
LOC_Os02g35760	LOC_Os12g31640	8.95324897
LOC_Os02g35760	LOC_Os08g09770	9.41151337
LOC_Os02g35310	LOC_Os02g47790	8.90821013
LOC_Os02g35760	LOC_Os04g35200	9.19228121
LOC_Os02g35590	LOC_Os04g32460	10.4662061
LOC_Os02g35500	LOC_Os06g04200	9.29003878
LOC_Os02g35760	LOC_Os07g42950	9.36505814
LOC_Os02g35500	LOC_Os05g44760	9.17633742
LOC_Os02g35760	LOC_Os06g10970	9.3806113
LOC_Os02g36400	LOC_Os03g27370	10.2467193
LOC_Os02g35310	LOC_Os03g44150	11.917501
LOC_Os02g34600	LOC_Os07g08170	9.28129491
LOC_Os02g35310	LOC_Os09g27750	8.95392504
LOC_Os02g35310	LOC_Os09g12570	9.66132996
LOC_Os02g35310	LOC_Os03g52460	9.2123269
LOC_Os02g34600	LOC_Os06g40180	8.87615853
LOC_Os02g34600	LOC_Os08g09950	9.73773091
LOC_Os02g35500	LOC_Os07g05580	8.8462719
LOC_Os02g35590	LOC_Os07g44430	10.6367706
LOC_Os02g34860	LOC_Os05g45810	8.87843483
LOC_Os02g35310	LOC_Os03g04260	9.76974488
LOC_Os02g35590	LOC_Os06g06880	9.48289668
LOC_Os02g35760	LOC_Os06g50300	9.55747135
LOC_Os02g34600	LOC_Os07g07060	8.89995936
LOC_Os02g36400	LOC_Os03g53790	9.27699047

InteractorA	InteractorB	Z score
LOC_Os02g35590	LOC_Os09g19954	10.1448211
LOC_Os02g35310	LOC_Os06g12990	9.33147823
LOC_Os02g34600	LOC_Os09g26380	9.56961899
LOC_Os02g35500	LOC_Os06g02380	8.97615517
LOC_Os02g35310	LOC_Os12g22680	9.80790551
LOC_Os02g34600	LOC_Os07g49400	9.55684091
LOC_Os02g35310	LOC_Os04g35200	9.42998584
LOC_Os02g35760	LOC_Os12g10730	10.5940083
LOC_Os02g35590	LOC_Os10g11810	8.8928036
LOC_Os02g35590	LOC_Os11g05290	9.07771531
LOC_Os02g35760	LOC_Os08g08500	9.15035331
LOC_Os02g35310	LOC_Os03g47000	9.43923627
LOC_Os02g35310	LOC_Os06g10910	9.77001129
LOC_Os02g35310	LOC_Os07g29750	9.10946289
LOC_Os02g35310	LOC_Os02g47800	9.60415014
LOC_Os02g35500	LOC_Os03g20700	9.99755654
LOC_Os02g34600	LOC_Os07g36140	9.49898013
LOC_Os02g35500	LOC_Os04g54390	9.78987222
LOC_Os02g36400	LOC_Os04g40950	8.87274143
LOC_Os02g35310	LOC_Os08g38900	8.86588494
LOC_Os02g36400	LOC_Os04g41960	10.0281138
LOC_Os02g35590	LOC_Os11g34450	9.09924402
LOC_Os02g35590	LOC_Os04g41960	9.11001975
LOC_Os02g35590	LOC_Os02g49720	8.85278412
LOC_Os02g35590	LOC_Os06g47320	8.854256
LOC_Os02g35500	LOC_Os08g42000	8.81585302
LOC_Os02g35760	LOC_Os10g40700	9.51999912
LOC_Os02g36710	LOC_Os11g14220	8.92021279
LOC_Os02g36400	LOC_Os05g38560	9.00941905
LOC_Os02g38200	LOC_Os03g52970	9.02099503
LOC_Os02g36400	LOC_Os12g42876	9.28102596
LOC_Os02g36710	LOC_Os02g38340	10.2832819
LOC_Os02g38340	LOC_Os04g16818	9.07922677
LOC_Os02g38200	LOC_Os12g12590	9.69878868
LOC_Os02g36710	LOC_Os05g28180	10.4803604
LOC_Os02g37420	LOC_Os02g50240	13.1470437
LOC_Os02g36400	LOC_Os05g39960	9.13379606
LOC_Os02g36710	LOC_Os02g52710	9.05370476
LOC_Os02g38210	LOC_Os11g33240	9.28085488
LOC_Os02g38210	LOC_Os02g52420	9.59333366
LOC_Os02g38210	LOC_Os06g07140	9.12669726
LOC_Os02g36974	LOC_Os07g48880	9.43445337
LOC_Os02g38200	LOC_Os11g03230	8.97088923

InteractorA	InteractorB	Z score
LOC_Os02g36974	LOC_Os12g08280	9.70814975
LOC_Os02g38200	LOC_Os12g22650	10.1743306
LOC_Os02g36400	LOC_Os07g26150	9.15929146
LOC_Os02g38200	LOC_Os09g12660	9.33453218
LOC_Os02g36974	LOC_Os04g41620	10.4347163
LOC_Os02g36710	LOC_Os09g12570	9.45847276
LOC_Os02g38200	LOC_Os11g29190	8.88801648
LOC_Os02g38200	LOC_Os12g36950	9.93494296
LOC_Os02g38340	LOC_Os04g32650	8.92852121
LOC_Os02g38200	LOC_Os12g07980	9.97893004
LOC_Os02g38200	LOC_Os02g39850	8.94492923
LOC_Os02g36710	LOC_Os08g44270	9.01772931
LOC_Os02g38210	LOC_Os02g55400	9.00844896
LOC_Os02g36400	LOC_Os05g46040	9.07074519
LOC_Os02g38200	LOC_Os03g64330	8.89278567
LOC_Os02g36974	LOC_Os06g07978	9.20588462
LOC_Os02g36974	LOC_Os06g06090	9.1790278
LOC_Os02g38340	LOC_Os03g06620	8.88577507
LOC_Os02g36710	LOC_Os02g52700	9.05370476
LOC_Os02g38200	LOC_Os10g38150	8.8252067
LOC_Os02g37420	LOC_Os11g10480	9.29111794
LOC_Os02g38200	LOC_Os03g13210	9.49700989
LOC_Os02g38200	LOC_Os08g09200	10.126821
LOC_Os02g38210	LOC_Os03g08530	10.4721572
LOC_Os02g36710	LOC_Os06g49470	9.32610534
LOC_Os02g38210	LOC_Os11g02440	10.7267454
LOC_Os02g36710	LOC_Os05g43820	8.89201434
LOC_Os02g38210	LOC_Os06g19960	10.0262983
LOC_Os02g36974	LOC_Os03g64210	8.80885534
LOC_Os02g36974	LOC_Os06g07878	9.20588462
LOC_Os02g37420	LOC_Os03g51740	8.92400673
LOC_Os02g36400	LOC_Os06g35540	9.10140152
LOC_Os02g36400	LOC_Os08g17784	8.80442768
LOC_Os02g36710	LOC_Os05g04470	11.0956869
LOC_Os02g38200	LOC_Os07g05160	8.80935168
LOC_Os02g36710	LOC_Os06g11240	9.47997851
LOC_Os02g38210	LOC_Os10g32550	8.88364773
LOC_Os02g36974	LOC_Os06g35660	9.00938787
LOC_Os02g38340	LOC_Os04g16772	9.0792411
LOC_Os02g36710	LOC_Os06g23780	8.88539732
LOC_Os02g38200	LOC_Os11g05470	12.3847067
LOC_Os02g36974	LOC_Os04g45290	10.0938477
LOC_Os02g38210	LOC_Os07g07060	8.99533722

InteractorA	InteractorB	Z score
LOC_Os02g36974	LOC_Os07g38860	13.7144657
LOC_Os02g38200	LOC_Os11g47560	8.91043507
LOC_Os02g36710	LOC_Os09g25150	9.43829009
LOC_Os02g36710	LOC_Os06g51050	10.8473974
LOC_Os02g38340	LOC_Os02g46130	10.1691966
LOC_Os02g36974	LOC_Os09g25150	10.4183368
LOC_Os02g38210	LOC_Os03g15120	11.4075506
LOC_Os02g36400	LOC_Os07g47990	9.59724497
LOC_Os02g36974	LOC_Os05g44760	9.31363137
LOC_Os02g38200	LOC_Os03g09810	9.46488959
LOC_Os02g36400	LOC_Os11g28340	9.06915951
LOC_Os02g38200	LOC_Os05g30480	9.30737833
LOC_Os02g37420	LOC_Os02g57040	9.55181068
LOC_Os02g38200	LOC_Os08g14570	8.92337003
LOC_Os02g37420	LOC_Os04g41310	10.1412114
LOC_Os02g38200	LOC_Os03g51200	11.0048364
LOC_Os02g38200	LOC_Os10g28080	9.21846501
LOC_Os02g36710	LOC_Os12g07050	8.98668443
LOC_Os02g38210	LOC_Os09g12230	8.88517569
LOC_Os02g36400	LOC_Os06g23760	8.80913603
LOC_Os02g37420	LOC_Os02g52230	8.82935349
LOC_Os02g36400	LOC_Os07g05180	9.00352463
LOC_Os02g36974	LOC_Os03g10190	9.93060843
LOC_Os02g38340	LOC_Os03g59060	8.85739222
LOC_Os02g38210	LOC_Os10g38140	9.88095525
LOC_Os02g36710	LOC_Os02g55300	9.1310003
LOC_Os02g38200	LOC_Os08g41990	9.86527795
LOC_Os02g38200	LOC_Os11g08445	9.8291201
LOC_Os02g36710	LOC_Os04g59200	9.8250924
LOC_Os02g38210	LOC_Os12g38760	10.3644378
LOC_Os02g38200	LOC_Os07g10660	9.04060008
LOC_Os02g38200	LOC_Os11g08440	9.15071707
LOC_Os02g36974	LOC_Os03g13200	10.6482476
LOC_Os02g36974	LOC_Os04g57410	8.81393192
LOC_Os02g38200	LOC_Os07g39290	9.27536158
LOC_Os02g38200	LOC_Os06g11280	9.4226709
LOC_Os02g36710	LOC_Os03g32270	10.0842027
LOC_Os02g36710	LOC_Os11g25330	8.97655867
LOC_Os02g37420	LOC_Os10g38640	9.398099
LOC_Os02g38200	LOC_Os07g42490	9.0762038
LOC_Os02g38200	LOC_Os03g22060	10.0908633
LOC_Os02g37420	LOC_Os05g41210	9.1158317
LOC_Os02g36400	LOC_Os11g19800	9.99620738

InteractorA	InteractorB	Z score
LOC_Os02g36974	LOC_Os03g53800	10.4375671
LOC_Os02g36974	LOC_Os06g08770	9.28631134
LOC_Os02g38200	LOC_Os08g28800	8.98749205
LOC_Os02g38200	LOC_Os05g51630	9.22029827
LOC_Os02g38210	LOC_Os06g40180	10.3475515
LOC_Os02g38840	LOC_Os09g38030	11.561349
LOC_Os02g40830	LOC_Os02g52230	9.26952812
LOC_Os02g38840	LOC_Os09g20090	10.8414027
LOC_Os02g38840	LOC_Os06g35630	8.82414229
LOC_Os02g38840	LOC_Os06g45100	9.28531596
LOC_Os02g39850	LOC_Os04g32010	9.15911507
LOC_Os02g38840	LOC_Os09g28420	9.64348903
LOC_Os02g38340	LOC_Os07g25024	9.07922677
LOC_Os02g38840	LOC_Os09g08880	8.93372915
LOC_Os02g38920	LOC_Os02g56130	9.21346335
LOC_Os02g38840	LOC_Os06g04030	9.93980628
LOC_Os02g38840	LOC_Os05g36270	9.98140109
LOC_Os02g38920	LOC_Os04g32010	8.85546993
LOC_Os02g38340	LOC_Os06g37500	8.87256806
LOC_Os02g40830	LOC_Os02g55370	8.97966535
LOC_Os02g39850	LOC_Os08g03040	9.15453117
LOC_Os02g38840	LOC_Os03g27310	9.93979792
LOC_Os02g38840	LOC_Os02g51830	9.1508888
LOC_Os02g40000	LOC_Os10g21266	9.25308759
LOC_Os02g38840	LOC_Os07g07320	11.4108711
LOC_Os02g38340	LOC_Os07g38860	9.80405152
LOC_Os02g38840	LOC_Os08g36320	9.0463534
LOC_Os02g39064	LOC_Os03g49380	8.81339564
LOC_Os02g39850	LOC_Os08g09370	8.84073319
LOC_Os02g38840	LOC_Os02g44550	11.1717016
LOC_Os02g39064	LOC_Os08g04180	8.96146275
LOC_Os02g38840	LOC_Os02g55300	9.24600363
LOC_Os02g39850	LOC_Os04g14680	10.603287
LOC_Os02g38840	LOC_Os09g23550	9.34233397
LOC_Os02g38840	LOC_Os11g32620	8.86367052
LOC_Os02g38920	LOC_Os08g14760	9.60437816
LOC_Os02g40830	LOC_Os03g58430	8.83750418
LOC_Os02g38340	LOC_Os06g36770	9.66929614
LOC_Os02g39850	LOC_Os04g42250	8.96353056
LOC_Os02g38840	LOC_Os02g57040	8.92074924
LOC_Os02g40000	LOC_Os03g63090	9.28414999
LOC_Os02g39850	LOC_Os03g57040	9.82380934
LOC_Os02g38920	LOC_Os10g21326	8.85182696

InteractorA	InteractorB	Z score
LOC_Os02g38840	LOC_Os04g33190	10.4002855
LOC_Os02g38920	LOC_Os04g34630	9.15213905
LOC_Os02g40830	LOC_Os03g42840	9.46325767
LOC_Os02g38920	LOC_Os11g37550	9.66559426
LOC_Os02g38920	LOC_Os04g16844	8.85182696
LOC_Os02g40000	LOC_Os02g50880	10.0506038
LOC_Os02g38840	LOC_Os08g10608	9.22760919
LOC_Os02g38840	LOC_Os08g04180	9.68152278
LOC_Os02g38340	LOC_Os09g24412	9.0792411
LOC_Os02g38840	LOC_Os09g23530	10.4015738
LOC_Os02g38840	LOC_Os06g35520	8.85462419
LOC_Os02g38840	LOC_Os11g24560	9.5271845
LOC_Os02g40000	LOC_Os04g19740	9.27098553
LOC_Os02g39064	LOC_Os09g08720	9.86755375
LOC_Os02g40000	LOC_Os03g48471	8.9832247
LOC_Os02g40000	LOC_Os08g34170	10.2371006
LOC_Os02g38920	LOC_Os08g23110	8.81090264
LOC_Os02g40000	LOC_Os05g41080	9.67832517
LOC_Os02g38920	LOC_Os04g40130	8.83550451
LOC_Os02g40000	LOC_Os03g62060	8.96147709
LOC_Os02g40000	LOC_Os04g59150	10.0711294
LOC_Os02g39850	LOC_Os10g25674	9.23328503
LOC_Os02g38920	LOC_Os08g03290	9.16930502
LOC_Os02g39064	LOC_Os06g28550	9.26147907
LOC_Os02g40000	LOC_Os05g39580	9.2938933
LOC_Os02g39850	LOC_Os03g61600	9.42478043
LOC_Os02g39850	LOC_Os02g57630	8.84607451
LOC_Os02g38840	LOC_Os06g08080	9.93472386
LOC_Os02g38340	LOC_Os10g21352	9.0792411
LOC_Os02g38340	LOC_Os07g26540	8.9601633
LOC_Os02g38340	LOC_Os07g05400	12.5311583
LOC_Os02g39850	LOC_Os02g46970	9.46265329
LOC_Os02g38920	LOC_Os10g27174	9.00241783
LOC_Os02g40830	LOC_Os03g06940	9.33406055
LOC_Os02g39850	LOC_Os03g01630	10.1462809
LOC_Os02g38340	LOC_Os10g25140	8.96465598
LOC_Os02g38840	LOC_Os04g20810	9.62894725
LOC_Os02g38340	LOC_Os07g43670	8.91636921
LOC_Os02g38920	LOC_Os03g56460	8.87918421
LOC_Os02g38840	LOC_Os04g10400	9.24654602
LOC_Os02g38920	LOC_Os04g40950	9.06113855
LOC_Os02g39850	LOC_Os04g33190	9.02158522
LOC_Os02g38840	LOC_Os05g37700	9.46823141

InteractorA	InteractorB	Z score
LOC_Os02g38840	LOC_Os04g28180	9.20834192
LOC_Os02g38840	LOC_Os03g45320	8.86879952
LOC_Os02g40830	LOC_Os03g60400	9.04751706
LOC_Os02g39850	LOC_Os07g28280	9.15668576
LOC_Os02g38840	LOC_Os04g46560	9.94756181
LOC_Os02g38920	LOC_Os03g07150	8.90023717
LOC_Os02g40000	LOC_Os03g31300	9.35302982
LOC_Os02g40830	LOC_Os03g11420	9.49625768
LOC_Os02g38920	LOC_Os03g41460	10.5282916
LOC_Os02g38920	LOC_Os03g63090	8.88377043
LOC_Os02g38920	LOC_Os08g25734	9.46688075
LOC_Os02g38840	LOC_Os11g05570	9.24683915
LOC_Os02g40000	LOC_Os09g10200	10.4967259
LOC_Os02g38340	LOC_Os08g15266	9.0792411
LOC_Os02g38840	LOC_Os05g33730	10.4613793
LOC_Os02g38340	LOC_Os06g43640	9.31245201
LOC_Os02g41590	LOC_Os05g50710	9.46791998
LOC_Os02g41470	LOC_Os05g49800	10.5608692
LOC_Os02g41470	LOC_Os07g31750	9.9459071
LOC_Os02g41590	LOC_Os05g06480	8.93758661
LOC_Os02g41650	LOC_Os05g49840	9.04290621
LOC_Os02g41650	LOC_Os04g26910	10.2132082
LOC_Os02g41470	LOC_Os03g58400	9.7980125
LOC_Os02g40830	LOC_Os04g55960	9.54569206
LOC_Os02g41470	LOC_Os03g12510	9.03382906
LOC_Os02g41590	LOC_Os09g23560	9.19343689
LOC_Os02g41590	LOC_Os10g31950	10.0498743
LOC_Os02g41470	LOC_Os06g11280	9.4219593
LOC_Os02g41650	LOC_Os02g41670	17.0985291
LOC_Os02g41670	LOC_Os07g02350	10.5945417
LOC_Os02g41470	LOC_Os10g42720	8.84958164
LOC_Os02g41630	LOC_Os07g43170	8.94991917
LOC_Os02g41670	LOC_Os03g17700	9.98859938
LOC_Os02g40830	LOC_Os07g46630	10.1540207
LOC_Os02g41470	LOC_Os03g52970	9.55216326
LOC_Os02g40830	LOC_Os09g34250	10.6908025
LOC_Os02g41670	LOC_Os07g22930	9.19362072
LOC_Os02g41630	LOC_Os02g41650	10.0192942
LOC_Os02g41650	LOC_Os12g13810	8.99051874
LOC_Os02g41650	LOC_Os07g34190	9.01652915
LOC_Os02g41590	LOC_Os02g55890	9.58518187
LOC_Os02g41630	LOC_Os06g01390	9.39936951
LOC_Os02g41630	LOC_Os04g43800	12.5987823

InteractorA	InteractorB	Z score
LOC_Os02g41650	LOC_Os04g43760	14.2539123
LOC_Os02g41670	LOC_Os04g43760	13.5312734
LOC_Os02g41470	LOC_Os04g40130	8.92671702
LOC_Os02g41470	LOC_Os11g24560	9.39817974
LOC_Os02g40830	LOC_Os12g10720	9.31300941
LOC_Os02g41670	LOC_Os03g15360	9.0018318
LOC_Os02g41630	LOC_Os12g33610	12.9920856
LOC_Os02g41630	LOC_Os02g45540	9.20791668
LOC_Os02g41630	LOC_Os04g32710	9.31940695
LOC_Os02g41470	LOC_Os02g46130	9.32741514
LOC_Os02g41470	LOC_Os11g39540	8.84750195
LOC_Os02g40830	LOC_Os05g34540	9.56010189
LOC_Os02g40830	LOC_Os07g02350	9.02931197
LOC_Os02g41630	LOC_Os11g08440	8.91678645
LOC_Os02g41470	LOC_Os03g20370	11.0949836
LOC_Os02g40830	LOC_Os07g48030	10.4735358
LOC_Os02g40830	LOC_Os06g08600	9.08184096
LOC_Os02g41470	LOC_Os04g37950	9.07439788
LOC_Os02g41670	LOC_Os03g52460	9.17042076
LOC_Os02g40830	LOC_Os05g35290	9.77050849
LOC_Os02g40830	LOC_Os05g44140	9.5444377
LOC_Os02g41470	LOC_Os10g21240	10.5875083
LOC_Os02g41630	LOC_Os11g48110	10.6540969
LOC_Os02g41590	LOC_Os03g49580	9.16073348
LOC_Os02g41470	LOC_Os05g48040	8.98061792
LOC_Os02g41630	LOC_Os09g08072	9.15058895
LOC_Os02g41670	LOC_Os12g33610	8.88527056
LOC_Os02g41630	LOC_Os10g38229	8.8742484
LOC_Os02g41670	LOC_Os06g06040	8.92708146
LOC_Os02g41650	LOC_Os05g35290	13.825758
LOC_Os02g41650	LOC_Os08g25570	8.93990105
LOC_Os02g41630	LOC_Os07g07320	9.02832022
LOC_Os02g41590	LOC_Os10g29470	9.55738128
LOC_Os02g40830	LOC_Os06g37150	8.92145263
LOC_Os02g41470	LOC_Os09g10260	9.30046067
LOC_Os02g41470	LOC_Os12g23170	8.88257506
LOC_Os02g41680	LOC_Os02g56180	8.95537706
LOC_Os02g41650	LOC_Os04g52361	8.91998796
LOC_Os02g41670	LOC_Os06g39230	9.01530831
LOC_Os02g40830	LOC_Os06g05690	8.9158943
LOC_Os02g41630	LOC_Os04g43760	14.8035716
LOC_Os02g41470	LOC_Os12g13390	9.28042051
LOC_Os02g41470	LOC_Os03g29570	9.84351907

InteractorA	InteractorB	Z score
LOC_Os02g41670	LOC_Os09g20090	9.12115419
LOC_Os02g41470	LOC_Os05g38230	11.4754114
LOC_Os02g41670	LOC_Os04g43800	10.2650581
LOC_Os02g41630	LOC_Os07g03368	9.41387986
LOC_Os02g40830	LOC_Os08g03040	8.83982849
LOC_Os02g41670	LOC_Os06g27770	8.83200068
LOC_Os02g41670	LOC_Os09g27420	9.52398483
LOC_Os02g41650	LOC_Os08g42000	10.6048327
LOC_Os02g40830	LOC_Os05g33570	9.07595612
LOC_Os02g41470	LOC_Os05g08430	9.67713158
LOC_Os02g41630	LOC_Os04g43400	8.96083995
LOC_Os02g41670	LOC_Os02g41680	9.31503278
LOC_Os02g41680	LOC_Os03g04169	9.20476783
LOC_Os02g40830	LOC_Os08g28800	8.83018944
LOC_Os02g41590	LOC_Os06g11200	9.38846486
LOC_Os02g40830	LOC_Os07g40290	8.93608893
LOC_Os02g40830	LOC_Os11g31530	9.33740816
LOC_Os02g41650	LOC_Os02g41680	9.15925906
LOC_Os02g40830	LOC_Os08g44210	8.9099064
LOC_Os02g41670	LOC_Os08g23730	8.8515817
LOC_Os02g40830	LOC_Os06g23780	9.3339876
LOC_Os02g41650	LOC_Os04g43800	17.9424686
LOC_Os02g41630	LOC_Os10g21250	8.83101762
LOC_Os02g41470	LOC_Os07g48780	11.0949836
LOC_Os02g40830	LOC_Os12g13390	9.21877497
LOC_Os02g40830	LOC_Os05g11730	10.55657
LOC_Os02g41650	LOC_Os03g23970	8.87864754
LOC_Os02g41470	LOC_Os07g47490	9.15614238
LOC_Os02g41650	LOC_Os05g40990	9.18755318
LOC_Os02g41590	LOC_Os09g23530	9.00855569
LOC_Os02g41670	LOC_Os08g39300	9.39407953
LOC_Os02g41650	LOC_Os04g40130	10.9473524
LOC_Os02g40830	LOC_Os06g06100	10.5146194
LOC_Os02g41670	LOC_Os08g42000	10.2241527
LOC_Os02g41630	LOC_Os02g41670	12.6262352
LOC_Os02g40830	LOC_Os10g25140	10.9215542
LOC_Os02g41470	LOC_Os09g25390	8.84511736
LOC_Os02g40830	LOC_Os06g10330	8.92484908
LOC_Os02g41650	LOC_Os12g33610	9.96558049
LOC_Os02g41670	LOC_Os10g08670	10.9049812
LOC_Os02g41590	LOC_Os08g32850	9.02288145
LOC_Os02g41470	LOC_Os03g38980	16.4635621
LOC_Os02g41630	LOC_Os12g42884	10.3049182

InteractorA	InteractorB	Z score
LOC_Os02g41650	LOC_Os08g29520	9.00305336
LOC_Os02g41630	LOC_Os05g35290	16.9052876
LOC_Os02g41590	LOC_Os09g08072	9.96153218
LOC_Os02g42810	LOC_Os06g35480	10.7995073
LOC_Os02g42320	LOC_Os03g52090	9.70700665
LOC_Os02g42810	LOC_Os12g07820	9.90419362
LOC_Os02g42810	LOC_Os04g57950	9.06554674
LOC_Os02g41860	LOC_Os04g36700	8.88403737
LOC_Os02g41680	LOC_Os06g49470	9.17477291
LOC_Os02g42320	LOC_Os07g01020	9.4764386
LOC_Os02g42810	LOC_Os03g05980	9.05834325
LOC_Os02g42320	LOC_Os04g53214	9.77882565
LOC_Os02g41860	LOC_Os10g22450	9.57521739
LOC_Os02g41860	LOC_Os08g16910	9.78178388
LOC_Os02g42710	LOC_Os05g37700	9.44211473
LOC_Os02g42810	LOC_Os12g17540	11.7201518
LOC_Os02g42320	LOC_Os05g29880	8.99963956
LOC_Os02g42810	LOC_Os06g45590	8.8050751
LOC_Os02g43010	LOC_Os02g55060	9.53492804
LOC_Os02g41680	LOC_Os04g43800	9.59953728
LOC_Os02g42810	LOC_Os06g29220	9.13868489
LOC_Os02g42320	LOC_Os03g16860	8.86643708
LOC_Os02g42520	LOC_Os06g50300	8.88645545
LOC_Os02g42320	LOC_Os05g34540	9.05412268
LOC_Os02g42710	LOC_Os03g15360	9.47800011
LOC_Os02g41680	LOC_Os04g56730	8.80879608
LOC_Os02g42520	LOC_Os04g18200	9.40567966
LOC_Os02g41860	LOC_Os07g49220	9.15362658
LOC_Os02g41680	LOC_Os12g16410	8.88694377
LOC_Os02g42810	LOC_Os12g40550	8.96138581
LOC_Os02g42320	LOC_Os08g09250	8.976069
LOC_Os02g42810	LOC_Os07g42600	9.85626574
LOC_Os02g41680	LOC_Os12g33610	16.9984196
LOC_Os02g41860	LOC_Os03g10940	9.06923035
LOC_Os02g42810	LOC_Os07g38910	9.10947595
LOC_Os02g41860	LOC_Os04g30800	8.98605075
LOC_Os02g42520	LOC_Os02g57260	9.30119339
LOC_Os02g42520	LOC_Os12g07980	9.02430855
LOC_Os02g41680	LOC_Os07g22930	9.51091515
LOC_Os02g42320	LOC_Os05g24580	10.1756303
LOC_Os02g42520	LOC_Os07g48010	9.17506883
LOC_Os02g42320	LOC_Os06g37080	9.84155983
LOC_Os02g42710	LOC_Os11g32650	9.13797052

InteractorA	InteractorB	Z score
LOC_Os02g42710	LOC_Os11g37970	9.73130031
LOC_Os02g42520	LOC_Os10g30840	10.831181
LOC_Os02g42520	LOC_Os08g03440	9.29924502
LOC_Os02g42320	LOC_Os11g32510	9.4337634
LOC_Os02g42520	LOC_Os11g10520	10.3138964
LOC_Os02g41860	LOC_Os02g46970	8.87310191
LOC_Os02g42520	LOC_Os04g48540	9.14183456
LOC_Os02g42810	LOC_Os04g39210	9.49425661
LOC_Os02g42320	LOC_Os12g44020	9.04583854
LOC_Os02g42810	LOC_Os06g45120	10.4549919
LOC_Os02g42810	LOC_Os11g03290	11.0566594
LOC_Os02g41680	LOC_Os04g43760	9.78203847
LOC_Os02g42710	LOC_Os05g10780	9.50051969
LOC_Os02g42810	LOC_Os02g52630	8.85142537
LOC_Os02g41860	LOC_Os12g16250	8.97934216
LOC_Os02g42320	LOC_Os09g27820	9.23689661
LOC_Os02g42810	LOC_Os12g36950	8.81966186
LOC_Os02g42710	LOC_Os07g48040	9.30411425
LOC_Os02g42520	LOC_Os09g25370	10.0390329
LOC_Os02g41680	LOC_Os05g35290	11.8970346
LOC_Os02g42810	LOC_Os06g37610	9.24125431
LOC_Os02g42520	LOC_Os11g10480	9.35742307
LOC_Os02g42810	LOC_Os06g05690	9.38699825
LOC_Os02g41860	LOC_Os07g34140	10.1386706
LOC_Os02g42520	LOC_Os02g57040	10.5855195
LOC_Os02g41680	LOC_Os05g19380	8.84529732
LOC_Os02g41680	LOC_Os03g58130	8.95611902
LOC_Os02g42810	LOC_Os07g07470	9.22929648
LOC_Os02g42520	LOC_Os03g13200	8.94207744
LOC_Os02g42810	LOC_Os11g38959	9.05834095
LOC_Os02g41680	LOC_Os11g14910	9.89478115
LOC_Os02g42520	LOC_Os08g44530	8.82816706
LOC_Os02g41680	LOC_Os10g26390	9.40801187
LOC_Os02g42710	LOC_Os04g44920	9.19378779
LOC_Os02g42520	LOC_Os12g10730	8.92312646
LOC_Os02g42810	LOC_Os05g41640	8.89092038
LOC_Os02g42810	LOC_Os03g62670	8.86736017
LOC_Os02g41860	LOC_Os05g02940	9.35420477
LOC_Os02g42810	LOC_Os07g44590	10.2295185
LOC_Os02g42810	LOC_Os06g15990	9.57225175
LOC_Os02g42520	LOC_Os04g28180	9.46143762
LOC_Os02g41680	LOC_Os04g53230	8.84756128
LOC_Os02g42810	LOC_Os12g07720	9.94633257

InteractorA	InteractorB	Z score
LOC_Os02g42520	LOC_Os03g02260	8.95755799
LOC_Os02g41860	LOC_Os04g58200	9.15663116
LOC_Os02g42810	LOC_Os07g48430	9.58754778
LOC_Os02g43010	LOC_Os11g14910	8.96692655
LOC_Os02g44000	LOC_Os11g26910	9.55308588
LOC_Os02g44630	LOC_Os06g39875	9.0841523
LOC_Os02g44630	LOC_Os02g56690	9.40295278
LOC_Os02g45540	LOC_Os02g46130	9.84876693
LOC_Os02g44550	LOC_Os04g18200	9.13338512
LOC_Os02g44000	LOC_Os07g07550	9.21427983
LOC_Os02g44550	LOC_Os03g40330	9.54215707
LOC_Os02g44080	LOC_Os07g05400	9.03772646
LOC_Os02g43010	LOC_Os04g37820	9.1176659
LOC_Os02g44550	LOC_Os12g41110	8.80900373
LOC_Os02g43010	LOC_Os12g44010	9.084624
LOC_Os02g43010	LOC_Os05g01050	9.50756658
LOC_Os02g44630	LOC_Os03g12270	9.46545377
LOC_Os02g44000	LOC_Os03g53800	9.1355854
LOC_Os02g45540	LOC_Os03g13170	9.23741997
LOC_Os02g44080	LOC_Os03g22060	9.16363553
LOC_Os02g43010	LOC_Os05g36010	9.16203511
LOC_Os02g43010	LOC_Os12g38180	11.0840615
LOC_Os02g44550	LOC_Os06g51084	8.83835487
LOC_Os02g44780	LOC_Os06g06100	8.83146633
LOC_Os02g44000	LOC_Os03g55150	10.9694429
LOC_Os02g45540	LOC_Os03g28400	9.31011355
LOC_Os02g44000	LOC_Os06g11290	9.53461904
LOC_Os02g44080	LOC_Os07g38890	10.907178
LOC_Os02g44780	LOC_Os10g31950	8.87870379
LOC_Os02g45540	LOC_Os03g08570	9.45624667
LOC_Os02g44000	LOC_Os05g19380	9.11665959
LOC_Os02g44780	LOC_Os07g48040	8.93119664
LOC_Os02g44000	LOC_Os04g42920	9.01733906
LOC_Os02g44000	LOC_Os06g51084	9.69955848
LOC_Os02g44780	LOC_Os04g39030	8.87469584
LOC_Os02g43010	LOC_Os04g09604	9.74347987
LOC_Os02g44780	LOC_Os06g05700	8.90647581
LOC_Os02g44550	LOC_Os03g46070	8.81786567
LOC_Os02g44550	LOC_Os06g50300	9.29280893
LOC_Os02g44000	LOC_Os05g46860	9.09035401
LOC_Os02g44780	LOC_Os03g61600	8.82315531
LOC_Os02g44000	LOC_Os07g42924	9.06478374
LOC_Os02g44000	LOC_Os08g39870	9.08187595

InteractorA	InteractorB	Z score
LOC_Os02g44630	LOC_Os07g34520	9.37715326
LOC_Os02g44630	LOC_Os05g05800	8.82474123
LOC_Os02g43010	LOC_Os12g36950	9.47105041
LOC_Os02g44550	LOC_Os11g32610	10.3553825
LOC_Os02g44550	LOC_Os04g33040	9.27119654
LOC_Os02g44550	LOC_Os08g03290	9.00539537
LOC_Os02g44080	LOC_Os07g40290	10.0559551
LOC_Os02g44080	LOC_Os05g05800	8.98137417
LOC_Os02g44000	LOC_Os08g06100	8.95646827
LOC_Os02g44780	LOC_Os06g29180	8.82450195
LOC_Os02g44080	LOC_Os05g46360	11.5476004
LOC_Os02g44780	LOC_Os03g42840	9.83696156
LOC_Os02g43010	LOC_Os07g37320	9.7624015
LOC_Os02g44000	LOC_Os06g44270	9.03503007
LOC_Os02g44000	LOC_Os09g34960	8.82410634
LOC_Os02g44550	LOC_Os07g44430	8.82816514
LOC_Os02g44000	LOC_Os04g20070	8.87824915
LOC_Os02g44000	LOC_Os05g49760	9.7033754
LOC_Os02g44550	LOC_Os12g02980	8.8618172
LOC_Os02g44780	LOC_Os04g42380	9.3384935
LOC_Os02g44550	LOC_Os11g01872	10.8393323
LOC_Os02g44000	LOC_Os05g29880	8.91995354
LOC_Os01g10590	LOC_Os06g48160	8.94206857
LOC_Os01g09700	LOC_Os05g29880	8.82937265
LOC_Os01g10110	LOC_Os06g40640	10.0226979
LOC_Os01g10820	LOC_Os05g44050	10.7482139
LOC_Os01g09700	LOC_Os02g52710	9.37157075
LOC_Os01g10820	LOC_Os01g14860	9.05366044
LOC_Os01g10110	LOC_Os01g27380	10.5611476
LOC_Os01g10590	LOC_Os02g52420	10.4655168
LOC_Os01g10290	LOC_Os12g10600	10.1739173
LOC_Os01g10290	LOC_Os04g59160	9.4888187
LOC_Os01g10590	LOC_Os09g31486	9.19792702
LOC_Os01g10590	LOC_Os02g01340	9.17602035
LOC_Os01g10820	LOC_Os01g71310	8.86456848
LOC_Os01g10590	LOC_Os03g22120	9.63314703
LOC_Os01g09700	LOC_Os10g34520	9.84991382
LOC_Os01g10110	LOC_Os03g58300	9.89421915
LOC_Os01g10290	LOC_Os06g08310	9.038489
LOC_Os01g10290	LOC_Os04g16680	8.88255718
LOC_Os01g10290	LOC_Os10g40700	9.45511435
LOC_Os01g09700	LOC_Os05g25490	16.0686588
LOC_Os01g09700	LOC_Os02g56180	9.23878989

InteractorA	InteractorB	Z score
LOC_Os01g10820	LOC_Os02g24634	8.94473696
LOC_Os01g10110	LOC_Os05g31020	9.06680972
LOC_Os01g10290	LOC_Os10g31950	9.17063848
LOC_Os01g10820	LOC_Os01g51410	8.9846041
LOC_Os01g09700	LOC_Os07g34140	9.71215001
LOC_Os01g10820	LOC_Os03g08020	9.34735935
LOC_Os01g09700	LOC_Os03g61600	9.70410123
LOC_Os01g10110	LOC_Os03g20370	9.09661101
LOC_Os01g10290	LOC_Os11g08445	8.91416661
LOC_Os01g10290	LOC_Os10g11260	8.98722648
LOC_Os01g10820	LOC_Os06g46000	9.52531729
LOC_Os01g10820	LOC_Os03g01630	8.91959507
LOC_Os01g10820	LOC_Os03g08050	9.34731756
LOC_Os01g10110	LOC_Os02g02410	9.1388566
LOC_Os01g10290	LOC_Os10g38950	8.85482823
LOC_Os01g10110	LOC_Os12g25700	8.88877349
LOC_Os01g10290	LOC_Os11g08470	9.15310302
LOC_Os01g10110	LOC_Os05g34170	10.8058948
LOC_Os01g10290	LOC_Os05g10780	9.76958056
LOC_Os01g10110	LOC_Os08g20270	10.6939264
LOC_Os01g09700	LOC_Os02g52700	9.37159162
LOC_Os01g09700	LOC_Os05g10780	9.52911529
LOC_Os01g10290	LOC_Os07g29440	9.09255094
LOC_Os01g10290	LOC_Os08g27840	10.7871699
LOC_Os01g10820	LOC_Os05g11550	8.95572477
LOC_Os01g10290	LOC_Os09g30412	9.2863707
LOC_Os01g10290	LOC_Os01g13120	9.32779379
LOC_Os01g10110	LOC_Os05g11550	9.54185107
LOC_Os01g10110	LOC_Os01g17330	9.18052392
LOC_Os01g10110	LOC_Os01g70300	9.68686615
LOC_Os01g10290	LOC_Os04g02050	9.23656264
LOC_Os01g10110	LOC_Os04g48060	9.4874932
LOC_Os01g10590	LOC_Os10g36650	9.64035628
LOC_Os01g10110	LOC_Os10g38950	10.4172811
LOC_Os01g10110	LOC_Os04g37640	11.3551164
LOC_Os01g10590	LOC_Os04g52280	9.30076284
LOC_Os01g09700	LOC_Os08g33370	9.79622615
LOC_Os01g10110	LOC_Os02g07060	9.48765169
LOC_Os01g10820	LOC_Os03g39610	8.83413678
LOC_Os01g10820	LOC_Os07g05580	8.94368872
LOC_Os01g10820	LOC_Os08g36900	8.96219852
LOC_Os01g10590	LOC_Os05g37884	8.82580296
LOC_Os01g10110	LOC_Os05g28940	12.276955

InteractorA	InteractorB	Z score
LOC_Os01g10590	LOC_Os10g41510	9.18968439
LOC_Os01g09700	LOC_Os03g11900	8.87557981
LOC_Os01g10110	LOC_Os01g73200	8.83540024
LOC_Os01g10290	LOC_Os04g12690	10.4873625
LOC_Os01g10590	LOC_Os04g40310	8.83714022
LOC_Os01g10590	LOC_Os02g52710	8.82541261
LOC_Os01g10820	LOC_Os06g40170	8.90310602
LOC_Os01g10820	LOC_Os01g74650	9.09031371
LOC_Os01g10110	LOC_Os02g18930	9.28958403
LOC_Os01g10110	LOC_Os01g62870	9.05029783
LOC_Os01g09700	LOC_Os10g37060	8.88213445
LOC_Os01g10290	LOC_Os09g17740	9.77558634
LOC_Os01g10110	LOC_Os05g31040	9.48887473
LOC_Os01g10290	LOC_Os09g31486	10.0394225
LOC_Os01g10290	LOC_Os02g44080	8.95101652
LOC_Os01g10590	LOC_Os03g26450	8.96745338
LOC_Os01g10820	LOC_Os07g36140	9.34741649
LOC_Os01g10820	LOC_Os03g61600	8.89689407
LOC_Os01g09700	LOC_Os05g43510	8.87375177
LOC_Os01g10820	LOC_Os03g31750	8.87378936
LOC_Os01g10590	LOC_Os05g01810	9.45827669
LOC_Os01g10820	LOC_Os02g57450	9.39592221
LOC_Os01g10820	LOC_Os07g08170	9.06322043
LOC_Os01g10110	LOC_Os06g11260	9.47530918
LOC_Os01g10110	LOC_Os01g50050	9.29114726
LOC_Os01g10110	LOC_Os11g08340	9.30535011
LOC_Os01g10590	LOC_Os08g40930	9.49210107
LOC_Os01g10290	LOC_Os03g49380	9.58033733
LOC_Os01g10820	LOC_Os03g08010	9.34732725
LOC_Os01g10820	LOC_Os02g01590	9.00244531
LOC_Os01g09700	LOC_Os03g51740	12.6261706
LOC_Os01g10290	LOC_Os01g52450	9.6832521
LOC_Os01g10820	LOC_Os05g49760	8.98752034
LOC_Os01g09700	LOC_Os05g48510	9.09944305
LOC_Os01g09700	LOC_Os08g34290	9.2095641
LOC_Os01g09700	LOC_Os07g11440	9.97969555
LOC_Os01g10820	LOC_Os08g40140	9.04765731
LOC_Os01g09700	LOC_Os04g58570	9.79019852
LOC_Os01g10290	LOC_Os06g10910	9.09893423
LOC_Os01g10820	LOC_Os07g10720	10.2512825
LOC_Os01g10290	LOC_Os01g16890	10.6651418
LOC_Os01g10820	LOC_Os05g19380	9.64779557
LOC_Os01g09700	LOC_Os11g03290	10.7359872

InteractorA	InteractorB	Z score
LOC_Os01g10590	LOC_Os05g04340	9.21390904
LOC_Os01g09700	LOC_Os09g39380	9.40916471
LOC_Os01g10290	LOC_Os02g57720	8.97492206
LOC_Os01g10590	LOC_Os02g52700	8.82539126
LOC_Os01g10820	LOC_Os06g47320	10.0869912
LOC_Os01g10820	LOC_Os01g52470	10.9769326
LOC_Os01g10290	LOC_Os06g36880	8.86231561
LOC_Os01g09700	LOC_Os10g01080	9.18695636
LOC_Os01g10590	LOC_Os02g02840	8.82082809
LOC_Os01g09700	LOC_Os05g35770	9.58326921
LOC_Os01g10110	LOC_Os07g43390	8.85562187
LOC_Os01g10110	LOC_Os01g16240	9.09660279
LOC_Os01g10110	LOC_Os07g48780	9.09654112
LOC_Os01g09700	LOC_Os02g19420	8.85760754
LOC_Os01g10290	LOC_Os10g41550	10.1603041
LOC_Os01g10820	LOC_Os04g24430	9.26854978
LOC_Os01g10110	LOC_Os04g44470	8.94947847
LOC_Os01g10290	LOC_Os06g39875	8.83788362
LOC_Os01g10820	LOC_Os06g42130	9.34459412
LOC_Os01g09700	LOC_Os07g17010	9.28708064
LOC_Os01g10110	LOC_Os08g03040	9.21987036
LOC_Os02g46130	LOC_Os02g51930	9.9431225
LOC_Os02g47110	LOC_Os04g33740	9.67048255
LOC_Os02g46130	LOC_Os07g22950	9.00280829
LOC_Os02g47020	LOC_Os12g06660	9.12220533
LOC_Os02g45540	LOC_Os04g44730	9.0019964
LOC_Os02g45940	LOC_Os12g22680	9.95584485
LOC_Os02g46970	LOC_Os05g07090	10.3286458
LOC_Os02g47020	LOC_Os04g10010	9.89942959
LOC_Os02g45940	LOC_Os10g01540	9.10660661
LOC_Os02g46130	LOC_Os05g04340	9.38776356
LOC_Os02g46970	LOC_Os03g08560	9.28318213
LOC_Os02g46970	LOC_Os06g44080	9.96049795
LOC_Os02g47110	LOC_Os03g49220	9.06477332
LOC_Os02g46130	LOC_Os06g15420	9.05411008
LOC_Os02g46130	LOC_Os06g36700	9.04195169
LOC_Os02g46970	LOC_Os05g37690	8.99937193
LOC_Os02g46970	LOC_Os05g34170	10.1919212
LOC_Os02g46970	LOC_Os03g07300	9.77522565
LOC_Os02g47020	LOC_Os10g21248	9.28916491
LOC_Os02g46970	LOC_Os07g44460	10.3451495
LOC_Os02g47020	LOC_Os07g07060	8.97262498
LOC_Os02g46970	LOC_Os11g01360	9.57280136

InteractorA	InteractorB	Z score
LOC_Os02g46970	LOC_Os11g31530	9.0604689
LOC_Os02g45540	LOC_Os06g11280	9.92166481
LOC_Os02g46970	LOC_Os11g08330	9.34757292
LOC_Os02g45940	LOC_Os10g22070	9.42850276
LOC_Os02g46970	LOC_Os07g38890	9.38660648
LOC_Os02g45940	LOC_Os02g56460	10.706
LOC_Os02g45940	LOC_Os11g10520	10.9140543
LOC_Os02g46970	LOC_Os09g25320	9.96049795
LOC_Os02g46970	LOC_Os07g07709	9.79232593
LOC_Os02g46970	LOC_Os06g35660	11.476739
LOC_Os02g46970	LOC_Os04g36800	8.97921281
LOC_Os02g45940	LOC_Os03g07150	9.73068597
LOC_Os02g46130	LOC_Os06g46000	9.16348124
LOC_Os02g45540	LOC_Os07g29750	8.86994086
LOC_Os02g46970	LOC_Os08g42000	9.3922786
LOC_Os02g46970	LOC_Os05g50890	9.65435807
LOC_Os02g46970	LOC_Os05g07720	9.29674279
LOC_Os02g45940	LOC_Os11g31620	9.02282073
LOC_Os02g46970	LOC_Os03g04060	9.89627406
LOC_Os02g47590	LOC_Os03g25300	9.85555624
LOC_Os02g46970	LOC_Os04g40990	10.3022455
LOC_Os02g46970	LOC_Os10g21268	10.7946656
LOC_Os02g45540	LOC_Os06g10910	8.86413341
LOC_Os02g46970	LOC_Os03g62670	9.26299711
LOC_Os02g46970	LOC_Os10g28350	9.82989469
LOC_Os02g45540	LOC_Os04g27980	8.91041035
LOC_Os02g45940	LOC_Os03g31300	10.4718022
LOC_Os02g45540	LOC_Os11g08460	8.93348001
LOC_Os02g46970	LOC_Os11g47570	8.99297602
LOC_Os02g47110	LOC_Os03g27280	8.86899851
LOC_Os02g45940	LOC_Os11g10480	8.92560379
LOC_Os02g46970	LOC_Os05g35330	10.7945605
LOC_Os02g45540	LOC_Os12g25120	9.97769142
LOC_Os02g45540	LOC_Os10g38640	9.45367502
LOC_Os02g46130	LOC_Os03g08800	9.66348976
LOC_Os02g46970	LOC_Os03g17700	10.5503643
LOC_Os02g46970	LOC_Os04g09540	8.80479472
LOC_Os02g47020	LOC_Os08g44810	8.93966124
LOC_Os02g46970	LOC_Os04g59450	9.29063173
LOC_Os02g45540	LOC_Os07g43390	10.1246324
LOC_Os02g46970	LOC_Os05g51480	10.5953125
LOC_Os02g47590	LOC_Os03g25280	9.8555576
LOC_Os02g45540	LOC_Os10g39120	9.71863687

InteractorA	InteractorB	Z score
LOC_Os02g45940	LOC_Os11g10510	8.86156807
LOC_Os02g45940	LOC_Os03g18740	9.04603168
LOC_Os02g46130	LOC_Os03g06620	9.20523416
LOC_Os02g46130	LOC_Os03g17470	9.25367701
LOC_Os02g45940	LOC_Os03g61600	8.88669481
LOC_Os02g46130	LOC_Os06g39875	9.16181654
LOC_Os02g47020	LOC_Os05g04520	9.05927207
LOC_Os02g46970	LOC_Os06g45100	9.93592252
LOC_Os02g45940	LOC_Os07g07709	10.0431544
LOC_Os02g47110	LOC_Os06g45120	9.57792397
LOC_Os02g46130	LOC_Os03g38980	9.50828742
LOC_Os02g46970	LOC_Os04g12690	8.97016046
LOC_Os02g46130	LOC_Os05g33150	9.53523209
LOC_Os02g45940	LOC_Os10g39170	9.48057701
LOC_Os02g47110	LOC_Os11g34450	9.32759543
LOC_Os02g46970	LOC_Os03g01530	9.17111371
LOC_Os02g46970	LOC_Os03g01770	9.29072227
LOC_Os02g46970	LOC_Os11g02100	9.16157556
LOC_Os02g47020	LOC_Os03g16900	10.5706144
LOC_Os02g46970	LOC_Os09g25150	9.38040954
LOC_Os02g46970	LOC_Os04g33480	9.5118431
LOC_Os02g45540	LOC_Os04g05080	9.793816
LOC_Os02g46970	LOC_Os07g34580	11.4139473
LOC_Os02g46970	LOC_Os11g47560	8.84652478
LOC_Os02g47020	LOC_Os03g10940	8.82068896
LOC_Os02g45540	LOC_Os07g42600	9.77206825
LOC_Os02g45940	LOC_Os07g07719	8.80395293
LOC_Os02g45540	LOC_Os07g44790	9.31011355
LOC_Os02g47590	LOC_Os03g04970	9.04876811
LOC_Os02g47110	LOC_Os11g07020	9.05930526
LOC_Os02g45540	LOC_Os04g45490	11.0053007
LOC_Os02g45540	LOC_Os05g09500	9.5125498
LOC_Os02g45540	LOC_Os07g30170	9.02130441
LOC_Os02g46970	LOC_Os10g38229	9.78413896
LOC_Os02g46970	LOC_Os04g45470	9.18451904
LOC_Os02g46970	LOC_Os09g30412	9.22803922
LOC_Os02g46970	LOC_Os12g25630	8.99580096
LOC_Os02g46970	LOC_Os11g39540	9.00256243
LOC_Os02g45940	LOC_Os03g58050	10.0431544
LOC_Os02g46970	LOC_Os04g53800	10.3461597
LOC_Os02g46130	LOC_Os06g51060	9.12228332
LOC_Os02g46970	LOC_Os04g58640	8.80625998
LOC_Os02g46970	LOC_Os03g58050	9.79235619

InteractorA	InteractorB	Z score
LOC_Os02g47020	LOC_Os07g26900	8.82871507
LOC_Os02g46970	LOC_Os08g33820	8.87253971
LOC_Os02g45940	LOC_Os03g51200	9.10305633
LOC_Os02g45540	LOC_Os06g35490	9.24483382
LOC_Os02g45540	LOC_Os07g39290	9.05723925
LOC_Os02g45540	LOC_Os09g39500	9.23741997
LOC_Os02g46970	LOC_Os11g04954	8.97501503
LOC_Os02g46970	LOC_Os03g55389	8.97928263
LOC_Os02g45540	LOC_Os04g32460	9.45040507
LOC_Os02g47790	LOC_Os03g11990	10.2204928
LOC_Os02g47800	LOC_Os10g21248	9.21361165
LOC_Os02g47800	LOC_Os04g41620	9.0334339
LOC_Os02g47790	LOC_Os07g08880	10.8072348
LOC_Os02g47800	LOC_Os05g09440	11.0280935
LOC_Os02g47610	LOC_Os03g04970	9.63260524
LOC_Os02g47610	LOC_Os04g55410	9.32780594
LOC_Os02g47610	LOC_Os04g58880	10.3241534
LOC_Os02g47800	LOC_Os10g39840	9.148228
LOC_Os02g47800	LOC_Os04g59200	9.14221488
LOC_Os02g47840	LOC_Os04g10010	10.4543099
LOC_Os02g47610	LOC_Os09g11230	10.3029027
LOC_Os02g47610	LOC_Os05g11710	9.69026667
LOC_Os02g47600	LOC_Os07g49400	9.1561438
LOC_Os02g47790	LOC_Os07g30990	9.07223687
LOC_Os02g47610	LOC_Os07g26540	8.84937127
LOC_Os02g47590	LOC_Os08g02410	9.93936177
LOC_Os02g47790	LOC_Os06g35560	10.1063199
LOC_Os02g47610	LOC_Os08g28800	11.6051357
LOC_Os02g47600	LOC_Os08g44960	9.91771315
LOC_Os02g47610	LOC_Os04g44870	8.8819467
LOC_Os02g47800	LOC_Os04g33470	9.33832967
LOC_Os02g47800	LOC_Os09g38620	8.88376237
LOC_Os02g47610	LOC_Os03g22530	9.31476132
LOC_Os02g47800	LOC_Os11g04880	9.09324747
LOC_Os02g48290	LOC_Os03g50250	9.09316747
LOC_Os02g47800	LOC_Os06g45590	10.5149391
LOC_Os02g47610	LOC_Os12g05410	10.4075718
LOC_Os02g47800	LOC_Os12g40830	8.84723565
LOC_Os02g47790	LOC_Os06g27770	8.81617281
LOC_Os02g47800	LOC_Os11g24560	9.40644415
LOC_Os02g47800	LOC_Os04g56920	8.86275409
LOC_Os02g47610	LOC_Os05g41060	8.85199521
LOC_Os02g47790	LOC_Os03g45270	9.02663056

InteractorA	InteractorB	Z score
LOC_Os02g47610	LOC_Os06g41810	9.04570019
LOC_Os02g47790	LOC_Os03g37950	9.32081035
LOC_Os02g47800	LOC_Os06g36700	9.87561802
LOC_Os02g47590	LOC_Os08g28800	8.90447028
LOC_Os02g47840	LOC_Os09g10270	11.0559263
LOC_Os02g47610	LOC_Os06g11260	10.3592744
LOC_Os02g47610	LOC_Os09g10270	9.08732344
LOC_Os02g47610	LOC_Os08g39420	9.31314088
LOC_Os02g47610	LOC_Os04g05080	9.41121294
LOC_Os02g47790	LOC_Os03g16980	9.45532754
LOC_Os02g47600	LOC_Os12g41110	10.7836104
LOC_Os02g47800	LOC_Os07g05940	9.9747101
LOC_Os02g47800	LOC_Os06g45670	9.69991189
LOC_Os02g47610	LOC_Os12g10600	11.0244881
LOC_Os02g47600	LOC_Os04g43400	8.93027079
LOC_Os02g47800	LOC_Os06g35520	9.59748958
LOC_Os02g47610	LOC_Os12g23630	9.82808625
LOC_Os02g47610	LOC_Os08g06550	12.1972329
LOC_Os02g47800	LOC_Os11g19800	9.08729213
LOC_Os02g47610	LOC_Os11g25100	9.10293538
LOC_Os02g47800	LOC_Os12g23170	10.1395165
LOC_Os02g47610	LOC_Os02g52390	9.02977985
LOC_Os02g47800	LOC_Os06g07140	9.49523605
LOC_Os02g47800	LOC_Os02g57040	12.0125603
LOC_Os02g47800	LOC_Os06g01390	9.66380975
LOC_Os02g47790	LOC_Os08g37490	8.90857975
LOC_Os02g47790	LOC_Os03g56810	9.54736038
LOC_Os02g47800	LOC_Os05g49830	8.91608476
LOC_Os02g47800	LOC_Os04g12900	11.4458463
LOC_Os02g47590	LOC_Os09g36900	8.83121833
LOC_Os02g47610	LOC_Os10g35480	8.80136154
LOC_Os02g47790	LOC_Os11g37950	8.86533242
LOC_Os02g47800	LOC_Os05g19380	9.71032798
LOC_Os02g47790	LOC_Os11g37550	9.49244404
LOC_Os02g47800	LOC_Os12g39630	8.86598163
LOC_Os02g47790	LOC_Os07g23850	9.60488328
LOC_Os02g47610	LOC_Os06g37660	9.37392018
LOC_Os02g47610	LOC_Os04g56920	10.1077714
LOC_Os02g48290	LOC_Os03g47770	9.66312859
LOC_Os02g47590	LOC_Os05g45590	9.01381015
LOC_Os02g47610	LOC_Os07g44450	10.1936906
LOC_Os02g47840	LOC_Os03g49260	12.4218494
LOC_Os02g47610	LOC_Os02g48290	9.12560682

InteractorA	InteractorB	Z score
LOC_Os02g47610	LOC_Os12g13800	11.4041243
LOC_Os02g47790	LOC_Os09g32810	10.7186262
LOC_Os02g47600	LOC_Os10g08670	8.93700045
LOC_Os02g47800	LOC_Os05g19150	8.94770057
LOC_Os02g47610	LOC_Os03g08530	9.48370655
LOC_Os02g47610	LOC_Os09g07830	8.9035385
LOC_Os02g47840	LOC_Os04g15920	9.25674915
LOC_Os02g47800	LOC_Os06g13450	10.4636949
LOC_Os02g47600	LOC_Os11g14910	8.81184363
LOC_Os02g47610	LOC_Os07g48050	9.0811811
LOC_Os02g47610	LOC_Os03g59710	9.06290444
LOC_Os02g47790	LOC_Os08g32620	8.88590027
LOC_Os02g47800	LOC_Os07g12200	8.97482469
LOC_Os02g47610	LOC_Os03g52970	9.46322863
LOC_Os02g47600	LOC_Os07g23730	8.92569859
LOC_Os02g47610	LOC_Os06g06880	9.95827162
LOC_Os02g48290	LOC_Os03g04060	9.21570877
LOC_Os02g47800	LOC_Os04g17064	9.23364428
LOC_Os02g47790	LOC_Os05g42150	9.21724933
LOC_Os02g47610	LOC_Os03g19930	9.26482084
LOC_Os02g47840	LOC_Os06g48180	9.62010739
LOC_Os02g47610	LOC_Os04g43400	8.94586681
LOC_Os02g47590	LOC_Os11g26850	8.93044643
LOC_Os02g47800	LOC_Os05g02310	9.06696227
LOC_Os02g47600	LOC_Os07g48060	8.82372097
LOC_Os02g48290	LOC_Os04g01740	9.01771611
LOC_Os02g47610	LOC_Os04g28870	9.42772384
LOC_Os02g47800	LOC_Os04g16740	9.17612443
LOC_Os02g47610	LOC_Os06g09910	12.3850789
LOC_Os02g47800	LOC_Os08g06060	8.83626547
LOC_Os02g47800	LOC_Os07g07320	9.42694325
LOC_Os02g47610	LOC_Os07g38030	8.85461286
LOC_Os02g47590	LOC_Os05g33140	9.54559344
LOC_Os02g47610	LOC_Os07g47990	9.09903676
LOC_Os02g47610	LOC_Os09g39810	10.478646
LOC_Os02g47610	LOC_Os06g02490	9.10479204
LOC_Os02g47610	LOC_Os03g07150	9.13694268
LOC_Os02g47610	LOC_Os04g45290	9.20215928
LOC_Os02g47790	LOC_Os08g03040	9.18822325
LOC_Os02g47790	LOC_Os10g23100	9.23758908
LOC_Os02g47590	LOC_Os08g34170	9.55784096
LOC_Os02g47800	LOC_Os03g15360	10.5451305
LOC_Os02g47610	LOC_Os08g33370	9.60690776

InteractorA	InteractorB	Z score
LOC_Os02g47790	LOC_Os03g22010	9.23160057
LOC_Os02g47790	LOC_Os07g44590	8.83006672
LOC_Os02g47790	LOC_Os05g36290	9.78724191
LOC_Os02g47590	LOC_Os09g19560	9.32863283
LOC_Os02g47800	LOC_Os10g38234	10.1990707
LOC_Os02g47800	LOC_Os06g24390	9.09993866
LOC_Os02g47800	LOC_Os05g45590	9.41011686
LOC_Os02g47790	LOC_Os06g04280	8.93226219
LOC_Os02g47610	LOC_Os03g52460	9.1691227
LOC_Os02g47800	LOC_Os10g33800	9.10043485
LOC_Os02g47800	LOC_Os12g42876	9.06695191
LOC_Os02g47790	LOC_Os04g58200	9.10559217
LOC_Os02g47610	LOC_Os03g38020	9.2735495
LOC_Os02g48290	LOC_Os03g12660	8.95343991
LOC_Os02g47610	LOC_Os06g05690	9.14675512
LOC_Os02g47600	LOC_Os09g10230	11.6981904
LOC_Os02g47800	LOC_Os04g47220	9.0964587
LOC_Os02g47610	LOC_Os06g35730	9.69026109
LOC_Os02g47610	LOC_Os07g07550	9.77254449
LOC_Os02g47610	LOC_Os12g04980	10.0922211
LOC_Os02g47800	LOC_Os04g24430	8.92171884
LOC_Os02g47610	LOC_Os08g39870	9.3316735
LOC_Os02g47800	LOC_Os06g35940	8.95551065
LOC_Os02g47790	LOC_Os10g41490	9.20019151
LOC_Os02g47590	LOC_Os07g11440	8.90738964
LOC_Os02g47790	LOC_Os06g11280	9.61087333
LOC_Os02g47610	LOC_Os06g39230	9.66073704
LOC_Os02g47610	LOC_Os10g08620	8.86836175
LOC_Os02g47610	LOC_Os03g56410	9.63893918
LOC_Os02g48290	LOC_Os07g44440	9.27811429
LOC_Os02g49720	LOC_Os04g33740	9.02492315
LOC_Os02g50240	LOC_Os10g27190	9.99936112
LOC_Os02g49720	LOC_Os05g04490	9.15630577
LOC_Os02g48290	LOC_Os07g09890	9.01592479
LOC_Os02g49720	LOC_Os03g63330	9.03005379
LOC_Os02g48290	LOC_Os10g39840	9.31808239
LOC_Os02g49720	LOC_Os06g37180	10.853096
LOC_Os02g50860	LOC_Os03g16980	8.95012975
LOC_Os02g48290	LOC_Os06g51084	9.63712935
LOC_Os02g50240	LOC_Os03g45320	10.3164982
LOC_Os02g49720	LOC_Os08g41990	9.39511944
LOC_Os02g49720	LOC_Os04g33240	8.80513208
LOC_Os02g49980	LOC_Os12g12514	8.83593837

InteractorA	InteractorB	Z score
LOC_Os02g49720	LOC_Os10g37210	8.82877615
LOC_Os02g50240	LOC_Os05g22724	9.73651291
LOC_Os02g49720	LOC_Os05g34540	9.04335031
LOC_Os02g49720	LOC_Os10g26010	8.97467352
LOC_Os02g48290	LOC_Os09g27820	10.6203126
LOC_Os02g49720	LOC_Os10g08670	10.6546503
LOC_Os02g50240	LOC_Os03g18130	9.06126627
LOC_Os02g50860	LOC_Os04g17650	9.1816707
LOC_Os02g50240	LOC_Os05g35290	9.12288869
LOC_Os02g49610	LOC_Os04g33190	10.7365474
LOC_Os02g49720	LOC_Os06g39230	10.6833921
LOC_Os02g50240	LOC_Os09g08910	9.12494597
LOC_Os02g50240	LOC_Os08g25734	9.36826517
LOC_Os02g49610	LOC_Os09g10230	8.96881856
LOC_Os02g48290	LOC_Os05g43510	10.444982
LOC_Os02g49720	LOC_Os10g25140	9.34063771
LOC_Os02g50240	LOC_Os09g10230	9.40474331
LOC_Os02g50240	LOC_Os06g04900	9.55571934
LOC_Os02g49720	LOC_Os05g07880	9.80545379
LOC_Os02g49980	LOC_Os05g23740	9.37406473
LOC_Os02g50860	LOC_Os03g50490	10.8925478
LOC_Os02g49980	LOC_Os12g22680	9.32315225
LOC_Os02g49610	LOC_Os11g08340	9.1593138
LOC_Os02g48290	LOC_Os08g09940	8.95132445
LOC_Os02g48290	LOC_Os11g29190	9.342669
LOC_Os02g50240	LOC_Os07g02340	8.83322194
LOC_Os02g48450	LOC_Os10g39840	9.16237217
LOC_Os02g49720	LOC_Os08g04180	9.97418787
LOC_Os02g49720	LOC_Os09g12660	11.5429885
LOC_Os02g49610	LOC_Os05g01970	9.56201659
LOC_Os02g48290	LOC_Os05g27940	9.06840623
LOC_Os02g49720	LOC_Os08g44960	8.80909724
LOC_Os02g49610	LOC_Os06g23440	9.1781219
LOC_Os02g48290	LOC_Os07g30090	9.67437053
LOC_Os02g49720	LOC_Os06g05690	9.64867927
LOC_Os02g50240	LOC_Os03g58260	10.3748398
LOC_Os02g50240	LOC_Os10g41550	8.80624294
LOC_Os02g49610	LOC_Os08g29170	9.05011829
LOC_Os02g50240	LOC_Os05g22722	8.90161941
LOC_Os02g48290	LOC_Os04g33970	9.25062841
LOC_Os02g49720	LOC_Os04g53230	9.5113209
LOC_Os02g49720	LOC_Os06g06300	9.04674894
LOC_Os02g49720	LOC_Os03g41438	10.1284684

InteractorA	InteractorB	Z score
LOC_Os02g48450	LOC_Os08g20420	9.01377601
LOC_Os02g49610	LOC_Os10g38340	8.95383769
LOC_Os02g50240	LOC_Os03g21460	10.7907287
LOC_Os02g50240	LOC_Os05g12240	8.81426599
LOC_Os02g50240	LOC_Os07g01560	9.53108457
LOC_Os02g50240	LOC_Os06g36840	9.49733649
LOC_Os02g50240	LOC_Os09g09230	9.58441082
LOC_Os02g50860	LOC_Os03g12290	8.87431198
LOC_Os02g49980	LOC_Os06g50300	9.20595466
LOC_Os02g50240	LOC_Os03g56840	9.09575861
LOC_Os02g50240	LOC_Os07g38860	8.96721664
LOC_Os02g50860	LOC_Os04g37490	8.85137164
LOC_Os02g49720	LOC_Os12g14070	8.8841467
LOC_Os02g50860	LOC_Os03g55070	8.83184688
LOC_Os02g48290	LOC_Os09g39570	9.47417105
LOC_Os02g50240	LOC_Os11g03290	9.91175063
LOC_Os02g48450	LOC_Os08g28820	9.66612254
LOC_Os02g49610	LOC_Os04g44924	8.90937344
LOC_Os02g50240	LOC_Os04g16828	8.90161941
LOC_Os02g50240	LOC_Os09g24924	8.83632151
LOC_Os02g49720	LOC_Os05g27940	9.51575479
LOC_Os02g50240	LOC_Os05g49760	9.31384506
LOC_Os02g49720	LOC_Os05g49760	9.00221991
LOC_Os02g50240	LOC_Os08g15276	8.90161941
LOC_Os02g50240	LOC_Os03g31300	9.08834032
LOC_Os02g49720	LOC_Os05g44050	9.32891416
LOC_Os02g50240	LOC_Os09g19560	9.0756011
LOC_Os02g50240	LOC_Os05g34540	9.10808731
LOC_Os02g48290	LOC_Os10g02040	9.27192787
LOC_Os02g49720	LOC_Os07g37550	9.36302109
LOC_Os02g49720	LOC_Os03g50490	9.42701837
LOC_Os02g48290	LOC_Os04g52440	8.88093103
LOC_Os02g48290	LOC_Os06g48180	9.33126437
LOC_Os02g49610	LOC_Os05g04340	8.92403377
LOC_Os02g50240	LOC_Os10g21342	8.90161941
LOC_Os02g51830	LOC_Os08g34210	10.8530615
LOC_Os02g51830	LOC_Os12g38180	9.05768295
LOC_Os02g51830	LOC_Os10g42280	8.83121057
LOC_Os02g51830	LOC_Os03g18810	9.88670648
LOC_Os02g51830	LOC_Os10g23100	9.03698933
LOC_Os02g51100	LOC_Os08g15276	9.08766358
LOC_Os02g51830	LOC_Os10g29470	10.5842809
LOC_Os02g51830	LOC_Os06g45710	9.56044082

InteractorA	InteractorB	Z score
LOC_Os02g51830	LOC_Os03g60620	9.28253919
LOC_Os02g51910	LOC_Os03g10370	8.81009694
LOC_Os02g51930	LOC_Os05g05680	10.7747792
LOC_Os02g50880	LOC_Os06g02144	9.05841496
LOC_Os02g51100	LOC_Os05g04450	10.1230411
LOC_Os02g51830	LOC_Os10g21250	10.2377724
LOC_Os02g50880	LOC_Os03g16900	9.09811303
LOC_Os02g51910	LOC_Os04g52361	9.66415036
LOC_Os02g51100	LOC_Os04g16828	9.08766358
LOC_Os02g51830	LOC_Os03g52970	9.0439398
LOC_Os02g51830	LOC_Os03g02260	9.45475598
LOC_Os02g51100	LOC_Os06g10340	8.87029462
LOC_Os02g51100	LOC_Os06g04270	8.87943594
LOC_Os02g51910	LOC_Os06g49970	9.32421568
LOC_Os02g51100	LOC_Os04g56580	9.70942332
LOC_Os02g50880	LOC_Os03g04169	10.3848284
LOC_Os02g51830	LOC_Os06g03770	9.88747718
LOC_Os02g51930	LOC_Os03g59020	9.47763989
LOC_Os02g51100	LOC_Os03g53230	9.46415788
LOC_Os02g51750	LOC_Os04g58680	9.97436037
LOC_Os02g51830	LOC_Os07g05940	9.2422477
LOC_Os02g51930	LOC_Os02g52420	9.54187357
LOC_Os02g50880	LOC_Os06g35520	9.22352351
LOC_Os02g51930	LOC_Os04g34630	8.89446002
LOC_Os02g51100	LOC_Os04g56920	9.38797518
LOC_Os02g51100	LOC_Os03g64050	10.5628057
LOC_Os02g50860	LOC_Os08g35740	9.43307313
LOC_Os02g50880	LOC_Os10g39840	9.61941723
LOC_Os02g50860	LOC_Os05g35320	8.80298886
LOC_Os02g51100	LOC_Os04g45290	8.98948349
LOC_Os02g50860	LOC_Os11g32650	8.83851653
LOC_Os02g51910	LOC_Os03g50885	9.36983929
LOC_Os02g51830	LOC_Os04g32460	10.4506482
LOC_Os02g51830	LOC_Os07g48040	9.36025045
LOC_Os02g51830	LOC_Os05g36270	9.70164268
LOC_Os02g51100	LOC_Os06g04030	13.7767652
LOC_Os02g51830	LOC_Os10g23900	8.88527931
LOC_Os02g50880	LOC_Os04g37950	8.86887616
LOC_Os02g51830	LOC_Os07g44430	8.93972124
LOC_Os02g50860	LOC_Os07g38540	9.54499851
LOC_Os02g51830	LOC_Os04g54330	9.09230022
LOC_Os02g50880	LOC_Os08g20730	9.82995465
LOC_Os02g51100	LOC_Os11g24560	11.0793709

InteractorA	InteractorB	Z score
LOC_Os02g51830	LOC_Os12g31370	9.22008287
LOC_Os02g50880	LOC_Os03g47610	10.1455889
LOC_Os02g51910	LOC_Os03g58050	11.2822446
LOC_Os02g51100	LOC_Os10g21342	9.08770968
LOC_Os02g51930	LOC_Os05g04450	13.5364228
LOC_Os02g51830	LOC_Os03g05730	9.20331443
LOC_Os02g51830	LOC_Os02g52710	9.21160378
LOC_Os02g51100	LOC_Os05g36270	9.24147436
LOC_Os02g51910	LOC_Os04g33740	9.93437126
LOC_Os02g51930	LOC_Os04g38600	9.00120083
LOC_Os02g51830	LOC_Os03g57290	11.6462305
LOC_Os02g51100	LOC_Os03g17690	9.74851363
LOC_Os02g51910	LOC_Os03g08530	9.44759788
LOC_Os02g51830	LOC_Os06g46940	9.23869418
LOC_Os02g50860	LOC_Os11g05730	8.89202896
LOC_Os02g51100	LOC_Os07g07470	10.4409223
LOC_Os02g51750	LOC_Os05g47540	8.99727007
LOC_Os02g51830	LOC_Os09g08072	8.90564171
LOC_Os02g51750	LOC_Os07g43390	9.53861467
LOC_Os02g50860	LOC_Os12g02370	9.07616175
LOC_Os02g50860	LOC_Os04g58710	9.05045062
LOC_Os02g51830	LOC_Os04g56580	11.916183
LOC_Os02g51830	LOC_Os05g37690	9.39874953
LOC_Os02g51830	LOC_Os04g25990	8.83047005
LOC_Os02g50880	LOC_Os06g35700	10.3442669
LOC_Os02g51910	LOC_Os02g56014	10.3168731
LOC_Os02g51930	LOC_Os02g57720	9.03023472
LOC_Os02g50860	LOC_Os12g23630	8.85942153
LOC_Os02g51930	LOC_Os04g56400	8.96921492
LOC_Os02g51830	LOC_Os03g50440	10.2381233
LOC_Os02g50860	LOC_Os06g06460	8.89202896
LOC_Os02g51830	LOC_Os04g47360	8.89131358
LOC_Os02g51100	LOC_Os06g34690	9.17595755
LOC_Os02g51910	LOC_Os06g07580	10.3168705
LOC_Os02g50880	LOC_Os04g45290	10.5093825
LOC_Os02g51910	LOC_Os04g57400	9.32188647
LOC_Os02g50880	LOC_Os06g36160	9.84266358
LOC_Os02g50880	LOC_Os10g41510	10.115041
LOC_Os02g51830	LOC_Os08g06550	8.82162577
LOC_Os02g51100	LOC_Os05g22722	9.08766358
LOC_Os02g50880	LOC_Os04g37460	9.12114342
LOC_Os02g51100	LOC_Os03g27310	13.776761
LOC_Os02g51830	LOC_Os05g12180	9.57175636

InteractorA	InteractorB	Z score
LOC_Os02g51100	LOC_Os04g40290	9.13036357
LOC_Os02g51100	LOC_Os04g58580	9.26918902
LOC_Os02g51100	LOC_Os04g52100	8.99968031
LOC_Os02g51100	LOC_Os03g16920	9.04674033
LOC_Os02g51930	LOC_Os04g20070	10.2923483
LOC_Os02g50860	LOC_Os09g07510	8.94226455
LOC_Os02g51910	LOC_Os03g42220	9.15002325
LOC_Os02g50880	LOC_Os08g39860	9.40182813
LOC_Os02g51830	LOC_Os05g47890	8.84131487
LOC_Os02g51930	LOC_Os03g61330	9.29319803
LOC_Os02g51830	LOC_Os12g07830	9.95269444
LOC_Os02g51910	LOC_Os06g06090	10.0090344
LOC_Os02g51930	LOC_Os04g43400	8.85034855
LOC_Os02g51930	LOC_Os05g26890	8.90239035
LOC_Os02g51100	LOC_Os06g36670	10.9012006
LOC_Os02g51910	LOC_Os05g31140	9.22894309
LOC_Os02g51930	LOC_Os03g15360	9.51017513
LOC_Os02g50880	LOC_Os03g08530	9.36034951
LOC_Os02g51830	LOC_Os12g16220	9.10513453
LOC_Os02g51910	LOC_Os12g13810	10.3677851
LOC_Os02g50880	LOC_Os07g34520	10.1621812
LOC_Os02g51910	LOC_Os07g07709	11.2822313
LOC_Os02g51830	LOC_Os03g07840	12.0630514
LOC_Os02g51100	LOC_Os12g13800	10.3499348
LOC_Os02g51830	LOC_Os12g25690	9.77782012
LOC_Os02g51830	LOC_Os07g36190	9.33981835
LOC_Os02g51830	LOC_Os12g02980	8.96193885
LOC_Os02g51830	LOC_Os03g51740	9.14898637
LOC_Os02g51830	LOC_Os06g10950	9.54775241
LOC_Os02g51930	LOC_Os05g15520	8.98494749
LOC_Os02g50860	LOC_Os05g45810	8.97509075
LOC_Os02g51930	LOC_Os04g56920	10.1809575
LOC_Os02g51750	LOC_Os09g10270	8.89769949
LOC_Os02g50880	LOC_Os06g43640	8.87842893
LOC_Os02g51830	LOC_Os09g21770	9.26610889
LOC_Os02g51930	LOC_Os05g38550	10.3801487
LOC_Os02g51930	LOC_Os03g55874	9.27048267
LOC_Os02g50860	LOC_Os11g02440	9.7556549
LOC_Os02g51100	LOC_Os05g40420	9.60502647
LOC_Os02g51100	LOC_Os07g03368	9.12079833
LOC_Os02g50860	LOC_Os09g20820	10.4565721
LOC_Os02g51830	LOC_Os02g52700	9.21160378
LOC_Os02g51830	LOC_Os04g39210	8.90448949

InteractorA	InteractorB	Z score
LOC_Os02g51930	LOC_Os05g34770	10.0652851
LOC_Os02g51100	LOC_Os05g47540	9.26725798
LOC_Os02g50860	LOC_Os06g06510	8.89202896
LOC_Os02g50880	LOC_Os03g49220	9.86167711
LOC_Os02g50880	LOC_Os03g06330	9.09088095
LOC_Os02g51100	LOC_Os11g25330	9.28395268
LOC_Os02g51910	LOC_Os07g48880	8.99936418
LOC_Os02g50880	LOC_Os03g04410	8.87759165
LOC_Os02g51930	LOC_Os04g44470	9.99343706
LOC_Os02g50860	LOC_Os05g36280	8.89202896
LOC_Os02g51100	LOC_Os03g18560	8.9959179
LOC_Os02g50880	LOC_Os12g14070	9.06507743
LOC_Os02g50880	LOC_Os12g38760	8.96089962
LOC_Os02g51100	LOC_Os08g09770	9.99884225
LOC_Os02g52230	LOC_Os02g56690	8.83906303
LOC_Os02g52230	LOC_Os05g40990	10.0341877
LOC_Os02g52230	LOC_Os09g10200	10.214097
LOC_Os02g52390	LOC_Os10g17680	9.08450847
LOC_Os02g52420	LOC_Os11g04880	9.93465977
LOC_Os02g52420	LOC_Os11g03230	9.45803592
LOC_Os02g52230	LOC_Os04g33040	9.09634012
LOC_Os02g51930	LOC_Os11g10480	9.77444162
LOC_Os02g52230	LOC_Os11g31530	8.84839097
LOC_Os02g52420	LOC_Os03g55070	9.94710376
LOC_Os02g52560	LOC_Os03g58320	9.40785929
LOC_Os02g52230	LOC_Os04g57380	9.21098217
LOC_Os02g52390	LOC_Os06g21980	9.11886373
LOC_Os02g52420	LOC_Os02g57040	9.12867609
LOC_Os02g52560	LOC_Os05g40420	9.08623252
LOC_Os02g52390	LOC_Os08g14760	10.3038067
LOC_Os02g52230	LOC_Os03g45960	9.49337136
LOC_Os02g52390	LOC_Os04g44730	8.8493042
LOC_Os02g52390	LOC_Os08g40740	9.56581904
LOC_Os02g52430	LOC_Os12g13390	8.80549308
LOC_Os02g52420	LOC_Os03g32050	9.55024811
LOC_Os02g52420	LOC_Os08g25570	9.39228209
LOC_Os02g52230	LOC_Os11g08330	8.84476594
LOC_Os02g52230	LOC_Os10g08022	8.84000097
LOC_Os02g52420	LOC_Os09g33500	8.8904784
LOC_Os02g52230	LOC_Os11g26910	11.6912401
LOC_Os02g52430	LOC_Os04g09604	9.17645825
LOC_Os02g52420	LOC_Os12g05590	9.21381207
LOC_Os02g52390	LOC_Os03g15880	8.86272127

InteractorA	InteractorB	Z score
LOC_Os02g51930	LOC_Os11g28340	9.4451676
LOC_Os02g52390	LOC_Os05g47640	10.591058
LOC_Os02g51930	LOC_Os06g06320	8.80866192
LOC_Os02g52230	LOC_Os11g37960	9.57869313
LOC_Os02g52430	LOC_Os05g07090	8.91209444
LOC_Os02g52420	LOC_Os08g44340	9.4430244
LOC_Os02g52430	LOC_Os09g37100	9.57725638
LOC_Os02g51930	LOC_Os08g37800	10.1542307
LOC_Os02g52230	LOC_Os06g11290	10.1066199
LOC_Os02g52230	LOC_Os09g24530	8.90754577
LOC_Os02g52420	LOC_Os12g43450	10.6992577
LOC_Os02g52390	LOC_Os07g36130	8.99751938
LOC_Os02g52390	LOC_Os02g56100	9.55632316
LOC_Os02g52390	LOC_Os07g48020	9.25310134
LOC_Os02g52560	LOC_Os04g41960	9.64373655
LOC_Os02g52420	LOC_Os06g35650	9.24670828
LOC_Os02g51930	LOC_Os08g31870	11.0578311
LOC_Os02g52230	LOC_Os04g39210	9.02100366
LOC_Os02g52230	LOC_Os04g48060	8.9516519
LOC_Os02g52230	LOC_Os04g36700	10.8346083
LOC_Os02g52420	LOC_Os12g34062	9.5253986
LOC_Os02g52420	LOC_Os03g64210	9.36206829
LOC_Os02g52390	LOC_Os10g37060	10.3122747
LOC_Os02g52230	LOC_Os06g36770	9.01961652
LOC_Os02g52230	LOC_Os05g33380	11.0727246
LOC_Os02g52230	LOC_Os09g20090	9.14820745
LOC_Os02g52430	LOC_Os07g08170	9.10719051
LOC_Os02g52230	LOC_Os06g05250	9.25907748
LOC_Os02g52230	LOC_Os10g40090	8.96361457
LOC_Os02g52390	LOC_Os06g41810	10.0892281
LOC_Os02g52560	LOC_Os05g44140	9.15942463
LOC_Os02g52390	LOC_Os07g30200	8.91376715
LOC_Os02g52390	LOC_Os03g53650	10.4021523
LOC_Os02g52420	LOC_Os04g56210	8.91156823
LOC_Os02g52230	LOC_Os03g26450	9.34941401
LOC_Os02g52390	LOC_Os04g24520	9.63470165
LOC_Os02g52420	LOC_Os06g12790	9.7898496
LOC_Os02g52420	LOC_Os07g19040	9.33923965
LOC_Os02g52420	LOC_Os05g01810	9.20731822
LOC_Os02g52230	LOC_Os06g01590	9.55035443
LOC_Os02g52420	LOC_Os03g40720	8.99903739
LOC_Os02g52390	LOC_Os03g17480	10.2880288
LOC_Os02g52230	LOC_Os05g05670	8.80541425

InteractorA	InteractorB	Z score
LOC_Os02g52230	LOC_Os05g41610	9.74748872
LOC_Os02g52390	LOC_Os03g59660	9.0205001
LOC_Os02g52560	LOC_Os03g59060	9.38576524
LOC_Os02g52560	LOC_Os06g04200	9.05758579
LOC_Os02g52230	LOC_Os05g37390	9.56466233
LOC_Os02g52230	LOC_Os06g39140	9.46836091
LOC_Os02g52430	LOC_Os07g31770	9.79342224
LOC_Os02g52560	LOC_Os04g16760	11.919849
LOC_Os02g51930	LOC_Os12g13380	9.95147962
LOC_Os02g52560	LOC_Os03g62070	9.4989805
LOC_Os02g52420	LOC_Os03g45410	9.28382173
LOC_Os02g52420	LOC_Os09g08120	9.14498851
LOC_Os02g51930	LOC_Os06g35490	9.62211057
LOC_Os02g52230	LOC_Os11g47560	9.29051488
LOC_Os02g52230	LOC_Os07g40290	9.04374505
LOC_Os02g52230	LOC_Os05g04690	9.27965109
LOC_Os02g51930	LOC_Os05g49200	9.15045744
LOC_Os02g52390	LOC_Os10g17660	9.08450847
LOC_Os02g52390	LOC_Os12g22650	9.62403618
LOC_Os02g52430	LOC_Os05g08960	8.9677549
LOC_Os02g52230	LOC_Os03g04250	10.8804725
LOC_Os02g52420	LOC_Os06g35660	11.2780173
LOC_Os02g51930	LOC_Os07g40290	9.19805626
LOC_Os02g52390	LOC_Os08g34170	9.32392476
LOC_Os02g52230	LOC_Os03g16210	9.46611929
LOC_Os02g52560	LOC_Os04g58640	9.12098983
LOC_Os02g52560	LOC_Os05g01675	11.919849
LOC_Os02g52230	LOC_Os03g37950	8.80701741
LOC_Os02g52230	LOC_Os07g12200	10.2892963
LOC_Os02g52420	LOC_Os11g42350	9.35413846
LOC_Os02g51930	LOC_Os06g40180	9.31595514
LOC_Os02g52390	LOC_Os07g31750	9.36207288
LOC_Os02g52230	LOC_Os05g46860	9.63629714
LOC_Os02g52420	LOC_Os07g49220	9.93290045
LOC_Os02g52560	LOC_Os04g42920	9.01205117
LOC_Os02g51930	LOC_Os06g44620	9.03522728
LOC_Os02g52420	LOC_Os06g03770	9.84337309
LOC_Os02g52230	LOC_Os06g40180	9.58896698
LOC_Os02g52420	LOC_Os06g40940	9.20821573
LOC_Os02g52560	LOC_Os05g27950	8.81021793
LOC_Os02g52230	LOC_Os04g52280	9.05283258
LOC_Os02g51930	LOC_Os11g24560	10.1289619
LOC_Os02g52390	LOC_Os10g26130	8.81309566

InteractorA	InteractorB	Z score
LOC_Os02g52420	LOC_Os03g10620	9.4747024
LOC_Os02g52420	LOC_Os03g48471	9.36417024
LOC_Os02g52560	LOC_Os05g45590	9.11620781
LOC_Os02g51930	LOC_Os10g07616	9.62741116
LOC_Os02g52390	LOC_Os04g52100	10.2345686
LOC_Os02g52230	LOC_Os10g07616	10.3958092
LOC_Os02g52420	LOC_Os11g06390	9.58107903
LOC_Os02g52420	LOC_Os12g13380	8.96122122
LOC_Os02g52230	LOC_Os04g59150	9.21584556
LOC_Os02g52230	LOC_Os03g10120	9.46216324
LOC_Os02g52560	LOC_Os04g55290	9.42140706
LOC_Os02g52420	LOC_Os10g39680	9.97385137
LOC_Os02g52430	LOC_Os03g58530	9.24031525
LOC_Os02g52230	LOC_Os03g49260	8.86290625
LOC_Os02g52420	LOC_Os09g39570	9.43806867
LOC_Os02g52420	LOC_Os11g31530	9.55800148
LOC_Os02g52230	LOC_Os11g19800	9.80475785
LOC_Os02g52430	LOC_Os08g33710	8.85588419
LOC_Os02g52420	LOC_Os05g33150	9.52055427
LOC_Os02g52700	LOC_Os07g48060	9.17042099
LOC_Os02g52590	LOC_Os10g37060	10.9800923
LOC_Os02g52640	LOC_Os03g08530	9.13880416
LOC_Os02g52710	LOC_Os05g35400	9.19601052
LOC_Os02g52630	LOC_Os09g08072	9.14209153
LOC_Os02g52560	LOC_Os08g28820	12.9792751
LOC_Os02g52710	LOC_Os07g48060	9.17035332
LOC_Os02g52710	LOC_Os04g59150	10.3191396
LOC_Os02g52560	LOC_Os07g07060	8.88426073
LOC_Os02g52590	LOC_Os03g10340	9.4681398
LOC_Os02g52560	LOC_Os12g17910	9.09078836
LOC_Os02g52630	LOC_Os09g39440	8.97674877
LOC_Os02g52610	LOC_Os07g27790	11.4965414
LOC_Os02g52700	LOC_Os06g48200	10.2334804
LOC_Os02g52700	LOC_Os10g41490	9.83404458
LOC_Os02g52610	LOC_Os06g14620	9.09073638
LOC_Os02g52610	LOC_Os04g28180	9.89663802
LOC_Os02g52710	LOC_Os06g37150	11.0422234
LOC_Os02g52700	LOC_Os03g57140	9.36521708
LOC_Os02g52700	LOC_Os07g37550	8.95645203
LOC_Os02g52560	LOC_Os08g34210	10.8855808
LOC_Os02g52710	LOC_Os07g06410	9.69767611
LOC_Os02g52640	LOC_Os11g37550	8.96132131
LOC_Os02g52710	LOC_Os04g46560	10.1055854

InteractorA	InteractorB	Z score
LOC_Os02g52560	LOC_Os12g07820	8.83993404
LOC_Os02g52590	LOC_Os09g26380	11.3031211
LOC_Os02g52700	LOC_Os03g05730	9.95847323
LOC_Os02g52560	LOC_Os10g38730	10.5086293
LOC_Os02g52700	LOC_Os10g38340	9.55105061
LOC_Os02g52640	LOC_Os11g47760	8.93658225
LOC_Os02g52610	LOC_Os03g50480	8.91183575
LOC_Os02g52700	LOC_Os06g42130	9.57803619
LOC_Os02g52700	LOC_Os10g42280	8.99698703
LOC_Os02g52610	LOC_Os06g11260	8.96707885
LOC_Os02g52700	LOC_Os12g08280	10.2921806
LOC_Os02g52590	LOC_Os05g44140	8.86260753
LOC_Os02g52700	LOC_Os10g20910	9.01319439
LOC_Os02g52630	LOC_Os03g11960	9.00331441
LOC_Os02g52710	LOC_Os06g37080	9.2465267
LOC_Os02g52700	LOC_Os05g35400	9.19606454
LOC_Os02g52700	LOC_Os08g34210	9.42404001
LOC_Os02g52700	LOC_Os04g59150	10.3191341
LOC_Os02g52560	LOC_Os09g23550	9.55216512
LOC_Os02g52710	LOC_Os03g56280	9.22289378
LOC_Os02g52610	LOC_Os03g21260	9.54057897
LOC_Os02g52710	LOC_Os06g48200	10.2334507
LOC_Os02g52640	LOC_Os06g51084	8.9899239
LOC_Os02g52560	LOC_Os07g43390	9.29118659
LOC_Os02g52640	LOC_Os08g35740	9.03412673
LOC_Os02g52590	LOC_Os10g42280	8.86038569
LOC_Os02g52700	LOC_Os03g56280	9.22281212
LOC_Os02g52630	LOC_Os05g33130	9.08440495
LOC_Os02g52630	LOC_Os03g64210	9.65077465
LOC_Os02g52700	LOC_Os08g32620	9.39226682
LOC_Os02g52640	LOC_Os12g08270	8.95007809
LOC_Os02g52710	LOC_Os04g52130	9.87270033
LOC_Os02g52590	LOC_Os03g10500	10.170226
LOC_Os02g52590	LOC_Os05g41210	10.893845
LOC_Os02g52640	LOC_Os06g36670	8.85007566
LOC_Os02g52560	LOC_Os12g33610	9.96752523
LOC_Os02g52710	LOC_Os06g42130	9.57803619
LOC_Os02g52710	LOC_Os03g50480	9.94759332
LOC_Os02g52710	LOC_Os03g57140	9.36537634
LOC_Os02g52590	LOC_Os03g41438	11.9607061
LOC_Os02g52710	LOC_Os05g49770	10.8198196
LOC_Os02g52640	LOC_Os08g25734	8.83355229
LOC_Os02g52640	LOC_Os11g19800	9.15959617

InteractorA	InteractorB	Z score
LOC_Os02g52560	LOC_Os10g42720	9.14625581
LOC_Os02g52610	LOC_Os04g53290	9.54660108
LOC_Os02g52700	LOC_Os06g37150	11.0422234
LOC_Os02g52700	LOC_Os04g24520	8.98173235
LOC_Os02g52640	LOC_Os05g46360	8.86092801
LOC_Os02g52590	LOC_Os07g34520	9.07012112
LOC_Os02g52630	LOC_Os08g14770	9.45545173
LOC_Os02g52700	LOC_Os07g06410	9.69767611
LOC_Os02g52630	LOC_Os03g01170	9.6485484
LOC_Os02g52640	LOC_Os06g35540	9.55861232
LOC_Os02g52640	LOC_Os03g60620	9.95402904
LOC_Os02g52630	LOC_Os08g29370	9.4727384
LOC_Os02g52710	LOC_Os03g05730	9.95847323
LOC_Os02g52590	LOC_Os07g48020	9.29737373
LOC_Os02g52700	LOC_Os03g50480	9.9475723
LOC_Os02g52610	LOC_Os11g33240	9.45664634
LOC_Os02g52630	LOC_Os04g12690	9.31127199
LOC_Os02g52700	LOC_Os04g52130	9.87270033
LOC_Os02g52590	LOC_Os03g17690	8.84426431
LOC_Os02g52700	LOC_Os03g45320	10.757997
LOC_Os02g52710	LOC_Os04g24520	8.98172877
LOC_Os02g52560	LOC_Os08g25734	9.13902566
LOC_Os02g52590	LOC_Os04g41310	9.50047115
LOC_Os02g52700	LOC_Os08g28190	9.96168196
LOC_Os02g52610	LOC_Os12g05410	8.9227672
LOC_Os02g52590	LOC_Os06g02490	9.02625711
LOC_Os02g52640	LOC_Os08g15292	10.3008423
LOC_Os02g52610	LOC_Os02g56100	9.66501551
LOC_Os02g52710	LOC_Os04g58710	10.4667357
LOC_Os02g52560	LOC_Os11g43360	9.74067748
LOC_Os02g52700	LOC_Os12g16200	9.08247724
LOC_Os02g52700	LOC_Os04g58710	10.4667431
LOC_Os02g52590	LOC_Os11g01872	8.99152943
LOC_Os02g52590	LOC_Os08g42910	8.98270877
LOC_Os02g52700	LOC_Os06g37080	9.2465267
LOC_Os02g52700	LOC_Os12g43630	10.0073031
LOC_Os02g52700	LOC_Os05g49770	10.8198196
LOC_Os02g52640	LOC_Os08g31870	10.6029116
LOC_Os02g52560	LOC_Os07g44440	8.92893918
LOC_Os02g52710	LOC_Os03g45320	10.758035
LOC_Os02g52710	LOC_Os07g37550	8.95645278
LOC_Os02g52610	LOC_Os05g25490	9.14552719
LOC_Os02g52700	LOC_Os04g46560	10.1055854

InteractorA	InteractorB	Z score
LOC_Os02g52700	LOC_Os09g07830	9.18133952
LOC_Os02g52590	LOC_Os03g21260	9.5143287
LOC_Os02g52610	LOC_Os06g05090	9.04991009
LOC_Os02g52560	LOC_Os07g43170	8.86412253
LOC_Os02g52610	LOC_Os03g49580	8.94521559
LOC_Os02g52590	LOC_Os03g49600	8.82228767
LOC_Os02g52610	LOC_Os03g62500	9.45335121
LOC_Os02g53790	LOC_Os06g11210	10.168459
LOC_Os02g52800	LOC_Os04g58200	9.38022862
LOC_Os02g55140	LOC_Os05g49760	8.93729626
LOC_Os02g53180	LOC_Os04g16680	9.50711808
LOC_Os02g52800	LOC_Os11g34450	9.29806931
LOC_Os02g53790	LOC_Os05g05620	9.94051326
LOC_Os02g52710	LOC_Os08g28190	9.96166793
LOC_Os02g52800	LOC_Os07g31770	9.03525313
LOC_Os02g53180	LOC_Os12g17910	9.43254321
LOC_Os02g52710	LOC_Os10g38340	9.55110747
LOC_Os02g52800	LOC_Os05g41230	8.96713397
LOC_Os02g53790	LOC_Os08g35420	9.00512621
LOC_Os02g55140	LOC_Os08g06100	9.01049338
LOC_Os02g53180	LOC_Os03g17980	9.04898974
LOC_Os02g55140	LOC_Os07g19040	9.01878981
LOC_Os02g52800	LOC_Os07g36190	9.49125187
LOC_Os02g53180	LOC_Os03g16920	8.92211655
LOC_Os02g53420	LOC_Os07g34260	8.94083607
LOC_Os02g53180	LOC_Os03g50290	9.31605393
LOC_Os02g55140	LOC_Os03g17470	8.81097126
LOC_Os02g53790	LOC_Os04g16770	9.00512621
LOC_Os02g53790	LOC_Os05g04450	8.82163138
LOC_Os02g53420	LOC_Os04g16740	10.2344419
LOC_Os02g52800	LOC_Os03g22530	9.31796252
LOC_Os02g52800	LOC_Os12g33610	9.12201513
LOC_Os02g52710	LOC_Os08g32620	9.39226682
LOC_Os02g55140	LOC_Os05g41210	9.14942084
LOC_Os02g53790	LOC_Os10g40090	9.18454166
LOC_Os02g53420	LOC_Os12g16240	8.87215929
LOC_Os02g52710	LOC_Os10g42280	8.99698703
LOC_Os02g53180	LOC_Os09g39810	9.1539664
LOC_Os02g55140	LOC_Os04g18200	9.0539884
LOC_Os02g52800	LOC_Os03g56280	8.9959804
LOC_Os02g53180	LOC_Os09g15400	9.70634538
LOC_Os02g53420	LOC_Os09g10300	9.69281984
LOC_Os02g53180	LOC_Os11g19800	9.33260215

InteractorA	InteractorB	Z score
LOC_Os02g55140	LOC_Os03g16740	9.1887746
LOC_Os02g53790	LOC_Os07g25150	9.377485
LOC_Os02g55140	LOC_Os04g14680	9.33759512
LOC_Os02g53420	LOC_Os03g18740	10.7734027
LOC_Os02g53420	LOC_Os07g47490	9.02374928
LOC_Os02g52800	LOC_Os08g14570	10.4125734
LOC_Os02g55140	LOC_Os09g34214	9.43526683
LOC_Os02g52710	LOC_Os12g43630	10.0073031
LOC_Os02g52800	LOC_Os05g25550	10.2012936
LOC_Os02g55140	LOC_Os04g55960	10.330959
LOC_Os02g53790	LOC_Os03g58260	8.90902643
LOC_Os02g53420	LOC_Os10g31940	9.22842981
LOC_Os02g53790	LOC_Os07g35940	10.2518672
LOC_Os02g52710	LOC_Os08g34210	9.42401974
LOC_Os02g53420	LOC_Os09g24990	9.22612991
LOC_Os02g52800	LOC_Os05g50890	9.01382218
LOC_Os02g53790	LOC_Os04g12950	9.02732989
LOC_Os02g53420	LOC_Os06g35540	9.43438522
LOC_Os02g53790	LOC_Os06g13450	9.77148227
LOC_Os02g53790	LOC_Os08g34280	8.90700826
LOC_Os02g53790	LOC_Os07g22930	9.00338399
LOC_Os02g52800	LOC_Os12g06620	10.0497468
LOC_Os02g53180	LOC_Os12g10720	9.0601982
LOC_Os02g53420	LOC_Os04g44920	9.17625524
LOC_Os02g55140	LOC_Os03g20710	10.6179607
LOC_Os02g53180	LOC_Os04g16874	9.11292316
LOC_Os02g53420	LOC_Os05g01490	8.8571404
LOC_Os02g55140	LOC_Os03g53860	9.57041455
LOC_Os02g53420	LOC_Os10g20910	8.81877397
LOC_Os02g53420	LOC_Os12g31370	9.36048798
LOC_Os02g52800	LOC_Os07g46830	9.15556806
LOC_Os02g55140	LOC_Os04g02820	9.22415746
LOC_Os02g55140	LOC_Os10g11140	8.80501967
LOC_Os02g53790	LOC_Os03g01640	9.31663096
LOC_Os02g53420	LOC_Os05g01600	9.36419015
LOC_Os02g55140	LOC_Os07g02340	9.18468732
LOC_Os02g53420	LOC_Os07g30170	9.42396602
LOC_Os02g52710	LOC_Os12g08280	10.2921806
LOC_Os02g53790	LOC_Os03g62670	9.03993135
LOC_Os02g52800	LOC_Os04g48060	9.31305226
LOC_Os02g52800	LOC_Os03g21950	9.13825483
LOC_Os02g52710	LOC_Os10g20910	9.01319439
LOC_Os02g55140	LOC_Os07g22650	9.04503041

InteractorA	InteractorB	Z score
LOC_Os02g53420	LOC_Os04g57400	10.568198
LOC_Os02g53790	LOC_Os09g38030	11.0575184
LOC_Os02g53790	LOC_Os06g35560	9.17851156
LOC_Os02g52800	LOC_Os07g05400	9.75622237
LOC_Os02g55140	LOC_Os03g28400	9.33174954
LOC_Os02g53180	LOC_Os03g08800	9.4166753
LOC_Os02g55140	LOC_Os03g62500	9.17792995
LOC_Os02g53180	LOC_Os05g12180	8.98954256
LOC_Os02g53180	LOC_Os04g37490	8.87085917
LOC_Os02g53180	LOC_Os09g30412	9.06196973
LOC_Os02g52800	LOC_Os06g15990	9.27154275
LOC_Os02g53790	LOC_Os12g22680	9.42529203
LOC_Os02g52710	LOC_Os09g07830	9.18133952
LOC_Os02g53420	LOC_Os05g40420	9.12714819
LOC_Os02g53420	LOC_Os03g02920	9.16776896
LOC_Os02g53420	LOC_Os04g39210	9.98578472
LOC_Os02g55140	LOC_Os03g01800	9.06788769
LOC_Os02g53420	LOC_Os06g36700	8.88867025
LOC_Os02g52710	LOC_Os12g16200	9.08242114
LOC_Os02g53420	LOC_Os07g07550	9.19230594
LOC_Os02g53790	LOC_Os03g10940	9.37482176
LOC_Os02g52800	LOC_Os09g36710	8.92014408
LOC_Os02g53420	LOC_Os09g24530	8.95388425
LOC_Os02g53420	LOC_Os03g26450	8.87731224
LOC_Os02g52800	LOC_Os09g08880	9.75506387
LOC_Os02g52800	LOC_Os05g06480	8.9383668
LOC_Os02g53420	LOC_Os07g38910	9.16713967
LOC_Os02g53420	LOC_Os07g16970	8.81999451
LOC_Os02g52800	LOC_Os09g24990	9.56773221
LOC_Os02g52710	LOC_Os10g41490	9.83423487
LOC_Os02g53790	LOC_Os05g34170	9.10056234
LOC_Os02g55140	LOC_Os07g36130	9.92125674
LOC_Os02g53180	LOC_Os09g09230	9.58668259
LOC_Os02g53790	LOC_Os09g12230	8.97046238
LOC_Os02g55140	LOC_Os03g31210	9.66272113
LOC_Os02g53180	LOC_Os08g38300	8.98317253
LOC_Os02g55140	LOC_Os07g44790	9.33174954
LOC_Os02g53420	LOC_Os03g50290	9.2128119
LOC_Os02g53790	LOC_Os12g22650	8.88119802
LOC_Os02g55140	LOC_Os10g26010	9.54025168
LOC_Os02g53790	LOC_Os10g28080	10.6014833
LOC_Os02g55420	LOC_Os09g24924	9.36257576
LOC_Os02g55400	LOC_Os04g24430	11.0659932

InteractorA	InteractorB	Z score
LOC_Os02g55890	LOC_Os06g37660	9.85835721
LOC_Os02g55400	LOC_Os02g56130	9.06130106
LOC_Os02g56014	LOC_Os09g08072	9.24315614
LOC_Os02g55370	LOC_Os04g16826	9.16387694
LOC_Os02g55420	LOC_Os10g38540	9.17412786
LOC_Os02g56014	LOC_Os04g39880	9.58060807
LOC_Os02g55420	LOC_Os04g48850	11.088177
LOC_Os02g55420	LOC_Os03g14530	8.82719493
LOC_Os02g55300	LOC_Os07g29750	8.9744056
LOC_Os02g55400	LOC_Os04g53920	8.98753321
LOC_Os02g55890	LOC_Os04g39880	9.55152042
LOC_Os02g55140	LOC_Os12g25700	11.1617896
LOC_Os02g55420	LOC_Os04g04230	9.64516184
LOC_Os02g55300	LOC_Os12g08270	10.1641494
LOC_Os02g55420	LOC_Os08g05910	9.24852736
LOC_Os02g55890	LOC_Os10g07040	9.04064349
LOC_Os02g55300	LOC_Os03g60620	9.4129198
LOC_Os02g55420	LOC_Os07g02350	10.215947
LOC_Os02g55400	LOC_Os04g33720	8.92749064
LOC_Os02g55890	LOC_Os05g50890	9.38063466
LOC_Os02g55890	LOC_Os12g01922	9.32983237
LOC_Os02g55890	LOC_Os04g09604	8.96639293
LOC_Os02g55370	LOC_Os10g42280	8.87486093
LOC_Os02g55400	LOC_Os09g28420	9.16138851
LOC_Os02g55420	LOC_Os06g39708	8.87039593
LOC_Os02g55370	LOC_Os10g38600	9.20054405
LOC_Os02g55300	LOC_Os05g43510	8.96844819
LOC_Os02g55400	LOC_Os12g17910	11.8679652
LOC_Os02g55420	LOC_Os10g38489	10.6842167
LOC_Os02g55300	LOC_Os12g07980	8.98648948
LOC_Os02g55370	LOC_Os04g18650	9.05672566
LOC_Os02g55300	LOC_Os06g37660	8.96493347
LOC_Os02g55420	LOC_Os06g45670	9.24126349
LOC_Os02g55400	LOC_Os09g23560	9.47344952
LOC_Os02g55300	LOC_Os08g35740	9.45956316
LOC_Os02g56014	LOC_Os05g09440	8.81291905
LOC_Os02g55400	LOC_Os04g53240	8.91864211
LOC_Os02g55300	LOC_Os07g05800	8.84528869
LOC_Os02g55890	LOC_Os03g51740	9.33605302
LOC_Os02g55420	LOC_Os03g16050	9.22633586
LOC_Os02g55370	LOC_Os10g28320	9.46935006
LOC_Os02g55300	LOC_Os08g37800	9.16022233
LOC_Os02g55420	LOC_Os10g38660	10.1062879

InteractorA	InteractorB	Z score
LOC_Os02g55300	LOC_Os10g38470	9.4550814
LOC_Os02g55300	LOC_Os04g53290	9.53541862
LOC_Os02g55890	LOC_Os05g06480	10.8409059
LOC_Os02g56014	LOC_Os09g32840	9.42595607
LOC_Os02g55890	LOC_Os07g07550	9.6275048
LOC_Os02g55890	LOC_Os04g55960	8.8282469
LOC_Os02g55400	LOC_Os08g35740	10.9984409
LOC_Os02g55300	LOC_Os06g33210	9.26040128
LOC_Os02g55300	LOC_Os04g36700	9.22790934
LOC_Os02g56014	LOC_Os12g05410	8.81617052
LOC_Os02g55370	LOC_Os07g46630	8.81309826
LOC_Os02g55370	LOC_Os04g59200	8.94340004
LOC_Os02g55400	LOC_Os09g23530	10.7682476
LOC_Os02g55370	LOC_Os05g04450	9.94627371
LOC_Os02g55370	LOC_Os07g47420	9.54462508
LOC_Os02g55420	LOC_Os10g14150	9.53303817
LOC_Os02g55370	LOC_Os05g37700	9.02608182
LOC_Os02g55890	LOC_Os06g08080	10.0856082
LOC_Os02g55300	LOC_Os12g01922	9.82365421
LOC_Os02g55400	LOC_Os04g55410	10.091806
LOC_Os02g55420	LOC_Os03g04169	9.23679801
LOC_Os02g55300	LOC_Os06g09450	8.90529838
LOC_Os02g55400	LOC_Os07g07060	9.63464881
LOC_Os02g55890	LOC_Os05g48040	9.06638251
LOC_Os02g55420	LOC_Os07g48430	9.26541754
LOC_Os02g55400	LOC_Os12g43440	8.88624905
LOC_Os02g55890	LOC_Os10g01570	9.80040082
LOC_Os02g55400	LOC_Os05g33150	9.2307029
LOC_Os02g55420	LOC_Os06g45100	9.09766401
LOC_Os02g55400	LOC_Os03g61920	9.09114345
LOC_Os02g55300	LOC_Os06g39140	9.99758569
LOC_Os02g55400	LOC_Os04g01740	9.35625767
LOC_Os02g55420	LOC_Os10g31950	10.1445409
LOC_Os02g55370	LOC_Os10g21344	9.16387694
LOC_Os02g55300	LOC_Os04g31960	8.86469033
LOC_Os02g55420	LOC_Os12g38770	9.33430063
LOC_Os02g56014	LOC_Os06g49470	8.80687157
LOC_Os02g55300	LOC_Os08g40740	8.93868469
LOC_Os02g55420	LOC_Os06g39140	8.91062076
LOC_Os02g55370	LOC_Os03g51600	8.87063574
LOC_Os02g55400	LOC_Os04g33470	9.47771439
LOC_Os02g56014	LOC_Os09g28420	8.86385068
LOC_Os02g56100	LOC_Os11g30290	8.80320428

InteractorA	InteractorB	Z score
LOC_Os02g56180	LOC_Os03g13160	9.08311127
LOC_Os02g56130	LOC_Os07g34140	9.27999661
LOC_Os02g56690	LOC_Os12g41110	10.1825479
LOC_Os02g56180	LOC_Os08g44370	9.2008283
LOC_Os02g56680	LOC_Os11g32260	10.9005151
LOC_Os02g56180	LOC_Os05g44340	9.02564213
LOC_Os02g56100	LOC_Os06g07210	9.11274553
LOC_Os02g56700	LOC_Os04g17064	9.00786018
LOC_Os02g56180	LOC_Os09g20090	9.96281698
LOC_Os02g56460	LOC_Os04g30800	9.05353911
LOC_Os02g56130	LOC_Os05g42190	11.3032015
LOC_Os02g56460	LOC_Os10g38234	9.05155266
LOC_Os02g56690	LOC_Os11g16590	8.93611517
LOC_Os02g56680	LOC_Os05g04690	9.28534298
LOC_Os02g56130	LOC_Os07g05180	8.80289405
LOC_Os02g56690	LOC_Os12g02980	8.92100029
LOC_Os02g56460	LOC_Os04g39840	9.40615911
LOC_Os02g56460	LOC_Os08g32620	9.38867257
LOC_Os02g56460	LOC_Os08g41340	9.40775157
LOC_Os02g56460	LOC_Os05g39050	10.706
LOC_Os02g56180	LOC_Os08g42560	9.50821238
LOC_Os02g56180	LOC_Os07g13980	9.60444577
LOC_Os02g56700	LOC_Os03g15360	10.8269358
LOC_Os02g56180	LOC_Os07g42490	10.0738139
LOC_Os02g56700	LOC_Os04g18650	10.338475
LOC_Os02g56180	LOC_Os03g16860	8.90753254
LOC_Os02g56460	LOC_Os03g02780	10.7059953
LOC_Os02g56700	LOC_Os03g62060	9.30693044
LOC_Os02g56180	LOC_Os05g28180	9.51780814
LOC_Os02g56180	LOC_Os03g63090	9.00565254
LOC_Os02g56460	LOC_Os10g38540	8.91200798
LOC_Os02g56690	LOC_Os10g21230	9.00569819
LOC_Os02g56130	LOC_Os04g33740	8.99922176
LOC_Os02g56460	LOC_Os03g04410	8.9813828
LOC_Os02g56460	LOC_Os06g36770	10.9455531
LOC_Os02g56460	LOC_Os12g06620	8.88269848
LOC_Os02g56680	LOC_Os06g37080	8.80756322
LOC_Os02g56100	LOC_Os10g01540	9.25559862
LOC_Os02g56460	LOC_Os05g51670	9.15472953
LOC_Os02g56130	LOC_Os03g27310	8.94473814
LOC_Os02g56680	LOC_Os09g07510	8.9406359
LOC_Os02g56460	LOC_Os07g36500	10.706
LOC_Os02g56100	LOC_Os07g31750	9.38976721

InteractorA	InteractorB	Z score
LOC_Os02g56180	LOC_Os05g45810	12.3291791
LOC_Os02g56100	LOC_Os12g22680	9.83951236
LOC_Os02g56100	LOC_Os08g37800	8.96533985
LOC_Os02g56460	LOC_Os04g16872	8.92066189
LOC_Os02g56690	LOC_Os04g41960	8.95872855
LOC_Os02g56690	LOC_Os09g27820	9.05119232
LOC_Os02g56130	LOC_Os04g55290	8.89694611
LOC_Os02g56690	LOC_Os10g38274	9.00569501
LOC_Os02g56100	LOC_Os07g43670	9.57008318
LOC_Os02g56690	LOC_Os05g37390	9.19878925
LOC_Os02g56130	LOC_Os09g04730	8.83701009
LOC_Os02g56180	LOC_Os11g33270	9.85462059
LOC_Os02g56460	LOC_Os10g21248	9.07729923
LOC_Os02g56180	LOC_Os07g42950	9.40148826
LOC_Os02g56460	LOC_Os06g12090	10.0937203
LOC_Os02g56180	LOC_Os06g12090	9.79668285
LOC_Os02g56130	LOC_Os06g04030	8.94473814
LOC_Os02g56130	LOC_Os10g31940	9.94266491
LOC_Os02g56180	LOC_Os04g18200	8.99579228
LOC_Os02g56460	LOC_Os05g38740	10.706
LOC_Os02g56130	LOC_Os11g33240	9.39226725
LOC_Os02g56680	LOC_Os04g17650	8.80454239
LOC_Os02g56130	LOC_Os04g47170	9.13417421
LOC_Os02g56700	LOC_Os03g25370	8.98187713
LOC_Os02g56690	LOC_Os04g37460	10.3915404
LOC_Os02g56690	LOC_Os12g38760	8.9330093
LOC_Os02g56680	LOC_Os09g07830	9.08327291
LOC_Os02g56100	LOC_Os09g31486	8.89595756
LOC_Os02g56100	LOC_Os03g45410	8.85586498
LOC_Os02g56460	LOC_Os04g56070	9.77481458
LOC_Os02g56460	LOC_Os10g39410	10.706
LOC_Os02g56130	LOC_Os04g01980	9.13121993
LOC_Os02g56690	LOC_Os03g47770	9.26156728
LOC_Os02g56180	LOC_Os12g08270	8.94412248
LOC_Os02g56680	LOC_Os07g38910	11.886975
LOC_Os02g56690	LOC_Os05g38760	9.0010813
LOC_Os02g56130	LOC_Os04g46620	8.99072843
LOC_Os02g56680	LOC_Os06g35630	8.85285446
LOC_Os02g56130	LOC_Os04g37820	9.67306508
LOC_Os02g56180	LOC_Os11g32520	8.80316731
LOC_Os02g56180	LOC_Os05g33130	9.23860136
LOC_Os02g56130	LOC_Os02g57260	10.7759657
LOC_Os02g56180	LOC_Os03g61970	9.2228094

InteractorA	InteractorB	Z score
LOC_Os02g56130	LOC_Os07g31750	9.18040147
LOC_Os02g56460	LOC_Os09g32640	9.29048763
LOC_Os02g56460	LOC_Os09g27820	9.01665353
LOC_Os02g56680	LOC_Os03g64030	9.46089163
LOC_Os02g56100	LOC_Os11g37550	11.4437867
LOC_Os02g56130	LOC_Os03g11990	8.85435646
LOC_Os02g56100	LOC_Os12g04980	10.2348244
LOC_Os02g56180	LOC_Os06g40940	9.19664687
LOC_Os02g56690	LOC_Os09g37100	9.99460029
LOC_Os02g56460	LOC_Os09g38020	10.706
LOC_Os02g56690	LOC_Os07g01760	9.24129917
LOC_Os02g56460	LOC_Os04g32010	9.32097534
LOC_Os02g56680	LOC_Os05g31040	8.9082371
LOC_Os02g56690	LOC_Os04g34630	9.18003836
LOC_Os02g56680	LOC_Os06g40190	8.93606428
LOC_Os02g56100	LOC_Os03g48310	9.07246059
LOC_Os02g56460	LOC_Os11g28340	9.78388848
LOC_Os02g56180	LOC_Os10g25130	8.84104424
LOC_Os02g56100	LOC_Os07g06970	9.18945991
LOC_Os02g56100	LOC_Os03g04410	9.10012465
LOC_Os02g56680	LOC_Os06g11290	8.80226807
LOC_Os02g56460	LOC_Os04g49420	10.706
LOC_Os02g56460	LOC_Os03g56410	9.22791007
LOC_Os02g56100	LOC_Os10g38740	8.94418047
LOC_Os02g56680	LOC_Os07g34190	8.84368361
LOC_Os02g56460	LOC_Os12g34062	10.2063405
LOC_Os02g56100	LOC_Os10g38690	9.85377811
LOC_Os02g56690	LOC_Os11g25330	9.0721466
LOC_Os02g56180	LOC_Os05g38550	9.80592741
LOC_Os02g56690	LOC_Os08g09770	9.18240872
LOC_Os02g56680	LOC_Os04g56950	10.7164287
LOC_Os02g56680	LOC_Os10g26600	11.7370931
LOC_Os02g56690	LOC_Os09g08072	11.0494852
LOC_Os02g56460	LOC_Os09g26340	10.706
LOC_Os02g56690	LOC_Os08g28820	9.30271497
LOC_Os02g56460	LOC_Os03g45320	9.28773385
LOC_Os02g56130	LOC_Os07g09680	9.20572034
LOC_Os02g56130	LOC_Os09g38030	8.90800834
LOC_Os02g56690	LOC_Os12g07820	9.07015677
LOC_Os02g56130	LOC_Os12g13810	8.85737
LOC_Os02g56180	LOC_Os05g04340	8.92926982
LOC_Os02g56130	LOC_Os06g01590	10.0927233
LOC_Os02g56180	LOC_Os03g45320	9.76965841

InteractorA	InteractorB	Z score
LOC_Os02g56130	LOC_Os03g57290	10.1773244
LOC_Os02g56680	LOC_Os03g48471	9.53428514
LOC_Os02g56100	LOC_Os10g08580	9.09911576
LOC_Os02g56100	LOC_Os06g23760	9.03599574
LOC_Os02g56690	LOC_Os07g25590	11.6159997
LOC_Os02g57450	LOC_Os03g16740	9.22721023
LOC_Os02g56700	LOC_Os04g39900	9.32230256
LOC_Os02g57040	LOC_Os07g34190	11.2502732
LOC_Os02g57240	LOC_Os04g01980	8.86397741
LOC_Os02g57150	LOC_Os03g63330	9.10535164
LOC_Os02g57150	LOC_Os03g15120	9.7794303
LOC_Os02g57260	LOC_Os09g10260	11.2640714
LOC_Os02g57040	LOC_Os04g44870	8.83619799
LOC_Os02g57040	LOC_Os06g37660	9.75096412
LOC_Os02g57260	LOC_Os03g40720	12.2479176
LOC_Os02g57040	LOC_Os12g22030	9.37167571
LOC_Os02g57040	LOC_Os07g08840	10.0158612
LOC_Os02g57150	LOC_Os04g53290	9.67716929
LOC_Os02g57150	LOC_Os03g05590	8.81956015
LOC_Os02g56700	LOC_Os08g14570	8.97359996
LOC_Os02g56700	LOC_Os07g02210	9.00469776
LOC_Os02g57260	LOC_Os10g26130	9.17594549
LOC_Os02g57260	LOC_Os08g40930	8.89489308
LOC_Os02g57040	LOC_Os12g05410	9.69036523
LOC_Os02g56720	LOC_Os06g09450	8.81138802
LOC_Os02g57450	LOC_Os03g13170	8.87224688
LOC_Os02g57150	LOC_Os04g45290	9.16471589
LOC_Os02g57240	LOC_Os04g44950	9.0391024
LOC_Os02g56720	LOC_Os09g23540	8.93562332
LOC_Os02g57040	LOC_Os10g11810	9.07016753
LOC_Os02g57150	LOC_Os05g41080	8.9767119
LOC_Os02g57040	LOC_Os11g10480	10.5826217
LOC_Os02g57150	LOC_Os06g05090	8.82372983
LOC_Os02g57150	LOC_Os08g34210	8.83006333
LOC_Os02g57040	LOC_Os12g01922	9.5641547
LOC_Os02g57150	LOC_Os10g02480	9.92630244
LOC_Os02g57150	LOC_Os10g40710	9.22404439
LOC_Os02g56700	LOC_Os08g28800	9.15584153
LOC_Os02g57260	LOC_Os03g05290	9.1419597
LOC_Os02g57240	LOC_Os06g37560	9.0186066
LOC_Os02g57150	LOC_Os10g23100	8.82520574
LOC_Os02g57260	LOC_Os10g30200	9.14044746
LOC_Os02g57260	LOC_Os05g33140	9.38575614

InteractorA	InteractorB	Z score
LOC_Os02g56720	LOC_Os04g44920	9.26955701
LOC_Os02g56700	LOC_Os09g27750	9.77832594
LOC_Os02g57150	LOC_Os11g16590	9.12052822
LOC_Os02g56700	LOC_Os10g39840	8.86819236
LOC_Os02g57040	LOC_Os11g33240	9.69531365
LOC_Os02g57260	LOC_Os03g51740	8.80278194
LOC_Os02g56700	LOC_Os09g14670	9.25722299
LOC_Os02g56700	LOC_Os07g25590	9.18758177
LOC_Os02g57450	LOC_Os04g32010	8.91467411
LOC_Os02g57150	LOC_Os03g01640	9.31257277
LOC_Os02g56700	LOC_Os08g33710	8.91657547
LOC_Os02g57260	LOC_Os12g06620	9.02499382
LOC_Os02g57260	LOC_Os03g38980	8.99315492
LOC_Os02g57040	LOC_Os05g09440	9.82658309
LOC_Os02g57450	LOC_Os06g04280	9.56960023
LOC_Os02g56700	LOC_Os09g08910	9.12413556
LOC_Os02g56720	LOC_Os06g28550	10.7132
LOC_Os02g57150	LOC_Os09g14670	8.9492586
LOC_Os02g57260	LOC_Os05g35400	9.17972103
LOC_Os02g57240	LOC_Os03g11970	9.61629605
LOC_Os02g57240	LOC_Os04g08350	10.2841252
LOC_Os02g57260	LOC_Os09g31502	8.80247129
LOC_Os02g57260	LOC_Os07g29440	8.83461051
LOC_Os02g57040	LOC_Os07g10720	9.13161737
LOC_Os02g56700	LOC_Os10g41190	8.89576611
LOC_Os02g57040	LOC_Os05g47545	9.81697131
LOC_Os02g57040	LOC_Os06g28550	10.4543791
LOC_Os02g56720	LOC_Os04g30420	8.98093661
LOC_Os02g56700	LOC_Os11g33240	9.09422396
LOC_Os02g56700	LOC_Os07g05400	9.47085477
LOC_Os02g57240	LOC_Os04g46560	8.82245428
LOC_Os02g57150	LOC_Os04g56210	9.6737885
LOC_Os02g57150	LOC_Os06g43640	11.264346
LOC_Os02g57240	LOC_Os04g10400	9.49126334
LOC_Os02g57260	LOC_Os05g11730	9.02154272
LOC_Os02g56700	LOC_Os12g22650	10.8059477
LOC_Os02g56700	LOC_Os07g03730	8.91511388
LOC_Os02g56720	LOC_Os03g59020	9.04567882
LOC_Os02g56720	LOC_Os03g28400	10.0400504
LOC_Os02g57150	LOC_Os04g38870	10.2894501
LOC_Os02g56700	LOC_Os09g31486	8.87202255
LOC_Os02g57150	LOC_Os04g29550	10.4409046
LOC_Os02g56700	LOC_Os11g19800	11.0295897

InteractorA	InteractorB	Z score
LOC_Os02g56700	LOC_Os11g08340	10.2125096
LOC_Os02g56720	LOC_Os08g42910	9.96142766
LOC_Os02g56720	LOC_Os03g28330	9.80000877
LOC_Os02g57150	LOC_Os03g55600	9.44984425
LOC_Os02g56720	LOC_Os04g31960	9.61201269
LOC_Os02g57240	LOC_Os10g28080	10.814205
LOC_Os02g56720	LOC_Os03g56410	9.09808722
LOC_Os02g56700	LOC_Os07g30170	10.2785673
LOC_Os02g56700	LOC_Os11g37640	8.87688486
LOC_Os02g57150	LOC_Os10g25140	9.09230688
LOC_Os02g57040	LOC_Os04g01740	9.61171387
LOC_Os02g57240	LOC_Os06g46340	9.0788484
LOC_Os02g56720	LOC_Os02g58480	10.2876008
LOC_Os02g57040	LOC_Os12g13390	9.37457473
LOC_Os02g57040	LOC_Os09g39570	10.1740321
LOC_Os02g57040	LOC_Os06g05090	9.11928872
LOC_Os02g57450	LOC_Os04g16826	8.96820148
LOC_Os02g57150	LOC_Os07g48880	9.88066767
LOC_Os02g57260	LOC_Os12g12560	8.92440916
LOC_Os02g57040	LOC_Os11g47560	10.2270911
LOC_Os02g57040	LOC_Os05g46290	10.6594914
LOC_Os02g57150	LOC_Os06g35490	9.27528273
LOC_Os02g57240	LOC_Os11g14040	9.57554835
LOC_Os02g57040	LOC_Os03g19930	9.41792764
LOC_Os02g56720	LOC_Os07g44790	10.0401079
LOC_Os02g56700	LOC_Os08g20270	8.81050406
LOC_Os02g56720	LOC_Os03g52970	9.15174724
LOC_Os01g11054	LOC_Os02g47110	9.94656526
LOC_Os01g10840	LOC_Os09g36900	9.58740105
LOC_Os01g10840	LOC_Os08g06060	10.2291193
LOC_Os01g10850	LOC_Os03g15880	9.3711825
LOC_Os01g10850	LOC_Os02g51930	9.20268051
LOC_Os01g10850	LOC_Os05g08430	9.95687411
LOC_Os01g11054	LOC_Os12g13800	8.91920591
LOC_Os01g10820	LOC_Os12g26290	8.99971122
LOC_Os01g11110	LOC_Os06g35560	9.18986626
LOC_Os01g10940	LOC_Os11g26860	9.07544251
LOC_Os01g11110	LOC_Os01g58380	10.3167335
LOC_Os01g10840	LOC_Os10g40600	8.92636292
LOC_Os01g11110	LOC_Os06g04900	8.82773822
LOC_Os01g10840	LOC_Os04g16760	8.90638392
LOC_Os01g10820	LOC_Os10g29620	9.0458128
LOC_Os01g10940	LOC_Os03g61360	9.92039497

InteractorA	InteractorB	Z score
LOC_Os01g10840	LOC_Os11g10520	8.91823956
LOC_Os01g10840	LOC_Os05g27940	8.92863427
LOC_Os01g10840	LOC_Os11g10480	9.55549756
LOC_Os01g10850	LOC_Os07g40580	9.34547035
LOC_Os01g10840	LOC_Os02g52610	9.17415804
LOC_Os01g10940	LOC_Os01g70220	9.29850497
LOC_Os01g11054	LOC_Os12g34874	8.83302995
LOC_Os01g11110	LOC_Os02g52630	8.94089761
LOC_Os01g10850	LOC_Os04g29550	9.08646809
LOC_Os01g10850	LOC_Os01g38970	9.33792751
LOC_Os01g10850	LOC_Os03g58430	9.49782678
LOC_Os01g10840	LOC_Os01g57945	9.31302688
LOC_Os01g10850	LOC_Os06g04200	9.14281547
LOC_Os01g10840	LOC_Os01g60790	9.10818114
LOC_Os01g11054	LOC_Os01g70525	9.16474614
LOC_Os01g11110	LOC_Os03g18580	9.03193316
LOC_Os01g11110	LOC_Os01g48600	8.89868643
LOC_Os01g10840	LOC_Os09g15400	9.42998179
LOC_Os01g10840	LOC_Os01g16890	9.27801482
LOC_Os01g10850	LOC_Os12g12580	10.0734543
LOC_Os01g11110	LOC_Os04g50880	8.94425128
LOC_Os01g10850	LOC_Os07g07060	9.08718966
LOC_Os01g10840	LOC_Os04g27060	9.69584563
LOC_Os01g10940	LOC_Os06g51050	8.91575529
LOC_Os01g11110	LOC_Os04g57380	9.7588622
LOC_Os01g11054	LOC_Os10g38340	9.0103213
LOC_Os01g11054	LOC_Os07g10590	8.90509229
LOC_Os01g10840	LOC_Os03g04250	9.20994595
LOC_Os01g10840	LOC_Os07g05180	9.53211019
LOC_Os01g11110	LOC_Os02g47590	9.23646782
LOC_Os01g10840	LOC_Os05g01675	8.90638392
LOC_Os01g10840	LOC_Os08g28800	10.874269
LOC_Os01g10840	LOC_Os02g46970	9.57496152
LOC_Os01g10840	LOC_Os05g19670	8.95779472
LOC_Os01g10850	LOC_Os01g71810	9.4650558
LOC_Os01g10850	LOC_Os03g44484	9.22916792
LOC_Os01g11110	LOC_Os01g18110	10.098182
LOC_Os01g10840	LOC_Os08g39870	9.9542007
LOC_Os01g10840	LOC_Os12g17540	9.40199537
LOC_Os01g10940	LOC_Os05g31040	11.2446799
LOC_Os01g10850	LOC_Os02g08100	9.17663463
LOC_Os01g10850	LOC_Os09g09270	9.94469104
LOC_Os01g10840	LOC_Os04g16844	9.31301418

InteractorA	InteractorB	Z score
LOC_Os01g10840	LOC_Os10g40090	9.01530904
LOC_Os01g11054	LOC_Os04g39864	11.735022
LOC_Os01g10840	LOC_Os07g38890	8.88363667
LOC_Os01g10840	LOC_Os03g11530	10.5299447
LOC_Os01g11110	LOC_Os01g65090	8.99895931
LOC_Os01g11054	LOC_Os04g01250	8.97027362
LOC_Os01g10850	LOC_Os02g36400	8.90096998
LOC_Os01g10840	LOC_Os06g36820	9.11883226
LOC_Os01g10840	LOC_Os10g17650	9.13391573
LOC_Os01g11110	LOC_Os04g48700	9.15776195
LOC_Os01g10840	LOC_Os02g24632	8.82816671
LOC_Os01g10840	LOC_Os12g02980	9.85338638
LOC_Os01g10840	LOC_Os04g36700	9.14268458
LOC_Os01g11054	LOC_Os03g20700	10.82894
LOC_Os01g10850	LOC_Os11g26850	9.05968536
LOC_Os01g10850	LOC_Os08g02410	10.2501688
LOC_Os01g10840	LOC_Os01g57964	8.90638392
LOC_Os01g10850	LOC_Os08g33710	9.2318063
LOC_Os01g10850	LOC_Os03g41460	9.80875949
LOC_Os01g11054	LOC_Os03g21260	9.63666787
LOC_Os01g10840	LOC_Os10g41510	8.81190017
LOC_Os01g10840	LOC_Os10g21326	9.31302125
LOC_Os01g10850	LOC_Os09g08072	10.1060702
LOC_Os01g10840	LOC_Os11g33240	9.72656009
LOC_Os01g10940	LOC_Os01g48680	8.80521085
