# Supplementary material for: Structure-based prediction of protein-protein interaction network in rice
Source: Genet Mol Biol. 2024 Feb 2;47(1):e20230068. doi: 10.1590/1678-4685-GMB-2023-0068 (PMC10849033; doi:10.1590/1678-4685-GMB-2023-0068)
Supplement: Table S4 - [file 1415-4757-GMB-47-01-e20230068-s4.pdf]

Genetics and Molecular Biology
https://doi.org/10.1590/1678-4685-GMB-2023-0068

Supplementary Material to “Structure-based prediction of protein-protein interaction network in rice”

Table S4. Trait-associated nonsynonymous SNPs at the predicted interface.

<table>
<tr><th>ID</th><th>Position</th><th>Alleles</th><th>Locus</th><th>Transcript</th><th>Consequence</th><th>Associated trait</th></tr>
<tr><td>osa177782</td><td>chr01:3897986</td><td>T/A</td><td>LOC_Os01g08020</td><td>LOC_Os01g08020.1</td><td>c.448A>T|p.Arg150Trp</td><td>plant height</td></tr>
<tr><td>osa732504</td><td>chr01:15217368</td><td>T/C</td><td>LOC_Os01g27260</td><td>LOC_Os01g27260.1</td><td>c.346A>G|p.Ile116Val</td><td>pericarp color</td></tr>
<tr><td>osa2371569</td><td>chr02:7721671</td><td>C/T</td><td>LOC_Os02g14130</td><td>LOC_Os02g14130.1</td><td>c.622G>A|p.Asp208Asn</td><td>spikelet number</td></tr>
<tr><td>osa2402363</td><td>chr02:8327191</td><td>T/C</td><td>LOC_Os02g14929</td><td>LOC_Os02g14929.1</td><td>c.1267A>G|p.Thr423Ala</td><td>spikelet number</td></tr>
<tr><td>osa2402396</td><td>chr02:8327697</td><td>G/T</td><td>LOC_Os02g14929</td><td>LOC_Os02g14929.1</td><td>c.837C>A|p.Asn279Lys</td><td>spikelet number</td></tr>
<tr><td>osa2402414</td><td>chr02:8328200</td><td>T/C</td><td>LOC_Os02g14929</td><td>LOC_Os02g14929.1</td><td>c.514A>G|p.Ile172Val</td><td>spikelet number</td></tr>
<tr><td>osa2971097</td><td>chr02:19220335</td><td>G/T</td><td>LOC_Os02g32490</td><td>LOC_Os02g32490.1</td><td>c.492G>T|p.Glu164Asp</td><td>root to shoot ratio</td></tr>
<tr><td>osa3705433</td><td>chr02:34759038</td><td>A/G</td><td>LOC_Os02g56690</td><td>LOC_Os02g56690.1</td><td>c.965A>G|p.Tyr322Cys</td><td>relative thousand kernel weight</td></tr>
<tr><td>osa3771773</td><td>chr03:493559</td><td>G/T</td><td>LOC_Os03g01800</td><td>LOC_Os03g01800.1</td><td>c.367C>A|p.Leu123Met</td><td>days to flowering trait</td></tr>
<tr><td>osa4248719</td><td>chr03:11713079</td><td>C/G</td><td>LOC_Os03g20700</td><td>LOC_Os03g20700.2</td><td>c.636C>G|p.Asn212Lys</td><td>seed weight</td></tr>
<tr><td>osa4291669</td><td>chr03:12679351</td><td>T/C</td><td>LOC_Os03g22120</td><td>LOC_Os03g22120.1</td><td>c.473A>G|p.Gln158Arg</td><td>germination index</td></tr>
<tr><td>osa7502585</td><td>chr05:6975956</td><td>A/G</td><td>LOC_Os05g12180</td><td>LOC_Os05g12180.1</td><td>c.1066T>C|p.Cys356Arg</td><td>germination index</td></tr>
<tr><td>osa9256088</td><td>chr06:13407809</td><td>A/G</td><td>LOC_Os06g22960</td><td>LOC_Os06g22960.1</td><td>c.319A>G|p.Ile107Val</td><td>leaf bronzing score</td></tr>
<tr><td>osa11264458</td><td>chr07:23522597</td><td>T/C</td><td>LOC_Os07g39290</td><td>LOC_Os07g39290.1</td><td>c.152T>C|p.Ile51Thr</td><td>grain length to width ratio</td></tr>
<tr><td>osa11307778</td><td>chr07:24556027</td><td>A/G</td><td>LOC_Os07g41050</td><td>LOC_Os07g41050.1</td><td>c.854A>G|p.Asp285Gly</td><td>grain length to width ratio</td></tr>
<tr><td>osa11368557</td><td>chr07:25980888</td><td>G/A</td><td>LOC_Os07g43390</td><td>LOC_Os07g43390.1</td><td>c.383G>A|p.Arg128His</td><td>grain length to width ratio</td></tr>
<tr><td>osa11371481</td><td>chr07:26036647</td><td>C/T</td><td>LOC_Os07g43510</td><td>LOC_Os07g43510.1</td><td>c.55G>A|p.Val19Met</td><td>grain length to width ratio</td></tr>
</table>

ID	Position	Alleles	Locus	Transcript	Consequence	Associated trait
osa11769077	chr08:5383250	A/G	LOC_Os08g09260	LOC_Os08g09260.1	c.439T>C|p.Tyr147His	panicle number
osa15426610	chr11:4450189	A/G	LOC_Os11g08445	LOC_Os11g08445.1	c.358A>G|p.Ser120Gly	plant height
osa15426611	chr11:4450190	G/A	LOC_Os11g08445	LOC_Os11g08445.1	c.359G>A|p.Ser120Asn	plant height
osa15426685	chr11:4451404	T/A	LOC_Os11g08445	LOC_Os11g08445.1	c.1573T>A|p.Tyr525Asn	plant height
osa15426686	chr11:4451416	G/A	LOC_Os11g08445	LOC_Os11g08445.1	c.1585G>A|p.Glu529Lys	plant height
osa15426688	chr11:4451423	T/C	LOC_Os11g08445	LOC_Os11g08445.1	c.1592T>C|p.Val531Ala	plant height
osa15427127	chr11:4466777	T/C	LOC_Os11g08460	LOC_Os11g08460.1	c.1592A>G|p.Asn531Ser	plant height
osa15427131	chr11:4466801	T/C	LOC_Os11g08460	LOC_Os11g08460.1	c.1568A>G|p.Gln523Arg	plant height
osa15427132	chr11:4466802	G/T	LOC_Os11g08460	LOC_Os11g08460.1	c.1567C>A|p.Gln523Lys	plant height
osa15427135	chr11:4466810	A/T	LOC_Os11g08460	LOC_Os11g08460.1	c.1559T>A|p.Val520Asp	plant height
osa15427136	chr11:4466813	T/A	LOC_Os11g08460	LOC_Os11g08460.1	c.1556A>T|p.Lys519Met	plant height
osa15427137	chr11:4466816	T/C	LOC_Os11g08460	LOC_Os11g08460.1	c.1553A>G|p.His518Arg	plant height
osa15427158	chr11:4466879	A/G	LOC_Os11g08460	LOC_Os11g08460.1	c.1490T>C|p.Val497Ala	plant height
osa15427159	chr11:4466882	C/T	LOC_Os11g08460	LOC_Os11g08460.1	c.1487G>A|p.Arg496Lys	plant height
osa15427160	chr11:4466883	T/G,C	LOC_Os11g08460	LOC_Os11g08460.1	c.1486A>G|p.Arg496Gly	plant height
osa15427745	chr11:4476885	T/A	LOC_Os11g08470	LOC_Os11g08470.1	c.1262A>T|p.Asn421Ile	plant height
osa15427752	chr11:4476997	C/T	LOC_Os11g08470	LOC_Os11g08470.1	c.1150G>A|p.Val384Ile	plant height
