# Supplementary material for: Structure-based prediction of protein-protein interaction network in rice
Source: Genet Mol Biol. 2024 Feb 2;47(1):e20230068. doi: 10.1590/1678-4685-GMB-2023-0068 (PMC10849033; doi:10.1590/1678-4685-GMB-2023-0068)
Supplement: Table S5 - [file 1415-4757-GMB-47-01-e20230068-s5.pdf]

Genetics and Molecular Biology
https://doi.org/10.1590/1678-4685-GMB-2023-0068

Supplementary Material to "Structure-based prediction of protein-protein interaction network in rice"

Table S5. Predicted changes in binding affinity (ΔΔG) caused by nonsynonymous SNPs.

InteractorA	InteractorB	Allele description	Associated trait	ΔΔG (kcal/mol)
LOC_Os01g08020	LOC_Os01g44260	osa177782|LOC_Os01g08020.1|c.448A>T|p.Arg150Trp	plant height	0.311
LOC_Os01g08020	LOC_Os05g27950	osa177782|LOC_Os01g08020.1|c.448A>T|p.Arg150Trp	plant height	0.172
LOC_Os01g27260	LOC_Os02g02560	osa732504|LOC_Os01g27260.1|c.346A>G|p.Ile116Val	pericarp color	-0.635
LOC_Os01g27260	LOC_Os02g52390	osa732504|LOC_Os01g27260.1|c.346A>G|p.Ile116Val	pericarp color	-0.306
LOC_Os01g27260	LOC_Os03g07150	osa732504|LOC_Os01g27260.1|c.346A>G|p.Ile116Val	pericarp color	-0.308
LOC_Os01g27260	LOC_Os03g22120	osa732504|LOC_Os01g27260.1|c.346A>G|p.Ile116Val	pericarp color	-0.499
LOC_Os01g27260	LOC_Os07g07550	osa732504|LOC_Os01g27260.1|c.346A>G|p.Ile116Val	pericarp color	-0.445
LOC_Os01g27260	LOC_Os08g37800	osa732504|LOC_Os01g27260.1|c.346A>G|p.Ile116Val	pericarp color	-0.552
LOC_Os01g27260	LOC_Os11g14910	osa732504|LOC_Os01g27260.1|c.346A>G|p.Ile116Val	pericarp color	-1.122
LOC_Os02g14130	LOC_Os04g43400	osa2371569|LOC_Os02g14130.1|c.622G>A|p.Asp208Asn	spikelet number	1.478
LOC_Os02g14929	LOC_Os05g46290	osa2402363|LOC_Os02g14929.1|c.1267A>G|p.Thr423Ala	spikelet number	-0.193
LOC_Os02g14929	LOC_Os09g20260	osa2402396|LOC_Os02g14929.1|c.837C>A|p.Asn279Lys	spikelet number	0.245
LOC_Os02g14929	LOC_Os09g36710	osa2402396|LOC_Os02g14929.1|c.837C>A|p.Asn279Lys	spikelet number	-0.769
LOC_Os02g14929	LOC_Os12g38770	osa2402396|LOC_Os02g14929.1|c.837C>A|p.Asn279Lys	spikelet number	-0.74
LOC_Os02g14929	LOC_Os01g59340	osa2402396|LOC_Os02g14929.1|c.837C>A|p.Asn279Lys	spikelet number	0.373
LOC_Os02g14929	LOC_Os01g73740	osa2402414|LOC_Os02g14929.1|c.514A>G|p.Ile172Val	spikelet number	-0.507

**InteractorA**	**InteractorB**	**Allele description**	**Associated trait**	**ΔΔG （kcal/mol）**
LOC_Os02g32490	LOC_Os03g42110	osa2971097|LOC_Os02g32490.1|c.492G>T|p.Glu164Asp	root to shoot ratio	-0.059
LOC_Os02g32490	LOC_Os03g55090	osa2971097|LOC_Os02g32490.1|c.492G>T|p.Glu164Asp	root to shoot ratio	0.226
LOC_Os02g32490	LOC_Os04g41960	osa2971097|LOC_Os02g32490.1|c.492G>T|p.Glu164Asp	root to shoot ratio	0.037
LOC_Os02g32490	LOC_Os08g08500	osa2971097|LOC_Os02g32490.1|c.492G>T|p.Glu164Asp	root to shoot ratio	0.01
LOC_Os02g32490	LOC_Os01g27490	osa2971097|LOC_Os02g32490.1|c.492G>T|p.Glu164Asp	root to shoot ratio	-0.328
LOC_Os02g32490	LOC_Os01g56880	osa2971097|LOC_Os02g32490.1|c.492G>T|p.Glu164Asp	root to shoot ratio	0.548
LOC_Os02g56690	LOC_Os03g47770	osa3705433|LOC_Os02g56690.1|c.965A>G|p.Tyr322Cys	relative thousand kernel weight	-1.129
LOC_Os02g56690	LOC_Os04g34630	osa3705433|LOC_Os02g56690.1|c.965A>G|p.Tyr322Cys	relative thousand kernel weight	-1.141
LOC_Os02g56690	LOC_Os04g37460	osa3705433|LOC_Os02g56690.1|c.965A>G|p.Tyr322Cys	relative thousand kernel weight	-0.966
LOC_Os02g56690	LOC_Os04g41960	osa3705433|LOC_Os02g56690.1|c.965A>G|p.Tyr322Cys	relative thousand kernel weight	-0.974
LOC_Os02g56690	LOC_Os05g37390	osa3705433|LOC_Os02g56690.1|c.965A>G|p.Tyr322Cys	relative thousand kernel weight	-0.926
LOC_Os02g56690	LOC_Os07g01760	osa3705433|LOC_Os02g56690.1|c.965A>G|p.Tyr322Cys	relative thousand kernel weight	-1.001
LOC_Os02g56690	LOC_Os07g25590	osa3705433|LOC_Os02g56690.1|c.965A>G|p.Tyr322Cys	relative thousand kernel weight	-1.506
LOC_Os02g56690	LOC_Os08g28820	osa3705433|LOC_Os02g56690.1|c.965A>G|p.Tyr322Cys	relative thousand kernel weight	-1.218
LOC_Os02g56690	LOC_Os09g08072	osa3705433|LOC_Os02g56690.1|c.965A>G|p.Tyr322Cys	relative thousand kernel weight	-0.604
LOC_Os02g56690	LOC_Os09g27820	osa3705433|LOC_Os02g56690.1|c.965A>G|p.Tyr322Cys	relative thousand kernel weight	-2.013
LOC_Os02g56690	LOC_Os09g37100	osa3705433|LOC_Os02g56690.1|c.965A>G|p.Tyr322Cys	relative thousand kernel weight	-0.762
LOC_Os02g56690	LOC_Os11g16590	osa3705433|LOC_Os02g56690.1|c.965A>G|p.Tyr322Cys	relative thousand kernel weight	-0.757
LOC_Os02g56690	LOC_Os11g25330	osa3705433|LOC_Os02g56690.1|c.965A>G|p.Tyr322Cys	relative thousand kernel weight	-0.404
LOC_Os02g56690	LOC_Os12g02980	osa3705433|LOC_Os02g56690.1|c.965A>G|p.Tyr322Cys	relative thousand kernel weight	-0.898
LOC_Os02g56690	LOC_Os12g07820	osa3705433|LOC_Os02g56690.1|c.965A>G|p.Tyr322Cys	relative thousand kernel weight	-1.218
LOC_Os02g56690	LOC_Os12g38760	osa3705433|LOC_Os02g56690.1|c.965A>G|p.Tyr322Cys	relative thousand kernel weight	-0.502
LOC_Os02g56690	LOC_Os12g41110	osa3705433|LOC_Os02g56690.1|c.965A>G|p.Tyr322Cys	relative thousand kernel weight	-1.411
LOC_Os02g56690	LOC_Os01g01650	osa3705433|LOC_Os02g56690.1|c.965A>G|p.Tyr322Cys	relative thousand kernel weight	-1.397

InteractorA	InteractorB	Allele description	Associated trait	ΔΔG (kcal/mol)
LOC_Os02g56690	LOC_Os01g21180	osa3705433|LOC_Os02g56690.1|c.965A>G|p.Tyr322Cys	relative thousand kernel weight	-1.147
LOC_Os02g56690	LOC_Os01g49690	osa3705433|LOC_Os02g56690.1|c.965A>G|p.Tyr322Cys	relative thousand kernel weight	-1.022
LOC_Os02g56690	LOC_Os01g60360	osa3705433|LOC_Os02g56690.1|c.965A>G|p.Tyr322Cys	relative thousand kernel weight	-1.549
LOC_Os02g56690	LOC_Os01g60410	osa3705433|LOC_Os02g56690.1|c.965A>G|p.Tyr322Cys	relative thousand kernel weight	-1.549
LOC_Os02g56690	LOC_Os02g18880	osa3705433|LOC_Os02g56690.1|c.965A>G|p.Tyr322Cys	relative thousand kernel weight	-0.931
LOC_Os02g56690	LOC_Os02g44630	osa3705433|LOC_Os02g56690.1|c.965A>G|p.Tyr322Cys	relative thousand kernel weight	-1.308
LOC_Os02g56690	LOC_Os02g52230	osa3705433|LOC_Os02g56690.1|c.965A>G|p.Tyr322Cys	relative thousand kernel weight	-1.055
LOC_Os03g01800	LOC_Os03g59020	osa3771773|LOC_Os03g01800.1|c.367C>A|p.Leu123Met	days to flowering trait	-0.199
LOC_Os03g01800	LOC_Os04g12960	osa3771773|LOC_Os03g01800.1|c.367C>A|p.Leu123Met	days to flowering trait	-0.35
LOC_Os03g01800	LOC_Os05g06480	osa3771773|LOC_Os03g01800.1|c.367C>A|p.Leu123Met	days to flowering trait	-0.195
LOC_Os03g01800	LOC_Os08g20270	osa3771773|LOC_Os03g01800.1|c.367C>A|p.Leu123Met	days to flowering trait	0.061
LOC_Os03g01800	LOC_Os08g40740	osa3771773|LOC_Os03g01800.1|c.367C>A|p.Leu123Met	days to flowering trait	-0.746
LOC_Os03g01800	LOC_Os10g38234	osa3771773|LOC_Os03g01800.1|c.367C>A|p.Leu123Met	days to flowering trait	-0.639
LOC_Os03g01800	LOC_Os10g38640	osa3771773|LOC_Os03g01800.1|c.367C>A|p.Leu123Met	days to flowering trait	-0.085
LOC_Os03g01800	LOC_Os01g55030	osa3771773|LOC_Os03g01800.1|c.367C>A|p.Leu123Met	days to flowering trait	-0.34
LOC_Os03g01800	LOC_Os01g62860	osa3771773|LOC_Os03g01800.1|c.367C>A|p.Leu123Met	days to flowering trait	-0.871
LOC_Os03g01800	LOC_Os01g64850	osa3771773|LOC_Os03g01800.1|c.367C>A|p.Leu123Met	days to flowering trait	0.225
LOC_Os03g01800	LOC_Os02g34600	osa3771773|LOC_Os03g01800.1|c.367C>A|p.Leu123Met	days to flowering trait	-0.51
LOC_Os03g01800	LOC_Os02g55140	osa3771773|LOC_Os03g01800.1|c.367C>A|p.Leu123Met	days to flowering trait	-0.771
LOC_Os03g01800	LOC_Os07g17010	osa3771773|LOC_Os03g01800.1|c.367C>A|p.Leu123Met	days to flowering trait	-0.354
LOC_Os03g20700	LOC_Os03g58050	osa4248719|LOC_Os03g20700.2|c.636C>G|p.Asn212Lys	seed weight	-0.683
LOC_Os03g20700	LOC_Os04g40874	osa4248719|LOC_Os03g20700.2|c.636C>G|p.Asn212Lys	seed weight	-0.909
LOC_Os03g20700	LOC_Os07g07709	osa4248719|LOC_Os03g20700.2|c.636C>G|p.Asn212Lys	seed weight	-0.683
LOC_Os03g20700	LOC_Os01g09320	osa4248719|LOC_Os03g20700.2|c.636C>G|p.Asn212Lys	seed weight	-0.849

InteractorA	InteractorB	Allele description	Associated trait	ΔΔG (kcal/mol)
LOC_Os03g22120	LOC_Os12g01922	osa4291669|LOC_Os03g22120.1|c.473A>G|p.Gln158Arg	germination index	-0.166
LOC_Os03g22120	LOC_Os01g51700	osa4291669|LOC_Os03g22120.1|c.473A>G|p.Gln158Arg	germination index	-0.156
LOC_Os05g12180	LOC_Os07g44740	osa7502585|LOC_Os05g12180.1|c.1066T>C|p.Cys356Arg	germination index	0.578
LOC_Os05g12180	LOC_Os08g36320	osa7502585|LOC_Os05g12180.1|c.1066T>C|p.Cys356Arg	germination index	0.158
LOC_Os05g12180	LOC_Os10g33900	osa7502585|LOC_Os05g12180.1|c.1066T>C|p.Cys356Arg	germination index	0.087
LOC_Os05g12180	LOC_Os02g10310	osa7502585|LOC_Os05g12180.1|c.1066T>C|p.Cys356Arg	germination index	-0.118
LOC_Os05g12180	LOC_Os03g02710	osa7502585|LOC_Os05g12180.1|c.1066T>C|p.Cys356Arg	germination index	0.141
LOC_Os06g22960	LOC_Os07g09890	osa9256088|LOC_Os06g22960.1|c.319A>G|p.Ile107Val	leaf bronzing score	1.368
LOC_Os06g22960	LOC_Os09g10270	osa9256088|LOC_Os06g22960.1|c.319A>G|p.Ile107Val	leaf bronzing score	0.153
LOC_Os06g22960	LOC_Os10g38600	osa9256088|LOC_Os06g22960.1|c.319A>G|p.Ile107Val	leaf bronzing score	0.175
LOC_Os06g22960	LOC_Os01g51060	osa9256088|LOC_Os06g22960.1|c.319A>G|p.Ile107Val	leaf bronzing score	-0.018
LOC_Os06g22960	LOC_Os01g51210	osa9256088|LOC_Os06g22960.1|c.319A>G|p.Ile107Val	leaf bronzing score	1.638
LOC_Os06g22960	LOC_Os03g52970	osa9256088|LOC_Os06g22960.1|c.319A>G|p.Ile107Val	leaf bronzing score	1.364
LOC_Os07g39290	LOC_Os12g23630	osa11264458|LOC_Os07g39290.1|c.152T>C|p.Ile51Thr	grain length to width ratio	-0.454
LOC_Os07g41050	LOC_Os10g08670	osa11307778|LOC_Os07g41050.1|c.854A>G|p.Asp285Gly	grain length to width ratio	-0.194
LOC_Os07g41050	LOC_Os11g28340	osa11307778|LOC_Os07g41050.1|c.854A>G|p.Asp285Gly	grain length to width ratio	-0.401
LOC_Os07g41050	LOC_Os01g08200	osa11307778|LOC_Os07g41050.1|c.854A>G|p.Asp285Gly	grain length to width ratio	-0.287
LOC_Os07g41050	LOC_Os02g08490	osa11307778|LOC_Os07g41050.1|c.854A>G|p.Asp285Gly	grain length to width ratio	-0.351
LOC_Os07g41050	LOC_Os03g17700	osa11307778|LOC_Os07g41050.1|c.854A>G|p.Asp285Gly	grain length to width ratio	-0.358
LOC_Os07g41050	LOC_Os05g06450	osa11307778|LOC_Os07g41050.1|c.854A>G|p.Asp285Gly	grain length to width ratio	-0.463
LOC_Os07g43390	LOC_Os01g12830	osa11368557|LOC_Os07g43390.1|c.383G>A|p.Arg128His	grain length to width ratio	0.374
LOC_Os07g43390	LOC_Os02g08100	osa11368557|LOC_Os07g43390.1|c.383G>A|p.Arg128His	grain length to width ratio	-0.05
LOC_Os07g43390	LOC_Os02g52560	osa11368557|LOC_Os07g43390.1|c.383G>A|p.Arg128His	grain length to width ratio	0.325
LOC_Os07g43390	LOC_Os03g02710	osa11368557|LOC_Os07g43390.1|c.383G>A|p.Arg128His	grain length to width ratio	0.055

InteractorA	InteractorB	Allele description	Associated trait	ΔΔG (kcal/mol)
LOC_Os07g43390	LOC_Os05g36010	osa11368557|LOC_Os07g43390.1|c.383G>A|p.Arg128His	grain length to width ratio	-0.181
LOC_Os07g43510	LOC_Os08g42560	osa11371481|LOC_Os07g43510.1|c.55G>A|p.Val19Met	grain length to width ratio	-0.907
LOC_Os07g43510	LOC_Os10g26390	osa11371481|LOC_Os07g43510.1|c.55G>A|p.Val19Met	grain length to width ratio	-0.533
LOC_Os07g43510	LOC_Os11g03980	osa11371481|LOC_Os07g43510.1|c.55G>A|p.Val19Met	grain length to width ratio	-1.187
LOC_Os07g43510	LOC_Os01g08350	osa11371481|LOC_Os07g43510.1|c.55G>A|p.Val19Met	grain length to width ratio	-0.6
LOC_Os07g43510	LOC_Os01g63270	osa11371481|LOC_Os07g43510.1|c.55G>A|p.Val19Met	grain length to width ratio	-0.95
LOC_Os07g43510	LOC_Os02g12730	osa11371481|LOC_Os07g43510.1|c.55G>A|p.Val19Met	grain length to width ratio	-0.706
LOC_Os07g43510	LOC_Os03g12270	osa11371481|LOC_Os07g43510.1|c.55G>A|p.Val19Met	grain length to width ratio	-0.501
LOC_Os07g43510	LOC_Os03g42840	osa11371481|LOC_Os07g43510.1|c.55G>A|p.Val19Met	grain length to width ratio	-0.884
LOC_Os08g09260	LOC_Os04g44730	osa11769077|LOC_Os08g09260.1|c.439T>C|p.Tyr147His	panicle number	-1.325
LOC_Os08g09260	LOC_Os06g35560	osa11769077|LOC_Os08g09260.1|c.439T>C|p.Tyr147His	panicle number	-1.894
LOC_Os11g08445	LOC_Os01g10290	osa15426610|LOC_Os11g08445.1|c.358A>G|p.Ser120Gly	plant height	-0.954
LOC_Os11g08445	LOC_Os06g10930	osa15426610|LOC_Os11g08445.1|c.358A>G|p.Ser120Gly	plant height	-0.634
LOC_Os11g08445	LOC_Os01g10290	osa15426611|LOC_Os11g08445.1|c.359G>A|p.Ser120Asn	plant height	-0.634
LOC_Os11g08445	LOC_Os06g10930	osa15426611|LOC_Os11g08445.1|c.359G>A|p.Ser120Asn	plant height	-0.355
LOC_Os11g08445	LOC_Os04g16740	osa15426685|LOC_Os11g08445.1|c.1573T>A|p.Tyr525Asn	plant height	0.501
LOC_Os11g08445	LOC_Os06g10930	osa15426685|LOC_Os11g08445.1|c.1573T>A|p.Tyr525Asn	plant height	0.08
LOC_Os11g08445	LOC_Os07g11440	osa15426685|LOC_Os11g08445.1|c.1573T>A|p.Tyr525Asn	plant height	1.086
LOC_Os11g08445	LOC_Os09g31120	osa15426685|LOC_Os11g08445.1|c.1573T>A|p.Tyr525Asn	plant height	0.221
LOC_Os11g08445	LOC_Os04g16740	osa15426686|LOC_Os11g08445.1|c.1585G>A|p.Glu529Lys	plant height	0.937
LOC_Os11g08445	LOC_Os07g11440	osa15426686|LOC_Os11g08445.1|c.1585G>A|p.Glu529Lys	plant height	2.165
LOC_Os11g08445	LOC_Os09g31120	osa15426686|LOC_Os11g08445.1|c.1585G>A|p.Glu529Lys	plant height	0.745
LOC_Os11g08445	LOC_Os07g11440	osa15426688|LOC_Os11g08445.1|c.1592T>C|p.Val531Ala	plant height	-1.328
LOC_Os11g08460	LOC_Os04g58640	osa15427127|LOC_Os11g08460.1|c.1592A>G|p.Asn531Ser	plant height	-0.058

InteractorA	InteractorB	Allele description	Associated trait	ΔΔG (kcal/mol)
LOC_Os11g08460	LOC_Os12g13380	osa15427131|LOC_Os11g08460.1|c.1568A>G|p.Gln523Arg	plant height	0.547
LOC_Os11g08460	LOC_Os12g38750	osa15427131|LOC_Os11g08460.1|c.1568A>G|p.Gln523Arg	plant height	0.552
LOC_Os11g08460	LOC_Os03g06940	osa15427131|LOC_Os11g08460.1|c.1568A>G|p.Gln523Arg	plant height	0.143
LOC_Os11g08460	LOC_Os04g44890	osa15427131|LOC_Os11g08460.1|c.1568A>G|p.Gln523Arg	plant height	0.685
LOC_Os11g08460	LOC_Os05g29880	osa15427131|LOC_Os11g08460.1|c.1568A>G|p.Gln523Arg	plant height	-0.1
LOC_Os11g08460	LOC_Os07g05150	osa15427131|LOC_Os11g08460.1|c.1568A>G|p.Gln523Arg	plant height	0.656
LOC_Os11g08460	LOC_Os07g25150	osa15427131|LOC_Os11g08460.1|c.1568A>G|p.Gln523Arg	plant height	0.466
LOC_Os11g08460	LOC_Os12g13380	osa15427132|LOC_Os11g08460.1|c.1567C>A|p.Gln523Lys	plant height	1.632
LOC_Os11g08460	LOC_Os12g38750	osa15427132|LOC_Os11g08460.1|c.1567C>A|p.Gln523Lys	plant height	2.183
LOC_Os11g08460	LOC_Os03g06940	osa15427132|LOC_Os11g08460.1|c.1567C>A|p.Gln523Lys	plant height	0.474
LOC_Os11g08460	LOC_Os04g44890	osa15427132|LOC_Os11g08460.1|c.1567C>A|p.Gln523Lys	plant height	1.868
LOC_Os11g08460	LOC_Os05g29880	osa15427132|LOC_Os11g08460.1|c.1567C>A|p.Gln523Lys	plant height	0.933
LOC_Os11g08460	LOC_Os07g05150	osa15427132|LOC_Os11g08460.1|c.1567C>A|p.Gln523Lys	plant height	2.113
LOC_Os11g08460	LOC_Os07g25150	osa15427132|LOC_Os11g08460.1|c.1567C>A|p.Gln523Lys	plant height	0.829
LOC_Os11g08460	LOC_Os03g06940	osa15427135|LOC_Os11g08460.1|c.1559T>A|p.Val520Asp	plant height	0.557
LOC_Os11g08460	LOC_Os04g44890	osa15427135|LOC_Os11g08460.1|c.1559T>A|p.Val520Asp	plant height	0.472
LOC_Os11g08460	LOC_Os05g29880	osa15427135|LOC_Os11g08460.1|c.1559T>A|p.Val520Asp	plant height	0.227
LOC_Os11g08460	LOC_Os07g05150	osa15427135|LOC_Os11g08460.1|c.1559T>A|p.Val520Asp	plant height	0.423
LOC_Os11g08460	LOC_Os07g06440	osa15427135|LOC_Os11g08460.1|c.1559T>A|p.Val520Asp	plant height	0.971
LOC_Os11g08460	LOC_Os01g19750	osa15427136|LOC_Os11g08460.1|c.1556A>T|p.Lys519Met	plant height	-0.125
LOC_Os11g08460	LOC_Os07g06440	osa15427136|LOC_Os11g08460.1|c.1556A>T|p.Lys519Met	plant height	-0.703
LOC_Os11g08460	LOC_Os12g13380	osa15427137|LOC_Os11g08460.1|c.1553A>G|p.His518Arg	plant height	-0.928
LOC_Os11g08460	LOC_Os04g44890	osa15427137|LOC_Os11g08460.1|c.1553A>G|p.His518Arg	plant height	0.156
LOC_Os11g08460	LOC_Os05g29880	osa15427137|LOC_Os11g08460.1|c.1553A>G|p.His518Arg	plant height	-0.296

InteractorA	InteractorB	Allele description	Associated trait	ΔΔG (kcal/mol)
LOC_Os11g08460	LOC_Os07g06440	osa15427137|LOC_Os11g08460.1|c.1553A>G|p.His518Arg	plant height	-0.758
LOC_Os11g08460	LOC_Os07g25150	osa15427137|LOC_Os11g08460.1|c.1553A>G|p.His518Arg	plant height	-0.447
LOC_Os11g08460	LOC_Os12g13380	osa15427158|LOC_Os11g08460.1|c.1490T>C|p.Val497Ala	plant height	-0.031
LOC_Os11g08460	LOC_Os04g44890	osa15427158|LOC_Os11g08460.1|c.1490T>C|p.Val497Ala	plant height	0.314
LOC_Os11g08460	LOC_Os07g06440	osa15427158|LOC_Os11g08460.1|c.1490T>C|p.Val497Ala	plant height	0.597
LOC_Os11g08460	LOC_Os12g13380	osa15427159|LOC_Os11g08460.1|c.1487G>A|p.Arg496Lys	plant height	-1.154
LOC_Os11g08460	LOC_Os02g08130	osa15427159|LOC_Os11g08460.1|c.1487G>A|p.Arg496Lys	plant height	0.019
LOC_Os11g08460	LOC_Os03g06940	osa15427159|LOC_Os11g08460.1|c.1487G>A|p.Arg496Lys	plant height	-0.73
LOC_Os11g08460	LOC_Os04g44890	osa15427159|LOC_Os11g08460.1|c.1487G>A|p.Arg496Lys	plant height	-0.822
LOC_Os11g08460	LOC_Os05g28280	osa15427159|LOC_Os11g08460.1|c.1487G>A|p.Arg496Lys	plant height	-1.594
LOC_Os11g08460	LOC_Os07g06440	osa15427159|LOC_Os11g08460.1|c.1487G>A|p.Arg496Lys	plant height	-1.282
LOC_Os11g08460	LOC_Os12g13380	osa15427160|LOC_Os11g08460.1|c.1486A>G|p.Arg496Gly	plant height	0.107
LOC_Os11g08460	LOC_Os02g08130	osa15427160|LOC_Os11g08460.1|c.1486A>G|p.Arg496Gly	plant height	0.019
LOC_Os11g08460	LOC_Os03g06940	osa15427160|LOC_Os11g08460.1|c.1486A>G|p.Arg496Gly	plant height	0.097
LOC_Os11g08460	LOC_Os04g44890	osa15427160|LOC_Os11g08460.1|c.1486A>G|p.Arg496Gly	plant height	0.061
LOC_Os11g08460	LOC_Os05g28280	osa15427160|LOC_Os11g08460.1|c.1486A>G|p.Arg496Gly	plant height	1.629
LOC_Os11g08460	LOC_Os07g06440	osa15427160|LOC_Os11g08460.1|c.1486A>G|p.Arg496Gly	plant height	-1.282
LOC_Os11g08470	LOC_Os05g01600	osa15427745|LOC_Os11g08470.1|c.1262A>T|p.Asn421Ile	plant height	1.411
LOC_Os11g08470	LOC_Os12g06620	osa15427752|LOC_Os11g08470.1|c.1150G>A|p.Val384Ile	plant height	1.214
LOC_Os11g08470	LOC_Os01g10290	osa15427752|LOC_Os11g08470.1|c.1150G>A|p.Val384Ile	plant height	0.705
