# Supplementary material for: Structure-based prediction of protein-protein interaction network in rice
Source: Genet Mol Biol. 2024 Feb 2;47(1):e20230068. doi: 10.1590/1678-4685-GMB-2023-0068 (PMC10849033; doi:10.1590/1678-4685-GMB-2023-0068)
Supplement: Figure S2 - [file 1415-4757-GMB-47-01-e20230068-s7.pdf]

Genetics and Molecular Biology

https://doi.org/10.1590/1678-4685-GMB-2023-0068

Supplementary Material to "Structure-based prediction of protein-protein interaction network in rice"

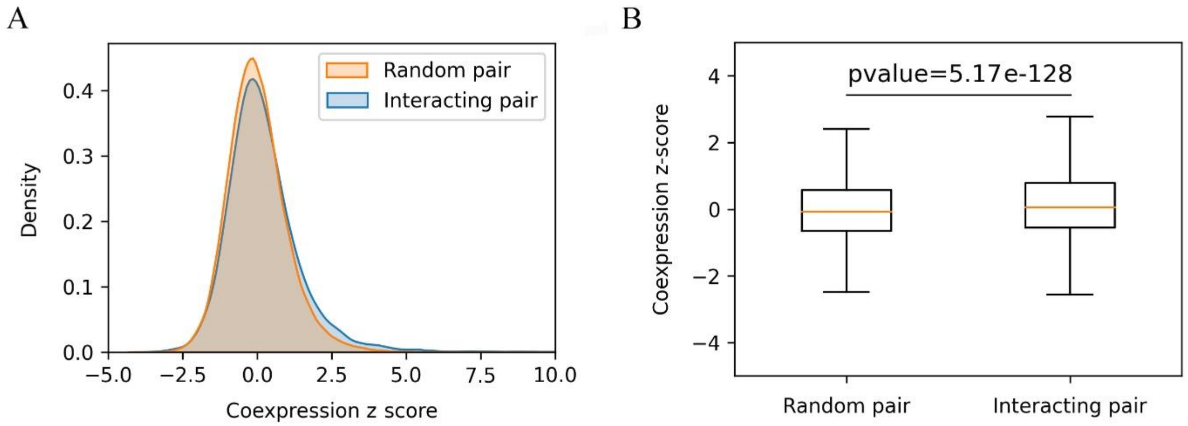

Figure S2. Coexpression analysis of PPIs predicted by the docking-based method. (A) Distribution of coexpression z scores for interacting genes and random pairs. (B) Coexpression comparison of interacting genes and random pairs. The boxplots indicate the interquartile ranges of these data. The bar in each boxplot indicates the median.
