# Supplementary material for: Structure-based prediction of protein-protein interaction network in rice
Source: Genet Mol Biol. 2024 Feb 2;47(1):e20230068. doi: 10.1590/1678-4685-GMB-2023-0068 (PMC10849033; doi:10.1590/1678-4685-GMB-2023-0068)
Supplement: Figure S3- [file 1415-4757-GMB-47-01-e20230068-s8.pdf]

Genetics and Molecular Biology

https://doi.org/10.1590/1678-4685-GMB-2023-0068

Supplementary Material to "Structure-based prediction of protein-protein interaction network in rice"

**Compartment (Protein number)**	chlo	cysk	cyto	cyto_nucl	E.R.	extr	golg	mito	nucl	nucl_plas	pero	plas	vacu
Chloroplast (632)	2136 (0.740)												
Cytoskeleton (59)	409 (0.420)	16 (0.757)											
Cytosol (754）	5313 (0.013)	508 (0.127)	3336 (5.34E-07)										
Cytosol and nucleus (8)	64 (0.122)	0 (1.000)	68 (0.392)	0 (1.000)									
Endoplasmic reticulum (36)	257 (0.267)	24 (0.449)	335 (0.010)	9 (0.005)	13 (0.022)								
Extracellular (121)	749 (0.998)	76 (0.583)	916 (0.993)	10 (0.604)	34 (0.982)	85 (0.255)							
Golgi (1)	4 (0.912)	0 (1.000)	0 (1.000)	0 (1.000)	0 (1.000)	2 (0.378)	0 (1.000)						
Mitochondria (108)	759 (0.252)	66 (0.665)	924 (0.086)	6 (0.907)	50 (0.130)	111 (0.997)	1 (0.692)	66 (0.354)					
Nucleus (193)	1444 (3.90E-04)	105 (0.961)	1684 (0.003)	18 (0.412)	76 (0.488)	203 (1.000)	1 (0.878)	214 (0.803)	272 (9.06E-07)				
Nucleus and plastids (3)	32 (0.011)	1 (0.855)	32 (0.083)	0 (1.000)	2 (0.328)	3 (0.755)	0 (1.000)	0 (1.000)	4 (0.874)	0 (1.000)			
Peroxisome (46)	352 (0.022)	18 (0.991)	436 (0.001)	6 (0.213)	21 (0.266)	73 (0.062)	0 (1.000)	59 (0.260)	100 (0.368)	2 (0.442)	19 (0.021)		
Plastids (112)	595 (1.000)	61 (0.912)	700 (1.000)	3 (0.997)	26 (0.999)	97 (1.000)	3 (0.123)	95 (1.000)	170 (1.000)	5 (0.302)	52 (0.719)	50 (0.989)	
Vacuole (58)	400 (0.463)	39 (0.401)	483 (0.354)	2 (0.961)	14 (0.980)	54 (0.997)	0 (1.000)	58 (0.902)	142 (0.036)	2 (0.564)	20 (0.968)	46 (0.999)	12 (0.945)

Figure S3. Subcellular localization analysis of the interacting proteins. For each compartment pair, the number represents the observed number of the interactions. Compartment pairs that showed enriched numbers of interactions are colored in red ($P < 0.01$).
